# Remarkable diversity of vomeronasal type 2 receptor (*OlfC*) genes of basal ray-finned fish and its evolutionary trajectory in jawed vertebrates

**DOI:** 10.1038/s41598-022-10428-0

**Published:** 2022-04-19

**Authors:** Zicong Zhang, Atsuhiro Sakuma, Shigehiro Kuraku, Masato Nikaido

**Affiliations:** 1 School of Life Science and Technology, Tokyo Institute of Technology, Tokyo, 152-8550 Japan; 2 Institute for the Advanced Study of Human Biology, Kyoto University, Kyoto, 606-8501 Japan; 3 Molecular Life History Laboratory, National Institute of Genetics, Shizuoka, 411-8540 Japan; 4 Laboratory for Phyloinformatics, RIKEN Center for Biosystems Dynamics Research (BDR), Kobe, 650-0047 Japan; 5 Department of Genetics, Graduate University for Advanced Studies, Sokendai, Shizuoka, 411-8540 Japan

**Keywords:** Molecular evolution, Evolutionary biology, Comparative genomics

## Abstract

The vomeronasal type 2 receptor (*V2R*, also called *OlfC*) multigene family is found in a broad range of jawed vertebrates from cartilaginous fish to tetrapods. *V2R*s encode receptors for food-related amino acids in teleost fish, whereas for peptide pheromones in mammals. In addition, *V2R*s of teleost fish are phylogenetically distinct from those of tetrapods, implying a drastic change in the *V2R* repertoire during terrestrial adaptation. To understand the process of diversification of *V2R*s in vertebrates from “fish-type” to “tetrapod-type”, we conducted an exhaustive search for *V2R*s in cartilaginous fish (chimeras, sharks, and skates) and basal ray-finned fish (reedfish, sterlet, and spotted gar), and compared them with those of teleost, coelacanth, and tetrapods. Phylogenetic and synteny analyses on 1897 *V2R*s revealed that basal ray-finned fish possess unexpectedly higher number of *V2R*s compared with cartilaginous fish, implying that *V2R* gene repertoires expanded in the common ancestor of Osteichthyes. Furthermore, reedfish and sterlet possessed various *V2R*s that belonged to both “fish-type” and “tetrapod-type”, suggesting that the common ancestor of Osteichthyes possess “tetrapod-type” *V2R*s although they inhabited underwater environments. Thus, the unexpected diversity of *V2R*s in basal ray-finned fish may provide insight into how the olfaction of osteichthyan ancestors adapt from water to land.

## Introduction

Olfaction is a critical chemosensory system for eliciting social behaviors in vertebrates, including reproduction, kin recognition, aggression, and feeding. The vertebrate olfaction system has experienced drastic changes in anatomy and neurophysiology during adaptation from water to land, one of the most important events is vertebrate evolution. Specifically, aquatic vertebrates detect water-soluble chemicals using their olfactory epithelium (OE), whereas terrestrial vertebrates detect both volatile and nonvolatile chemicals by differentiating their OE into main olfactory epithelium (MOE) and vomeronasal organ (VNO). Accompanied by the terrestrial adaptation of olfaction, the chemosensory receptors are proposed to have undergone major innovative diversification, although the detailed evolutionary process of diversification remains to be poorly understood at the DNA level.

Olfaction of vertebrates is mainly composed of four types of G protein-coupled receptors (GPCRs), namely, the olfactory receptor (*OR*), vomeronasal receptor type I (*V1R*), vomeronasal receptor type II (*V2R*), and trace amine-associated receptor (*TAAR*), all forming multigene families^1^. In teleost fish, *V2R*s, also referred to as *OlfC*s (olfactory receptors classified as type C GPCRs)^2,3^, are expressed in OE of the nasal cavity. Several independent studies have shown that teleost *V2R*s detect amino acids that elicit certain feeding behaviors. For example, *V2R*s are expressed in microvillous sensory neurons of zebrafish and respond to amino acids, but not bile acids or sex pheromones^4^. In addition, the genetic blockage of neural transmission in the *V2R*-expressing neurons abolishes the attractive response to a mixture of amino acids^5^. However, given that Yang et al.^6^ have proposed a possible contribution of some *V2R*s to elicit fright reactions, it is premature to rule out the possibility that *V2R*s detect some chemicals other than amino acids. In tetrapods, VNO is a specific organ that mainly detect pheromones^1^. *V2R*s are specifically expressed in the VNO of mice^7^, frogs^8^, and reptiles^9^. Hence, they are believed to encode pheromone receptors. Indeed, it has been shown that *V2R*s detect peptide pheromones^10,11^ and peptides for the major histocompatibility complex^12^.

Until now, phylogenetic analyses have revealed that teleost fish possess 20–60 *V2R*s, which are divided into 16 subfamilies^2,13,14^. On the other hand, mammalians possess variable number of *V2R*s (0–121), which are genetically closely related to each other^1,13,15,16^. In the phylogenetic tree, *V2R*s of teleost fish are distinct from those of tetrapods, which were described as “fish-like” and “tetrapod-like”^17^. Similar distinctive evolution was also observed between *V1R*s of teleost fish and tetrapods^18^, which were named “fish-type” (f-*V1R*s) and “tetrapod-type” (t-*V1R*s), respectively^19–21^. In accordance with previous studies, we hereafter use the name of "fish-type" *V2R*s (f-*V2R*s) for the putative amino acid receptor in teleost fish and "tetrapod-type" *V2R*s (t-*V2R*s) for the putative peptide receptor in tetrapods. The f-*V2R*s and t-*V2R*s are also found to be distinct in their synteny relationships in that the f-*V2R*s form single large cluster in particular chromosomes^13,14,16^ and t-*V2R*s are scattered on several chromosomes^22^. It is notable that the coelacanth, which is the close relatives of tetrapods, have both f-*V2R*s and t-*V2R*s^17^, showing that the coelacanth is an important organism as it serves as a missing link to fill an evolutionary gap between vertebrates under water and land^19,21^. Recent studies on several shark genomes have shown that olfaction in cartilaginous fish is dominated by f-*V2R*s rather than conventional *OR*s^23–25^, which is consistent with the ultrastructural observation that the presence of only microvillous sensory neurons in OE^26^ and with the immunohistochemical observations that most neurons in OE are positive for Go antibodies^27^. In addition, a previous study has shown that *V2R*s were absent in lamprey genomes^28^. Based on the above findings, *V2R*s are considered to have originated in the jawed vertebrate ancestor before the split between the two extant descendant lineages, that is, cartilaginous fish and Osteichthyes (ray-finned and lobed-finned fish including tetrapods).

In this study, we have performed a comprehensive exploration and phylogenetic analyses for *V2R*s in nine cartilaginous fish species (one elephant shark, one rabbit fish, four sharks, two skates, and sawfish), three basal ray-finned fish (reedfish, sterlet, and spotted gar), two teleost fish (eel, zebrafish), a coelacanth, two amphibians (western clawed frog and caecilian), an anole lizard, and a mouse to elucidate the process of diversification of f-*V2R*s and t-*V2R*s in vertebrates. Especially, focusing on basal ray-finned fish, which retain many ancestral characters of Osteichthyes, is of primary importance in understanding the evolution and diversification of *V2R*s from fish to tetrapods^29^. As a result, we have characterized 9–42 *V2R*s in cartilaginous fish, and a large amount (47–189) of *V2R*s in basal ray-finned fish. Phylogenetic analyses of *V2R*s of 19 various vertebrate species have revealed the existence of t-*V2R*s in the genomes of reedfish and sterlet, implying the presence of an evolutionary seed for mammalian peptide pheromone receptors in the basal ray-finned fish. The results of this study including; (1) genomic survey and identification of *V2R*s, (2) fine-scale phylogenetic and synteny analyses, and (3) mRNA expression profiles on the OE, would provide important insights into understanding the process of diversification of *V2R*s during vertebrate evolution.

## Results

### Characterization of *V2R*s from the genomes of a broad range of vertebrates

We explored *V2R* gene repertoires from the genomes of nine cartilaginous fish (one elephant shark, one rabbit fish, four sharks, and three rays), three basal ray-finned fish (reedfish, sterlet, and spotted gar), two teleost fish (zebrafish, Japanese eel), a coelacanth, two amphibians (western clawed frog, caecilian), an anole lizard, and a mouse using a software “fate”^30^. The copy numbers of *V2R*s in individual species are summarized and provided in Table 1. The number of *V2R*s in teleost fish was mostly comparable to previous studies with some update. For example, zebrafish that possessed 72 *V2R*s, which was larger than in previous studies^2,13^. The numbers of *V2R*s in cartilaginous fish ranged from 8 (rays) to 41 (chimera), which were found to be smaller than those of the teleost fish. In contrast, basal ray-finned fish possessed an unexpectedly large number of *V2R*s. In reedfish, we identified 188 intact *V2R*s, which was the largest in all ray-finned fish studied until now. The numbers of *V2R*s in sterlet and spotted gars were also high (46 and 49, respectively). The copy number of *V2R*s in the western clawed frog (691), which far exceeded that previous studies^15^, was the largest among vertebrates studied so far; it is much higher than those of other tetrapods, such as caecilian (275), anole lizard (64), and mouse (154). It is noteworthy that we found no *V2R*s in the genomes of agnathans and amphioxus.

**Table 1:** Number of intact *V2R* genes identified in the genomes of broad range of vertebrates.

Scientific name	Common name	Assembly name	V2R2	ancV2R	t-V2R	f-V2R															
			Sum	Sum	Sum	Sum	Sub 1	Sub 2	Sub 3	Sub 4–9	Sub 10	Sub 11	Sub 12	Sub 13	Sub 14	Sub 15	Sub 16	Sub a1	Sub a2	Sub a3	Unplaced
*Callorhinchus milii*	Elephant shark	Callorhinchus_milii-6.1.3	1	1	**0**	26	1	**0**	**0**	**0**	**0**	**0**	1	**0**	1	**0**	**0**	**0**	19	2	2
*Hydrolagus affinis*	Small-eyed rabbitfish	UP_Haf	1	1	**0**	39	**0**	**0**	**0**	**0**	**0**	**0**	**0**	**0**	1	**0**	**0**	**0**	36	**0**	2
*Scyliorhinus torazame*	Cloudy catshark	Storazame_v1.0	1	1	**0**	21	**0**	**0**	1	**0**	**0**	**0**	1	4	1	**0**	**0**	**0**	13	1	**0**
*Carcharodon carcharias*	Great white shark	ASM360424v1	1	1	**0**	12	2	**0**	1	**0**	**0**	**0**	**0**	**0**	**0**	**0**	**0**	**0**	8	**0**	1
*Rhincodon typus*	Whale shark	ASM164234v2	1	**0**	**0**	21	2	**0**	1	**0**	**0**	**0**	**0**	1	**0**	**0**	**0**	**0**	16	**0**	1
*Chiloscyllium punctatum*	Brownbanded bambooshark	Cpunctatum_v2.1	1	1	**0**	24	1	**0**	1	**0**	**0**	**0**	**0**	2	1	**0**	**0**	**0**	18	1	**0**
*Leucoraja erinacea*	Little skate	LER_WGS_1	1	1	**0**	6	1	**0**	1	**0**	**0**	**0**	**0**	1	**0**	**0**	**0**	**0**	2	1	**0**
*Amblyraja radiata*	LER_WGS_1	sAmbRad1.pri	1	1	**0**	6	1	**0**	1	**0**	**0**	**0**	**0**	1	**0**	**0**	**0**	**0**	2	1	**0**
*Pristis pectinata*	Smalltooth sawfish	sPriPec2.pri	1	1	**0**	22	2	**0**	1	**0**	**0**	**0**	**0**	**0**	**0**	**0**	**0**	**0**	18	1	**0**
*Erpetoichthys calabaricus*	Reedfish	Reedfish	1	1	3	183	1	2	1	**0**	4	1	1	2	1	1	67	101	**0**	**0**	1
*Acipenser ruthenus*	Sterlet	ASM1064508v1	1	1	3	41	1	11	1	**0**	1	2	1	**0**	1	**0**	1	21	**0**	**0**	1
*Lepisosteus oculatus*	Spotted gar	LepOcu1	1	1	**0**	47	**0**	1	2	**0**	1	**0**	1	1	1	1	9	29	**0**	**0**	1
*Anguilla japonica*	Japanese eel	Ajp_01	1	**0**	**0**	63	1	4	2	39	1	4	1	2	1	3	5	**0**	**0**	**0**	**0**
*Danio rerio*	Zebrafish	GRCz11	1	**0**	**0**	71	2	6	1	24	1	3	1	6	2	2	23	**0**	**0**	**0**	**0**
*Latimeria chalumnae*	Coelacanth	LatCha1	1	**0**	75	13	1	1	1	**0**	**0**	1	4	1	1	**0**	**0**	**0**	**0**	**0**	3
*Xenopus tropicalis*	Western clawed frog	UCB_Xtro_10.0	1	1	689	**0**	**0**	**0**	**0**	**0**	**0**	**0**	**0**	**0**	**0**	**0**	**0**	**0**	**0**	**0**	**0**
*Geotrypetes seraphini*	Caecilian	aGeoSer1.1	1	**0**	273	1	**0**	**0**	**0**	**0**	**0**	**0**	**0**	**0**	1	**0**	**0**	**0**	**0**	**0**	**0**
*Anolis carolinensis*	Anole lizard	AnoCar2.0	1	**0**	63	**0**	**0**	**0**	**0**	**0**	**0**	**0**	**0**	**0**	**0**	**0**	**0**	**0**	**0**	**0**	**0**
*Mus musculus*	Mouse	GRCm38.p6	7	**0**	147	**0**	**0**	**0**	**0**	**0**	**0**	**0**	**0**	**0**	**0**	**0**	**0**	**0**	**0**	**0**	**0**

The bold highlighted the cells in which no genes were found in the corresponding subfamilies.

### Evolution of f-*V2R*s of vertebrates

**Figure 1:**
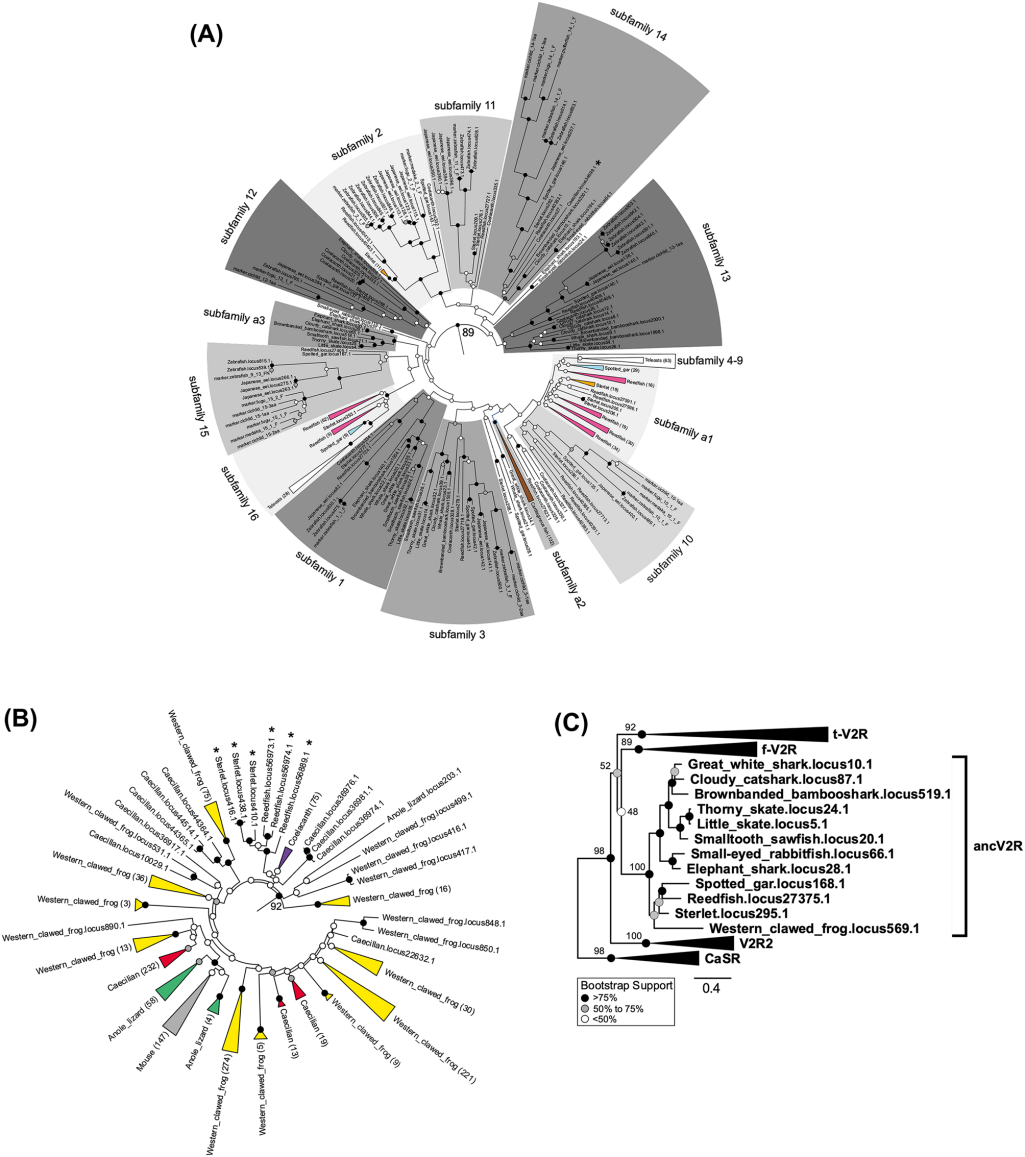
Phylogenetic relationships of *V2R*s of a broad range of vertebrates from cartilaginous fish to tetrapods constructed using RAxML-NG v.1.0.1 (https://github.com/amkozlov/raxml-ng). (**A**) Phylogenetic tree of f-*V2R*s. Note that f-*V2R*s were subdivided into 16 known and 3 novel subfamilies, as indicated by gray thick bars. Triangles in red, orange, blue, white, and brown indicate the expanded *V2R* clusters specific to reedfish, sterlet, spotted gar, cartilaginous fish, and teleost fish, respectively. Only one f-*V2R*s found in the caecilian species was marked using an asterisk. (**B**) Phylogenetic tree of t-*V2R*s. Triangles in violet, pink, yellow, green, and gray indicate expanded *V2R* clusters specific to coelacanth, caecilian, western clawed frog, anole lizard, and mouse, respectively. Asterisks were used to mark the t-*V2R*s identified in reedfish and sterlet. Note that the t-*V2R*s, in contrast to the f-*V2R*s, are composed of many clusters that are expanded in a species-specific manner. (**C**) Overview of the phylogenetic tree of all *V2R*s showing novel orthologous clade *ancV2R*. The calcium-sensing receptor (*CaSR*) gene was used for outgrouping all *V2R*s. The OTU names consist of the common name and locus as summarized in Supplementary Table S1. The f-*V2R*s, t-*V2R*s, and *V2R2* clades were compressed into black triangles. The number on the branches indicates the bootstrap support values for particular nodes. Note that the grouping of the orthologous *ancV2R*s of cartilaginous fish, basal ray-finned fish, and western clawed frog was suggested by maximum bootstrap support (100%). The numbers next to triangles indicate the copy number of *V2Rs* included in the clusters. The filled circles on each node indicate bootstrap supports (black > 75, 75 ≧ gray ≧ 50, 50 > white). Scale bar indicates the number of amino acid substitutions per site.

To classify and infer the evolutionary history of *V2R*s in vertebrates, we constructed a phylogenetic tree of all *V2R*s identified in the study (1897 from 19 species) using maximum likelihood algorithm in RAxML^31^. The phylogenetic tree with all sequences shows a clear separation of the two *V2R* clades, each of which consists of the sequences of f-*V2R*s of teleost fish and t-*V2R*s of tetrapods, respectively (Supplementary File 2). Therefore, the phylogenetic trees of the two clades consisting of f-*V2R*s (Fig. 1A) and t-*V2R*s (Fig. 1B) were shown separately for better visibility of the details. A simplified phylogenetic tree of f-*V2R*s, t-*V2R*s, and other two additional clades are shown in Fig. 1C.

In the phylogenetic tree of f-*V2R* clade, we identified all known 16 subfamilies^13,14^ in addition to the newly identified subfamilies “a1”, “a2” and “a3” (Fig. 1A). Notably, over half of the *V2R*s of the basal ray-finned fish were included “a1”. Also, we have found a cartilaginous fish-specific subfamily “a2” and “a3” (Table 1, Figs. 1A, S1). In contrast to ray-finned fish in which *V2R* subfamilies were highly diverse, cartilaginous fishes were relatively less diverse (Table 1). One exceptional finding was that only one sequence of caecilian *V2R* was classified as being a member of the f-*V2R* subfamily 14 (Table 1, Fig. 1A, marked with an asterisk).

### Evolution of t-*V2R*s of vertebrates

The t-*V2R*s clade were shown to be mainly dominated by closely related *V2R*s of tetrapods that were genetically closely related each other^9,13,15^. Notably, *V2R*s of amphibians are highly diverse in that they are subdivided into many species-specific clusters, while all *V2R*s of mouse belonged to a single cluster (Fig. 1B, Table 1). The existence of 75 *V2R*s of coelacanth in t-*V2R* clade was consistent with a previous study (Fig. 1A, Table 1)^17^. One of the most striking results of this study was that six *V2R* sequences of ray-finned fishes (reedfish and sterlet) were classified into t-*V2R*s. Indeed, in the phylogenetic tree, these *V2R*s were sister group of the coelacanth *V2R* cluster (Fig. 1B and Supplementary File S2). Although the existence of *V2R*s of basal ray-finned fish in t-*V2R* clade leads to a slight confusion in definitions, we will continue to adopt this nomenclature, taking into account that; 1. classification in previous studies used these names for each clade^17,19,21^, and 2. dual character (fish-like as well as tetrapod-like) of the basal ray-finned fish is meaningful in discussing vertebrate evolution (see discussion).

### Newly identified *V2R* orthologs conserved from cartilaginous fish to amphibians

Previous studies have shown that *V2R*s were divided into three well-supported clades, namely, the *V2R2*s, f-*V2R*s, and t-*V2R*s^17,22,28^. The orthologous *V2R2*s were shared in all vertebrates with a few species-specific duplications in mice^28,32^. In this study, *V2R2* ortholog of all vertebrates was located at the basal position of the *V2R* tree (Table 1, Fig. 1C, Supplementary Fig. 1). In addition to the three clades, we identified a novel clade, in which *V2R*s of cartilaginous fish, basal ray-finned fish, and amphibians were included (Fig. 1C). A closer inspection of the genome assembly did not reveal any apparent errors that could lead to the generation of the artifact sequences. Since this clade contained just one *V2R* from each species, and the tree topology was identical to the species tree, these *V2R* sequences were considered to be orthologous. This orthologous *V2R*s were evolutionarily distinct from the f-*V2R*s and t-*V2R*s, which were amplified in a species-specific manner. Considering that only one ortholog has been retained since quite a long time ago, we named them *anc* (ancient) *V2R*. In this study, highly conserved *ancV2R* sequences were found in the genomes of cartilaginous fish to amphibians, but not in teleost fish, coelacanth, and mammals (Fig. 1C).

### Conserved gene clusters of f-*V2R*s

**Figure 2:**
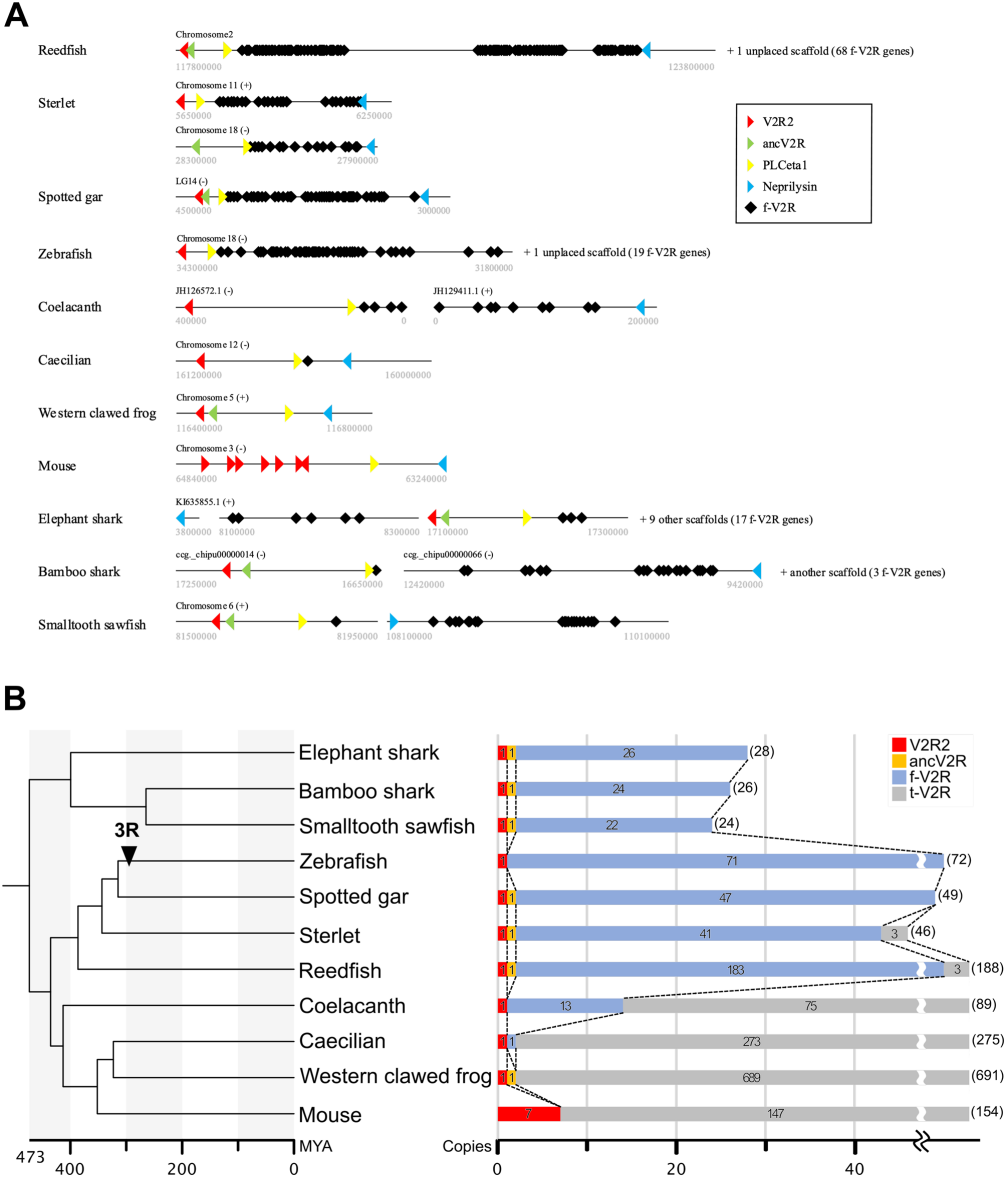
(**A**) Synteny relationships for the f-*V2R* clusters among basal ray-finned fish (reedfish, sterlet, spotted gar), teleost fish (zebrafish), lobe-finned fish (coelacanth), tetrapods (caecilian, western clawed frog, mouse), and cartilaginous fish (elephant shark, bamboo shark, and smalltooth sawfish), respectively. Triangles in yellow, blue, red, and green indicate two landmark genes (*PLC eta1*, *neprilysin*), *V2R2*, and *ancV2R*, respectively. Black squares indicate f-*V2R*s. Indicated at the upper-left of each line were several chromosomes or scaffolds and its directions. Indicated below the ends of the line are the start and end of the cluster regions. Unplaced scaffolds are not shown in this figure. Note that f-*V2R*s were flanked by two landmark genes and that *V2R2*s and *ancV2R*s were located in tandem close to the clusters. No t-*V2R*s were observed in these cluster regions. In the elephant shark, some f-*V2R*s are located outside the cluster because the cluster regions were not properly assembled. (**B**) Changes in the number of *V2R*s during vertebrate evolution. The phylogenetic tree with timescale for 11 representative vertebrates (left) and the number of *V2R*s in these species (right) are shown. The timing of teleost-specific third round whole genome duplication (3R) is indicated by arrow. The color and number on the bar graph indicate the clade and copy number, respectively. The total copy number of *V2R*s is shown in parentheses on the right of the graph. MYA: million years ago.

In addition to phylogenetic analysis, the synteny relationships have provided important insight into the classification of *V2R*s. Previous studies have shown that the *V2R*s in teleost were clustered in one particular chromosomal region, which was flanked by two landmark genes, *phospholipase C* (*PLC*) *eta1* and *neprilysin*^13,14,16^. In contrast, no *V2R*s were found between these two genes in tetrapods, and t-*V2R*s were scattered into several chromosomes^22^. Therefore, to ascertain if the *V2R*s are f-*V2R*s or t-*V2R*s, we examined the synteny relationships, in which the gene arrangements in the genomic region of two landmark genes in various vertebrates from cartilaginous fish to mammals were summarized (Fig. 2A). Notably, *V2R2* and *ancV2R* was found in tandem of the neighboring regions of the *PLC eta1*. No *V2R* was found in tetrapods in this region, except for only one caecilian *V2R*, which was classified as f-*V2R*s in the phylogenetic tree (Fig. 1A). Importantly, we revealed that f-*V2R*s were located in this cluster region. In addition, three t-*V2R*s identified in reedfish and sterlet were located on different chromosomes (chr.3 in reedfish, chr.52, 53 and VTUV01000346.1 in sterlet, Supplementary Table S1). In sterlet, we identified two distinct chromosomal regions of the f-*V2R* clusters, which were due to polyploidization specific to this group^33^. The synteny of coelacanth also showed a conservation of the f-*V2R* cluster. Overall, the phylogenetic and synteny analyses both supported the conservation of the cluster for f-*V2R*s as well as the existence of the t-*V2R*s in basal ray-finned fish.

### The copy number of *V2R*s in vertebrate evolution

We investigate the evolutionary trends of *V2R*s in terms of copy number changes by mainly focusing on f-*V2R*s and t-*V2R*s (Fig. 2B). The copy numbers of *V2R2* and *ancV2R* were constant, which were mostly one in any species. The cartilaginous fish possess relatively small number of *V2R*s, which are dominated by f-*V2R*s. The *V2R*s suddenly increased in ray-finned fish, which were also dominated by f-*V2R*s. Notably, three copies of t-*V2R*s emerged in basal ray-finned fish, represented by reedfish and sterlet, but were lost in teleost fish. Then, f-*V2R*s decreased and t-*V2R*s increased in coelacanth and tetrapods. Thus, the changes in the proportion of f-*V2R*s and t-*V2R*s in the genomes appears to coincide with evolutionary transition of vertebrates from water to land. Overall, it is obvious that sudden increase of *V2R*s occurred three times, namely, in the common ancestor of Osteichthyes, in the common ancestor of tetrapods, and in Western clawed frog. By contrast, sudden increase of *V2R*s was not observed in the lineage of teleost fish.

### Expression of *V2R*s in the olfactory epithelium of basal ray-finned fish

**Figure 3:**
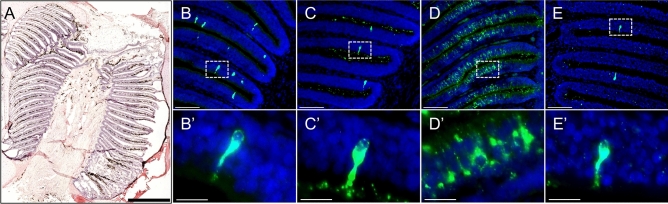
The expression patterns of *V2R*s in the olfactory epithelium of the basal ray-finned fish, *Polypterus senegalus*. Overall view of a HE-stained horizontal section of the olfactory organ, showing that lamellae of olfactory organ are stained blue-violet, and the nerve bundles are stained red-violet (**A**). Expression of four *V2R* genes were confirmed by FISH on horizontal sections of the olfactory organ of the bichir using DIG-labeled RNA antisense probes for f-*V2R* (**B**) and t-*V2R* (**C**), *V2R2* (**D**), and *ancV2R* (**E**). Green indicated the expression signals. The blue area indicates the cell nucleus stained with DAPI. *V2R2* was globally expressed in the deep layers of olfactory lamellae (**D**). In contrast, f-*V2R*, t-*V2R*, and *ancV2R* were sparsely expressed in a small number of neurons in the deeper layers of the olfactory lamellae (**B**, **C**, and **E**). (**B**’-**E**') High magnification view of the dotted squares in **B**–**E**. Scale bars show 1 mm (**A**), 100 µm (**B**–**E**) and 20 µm (**B**'–**E**').

To evaluate the functional role of *V2R*s found in the genomes of basal ray-finned fish, we examined the cellular expression patterns for these receptors in the OE. The locations of transcripts for four different *V2R*s were detected by in situ hybridization on frozen sections of the OE of *Polypterus senegalus* (bichir), which is a basal ray-finned fish closely related to reedfish (Fig. 3). The probes of the four *V2R*s f-*V2R*, t-*V2R*, *V2R2*, and *ancV2R* each of which has been identified as a distinct clade in the phylogenetic tree (Fig. 1C), were used in the experiments. The expression of a member of the f-*V2R*s showed a sparse pattern in the sensory cells of the OE, typical of canonical *V2R*s (Fig. 3B). The expression of a member of t-*V2R*s, which was newly identified in basal ray-finned fish, also showed similar sparse pattern in the OE of *P. senegalus* (Fig. 3C). The expression of *V2R2* has showed widespread patterns in their OE (Fig. 3D). The expression pattern of *V2R2* in *P. senegalus* was consistent with the ubiquitous expression in zebrafish^34^ and mouse^32^. The expression of *ancV2R* showed a sparse pattern in their OE (Fig. 3E). Overall, the *V2R*s belonging to four clades were all expressed in the OE, suggesting their functions as olfactory receptors. However, the patterns of expressions were ubiquitous in *V2R2,* while they were sparse in *V2R*s of other clades (Table 2).

**Table 2:** Summary of current knowledge on four *V2R* clades.

	*V2R2*	*ancV2R*	f-*V2R*	t-*V2R*
**Specific characters**				
Expression	Broad^a,^^32^	Sparse^b^*	Sparse^5,35^	Sparse^7,10^
Gene number	One or a few^32^	One*	Expanded^2,13,14^	Expanded^7,9,15^
Ligands	Amino aicds^34^	Unknown	Amino acids^5^	Peptides^10–12^
Habitat (water/land)	Both	Both	Mainly water	Mainly land
**Presence (P)/absence (A)**				
Cartilaginous fish	P	P*	P	A
Basal ray-finned fish	P	P*	P*	P*
Teleost fish	P	A	P	A
Coelacanth	P	A	P	P
Amphibians	P	P*	P*	P
Reptiles	P	A	A	P
Mammals	P	A	A	P

*Newly found in this study.

^a^broadly expressed in all vomeronasal neurons and co-expressed with the other *V2R*s.

^b^sparsely expressed according to one neuron one receptor rule.

## Discussion

In this study, we have conducted a comprehensive exploration of *V2R* sequences from the genomes of 19 vertebrate species. Phylogenetic analyses of a large number of *V2R*s allowed us to gain a panoramic view of the diversity in terms of copy number and repertoire of *V2R*s across vertebrates. According to a recent study by Bi et al.^29^, more than 50 *V2R*-like sequences in hagfish and a few in amphioxus were observed. However, our phylogenetic analyses revealed that they did not form a cluster with known *V2R*s. Therefore, we need to be still cautious to designate these sequences as *V2R*s, reaching a conclusion that no typical *V2R*s exist in agnathans and amphioxus. In addition, we need to remain careful about the detail copy numbers, as it depends on the quality of the genome assemblies, which were relatively low in some cartilaginous fish. Based on the current data set obtained in this study, we here discuss the evolution of *V2R*s and how these factors drive the adaptive evolution of the olfactory system in vertebrates.

It is obvious that *V2R*s were abundant in ray-finned fish compared to cartilaginous fish, which is achieved by a species-specific expansion of f-*V2R*s. Notably, species-specific expansions of *V2R*s did not occur uniformly in all subfamilies, but were rather concentrated in certain ones. For example, the expansion of *V2R*s was mainly observed in subfamilies 4–9, 16, ‘a1’, and ‘a2’, while the copy numbers of other subfamilies remained one or two. At present, *V2Rs* are expected to detect amino acids and their derivatives, eliciting feeding behaviors in teleost fish^4,5,35^. It is reasonable to assume that a limited number of amino acids in diets were received by evolutionarily conserved *V2R* subfamilies. However, it is plausible to assume that the *V2R* subfamily with frequent lineage-specific gene duplications is responsible for detecting some species-specific variable chemicals for social communication. For example, Yambe et al.^36^ showed that an amino acid derivative, L-kynurenine, secreted in the female urine, acts as the male-attracting pheromone in masu salmon. In addition, a previous study showed a possible correlation between expansions of *V2R*s in subfamily 9 and the evolution of fright reactions in teleost fish^6^. Thus, to elucidate the function of *V2Rs* in ray-finned fish in addition to amino acid detection, it is necessary to further examine the *V2Rs* from a multidisciplinary framework, including the ligand binding, and behavioral experiments using candidate chemicals.

We examined the possible link between *V2R* expansion and whole genome duplication (WGD). First, we precisely examined the presence/absence of 2:1 relationship for Western clawed frog, in which *V2R*s were expanded (Fig. 1B, Supplementary File 1). As a result, we failed to find such relationship between *V2R*s of Western clawed frog and the other species. The results suggested that the expansion of *V2R*s in Western clawed frog was not by WGD but by species-specific gene expansion. Indeed, it has been shown that Western clawed frog did not experience WGD specific to this species^39^. Previous studies have revealed that the third round of WGD has occurred in the common ancestor of Teleostei, namely, after the split of teleost fish and spotted gar about 300 million years ago (called 3R, Fig. 2B)^37,38^. However, expansion of *V2R*s was not observed in the lineage of teleost fish. We precisely examined the 2:1 relationship for each *V2R* of teleost fish and spotted gar by checking the phylogenetic tree, again failed to find such relationship (Fig. 1A, Supplementary File 1). In addition, *V2R*s of teleost fish were located at only one genomic region (Supplementary Table S1, Fig. 2A). Therefore, WGD is unlikely to have made a significant impact on the expansion of *V2R*s during vertebrate evolution. Recent study on the keratin multigene family also revealed no apparent link between the gene expansion and WGD^40^. In multigene families such as chemoreceptor genes and keratin genes, tandem duplication, not WGD, may be the main driving force to increase copy number.

We showed that orthologous sequences of *V2R2* and newly identified *ancV2R* have long been conserved during the evolution of vertebrates. Although the order of divergence among *ancV2R*, t-*V2R*s and f-*V2R*s are uncertain due to the lack of sufficient bootstrap support, *ancV2R* is expected to have first emerged by tandem duplication from *V2R2*. The existence of *V2R2* and *ancV2R* in close genomic proximity may also imply this scenario (Fig. 2A). Studies by Silvotti et al^32^*.* in the mouse and DeMaria et al^34^*.* in zebrafish have revealed that highly conserved *V2R2* was expressed in a broad area of the OE and was co-expressed with one of the many canonical *V2R*s. Consistent with previous studies, *V2R2* of *Polypterus senegalus* showed widespread expression patterns in the OE (Fig. 3D), while the f-*V2R*s and t-*V2R*s showed sparse patterns (Fig. 3B, C). Although *ancV2R* was similar to *V2R2* in terms of evolutionary conservation and genomic proximity, the pattern of expression in the OE was sparse rather than widespread (Table 2). It is implicative to note that *ancV2R* has characteristics between *V2R2* and canonical f-*V2R*s and t-*V2R*s. Thus, taking the evolutionary conservation as well as the sparse pattern of expression into account, *ancV2R* may have retained ancestral nature inherited from a protogene before the split between f-*V2R*s and t-*V2R*s, which are now highly diversified in jawed vertebrate genomes. It was of interest that the expression patterns of *V2R2*, *ancV2R*, and f-*V2R*s were distinct despite their location on the same genomic cluster. A detailed investigation into this genomic region would lead to the elucidation of a *cis*-regulatory mechanism that controls the expression of canonical *V2R*s, as to say “one neuron one receptor” rule^41^.

**Figure 4:**
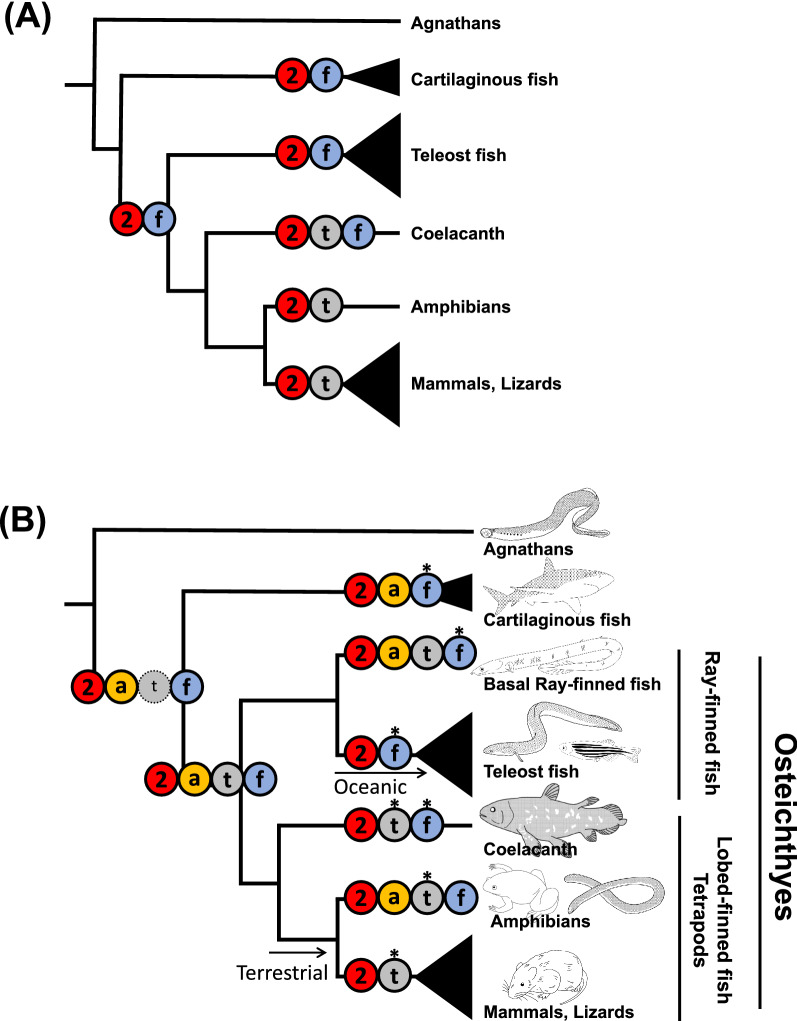
Evolutionary scenarios of *V2R*s during vertebrate evolution proposed by previous (**A**) and this (**B**) studies. The presence/absence of the four major *V2R* clades was plotted on the phylogenetic tree of vertebrates from agnathans to mammals. The red circle with “2,” yellow with “a,” gray with “t,” and blue with “f” indicate *V2R2*, *ancV2R*, t-*V2R*s, and f-*V2R*s, respectively. (**A**) Except for *V2R2*, teleost fish and tetrapods possess only f-*V2R*s or t-*V2R*s, respectively, while the coelacanth possesses both *V2R*s. Common ancestor of Osteichthyes was expected to possess *V2R2* and f-*V2R*s. (**B**) The large-scale analyses revealed the existence of t-*V2R*s in basal ray-finned fish and identified a novel clade of *ancV2R*. In contrast to basal ray-finned fish with all four clades of *V2R*s, teleost fish, mammals, and lizards possess only two of them. The reduction of specific *V2R* clades in these lineages would be due to adaptation to specific oceanic and terrestrial environments. Note that the origin of t-*V2R*s dates back to the common ancestor of extant Osteichthyes, but its antiquity in the jawed vertebrate ancestor remains to be examined with complete genome sequences of more cartilaginous fish (dotted circle with “t” inside). Asterisks above the circles indicate clades with substantial copy numbers due to gene expansions in each extant species. The illustration of the animals was drawn by using free software Vectr (https://vectr.com).

The previous (A) and present (B) hypotheses for evolutionary scenarios of the four clades of *V2R* in vertebrates from agnathans (lamprey and hagfish) to mammals were shown in Fig. 4, by marking the distribution of these four *V2R* clades in extant species and two ancestral nodes. In addition, the character of the four clades of *V2R*s were summarized in Table 2. The newly characterized *V2R*s in cartilaginous fish, basal ray-finned fish and caecilian provided new insights into *V2R* evolution in vertebrates. No *V2R* was found in the agnathans, implying the acquisition of a *V2R*-mediated olfaction as a common ancestor of jawed vertebrates (cartilaginous fish and Osteichthyes). Given that agnathans have three other types of chemoreceptor genes, namely, *OR*s, *TAAR*s, and *V1R*s, it is likely that *V2R*s were originated later than these three receptors^28,42^. *V2R2* was conserved in all extant jawed vertebrates with no exceptions, which suggests the highly important and fundamental role of *V2R2* in the detection and subsequent signaling pathway for olfactory substances in both underwater and terrestrial environments. Importantly, except for *V2R2*, mammal and teleost fish possessed only specific clades of *V2R*s, namely, t-*V2R*s and f-*V2R*s, respectively. In contrast, basal ray-finned fish and one amphibian (caecilian) possessed both t-*V2R*s and f-*V2R*s. This finding suggests that all four clades of *V2R*s were present at least in the common ancestor of Osteichthyes, or even earlier in the common ancestor of jawed vertebrates. However, subsequently, the repertoires of *V2R*s were lost in teleost fish and mammals during adaptation to their specific environments. It is interesting to note that one copy of f-*V2R* is exceptionally retained in caecilian. This may reflect its ancestral position in Amphibia, its water-dependent habitat, and/or subterranean lifestyle, but further studies are needed to examine these possibilities. Because the previous studies of *V2R*s have been limited to teleost fish and mammalians, the diversity of *V2R*s was underestimated. Thus, the genome sequences of non-model animals, which become available due to the recent advancement of sequence technique, allowed us to uncover the hidden and remarkable diversity in the genomes of vertebrates.

One of the important discussions is the timing of *V2R*s diversification. The repertoires of the subfamily of the f-*V2R*s are abundant in ray-finned fish and coelacanth compared with those in cartilaginous fish (Table 1). In addition, all four clades of *V2R*s were shown to be present in basal ray-finned fish and amphibians. The abundance of copy numbers and repertoires in these groups suggests that *V2R*s were highly diversified in the common ancestor of Osteichthyes. The diversification of *V2R*s is not caused by WGD, but is most likely a signature of adaptive evolution. Given that the olfaction-related genes also emerge in the common ancestor of the Osteichthyes (e.g., *ancV1R*^43^; *OMP*^44^), it might be possible that an innovative evolution of the olfactory system occurred in this timing.

It is also worth mentioning here that the polypterids (bichir and reedfish) possesses large paired openings (spiracles) on top of their head, in which they use for breathing air^45^. Similar spiracle-like structures were observed in the fossil records of stem tetrapods^46^. Thus, breathing air using spiracles may have been an important respiratory strategy in the stem Osteichthyes, which inhabit shallow freshwater environments and use lungs in addition to gills for respiration^47^. Specifically, the evolution of air-breathing by spiracles may increase the opportunity to raise their head above water, which led to the acquisition of the primitive capabilities of detecting airborne chemicals before terrestrial adaptation. Thus, such dual functional roles of the olfactory system in stem Osteichthyes were related to the diversification of *V2R*s, including f-*V2R*s and t-*V2R*s. However, at this moment, both types of *V2R*s are expected to detect non-volatile ligands such as amino acids and peptides (Table 2), thus the adaptive significance of the emergence of t-*V2R*s in stem Osteichthyes remains unclear. Deorphanization of *V2R*s and/or the exploration of *V2R* expressions in various organs including non-OE in basal ray-finned fish in the near future is necessary to evaluate the above possibility.

## Materials and methods

### Sequence retrieval

To estimate the evolutionary history of *V2R*s, we conducted a comprehensive exploration of *V2R* sequences in the genome assemblies of a broad range of vertebrates, including nine cartilaginous fishes and three basal ray-finned fishes. In addition to two teleost fishes (Japanese eel and zebrafish), coelacanth, two amphibians (caecilian and western clawed frog), anole lizard, and mouse were explored (Table 1). To identify the *V2R* sequences from the genome assemblies, we performed tBLASTn searches using the transmembrane (TM) domain of *V2R*s as queries against all of the genomic sequences. The query sequences were generated by aligning *V2R* sequences of mouse, anole lizard, tropical clawed frog, coelacanth, spotted gar, and zebrafish deposited in the Ensembl database and of elephant shark and cloudy catshark^24^ using the MA–T –dash option^48^. Then, the TM domains, from 50-aa sequences 7n1upstream of the 1st TM region to 50-aa downstream of the 7th TM region, were extracted using a protein structure of the glutamate receptor (PDB: 4OR2)^49^ as reference.

Next, the *V2R*-coding sequences were predicted for tBLASTn hit regions using GeneWise v.2.4.1^50^. The above procedures were conducted by using a software “fate”, which is developed specifically for the identification of multiple gene families^30^. Sequences shorter than 600 nucleotides were discarded. The homology of intact outputs was then determined by the next phylogenetic analysis. The queries used in the BLAST search were critical to the estimated number of *V2R*s. Indeed, the number of *V2R*s identified in this study was consistent with previous studies, whereas a few differences were observed in some cases. For example, the numbers of *V2R*s in elephant shark and catshark identified in this study (29 and 24 copies) were slightly smaller than those reported by Sharma et al.^24^. The difference in query sequences used for the BLAST search reflects results. Specifically, we used the seven-transmembrane regions of the *V2R*, whereas Sharma et al.^24^ used the entire region of *V2R*, including the extracellular Venus flytrap module region (VFTM)^51^ The VFTM region of the *V2R* protein has been determined to be highly diverse and difficult in predicting the exon–intron structure of the gene. This result affects the judgment of intact or pseudogenes. Therefore, we compared the number of *V2R*s estimated under a unified condition using the seven-transmembrane region as a query.

### Phylogenetic and synteny analyses

Sequences were translated into amino acid sequences and aligned using MAFFT with the -ginsi option^52^ and default parameters. The sites with < 50% coverage among all sequences were removed. Maximum likelihood trees for V2R genes were then inferred using RAxML-NG v.1.0.1^31^ with the JTT + G + F amino acid substitution model. This result was the best fitting model selected by the ModelTest-NG^53,54^ based on AIC scores. Rapid bootstrap analyses were performed using 100 replicates to assess the reliability of nodes. The *V2R* sequences for coelacanth, western clawed frog, and mouse were then clustered based on the criteria of 80% similarity at the amino acid level to save computational costs. To avoid false positives in identification of the *V2R* genes and prevent long branch attractions, we obtained other GPCR family C sequences from GenBank or Ensembl for use as outgroups, including CaSR (NM_013803.3), Tas1r1 to Tas1r3 (ENSMUSG00000028950, ENSMUSG00000028738, ENSMUSG00000029072), GPCR6 (NM_153071.1), GRM1a to GRM8 (NM_016976.1, NM_001160353.1, NM_181850.2, NM_001081414.2, NM_001081414.2, NM_173372.2, NM_177328.3, NM_001361125.1), and GABA B1 to GABA B2 (NM_019439.3 and NM_001081141.2), for constructing an initial gene tree. All genes in the sister clade to *CaSR* were named homologs of *V2R* (including *V2R2*). Using these genes, we constructed the *V2R* gene tree again (*CaSR* was used as an outgroup). We also included sequences of all 16 teleost fish subfamilies classified in previous studies^13,14^, as markers to indicate *V2R* subfamilies.

The synteny relationships of f-*V2R*s of vertebrates were then illustrated based on the genomic location of the identified *V2R*s (Supplementary Table S1). The numeric data for the genomic position of *V2R*s, which were identified as f-*V2R*s, *V2R2*, and *ancV2R* in the phylogenetic tree, were then compiled, with those of *PLC eta1* and *neprilysin*.

### Histology and in situ hybridization

Bichir (*Polypterus senegalus*) individuals of 11–25 cm long, which were used for the preparation of frozen sections and the extraction of total RNA from the olfactory organs, were purchased from a commercial supplier; they were kept under standard conditions suitable for tropical fish breeding until experimental manipulations. All experimental studies using the animals were approved by the Institutional Animal Experiment Committee of the Tokyo Institute of Technology were performed in accordance with the institutional, governmental ARRIVE guidelines. In addition, all methods were performed in accordance with the relevant guidelines and regulations. TRIzol (Invitrogen) was then used for total RNA extraction from the olfactory organs of the bichir. Using the total RNA extracted from the olfactory organs of the bichir, cDNA was synthesized by reverse transcription reaction using SuperScript III RTase (Invitrogen). Coding regions of *V2R* were amplified by PCR using the primer sets, which were designed on the basis of *V2R* sequences of reedfish, as has been summarized in Supplementary Table S2. The PCR products were cloned using the pGEM-T vector (Promega) and the DH5α strain of *E. coli*. Digoxigenin-labeled RNA probes were synthesized using the plasmid vector as a template using T7 or SP6 RNA Polymerase (Roche) and DIG RNA labeling mix (Roche) as well. The olfactory organs of the bichir were then fixed with 4% PFA, replaced with sucrose, and embedded in an O.C.T compound (Sakura Finetek). In situ hybridization was performed according to the method as previously described^43,44^. Briefly, hybridization was performed using DIG-labeled RNA probes. The antibody reaction was conducted using anti-digoxigenin-POD, Fab fragments (Sigma-Aldrich), or anti-fluorescein-POD, Fab fragments (PerkinElmer). The signal was then amplified with Tyramide Signal Amplification Plus Biotin kit (Kiko Tech), and detected by streptavidin, Alexa Fluor 488 conjugate (Thermo Fisher). Finally, the sections were sealed using a VECTASHIELD Mounting Medium with DAPI (VECTOR). The sealed sections were observed using a fluorescence microscope Axioplan (Carl Zeiss). All fluorescence photographs were taken using an Axiocam 503 color (Carl Zeiss) and optimized for brightness and contrast in Adobe Photoshop.

For histological observation, the frozen sections of the bichir olfactory organs were stained with hematoxylin for 4 min and washed with tap water. The sections were then stained with eosin for 10 min and treated with 70% ethanol for 1 min, 80% ethanol for 1 min, 90% ethanol for 1 min, and 100% ethanol for 5 min three times. The sections were then treated with xylene for 5 min and three times and sealed in ENTELLAN NEW (MERCK).

## Supplementary Information

Supplementary Information.
