# Supplementary material for: Remarkable diversity of vomeronasal type 2 receptor (OlfC) genes of basal ray-finned fish and its evolutionary trajectory in jawed vertebrates
Source: Sci Rep. 2022 Apr 19;12:6455. doi: 10.1038/s41598-022-10428-0 (PMC9018814; doi:10.1038/s41598-022-10428-0)
Supplement: Supplementary file 1 — Supplementary Information. [file 41598_2022_10428_MOESM1_ESM.pdf]

Supplementary Information

Remarkable diversity of vomeronasal type 2 receptor (*OlfC*) genes of basal ray-finned fish and its evolutionary trajectory in jawed vertebrates

Zicong Zhang, Atsuhiro Sakuma, Shigehiro Kuraku and Masato Nikaido

Supplementary Figure S1. A phylogenetic tree of subfamily “a2” and the cartilaginous fish-specific *V2R*s constructed using RAxML-NG v.1.0.1 (https://github.com/amkozlov/raxml-ng). Note that this subfamily consists of 132 *V2R*s, most of which were expanded in species-specific birth and death processes. Only one orthologous *V2R* was shared among cartilaginous fish (indicated by asterisk).

Supplementary Table S1. Summary of the intact *V2R*s in the genomes of vertebrates. Gene name, locus (position in the chromosomes, scaffolds), and directions were summarized in a bed file format.

Supplementary Table S2. PCR primers used to amplify four *V2R* genes of *Polypterus senegalus*.

Supplementary File 1. The aligned *V2R* sequences of all 1897 intact *V2R*s identified from various vertebrates.

Supplementary File 2. The Newick format phylogenetic tree of all 1897 intact *V2R*s identified from 19 vertebrates.

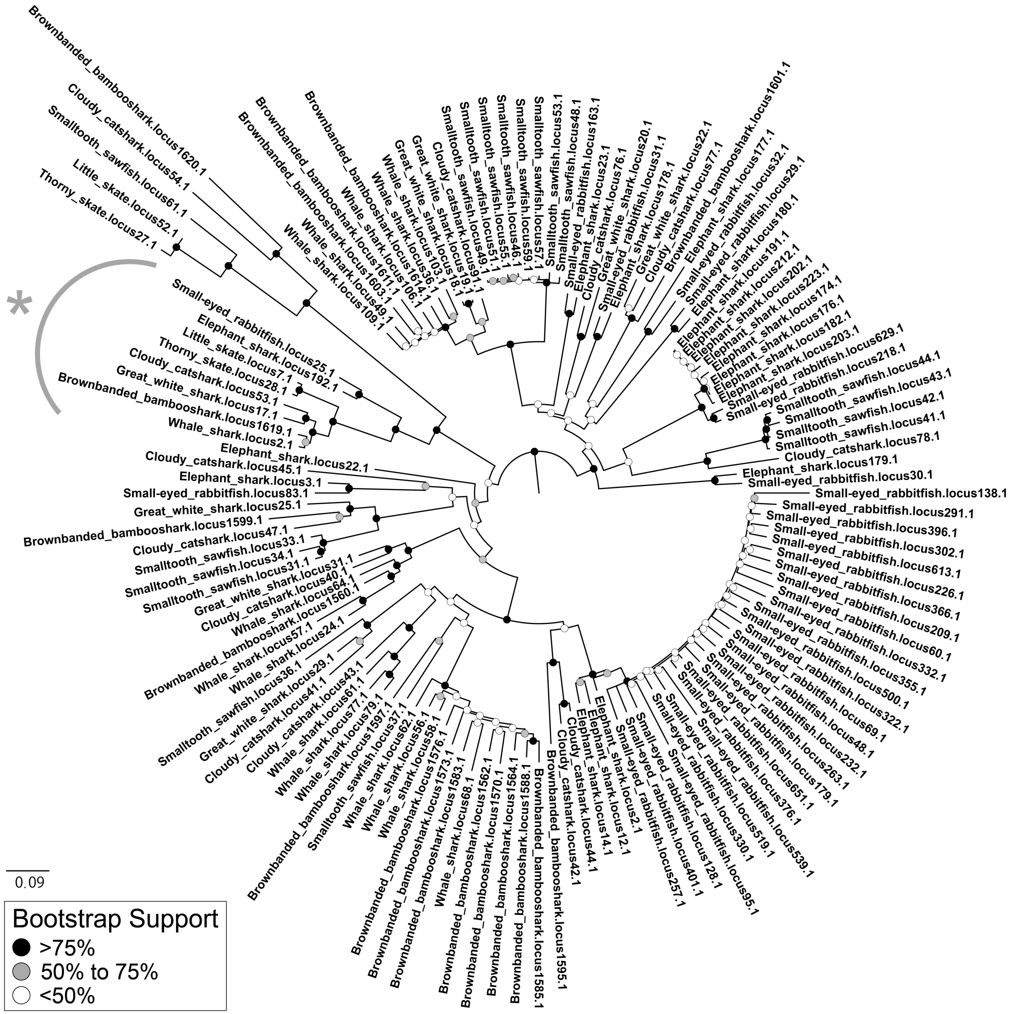

Supplementary Figure S1. A phylogenetic tree of subfamily "a2" and the cartilaginous fish-specific *V2R*s. Note that this subfamily consists of 132 *V2R*s, most of which were expanded in species-specific birth and death processes. Only one orthologous *V2R* was shared among cartilaginous fish (indicated by asterisk).

Table S1. Summary of the intact V2Rs in the genomes of vertebrates. Gene name, locus (position in the chromosomes, scaffolds), and directions were summarized in a bed file format.

Elephant_shark

KI635855.1	8113726	8114764	locus2.1	848.45	-	8113726	8114764	0,0,255	1	1038	0
KI635855.1	8119816	8120716	locus3.1	742.45	-	8119816	8120716	0,0,255	1	900	0
KI635855.1	8177093	8178050	locus12.1	797.41	-	8177093	8178050	0,0,255	1	957	0
KI635855.1	8192636	8193665	locus14.1	843.28	-	8192636	8193665	0,0,255	1	1029	0
KI635855.1	8227763	8228576	locus22.1	669.02	-	8227763	8228576	0,0,255	1	813	0
KI635855.1	8240920	8241826	locus23.1	735.32	+	8240920	8241826	0,0,255	1	906	0
KI635855.1	17102292	17103270	locus27.1	793.77	-	17102292	17103270	0,0,255	1	978	0
KI635855.1	17115829	17116807	locus28.1	792.8	-	17115829	17116807	0,0,255	1	978	0
KI635855.1	17233500	17234400	locus29.1	742.05	+	17233500	17234400	0,0,255	1	900	0
KI635855.1	17241521	17242424	locus30.1	745.96	+	17241521	17242424	0,0,255	1	903	0
KI635855.1	17252805	17253687	locus31.1	733.15	+	17252805	17253687	0,0,255	1	882	0
KI635949.1	2150304	2151231	locus140.1	760.16	-	2150304	2151231	0,0,255	1	927	0
KI636120.1	1422	2370	locus174.1	789.26	+	1422	2370	0,0,255	1	948	0
KI636120.1	134939	135887	locus176.1	790.01	+	134939	135887	0,0,255	1	948	0
KI636120.1	145558	146461	locus177.1	750.83	+	145558	146461	0,0,255	1	903	0
KI636120.1	153579	154395	locus178.1	669.82	+	153579	154395	0,0,255	1	816	0
KI636120.1	160538	161528	locus179.1	819.89	+	160538	161528	0,0,255	1	990	0
KI636120.1	170622	171534	locus180.1	751.98	+	170622	171534	0,0,255	1	912	0
KI636120.1	387729	388536	locus182.1	659.2	-	387729	388536	0,0,255	1	807	0
KI636613.1	1773	2580	locus191.1	742.52	-	1773	2580	0,0,255	1	807	0

KI636613.1	10429	11227	locus192.1	656.84	-	10429	11227	0,0,255	1	798	0
KI636613.1	26870	27686	locus193.1	671.53	-	26870	27686	0,0,255	1	816	0
KI636730.1	18084	18972	locus194.1	734.37	+	18084	18972	0,0,255	1	888	0
KI637190.1	13622	14435	locus202.1	759.66	+	13622	14435	0,0,255	1	813	0
KI637259.1	1855	2761	locus206.1	742.5	-	1855	2761	0,0,255	1	906	0
KI637457.1	7146	7953	locus212.1	749.02	-	7146	7953	0,0,255	1	807	0
AAVX020441	1726	2539	locus203.1	763.66	+	1726	2539	0,0,255	1	813	0
AAVX020477	5736	6549	locus223.1	752.16	+	5736	6549	0,0,255	1	813	0

Small-eyed_rabbitfish

JAAILG010023761.1	7699	8602	locus128.1	604.77	-	7699	8602	0,0,255	1	903	0
JAAILG010115746.1	0	840	locus500.1	606.08	-	0	840	0,0,255	1	840	0
JAAILG010055919.1	747	1792	locus322.1	609.36	+	747	1792	0,0,255	2	274,755	0,290
JAAILG010059427.1	1243	2146	locus332.1	593.78	-	1243	2146	0,0,255	1	903	0
JAAILG010064328.1	344	1385	locus355.1	619.77	-	344	1385	0,0,255	1	1041	0
JAAILG010015209.1	3558	4599	locus69.1	614.77	-	3558	4599	0,0,255	1	1041	0
JAAILG010072115.1	1336	2239	locus376.1	643.25	-	1336	2239	0,0,255	1	903	0
JAAILG010181541.1	0	717	locus651.1	522.45	+	0	717	0,0,255	1	717	0
JAAILG010021320.1	2785	3790	locus110.1	625.21	+	2785	3790	0,0,255	1	1005	0
JAAILG010169968.1	2	743	locus629.1	567.5	+	2	743	0,0,255	1	741	0
JAAILG010025835.1	7080	8013	locus138.1	587.29	+	7080	8013	0,0,255	1	933	0
JAAILG010047614.1	3306	4197	locus291.1	531.82	+	3306	4197	0,0,255	1	891	0
JAAILG010076904.1	196	1096	locus396.1	643.25	+	196	1096	0,0,255	1	900	0
JAAILG010163260.1	2	815	locus613.1	579.76	-	2	815	0,0,255	1	813	0
JAAILG010050407.1	0	852	locus302.1	568.11	+	0	852	0,0,255	1	852	0
JAAILG010036036.1	3007	4064	locus226.1	604.91	-	3007	4064	0,0,255	2	882,24	0,1033
JAAILG010068946.1	474	1614	locus366.1	615.26	-	474	1614	0,0,255	1	1140	0
JAAILG010032669.1	1	676	locus209.1	515.88	+	1	676	0,0,255	1	675	0
JAAILG010013374.1	9399	10308	locus60.1	588.39	-	9399	10308	0,0,255	1	909	0
JAAILG010010345.1	9179	10082	locus48.1	622.76	+	9179	10082	0,0,255	1	903	0
JAAILG010036747.1	1425	2385	locus232.1	628.76	+	1425	2385	0,0,255	1	960	0
JAAILG010043639.1	3949	4756	locus263.1	550.51	-	3949	4756	0,0,255	1	807	0
JAAILG010030383.1	2	812	locus179.1	546.14	+	2	812	0,0,255	1	810	0

JAAILG010119096.1	1	946	locus519.1	562.42	+	1	946	0,0,255	1	945	0
JAAILG010126192.1	70	1072	locus539.1	586.77	-	70	1072	0,0,255	1	1002	0
JAAILG010058509.1	15	786	locus330.1	535.47	+	15	786	0,0,255	1	771	0
JAAILG010018604.1	329	1187	locus95.1	521.4	-	329	1187	0,0,255	1	858	0
JAAILG010077329.1	0	813	locus401.1	545.63	+	0	813	0,0,255	1	813	0
JAAILG010042405.1	110	1301	locus257.1	612.99	+	110	1301	0,0,255	3	24,830,37	0,156,1154
JAAILG010017782.1	7477	8284	locus83.1	645.51	-	7477	8284	0,0,255	1	807	0
JAAILG010007213.1	1158	2106	locus29.1	672.75	-	1158	2106	0,0,255	1	948	0
JAAILG010007213.1	8266	9169	locus30.1	527.08	-	8266	9169	0,0,255	1	903	0
JAAILG010007213.1	23226	24129	locus32.1	602.92	-	23226	24129	0,0,255	1	903	0
JAAILG010028109.1	8313	9108	locus163.1	607.02	+	8313	9108	0,0,255	1	795	0
JAAILG010007213.1	15424	16327	locus31.1	661.2	-	15424	16327	0,0,255	1	903	0
JAAILG010034634.1	0	720	locus218.1	628.84	-	0	720	0,0,255	1	720	0
JAAILG010006824.1	23636	24434	locus25.1	567.52	+	23636	24434	0,0,255	1	798	0
JAAILG010006824.1	9779	10721	locus24.1	653.62	+	9779	10721	0,0,255	1	942	0
JAAILG010022906.1	393	1185	locus115.1	667.73	-	393	1185	0,0,255	1	792	0
JAAILG010013651.1	13704	14709	locus64.1	663.87	+	13704	14709	0,0,255	1	1005	0
JAAILG010014301.1	11755	12670	locus66.1	597.55	-	11755	12670	0,0,255	1	915	0

Cloudy_catshark

BFAA01000307.1	373103	374000	locus7.1	742.33	+	373103	374000	0,0,255	1	897	0
BFAA01000307.1	381643	382540	locus8.1	744.04	-	381643	382540	0,0,255	1	897	0
BFAA01000307.1	420820	421711	locus10.1	732.32	-	420820	421711	0,0,255	1	891	0
BFAA01000307.1	502254	503061	locus12.1	660.24	-	502254	503061	0,0,255	1	807	0
BFAA01000307.1	575562	576369	locus14.1	661.03	-	575562	576369	0,0,255	1	807	0
BFAA01000307.1	612935	613727	locus15.1	646.05	-	612935	613727	0,0,255	1	792	0
BFAA01001691.1	123489	124302	locus39.1	676.81	-	123489	124302	0,0,255	1	813	0
BFAA01001691.1	152837	153758	locus40.1	769.15	-	152837	153758	0,0,255	1	921	0
BFAA01001691.1	185319	186114	locus41.1	661.49	+	185319	186114	0,0,255	1	795	0
BFAA01001691.1	206554	207274	locus42.1	590.43	+	206554	207274	0,0,255	1	720	0
BFAA01001691.1	250024	250924	locus43.1	748.43	+	250024	250924	0,0,255	1	900	0
BFAA01001691.1	267255	268047	locus44.1	657.03	-	267255	268047	0,0,255	1	792	0
BFAA01001691.1	311246	312044	locus45.1	662.84	-	311246	312044	0,0,255	1	798	0
BFAA01001691.1	352153	353116	locus47.1	798.92	-	352153	353116	0,0,255	1	963	0
BFAA01002101.1	33077	33890	locus53.1	670.41	+	33077	33890	0,0,255	1	813	0
BFAA01002101.1	87681	88575	locus54.1	740.75	+	87681	88575	0,0,255	1	894	0
BFAA01004758.1	192202	193105	locus65.1	745.41	+	192202	193105	0,0,255	1	903	0
BFAA01008259.1	16048	16942	locus76.1	746.63	-	16048	16942	0,0,255	1	894	0
BFAA01008259.1	53813	55076	locus77.1	1038.67	-	53813	55076	0,0,255	1	1263	0
BFAA01008259.1	83740	84511	locus78.1	631.29	-	83740	84511	0,0,255	1	771	0
BFAA01008733.1	38466	39354	locus79.1	714.45	+	38466	39354	0,0,255	1	888	0
BFAA01012745.1	7247	8267	locus87.1	820.73	-	7247	8267	0,0,255	1	1020	0
BFAA01013518.1	10111	10996	locus91.1	734.91	+	10111	10996	0,0,255	1	885	0

Great_white_shark

QUOW01000919.1	4438299	4439199	locus10.1	724.65	+	4438299	4439199	0,0,255	1	900	0
QUOW01000919.1	4483961	4484849	locus11.1	709.15	+	4483961	4484849	0,0,255	1	888	0
QUOW01001159.1	1331958	1332810	locus17.1	707.47	-	1331958	1332810	0,0,255	1	852	0
QUOW01001159.1	1833390	1834329	locus18.1	775	-	1833390	1834329	0,0,255	1	939	0
QUOW01001159.1	1869177	1870107	locus19.1	766.41	-	1869177	1870107	0,0,255	1	930	0
QUOW01001159.1	1899640	1900639	locus20.1	827.63	-	1899640	1900639	0,0,255	1	999	0
QUOW01001159.1	1959492	1960395	locus22.1	743.75	-	1959492	1960395	0,0,255	1	903	0
QUOW01001159.1	2812522	2813326	locus25.1	657.98	+	2812522	2813326	0,0,255	1	804	0
QUOW01001159.1	2927235	2928186	locus29.1	787.58	-	2927235	2928186	0,0,255	1	951	0
QUOW01001159.1	2956131	2957067	locus31.1	782.69	+	2956131	2957067	0,0,255	1	936	0
QUOW01001159.1	2971974	2972874	locus32.1	749.9	+	2971974	2972874	0,0,255	1	900	0
QUOW01001159.1	3010165	3010981	locus33.1	671.94	-	3010165	3010981	0,0,255	1	816	0
QUOW01001159.1	3042198	3043008	locus34.1	661.25	-	3042198	3043008	0,0,255	1	810	0
QUOW01001159.1	3094851	3095781	locus35.1	769.27	+	3094851	3095781	0,0,255	1	930	0

Whale_shark

LVEK02000002.1	86491	87304	locus2.1	674.97	-	86491	87304	0,0,255	1	813	0
LVEK02000776.1	6938	7745	locus9.1	652.74	-	6938	7745	0,0,255	1	807	0
LVEK02002323.1	78960	79698	locus20.1	482.54	-	78960	79698	0,0,255	1	738	0
LVEK02002323.1	131462	132272	locus21.1	663.66	+	131462	132272	0,0,255	1	810	0
LVEK02002323.1	148000	148876	locus22.1	716.51	+	148000	148876	0,0,255	1	876	0
LVEK02002323.1	168028	168928	locus23.1	745.34	-	168028	168928	0,0,255	1	900	0
LVEK02002323.1	193877	194678	locus24.1	665.7	-	193877	194678	0,0,255	1	801	0
LVEK02014336.1	34850	35867	locus36.1	815.4	-	34850	35867	0,0,255	1	1017	0
LVEK02019665.1	27856	28753	locus49.1	750.31	+	27856	28753	0,0,255	1	897	0
LVEK02029290.1	13908	14739	locus56.1	691.32	-	13908	14739	0,0,255	1	831	0
LVEK02029290.1	22329	23130	locus57.1	657.15	-	22329	23130	0,0,255	1	801	0
LVEK02029290.1	34843	35677	locus58.1	680.59	-	34843	35677	0,0,255	1	834	0
LVEK02029290.1	78214	79030	locus61.1	682.6	+	78214	79030	0,0,255	1	816	0
LVEK02029290.1	102240	103086	locus62.1	702.1	+	102240	103086	0,0,255	1	846	0
LVEK02029290.1	139969	140770	locus64.1	659.99	+	139969	140770	0,0,255	1	801	0
LVEK02029290.1	208421	209234	locus68.1	676.47	+	208421	209234	0,0,255	1	813	0
LVEK02029292.1	77897	78809	locus77.1	768.43	-	77897	78809	0,0,255	1	912	0
LVEK02029292.1	117038	117833	locus79.1	725.49	-	117038	117833	0,0,255	1	795	0
LVEK02042295.1	21726	22614	locus98.1	709.9	-	21726	22614	0,0,255	1	888	0
LVEK02047597.1	15668	16601	locus103.1	781.08	-	15668	16601	0,0,255	1	933	0
LVEK02047597.1	59769	60666	locus106.1	749.69	-	59769	60666	0,0,255	1	897	0
LVEK02047597.1	91482	92457	locus109.1	810.72	-	91482	92457	0,0,255	1	975	0

Brownbanded_bambooshark

ccg_chipu00000014	16653850	16654837	locus518.1	819.81	-	16653850	16654837	0,0,255	1	987	0
ccg_chipu00000014	17030173	17031091	locus519.1	748.83	+	17030173	17031091	0,0,255	1	918	0
ccg_chipu00000014	17087991	17088975	locus520.1	802.18	+	17087991	17088975	0,0,255	1	984	0
ccg_chipu00000066	9822515	9823544	locus1554.1	856.39	+	9822515	9823544	0,0,255	1	1029	0
ccg_chipu00000066	9842281	9843094	locus1555.1	675.68	-	9842281	9843094	0,0,255	1	813	0
ccg_chipu00000066	9914503	9915334	locus1560.1	689.82	-	9914503	9915334	0,0,255	1	831	0
ccg_chipu00000066	9931637	9932597	locus1562.1	800.74	-	9931637	9932597	0,0,255	1	960	0
ccg_chipu00000066	9955952	9956888	locus1564.1	777.09	-	9955952	9956888	0,0,255	1	936	0
ccg_chipu00000066	10051002	10051998	locus1570.1	827.6	-	10051002	10051998	0,0,255	1	996	0
ccg_chipu00000066	10099547	10100489	locus1573.1	786.17	-	10099547	10100489	0,0,255	1	942	0
ccg_chipu00000066	10132160	10132985	locus1576.1	696.93	+	10132160	10132985	0,0,255	1	825	0
ccg_chipu00000066	10197679	10198486	locus1583.1	787.25	+	10197679	10198486	0,0,255	1	807	0
ccg_chipu00000066	10234631	10235486	locus1585.1	740.89	+	10234631	10235486	0,0,255	1	855	0
ccg_chipu00000066	10280061	10280892	locus1588.1	805.45	+	10280061	10280892	0,0,255	1	831	0
ccg_chipu00000066	10373096	10374056	locus1595.1	798.17	-	10373096	10374056	0,0,255	1	960	0
ccg_chipu00000066	10405846	10406776	locus1597.1	782.16	-	10405846	10406776	0,0,255	1	930	0
ccg_chipu00000066	10445892	10446786	locus1599.1	736.24	+	10445892	10446786	0,0,255	1	894	0
ccg_chipu00000066	11225393	11226296	locus1601.1	741.09	-	11225393	11226296	0,0,255	1	903	0
ccg_chipu00000066	11279302	11280139	locus1603.1	688.28	+	11279302	11280139	0,0,255	1	837	0
ccg_chipu00000066	11367106	11368003	locus1611.1	749.49	+	11367106	11368003	0,0,255	1	897	0
ccg_chipu00000066	11407717	11408596	locus1614.1	736.64	+	11407717	11408596	0,0,255	1	879	0
ccg_chipu00000066	11886330	11887143	locus1619.1	673.85	+	11886330	11887143	0,0,255	1	813	0
ccg_chipu00000066	11916072	11916963	locus1620.1	743.73	+	11916072	11916963	0,0,255	1	891	0

ccg_chipu00000105	698011	698893	locus1998.1	727.07	-	698011	698893	0,0,255	1	882	0
ccg_chipu00000105	755174	756184	locus2000.1	630.26	-	755174	756184	0,0,255	2	773,40	0,970
ccg_chipu00000105	777718	778645	locus2001.1	757.73	-	777718	778645	0,0,255	1	927	0

Little_skate

AESE010035648.1	314	1205	locus4.1	733.91	-	314	1205	0,0,255	1	891	0
AESE010036109.1	70	988	locus5.1	740.6	+	70	988	0,0,255	1	918	0
AESE010044841.1	1830	2760	locus7.1	770.64	-	1830	2760	0,0,255	1	930	0
AESE010104795.1	31	967	locus14.1	776.12	-	31	967	0,0,255	1	936	0
AESE010131607.1	198	1011	locus16.1	664.49	+	198	1011	0,0,255	1	813	0
AESE011510678.1	449	1421	locus49.1	792.79	-	449	1421	0,0,255	1	972	0
AESE011557639.1	103	1003	locus52.1	756.07	-	103	1003	0,0,255	1	900	0
AESE011619611.1	1005	1899	locus54.1	735.42	+	1005	1899	0,0,255	1	894	0

Thorny_skate

scaffold_16	12510531	12511359	locus23.1	668.39	-	12510531	12511359	0,0,255	1	828	0
scaffold_16	12541614	12542532	locus24.1	741.46	-	12541614	12542532	0,0,255	1	918	0
scaffold_16	12736487	12737378	locus25.1	733.91	+	12736487	12737378	0,0,255	1	891	0
scaffold_16	34257392	34258286	locus26.1	735.65	-	34257392	34258286	0,0,255	1	894	0
scaffold_16	35960327	35961227	locus27.1	756.88	-	35960327	35961227	0,0,255	1	900	0
scaffold_16	35978605	35979535	locus28.1	773.02	-	35978605	35979535	0,0,255	1	930	0
scaffold_16	36517683	36518619	locus31.1	775.46	-	36517683	36518619	0,0,255	1	936	0
scaffold_16	36556572	36557385	locus32.1	663.82	+	36556572	36557385	0,0,255	1	813	0

Smalltooth_sawfish

CM019860.1	81599275	81600256	locus19.1	797.55	-	81599275	81600256	0,0,255	1	981	0
CM019860.1	81632010	81633054	locus20.1	848.17	-	81632010	81633054	0,0,255	1	1044	0
CM019860.1	81858781	81859684	locus21.1	741.72	+	81858781	81859684	0,0,255	1	903	0
CM019860.1	108428013	108428826	locus24.1	662.55	-	108428013	108428826	0,0,255	1	813	0
CM019860.1	108543764	108545009	locus31.1	1020.13	-	108543764	108545009	0,0,255	1	1245	0
CM019860.1	108592296	108593187	locus33.1	735.25	+	108592296	108593187	0,0,255	1	891	0
CM019860.1	108611520	108612660	locus34.1	871.47	+	108611520	108612660	0,0,255	1	1140	0
CM019860.1	108680533	108681346	locus36.1	670.28	+	108680533	108681346	0,0,255	1	813	0
CM019860.1	108687346	108688162	locus37.1	677.69	-	108687346	108688162	0,0,255	1	816	0
CM019860.1	108731703	108732516	locus38.1	668.05	+	108731703	108732516	0,0,255	1	813	0
CM019860.1	108749465	108750281	locus39.1	668.97	-	108749465	108750281	0,0,255	1	816	0
CM019860.1	109346601	109347492	locus41.1	740.71	+	109346601	109347492	0,0,255	1	891	0
CM019860.1	109360872	109361763	locus42.1	740.2	+	109360872	109361763	0,0,255	1	891	0
CM019860.1	109372416	109373307	locus43.1	741.74	+	109372416	109373307	0,0,255	1	891	0
CM019860.1	109387080	109387971	locus44.1	739.03	+	109387080	109387971	0,0,255	1	891	0
CM019860.1	109415317	109416235	locus46.1	744.7	+	109415317	109416235	0,0,255	1	918	0
CM019860.1	109432060	109432978	locus48.1	758.93	+	109432060	109432978	0,0,255	1	918	0
CM019860.1	109448836	109449754	locus49.1	756.19	+	109448836	109449754	0,0,255	1	918	0
CM019860.1	109470582	109471500	locus51.1	748.05	+	109470582	109471500	0,0,255	1	918	0
CM019860.1	109500707	109501625	locus53.1	745.54	+	109500707	109501625	0,0,255	1	918	0
CM019860.1	109533410	109534328	locus55.1	743.05	+	109533410	109534328	0,0,255	1	918	0
CM019860.1	109551998	109552916	locus57.1	757.54	+	109551998	109552916	0,0,255	1	918	0
CM019860.1	109574654	109575572	locus59.1	757.95	+	109574654	109575572	0,0,255	1	918	0

CM019860.1	109725444	109726473	locus61.1	855.7	+	109725444	109726473	0,0,255	1	1029	0

Reedfish

LR536433.1	117940254	117941241	locus27372.1	802.65	-	117940254	117941241	0,0,255	1	987	0
LR536433.1	118001930	118002821	locus27375.1	723.23	-	118001930	118002821	0,0,255	1	891	0
LR536433.1	118551605	118552418	locus27405.1	660.16	+	118551605	118552418	0,0,255	1	813	0
LR536433.1	118591654	118592458	locus27408.1	650.02	+	118591654	118592458	0,0,255	1	804	0
LR536433.1	118605859	118606663	locus27410.1	650.46	+	118605859	118606663	0,0,255	1	804	0
LR536433.1	118620442	118621252	locus27411.1	665.16	+	118620442	118621252	0,0,255	1	810	0
LR536433.1	118685126	118686113	locus27417.1	817.34	+	118685126	118686113	0,0,255	1	987	0
LR536433.1	118700162	118700975	locus27418.1	665.85	+	118700162	118700975	0,0,255	1	813	0
LR536433.1	118720451	118721261	locus27420.1	723.95	+	118720451	118721261	0,0,255	1	810	0
LR536433.1	118744336	118745149	locus27423.1	663.48	+	118744336	118745149	0,0,255	1	813	0
LR536433.1	118762868	118763681	locus27424.1	661.8	+	118762868	118763681	0,0,255	1	813	0
LR536433.1	118784814	118785627	locus27426.1	662.36	+	118784814	118785627	0,0,255	1	813	0
LR536433.1	118801657	118802581	locus27427.1	759.44	+	118801657	118802581	0,0,255	1	924	0
LR536433.1	118821122	118821935	locus27432.1	659.77	+	118821122	118821935	0,0,255	1	813	0
LR536433.1	118839755	118840565	locus27436.1	661.69	+	118839755	118840565	0,0,255	1	810	0
LR536433.1	118867951	118868761	locus27441.1	663.32	+	118867951	118868761	0,0,255	1	810	0
LR536433.1	118881454	118882264	locus27442.1	657.56	+	118881454	118882264	0,0,255	1	810	0
LR536433.1	118905319	118906129	locus27444.1	658.12	+	118905319	118906129	0,0,255	1	810	0
LR536433.1	118945412	118946225	locus27451.1	661.31	+	118945412	118946225	0,0,255	1	813	0
LR536433.1	118959787	118960597	locus27452.1	658.85	+	118959787	118960597	0,0,255	1	810	0
LR536433.1	118967678	118968605	locus27454.1	763.77	+	118967678	118968605	0,0,255	1	927	0
LR536433.1	118990787	118991597	locus27456.1	662.17	+	118990787	118991597	0,0,255	1	810	0
LR536433.1	119010827	119011709	locus27457.1	726.36	+	119010827	119011709	0,0,255	1	882	0

LR536433.1	119027494	119028301	locus27461.1	655.55	+	119027494	119028301	0,0,255	1	807	0
LR536433.1	119049701	119050508	locus27464.1	656.36	+	119049701	119050508	0,0,255	1	807	0
LR536433.1	119069853	119070648	locus27467.1	643.87	+	119069853	119070648	0,0,255	1	795	0
LR536433.1	119083036	119083831	locus27468.1	645.06	+	119083036	119083831	0,0,255	1	795	0
LR536433.1	119098554	119099349	locus27472.1	647.14	+	119098554	119099349	0,0,255	1	795	0
LR536433.1	119139581	119140376	locus27474.1	644.99	+	119139581	119140376	0,0,255	1	795	0
LR536433.1	119157263	119158073	locus27478.1	662.53	+	119157263	119158073	0,0,255	1	810	0
LR536433.1	119171291	119172122	locus27482.1	672.7	+	119171291	119172122	0,0,255	1	831	0
LR536433.1	119202381	119203224	locus27487.1	681.29	+	119202381	119203224	0,0,255	1	843	0
LR536433.1	119234880	119235732	locus27491.1	688.83	+	119234880	119235732	0,0,255	1	852	0
LR536433.1	119269856	119270696	locus27495.1	678.65	+	119269856	119270696	0,0,255	1	840	0
LR536433.1	119316358	119317153	locus27496.1	651.64	+	119316358	119317153	0,0,255	1	795	0
LR536433.1	119346971	119347766	locus27498.1	652.07	+	119346971	119347766	0,0,255	1	795	0
LR536433.1	119368834	119369629	locus27501.1	652.72	+	119368834	119369629	0,0,255	1	795	0
LR536433.1	119402263	119403070	locus27507.1	653.78	+	119402263	119403070	0,0,255	1	807	0
LR536433.1	119424287	119425100	locus27509.1	662.36	+	119424287	119425100	0,0,255	1	813	0
LR536433.1	119432190	119433153	locus27510.1	791.37	+	119432190	119433153	0,0,255	1	963	0
LR536433.1	119446425	119447271	locus27514.1	689.21	+	119446425	119447271	0,0,255	1	846	0
LR536433.1	119458643	119459453	locus27515.1	657.55	+	119458643	119459453	0,0,255	1	810	0
LR536433.1	119483502	119484429	locus27517.1	770.73	+	119483502	119484429	0,0,255	1	927	0
LR536433.1	119516598	119517399	locus27518.1	654.75	+	119516598	119517399	0,0,255	1	801	0
LR536433.1	119538141	119538936	locus27521.1	649.2	+	119538141	119538936	0,0,255	1	795	0
LR536433.1	119550328	119551123	locus27524.1	650.58	+	119550328	119551123	0,0,255	1	795	0
LR536433.1	119567728	119568661	locus27527.1	767.39	+	119567728	119568661	0,0,255	1	933	0

LR536433.1	119598801	119599605	locus27530.1	649.85	+	119598801	119599605	0,0,255	1	804	0
LR536433.1	119613792	119614785	locus27531.1	811.83	+	119613792	119614785	0,0,255	1	993	0
LR536433.1	119645834	119646641	locus27535.1	663.37	+	119645834	119646641	0,0,255	1	807	0
LR536433.1	119679292	119680087	locus27543.1	647.65	+	119679292	119680087	0,0,255	1	795	0
LR536433.1	121169062	121169962	locus27546.1	739.57	+	121169062	121169962	0,0,255	1	900	0
LR536433.1	121188689	121189685	locus27548.1	823.46	+	121188689	121189685	0,0,255	1	996	0
LR536433.1	121214242	121215055	locus27551.1	668.03	+	121214242	121215055	0,0,255	1	813	0
LR536433.1	121236519	121237419	locus27552.1	741.46	+	121236519	121237419	0,0,255	1	900	0
LR536433.1	121251987	121252929	locus27554.1	779.95	+	121251987	121252929	0,0,255	1	942	0
LR536433.1	121288013	121288817	locus27559.1	660.83	+	121288013	121288817	0,0,255	1	804	0
LR536433.1	121306580	121307537	locus27563.1	799.03	+	121306580	121307537	0,0,255	1	957	0
LR536433.1	121331732	121332695	locus27565.1	794.85	+	121331732	121332695	0,0,255	1	963	0
LR536433.1	121351273	121352083	locus27567.1	663.72	+	121351273	121352083	0,0,255	1	810	0
LR536433.1	121364526	121365339	locus27570.1	669.44	+	121364526	121365339	0,0,255	1	813	0
LR536433.1	121378187	121379027	locus27573.1	693.75	+	121378187	121379027	0,0,255	1	840	0
LR536433.1	121391279	121392179	locus27575.1	748.18	+	121391279	121392179	0,0,255	1	900	0
LR536433.1	121404061	121404865	locus27576.1	666.01	+	121404061	121404865	0,0,255	1	804	0
LR536433.1	121426923	121427817	locus27579.1	742.36	+	121426923	121427817	0,0,255	1	894	0
LR536433.1	121444619	121445417	locus27584.1	657.72	+	121444619	121445417	0,0,255	1	798	0
LR536433.1	121464392	121465190	locus27585.1	658.73	+	121464392	121465190	0,0,255	1	798	0
LR536433.1	121482148	121482946	locus27586.1	657.99	+	121482148	121482946	0,0,255	1	798	0
LR536433.1	121495258	121496071	locus27587.1	663.66	+	121495258	121496071	0,0,255	1	813	0
LR536433.1	121516051	121516855	locus27591.1	662.66	+	121516051	121516855	0,0,255	1	804	0
LR536433.1	121580539	121581478	locus27596.1	775.84	+	121580539	121581478	0,0,255	1	939	0

LR536433.1	121604253	121605189	locus27597.1	781.36	+	121604253	121605189	0,0,255	1	936	0
LR536433.1	121614609	121615569	locus27599.1	793.08	+	121614609	121615569	0,0,255	1	960	0
LR536433.1	121632443	121633463	locus27603.1	850.84	+	121632443	121633463	0,0,255	1	1020	0
LR536433.1	121676535	121677516	locus27607.1	808.04	+	121676535	121677516	0,0,255	1	981	0
LR536433.1	121698722	121699658	locus27608.1	780.08	+	121698722	121699658	0,0,255	1	936	0
LR536433.1	121736726	121737539	locus27613.1	666.68	+	121736726	121737539	0,0,255	1	813	0
LR536433.1	121755062	121755998	locus27617.1	773.95	+	121755062	121755998	0,0,255	1	936	0
LR536433.1	121787932	121788844	locus27623.1	756.86	+	121787932	121788844	0,0,255	1	912	0
LR536433.1	121809990	121810818	locus27626.1	774.88	+	121809990	121810818	0,0,255	1	828	0
LR536433.1	121822990	121823791	locus27629.1	655.34	+	121822990	121823791	0,0,255	1	801	0
LR536433.1	121849857	121850664	locus27632.1	657.83	+	121849857	121850664	0,0,255	1	807	0
LR536433.1	121858788	121859616	locus27634.1	679.11	+	121858788	121859616	0,0,255	1	828	0
LR536433.1	121882597	121883425	locus27636.1	680.32	+	121882597	121883425	0,0,255	1	828	0
LR536433.1	121897316	121898159	locus27637.1	685.02	+	121897316	121898159	0,0,255	1	843	0
LR536433.1	121924644	121925544	locus27638.1	733.31	+	121924644	121925544	0,0,255	1	900	0
LR536433.1	121947435	121948335	locus27640.1	732.58	+	121947435	121948335	0,0,255	1	900	0
LR536433.1	121955985	121956885	locus27641.1	736.85	+	121955985	121956885	0,0,255	1	900	0
LR536433.1	121981821	121982721	locus27646.1	736.85	+	121981821	121982721	0,0,255	1	900	0
LR536433.1	122003860	122004799	locus27647.1	773.45	+	122003860	122004799	0,0,255	1	939	0
LR536433.1	122041492	122042392	locus27649.1	737.24	+	122041492	122042392	0,0,255	1	900	0
LR536433.1	122056758	122057670	locus27651.1	744.52	+	122056758	122057670	0,0,255	1	912	0
LR536433.1	122070589	122071402	locus27654.1	709.66	+	122070589	122071402	0,0,255	1	813	0
LR536433.1	122089569	122090367	locus27657.1	680.2	+	122089569	122090367	0,0,255	1	798	0
LR536433.1	122103522	122104422	locus27659.1	740.95	+	122103522	122104422	0,0,255	1	900	0

LR536433.1	122487544	122488432	locus27661.1	734.27	+	122487544	122488432	0,0,255	1	888	0
LR536433.1	122507488	122508382	locus27663.1	733.79	+	122507488	122508382	0,0,255	1	894	0
LR536433.1	122520763	122521696	locus27665.1	771.93	+	122520763	122521696	0,0,255	1	933	0
LR536433.1	122551304	122552099	locus27670.1	657.82	+	122551304	122552099	0,0,255	1	795	0
LR536433.1	122564421	122565399	locus27671.1	807.14	+	122564421	122565399	0,0,255	1	978	0
LR536433.1	122587097	122588006	locus27677.1	752.71	+	122587097	122588006	0,0,255	1	909	0
LR536433.1	122614338	122615151	locus27679.1	665.32	+	122614338	122615151	0,0,255	1	813	0
LR536433.1	122633517	122634312	locus27683.1	653.68	+	122633517	122634312	0,0,255	1	795	0
LR536433.1	122642200	122643100	locus27685.1	737.8	+	122642200	122643100	0,0,255	1	900	0
LR536433.1	122667448	122668348	locus27689.1	739	+	122667448	122668348	0,0,255	1	900	0
LR536433.1	122693582	122694506	locus27690.1	768.94	+	122693582	122694506	0,0,255	1	924	0
LR536433.1	122738904	122739702	locus27693.1	658.15	+	122738904	122739702	0,0,255	1	798	0
LR536433.1	122753828	122754632	locus27697.1	664.1	+	122753828	122754632	0,0,255	1	804	0
LR536433.1	122764068	122764869	locus27699.1	665.39	+	122764068	122764869	0,0,255	1	801	0
LR536433.1	122776725	122777529	locus27703.1	663.3	+	122776725	122777529	0,0,255	1	804	0
LR536433.1	122815883	122816684	locus27707.1	662.53	+	122815883	122816684	0,0,255	1	801	0
LR536433.1	122832262	122833054	locus27711.1	649.32	+	122832262	122833054	0,0,255	1	792	0
LR536433.1	122853324	122854200	locus27713.1	726.74	+	122853324	122854200	0,0,255	1	876	0
LR536433.1	122879352	122880165	locus27719.1	669.35	+	122879352	122880165	0,0,255	1	813	0
LR536433.1	122924618	122925533	locus27724.1	763.93	+	122924618	122925533	0,0,255	1	915	0
LR536433.1	122932964	122933768	locus27727.1	661.2	-	122932964	122933768	0,0,255	1	804	0
LR536434.1	273369089	273370001	locus56889.1	750.66	+	273369089	273370001	0,0,255	1	912	0
LR536434.1	274753008	274753857	locus56973.1	694.41	-	274753008	274753857	0,0,255	1	849	0
LR536434.1	274825717	274826566	locus56974.1	687.69	-	274825717	274826566	0,0,255	1	849	0

CAADRL0100015[illegible]	21326	22148	locus40330.1	668.59	+	21326	22148	0,0,255	1	822	0
CAADRL0100015[illegible]	41157	41967	locus40334.1	658.34	+	41157	41967	0,0,255	1	810	0
CAADRL0100015[illegible]	61215	62148	locus40336.1	768.37	+	61215	62148	0,0,255	1	933	0
CAADRL0100015[illegible]	85917	86850	locus40339.1	730.28	+	85917	86850	0,0,255	1	933	0
CAADRL0100015[illegible]	114892	115825	locus40342.1	767.98	+	114892	115825	0,0,255	1	933	0
CAADRL0100015[illegible]	140049	140982	locus40344.1	728.28	+	140049	140982	0,0,255	1	933	0
CAADRL0100015[illegible]	160432	161359	locus40347.1	763.53	+	160432	161359	0,0,255	1	927	0
CAADRL0100015[illegible]	176758	177763	locus40349.1	832.76	+	176758	177763	0,0,255	1	1005	0
CAADRL0100015[illegible]	190866	191796	locus40350.1	765.59	+	190866	191796	0,0,255	1	930	0
CAADRL0100015[illegible]	231602	232571	locus40354.1	792.46	+	231602	232571	0,0,255	1	969	0
CAADRL0100015[illegible]	339549	340359	locus40363.1	755.24	+	339549	340359	0,0,255	1	810	0
CAADRL0100015[illegible]	361336	362149	locus40365.1	659.01	+	361336	362149	0,0,255	1	813	0
CAADRL0100015[illegible]	373831	374734	locus40368.1	740.41	+	373831	374734	0,0,255	1	903	0
CAADRL0100015[illegible]	393159	393957	locus40371.1	697.9	+	393159	393957	0,0,255	1	798	0
CAADRL0100015[illegible]	419653	420463	locus40372.1	662.06	+	419653	420463	0,0,255	1	810	0
CAADRL0100015[illegible]	450271	451066	locus40375.1	650.21	+	450271	451066	0,0,255	1	795	0
CAADRL0100015[illegible]	469155	469980	locus40378.1	668.29	-	469155	469980	0,0,255	1	825	0
CAADRL0100015[illegible]	494094	494916	locus40381.1	665.33	-	494094	494916	0,0,255	1	822	0
CAADRL0100015[illegible]	500715	501561	locus40382.1	684.92	-	500715	501561	0,0,255	1	846	0
CAADRL0100015[illegible]	525833	526649	locus40383.1	664.12	+	525833	526649	0,0,255	1	816	0
CAADRL0100015[illegible]	545218	546088	locus40385.1	714.39	-	545218	546088	0,0,255	1	870	0
CAADRL0100015[illegible]	586244	587174	locus40391.1	774.42	-	586244	587174	0,0,255	1	930	0
CAADRL0100015[illegible]	599660	600560	locus40393.1	750.52	-	599660	600560	0,0,255	1	900	0
CAADRL0100015[illegible]	656664	657576	locus40403.1	766.49	-	656664	657576	0,0,255	1	912	0

CAADRL0100015:	698980	699886	locus40406.1	744.31	+	698980	699886	0,0,255	1	906	0
CAADRL0100015:	718441	719266	locus40408.1	681.08	+	718441	719266	0,0,255	1	825	0
CAADRL0100015:	725661	726477	locus40409.1	662.85	-	725661	726477	0,0,255	1	816	0
CAADRL0100015:	782053	782962	locus40415.1	757.66	+	782053	782962	0,0,255	1	909	0
CAADRL0100015:	808493	809303	locus40417.1	669.33	+	808493	809303	0,0,255	1	810	0
CAADRL0100015:	819983	820796	locus40419.1	669.12	+	819983	820796	0,0,255	1	813	0
CAADRL0100015:	828710	829511	locus40420.1	667.6	+	828710	829511	0,0,255	1	801	0
CAADRL0100015:	845794	846601	locus40424.1	662.49	+	845794	846601	0,0,255	1	807	0
CAADRL0100015:	860789	861602	locus40427.1	662.62	+	860789	861602	0,0,255	1	813	0
CAADRL0100015:	883318	884131	locus40429.1	672.36	+	883318	884131	0,0,255	1	813	0
CAADRL0100015:	914059	914866	locus40433.1	664.85	+	914059	914866	0,0,255	1	807	0
CAADRL0100015:	941278	942091	locus40435.1	671.79	+	941278	942091	0,0,255	1	813	0
CAADRL0100015:	962312	963311	locus40436.1	827.13	+	962312	963311	0,0,255	1	999	0
CAADRL0100015:	998814	999714	locus40437.1	741.02	+	998814	999714	0,0,255	1	900	0
CAADRL0100015:	1076756	1077656	locus40443.1	740.82	+	1076756	1077656	0,0,255	1	900	0
CAADRL0100015:	1106686	1107649	locus40448.1	800.5	+	1106686	1107649	0,0,255	1	963	0
CAADRL0100015:	1125519	1126458	locus40451.1	774.11	+	1125519	1126458	0,0,255	1	939	0
CAADRL0100015:	1147897	1148710	locus40453.1	665.01	-	1147897	1148710	0,0,255	1	813	0
CAADRL0100015:	1195602	1196541	locus40458.1	727.49	+	1195602	1196541	0,0,255	1	939	0
CAADRL0100015:	1220414	1221314	locus40460.1	739.44	+	1220414	1221314	0,0,255	1	900	0
CAADRL0100015:	1237200	1238139	locus40462.1	778.97	+	1237200	1238139	0,0,255	1	939	0
CAADRL0100015:	1268277	1269237	locus40464.1	792.43	+	1268277	1269237	0,0,255	1	960	0
CAADRL0100015:	1313230	1314025	locus40468.1	701.98	+	1313230	1314025	0,0,255	1	795	0
CAADRL0100015:	1330491	1331430	locus40470.1	778.48	+	1330491	1331430	0,0,255	1	939	0

CAADRL0100015[illegible]	1354663	1355476	locus40471.1	663.6	-	1354663	1355476	0,0,255	1	813	0
CAADRL0100015[illegible]	1387471	1388284	locus40475.1	668.12	-	1387471	1388284	0,0,255	1	813	0
CAADRL0100015[illegible]	1403119	1403932	locus40478.1	667.11	-	1403119	1403932	0,0,255	1	813	0
CAADRL0100015[illegible]	1421907	1422837	locus40479.1	765.53	-	1421907	1422837	0,0,255	1	930	0
CAADRL0100015[illegible]	1436454	1437402	locus40481.1	777.6	-	1436454	1437402	0,0,255	1	948	0
CAADRL0100015[illegible]	1452346	1453153	locus40483.1	709.35	-	1452346	1453153	0,0,255	1	807	0
CAADRL0100015[illegible]	1461893	1462784	locus40486.1	733.64	-	1461893	1462784	0,0,255	1	891	0
CAADRL0100015[illegible]	1500626	1501442	locus40490.1	669.9	-	1500626	1501442	0,0,255	1	816	0
CAADRL0100015[illegible]	1520255	1521053	locus40492.1	653.88	-	1520255	1521053	0,0,255	1	798	0
CAADRL0100015[illegible]	1537080	1538124	locus40497.1	871.1	-	1537080	1538124	0,0,255	1	1044	0
CAADRL0100015[illegible]	1554762	1555557	locus40499.1	722.73	-	1554762	1555557	0,0,255	1	795	0
CAADRL0100015[illegible]	1567042	1567837	locus40501.1	723.17	-	1567042	1567837	0,0,255	1	795	0
CAADRL0100015[illegible]	1592090	1592885	locus40503.1	714.17	-	1592090	1592885	0,0,255	1	795	0
CAADRL0100015[illegible]	1614762	1615692	locus40506.1	768.64	-	1614762	1615692	0,0,255	1	930	0
CAADRL0100015[illegible]	1631155	1631950	locus40509.1	705.69	-	1631155	1631950	0,0,255	1	795	0
CAADRL0100015[illegible]	1647576	1648380	locus40513.1	661.85	-	1647576	1648380	0,0,255	1	804	0
CAADRL0100015[illegible]	1667137	1668076	locus40514.1	771.35	-	1667137	1668076	0,0,255	1	939	0
CAADRL0100015[illegible]	2423742	2424705	locus40515.1	794.85	-	2423742	2424705	0,0,255	1	963	0
CAADRL0100015[illegible]	2449079	2450036	locus40517.1	799.19	-	2449079	2450036	0,0,255	1	957	0
CAADRL0100015[illegible]	2478025	2478967	locus40520.1	780.09	-	2478025	2478967	0,0,255	1	942	0
CAADRL0100015[illegible]	2490512	2491412	locus40523.1	742.43	-	2490512	2491412	0,0,255	1	900	0

Sterlet

CM021261.1	5650468	5651500	locus180.1	599.3	-	5650468	5651500	0,0,255	1	1032	0
CM021261.1	5775258	5776053	locus182.1	606.8	-	5775258	5776053	0,0,255	1	795	0
CM021261.1	5784168	5785152	locus183.1	607.3	-	5784168	5785152	0,0,255	1	984	0
CM021261.1	5798180	5798975	locus184.1	604.8	+	5798180	5798975	0,0,255	1	795	0
CM021261.1	5806225	5807020	locus185.1	599.8	+	5806225	5807020	0,0,255	1	795	0
CM021261.1	5815076	5815871	locus186.1	594.8	+	5815076	5815871	0,0,255	1	795	0
CM021261.1	5824945	5825869	locus187.1	576.1	+	5824945	5825869	0,0,255	1	924	0
CM021261.1	5837160	5838120	locus188.1	604.8	-	5837160	5838120	0,0,255	1	960	0
CM021261.1	5847503	5848298	locus189.1	606.3	-	5847503	5848298	0,0,255	1	795	0
CM021261.1	5881419	5882241	locus191.1	605.3	+	5881419	5882241	0,0,255	1	822	0
CM021261.1	5897511	5898450	locus192.1	589.3	+	5897511	5898450	0,0,255	1	939	0
CM021261.1	5907782	5908682	locus193.1	579.8	+	5907782	5908682	0,0,255	1	900	0
CM021261.1	5920448	5921348	locus194.1	585.8	+	5920448	5921348	0,0,255	1	900	0
CM021261.1	5934387	5935287	locus195.1	587.3	+	5934387	5935287	0,0,255	1	900	0
CM021261.1	5944272	5945172	locus196.1	591.8	+	5944272	5945172	0,0,255	1	900	0
CM021261.1	5952262	5953075	locus197.1	586.3	+	5952262	5953075	0,0,255	1	813	0
CM021261.1	5961166	5962066	locus199.1	592.3	+	5961166	5962066	0,0,255	1	900	0
CM021261.1	6067895	6068774	locus200.1	603.3	+	6067895	6068774	0,0,255	1	879	0
CM021261.1	6087488	6088439	locus201.1	590.8	+	6087488	6088439	0,0,255	1	951	0
CM021261.1	6097731	6098544	locus202.1	591.3	+	6097731	6098544	0,0,255	1	813	0
CM021261.1	6110866	6111679	locus203.1	602.3	+	6110866	6111679	0,0,255	1	813	0
CM021261.1	6125954	6126881	locus204.1	600.3	+	6125954	6126881	0,0,255	1	927	0
CM021261.1	6136253	6137141	locus205.1	594.8	+	6136253	6137141	0,0,255	1	888	0

CM021261.1	6145025	6145913	locus206.1	596.8	+	6145025	6145913	0,0,255	1	888	0
CM021261.1	6159344	6160211	locus208.1	604.2	+	6159344	6160211	0,0,255	1	867	0
CM021261.1	6166453	6167362	locus209.1	576.4	-	6166453	6167362	0,0,255	1	909	0
CM021268.1	27942084	27942996	locus276.1	568.9	+	27942084	27942996	0,0,255	1	912	0
CM021268.1	27945509	27946400	locus277.1	529	-	27945509	27946400	0,0,255	1	891	0
CM021268.1	27958905	27959805	locus278.1	531.6	-	27958905	27959805	0,0,255	1	900	0
CM021268.1	27967605	27968418	locus280.1	590.3	-	27967605	27968418	0,0,255	1	813	0
CM021268.1	27995822	27996764	locus281.1	597.3	-	27995822	27996764	0,0,255	1	942	0
CM021268.1	28012722	28013535	locus282.1	594.3	-	28012722	28013535	0,0,255	1	813	0
CM021268.1	28024304	28025183	locus283.1	598.3	-	28024304	28025183	0,0,255	1	879	0
CM021268.1	28042484	28043453	locus284.1	586.3	-	28042484	28043453	0,0,255	1	969	0
CM021268.1	28069288	28070188	locus285.1	578.3	-	28069288	28070188	0,0,255	1	900	0
CM021268.1	28093726	28094536	locus286.1	572.7	-	28093726	28094536	0,0,255	1	810	0
CM021268.1	28100654	28101560	locus287.1	601.8	-	28100654	28101560	0,0,255	1	906	0
CM021268.1	28118261	28119077	locus288.1	606.9	+	28118261	28119077	0,0,255	1	816	0
CM021268.1	28135289	28136084	locus289.1	601.8	+	28135289	28136084	0,0,255	1	795	0
CM021268.1	28141557	28142364	locus290.1	588.3	+	28141557	28142364	0,0,255	1	807	0
CM021268.1	28149043	28149859	locus292.1	559.3	-	28149043	28149859	0,0,255	1	816	0
CM021268.1	28160236	28161043	locus293.1	572.4	+	28160236	28161043	0,0,255	1	807	0
CM021268.1	28260625	28261522	locus295.1	544.7	+	28260625	28261522	0,0,255	1	897	0
CM021302.1	1738706	1740130	locus410.1	438.8	+	1738706	1740130	0,0,255	2	333,435	0,989
CM021306.1	1933890	1934907	locus416.1	496.1	+	1933890	1934907	0,0,255	1	1017	0
VTUV010003	79217	80234	locus438.1	495.6	-	79217	80234	0,0,255	1	1017	0

Spotted_gar

LG14	3737208	3738021	locus100.1	664.65	-	3737208	3738021	0,0,255	1	813	0
LG14	3760919	3761888	locus105.1	807.65	-	3760919	3761888	0,0,255	1	969	0
LG14	3782446	3783259	locus110.1	671.69	-	3782446	3783259	0,0,255	1	813	0
LG14	3796418	3797348	locus113.1	770.65	-	3796418	3797348	0,0,255	1	930	0
LG14	3831636	3893202	locus120.1	635.44	-	3831636	3893202	0,0,255	2	354,456	0,61110
LG14	3869152	3870106	locus128.1	793.12	-	3869152	3870106	0,0,255	1	954	0
LG14	3879025	3879829	locus130.1	662.39	-	3879025	3879829	0,0,255	1	804	0
LG14	3914055	3915003	locus135.1	787.93	-	3914055	3915003	0,0,255	1	948	0
LG14	3944736	3945552	locus137.1	661.82	+	3944736	3945552	0,0,255	1	816	0
LG14	3957616	3958456	locus140.1	689.85	-	3957616	3958456	0,0,255	1	840	0
LG14	4014355	4015309	locus143.1	798.36	+	4014355	4015309	0,0,255	1	954	0
LG14	4033923	4034787	locus146.1	697.44	-	4033923	4034787	0,0,255	1	864	0
LG14	4047446	4048346	locus147.1	733.65	+	4047446	4048346	0,0,255	1	900	0
LG14	4055702	4056599	locus148.1	736.46	+	4055702	4056599	0,0,255	1	897	0
LG14	4073886	4074819	locus150.1	769	+	4073886	4074819	0,0,255	1	933	0
LG14	4089462	4090362	locus152.1	734.09	+	4089462	4090362	0,0,255	1	900	0
LG14	4111372	4112299	locus154.1	760.8	+	4111372	4112299	0,0,255	1	927	0
LG14	4131658	4168717	locus156.1	732.29	-	4131658	4168717	0,0,255	2	691,245	0,36814
LG14	4184445	4185375	locus161.1	760.34	-	4184445	4185375	0,0,255	1	930	0
LG14	4202753	4203653	locus163.1	737.93	-	4202753	4203653	0,0,255	1	900	0
LG14	4213897	4214782	locus166.1	726.91	+	4213897	4214782	0,0,255	1	885	0
LG14	4216999	4217803	locus167.1	658.36	-	4216999	4217803	0,0,255	1	804	0
LG14	4332432	4333272	locus168.1	674.51	+	4332432	4333272	0,0,255	1	840	0

LG14	3196387	3197329	locus28.1	794.09	-	3196387	3197329	0,0,255	1	942	0
LG14	3367873	3368686	locus42.1	640.02	-	3367873	3368686	0,0,255	1	813	0
LG14	3374600	3375413	locus43.1	663.01	-	3374600	3375413	0,0,255	1	813	0
LG14	3392201	3393014	locus48.1	667.88	-	3392201	3393014	0,0,255	1	813	0
LG14	3416727	3417717	locus52.1	827.37	-	3416727	3417717	0,0,255	1	990	0
LG14	3434304	3435237	locus55.1	654.94	-	3434304	3435237	0,0,255	1	933	0
LG14	3463431	3464235	locus62.1	670.37	-	3463431	3464235	0,0,255	1	804	0
LG14	3512907	3513720	locus66.1	674.27	-	3512907	3513720	0,0,255	1	813	0
LG14	3532059	3532872	locus69.1	666.19	-	3532059	3532872	0,0,255	1	813	0
LG14	3538497	3539295	locus70.1	661.27	-	3538497	3539295	0,0,255	1	798	0
LG14	3545231	3546047	locus71.1	670.87	-	3545231	3546047	0,0,255	1	816	0
LG14	3551595	3552408	locus73.1	668.69	-	3551595	3552408	0,0,255	1	813	0
LG14	3557557	3558370	locus74.1	668.17	-	3557557	3558370	0,0,255	1	813	0
LG14	3573181	3573994	locus76.1	667.94	-	3573181	3573994	0,0,255	1	813	0
LG14	3581356	3582301	locus77.1	781.49	-	3581356	3582301	0,0,255	1	945	0
LG14	3591299	3592112	locus78.1	667.75	-	3591299	3592112	0,0,255	1	813	0
LG14	3612283	3613096	locus81.1	675.4	-	3612283	3613096	0,0,255	1	813	0
LG14	3621680	3622478	locus83.1	661.79	-	3621680	3622478	0,0,255	1	798	0
LG14	3646917	3647766	locus87.1	700	-	3646917	3647766	0,0,255	1	849	0
LG14	3657134	3657947	locus88.1	673.84	-	3657134	3657947	0,0,255	1	813	0
LG14	3668870	3669683	locus89.1	770.42	-	3668870	3669683	0,0,255	1	813	0
LG14	3676900	3677860	locus90.1	796.81	-	3676900	3677860	0,0,255	1	960	0
LG14	3692596	3693415	locus92.1	673.31	-	3692596	3693415	0,0,255	1	819	0
LG14	3701843	3702656	locus94.1	671.97	-	3701843	3702656	0,0,255	1	813	0

LG14	3720159	3721134	locus96.1	815.51	-	3720159	3721134	0,0,255	1	975	0
LG14	4343220	4344249	locus169.1	833.96	+	4343220	4344249	0,0,255	1	1029	0

Japanese_eel

BEWY01000013.1	25314840	25315641	locus82.1	661.57	-	25314840	25315641	0,0,255	1	801	0
BEWY01000017.1	8079321	8080224	locus99.1	752.6	-	8079321	8080224	0,0,255	1	903	0
BEWY01000017.1	8086131	8087052	locus101.1	769.19	-	8086131	8087052	0,0,255	1	921	0
BEWY01000017.1	8107616	8108507	locus106.1	742.58	-	8107616	8108507	0,0,255	1	891	0
BEWY01000017.1	8114233	8115136	locus108.1	751.98	-	8114233	8115136	0,0,255	1	903	0
BEWY01000017.1	8121556	8122483	locus110.1	708.44	-	8121556	8122483	0,0,255	1	927	0
BEWY01000017.1	8135913	8136714	locus113.1	658.81	-	8135913	8136714	0,0,255	1	801	0
BEWY01000017.1	8150012	8150819	locus115.1	663.22	-	8150012	8150819	0,0,255	1	807	0
BEWY01000017.1	8229593	8230400	locus123.1	664.72	-	8229593	8230400	0,0,255	1	807	0
BEWY01000017.1	8261792	8262599	locus128.1	663.5	-	8261792	8262599	0,0,255	1	807	0
BEWY01000017.1	8309997	8310798	locus138.1	658.32	+	8309997	8310798	0,0,255	1	801	0
BEWY01000017.1	8319418	8320219	locus140.1	658.96	+	8319418	8320219	0,0,255	1	801	0
BEWY01000017.1	8326044	8326857	locus141.1	665.03	-	8326044	8326857	0,0,255	1	813	0
BEWY01000017.1	8335094	8335907	locus142.1	667.19	-	8335094	8335907	0,0,255	1	813	0
BEWY01000017.1	8345255	8346155	locus143.1	746.84	-	8345255	8346155	0,0,255	1	900	0
BEWY01000017.1	8353784	8354492	locus145.1	588.04	-	8353784	8354492	0,0,255	1	708	0
BEWY01000017.1	8379800	8380607	locus149.1	663.2	-	8379800	8380607	0,0,255	1	807	0
BEWY01000017.1	8392702	8393656	locus151.1	789.97	-	8392702	8393656	0,0,255	1	954	0
BEWY01000017.1	8401849	8402785	locus153.1	776.6	-	8401849	8402785	0,0,255	1	936	0
BEWY01000017.1	8411598	8412411	locus155.1	727.13	-	8411598	8412411	0,0,255	1	813	0
BEWY01000017.1	8431168	8431981	locus158.1	669.19	-	8431168	8431981	0,0,255	1	813	0
BEWY01000017.1	8436739	8437540	locus159.1	659.12	-	8436739	8437540	0,0,255	1	801	0
BEWY01000017.1	8441411	8442224	locus160.1	774.58	-	8441411	8442224	0,0,255	1	813	0

BEWY01000017.1	8456849	8457866	locus162.1	846.09	-	8456849	8457866	0,0,255	1	1017	0
BEWY01000017.1	8464218	8465205	locus165.1	816.91	-	8464218	8465205	0,0,255	1	987	0
BEWY01000017.1	8472235	8473201	locus166.1	800.98	-	8472235	8473201	0,0,255	1	966	0
BEWY01000017.1	8532832	8533621	locus175.1	761.01	-	8532832	8533621	0,0,255	1	789	0
BEWY01000017.1	8570902	8571793	locus182.1	738.61	-	8570902	8571793	0,0,255	1	891	0
BEWY01000017.1	8579213	8580026	locus184.1	719.9	-	8579213	8580026	0,0,255	1	813	0
BEWY01000017.1	8591650	8592541	locus186.1	740.82	-	8591650	8592541	0,0,255	1	891	0
BEWY01000017.1	8636939	8637752	locus189.1	734.53	-	8636939	8637752	0,0,255	1	813	0
BEWY01000017.1	8647986	8648877	locus191.1	739.88	-	8647986	8648877	0,0,255	1	891	0
BEWY01000017.1	8672305	8673103	locus193.1	656.44	-	8672305	8673103	0,0,255	1	798	0
BEWY01000017.1	8697135	8697951	locus197.1	761.88	-	8697135	8697951	0,0,255	1	816	0
BEWY01000017.1	8713899	8714712	locus199.1	734.03	-	8713899	8714712	0,0,255	1	813	0
BEWY01000017.1	8734269	8735160	locus202.1	738.1	-	8734269	8735160	0,0,255	1	891	0
BEWY01000105.1	99827	100586	locus217.1	637.25	+	99827	100586	0,0,255	1	759	0
BEWY01000105.1	105898	106801	locus219.1	752.91	+	105898	106801	0,0,255	1	903	0
BEWY01000105.1	121675	122578	locus223.1	751.99	+	121675	122578	0,0,255	1	903	0
BEWY01000105.1	137524	138427	locus227.1	753.14	+	137524	138427	0,0,255	1	903	0
BEWY01000105.1	173486	174389	locus234.1	752.52	+	173486	174389	0,0,255	1	903	0
BEWY01000105.1	186258	187185	locus236.1	717.5	+	186258	187185	0,0,255	1	927	0
BEWY01000105.1	196767	197706	locus238.1	790.11	+	196767	197706	0,0,255	1	939	0
BEWY01000105.1	262761	263763	locus247.1	838.51	+	262761	263763	0,0,255	1	1002	0
BEWY01000105.1	298316	299300	locus250.1	828.08	+	298316	299300	0,0,255	1	984	0
BEWY01000105.1	353454	354261	locus256.1	665.06	+	353454	354261	0,0,255	1	807	0
BEWY01000105.1	358286	359243	locus257.1	783.98	-	358286	359243	0,0,255	1	957	0

BEWY01000105.1	372732	373632	locus260.1	734.34	+	372732	373632	0,0,255	1	900	0
BEWY01000105.1	385228	386128	locus262.1	732.38	+	385228	386128	0,0,255	1	900	0
BEWY01000105.1	389037	389760	locus263.1	592.07	-	389037	389760	0,0,255	1	723	0
BEWY01000105.1	398950	399748	locus266.1	646.44	-	398950	399748	0,0,255	1	798	0
BEWY01000105.1	410820	411720	locus270.1	732.18	+	410820	411720	0,0,255	1	900	0
BEWY01000105.1	420781	421681	locus272.1	734.6	+	420781	421681	0,0,255	1	900	0
BEWY01000105.1	429555	430455	locus274.1	735.18	+	429555	430455	0,0,255	1	900	0
BEWY01000105.1	431969	432767	locus275.1	649.37	-	431969	432767	0,0,255	1	798	0
BEWY01000105.1	542413	543406	locus277.1	786.49	+	542413	543406	0,0,255	1	993	0
BEWY01004646.1	5610	6552	locus298.1	781.42	-	5610	6552	0,0,255	1	942	0
BEWY01074007.1	6472	7264	locus360.1	728.72	-	6472	7264	0,0,255	1	792	0
BEWY01083235.1	13034	13826	locus380.1	729.74	+	13034	13826	0,0,255	1	792	0
BEWY01083235.1	37898	38708	locus384.1	656.34	-	37898	38708	0,0,255	1	810	0
BEWY01083235.1	123662	124628	locus394.1	788.26	+	123662	124628	0,0,255	1	966	0
BEWY01083235.1	133413	134385	locus396.1	797.85	+	133413	134385	0,0,255	1	972	0
BEWY01083235.1	164818	165619	locus400.1	655.88	+	164818	165619	0,0,255	1	801	0
BEWY01083235.1	190081	190885	locus404.1	706.05	+	190081	190885	0,0,255	1	804	0

Zebrafish

18	31912757	31913675	locus473.1	749.34	-	31912757	31913675	0,0,255	1	918	0
18	31965346	31966228	locus474.1	724.98	-	31965346	31966228	0,0,255	1	882	0
18	32136080	32136995	locus475.1	769.24	-	32136080	32136995	0,0,255	1	915	0
18	32566599	32567412	locus478.1	664.62	+	32566599	32567412	0,0,255	1	813	0
18	32617843	32618647	locus479.1	664.85	+	32617843	32618647	0,0,255	1	804	0
18	32674235	32675048	locus480.1	665.91	-	32674235	32675048	0,0,255	1	813	0
18	32705492	32706302	locus481.1	664.6	-	32705492	32706302	0,0,255	1	810	0
18	32783337	32784267	locus482.1	772.44	+	32783337	32784267	0,0,255	1	930	0
18	32918822	32919974	locus485.1	959.62	+	32918822	32919974	0,0,255	1	1152	0
18	32953321	32954131	locus486.1	664.51	+	32953321	32954131	0,0,255	1	810	0
18	32981139	32982171	locus487.1	858.96	+	32981139	32982171	0,0,255	1	1032	0
18	32994991	32995792	locus488.1	658.52	+	32994991	32995792	0,0,255	1	801	0
18	32999330	33000125	locus489.1	652.27	+	32999330	33000125	0,0,255	1	795	0
18	33012244	33013057	locus490.1	679.23	+	33012244	33013057	0,0,255	1	813	0
18	33035786	33036740	locus492.1	798.58	+	33035786	33036740	0,0,255	1	954	0
18	33055358	33056327	locus493.1	807.96	+	33055358	33056327	0,0,255	1	969	0
18	33078427	33079231	locus494.1	663.5	-	33078427	33079231	0,0,255	1	804	0
18	33081323	33082130	locus495.1	670.88	-	33081323	33082130	0,0,255	1	807	0
18	33102917	33103808	locus497.1	743.91	+	33102917	33103808	0,0,255	1	891	0
18	33134526	33135417	locus498.1	744.82	+	33134526	33135417	0,0,255	1	891	0
18	33174818	33175628	locus500.1	660.55	+	33174818	33175628	0,0,255	1	810	0
18	33188586	33189549	locus501.1	802.06	+	33188586	33189549	0,0,255	1	963	0
18	33215966	33216764	locus502.1	760.02	+	33215966	33216764	0,0,255	1	798	0

18	33239653	33240538	locus503.1	744.53	+	33239653	33240538	0,0,255	1	885	0
18	33250320	33251205	locus504.1	743.88	-	33250320	33251205	0,0,255	1	885	0
18	33267647	33268451	locus505.1	667.79	+	33267647	33268451	0,0,255	1	804	0
18	33275108	33275912	locus506.1	666.32	+	33275108	33275912	0,0,255	1	804	0
18	33280463	33281264	locus507.1	663.32	+	33280463	33281264	0,0,255	1	801	0
18	33293140	33294088	locus509.1	791.53	+	33293140	33294088	0,0,255	1	948	0
18	33313860	33314799	locus511.1	781.09	+	33313860	33314799	0,0,255	1	939	0
18	33330738	33331641	locus513.1	749.82	+	33330738	33331641	0,0,255	1	903	0
18	33333410	33334214	locus514.1	654.04	-	33333410	33334214	0,0,255	1	804	0
18	33343400	33344315	locus516.1	747.61	+	33343400	33344315	0,0,255	1	915	0
18	33356124	33356964	locus517.1	677.58	+	33356124	33356964	0,0,255	1	840	0
18	33377105	33378068	locus519.1	780.97	+	33377105	33378068	0,0,255	1	963	0
18	33388851	33389754	locus520.1	736.19	+	33388851	33389754	0,0,255	1	903	0
18	33410734	33411637	locus521.1	736.26	+	33410734	33411637	0,0,255	1	903	0
18	33423656	33424553	locus522.1	740.42	+	33423656	33424553	0,0,255	1	897	0
18	33472684	33473581	locus524.1	741.22	+	33472684	33473581	0,0,255	1	897	0
18	33492710	33493613	locus525.1	739.94	+	33492710	33493613	0,0,255	1	903	0
18	33525109	33526012	locus526.1	739.49	+	33525109	33526012	0,0,255	1	903	0
18	33553111	33553897	locus527.1	662.42	+	33553111	33553897	0,0,255	1	786	0
18	33560836	33561739	locus528.1	737.8	+	33560836	33561739	0,0,255	1	903	0
18	33576322	33577222	locus530.1	738.05	+	33576322	33577222	0,0,255	1	900	0
18	33583019	33583907	locus531.1	730.29	+	33583019	33583907	0,0,255	1	888	0
18	33594790	33595693	locus532.1	734.25	+	33594790	33595693	0,0,255	1	903	0
18	33655522	33656425	locus533.1	738.8	+	33655522	33656425	0,0,255	1	903	0

18	33678329	33679250	locus534.1	753.38	+	33678329	33679250	0,0,255	1	921	0
18	33687552	33688455	locus535.1	737.61	+	33687552	33688455	0,0,255	1	903	0
18	33820994	33821891	locus536.1	729.12	+	33820994	33821891	0,0,255	1	897	0
18	33917456	33918356	locus538.1	733.51	+	33917456	33918356	0,0,255	1	900	0
18	33972830	33973631	locus539.1	752.4	-	33972830	33973631	0,0,255	1	801	0
18	34241448	34242558	locus541.1	888.81	+	34241448	34242558	0,0,255	1	1110	0
CHR_ALT_CTG	32237932	32238949	locus795.1	835.4	-	32237932	32238949	0,0,255	1	1017	0
CHR_ALT_CTG	33902127	33903027	locus814.1	733.51	+	33902127	33903027	0,0,255	1	900	0
CHR_ALT_CTG	33974726	33975656	locus815.1	767.53	-	33974726	33975656	0,0,255	1	930	0
CHR_ALT_CTG	31965346	31966228	locus828.1	724.98	-	31965346	31966228	0,0,255	1	882	0
CHR_ALT_CTG	32128657	32129449	locus830.1	756.55	-	32128657	32129449	0,0,255	1	792	0
CHR_ALT_CTG	33188586	33189549	locus840.1	802.06	+	33188586	33189549	0,0,255	1	963	0
CHR_ALT_CTG	33215966	33216764	locus841.1	760.02	+	33215966	33216764	0,0,255	1	798	0
CHR_ALT_CTG	33239656	33240541	locus842.1	743.34	+	33239656	33240541	0,0,255	1	885	0
CHR_ALT_CTG	33250335	33251220	locus843.1	742.9	-	33250335	33251220	0,0,255	1	885	0
CHR_ALT_CTG	33270242	33271046	locus844.1	666.72	+	33270242	33271046	0,0,255	1	804	0
CHR_ALT_CTG	33277581	33278385	locus845.1	666.32	+	33277581	33278385	0,0,255	1	804	0
CHR_ALT_CTG	33283107	33283908	locus846.1	663.44	+	33283107	33283908	0,0,255	1	801	0
CHR_ALT_CTG	33318122	33319070	locus848.1	791.37	+	33318122	33319070	0,0,255	1	948	0
CHR_ALT_CTG	33332802	33333732	locus850.1	765.69	+	33332802	33333732	0,0,255	1	930	0
CHR_ALT_CTG	33348050	33348953	locus852.1	749.82	+	33348050	33348953	0,0,255	1	903	0
CHR_ALT_CTG	33350668	33351472	locus853.1	654.05	-	33350668	33351472	0,0,255	1	804	0
CHR_ALT_CTG	33360501	33361416	locus855.1	747.69	+	33360501	33361416	0,0,255	1	915	0
CHR_ALT_CTG	33373428	33374268	locus856.1	677.08	+	33373428	33374268	0,0,255	1	840	0

CHR_ALT_CTG	33394681	33395644	locus858.1	780.97	+	33394681	33395644	0,0,255	1	963	0

Coelacanth

JH126572.1	1660	2485	locus20.1	731.18	+	1660	2485	0,0,255	1	825	0
JH126572.1	19350	20253	locus21.1	741.68	+	19350	20253	0,0,255	1	903	0
JH126572.1	48890	49700	locus26.1	660.1	-	48890	49700	0,0,255	1	810	0
JH126572.1	68313	69111	locus27.1	654.6	-	68313	69111	0,0,255	1	798	0
JH126572.1	371473	372460	locus28.1	789.35	+	371473	372460	0,0,255	1	987	0
JH127178.1	3304	4285	locus131.1	794.96	+	3304	4285	0,0,255	1	981	0
JH127178.1	43758	44580	locus132.1	663.86	+	43758	44580	0,0,255	1	822	0
JH127178.1	199116	200004	locus136.1	688.45	+	199116	200004	0,0,255	2	405,471	0,417
JH127178.1	398452	399292	locus141.1	675.39	+	398452	399292	0,0,255	1	840	0
JH127178.1	441211	442123	locus142.1	751.2	-	441211	442123	0,0,255	1	912	0
JH127178.1	555152	556013	locus143.1	698.43	-	555152	556013	0,0,255	1	861	0
JH127178.1	625370	626363	locus145.1	814.91	-	625370	626363	0,0,255	1	993	0
JH127178.1	688571	689519	locus147.1	781.16	-	688571	689519	0,0,255	1	948	0
JH127178.1	892366	893278	locus150.1	748.03	-	892366	893278	0,0,255	1	912	0
JH127178.1	1106224	1107133	locus153.1	740.34	-	1106224	1E+06	0,0,255	1	909	0
JH127874.1	146802	147708	locus199.1	740.66	-	146802	147708	0,0,255	1	906	0
JH127874.1	180783	181749	locus200.1	791.13	-	180783	181749	0,0,255	1	966	0
JH127874.1	197051	197873	locus201.1	666.56	-	197051	197873	0,0,255	1	822	0
JH127874.1	306245	307157	locus203.1	746.91	-	306245	307157	0,0,255	1	912	0
JH127874.1	387160	388078	locus205.1	757.63	-	387160	388078	0,0,255	1	918	0
JH127874.1	453413	454304	locus206.1	726.88	-	453413	454304	0,0,255	1	891	0
JH127874.1	487820	488777	locus208.1	790.1	-	487820	488777	0,0,255	1	957	0
JH127874.1	506541	507441	locus209.1	742.35	-	506541	507441	0,0,255	1	900	0

JH128519.1	116280	117186	locus248.1	741.83	-	116280	117186	0,0,255	1	906	0
JH128519.1	188965	189877	locus250.1	756.82	-	188965	189877	0,0,255	1	912	0
JH128519.1	224898	225807	locus251.1	753.39	-	224898	225807	0,0,255	1	909	0
JH128519.1	335001	335913	locus252.1	752.03	-	335001	335913	0,0,255	1	912	0
JH128519.1	369706	370615	locus253.1	764.11	-	369706	370615	0,0,255	1	909	0
JH128552.1	157158	158127	locus261.1	800.79	-	157158	158127	0,0,255	1	969	0
JH128552.1	284063	285002	locus262.1	769.07	-	284063	285002	0,0,255	1	939	0
JH128635.1	151162	151984	locus268.1	671.41	+	151162	151984	0,0,255	1	822	0
JH128635.1	177991	178813	locus269.1	665.54	+	177991	178813	0,0,255	1	822	0
JH128635.1	220401	221205	locus270.1	659.63	+	220401	221205	0,0,255	1	804	0
JH128635.1	244179	245097	locus271.1	762.57	+	244179	245097	0,0,255	1	918	0
JH128635.1	266319	267024	locus272.1	654.07	+	266319	267024	0,0,255	1	705	0
JH128635.1	316205	317153	locus274.1	779.83	+	316205	317153	0,0,255	1	948	0
JH128635.1	334892	335801	locus276.1	751.47	+	334892	335801	0,0,255	1	909	0
JH128635.1	351029	351845	locus277.1	666	+	351029	351845	0,0,255	1	816	0
JH128635.1	369080	369992	locus278.1	747.08	+	369080	369992	0,0,255	1	912	0
JH129025.1	36670	37627	locus290.1	779.51	-	36670	37627	0,0,255	1	957	0
JH129025.1	150061	150883	locus292.1	659.25	-	150061	150883	0,0,255	1	822	0
JH129025.1	296338	297250	locus294.1	744.78	-	296338	297250	0,0,255	1	912	0
JH129025.1	315192	316014	locus295.1	657.34	-	315192	316014	0,0,255	1	822	0
JH129158.1	46643	47465	locus301.1	665.88	-	46643	47465	0,0,255	1	822	0
JH129158.1	68588	69488	locus302.1	737.09	-	68588	69488	0,0,255	1	900	0
JH129158.1	122796	123696	locus304.1	738.76	-	122796	123696	0,0,255	1	900	0
JH129158.1	147069	148185	locus305.1	925.87	-	147069	148185	0,0,255	1	1116	0

JH129158.1	168676	169621	locus306.1	775.07	-	168676	169621	0,0,255	1	945	0
JH129158.1	201631	202582	locus308.1	778.93	-	201631	202582	0,0,255	1	951	0
JH129158.1	219866	220778	locus309.1	749.18	-	219866	220778	0,0,255	1	912	0
JH129158.1	262915	263818	locus311.1	740.66	-	262915	263818	0,0,255	1	903	0
JH129405.1	46480	47374	locus318.1	743.84	+	46480	47374	0,0,255	1	894	0
JH129405.1	65006	65828	locus319.1	658.99	+	65006	65828	0,0,255	1	822	0
JH129405.1	109737	110646	locus320.1	737.27	+	109737	110646	0,0,255	1	909	0
JH129405.1	160614	161436	locus321.1	658.49	+	160614	161436	0,0,255	1	822	0
JH129405.1	224732	225554	locus322.1	660.9	+	224732	225554	0,0,255	1	822	0
JH129405.1	244984	245941	locus324.1	783.13	+	244984	245941	0,0,255	1	957	0
JH129411.1	1149	2052	locus325.1	742.24	-	1149	2052	0,0,255	1	903	0
JH129411.1	39781	40594	locus327.1	662.34	+	39781	40594	0,0,255	1	813	0
JH129411.1	53068	53881	locus328.1	669.5	+	53068	53881	0,0,255	1	813	0
JH129411.1	58223	59195	locus329.1	812.66	-	58223	59195	0,0,255	1	972	0
JH129411.1	75954	76767	locus330.1	672.81	-	75954	76767	0,0,255	1	813	0
JH129411.1	104826	105624	locus332.1	649.76	+	104826	105624	0,0,255	1	798	0
JH129411.1	112135	112978	locus333.1	680.99	-	112135	112978	0,0,255	1	843	0
JH129411.1	151203	152100	locus334.1	741.54	+	151203	152100	0,0,255	1	897	0
JH129411.1	159092	160079	locus335.1	814.75	-	159092	160079	0,0,255	1	987	0
JH129675.1	198759	199656	locus341.1	742.66	+	198759	199656	0,0,255	1	897	0
JH130005.1	14273	15185	locus346.1	743.06	+	14273	15185	0,0,255	1	912	0
JH130005.1	29657	30476	locus347.1	669.51	+	29657	30476	0,0,255	1	819	0
JH130005.1	95436	96579	locus349.1	943.01	+	95436	96579	0,0,255	1	1143	0
JH130034.1	33862	34774	locus350.1	748.52	-	33862	34774	0,0,255	1	912	0

JH130034.1	105499	106321	locus351.1	694.56	+	105499	106321	0,0,255	1	822	0
JH130241.1	69336	70233	locus352.1	747.86	+	69336	70233	0,0,255	1	897	0
JH130241.1	93697	94624	locus353.1	752.88	+	93697	94624	0,0,255	1	927	0
JH130857.1	96	1059	locus361.1	790.63	+	96	1059	0,0,255	1	963	0
JH130857.1	50871	51774	locus362.1	739.24	+	50871	51774	0,0,255	1	903	0
JH131058.1	22511	23402	locus365.1	727.14	-	22511	23402	0,0,255	1	891	0
JH131250.1	18165	19140	locus370.1	807.26	+	18165	19140	0,0,255	1	975	0
JH131452.1	20233	21145	locus373.1	754.96	-	20233	21145	0,0,255	1	912	0
JH131509.1	14057	14876	locus374.1	660.9	+	14057	14876	0,0,255	1	819	0
JH131653.1	7876	8698	locus375.1	669.88	-	7876	8698	0,0,255	1	822	0
JH131709.1	6287	7235	locus377.1	765.71	-	6287	7235	0,0,255	1	948	0
JH131961.1	2494	3442	locus380.1	775.96	+	2494	3442	0,0,255	1	948	0
JH132926.1	10416	11319	locus388.1	739.11	+	10416	11319	0,0,255	1	903	0
JH133047.1	3187	4009	locus389.1	657.73	+	3187	4009	0,0,255	1	822	0
AFYH0127316[illegible]	2923	3871	locus395.1	781.77	-	2923	3871	0,0,255	1	948	0
AFYH0127679[illegible]	3638	4595	locus404.1	783.83	-	3638	4595	0,0,255	1	957	0
AFYH0129023[illegible]	206	1118	locus412.1	742.53	-	206	1118	0,0,255	1	912	0
AFYH0129139[illegible]	12	990	locus413.1	801.58	+	12	990	0,0,255	1	978	0

Western_clawed_frog

NC_030677.2	835521	836406	locus2.1	637.43	+	835521	836406	0,0,255	1	885	0
NC_030677.2	878688	879573	locus3.1	641.43	+	878688	879573	0,0,255	1	885	0
NC_030677.2	922748	923624	locus4.1	633.93	+	922748	923624	0,0,255	1	876	0
NC_030677.2	966988	967894	locus5.1	629.43	-	966988	967894	0,0,255	1	906	0
NC_030677.2	978999	979872	locus6.1	631.43	-	978999	979872	0,0,255	1	873	0
NC_030677.2	1036057	1036933	locus8.1	628.43	-	1036057	1036933	0,0,255	1	876	0
NC_030677.2	1054632	1055574	locus9.1	633.92	-	1054632	1055574	0,0,255	1	942	0
NC_030677.2	1190223	1191147	locus11.1	639.22	-	1190223	1191147	0,0,255	1	924	0
NC_030677.2	1227192	1228062	locus12.1	637.22	-	1227192	1228062	0,0,255	1	870	0
NC_030677.2	1261421	1262291	locus13.1	637.22	-	1261421	1262291	0,0,255	1	870	0
NC_030677.2	1306926	1307853	locus14.1	650.41	-	1306926	1307853	0,0,255	1	927	0
NC_030677.2	1327276	1328146	locus15.1	640.92	-	1327276	1328146	0,0,255	1	870	0
NC_030677.2	1384714	1385695	locus16.1	634.93	-	1384714	1385695	0,0,255	1	981	0
NC_030677.2	1403382	1404261	locus17.1	633.22	-	1403382	1404261	0,0,255	1	879	0
NC_030677.2	1425079	1426057	locus18.1	633.43	-	1425079	1426057	0,0,255	1	978	0
NC_030677.2	1450650	1451454	locus19.1	566.51	-	1450650	1451454	0,0,255	1	804	0
NC_030677.2	1468775	1469648	locus20.1	646.41	-	1468775	1469648	0,0,255	1	873	0
NC_030677.2	1517986	1518880	locus21.1	629.43	-	1517986	1518880	0,0,255	1	894	0
NC_030677.2	1540752	1541622	locus22.1	620.93	-	1540752	1541622	0,0,255	1	870	0
NC_030677.2	1560142	1560970	locus23.1	629.92	-	1560142	1560970	0,0,255	1	828	0
NC_030677.2	1636094	1637054	locus24.1	621.43	-	1636094	1637054	0,0,255	1	960	0
NC_030677.2	1662244	1663204	locus25.1	617.94	-	1662244	1663204	0,0,255	1	960	0
NC_030677.2	1699111	1700077	locus26.1	633.42	-	1699111	1700077	0,0,255	1	966	0
NC_030677.2	1736883	1737756	locus27.1	621.43	-	1736883	1737756	0,0,255	1	873	0
NC_030677.2	1774370	1775381	locus28.1	620.44	-	1774370	1775381	0,0,255	1	1011	0
NC_030677.2	1789862	1790735	locus29.1	628.42	-	1789862	1790735	0,0,255	1	873	0
NC_030677.2	1809246	1810119	locus30.1	617.93	-	1809246	1810119	0,0,255	1	873	0
NC_030677.2	1829973	1830846	locus31.1	634.93	-	1829973	1830846	0,0,255	1	873	0
NC_030677.2	1851224	1852097	locus32.1	630.92	-	1851224	1852097	0,0,255	1	873	0
NC_030677.2	1906882	1907761	locus33.1	641.92	-	1906882	1907761	0,0,255	1	879	0
NC_030677.2	1928336	1929215	locus34.1	606.93	-	1928336	1929215	0,0,255	1	879	0
NC_030677.2	1976970	1977888	locus35.1	622.43	+	1976970	1977888	0,0,255	1	918	0
NC_030677.2	1983496	1984372	locus36.1	645.42	-	1983496	1984372	0,0,255	1	876	0
NC_030677.2	2037099	2037981	locus37.1	607.73	+	2037099	2037981	0,0,255	1	882	0
NC_030677.2	2053097	2053973	locus38.1	641.92	-	2053097	2053973	0,0,255	1	876	0
NC_030677.2	2068837	2069719	locus39.1	610.65	-	2068837	2069719	0,0,255	1	882	0
NC_030677.2	2101674	2102556	locus40.1	614.65	-	2101674	2102556	0,0,255	1	882	0
NC_030677.2	2145356	2146238	locus41.1	617.65	-	2145356	2146238	0,0,255	1	882	0
NC_030677.2	2179456	2180338	locus42.1	615.15	-	2179456	2180338	0,0,255	1	882	0
NC_030677.2	2215653	2216535	locus43.1	613.15	-	2215653	2216535	0,0,255	1	882	0
NC_030677.2	2254398	2255280	locus44.1	619.14	-	2254398	2255280	0,0,255	1	882	0
NC_030677.2	2285226	2286108	locus45.1	610.15	-	2285226	2286108	0,0,255	1	882	0
NC_030677.2	2329873	2330755	locus46.1	617.65	-	2329873	2330755	0,0,255	1	882	0

NC_030677.2	2353559	2354441	locus47.1	621.14	-	2353559	2354441	0,0,255	1	882	0
NC_030677.2	2395332	2396208	locus48.1	616.15	-	2395332	2396208	0,0,255	1	876	0
NC_030677.2	2431892	2432744	locus49.1	560.01	+	2431892	2432744	0,0,255	1	852	0
NC_030677.2	2451984	2452863	locus50.1	636.93	+	2451984	2452863	0,0,255	1	879	0
NC_030677.2	2493040	2493922	locus51.1	618.15	+	2493040	2493922	0,0,255	1	882	0
NC_030677.2	2539886	2540768	locus52.1	616.65	+	2539886	2540768	0,0,255	1	882	0
NC_030677.2	2549561	2550467	locus53.1	628.91	-	2549561	2550467	0,0,255	1	906	0
NC_030677.2	2568567	2569446	locus54.1	632.91	-	2568567	2569446	0,0,255	1	879	0
NC_030677.2	2584831	2585710	locus55.1	626.43	-	2584831	2585710	0,0,255	1	879	0
NC_030677.2	2603686	2604565	locus56.1	627.42	-	2603686	2604565	0,0,255	1	879	0
NC_030677.2	2619302	2620181	locus57.1	630.93	-	2619302	2620181	0,0,255	1	879	0
NC_030677.2	2655549	2656422	locus58.1	638.42	-	2655549	2656422	0,0,255	1	873	0
NC_030677.2	2701255	2702128	locus59.1	635.42	-	2701255	2702128	0,0,255	1	873	0
NC_030677.2	2742536	2743409	locus60.1	626.93	-	2742536	2743409	0,0,255	1	873	0
NC_030677.2	2769007	2769883	locus61.1	622.42	+	2769007	2769883	0,0,255	1	876	0
NC_030677.2	2777561	2778500	locus62.1	647.41	-	2777561	2778500	0,0,255	1	939	0
NC_030677.2	2821628	2822522	locus63.1	644.9	-	2821628	2822522	0,0,255	1	894	0
NC_030677.2	2854856	2855753	locus64.1	654.91	-	2854856	2855753	0,0,255	1	897	0
NC_030677.2	2868389	2869286	locus65.1	649.91	-	2868389	2869286	0,0,255	1	897	0
NC_030677.2	2892540	2893413	locus66.1	633.14	-	2892540	2893413	0,0,255	1	873	0
NC_030677.2	2921145	2922048	locus67.1	480.99	-	2921145	2922048	0,0,255	1	903	0
NC_030677.2	2946553	2947456	locus68.1	476.49	-	2946553	2947456	0,0,255	1	903	0
NC_030677.2	2975104	2975983	locus69.1	629.92	-	2975104	2975983	0,0,255	1	879	0
NC_030677.2	2992226	2993168	locus70.1	620.42	-	2992226	2993168	0,0,255	1	942	0
NC_030677.2	3018161	3019037	locus71.1	613.94	-	3018161	3019037	0,0,255	1	876	0
NC_030677.2	3039623	3040496	locus72.1	633.42	-	3039623	3040496	0,0,255	1	873	0
NC_030677.2	3069791	3070664	locus73.1	633.42	-	3069791	3070664	0,0,255	1	873	0
NC_030677.2	3116380	3117253	locus74.1	629.92	-	3116380	3117253	0,0,255	1	873	0
NC_030677.2	3142185	3143121	locus75.1	630.92	-	3142185	3143121	0,0,255	1	936	0
NC_030677.2	3160182	3161115	locus76.1	641.91	-	3160182	3161115	0,0,255	1	933	0
NC_030677.2	3198220	3199114	locus77.1	614.43	-	3198220	3199114	0,0,255	1	894	0
NC_030677.2	3208838	3209732	locus78.1	605.94	-	3208838	3209732	0,0,255	1	894	0
NC_030677.2	3221271	3222129	locus79.1	609.93	-	3221271	3222129	0,0,255	1	858	0
NC_030677.2	3249544	3250438	locus80.1	615.92	-	3249544	3250438	0,0,255	1	894	0
NC_030677.2	3280645	3281539	locus81.1	608.43	-	3280645	3281539	0,0,255	1	894	0
NC_030677.2	3303980	3304856	locus82.1	639.91	-	3303980	3304856	0,0,255	1	876	0
NC_030677.2	3339154	3340030	locus83.1	637.92	-	3339154	3340030	0,0,255	1	876	0
NC_030677.2	3358019	3359024	locus84.1	639.92	-	3358019	3359024	0,0,255	1	1005	0
NC_030677.2	3389547	3390420	locus85.1	625.72	-	3389547	3390420	0,0,255	1	873	0
NC_030677.2	3424640	3425447	locus86.1	638.42	-	3424640	3425447	0,0,255	1	807	0
NC_030677.2	3440294	3441188	locus87.1	623.42	-	3440294	3441188	0,0,255	1	894	0
NC_030677.2	3459493	3460471	locus88.1	611.43	-	3459493	3460471	0,0,255	1	978	0
NC_030677.2	3481486	3482329	locus89.1	644.91	-	3481486	3482329	0,0,255	1	843	0
NC_030677.2	3495126	3496029	locus90.1	599.47	-	3495126	3496029	0,0,255	1	903	0

NC_030677.2	3514380	3515283	locus91.1	583.48	-	3514380	3515283	0,0,255	1	903	0
NC_030677.2	3549805	3550699	locus92.1	574.49	-	3549805	3550699	0,0,255	1	894	0
NC_030677.2	3594496	3595399	locus93.1	574.99	-	3594496	3595399	0,0,255	1	903	0
NC_030677.2	3632369	3633272	locus94.1	576.49	-	3632369	3633272	0,0,255	1	903	0
NC_030677.2	3667415	3668309	locus95.1	572.49	-	3667415	3668309	0,0,255	1	894	0
NC_030677.2	3706490	3707384	locus96.1	580.98	-	3706490	3707384	0,0,255	1	894	0
NC_030677.2	3760561	3761497	locus97.1	497.98	-	3760561	3761497	0,0,255	1	936	0
NC_030677.2	3787950	3788865	locus98.1	466.48	+	3787950	3788865	0,0,255	1	915	0
NC_030677.2	3806621	3807524	locus100.1	582.44	+	3806621	3807524	0,0,255	1	903	0
NC_030677.2	3821903	3822806	locus101.1	492.47	+	3821903	3822806	0,0,255	1	903	0
NC_030677.2	3858541	3859444	locus102.1	477.99	+	3858541	3859444	0,0,255	1	903	0
NC_030677.2	3873752	3874652	locus103.1	484.98	+	3873752	3874652	0,0,255	1	900	0
NC_030677.2	3887777	3888674	locus104.1	623.92	-	3887777	3888674	0,0,255	1	897	0
NC_030677.2	3935515	3936469	locus105.1	627.92	+	3935515	3936469	0,0,255	1	954	0
NC_030677.2	3956234	3957110	locus106.1	636.41	+	3956234	3957110	0,0,255	1	876	0
NC_030677.2	3980795	3981716	locus107.1	628.42	+	3980795	3981716	0,0,255	1	921	0
NC_030677.2	3984076	3985012	locus108.1	502.98	-	3984076	3985012	0,0,255	1	936	0
NC_030677.2	4036195	4037128	locus109.1	627.92	+	4036195	4037128	0,0,255	1	933	0
NC_030677.2	4065319	4066195	locus110.1	623.73	+	4065319	4066195	0,0,255	1	876	0
NC_030677.2	4116921	4117818	locus111.1	629.92	+	4116921	4117818	0,0,255	1	897	0
NC_030677.2	4146605	4147502	locus112.1	627.42	+	4146605	4147502	0,0,255	1	897	0
NC_030677.2	4201613	4202510	locus113.1	634.42	+	4201613	4202510	0,0,255	1	897	0
NC_030677.2	4240714	4241608	locus114.1	637.92	+	4240714	4241608	0,0,255	1	894	0
NC_030677.2	4282340	4283213	locus115.1	630.41	+	4282340	4283213	0,0,255	1	873	0
NC_030677.2	4306664	4307537	locus116.1	628.91	+	4306664	4307537	0,0,255	1	873	0
NC_030677.2	4332295	4333168	locus117.1	631.91	+	4332295	4333168	0,0,255	1	873	0
NC_030677.2	4361025	4361889	locus118.1	637.41	+	4361025	4361889	0,0,255	1	864	0
NC_030677.2	4375708	4376581	locus120.1	616.93	-	4375708	4376581	0,0,255	1	873	0
NC_030677.2	4413199	4414078	locus121.1	630.42	+	4413199	4414078	0,0,255	1	879	0
NC_030677.2	4424790	4425669	locus122.1	654.41	-	4424790	4425669	0,0,255	1	879	0
NC_030677.2	4452458	4453451	locus123.1	629.42	-	4452458	4453451	0,0,255	1	993	0
NC_030677.2	4480290	4481073	locus124.1	620.72	-	4480290	4481073	0,0,255	1	783	0
NC_030677.2	4532903	4533800	locus126.1	497.48	-	4532903	4533800	0,0,255	1	897	0
NC_030677.2	4605693	4606563	locus127.1	626.42	-	4605693	4606563	0,0,255	1	870	0
NC_030677.2	4636237	4637035	locus128.1	619.2	-	4636237	4637035	0,0,255	1	798	0
NC_030677.2	4662954	4663860	locus129.1	491.99	-	4662954	4663860	0,0,255	1	906	0
NC_030677.2	4716277	4717177	locus130.1	483.03	+	4716277	4717177	0,0,255	1	900	0
NC_030677.2	4776011	4776911	locus131.1	489.77	+	4776011	4776911	0,0,255	1	900	0
NC_030677.2	4808558	4809458	locus132.1	494.77	+	4808558	4809458	0,0,255	1	900	0
NC_030677.2	4846804	4847704	locus133.1	482.77	+	4846804	4847704	0,0,255	1	900	0
NC_030677.2	4861408	4862305	locus134.1	632.41	-	4861408	4862305	0,0,255	1	897	0
NC_030677.2	4890474	4891428	locus135.1	630.42	-	4890474	4891428	0,0,255	1	954	0
NC_030677.2	5015936	5016833	locus136.1	640.42	-	5015936	5016833	0,0,255	1	897	0
NC_030677.2	5071397	5072303	locus138.1	499.48	-	5071397	5072303	0,0,255	1	906	0

NC_030677.2	5103811	5104612	locus139.1	459.91	-	5103811	5104612	0,0,255	1	801	0
NC_030677.2	5147625	5148531	locus140.1	486.49	+	5147625	5148531	0,0,255	1	906	0
NC_030677.2	5197550	5198456	locus141.1	474.99	+	5197550	5198456	0,0,255	1	906	0
NC_030677.2	5206604	5207510	locus142.1	497.96	-	5206604	5207510	0,0,255	1	906	0
NC_030677.2	5249709	5250585	locus144.1	642.41	+	5249709	5250585	0,0,255	1	876	0
NC_030677.2	5279106	5280000	locus145.1	635.91	+	5279106	5280000	0,0,255	1	894	0
NC_030677.2	5306749	5307646	locus146.1	629.41	+	5306749	5307646	0,0,255	1	897	0
NC_030677.2	5311195	5312095	locus148.1	489.77	-	5311195	5312095	0,0,255	1	900	0
NC_030677.2	5348557	5349457	locus149.1	493.77	-	5348557	5349457	0,0,255	1	900	0
NC_030677.2	5396571	5397471	locus150.1	495.77	-	5396571	5397471	0,0,255	1	900	0
NC_030677.2	5442351	5443182	locus151.1	611.93	-	5442351	5443182	0,0,255	1	831	0
NC_030677.2	5460272	5461103	locus152.1	616.93	-	5460272	5461103	0,0,255	1	831	0
NC_030677.2	5478893	5479724	locus153.1	498.47	-	5478893	5479724	0,0,255	1	831	0
NC_030677.2	5499045	5499876	locus154.1	508.97	-	5499045	5499876	0,0,255	1	831	0
NC_030677.2	5529137	5551850	locus156.1	577.04	-	5529137	5551850	0,0,255	4	822,8,7,9	0,868,12696,22704
NC_030677.2	5584329	5585208	locus157.1	639.91	+	5584329	5585208	0,0,255	1	879	0
NC_030677.2	5617299	5618178	locus158.1	646.41	+	5617299	5618178	0,0,255	1	879	0
NC_030677.2	5658779	5659787	locus159.1	634.92	+	5658779	5659787	0,0,255	1	1008	0
NC_030677.2	5691252	5692146	locus160.1	633.91	+	5691252	5692146	0,0,255	1	894	0
NC_030677.2	5731978	5732947	locus161.1	625.42	+	5731978	5732947	0,0,255	1	969	0
NC_030677.2	5776826	5777717	locus162.1	625.93	+	5776826	5777717	0,0,255	1	891	0
NC_030677.2	5799201	5800035	locus163.1	645.91	+	5799201	5800035	0,0,255	1	834	0
NC_030677.2	5838950	5839829	locus164.1	702.89	+	5838950	5839829	0,0,255	1	879	0
NC_030677.2	5866601	5867492	locus165.1	686.4	+	5866601	5867492	0,0,255	1	891	0
NC_030677.2	5889423	5890302	locus166.1	603.44	+	5889423	5890302	0,0,255	1	879	0
NC_030677.2	5900397	5901273	locus167.1	636.41	+	5900397	5901273	0,0,255	1	876	0
NC_030677.2	5938585	5939485	locus168.1	491.77	+	5938585	5939485	0,0,255	1	900	0
NC_030677.2	5964465	5965359	locus169.1	638.92	-	5964465	5965359	0,0,255	1	894	0
NC_030677.2	5980729	5981626	locus170.1	640.91	-	5980729	5981626	0,0,255	1	897	0
NC_030677.2	6051208	6052114	locus172.1	475.98	+	6051208	6052114	0,0,255	1	906	0
NC_030677.2	6060145	6061051	locus173.1	488.48	+	6060145	6061051	0,0,255	1	906	0
NC_030677.2	6088582	6089488	locus174.1	493.48	+	6088582	6089488	0,0,255	1	906	0
NC_030677.2	6097290	6098196	locus175.1	482.99	+	6097290	6098196	0,0,255	1	906	0
NC_030677.2	6112030	6112936	locus176.1	486.48	+	6112030	6112936	0,0,255	1	906	0
NC_030677.2	6141782	6142709	locus177.1	482.49	+	6141782	6142709	0,0,255	1	927	0
NC_030677.2	6165663	6166569	locus178.1	492.99	+	6165663	6166569	0,0,255	1	906	0
NC_030677.2	6213981	6214887	locus180.1	501.47	-	6213981	6214887	0,0,255	1	906	0
NC_030677.2	6267901	6268831	locus183.1	495.47	+	6267901	6268831	0,0,255	1	930	0
NC_030677.2	6317180	6318077	locus184.1	488.48	+	6317180	6318077	0,0,255	1	897	0
NC_030677.2	6353631	6354414	locus185.1	619.72	+	6353631	6354414	0,0,255	1	783	0
NC_030677.2	6418203	6418986	locus187.1	624.92	+	6418203	6418986	0,0,255	1	783	0
NC_030677.2	6474834	6475785	locus188.1	639.41	+	6474834	6475785	0,0,255	1	951	0
NC_030677.2	6570546	6571443	locus190.1	625.93	-	6570546	6571443	0,0,255	1	897	0
NC_030677.2	6643516	6644446	locus191.1	640.41	+	6643516	6644446	0,0,255	1	930	0

NC_030677.2	6678414	6679314	locus192.1	703.62	+	6678414	6679314	0,0,255	1	900	0
NC_030677.2	6689068	6690076	locus193.1	620.71	+	6689068	6690076	0,0,255	1	1008	0
NC_030677.2	6939211	6940171	locus194.1	622.92	+	6939211	6940171	0,0,255	1	960	0
NC_030677.2	6964861	6965659	locus195.1	619.43	+	6964861	6965659	0,0,255	1	798	0
NC_030677.2	6971533	6972514	locus196.1	605.44	-	6971533	6972514	0,0,255	1	981	0
NC_030677.2	6997627	6998608	locus197.1	595.95	-	6997627	6998608	0,0,255	1	981	0
NC_030677.2	7037919	7038813	locus198.1	619.93	-	7037919	7038813	0,0,255	1	894	0
NC_030677.2	7056246	7057143	locus199.1	614.43	-	7056246	7057143	0,0,255	1	897	0
NC_030677.2	7071603	7072500	locus200.1	625.43	-	7071603	7072500	0,0,255	1	897	0
NC_030677.2	7128878	7129802	locus202.1	511.97	+	7128878	7129802	0,0,255	1	924	0
NC_030677.2	7156126	7156924	locus203.1	612.97	+	7156126	7156924	0,0,255	1	798	0
NC_030677.2	7190191	7191067	locus204.1	638.41	+	7190191	7191067	0,0,255	1	876	0
NC_030677.2	7223913	7224789	locus205.1	638.41	+	7223913	7224789	0,0,255	1	876	0
NC_030677.2	7254821	7255697	locus206.1	635.41	+	7254821	7255697	0,0,255	1	876	0
NC_030677.2	7275315	7276191	locus207.1	633.91	+	7275315	7276191	0,0,255	1	876	0
NC_030677.2	7296340	7297216	locus208.1	631.92	+	7296340	7297216	0,0,255	1	876	0
NC_030677.2	7316177	7317053	locus209.1	633.42	+	7316177	7317053	0,0,255	1	876	0
NC_030677.2	7353454	7354330	locus210.1	629.92	+	7353454	7354330	0,0,255	1	876	0
NC_030677.2	7388298	7389174	locus211.1	636.41	+	7388298	7389174	0,0,255	1	876	0
NC_030677.2	7418154	7419027	locus212.1	635.43	+	7418154	7419027	0,0,255	1	873	0
NC_030677.2	7460584	7461460	locus213.1	622.44	+	7460584	7461460	0,0,255	1	876	0
NC_030677.2	7473810	7474686	locus214.1	642.41	+	7473810	7474686	0,0,255	1	876	0
NC_030677.2	7479293	7480172	locus215.1	625.92	-	7479293	7480172	0,0,255	1	879	0
NC_030677.2	7495573	7496512	locus216.1	602.93	-	7495573	7496512	0,0,255	1	939	0
NC_030677.2	7519463	7520402	locus217.1	613.43	-	7519463	7520402	0,0,255	1	939	0
NC_030677.2	7544993	7545929	locus218.1	609.93	-	7544993	7545929	0,0,255	1	936	0
NC_030677.2	7568816	7569755	locus219.1	607.93	-	7568816	7569755	0,0,255	1	939	0
NC_030677.2	7588599	7589538	locus221.1	606.94	-	7588599	7589538	0,0,255	1	939	0
NC_030677.2	7607166	7608105	locus222.1	608.43	-	7607166	7608105	0,0,255	1	939	0
NC_030677.2	7627433	7628327	locus224.1	610.93	-	7627433	7628327	0,0,255	1	894	0
NC_030677.2	7647375	7648272	locus225.1	642.91	-	7647375	7648272	0,0,255	1	897	0
NC_030677.2	7667698	7668511	locus226.1	641.45	-	7667698	7668511	0,0,255	1	813	0
NC_030677.2	7705636	7706512	locus227.1	630.92	+	7705636	7706512	0,0,255	1	876	0
NC_030677.2	7738892	7739768	locus228.1	605.93	-	7738892	7739768	0,0,255	1	876	0
NC_030677.2	7770787	7771657	locus229.1	629.92	-	7770787	7771657	0,0,255	1	870	0
NC_030677.2	7791627	7792506	locus230.1	623.43	-	7791627	7792506	0,0,255	1	879	0
NC_030677.2	7823648	7824527	locus231.1	622.93	-	7823648	7824527	0,0,255	1	879	0
NC_030677.2	7856914	7857793	locus232.1	625.93	-	7856914	7857793	0,0,255	1	879	0
NC_030677.2	7881908	7882787	locus233.1	624.94	-	7881908	7882787	0,0,255	1	879	0
NC_030677.2	7922676	7923555	locus234.1	626.93	-	7922676	7923555	0,0,255	1	879	0
NC_030677.2	8011457	8012351	locus236.1	621.93	+	8011457	8012351	0,0,255	1	894	0
NC_030677.2	8081627	8082506	locus237.1	623.93	+	8081627	8082506	0,0,255	1	879	0
NC_030677.2	8096116	8097049	locus238.1	635.42	-	8096116	8097049	0,0,255	1	933	0
NC_030677.2	8172828	8173722	locus239.1	615.93	+	8172828	8173722	0,0,255	1	894	0

NC_030677.2	8246276	8247110	locus240.1	622.42	+	8246276	8247110	0,0,255	1	834	0
NC_030677.2	8269738	8270632	locus241.1	618.93	+	8269738	8270632	0,0,255	1	894	0
NC_030677.2	8277431	8278162	locus242.1	220.99	-	8277431	8278162	0,0,255	3	########	0,275,440
NC_030677.2	8289029	8289938	locus243.1	615.43	+	8289029	8289938	0,0,255	1	909	0
NC_030677.2	8291931	8292828	locus244.1	621.42	-	8291931	8292828	0,0,255	1	897	0
NC_030677.2	8335313	8336207	locus245.1	627.42	+	8335313	8336207	0,0,255	1	894	0
NC_030677.2	8367032	8368007	locus246.1	618.42	+	8367032	8368007	0,0,255	1	975	0
NC_030677.2	8400144	8401116	locus247.1	612.93	+	8400144	8401116	0,0,255	1	972	0
NC_030677.2	8422551	8423448	locus248.1	633.43	+	8422551	8423448	0,0,255	1	897	0
NC_030677.2	8426584	8427370	locus249.1	619.93	-	8426584	8427370	0,0,255	1	786	0
NC_030677.2	8445527	8446421	locus250.1	626.43	-	8445527	8446421	0,0,255	1	894	0
NC_030677.2	8466696	8467590	locus251.1	617.92	-	8466696	8467590	0,0,255	1	894	0
NC_030677.2	8505718	8506612	locus252.1	618.43	-	8505718	8506612	0,0,255	1	894	0
NC_030677.2	8547622	8548597	locus254.1	610.93	-	8547622	8548597	0,0,255	1	975	0
NC_030677.2	8574731	8575676	locus255.1	622.43	-	8574731	8575676	0,0,255	1	945	0
NC_030677.2	8595310	8596204	locus256.1	619.93	-	8595310	8596204	0,0,255	1	894	0
NC_030677.2	8614680	8615556	locus257.1	634.43	-	8614680	8615556	0,0,255	1	876	0
NC_030677.2	8677649	8678615	locus258.1	635.92	+	8677649	8678615	0,0,255	1	966	0
NC_030677.2	8683850	8684726	locus259.1	641.42	-	8683850	8684726	0,0,255	1	876	0
NC_030677.2	8697403	8698279	locus260.1	636.92	-	8697403	8698279	0,0,255	1	876	0
NC_030677.2	8740732	8741608	locus261.1	632.42	-	8740732	8741608	0,0,255	1	876	0
NC_030677.2	8776365	8777241	locus262.1	630.42	-	8776365	8777241	0,0,255	1	876	0
NC_030677.2	8809061	8810027	locus263.1	638.43	-	8809061	8810027	0,0,255	1	966	0
NC_030677.2	8882872	8883802	locus265.1	498.47	-	8882872	8883802	0,0,255	1	930	0
NC_030677.2	8923168	8924107	locus266.1	498.47	-	8923168	8924107	0,0,255	1	939	0
NC_030677.2	8974456	8975395	locus268.1	499.46	-	8974456	8975395	0,0,255	1	939	0
NC_030677.2	9039083	9039965	locus269.1	614.66	+	9039083	9039965	0,0,255	1	882	0
NC_030677.2	9087616	9088555	locus270.1	620.15	+	9087616	9088555	0,0,255	1	939	0
NC_030677.2	9143139	9144111	locus271.1	608.25	+	9143139	9144111	0,0,255	1	972	0
NC_030677.2	9174874	9175759	locus272.1	617.16	+	9174874	9175759	0,0,255	1	885	0
NC_030677.2	9200577	9201450	locus274.1	605.76	-	9200577	9201450	0,0,255	1	873	0
NC_030677.2	9239717	9240599	locus275.1	622.15	-	9239717	9240599	0,0,255	1	882	0
NC_030677.2	9285903	9286785	locus277.1	613.65	-	9285903	9286785	0,0,255	1	882	0
NC_030677.2	9327549	9328428	locus278.1	618.14	-	9327549	9328428	0,0,255	1	879	0
NC_030677.2	9348790	9349693	locus279.1	483.98	-	9348790	9349693	0,0,255	1	903	0
NC_030677.2	9387125	9388019	locus280.1	630.42	+	9387125	9388019	0,0,255	1	894	0
NC_030677.2	9410297	9411170	locus282.1	620.93	+	9410297	9411170	0,0,255	1	873	0
NC_030677.2	17430992	17431916	locus283.1	477	+	17430992	17431916	0,0,255	1	924	0
NC_030677.2	17478457	17479354	locus285.1	585.46	+	17478457	17479354	0,0,255	1	897	0
NC_030677.2	17496128	17497025	locus286.1	579.46	+	17496128	17497025	0,0,255	1	897	0
NC_030677.2	17524962	17525859	locus287.1	571.46	+	17524962	17525859	0,0,255	1	897	0
NC_030677.2	17546542	17547421	locus288.1	573.97	+	17546542	17547421	0,0,255	1	879	0
NC_030677.2	17573752	17574649	locus290.1	587.46	+	17573752	17574649	0,0,255	1	897	0
NC_030677.2	17598296	17599193	locus291.1	578.96	+	17598296	17599193	0,0,255	1	897	0

NC_030677.2	17616658	17617555	locus292.1	578.46	+	17616658	17617555	0,0,255	1	897	0
NC_030677.2	17641760	17642639	locus294.1	561.98	+	17641760	17642639	0,0,255	1	879	0
NC_030677.2	17669122	17670001	locus295.1	566.47	+	17669122	17670001	0,0,255	1	879	0
NC_030677.2	17689807	17690704	locus296.1	573.97	+	17689807	17690704	0,0,255	1	897	0
NC_030678.2	21334	27928	locus332.1	629.59	+	21334	27928	0,0,255	2	9,879	0,5715
NC_030678.2	178164867	1.78E+08	locus374.1	689.69	-	178164867	178165770	0,0,255	1	903	0
NC_030678.2	178209944	1.78E+08	locus375.1	689.69	-	178209944	178210847	0,0,255	1	903	0
NC_030678.2	178237350	1.78E+08	locus376.1	690.19	-	178237350	178238253	0,0,255	1	903	0
NC_030678.2	178311473	1.78E+08	locus377.1	683.2	-	178311473	178312376	0,0,255	1	903	0
NC_030678.2	178396223	1.78E+08	locus378.1	689.7	+	178396223	178397132	0,0,255	1	909	0
NC_030678.2	178416973	1.78E+08	locus379.1	685.24	-	178416973	178417876	0,0,255	1	903	0
NC_030678.2	178470580	1.78E+08	locus380.1	692.74	-	178470580	178471483	0,0,255	1	903	0
NC_030678.2	178501805	1.79E+08	locus381.1	684.74	-	178501805	178502708	0,0,255	1	903	0
NC_030678.2	178540754	1.79E+08	locus382.1	693.73	-	178540754	178541657	0,0,255	1	903	0
NC_030678.2	178573630	1.79E+08	locus383.1	692.23	-	178573630	178574533	0,0,255	1	903	0
NC_030678.2	178654064	1.79E+08	locus385.1	704.23	+	178654064	178654967	0,0,255	1	903	0
NC_030678.2	178665401	1.79E+08	locus386.1	695.73	-	178665401	178666304	0,0,255	1	903	0
NC_030678.2	178709921	1.79E+08	locus388.1	694.73	-	178709921	178710818	0,0,255	1	897	0
NC_030678.2	178733260	1.79E+08	locus389.1	685.74	-	178733260	178734157	0,0,255	1	897	0
NC_030679.2	145271855	1.45E+08	locus416.1	442.21	+	145271855	145272815	0,0,255	1	960	0
NC_030679.2	145283085	1.45E+08	locus417.1	436.71	+	145283085	145284045	0,0,255	1	960	0
NC_030679.2	145622605	1.46E+08	locus418.1	717.92	+	145622605	145623526	0,0,255	1	921	0
NC_030679.2	145630946	1.46E+08	locus419.1	717.92	+	145630946	145631867	0,0,255	1	921	0
NC_030679.2	145647365	1.46E+08	locus420.1	622.89	+	145647365	145648214	0,0,255	1	849	0
NC_030679.2	145712850	1.46E+08	locus421.1	708.35	+	145712850	145713801	0,0,255	1	951	0
NC_030679.2	145728373	1.46E+08	locus422.1	558.76	+	145728373	145729291	0,0,255	1	918	0
NC_030679.2	145741602	1.46E+08	locus423.1	593.17	+	145741602	145742487	0,0,255	1	885	0
NC_030679.2	145755902	1.46E+08	locus424.1	718.6	+	145755902	145756820	0,0,255	1	918	0
NC_030679.2	145770103	1.46E+08	locus425.1	705.18	+	145770103	145771000	0,0,255	1	897	0
NC_030679.2	145809884	1.46E+08	locus426.1	519.79	+	145809884	145810895	0,0,255	1	1011	0
NC_030679.2	145823381	1.46E+08	locus427.1	508.43	+	145823381	145824350	0,0,255	1	969	0
NC_030679.2	145840075	1.46E+08	locus428.1	650.23	+	145840075	145840975	0,0,255	1	900	0
NC_030679.2	145857765	1.46E+08	locus429.1	681.21	+	145857765	145858644	0,0,255	1	879	0
NC_030679.2	145888657	1.46E+08	locus431.1	696.71	+	145888657	145889536	0,0,255	1	879	0
NC_030679.2	145898777	1.46E+08	locus432.1	686.71	+	145898777	145899656	0,0,255	1	879	0
NC_030679.2	145909288	1.46E+08	locus433.1	681.73	+	145909288	145910284	0,0,255	1	996	0
NC_030679.2	145930741	1.46E+08	locus434.1	664.22	+	145930741	145931611	0,0,255	1	870	0
NC_030679.2	145942731	1.46E+08	locus435.1	720.69	+	145942731	145943595	0,0,255	1	864	0
NC_030679.2	145958197	1.46E+08	locus436.1	684.22	+	145958197	145959070	0,0,255	1	873	0
NC_030679.2	145975594	1.46E+08	locus437.1	675.22	+	145975594	145976467	0,0,255	1	873	0
NC_030679.2	145994586	1.46E+08	locus438.1	675.73	+	145994586	145995459	0,0,255	1	873	0
NC_030679.2	146006472	1.46E+08	locus439.1	675.73	+	146006472	146007351	0,0,255	1	879	0
NC_030679.2	146020705	1.46E+08	locus441.1	676.72	+	146020705	146021584	0,0,255	1	879	0
NC_030679.2	146032668	1.46E+08	locus442.1	657.23	+	146032668	146033547	0,0,255	1	879	0

NC_030679.2	146046333	1.46E+08	locus443.1	645.73	+	146046333	146047218	0,0,255	1	885	0
NC_030679.2	146059583	1.46E+08	locus444.1	650.72	+	146059583	146060462	0,0,255	1	879	0
NC_030679.2	146075269	1.46E+08	locus445.1	592.62	+	146075269	146076166	0,0,255	1	897	0
NC_030679.2	146087607	1.46E+08	locus446.1	608.36	+	146087607	146088495	0,0,255	1	888	0
NC_030679.2	146096117	1.46E+08	locus447.1	625.61	+	146096117	146097017	0,0,255	1	900	0
NC_030679.2	146109752	1.46E+08	locus448.1	606.1	+	146109752	146110679	0,0,255	1	927	0
NC_030679.2	146119495	1.46E+08	locus449.1	610.62	+	146119495	146120413	0,0,255	1	918	0
NC_030679.2	146130416	1.46E+08	locus451.1	595.88	+	146130416	146131316	0,0,255	1	900	0
NC_030679.2	146141168	1.46E+08	locus452.1	657.35	+	146141168	146142068	0,0,255	1	900	0
NC_030679.2	146153466	1.46E+08	locus453.1	623.6	+	146153466	146154420	0,0,255	1	954	0
NC_030679.2	146166997	1.46E+08	locus454.1	625.37	+	146166997	146167777	0,0,255	1	780	0
NC_030679.2	146180285	1.46E+08	locus455.1	621.11	+	146180285	146181182	0,0,255	1	897	0
NC_030679.2	146190904	1.46E+08	locus456.1	606.87	+	146190904	146191804	0,0,255	1	900	0
NC_030679.2	146205635	1.46E+08	locus457.1	587.39	+	146205635	146206415	0,0,255	1	780	0
NC_030679.2	146210829	1.46E+08	locus458.1	620.11	-	146210829	146211807	0,0,255	1	978	0
NC_030679.2	146231789	1.46E+08	locus459.1	598.12	+	146231789	146232698	0,0,255	1	909	0
NC_030679.2	146241341	1.46E+08	locus460.1	602.61	+	146241341	146242241	0,0,255	1	900	0
NC_030679.2	146390737	1.46E+08	locus463.1	606.37	-	146390737	146391640	0,0,255	1	903	0
NC_030679.2	146415510	1.46E+08	locus464.1	616.85	-	146415510	146416416	0,0,255	1	906	0
NC_030679.2	146427112	1.46E+08	locus465.1	661.84	-	146427112	146428009	0,0,255	1	897	0
NC_030679.2	146436266	1.46E+08	locus466.1	729.31	-	146436266	146437106	0,0,255	1	840	0
NC_030679.2	146445846	1.46E+08	locus467.1	616.36	-	146445846	146446746	0,0,255	1	900	0
NC_030679.2	146457394	1.46E+08	locus468.1	611.12	-	146457394	146458294	0,0,255	1	900	0
NC_030679.2	146483646	1.46E+08	locus469.1	610.38	+	146483646	146484546	0,0,255	1	900	0
NC_030679.2	146495074	1.46E+08	locus470.1	628.6	+	146495074	146495974	0,0,255	1	900	0
NC_030679.2	146508942	1.47E+08	locus471.1	621.61	+	146508942	146509842	0,0,255	1	900	0
NC_030679.2	146521524	1.47E+08	locus472.1	623.61	+	146521524	146522445	0,0,255	1	921	0
NC_030679.2	146541550	1.47E+08	locus473.1	617.86	+	146541550	146542390	0,0,255	1	840	0
NC_030679.2	146555963	1.47E+08	locus474.1	619.12	+	146555963	146556863	0,0,255	1	900	0
NC_030679.2	146568197	1.47E+08	locus475.1	625.85	+	146568197	146569085	0,0,255	1	888	0
NC_030679.2	146573705	1.47E+08	locus476.1	620.35	-	146573705	146574575	0,0,255	1	870	0
NC_030679.2	146585402	1.47E+08	locus477.1	629.61	-	146585402	146586296	0,0,255	1	894	0
NC_030679.2	146598469	1.47E+08	locus478.1	628.86	-	146598469	146599357	0,0,255	1	888	0
NC_030679.2	146617463	1.47E+08	locus479.1	612.36	+	146617463	146618336	0,0,255	1	873	0
NC_030679.2	146626905	1.47E+08	locus480.1	591.26	+	146626905	146627682	0,0,255	1	777	0
NC_030679.2	146637775	1.47E+08	locus481.1	618.62	+	146637775	146638669	0,0,255	1	894	0
NC_030679.2	146650644	1.47E+08	locus482.1	621.85	+	146650644	146651409	0,0,255	1	765	0
NC_030679.2	146656544	1.47E+08	locus483.1	622.12	+	146656544	146657435	0,0,255	1	891	0
NC_030679.2	146668798	1.47E+08	locus484.1	627.11	+	146668798	146669704	0,0,255	1	906	0
NC_030679.2	146677964	1.47E+08	locus485.1	609.12	+	146677964	146678864	0,0,255	1	900	0
NC_030679.2	146686602	1.47E+08	locus486.1	610.61	+	146686602	146687502	0,0,255	1	900	0
NC_030679.2	146698121	1.47E+08	locus487.1	621.61	+	146698121	146699021	0,0,255	1	900	0
NC_030679.2	146709546	1.47E+08	locus488.1	610.11	+	146709546	146710440	0,0,255	1	894	0
NC_030679.2	146722272	1.47E+08	locus489.1	622.86	+	146722272	146723196	0,0,255	1	924	0

NC_030679.2	146727993	1.47E+08	locus490.1	702	-	146727993	146728956	0,0,255	1	963	0
NC_030679.2	146746937	1.47E+08	locus491.1	610.62	+	146746937	146747837	0,0,255	1	900	0
NC_030679.2	146752351	1.47E+08	locus492.1	724.56	-	146752351	146753251	0,0,255	1	900	0
NC_030679.2	146761250	1.47E+08	locus493.1	612.62	-	146761250	146762237	0,0,255	1	987	0
NC_030679.2	146772261	1.47E+08	locus494.1	628.85	-	146772261	146773161	0,0,255	1	900	0
NC_030679.2	146791494	1.47E+08	locus496.1	621.61	-	146791494	146792394	0,0,255	1	900	0
NC_030679.2	146812154	1.47E+08	locus497.1	715.04	+	146812154	146812946	0,0,255	1	792	0
NC_030679.2	146824901	1.47E+08	locus498.1	721.91	+	146824901	146825834	0,0,255	1	933	0
NC_030679.2	146847230	1.47E+08	locus499.1	398.13	+	146847230	146848133	0,0,255	1	903	0
NC_030680.2	24522	25397	locus502.1	632.86	+	24522	25397	0,0,255	2	30,834	0,41
NC_030680.2	24688609	24689527	locus505.1	409.89	+	24688609	24689527	0,0,255	1	918	0
NC_030680.2	24711758	24712661	locus506.1	394.9	+	24711758	24712661	0,0,255	1	903	0
NC_030680.2	24741300	24742218	locus507.1	399.9	+	24741300	24742218	0,0,255	1	918	0
NC_030680.2	24791734	24792631	locus508.1	396.4	+	24791734	24792631	0,0,255	1	897	0
NC_030680.2	128529424	1.29E+08	locus531.1	697.86	-	128529424	128530360	0,0,255	1	936	0
NC_030680.2	152998213	1.53E+08	locus536.1	633.33	+	152998213	153006860	0,0,255	3	26,14,896	0,6824,7751
NC_030681.2	116456528	1.16E+08	locus568.1	697.58	-	116456528	116457512	0,0,255	1	984	0
NC_030681.2	116482565	1.16E+08	locus569.1	695.8	-	116482565	116483405	0,0,255	1	840	0
NC_030681.2	154715071	1.55E+08	locus578.1	413.96	-	154715071	154715854	0,0,255	1	783	0
NC_030681.2	154750009	1.55E+08	locus580.1	415.47	-	154750009	154750792	0,0,255	1	783	0
NC_030681.2	154764974	1.55E+08	locus581.1	419.46	-	154764974	154765931	0,0,255	1	957	0
NC_030681.2	154780095	1.55E+08	locus582.1	418.96	-	154780095	154781025	0,0,255	1	930	0
NC_030681.2	154800559	1.55E+08	locus583.1	419.47	-	154800559	154801342	0,0,255	1	783	0
NC_030681.2	154841651	1.55E+08	locus584.1	412.48	-	154841651	154842563	0,0,255	1	912	0
NC_030681.2	154860552	1.55E+08	locus585.1	411.48	-	154860552	154861518	0,0,255	1	966	0
NC_030681.2	154877224	1.55E+08	locus586.1	410.49	-	154877224	154878022	0,0,255	1	798	0
NC_030681.2	154899877	1.55E+08	locus587.1	421.97	-	154899877	154900789	0,0,255	1	912	0
NC_030681.2	154924047	1.55E+08	locus588.1	424.46	-	154924047	154924851	0,0,255	1	804	0
NC_030681.2	154955745	1.55E+08	locus589.1	414.38	-	154955745	154956606	0,0,255	1	861	0
NC_030681.2	154980120	1.55E+08	locus590.1	382.77	-	154980120	154980893	0,0,255	2	742,14	0,759
NC_030681.2	155007217	1.55E+08	locus591.1	423.46	-	155007217	155008129	0,0,255	1	912	0
NC_030681.2	155038792	1.55E+08	locus592.1	420.4	-	155038792	155039734	0,0,255	1	942	0
NC_030681.2	155055448	1.55E+08	locus593.1	423.46	-	155055448	155056252	0,0,255	1	804	0
NC_030681.2	155078235	1.55E+08	locus594.1	414.35	-	155078235	155079186	0,0,255	1	951	0
NC_030681.2	155188260	1.55E+08	locus596.1	414.47	-	155188260	155189190	0,0,255	1	930	0
NC_030681.2	155220413	1.55E+08	locus597.1	429.96	-	155220413	155221343	0,0,255	1	930	0
NC_030681.2	155249835	1.55E+08	locus598.1	421.97	-	155249835	155250750	0,0,255	1	915	0
NC_030681.2	155268003	1.55E+08	locus599.1	423.47	-	155268003	155268810	0,0,255	1	807	0
NC_030681.2	155288745	1.55E+08	locus600.1	416.97	-	155288745	155289675	0,0,255	1	930	0
NC_030681.2	155309287	1.55E+08	locus601.1	432.45	-	155309287	155310217	0,0,255	1	930	0
NC_030681.2	155324442	1.55E+08	locus602.1	423.97	-	155324442	155325249	0,0,255	1	807	0
NC_030681.2	155340816	1.55E+08	locus603.1	411.47	-	155340816	155341746	0,0,255	1	930	0
NC_030681.2	155361948	1.55E+08	locus604.1	414.47	-	155361948	155362878	0,0,255	1	930	0
NC_030681.2	155383950	1.55E+08	locus605.1	409.47	-	155383950	155384880	0,0,255	1	930	0

NC_030681.2	155399948	1.55E+08	locus606.1	409.88	-	155399948	155400878	0,0,255	1	930	0
NC_030681.2	155417584	1.55E+08	locus607.1	423.96	-	155417584	155418514	0,0,255	1	930	0
NC_030681.2	155441606	1.55E+08	locus608.1	420.47	-	155441606	155442536	0,0,255	1	930	0
NC_030681.2	155462515	1.55E+08	locus609.1	420.46	-	155462515	155463445	0,0,255	1	930	0
NC_030681.2	155517971	1.56E+08	locus611.1	403.48	+	155517971	155518874	0,0,255	1	903	0
NC_030681.2	155536170	1.56E+08	locus612.1	416.97	+	155536170	155537091	0,0,255	1	921	0
NC_030681.2	155548209	1.56E+08	locus613.1	402.88	+	155548209	155549094	0,0,255	1	885	0
NC_030681.2	155554970	1.56E+08	locus614.1	419.48	-	155554970	155555885	0,0,255	1	915	0
NC_030681.2	155571529	1.56E+08	locus615.1	406.98	-	155571529	155572393	0,0,255	1	864	0
NC_030681.2	155588287	1.56E+08	locus616.1	412.97	-	155588287	155589148	0,0,255	1	861	0
NC_030681.2	155623117	1.56E+08	locus618.1	414.46	-	155623117	155623978	0,0,255	1	861	0
NC_030681.2	155673182	1.56E+08	locus619.1	413.97	+	155673182	155674079	0,0,255	1	897	0
NC_030681.2	155683846	1.56E+08	locus620.1	422.37	-	155683846	155684713	0,0,255	1	867	0
NC_030681.2	155706852	1.56E+08	locus621.1	425.97	-	155706852	155707782	0,0,255	1	930	0
NC_030681.2	155733012	1.56E+08	locus622.1	419.98	-	155733012	155733927	0,0,255	1	915	0
NC_030681.2	155750174	1.56E+08	locus623.1	431.46	-	155750174	155751038	0,0,255	1	864	0
NC_030681.2	155772749	1.56E+08	locus624.1	418.47	-	155772749	155773607	0,0,255	1	858	0
NC_030681.2	155795189	1.56E+08	locus625.1	398.49	-	155795189	155796053	0,0,255	1	864	0
NC_030681.2	155806344	1.56E+08	locus626.1	423.47	-	155806344	155807256	0,0,255	1	912	0
NC_030681.2	155863270	1.56E+08	locus627.1	419.98	-	155863270	155864182	0,0,255	1	912	0
NC_030681.2	155893139	1.56E+08	locus628.1	428.97	-	155893139	155894000	0,0,255	1	861	0
NC_030681.2	155916395	1.56E+08	locus629.1	429.98	-	155916395	155917301	0,0,255	1	906	0
NC_030681.2	155934595	1.56E+08	locus630.1	405	-	155934595	155935510	0,0,255	1	915	0
NC_030681.2	155961910	1.56E+08	locus631.1	422.48	+	155961910	155962816	0,0,255	1	906	0
NC_030681.2	155982143	1.56E+08	locus632.1	424.98	+	155982143	155983055	0,0,255	1	912	0
NC_030681.2	156021443	1.56E+08	locus634.1	376.01	+	156021443	156022363	0,0,255	2	555,354	0,566
NC_030681.2	156049531	1.56E+08	locus635.1	416.48	+	156049531	156050419	0,0,255	1	888	0
NC_030681.2	156069827	1.56E+08	locus636.1	405.38	+	156069827	156070715	0,0,255	1	888	0
NC_030681.2	156084413	1.56E+08	locus637.1	424.35	+	156084413	156085334	0,0,255	1	921	0
NC_030681.2	156100820	1.56E+08	locus638.1	418.36	+	156100820	156101708	0,0,255	1	888	0
NC_030681.2	156139821	1.56E+08	locus639.1	409.98	+	156139821	156140715	0,0,255	1	894	0
NC_030681.2	156148158	1.56E+08	locus640.1	407.98	-	156148158	156149040	0,0,255	1	882	0
NC_030681.2	156207645	1.56E+08	locus641.1	412.48	+	156207645	156208530	0,0,255	1	885	0
NC_030681.2	156221196	1.56E+08	locus642.1	419.65	+	156221196	156222090	0,0,255	1	894	0
NC_030681.2	156238562	1.56E+08	locus643.1	396.74	+	156238562	156239411	0,0,255	1	849	0
NC_030681.2	156261594	1.56E+08	locus644.1	410.38	+	156261594	156262482	0,0,255	1	888	0
NC_030681.2	156282376	1.56E+08	locus645.1	402.98	+	156282376	156283264	0,0,255	1	888	0
NC_030681.2	156291784	1.56E+08	locus646.1	419.97	+	156291784	156292669	0,0,255	1	885	0
NC_030681.2	156311492	1.56E+08	locus647.1	413.47	+	156311492	156312377	0,0,255	1	885	0
NC_030681.2	156343383	1.56E+08	locus648.1	413.84	+	156343383	156344268	0,0,255	1	885	0
NC_030681.2	156374263	1.56E+08	locus649.1	412.48	+	156374263	156375148	0,0,255	1	885	0
NC_030681.2	156390154	1.56E+08	locus650.1	416.96	+	156390154	156391039	0,0,255	1	885	0
NC_030681.2	156449612	1.56E+08	locus651.1	409.98	+	156449612	156450494	0,0,255	1	882	0
NC_030681.2	156493612	1.56E+08	locus653.1	410.48	+	156493612	156494497	0,0,255	1	885	0

NC_030681.2	156531038	1.57E+08	locus654.1	415.97	+	156531038	156532187	0,0,255	1	1149	0
NC_030681.2	156565268	1.57E+08	locus655.1	412.35	+	156565268	156566150	0,0,255	1	882	0
NC_030681.2	156583720	1.57E+08	locus656.1	403.97	-	156583720	156584605	0,0,255	1	885	0
NC_030681.2	156612842	1.57E+08	locus657.1	405.47	-	156612842	156613727	0,0,255	1	885	0
NC_030681.2	156668846	1.57E+08	locus658.1	410.97	-	156668846	156669848	0,0,255	1	1002	0
NC_030681.2	156684798	1.57E+08	locus659.1	404.07	-	156684798	156685683	0,0,255	1	885	0
NC_030681.2	156715443	1.57E+08	locus660.1	403.48	-	156715443	156716262	0,0,255	1	819	0
NC_030681.2	156729671	1.57E+08	locus661.1	406.48	-	156729671	156730553	0,0,255	1	882	0
NC_030681.2	156744834	1.57E+08	locus662.1	420.97	-	156744834	156745722	0,0,255	1	888	0
NC_030681.2	156820331	1.57E+08	locus663.1	406.98	+	156820331	156821216	0,0,255	1	885	0
NC_030681.2	156840266	1.57E+08	locus664.1	406.47	-	156840266	156841151	0,0,255	1	885	0
NC_030681.2	156898140	1.57E+08	locus665.1	399.48	-	156898140	156899025	0,0,255	1	885	0
NC_030681.2	156926649	1.57E+08	locus666.1	399.98	-	156926649	156927534	0,0,255	1	885	0
NC_030681.2	156941228	1.57E+08	locus667.1	413.98	-	156941228	156942113	0,0,255	1	885	0
NC_030681.2	156972041	1.57E+08	locus668.1	410.48	-	156972041	156972926	0,0,255	1	885	0
NC_030681.2	157014199	1.57E+08	locus669.1	402.16	-	157014199	157015084	0,0,255	1	885	0
NC_030681.2	157046627	1.57E+08	locus670.1	418.46	-	157046627	157047521	0,0,255	1	894	0
NC_030681.2	157095759	1.57E+08	locus671.1	403.98	-	157095759	157096641	0,0,255	1	882	0
NC_030681.2	157140496	1.57E+08	locus672.1	412.97	-	157140496	157141378	0,0,255	1	882	0
NC_030681.2	157188927	1.57E+08	locus673.1	408.84	-	157188927	157189812	0,0,255	1	885	0
NC_030681.2	157214151	1.57E+08	locus674.1	412.48	-	157214151	157215111	0,0,255	1	960	0
NC_030681.2	157234518	1.57E+08	locus675.1	407.98	-	157234518	157235403	0,0,255	1	885	0
NC_030681.2	157256298	1.57E+08	locus676.1	413.64	-	157256298	157257183	0,0,255	1	885	0
NC_030681.2	157272283	1.57E+08	locus677.1	406.48	-	157272283	157273165	0,0,255	1	882	0
NC_030681.2	157305469	1.57E+08	locus678.1	413.47	-	157305469	157306354	0,0,255	1	885	0
NC_030681.2	157333314	1.57E+08	locus679.1	421.47	-	157333314	157334196	0,0,255	1	882	0
NC_030681.2	157369443	1.57E+08	locus680.1	413.47	-	157369443	157370328	0,0,255	1	885	0
NC_030681.2	157397213	1.57E+08	locus681.1	371.42	-	157397213	157398088	0,0,255	2	674,190	0,685
NC_030681.2	157467229	1.57E+08	locus682.1	403.49	-	157467229	157468117	0,0,255	1	888	0
NC_030681.2	157492880	1.57E+08	locus683.1	411.36	-	157492880	157493744	0,0,255	1	864	0
NC_030681.2	157507457	1.58E+08	locus684.1	417.48	-	157507457	157508351	0,0,255	1	894	0
NC_030681.2	157526766	1.58E+08	locus685.1	413.47	-	157526766	157527657	0,0,255	1	891	0
NC_030681.2	157564389	1.58E+08	locus686.1	410.48	+	157564389	157565271	0,0,255	1	882	0
NC_030681.2	157601956	1.58E+08	locus687.1	418.97	+	157601956	157602841	0,0,255	1	885	0
NC_030681.2	157611434	1.58E+08	locus688.1	408.98	-	157611434	157612322	0,0,255	1	888	0
NC_030681.2	157650119	1.58E+08	locus689.1	415.86	-	157650119	157651007	0,0,255	1	888	0
NC_030681.2	157664628	1.58E+08	locus690.1	423.35	-	157664628	157665549	0,0,255	1	921	0
NC_030681.2	157678611	1.58E+08	locus691.1	406.17	-	157678611	157679604	0,0,255	1	993	0
NC_030681.2	157699836	1.58E+08	locus692.1	416.98	-	157699836	157700724	0,0,255	1	888	0
NC_030681.2	157785646	1.58E+08	locus695.1	422.48	-	157785646	157786558	0,0,255	1	912	0
NC_030681.2	157805793	1.58E+08	locus696.1	422.98	-	157805793	157806699	0,0,255	1	906	0
NC_030681.2	157833133	1.58E+08	locus697.1	406	+	157833133	157833997	0,0,255	1	864	0
NC_030681.2	157905071	1.58E+08	locus698.1	419.98	+	157905071	157905983	0,0,255	1	912	0
NC_030681.2	157970739	1.58E+08	locus699.1	407.49	+	157970739	157971603	0,0,255	1	864	0

NC_030681.2	157993126	1.58E+08	locus700.1	421.47	+	157993126	157993984	0,0,255	1	858	0
NC_030681.2	158015775	1.58E+08	locus701.1	431.46	+	158015775	158016639	0,0,255	1	864	0
NC_030681.2	158032917	1.58E+08	locus702.1	419.98	+	158032917	158033832	0,0,255	1	915	0
NC_030681.2	158054736	1.58E+08	locus703.1	427.97	+	158054736	158055666	0,0,255	1	930	0
NC_030681.2	158077522	1.58E+08	locus704.1	421.38	+	158077522	158078389	0,0,255	1	867	0
NC_030681.2	158087565	1.58E+08	locus705.1	413.97	-	158087565	158088462	0,0,255	1	897	0
NC_030681.2	158138791	1.58E+08	locus706.1	415.47	+	158138791	158139652	0,0,255	1	861	0
NC_030681.2	158174060	1.58E+08	locus708.1	414.47	+	158174060	158174921	0,0,255	1	861	0
NC_030681.2	158194035	1.58E+08	locus709.1	406.48	+	158194035	158194899	0,0,255	1	864	0
NC_030681.2	158208129	1.58E+08	locus710.1	421.97	+	158208129	158209044	0,0,255	1	915	0
NC_030681.2	158220481	1.58E+08	locus711.1	401.88	-	158220481	158221366	0,0,255	1	885	0
NC_030681.2	158232497	1.58E+08	locus712.1	414.97	-	158232497	158233418	0,0,255	1	921	0
NC_030681.2	158251920	1.58E+08	locus713.1	401.85	-	158251920	158252805	0,0,255	1	885	0
NC_030681.2	158263521	1.58E+08	locus714.1	415.96	-	158263521	158264439	0,0,255	1	918	0
NC_030681.2	158301563	1.58E+08	locus715.1	425.47	+	158301563	158302493	0,0,255	1	930	0
NC_030681.2	158322953	1.58E+08	locus716.1	417.97	+	158322953	158323883	0,0,255	1	930	0
NC_030681.2	158346689	1.58E+08	locus717.1	423.96	+	158346689	158347619	0,0,255	1	930	0
NC_030681.2	158364958	1.58E+08	locus718.1	410.48	+	158364958	158365888	0,0,255	1	930	0
NC_030681.2	158402803	1.58E+08	locus720.1	415.98	+	158402803	158403733	0,0,255	1	930	0
NC_030681.2	158421225	1.58E+08	locus721.1	416.47	+	158421225	158422155	0,0,255	1	930	0
NC_030681.2	158437203	1.58E+08	locus722.1	422.48	+	158437203	158438133	0,0,255	1	930	0
NC_030681.2	158452420	1.58E+08	locus723.1	430.45	+	158452420	158453350	0,0,255	1	930	0
NC_030681.2	158487897	1.58E+08	locus725.1	424.96	+	158487897	158488704	0,0,255	1	807	0
NC_030681.2	158507316	1.59E+08	locus726.1	423.97	+	158507316	158508231	0,0,255	1	915	0
NC_030681.2	158534968	1.59E+08	locus727.1	429.96	+	158534968	158535898	0,0,255	1	930	0
NC_030681.2	158607532	1.59E+08	locus728.1	425.47	+	158607532	158608462	0,0,255	1	930	0
NC_030681.2	158640124	1.59E+08	locus729.1	424.47	+	158640124	158640952	0,0,255	1	828	0
NC_030681.2	158695737	1.59E+08	locus730.1	349.89	+	158695737	158696547	0,0,255	1	810	0
NC_030681.2	158757522	1.59E+08	locus731.1	405.98	+	158757522	158758488	0,0,255	1	966	0
NC_030681.2	158773404	1.59E+08	locus732.1	416.98	+	158773404	158774259	0,0,255	1	855	0
NC_030681.2	158823975	1.59E+08	locus734.1	418.96	+	158823975	158824905	0,0,255	1	930	0
NC_030681.2	158838710	1.59E+08	locus735.1	422.96	+	158838710	158839667	0,0,255	1	957	0
NC_030681.2	158852936	1.59E+08	locus736.1	422.46	+	158852936	158853719	0,0,255	1	783	0
NC_030681.2	158859137	1.59E+08	locus737.1	415.46	-	158859137	158860085	0,0,255	1	948	0
NC_030681.2	158885939	1.59E+08	locus738.1	419.96	-	158885939	158886824	0,0,255	1	885	0
NC_030681.2	158939322	1.59E+08	locus739.1	416.46	+	158939322	158940219	0,0,255	1	897	0
NC_030681.2	158972669	1.59E+08	locus741.1	413.96	+	158972669	158973716	0,0,255	1	1047	0
NC_030681.2	159234119	1.59E+08	locus742.1	410.47	-	159234119	159235007	0,0,255	1	888	0
NC_030681.2	159253212	1.59E+08	locus743.1	412.66	-	159253212	159254094	0,0,255	1	882	0
NC_030681.2	159274453	1.59E+08	locus744.1	411.47	-	159274453	159275335	0,0,255	1	882	0
NC_030681.2	159290872	1.59E+08	locus745.1	411.9	-	159290872	159291884	0,0,255	2	875,7	0,1005
NC_030681.2	159357840	1.59E+08	locus746.1	419.47	+	159357840	159358722	0,0,255	1	882	0
NC_030681.2	159372800	1.59E+08	locus747.1	413.47	+	159372800	159373682	0,0,255	1	882	0
NC_030681.2	159391780	1.59E+08	locus748.1	411.98	+	159391780	159392662	0,0,255	1	882	0

NC_030681.2	159410028	1.59E+08	locus749.1	411.66	+	159410028	159410910	0,0,255	1	882	0
NC_030681.2	159423069	1.59E+08	locus750.1	403.98	+	159423069	159423888	0,0,255	1	819	0
NC_030681.2	159441586	1.59E+08	locus751.1	401.42	+	159441586	159442465	0,0,255	1	879	0
NC_030681.2	159458246	1.59E+08	locus752.1	410.97	+	159458246	159459128	0,0,255	1	882	0
NC_030681.2	159476719	1.59E+08	locus753.1	409.47	+	159476719	159477607	0,0,255	1	888	0
NC_030681.2	160299084	1.6E+08	locus754.1	398.48	+	160299084	160299957	0,0,255	1	873	0
NC_030681.2	160339058	1.6E+08	locus755.1	400.97	+	160339058	160339976	0,0,255	1	918	0
NC_030681.2	160350807	1.6E+08	locus756.1	415.98	-	160350807	160351680	0,0,255	1	873	0
NC_030681.2	160389885	1.6E+08	locus757.1	394.48	+	160389885	160390758	0,0,255	1	873	0
NC_030681.2	160421331	1.6E+08	locus758.1	409.47	+	160421331	160422204	0,0,255	1	873	0
NC_030681.2	160430947	1.6E+08	locus759.1	398.48	+	160430947	160431820	0,0,255	1	873	0
NC_030681.2	160447076	1.6E+08	locus760.1	402.47	+	160447076	160447949	0,0,255	1	873	0
NC_030681.2	160463911	1.6E+08	locus761.1	402.47	+	160463911	160464784	0,0,255	1	873	0
NC_030681.2	160479263	1.6E+08	locus762.1	402.47	+	160479263	160480136	0,0,255	1	873	0
NC_030681.2	160497053	1.6E+08	locus763.1	407.48	+	160497053	160497974	0,0,255	1	921	0
NC_030681.2	160533515	1.61E+08	locus764.1	403.97	+	160533515	160534457	0,0,255	1	942	0
NC_030681.2	160556685	1.61E+08	locus765.1	401.97	+	160556685	160557558	0,0,255	1	873	0
NC_030681.2	160566538	1.61E+08	locus766.1	402.47	-	160566538	160567411	0,0,255	1	873	0
NC_030681.2	160595058	1.61E+08	locus767.1	401.47	-	160595058	160596000	0,0,255	1	942	0
NC_030681.2	160633412	1.61E+08	locus768.1	404.97	-	160633412	160634285	0,0,255	1	873	0
NC_030681.2	160654222	1.61E+08	locus769.1	399.48	-	160654222	160655095	0,0,255	1	873	0
NC_030681.2	160685334	1.61E+08	locus770.1	401.46	-	160685334	160686207	0,0,255	1	873	0
NC_030681.2	160714142	1.61E+08	locus771.1	400.98	-	160714142	160715084	0,0,255	1	942	0
NC_030681.2	160754668	1.61E+08	locus772.1	403.97	-	160754668	160755610	0,0,255	1	942	0
NC_030681.2	160785237	1.61E+08	locus773.1	400.97	-	160785237	160786110	0,0,255	1	873	0
NC_030681.2	160800059	1.61E+08	locus774.1	400.98	-	160800059	160800932	0,0,255	1	873	0
NC_030681.2	160881173	1.61E+08	locus776.1	400.97	+	160881173	160882115	0,0,255	1	942	0
NC_030681.2	160934621	1.61E+08	locus778.1	399.48	+	160934621	160935494	0,0,255	1	873	0
NC_030681.2	160970410	1.61E+08	locus779.1	393.98	+	160970410	160971283	0,0,255	1	873	0
NC_030681.2	160997296	1.61E+08	locus780.1	398.47	+	160997296	160998169	0,0,255	1	873	0
NC_030681.2	161951424	1.62E+08	locus781.1	467.36	-	161951424	161952333	0,0,255	1	909	0
NC_030681.2	162768620	1.63E+08	locus782.1	710.9	-	162768620	162769517	0,0,255	1	897	0
NC_030681.2	162797056	1.63E+08	locus783.1	705.9	-	162797056	162797902	0,0,255	1	846	0
NC_030681.2	162852634	1.63E+08	locus785.1	709.4	+	162852634	162853480	0,0,255	1	846	0
NC_030681.2	163387067	1.63E+08	locus786.1	406.41	-	163387067	163387985	0,0,255	1	918	0
NC_030681.2	163806574	1.64E+08	locus787.1	613.69	-	163806574	163820415	0,0,255	3	91,827,9	0,232,13832
NC_030682.2	133471	134397	locus788.1	612.39	+	133471	134397	0,0,255	2	30,885	0,41
NC_030683.2	102962369	1.03E+08	locus847.1	574.59	+	102962369	102963374	0,0,255	2	692,259	0,746
NC_030683.2	103145211	1.03E+08	locus848.1	571.99	-	103145211	103146132	0,0,255	1	921	0
NC_030683.2	103161507	1.03E+08	locus849.1	569.92	-	103161507	103162431	0,0,255	2	250,620	0,304
NC_030683.2	103178607	1.03E+08	locus850.1	694.41	-	103178607	103179531	0,0,255	1	924	0
NC_030683.2	103205220	1.03E+08	locus852.1	559.11	-	103205220	103206099	0,0,255	2	241,584	0,295
NC_030683.2	103234456	1.03E+08	locus853.1	580.91	-	103234456	103235323	0,0,255	2	247,566	0,301
NC_030683.2	103239411	1.03E+08	locus854.1	561.61	-	103239411	103240359	0,0,255	2	247,647	0,301

NC_030683.2	103252046	1.03E+08	locus855.1	569.92	-	103252046	103252931	0,0,255	2	247,584	0,301
NC_030683.2	103264154	1.03E+08	locus856.1	572.09	-	103264154	103265039	0,0,255	2	247,584	0,301
NC_030683.2	103274702	1.03E+08	locus857.1	649.88	-	103274702	103275575	0,0,255	2	253,566	0,307
NC_030683.2	103292091	1.03E+08	locus858.1	587.9	-	103292091	103293078	0,0,255	2	274,659	0,328
NC_030683.2	103314139	1.03E+08	locus860.1	585.17	-	103314139	103315072	0,0,255	2	247,632	0,301
NC_030683.2	103330636	1.03E+08	locus862.1	584.96	-	103330636	103331533	0,0,255	2	259,584	0,313
NC_030683.2	103342877	1.03E+08	locus863.1	575.09	-	103342877	103343813	0,0,255	2	259,623	0,313
NC_030683.2	103357233	1.03E+08	locus864.1	445.53	-	103357233	103358124	0,0,255	2	271,566	0,325
NC_030683.2	103369450	1.03E+08	locus865.1	444.2	-	103369450	103370341	0,0,255	2	271,566	0,325
NC_030683.2	103380080	1.03E+08	locus866.1	436.71	-	103380080	103380971	0,0,255	2	271,566	0,325
NC_030683.2	103388898	1.03E+08	locus867.1	445.21	-	103388898	103389789	0,0,255	2	271,566	0,325
NC_030684.2	1271851	1272634	locus875.1	668.98	-	1271851	1272634	0,0,255	1	783	0
NC_030684.2	64857632	64858601	locus890.1	398.45	+	64857632	64858601	0,0,255	1	969	0
NC_030684.2	120151819	1.2E+08	locus903.1	437.88	-	120151819	120152740	0,0,255	1	921	0
NC_030684.2	120266315	1.2E+08	locus905.1	401.37	+	120266315	120267224	0,0,255	1	909	0
NC_030684.2	120272888	1.2E+08	locus906.1	395.88	-	120272888	120273809	0,0,255	1	921	0
NC_030684.2	120367889	1.2E+08	locus908.1	674.92	+	120367889	120368780	0,0,255	1	891	0
NC_030684.2	120508348	1.21E+08	locus909.1	438.89	+	120508348	120509248	0,0,255	1	900	0
NC_030684.2	120562151	1.21E+08	locus910.1	436.38	+	120562151	120563081	0,0,255	1	930	0
NC_030684.2	120584772	1.21E+08	locus911.1	439.38	+	120584772	120585672	0,0,255	1	900	0
NC_030684.2	120609590	1.21E+08	locus912.1	429.39	+	120609590	120610520	0,0,255	1	930	0
NC_030684.2	120633374	1.21E+08	locus913.1	420.14	+	120633374	120634268	0,0,255	1	894	0
NC_030684.2	120655966	1.21E+08	locus914.1	437.37	+	120655966	120656923	0,0,255	1	957	0
NC_030684.2	120758816	1.21E+08	locus915.1	420.89	+	120758816	120759713	0,0,255	1	897	0
NC_030684.2	120799205	1.21E+08	locus916.1	420.9	+	120799205	120800102	0,0,255	1	897	0
NC_030684.2	120840845	1.21E+08	locus917.1	407.9	+	120840845	120841742	0,0,255	1	897	0
NC_030684.2	120878982	1.21E+08	locus918.1	411.9	+	120878982	120879879	0,0,255	1	897	0
NC_030684.2	120921959	1.21E+08	locus919.1	409.9	+	120921959	120922856	0,0,255	1	897	0
NC_030684.2	120941613	1.21E+08	locus920.1	412.4	+	120941613	120942510	0,0,255	1	897	0
NC_030684.2	121006034	1.21E+08	locus922.1	431.9	+	121006034	121006931	0,0,255	1	897	0
NC_030684.2	121030642	1.21E+08	locus923.1	421.4	+	121030642	121031539	0,0,255	1	897	0
NC_030684.2	121062894	1.21E+08	locus924.1	422.89	+	121062894	121063797	0,0,255	1	903	0
NC_030684.2	122053742	1.22E+08	locus926.1	440.38	+	122053742	122054630	0,0,255	1	888	0
NC_030684.2	122093658	1.22E+08	locus927.1	447.37	+	122093658	122094546	0,0,255	1	888	0
NC_030684.2	122132340	1.22E+08	locus928.1	448.36	+	122132340	122133228	0,0,255	1	888	0
NC_030684.2	122167756	1.22E+08	locus929.1	450.36	+	122167756	122168644	0,0,255	1	888	0
NC_030684.2	122185836	1.22E+08	locus930.1	450.86	+	122185836	122186724	0,0,255	1	888	0
NC_030684.2	122205002	1.22E+08	locus931.1	447.37	+	122205002	122205890	0,0,255	1	888	0
NC_030684.2	122222015	1.22E+08	locus932.1	440.86	+	122222015	122222903	0,0,255	1	888	0
NC_030684.2	122268448	1.22E+08	locus933.1	446.86	+	122268448	122269330	0,0,255	1	882	0
NC_030684.2	122343970	1.22E+08	locus934.1	459.85	+	122343970	122344870	0,0,255	1	900	0
NC_030684.2	122360722	1.22E+08	locus935.1	453.35	+	122360722	122361613	0,0,255	1	891	0
NC_030684.2	122383303	1.22E+08	locus936.1	449.37	-	122383303	122384206	0,0,255	1	903	0
NC_030684.2	122409955	1.22E+08	locus937.1	456.86	-	122409955	122410858	0,0,255	1	903	0

NC_030684.2	122431682	1.22E+08	locus938.1	453.86	-	122431682	122432585	0,0,255	1	903	0
NC_030684.2	122447326	1.22E+08	locus939.1	465.36	-	122447326	122448229	0,0,255	1	903	0
NC_030684.2	122462640	1.22E+08	locus940.1	410.16	-	122462640	122463537	0,0,255	1	897	0
NC_030684.2	122472585	1.22E+08	locus941.1	396.66	-	122472585	122473509	0,0,255	1	924	0
NC_030684.2	123196134	1.23E+08	locus942.1	696.76	-	123196134	123197022	0,0,255	1	888	0
NC_030684.2	123225062	1.23E+08	locus943.1	400.39	-	123225062	123226001	0,0,255	1	939	0
NC_030684.2	123242839	1.23E+08	locus944.1	395.08	-	123242839	123243616	0,0,255	1	777	0
NC_030684.2	123272794	1.23E+08	locus945.1	400.07	-	123272794	123273586	0,0,255	1	792	0
NC_030684.2	123299228	1.23E+08	locus946.1	389.08	-	123299228	123300146	0,0,255	1	918	0
NC_030684.2	123335285	1.23E+08	locus947.1	398.08	-	123335285	123336203	0,0,255	1	918	0
NC_030684.2	123445890	1.23E+08	locus948.1	632.34	-	123445890	123446796	0,0,255	1	906	0
NC_030684.2	123481506	1.23E+08	locus950.1	678.32	+	123481506	123482607	0,0,255	1	1101	0
NC_030684.2	123503503	1.24E+08	locus951.1	671.32	+	123503503	123504517	0,0,255	1	1014	0
NC_030684.2	123534002	1.24E+08	locus952.1	676.83	+	123534002	123534911	0,0,255	1	909	0
NC_030684.2	123561352	1.24E+08	locus953.1	667.32	+	123561352	123562432	0,0,255	1	1080	0
NC_030684.2	123568064	1.24E+08	locus955.1	639.83	-	123568064	123568961	0,0,255	1	897	0
NC_030684.2	123603898	1.24E+08	locus957.1	684.81	+	123603898	123604807	0,0,255	1	909	0
NC_030684.2	123611296	1.24E+08	locus958.1	641.84	-	123611296	123612202	0,0,255	1	906	0
NC_030684.2	123625639	1.24E+08	locus959.1	670.82	-	123625639	123626548	0,0,255	1	909	0
NC_030684.2	123642642	1.24E+08	locus960.1	674.32	-	123642642	123643551	0,0,255	1	909	0
NC_030684.2	123665314	1.24E+08	locus961.1	667.32	-	123665314	123666226	0,0,255	1	912	0
NC_030684.2	123693946	1.24E+08	locus962.1	671.82	-	123693946	123694948	0,0,255	1	1002	0
NC_030684.2	123719758	1.24E+08	locus963.1	680.82	-	123719758	123720757	0,0,255	1	999	0
NC_030684.2	123742969	1.24E+08	locus964.1	666.32	-	123742969	123744058	0,0,255	1	1089	0
NC_030684.2	123770066	1.24E+08	locus965.1	667.32	-	123770066	123770978	0,0,255	1	912	0
NC_030684.2	123789471	1.24E+08	locus966.1	674.82	-	123789471	123790383	0,0,255	1	912	0
NC_030684.2	123822532	1.24E+08	locus967.1	679.82	-	123822532	123823444	0,0,255	1	912	0
NC_030684.2	123845006	1.24E+08	locus968.1	667.33	-	123845006	123845918	0,0,255	1	912	0
NC_030684.2	123873896	1.24E+08	locus969.1	672.82	-	123873896	123874805	0,0,255	1	909	0
NC_030684.2	123891709	1.24E+08	locus971.1	666.33	-	123891709	123892798	0,0,255	1	1089	0
NC_030684.2	123913965	1.24E+08	locus972.1	659.33	-	123913965	123914988	0,0,255	1	1023	0
NC_030684.2	123933769	1.24E+08	locus973.1	673.32	-	123933769	123934678	0,0,255	1	909	0
NC_030684.2	123956079	1.24E+08	locus974.1	672.32	-	123956079	123956988	0,0,255	1	909	0
NC_030684.2	123989850	1.24E+08	locus975.1	669.82	-	123989850	123990747	0,0,255	1	897	0
NC_030684.2	124008005	1.24E+08	locus976.1	667.82	-	124008005	124008911	0,0,255	1	906	0
NC_030684.2	124050641	1.24E+08	locus977.1	655.75	+	124050641	124051454	0,0,255	1	813	0
NC_030684.2	124073126	1.24E+08	locus978.1	701.21	+	124073126	124073939	0,0,255	1	813	0
NC_030684.2	124099565	1.24E+08	locus979.1	627.83	+	124099565	124100507	0,0,255	1	942	0
NC_030684.2	124120524	1.24E+08	locus980.1	402.3	+	124120524	124121448	0,0,255	1	924	0
NC_030684.2	124141358	1.24E+08	locus981.1	398.81	+	124141358	124142243	0,0,255	1	885	0
NC_030684.2	124161509	1.24E+08	locus982.1	379.33	+	124161509	124162430	0,0,255	1	921	0
NC_030684.2	124186313	1.24E+08	locus983.1	379.24	+	124186313	124187291	0,0,255	1	978	0
NC_030684.2	124199647	1.24E+08	locus984.1	374.32	+	124199647	124200568	0,0,255	1	921	0
NC_030684.2	124217234	1.24E+08	locus985.1	371.89	+	124217234	124218158	0,0,255	1	924	0

NC_030684.2	124238497	1.24E+08	locus986.1	375.89	+	124238497	124239421	0,0,255	1	924	0
NC_030684.2	124268262	1.24E+08	locus987.1	377.31	+	124268262	124269186	0,0,255	1	924	0
NC_030684.2	124300972	1.24E+08	locus988.1	387.89	+	124300972	124301896	0,0,255	1	924	0
NC_030684.2	124319645	1.24E+08	locus989.1	385.21	+	124319645	124320533	0,0,255	1	888	0
NC_030684.2	124341900	1.24E+08	locus990.1	385.32	+	124341900	124342821	0,0,255	1	921	0
NC_030684.2	124365525	1.24E+08	locus991.1	391.23	+	124365525	124366452	0,0,255	1	927	0
NC_030684.2	124394031	1.24E+08	locus992.1	381.81	+	124394031	124394940	0,0,255	1	909	0
NC_030684.2	124408758	1.24E+08	locus993.1	401.87	-	124408758	124409667	0,0,255	1	909	0
NC_030684.2	124439478	1.24E+08	locus994.1	431.82	-	124439478	124440378	0,0,255	1	900	0
NC_030684.2	124471663	1.24E+08	locus995.1	420.81	-	124471663	124472563	0,0,255	1	900	0
NC_030684.2	124506771	1.25E+08	locus997.1	665.42	+	124506771	124507602	0,0,255	1	831	0
NC_030684.2	124514272	1.25E+08	locus998.1	420.32	-	124514272	124515172	0,0,255	1	900	0
NC_030684.2	124525548	1.25E+08	locus999.1	434.06	-	124525548	124526448	0,0,255	1	900	0
NC_030684.2	124542568	1.25E+08	locus1000.1	704.23	-	124542568	124543459	0,0,255	1	891	0
NC_030684.2	124557985	1.25E+08	locus1001.1	715.81	-	124557985	124558879	0,0,255	1	894	0
NC_030684.2	124632930	1.25E+08	locus1003.1	709.06	-	124632930	124633830	0,0,255	1	900	0
NC_030684.2	124657304	1.25E+08	locus1004.1	688.56	-	124657304	124658204	0,0,255	1	900	0
NC_030684.2	124672716	1.25E+08	locus1005.1	606.12	-	124672716	124673637	0,0,255	1	921	0
NC_030684.2	124676823	1.25E+08	locus1006.1	606.12	-	124676823	124677744	0,0,255	1	921	0
NC_030684.2	124727027	1.25E+08	locus1007.1	701.91	-	124727027	124727921	0,0,255	1	894	0
NC_030684.2	124754575	1.25E+08	locus1009.1	693.91	-	124754575	124755469	0,0,255	1	894	0
NC_030684.2	124770146	1.25E+08	locus1011.1	673.85	-	124770146	124771046	0,0,255	1	900	0
NC_030684.2	125117771	1.25E+08	locus1014.1	702.41	+	125117771	125118665	0,0,255	1	894	0
NC_030684.2	127798466	1.28E+08	locus1017.1	611.84	-	127798466	127799336	0,0,255	1	870	0
NC_030684.2	127817942	1.28E+08	locus1018.1	607.35	-	127817942	127818812	0,0,255	1	870	0
NC_030684.2	127830771	1.28E+08	locus1019.1	610.84	-	127830771	127831719	0,0,255	1	948	0
NC_030684.2	127843652	1.28E+08	locus1020.1	609.35	-	127843652	127844588	0,0,255	1	936	0
NC_030684.2	127856269	1.28E+08	locus1021.1	615.84	-	127856269	127857205	0,0,255	1	936	0
NC_030684.2	127876097	1.28E+08	locus1022.1	602.35	-	127876097	127877033	0,0,255	1	936	0
NC_030684.2	127897746	1.28E+08	locus1023.1	619.84	-	127897746	127898682	0,0,255	1	936	0
NC_030685.2	16235	17110	locus1028.1	630.37	-	16235	17110	0,0,255	2	834,30	0,845
NC_030686.2	52381478	52388307	locus1062.1	636.09	-	52381478	52388307	0,0,255	2	876,9	0,6820

Caecilian

CABPRQ010(	27082	27886	locus46850.1	682.52	-	27082	27886	0,0,255	1	804	0
LR699147.1	250585478	250586369	locus10029.1	724.98	-	250585478	250586369	0,0,255	1	891	0
LR699150.1	90409978	90410893	locus22632.1	737.78	-	90409978	90410893	0,0,255	1	915	0
LR699154.1	160605649	160606597	locus34699.1	793.96	-	160605649	160606597	0,0,255	1	948	0
LR699154.1	161065784	161066675	locus34702.1	706.47	+	161065784	161066675	0,0,255	1	891	0
LR699155.1	119403663	119404602	locus36723.1	768.65	-	119403663	119404602	0,0,255	1	939	0
LR699155.1	119464874	119465780	locus36726.1	741.42	-	119464874	119465780	0,0,255	1	906	0
LR699155.1	119507874	119508780	locus36727.1	742.47	+	119507874	119508780	0,0,255	1	906	0
LR699155.1	119547583	119548480	locus36728.1	733.6	+	119547583	119548480	0,0,255	1	897	0
LR699155.1	119571081	119572020	locus36730.1	762.85	+	119571081	119572020	0,0,255	1	939	0
LR699155.1	119656359	119657253	locus36732.1	731.07	+	119656359	119657253	0,0,255	1	894	0
LR699155.1	119715611	119716505	locus36733.1	729.17	+	119715611	119716505	0,0,255	1	894	0
LR699155.1	119764824	119765718	locus36734.1	732.67	+	119764824	119765718	0,0,255	1	894	0
LR699155.1	119808317	119809211	locus36735.1	735.65	+	119808317	119809211	0,0,255	1	894	0
LR699155.1	119846364	119847258	locus36736.1	729.91	+	119846364	119847258	0,0,255	1	894	0
LR699155.1	119901002	119901797	locus36738.1	640.16	+	119901002	119901797	0,0,255	1	795	0
LR699155.1	120040733	120041627	locus36740.1	732.32	+	120040733	120041627	0,0,255	1	894	0
LR699155.1	120138907	120139801	locus36742.1	731.04	+	120138907	120139801	0,0,255	1	894	0
LR699155.1	120235603	120236497	locus36743.1	732.56	+	120235603	120236497	0,0,255	1	894	0
LR699155.1	120291050	120291944	locus36744.1	725.55	+	120291050	120291944	0,0,255	1	894	0
LR699155.1	130972543	130973506	locus36860.1	751.11	-	130972543	130973506	0,0,255	1	963	0
LR699155.1	131064468	131065407	locus36862.1	760.99	+	131064468	131065407	0,0,255	1	939	0
LR699155.1	134146095	134147004	locus36882.1	733.24	-	134146095	134147004	0,0,255	1	909	0

LR699155.1	134186404	134187313	locus36883.1	732.41	-	134186404	134187313	0,0,255	1	909	0
LR699155.1	134230892	134231801	locus36884.1	732.41	-	134230892	134231801	0,0,255	1	909	0
LR699155.1	134283980	134284889	locus36885.1	731.12	-	134283980	134284889	0,0,255	1	909	0
LR699155.1	134330989	134331898	locus36886.1	735.73	-	134330989	134331898	0,0,255	1	909	0
LR699155.1	134429384	134430296	locus36888.1	739.78	-	134429384	134430296	0,0,255	1	912	0
LR699155.1	134522068	134522977	locus36891.1	734.17	-	134522068	134522977	0,0,255	1	909	0
LR699155.1	134566990	134567899	locus36892.1	731.67	-	134566990	134567899	0,0,255	1	909	0
LR699155.1	134756206	134757115	locus36893.1	731.67	-	134756206	134757115	0,0,255	1	909	0
LR699155.1	134803810	134804719	locus36895.1	731.12	-	134803810	134804719	0,0,255	1	909	0
LR699155.1	134837467	134838379	locus36896.1	737.01	-	134837467	134838379	0,0,255	1	912	0
LR699155.1	134875233	134876142	locus36898.1	734.94	-	134875233	134876142	0,0,255	1	909	0
LR699155.1	134979490	134980399	locus36900.1	734.19	-	134979490	134980399	0,0,255	1	909	0
LR699155.1	135520728	135521622	locus36907.1	733.12	+	135520728	135521622	0,0,255	1	894	0
LR699155.1	135560679	135561573	locus36908.1	733.25	+	135560679	135561573	0,0,255	1	894	0
LR699155.1	135595258	135596161	locus36909.1	737.13	+	135595258	135596161	0,0,255	1	903	0
LR699155.1	135626048	135626942	locus36910.1	734.05	+	135626048	135626942	0,0,255	1	894	0
LR699155.1	135653943	135654846	locus36911.1	738.91	+	135653943	135654846	0,0,255	1	903	0
LR699155.1	135664608	135665511	locus36912.1	740.82	-	135664608	135665511	0,0,255	1	903	0
LR699155.1	135711963	135712857	locus36913.1	733.92	-	135711963	135712857	0,0,255	1	894	0
LR699155.1	135738061	135738955	locus36915.1	734.03	-	135738061	135738955	0,0,255	1	894	0
LR699155.1	135907700	135908600	locus36917.1	735.49	-	135907700	135908600	0,0,255	1	900	0
LR699155.1	136253768	136254662	locus36920.1	739.05	-	136253768	136254662	0,0,255	1	894	0
LR699155.1	136293371	136294265	locus36921.1	737.63	-	136293371	136294265	0,0,255	1	894	0
LR699155.1	136417898	136418792	locus36926.1	728.34	-	136417898	136418792	0,0,255	1	894	0

LR699155.1	136464911	136465832	locus36928.1	750.97	-	136464911	136465832	0,0,255	1	921	0
LR699155.1	136515857	136516778	locus36931.1	751.82	-	136515857	136516778	0,0,255	1	921	0
LR699155.1	136596668	136597589	locus36934.1	748.68	-	136596668	136597589	0,0,255	1	921	0
LR699155.1	136647593	136648514	locus36937.1	749.62	-	136647593	136648514	0,0,255	1	921	0
LR699155.1	136711929	136712850	locus36939.1	757.58	+	136711929	136712850	0,0,255	1	921	0
LR699155.1	136752845	136753766	locus36940.1	749.95	+	136752845	136753766	0,0,255	1	921	0
LR699155.1	136818561	136819482	locus36942.1	747.39	+	136818561	136819482	0,0,255	1	921	0
LR699155.1	136867269	136868163	locus36943.1	734.31	+	136867269	136868163	0,0,255	1	894	0
LR699155.1	136924397	136925291	locus36944.1	732.94	+	136924397	136925291	0,0,255	1	894	0
LR699155.1	137012054	137012948	locus36946.1	734.5	+	137012054	137012948	0,0,255	1	894	0
LR699155.1	137037904	137038798	locus36947.1	736.11	+	137037904	137038798	0,0,255	1	894	0
LR699155.1	137064077	137064971	locus36948.1	732.87	+	137064077	137064971	0,0,255	1	894	0
LR699155.1	137136386	137137211	locus36949.1	704.06	+	137136386	137137211	0,0,255	1	825	0
LR699155.1	137228543	137229437	locus36950.1	731.68	+	137228543	137229437	0,0,255	1	894	0
LR699155.1	137281666	137282560	locus36951.1	724.89	+	137281666	137282560	0,0,255	1	894	0
LR699155.1	137325379	137326264	locus36954.1	729.09	+	137325379	137326264	0,0,255	1	885	0
LR699155.1	137366651	137367545	locus36955.1	735.19	+	137366651	137367545	0,0,255	1	894	0
LR699155.1	137458179	137459073	locus36959.1	734.7	-	137458179	137459073	0,0,255	1	894	0
LR699155.1	137472008	137472890	locus36960.1	721.13	-	137472008	137472890	0,0,255	1	882	0
LR699155.1	137523138	137524032	locus36962.1	731.98	-	137523138	137524032	0,0,255	1	894	0
LR699155.1	137553143	137554037	locus36963.1	732.51	-	137553143	137554037	0,0,255	1	894	0
LR699155.1	137767704	137768613	locus36974.1	745.84	+	137767704	137768613	0,0,255	1	909	0
LR699155.1	137790050	137790959	locus36976.1	746.2	+	137790050	137790959	0,0,255	1	909	0
LR699155.1	137840577	137841486	locus36981.1	746.2	+	137840577	137841486	0,0,255	1	909	0

LR699157.1	95062657	95063512	locus40274.1	694.58	+	95062657	95063512	0,0,255	1	855	0
LR699157.1	95113206	95114118	locus40276.1	752.94	+	95113206	95114118	0,0,255	1	912	0
LR699157.1	95227104	95228025	locus40278.1	759.19	-	95227104	95228025	0,0,255	1	921	0
LR699157.1	95537468	95538362	locus40280.1	748.19	-	95537468	95538362	0,0,255	1	894	0
LR699157.1	95669367	95670261	locus40281.1	730.55	-	95669367	95670261	0,0,255	1	894	0
LR699157.1	95806893	95807787	locus40283.1	733.65	-	95806893	95807787	0,0,255	1	894	0
LR699157.1	95911461	95912373	locus40284.1	746.6	-	95911461	95912373	0,0,255	1	912	0
LR699157.1	96085334	96086237	locus40286.1	738.18	-	96085334	96086237	0,0,255	1	903	0
LR699157.1	96327021	96327939	locus40288.1	763.81	+	96327021	96327939	0,0,255	1	918	0
LR699157.1	96547458	96548340	locus40291.1	721.99	+	96547458	96548340	0,0,255	1	882	0
LR699157.1	97245280	97246174	locus40294.1	734.61	+	97245280	97246174	0,0,255	1	894	0
LR699157.1	97482784	97483696	locus40296.1	753.26	-	97482784	97483696	0,0,255	1	912	0
LR699157.1	97702293	97703307	locus40297.1	833.83	-	97702293	97703307	0,0,255	1	1014	0
LR699157.1	97880269	97881163	locus40302.1	730.47	-	97880269	97881163	0,0,255	1	894	0
LR699157.1	97970706	97971609	locus40303.1	736.29	-	97970706	97971609	0,0,255	1	903	0
LR699157.1	98087627	98088521	locus40306.1	731.31	+	98087627	98088521	0,0,255	1	894	0
LR699157.1	98251224	98252130	locus40307.1	745.06	+	98251224	98252130	0,0,255	1	906	0
LR699157.1	98300680	98301586	locus40308.1	744.23	+	98300680	98301586	0,0,255	1	906	0
LR699157.1	98424987	98425893	locus40309.1	745.15	+	98424987	98425893	0,0,255	1	906	0
LR699157.1	98544740	98545646	locus40310.1	744.2	+	98544740	98545646	0,0,255	1	906	0
LR699157.1	98605968	98606874	locus40311.1	744.79	+	98605968	98606874	0,0,255	1	906	0
LR699157.1	98689183	98690074	locus40312.1	728.82	-	98689183	98690074	0,0,255	1	891	0
LR699157.1	98944360	98945254	locus40313.1	726.04	-	98944360	98945254	0,0,255	1	894	0
LR699157.1	99010740	99011709	locus40314.1	755.54	-	99010740	99011709	0,0,255	1	969	0

LR699157.1	99080992	99081886	locus40315.1	732.31	-	99080992	99081886	0,0,255	1	894	0
LR699157.1	99271936	99272830	locus40317.1	730.47	-	99271936	99272830	0,0,255	1	894	0
LR699157.1	99354872	99355766	locus40318.1	731.14	-	99354872	99355766	0,0,255	1	894	0
LR699157.1	99494894	99495788	locus40321.1	731.7	-	99494894	99495788	0,0,255	1	894	0
LR699157.1	99570440	99571334	locus40322.1	736.27	-	99570440	99571334	0,0,255	1	894	0
LR699157.1	99679259	99680153	locus40323.1	731.33	-	99679259	99680153	0,0,255	1	894	0
LR699157.1	99799023	99799917	locus40325.1	733.22	-	99799023	99799917	0,0,255	1	894	0
LR699157.1	99963283	99964186	locus40327.1	751.45	+	99963283	99964186	0,0,255	1	903	0
LR699157.1	100011049	100012000	locus40328.1	779.48	+	100011049	100012000	0,0,255	1	951	0
LR699157.1	100063084	100064029	locus40329.1	779.86	+	100063084	100064029	0,0,255	1	945	0
LR699157.1	100161462	100162446	locus40331.1	770.08	-	100161462	100162446	0,0,255	1	984	0
LR699157.1	100234236	100235139	locus40332.1	741.81	-	100234236	100235139	0,0,255	1	903	0
LR699157.1	100446073	100446997	locus40334.1	756.79	+	100446073	100446997	0,0,255	1	924	0
LR699157.1	100530273	100531284	locus40335.1	827.25	+	100530273	100531284	0,0,255	1	1011	0
LR699157.1	100619386	100620310	locus40336.1	758.04	+	100619386	100620310	0,0,255	1	924	0
LR699157.1	100704039	100704963	locus40337.1	758.39	+	100704039	100704963	0,0,255	1	924	0
LR699157.1	100783653	100784577	locus40338.1	753.59	+	100783653	100784577	0,0,255	1	924	0
LR699157.1	100804798	100805692	locus40339.1	731.55	-	100804798	100805692	0,0,255	1	894	0
LR699157.1	100923341	100924262	locus40341.1	751.71	+	100923341	100924262	0,0,255	1	921	0
LR699157.1	100935936	100936830	locus40342.1	733.34	-	100935936	100936830	0,0,255	1	894	0
LR699157.1	101039811	101040732	locus40343.1	753.07	-	101039811	101040732	0,0,255	1	921	0
LR699157.1	101116783	101117704	locus40344.1	752.57	-	101116783	101117704	0,0,255	1	921	0
LR699157.1	101222064	101222985	locus40345.1	752.46	+	101222064	101222985	0,0,255	1	921	0
LR699157.1	101289913	101290807	locus40348.1	724.96	-	101289913	101290807	0,0,255	1	894	0

LR699157.1	101386980	101387883	locus40349.1	741.07	+	101386980	101387883	0,0,255	1	903	0
LR699157.1	101499018	101499912	locus40354.1	736.71	+	101499018	101499912	0,0,255	1	894	0
LR699157.1	101567284	101568202	locus40355.1	760.85	+	101567284	101568202	0,0,255	1	918	0
LR699157.1	101618109	101619030	locus40356.1	757.17	+	101618109	101619030	0,0,255	1	921	0
LR699157.1	101742020	101742932	locus40360.1	750.06	+	101742020	101742932	0,0,255	1	912	0
LR699157.1	101846600	101847500	locus40361.1	737.81	+	101846600	101847500	0,0,255	1	900	0
LR699157.1	101993509	101994409	locus40362.1	738.59	+	101993509	101994409	0,0,255	1	900	0
LR699157.1	102069442	102070336	locus40363.1	728.79	+	102069442	102070336	0,0,255	1	894	0
LR699157.1	102147284	102148208	locus40364.1	758.06	+	102147284	102148208	0,0,255	1	924	0
LR699157.1	102223317	102224238	locus40365.1	759.32	+	102223317	102224238	0,0,255	1	921	0
LR699157.1	102375245	102376280	locus40369.1	845.14	+	102375245	102376280	0,0,255	1	1035	0
LR699157.1	102555817	102556711	locus40370.1	786.69	+	102555817	102556711	0,0,255	1	894	0
LR699157.1	102681207	102682128	locus40371.1	756.9	+	102681207	102682128	0,0,255	1	921	0
LR699157.1	102925797	102926808	locus40376.1	820.93	+	102925797	102926808	0,0,255	1	1011	0
LR699157.1	102934062	102935073	locus40377.1	820.07	+	102934062	102935073	0,0,255	1	1011	0
LR699157.1	103103897	103104908	locus40379.1	818.63	+	103103897	103104908	0,0,255	1	1011	0
LR699157.1	103311773	103312784	locus40382.1	820.75	+	103311773	103312784	0,0,255	1	1011	0
LR699157.1	103483139	103484033	locus40384.1	790.69	+	103483139	103484033	0,0,255	1	894	0
LR699157.1	103578627	103579521	locus40385.1	736.97	+	103578627	103579521	0,0,255	1	894	0
LR699157.1	103609951	103610899	locus40387.1	761.15	-	103609951	103610899	0,0,255	1	948	0
LR699157.1	103665607	103666531	locus40390.1	760.77	-	103665607	103666531	0,0,255	1	924	0
LR699157.1	103809421	103810372	locus40392.1	793.88	+	103809421	103810372	0,0,255	1	951	0
LR699157.1	103836885	103837842	locus40393.1	775.76	-	103836885	103837842	0,0,255	1	957	0
LR699157.1	104036745	104037648	locus40394.1	741.54	-	104036745	104037648	0,0,255	1	903	0

LR699157.1	104092837	104093731	locus40395.1	732.66	+	104092837	104093731	0,0,255	1	894	0
LR699157.1	104129395	104130289	locus40397.1	731.61	-	104129395	104130289	0,0,255	1	894	0
LR699157.1	104202391	104203285	locus40400.1	736.04	-	104202391	104203285	0,0,255	1	894	0
LR699157.1	104252249	104253143	locus40401.1	733.38	-	104252249	104253143	0,0,255	1	894	0
LR699157.1	104351667	104352558	locus40402.1	727.87	-	104351667	104352558	0,0,255	1	891	0
LR699157.1	104411664	104412558	locus40403.1	733.24	-	104411664	104412558	0,0,255	1	894	0
LR699157.1	104478208	104479102	locus40404.1	728.81	-	104478208	104479102	0,0,255	1	894	0
LR699157.1	104549155	104550049	locus40406.1	735.19	-	104549155	104550049	0,0,255	1	894	0
LR699157.1	104604086	104604980	locus40407.1	732.32	-	104604086	104604980	0,0,255	1	894	0
LR699157.1	104681956	104682850	locus40408.1	730.93	-	104681956	104682850	0,0,255	1	894	0
LR699157.1	104732008	104732977	locus40409.1	798.41	+	104732008	104732977	0,0,255	1	969	0
LR699157.1	104793182	104794124	locus40410.1	773.99	+	104793182	104794124	0,0,255	1	942	0
LR699157.1	104815528	104816434	locus40412.1	744.49	+	104815528	104816434	0,0,255	1	906	0
LR699157.1	104822752	104823652	locus40413.1	743.5	-	104822752	104823652	0,0,255	1	900	0
LR699157.1	104854916	104855816	locus40414.1	737.94	-	104854916	104855816	0,0,255	1	900	0
LR699157.1	104884858	104885749	locus40415.1	733.84	-	104884858	104885749	0,0,255	1	891	0
LR699157.1	104900179	104901073	locus40416.1	731.41	-	104900179	104901073	0,0,255	1	894	0
LR699157.1	105023369	105024260	locus40417.1	738.04	+	105023369	105024260	0,0,255	1	891	0
LR699157.1	105139484	105140375	locus40418.1	734.02	-	105139484	105140375	0,0,255	1	891	0
LR699157.1	105229068	105229899	locus40420.1	709.56	-	105229068	105229899	0,0,255	1	831	0
LR699157.1	105285046	105286012	locus40421.1	797.67	-	105285046	105286012	0,0,255	1	966	0
LR699157.1	105298256	105299222	locus40422.1	797.67	-	105298256	105299222	0,0,255	1	966	0
LR699157.1	105400441	105401377	locus40424.1	772.72	+	105400441	105401377	0,0,255	1	936	0
LR699157.1	105474214	105475108	locus40426.1	750.85	+	105474214	105475108	0,0,255	1	894	0

LR699157.1	105486202	105487183	locus40428.1	809.23	-	105486202	105487183	0,0,255	1	981	0
LR699157.1	105564377	105565271	locus40429.1	729.63	+	105564377	105565271	0,0,255	1	894	0
LR699157.1	105675363	105676299	locus40431.1	766.93	+	105675363	105676299	0,0,255	1	936	0
LR699157.1	105744248	105745139	locus40432.1	732.25	+	105744248	105745139	0,0,255	1	891	0
LR699157.1	105756765	105757740	locus40433.1	755.91	-	105756765	105757740	0,0,255	1	975	0
LR699157.1	105836184	105837084	locus40434.1	743.59	-	105836184	105837084	0,0,255	1	900	0
LR699157.1	105880682	105881576	locus40435.1	729.37	-	105880682	105881576	0,0,255	1	894	0
LR699157.1	106038323	106039244	locus40438.1	752.75	+	106038323	106039244	0,0,255	1	921	0
LR699157.1	106120230	106121130	locus40440.1	734.33	+	106120230	106121130	0,0,255	1	900	0
LR699157.1	106204843	106205764	locus40441.1	749.76	+	106204843	106205764	0,0,255	1	921	0
LR699157.1	106271978	106272863	locus40443.1	724.59	+	106271978	106272863	0,0,255	1	885	0
LR699157.1	106353867	106354770	locus40446.1	741.4	+	106353867	106354770	0,0,255	1	903	0
LR699157.1	106370625	106371528	locus40447.1	741.2	-	106370625	106371528	0,0,255	1	903	0
LR699157.1	106469627	106470551	locus40448.1	755.44	+	106469627	106470551	0,0,255	1	924	0
LR699157.1	106499862	106500786	locus40450.1	757.5	-	106499862	106500786	0,0,255	1	924	0
LR699157.1	106648955	106649879	locus40454.1	762.44	-	106648955	106649879	0,0,255	1	924	0
LR699157.1	106701054	106702041	locus40455.1	826.28	-	106701054	106702041	0,0,255	1	987	0
LR699157.1	106796058	106796952	locus40458.1	733.6	-	106796058	106796952	0,0,255	1	894	0
LR699157.1	106837933	106838827	locus40460.1	734.89	-	106837933	106838827	0,0,255	1	894	0
LR699157.1	106887433	106888327	locus40462.1	734.95	-	106887433	106888327	0,0,255	1	894	0
LR699157.1	106935667	106936561	locus40463.1	736.51	-	106935667	106936561	0,0,255	1	894	0
LR699157.1	106974811	106975735	locus40464.1	766.03	-	106974811	106975735	0,0,255	1	924	0
LR699157.1	107027682	107028624	locus40466.1	773.44	-	107027682	107028624	0,0,255	1	942	0
LR699157.1	107131560	107132490	locus40467.1	761.81	+	107131560	107132490	0,0,255	1	930	0

LR699157.1	107172022	107172967	locus40469.1	780.54	-	107172022	107172967	0,0,255	1	945	0
LR699157.1	107313862	107314756	locus40470.1	737.26	+	107313862	107314756	0,0,255	1	894	0
LR699157.1	107429242	107430136	locus40473.1	737.43	+	107429242	107430136	0,0,255	1	894	0
LR699157.1	107505401	107506295	locus40476.1	730.55	+	107505401	107506295	0,0,255	1	894	0
LR699157.1	107564708	107565602	locus40477.1	732.53	+	107564708	107565602	0,0,255	1	894	0
LR699157.1	107632749	107633643	locus40480.1	735.98	+	107632749	107633643	0,0,255	1	894	0
LR699157.1	107670002	107670896	locus40481.1	731.73	+	107670002	107670896	0,0,255	1	894	0
LR699157.1	107727124	107728009	locus40485.1	725.34	+	107727124	107728009	0,0,255	1	885	0
LR699157.1	107804017	107804911	locus40486.1	732.26	+	107804017	107804911	0,0,255	1	894	0
LR699157.1	107821483	107822503	locus40487.1	768.71	-	107821483	107822503	0,0,255	1	1020	0
LR699157.1	107882045	107883038	locus40489.1	826.11	-	107882045	107883038	0,0,255	1	993	0
LR699157.1	107986510	107987413	locus40490.1	739.22	+	107986510	107987413	0,0,255	1	903	0
LR699157.1	108008100	108009003	locus40491.1	739.2	+	108008100	108009003	0,0,255	1	903	0
LR699157.1	108026740	108027664	locus40492.1	769.76	-	108026740	108027664	0,0,255	1	924	0
LR699157.1	108137460	108138348	locus40494.1	725.41	+	108137460	108138348	0,0,255	1	888	0
LR699157.1	108275397	108276285	locus40497.1	723.63	+	108275397	108276285	0,0,255	1	888	0
LR699157.1	108300028	108300916	locus40498.1	729.17	+	108300028	108300916	0,0,255	1	888	0
LR699157.1	108353043	108353931	locus40499.1	727.94	+	108353043	108353931	0,0,255	1	888	0
LR699157.1	108532693	108533581	locus40502.1	727.77	+	108532693	108533581	0,0,255	1	888	0
LR699157.1	108625788	108626676	locus40504.1	724.28	+	108625788	108626676	0,0,255	1	888	0
LR699157.1	108680607	108681501	locus40506.1	734.57	+	108680607	108681501	0,0,255	1	894	0
LR699157.1	108789178	108790135	locus40508.1	787.9	+	108789178	108790135	0,0,255	1	957	0
LR699157.1	108839533	108840427	locus40509.1	726.89	+	108839533	108840427	0,0,255	1	894	0
LR699157.1	108911615	108912518	locus40511.1	738.37	+	108911615	108912518	0,0,255	1	903	0

LR699157.1	108986539	108987505	locus40513.1	801.07	+	108986539	108987505	0,0,255	1	966	0
LR699157.1	109003250	109004162	locus40514.1	753.31	-	109003250	109004162	0,0,255	1	912	0
LR699157.1	109262621	109263551	locus40517.1	767.45	-	109262621	109263551	0,0,255	1	930	0
LR699157.1	109362718	109363603	locus40518.1	735	-	109362718	109363603	0,0,255	1	885	0
LR699157.1	109423540	109424422	locus40519.1	696.2	-	109423540	109424422	0,0,255	1	882	0
LR699157.1	109521576	109522458	locus40520.1	726.59	+	109521576	109522458	0,0,255	1	882	0
LR699157.1	109608190	109609075	locus40522.1	729.89	+	109608190	109609075	0,0,255	1	885	0
LR699157.1	109658305	109659190	locus40523.1	731.83	+	109658305	109659190	0,0,255	1	885	0
LR699157.1	109701965	109702850	locus40524.1	733.11	+	109701965	109702850	0,0,255	1	885	0
LR699157.1	109732771	109733656	locus40525.1	733.82	-	109732771	109733656	0,0,255	1	885	0
LR699157.1	109776449	109777373	locus40526.1	762.86	-	109776449	109777373	0,0,255	1	924	0
LR699157.1	109807220	109808105	locus40527.1	710.92	-	109807220	109808105	0,0,255	1	885	0
LR699157.1	109871761	109872760	locus40528.1	779.04	-	109871761	109872760	0,0,255	1	999	0
LR699157.1	109922567	109923452	locus40530.1	726.37	-	109922567	109923452	0,0,255	1	885	0
LR699157.1	110034098	110035016	locus40531.1	743.94	-	110034098	110035016	0,0,255	1	918	0
LR699157.1	110126450	110127386	locus40533.1	772.72	-	110126450	110127386	0,0,255	1	936	0
LR699157.1	110215511	110216396	locus40534.1	720.18	-	110215511	110216396	0,0,255	1	885	0
LR699157.1	110309727	110310612	locus40537.1	728.66	-	110309727	110310612	0,0,255	1	885	0
LR699157.1	110417353	110418238	locus40538.1	727.42	-	110417353	110418238	0,0,255	1	885	0
LR699157.1	110468381	110469266	locus40539.1	730.56	-	110468381	110469266	0,0,255	1	885	0
LR699157.1	110531067	110532045	locus40540.1	755.65	-	110531067	110532045	0,0,255	1	978	0
LR699157.1	110562731	110563718	locus40541.1	789.37	-	110562731	110563718	0,0,255	1	987	0
LR699157.1	112662266	112663187	locus40565.1	757.04	+	112662266	112663187	0,0,255	1	921	0
LR699157.1	112706537	112707461	locus40566.1	764.67	+	112706537	112707461	0,0,255	1	924	0

LR699157.1	112789869	112790793	locus40568.1	763	+	112789869	112790793	0,0,255	1	924	0
LR699157.1	112866282	112867206	locus40569.1	762.96	+	112866282	112867206	0,0,255	1	924	0
LR699157.1	112939465	112940389	locus40570.1	761.44	+	112939465	112940389	0,0,255	1	924	0
LR699157.1	113004018	113004912	locus40571.1	745.7	+	113004018	113004912	0,0,255	1	894	0
LR699157.1	113055734	113056658	locus40572.1	764.18	+	113055734	113056658	0,0,255	1	924	0
LR699157.1	113132402	113133326	locus40573.1	761.45	+	113132402	113133326	0,0,255	1	924	0
LR699157.1	113195399	113196323	locus40574.1	761.14	+	113195399	113196323	0,0,255	1	924	0
LR699157.1	113280057	113280981	locus40578.1	763.58	-	113280057	113280981	0,0,255	1	924	0
LR699157.1	113328484	113329408	locus40579.1	765.33	-	113328484	113329408	0,0,255	1	924	0
LR699157.1	113385393	113386317	locus40580.1	762.44	-	113385393	113386317	0,0,255	1	924	0
LR699157.1	113451104	113452028	locus40581.1	762.59	-	113451104	113452028	0,0,255	1	924	0
LR699157.1	113526897	113527821	locus40582.1	760.9	-	113526897	113527821	0,0,255	1	924	0
LR699157.1	113612402	113613326	locus40584.1	765.6	-	113612402	113613326	0,0,255	1	924	0
LR699157.1	113658588	113659512	locus40585.1	763.7	-	113658588	113659512	0,0,255	1	924	0
LR699157.1	113720965	113721859	locus40586.1	738.53	-	113720965	113721859	0,0,255	1	894	0
LR699161.1	15068330	15069230	locus44250.1	728.91	+	15068330	15069230	0,0,255	1	900	0
LR699161.1	15109005	15109938	locus44251.1	756.91	+	15109005	15109938	0,0,255	1	933	0
LR699161.1	15137860	15138913	locus44252.1	848.06	-	15137860	15138913	0,0,255	1	1053	0
LR699161.1	15159019	15159952	locus44253.1	757.76	-	15159019	15159952	0,0,255	1	933	0
LR699161.1	15580906	15581815	locus44258.1	737.06	+	15580906	15581815	0,0,255	1	909	0
LR699161.1	15686262	15687195	locus44261.1	759.51	+	15686262	15687195	0,0,255	1	933	0
LR699161.1	16326175	16326979	locus44273.1	690.01	+	16326175	16326979	0,0,255	1	804	0
LR699161.1	17752896	17753814	locus44290.1	741.03	-	17752896	17753814	0,0,255	1	918	0
LR699161.1	18086294	18087188	locus44297.1	723.51	+	18086294	18087188	0,0,255	1	894	0

LR699161.1	18177544	18178450	locus44300.1	730.75	+	18177544	18178450	0,0,255	1	906	0
LR699161.1	23691778	23692696	locus44364.1	753	-	23691778	23692696	0,0,255	1	918	0
LR699161.1	23722581	23723517	locus44365.1	785.11	-	23722581	23723517	0,0,255	1	936	0
LR699161.1	28638713	28639643	locus44437.1	752.52	+	28638713	28639643	0,0,255	1	930	0
LR699161.1	28670387	28671317	locus44439.1	751.92	+	28670387	28671317	0,0,255	1	930	0
LR699161.1	28690143	28691070	locus44440.1	749.12	+	28690143	28691070	0,0,255	1	927	0
LR699161.1	28718812	28719694	locus44442.1	733.44	+	28718812	28719694	0,0,255	1	882	0
LR699161.1	28742883	28743810	locus44444.1	751.51	+	28742883	28743810	0,0,255	1	927	0
LR699161.1	28961223	28962156	locus44450.1	757.22	+	28961223	28962156	0,0,255	1	933	0
LR699161.1	28973488	28974391	locus44451.1	732.7	-	28973488	28974391	0,0,255	1	903	0
LR699161.1	28991930	28992833	locus44452.1	733.92	-	28991930	28992833	0,0,255	1	903	0
LR699161.1	32961355	32962441	locus44514.1	914.35	+	32961355	32962441	0,0,255	1	1086	0

Anole_lizard

2	193798122	193799079	locus37.1	780.54	+	193798122	193799079	0,0,255	1	957	0
3	15721862	15722747	locus40.1	705.1	-	15721862	15722747	0,0,255	1	885	0
GL343220.1	94617	95520	locus96.1	733.75	-	94617	95520	0,0,255	1	903	0
GL343220.1	113516	114419	locus97.1	731.68	-	113516	114419	0,0,255	1	903	0
GL343220.1	128250	129024	locus98.1	710.76	-	128250	129024	0,0,255	1	774	0
GL343220.1	149925	150825	locus99.1	732.8	-	149925	150825	0,0,255	1	900	0
GL343220.1	159930	160821	locus100.1	732.7	-	159930	160821	0,0,255	1	891	0
GL343220.1	174203	175094	locus101.1	732.74	-	174203	175094	0,0,255	1	891	0
GL343220.1	187800	188691	locus102.1	723.25	-	187800	188691	0,0,255	1	891	0
GL343220.1	200234	201125	locus103.1	727.93	-	200234	201125	0,0,255	1	891	0
GL343220.1	212181	213111	locus104.1	765.26	-	212181	213111	0,0,255	1	930	0
GL343220.1	226433	227324	locus105.1	727.08	-	226433	227324	0,0,255	1	891	0
GL343220.1	241426	242326	locus106.1	725.66	-	241426	242326	0,0,255	1	900	0
GL343220.1	254373	255273	locus107.1	731.84	-	254373	255273	0,0,255	1	900	0
GL343220.1	284822	285722	locus108.1	734.78	+	284822	285722	0,0,255	1	900	0
GL343220.1	299440	300334	locus109.1	723.63	+	299440	300334	0,0,255	1	894	0
GL343220.1	327484	328570	locus111.1	905.18	+	327484	328570	0,0,255	1	1086	0
GL343220.1	343063	344074	locus112.1	820.88	+	343063	344074	0,0,255	1	1011	0
GL343220.1	355553	356444	locus113.1	724.51	+	355553	356444	0,0,255	1	891	0
GL343220.1	365185	366106	locus114.1	767.85	+	365185	366106	0,0,255	1	921	0
GL343220.1	392927	393749	locus115.1	646.51	+	392927	393749	0,0,255	1	822	0
GL343220.1	397437	398328	locus117.1	716.61	-	397437	398328	0,0,255	1	891	0
GL343220.1	411614	412556	locus119.1	762.71	-	411614	412556	0,0,255	1	942	0

GL343220.1	422000	422936	locus120.1	772.93	-	422000	422936	0,0,255	1	936	0
GL343220.1	433763	434663	locus121.1	728.18	-	433763	434663	0,0,255	1	900	0
GL343220.1	449524	450424	locus122.1	727.67	-	449524	450424	0,0,255	1	900	0
GL343220.1	464902	465865	locus123.1	761.78	-	464902	465865	0,0,255	1	963	0
GL343220.1	476931	477852	locus124.1	737.99	-	476931	477852	0,0,255	1	921	0
GL343220.1	487819	488782	locus125.1	773.63	-	487819	488782	0,0,255	1	963	0
GL343220.1	508308	509229	locus127.1	747.49	+	508308	509229	0,0,255	1	921	0
GL343220.1	1575221	1576208	locus129.1	816.65	-	1575221	1576208	0,0,255	1	987	0
GL343220.1	1587051	1587942	locus130.1	732.62	-	1587051	1587942	0,0,255	1	891	0
GL343253.1	601063	601996	locus135.1	758.35	+	601063	601996	0,0,255	1	933	0
GL343253.1	618252	619146	locus137.1	730.13	+	618252	619146	0,0,255	1	894	0
GL343253.1	780204	781149	locus139.1	778.79	+	780204	781149	0,0,255	1	945	0
GL343253.1	934580	935480	locus140.1	735.63	+	934580	935480	0,0,255	1	900	0
GL343253.1	1098933	1099848	locus141.1	756.56	+	1098933	1099848	0,0,255	1	915	0
GL343253.1	1137573	1138464	locus142.1	725.15	+	1137573	1138464	0,0,255	1	891	0
GL343253.1	1475928	1476840	locus143.1	748.49	+	1475928	1476840	0,0,255	1	912	0
GL343253.1	1494215	1495115	locus144.1	740.4	+	1494215	1495115	0,0,255	1	900	0
GL343253.1	1899873	1900782	locus145.1	744.49	-	1899873	1900782	0,0,255	1	909	0
GL343253.1	1917521	1918412	locus146.1	730.88	-	1917521	1918412	0,0,255	1	891	0
GL343253.1	1931433	1932225	locus147.1	645.86	-	1931433	1932225	0,0,255	1	792	0
GL343253.1	1959004	1960042	locus148.1	846.78	+	1959004	1960042	0,0,255	1	1038	0
GL343253.1	1963640	1964537	locus149.1	740.71	-	1963640	1964537	0,0,255	1	897	0
GL343253.1	1975044	1975962	locus150.1	750.76	-	1975044	1975962	0,0,255	1	918	0
GL343253.1	1989115	1990021	locus152.1	747.43	-	1989115	1990021	0,0,255	1	906	0

GL343287.1	1752846	1753809	locus155.1	773.89	+	1752846	1753809	0,0,255	1	963	0
GL343438.1	218253	219153	locus175.1	728.6	+	218253	219153	0,0,255	1	900	0
GL343491.1	140613	141510	locus177.1	737.45	+	140613	141510	0,0,255	1	897	0
GL343491.1	153746	154643	locus178.1	738.26	+	153746	154643	0,0,255	1	897	0
GL343491.1	165893	166793	locus179.1	740.81	+	165893	166793	0,0,255	1	900	0
GL343491.1	172830	173730	locus180.1	722.27	-	172830	173730	0,0,255	1	900	0
GL343491.1	214856	215756	locus181.1	728.35	-	214856	215756	0,0,255	1	900	0
GL343491.1	226456	227356	locus182.1	728.9	-	226456	227356	0,0,255	1	900	0
GL343514.1	253785	254691	locus186.1	747.63	+	253785	254691	0,0,255	1	906	0
GL343514.1	268411	269341	locus187.1	759.26	+	268411	269341	0,0,255	1	930	0
GL343514.1	281254	282160	locus188.1	740.5	+	281254	282160	0,0,255	1	906	0
GL343514.1	304686	305592	locus190.1	740.14	+	304686	305592	0,0,255	1	906	0
GL343520.1	399815	400715	locus183.1	718.66	-	399815	400715	0,0,255	1	900	0
GL343520.1	434981	435863	locus184.1	707.47	-	434981	435863	0,0,255	1	882	0
GL344165.1	51383	52262	locus203.1	716.55	-	51383	52262	0,0,255	1	879	0
AAWZ020352	30170	31106	locus204.1	761.36	-	30170	31106	0,0,255	1	936	0
AAWZ020387	59	779	locus211.1	580.84	+	59	779	0,0,255	1	720	0

Mouse

CM000995.2	182000184	182001087	locus17.1	743.69	+	182000184	182001087	0,0,255	1	903	0
CM000996.2	64104486	64105458	locus22.1	785.51	+	64104486	64105458	0,0,255	1	972	0
CM000996.2	64116431	64117403	locus23.1	785.52	-	64116431	64117403	0,0,255	1	972	0
CM000996.2	64258960	64260181	locus26.1	1001.5	-	64258960	64260181	0,0,255	1	1221	0
CM000996.2	64339564	64340671	locus28.1	901.25	-	64339564	64340671	0,0,255	1	1107	0
CM000996.2	64490820	64491849	locus33.1	831.03	-	64490820	64491849	0,0,255	1	1029	0
CM000996.2	64537560	64538532	locus35.1	791.09	-	64537560	64538532	0,0,255	1	972	0
CM000996.2	64690659	64691631	locus41.1	791.08	-	64690659	64691631	0,0,255	1	972	0
CM000997.2	156338145	156339060	locus63.1	745.28	+	156338145	156339060	0,0,255	1	915	0
CM000998.2	108797192	108798125	locus74.1	765.89	-	108797192	108798125	0,0,255	1	933	0
CM000998.2	108842946	108843849	locus75.1	733.96	-	108842946	108843849	0,0,255	1	903	0
CM000998.2	108995538	108996441	locus77.1	739.25	-	108995538	108996441	0,0,255	1	903	0
CM000998.2	109046872	109047775	locus78.1	742.9	-	109046872	109047775	0,0,255	1	903	0
CM000998.2	109085848	109086706	locus79.1	700.55	-	109085848	109086706	0,0,255	1	858	0
CM000998.2	109156067	109156922	locus80.1	694.16	-	109156067	109156922	0,0,255	1	855	0
CM000998.2	109215501	109216404	locus82.1	737.9	-	109215501	109216404	0,0,255	1	903	0
CM000998.2	109286268	109287171	locus83.1	735.87	-	109286268	109287171	0,0,255	1	903	0
CM000998.2	109363578	109364481	locus85.1	738.41	+	109363578	109364481	0,0,255	1	903	0
CM000998.2	109452484	109453387	locus86.1	739.61	+	109452484	109453387	0,0,255	1	903	0
CM000998.2	109566217	109567102	locus87.1	732.64	-	109566217	109567102	0,0,255	1	885	0
CM000998.2	138532199	138533006	locus90.1	667.82	+	138532199	138533006	0,0,255	1	807	0
CM000998.2	151513132	151514023	locus92.1	724.66	-	151513132	151514023	0,0,255	1	891	0
CM000998.2	151561660	151562464	locus93.1	646.37	-	151561660	151562464	0,0,255	1	804	0

CM000999.2	123335559	123336537	locus105.1	802.7	+	123335559	123336537	0,0,255	1	978	0
CM000999.2	123385261	123386152	locus106.1	725.55	-	123385261	123386152	0,0,255	1	891	0
CM000999.2	123492507	123493398	locus108.1	727.02	-	123492507	123493398	0,0,255	1	891	0
CM000999.2	123609757	123610648	locus110.1	727.02	-	123609757	123610648	0,0,255	1	891	0
CM000999.2	123741348	123742239	locus111.1	733.71	+	123741348	123742239	0,0,255	1	891	0
CM000999.2	123815389	123816280	locus112.1	728	+	123815389	123816280	0,0,255	1	891	0
CM000999.2	123822813	123823704	locus113.1	726.23	-	123822813	123823704	0,0,255	1	891	0
CM000999.2	124061144	124062035	locus114.1	725.35	+	124061144	124062035	0,0,255	1	891	0
CM000999.2	124081695	124082586	locus115.1	724.68	-	124081695	124082586	0,0,255	1	891	0
CM000999.2	124138730	124139621	locus116.1	721.54	-	124138730	124139621	0,0,255	1	891	0
CM000999.2	124191595	124192498	locus117.1	734.08	-	124191595	124192498	0,0,255	1	903	0
CM001000.2	5480640	5481543	locus120.1	748.14	-	5480640	5481543	0,0,255	1	903	0
CM001000.2	7231326	7232229	locus122.1	745.26	-	7231326	7232229	0,0,255	1	903	0
CM001000.2	7312273	7313176	locus124.1	747.85	-	7312273	7313176	0,0,255	1	903	0
CM001000.2	7383984	7384914	locus126.1	766.13	-	7383984	7384914	0,0,255	1	930	0
CM001000.2	7417669	7418572	locus128.1	748.98	-	7417669	7418572	0,0,255	1	903	0
CM001000.2	7463968	7464871	locus130.1	746.14	-	7463968	7464871	0,0,255	1	903	0
CM001000.2	7550966	7551896	locus133.1	765.78	-	7550966	7551896	0,0,255	1	930	0
CM001000.2	7642128	7643058	locus134.1	760.59	-	7642128	7643058	0,0,255	1	930	0
CM001000.2	7671828	7672731	locus136.1	745.58	-	7671828	7672731	0,0,255	1	903	0
CM001000.2	7786150	7787080	locus140.1	764.66	-	7786150	7787080	0,0,255	1	930	0
CM001000.2	7876652	7877555	locus143.1	747.96	-	7876652	7877555	0,0,255	1	903	0
CM001000.2	7948287	7949217	locus145.1	767.19	-	7948287	7949217	0,0,255	1	930	0
CM001000.2	8102367	8103297	locus150.1	763.48	-	8102367	8103297	0,0,255	1	930	0

CM001000.2	8137904	8138807	locus151.1	748.69	-	8137904	8138807	0,0,255	1	903	0
CM001000.2	8184212	8185115	locus153.1	745.53	-	8184212	8185115	0,0,255	1	903	0
CM001000.2	8244624	8245554	locus155.1	765.17	-	8244624	8245554	0,0,255	1	930	0
CM001000.2	8304225	8305155	locus157.1	764.59	-	8304225	8305155	0,0,255	1	930	0
CM001000.2	8367459	8368389	locus159.1	766.43	-	8367459	8368389	0,0,255	1	930	0
CM001000.2	8399201	8400104	locus161.1	748.72	-	8399201	8400104	0,0,255	1	903	0
CM001000.2	8471468	8472371	locus163.1	746.79	-	8471468	8472371	0,0,255	1	903	0
CM001000.2	8667317	8668220	locus170.1	746.95	-	8667317	8668220	0,0,255	1	903	0
CM001000.2	8738845	8739775	locus172.1	766.83	-	8738845	8739775	0,0,255	1	930	0
CM001000.2	8801137	8802040	locus174.1	749.47	-	8801137	8802040	0,0,255	1	903	0
CM001000.2	8872179	8873109	locus176.1	763.4	-	8872179	8873109	0,0,255	1	930	0
CM001000.2	8907733	8908636	locus177.1	748.76	-	8907733	8908636	0,0,255	1	903	0
CM001000.2	9014749	9015679	locus181.1	763.75	-	9014749	9015679	0,0,255	1	930	0
CM001000.2	9074795	9075725	locus183.1	765.18	-	9074795	9075725	0,0,255	1	930	0
CM001000.2	9176901	9177831	locus185.1	765.75	-	9176901	9177831	0,0,255	1	930	0
CM001000.2	9206102	9207005	locus187.1	747.24	-	9206102	9207005	0,0,255	1	903	0
CM001000.2	9326023	9326953	locus191.1	764.63	-	9326023	9326953	0,0,255	1	930	0
CM001000.2	9373547	9374450	locus192.1	747.54	-	9373547	9374450	0,0,255	1	903	0
CM001000.2	9547710	9548613	locus198.1	749.47	-	9547710	9548613	0,0,255	1	903	0
CM001000.2	9642620	9643523	locus200.1	747.54	-	9642620	9643523	0,0,255	1	903	0
CM001000.2	9816781	9817684	locus206.1	749.47	-	9816781	9817684	0,0,255	1	903	0
CM001000.2	9886277	9887207	locus208.1	764.31	-	9886277	9887207	0,0,255	1	930	0
CM001000.2	9927623	9928526	locus210.1	749.43	-	9927623	9928526	0,0,255	1	903	0
CM001000.2	9976244	9977147	locus212.1	746.8	-	9976244	9977147	0,0,255	1	903	0

CM001000.2	10037234	10038164	locus214.1	764.73	-	10037234	10038164	0,0,255	1	930	0
CM001000.2	10087197	10088100	locus216.1	748.3	-	10087197	10088100	0,0,255	1	903	0
CM001000.2	10158651	10159554	locus218.1	753.88	-	10158651	10159554	0,0,255	1	903	0
CM001000.2	12581469	12582444	locus220.1	788.01	-	12581469	12582444	0,0,255	1	975	0
CM001000.2	12615232	12616207	locus221.1	791.23	-	12615232	12616207	0,0,255	1	975	0
CM001000.2	12651705	12652608	locus222.1	733.6	-	12651705	12652608	0,0,255	1	903	0
CM001000.2	12693997	12694900	locus223.1	732.95	-	12693997	12694900	0,0,255	1	903	0
CM001000.2	41399731	41400661	locus224.1	759.8	-	41399731	41400661	0,0,255	1	930	0
CM001000.2	41719004	41719919	locus227.1	742.04	+	41719004	41719919	0,0,255	1	915	0
CM001000.2	41744712	41745639	locus229.1	757.77	-	41744712	41745639	0,0,255	1	927	0
CM001000.2	41836880	41837807	locus231.1	757.92	-	41836880	41837807	0,0,255	1	927	0
CM001000.2	41937526	41938462	locus232.1	765.04	-	41937526	41938462	0,0,255	1	936	0
CM001000.2	42011791	42012727	locus234.1	758.49	-	42011791	42012727	0,0,255	1	936	0
CM001000.2	42194876	42195776	locus237.1	732.81	+	42194876	42195776	0,0,255	1	900	0
CM001000.2	42299924	42300755	locus239.1	695.34	+	42299924	42300755	0,0,255	1	831	0
CM001000.2	42429782	42430718	locus243.1	762.82	-	42429782	42430718	0,0,255	1	936	0
CM001000.2	42764437	42765355	locus244.1	747.33	-	42764437	42765355	0,0,255	1	918	0
CM001000.2	42903250	42904168	locus246.1	747.55	-	42903250	42904168	0,0,255	1	918	0
CM001000.2	43047188	43048106	locus248.1	746.62	+	43047188	43048106	0,0,255	1	918	0
CM001000.2	84940168	84941215	locus254.1	852.34	-	84940168	84941215	0,0,255	1	1047	0
CM001000.2	84994644	84995562	locus255.1	750.26	-	84994644	84995562	0,0,255	1	918	0
CM001000.2	85136239	85137202	locus261.1	787.5	-	85136239	85137202	0,0,255	1	963	0
CM001000.2	85221517	85222480	locus264.1	791.86	-	85221517	85222480	0,0,255	1	963	0
CM001000.2	85406375	85407290	locus268.1	751.81	-	85406375	85407290	0,0,255	1	915	0

CM001000.2	85558702	85559620	locus271.1	748.5	-	85558702	85559620	0,0,255	1	918	0
CM001000.2	85623629	85624547	locus276.1	760.34	+	85623629	85624547	0,0,255	1	918	0
CM001000.2	85737783	85738746	locus280.1	793.66	-	85737783	85738746	0,0,255	1	963	0
CM001000.2	85857546	85858461	locus286.1	751.9	-	85857546	85858461	0,0,255	1	915	0
CM001000.2	85951866	85952781	locus289.1	753.33	-	85951866	85952781	0,0,255	1	915	0
CM001000.2	86148041	86148965	locus298.1	760.91	-	86148041	86148965	0,0,255	1	924	0
CM001000.2	86225205	86226111	locus302.1	752.48	-	86225205	86226111	0,0,255	1	906	0
CM001000.2	86811114	86812032	locus305.1	757.2	+	86811114	86812032	0,0,255	1	918	0
CM001000.2	86884870	86885788	locus307.1	755.19	+	86884870	86885788	0,0,255	1	918	0
CM001000.2	86954253	86955177	locus309.1	761.59	+	86954253	86955177	0,0,255	1	924	0
CM001000.2	87037050	87037968	locus311.1	760.7	+	87037050	87037968	0,0,255	1	918	0
CM001001.2	3139124	3140009	locus317.1	721.15	+	3139124	3140009	0,0,255	1	885	0
CM001002.2	124428075	124429008	locus338.1	763.2	-	124428075	124429008	0,0,255	1	933	0
CM001003.2	79193979	79194933	locus345.1	788.47	+	79193979	79194933	0,0,255	1	954	0
CM001003.2	79292956	79293874	locus346.1	762.26	+	79292956	79293874	0,0,255	1	918	0
CM001003.2	79395839	79396766	locus347.1	763.21	+	79395839	79396766	0,0,255	1	927	0
CM001003.2	79491347	79492154	locus348.1	656.54	+	79491347	79492154	0,0,255	1	807	0
CM001003.2	130385799	130386705	locus351.1	743.8	-	130385799	130386705	0,0,255	1	906	0
CM001003.2	130418260	130419166	locus352.1	744.36	-	130418260	130419166	0,0,255	1	906	0
CM001003.2	130446198	130447098	locus353.1	736.22	-	130446198	130447098	0,0,255	1	900	0
CM001003.2	130471820	130472720	locus354.1	733.27	-	130471820	130472720	0,0,255	1	900	0
CM001007.2	51417979	51418882	locus389.1	735.54	+	51417979	51418882	0,0,255	1	903	0
CM001007.2	51460056	51460854	locus391.1	695.29	+	51460056	51460854	0,0,255	1	798	0
CM001010.2	17733237	17734167	locus403.1	758.77	+	17733237	17734167	0,0,255	1	930	0

CM001010.2	18184353	18185178	locus409.1	699.47	+	18184353	18185178	0,0,255	1	825	0
CM001010.2	18243569	18244394	locus410.1	769.85	-	18243569	18244394	0,0,255	1	825	0
CM001010.2	18325619	18326441	locus413.1	767.65	+	18325619	18326441	0,0,255	1	822	0
CM001010.2	18451538	18452324	locus415.1	733.92	+	18451538	18452324	0,0,255	1	786	0
CM001010.2	18597338	18598157	locus419.1	760.9	+	18597338	18598157	0,0,255	1	819	0
CM001010.2	18706327	18707149	locus421.1	767.12	+	18706327	18707149	0,0,255	1	822	0
CM001010.2	18947249	18948071	locus426.1	720.52	+	18947249	18948071	0,0,255	1	822	0
CM001010.2	19080489	19081311	locus430.1	722.02	+	19080489	19081311	0,0,255	1	822	0
CM001010.2	19175935	19176751	locus432.1	692.39	+	19175935	19176751	0,0,255	1	816	0
CM001010.2	19303725	19304643	locus434.1	752.41	+	19303725	19304643	0,0,255	1	918	0
CM001010.2	19393768	19394590	locus436.1	762.84	+	19393768	19394590	0,0,255	1	822	0
CM001010.2	19531211	19532060	locus440.1	681.07	+	19531211	19532060	0,0,255	1	849	0
CM001010.2	19811540	19812536	locus448.1	827.96	+	19811540	19812536	0,0,255	1	996	0
CM001010.2	19986867	19987782	locus452.1	749.56	-	19986867	19987782	0,0,255	1	915	0
CM001010.2	20029424	20030246	locus453.1	699.09	-	20029424	20030246	0,0,255	1	822	0
CM001010.2	20208229	20209144	locus456.1	757.96	-	20208229	20209144	0,0,255	1	915	0
CM001010.2	20267546	20268545	locus458.1	838.63	-	20267546	20268545	0,0,255	1	999	0
CM001010.2	20374938	20375772	locus462.1	677.49	+	20374938	20375772	0,0,255	1	834	0
CM001010.2	20540516	20541434	locus465.1	754.13	-	20540516	20541434	0,0,255	1	918	0
CM001010.2	20573828	20574746	locus467.1	758.67	-	20573828	20574746	0,0,255	1	918	0
CM001010.2	22547940	22548753	locus469.1	659.26	-	22547940	22548753	0,0,255	1	813	0
CM001010.2	22618320	22619133	locus472.1	658.26	+	22618320	22619133	0,0,255	1	813	0
CM001010.2	22958001	22958814	locus474.1	657.06	+	22958001	22958814	0,0,255	1	813	0
CM001010.2	23076612	23077425	locus476.1	656.3	+	23076612	23077425	0,0,255	1	813	0

CM001010.2	23149863	23150676	locus477.1	652.21	+	23149863	23150676	0,0,255	1	813	0
CM001010.2	23290933	23291842	locus481.1	733.58	-	23290933	23291842	0,0,255	1	909	0
CM001010.2	23359219	23360128	locus484.1	731.67	+	23359219	23360128	0,0,255	1	909	0
CM001010.2	23400955	23401864	locus486.1	736.73	+	23400955	23401864	0,0,255	1	909	0
CM001010.2	23441010	23441823	locus487.1	673.2	-	23441010	23441823	0,0,255	1	813	0
CM001010.2	23459674	23460487	locus489.1	699.6	-	23459674	23460487	0,0,255	1	813	0
CM001010.2	55592340	55593123	locus493.1	736.42	-	55592340	55593123	0,0,255	1	783	0
CM001010.2	57508782	57509715	locus494.1	771.92	-	57508782	57509715	0,0,255	1	933	0
CM001013.2	124127777	124128680	locus508.1	734.21	-	124127777	124128680	0,0,255	1	903	0
KB469738.3	197845	198778	locus755.1	763.2	-	197845	198778	0,0,255	1	933	0
KB469742.1	904911	905817	locus758.1	83.15	-	904911	905817	0,0,255	1	906	0

Table S2. PCR primers used to amplify four V2R genes of *Polypterus senegalus*

Gene	Forward	Reverse
V2R2	5'-CCAACGTCTTCTGATTGTG-3'	5'-GACTCCGTGATGTTACTGCT-3'
ancV2R	5'-GCAAGACGAGATCAATGTG-3'	5'-TTGTGCTGATGTATCCTGG-3'
f-V2R65700	5'-TGTATGACACTGCTGAAGTGC-3'	5'-CTTTCCGTGCATACCGATA-3'
t-V2R56889	5'-GCAGGTACTGAGATTGTGTAGG-3'	5'-CAATGCTGTCACTGACAGAC-3'

Supplementary File 1. The aligned V2R sequences of all 1897 intact V2Rs identified from various vertebrates.

>Japanese_eel.locus298.1
CVQCHSDYWSSKKRDECILKETEYLSYQEIMGILLASISLLGTCTTSIVVTIFFIYRHTPIVKANNSELSFLLLFSLKLCFLCSLTFIGRPSEWSCMLRHTAFGITFVLCISCVLGKTIVVLMAFRATLPGSNVMKWFGPLQQRMSVLA
FTLIQVLICVLWLTISPPFPNKNMKHYKEKIILECDVGSAVGFWAVLGYIGLLSILCFILAFLARKLPDNFNEAKLITFSMLIFCAVWITFIPAYVSSPGKFTVAVEIFAILASSFGMLCCIFIPKCYIILLKPDKNNKKNLMGKGPL
>Japanese_eel.locus155.1

MGILLASISLLGTCTTSIVATIFFRYRHTPIVKANNSELSFLLLFSLKLCFLCSLTFIGRPSEWSCMLRHTAFGITFVLCISCVLGKTIVVLMAFRATLPGSNVMKWFGPLQQRLSVLAFTLIQVLICVLWLTISPPFPNKNMKHYKEK
IILECDVGSALGFWSVLGYIGLLSILCFVLAFLARKLPDNFNEAKFITFSMLIFCAVWITFIPAYVSSPGKFTVAVEIFAILASSFGMLFCIFIPKCYIILLKPDKNNKKNLMGNRPP
>Japanese_eel.locus153.1
CVQCHSDYWSSKKRDECLSKETEYLSYHEIMGILLASISLLGTCTTSIVVIIFFRYRHTPIVKANNSELSFLLLFSLKLCFLCSLTFIGRPSEWSCMLRHTAFGITFVLCISCVLGKTIVVLMAFRATLPGSNVMKWFGPLQQRLSVLA
FTLIQVLICVLWLTISPPFPNKNMKHYKEKIILECDVGSAVGFWAVLGYIGLLSILCFVLAFLARKLPDNFNEAKYITFSMLIFCAVWITFIPAYVSSPGKFTVAVEIFAILASSFGMLFCIFIPKCYIILLKPEKNNKKNLMGKMLS
>Japanese_eel.locus145.1

ILGYLPAVFFILGVCFTTTVAIIIFWYRHNAITRANNSELSFLLLFSLKLCFLCSLTFIGRPSEWSCMLRHTAFGITFVLCISCVLGKTIVVLMAFRATLPGSNVMKWFGPLQQRLSVLAFTLIQVLICVLWLTISPPFPTKNMKHFKE
KIILECDLGSAVGFWAVLGYIGLLSILCFVLVFLVRKLP--FGESTFLLLCMLICHAVWIALIQMYVRTPTKYTLIIKIVTTSTTSNH-------------------------------
>Japanese_eel.locus151.1
CVRCPFEYWSNKRGDACVLKEIEFLSFGEVMGILLVFFSLTGVLFTAMTALIFYSNRKTPIVRANNSELSFLLLFSLKLCFLCSLTFIGRPSEWSCMLRHTAFGITFVLCISCVLGKTIVVLMAFRATLPGSNVMKWFGPLQQRLSVLA
FTLIQVLICVLWLTISPPFPNKNTKHYKEKIILECDVGSAVGFWAVLGYIGLLSLLCFVLAFLARKLPDNFNEAKFITFSMLIFCAVWITFIPAYVSSPGKFTVAVEIFAILASSFGLLFCIFIPKCYIILLKPEKNTKKHVMGKTSN
>Western_clawed_frog.locus42.1
------MEWPNRKKTKCIPKTVDYLSYH-
VLSAFFSATSALFLLASLLILGVFITYRDSPIVRANNRSLSFLLLVSIKLSFLSVFLFLGRPVDITCMLRIITFGITFSIAVSSLLAKTIMVCVAFKATKPGSSWRKWLGVKLSNSVVLFCSSIQIIICMTWLAISPPFQELDIHTSPG
TIIIQCNEGSAIGFYSVIGYMGLLAAVSFVLAFLARSLPDSFNEAKYITFSMLLFCSVWITMIPAYLSTKGKNTVCVEIFAILTSSAGLLACIFLPKCYIIQFRSEINTKSQLLGNRQN
>Western_clawed_frog.locus41.1
------MEWPNRKKTKCIPKTVDFLSYH-
VLSAFFSATSALFFLASLLILGVFITYRDSPIVRANNRSLSFLLLVSIKLSFLSVFLFLGCPVDITCMLRIITFGITFSIAVSSLLAKTIMVCVAFKATKPGSSWRKWLGVKLSNSVVLFCSSIQIIICMTWLAISPPFQELDIHTSPG
TIIIQCNEGSAIGFYSVIGYMGLLAAVSFVLAFLARSLPDSFNEAKYITFSMLLFCSVWITMIPAYLSTKGKNTVCVEIFAILTSSAGLLACIFLPKCYIIQFRSEMNTKSQLLGNRQN
>Western_clawed_frog.locus44.1
------MEWPNRKKTQCIPKSVDYLSYH-
ILSAFFSATSALFFLASLLILGVFITYRDSPIVRANNRSLSFLLLVSIKLSFLSVFLFLGRPVDITCMLRNVTFGITFSIAVSSLLAKTIMVCVAFKATKPGSSWRKWLGVKLSNSVVLFCSSIQIIICMTWLAISPPFQELDIHTSPG
TIIIQCNEGSAIGFYSVIGYMGLLAAVSFVLAFLARSLPDSFNEAKYITFSMLLFCSVWITMIPAYLSTKGKNTVCVEIFAILTSSAGLLASIFLPKCYIIQFRSEINTKSQLLGNRQN
>Western_clawed_frog.locus52.1
------MEWPNRKKTKCIPKTVDYLSYH-
ILSAFFSATSALFFLASLLILGVFITYRDTPIVRANNRSLSFLLLVSIKLSFLSVFLFLGRPVDITCMLRIITFGITFSIAVSSLLAKTIMVCVAFKATKPGSSWRKWLGVKLSNSVVLFCSSIQIIICMTWLAISPPFQELDIHTSPG
TIIIQCNEGSAIGFYSVIGYMGLLAAVSFVLAFLARSLPDSFNEAKYITFSMLLFCSVWITMIPAYLSTKGKNTVCVEIFAILTSSAGLLACIFLPKCYIIQFRSEINTKSQLLGNRQN
>Western_clawed_frog.locus47.1
------MEWPNRKKNKCIPKTVDFLSYH-
ILSAFFSAASALFFLASLLILGVFITYRDTPIVRANNRSLSFLLLVSIKLSFLSVFLFLGRPVDITCMLRIITFGITFSIAVSSLLAKTIMVCVAFKATKPGSSWRKWLGVKLSNSVVLFCSSIQIIICMTWLAISPPFQELDIHTSPG
TIIIQCNEGSAIGFYSVIGYMGLLAAVSFVLAFLARSLPDSFNEAKYITFSMLLFCSVWITMIPAYLSTKGKNTVCVEIFAILTSSAGLLACIFLPKCYIIQFRSEINTKSQLLGNRQN
>Western_clawed_frog.locus39.1
------MEWPNRKKTKCIPKTVDFLSYH-
ILSAFFSATSVLFFLASLLILGVFIAHQDTPIVRANNRSLSFLLLVSIKLSFLSVFLFLGRPVDITCMMRIITFGITFSIAVSSLLAKTIMVCVAFKATKPGSSWRKWLGVKLSNSVVLFCSSIQIIICMTWLAISPPFQELDIHTSPG
TIIIQCNEGSAIGFYSVIGYMGLLAAVSFVLAFLARSLPDSFNEAKYITFSMLLFCSVWITMIPAYLSTKGKNTVCVEIFAILNSSAGLLACIFLPKCYIIQFRAEINTKSLLLGNRQN
>Western_clawed_frog.locus43.1
------MEWPNRKKTKCIPKTVDFLSYH-
ILSAFFSATSALFFLASLLILGVFIAHRETPIVRANNRSLSFLLLVSIKLSFLSVFLFLGRPVDITCMLRIITFGITFSIAVSSLLAKTIMVCVAFKATKPGSSWRKWLGVKLSNSVVLFCSSIQIIICMTWLAISPPFQELDIHTSPG
TIIIQCNEGSAIGFYSVIGYMGLLAAVSFVLAFLARSLPDSFNEAKYITFSMLLFCSVWITMIPAYLSTKGKNTVCVEIFAILTSSAGLLACIFLPKCYIIQFRSEINTKSQLLGNRQN
>Western_clawed_frog.locus40.1
------MEWPNRKKTKCIPKTEDYLSYH-
ILFAFFSATSALFFLTSLLILGVFIAHRKTPIVRANNRSLSFLLLVSIKLSFLSVFLFLGRPVDITCMLRIITFGITFSIAVSSLLAKTIMVCVAFKATKPGSSWRKWLGVKLSNSVVLFCSSIQIIICMTWLAISPPFQELDIHTSPG
TIIIQCNEGSAIGFYSVIGYMGLLAAVSFVLAFLARSLPDSFNEAKYITFSMLLFCSVWITMIPAYLSTKGKNTVCVEIFAILTSSAGLLACIFLPKCYIIQFRSEINTKSQLFGNRQN
>Western_clawed_frog.locus45.1
------MEWPNRKKTKCIPKTVDYLSYH-
ILSPFFSATSALFFLTSLLILGVFIAHRETPIVRANNRSLSFLLLVSIKLSFLSVFLFLGRPVDITCMLRIITFGITFSIAVSSLLAKTIMVCVAFKATKPGSSWRKWLGVKLSNSVVLFCSSIQIIICMTWLAISPPFQELDIHTSPG
TIIIQCNEGSDIGFYSVIGYMGLLAAVSFVLAFLARSLPDSFNEAKYITFSMLLFCSVWITMIPAYLSTKGKNTVCVEIFAILTSSAGLLACIFLPKCYIIQFRSEINTKSQLFGNRQN
>Western_clawed_frog.locus46.1
------MEWPNRKKTKCIPKTVDFLSYH-
ILSAFFSGTSALFFLTSLLILGVFITHRETPIVRANNRSLSFLLLVSIKLSFLSVFLFLGRPVDITCMLRIITFGITFSIAVSSLLAKTIMVCVAFKATKPGSSWRKWLGVKLSNSVVLFCSSIQIIICMTWLAISPPFQELDIHTSPG
TIIIQCNEGSAIGFYSVIGYMGLLAAVSFVLAFLARSLPDSFNEAKYITFSMLLFCSVWITMIPAYLSTKGKNTVCVEIFAILTSSAGLLACIFLPKCYIIQFRSEINTKSQLLGNRQN
>Western_clawed_frog.locus51.1
------MEWPNRKKTECIPKTVDFLSYH-
ILSAFFSATSVLFFLTSLLILGVFIAHRGTPIVRANNRSLSFLLLVSIKLSFLSVFLFLGRPVDITCMLRIITFGITFSIAVSSLLAKTIMVCVAFKATKPGSSWRKWLGVKLSNSVVLFCSSIQIIICMTWLAISPPFQELDIHTSPG
TIIIQCNEGSAIGFYSVIGYMGLLAAVSFVLAFLARSLPDSFNEAKYITFSMLLFCSVWITMIPAYLSTKGKNTVCVEIFAILTSSAGLLACIFLPKCYIIQFRSEINTKSQLLGNRQN
>Western_clawed_frog.locus48.1
------MEWPNRKKTECIPKTVDFLSYH-
ILSAFFSATSALFFIASLLILGVFIAHRETPIVRANNRSLSFLLLVSIKLSFLSVFLFLGRPVDITCMLRIITFGITFSIAVSSLLAKTIMVCVAFKATKPGSSWRKWLGVKLSNSVVLFCSSIQIIICMTWLAISPPFQELDIHTSPG
TIIIQCNEGSAIGFYSVIGYMGLLAAVSFVLAFLARSLPDSFNEAKYITFSMLLFCSVWITMIPAYLSTKGKNTVCVEIFAILTSSAGLLACIFLPKCYTILLRPEMNTKNNLLGNKDN
>Western_clawed_frog.locus203.1

MSIFFSAASAVFFLAVLVILGVFIAHRETPIVRANNRSLSFFLLVSIKLSFLSVFLFLGRPVDITCMLRIITFGITFSIAVSSLLAKTIMVCVAFKATKPGSSWRKWLGVKLSNSVVLFCSSIQIIICMTWLAISPPFQELDIHTSPGT
IIIQCNEGSAIGFYSVIGYMGLLAAVSFVLAFLARSLPDSFNEAKYITFSMLLFCSVWITMIPAYLSTKGKNTVCVEIFAILTSSAGLLACIFLPKCYIILFRPEINTKSHLLENK--
>Western_clawed_frog.locus274.1

MEWSSKKRTQCIAKMEDFLVNTSAFAVFFSASSLLFFLTTLLILGVFIAHRETPIVRANNRSLSFLLLVSIKLSFLSVFLFLGRPVDITCMLRIITFGITFSIAVSSLLAKTIMVCVAFKATKPGSSWRKWLGVKLSNSVVLFCSSIQI
IICMTWLAISPPFQELDIHTSPGTIIIQCNEGSAIGFYSVIGYMGLLAAVSFVLAFLARSLPDSFNEAKYITFSMLLFCSVWITMIPAYLSTKGKNTVCVEIFAILTSSAGLLACIFLPKCYSIFCRPKLNTKSYIIRMKA-
>Western_clawed_frog.locus271.1
CIPCPKMEWSSKKRIQCIAKMEDFLVNS-
AFAVFFSASSLLFFLTTLLILGVFIAHRDTPIVRANNRSLSFLLLVSIKLSFLSVFLFLGRPVDITCMLRIITFGITFSIAVSSLLAKTIMVCVAFKATKPGSSWRKWLGVKLSNSVVLFCSSIQIIICMTWLAISPPFQELDIHTSPG
TIIIQCNEGSAIGFYSVIGYMGLLAAVSFVLAFLARSLPDSFNEAKYITFSMLLFCSVWITMIPAYLSTKGKNTVCVEIFAILTSSAGLLACIFLPKCYFIQFRSEMNTKSNLLGNRQY
>Western_clawed_frog.locus278.1
------MEWSNKKRIQCIAKMEDFLVYN-
VFSVFFSASSVLFFLTTLLILEVFIAHRDTPIVRANNRSLSFLLLVSIKLSFLSVFLFLGRPVDITCMLRIITFGITFSIAVSSLLAKTIMVCVAFKATKPGSSWRKWLGVKLSNSVVLFCSSIQIIICMTWLAISPPFQELDIHTSPG
TIIIQCNEGSAIGFYSVIGYMGLLAAVSFVLAFLARSLPDSFNEAKYITFSMLLFCSVWITMIPAYLSTKGKNTVCVEIFAILTSSAGLLACIFLPKCYVILFRTEMNTKS-LLGNRQY
>Western_clawed_frog.locus275.1
------MEWSNKKRTQCIAKMEDFLVYN-
VISVFFSASSVLFFLTTLLILGVFIAHRDTPIVRANNRSLSFLLLVSIKLSFLSVFLFLGRPVDITCMLRIITFGITFSIAVSSLLAKTIMVCVAFKATKPGSSWRKWLGVKLSNSVVLFCSSIQIIICMTWLAISPPFQELDIHTSPG
TIIIQCNEGSAIGFYSVIGYMGLLAAVSFVLAFLARSLPDSFNEAKYITFSMLLFCSVWITMIPAYLSTKGKNTVCVEIFAILTSSAGLLACIFLPKCYIIQFRSEMNTKSNLFRNRQY
>Western_clawed_frog.locus277.1
------MEWSNKKRTQCIAKMEDVLVYN-
VISVFFSASSVLFFLTTLLILGVFIAHRDTPIVRANNRSLSFLLLVSIKLSFLSVFLFLGRPVDITCMLRIITFGITFSIAVSSLLAKTIMVCGAFKATKPGSSWRKWLGVKLSNSVVLFCSSIQIIICMTWLAISPPFQQLDIHTSPG
TIIIQCNEGSAIGFYSVIGYMGLLAAVSFVLAFLARSLPDSFNEAKYITFSMLLFCSVWITMIPAYLSTKGKNTVCVEIFAILTSSAGLLACIFLPKCYIIQFRSEMNTKSNLFRNRQY
>Western_clawed_frog.locus272.1
------MEWSNKKRTKCIAKIEDFLVYN-
VISEFFSASSVLFFLTTLLILGVFIAHRETPIVRANNRSLSFLLLVSIKLSFLSVFLFLGRPVDITCMLRIITFGITFSIAVSSLLAKTIMVCVAFKATKPGSSWRKWLGVKLSNSVVLFCSSIQIIICMTWLAISPPFQELDIHTSPG
TIIIQCNEGSAIGFYSVIGYMGLLAAVSFVLAFLARSLPDSFNEAKYITFSMLLFCSVWITMIPAYLSTKGKNTVCVEIFAILTSSAGLLACIFLPKCYIIQFRSEMNTKS-LLGNRQY
>Western_clawed_frog.locus270.1
CIPCPKMEWSNKKRTQCIAKMEDLLVYN-
VISVFFSASSVLFFLTTLLILGVFIAHRETPIVRANNRSLSFLLLVSIKLSFLSVFLFLGRPVDITCMLRIITFGITFSIAVSSLLAKTIMVCVAFKATKPGSSWRKWLGVKLSNSVVLFCSSIQIIICMTWLAISPPFQELDIHTSPG
TIIIQCNEGSAIGFYSVIGYMGLLAAVSFVLAFLARSLPDSFNEAKYITFSMLLFCSVWITMIPAYLSTKGKNTVCVEIFAILTSSAGLLACIFLPKCYIILFRSAMNTKSNLLGNRQY
>Western_clawed_frog.locus269.1
------MEWSNKKRTQCIAKMEDVLVYN-
VISVFFSASSVLFFLTTLLILGVFIEHRETPIVRANNRSLSFLLLVSIKLSFLSVFLFLGRPVDITCMLRIITFGITFSIAVSSLLAKTIMVCVAFKATKPGSSWRKWLGVKLSNSVVLFCSSIQIIICMTWLAISPPFQELDIHTSPG
TIIIQCNEGSAIGFYSVIGYMGLLAAVSFVLAFLARSLPDSFNEAKYITFSMLLFCSVWITMIPAYLSTKGKNTVCVEIFAILTSSAGLLACIFLPKCYIILFRTEMNRKSNLLGNRQY
>Western_clawed_frog.locus113.1
CIRCPDLEWPNKNRTICILKLEEFLSYNDAIAVVLSSISVLFFLITLLVLVVFISNHDSPIVRANNRSLSFLLLVSIKLSFLSVFLFLGRPVDITCMLRIITFGITFSIAVSSLLAKTIMVCVAFKATKPGSSWRKWLGVKLSNSVVLF
CSSIQIIICMTWLAISPPFQELDIHTSPGTIIIQCNEGSAIGFYSVIGYMGLLAAVSFVLAFLARSLPDSFNEAKYITFSMLLFCSVWITMIPAYLSTKGKNTVCVEIFAILTSSAGLLASIFLPKCYTILYRPEINTKAYLLGNKSL
>Western_clawed_frog.locus111.1
CIRCPDLEWPNKNRTICILKLEEFLSYNDAIAVVLLSISVLFFLITLLVLVVFISNHDTPIVRANNRSLSFLLLVSIKLSFLSVFLFLGRPVDITCMLRIITFGITFSIAVSSLLAKTIMVCVAFKATKPGSSWRKWLGVKLSNSVVLF
CSSIQIIICMTWLAISPPFQELDIHTSPGTIIIQCNEGSAIGFYSVIGYMGLLAAVSFVLAFLARSLPDSFNEAKYITFSMLLFCSVWITMIPAYLSTKGKNTVCVEIFAILTSSAGLLASIFLPKCYTILYRPEINTKANLLRNKSL
>Western_clawed_frog.locus112.1
CIRCPDLEWPNKNRTICILKLEEFLSYNDAIAVVLLSISVLFFLITLLVLVVFISNHDTPIVRANNRSLSFLLLVSIKLSFLSVFLFLGRPVDITCMLRIITFGITFSIAVSSLLAKTIMVCVAFKATKPGSSWRKWLGVKLSNSVVLF
CSSIQIIICMTWLAISPPFQELDIHTSPGTIIIQCNEGSAIGFYSVIGYMGLLAAVSFVLAFLARSLPDSFNEAKYITFSMLLFCSVWITMIPAYLSTKGKNTVCVEIFAILTSSAGLLACIFLPKCYTILYRPEINTKSNLLGNKSL
>Western_clawed_frog.locus104.1
CIRCPDLEWPNKNRTICILKLEEFLSYNDAIAVVLSSISVFFFLITLLVLVVFISNHDTPIVRANNRSLSFLLLVSIKLSFLSVFLFLGRPVDITCMLRIITFGITFSIAVSSLLAKTIMVCVAFKATKPGSSWRKWLGVKLSNSVVLF
CSSIQIIICMTWLAISPPFQELDIHTSPGTIIIQCNEGSAIGFYSVIGYMGLLAAVSFVLAFLARSLPDSFNEAKYITFSMLLFCSVWITMIPAYLSTKGKNTVCVEIFAILTSSAGLLACMFLPKCYIIVFRVEINVKSQLFGNKLK
>Western_clawed_frog.locus114.1
CIRCPDLEWPNKNRTICIVKTEDFLSYNDAIAVVLSSISVLFFFLTLLIFVVFLTYRGTPIVRANNRSLSFLLLVSIKLSFLSVFLFLGRPVDITCMLRIITFGITFSIAVSSLLAKTIMVCVAFKATKPGSSWRKWLGVKLSNSVVLF
CSSIQIIICMTWLAISPPFQELDIHTSPGTIIIQCNEGSAIGFYSVIGYMGLLAAVSFVLAFLARSLPDSFNEAKYITFSMLLFCSVWITMIPAYLSTKGKNTVCVEIFAILTSSAGLLASIFLPKCYTILYRPEMNTKCHFLGNKT-

```
>Western_clawed_frog.locus160.1
CIRCPDLEWPNKNRTICIAKTEDFLSYNDAIAVVLSSISVFFFFLSLLIFVVFLTFRDSPIVRANNRSLSFLLLVSIKLSFLSVFLFLGRPVDITCMLRIITFGITFSIAVSSLLAKTIMVCVAFKATKPGSSWRKWLGVKLSNSVVLF
CSSIQIIICMTWLAISPPFQELDIHTSPGTIIIQCNEGSAIGFYSVIGYMGLLAAVSFVLAFLARSLPDSFNEAKYITFSMLLFCSVWITMIPAYLSTKGKNTVCVEIFAILTSSAGLLACIFLPKCYIILFRPEINTKSYLVRIQF-
>Western_clawed_frog.locus136.1
CIRCPDLEWPNKNRTICIVKTEDFLSYNDAITVVLSSISALFFFLTLLILGVFIANRDSPIVRANNRSLSFLLLVSIKLSFLSVFLFLGRPVDITCMLRIITFGITFSIAVSSLLAKTIMVCVAFKATKPGSSWRKWLGVKLSNSVVLF
CSSIQIIICMTWLAISPPFQELDIHTSPGTIIIQCNEGSAIGFYSVIGYMGLLAAVSFVLAFLARSLPDSFNEAKYITFSMLLFCSVWITMIPAYLSTKGKNTVCVEIFAILTSSAGLLACIFLPKCYIILLRPEMNSKSNLLGNKSL
>Western_clawed_frog.locus159.1
CIRCPDLEWPNKNRTICIVKTEDFLSYNDAIAVVLSSISVLFFFFSLLILGVFIANRDSPIVRANNRSLSFLLLVSIKLSFLSVFLFLGRPVDITCMLRIITFGITFSIAVSSLLAKTIMVCVAFKATKPGSSWRKWLGVKLSNSVVLF
CSSIQIIICMTWLAISPPFQELDIHTSPGTIIIQCNEGSAIGFYSVIGYMGLLAAVSFVLAFLARSLPDSFNEAKYITFSMLLFCSVWITMIPAYLSTKGKNTVCVEIFAILTSSAGLLACIFLPKCYTILYRPEINTKSNLLGNKSL
>Western_clawed_frog.locus190.1
CITCPDLEWPNKNRTICIVKTEDCLSYNDVISIIFSSISVLFFLITLLILVVFISNHDTPIVRANNRSLSFLLLVSIKLSFLSVFLFLGRPVDITCMLRIITFGITFSIAVSSLLAKTIMVCVAFKATKPGSSWRKWLGVKLSNSVVLF
CSSIQIIICMTWLAISPPFQELDIHTSPGTIIIQCNEGSAIGFYSVIGYMGLLAAVSFVLAFLARSLPDSFNEAKYITFSMLLFCSVWITMIPAYLSTKGKNTVCVEIFAILTSSAGLLACIFLPKCYTILYRPEMNKKSHLLGNKAS
>Western_clawed_frog.locus231.1
------
MEWPNKKRTICIAKTEVFLSYNDVISVILSSISVFFFVITVMILGVFIINRDTPIVRANNQSLSFLLLVSIKLSFLSVFLFLGRPVDITCMLRIITFGITFSIAVSSLLAKTIMVCVAFKATKPGSSWRKWLGVKLSNSVVLFCSSIQI
IICMTWLAISPPFQELDIHTSPGTIIIQCNEGSAIGFYSVIGYMGLLAAVSFVLAFLARSLPDSFNEAKYITFSMLLFCSVWITMIPAYLSTKGKNTVCVEIFAILTSSAGLLACIFLPKCYTIFFKPEMNTKSQLLGNKLH
>Western_clawed_frog.locus233.1
------
MEWPNKKRTICIAKTEVFLSYNDVISVIFSSISVFFFVITVMILGVFIINRDSPIVRANNRSLSFLLLVSIKLSFLSVFLFLGRPVDITCMLRIITFGITFSIAVSSLLAKTIMVCVAFKATKPGSSWRKWLGVKLPNSVVLFCSSIQI
IICMTWLAISPPFQELDIHTSPGTIIIQCNEGSAIGFYSVIGYMGLLAAVSFVLAFLARSLPDSFNEAKYITFSMLLFCSVWITMIPAYLSTKGKNTVCVEIFAILTSSTGLLACIFLPKCYTIFFKPEMNTKSQLLGNKLH
>Western_clawed_frog.locus230.1
------
MEWPNKKRTFCIAKTEAFLSYNDVISVIFSSISVFFFVITVMILGVFIINQDSPIVRANNRSLSFLLLVSIKLSFLNVFLFLGRPVDITCMLRIITFGITFSIAVSSLLAKTIMVCVAFKATKPGSSWRKWLGVKLSNSVVLFCSSIQI
IICMTWLAISPPFQELDIHTSPGTIIIQCNEGSAIGFYSVIGYMGLLAAVSFVLAFLARSLPDSFNEAKYITFSMLLFCSVWITMIPAYLSTKGKNTVCVEIFAILTSSAGLLACIFLPKCYTIFFKPEMNMKSQLFGNKQH
>Western_clawed_frog.locus232.1
------
MEWPNKKRTFCIAKTEVFLSYNDVISVIFSSISVFFFVITVMILGVFIINQDTPIVRANNRSLSFLLLVSIKLSFLSVFLFLGRPVDITCMLRIITFGITFSIAVSSLLAKTIMVCVAFKATKPGSSWRKWLGVKLSNSVVLFCSSIQI
IICMTWLAISPPFQELDIHTSPGTIIIQCNEGSAIGFYSVIGYMGLLAAVSFVLAFLARSLPDSFNEAKYITFSMLLFCSVWITMIPAYLSTKGKNTVCVEIFAILTSSAGLLACIFLPKCYTIFFKPEMNTKSQLLGNKLH
>Western_clawed_frog.locus237.1
------
MEWPNKKRTICIAKTEVFLSYNDVISVIFSSISVFFFVITVMILGVFIINQDSPIVRANNRSLSFLLLVSIKLSFLSVFLFLGRPVDITCMLRIITFGIPFSIAVSSLLAKTIMVCVAFKATKPGSSWRKWLGVKLSNSVVLFCSSIQI
IICMTWLAISPPFQELDIHTSPGTIIIQCNEGSAIGFYSVIGYMGLLAAVSFVLAFLARSLPDSFNEAKYITFSMLLFCSVWITMIPAYLSTKGKNTVCVEIFAILTSSAGLLACIFLPKCYTICFKPEMNTKSQLLGNKLQ
>Western_clawed_frog.locus234.1
------
MEWPNKKRTICIAKTEVFLSYNDAISVIFSSISVFFFVITVMILGVFIINQDSPIVRANNRSLSFLLLVSIKLSFLSVFLFLGRPVDITCMLRIITFGITFSIAVSSLLAKTIMVCVAFKATKPGSSWRKWLGVKLSNSVVLFCSSIQI
IICMTWLAISPPFQELDIHTSPGTIIIQCNEGSAIGFYSVIGYMGLLAAVSFVLAFLARSLPDSFNEAKYITFSMLLFCSVWITMIPAYLSTKGKNTVCVEIFAILASSAGLLACIFLPKCYTIFFKPEMNTKSQLLGNKLQ
>Western_clawed_frog.locus121.1
------
MEWPNNNRTNCIARTEDFLSLNDVISVFFSSVSLLLFVITLLILGVFISHQDTPIVRANNRSLSFLLLVSIKLSFLSVFLFLGRPVDITCMLRIITFGITFSIAVSSLLAKTIMVCVAFKATKPGSSWRKWLGVKLSNSVVLFCSSIQI
IICMTWLAISPPFQELDIHTSPGTIIIQCNEGSAIGFYSVIGYMGLLAAVSFVLAFLARSLPDSFNEAKYITFSMLLFCSVWITMIPAYLSTKGKNTVCVELFAILTSSAGLLGCIFLPKCYAILLRPEKNTKSHLFANKSQ
>Western_clawed_frog.locus163.1
--------------------
MEDFLSYSDGISVFFYSISVLFFLLNLLIFGVFIRHLDTPIVRANNRSLSFLLLVSIKLSFLSVFLFLGRPVDITCMLRIITFGITFSIAVSSLLAKTIMVCVAFKATKPGSSWRKWLGVKLSNSVVLFCSSIQIIICMTWLAISPPFQ
ELDIHTSPGTIIIQCNEGSAIGFYSVIGYMGLLAAVSFVLAFLARSLPDSFNEAKYITFSMLLFCSVWITMIPAYLSTKGKNTVCVEIFAILTSSAGLLASIFLPKCYIILLKCQMNTKSHLLGNKH-
>Western_clawed_frog.locus162.1
------
MEWPNEKRNQCIAKMEDFLSYSDGISVLFYSISVLFFLLNVLILGVFIRHLDTPIVRANNRSLSFLLLVSIKLSFLCVFLFLGRPVDITCMLRIITFGITFSIAVSSLLAKTIMVCVAFKATKPGSSWRKWLGVKLSNSVVLFCSSIQI
IICMTWLAISPPFQELDIHTSPGTIIIQCNEGSAIGFYSVIGYMGLLAAVSFVLAFLARSLPDSFNEAKYITFSMLLFCSVWITMIPAYLSTKGKNTVCVEIFAILTSSAGLLASIFLPKCYIILFRPERNMKSAMFGNKTE
>Western_clawed_frog.locus294.1
------
MEWPNKENTRCIPKKEDFLSYTDVISIVLSSISVLFLLITFLILGVFIKYCDTPIVRANNRSLSFLLLVSIKLSFLSVFLFLGRPGDITCRLRNITYGIAFSIAVSSLLAKTIMVYIAFKSTKPGSSWGKWMGVKLSRSVVLVFSSIQI
IICITWLAISPPFQELDIHTYPGTIIIQCNEGSALGFYSVIGYMGLLAAVSFVLAFLARSLPDSFNEAKYITFSMLLFCSVWITMIPAYLSTKGKNTVCVEIFAIVTSSAGLLGCIFLTKCYIILFRPKINVRSQLFGNKTR
>Western_clawed_frog.locus295.1
------
MEWPNKKNTRCIPKKEDFLSYTDVISIVLSSISVLFLLITFLILGVFIKYRDTPIVRANNRSLSFLLLVSIKLSFLSVFLFLGRPGDITCRLRNITYGIAFSIAVSSLLAKTIMVYIAFKSTKPGSSWGKWMGVKLSKSVVLVFSSIQI
IICITWLAISPPFQELDIHTYPGTIIIQCNEGSALGFYSVIGYMGLLAAVSFVLAFLARSLPDSFNEAKYITFSMLLFCSVWITMIPAYLSTKGKNTVCVEIFAIVTSSAGLLGCIFVTKCYIILFRPKMNVRSQLLGNKTR
>Western_clawed_frog.locus296.1
CFRCPDLEWPNKENTQCIPKKEDFLSYTDVISIVLSSISVLFLLITFLILGVFIKYSDTPIVRANNRSLSFLLLVSIKLSFLSVFLFLGRPGDITCRLRNITYGIAFSIAVSSLLAKTIMVYIAFKSTKPGSSWGKWMGVKLSKSVVLV
FSSIQIIICITWLAISPPFQELDIHTYPGTIIIQCNEGSALGFYSVIGYMGLLAAVSFVLAFVARSLPDSFNEAKYITFSMLLFCSVWITMIPAYLSTKGKNTVCVEIFAIVTSSAGLLGCIFLTKCYIILFRPKMNVRSQLFGNKPR
>Western_clawed_frog.locus286.1
CFRCPDLEWPNKENTQCIPKKEDFLSYTDVISIVLSSISVLFLLITFLILGVYIKYRDTPIVRANNRSLSFLLLVSIKLSFLSVFLFLGRPGDITCRLRNITYGIAFSIAVSSLLAKTIMVYIAFKSTKPGSSWGKWMGVKLSRSVVLV
FSSIQIIICITWLAISPPFQELDIHTYPGTIIIQCNEGSALGFYSVIGYMGLLAAVSFVLAFLARSLPDSFNEAKYITFSMLLFCSVWITMIPAYLSTKGKNTVCVEIFAIVTSSAGLLGCIFLPKCYIILFRPEINVRSKLLGNKSR
>Western_clawed_frog.locus288.1
------
MEWPNKEKTQCIPKKEDFLSYTDVISIVLSSISVLFLLITFLILGVFIKYSDTPIVRANNRSLSFLLLVSIKLSFLSVFLFLGRPGDITCRLRNITYGIAFSIAVSSLLAKTIMVYIAFKSTKPGSSWGKWMGVKLSKSVVLVFSSIQI
IICITWLAISPPFQELDIHTYPGTIIIQCNEGSALGFYSVIGYMGLLAAVSFVLAFLARSLPDSFNEAKYITFSMLLFCSVWITMIPAYLSTKGKNTVCVEIFAIVTSSAGLLGCIFLPKCYIILFRPEMNVRSQLFGNKSR
>Western_clawed_frog.locus291.1
CFRCPDLEFPNKRRNQCIPKKEDFLSYTDVISIVLSSISVLFLLITFLILGVFIKYRDTPIVRANNRSLSFLLLVSIKLSFLSVFLFLGRPGDITCRLRNITYGIAFSIAVSSLLAKTIMVYIAFKSTKPGSSWEKWMGVKLSRSVVLV
FSSIQIIICITWLAISPPFQELDIHTYPGTIIIQCNEGSALGFYSVIGYMGLLAAVSFVLAFLARSLPDSFNEAKYITFSMLLFCSVWITMIPAYLSTKGKNTVCVEIFAIVTSSAGLLGCIFLTKCYIILFRPEMNVRSQLLGNKTR
>Western_clawed_frog.locus287.1
CFRCPDLEFPNKRRNQCIPKKEDFLSYTDVISIVLSSISVLFLLITFLILGVFIKYRDSPIVRANNRSLSFLLLVSIKLSFLSVFLFLGRPGDITCRLRNITYGIAFSIAVSSLLAKTIMVYIAFKSTKPGSSWEKWMGVKLSRSVVLV
FSSIQIIICITWLAISPPFQELDIHTYPGTIIIQCNEGSALGFYSVIGYMGLLAVVSFVLAFLARSLPDSFNEAKYITFSMLLFCSVWITMIPAYLSTKGKNTVCVEIFAIVTSSAGLLGCIFLTKCYIIFFRPEFHVKSHLFRNKTK
>Western_clawed_frog.locus292.1
CFRCPDLEFPNKRRNQCIPKKEDFLSYTDVISIVLSSISVLFLLITFLILGVFIKYRDSPIVRANNRSLSFLLLVSIKLSFLSVFLFLGRPGDITCRLRNITYGIAFSIAVSSLLAKTIMVYIAFKSTKPGSSWGKWMGVKLSKSVVLV
FSSIQIIICITWLAISPPFQELDIHTYPGTIIIQCNEGSALGFYSVIGYMGLLAAVSFVLAFLARSLPDSFNEAKYITFSMLLFCSVWITMIPAYLSTKGKNTVCVEIFAIVTSSAGLLGCIFLTKCYIILFRPKMNIRSQLFGNKTR
>Western_clawed_frog.locus285.1
CFRCPDLEWPNNRKNQCIPKKEDFLSYTDVISIVLSSVSVLFLLITFLILGVFIKYRDSPIVRANNRSLSFLLLVSIKLSFLSVFLFLGRPGDITCRLRNITYGIAFSIAVSSLLAKTIMVYIAFKSTKPGSSWGKWMGVKLSKSVVLV
FSSIQIIICITWLAIFPPFQELDIHTYPGTIIIQCNEGSALGFYSVIGYMGLLAAVSFVLAFLARSLPDSFNEAKYITFSMLLFCSVWITMIPAYLSTKGKNTVCVEIFAIVTSSAGLLGCIFLPKCYIILFRPKMNVRSQLLGNKSR
>Western_clawed_frog.locus290.1
CFRCPDLEWPNNRKNQCIPKKEDFLSYTDVISIVLSSISVLFLLITFLILGVFIKYRDSPIVRANNRSLSFLLLVSIKLSFLSVFLFLGRPGDITCRLRNITYGIAFSIAVSSLLAKTIMVYIAFKSTKPGSSWGKWMGVKLSKSVVLV
FSSIQIIICMTWLAISPPFQELDIHTYPGTIIIQCNEGSALGFYSVIGYMGLLAAVSFVLAFLARSLPDSFNEAKYITFSMLLFCSVWITMIPAYLSTKGKNTVCVEIFAIVTSSAGLLGCIFLSKCYIILFRPEMNVKSQLLGNKPR
>Western_clawed_frog.locus215.1
------
MEWPNKRKNQCIPRREDFLSYSDKISVCLSVIAILFFLITLWISAVFVTHRETVIVRANNRSLSFLLLVSIKLSFLSVFLFLGRPVDITCMLRIITFGITFSIAVSSLLAKTIMVCVAFKATKPGSSWRKWLGVKLSNSVVLFCSSIQI
IICMTWLAISPPFQELDIHTSPGTIIIQCNEGSAIGFYSVIGYMGLLAAVSFVLAFLARSLPDSFNEAKYITFSMLLFCSVWITMIPAYLSTKGKNTVCVEIFAILTSSAGLLASIFLPKCYIILFKPEINVKSHLFGNKSR
>Western_clawed_frog.locus218.1
CLRCPAIEWTNEWRNQCFPKTEDFLLHTDAIAVVLSSLAVSLFVLTLFILGVLITYQDTPIVRANNRSLSFLLLVSIKLSFLSVFLFLGRPVDITCMLRIITFGITFSIAVSSLLAKTIMVCVAFKATKPGSSWRKWLGVKLSNSVVLF
CSSIQIIICMTWLAISPPFQELDIHTSPGTIIIQCNEGSAIGFYSVIGYMGLLAAVSFVLAFLARSLPDSFNEAKYITFSMLLFCSVWITMIPAYLSTKGKNTVCVEIFAILTSSAGLLACIFLPKCYIIMFRPEMNTKSNLLYN-V-
>Western_clawed_frog.locus219.1
CLRCPAIEWTNEWRNQCFPKTEDFLLHTDAIAVVLSSLAVSLFVLTLFILGVFITYQDTPIVRANNRSLSFLLLVSIKLSFLSVFLFLGRPVDITCMLRIITFGITFSIAVSSLLAKTIMVCVAFKATKPGSSWRKWLGVKLSNSVVLF
CSSIQIIICMTWLAISPPFQELDIHTSPGTIIIQCNEGSAIGFYSVIGYMGLLAAVSFVLAFLARSLPDTFNEAKYITFSMLLFCSVWITMIPAYLSTKGKNTVCVEIFAILTSSAGLLACIFLPKCYIILFRPEINVKSHVFGKNM-
>Western_clawed_frog.locus216.1
CLRCPAIEWTNEWRNQCFAKTEDFLLHTDAIVVVLSSLAVSLFVLTLFILGVFIKNQDTPIVRANNRSLSFLLLVSIKLSFLSVFLFLGRPVDITCMLRIITFGITFSIAVSSLLAKTIMVCVAFKATKPGSSWRKWLGVKLSNSVVLF
CSSIQIIICMTWLAISPPFQELDIHTSPGTIIIQCNEGSAIGFYSVIGYMGLLAAVSFVLAFLARSLPDSFNEAKYITFSMLLFCSVWITMIPAYLSTKGKNTVCVEIFAILTSSAGLLACIFLPKCYIILFRPEINVKSHVFGKNM-
>Western_clawed_frog.locus221.1
CHRCPAIEWTNEWRNQCIAKTEDFLLHTDAIAVVLSSLAVSLFVLTLFILGVFITYQETPIVRVNNRSLSFLLLVSIKLSFLSVFLFLGRPVDITCMLRIITFGITFSIAVSSLLAKTIMVCVAFKATKPGSSWRKWLGVKLSNSVVLF
CSSIQIIICMTWLAISPPFQELDIHTSPGTIIIQCNEGSAIGFYSVIGYMGLLAAVSFVLAFLARSLPDSFNEAKYITFSMLLFCSVWITMIPAYLSTKGKNTVCVEIFAILASSAGLLACIFLPKCYIILFRPEINVKSHVFGKNM-
>Western_clawed_frog.locus222.1
CLRCPAIEWTNEWRNQCIAKTEDFLLHTDAIAVVLSSLAVTFFVLTLFILGVFLTYQDTPIVRANNRGLSFLLLVSIKLSFLSVFLFLGRPVDITCMLRIITFGITFSIAVSSLLAKTIMVCVAFKATKPGSSWRKWLGVKLSNSVVLF
CSSIQIIICMTWLAISPPFQELDIHTSPGTIIIQCNEGSAIGFYSVIGYMGLLAAVSFVLAFLARSLPDSFNEAKYITFSMLLFCSVWITMIPAYLSTKGKNTVCVEIFAILASSAGLLACIFLPKCFIILFRPEINVKSHVFGKNM-
>Western_clawed_frog.locus224.1
CLRCPAIEWTNEWRNQCIAKTEDFLLHTDAIAVVLSSLAVSLFVLTLFMLGVFITYQDTPIVRANNRSLSFLLLVSIKLSFLSVFLFLGRPVDITCMLRIITFGITFSIAVSSLLAKTIMVCVAFKATKPGSSWRKWLGVKLSNSVVLF
CSSIQIIICMTWLAISPPFQELDIHTSPGTIIIQCNEGSAIGFYSVIGYMGLLAAVSFVLAFLARSLPDSFNEAKYITFSMLLFCSVWITMIPAYLSTKGKNTVCVEIFAILTSSAGLLACIFLPKCYIILFRPEINVKSHVFGKNM-
>Western_clawed_frog.locus217.1
CLRCPAIEWTNEWRNQCIAKTEDFLLYTDAIAVVLSSLAVSLFLLTLFILGVFITYQDTPIVRANNRSLSFLLLVSIKLSFLSVFLFLGRPVDITCMLRIITFGITFSIAVSSLLAKTIMVCVAFKATKPGSSWRKWLGVKLSNSVVLF
CSSIQIIICMTWLAISPPFQELDILTSPGTIIIQCNEGSAIGFYSVIGYMGLLAAVSFVLAFLARSLPDSFNEAKYITFSMLLFCSVWITMIPAYLSTKGKNTVCVEIFAILTSSAGLLACIFLPKCYIILFRPEINVKSHLFGKNM-
>Western_clawed_frog.locus53.1
CLRCPDIEWPNEWRNQCFAKTVDFLSYTDAISVVLSSVSVLFFFLSLLILGVFIKHRDSPIVRANNRSLSFLLLVSIKLSFLSVFLFLGRPVDITCMLRIITFGITFSIAVSSLLAKTIMVCVAFKATKPGSSWRKWLGVKLSNSVVLF
CSSIQIIICMTWLAISPPFQELDIHTSPGTIIIQCNEGSAIGFYSVIGYMGLLAAVSFVLAFLARSLPDSFNEAKYITFSMLLFCSVWITMIPAYLSTKGKNTVCVEIFAILTSSAGLLASIFLPKCYIILVVPEINVKSNLFGNKPG
>Western_clawed_frog.locus49.1
-------------MNGGIS-----
VLHTDAISVVLSSVSVLFFFLSLLIFRVFIKHRETPIVRANNRSLSFLLLVSIKLSFLSVFLFLGRPVDITCMLRIITFGITFSIAVSSLLAKTIMVCVAFKATKPGSSWRKWLGVKLSNSVVLFCSSIQIIICMTWLAISPPFQELDI
HTSPGTIIIQCNEGSAIGFYSVIGYMGLLAAVSFVLAFLARSLPDSFNEAKYITFSMLLFCSVWITMIPAYLSTKGKNTVCVEIFAILTSSAGLLACIFLPKCYLILVVPEINVKSNLFGNKPG
>Western_clawed_frog.locus9.1
------
MEWPNEQKDQCIPKIEQFLSYNDVISVVLSSISVLFFLITLLILGVFISYWDTPIVRANNRSLSFLLLVSIKLSFLSVFLFLGRPVDITCMLRIITFGITFSIAVSSLLAKTIMVCVAFKATKPGSSWRKWLGVKLSNSVVLFCSSIQI
IICMTWLAISPPFQELDIHTSPGTIIIQCNEGSAIGFYSVIGYMGLLAAVSFVLACLARSLPDSFNEAKYITFSMLLFCSVWITMIPAYLSTKGKNTVCVEIFAVLTSSAGLLACIFLPKCYVIFLLPERNTKSHMLGNKRQ
>Western_clawed_frog.locus8.1
```

```
------
MEWPNDQKDQCIPKIEEFLSYNDVISVVLSSISVLFFLITLLILGVFISYWDTPIVRANNRSLSFLLLVSIKLSFLSVFLFLGRPVDITCMLRIITFGITFSIAVSSLLAKTIMVCVAFKATKPGSSWRKWLGVKLSNSVVLFCSSIQI
IICMTWLAISPPFQELDIHTSPGTIIIQCNEGSAIGFYSVIGYMGLLAAVSFVLAFLARSLPDSFNEAKYITFSMLLFCSVWITMIPAYLSTKGKNTVCVEIFAILTSSAGLLACIFLPKCYIIYFLPVQNTKSYVLGNKD-
>Western_clawed_frog.locus166.1
------
MEWPTKKKKWCRPKRIEFLSYKDVISVLYAMFSFIFFLITVLVLAVFISYQDTPIVRANNRSLSFLLLVSIKLSFLSVFLFLGRPVDITCMLRIITFGITFSIAVSSLLAKTIMVCVAFKATKPGSSWRKWLGVKLSNSVVLFCSSIQI
IICMTWLAISPPFQELDIHTSPGTIIIQCNEGSAIGFYSVIGYMGLLAAVSFVLAFLARSLPDSFNEAKYITFSMLLFCSVWITMIPAYLSTKGKNTVCVEIFAILASNAGLLVCIFLPKCFIILFRPERNIKSVMFGRNNV
>Western_clawed_frog.locus61.1
------
MEWPDEMKKICIKKREAFLSYNEVISLVFSSVVVFFFLITMLILGMFVAYWDTPIVRANNRSLSFLLLVSIKLSFLSVFLFLGRPVDITCMLRIITFGITFSIAVSSLLAKTIMVCVAFKATKPGSSWRKWLGVKLSNSVVLFCSSIQI
IICMTWLAISPPFQELDIHTSPGTIIIQCNEGSAIGFYSVIGYMGLLAAVSFVLAFLARSLPDSFNEAKYITFSMLLFCSVWITMIPAYLSTKGKNTVCVEIFAILTSSAGLLACIFLPKCYIIMFKPKMNTKTNLLGKSQ-
>Western_clawed_frog.locus105.1
CIRCSDMEWPNKKRNQCIEKMEDFLSYNDVLTVIFASISVLLFVITLMILGVFITNWDTPIVRANNRSLSFLLLVSIKLSFLSVFLFLGRPVDITCMMRIITFGITFSIAVSSLLAKTIMVCVAFKATKPGSSWRKWLGVKLSNSVVLF
CSSIQIIICMTWLAISPPFQELDIHTSPGTIIIQCNEGSAIGFYSVIGYMGLLAAVSFVLAFLARSLPDSFNEAKYITFSMLLFCSVWITMIPAYLSTKGKNTVCVEIFAILTSSAGLLACIFLPKCYTILLRPDVNTKSFLLGTKP-
>Western_clawed_frog.locus106.1
------
MEWPNEKRNQCIEKMEDFLSYNDVLTVIFASISVLLFVITLMILTVFIIFRDTPVVRANNRSLSFLLLVSIKLSFLSVFLFLGRPVDITCMLRIITFGITFSIAVSSLLAKTIMVCVAFKATKPGSSWRKWLGVKLSNSVVLFCSSIQI
IICMTWLAISPPFQELDIHTSPGTIIIQCNEGSAIGFYSVIGYMGLLAAVSFVLAFLARSLPDTFNEAKYITFSMLLFCSVWITMIPAYLSTKGKNTVCVEIFAILTSSAGLLASIFLPKCYIILLRPEINTKSFLLGNKP-
>Western_clawed_frog.locus107.1
CIRCSDMEWPNEKRNKCTKKMEDFLSYNDVMTVIFSSISVLLFVITLMILGVFITNWDTPIVRANNRSLSFLLLVSIKLSFLSVFLFLGRPVDITCMLRIITFGITFSIAVSSLLAKTIMVCVAFKATKPGSSWRKWLGVKLSNSVVLF
CSSIQIIICMTWLAISPPFQELDIHTSPGTIIIQCNEGSAIGFYSVIGYMGLLAAVSFVLAFLARSLPDSFNEAKYITFSMLLFCSVWITMIPAYLSTKGKNTVCVEIFAILTSSAGLLASIFLPKCYVIMFRPEMNHKSGILGGNN-
>Western_clawed_frog.locus226.1
------------------------------
MSLIFLSISVLIFLVTLLILGVFITYQDTPIVRANNRSLSFLLLVSIKLSFLSVFLFLGRPVDITCMLRIITFGITFSIAVSSLLAKTIMVCVAFKATKPGSSWRKWLGVKLSNSVVLFCSSIQIIICMTWLAISPPFQELDIHTSPGT
IIIQCNEGSAIGFYSVIGYMGLLAAVSFVLAFLARSLPDSFNEAKYITFSMLLFCSVWITMIPAYLSTKGKNTVCVEIFAIITSSAGLLACIFLPKCYIILFRPEMNTKSHLLGNRIE
>Western_clawed_frog.locus213.1
------
MEWPNEKKNWCIERMKDFLSYNDVISVVFVSICLLLFVLTLLILIVFVIYRDTPIVRANNRSLSFLLLVSIKLSFLSVFLFLGRPVDITCMLRIITFGITFSIAVSSLLAKTIMVCVAFKATKPGSSWRKWLGVKLSNSVVLFCSSIQI
IICMTWLAISPPFQELDIHTSPGTIIIQCNEGSAIGFYSVIGYIGLLAAVSFVLAFLARSLPDSFNEAKYITFSMLLFCSVWITMIPAYLSTKGKNTVCVEIFAILTSSAGLLACIFLPKCYIILYRPEMNKKSHLLGNKV-
>Western_clawed_frog.locus227.1
------
MEWPNEKKNQCTEKMEDFLSYDDVISVFFSSISVLFFVITVLILRVLIIYRDTPIVRANNRSLSFLLLVSIKLSFLSVFLFLGRPVDITCMLRIITFGITFSIAVSSLLAKTIMVCVAFKATKPGSSWRKWLGVKVSNSVVLFCSSIQI
IICMTWLAISPPFQELDIHTSPGTIIIQCNEGSAIGFYSVIGYMGLLAAVSFVLAFLARSLPDSFNEAKYITFSMLLFCSVWITMIPAYLSTKGKNTVCVEIFAILTSSAGLLGCIFLPKCYIIIYRHEMNTKSHLLGKKA-
>Western_clawed_frog.locus212.1
------
MEWPNEKRNRCIAKMENFLSYNYGISVCFSSISVLLFVITLLILAVFISYRDTPIVRANNRSLSFLLLVSIKLSFLSVFLFLGRPVDITCMLRIITFGITFSIAVSSLLAKTIMVCVAFKATKPGSSWRKWLGVKLSNSVVLFCSSIQI
IICMTWLAISPPFQELDIHTSPGTIIIQCNEGSAIGFYSVIGYMGLLAAVSFVLAFLARSLPDSFNEAKYITFSMLLFCSVWITMIPAYLSTKGKNTVCVEIFAILTSSAGLLACIFLPKCYIILYRSDMNTKSHLLGNK--
>Western_clawed_frog.locus193.1
CSKCPDMEWPNRKRNECVAGWEDFLSY-
DVISVVLVSVAVFLYLMALLILGVFISYRDTPIVRANNRSLSFLLLVSIKLSFLSVFLFLGRPVDITCMLRIITFGITFSIAVSSLLAKTIMVCVAFKATKPGSSWRKWLGVKLSNSVVLFCSSIQIIICMTWLAISPPFQELDIHTSP
GTIIIQCNEGSAIGFYSVIGYMGLLAAVSFVLAFLARSLPDTFNEAKYITFSMLLFCSVWITMIPAYLSTKGKNTVCVEIFAILTSSAGLLASIFLPKCYIILYRPEMNTKSQLLGNKSL
>Western_clawed_frog.locus71.1
------
MEWPNEKRNQCVAKMEDFLSYNDVISAVFISVSICLYLVALLILGVFIKYRDTPIVRANNRSLSFLLLVSIKLSFLSVFLFLGRPVDITCMLRIITFGITFSIAVSSLLAKTIMVCVAFKATKPGSSWRKWLGVKLSNSVVLFCSSIQI
IICMTWLAISPPFQELDIHTSPGTIIIQCNEGSAIGFYSVIGYMGLLAAVSFVLAFLARSLPDSFNEAKYITFSMLLFCSVWITMIPAYLSTKGKNTVCVEIFAILTSSAGLLFCIFLPKFYLICFKPKLNIKSNLLGNKS-
>Western_clawed_frog.locus188.1
CIRCSDMEWPNEKRNRCMTKTEEFLSYNDLISVFISSISVLFFLITLLVLVVFISNHDTPIVRANNRSMSFLLLVSIKLSFLSVFLFLGRPVDITCMLRIITFGITFSIAVSSLLAKTIMVCVAFKATKPGSSWRKWLGVKLSNSVVLF
CSSIQIIICMTWLAISPPFQELDIHTSPGTIIIQCNEGSAIGFYSVIGYMGLLAAVSFVLAFLARSLPDSFNEAKYITFSMLLFCSVWITMIPAYLSTKGKNTVCVEIFAILTSSAGLLASIFLPKCYVILFKPEINTKSHVFANKS-
>Western_clawed_frog.locus79.1
------------
MRDQCIPKIEEFLSYDSGIPVFISAVTAILLLIAVIILGVFITYRDTPIMRANNRSLSFLLLVSIKLSFLSVFLFLGRPVDITCMLRIITFGITFSIAVSSLLAKTIMVCVAFKATKPGSSWRKWLGVKLSNSVVLFCSSIQIIICMTW
LAISPPFQELDIHTSPGTIIIQCNEGSAIGFYSVIGYMGLLAAVSFVLAFLARSLPDSFNEAKYITFSMLLFCSVWITMIPAYLSTKGKNTVCVEIFAILTSSAGLLACIFLPKCYIILFKTENNRKSNLLVIKS-
>Western_clawed_frog.locus80.1
CLRCPYNEWPNQRRNQCIPKIEEFLSYDSGIPVFISAVTAILLLIAVIILGVFITYRDTPIMRANNRSLSFLLLVSIKLSFLSVFLFLGRPVDITCMLRIITFGITFSIAVSSLLAKTIMVCVAFKATKPGSSWRKWLGVKLSNSVVLF
CSSIQIIICMTWLAISPPFQELDIHTSPGTIIIQCNEGSAIGFYSVIGYMGLLAAVSFVLAFLARSLPDSFNEAKYITFSMLLFCSVWITMIPAYLSTKGKNTVCVEIFAILTSSAGLLASIFLPKCYIILFKTENNRKSNLLVIKS-
>Western_clawed_frog.locus81.1
CIRCPYNEWPNQRRNQCIPKIEEFLSYDSVIPVFISAVTAILLLITVIILGVFIIYWDSPIVRANNRSLSFLLLVSIKLSFLSVFLFLGCPVDITCMLRIITFGITFSIAVSSLLAKTIMVCVAFKATKPGSSWRKWLGVKLSNSVVLF
CSSIQIIICMTWLAISPPFQELDIHTSPGTIIIQCNEGSAIGFYSVIGYMGLLAAVSFVLAFLARSLPDSFNEAKYITFSMLLFCSVWITMIPAYLSTKGKNTVCVEIFAILTSSAGLLASIFLPKCYIILFITENNRKSNLLVIKS-
>Western_clawed_frog.locus78.1
CIRCPYNEWPNQRRNQCIPKIEEFLSYDSVIPVFISAVSAILLLITVIILGVFIAHRETSIVRANNQSLSFLLLVSIKLSFLSVFLFLGRPVDITCMLRIITFGITFSIAVSSLLAKTIMVCVAFKATKPGSSWRKWLGVKLSNSVVLF
CSSIQIIICMTWLAISPPFQELDIHTSPGTIIIQCNEGSAIGFYSVIGYMGLLAAVSFVLAFLARSLPDSFNEAKYITFSMLLFCSVWITMIPAYLSTKGKNTVCVEIFAILTSSAGLLACIFLPKCYIILFITENNRKSNLLVIKS-
>Western_clawed_frog.locus77.1
CIRCPYNEWPNQRKNQCISKIEEFLSYNSVIPVFISAVSAILLLFTVIILGVFIAHWDTPIVRANNRSLSFLLLVSIKLSFLSVFLFLGRPVDITCMLRIITFGITFSIAVSSLLAKTIMVCVAFKATKPGSSWRKWLGVKLSNSVVLF
CSSIQIIICMTWLAISPPFQELDIHTSPGTIIIQCNEGSAIGFYSVIGYMGLLAAVSFVLAFLARSLPDSFNEAKYITFSMLLFCSVWITMIPAYLSTKGKNTVCVEIFAILTSSAGLLACIFLPKCYIILFLTEINKKSNLLVIKT-
>Western_clawed_frog.locus87.1
CARCPYNEWPNEKRNQCIARIEEFLSYDNVISVFFSVISALLLLMNMMISGVFISYRETPIVRANNRSLSFLLLVSIKLSFLSVFLFLGRPVDITCMLRIITFGITFSIAVSSLLAKTIMVCVAFKATKPGSSWRKWLGVKLSNSVVLF
CSSIQIIICMTWLAISPPFQELDIHTSPGTIIIQCNEGSAIGFYSVIGYMGLLAAVSFVLAFLARSLPDSFNEAKYITFSMLLFCSVWITMIPAYLSTKGKNTVCVEIFAILTSSAGLLASIFLPKCYTVLFRADINIKSHLLVMKT-
>Western_clawed_frog.locus88.1
CFTCPYNEWPNEKRNQCIARIEEFLSYDNVIPVFFSVISALLLLINVMISGVFISYRDTPIVRANNRSLSFLLLVSIKLSFLSVFLFLGRPVDITCMLRIITFGITFSIAVSSLLAKTIMVCVAFKATKPGSSWRKWLGVKLSNSVVLF
CSSIQIIICMTWLAISPPFQELDIHTSPGTIIIQCNEGSAIGFYSVIGYMGLLAAVSFVLAFLARSLPDSFNEAKYITFSMLLFCSVWITMIPAYLSTKGKNTVCVEIFAILTSSAGLLFCIFLPKCYTILFRTDINQKLNLLVIKT-
>Western_clawed_frog.locus200.1
CIRCPYNEWPNEKRNQCIAKIEEFLSYNNAIPVFFSVISALLLLKTVMILGVFIFYRDSPIVRANNRSLSFLLLVSIKLSFLSVFLFLGRPVDITCMLRIITFGITFSIAVSSLLAKTIMVCVAFKATKPGSSWRKWLGVKLSNSVVLF
CSSIQIIICMTWLAISPPFQELDIHTSPGTIIIQCNEGSAIGFYSVIGYMGLLAAVSFVLAFLARSLPDSFNEAKYITFSMLLFCSVWITMIPAYLSTKGKNTVCVEIFAIITSSAGLLACIFLPKCYIILLRPERNRKEYLIGRSNN
>Western_clawed_frog.locus199.1
CIRCPYNEWPNEKRNQCIAKIEEYLSYDNAIPVFFSVISALLLLKTVIILGVFIFYRDTPIVRANNRSLSFLLLVSIKLSFLSVFLFLGRPVDITCMLRIITFGITFSIAVSSLLAKTIMVCVAFKATKPGSSWRKWLGVKMSNSVVLF
CSSIQIIICMTWLAISPPFQELDIHTSPGTIIIQCNEGSAIGFYSVIGYMGLLAAVSFVLAFLARSLPDSFNEAKYITFSMLLFCSVWITMIPAYLSTKGKNTVCVEIFAIISSSCGLLFCIFLPKCYIILFKPEMNRKEYLTGRLNN
>Western_clawed_frog.locus198.1
CIRCPYNEWPNEKRNRCIAKIEEYLSYDNAIPVFFSVISALLLLKTVMISGVFIFYRDSPIVRANNRSLSFLLLVSIKLSFLSVFLFLGRPVDITCMLRIITFGITFSIAVSSLLAKTIMVCVAFKATKPGSSWRKWLGVKLSNSVVLF
CSSIQIIICMTWLAISPPFQELDIHTSPGTIIIQCNEGSAIGFYSVIGYMGLLAAVSFVLAFLARSLPDSFNEAKYITFSMLLFCSVWITMIPAYLSTKGKNTVCVEIFAILTSSAGLLACIFLPKCYIILFRTEMNNKSQLLVIKT-
>Western_clawed_frog.locus196.1
CFTCPYNEWSNENKNQCIAKIEVFLSYNDTIAVVFFAILAFLLFMTIMILAIFIYYLDTPIVRANNRSLSFLLLVSIKLSFLSVFLFLGRPVDITCMLRIITFGITFSIAVSSLLAKTIMVCVAFKATKPGSSWRKWLGVKLSNSVVLF
CSSIQIIICMTWLAISPPFQELDIHTSPGTIIIQCNEGSAIGFYSVIGYMGLLAAVSFVLAFLARSLPDSFNEAKYITFSMLLFCSVWITMIPAYLSTKGKNTVCVEIFAILTSSAGLLACIFLPKCYIILFRTEMNRKSNLLVIKTG
>Western_clawed_frog.locus197.1
CFTCPYNEWSNENKNQCIAKIEVFLSYNDTIAVVFFAILAFLLFITMMILAIFIYYLDTPIVRANNRGLSFLLLVSIKLSFLSVFLFLGRPVDITCMLRNVTFGITFSIAVSSLLAKTIMVCVAFKATKPGSSWRKWLGVKLSNSVVLF
CSSIQIIICMTWLAISPPFQELDIHTSPGTIIIQCNEGSAIGFYSVIGYMGLLAAVSFVLAFLARSLPDSFNEAKYITFSMLLFCSVWITMIPAYLSTKGKNTVCVEIFAILTSSAGLLACIFLPKCYNILFRTEMNRKSNLLVIKTG
>Western_clawed_frog.locus280.1
CIRCPYNEWPNEKRNQCIAKTEEFLSYTDVISVCFSVISVLFLLITVMILGVFIAHRDTPIVRANNRSLSFLLLVSIKLSFLSVFLFLGRPVDITCMLRIITFGITFSIAVSSLLAKTIMVCVAFKATKPGSSWRKWLGVKLSNSVVLF
CSSIQIIICMTWLAISPPFQELDIHTSPGTIIIQCNEGSAIGFYSVIGYMGLLAAVSFVLAFLARSLPDSFNEAKYITFSMLLFCSVWITMIPAYLSTKGKNTVCVEIFAILTSSAGLLACIFLPKCYIIMFRTEMNKKSHLLVMKS-
>Western_clawed_frog.locus75.1
CIRCPYNEWPNEKKDKCIARIEEFLSYTDVISVFFSSLSVLLFLITQLILQVFISYRDSPIVRANNRSLSFLLLVSIKLSFLSVFLFLGRPVDITCMLRIITFGITFSIAVSSLLAKTIMVCVAFKATKPGSSWRKWLGVKLSNSVVLF
CSSIQIIICMTWLAISPPFQELDIHTSPGTIIIQCNEGSAIGFYSVIGYMGLLAAVSFVLAFLARSLPDSFNEAKYITFSMLLFCSVWITMIPAYLSTKGKNTVCVEIFAILTSSAGLLACIFLPKCYIILFRTENNRKSHLLKNIKT
>Western_clawed_frog.locus76.1
CIRCPYNEWPNETKNKCIARIEEFLSYTDVISVFFSSVSVLFFLITQLILQVFISYRDTPIVRANNRSLSFLLLVSIKLSFLSVFLFLGRPVDITCMLRIITFGITFSIAVSSLLAKTIMVCVAFKATKPGSSWRKWLGVKLSNSVVLF
CSSIQIIICMTWLAISPPFQELDIHTSPGTIIIQCNEGSAIGFYSVIGYMGLLAAVSFVLAFLARSLPDSFNEAKYITFSMLLFCSVWITMIPAYLSTKGKNTVCVEIFAILTSSAGLLASIFLPKCYIIMLSPELNTKSHLLGNQA-
>Western_clawed_frog.locus72.1
------
MEWPNEKRNQCVAKAEEFLSYNDVISIFFSAVSIIFFLITQLLIVVFIIYRDSPIVRANNRSLSFLLLVSIKLSFLSVFLFLGRPVDITCMLRIITFGITFSIAVSSLLAKTIMVCVAFKATKPGSSWRKWLGVKLSNSVVLFCSSIQI
IICMTWLAISPPFQELDIHTSPGTIIIQCNEGSAIGFYSVIGYMGLLAAVSFVLAFLARSLPDSFNEAKYITFSMLLFCSVWITMIPAYLSTKGKNTVCVEIFAILTSSAGLLASIFLPKCYIILFRPENNRKSHLLIKT--
>Western_clawed_frog.locus73.1
------
MEWPNEKRNQCVAKAEEFLSYNDVISIFFSAVSIIFFLLTQLLIVVFIIYRDSPIVRANNRSLSFLLLVSIKLSFLSVFLFLGRPVDITCMLRIITFGITFSIAVSSLLAKTIMVCVAFKATKPGSSWRKWLGVKLSNSVVLFCSSIQI
IICMTWLAISPPFQELDIHTSPGTIIIQCNEGSAIGFYSVIGYMGLLAAVSFVLAFLARSLPDSFNEAKYITFSMLLFCSVWITMIPAYLSTKGKNTVCVEIFAILTSSAGLLASIFLPKCYIILFRPENNRKSHLLIKT--
>Western_clawed_frog.locus74.1
------
MEWPNEKRNQCVAKAEEFLSYNDVISIFFSAVSIIFFLITQLLIVVFIIYRDSPIVRANNRSLSFLLLVSIKLSFLSVFLFLGRPVDITCMLRIITFGITFSIAVSSLLAKTIMVCVAFKATKPGSSWRKWLGVKLSNSVVLFCSSIQI
IICMTWLAISPPFQELDIHTSPGTIIIQCNEGSAIGFYSVIGYMGLLAAVSFVLAFLARSLPDSFNEAKYITFSMLLFCSVWITMIPAYLSTKGKNTVCVEIFAILTSSAGLLACIFLPKCYIILFRPDNNRKSHLLIKT--
>Western_clawed_frog.locus54.1
------
MEWPNEKRNQCVSKTEEFLSYNDVISISFSAVSIIFFLITQLLIVVFIIHMDTPIVRANNRSLSFLLLVSIKLSFLSVFLFLGRPVDITCMLRIITFGITFSIAVSSLLAKTIMVCVAFKATKPGSSWRKWLGVKLSNSVVLFCSSIQI
IICMTWLAISPPFQELDIHTSPGTIIIQCNEGSAIGFYSVIGYMGLLAAVSFVLAFLARSLPDSFNEAKYITFSMLLFCSVWITMIPAYLSTKGKNTVCVEIFAILTSSAGLLASIFLPKCYIILFRPEINAKSYLFQNKSM
>Western_clawed_frog.locus56.1
------
MEWPNEKRNQCVAKTEEFLSYNDVISISFSAVSIIFFLITQLLIVVFIIHMDTPIVRANNRSLSFLLLVSIKLSFLSVFLFLGRPVDITCMLRIITFGITFSIAVSSLLAKTIMVCVAFKATKPGSSWRKWLGVKLSNSVVLFCSSIQI
IICMTWLAISPPFQELDIHTSPGTIIIQCNEGSAIGFYSVIGYMGLLAAVSFVLAFLARSLPDSFNEAKYITFSMLLFCSVWITMIPAYLSTKGKNTVCVEIFAILTSSTGLLACIFLPKCYIILFRPEINAKSYLFQNKSM
>Western_clawed_frog.locus243.1
CIRCPALEWPNDKRSQCIAKTEEFLSFDGAISASLLSITVLFSLITLLMLRIFILYRDSPIVRANNRSLSFLLLVSIKLSFLSVFLFLGRPVDITCMLRIITFGITFSIAVSSLLAKTIMVCVAFKATKPGSSWRKWLGVKLSNSVVLF
CSSIQITICMTWLAISPPFQELDIHTSPGTIIIQCNEGSAIGFYSVIGYMGLLAAVSFGLAFLARSLPDSFNEAKYITFSMLLFCSVWITMIPAYLSTKGKNTVCVEIFAILTSSAGLLACIFLPKCYIILLKPEMNTKQYLRGTNKE
>Western_clawed_frog.locus241.1
```

```
CIRCPDLEWPNDKKNQCIAKTEEFLSFDGAISASLLSITVLFSLITLLMLRIFILNRDSPIVRANNRSLSFLLLVSIKLSFLSVFLFLGRPVDITCMLRIITFGITFSIAVSSLLAKTIMVCVAFKATKPGSSWRKWLGVKLSNSVVLF
CSSIQIIICMTWLAISPPFQELDIHTSPGTIIIQCNEGSAIGFYSVIGYMGLLAAVSFVLAFLARSLPDSFNEAKYITFSMLLFCSVWITMIPAYLSTKGKNTVCVEIFAILTSSAGLLACIFLPKCYIIMFRPEMNQKSCLLGSKT-
>Western_clawed_frog.locus256.1
CIRCPDLEWPNNKRNQCIVKTEEFLSFDGTISVSLLSITVLFSFITLLMLRIFILYRDTPIVRANNRSLSFLLLVSIKLSFLSVFLFLGRPVDITCMLRIITFGITFSIAVSSLLAKTIMVCVAFKATKPGSSWRKWLGVKLSNSVVLF
CSSIQIIICMTWLAISPPFQELDIHTSPGTIIIQCNEGSAIGFYSVIGYMGLLAAVSFVLAFLARSLPDSFNEAKYITFSMLLFCSVWITMIPAYLSTKGKNTVCVEIFAILTSSAGLLACIFLPKCYIIMFRPEMNQKSCLLGSKT-
>Western_clawed_frog.locus255.1
CIRCPDLEWPNDKRNQCIAKTEEFLSFDGTISVSLLSITVLFSLITLLMLRIFILYRDSPIVRANNRSLSFLLLVSIKLSFLSVFLFLGRPVDITCMLRIITFGITFSIAVSSLLAKTIMVCVAFKATKPGSSWRKWLGVKLSNSVVLF
CSSIQIIICMTWLAISPPFQELDIHTSPGTIIIQCNEGSAIGFYSVIGYMGLLAAVSFVLAFLARSLPDSFNEAKYITFSMLLFCSVWITMIPAYLSTKGKNTVCVEIFAILTSSAGLLACIFLPKCYIIMFRPEMNQKSCLLGGKT-
>Western_clawed_frog.locus252.1
CIRCPDLEWPNDQRNQCIAKTVEFLSFDGTISVSLLSTTVLFNLTTLLMLRIFISYRDTPIVRANNRSLSFLLLVSIKLSFLSVFLFLGRPVDITCMLRIITFGITFSIAVSSLLAKTIMVCVAFKATKPGSSWRKWLGVKLSNSVVLF
CSSIQIIICMTWLAISPPFQELDIHTSPGTIIIQCNEGSAIGFYSVIGYMGLLAAVSFVLAFLARSLPDSFNEAKYITFSMLLFCSVWITMIPAYLSTKGKNTVCVEIFAILTSSAGLLACIFLPKCYIIMFRPEMNQKSCLLGSKT-
>Western_clawed_frog.locus244.1
CFRCPDLEWPNEKRNQCIAKTEEFLSFDGTISTSLLSITVLFSLTTLLMLRIFISYRDSPIVRANNRSLSFLLLVSIKLSFLSVFLFLGRPVDITCMLRIITFGITFSIAVSSLLAKTIMVCVAFKATKPGSSWRKWLGVKLSNSVVLF
CSSIQIIICVTWLAISPPFQELDIHTSPGTIIIQCNEGSAIGFYSVIGYMGLLAAVSFVLAFLARSLPDSFNEAKYITFSMLLFCSVWITMIPAYLSTKGKNTVCVEIFAILTSSAGLLACIFLPKCYIIMFRPEINQKSCLLGNKAF
>Western_clawed_frog.locus239.1
CIRCPALEWPNDKRDQCIAKTEEFLSFDGTISVSLLSITVLFSLITLLMLRIFILYRDSPIVRANNRSLSFLLLVSIKLSFLSVFLFLGRPVDITCMLRIITFGITFSIAVSSLLAKTIMVCVAFKATKPGSSWRKWLGVKLSNSVVLF
CSSIQIIICMTWLAISPPFQELDIHTSPGTIIIQCNEGSAIGFYSVIGYMGLLAAVSFVLAFLARSLPDSFNEAKYITFSMLLFCSVWITMIPAYLSTKGKNTVCVEIFAILTSSAGLLACIFLPKCYIIMFRPEMNQKSCLLGNKT-
>Western_clawed_frog.locus254.1
CIRCPALEWPNDKRSQCIAKTEEFLSFDGTISVSLLSITFLFSLITLLMLRMFISYRDSPIVRANNRSLSFLLLVSIKLSFLSVFLFLGRPVDITCMLRIITFGITFSIAVSSLLAKTIMVCVAFKATKPGSSWRKWLGVKLPNSVVLF
CSSIQIIICMTWLAISPPFQELDIHTSPGTIIIQCNEGSAIGFYSVIGYMGLLAAVSFVLAFLARSLPDSFNEAKYITFSMLLFCSVWITMIPAYLSTKGKNTVCVEIFAILTSSAGLLACIFLPKCYIIMFRPEMNQKSSLLGSKT-
>Western_clawed_frog.locus251.1
CIRCPALEWPNDKRNQCIAKTEEFLSFDGTISVSLLSITVLFSLITLLMLRMFISYRDSPIVRANNRSLSFLLLVSIKLSFLSVFLFLGRPVDITCMLRIITFGITFSIAVSSLLAKTIMVCVAFKATKPGSSWRKWLGVKLSNSVVLF
CSSIQIIICMTWLAISPPFQELDIHTSPGTIIIQCNEGSAIGFYSVIGYMGLLAAVSFVLAFLARSLPDSFNEAKYITFSMLLFCSVWITMIPAYLSTKGKNTVCVEIFAILTSSAGLLACIFLPKCYIIMFRPEMNHKSSLLGSKT-
>Western_clawed_frog.locus247.1
CIRCPALEWPNDKRNQCIAKIEEFLSFNGTISVSLLSITVLFSLITLLMLRIFISYRDSPIVRANNRSLSFLLLVSIKLSFLSVFLFLGRPVDITCMLRIITFGITFSIAVSSLLAKTIMVCVAFKATKPGSSWRKWLGVKLSNSVVLF
CSSIQIIICMTWLAISPPFQELDIHTSPGTIIIQCNEGSAIGFYSVIGYMGLLAAVSFVLAFLARSLPDSFNEAKYITFSMLLFCSVWITMIPAYLSTKGKNTVCVEIFAIFTSSAGLLACIFLPKFYIIMFRPEMNCKSGILGGKN-
>Western_clawed_frog.locus246.1
CIRCPALEWPNDKKNQCIAKTEEFLSFDGTISVSLLSITVLFSIITLLMLRIFISYRDSPIVRANNRSLSFLLLVSIKLSFLSVFLFLGRPVDITCMLRIITFGITFSIAVSSLLAKTIMVCVAFKATKPGSSWRKWLGVKLSNSVVLF
CSSIQIIICMTWLAISPPFQELDIHTSPGTIIIQCNEGSAIGFYSVIGYMGLLAAVSFVLAFLARSLPDSFNEAKYITFSMLLFCSVWITMIPAYLSTKGKNTVCVEIFAILTSSAGLLACIFLPKCYIIMFRPEMNQKSCLLGSKT-
>Western_clawed_frog.locus240.1
--------------------
MVEFLSFDGAISASLLSTTVLFFLITLLILGIFISYRDSPIVRANNRSLSFLLLVSIKLSFLSVFLFLGRPVDITCMLRIITFGITFSIAVSSLLAKTIMVCVAFKATKPGSAWRKWLGVKLSNSVVLFCSSIQIIICMTWLAISPPFQ
ELDIHTSPGTIIIQCNEGSAIGFYSVIGYMGLLAAVSFVLAFLARSLPDSFNEAKYITFSMLLFCSVWITMIPAYLSTKGKNTVCVEIFAILTSSAGLLACIFLPKCYIIMFRPEMNQKSCLLGSKT-
>Western_clawed_frog.locus245.1
CIRCPALEWPNDKRNQCIAKTEEFLSFDGTISVSLLSITVLFFLITLLILGIFISYRDTPIVRANNRSLSFLLLVSIKLSFLSVFLFLGRPVDITCMLRIITFGITFSIAVSSLLAKTIMVCVAFKATKPGSSWRKWLGVKLSNSVVLF
CSSIQIIICMTWLAISPPFQELDIHTSPGTIIIQCNEGSAIGFYSVIGYMGLLAAVSFVLAFLARSLPDSFNEAKYITFSMLLFCSVWITMIPAYLSTKGKNTVCVEIFAILTSSAGLLACIFLPKCYIIMFRPEMNQKACLLGSKT-
>Western_clawed_frog.locus236.1
CIRCPALEWPNHKRNQCIAKTEEFLSFDGTISVSLLSITVLFSLITLLILGIFVIYRDSPIVRANNRSLSFLLLVSIKLSFLSVFLFLGRPVDITCMLRIITFGITFSIAVSSLLAKTIMVCVAFKATKPGSSWRKWLGVKLSNSVVLF
CSSIQIIICMTWLAISPPFQELDIHTSPGTIIIQCNEGSAIGFYSVIGYMGLLAAVSFVLAFLARSLPDSFNEAKYITFSMLLFCSVWITMIPAYLSTKGKNTVCVEIFAILTSSAGLLACIFLPKCYIIMFRPEMNHKSCLLGSKT-
>Western_clawed_frog.locus250.1
CIRCPDLEWPNDKRNQCIVKTEEFLLFDGTISVSLLSITVLFSLITLFILGVFITYRDSPIVRANNRGLSFLLLVSIKLSFLSVFLFLGRPVDITCMLRIITFGITFSIAVSSLLAKTIMVCVAFKATKPGSSWRKWLGVKLSNSVVLF
CSSIQIIICMTWLAISPPFQELDIHTSPGTIIIQCNEGSAIGFYSVIGYMGLLAAVSFVLAFLARSLPDSFNEAKYITFSMLLFCSVWITMIPAYLSTKGKNTVCVEIFAILTSSAGLLACIFLPKCYIIMFRPEMNQKSCLLGSKT-
>Western_clawed_frog.locus263.1
CLRCPDMEWPNENKNHCLSKTEEFLSFNDTISVFLLSMPILFFFITLLILGIFIIYRDTPIVRANNRSLSFLLLVSIKLSFLSVFLFLGRPVDITCMLRIITFGITFSIAVSSLLAKTIMVCVAFKATKPGSSWRKWLGVKLSNSVVLF
CSSIQIIICMTWLAISPPFQELDIHTSPGTIIIQCNEGSAIGFYSVIGYMGLLAAVSFVLAFLARSLPDSFNEAKYITFSMLLFCSVWITMIPAYLSTKGKNTVCVEIFAILTSSAGLLACIFLPKCYIILFRPEMNHKSYLLGSKT-
>Western_clawed_frog.locus258.1
CLRCPDMEWPNENKNQCLAKTEEFLSFNDTISVFLLSMPILFFFITLLILGIFIINQDTPIVRANNRSLSFLLLVSIKLSFLSVFLFLGRPVDITCMLRIITFGITFSIAVSSLLAKTIMVCVAFKATKPGSSWRKWLGVKLSNSVVLF
CSSIQIIICMTWLAISPPFQELDIHTSPGTIIIQCNEGSAIGFYSVIGYMGLLAAVSFVLAFLARSLPDSFNEAKYITFSMLLFCSVWITMIPAYLSTKGKNTVCVEIFAILTSSAGLLACIFLPKCYIIMFRPEMNQKSRLLGSKT-
>Western_clawed_frog.locus257.1
------
MEWPNENKNQCLAKPEEFLSLNDTISVFLLSMPILFFFITLLILGIFIIYRDTPIVRANNRSLSFLLLVSIKLSFLSVFLFLGRPVDITCMLRIITFGITFSIAVSFLLAKTIMVCVAFKATKPGSSWRKWLGVKLSNSVVLFCSSIQI
IICMTWLAISPPFQELDIHTSPGTIIIQCNEGSAIGFYSVIGYMGLLAAVSFVLAFLARSLPDSFNEAKYITFSMLLFCSVWITMIPAYLSTKGKNTVCVEIFAILTSSAGLLACIFLPKCYIIMFRPEMNHKSSLLGSKT-
>Western_clawed_frog.locus262.1
------
MEWPNENKNQCLAKPEEFLSFNDTISLFLLSMPILLFFITLLMLGIFIIYRDTPIVRANNRSLSFLLLVSIKLSFLSVFLFLGRPVDITCMLRIITFGITFSIAVSSLLAKTIMVCVAFKATKPGSSWRKWLGVKLSNSVVLFCSSIQI
IICMTWLAISPPFQELDIHTSPGTIIIQCNEGSAIGFYSVIGYMGLLAAVSFVLAFLARSLPDSFNEAKYITFSMLLFCSVWITMIPAYLSTKGKNTVCVEIFAILTSSAGLLACIFLPKCYIIMFRPEMNHKSCLLGIKT-
>Western_clawed_frog.locus260.1
------
MEWPNENKNRCLAKTEEFLSFNDTISVFLLSMPILFFFITLLILGIFIINRDSPIVRANNRSLSFLLLVSIKLSFLSVFLFLGRPVDITCMLRIITFGITFSIAVSSLLAKTIMVCVAFKATKPGSSWRKWLGVKLSNSVVLFCSSIQI
IICMTWLAISPPFQELDIHTSPGTIIIQCNEGSAIGFYSVIGYMGLLAAVSFVLAFLARSLPDSFNEAKYITFSMLLFCSVWITMIPAYLSTKGKNTVCVEIFAILTSSAGLLACIFLPKCYIIMFRPEMNHKSCLLGIKT-
>Western_clawed_frog.locus261.1
------
MEWPNENKNQCLSKTEEFLSFNDTISVFLLSMPILFFFITLLILVIFVIYLDSPIVRANNRSLSFLLLVSIKLSFLSVFLFLGRPVDITCMLRIITFGITFSIAVSSLLAKTIMVCVAFKATKPGSSWRKWLGVKMSNSVVLFCSSIQI
IICMTWLAISPPFQELDIHTSPGTIIIQCNEGSAIGFYSVIGYMGLLAAVSFVLAFLARSLPDSFNEAKYITFSMLLFCSVWITMIPAYLSTKGKNTVCVEIFAILTSSAGLLACIFLPKCYIIMFRPEMNQKSCLLGSKT-
>Western_clawed_frog.locus259.1
------
MEWPNENKNQCLAKTEEFLSFNDTISVFLLSMPILFFFITLLILGIFVIYRDSPIVRANNRSLSFLLLVSIKLSFLSVFLFLGRPVDITCMLRIITFGITFSIAVSSLLAKTIMVCVAFKATKPGSSWRKWLGVKLSNSVVLFCSSIQI
IICMTWLAISPPFQELDIHTSPGTIIIQCNEGSAIGFYSVIGYMGLLAAVSFVLAFLARSLPDSFNEAKYITFSMLLFCSVWITMIPAYLSTKGKNTVCVEIFAILTSSAGLLACIFLPKCYIIMFRPEMNHKSCLLGSKT-
>Western_clawed_frog.locus134.1
CLKCPDLEWPNEQRNQCIARTEEFLSFDCTIAAFLSPVTILFYIITLLILGIFITFRDSPIVRANNRSLSFLLLVSIKLSFLSVFLFLGRPVDITCMLRIITFGITFSIAVSSLLAKTIMVCVAFKATKPGSSWRKWLGVKLSNSVVLF
CSSIQIIICMTWLAISPPFQELDIHTSPGTIIIQCNEGSAIGFYSVIGYMGLLAAVSFVLAFLARSLPDSFNEAKYITFSMLLFCSVWITMIPAYLSTKGKNTVCVEIFAILTSSAGLLACIFLPKCYTVFFRSEINTKSHLFRNNYH
>Western_clawed_frog.locus170.1
CLKCPDLEWPNEQRNQCIARTEEFLSFDCTIAEFLSSVSILFYIITLLILGIFITFRGTPIVRANNRSLSFLLLVSIKLSFLSVFLFLGRPVDITCMLRIITFGITFSIAVSSLLAKTIMVCVAFKATKPGSSWRKWLGVKLSNSVVLF
CSSIQIIICMTWLAISPPFQELDIHTSPGTIIIQCNEGSAIGFYSVIGYMGLLAAVSFVLAFLARSLPDSFNEAKYITFSMLLFCSVWITMIPAYLSTKGKNTVCVEIFAILTSSAGLLASIFLPKCYIIMLGPEMNTKSHLFSNNHH
>Western_clawed_frog.locus146.1
CLKCPDVEWPNEQRNQCIAKTEEFLSFDCTIAAFLSSVTILFYIITLLILGIFITFRDSPIVRANNRSLSFLLLVSIKLSFLSVFLFLGRPVDITCMLRIITFGITFSIAVSSLLAKTIMVCVAFKATKPGSSWRKWLGVKLSNSVVLF
CSSIQIIICMTWLAISPPFQEVDIHTSPGTIIIQCNEGSAIGFYSVIGYMGLLAAVSFVLAFLARSLPDSFNEAKYITFSMLLFCSVWITMIPAYLSTKGKNTVCVEIFAILTSSAGLLACIFLPKCYVIMFEPETNQKLCLLRSKTL
>Western_clawed_frog.locus145.1
CLKCPDVEWPNEKSNQCIARTEEFLSFDCTIAAFLSSVTIVFYIITLLILGIFITFRDSPIVRANNRSLSFLLLVSIKLSFLSVFLFLGRPVDITCMLRIITFGITFSIAVSSLLAKTIMVCVAFKATKPGSSWRKWLGVKLSNSVVLF
CSSIQIIICMTWLAISPPFQELDIHTSPGTIIIQCNEGSAIGFYSVIGYMGLLAAVSFVLAFLARSLPDSFNEAKYITFSMLLFCSVWITMIPAYLSTKGKNTVCVEIFAILTSSAGLLASIFLPKCYTIILRPEMNQKSCLLGHIT-
>Western_clawed_frog.locus144.1
------
MEWPNEKNNECVTKTEEYLSFNDIISAFLSSLSVLLFLLSLQILGIFITFRDTPIVRANNRSLSFLLLVSIKLSFLSVFLFLGRPVDITCMLRIITFGITFSIAVSSLLAKTIMVCVAFKATKPGSSWRKWLGVKLSNSVVLFCSSIQI
IICMTWLAISPPFQELDIHTSPGTIIIQCNEGSAIGFYSVIGYMGLLAAVSFVLAFLARSLPDSFNEAKYITFSMLLFCSVWITMIPAYLSTKGKNTVCVEIFAILTSSAGLLASIFLPKCYIIILRPEMNQKSCLLGHIT-
>Western_clawed_frog.locus169.1
CYRCPDLEWPNEKRNQCIARTEDCLSYSDGISVFLTSISVLFFLITLLIFGVFVLYRDSPIVRANNRSLSFLLLVSIKLSFLSVFLFLGRPVDITCMLRIITFGITFSIAVSSLLAKTIMVCVAFKATKPGSSWRKWLGVKLSNSVVLF
CSSIQIIICMTWLAISPPFQELDIHTSPGTIIIQCNEGSAIGFYSVIGYMGLLAAVSFVLAFLARSLPDSFNEAKYITFSMLLFCSVWITMIPAYLSTKGKNTVCVEIFAILTSSAGLLASIFLPKCYIIMCKPERNQKSCLLQSKT-
>Western_clawed_frog.locus248.1
CFRCPDLEWPNEKRNQCIAKTEEFLSHTDTISIFLSSIPILFFLISLLILGVFIAHRETPIVRANNQSLSFLLLVSIKLSFLSVFLFLGRPVDITCMLRIITFGITFSIAVSSLLAKTIMVCVAFKATKPGSSWRKWLGVKLSNSVVLF
CSSIQIIICMTWLAISPPFQELDIHTSPGTIIIQCNEGSAIGFYSVIGYMGLLAAVSFVLAFLARSLPDSFNEAKYITFSMLLFCSVWITMIPAYLSTKGKNTVCVEIFAILTSSAGLLACIFLPKCYIIMFRPEMNQKSHLLGNKAF
>Western_clawed_frog.locus238.1
CFRCPDTEWPNANKNVCIVKGAEFLSYNDVISVFFSSISVLLFVITLLILGIFIIYRDTPIVRANNRSLSFLLLVSIKLSFLSVFLFLGRPVDITCMLRIITFGITFSIAVSSLLAKTIMVCVAFKATKPGSSWRKWLGVKLSNSVVLF
CSSIQIIICMTWLAISPPFQELDIHTSPGTIIIQCNEGSAIGFYSVIGYMGLLAAVSFVLAFLARSLPDSFNEAKYITFSMLLFCSVWITMIPAYLSTKGKNTVCVEIFAILTSSAGLQGCIFLPKCYTILFRPEINSKSFLRVNKFC
>Western_clawed_frog.locus109.1
CIRCPDTEWPNANKSACVVKGAEFLSYNDVISVFFSSISVLLFVITLLILGIFIIYRDTPIVRANNRSLSFLLLVSIKLSFLSVFLFLGRPVDITCMLRIVTFGITFSIAVSSLLAKTIMVCVAFKATKPGISWRTWVGVKLSNSVVLF
CSSIQIIICMTWLAISPPFQELDIHTSHGTIIIQCNEGSAIGFYSVIGYMGLLAAVSFVLAFLARSLPDSFNEAKYITFSMLLFCSVWITMIPAYLSTKGKNTVCVEIFAILTSSAGLLACIFLPKCYTILFRPEINTKSILRGNKFC
>Western_clawed_frog.locus110.1
------
MEAPNEKRNQCIAKSVEFLSYNDVISEFLSSISVLLVVVTLLILGVFIQYRDSPIVRANNRSLSFLLLVSIKLSFLSVFLFLGRPVDITCMLRIITFGITFSIAVSSLLAKTIMVCVAFKATKPGSSWRKWLGVKLSNSVVLFCSSIQI
IICMTWLAISPPFQELDIHTSPGTIIIQCNEGSAIGFYSVIGYMGLLAAVSFVLAFLARSLPDSFNEAKYITFSMLLFCSVWITMIPAYLSTKGKNTVCVEIFAILTSSVGLLACIFLPKCYTILYRPEINTKSILLGNKSL
>Western_clawed_frog.locus86.1
------------------------------
MISVFFSFMSVFFFIVTLMISGVFITYRDSPIVRANNRSLSFLLLVSIKLSFLSVFLFLGRPVDITCMLRIITFGITFSIAVSSLLAKTIMVCVAFKATKPGSSWRKWLGVKLSNSVVLFCSSIQIIICMTWLAISPPFQELDIHTSPG
TIIIQCNEGSAIGFYSVIGYMGLLAAVSFVLAFLARSLPDSFNEAKYITFSMLLFCSVWITMIPAYLSTKGKNTVCVEIFAILTSSAGLLASIFLPKCYTILFMSAMNKKFNLLRNKSL
>Western_clawed_frog.locus225.1
CFRCPSLEWPNEKRNQCIAKVEEFLSYNDVISLFVSTVSILFFLTTLLILGVFISYQDSPIVRANNRSLSFLLLISIKLSFLSVFLFLGRPVDITCMLRIITFGITFSIAVSSLLAKTIMVCVAFKATKPGSSWRKWLGVKLSNSVVLF
CSSIQIIICMTWLAISPPFQELDIHTSPGTIIIQCNEGSAIGFYSVIGYMGLLAAVSFVLAFLARSLPDSFNEAKYITFSMLLFCSVWITMIPAYLSTKGKNTVCVEIFAILTSSAGLLACIFLPKCYTILLRPERNTTPQLNVNKSY
>Western_clawed_frog.locus127.1
------
MEWPNEKRNQCIARTEQFLSYDNVISDAFISVSILFSLITVIVLGIFVLYRDSPIVRANNRSLSFLLLVSIKLSFLSVFLFLGRPVDITCMLRIITFGITFSIAVSSLLAKTIMVCVAFKATKPGSSWRKWLGVKLSNSVVLFCSSIQI
IICMTWLAISPPFQELDIHTSPGTIIIQCNEGSAIGFYSVIGYMGLLAAVSFVLAFLARSLPDSFNEAKYITFSMLLFCSVWITMIPAYLSTKGKNTVCVEIFAILTSSAGLLFCIFLPKCYIILFRAETNVKSHLFKK---
>Western_clawed_frog.locus157.1
------
MEWPNEKRNQCIAKTEQFLSYNDVISDAFISVSILFSLITVIVLGIFVLYRDSPIVRANNRSLSFLLLVSIKLSFLSVFLFLGRPVDITCMLRIITFGITFSIAVSSLLAKTIMVCVAFKATKPGSSWRKWLGVKLSNSVVLFCSSIQI
IICMTWLAISPPFQELDIHTSPGTIIIQCNEGSAIGFYSVIGYMGLLAAVSFVLAFLARSLPDSFNEAKYITFSMLLFCSVWITMIPAYLSTKGKNTVCVEIFAILTSSAGLLASIFLPKCYMILFRPEMNTKSNLLGNKFL
>Western_clawed_frog.locus187.1
---------------------------------
MFFSAFSVLSFFITLLILRVFISYRDTPIVRANNRSLSFLLLVSIKLSFLSVFLFLGRPVDITCMLRIITFGITFSIAVSSLLAKTIMVCVAFKATKPGSSWRKWLGVKLSNSVVLFCSSIQIIICMTWLAISPPFQELDIHTSPGTII
```

```
IQCNEGSAIGFYSVIGYMGLLAAVSFVLAFLARSLPDSFNEAKYITFSMLLFCSVWITMIPAYLSTKGKNTVCVEIFAILTSSAGLLACIFLPKCYIILFKPAMNTKKNLL-----
>Western_clawed_frog.locus185.1
--------------------------------
MFFSAFSVLSFFITLLILGVFISYRDSPIVRANNRSLSFLLLVSIKLSFLSVFLFLGRPVDITCMLRIITFGITFSIAVSSLLAKTIMVCVAFKATKPGSSWRKWLGVKLSNSVVLFCSSIQIIICMTWLAISPPFQELDIHTSPGTII
IQCNEGSAIGFYSVIGYMGLLAAVSFVLAFLARSLPDSFNEAKYITFSMLLFCSVWITMIPAYLSTKGKNTVCVEIFAILTSSAGLLACIFLPKCYIIMFKPAMNTKKNVL-----
>Western_clawed_frog.locus123.1
CFRCKDTEWPNKKRTQCIAKAEAFLSYDDVISMFFSAFSVLSFFITLLILGVFISYRDSPIVRANNRSLSFLLLVSIKLSFLSVFLFLGRPVDITCMLRIITFGITFSIAVSSLLAKTIMVCVAFKATKPGSSWRKWLGVKLSNSVVLF
CSSIQIIICMTWLAISPPFQELDIHTSPGTIIIQCNEGSAIGFYSVIGYMGLLAAVSFVLAFLARSLPDSFNEAKYITFSMLLFCSVWITMIPAYLSTKGKNTVCVEIFAILTSSAGLLACIFLPKCYIIMFKPEINTKKNLF-----
>Western_clawed_frog.locus124.1
--------------------------------
MFFSAVSVLSFFITLLILGVFISYRDSPIVRANNRSLSFLLLVSIKLSFLSVFLFLGRPVDITCMLRIITFGITFSIAVSSLLAKTIMVCVAFKATKPGSSWRKWLGVKLSNSVVLFCSSIQIIICMTWLAISPPFQELDIHTSPGTII
IQCNEGSAIGFYSVIGYMGLLAAVSFVLAFLARSLPDSFNEAKYITFSMLLFCSVWITMIPAYLSTKGKNTVCVEIFAILTSSAGLLACIFLPKCYIIMFKPEMNTKKNLF-----
>Western_clawed_frog.locus128.1
--------------------------------
MFFSAVSVLSFFISLLILGVFISYRDSPIVRANNRSLSFLLLVSIKLSFLSVFLFLGRPVDITCMLRIITFGITFSIAVSSLLAKTIMVCVAFKATKPGSSWRKWLGVKLSNSVVLFCSSIQIIICMTWLAISPPFQELDIHTSPGTII
IQCNEGSAIGFYSVIGYMGLLAAVSFVLAFLARSLPDSFNEAKYITFSMLLFCSVWITMIPAYLSTKGKNTVCVEIFAILTSSAGLLFCIFLPKCYVIMFRPEINSKSKLLGNRSF
>Western_clawed_frog.locus195.1
--------------------------------
MLFSAVSILFFFITLLILVVFISYRDTPIVRANNRILSFLLLVSIKLSFLSVFLFLGRPVDITCMLRIITFGITFSIAVSSLLAKTIMVCVAFKATKPGSSWRKWLGVKLSNSVVLFCSSIQIIICMTWLAISPPFQELDIHTSPRTII
IQCNEGSAIGFYSVIGYMGLLAAVSFVLAFLARSLPDSFNEAKYITFSMLLFCSVWITMIPAYLSTKGKNTVCVEIFAILTSSAGLLACIFLPKCYIILFNSEMNTKKYLLGNKDV
>Western_clawed_frog.locus194.1
CFQCKETEWPNGKRTRCIAKVKEFLSYDDVISMVFSAVSVLFFFITLLILGVFISYRDSPIVRANNRSLSFLLLVSIKLSFLSVFLFLGRPVDITCMLRIITFGITFSIAVSSLLAKTIMVCVAFKATKPGSSWRKWLGVKLSNSVVLF
CSSIQIIICMTWLAISPPFQELDIHTSPGTIIIQCNEGSAIGFYSVIGYMGLLAAVSFVLAFLARSLPDSFNEAKYITFSMLLFCSVWITMIPAYLSTKGKNTVCVEIFAILTSSAGLLACIFLPKCYTIFFKPEMNTKKYLL-----
>Western_clawed_frog.locus82.1
------
MEWPNENRTQCIAKVEEFLSYNDIISIIFSAFSILFFLITMLILILFITYRDSPIVRANNRSLSFLLLVSIKLSFLSVFLFLGRPVDITCMLRIITFGITFSIAVSSLLAKTIMVCVAFKATKPGSSWRKWLGVKLSNSVVLFCSSIQI
IICMTWLAISPPFQELDIHTSPGTIIIQCNEGSAIGFYSVIGYMGLLAAVSFVLAFLARSLPDSFNEAKYITFSMLLFCSVWITMIPAYLSTKGKNTVCVEIFAILTSSAGLLACIFVPKCYVILIRPDLNTKTQLLGNKM-
>Western_clawed_frog.locus83.1
------
MEWPNENRTQCIAKVEEFLSYNDIISITFSAISILFFLITMLILILFITYWDSPIVRANNRSLSFLLLVSIKLSFLSVFLFLGRPVDITCMLRIITFGITFSIAVSSLLAKTIMVCVAFKATKPGSSWRKWLGVKLSNSVVLFCSSIQI
IICMTWLAISPPFQELDIHTSPGTIIIQCNEGSAIGFYSVIGYMGLLAAVSFVLAFLARSLPDSFNEAKYITFSMLLFCSVWITMIPAYLSTKGKNTVCVEIFAILTSSAGLLACIFVPKCYVILIRPDLNTKTQLLGNKM-
>Western_clawed_frog.locus85.1
------MEWPNENRTQCIAKLEEFLSY-
DIISIIFSAISILFFLITMLILILFIKNWGTPIVRANNRSLSFLLLVSIKLSFLSVFLFLGRPVDITCMLRIITFGITFSIAVSSLLAKTIMVCVAFKATKPGSSWRKWLGVKLSNSVVLFCSSIQIIICMTWLAISPPFQELDIHTSP
GTIIIQCNEGSAIGFYSVIGYMGLLAAVSFVLAFLARSLPDSFNEAKYITFSMLLFCSVWITMIPAYLSTKGKNTVCVEIFAILTSSAGLLACIFLPKCYVILIKSEMNTKTHLLGNKM-
>Western_clawed_frog.locus84.1
CVHCPDMEWPNENRTQCIAKVEEFLSYNDIISIIFSAISILFFLITMLILILFIKNWDSPIVRANNRSLSFLLLVSIKLSFLSVFLFLGRPVDITCMLRIITFGITFSIAVSSLLAKTIMVCVAFKATKPGSSWRKWLGVKLSNSVVLF
CSSIQIIICMTWLAISPPFQELDIHTSPGTIIIQCNEGSAIGFYSVIGYMGLLAAVSFVLAFLARSLPDSFNEAKYITFSMLLFCSVWITMIPAYLSTKGKNTVCVEIFAILTSSAGLLACIFLPKCYIILIKSEMNTKTHLLGNKM-
>Western_clawed_frog.locus135.1
CIQCPEMEWANNKRTKCIAKTDEFLSYNDIIAVIFSSIAVLFFLITMLLLRFFITFRDSPIVRANNRSLSFLLLVSIKLSFLSVFLFLGRPVDITCMLRIITFGITFSIAVSSLLAKTIMVCVAFKATKPGSSWRKWLGVKLSNSVVLF
CSSIQIIICMTWLAISPPFQELDIHTSPGTIIIQCNEGSAIGFYSVIGYMGLLAAVSFVLAFLARSLPDSFNEAKYITFSMLLFCSVWITMIPAYLSTKGKNTVCVEIFAILTSSAGLLACIFLPKCYVILFRPEINTKSHLLRIQF-
>Western_clawed_frog.locus161.1
CIQCPEMEWADNKRTKCIAKTDEFLSYNDIIAVIFSSITVLFFLITVLILRVFITYWDSPIVRANNRSLSFLLLVSIKLSFLSVFLFLGRPVDITCMLRIITFGITFSIAVSSLLAKTIMVCVAFKATKPGSSWRKWLGVKLSNSVVLF
CSSIQIIICMTWLAISPPFQELDIHTSPGTIIIQCNEGSAIGFYSVIGYMGLLAAVSFVLAFLARSLPDSFNEAKYITFSMLLFCSVWITMIPAYLSTKGKNTVCVEIFAILTSSASLLASIFLPKCYIILFRPEINTKSNLLGNQKV
>Western_clawed_frog.locus228.1
------
MERPNTRRTKCTAKTEEFLSYNDVISIILSLISVSSFFISVLILGVFITYRDTPIVRANNRSLSFLLLVSIKLSFLSVFLFLGRPVDITCMLRNVTFGITFSIAVSSLLAKTIMVCVAFKATKPGSSWRKWLGVKLSNSVVLFCSSIQI
IICMTWLAISPPFQELDIHTSPGTIIIQCNEGSAIGFYSVIGYMGLLAAVSFVLAFLARSLPDSFNEAKYITFSMLLFCSVWITMIPAYLSTKGKNTVCVEIFAILTSSAGLLACIFLPKCYTIVFRPEINTRSYLRGKQF-
>Western_clawed_frog.locus229.1
------
MEWTNNKRTKCITKTEEFLSYNDLISVIFSSISVLFFLTTVLVLSVFITYRDSPIVRANNRSLSFLLLVSIKLSFLSVFLFLGRPVDITCMLRIITFGITFSIAVSSLLAKTIMVCVAFKATKPGSSWRKWLGVKLSNSVALFCSSIQI
IICMTWLAISPPFQELDIHTSPGTIIIQCNEGSAIGFYSVIGYMGLLAAVSFVLAFLARSLPDSFNEAKYITFSMLLFCSVWITMIPAYLSTKGKNTVCVEIFAILTSSAGLLACIFLPKCYTIVFRPEINKKSHLLGN---
>Western_clawed_frog.locus214.1
------
MEWPNDKRTKCIARTEVFLSFNDVISLFFSSVSLLFFLLTLLILGVFIIYRDSPIVRANNRSLSFLLLVSIKLSFLSVFLFLGRPVDITCMLRIIAFGITFSIAVSSLLAKTIMVCVAFKATKPGSSWRKWLGVKLSNSVVLFCSSIQI
IICMTWLAISPPFQELDIHTSPGTIIIQCNEGSAIGFYSVIGYMGLLAAVSFVLAFLARSLPDSFNEAKYITFSMLLFCSVWITMIPAYLSTKGKNTVCVEIFAILTSSAGLLACIFLPKCYIILFRSEINTKSHLLGNKY-
>Western_clawed_frog.locus37.1
------
MEWPNKMKTKCVPKAVEYLLYSDGISLFFSSTSGFFFLINLLILGTFTLYQSSPIVRANNRSLSFLLLVSIKLSFLSVFLFLGRPVDITCMLRIITFGITFSIAVSSLLAKTIMVCVAFKATKPGSSWRKWLGVKLSNSVVLFCSSIQI
IICMTWLAISPPFQELDIHTSPGTIIIQCNEGSAIGFYSVIGYMGLLAAVSFVLAFLARSLPDSFNEAKYITFSMLLFCSVWITMIPAYLSTKGKNTVCVEIFAILTSSAGLLACIFLPKCYIILFRPEINAKCHLLTKQTR
>Western_clawed_frog.locus69.1
------
MEWPNVKKDQCIPKTEEFLPYHDGISVFFSAVSLLFALITLLILRVFIVYQDSPIVRANNRSLSFLLLVSIKLSFLSVFLFLGRPVDITCMLRIITFGITFSIAVSSLLAKTIMVCVAFKATKPGSSWRKWLGVKLSNSVVLFCSSIQI
IICMTWLAISPPFQELDIHTSPGTIIIQCNEGSAIGFYSVIGYMGLLAAVSFVLAFLARSLPDSFNEAKYITFSMLLFCSVWITMIPAYLSTKGKNTVCVEIFAILTSSAGLLASIFLPKCYTILIRPETNKKTHFHGNKSL
>Western_clawed_frog.locus5.1
CARCPDLQWPNENKTQCITSTETFLSYSDGISVVFFSISLLFFLKTVLIFGVFIIYRETPIVRANNRSLSFLLLVSIKLSFLSVFLFLGRPVDITCMLRIITFGITFSIAVSSLLAKTIMVCVAFKATKPGSSWRKWLGVKLSNSVVLF
CSSIQIIICTTWLAISPPFQELDIHTSPGTIIIQCNEGSAIGFYSVIGYMGLLAAVSFVLAFLARSLPDSFNEAKYITFSMLLFCSVWITMIPAYLSTKGKNTVCVEIFAILTSSAGLLACIFLPKCYIILLIPENNTKSNLLGGKVL
>Western_clawed_frog.locus208.1
------
MEWPNEQKDQCIPKIEEFLSFVDVISVFFSFISFIFFLITEVILGIFLKYRDTPIVRANNRSLSFLLLVSIKLSFLSVFLFLGRPVDITCMLRIITFGITFSIAVSSLLAKTIMVCVAFKATKPGSSWRKWLGVKLSNSVVLFCSSIQI
IICMTWLAISPPFQELDIHTSPGTIIIQCNEGSAIGFYSVIGYMGLLAAVSFVLAFLARSLPDSFNEAKYITFSMLLFCSVWITMIPAYLSTKGKNTVCVEIFAILTSSAGLLACIFLPKCYIILFRPEINTKSHLLKTTY-
>Western_clawed_frog.locus210.1
------
MEWPNEQKDQCIPKIEEFLSFADVISVFFSFISVLFFVITAVTLGIFLKYQDSPIVRANNRSLSFLLLVSIKLSFLSVFLFLGRPVDITCMLRIITFGITFSIAVSSLLAKTIMVCVAFKATKPGSSWRKWLGVKLPNSVVLFCSSIQI
IICMTWLAISPPFQELDIHTSPGTIIIQCNEGSAIGFYSVIGYMGLLAAVSFVLAFLARSLPDSFNEAKYITFSMLLFCSVWITMIPAYLSTKGKNTVCVEIFAILTSSAGLLACIFLPKCYIILFRPEINTKSHLLENKS-
>Western_clawed_frog.locus209.1
------
MEWPNEQKDQCIPKIQEFLSYVDVISVFFSFISFLFFLITEVILGIFLKYRDSPIVRANNRSLSFLLLVSIKLSFLSVFLFLGRPVDITCMLRIITFGITFSIAVSSLLAKTIMVCVAFKATKPGSSWRKWLGVKLSNSVVLFCSSIQI
IICMTWLAISPPFQELDIHTSPGTIIIQCNEGSAIGFYSVIGYMGLLAAVSFVLAFLARSLPDSFNEAKYITFSMLLFCSVWITMIPAYLSTKGKNTVCVEIFAILTSSAGLLACIFLPKCYIILFTPEINTKSHLLENKY-
>Western_clawed_frog.locus207.1
------
MEWPNENKIQCFAKMEEFLSYVDGISMFFSFISLLFFLITEVILGIFLKYQDSPIVRANNRSLSFLLLVSIKLSFLSVFLFLGRPVDITCMLRIITFGITFSIAVSSLLAKTIMVCVAFKATKPGSSWRKWLGVKLSNSVVLFCSSIQI
IICMTWLAISPPFQELDIHTSPGTIIIQCNEGSAIGFYSVIGYMGLLAAVSFVLAFLARSLPDSFNEAKYITFSMLLFCSVWITMIPAYLSTKGKNTVCVEIFAILTSSAGLLASIFLPKCYIIMFRPEINTKSHLLENKY-
>Western_clawed_frog.locus206.1
------
MEWPNENKIQCFARMEEFLSYVDFISLFFSFFSLLFFLVTAVILGIFLKYRDSPIVRANNRSLSFLLLVSIKLSFLSVFLFLGRPVDITCMLRIITFGITFSIAVSSLLAKTIMVCVAFKATKPGSSWRKWLGVKLSNSVVLFCSSIQI
IICMTWLAISPPFQELDIHTSPGTIIIQCNEGSAIGFYSVIGYMGLLAAVSFVLAFLARSLPDSFNEAKYITFSMLLFCSVWITMIPAYLSTKGKNTVCVEIFAILTSSAGLLACIFLPKCYIILFRPEINTKSHLFENKY-
>Western_clawed_frog.locus205.1
------
MEWPNENKTQCFARMEEFLSYVDVISVFFSFISLLFFLITEVILGIFLNYRDTPIVRANNRSLSFLLLVSIKLSFLSVFLFLGRPVDITCMLRIITFGITFSIAVSSLLAKTIMVCVAFKATKPGSSWRKWLGVKLSNSVVLFCSSIQI
IICMTWLAISPPFQELDIHTSPGTIIIQCNEGSAIGFYSVIGYMGLLAAVSFVLAFLARSLPDSFNEAKYITFSMLLFCSVWITMIPAYLSTKGKNTVCVEIFAILTSSAGLLACIFLPKCYIILFRPEINTKSHLLENKY-
>Western_clawed_frog.locus204.1
------
MEWPNENKTQCFARMEEFLSYVDVISVFFSFISLLFFLITEVILGIFLNYRDTPIVRANNRSLSFLLLVSIKLSFLSVFLFLGRPVDITCMLRIITFGITFSIAVSSLLAKTIMVCVAFKATKPGSSWRKWLGVKLSNSVVLFCSSIQI
IICMTWLAISPPFQELDIHTSPGTIIIQCNEGSAIGFYSVIGYMGLLAAVSFVLAFLARSLPDSFNEAKYITFSMLLFCSVWITMIPAYLSTKGKNTVCVEIFAILTSSAGLLACIFLPKCYIILFRPEINTKSHLLENKY-
>Western_clawed_frog.locus211.1
------
MEWSNQNKSQCFARMEEFLSYVDVISVFFSFISVLFFLITVVILGIFLKYRDTPIVRANNRSLSFLLLVSIKLSFLSVFLFLGRPVDITCMLRIITFGITFSIAVSSLLAKTIMVCVAFKATKPGSSWRKWLGVKLSNSVVLFCSSIQI
IICMTWLAISPPFQELDIHTSPGTIIIQCNEGSAIGFYSVIGYMGLLAAVSFVLAFLARSLPDSFNEAKYITFSMLLFCSVWITMIPAYLSTKGKNTVCVEIFAILTSSAGLLACIFLPKCYIILFRPEINTKSHLLENKY-
>Western_clawed_frog.locus33.1
------
MEWPNEKMIQCIARNEEFLSYDNVVSAIFSFISVLFFLITEVILGFFITYRDRPIVRANNRSLSFLLLVSIKLSFLSVFLFLGRPVDITCMLRIITFGITFSIAVSSLLAKTIMVCVAFKATKPGSSWRKWLGVKLSNSVVLFCSSIQI
IICMTWLAISPPFQELDIHTSPGTIIIQCNEGSAIGFYSVIGYMGLLAAVSFVLAFLARSLPDSFNEAKYITFSMLLFCSVWITMIPAYLSTKGKNTVCVEIFAILTSSAGLLASIFLPKCYIILLRPDMNTKSYLHYNNNV
>Western_clawed_frog.locus282.1
------
MEWPNEQKDQCIAKMEEFLSFDDVISAIFLFISVLLFLITGGIMRIFIIYRDSPIVRANNRSLSFLLLVSIKLSFLSVFLFLGRPVDITCMLRIITFGITFSIAVSSLLAKTIMVCVAFKATKPGSSWRKWLGVKLSNSVVLFCSSIQI
IICMTWLAISPPFQELDIHTSPGTIIIQCNEGSAIGFYSVIGYMGLLAAVSFVLAFLARSLPDSFNEAKYITFSMLLFCSVWITMIPAYLSTKGKNTVCVEIFAILTSSAGLLACIFLPKCYIILLRPEINLKTSLLRNQ--
>Western_clawed_frog.locus22.1
------
MEWPNEKKSQCIAKMEEFLSYGNVISVICLFISVLLFLITEVILGVFIKYRDSPIVRANNRSLSFLLLVSIKLSFLSVFLFLGRPVDITCMLRIITFGITFSIAVSSLLAKTIMVCVAFKATKPGSSWRKWLGVKLSNSVVLFCSSIQI
IICMTWLAISPPFQELDIHTSPGTIIIQCNEGSAIGFYSVIGYMGLLAAVSFVLAFLARSLPDSFNEAKYITFSMLLFCSVWITMIPAYLSTKGKNTVCVEIFAILTSSAGLLACIFLPKCYTILLRPELNIKTSLLRN---
>Western_clawed_frog.locus23.1
--------------------
MEEFLSYDDVISVICLFISVLFFLITEVILGVFIKYQDTPIVRANNRSLSFLLLVSIKLSFLSVFLFLGRPVDITCMLRIITFGITFSIAVSSLLAKTIMVCVAFKATKPGSSWRKWLGVKLSNSVVLFCSSIQIIICMTWLAISPPFQ
ELDIHTSPGTIIIQCNEGSAIGFYSVIGYMGLLAAVSFVLAFLARSLPDSFNEAKYITFSMLLFCSVWITMIPAYLSTKGKNTVCVEIFAILTSSAGLLASIFLPKCYTILLRPELNIKTSLLRN---
>Western_clawed_frog.locus21.1
CIRCPLVEWPNEKKSQCIAKTEEFLSYNDVISVICLFISILFFLLTEVILGVFISYRDTPIVRANNRSLSFLLLVSIKLSFLSVFLFLGRPVDITCMLRIITFGITFSIAVSSLLAKTIMVCVAFKATKPGSSWRKWLGVKLSNSVVLF
CSSIQIIICMTWLAISPPFQELDIHTSPGTIIIQCNEGSAIGFYSVIGYMGLLAAVSFVLAFLARSLPDSFNEAKYITFSMLLFCSVWITMIPAYLSTKGKNTVCVEIFAILTSSAGLLACIFLPKCYTILLRPDINMKSNLLRNKV-
```

```
>Western_clawed_frog.locus28.1
CIQCPDMEWPNEKKIQCIAKTEEFLSYDGIISVIFLFTSVLFFLITEAILGVFIKYWDSPIVRANNRSLSFLLLVSIKLSFLSVFLFLGRPVDITGMLRIITFGITFSIAVSSLLAKTIMVCVAFKATKPGSSWRKWLGVKLSNSVVLF
CSSIQIIICMTWLAISPPFQELDIHTSPGTIIIQCNEGSAIGFYSVIGYMGLLAAVSFVLAFLARSLPDSFNEAKYITFSMLLFCSVWITMIPAYLSTKGKNTVCVEIFAILTSSAGLLASIFLPKCYTILLRPNVNTKPNFLRNKYK
>Western_clawed_frog.locus29.1
------
MEWPNEKKIQCIAKTEEFLSYDGIISVFFLFFSVLFFLITEVILGVFIKYQDTPIVRANNRSLSFLLLVSIKLSFLSVFLFLGRPVDITCMLRIITFGITFSIAVSSLLAKTIMVCVAFKATKPGSSWRKWLGVKLSNSVVLFCSSIQI
IICMTWLAISPPFQELDIHTSPGTIIIQCNEGSAIGFYSVIGYMGLLAAVSFVLAFLARSLPDSFNEAKYITFSMLLFCSVWITMIPAYLSTKGKNTVCVEIFAILTSSAGLLASIFLPKCYTILLRPDVNMKTNFLRKK--
>Western_clawed_frog.locus30.1
------
MEWPNEKKIQCIAKTEEFLSYDGIISVIFLFFSVLFFLITEVILGVFIKHQDTPIVRANNRSLSFLLLVSIKLSFLSVFLFLGRPVDITCMLRIITFGITFSIAVSSLLAKTIMVCVAFKATKPGSSWRKWLGVKLSNSVVLFCSSIQI
IICMTWLAISPPFQELDIHTSPGTIIIQCNEGSAIGFYSVIGYMGLLAAVSFVLAFLARSLPDSFNEAKYITFSMLLFCSVWITMIPAYLSTKGKNTVCVEIFAILTSSAGLLACIFLPKCYTILLRPDVRIKTNLLRKK--
>Western_clawed_frog.locus24.1
CIQCPDMEWPNEKKIQCIAKTEEFLSYDDVISIIFSFISVLFLLITGQILGVFTKHRNTPIVRANNRGLSFVLLVSIKLSFLSVLLFLGRPVDITCMLRIITFGITFSIAVSSLLAKTIMVCVAFKATKPGSSWRKWLGVKLSNSVVLF
CSSIQIIICMTWLAISPPFQELDIHTSPGTIIIQCNEGSAIGFYSVIGYMGLLAAVSFVLAFLARSLPDSFNEAKYITFSMLLFCSVWITMIPAYLSTKGKNTVCVEIFAILTSSAGLLACIFLPKCYTILLRPELNMKTSLLRN---
>Western_clawed_frog.locus25.1
CIQCPDMEWPNEKKIQCIAKLEEFLSYDDVISIIFSFISVFFLLITGEILGVFIKHCNTPIVRANNRGLSFLLLVSIKLSFLSVFLFLGRPVDITCMLRIITFGITFSIAVSSLLAKTIMVCVAFKATKPGSSWRKWLGVKLSNSVVLF
CSSIQIIICMTWLAISPPFQELDIHTSPGTIIIQCNEGSAIGFYSVIGYMGLLAAVSFVLAFLARSLPDSFNEAKYITFSMLLFCSVWITMIPAYLSTKGKNTVCVEIFAILTSSAGLLACIFLPKCYTILLRPGVNMKSNLLRSKCK
>Western_clawed_frog.locus117.1
------
MEWPNEEKIQCFAKMEEFLSYDDVISIIFSSISVLFFLITLLVLVLFISNHDTPIVRANNRSLSFLLLVSIKLSFLSVFLFLGRPVDITCMLRIITFGITFSIAVSSLLAKTIMVCVAFKATKPGSSWRKWLGVKLSNSVVLFCSSIQI
IICMTWLAISPPFQELDIHTSPGTIIIQCNEGSAIGFYSVIGYMGLLAAVSFVLAFLARSLPDSFNEAKYITFSMLLFCSVWITMIPAYLSTKGKNTVCVEIFAILTSSAGLLACIFLPKCYTILLRPDVNTKISLLRNK--
>Western_clawed_frog.locus116.1
------
MEWPNEEKIKCFAKMEEFLSYDHVISMIFSSISVLFFLITLLVLVVFISNHDTPIVRANNRSLSFLLLVSIKLSFLSVFLFLGRPVDITCMLRIITFGITFSIAVSSLLAKTIMVCVAFKATKPGSSWRKWLGVKLSNSVVLFCSSIQI
IICMTWLAISPPFQELDIHTSPGTIIIQCNEGSAIGFYSVIGYMGLLAAVSYVLAFLARSLPDSFNEAKYITFSMLLFCSVWITMIPAYLSTKGKNTVCVEIFAILTSSAGLLASIFLPKCYTILLRLDVNTKISLFRNK--
>Western_clawed_frog.locus115.1
------
MEWPNEEKIQCFAKIKEFLSYDDVISIIFSSISVLFFLITLLVLVLFISNHDSPIVRANNRSLSFLLLVSIKLSFLSVFLFLGRPVDITCMLRIITFGITFSIAVSSLLAKTIMVCVAFKATKPGSSWRKWLGVKLSNSVVLFCSSIQI
IICMTWLAISPPFQELDIHTSPGTIIIQCNEGSAIGFYSVIGYMGLLAAVSFVLAFLARSLPDSFNEAKYITFSMLLFCSVWITMIPAYLSTKGKNTVCVEIFAILTSSAGLLASIFLPKCYTILLRPDVNMKTSLLRNK--
>Western_clawed_frog.locus118.1
------
MEWPNEEKIKCFAMMEEFLSYDDVISMIFSLISVLFFLITLLVLVLFITYRDSPIVRANNRSLSFLLLVSIKLSFLSVFLFLGRPVDITCMLRIITFGITFSIAVSSLLAKTIMVCVAFKATKPGSSWRKWLGVKLSNSVVLFCSSIQI
IICMTWLAISPPFQELDIHTSPGTIIIQCNEGSAIGFYSVIGYMGLLAAVSFVLAFLARSLPDSFNEAKYITFSMLLFCSVWITMIPAYLSTKGKNTVCVEIFAILTSSAGLLASIFLPKCYIIMFKPEMNNKKNLL-----
>Western_clawed_frog.locus32.1
------
MEWPNEQQNQCIPKMEEFLSYNDVISVSLSSISVLFFLITLLVLGLFISHWDTPIVRANNRSLSFLLLVSIKLSFLSVFLFLGRPVDITCMLRIITFGITFSIAVSSLLAKTIMVCVAFKATKPGSSWRKWLGVKLSNSVVLFCSSIQI
IICMTWLAISPPFQELDIHTSPGTIIIQCNEGSAIGFYSVIGYMGLLAAVSFVLAFLARSLPDSFNEAKYITFSMLLFCSVWITMIPAYLSTKGKNTVCVEIFAILTSSAGLLACIFLPKCYTILLRPDVNMKSNLLRNN--
>Western_clawed_frog.locus31.1
------
MEWPNQQKNQCIPKMEEFLSYNDVISVFLSSISILFFLITLLILGVFISHWDSPIVRANNRSLSFLLLVSIKLSFLSVFLFLGRPVDITCMLRIITFGITFSIAVSSLLAKTIMVCVAFKATKPGSSWRKWLGVKLSNSVVLFCSSIQI
IICMTWLAISPPFQELDIHTSPGTIIIQCNEGSAIGFYSVIGYMGLLAAVSFVLAFLARSLPDSFNEAKYITFSMLLFCSVWITMIPAYLSTKGKNTVCVEIFAILTSSAGLLFCIFLPKCYTILLRPDVKMKTNFLRKK--
>Western_clawed_frog.locus35.1
CMRCPDMEWPNKKKDQCVSKMEDYLSYKELISVLFSLISVTFALIAIVILGVFIFYMDSPIVRANNRSLSFLLLVSIKLSFLSVFLFLGRPVDITCMLRIITFGITFSIAVSSLLAKTIMVCVAFKATKPGSSWRKWLGVKLSNSVVLF
CSSIQIIICMTWLAISPPFQELDIHTSPGTIIIQCNEGSAIGFYSVIGYMGLLAAVSFVLAFLARSLPDSFNEAKYITFSMLLFCSVWITMIPAYLSTKGKNTVCVEIFAILTSSAGLLACIFLPKCYIILLRPDINMKRNLRRK---
>Western_clawed_frog.locus34.1
------
MEWPNRKKTRCIPKMEDFLSYNHVISLFFSVASVIFFLKTQLIIVVFIIHIDTPIVRANNRNLSFLLLVSIKLSFLSVFLFLGRPVDITCMLRIITFGITFSIAVSSLLAKTIMVCVAFKATKPGSSWRKWLGVKLSNSVVLFCSSIQI
IICMTWLAISPPFQELDIHTSPGTIIIQCNEGSAIGFYSVIGYMGLLAAVSFVLAFLARSLPDTFNEAKYITFSMLLFCSVWITMIPAYLSTKGKNTVCVEIFAILTSSAGLLACIFLPKCYTILLRPEINMNRNLLRNKDK
>Western_clawed_frog.locus62.1
CTQCPDMEWPNEMRNQCIQREEVFLSYNDVISIFFSAFSVLFFLITQLILQVFITYRDTPIVRANNRSLSFLLLVSIKLSFLSVFLFLGRPVDITCMLRIITFGITFSIAVSSLLAKTIMVCVAFKATKPGSSWRKWLGVKLSNSVVLF
CSSIQIIICMTWLAISPPFQELDIHTSPGTIIIQCNEGSAIGFYSVIGYMGLLAAVSFVLAFLARSLPDSFNEAKYITFSMLLFCSVWITMIPAYLSTKGKNTVCVEIFAILTSSAGLLASIFLPKCYIMMFRPEMNTKTHLHGNK--
>Western_clawed_frog.locus58.1
------
MEWPNEMKDQCIQREEVFLSYNDVISICFSAVSILFFLITQFILQVFIKHRDTPIVRANNRSLSFLLLVSIKLSFLSVFLFLGRPVDITCMLRIITFGITFSIAVSSLLAKTIMVCVAFKATKPGSSWRKWLGVKLSNSVVLFCSSIQI
IICMTWLAISPPFQELDIHTSPGTIIIQCNEGSAIGFYSVIGYMGLLAAVSFVLAFLARSLPDSFNEAKYITFSMLLFCSVWITMIPAYLSTKGKNTVCVEIFAILTSSAGLLASIFLPKCYTILLRPEMNTKNNLLGDK--
>Western_clawed_frog.locus59.1
------
MEWPNEMRNQCILREEVFLSYNDVISIFFSAASVIYFLITQLIIVVFIKHMDSPIVRANNRSLSFLLLVSIKLSFLSVFLFLGRPVDITCMLRIITFGITFSIAVSSLLAKTIMVCVAFKATKPGSSWRKWLGVKLSNSVVLFCSSIQI
IICMTWLAISPPFQELDIHTSPGTIIIQCNEGSAIGFYSVIGYMGLLAAVSFVLAFLARSLPDSFNEAKYITFSMLLFCSVWITMIPAYLSTKGKNTVCVEIFAILTSSAGLLACIFLPKCYTILLRPEMNMKTNLLGNK--
>Western_clawed_frog.locus26.1
CTQCSDMEWPNEMKDQCLEKKRDFLSYNNVISIFFSTVSVLFFLITQMVLGVFITYMDSPIVRANNRSLSFLLLVSIKLSFLSVFLFLGRPVDITCMLRIITFGITFSIAVSSLLAKTIMVCVAFKATKPGSSWRKWLGVKLSNSVVLF
CSSIQIIICMTWLAISPPFQELDIHTSPGTIIIQCNEGSAIGFYSVIGYMGLLAAVSFVLAFLARSLPDSFNEAKYITFSMLLFCSVWITMIPAYLSTKGKNTVCVEIFAILTSSAGLLACIFLPKCYTILLRPDVNVKTNLLRNN--
>Western_clawed_frog.locus27.1
------
MECPNEMKNQCLEKKKDFLSYNDAISIFFSTVSVLFFLITQVVLRVFIIHRGTPIVRANNRSLSFLLLVSIKLSFLSVFLFLGRPVDITCMLRIITFGITFSIAVSSLLAKTIMVCVAFKATKPGSSWRKWLGVKLSNSVVLFCSSIQI
IICMTWLAISPPFQELDIHTSPGTIIIQCNEGSAIGFYSVIGYMGLLAAVSFVLAFLARSLPDSFNEAKYITFSMLLFCSVWITMIPAYLSTKGKNTVCVEIFAILTSSAGLLACIFLPKCYTILLRPDVNIKTNLLRNN--
>Western_clawed_frog.locus120.1
------
MEWPNEKNIQCIASKEDFLSYSDKISLFISPIVIFFLFITLLILRVFVMHWDSPIVRANNRSLSFLLLVSIKLSFLSVFLFLGRPVDITCMLRIITFGITFSIAVSSLLAKTIMVCVAFKATKPGSSWRKWLGVKLSNSVVLFCSSIQI
IICMTWLAISPPFQELDIHTSPGTIIIQCNEGSAIGFYSVIGYMGLLAAVSFVLAFLARSLPDSFNEAKYITFSMLLFCSVWITMIPAYLSTKGKNTVCVEIFAILTSSAGLLASIFLPKCYTILLRPEMNMKTSLIRNK--
>Western_clawed_frog.locus50.1
------
MEWPNEKKDHCIASREDFLSFNDTISVLCSSISILFFLITSWILGVFITYRDSPIVRANNRSLSFLLLVSIKLSFLSVFLFLGRPVDITCMLRIITFGITFSIAVSSLLAKTIMVCVAFKATKPGSSWRKWLGVKLSNSVVLFCSSIQI
IICMTWLAISPPFQELDIHTSPGTIIIQCNEGSAIGFYSVIGYMGLLAAVSFVLAFLARSLPDSFNEAKYITFSMLLFCSVWITMIPAYLSTKGKNTVCVEIFAILTSSAGLLACIFLPKCYIILIRPEMNTKTYLLGNKDN
>Western_clawed_frog.locus60.1
------
MEWPNERKDQCIASKDDFLSYNDTVSVLCSSISILFFLITSWISGVFITYRDSPIVRANNRSLSFLLLVSIKLSFLSVFLFLGRPVDITCMLRIITFGITFSIAVSSLLAKTIMVCVAFKATKPGSSWRKWLGVKLSNSVVLFCSSIQI
IICMTWLAISPPFQELDIHTSPGTIIIQCNEGSDIGFYSVIGYMGLLAAVSFVLAFLARSLPDSFNEAKYITFSMLLFCSVWITMIPAYLSTKGKNTVCVEIFAILTSSAGLLACIFLPKCYTILLRPEINLKTNLLGNK--
>Western_clawed_frog.locus191.1
CFPCPDMEWPNEGKDQCITRKEDFLSYNNVISVFVSSVAIFFFLITLLIFDIFITFRDSPIVRANNRSLSFLLLVSIKLSFLSVFLFLGRPVDITCMLRIITFGITFSIAVSSLLAKTIMVCVAFKATKPGSSWRKWLGVKLSNSVVLF
CSSIQIIICMTWLAISPPFQELDIHTSPGTIIIQCNEGSAIGFYSVIGYMGLLAAVSFVLAFLARSLPDSFNEAKYITFSMLLFCSVWITMIPAYLSTKGKNTVCVEIFAILTSSAGLLASIFLPKCYTILFRPEMNVKSFLLGNK--
>Western_clawed_frog.locus19.1
----------------------------
DLISAFISLVTILFFLVTQLILGVFIKYQVTPIVRANNRSLSFLLLVSIKLSFLSVFLFLGRPVDITCMLRIITFGITFSIAVSSLLAKTIMVCVAFKATKPGSSWRKWLGVKLSNSVVLFCSSIQIIICMTWLAISPPFQELDIHTSP
GTIIIQCNEGSAIGFYSVIGYMGLLAAVSFVLAFLARSLPDSFNEAKYITFSMLLFCSVWITMIPAYLSTKGKNTVCVEIFAILTSSAGLLACIFLPKCYIILFRPKRNTKMNLFYKA--
>Western_clawed_frog.locus18.1
CIRCPDMEWPNKNKHQCIGRNADFLSYKDLISIFISIASVLFFLVTQLILGVFISYRDTPIVRANNWGLSFLLLVSIKLSFLSVFLFLGRPVDITCMLRIITFGITFSIAVSSLLAKTIMVCVAFKATKPGSSWRKWLGVKLSNSVVLF
CSSIQIIICMTWLAISPPFQELDIHTSPGTIIIQCNEGSAIGFYSVIGYMGLLAAVSFVLAFLARSLPDSFNEAKYITFSMLLFCSVWITMIPAYLSTKGKNTVCVEIFAILTSSVGLLACIFLPKCYIILLKPERNKKMNLLYKA--
>Western_clawed_frog.locus20.1
------
MEWPNEMKNQCIRRNEDFLSYNDLISIFISVVSVLFFLLTQLILVVFIQYRDSPIVRANNRSLSFLLLVSIKLSFLSVFLFLGRPVDITCMLRIITFGITFSIAVSSLLAKTIMVCVAFKATKPGSSWRKWLGVKLSNSVVLFCSSIQI
IICMTWLAISPPFQELDIHTSPGTIIIQCNEGSAIGFYSVIGYMGLLAAVSFVLAFLARSLPDSFNEAKYITFSMLLFCSVWITMIPAYLSTKGKNTVCVEIFAIVTSSAGIIACIFLPKCYVILLRPERNTKTNLLHFC--
>Western_clawed_frog.locus16.1
CIRCSDMEWPNEMKNQCIGRKEEFLSYEDLISVFISLVSVLFGLITQLILGIFIKYRDSPIVRANNRSLSFLLLVSIKLSFLSVFLFLGRPVDITCMLRIITFGITFSIAVSSLLAKTIMVCVAFKATKPGSSWRKWLGVKLSNSVVLF
CSSIQIIICMTWLAISPPFQELDIHTSPGTIIIQCNEGSAIGFYSVIGYMGLLAAVSFVLAFLARSLPDSFNEAKYITFSMLLFCSVWITMIPAYLSTKGKNTVCVEIFAILTSSAGLLACIFLLKCYIILLKPERNTKTNLFYKA--
>Western_clawed_frog.locus17.1
------MEWPNE-
MNQCIGRKEEFLSYEDLISVFISLVSVLFGLKTQLILGVFIKYLDSPIVRANNRSLSFLLLVSIKLSFLSVFLFLGRPVDITCMLRIITFGITFSIAVSSLLAKTIMVCVAFKATKPGSSWRKWLGVKLSNSVVLFCSSIQIIICMTWL
AISPPFQELDIHTSPGTIIIQCNEGSAIGFYSVIGYMGLLAAVSFVLAFLARSLPDSFNEAKYITFSMLLFCSVWITMIPAYLSTKGKNTVCVEIFAILTSSAGLLASIFLPKCYIILLRPERNTKLICFTKPNN
>Western_clawed_frog.locus12.1
------MEWPNEM-
NQCIRRNEEFLSYKDLISICISLVSVFFILTTQLILGVSIKYRDSPIVRANNRSLSFLLLVSIKLSFLSVFLFLGRPVDITCMLRIITFGITFSIAVSSLLAKTIMVCVAFKATKPGSSWRKWLGVKLSNSVVLFCSSIQIIICMTWLA
ISPPFQELDIHTSPGTIIIQCNEGSAIGFYSVIGYMGLLAAVSFVLAFLARSLPDSFNEAKYITFSMLLFCSVWITMIPAYLSTKGKNTVCVEIFAILTSSAGLLACIFLPKCYIILLRPEMNTKNNLLYKD--
>Western_clawed_frog.locus13.1
------MEWPNEM-
NQCIRRNEEFLSYKDLISAFISLVSVFFILTTQLILGVSIKYRDTPIVRANNRSLSFLLLVSIKLSFLSVFLFLGRPVDITCMLRIITFGITFSIAVSSLLAKTIMVCVAFKATKPGSAWRKWLGVKLSNSVVLFCSSIQIIICMTWLA
ISPPFQELDIHTSPGTIIIQCNEGSAIGFYSVIGYMGLLAAVSFVLAFLARSLPDSFNEAKYITFSMLLFCSVWITMIPAYLSTKGKNTVCVEIFAILTSSAGLLACIFLPKCYIILLKPEMNTRSNLLYKD--
>Western_clawed_frog.locus14.1
CIKCPDMEWPNDKKNQCIRRNEEFLSYKDLISAFISLVSVLFILTTQLILGVFMKYRDSPIVRANNRSLSFLLLVSIKLSFLSVFLFLGRPVDITCMLRIITFGITFSIAVSSLLAKTIMVCVAFKATKPGSSWRKWLGVKLSNSVVLF
CSSIQIIICMTWLAISPPFQELDIHTSPGTIIIQCNEGSAIGFYSVIGYMGLLAAVSFVLAFLARSLPDSFNEAKYITFSMLLFCSVWITMIPAYLSTKGKNTVCVEIFAILTSSAGLLASIFLPKCYVILLRPEMNTKTNLLYKD--
>Western_clawed_frog.locus15.1
------
MEWPNEMKNQCIGRKEEFLSYKDLISVFISLVSAFFILTTQLILGVFIKYRDTPIVRANNRSLSFLLLVSIKLSFLSVFLFLGRPVDITCMLRIITFGITFSIAVSSLLAKTIMVCVAFKATKPGSSWRKWLGVKLSNSVVLFCSSIQI
IICMTWLAISPPFQELDIHTSPGTIIIQCNEGSAIGFYSVIGYMGLLAAVSFVLAFLARSLPDSFNEAKYITFSMLLFCSVWITMIPAYLSTKGKNTVCVEIFAILTSSAGLLACIFLPKCYIILLRPEMNTKSCLFTN---
>Western_clawed_frog.locus11.1
CTKCPDMEWPNEM-
NQCIGRKEEFLSYKDLISICISMISVLLFLMTQLILGVFIKYRDSPIVRANNRSLSFLLLVSIKLSFLSVFLFLGRPVDITCMLRIITFGITFSIAVSSLLAKTIMVCVAFKATKPGSSWRKWLGVKLSNSVVLFCSSIQIIICMTWLA
ISPPFQELDIHTSPGTIIIQCNEGSAIGFYSVIGYMGLLAAVSFVLAFLARSLPDSFNEAKYITFSMLLFCSVWITMIPAYLSTKGKNTVCVEIFAILTSSAGLLACIFLPKCYIILLKPEMNTKSNLLYKV--
```

```
>Western_clawed_frog.locus2.1
------
MEWPNEMKNQCIGRKEEFLSYKDLISAFISLVSLLFGLKTLLILGIFIKYRDSPIVRANNRSLSFLLLVSIKLSFLSVFLFLGRPVDITCMLRIITFGITFSIAVSSLLAKTIMVCVAFKATKPGSSWRKWLGVKLPNSVVLFCSSIQI
IICMTWLAISPPFQELDIHTSPGTIIIQCNEGSAIGFYSVIGYMGLLAAVSFVLAFLARSLPDSFNEAKYITFSMLLFCSVWITMIPAYLSTKGKNTVCVEIFAILTSSAGLLACIFLPKCYIIQFRSEMNTKCHLLGNRQY
>Western_clawed_frog.locus3.1
------
MEWPNEKKNQCIGRKEEFLSYKDLISVFISLVSVLFGLKTQLILGIFIKYRDSPIVRANNRSLSFLLLVSIKLSFLSVFLFLGRPVDITCMLRIITFGITFSIAVSSLLAKTIMVCVAFKATKPGSSWRKWLGVKLSNSVVLFCSSIQI
IICMTWLAISPPFQELDIHTSPGTIIIQCNEGSAIGFYSVIGYMGLLAAVSFVLAFLARSLPDSFNEAKYITFSMLLFCSVWITMIPAYLSTKGKNTVCVEIFAILTSSAGLLACIFLPKCYIIQFRSEMNTKCHLLGNRQY
>Western_clawed_frog.locus4.1
------
MEWPNEKKNQCIGRKEEFLSYKDLISAFISLVSVLFGLKTQLILGIFIKYLDSPIVRANNRSLSFLLLVSIKLSFLSVFLFLGRPVDITCMLRIITFGITFSIAVSSLLAKTIMVCVAFKATKPGSSWRKWLGVKLSNSVVLFCSSIQI
IICMTWLAISPPFQELDIHTSPGTIIIQCNEGSAIGFYSVIGYMGLLAAVSFVLAFLARSLPDSFNEAKYITFSMLLFCSVWITMIPAYLSTKGKNTVCVEIFAILTSSAGLLACIFLPKCYIILLIPENNTKSNLLGGKC-
>Western_clawed_frog.locus6.1
------
MEWPNEKKNQCIGRKEEFLSYEDLISAFISLVSVLFGLITQLILGVFIKYWDTPIVRANNRSLSFLLLVSIKLSFLSVFLFLGRPVDITCMLRIITFGITFSIAVSSLLAKTIMVCVAFKATKPGSSWRKWLGVKLSNSVVLFCSSIQI
IICMTWLAISPPFQELDIHTSPGTIIIQCNEGSAIGFYSVIGYMGLLAAVSFVLAFLARSLPDSFNEAKYITFSMLLFCSVWITMIPAYLSTKGKNTVCVEIFAILTSSAGLLACIFLPKCYTILLRPELNMKTNLLGNK--
>Western_clawed_frog.locus787.1
------
MEWPMREKNQCIARTEQFLSCNDGISVFFSSISDLSFLTTLLILGAFITYRDSPIVRANNRSLSFLLLVSIKLSFLSVFLFLGRPVDITCMLRIITFGITFSIAVSSLLAKTIMVCVAFKATKPGSSWRKWLGVKLSNSVVLFCSSIQI
IICMTWLAISPPFQELDIHTSPGTIIIQCNEGSAIGFYSVIGYMGLLAAVSFVLAFLARSLPDSFNEAKYITFSMLLFCSVWITMIPAYLSTKGKNTVCVEIFAILTSSAGLLASIFLPKCYFILVRPRPRTCSLLLGGRT-
>Western_clawed_frog.locus332.1
------
MEWPMREKNQCIAGTEQFLLCNDGISVFFSSISDMSFLTTLLILGAFITYRDSPIVRANNRSLSFLLLVSIKLSFLSVFLFLGRPVDITCMLRIITFGITFSIAVSSLLAKTIMVCVAFKATKPGSSWRKWLGVKLSNSVVLFCSSIQI
IICMTWLAISPPFQELDIHTSPGTIIIQCNEGSAIGFYSVIGYMGLLAAVSFVLAFLARSLPDSFNEAKYITFSMLLFCSVWITMIPAYLSTKGKNTVCVEIFAILTSSAGLLASIFLPKCYIILFRPEMNTKSHLLGNKFS
>Western_clawed_frog.locus1062.1
------
MEWPMREKNQCIARTEQFLLCNDGISVFFSSISDLSFLTTLLILGVFITYQDSPIVRANNRSLSFLLLVSIKLSFLSVFLFLGRPVDITCMLRIITFGITFSIAVSSLLAKTIMVCVAFKATKPGSSWRKWLGVKLSNSVVLFCSSIQI
IICMTWLAISPPFQELDIHTSPGTIIIQCNEGSAIGFYSVIGYMGLLAAVSFVLAFLARSLPDSFNEAKYITFSMLLFCSVWITMIPAYLSTKGKNTVCVEIFAILTSSAGLLASIFLPKCYIILLRPEMNTKSHLLGNRT-
>Western_clawed_frog.locus788.1
------MEWPNERK-----
KSEQFLLCNDGISVFFSSISDLSFLITLLILGAFITYWDSPIVRANNRSLSFLLLVSIKLSFLSVFLFLGRPVDITCMLRIITFGITFSIAVSSLLAKTIMVCVAFKATKPGSSWRKWLGVKLSNSVVLFCSSIQIIICMTWLAISPPF
QELDIHTSPGTIIIQCNEGSAIGFYSVIGYMGLLAAVSFVLAFLARSLPDSFNEAKYITFSMLLFCSVWITMIPAYLSTKGKNTVCVEIFAILTSSAGLLASIFLPKCYIILLRPEMNTKSHLLGNRTY
>Western_clawed_frog.locus158.1
------
MEWPNEKRNQCIARTEQFLSYDDGISVFFSSTSVLIFFITLVILGVFIANRDSPIVRANNRSLSFLLLVSIKLSFLSVFLFLGRPVDITCMLRIITFGITFSIAVSSLLAKTIMVCVAFKATKPGSSWRKWLGVKLSNSVVLFCSSIQI
IICMTWLAISPPFQELDIHTSPGTIIIQCNEGSAIGFYSVIGYMGLLAAVSFVLAFLARSLPDSFNEAKYITFSMLLFCSVWITMIPAYLSTKGKNTVCVEIFAILTSSAGLLASIFLPKCYIILLRPEMNSKSNLLGNKSS
>Western_clawed_frog.locus156.1
------
MEWPLDNKNKFIAGTERFISCDDGISVIFSSTSVLIFFITLGILGVFIANWDSPIVRANNRSLSFLLLVSIKLSFLSVFLFLGRPVDITCMLRIITFGITFSIAVSSLLAKTIMVCVAFKATKPGSSWRKWLGVKLSNSVVLFCSSIQI
IICMTWLAISPPFQELDIHTSPGTIIIQCNEGSAIGFYSVIGYMGLLAAVSFVLAFLARSLPDSFNEAKYITFSMLLFCSVWITMIPAYLSTKGKNTVCVEIFAILTSSAGLLASIFMPPMAKVQNNISFST----------
>Western_clawed_frog.locus502.1
------MEWPNERK-----
KSEQFLSYSDMISVFFSSISVLIFLTTLLILGAFITYRDSPIVRANNRSLSFLLLVSIKLSFLSVFLFLGRPVDITCMLRIITFGITFSIAVSSLLAKTIMVCVAFKATKPGSSWRKWLGVKLSNSVVLFCSSIQIIICMTWLAISPPF
QELDIHTSPGTIIIQCNEGSAIGFYSVIGYMGLLAAVSFVLAFLARSLPDSFNEAKYITFSMLLFCSVWITMIPAYLSTKGKNTVCVEIFAILTSSAGLLASIFLPKCYIILLRPEMNTKSHLLGNKFS
>Western_clawed_frog.locus1028.1
------MEWPNERK-----
KSEQFLSYSDMISVFFSSISVLFFLITLLILGFFITYRDSPIVRANNRSLSFLLLVSIKLSFLSVFLFLGRPVDITCMLRIITFGITFSIAVSSLLAKTIMVCVAFKATKPGSSWRKWLGVKLSNSVVLFCSSIQIIICMTWLAISPPF
QELDIHTSPGTIIIQCNEGSAIGFYSVIGYMGLLAAVSFVLAFLARSLPDSFNEAKYITFSMLLFCSVWITMILAYLSTKGKNTVCVEIFAILTSSAGLLASIFLPKCYIILFRPEMNTKSHLLGNKFS
>Western_clawed_frog.locus536.1
------
MEWPNERKNRILIGTERFLSYSDMISVFFSSISVLIFLITLLILGVFISYRDSPIVRANNRSLSFLLLVSIKLSFLSVFLFLGRPVDITCMLRIITFGITFSIAVSSLLAKTIMVCVAFKATKPGSSWRKWLGVKLSNSAVLFCSSIQI
IICMTWLAISPPFQELDIHTSPGTIIIQCNEGSAIGFYSVIGYMGLLAAVSFVLAFLARSLPDSFNEAKYITFSMLLFCSVWITMIPAYLSTKGKNTVCVEIFAILTSSAGLLASIFLPKCYIILLRPEMNTKSHLLGNKAS
>Western_clawed_frog.locus165.1
------
MEWPNENKNQCIIRNEEFLSYNYMISVFFSSISVLFFLTTLLILGVFITFRNSPIVRANNRSLSFLLLVSIKLSFLSVFLFLGRPVDITCMLRIITFGITFSIAVSSLLAKTIMVCVAFKATKPGSSWRKWLGVKLSNSVVLFCSSIQI
IICMTWLAISPPFQELDIHTSPGTIIIQCNEGSAIGFYSVIGYMGLLAAVSFVLAFLARSLPDSFNEAKYITFSMLLFCSVWITMIPAYLSTKGKNTVCVEIFAILTSSAGLLASIFLPKCYIILLRPEMNMKSVMFGNKTK
>Western_clawed_frog.locus164.1
------
MEWPNENKNQCIIRNEEFLSYNDMISVFFSSISVLFFLTTLLILGVFITFRNSPIVRANNRSLSFLLLVSIKLSFLSVFLFLGRPVDITCMLRIITFGITFSIAVSSLLAKTIMVCVAFKATKPGSSWRKWLGVKLSNSVVLFCSSIQI
IICMTWLAISPPFQELDIHTSPGTIIIQCNEGSAIGFYSVIGYMGLLAAVSFVLAFLARSLPDSFNEAKYITFSMLLFCSVWITMIPAYLSTKGKNTVCVEIFAILTSSAGLLASIFLPKCYIILLKCQMNTKSHLLGNKKY
>Western_clawed_frog.locus38.1
------
MEWPNEQRNQCVAKTEEFLSYNDVISIFLPSLSVVLYLCTVLILAIFIKYRDSPIVRANNRSLSFLLLVSIKLSFLSVFLFLGRPVDITCMLRIITFGITFSIAVSSLLAKTIMVCVAFKATKPGSSWRKWLGVKLSNSVVLFCSSIQI
IICMTWLAISPPFQELDIHTSPGTIIIQCNEGSAIGFYSVIGYMGLLAAVSFVLAFLARSLPDSFNEAKYITFSMLLFCSVWITMIPAYLSTKGKNTVCVEIFAILTSSAGLLASIFLPKCYIILYRPEMNTRTFVFANKI-
>Western_clawed_frog.locus36.1
------
MEWPNEQRNQCIARTEEFLSYNDVISIFLPSLSVVLYLCTVLILAIFIKYRDSPIVRANNRSLSFLLLVSIKLSFLSVFLFLGRPVDITCMLRIITFGITFSIAVSSLLAKTIMVCVAFKATKPGSSWRKWLGVKLSNSVVLFCSSIQI
IICMTWLAISPPFQELDIHTSPGTIIIQCNEGSAIGFYSVIGYMGLLAAVSFVLAFLARSLPDSFNEAKYITFSMLLFCSVWITMIPAYLSTKGKNTVCVEIFAILTSSAGLLASIFLPKCYIILYRPEMNTRTFVFANKI-
>Western_clawed_frog.locus57.1
------
MEWPNEKRNQCVARTEEFLSYDDVISICLLSLSVVLYLCTVLITTCFITYRDSPIVRANNRSLSFLLLVSIKLSFLSVFLFLGRPVDITCMLRIITFGITFSIAVSSLLAKTIMVCVAFKATKPGSSWRKWLGVKLSNSVVLFCSSIQI
IICMTWLAISPPFQELDIHTSPGTIIIQCNEGSAIGFYSVIGYMGLLAAVSFVLAFLARSLPDSFNEAKYITFSMLLFCSVWITMIPAYLSTKGKNTVCVEIFAILTSSAGLLFCIFLPKCYTILLRPEINMKSNLLGNKYK
>Western_clawed_frog.locus55.1
------
MEWPNEKRNQCVARTEEFLSYDDVISICLLSLSVVLYLCTVLITTCFIIYRDTPIVRANNRSLSFLLLVSIKLSFLSVFLFLGRPVDITCMLRIITFGITFSIAVSSLLAKTIMVCVAFKATKPGSSWRKWLGVKLSNSVVLFCSSIQI
IICMTWLAISPPFQELDIHTSPGTIIIQCNEGSAIGFYSVIGYMGLLAAVSFVLAFLARSLPDSFNEAKYITFSMLLFCSVWITMIPAYLSTKGKNTVCVEIFAILTSSAGLLFCIFLPKCYTILLRPEINMKSNLLGNKYK
>Western_clawed_frog.locus64.1
CFRCPDLEWPNEKKNQCIARTEEFLPYNDVISVFVSSFSVLFFLLTVLILGVFITYRDSPIVRANNRSLSFLLLVSIKLSFLSVFLFLGRPVDITCMLRIITFGITFSIAVSSLLAKTIMVCVAFKATKPGSSWRKWLGVKLSNSVVLF
CSSIQIIICMTWLAISPPFQELDIHTSPGTIIIQCNEGSAIGFYSVIGYMGLLAAVSFVLAFLARSLPDSFNEAKYITFSMLLFCSVWITMIPAYLSTKGKNTVCVEIFAILTSSAGLLACIFLPKCYIILFRSKMNTKTHLLKNKS-
>Western_clawed_frog.locus65.1
CFRCPDLEWPNEKKNQCIARTEEFLPYNDVISLFVSSFSVLFFLLTVLILGVFISYRDSPIVRANNRSLSFLLLVSIKLSFLSVFLFLGRPVDITCMLRIITFGITFSIAVSSLLAKTIMVCVAFKATKPGSSWRKWLGVKLSNSVVLF
CSSIQIIICMTWLAISPPFQELDIHTSPGTIIIQCNEGSAIGFYSVIGYMGLLAAVSFVLAFLARSLPDSFNEAKYITFSMLLFCSVWITMIPAYLSTKGKNTVCVEIFAILTSSAGLLACIFLPKCYIIMFRPEMNTKTHLLKNKS-
>Western_clawed_frog.locus63.1
CFRCPDLEWPNEKKNQCIARTEEFLSYNDVISVFFSSFSLLFFLLTVLILGVFFTYRDTPIVRANNRSLSFLLLVSIKLSFLSVFLFLGRPVDITCMLRNISFGITFSIAVSSLLAKTIMVCVAFKATKPGSSWRKWLGVKLSNSVVLF
CSSIQIIICMTWLAISPPFQELDIHTSPGTIIIQCNEGSAIGFYSVIGYMGLLAAVSFVLAFLARSLPDSFNEAKYITFSMLLFCSVWITMIPAYLSTKGKNTVCVEIFAILTSSAGLLASIFLPKCYTFLFTPEINTKTHLFVNKL-
>Western_clawed_frog.locus66.1
------MEWPNRKSTQCTARTEEFLSYK-
IISVIFSSVSVFFFLITLLILGVFISYRDSPIVRANNRSLSFLLLVSIKLSFLSVFLFLGRPVDITCMLRIITFGITFSIAVSSLLAKTIMVCVAFKATKPGSSWRKWLGVKLSNSVVLFCSSIQIIICMTWLAISPPFQELDIHTSPG
TIIIQCNEGSAIGFYSVIGYMGLLAAVSFVLAFLARSLPDSFNEAKYITFSMLLFCSVWITMIPAYLSTKGKNTVCVEIFAILTSSAGLLACIFLPKCYIIMFRPEINTKTHLFGNIS-
>Western_clawed_frog.locus122.1
------
MEWPNENKNQCIARTEQFLSYNDVISIIFSSISVLFFLITLLILGVFISYRDSPIVRANNRSLSFLLLVSIKLSFLSVFLFLGRPVDITCMLRIITFGITFSIAVSSLLAKTIMVCVAFKATKPGSSWRKWLGVKLSNSVVLFCSSIQI
IICMTWLAISPPFQELDIHTSPGTIIIQCNEGSAIGFYSVIGYMGLLAAVSFVLAFLARSLPDSFNEAKYITFSMLLFCSVWITMIPAYLSTKGKNTVCVEIFAILTSSAGLLACIFLPKCYVIMFRPELNTKSCLFEHNSR
>Western_clawed_frog.locus70.1
CYKCPDMEWPNEKRDQCILKSEDFLSYNHEISMLFGSVSLFLSVLTVLIMGIFLKYRDTPIVRANNRSLSFLLLVSIKLSFLSVFLFLGRPVDITCMLRIITFGITFSIAVSSLLAKTIMVCVAFKATKPGSSWRKWLGVKLSNSVVLF
CSSIQIIICMTWLAISPPFQELDIHTSPGTIIIQCNEGSAIGFYSVIGYMGLLAAVSFVLAFLARSLPDSFNEAKYITFSMLLFCSVWITMIPAYLSTKGKNTVCVEIFAILTSSAGLLACIFLPKCYIILITPETNKKFYLTGKTCG
>Western_clawed_frog.locus89.1
-----------------
MTKSEDFLSYNDIISMFFSSISVFFFVITLMILGTFITYRDSPIVRANNRSLSFLLLVSIKLSFLSVFLFLGRPVDITCMLRIITFGITFSIAVSSLLAKTIMVCVAFKATKPGSSWRKWLGVKLSNSVVLFCSSIQIIICMTWLAISP
PFQELDIHTSPGTIIIQCNEGSAIGFYSVIGYMGLLAAVSFVLAFLARSLPDSFNEAKYITFSMLLFCSVWITMIPAYLSTKGKNTVCVEIFAILTSSAGLLACIFLPKCYIIMMRPEMNTKSYVLGNNT-
>Western_clawed_frog.locus139.1
CYKCEDTEWPNQEKTLCIEKQIEFLSYGDPLTLISIISSVILFIIAAVILGIFISFRDTPVVRANNHTLSFILLVSIKLSFLSVFLFLGRPVDITCMLRQTSFGITFSIAVSCVLAKTLMVSIAFKATKPGSPWRKWVGVKLANGLVFI
CSLIQFLISVIWLVIAPPYVEHNTHSEPGKIIIQCNESSVVAFYIVLSYMGLLASVSFIVAFLARSLPDSFNEAKYITFSMLLFCSVWITMIPAYLSTKGKYMVAVEIFAIIS-----------------------------------
>Western_clawed_frog.locus175.1
CFTCEDNEWPNQEKTLCIEKQIEFLSYGDPLTLISIISSVILFIIAAVILGIFISFRDTPVVRANNHTLSFILLVSIKLSFLSVFLFLGRPVDITCMLRQTSFGITFSIAVSCVLAKTLMVSIAFKATKPGSPWRKWVGVKLANGLVFI
CSLIQFLISVIWLVIAPPYVEQNTHSEPGKIIIQCNESSVVAFYVVLSYMGLLASVSFIVAFLARSLPDSFNEAKYITFSMLLFCSVWITMIPAYLSTKGKYMVAVEIFAIISSSCGLLFCIFLPKCYIILFKPELNSKQYLLGKYNT
>Coelacanth.locus21.1
-MICPPDFWSNVQKDFCLPKEVEYLSFMDRMGIVLLTVALIGTGLTFAITAVFARYRNTPIVKANNSELSFLILFSLALCFLCSITFIGQPTMWSCKLRHVAFGIIFAFCISCILGKTIVVLMAFRANLPGNNVMRWFGPRQQRAIIIA
TTFIQIIISTVWMIIAPPLPVKNGKYLNSRIILECDVGSLVAFALVLSYIGLLSCICFVLAFLARNLPDSFNEAKYITFSMLIFFAVWISFIPAYISTAGKYTIAVEIFAILSSNLGLLLCLFAPKCYIILLKPEKNSKKNLMNRFTN
>Coelacanth.locus20.1
---------------------------
MDSMGIVLLTVALIGTGLTFAITAVFARYRNTPIVKANNSELSFLILFSLALCFLCSITFIGQPTMWSCKLRHVAFGIIFAFCISCILGKTIVVLMAFRANLPGNNVMRWFGPRQQRAIIIATTFIQIIISTVWMIIAPPLPVKNGKYL
NSRIILECDVGSLVAFALVLSYIGLLSCICFVLAFLARNLPDSFNEAKYITFSMLIFFAVWISFIPAYISTAGKYTIAVEIFAILSSNFGLLLCLFAPKCYIILLKPEKNSKKSLMNRFTN
>Coelacanth.locus325.1
-MICPPDFWSNVQKDFCLPKEVEYLSFMDSMGIVLLTVALLGTCLTFAITAVFARYRNTPIVKANNSKLSFLILFSLALCFLCSITFIGQPTMWSCKLRHVAFGIIFAFCISCILGKTIVVLMAFRANLPGNNVMRWFGPRQQRAIIIA
TTFIQIIISTVWMIIAPPLPVKNGKYLNSRIILECDVGSLVAFALVLSYIGLLSCICFVLAFLARNLPDSFNEAKYITFSMLIFFAVWISFIPAYISTAGKYTIAVEIFAILSSNFGLLLCLFAPKCYIILLKPEKNSKKSLMNRFTN
>Coelacanth.locus333.1
---------------------
```

```
MEYLSFEDTTGIILLSAALFGICLTLTITVIFAYYRNSPIVKANNSELSFLILFSLALCFLCSVTFIGQPTVWSCMLRHTVFGITFVLSISCILGKTVVVLIAFKAKLPANNMTKWFGPIQQKALILICTGVQIIISVVWLIIAPPLPM
KNIKHQSSRIILECNVGSVVAYSLVLGYIGLLSCICFVLAFLARKLPDNFNEAKFITFSMLIFFAVWLTFIPAYISTPGKYTVAVEIFAILSSSFGLLICIFAPKCYIILLRPEKNTKKQLMGRISS
>Western_clawed_frog.locus782.1
---------------
MCVPKVIEFLSYQEPLGIFLAVTVIIFFLITLSILFIFMKYRGTPIIKATNRELSFILLVSLTLCFLCCLIFIGSPSPMTCPLRQTLFMVVFSISISSVLAKTIMVIVAFKATKMHSPLRKWLGPKIPRTVVALCTTAQVGICGAWLLL
SPPFPQVNAEIEKHKLIRECHEGQSLFFYVTLGFMGFLAMVSFFAAFLARNLPGSYNEAKLITFSMLVFCSVWVSFIPAHLSTKGKYTVSVQIFAILASSAGLLACIFLPKCYIVLLRPDRNSRGRLTSGASR
>Caecillan.locus36862.1
CTTCPEDQWSNPNRDACIPKMITFLSYEELLGIVLILISIVFFLITAVILGIFIYYRDTPIVRANNRNLSYILLISFMLSFLCTLVFIGRPKKVTCLLRQTVFVITFSISLSSVLAKTITVVTAFHATKPGSKLHKWMSSRVSVMIVLC
CSLIQTVLCLAWLFTAPPFPYLNMKSEIGLILIECNEGSIVAFYCVLGYLGFLAGVSFIVAFLARNLPDSFNEAKYITFSMLVFCSVWVSFIPTYLSTKGKYMVAVEIFAILASSAGLLGCIFMPKCYIILLKPDRNSRKYLTKNLNE
>Caecillan.locus36730.1
CITCPEDQWSNPNRDACIPKMITFLSYEELLGIVLILISIVFFLITAVILGIFIYYRDTPIVRANNRNLSYILLISFMLSFLCTLVFIGRPKKVTCLLRQTIFVITFYISLSSVLAKTITVVTAFHATKPGSKFQKWMGSRVSVTIVLC
CSLIQTVLCLAWLFTAPPFPYLNMKSEIGLILIECNEGSIVAFYCVLGYLGFLAGVSFIVAFLARNLPDSFNEAKYITFSMLVFCSVWVSFIPTYLSTKGKYMVAVEIFAILASSAGLLGCIFMPKCYIILLKPDRNSRKYLTKNLNE
>Western_clawed_frog.locus689.1
CIKCSEYEKSNTEQDGCIPKNINFLSYKDILGATLSSITIICAVTCAVILGIFIKYRETPIVRANNRYLSCLLLISLMLCFLCTLLFIGRPTQICCLLRQVTFGVVFTISVSSVLAKTLTVIIAFNATKPGSKLKKYVGTQLAIVLVIV
CSLGEIIISIVWLVSNPPFSETDTLSDPENIILLCNEGSDFFFFCIIGYIGTLALLSFIAAFLAKDFPDRFNEAKNITFSMLGFCSVWGTFVPAYLSSKGSRMVAVEIFAILSSSAGLLGCIFIPKCYIIFIRPELNTKDTIVRKR--
>Western_clawed_frog.locus638.1
CIKCSEYEKSNTEQDGCIPKNINFLSYKDILGATLSSITILCAVTCAVILGIFIKYRETPIVRANNRYLSCLLLISLMLCFLCTLLFIGRPTQICCLLRQVTFGIVFTISVSSVLAKTLTVIIAFNATKPGSKLKKYVGTQLAIILVIV
CSLGEIIISIVWMVSYPPFSETDTLSDPDYIIVQCNEGSGCFFFCIIGYIGSLALLSFIAAFLAKDFPDRFNEAKNITFSMLGFCSVWGAFVPAYLSSKGSRMVAVEIFAILSSSAGLLGCIFIPKCYIIFIRPELNTKDTIVRKR--
>Western_clawed_frog.locus582.1
CERCSEYQKSNTERTECLLKAINFLSYTDTMGASLTAIAFILFITASVVLGIFVKYWETPIVRANNQHLSCLLLISLMLCFLCTLLFIGRPTQICCLLRQVTFGIVFTISVSSVLAKTLTVIIAFNATKPGSKLKKYVGTQLAIVLVII
CSLGSTGISAVWMASHPPFLEADTVSEMDTVILLCNEGSVTFFFCIIGYIGTLALLSFIAAFLAKDFPDRFNEAKNITFSMLGFCSVWGAFVPAYLSSKGSRMVAVEIFAILSSSAGLLACIFAPKCYIIFLQPELNKRTL-------
>Small-eyed_rabbitfish.locus128.1
CLTCPLEYWSIDKRDHCLLKEIEFLSLEETLRIVLVTLALLGVCLTISVFTAFYVHRDTTIVKANNSDLSFLLLVALALCFLCSVTFIVEPSVWSCRLRHMAFGITFVLCISCVLGKTIVVVMAFNATLPSNNMMKWFGPTQQRLSFFI
LTLIQCLICTIWLSLSPPYPLEISEYFTERIVFECHVGSAVAFYCVLGYIGLLSCVCIGVAFLARRLPDNFNEAKHITFSMLIFCAVWITFIPAYVSSPGKYTVAVEIFAILATSFVLLACIFAPKCYIILLKKEENTKKHMMGKMAS
>Reedfish.locus27498.1
------------------------------
MGIALTTLSLSGTCLSICVLTIFIYYRNTPVVKANNSELSFLLLISLTLCFVCALCFIGKPSHVTCMLRHVMFGISFVLCISCILVKTIVVIMAFKATLPGNNIMKWFGAAQQRGTVFFFTFIQSLICIIWLSTAPPFPVRNSKNINTK
IILECDIGSLTGFSCLLGYIGLLACICFLLAFLARNLPDTFNEARFITFSMLIFCAVWITFIPAYISSPGKYTVAVEIFAILASSFGVLLSIFAPKCYIILLKPEENNKRALMGR---
>Reedfish.locus27501.1
------------------------------
MGIALTTLSLSGTCLSICVLTIFIYYRNTPVVKANNSELSFLLLISLTLCFVCALCFIGKPSHVTCMLRHVMFGISFVLCISCILVKTIVVIMAFKATLPGNNIMKWFGAAHQRGTVFFFTFIQSLICIIWLSTAPPFPVRNSKNINTK
IILECEIGSLTGFSCLLGYVGLLACICFLLAFLARNLPDTFNEARFITFSMLIFCAVWITFIPAYISSPGKYTVAVEIFAILASSFGVLLSIFAPKCYIILLKPEENNKRALMGR---
>Reedfish.locus27496.1
------------------------------
MGIALTTLSLSGTCLSICVLTIFIYYRNTPVVKANNSELSFLLLISLTLCFVCALCFIGKPSHVTCMLRHVMFGISFVLCISCILVKTIVVIMAFKATLPGNNIMKWFGATQQRGTVLFFTFIQSLICIIWLSTAPPFPARNSKNFNTK
IILECDIGSLTGFSCLLGYIGLLACICFLLAFLARNLPDTFNEARFITFSMLIFCAVWITFIPAYISSPGKYTVAVEIFAILASSFGVLLSIFAPKCYIILLKPEKNKKRALMGR---
>Reedfish.locus40354.1
CIKCPDDLWSNAGKNQCVLKEIEFLSYDESMGVILTIASLFGASISVCILIIFIHYRNTPVVKANNSELSFLLLISLILCFLCSLSFIGQPSDITCMFRHVMFGISFVLCISCILVKTIVVIMAFKATLPGSNIMKLFGVAQQRGTIFF
LTFIQSMICCIWLVAAPPFPTKNSKYLNTKIILECDIGSLIAFSCTLGYVGLLSCICFLIAFLARNLPDTFNEAKFITFSMLIFCSVWITFIPAYISSPGKHTVAVEIFAILASSFGVTIAIFAPKCYIILLKPERNNKKALMGRVVS
>Reedfish.locus27514.1
------------------
MKEIEFLSYEDSMGLTLTTTALTGVCLSLGILSIFIHYRHTPVVKANNSELSFLLLASLTLCFFCALCFIGQPTNLSCMLRHVMFGISFVLCISCILVKTIVVIMAFKAALPGNNLMKWFGVPQQRGTVFFFTLIQSVICIVWLSTAPP
VPNKNTKIQSVKIIFECDVGSLTGFSCLLGYIGFLAGISFILAFLARNLPDSFNEAKFITFSMLIFCAVWITFIPAYISSPGKYTVAVEIFAILASSFGVLFAIFLPKCYIILIKPEKNNKKALMGKPAT
>Reedfish.locus27524.1
------------------------------
MGITLTTTALSGTCLSIGILMIFIQYRNTPVVKANNSELSFLLLVSLTLCFLCSLCFIGQPSHFTCVFMYAIFGISFVLCISCILVKTLVVIMAFKATLPGSNAMKWFGIAQQRGTVFFFTAIQLLICVIWLSISPPFPSKNTKYQNSK
IILECDIASLFGFSCLLGYIGLLACICFILAFLARKLPDNFNEAKFITFSMLIFCTVWITFIPAYISSPGKYTVAVEVFAILASSFGILFAIFTPKCYIILLKPEQNTKKALMGR---
>Reedfish.locus27417.1
CSRCLPDFWSNQGKNQCVLKEIEFLSYEDVMGIILTTTALLGTCLSVAILAIFIQYRNTPVVKANNSELSFLLLVSLKLCFLCALCFIGQPSQLTCILRHLMFGISFALCISCILVKTIVVIMAFKATLPGNNIMKWFGVSQQRGTVFF
FTLIQAVICLIWFCTSPPGPAKNIKYQNSKIILECDVGSQTGFSCLLGYIGLLACVCFLSAFLARNLPDTFNEAKFITFSMLIFCAVWITFIPAYISTPGKYTVAVEIFAILASAFGILLAIFTPKCYVILLKPERNTKKALMEKSIA
>Reedfish.locus27517.1
CIQCPRDQWSNPGRNCCLLKEVEFLSYSDAMGITLTTAALFGASFSCMVLAIFIHHRYTPVVKANNSEMSFVLLVSLTLCFLCSLCFIGQPSNFTCIIRHVVFGISFVVCVSSILVKTIVVLMAFTATLPGNNTMKWFGVMQQRCTIFF
LTCVQSLICTFWLTTAPPFPTQNTLSQNAKILLECNIGSSIGFSCLLGYIGLLACICFFLAFLARNLPDTFNEAKFITFSMLIFCTVWITFIPAYISSPGKYTVAVEVFAILASSFGLLFLIFAPKCYIIILKPKSNSRKALMGRSNV
>Reedfish.locus27515.1
------------------------------
MGITLSTTALSGASFSVAVLVIFMQYRYTPVVKANNSELSFLLLTSLTLCFLCALCFIGQPSYLTCMLRHVVFGISFVLCISCILVKTIVVIMAFRATMPGNNVMKWFGTAQQRGTVFLFTFIQSLICIIWLMVAPPIPARNTKYQNAK
VILECDVGSLTGFSCLLGYIGLLACICFILAFLARNLPDTFNETKFITFSMLIFCAVWITFIPAYISSPGKYTVAVEIFAILASSFGLLFAIFAPKCYIIILKPERNSKRALMSKSAL
>Reedfish.locus27510.1
CIKCTSEFWPNSGRNECVLKEIEFLSYEDSMGITLTTTALSGACLSIGVLAVFIHYRNTPVVKANNSELSFLLLVSLILCFLCALCFIGQPSHLTCMIRHVLFGISFVLSVSCILVKTIVVIMAFKSTLPGHNIMKWFGTSQQRGTVFF
FTFIQSLICIIWLTIAPPIPAKNSKYQNSKIIFECDIGSIAGFACLMGYIGLLTCISFLLAFLARNLPDTFNEAKFITFSMLIFCAVWITFIPAYLSTPGKYTVAVEIFAILASSFGLLFSIFAPKCYIILIRPELNTKKALMGRGES
>Reedfish.locus40336.1
CIRCLPEFWSNRDRSQCVLKEIEFLSYDDDMGITLTATACFGACLSLGVLSVFLHYRNTPVVKANNSELSFLLLVSLTLCFLCALCFIGEPTYVTCMLRHVVFGVSFALCISCILVKTIVVIIAFKATLPGNNLMKWFGVAQQRGTVFI
LAFIQALICLMWLTTAPPFPTKNIKYQNSKVIFECDLGSVTGFSCLLGYIGLLAVICFMIAFLARNLPDNFNEAKFITFSMLIFCAVWITFIPAYISSPGKHTVAVEIFAILASSFGVLIAIFAPKCYIILIKPELNTKKALMNRGAP
>Reedfish.locus40342.1
CIRCLPEFWSNRDRSQCVLKEIEFLSYDDDMGITLTATACFGACLSLGVLSVFLHYRNTPVVKANNSELSFLLLVSLTLCFLCALCFIGEPTYVTCMLRHVVFGVSFALCISCILVKTIVVIIAFKATLPGNNLMKWFGVSQQRGIVFI
LAFIQALICLMWLTTAPPFPTKNIKYQNSKVIFECDLGSVTGFSCLLGYIGLLAAICFMIAFFARNLPDNFNEAKFIPFSMLIFCAVWITFIPAYISSPGKLTVAVEIFAILASSFGVLIAIFAPKCYIILIKPELNTKKALMNRGAP
>Reedfish.locus40347.1
CIRCLTEFWSNRDRSQCVLKEIEFLSYDDAMGITLTATACFGACLSLGVLSVFLHYRNTPVVKANNSELSFLLLVSLTLCFLCALCFIGEPTYVTCMLRHVVFGVSFALCISCILVKTIVVIIAFKATLPGNNLMKWFGIAQQRGTVFI
LAFIQALICLMWLTTAPPFPTKNTKYQNSKVIFECDLGSVTGFSCLLGYIGLLAAICFMIAFLARNLPDNFNEAKFITFSMLIFCAVWLTFIPAYISSPGKHTVAVEIFAILASSFGVLIAIFAPKCYIILIKPELNTKKALMNRGAP
>Reedfish.locus27454.1
CIKCPSDFWSNFNKTKCVLKEVDFLSFTEVIGIILTVSSGCGVCLCLSTIAVFIHYRTSPLVKANNLELSFILLISLTLCFLCALGFIGQPTQLTCIVRHVVFGISFALCVSCILVKTIVVIMAFKAVVPSNKILKWFGAAQQRGAVLM
LTLFQTLICIVWFTTAPPFPYKNTKYEKFKVILECDVGSVVGFSCLLGYIGLLAGVCFALAFLARNLPDNFNEARLITFSMLIFCAVWITFIPTYIGSPGKYAVIVEVFAILTSSFGLLIIKFSPRCYVILFRADLNTKKAIMGRGRP
>Reedfish.locus27452.1
------------------------------
MGIVLTIILLSGAFLSLAALAVFLYYRNTPVVKANNSGLSFLLLVSLTLCFLCALCFIGQPSGLTCMLRHAVFGLSFVLSVSCILVKTIVVVMAFKATMPGGNVVKWFGVTQQKGTVFFFTSIQCVICVAWLTTAPPFPNKNTKYQTLK
IIFECDVGSLTGFSCLLGYIGLLAGACCLLASLARNLPDNFNEAKYITFSMLIFCVVWITFIPAYISSPGKFTVAVEVFAILASSFGILFAIFAPKCYIILLKPELNSKKALIRRQFP
>Reedfish.locus27461.1
------------------------------
MGITLTTVSLFGVCLSICVLVVFIIYRNTPVVKANNSELSFLLLVSLTFCFLCALCFIGQPTDLTCILRHIVFGISFVLCVSCILVKTIVVIVAFKATLPGNNMMKWFGVSQQRGTIFLFTLIQSIICIIWLSTAPPSPVKNTKYQNEK
IIFECDNGSIVGFSSVLGYIGLLACTCFAIAFLARNLPDTFNEAKFITFSMLLFCAVWISFIPAYISSPGKYTVAVEIFAILASSFGVLFAIFSPKCFIILLKPELNNKKSLMGKLAS
>Reedfish.locus27464.1
------------------------------
MGITLTTVSLFGVCLSICVLVVFIIYRNTPVVKANNSELSFLLLVSLTFCFLCALCFIGQPTDLTCILRHIVFGISFVLCVSCILVKTIVVIVAFKATLPGNNMMKWFGVSQQRGTIFLFTLIQSIICIIWLSTAPPSPVKNTKYQNEK
IIFECDNGSIVGFSSVLGYIGLLACTCFAIAFLSRNLPDTFNEAKFITFSMLLFCAVWISFIPAYISSPGKYTVAVEVFAILASSFGLLLAIFSPKCFIILLKPELNNKKSILGKVNP
>Reedfish.locus27456.1
------------------------------
MGITLSTVSAFGACLSLGVLAVFIYYRNTPVVKANNSELSFLLLVSLTFCFLCALCFIGQPSHLTCMLRHVVFGISFVLCISCILVKTIVVIMAFKSTLPGTNLMKWFGVAQQRGTVVFFTLIQSIICIIWLSTNPPGPLKNTKYQNGK
IIFECNIGSVTGFSSVLGYIGLLACICFLSAFLARKLPDNFNEAKFITFSMLIFCAVWITFIPAYVSSPGKYTVAVEVFAILASSYGILFAIFAPKCYIILFKPEKNTKKALMGRADP
>Reedfish.locus40375.1
------------------------------
MGMTLAVIALLGACMSLFVLVIFMQYRSTPIVKANNSELSFLLLISLTFSFLSALCFIGQPSHWTCMLRHVLLGISLVVCISCILVKTIVVIMAFKATLPGNNMMKWFGVAQQRGTIICFTLIQALICTIWLSTTPPFPLRNTKYQNDK
IIFECSIGSLIGFGCLLGYVGFLACISFLLAFLARHLPDTFNEAKFITFSMLIFCAVWITFIPAYISSPGKYTVAVEIFAILASSFGLLFAIFAPKCYIILLKPELNTKKAMMGR---
>Mouse.locus248.1
CVQCPESHYANSEKNHCLQKSVSFLAYEEPLGMGLTITALDFSVLTAVVLVVFVKHRDTPIVKANNRVLSYTLLLTLIICFLSCLLYIRQPNTATCILQQTAFGTLFTVALSTVLAKAIVVVTAFKITSPARMVRWLIVSGAPNSIIPI
CTLIQLIIYGIWLATSPPFIDQNAHVEHGHIIIMCNKGSAVAFHCVLGYLCFLALGSYSMAFLSRNLPDTFNEAKYLSFSMQVFFCVWITFLPVYHSTKGKVMVAMEVFSVLASSIALLALIFAPKCYIILLRPDKNSRLDIRHKIHS
>Zebrafish.locus480.1
------------------------------
MGIVLVFFSLFGAGLTVLVAILFYSKKDTPIVKANNSELSFLLLFSLTLCFLCSLTFIGQPTQWSCMLRHTAFGITFVLCISCVLGKTLLVLMAFKATLPGSNIMKWFGPVQQRLSVLAFTFIQVLICVLWLSISPPFPHKNMKYYEEK
IILECSLGSTIGFWAILGYIGLLAALCFILAFLARTLPDNFNEAKFITFSMLIFCAVWITFIPAYVSSPGKFTVAVEIFAILSSSISLLLCIFAPKCYIILLKPEQNTKQYMLGKTTS
>Western_clawed_frog.locus661.1
CTKCSKYEKSNSGRNSCIPRDINCLSYGDQLGSTFSSISVILSITCAVILGIFIKYRETPIVRANNRYLSCLLLISLVLCFLCTLLFIGRPTQICCLLRQVTFGVVFTISVSSVLAKTLTVIIAFNATKPGSKLKKYVGTQLSIILVIV
CCLGEIIISIVWLVSNPPYPEADTLSDADTIIVLCNEGSGCFFFCIIGYIGMLALLSFIAAFLAKDFPDRFNEAKNITFSMLGFCSVWGAFVPAYLSSKGSRMVAVEIFAILSSSAGLLGCIFIPKCYIIFLRPELNTKAN--RII--
>Western_clawed_frog.locus658.1
CTKCSKYEKSNSGRTSCIPRDINYLSYDDHLGSTLSSISVILSITCAVILGIFIKYRETPIVRANNRYLSCLLLISLMLCFLCTLLFIGRPTQICCLLRQVTFGVVFTISVSSVLAKTLTVIIAFNATKPGSKLKKYVGTQLAIILVIV
CCLGEIIISIVWLASNPPFPEDDTLSDADIIILQCNEGSGSFFFCIIGYIGTLALLSFIAAFLAKDFPDRFNEAKNITFSMLGFCSVWGAFVPAYLSSKGSSMLAVEIFAILSSSAGLLGCIFIPKCYMLFLRPELNTKAN--RII--
>Western_clawed_frog.locus660.1
--------------------
MNYLSYDEHLGSTFSSISVILSITCAVILGIFIKYRETPIVRANNRYLSCLLLISLMLCFLCTLLFIGRPTQICCLLRQVTFGVVFTISVSSVLAKTLTVIIAFNATKPGSKLKKYVGTQLAIILVNVCSLGEIIISIVWLASSPPFPE
DEILSDADTIILLCNEGSGCFFFCIIGYIGTLALLSFIAAFLAKDFPDRFNEAKNITFSMLGFCSVWGAFVPAYLSSKGSRMVAVEIFAILSSSAGLLGCIFIPKCYIIFLRPELNTKAN--RII--
>Western_clawed_frog.locus750.1
--------------------
MNYISYDDHLGSTFSSISVILSVTCAVILGIFIKYRETPIVRANNRYLSCLLLISLMLCFLCTLLFIGRPTQICCLLRQVTFGIVFTISVSSVLAKTLTVIIAFNATKPGSKLKKYVGTQLAIILVIVCSLGEIIISIVWLASNPPFPE
DEILSDANTIILLCNEGSGCFFFCIIGYIGTLALLSFIAAFLAKDFPDRFNEAKNITFSMLGFCSVWGAFVPAYLSSKGSRMVAVEIFAILSSSAGLLGCIFIPKCYIIFLRPELNTKAN--RII--
>Caecillan.locus40469.1
CIKCPANQWSNQKRDTCIPKRIIFLSYEEPLGISMTIISMFLCFISVVILGIFIKYQHTPIVKANNRSLSYILLISLMLCFLCSLLFIGHPDKLTCILRQITFGITFSISLSSVLAKTITVVIAFQATKPKSKLRNWMGSRVSNSIVLC
CSLLQTLVCLVWMCTAPPFPYHNSESEIGTIIIECNEGSIIAFYSVIGYLGLLAGISFVVAFFARNLPDTFNEAKHITFSMVVFCSVWVAFIPTYLSTKGKYMVVVEIFSMLASSAGLLACIFIPKCYRIILRPEENNKKYLTRHVKT
>Caecillan.locus40329.1
CIKCPENQWSNQKRDACIPKRIIFLSYEEPLGISMTIISMFLCFISVVILGIFIKYQHTPIVKANNRSLSYILLISLMLCFLCSLIFIGHPDKLTCILRQITFGITFSIALSSVLAKTITVVIAFQATKPKSKLRNWMGSSLSNSIVLC
```

```
CSLLQTLVCLVWMCTAPPFPYHNSESEIGTIIIECNEGSIIAFYSVIGYLGLLAGISFVVAFFARNLPDTFNEAKHITFSMVVFCSVWVAFIPTYLSTRGKYMVAVEIFSMLASSAGLLGYIFIHKCYIILLRPETNNKKYLTKHVKT
>Caecillan.locus40454.1
CIRCPEDQWPNEKRDVCVPKIISYLAHEEILGIILTLISIFFFFITAVILGIFIYYQDTPIVKSNNRNLSYILLVSLMLCFLCSLIFIGHPDEVTCVLRQTAFGLTFSISLSSVLAKTITVVMAFQSTKPGSKFRKWMGSRVSYSIVLS
CSFLQVLLCLVWLGVAPPFPHANMQAEDGTIQIECNEGSAIAFYFVLGYLGFLAGISFIVAFLARNLPGSFNEAKHITFSMLVFCSVWVSFIPTYLSTRGKYMVAVEIFAILASCSGILGCIFIPKCYIILLKPDRNNRKYITKK---
>Reedfish.locus27670.1
------------------------------
MGILLVAFAILGAAFTIAIAVIFFYFRDTPIVRANNSELSFLLLFSLTLCFLCSITFIGEPSDWSCMLRHTAFGITFVLCISCVLGKTVVVLMAFRATLPGNNIMKWFGPTQQRLSVLFFTFIQCIICFLWLITSPPFP--
NMAYYVEKIILECNLGSITAFYSVLGYIGLLSAMCFALAFLARTLPDNFNEAKYITFSMLIFCAVWITFIPAYISSPGKYTVAVEIFAILASSFSLLFCIFAPKCYIILLKPERNTKKYMMGKTT-
>Caecillan.locus36963.1
CTICPEDQWPNKKRDACIPKVIIFLSYEEPLGIALTLISIFFFLITAAILGIFIYYRDTPIVRANNRDLSYILLISLMLCFLCSLIFIGQPNEASCILRHTTFGITFSIALSSILAKTITVVTAFQATKPGSKLRKWMGSRVSNSIVLS
CSLIQTILCLAWLFTAPPFPYLNMRSEIGTILIECNEGSVIAFYCVLGYLGFLSGVSFIIAFLARNLPDSFNEAKYITFSMLVFCSVWISFIPTYLSTKNKYMVAVEIFALLASSAGLLGCIFMPKCYIILLRPDMNIRKYLTKN---
>Japanese_eel.locus113.1
------------------------------
MGLTLTVIAVVGACLTVAVLAVFLYHRNTPIVKVNNSELSFFILLSLTLCFLCALVFIGEPTSWSCMLRHTAFSITFSLCISCILGKTLVVLAAFTATRPGNNLMKWLGPKQQRLIISVCTFIQVIICAVWLITAPPFPNKNSQYHRSR
IILECSVGSEQAFWCVLGYIGLLAALCFILAFLARKLPGNFNEAKYITFSMLIFCAVWLAFVPAYVSSPGKFTVAVEIFAILASSFGLLLCLFTPKCYIILLKPQKNTKQHLMGKD--
>Japanese_eel.locus128.1
------------------------------
MGLTLTVIAVVGACLTIAVLAVFLYHRNTPIVKVNNSELSFFILLSLTLCFLCALVFIGEPTSWSCMLRHTAFSITFSLCISCILGKTLVVLAAFTATRPGNNLMKWLGPKQQRFIISACTFIQVIICAVWLITAPPFPNKNNQYHRSR
IILECSVGSEQAFWCVLGYIGLLAALCFILAFLARKLPGNFNEAKYITFSMLIFCAVWLAFVPAYISSPGKFTVAVEIFAILASSFGLLLCLFAPKCYIILLKPQKNTKQHLMGKVVT
>Reedfish.locus40503.1
------------------------------
MGILLVTLSVLGALLTLLVGLVFYCHRETPIVRANNSELSFLLLFSLTLCFLCSLTFIGQPSDWSCMLRHTAFGIAFVMCISCVLGKTIVVLMAFRSTLPGSNVMKWFGVTQQRLSVCSFTLIQVIICLLWLLTSPPFPSKNMKHYTEI
VILECDLGSTAAFSAVLGYIGLLALFCFVLAFLARKLPDNFNEAKFITFSILIFCAIWITFIPAYISSPGKYTVAVEIFAILASSFGLLFCIFFPKCYIILLKPEKNTKKSIMAK---
>Reedfish.locus40509.1
------------------------------
MGILLVTLSVLGALLTLSICLVFYRHRETPIVRANNSELSFLLLFSLTLCFLCSLTFIGQPSDWSCMLRHTAFGVAFVMCISCVLGKTIVVLMAFRSTLPGSNVMKWFGVTQQRLSVCSFTLIQVIICFLWLLTSPPFPSKNMKHYTEI
IILECNLGSTAAFSAVLGYIGLLALFCFVLAFLARKLPDNFNEAKFITFSILIFCAVWITFIPAYISSPGKYTVAVEIFAILASSFGLLFCIFFPKCYIILLKPEKNTKKSIMAK---
>Reedfish.locus40499.1
------------------------------
MGILLVTLSVLGALLTLLVGLVFYYHRETPIVRANNSELSFLLLFSLTLCFLCSLTFIGQPSDWSCMLRHTAFGIAFVMCISCVLGKTIVVLMAFRSTLPGSNVMKWFGVTQQRLSVCSFTLIQVIICLLWLLSSPPFPNKNMKHYTEI
IILECDLGSTAAFYAVLGYIGLLALFCFVLAFLARKLPDNFNEAKFITFSILIFCAVWITFIPAYISSPGKYTVAVEIFAILASSFGLLFCIFFPKCYIILLKPEKNTKKQIMAK---
>Reedfish.locus40501.1
------------------------------
MGILLVTLSVLGALLTLLVGLVFYYHRETPIVRANNSELSFLLLFSLTLCFLCSLTFIGQPSDWSCMLRHTAFGIAFVMCISCVLGKTIVVLMAFRSTLPGSNVMKWFGVTQQRLSVCSFTLIQVIICLLWLLNSPPFPNKNMKHYTEI
IILECDLGSTAAFYAVLGYIGLLALFCFVLAFLARKLPDNFNEAKFITFSILIFCAVWITFIPAYISSPGKYTVAVEIFAILASSFGLLFCIFFPKCYIILLKPEKNTKKQIMAK---
>Reedfish.locus40506.1
CLKCPVEYKPNKQKDECILKEIEYLSFQEIMGILLVTLSVLGALLTLLVGLVFYYHRETPIVRANNSELSFLLLFSLTLCFLCSLTFIGEPSDWSCMLRHTAFGIAFVMCISCVLGKTIVVLMAFRSTLPGSNVMKWFGVTQQRLSVCS
FTLIQVIICLLWLLNSPPFPNKNIKHYTEIIILECDLGSTAAFYAVLGYIGLLALFCFVLAFLARKLPDNFNEAKFITFSILIFCAVWITFIPAYISSPGKYTVAVEIFAILASSFGLLFCIFFPKCYIILLKPERNTKKNIMAK---
>Reedfish.locus40497.1
CLRCLPEYRSNKGNDNCIMKEIEYLSFQEIMGILLATLSLLGVFLTGFVSFVFFIKRNTPIVRANNSELSFLLLFSLTLCFLCSLTFIGEPSDWSCMLRHTAFGIAFVLCISCILGKTIVVLIAFRATLPGSNVMKWFGAPQQRLSVCS
FTLIQVIICIVWLMKSPPIPFKNMKHYVDIVILECDLGSVAAFSAVLGYIGFLALFCFILAFLARHLPDNFNEAKFITFSMLIFCAVWITFIPAYISSPGKYTVAVEIFAILASSFGLLFCIFIPKCYIILLKPEQNTKKHIMGKQHE
>Reedfish.locus27408.1
------------------------------
MGITLTTTALLGAFISVAVFAIFLFFRNTPVVKANNSELSFLLLLSLTLCFLCSLCFIGKPTDVTCMLRHVMFGISFVLCISCILVKTIVVIMAFKATLPGNNIMKWFGVSKQRSTVLLFTLLQSLICLIWLITAPPAPSKNTKYQNLK
MILECDVGSVVGFACLLGYIGFLACLCFLLAFLARNLPDTFNEAKFITFSMLIFCAVWITFIPAYISSPGKYTVAVEVFAILASSFGLLVAIFAPKCYIILFKPEKNAKKALLGRSA-
>Caecillan.locus40537.1
CTKCPEDQWPNQKRDACIPKVIIFLSYEEPLGLTLTFICLFLFLINVVILGLFIHYKDTPIVKANNRELSYILLISLMLCFLCSLVFIGHPDKLTCILRQTAFGIMFSIALSSILAKTTTVVMAFHATKPGSKLRKWMGTRISVSIVLS
CSLIQTAVCLVWLSTAPPFPYLNMKSEIGTILSECNEGSIVAFYCVLGYLGFLAGISFIVAFLARNLPDRFNEAKYITFSMLVFCNVWISFIPTYLSTKGKYMVAVEVFAILASSAGLLGCIFFPKCYIILLRPEQKSGKYL------
>Caecillan.locus40539.1
CTKCPEDQWPNQKRDACIPKVIIFLSYEEPLGLTLTFICLFLFLINVVILGLFIHYKDTPIVKANNRELSYILLTSLMLCFLCSLVFIGHPDKLTCILRQTAFGIMFSIALSSILAKTITVVMAFHATKPGSKLRKWMGTRISVSIVLS
CSLIQTAVCLVWLSTAPPFPYLNMKSEIGTILSECNEGSIVAFYCVLGYLGFLAGISFIVAFLARNLPDRFNEAKYITFSMLVFCNVWISFIPTYLSTKGKYMVAVEVFAILASSAGLLGCIFFPKCYIILLRPEQKNGKYL------
>Caecillan.locus40538.1
CTKCPEDQWPNQKSDACIPKVIIFLSYEEPLGLTLTFICLFLFLINVVILGLFIHYKDTPIVKANNRELSYILLISLMLCFLCSLVFIGHPDKLTCILRQTAFGIMFSIALSSILAKTITVVMAFHATKPGSKLRKWMGTRISVSIVLS
CSLIQTAVCLVWLSTAPPFPYLNMRSEIGTILIECNEGSIVAFYCVLAYLGFLAGISFIVAFLARNLPDRFNEAKYITFSMLVFCNVWISFIPTYLSTKGKYMVAVEVFAILASSAGLLGCIFFPKCYIILLRPEQKSGKYL------
>Caecillan.locus40540.1
CTKCPEDQWPNQKRDACILKMISFLTFKEPLGLTLTFICLFLFLINVVILGLFIHYKDTPIVKANNQALSYILLISLMLCFLCSLVFIGHPDKLTCILRQTAFGIMFSIALSSILTKTITVIMAFHATKPGSKLRKWMGTRVSVSIVLS
CSLIQTAVCLVWLSTAPPFPYPNMKSEIGTILSECNEGSIVAFYCVLGYLGFLAGISFIVAFLARNLPDRFNEAKYITFSMLVFCNVWISFIPTYLSTKGKYMVAVEVFAILASTAGLLGCIFFPKCYVILLRPERNSRKALIL----
>Caecillan.locus40534.1
CTKCPEDQWPNQKRDACIPKVIVFLSYEEPFGLTLTFICLFLLLINVVILGLFIHYKDTPIVKANNQELSYILLISLMLCFLCSLVFIGHPDKLTCILRQTAFGIMFSIALSSILAKTITVVMAFHATKPGSKLRKWMGTRISVSIVLS
CSLIQTAVCLVWLSTAPPFPYLNMKSEIGTILSECNEGSIVAFYCVLGYLGFLAGISFIVAFLARNLPDRFNEAKYITFSMLVFCNVWISFIPTYLSTKGKYMVAVEVFAILASSAGLLGCIFFPKCYVILLRPEKNSRKPL------
>Caecillan.locus40533.1
CTKCPEDQWPNQKRDACIPKVIIFLSYEEPLGLTLTFICLFLFLINVVILGLFIHYKDTPIVKANNRELSYILLISLMLCFLCSLVFIGHPDKLTCILRQTTFGIMFSIALSSILAKTITVVMAFHATKPGSKLRKWMGSRISVSIVLS
CTLIQTAVCLVWLSTAPPFPYLNMGSEIGTILSECNEGSIVAFYCVLGYLGFLAGISFIVAFLARNLPDRFNEAKYITFSMLVFCNVWISFIPTYLSTKGKYMVAVEVFAILASSAGLLGCIFFPKCYVILLRPERNSRKPNMKKINY
>Caecillan.locus40531.1
CTKCPEDQWPNQKRDACIPKVIIFLSYEEPLGLTLTFICLFLFLINVVILGLFIHYKDTPIVKANNRELSYILLISLMLCFLCSLVFIGHPDKLTCILRQTTFGIMFSIALSSILAKTITVVMAFHATKPGSKLRKWMGSRISVSIVLS
CTLIQTAVCLVWLSTAPPFPYLNMGSEIGTILSECNEGSIVAFYCVLGYLGFLAGISFIVAFLARNLPDRFNEAKYITFSMLVFCNVWISFIPTYLSTKGKYMVAVEVFAILASSAGLLGCIFFPKCYVILLRPERNSRKHI------
>Caecillan.locus40527.1
CTKCPEDQWPNQKRDACIPKVIIFLSYEEPVGLTLTFICLFLFLINVVILGLFIHYKDTPIVKANNRELSYILLISLMLCFLCSLVFIGHPDKLTCILRQIAFGIMFSIALSSILAKTITVVLAFHATKPGSKLRKWMGTRISVSIVLS
CSLIQTAVCLVWLSTVPPFPYLNMRSEIGTILIECNEGSIVAFYCVLGYLGFLAGISFIVAFLARNLPDRFNEAKYITFSMLVFCNVWISFIPTYLSTKGKYMVAVEVFAILASSAGLLGCIFFPKCYIILLRPERNSRKRI------
>Caecillan.locus40520.1
CTKCPEDQWPNQKRDACIPKVIVFLSYDEPVGLTLTFICLFLFLINVVILGLFIHYKDTPIVKANNRELSYILLISLMLCFLCSMVFIGHPDKLTCILRQTAFGIMFSISLSSILAKTITVVLAFHATKPGSKLRKWMGTRISVSIVLS
CSLIQTAVCLVWLSTVPPFPYLNMRSEIGTIVSECNEGSIVAFYCVLGYLGFLAGISFIVAFLARNLPDRFNEAKYITFSMLVFCNVWISFIPTYLSTKGKYTVAVEVFAILASSAGLLGCIFFPKCYIILLRPERNSRKR-------
>Caecillan.locus40530.1
CTKCPDDQWPNQKRDACIPKVIIFLSYEETLGLTLTFICLFLFLINVVILGLFVHYKDTPIVKSNNRELSNILLISLMLCFLCSLVFIGHPDKLTCILRQTAFGIMFSIALSSILAKTITVVLAFHATKPGSKLQKWMGTRISVSIVLF
FSLIQTAVCLVWLSIAPPFPYLNMKSEIGTILSECNEGSIVAFYCVLGYLGFLAGISFIVAFLARNLPDRFNEAKYITFSMLVFCNVWISFIPTYLSTKGKYMVAVEVFAILASSAGLLGCIFFPKCYIILIRPERNSRKRI------
>Caecillan.locus40528.1
CTKCPEDQWPNQKRDACIPKVIIFLSYEETLGLTLTFICLFLFLINVVILGLFIHYKDTPIVKANNRELSYILLISLMLCFLCSLVFIGNPDKLTCILRQTAFGIIFSIALSSILIKTITVVLAFHATKPGSKLQKWMGTRISVSIVLF
CSLIQTAVCLVWLSIAPPFPYLNMKSEIGTILSECNEGSIVAFYCVLGYLGFLAGISFIVAFLARNLPDRFNEAKYITFSMLVFCNVWISFIPTYLSTKGKYMVAVEVFAILASSAGLLGCIFFPKCYIILIRPERNSRKRI------
>Caecillan.locus40522.1
CTKCPEDQWPNQKRDACIPKVIVFLSYDEPVGLTLTFICLFLFLINVVILGLFIHYKDTPIVKANNRELSYILLISLMLCFLCSLVFIGHPDKLTCILRQTAFGIMFSIALSSILAKTITVVLAFHTTKPGSKLRKWMGTRISVSIVLS
CSLIQTAVCLAWLFIAPPFPYLNMRSEIGTILIECNEGSIVAFYCVLGYLGFLAGISFIVAFLARNLPDRFNEAKYITFSMLVFCNVWISFIPTYLSTKGKYMVAVEVFAILASSAGLLGCIFFPKCYIILLRPERNSRKRI------
>Caecillan.locus40523.1
CTKCPEDQWPNQKRDACIPKVITFLSYEEPLGIMLTFICLFLFFINTVILGIFIHYRDTPIVKANNRELSYILLISLMLCFLCSLVFIGHPDKFTCILRQTAFGITFSIALSSILAKTVTVVLAFHATKPGSKLRKWMGSRTSVSIVLS
CSLIQTAVCLAWLFIAPPFPHLNMRSEIGTIVSECNEGSIVAFYCVLAYLGFLAGISFIVAFLARNLPDRFNEAKYITFSMLVFCNVWISFIPTYLSTKGKYMVAVEVFAILASSAGLLGCIFFPKCYIILLRPERNSRKCL------
>Caecillan.locus40525.1
CTKCPEDQWPNQKRDACIPKVIVFLSYDEPLGIMLTFICLFLFFINTVILGIFIHYKDTPIVKANNRELSYILLISLMLCFLCSLVFIGHPDKLTCILRQTAFGITFSIALSTILAKTITVVLAFHATKPGSKLRKWMGSRTSVSIVLS
CSLIQTAVCLAWLFIAPPFPYLNMRSEIGTIVSECNEGSIVAFYCVLGYLGFLAGISFIVAFLARNLPDRFNEAKYITFSMLVFCNVWISFIPTYLSTKGKYMVAVEVFAILASSAGLLGCIFFPKCYIILLRPERNSRKWL------
>Caecillan.locus40519.1
CTKCPEDQWPNQKRDACIPKVIIFLSYEEPLGLMLTFICLFLFLINVVILGLFIHYKDTPIVKANNRELSYILLISLMLCFLCSLVFIGHPDKLTCILRQTAFGITFSIALSSILAKTVTVVLAFHATKPGSKLRKWMGSRISVSIVLS
CSLIQTAVCLAWLFIAPPFPYLNMRSEIGTIVSECNEGSIVAFYCVLGYLGFLAGISFIVAFLARNLPDRFNEAKYITFSMLVFCNVWISFIPTYLSTKGKYMVAVEVFAILASSAGLLGCIFFPKCYVILLRPERNSRKR-------
>Caecillan.locus40518.1
CTKCPEDQWPNQKRDACIPKVITFLSYEEPLGLMLTFICLFLFLINVVILGIFIHYKDTPIVKANNQELSYILLISLMLCFLCSLVFIGHPNKLTCILRQTAFGIMFSIALSSILAKTITVVLAFHATKPGSKLQKWMGSRVSLSIVLF
CSFIQTAVCLAWLFIAPPFPYLNMRSEFGTILSECNEGSIVAFYCVLGYLGFLAGISFIVAFLARNLPDRFNEAKYITFSMLVFCNVWISFIPTYLSTKDKYMVAVEVFAILASSAGLLGCIFFPKCYIILLRPEQNCRKYL------
>Caecillan.locus40517.1
CTKCPEDQWPNQKRDACIPKVITFLSYEEPLGLTLTFICLFLFLINVVILAIFIHYKDTPIVKANNRELSYILLISLMLCFLCSLVFIGHPDKLTCILRQTAFGIMFSIALSSILAKTITVVLAFHATKPGSKLQKWMGSRISVSIILS
CSLIQTAVCLAWLFIAPPFPYLNMRSEFGTILSECNEGSIVAFYCVLGYLGILAGISFIVAFLARNLPDRFNEAKYITFSMLVLYNVWISFIPTYLSTKGKYMVAVEIFAILASSAGLLGCIFFPKCYIILLRPERNCRKYL------
>Caecillan.locus40526.1
CTKCPEDQCPNQKREACIPKEITFLSYEEPLRLMLTFICLFLFLINIVILAIFIHYKDTPIVKANNRELSYILLISLMLCFLCSLVFIGHPDKLTCILRQTGFGIMFSIALSSILAKTITVVLAFHVTKPGSKLQRWMGSRISVSIVLS
CSFIQTAVCFAWLFIAPPFPYLNMRSEIGTILSECNEGSIVAFYCVLGYLGFLAGISFIVAFLARNLPDRFNEAKYITFSMLVFCNVWISFIPTYLSTKVKYMVAVEVFAILASSAGLLGCIFFPKCYIILLRRERNNRK--------
>Caecillan.locus40524.1
CTKCPEEQWPNKKRDSCIPKVITFLSYEEPVGLTLTFICLFLFLINTIILGIFIHYKDTPIVKANNRELSYILLISLMLCFLCSLLFICHPDKLTCILRQTAFGIIFSIALSSILAKTITVVLAFHATKPGSKLRKWMGTRISVSIVLS
CSFIQTAVCFAWLFIAPPFPYLNMRSEIGTIVSECNEGSIVAFYCVLGYLGFLAGISFIVAFLARNLPDRFNEAKYITFSMLVFCNVWISFIPTYLSTKGKYMVAVEVFAVLASSAGLLGCIFFPKCYIILLRPERNSRKCL------
>Anole_lizard.locus106.1
CSQCQKGYYPNKDQDLCIPKVVIFLSYAEPLGIGLATSAVALSFITTLVLAIFVKYNNTPIVKVNNRMLSYTLLISLLLSFLCTLLFIGRPQKMTCLLRQTVFGMVFSVVISCLLAKAITVVVAFMANKPGSKLKKWIGKRLAISIILL
CSLTQVMICSLNLATSPPFPDSDTDSFLQNNVLECNEGSGTMLYCVLGFMGFLAIISFIVAFLARKLPDSFNEAKFITFSMLLFCSVWLTFVPTYLSSRGKYMVAVEIFSILASSAGLLAFIFFPKCYIILFRPEQNKKEQLMRRKC-
>Anole_lizard.locus108.1
CFQCQTGYYPNKNHNICLRKVITFLSYEEPLGIGLATSAVALSFITALVLAIFVKYRNTPIVKVNNRMLSYTLLISLLLSFLCTLLFIGQPRRMTCLLRQTAFGIVFSVVISCLLAKTTTVVMAFMANKPGSTMNKWMGKRLATSIVLS
CSFTQAMICTLWLATSPPFPDSDTESDTQNSVLQCNEGSDFMFYCVLGFMGFLAIISFTVAFLARKLPDSFNEAKFITFSMLLFCSVWLTFVPTYLSTKGKYMVAVEIFSILASSAGLLGFIFFPKCYIVLFRPEQNKREQLMKRKG-
>Anole_lizard.locus125.1
CIECQEDQYSNDVRDFCIQKSRSFLSYEEPLGIALTTLAVSFSFITILVLAVFLKYSDTPIVKANNRSLSYTLLLALLLSFLSTFLFIGEPQKVTCLLRQTVFGVIFSVAVSCILAKTTIVVLAFLATKPGSRMRKWIGRRLASSIVIF
SSFIQTIICLVWLAISPPFPDLDTRSMATEIVLECNEGSTVLFYCVLGFMGMLAFISFTVAFLARKLPDSFNEAKCITFSMLLFCSVWLCFVPAYLSTKGKYTVAMEIFSILASSVGLLGCIFSPKCFIILVHPTLNKQGQLVIQKKK
>Anole_lizard.locus123.1
CIECQEDQYSNNAKDFCIQKSRSFLSYEEPLGIALTTLAVSFSFITILVLAVFLKYSDTPIVKANNRSLSYTLLLALLLSFLSTFLFIGEPQKVTCLLRQTVFGVIFSVAVSCILAKTTIVVLAFLATKPGSRMRKWIGRRLASSIVIS
SSFIQTIICLVWLAISPPFPDLDTRSMATEIVLECNEGSTVLFSCVLGFMGMLAFISFTVAFLARKLPDSFNEAKCITFSMLLFCSVWLCFVPAYLSTKGKYTVAVEIFSILASSVGLLGCIFSPKCFIILVHPTLNKQGQLVIQKRK
>Anole_lizard.locus121.1
CIPCPEDQHPSHGQDSCIPKAISFLSFEEPLGISFATIAVSFSFITALVLGIFIKHRTTPIVRANNSNLTFTLLTSLLLSFLCAFLFIGQPSKISCLFQQVAFALMFSLAVACILSKTTIVILAFMATKPESRTRKWMGKQFSTSIVLS
CVLVQAAICILWLATSPPFPDFNTQSMPEEIVLECNEGSSVMFYSVLGFMGSLALISFTVAFLARKLPDSYNEAKFITFSMLVFCSVWLSFVPTYLSIKGKYMVAVEIFSILASGGSLLFCIFSPKCFIIILRPELNKRGQLMRRRN-
>Anole_lizard.locus122.1
```

```
CIQCPEDQHPSHGQDSCIPKAISFLSFEEPLGISLATIAASFSFITALVLGIFIKHRTTPIVRANNSNLTFTLLTSLLLSFLCAFLFIGQPSKMSCLFQQVAFALMFSLAVACILSKTTIVILAFMATKPESRTRKWMGKQFSTSIVLS
CFLVQAAICILWLATSPPFPDFNTQSMPEEIVLECNEGSSVMFYSVLGFMGSLALISFTVAFLARKLPDSYNEAKFITFSMLVFCSVWLSFVPTYLSIKGKYMVAVEIFSILASGGSLLFCIFSPKCFIIILRPELNKRGQLMRRRN-
>Western_clawed_frog.locus697.1
--------
MSNEQRTACVPKMKNYLSYEETLGASLASIALVLFLTTSAVQGVFVKYWHTTIVKANNQNLSCLLLISLMLCFLCTLLFIGRPTQICCLLRQITFGIVFTISVSSVLAKTLTVIIAFNATKPGSTLKKFVGFQVPIVLVTICSLGEIAI
CIVWMTFDPPFLEIDIFSERDTIILQCNEGSVTFFFSIIGYIGTLALLSFIAAFLAKDFPDRFNEAKNITFSMLGFCSVWGAFVPAYLSSKGSRMVAVEIFAILSSSAGLLACIFGPKCYIIFLRPQLNTKVTAYQIH--
>Western_clawed_frog.locus630.1
CFRCPGYEMSNEQRTACVPKMKNYLSYGETLGASLASIALVLFLTTSAVQGVFVKYWHTTIVKANNQNLSCLLLISLMLCFLCTLLFIGRPTQICCLLRQVTFGIVFTISVSSVLAKTLTVIIAFNATKPGSTLKKFVGFQVPIVLVTI
CSLGEIAICIVWMTFDPPFLETDIFSERDTIILQCNEGSVTFFFSIIGYIGTLALLSFIAAFLAKDFPDRFNEAKNITFSMLGFCSVWGAFVPAYLSSKGSRMVAVEIFAILSSSAGLLGCIFGPKCYIIFLRPQLNTKVTAYQIH--
>Caecillan.locus40422.1
CWKCPEDQWPNQKRDSCIPKVITFLSYEEPLGIALTSITTFFNLLTVFILGIFIYYKDTPIVKANNRNLSYILFISLMLCFLCSLIFMGHPNKVTCILRQTAFGITFTISLSSILSKTITVVMAFYATKPGSKLRKWMGSRVSLSIVLS
CSLLQIILCLSWLFTAPPFPYFNMDLETGKILIECNDGSAIAFYCVLGYLGFLAGVSFIVAFLARNLPDSFNEAKYITFSMLVFCSVWISFIPAYLSSKGKYMVAVEIFAILTSSAGLLGCIFFPKCYIILLRPDKNNRNYLIKTIG-
>Caecillan.locus40421.1
CWKCPEDQWPNQKRDSCIPKVITFLSYEEPLGIALTSITTFFNLLTVFILGIFIYYKDTPIVKANNRNLSYILFISLMLCFLCSLIFMGHPNKVTCILRQTAFGITFTISLSSILSKTITVVMAFYATKPGSKLRKWMGSRVSLSIVLS
CSLLQIILCLSWLFTAPPFPYFNMDLETGKILIECNDGSAIAFYCVLGYLGFLAGVSFIVAFLARNLPDSFNEAKYITFSMLVFCSVWISFIPAYLSSKGKYMVAVEIFAILTSSAGLLGCIFFPKCYIILLRPDKNNRNYLIKTIG-
>Caecillan.locus40428.1
CWKCPEDQWPNQKRDSCIPKVITFLSYEEPLGIALTSISIFFILLTVFILGIFIYYKDTPIVKANNRNLSYILLISLMLCFLCSLIFMGHLNKVTCILRQTAFGITFTISLSSILSKTITVVMAFYATKPGSKLRKWMGSRVSLSIVLS
CSLLQTILCLSWLFTAPPYPYFNMHLETGKILIECNDGSTIAFYCVLGYLGFLAGVSFIVAFLARNLPDSFNEAKYITFSMLVFCSVWIFFIPAYLSSKGKYMVAVEIFAILTSSAGLLGCIFLPKCYIILLRPDKNNRTYLIKTIG-
>Caecillan.locus40420.1
--------------------
MITFLSYEEPLGIALTSITTFFILLTVVILGIFIYYKDTPIVKANNRKLSYILLISFIFCFTCSLIFIGHPNKVTCILRQTAFGITFTISLSSILSKTITVVMAFYATKPGSKLRKWMGSRVSLSIVLSCSLLQTILCLSWLFTAPPFP
YFNMHLETGKILIECNDGSVIAFYCVLGYLGFLAGISFIVAFLARNLPDSFNEAKYITFSMLVFCSVWISFIPTYLSSKGKYMVAVEIFAILASSAGLLGCIFFPKCYIILLRPDKNNRNYLIKTIG-
>Caecillan.locus40415.1
CWKCPEDQWPNQKRDNCIPKVITFLSYEEPLGLALTSIITFFFLVTVVILGIFINYKDTPIVKANNRNLSYILLISLMLCFLCSLIFMGHPNKVTCILRQTAFGITFTITLSSILSKTIIVVMAFYATKPGSKLRKWMRSRVSLSIVLS
CSLLQTVLCLSWLLTAPPFPYLNMQSETGKILIECNEGSVIAFYCVMGYLGFLAGISFIVAFLARNLPDSFNEAKHITFSMLVFYSVWISFIPTYLSSKGKYMVAVEIFSILASSAGLLGCIFFPKCYIILLRPERNNRNYLTKTIE-
>Caecillan.locus40418.1
CWKCPEDQWPNKKGDNCIPKVITFLSYEEPLGLTLTSITTFFFLVTVVILGIFINYKDTPIVKANNRNLSYILLISLMLCFLCSLIFMGHPNKVTCILRQTAFGITFTISLSSILSKTIIVVMAFYATKPGSKLRKWMRSRVSLSIVLS
CSLLQTVLCLSWLFTAPPFPYLNMQSETGKILIECNEGSVIAFYCVLGYLGFLAGISFIVAFLARNLPDSFNEAKYITFSMLVFCSVWISFIPTYLSSKGKYMVAVEIFAILASSAGLLSCIFFPKCYVILLRPERNNRNYLTKTIE-
>Caecillan.locus40417.1
CWKCPEDQWPNQKRDSCIPKVINFLSYEEPLGIVLTSITTFFFLVTVVVLGIFINYKDTPIVKANNRNLSYILLISLMLCFLCSLIFIGHPNMVTCILRHTAFGITFTISLSSILSKTIIVIMAFYATKPGSKLQKWMGSRVSLSIVLS
CSLLQTVLCLGWLFTAPPFPYLNMHSEPGTILIECNEGSAIAFNCVLGYLGFLAGISFIVAFLARNLPDSFNEAKYITFSMLVFCSVWISFIPTYLSNKGKYMVAVEIFAILASSAGLLGCIFLPKCYIILLRPERNNRNYLTKTIG-
>Caecillan.locus40409.1
CWKCPEDQWPNQKRDSCIPKTINFLAYEEPLGIVLTSITIFFFLVTVVVLGIFINYKDTPIVKANNKNLSYILLISLMLCFLCSLIFIGHPNKVTCILRQTVFGIAFTISLSSILSKTIIVVMAFYATKPGSKLQKWMGSRVSLSIVLS
CSLLQTVLCLVWLFTAPPFPYCNMHSEIGKILIECNEGSVIAFYYVLGYLGFLAGISFIVAFLARNLPDSFNEAKCITFSMLVFCSVWISFIPTYLSSKGKYMVAVEIFAILASSAGLLGCIFLPKCYIILLRPDRNNRNYLIKTIG-
>Caecillan.locus40413.1
CWKCPEDQWPNQRRDSCIPKVITFLSYEEPLGIAFTSISSFFFLVTAVILGIFINYRDTALVRANNRDLSYILLISLMICFLCSLIFIGHPNKVTCILRHMIFGITFAISLSSILSKTITVVMAFHATKPGSKLLKWMGSRVSLSIVLS
CSFIQSDLCLAWLFIAPPFPYLNMHSEPGTILIECNDGSVIIFYCVMGYLGFLAAISFIVAFLARNLPDSFNEAKYITFSMLVFCSVWISFIPAYLSTKGKYMVAVEIFAILASSAGLLGCIFIPKCYIILLRPDRNSRKYLTKTTEG
>Caecillan.locus40414.1
CWKCPEDQWPTDKRNACIPKVITFLSYEEPLGIALTFITTFFFLVTAVILGIFINYQDTPIVRANNRDLSYILLISLMLCFLCSLIFIGHPNKVTCILRQTAFGISFAISLSSILSKTITVFMAFHATKPGSKLRKWMGSRVSVSIVLS
CSLLQTVLSLTWLLIAPPFPYLNIRSEIGTILIECNEGSVIAFYCVLGYLGFLAGTSFIVAFLARNLPDSFNEAKYITFSILVFCSVWISFIPTYLSTKGKYMVAVEIFAILASSTGILGCIFLPKCYIILLRPDMNSRKYLTKTTGL
>Caecillan.locus40284.1
CWKCPENQWPNQKRDACIPKVIVFLSFEEPLGIALTIISIFFFIITAVILRIFFNYQNTPIVRANNHVLSYILLISLMLCFLCSLIFIGHPNEVNCILRQTVFGISFSIALSSILSKTITVVMAFQATKPGSKLRKWMGPRVSVSIVLS
CSLIQTILCLAWLFTAPPFPYINMRSEIEIILIECNEGSIVAFYCVLSYLGFLAGISFIVAFLARNLPDSFNEAKYITFSMLVFCSVWISFIPTYLSTKGKYMVAVEIFAILASSAGLLGCIFFPKCYIILLKPERNSKKYLTKTIMG
>Caecillan.locus40410.1
CWKCPEDQWPNHKRDACIPKVITFLSYEEPLGIVLTSMSIFFFLTTAAILGIFFNYRDTPIVRANNQDLSYILIISLLFCFLCSLVFIGRPNKVTCILRQTVFGIIFSIAVSSILAKTITVVMVFHATKPGSKLQKWMGSRVSVSIVLS
FSLIQTVLCLAWLFTAPPFAYLNMRSEIGTILIECNEGSVIAFYCVLGYLGFLAVISFIIAFLARNLPDSFNEAKYITFSMLVFCSVWISFIPTYLSTKGKYMVAVEIFAILASSAGLLGCIFFPKCYIILLKPERNSKKYLTKNTGG
>Caecillan.locus40431.1
CTSCPEDQWSNQKRDACIPKIITFLSYKDPLGIALTSTSIFFFLITAAILGIFINYRDTPIVKANNRDLSYVILISLMLSFLCSLVFIGHPENITCILRQTAFGIIFSITLSSILAKTITVVMAFHATKPGNKFRKWIGSRVSVSIVLC
CSLIQTVLCLVWLFTAPPFPYLNMRSEIGIIIIECNEGSAIAFYCVLGYLGFLAGVSFIIAFLARNLPDSFNEAKYITFSMLVFCSVWISFIPTYLSTKGKYMVAVEIFAILASTVGILGCIFLPKCCIILMRPERNNRKYIKKNVTT
>Caecillan.locus40443.1
CTICPEDQWPNQKRDACIPKIITFLSYKDNLGIALTFTSIFFFLIAAAILRIFINYRDTPIVRANNRDLSYILLISLMLSFLCSLLFIGQPENITCILRQTAFGIIFSITLSSLLAKTITVVMAFHATKPGNTFRKWMGSRVSVTIILC
CSLIQTVLCLAWLFTAPPFAYLNMISEIGIIIIECNEGSVIAFYCVLGYLGFLAGISFIIAFLARNLPDSFNEAKYITFSMMVFCSVWISFIPTYLSTKGKSMVAVEIFAILASSGGILGCIFFPKCYIILFRPEKNSKKDLRKI---
>Spotted_gar.locus156.1
CFKCPPEFWSNPNRDQCVLKEIEFLSYGESLGISLTTVSVLGALFSTAVLAVFIFYRSTPVVRANNSELSFLLLLSLILCFLCSLLFIGHPLHLTCMLRHVVFGISFVLCISCILVKTIVVIMAFRATLPDNNVMKWFGAAQQRGTVFV
FTVIQALICIIWLSTAPPVPFKNTQYQNSKIIFECYVGSVTGFIFLLGYIGLLASVCFVLAFLARNLPDNFNEAKFITFSMLIFCAVWIAFIPTYVSSPGKYSDTVEIFAILASSFGLLIAIFAPKCYIIVLRPEKNTKKALMGRAAT
>Caecillan.locus40485.1
CTTCPEDQWPNKKRDACIPKVKTFLSYEEPLGIALTSIGLFFFLVTAVILGIFIYYRDTPIVRANNRDLSYIFLISLMLCFLCSLLFIGHPEDVTCILRQTTFGITFSITLSSILAKTITVVMAFHATKPGSKFRKWMGSRVSNSIVLS
SSFIETILCLSWLFSAPPFAYLNMKSETGTILIECNEGSVIAFYCVLGYLGFLAGVSFIIAFLARNLPDSFNEAKYITFSMLVFCSVWISFIPTYLSTKGKYMVAVEIFAILASSAGLLGCILFPKCYIILLRPERNSKKYLTKT---
>Caecillan.locus40486.1
CTTCPEDQWPNKKRDACIPKVKIFLSYEEPLGIALTSIGLFLFLVTAVILGIFIYYRDTPIVRANNRDLSYIFLISLMLCFLCSLLFIGHPEDVTCILRQTTFGITFSITLSSILAKTITVVMAFHATKPGSKFRKWMGSRVSNSIILS
SSFIETVLCLSWFFSAPPFAYLNMKSETGTILIECNEGSVIAFYCVLGYLGFLAGVSFIIAFLARNLPDSFNEAKYITFSMLVFCSVWISFIPTYLSTKGKYMVAVEIFAILASSAGLLGCILFPKCYIILLRPERNSKKYLSKT---
>Caecillan.locus36955.1
CTTCPENEWPNHNRDACIRKVKTFLSYEEPLGIALTSISLFFFLVTAVILGIFIYYRDTPIVRANNRDLSYIFLISLMLCFLCSLVFIGHPEDVTCILRQTTFGITFSVTLSSILAKTITVVMAFHATKPGSKLRKWMGSRVSNSIVLS
CSLIQTILCLSWLFTAPPFACLNMRSEIGTILIECNEGSVIAFYCVLGYLGFLAGVSFIIAFLARNLPDSFNEAKYIIFSMLVFCSVWISFIPTYLSTKGKYMVAVEIFAILASSAGLLGCIFFPKCYIILLRPEKNSKKYLTKT---
>Caecillan.locus40412.1
CTICPEDQWPNQKRDACILKVKTFLSYEEPLGITLTSSSLFFFLVTAVILGIFIYYRDTPIVRANNQDLSYIFLISLKLCFLCSLLFIGHPEDVTCILRQSTFGITFSITLSSILANTITVVMAFHATKPGSKLRKWMGSRVSNSIVLS
CSLIQTVLCLAWLFTAPPFAYLNMRSEIGTILIECNEGSVIAFYCVLGYLGFMAGISFIVAFLARNLPDRYNEAKYITFSMLVFCSVWISFIPTYLSTKGKYMVAVEIFAILASSTGLLGCIFFPKCYFILLRSERNSKKYLTKKKHN
>Western_clawed_frog.locus620.1
--------
MSNKERTACVSKFINYLSYEDTLGASLASVALMLFLTTSTVQGIFVKYWETPIVRANNQNLSCLLLISLMLCFLCTLLFIGRPTQICCLLRQVTFGIVFTISVSSVLAKTLTVIIAFNATKPGSKLKKYVGTQLAIVLVIICSLGKIGI
STMWMASCPPFLEADMFSEMDTIILKCNEGSVTFFFSIIGYIGTLALLSFIAAFLAKDFPDRFNEAKNITFSMLGFCSVWGAFVPAYLSSKGSRMVAVEIFAILSSSAGLLGCIFAPKCYIIFIRPELNTRVVVVRNQ--
>Cloudy_catshark.locus41.1
------------------------------
MGIILLAVALFGACITMGVFSTFYFYKDTPVVKANNSELSFLLLVALTLCFLCSIAFIGEPSVWSCILRHTAFAVIFVLCISCVLSKTVVVVMAFNAKFPNHNMMKWFGPTQQRLTVAFLTMVQCLICTVWLSTLPPYPRKNSAYAKNR
IIFECNVGSAINFYCALGYIGVLSCVCFLAAFLARQLPNNFNEAKHITFSMLIFCAVWVTFIPAYISSPGKYAVAVEVFAILASSFGLLICIFAPKCYIILFKQSENTKRHIMGH---
>Western_clawed_frog.locus700.1
--------
MSNIRRTGCILKTMNYLSYKDTLGASLASIALVLFLTTSAVQGIFMKYWETPIVRANNQHLSCLLLISLMLCFLCTLLFIGRPTQICCLLRQVTFGIVFTISVSSVLAKTLTVIIAFNATKPGSKLKKYVGTQLAILLVLMCTLGKIGI
SVVWMASNPPFLEADTFSEMETIILLCNEGSVTFFFCIIGYIGTLALLSFIAAFLAKDFPDRFNEAKNITFSMLGFCSVWGAFVPAYLSSKGSRMVAVEIFAILASSAGLLACIFVPKCFIIFFRPKLNKRANVLK----
>Zebrafish.locus486.1
------------------------------
MGIVLVIFSLFGVTLTVLVTFLFYSKKDTPIVKANNSELSFLLLFSLTLCFLCSLTFIGRPTEWSCMLRHTTFGITFVLCISCILGKTIVVLMAFRATIPGNNMIKWFGPVQQRLSVLAFTLIQILICVVWLTISPPFPSKNIKYYKEK
IILECSLGSTIGFWIVLGYIGLLAVLCFILAFLARTLPDNFNEAKFITFSILIFCAVWITFIPAYVSSPGKLTVAVEIFAILASSFGLLFCIFAPKCYIILFKPEQNTKQHIMGKTIS
>marker.zebrafish_9_3_F
---
CPEEYWPNTEKSKCVLKIVEFLSFTELMGIVLVIFSLFGVTLTVLVTFLFYSKKDTPIVKANNSELSFLLLFSLTLCFLCSLTFIGRPTEWSCMLRHTTFGITFVLCISCILGKTIVVLMAFRATIPGNNMIKWFGPVQQRLSVLAFTL
IQILICVVWLTISPPFPSKNIKYYKEKIILECSLGSTIGFWIVLGYIGLLAVLCFILAFLARTLPDNFNEAKFITFSILIFCAVWITFIPAYVSSPGKLTVAVEIFAILASSFGLLFCIFAPKCYIILFKPEQNTKQHIMGKT--
>marker.zebrafish_9_11_F
---
CPGEYWSNAEKNRCVLKTVEFLSFTEVMGIVLVFFSLFGVGLTVLVAILFYSKKETPIVKANNSELSFLLLFSLTLCFLCSLTFIGRPTEWSCMLRHTTFGITFVLCISCVLGKTVVVLMAFRATHPGKDIMKWFGPVQQRLSVIALTL
IQVLICVLWLTISPPYPYKNMKYFKEKTILECNLGSTIGFSAVLGYIGLLAVLCFFLAFLARTLPDKFNEAKFITFSMLIFCAVWITFIPSYVSSPGKLTVAVEIFAILASSFGLLFCIFAPKCYVILLKPEQNTKQHIMGKI--
>Zebrafish.locus478.1
------------------------------
MGIVLVFFSLFGVGLTVLVAILFYSKKETPIVKANNSELSFLLLFSLTLCFLCSLTFIGRPTEWSCMLRHTTFGITFVLCISCVLGKTVVVLMAFRATHPGKDIMKWFGPVQQRLSVIALTLIQVLICVLWLTISPPYPYKNMKYFKEK
TILECNLGSTIGFSAVLGYIGLLAVLCFFLAFLARTLPDKFNEAKFITFSMLIFCAVWITFIPSYVSSPGKLTVAVEIFAILASSFGLLFCIFAPKCYVILLKPEQNTKQHIMGKISS
>Zebrafish.locus481.1
------------------------------
MGIILVFFSLFGAGLTVLVAILFYSKKDTPIVKANNSELSFLLLFSLTLCFLCSIPFIGRPTQWSCMLRHTAFGITFVLCISCILGKTIVVLMAFKATLPGSNIMKWFGPVQQRLSVLAFTLIQGLICVIWLTKSPPFPYKNMKYFQEQ
IILECSVGSTIGFWAVLGYIGLLAVLCFILAFLARTLPDNFNEAKFITFSMLIFCAVWITFIPAYVSSPGKLTVAVEIFAILASSFGLLFCIFAPKCYIILLQPEQNTKQQMMAKTPS
>marker.zebrafish_9_8_F
---
CPGEYWSNANKNKCVTKAVEFLSFTEVMGIILVFFSLFGAGLTVLVAILFYSKKDTPIVKANNSELSFLLLFSLTLCFLCSIPFIGRPTQWSCMLRHTAFGITFVLCISCILGKTIVVLMAFKATLPGSNIMKWFGPVQQRLSVLAFTL
IQGLICVIWLTKSPPFPYKNMKYFQEQIILECSVGSTIGFWAVLGYIGLLAVLCFILAFLARTLPDNFNEAKFITFSMLIFCAVWITFIPAYVSSPGKLTVAVEIFAILASSFGLLFCIFAPKCYIILLQPEQNTKQQMMAKT--
>marker.zebrafish_9_7_F
---
CQGEYWSNADKNKCVQKSVEFLSFTEVMGIVLVFFSLFGAGLTVLVAILFYSKKDTPIVKANNSELSFLLLFSLTLCFLCSLTFIGQPTQWSCMLRHTAFGITFVLCISCVLGKTLLVLMAFKATLPGSNIMKWFGPVQQRLSVLAFTF
IQVLICVLWLSISPPFPHKNMKYYEEKIILECSLGSTIGFWAILGYIGLLAALCFILAFLARTLPDNFNEAKFITFSMLIFCAVWITFIPAYVSSPGKFTVAVEIFAILSSSISLLLCIFAPKCYIILLKPEQNTKQYMLGKT--
>marker.zebrafish_9_1_F
---
CQGEYWSNVEKNKCVLKAVEFLSFTDFMGIVLVFFSLFGVGLTVLVAILFYSKKDTPIVKANNSELSFLLLLSLTLCFLCSLTFIGRPTEWSCMLRHTAFGITFVLCISCVLGKTIVVLMAFKATLPGSNVMKWFGPVQQRLSVLAFTL
IQVLICVLWLTMSSPFPYKNMKYYKEKIILECSLGSVIGFWAVLGYIGLLAALCFILAFLARTLPDNFNEAKFITFSMLIFCAVWVTFIPAYVSSPGKYTVAVEIFAILASTFGLLFCIFVPKCHIIIFKPEQNTKQHMMGKN--
>Zebrafish.locus487.1
CKQCPGEYWSNDKKNKCVLKAVEFLSFTEVMGVLLLFVSLFGVGLTTLVAILFYNKKDTPIVRANNSELSFLLLFSLILCFLCSITFIGRPTEWSCMLRHTAFGITFVLCISCVLGKTIVVLMAFKATHPGSNVMKWFGPVQQRLSVLV
FTFIQVIICVLWLTISPPFPYRNMKYYKEKIILECSLGSIVGFWAVLGYIGLLAALCFILAFLARKLPDNFNEAKFITFSMLIFCAVWITFIPAYFSSPGKLTVAVEIFAILASSIGLLFCIFAPKCYIILCKPEQNTKQHIMGKNN-
>marker.zebrafish_9_4_F
---
CPGEYWSNDKKNKCVLKAVEFLSFTEVMGVLLLFVSLFGVGLTTLVAILFYNKKDTPIVRANNSELSFLLLFSLILCFLCSITFIGRPTEWSCMLRHTAFGITFVLCISCVLGKTIVVLMAFKATHPGSNVMKWFGPVQQRLSVLVFTF
IQVIICVLWLTISPPFPYRNMKYYKEKIILECSLGSIVGFWAVLGYIGLLAALCFILAFLARKLPDNFNEAKFITFSMLIFCAVWITFIPAYFSSPGKLTVAVEIFAILASSIGLLFCIFAPKCYIILCKPEQNTKQHIMGKN--
```

```
>marker.zebrafish_9_5_F
---
CPLEYWSNTEKNKCVFKSVEFLSFTEVMGVVLVFFSLFGVGLTMLVAILFYNKKDTPIVKANNSELSFLLLFSLTLCFLCSLTFIGRPTEWSCMLRHTTFGITFVLCISCVLGKTIVVLMAFKATLPGSNIMKWFGPVQQRLSVLAFTL
IQVIICVLWLIISPPFPYKNMKYYKEKIILECSLGATICFSAVLFYIGLLAILCFILAFLARKLPDNFNEAKFITFSMLIFCAVWITFIPAYVSSPGKFTVAVEIFAILASSFGLLFCIFVPKCYIILFKNEQNTKQHIMGKI--
>Zebrafish.locus488.1
----------------------------
MGVVLVFFSLFGVGLTMLVAILFYNKKDTPIVKANNSELSFLLLFSLTLCFLCSLTFIGRPTEWSCMLRHTTFGITFVLCISCVLGKTIVVLMAFKATLPGSNIMKWFGPVQQRLSVLAFTLIQVIICVLWLIISPPFPYKNMKYYKEK
IILECSLGATICFSAVLFYIGLLAILCFILAFLARKLPDNFNEAKFITFSMLIFCAVWITFIPAYVSSPGKFTVAVEIFAILASSFGLLFCIFVPKCYIILFKNEQNTKQHIMGKIK-
>Zebrafish.locus482.1
CKQCPREYWSNGEKNKCVLKAIEFLSFTEIMGIVLVCFSLFGVGLTAVVAILFWSKMDTPIVKANNSELSFLLLFSLTLCFLCSLTFIGRPTEWSCMLRHTAFGITFVLCISCVLGKTIVVLMVFKATLPGSNVMKWFGPTQQRLSVLA
FTFIQVLICVLWLTISPPFPNKNMTYYKEKIIIECSLGSTISFWAVLGYIGLLAVLCFILAFLARKLPDNFNEAKFITFSMLIFCAVWITFIPAYVSSPGKFTVAVEIFAILASSFGLLLCIFAPKCYIIICKPEQNTKQHVMGKTSL
>marker.zebrafish_9_9_F
---
CPREYWSNGEKNKCVLKAIEFLSFTEIMGIVLVCFSLFGVGLTAVVAILFWSKMDTPIVKANNSELSFLLLFSLTLCFLCSLTFIGRPTEWSCMLRHTAFGITFVLCISCVLGKTIVVLMVFKATLPGSNVMKWFGPTQQRLSVLAFTF
IQVLICVLWLTISPPFPNKNMTYYKEKIIIECSLGSTISFWAVLGYIGLLAVLCFILAFLARKLPDNFNEAKFITFSMLIFCAVWITFIPAYVSSPGKFTVAVEIFAILASSFGLLLCIFAPKCYIIICKPEQNTKQHVMGKT--
>Western_clawed_frog.locus92.1
CLQCPDNEWPNQGKNMCIEKQTDFLSYDGSLTLSFAFISVICFLMNEIIFGIFISHRDTPVVKANNRDLSFILLIAIKLSFLSVFLFLGRPVDITCMLRIITFGITFSIAVSSLLAKTIMVCVAFKATKPGSSWRKWLGVKLSNSVVLF
CSSIQIIICMTWLAISPPFQELDIHTSPGTIIIQCNEGSAIGFYSVIGYMGLLAAVSFVLAFLARSLPDSFNEAKYITFSMLLFCSVWITMIPAYLSTKGKYMVAVEIFAIISSSCGLLFCIFLPKCYIIVLKPETNRRQYLLGR---
>Western_clawed_frog.locus93.1
CLQCPDNEWPNQGKNMCIEKQTDFLSYDGSLTLSFALISVICFLMNEIIFGIFISYQDTPVVKANNRDLSFILLIAIKLSFLSVFLFLGRPVDITCMLRIITFGITFSIAVSSLLAKTIMVCVAFKATKPGSSWRKWLGVKLSNSVVLF
CSSIQIIICMTWLAISPPFQELDIHTSPGTIIIQCNEGSAIGFYSVIGYMGLLAAVSFVLAFLARSLPDSFNEAKYITFSMLLFCSVWITMIPAYLSTKGKYMVAVEIFAIISSSCGLLFCIFLPKCYIIMLKPETNRRQYLLGRLTL
>Western_clawed_frog.locus91.1
CLQCPDNEWPNQGKNMCIEKQTDFLSYDGSLTLSFAFISVICFLMNKIIFGIFISYRDTPVVKANNRDLSFILLIAIKLSFLSVFLFLGRPVDITCMLRIITFGITFSIAVSSLLAKTIMVCVAFKATKPGSSWRKWLGVKLSNSVVLF
CSSIQIIICMTWLAISPPFQELDIHTSPGTIIIQCNEGSAIGFYSVIGYMGLLAAVSFVLAFLARSLPDSFNEAKYITFSMLLFCSVWITMIPAYLSTKGKYMVAVEIFAIISSSCGLLFCIFLPKCYIIMLKPEMNTKQYLLGRLNT
>Western_clawed_frog.locus94.1
CLQCPDNEWPNQGKNMCIEKQTDFLSYDGSLTLSFAFISVICFLMNEIIFGIFISYRDTPVVKANNRDLSFILLIAIKLSFLSVFLFLGRPVDITCMLRIITFGITFSIAVSSLLAKTIMVCVAFKATKPGSSWRKWLGVKLSNSVVLF
CSSIQIIICMTWLAISPPFQELDIHTSPGTIIIQCNEGSAIGFYSVIGYMGLLAAVSFVLAFLARSLPDSFNEAKYITFSMLLFCSVWITMIPAYLSTKGKYMVAVEIFAIISSSCGLLFCIFLPKCYIILFNPEMNRRQYLLGRLNK
>Western_clawed_frog.locus95.1
CLQCPDNEWPNQGKNLCIEKQTDFLSYDGSLTLSFAFISVICFLLTEIIFGIFISYRDTPVVKANNRDLSFILLIAIKLSFLSVFLFLGRPVDITCMLRIITFGITFSIAVSSLLAKTIMVCVAFKATKPGSSWRKWLGVKLSNSVVLF
CSSIQIIICMTWLAISPPFQELDIHTSPGTIIIQCNEGSAIGFYSVIGYMGLLAAVSFVLAFLARSLPDSFNEAKYITFSMLLFCSVWITMILAYLSTKGKYMVAVEIFAIISSSCGLLFCIFLPKCYIILFHPEMNRRQHLLGR---
>Western_clawed_frog.locus96.1
CLQCPDNEWPNRGKNMCIEKQTDFLSYDGSLTLSFAFISVICFLMNEIIFGIFISYRDTPVVKANNRDLSFILLIAIKLSFLSVFLFLGHPVDITCMLRIITFGIPFSIAVSSLLAKTIMVCVAFKATKPGSSWRKWLGVKLSNSVVLF
CSSIQIIICMTWLAISPPFQELDIHTSPGTIIIQCNEGSAIGFYSVIGYMGLLAAVSFVLAFLARSLPDSFNEAKYITFSMLLFCSVWITMIPAYLSTKGKNTVCVEIFAIISSSCGLLFCIFLPKCYIILFHPEMNRRQYLLGR---
>Western_clawed_frog.locus90.1
CLQCPDNEWPNQGKNLCIEKQTDFLSYDGSLTLSFAFISAICFLMTEIIFGIFISYQDTPVVKANNRDLSFILLIAIKLSFLSVFLFLGRPVDITCMLRIITFGITFSIAVSSLLAKTIMVCVAFKATKPGSSWRKWLGVKLSNSVVLF
CSSIQIIICMTWLAISPPFQELDIHTSPGTIIIQCNEGSAIGFYSVIGYMGLLAAVSFVLAFLARSLPDSFNEAKYITFSMLLFCSVWITMIPAYLSTKGKNTVCVEIFAILTSSVGLLASIFLPKCYIILFKPEMNTKQCLLKDINI
>Coelacanth.locus404.1
CLECPYDHWSNDRRDKCIPKPIEFLSYKELLGAILSSISLFSSLIPAVILCTFIRYRNTPIVKANNRELSYVLLLALVLCFLCCLIFIGQPMVVTCILRQAVFGIVFALCVTCVLAKTIMVVIAFKATNPDSNLKKWVGPKLPNTIVFV
CTLLQVIICIAWLSSSPPFPEQNMKSEPGKIIIECNEGSTVAFWCMLGYMGLLAIISFIVAFLARSLPGSFNEAKFITFSMLVFVCVWLSFIPAYLSTKGKYMVAVEIFAILASSAGLLACIFFPKCYIILLRPDINTRENLIGKEAF
>Coelacanth.locus350.1
CLKCPEDHWSNERKDQCIPKSVEFLSYKEPLGAILASISVFCSLIPAAILCTFFRYRDTPIVKANNRELSYLLLVALILCFLCCLIFIGQPLEVSCILRQTAFGIIFAFGVSCVLAKTIIVVIAFNATKPNSNVKRWVGHKLSSIILLT
CTLLQVVICIAWLSSYPPFPERNMKSQPGKIIIECNEGSIIAFWCMLGYMGLLAIVSFIVAFIARNLPDSFNEAKFITFSMLVFVTVWLSFIPAYLSTKGKYMVAVEVFAILASSAGLMACIFFPKCYIILLRPDINTRDYLKGKESS
>Coelacanth.locus413.1
CLECPDDHYSNDERDKCIPKSIEFLSYKEPLGSILSSTSIFSSIIPAAILCIFIRYRDTPIVKANNRELSYLLLLALVLCFLCCLIFIGQPTAVTCILRQTAFGIIFAFSVACVLAKTIMVVIAFNATKPNSNLKKWVGHKLSNTIVFL
CTLLQIIICIAWLASFPPFPEQNVKSQTRKIITECNEGSTVAFWCMLGYMGLLATVSFIVAFLARNLPDSFNEAKFITFSMLVFVTVWLSFIPAYLSTKGKYMVAVEIFAILASSAGLLVCIFFPKCYIILFRPDINTREYLMGKGNK
>Coelacanth.locus365.1
--
QCPDDHWSNARRDKCIPKLIEFLSYKETLGVILASISIVSSLLPAAILYTFLKYHETPVVKANNCELSYLLLFALMLCSLCCLIFIGRPVAVTCALRQTAFGIVFALSVSCVLAKTIIVVIAFNATKPNSSLKKWVGHKLSNTIVSMGT
LLQVIICIIWLTSSPPFPQQNMKSEPGKIIIECNEGSPVAFWCMLGYMGLLATVSFIVAFLARNLPDSFNEAKFITFSMLVFVTVWLSFIPAYLSTRGKYMVAVEIFAILTSSAGLLICLFFPKCYIILLRPDINTREYLMGKGSQ
>Coelacanth.locus248.1
CLECPDDHWSNDRRDTCVPKSIEFLSYTENLGIILASASILFSFIPAAILCTFIRYRETPIVKANNRELSYVLLLALVLCFLCCLIFIGQPVMVTCMLRQTAFGIIFAFSVSCVLAKTIMVVIAFNATKPNSSLKKWVGPKLSNTIVIV
CTVVQVIICVIWLASSPPFPEENMKSEPRKLIIECNEGSTVAFWCMLGYMGLLATISFIVAFLARNLPDSFNEAKLITFSMLVFVIVWLSFIPAYLSTKGKYMVAVEIFAILASSAGLLACIFFPKCYIILLRPDINTREYLMGKGTF
>Coelacanth.locus143.1
--------------
MCVPKSIEFLSYTENLGIILASTSILFSFIPAAILCTFIRYRETPIVKANNRELSYVLLLALVLCFLCCLIFIGQPVMVTCMLRQTTFGIIFAFSISCVLAKTIMVVIAFTATKPNSSLKKWVGPKVSNTIVIVRTLVQIIICVTWLAS
SPPFPEENMKPDPGKIIIECNEGSTVAFWCMLGYMGLLASVSFIVAFIARNLPDSFNEAKLITFSMLVFVSVWLSFIPAYLSTKGKYMVAVEIFAILASSTGLLLCIFFPKCYIILLRPDINTREFLMGKGTF
>Coelacanth.locus142.1
CLECPDDQWSNNRRDKCIPKAIEFLSYKETLGAILASISILFSIIPVAILCIFIRNRETPIVKANNRELSYLLLSALVLCFLCCLIFIGQPVVVTCMLRQTAFGIIFVFSVSCVLAKTIMVVVAFNATKPNSNLKKWVGPKLSNTIVLM
CTLLQVIICIVWLSNFPPFPEENMKSQPGKIIFECNEGSVVAFWCVLGYMGLLATVCFIVAFLARNLPDSFNEAKFITFSMLVFVSVWLSFIPAYLSTKGKYMVAVEIFAILASGGGLMACIFFPKCYIILLRPDINTKEYLMGKGTF
>Coelacanth.locus311.1
-MECPHNHYSNGRRDECIPKSIEFLSYKEPLGAILASISILSSLIPSAILCTFVRHHDTPIVKANNRELSYFLLLALVLCFLCCLIFIGQPVLATCMLRQTAFGVIFAFSVSCVLAKTIMVVIAFNATKPNSNLKKWVGHKLSNTIVFV
CTLLQVIICTVWLASSPPFPEQNMKSETRKIIIQCNAGSTLAFWFMLGYMGMLAIISFIVAFLARNLPDSFNEAKFITFSMLVFVCVWLSFIPAYLSTKGKYMVAVEIFAILASSTGLLGCIFFPKVYIIVLRPEMNTREFLMGKGTF
>Coelacanth.locus150.1
CTECPFDHYSNDRRDECIPKSIEFLSYKETLGAILASTSIFSSLIPVVILCIFIRHHDTPIVKANNRELSFLLLLGLVLCFLCCLIFIGRPVVITCLLRQTAFAIVFTLSVSCVLAKTIMVVIAFNATKPNSNFKKWVGPKLSNTIVFV
CTFLQVIICIAWLTSSPPFPEQNMNSEPGKIIIGCNEGSTVAFWFMLGYMGLLAFVSFVVAFLARNLPDSFNEAKFITFSMLVFVSVWLAFIPAYLSTRGKYMVAVEIFAILASSAGLLACIFFPKCYIILLRPEINTKEYLMGKGTF
>Coelacanth.locus153.1
-QECPYDHWSNDRQDKCIPKSIEFLSFKESLGIIVVSITVSSSLIPAASLYIFLRYRDTPIVKANNRELSYLLLLALVLCFLCSLIFIGEPMIVTCILHQITFGIIFAFSVSCVLAKTIKVVIAFKATNPNSNLKKWVGLKLPNTVVFA
CTLLQVIICMAWLTSSPPFSEKNMNSEPGKIIIECNEGSTVAFYCMLGYLGLLAIVSFIMAFFARNLPNNFNEAKFITFSMLVFVSVWLSFIPAYLSTKGKYMVAVEIFAILASSAGLLACIFFPKCYFILLKPEINTRENLMGRETF
>Coelacanth.locus373.1
RQQCPHNHWSNDRRDKCIPKTIEFLSFKEPLGVILSCISIISSLIPAAILYTFVRYRDTPIVKGNNRELSYLLLLALMLCFLCCLIFIGQPMMVNCILRQTAFGIVFTFSVSCVLAKTIMVIIAFKATNPNSNLKKWLGPKLPNTIVFI
CTLFQVVICIAWLTSSPPFPEKNMNSKPGMIIIECNEGSTAAFWCMLGYMGLLAIVSFIVAFLAQNLPGSFNEAKFITFSMLVFVSIWLSFIPAYLSTKGKYMVAAEIFAMLASSAGLLSCIFFPKCYIIVLRPDINTRENLMGKVDF
>Caecillan.locus40569.1
CIRCQEDQWPNENRDACFPKIISFLSHEETLGIILTLISIFFFFITAVILGIFIYYQATPIVKSNNRNLSYIILVSLMLCFLCSLIFIGHPDKVTCVLRQVAFGITFSISLSSVLAKTITVVMAFHSTKPGSKLRKWMGSRVSYSIVLS
CSLLQVFLCLIWLGAAPPFPHLNMEGEDGTIQIECNEGSAIAFYCVLGYLGFLAGISFIVAFLARNLPGSFNEAKHITFSMLVFCSVWVSFIPTYLSTRGKYMVAVEIFAILASGFGILGCIFIPKCYIILLRPERNNRKYIAKK---
>Elephant_shark.locus223.1
---------------------------
MLGGVLLTLALVGVSTTISIAIVFFTHRDTPIVKANNSELSYLLLFALTLCFLCSVTFIGEPSVWACMLCHTAFAIAFVLCISCILIKTILVILAFKATLPNNNMAKLFGPAQQRWSVFLLTSVQCLICTLWLTISHPFPMRNTEYYRE
IIILECDLGSVSAFYSMAGYIAFLAGVSFVLAFLARKLPDNFNEAQCITFSMLTFCAVWITFIPVYISSPGKYTIAVEVFAILASSFGLLFCIFVPKCYIILIKPEINTRKHLLGKVPV
>Elephant_shark.locus202.1
---------------------------
MLGGVLLTLALVGVSTTISIAIVFFTHRDTPIVKANNSELSYLLLFALTLCFLCSVTFIGEPSVWACMLCHTAFAIAFVLCISCILIKTILVILAFKATLPNNNMAKLFGPAQQRWSVFLLTSVQCLICTLWLTISHPFPMRNTEYYRE
IIILECDLGSVSAFYSMAGYIAFLAGVSFVLAFLARKLPDNFNEAQCITFSMLTFCAVWITFIPVYISSPGKYTVAVEVFAILASSFGLLFCIFVPKCYIILIKPEMNTKKHLMGKVPV
>Anole_lizard.locus101.1
CFQCPEDHYPNKIRDFCIPKYIIFLGFEDPFGTGLASSALFLFCVTVVVLGIFLKHRDTPIVKANNRTLTYTLLLSLLLSFLCALLFIGKPQKMTCLLRQTAFGIIFSVAISSVLAKTITVILAFQTTKPVSCLRKWLGKRTATSIIIF
CFLIQVTICIVWLSTFPPFPDFGVISMAEEISLECNEGSVSMFYCVLGFMGFLSICSFTSAFFARKLPDSFNEAKFITISMLVFCSVWISFVPAYLSSKGKHMVAVEIFSILASSAGLLGFIFFPKCYIIVMRPDMNIRKQLIKRKH-
>Anole_lizard.locus104.1
CFQCPEDHYPNKKRDFCIPKYIIFLGFEDPFGTGLASFALFLFCVTVVVLGIFLKHRDTPIVKANNRTLTYTLLLSLLLSFLCALLFIGKPQKMTCLLRQTAFGIIFSVAISSVLAKTITVILAFQATKPESKWRKWLGKRMATSIVLS
SLLIQATICTVWLSTFPPFPDLDVNSMAEEIILECNEGSITMFYCLLGFMGFLSICSFTSAFFARKLPDSFNEAKFITFSMLVFCSVWISFVPTYLSSKGKYMVAVEIFSILASSAGLLGFIFFPKCYIIILRPDMNTKEQVMKRKH-
>Anole_lizard.locus103.1
CFQCPEDHYPNKKRDFCIPKSLTFLTFEEALGTSLASSALLLFCVTSVVLGIFLKHRDTPIVKANNRTLTYTLLLSLLLSFLCALLFIGKPQKMTCLLRQTAFGIIFLVAISSVLAKTITVILAFQATKPECMWRKWLGKRMATSIVLS
SLLIQATICTVWLSTFPPFPDLDVNSMAEEVILKCNEGSITMFYCLLGFMGFLSICSFTSAFFARKLPDSFNEAKFITFSMLVFCSVWISFVPTYLSSKGKYMVAVEIFSILASSAGLLGFIFFPKCYIIILRPDMNTKEQVMKRKH-
>Anole_lizard.locus100.1
CFQCPEDHYPNKKRDFCIPKSLTFLTFEETLGRSLASTALFLFCVTAVVLWIFLKHRDTPIVKANNRTLTYTLLLSLLLSFLCALLFIGKPQKMTCLFRQTAFGIIFSVAISSVLAKTITVILAFQATKPESKWRKWLGKRMATSIVLS
SLLIQATICTVWLSTFPPFPDLDVNSMAEEVILKCNEGSITMFYCLLGFMGFLFICSFTSAFFARKLPDSFNEAKFITFSMLVFCSVWISFVPTYLSSKGKYMVAVEIFSILASSAGLFGFIFFPKCYIIILRPDMNTKEKIMKRKN-
>Anole_lizard.locus105.1
CFQCPEDHYPNKKRDFCIPKSLTFLTFEEALGTSLASSALFLFCVTSVVLWIFLKHRDTPIVKANNRTLTYTLLLSLLLSFLCALLFIGKPQKMTCLFRQTAFGIIFSVAISSVLAKTITVILAFQATKPESKLRKWLGKRMATSIVLS
SLLIQTTICTVWLSTFPPFPDLDVNSMAEEVILKCNEGSITMFYCLLGFMGFLSICSFTSAFFARKLPDSFNEAKFITFSMLVFCSVWISFVPTYLSSKGKYMVAVEIFSILASSAGLLGFIFFPKCYIIILRPDMNTKEQVMKRKH-
>Western_clawed_frog.locus458.1
CLPCSWDTWPNLQQDRCLPRPTEFLSYGDPLGYSLAAASVFSSLTPLFILGVFYFFKKTPIVRANNYSLSCLLLLSLFLCFLCSLGFIGYPQPEKCLLRQVAFGMVFALCISCVLAKTITVVIAFNATKPGSRLRKWTGVKVSSSIIIV
CTSIDFCLCVIWVIYFPPFSELDTYTKPGFIIVSCNEGSPTAFWCMLGYLGFLASISFIVAFLARRLPDSFNEAKLITFSMLAFLSVWVSFIPAYLSARGMYTVAMEVFAILSSSWAVVGCIFVSKCFIVLFRPNLNSREHLMGRGRR
>Western_clawed_frog.locus497.1
--------------------------------
MATMSVSSSAVPLGILSIFIVYQTTPIVRANNYSLSCLLLLSLFLCFLCSLGFIGYPQPEKCLLRQVAFGMVFALCISCVLAKTITVVIAFNATKPGSRLRKWTGVKVSKSVVMFCTCIQFSICVLWVSFSPPFPEQDTKTQPGVIIYS
CNEGSPFAFWIMLGYLGLLASISFIVAFLARRLPDSFNEAKLITFSMLAFLSVWVSFIPAYLSARGMYTVAMEVFAILSSSWAVVGCIFVPKCFIVLFRPNMNSREHLMGKGQR
>Western_clawed_frog.locus449.1
CHPCSWDTWPNLQQDRCLPRPTEFLSYGDPLGYTLAAIAILSSLIPAAILGVFICFKKTPIVRANNYSLSCLLLLSLFLCFLCSLGFIGYPQPEKCLLRQVAFGMVFALCISCVLAKTITVVIAFNATKPGSRLRKWTGVKLSCSIIIL
SSFIQLCICGMWLIFSPSFPELDTDTKPGIIILNCNEGSPTAFWCMLGYLGLLASISFIVAFLARRLPDSFNEAKLITFSMLAFLSVWVSFIPAYLSARGMYTVAMEVFAILSSSWAVVGCIFVPKCFIVLFRPNMNSREHLTIKG--
>Western_clawed_frog.locus493.1
CHPCSWDTWPNLQQDRCLPRPTEFLSYGDPLGYSLTAFSVFSSLIPLAILGVFICYKKTPIVRANNYSLSCLLLLSLFLCFLCSLGFIGYPQPEKCLLRQVAFGMVFALCISCVLAKTITVVIAFNATKPGSRLRKWTGVRVSYWITVF
GTLIQLFVCVMWLIFSPPFPELDTITSPGVIIVTCNEGSPTAFWCMLGYLGLLASISFIVAFLARRLPDSFNEAKLITFSMLAFLSVWVSFIPAYLSARGMYTVAMEVFAILSSSWAVVGCIFVPKCLIVMFRPNMNSRELLMGKRSK
>Western_clawed_frog.locus167.1
------
MEWPNEWRNQCFSKTEEFLSYNDVISVVLSSLSVFFFFLTLLIFGLFITHRDSPIVRANNRSLSFLLLVSIKLSFLSVFLFLGRPVDITCMLRIITFGITFSIAVSSLLAKTIMVCVAFKATKPGSSWRKWLGVKLSNSVVLFCSSIQI
IICMTWLAISPPFQELDIHTSHGTIIIQCNEGSAIGFYSVIGYMGLLAAVSFVLAFLARSLPDSFNEAKYITFSMLLFCSVWITMIPAYLSTKGKNTVCVEIFAILSSSAGLLASIFLPKCYIILLKPEMNTKQYLLGNNK-
>Western_clawed_frog.locus152.1
--------------------
MEFLSYDDSLTVAFILLSLVFFIIAAVILGIFISYRDSPIVRANNRSLSFLLLVSIKLSFLSVFLFLGRPVDITCMLRIITFGITFSIAVSSLLAKTIMVCVAFKATKPGSSWRKWLGVKLSNSVVLFCSSIQIIICMTWLAISPPFQE
LDIHTSPGTIIIQCNEGSAIGFYSVIGYMGLLAAVSFVLAFLARSLPDSFNEAKYITFSMLLFCSVWITMIPAYLSTKGKNTVCVEIFAILTSSAGLLASIFLPKCYIILLKPEMNTKEYLLGNNK-
```

```
>Western_clawed_frog.locus151.1
--------------------
MEFLSYDDSLTVAFILLSLVFFIIAAVILGIFISYRDSPIVRANNRSLSFLLLVSIKLSFLSVFLFLGRPVDITCMLHIITFGITFSIAVSSLLAKTIMVCVAFKATKPGSSWRKWLGVKLSNSVVLFCSSIQIIICMTWLAISPPFQE
LDIHTSPGTIIIQCNEGSAIGFYSVIGYMGLLAAVSFVLAFLARSLPDSFNEAKYITFSMLLFCSVWITMIPAYLSTKGKNTVCVEIFAILTSSAGLLASIFLPKCYIILLKPEMNSKQCLLGNNK-
>Western_clawed_frog.locus249.1
--------------------------------------
MFFLIAAVILGIFILYRDTPIVRANNRSLSFLLLVSIKLSFLSVFLFLGRPVDITCMLRIITFGITFSIAVSSLLAKTIMVCVAFKATKPGSSWRKWLGVKLSNSVVLFCSSIQIIICMTWLAISPPFQELDIHTSPGTIIIQCNEGSA
IGFYSVIGYMGLLAAVSFVLAFLARSLPDSFNEAKYITFSMLLFCSVWITMIPAYLSTKGKNTVCVEIFAILTSSAGLLACIFLPKCYIILLKPEMNTKQYLLGNNKE
>Western_clawed_frog.locus100.1
CIKCHIYEWPNEEKTMCIEKQTEFLSYDDSLTLVFIVLSLVSVLLVTVILGIFISFRDTPVVRANNHTLSFLLLVSIKLSFLSVFLFLGRPVDITCMLRIITFGITFSIAVSSLLAKTIMVCVAFKATKPGSSWRKWLGVKLSNSVVLF
CSSIQIIICMTWLAISPPFQELDIHTSPGTIIIQCNEGSAIGFYSVIGYMGLLAAVSFVLAFLARSLPDSFNEAKYITFSMLLFCSVWITMIPAYLSTKGKYMVAVEIFAIISSSCGLLFCIFLPKCYIIILKPEMNTKQYLLGNNK-
>Western_clawed_frog.locus695.1
CFRCPGYEMSNKQRTACVPKMINYLSYEETLGASLASIALVLFLTTSAVQGVFVKYWETPIVRANNQHLSCLLLISLMLCFLCTLLFIGRPTQICCLLRQVTFGIVFTISVSSVLAKTLTVIIAFNATKPGSKLKKCIGSQVPIVFVTI
CSLGKIGICIVWMAFDPPFLKADMFSERNTIILQCNEGSVTFFFCIIGYIGTLALLSFIVAFLAKDFPDRFNEAKNITFSMLGFCSVWGAFVPAYLSSKGSRMVAVEIFAILSSSAGLLGCIFAPKCYIIFLRPELNTKVTACQIH--
>Western_clawed_frog.locus632.1
CFRCPGYEMSNKQRTACVPKMINYLSYEETLGASLASIALVLFLTTSAVLGIFVKYWETPIVRANNQHLSCLLLISLMLCFLCTLLFIGRPTQICCLLRQVTFGIVFTISVSSVLAKTLTVIIAFNATKPGSKLKKCIGSQVPIVFVTI
CSLGKIGICIVWMAFDPPFLKADMFSERNTIILQCNEGSVTFFFCIIGYIGTLALLSFIVAFLAKDFPDRFNEAKNITFSMLGFCSVWGAFVPAYLSSKGSRMVAVEIFAILSSSAGLLGCIFAPKCYIIFLRPELNTKVTACQIH--
>Western_clawed_frog.locus627.1
CFRCPGYEMSNKQRTACVPKMINYLSYEETLGASLASIALVLFLTTSAVQGVFVKYWETPIVRANNRNLSCLLLISLMLCFLCTLLFIGRPTQICCLLRQVTFGVVFTISVSSVLAKTLTVIIAFNATKPGSKLKKCISSQVPIVFVTI
CSLGKIGICLIWMAFDPPFLEVDIFSERNTIILQCNEGSVTFFFCIIGYIGTLALLSFIAAFLAKDFPDRFNEAKNITFSMLGFCSVWGAFVPAYLSSKGSRMVAVEIFAILSSSAGLLGCIFAPKCYIIFLRPQLNTKVTVCRIH--
>Western_clawed_frog.locus698.1
CFRCPGYEMSNKQRTACVPKMINYLSYEETLGASLASIALVLFLTTSAVQGVFVKYWETPIVRANNRNLSCLLLISLMLCFLCTLLFIGRPTQICCLLRQVTFGVVFTISVSSVLAKTLTVIIAFNATKPGSKLKKCISSQVPIVFVTI
CSLGKIGICLIWMAFDPPFLEVDIFSERNTIILQCNEGSVTFFFCIIGYIGTLALLSFIAAFLAKDFPDRFNEAKNITFSMLGFCSVWGAFVPAYLSSKGSRMVAVEIFAILSSSAGLLGCIFAPKCYIIFLRPQLNTKVTVCRIH--
>Western_clawed_frog.locus626.1
CLKCPGYEMSNKQRTACVPKMINYLSYEETLGASLASIALVLFLTTSAVLGIFVKYWETPIVRANNQNLSCLLLISLMLCFLCTLLFIGRPTQICCLLRQVTFGIVFTISVSSVLAKTLTVIIAFNATKPGSKLKKCIGSQVPIVFVTI
CSLGKIGICLIWMMFHPPFLEVDILSERDTIILQCNEGSVTFFFCIIGYIGTLALLSFIAAFLAKDFPDRFNEAKNITFSMLGFCSVWGAFVPAYLSSKGSRMVAVEIFAILFSSAGLLGCIFIPKCYIIFLRPELNTRVAVNQNH--
>Western_clawed_frog.locus696.1
CFRCPGYEMSNEQRTACVPKMINYLSYEETLGASLASIALVLFLTTSAVQGVFVKNWDTPIVRANNQNLSCLLLISLMLCFLCTLLFIGRPTQICCLLRQVTFGIVFTISVSSVLAKTLTVIIAFNATKPGSKLKKCIGSQVPIVFVTI
CSLGEIGICLIWMTFDPPFLETDILSERDTIILQCNEGSVTFFFCIIGYIGTLALLSFIAAFLAKDFPDRFNEAKNITFSMLGFCSVWGAFVPAYLSSKGSRMVAVEIFAILSSSAGLLGCIFAPKCYIIFLRPQLNTKVIA---Q--
>Western_clawed_frog.locus631.1
CFRCPGYEMSNEQRTACVPKMINYLSYEETLGASLASIALVLFLTTSAVQGVFVKNWDTPIVRANNQNLSCLLLISLMLCFLCTLLFIGRPTQICCLLRQVTFGIVFTISVSSVLAKTLTVIIAFNATKPGSKLKKCIGSQVPIVFVTI
CSLGEIGICLIWMTFDPPFLETDILSERDTIILKCNEGSVTFFFCIIGYIGTLALLSFIAAFLAKDFPDRFNEAKNITFSMLGFCSVWGAFVPAYLSSKGSRMVAVEIFAILSSSAGLLGCIFAPKCYIIFLRPQLNTKVIA---Q--
>Western_clawed_frog.locus628.1
--------
MSNKQRTACVPKMINYLSYEETLGASLASIALVLFLTTSAVQGVFVKYWETPIVRANNRYLNCLLLISLMLCFLCTLLFIGRPTQICCLLRQVTFGVVFTISVSSVLAKTLTVIIAFNATKPGSKLKKYVGTQLAIILVIICSLGEIGI
CLIWMTFDPPFLEVDILSERDTIILQCNEGSVTFFFCIIGYIGTLALLSFIAAFLAKDFPDRFNEAKNITFSMLGFCSVWGAFVPAYLSSKGSRMVAVEIFAILSSSAGLLGCIFAPKCYIIFLRPQLNIKVTA-QNK--
>Western_clawed_frog.locus629.1
CFRCPGYEMSNKQRTACVPKMINYLSYEETLGASLASIALVLFLTTSAVQGVFVKYWKTPIVRANNRNLSCLLLISLMLCFLCTLLFIGRPTQICCLLRQVTFGIVFTISVSSVLAKTLTVIIAFNATKPGSKLKKYVGTQLPIVLVTI
CSLGEIGICLIWMASDPPFVEVDILSERDTIILQCNEGSVTFFFCIIGYIGTLALLSFIAAFLAKDFPDRFNEAKNITFSMLGFCSVWGAFVPAYLSSKGSRMVAVEIFAILSSSAGLLGCIFAPKCYIIFLRPQLNTKVIA---Q--
>Western_clawed_frog.locus702.1
CFRCPGYEKSNKQRTACVPKMINYLSYGETLGASLASIALVLFLTTSAVQGVFVKYWETPIVRANNRYLSCLLLISLMLCFLCTLLFIGRPTQICCLLRQVTFGVVFTISVSSVLAKTLTVIIAFNATKPGSKLKKYVGTQLPIVLVTF
CSLGKIGICIVWMTYDPPFLEADILSERDTITLQCNEGSGYFFFCIIGYIGTLALLSFIAAFLAKDFPDRFNEAKNITFSMLGFCSVWGAFVPAYLSSKGSRMVAVEIFAILSSSAGLLGCIFAPKCYIIFLKPGLNTRVAAARNY--
>Western_clawed_frog.locus622.1
CFRCPGYEKSNKQRTACVPKMINYLSYGETLGASLASIALVLFLTTSAVQGVFVKYWETPIVRANNRYLSCLLLISLMLCFLCTLLFIGRPTQICCLLRQVTFGVVFTISVSSVLAKTLTVIIAFNATKPGSKLKKYVGTQLPIVLVTF
CSLGKIGICIVWMTYDPPFLEADILSERDTITLQCNEGSGYFFFCIIGYIGTLALLSFIAAFLAKDFPDRFNEAKNITFSMLGFCSVWGAFVPAYLSSKGSRMVAVEIFAILSSSAGLLGCIFAPKCYIIFLKPGLNTRVAAARNY--
>Western_clawed_frog.locus614.1
CFRCPGYEMSNKQRTACVPKMINYLSYEETLGASLASIALVLFLTTSAVQGVFVKYWETPIVRANNQNLSCLLLISLMLCFLCTLLFIGRPTQICCLLRQVTFGIVFTISVSSVLAKTLTVIIAFNATKPGSKLKKYVGTQLAIVLVTI
CSLGSTVISAVWMASHPPFLEADTVSEMDTIILKCNEGSVTFFFCIIGYIGTLALLSFIAAFLAKDFPDRFNEAKNITFSMLGFCSVWGAFVPAYLSSKGSRMVAVEIFAILASSAGLLGCIFAPKCYIIFHRPELNTRVAISCSN--
>Western_clawed_frog.locus710.1
CFRCPGYEMSNKQRTACVPRMKNYLSYEETLGASLASIALVLFLTTSIVLGIFVKYWETPIVRANNQHLSCLLLISLMLCFLCTLLFIGRPTQICCLLRQVTFGIVFTISVSSVLAKTLTVIIAFNATKPGSKLKKYVGTQLAIVLVII
CSMGSTVISAVWMASHPPFLEADTVSEMDTIILKCNEGSVTFFFCIIGYIGTLALLSFIAAFLAKDFPDRFNEAKIITFSMLGFCSVWGAFVPAYLSSKGSRMVAVEIFAILSSSAGLLGCIFAPKCYIIFHRPELNRRVAISCSN--
>Western_clawed_frog.locus621.1
CFRCPGYEMSNKQRTACVPKMINYLSYEETLGASLASIALVLFLTASAVQGVFVKYWETPIVRANNQNLSCLLLISLMLCFLCTLLFIGRPTQICCLLRQVTFGIVFTISVSSMLAKTLTVIIAFNATKPGSKLKKYVGTQLATILVIV
CSLGTIGISTVWMFSNPPFLEVDMFSKMDTIILQCNEGSVTFFFCIIGYIGTLALLSFIAAFLAKDFPDRFNEAKNITFSMLGFCSVWGAFVPAYLSSKGSRMVAVEIFAILSSSAGLLACIFAPKCYIIFLRPELNTRVAAARNY--
>Western_clawed_frog.locus703.1
CFRCPGYEMSNEQRTACVPKMINYLSYEETLGASLASIALVLFLTTSAVQGVFVKYWETPIVRANNQNLSCLLLISLMLCFLCTLLFIGRPTQICCLLRQVTFGIVFTISVSSVLAKTLTVIIAFNATKPGSKLKKYVGTQLAIVLVII
CSLGTIGISTVWMASNPPFLEADMFSEMDTIILKCNEGSVTFFFCIIGYMGTLALLSFIAAFLAKDFPDRFNEAKNITFSMLGFCSVWGAFVPAYLSSKGSRMVAVEIFAILSSSAGLLACIFAPKCYIIFLRPELNTRVAAARNY--
>Western_clawed_frog.locus704.1
--------
MSNKERTACVSKFINYLSYEDTLGASLASVALMLFLTTSTVQGIFVKYWETPIVRANNQNLSCLLLISLMLCFLCTLLFIGRPTQICCLLRQVTFGIVFTISVSSVLAKTLTVIIAFNATKPGSKLKKYVGTQLAIVLVIICSLGKIGI
STMWMASCPPFLEADMFSEMDTIILKCNEGSVTFFFCIIGYIGTLALLSFIAAFLAKDFPDRFNEAKNITFSMLGFCSVWGAFVPAYLSSKGSRMVAVEIFAILSSSAGLLGCIFAPKCYIIFIRPELNTRVVVVRNQ--
>Western_clawed_frog.locus732.1
--------
MSNINRTACNPKTMNYLSYKDTLGASLASFALVLFLTTSTVQGTLLKYWETPIVRANNQNLSCLLLISLMLCFLCTLLFIGRPTQICCLLRQVTFGIVFTISVSSVLAKTLTVIIAFNATKPGSKLKKYVGTQLPIVLVIMCTLGKIGI
STVWVASYPPFLEADMFSKMDTIILQCNEGSVTFFFCIIGYIGTLALLSFIAAFLAKDFPDRFNEAKNITFSMLGFCSVWGAFVPAYLSSKGSRMLAVEIFAILSSSAGLLGCIFLPKCFIIFSRPEMNTKKLLS-----
>Western_clawed_frog.locus584.1
CFKCSEYLMSNINRTACNPKTMNYLSYEETLGASLASIALVLFLTTSAVQGIFVKYWDTPIVRANNQNLSCLLLISLMLCFLCTLLFIGRPTQICCLLRQVTFGIVFTISVSSVLAKTLTVIIAFNATKPGSKLTKYVGTQLSILFVIM
CTLGTIGISTVWVASNPPFLEADMFSKMDTIILLCNEGSVTFFFCIIGYIGTLALLSFIAAFLAKDFPDRFNEAKNITFSMLGFCSVWGAFVPAYLSSKGSRMVAVEIFAILSSSARLLGCIFLPKCFIIFLRPEMNTKKAVVLK---
>Western_clawed_frog.locus699.1
--------
MSNIRRTGCISKTMNYLSYKDALGASLASIALVLFLTTSAVQGIFVKYWETPIVRANNQNLSCLLLISLKLCFLCSLLFIGHPTQISCCLRQVTFGIVFTISVSSVLAKTLTVIIAFNATKPGSKLTKYVGTQLSILFVIMCTLGTTGI
STVWVASYPPFLEADMFSEMDTIILLCNEGSVTFFFCIIGYIGTLALLSFIAAFLAKDFPDRFNEAKNITFSMLGFCSVWGAFVPAYLSSKGSRMVAVEIFAILSSSAGLLGCIFGPKCYIIFFRHELNTRATVVLKL--
>Western_clawed_frog.locus625.1
--------
MSNIRRTGCISKTMNYLSYKDALGASLASIALVLFLTTSAVQGIFVKYWETPIVRANNQNLSCLLLISLKLCFLCSLLFIGHPTQISCCLRQVTFGIVFTISVSSVLAKTLTVIIAFNATKPGSKLTKYVGTQLSILFVIMCTLGTTGI
STVWVASYPPFLEADMFSEMETIILLCNEGSVTFFFCIIGYIGTLALLSFIAAFLAKDFPDRFNEAKNITFSMLGFCSVCGAFVPAYLSSKGSRMVAVEIFAILSSSAGLLGCIFGPKCYIIFFRHEQNTRATVVLKL--
>Western_clawed_frog.locus624.1
--------
MSNIRRTGCISKTVNFLSYKDTLGASLASIALVLFLTTSAVQGIFVKYWETPIVRANNQNLSCLLLISLMLCFLCTLLFIGRPTQICCLLRQVTFGIVFTISVSSVLAKTLTVIIAFNATKPGSKLKKYVGTQLAILLVLMCTLGKIGI
SVVWMVSNPPFLEADTFSEMDTIILLCNEGSVTFFFCIIGYIGTLALLSFIAAFLAKDFPDRFNEAKNITFSMLGFCSVWGAFVPAYLSSKGSRMVAVEIFAILASSAGLLACIFVPKCFIIFFRPKLNKRANVLK----
>Western_clawed_frog.locus623.1
--------
MSNKQRTVCIPKSVNYLSFEDTLGASLASIALVLFLTTSVVQGTFIKYWETPIVRANNQHLSCLLLISLMLCFLCTLLFIGRPTQICCLLRQVTFGIVFTISVSSVLAKTLTVIIAFNATKPGSKLKKYVGTQLAILLVLMCTLGKIGI
SVVWMASNPPFLEADTFSEMDTIILLCNEGSVAFFFCIIGYMGTLALLSFIAAFLAKDFPDRFNEAKNITFSMLGFCSVWGAFVPAYLSSKGSRMVAVEIFAILSSSAGLLACIFLPKCFIIFFRPEINTRETVVLKR--
>Western_clawed_frog.locus701.1
--------
MSNKQRTVCIPKSVNYLSFEDTLGASLASIALVLFLTTSVVQGTFIKYWETPIVRANNQHLSCLLLISLMLCFLCTLLFIGRPTQICCLLRQVTFGIVFTISVSSVLAKTLTVIIAFNATKPGSKLKKYVGTQLAILLVLMCTLGKIGI
SVVWMASNPPFLEADTFSEMDTIILLCNEGSVAFFFCIIGYMGTLALLSFIAAFLAKDFPDRFNEAKNITFSMLGFCSVWGAFVPAYLSSKGSRMVAVEIFAILSSSAGLLACIFLPKCFIIFFRPEINTRETVVLKR--
>Western_clawed_frog.locus616.1
--------
MPDRKRTVCIPKATNYLSYTDTMGTSLTAIAFILFITASVVLGIFVKYWETPIVRANNQNLSCLLLISLMLCFLCTLLFIGRPTQICCLLRQVTFGVVFTISVSSVLAKTLTVIIAFNATKPGSKLKKYVGTQLPIILVIVCSFEEMLI
STVWMVSNPPFLEADMFSEMDTIVLLCNEGSVTFFFCIIGYIGTLALLSFIAAFLAKDFPDRFNEAKNITFSMLGFCSVWGAFVPAYLSSKGSRMVAVEIFAILSSSAGLLACIFAPKCYIIYLRPELNSRKTTIRK---
>Western_clawed_frog.locus708.1
--------
MPDRKRTVCIPKATNYLSYTDTMGTSLTAIAFILFITASVVLGIFVKYWETPIVRANNQNLSCLLLISLMLCFLCTLLFIGRPTQICCLLRQVTFGVVFTISVSSVLAKTLTVIIAFNATKPGSKLKKYVGTQLAIILVIVCSFEEMLI
STVWMASNPPFLEADMFSEMDTIVLLCNEGSVTFFFCIIGYIGTLALLSFIAAFLAKDFPDRFNEAKNITFSMLGFCSVWGAFVPAYLSSKGSRMVAVEIFAILSSSAGLLGCIFAPKCYIIYLRPELNSRKTTIRK---
>Western_clawed_frog.locus706.1
--------
MPDRKRTACIPKATNYLSYIDTLGTSLTAIAFILFITASVVLGIFVKYWETPIVRANNQNLSCLLLISLMLCFLCTLLFIGRPTQICCLLRQVTFGIVFTISVSSVLAKTLTVIIAFNATKPGSKLKKYVGTQLAIVLVIVCSFGEMLI
STVWMASNPPFLEADMFSEMDTIVLLCNEGSVTFFFCIIGYIGTLALLSFIAAFLAKDFPDRFNEAKNITFSMLGFCSVWGAFVPAYLSSKGSRMVAVEIFAILASSAGLLGCIFAPKCYIIYLRPELNSRKTTIRK---
>Western_clawed_frog.locus615.1
--------
MPDTKRIVCIPKATNYLSFIDTLGASLTAIAFILFITASVVLGIFVKYWETPIVRANNRYLSCLLLISLMLCFLCTLLFIGRPTQICCLLRQVTFGIVFTISVSSVLAKTLTVIIAFNATKPGSKLKKYVGTQLAIVLVIVCCLGEIII
STVWMASNPPLVEADMVSKMDTIVLLCNEGSVAFFFCIIGYIGTLALLSFIAAFLAKDFPDRFNEAKNITFSMLGFCSVWGAFVPAYLSSKGSRMVAAEIFAILSSSAGLLACIFGPKCYIIYLRPELNSRKTTIRKY--
>Western_clawed_frog.locus709.1
--------
MPDTKRIVCIPKATNYLSFIDTLGASLTAIAFILFITASVVLGIFVKYWDTPIVRANNRYLSCLLLISLMLCFLCTLLFIGRPTQICCLLRQVTFGIVFTISVSSVLAKTLTVIIAFNATKPGSKLKKYVGTQLAIVLVIVCCLGEIII
STVWMVSNPPLVEADIVSKMDTIVLLCNEGSVAFFFCIIGYIGTLALLSFIAAFLAKDFPDRFNEAKNITFSMLGFCSVWGAFVPAYLSSKGSRMVAAEIFAILSSSAGLLGCIFGPKCYIIYLRPELNSRKTTIRKY--
>Western_clawed_frog.locus618.1
--------
MPDRKRTACIPKATNYLSYIDTLGASLTAIAFILFITASVVLGIFVKYWETPIVRANNRYLSCLLLISLMLCFLCTLLFIGRPSQICCLLRQVTFGVVFTISVSSVLAKTLTVIIAFNATKPGSKLKKYVGTQLAIVLVIVCSFGEMLI
STVWMASNPPFLEADMFSEMDTIVLLCNEGSVTFFFCIIGYMGTLALLSFIAAFLAKDFPDRFNEAKNITFSMLGFCSVWGAFVPAYLSSKGSRMVAVEIFAILASSAGLLGCIFAPKCYIIYLKPELNSRKTTIRK---
>Western_clawed_frog.locus735.1
CERCSEYQKSNTERTECLLKAINFLSYTDTMGASLTVIAFILFITASVVLGIFVKYWETPIVRANNQNLSCLLLISLMLCFLCTLLFIGRPTQICCLLRQVTFGIVFTISVSSVLAKTLTVIIAFNATKPGSKLKKYVGTQLAIVLVII
CSLGSTGISAVWMASYPPFLEADTVSEMETVILMCNEGSVSLFFCSIGYMGTLALLSFIAAFLAKDFPDRFNEAKNITFSMLGFCSVWGAFVPAYLSSKGSRMVAVEIFAILASSAGLLGCIFAPKCYIIFLGPDLNKRTF-------
>Western_clawed_frog.locus581.1
CERCSEYQKSNTERTECLLKAINFLSYTDTMGASLTVIAFILFITASVVLGIFVKYWETPIVRANNQHLSCLLLISLMLCFLCTLLFIGRPTQICCLLRQVTFGTVFTISVSSVLAKTLTVIIAFNATKPGSKLKKYVGTQLAIVLVII
CSLGSTGISAVWMASYPPFLEADTVSEMETVILMCNEGSVSLFFCSIGYMGTLALLSFIAAFLAKDFPDRFNEAKNITFSMLGFCSVWGAFVPAYLSSKGSRMVAVEIFAILASSAGLLGCIFAPKCYIIFLGPDLNKRTF-------
```

```
>Western_clawed_frog.locus583.1
------------------------------
MGASLTAIAFILFITASVVLGIFVKYWETPIVRANNQHLSCLLLISLMLCFLCTLLFIGRPTQICCLLRQVTFGIVFTISVSSVLAKTLTVIIAFNATKPGSKLKKYVGTQLAIVLVIICSLGSTGISAVWMASHPPFLEADTVSEMDT
VILMCNEGSVFLFFCSIGYMGTLALLSFIAAFLAKDFPDRFNEAKNITFSMLGFCSVWGAFVPAYLSSKGSRMVAVEICAILASSAGLLGCIFAPKCYIIFLRPELNKRTL-------
>Western_clawed_frog.locus578.1
------------------------------
MGASLTAIAFILFITASVVLGIFVKYWETPIVRANNQHLSCLLLISLMLCFLCTLLFIGRPTQICCLLRQVTFGVVFTISVSSVLAKTLTVIIAFNATKPGSKLKKYVGTQLAIVLVIICSLGSTVISAVWMASHPPFLEADTVSEMDT
VILMCNEGSVFFFFSAIGYIGTLALLSFIAAFLAKDFPDRFNEAKNITFSMLGFCSVWGAFVPAYLSSKGSRMVAVEIFAILASSAGLLGCIFTPKCYIIFLRPELNTRTF-------
>Western_clawed_frog.locus741.1
CESCSEYQKSNTERTECLLKAINFLSYTDTMGASLTAIAFILFITASVVLGIFVKYWETPIVRANNQHLSCLLLISLMLCFLCTLLFIGRPTQICCLLRQVTFGVVFTISVSSVLAKTLTVIIAFNATKPGSKLKKYVGTQLAIVLVII
CSLGSTVISAVWMASHPPFLEADTVSEMDTVILMCNEGSVFFFFSAIGYIGTLALLSFIAAFLAKDFPDRFNEAKNITFSMLGFCSVWGAFVPAYLSSKGSRMVAVEIFAILASSAGLLGCIFAPKCYIIFLRPELNTRTF-------
>Western_clawed_frog.locus619.1
CERCSEYQKSNTERTECLLKAINFLSYTDTMGASLTAIAFILFITASVVLGIFVKYWETPIVRANNQNLSCLLLISLMLCFLCTLLFIGRPTQICCLLRQVTFGIVFTISVSSVLAKTLTVIIAFNATKPGSKLKKYVGTQLAIVLVII
CSLGSTGISAVWMASHPPFLEADTVSEMDTVFLMCNEGSVLFFFCAIGYMTALALFSFIAAFLAKDFPDRFNEAKNITFSMLGFCSVWGAFVPAYLSSKGSRMVAVEIFAILSSSAGLLGCIFAPKCYIIFLQPELNTRTF-------
>Western_clawed_frog.locus705.1
CERCSEYQKSNTERTECLLKAINFLSYTDTMGASLTAIAFILFITASVVLGIFVKYWETPIVRANNQNLSCLLLISLMLCFLCTLLFIGRPTQICCLLRQVTFGIVFTISVSSVLAKTLTVIIAFNATKPGSKLKKYVGTQLAIVLVII
CSLGSTGISAVWMASHPPFLEADTVSEMDTVFLMCNEGSVLFFFCAIGYMTALALFSFIAAFLAKDFPDRFNEAKNITFSMLGFCSVWGAFVPAYLSSKGSRMVAVEIFAILSSSAGLLGCIFAPKCYIIFLQPELNTRTF-------
>Western_clawed_frog.locus736.1
------------------------------
MGASLTAIAFILFITASVVLGIFVKYWETPIVRANNQHLSCLLLISLMLCFLCTLLFIGRPSQICCLLRQVTFGIVFTISVSSVLAKTLTVIIAFNATKPGSKLKKYVGTQLAIVLVIICSLGSTGISAVWMASHPPFLEADTVSEMDT
VILMCNEGSVFFFFCAIGYMGAPAVLSFIAAFLAKDFPDRFNEAKNITFSMLVFCSVWGAFVPAYLSSKGSRMVAVEIFAILSSSAGLLGCIFAPKCYIIFLQPELNTRTF-------
>Western_clawed_frog.locus580.1
------------------------------
MGASLTAIAFILFITASVVLGIFVKYWDTPIVRANNRYLSCLLLISLMLCFLCTLLFIGRPTQICCLLQQVTFGVVFTISVSSVLAKTLTVIIAFNATKPGSKLKKYVGTQLAIVLVIICSLGSTVISAVWMASHPPFLEADTVSEMDT
VILMCNEGSVTFFFCIIGYIGTLALLSFIAAFLAKDFPDRFNEAKNITFSMLGFCSVWGAFVPAYLSSKGSRMVAVEIFAILSSSAGLLGCIFAPKCYIIFLQPELNTKTF-------
>Western_clawed_frog.locus737.1
CERCSEYQKSNTERTECLLKAINFLSYTDTMGASLTAIAFILFITASVVLGIFVKYWETPIVRANNRYLSCLLLISLMLCFLCTLLFIGRPTQICCLLRQVIFGVVFTTSVSSVLAKTLTVIIAFNATKPGSKLKKYVGTQLAIVLVII
CSLGSTVISAVWMASHPPFLEADTVSEMDTVILTCNEGSVFFFFCIIGYIGTLALLSFIAAFLAKDFPDRFNEAKNITFSMLGFCSVWGAFVPAYLSSKGSRMVAVEIFAILSSSAGLLGCIFAPKCYIIFLQPELNTKTF-------
>Western_clawed_frog.locus734.1
CERCSEYQKSNTERTECLLKAINFLSYTDTMGASLTAIAFILFITASVVLGIFVKYWETPIVRANNQHLSCLLLISLMLCFLCTLLFIGRPTQICCLLRQVTFGIVFTISVSSVLAKTLTVIIAFNATKPGSKLKKYVGTQLAIVLVII
CSLGSTGISAVWMASHPPFLEADTVSEMDTVILLCNEGSVTFFFCIIGYIGTLALLSFIAAFLAKDFPDRFNEAKNITFSMLGFCSVWGAFVPAYLSSKGSRMVAVEIFAILSSSAGLLACIFAPKCYIIFLQPELNKRTL-------
>Western_clawed_frog.locus739.1
CERCSEYQKSNTERTECLLKAINFLSYTDTMGASLTAIAFILFITASVVLGIFVKYWETPIVRANNQNLSCLLLISLMLCFLCTLLFIGRPTQICCLLQQVTFGIVFTISVSSVLAKTLTVIIAFNATKPGSKLKKYVGTQLAIVLVII
CSLGTTGISAVWMASHPPFLEADTVSEMDTVILLCNEGSVTFFFCIIGYIGTLALLSFIAAFLAKDFPDRFNEAKNITFSMLGFCSVWGAFVPAYLSSKGSRMVAVEIFAILSSSAGLLGCIFAPKCYIIFLQPELNTKTF-------
>Western_clawed_frog.locus731.1
CSKCPEDRKSNLQRTDCIPKTINYISYMDSMGFGLTTITLILFFAVYAVFTIFLKYWHTPIVRANNQNLSCLLLISLMLCSLCTLLFIGRPTQICCLLRQVTFGVVFTISVSSVLAKTLTVIIAFNSTKPGSKLKKYVGTQLSFILVIV
CSLGEMGISVGWVASYPPYVEADMISEKDIIILQCNEGSVTFFFCIIGYIGTLALLSFIAAFLAKDFPDRFNEAKNITFSMLGFCSVWVAFVPAYLSSKGSRMVAVEIFAILASSSGLLGCIFVPKCYIIYIKPELNKRETRALVF--
>Western_clawed_frog.locus585.1
CSKCPEDRKSNLQRTDCIPKTINYISYMDSMGFGLTTITLILFFAVYAVFTIFLKYWHTPIVRANNQNLSCLLLISLMLCFLCTLLFIGRPTQICCLLRQVTFGVVFTISVSSVLAKTLTVIIAFNSTKPGSKLKKYVGTQLSFILVIV
CSLGEMGISVAWVASYPPYVEADMISEKDIIILQCNEGSVAFFFCIIGYIGTLALLSFIAAFLAKDFPDRFNEAKNITFSMLGFCSVWVAFVPAYLSSKGSRMVAVEIFAILASSSGLLGCIFVPKCYIIYIKPELNKRETRALVF--
>Western_clawed_frog.locus586.1
---------------------------
MDSMGFGLTTITLILFFAVYAVFTIFLKYWHTPIVRANNQHLSCLILISLMLCSLCTLLFIGRPTQICCLLRQITFGIVFTISVSSVLAKTLTVIIAFNATKPGSKLKKYVGTQLAIILVIVCCLAEMGISVGWVASYPPYVEADMISE
KDIIILQCNEGSVAFFFCIIGYIGTLALLSFIAAFLAKDFPDRFNEAKNITFSMLGFCSVWGAFVPAYLSTKGSRMVAVEIFAILSSSAGLLGCIFLPKCFIIFLRPEMNTKKLLS-----
>Western_clawed_frog.locus592.1
-MKCPEDQKPNRQRTDCVPKAINYLSYMDTLGASLASIALVFSITASVVLGISVKYWETPIVRANNQNLSCLLLISLMLCFLCTLLFIGRPTQICCLLRQVTFGIVFTISVSSVLAKTLTVIIAFNATKPGSKLKKYVGTQLSVLVVFT
CSSGETAISVAWIVSSPPFPDTDTSFETD-IFLQCNEGSVTFFFCIIGYIGTLALLSFIAAFLAKDFPDRFNEAKNITFSMLGFCSVWGAFVPAYLSSKGSRMVAVEIFAILSSSAGLLICIFAPKCYIIFIRPELNKKENCCASKRS
>Western_clawed_frog.locus589.1
--------
MSNWQKTDCVPKALNYLSYMDTLGASLTAIAFILFITASAVQGIFVKYWETPIVRANNQNLSCLLLISLMLCFLCTLLFIGRPTQICCLLRQVTFGVVFTISVSSVLAKTLTVIIAFNATKPGSKLTMYVGTQLSITLVLVCSLGETGI
SAAWMASNPPFLEADTSSETDTIILQCNEGSVTFFFCIIGYIGTLALLSFIAAFLAKDFPDRFNEAKNITFSMLGFCSVWGAFVPAYLSSKGSRMVAVEIFAILSSSAGLLGCIFAPKCYIIFIRPELNKKRKCCST---
>Western_clawed_frog.locus609.1
CVKCPEDQKSNRQKTDCVPKALNFLSYMDTLGASLASAAIILFITTSVVMGIFVKYWETPIVRANNQNLSCLLLISLMLCFLCTLLFIGRPTQICCLLRQVTFGIVFTISVSSVLAKTLTVIIAFNATKPGSKLKKYVGTQLAIVLVII
CSLGSTGISAVWMASYPPFLEVDMFSEMDTIILLCNEGSVTFFFCIIGYIGTLALLSFIAAFLAKDFPDRFNEAKNITFSMLGFCSVWGAFVPAYLSSKGSRMVAVEIFAILSSSAGLLGCIFAPKCYIIFLRPELNIREIVARKA--
>Western_clawed_frog.locus715.1
CVKCPEDQKSNRQKTDCVPKALNYLSYMDTLGASLASAAIILFITTSVVLGIFVKYWDTPIVRANNQNLSCLLLISLMLCFLCTLLFIGRPTQICCLLRQVTFGIVFTISVSSVLAKTLTVIIAFNATKPGSKLKKYVGTQLAIVLVII
CSLGSTGISAVWMASYPPFLEVDMFSEMDTIILLCNEGSVTFFFCIIGYIGTLALLSFIAAFLAKDFPDRFNEAKNITFSMLGFCSVWGAFVPAYLSSKGSRMVAVEIFAILSSSAGLLGCIFAPKCYIIFLRPELNIREIVAIKA--
>Western_clawed_frog.locus726.1
CVKCPEDQKSNRQKTDCVPKALNYLSYMDTLGASLASTAIILFITTSVVMGIFVKYWDTPIVRASNRYLSCLLLISLMLCFLCTLLFIGRPTKIFCLVRQAAFGIIFIISVSTVLAKTLTVIIAFNATKPGSKLKKYVGTQLAILVVSA
CFSVVTLICIVWMASSPPFPETDTYSETDTIILLCNEGSVAFFFCIIGYMGTLALLSFIAAFLAKDFPDRFNEAKNITFSMLGFCSVWGAFVPAYLSSKGSRMVAVEIFAILSSSAGLLGCMFAPKLYIIFLRPELNTREIVVKRS--
>Western_clawed_frog.locus598.1
CVKCPEDQKSNRQKTDCVPKALNYLSYMDTLGASLASTAIILFITTSVVMGIFVKYWDTPIVRASNRYLSCLLLISLMLCFLCTLLFIGRPTKIFCLVRQAAFGIIFIISVSTVLAKTLTVIIAFNATKPGSKLKKYVGTQLAILVVSA
CFSVVTLICIVWMASSPPFPETDTYSETDTIILLCNEGSVTFFFCIIGYIGTLALLSFIAAFLAKDFPDRFNEAKNITFSMLGFCSVWGAFVPAYLSSKGSRMVAVEIFAILSSSAGLLGCMFAPKLYIIFLRPELNTREIVVKRS--
>Western_clawed_frog.locus727.1
CVKCPEDQKSNRQKTDCVPKALNYLSYMDTLGASLASAAIILFITASVVLGIFVKYWDTPIVRASNRNLSCLLLISLMLCFLCTLLFIGRPTKIFCLVRQAAFGIIFIISVSTVLAKTLTVIIAFNATKPGSKLKKYVGTQLAIFIVSA
CSLCVTLICVVWMASSPPFPEADTYSETDTIILLCNEGSVTFFFCIIGYIGTLALLSFIAAFLAKDFPDRFNEAKNITFSMLGFCSVWGAFVPAYLSSKGSRMVAVEIFAILSSSAGLLICIFAPKCFIIFLRPELNTREIVVKRS--
>Western_clawed_frog.locus597.1
CVKCPEDQKSNRQKTDCVPKALNYLSYMDTLGASLASAAIILFITASVVLGIFVKYWDTPIVRASNRNLSCLLLISLMLCFLCTLLFIGRPTKIFCLVRQAAFGIIFIISVSTVLAKTLTVIIAFNATKPGSKLKKYVGTQLAIFIVSA
CSLCVTLICVVWMASSPPFPEADTYSETDTIILLCNEGSVTFFFCIIGYIGTLALLSFIAAFLAKDFPDRFNEAKNITFSMLGFCSVWGAFVPAYLSSKGSRMVAVEIFAILSSSAGLLICIFAPKCFIIFLRPELNTREIVVKRS--
>Western_clawed_frog.locus594.1
CVKCPEDQKSNRQKTDCVPKALNYLSYMDTLGASLASTAIILFITTSVVMGIFVKYWDTPIVRASNQNLSCLLLISLMLCFLCTLLFIGRPTKIICLVRQATFGIIFMISVSTVLAKTLTVIIAFNATKPGSKLKKYVGTQLSILVISA
SFLGVTLICIVWMASSPPFPEADNYSETDTIILLCNEGSVIFFFCIIGYIGTLALLSFIAAFLAKDFPDQFNEAKNITFSMLGFCSVWGAFVPAYLSSKGSRMVAVEIFAILSSSAGLLACIFAPKCYIIFFQPEMNIREIVVRKTSP
>Western_clawed_frog.locus608.1
CVKCPEDQKSNRQKTDCVPKALNYLSYMDTLGASLASAAIILFITTSVVLGIFVKYWETPIVRANNQHLSCLLLISLMLCFLCTLLFIGRPTKIICLVRQAAFGIIFTISVSTVLAKTLTVIIAFNATKPGSKLKKYVGTQLAILVVSA
CSLGETVICVVWMASSPPFPEDDTLSDPDYIILLCNEGSGYFFFCIIGYIGTLALLSFIAAFLAKDFPDRFNEAKNITFSMLGFCSVWGAFVPAYLSSKGSRMVAVEIFAILSSSAGLLGCIFAPKCYIIFHRPELNIRETVARKA--
>Western_clawed_frog.locus716.1
CVKCPEDQKSNRQKTDCVPKALNYLSYMDTLGASLASAAIILFITTSVVLGIFVKYWETPIVRANNQHLSCLLLISLMLCFLCTLLFIGRPTKIICLVRQAAFGIIFTISVSTVLAKTLTVIIAFNATKPGSKLKKYVGTQLAILVVSA
CSLGETVICVVWMASSPPFPEDDTLSDPDYIILLCNEGSGSFFFCIIGYIGTLALLSFIAAFLAKDFPDRFNEAKNITFSMLGFCSVWGAFVPAYLSSKGSRMVAVEIFAILSSSAGLLGCIFAPKCYIIFHRPELNIRETVARKA--
>Western_clawed_frog.locus590.1
---------------------------MDTLG------
AIILFITTSAVLGIFVKYLETPIVRANNQNLSCLLLISLMLCFLCTLLFIGHPTQICCLIRQVTFGIVFTISVSSVLAKTLTVIIAFNATKPGSKLKKYVGTQLAILVVSACFSVVTLICVVWMASSPPFPEADTSSETDTIILLCNEG
SVTFFFCIIGYMGTLALLSFIAAFLAKDFPDRFNEAKNITFSMLGFCSVWGAFVPAYLSSKGSRMVAVEIFAILASSAGLLGCIFAPKCYIIFLDQR-------------
>Western_clawed_frog.locus729.1
---------------------------
MDTLGASLASAAIILFITTSAVLGIFVKYWETPIVRANNQNLSCLLLISLMLCFLCTLLFIGHPTQICCLIRQVTFGIVFTISVSSVLAKTLTVIIAFNATKPGSKLKKYVGTQLAILVVSACFSVVTLICVVWMASSPPFPEADTSSE
TDTIILLCNEGSVTFFFCIIGYMGTLALLSFIAAFLAKDFPDRFNEAKNITFSMLGFCSVWGAFVPAYLSSKGSRMVAVEIFAILASSAGLLGCIFAPKCYIIFFRPEMNIREIVVRKTSP
>Western_clawed_frog.locus603.1
CVKCPEDQKSNRQKTDCIHKALNYLSYMDTLGASLASTAIILFITTSVVLGIFVKYWETPIVRANNQNLSCLLLISLMLCFLCTFLFIGRPTQICCLLRQVTFGIVFTISVSSVLAKTLTVIIALNATKPGSKATRYVGTQMSIFIVSA
CSLGVTLICVVWMASSPPFPEADTSSENDTIILLCNEGSVTFFFCIIGYIGTLALLSFIAAFLAKDFPDRFNEAKNITFSMLGFCSVWGAFVPAYLSSKGSRMVAVEIFAILSSSAGLLGCIFSPKLYIIFLRPELNIRGSVVRRS--
>Western_clawed_frog.locus721.1
CVKCPEDQKSNRQKTDCVPKALNYLSYMDTLGASLASAAIILFITTSVVMGIFVKYWETPIVRANNQHLSCLLLISLMLCFLCTLLFIGRPTQICCLLRQVTFGIVFTISVSSVLAKTLTVIIAFNATKPGSNATRYVGTQMSIFIVFA
CSLGVTLICIIWMASSPPFPEADTSSETDTIILLCNEGSVTFFFCIIGYIGTLALLSFIAAFLAKDFPDRFNEAKNITFSMLGFCSVWGAFVPAYLSSKGSRMVAVEIFAILSSSAGLLGCICAPKLYIIFLRPELNIRGSVVRRT--
>Western_clawed_frog.locus596.1
CVKCPEDQKSNRQKTDCVPKALNYLSYMDTLGASLASAAIILFITASVVMGIFVKYWETPIVRANNQNLSCLLLISLMLCFLCTLLFIGRPTQICCLLRQVTFGIVFTSSVSSVLAKTLTVIIAFNATKPGSKATRYVGTQLSIFIVSA
CSLGVTLICVVWMASSPPFPDADTFSETDTIILLCNEGSIIFFFCIIGYIGTMALLSFIAAFLAKDFPDRFNEAKNITFSMLGFCSVWGAFVPAYLSSKGSRMVAVEIFAILSSSAGLFGCIFAPKLYIIFLRPELNIRETVVKRS--
>Western_clawed_frog.locus722.1
CVKCPEDQKSNRQKTDCVPKALNYLSYMDTLGASLASAAIILFIKTSAVLGIFVKYWETPIVRANNQHLSCLLLISLMLCFLCTLLFIGHPTKIFCLVRQVTFGIIFTISVSSVLAKTLTVIIAFNATKPGSKATRYVGTQMSILVVFA
CSLGVTLICIIWMASSPPFPDVDTSSETDTIILLCNEGSVAFFFCIIGYIGTLALLSFIAAFLAKDFPDRFNEAKNITFSMLGFCSVWGAFVPAYLSSKGSRMVAVEIFAILSSSAGLLICIFAPKCYIIFLRPKLNIREIVIRRT--
>Western_clawed_frog.locus602.1
---------------------------
MDTLGASLASTAIILFIKTSAVLGIFVKYWETPIVRANNQNLSCLLLISLMLCFLCTLLFIGRPTQICCLLRQVTFGIVFTISVSSVLAKTLTVIIAFNATKPGSKAARYVGTQMSILVVFACSLGVTLICIIWMASSPPFPETDTSSE
TDTIILLCNEGSVAFFFCIIGYIGTLALLSFIAAFLAKDFPDRFNEAKNITFSMLGFCSVWGAFVPAYLSSKGSRMVAVEIFAILSSSAGLLICIFAPKCYIIFLRPKLNIREIVIRRT--
>Western_clawed_frog.locus728.1
CDKCPEDHKSNRQKTDCVPKALNYLSYMDTLGASLASAAIILFITTSVVMGIFVKYWETPIVRANNQHLSCLLLISLMLCFLCTLLFIGRPTQICCLLRQVTFGIVFTISVSSVLAKTLTVIIAFNATKPGSKATRYVGTQLSIFIVFA
CSLGVTLICIVWMASSPPFPETDTSSETDTIILLCNEGSVTFFFCIIGYIGTLALLSFIAAFLAKDFPDRFNEAKNITFSMLGFCSVWGAFVPAYLSSKGSRMVAVEIFAILSSSAGLLICIFAPKCFIIFLRPELNIRETVVKRS--
>Western_clawed_frog.locus605.1
CVKCPEDQKSNRQKTDCVPKALNYLSYMDTLGASLASAAIILFITTSVVMGVFVKYWETPIVRANNQNLSCLLLISLMLCFLCTLLFIGRPTQICCLLRQVTFGIVFTISVSSVLAKTLTVIIAFNATKPGSKATWFVGTQLSIFIVSA
CSLGVTLISVVWMASSPPFPEADASSETDTIILLCKEGSIVFFFCIIGYIGTLALLSFIAAFLAKDFPDRFNEAKNITFSMLGFCSVWGAFVPAYLSSKGSRMVAVEIFAILSSSAGLLGCIFAPKCYIIFLRPELNIREIVARKA--
>Western_clawed_frog.locus717.1
CEKCPEDQKSNRQKTDCVPKALNYLSYMDTLGASLASTAIILFITTSVVMGIFVKYWDTPIVRANNQNLSCLLLISLMLCFLCTLLFIGRPTQICCLLRQVTFGIVFTSSVSSVLAKTLTVIIAFNATKPGSKLKKYVGSQLSIVLVIA
CSLGVTLICVVWMASSPPFPEADTSYETDIIILLCNEGSVTFFFCIIGYIGTLALLSFIAAFLAKDFPDRFNEAKNITFSMLGFCSVWGAFVPAYLSSKGSRMVAVEIFAILSSSAGLLGCIFAPKCFIIFLRPELNIREIVVRKA--
>Western_clawed_frog.locus607.1
CEKCPEDQKSNRQKTDCVPKALNYLSYMDTLGASLASTAIILFITTSVVMGIFVKYWDTPIVRANNQNLSCLLLISLMLCFLCTLLFIGRPTQICCLLRQVTFGIVFTSSVSSVLAKTLTVIIAFNATKPGSKLKKYVGSQLSIVLVIA
CSLGVTLICVVWMASSPPFPEADTSYETDIIILLCNEGSVTFFFCIIGYIGTLALLSFIAAFLAKDFPDRFNEAKNITFSMLGFCSVWGAFVPAYLSSKGSRMVAVEIFAILSSSAGLLGCIFAPKCFIIFLRPELNIREIVVRKA--
>Western_clawed_frog.locus730.1
---------------------------MDTLGASLASAAIILFITTSVVMGIFGKYCGEPITKIS----
AVSLLISLMLCSSGTLLFIGRPTQICCLLRQVTFGIVFTISVSSVLAKTLTVIIAFNATKPGSKLTLYVGTQLAMLVVSACSLGETVISVVWMASCPPFPDADTSSKMDTIVLLCNEGSELFFFCIIGYIGTLALLSFIAAFLAKDFPD
RFNEAKNITFSMLGFCSVWGAFVPAYLSSKGSRMVAVEIFAILSSSAGLLICIFVPKCYIIFKRPELNKKRKFCST---
```

```
>Western_clawed_frog.locus588.1
---------------------------
MDTLGASLASAAIILFITTSVVMGIFVKYWDTPIVRANNQNLSCLLLISLMLCFLCTLLFIGRPTQICCLLRQVTFGIVFTISVSSVLAKTLTVIIAFNATKPGSKLTLYVGTQLAMLVVSACSLGETVISVVWMASCPPFPDADTSSK
MDTIVLLCNEGSELFFFCIIGYIGTLALLSFIAAFLAKDFPDRFNEAKNITFSMLGFCSVWGAFVPAYLSSKGSRMVAVEIFAILSSSAGLLICIFVPKCYIIFKRPELNKKRKFCS-T--
>Western_clawed_frog.locus593.1
---------------------------
MDTLGASLASAAIILFITTSAVMGIFVKYWETPIVRANNQNLSCLLLISLMLCFLCTLLFIGRPTQICCLLRQVTFGIVFTISVSSVLAKTLTVIIAFNATKPGSKLKKYVGTQLSILVVSACSLGETVISVVWMASSPPFPDADTSSK
MDTIVLMCNEGSNTFFFCIIGYIGTLALLSFIAAFLAKDFPDRFNEAKNITFSMLGFCSVWGAFVPAYLSSKGSRMVAVEIFAILSSSAGLLICIFVPKCYIIFKRPELNKMRKICS-T--
>Western_clawed_frog.locus725.1
---------------------------
MDTLGASLASAAIILFITASVVLGIFVKYWETPIVRANNQNLSCLLLISLMLCFLCTLLFIGRPTQICCLLRQVTFGIIFTISVSSVLAKTLTVIIAFNATKPGSKLTMYVGTQLAILVVSACSLGETVISVVWMASSPPFPEADTSSK
MDTIILLCNEGSNFFFFCIIGYIGTLALLSFIAAFLAKDFPDRFNEAKNITFSMLGFCSMWGAFVPAYLSSKGSRMVAVEIFAILSSSAGLLICIFAPKCYIIFLRPELNIREIVVRRS--
>Western_clawed_frog.locus599.1
---------------------------
MDTLGASLASAAIILFITTSAVLGIFVKYWETPIVRANNQNLSCLLLISLMLCFLCTLLFIGRPTQICCLLRQVTFGIIFTISVSSVLAKTLTVIIAFNATKPGSKLTMYVGTQLAILVVSACSLGETVISVVWMASSPPFPEADTSSK
MDTIILLCNEGSNFFFFCIIGYIGTLALLSFIAAFLAKDFPDRFNEAKNITFSMLGFCSMWGAFVPAYLSSKGSRMVAVEIFAILSSSAGLLICIFAPKCYIIFLRPELNIREIVVRRS--
>Western_clawed_frog.locus723.1
CVKCPEDQKSNRQKTDCVPKALNYLSYMDTLGASLASTAIILFITASVVMGIFVKYWETPIVRANNQNLSCLLLISLMLCFLCTLLFIGRPTQICCLLRQVTFGIVFTISVSSVLAKTLTVIIAFNATKPGSKLKKYVGTQLAIVLVIV
CSLGETVISVVWMASSPPFPEADTSSKMDTIVLLCNEGSNFFFFCIIGYIGTLALLSFIAAFLAKDFPDRFNEAKNITFSMLGFCSVWGAFVPAYLSSKGSRMVAVEIFAILSSSAGLLGCIFAPKCYIIFLRPDLNIREIVVRRT--
>Western_clawed_frog.locus601.1
CVKCPEDQKSNRQKTDCVPKALNYLSYMDTLGASLASAAIILFITASVVMGIFVKYWDTPIVRANNQNLSCLLLISLMLCFLCTLLFIGRPTQICCLLRQVTFGIVFTISVSSVLAKTLTVIIAFNATKPGSKLKKYVGTQLAIVLVIV
CSLGETVISVVWMASSPPFPEADTSSKMDTIVLLCNEGSNFFFFCIIGYIGTLALLSFIAAFLAKDFPDRFNEAKNITFSMLGFCSVWGAFVPAYLSSKGSRMVAVEIFAILSSSAGLLGCIFAPKCYIIFLRPDLNIREIVVRRT--
>Western_clawed_frog.locus718.1
CVKCPEDQKSNRQKTDCVPKALNYLSYMDTLGASLASAAIILFITTSAVFRIFVKYWETPIVRANNQNLSCLLLISLMLCFLCTLLFIGRPTQICCLLRQVTFGIVFTVSVSSVLAKTLTVIIAFNATKPGSKLKKYVGTQLSIILVIV
CSLVEFLISAVWLAYNPPFPKADTVSDPDYIILLCNEGSELFFFCIIGYIGTLALLSFIAAFLAKDFPDRFNEAKNITFSMLGFCSVWGAFVPAYLSSKGSRMVAVEIFAILSSSAGLFGCIFAPKCYIIFLRPELNIREIVVRKS--
>Western_clawed_frog.locus606.1
CVKCPEDQKSNRQKTDCVPKALNYLSYMDTLGASLASAAIILFITTSAVFRIFVKYWDTPIVRANNQNLSCLLLISLMLCFLCTLLFIGRPIQICCLLRQVTFGIVFTVSVSSVFAKTLTVIIAFNATKPGSKLKKYVGTQLSIILVIL
CSLVEFVISAVWLAYNPPFPEADTVSDPDYIILLCNEGSELFFFCIIGYIGTLALLSFIAAFLAKDFPDRFNEAKNITFSMLGFCSAWGAFVPAYLSSKGSRMVAVEIFAILSSSAGLLGCIFAPKCYIIFLRPELNIREIVVRKS--
>Western_clawed_frog.locus600.1
CVKCPEDQKSNRQKTDCVPKALNYLSYMDTLGASLASAAIILFITTSVVLGIFVKYWDTPIVRASNQHLSCLLLISLMLCFLCTLLFIGRPTQICCLLRQVTFGIVFTISVSSVLAKTLTVIIAFNATKPGSKLKKYVGTQLAIILVIV
CSLGEIVISAVWLAYNPPFPEADTVSDPDYIILLCNEGSELFFFCIIGYIGTLALLSFIAAFLAKDFPDRFNEAKNITFSMLGFCSVWGAFVPAYLSSKGSRMVAVEIFAILSSSAGLLICIFAPKCYIIFLRPHLNIREIVVRRT--
>Western_clawed_frog.locus720.1
CVKCPEDQKSNRQKTDCVPKALNYLSYMDTLGASLASAAIILFITASVVLGIFVKYWETPIVRANNQHLSCLLLISLMLCFLCTLLFIGRPTQICCLLRQVTFGIVFTISVSSVLAKTLTVIIAFNATKPGSKLKKYVGTQLAIILVIV
CSLGEIVISAVWLAYNPPFPEADTVSDPDYIILLCNEGSELFFLCIIGYIGTLALLSFIAAFLAKDFPDRFNEAKNITFSMLGFCSVWGAFVPAYLSSKGSRMVAVEIFAVLSSSAGLLGCIFAPKCYIIFLRPELNIREIVARKA--
>Western_clawed_frog.locus604.1
CVKCPEDQKSNRQKTDCVPKALNYLSYMDTLGASLASAAIILFITASVVMGIFVKYSETPIVRANNQNLSCLLLISLMLCFLCTLLFIGRPTQICCLLRQVTFGIVFTISVSSVLAKTLTVIIAFNATKPGSKLKKYVGTQLATILVIV
CSLGEIVISAVWLAYNPPFPEADTVSDPDYIILLCNEGSELFFFCIIGYMGTLALLSFIAAFLAKDFPDRFNEAKNITFSMLGFCSVWGAFVPAYLSSKGSRMVAVEIFAVLSSSAGLLGCIFAPKCYIIFLRPELNIREIVASKS--
>Western_clawed_frog.locus591.1
CTKCPEDQKSNRQKTDCVPKAINYLSYMDTLGASLASAAIILFITASVVMGIFVKYWETPIVRANNQHLSCLLLISLMLCFLCTLLFIGRPTQICCLLRQVIFGIVFTISVSSVLAKTLTVIIAFNATKPGSKLKKYVGTQLAIILVIV
CSLVEIVMSAVWLASNPPFPEADTLSDPNYIILLCNEGSGFFFFCIIGYIGTLALLSFIAAFLAKDFPDRFNEAKNITFSMLGFCSVWGAFVPAYLSSKGSRMVAVEIFAILSSSAGLLICIFTPKCYIIFIRPELNKR---------
>Western_clawed_frog.locus587.1
CTKCPEDQKSNRQRTDCVPKAINYLSYMDTLGASLAFASIILFITASVVLGIFVKYWETPIVRANNQHLSFLLLISLMLCFLCTLLFIGRPTQICCLLRQVTFGIVFTTSVSSVLAKTLTVIIAFNATKPGSKLKKYVGTQLAIVLVIV
CSLVEIVISAVRLAFNPPFPEADTFSDPDYIILLCNEGSDFFFFCIIGYIGTLALLSFIAAFLAKDFPDRFNEAKNITFSMLGFCSVWGAFVPAYLSSKGSRMVAVEIFAILSSSAGLLICIFTPKCYIIFIRPELNKR---------
>Western_clawed_frog.locus760.1
CFRCSEYEKSNKQRTGCVPKEINYLSYTDTLGASLTSIALVLFIAASVVLGIFVRYWETPIVRANNQHLSFLLLISLMLCFLCTLLFIGRPTQICCLLRQVTFGIVFTISVSSVLAKTLTVIIAFNATKPGSKLKKYVGTQLATILVIV
CCLGEMMISAVWMASNPPFLDADTLTDINTVFLMCNEGSVLFFFSVIGYMTALALFSFIAAFLAKDFPDRFNEAKNITFSMLGFCSVWGAFVPAYLSSKGSRMVAVELFAILSSSAGLLACIFVPKCYIIFFKPGLNKRKM-------
>Western_clawed_frog.locus761.1
CFRCSEYEKSNKQRTGCVPKEINYLSYTDTLGASLTSIALVLFIAASVVLGIFVRYWETPIVRANNQHLSFLLLISLMLCFLCTLLFIGRPTQICCLLRQVTFGIVFTISVSSVLAKTLTVIIAFNATKPGSKLKKYVGTQLATILVIV
CCLGEMMISAVWMASNPPFLDADTLTDINTVFLMCNEGSVLFFFSVIGYMTALALFSFIAAFLAKDFPDRFNEAKNITFSMLGFCSVWGAFVPAYLSSKGSRMVAVELFAILSSSAGLLACIFVPKCYIIFFKPGLNKRKM-------
>Western_clawed_frog.locus759.1
CFRCSEYEKSNKQRTGCVPKEINYLSYTDTLGASLTSIALVLFIAASVVLGIFVRYWETPIVRANNQHLSFLLLISLMLCFLCTLLFIGRPTQICCLLRQVTFGIVFTISVSSVLAKTLTVIIAFNATKPGSKLKKYVGTQLATILVTV
CCLGEMMISAVWMAFNPPFLDADTLTDINTVFLMCNEGSVLFFFSVIGYMTALALFSFIAAFLAKDFPDRFNEAKNITFSMLGFCSVWGAFVPAYLSSKGSRMVAAEIFAILSSSAGLLACIFVPKCYIIFFKPGLNKRKM-------
>Western_clawed_frog.locus762.1
CFLCSEYEKSNKQRTGCVPKEINYLSYTDTLGASLTSIALVLFIAASVVLGIFVRYWETPIVRANNQHLSFLLLISLMLCFLCTLLFIGRPTQICCLLRQVTFGIVFTISVSSVLAKTLTVIIAFNATKPGSKLKKYVGTQLATILVIV
CCLGEMMISAVWMASNPPFLDADTLTDINTVFLMCNEGSVLFFFSVIGYMTALALFSFIAAFLAKDFPDRFNEAKNITFSMLGFCSVWGAFVPAYLSSKGSRMVAAEIFAILSSSAGLLACIFVPKCYIIFFKPELNKRKM-------
>Western_clawed_frog.locus765.1
CFKCFDYEKSNKQRTGCVPKEINYLSYTDTLGASLTSIALVLFIAASVVLGIFVRYWETPIVRANNQNLSFLLLISLMLCFLCTLLFIGRPTQICCLLRQVTFGIVFTISVSSVLAKTLTVIIAFNATKPGSKLKKYVGTQLATILVIV
CCLGEMMISAVWMASNPPFLDADTLTDINTVFLMCNEGSVLFFFSVIGYMTALALFSFIAAFLAKDFPDRFNEAKNITFSMLGFCSVWGAFVPAYLSSKGSRMVAAEIFAILSSSAGLLACIFVPKCYIIFFKPEQNKRKM-------
>Western_clawed_frog.locus757.1
CFRCSEYEKSNKQRTGCVPKEINYLSYTDTLGATLTSIALVLFIAASVVLGIFVRYWETPIVRANNQHLSFLLLISLMLCFLCTLLFIGRPTQICCLLRQVTFSIIFTISVSTVLAKTLTVIIAFNATKPGSKLKKYVGTQLATILVTV
CCLGEMMISAVWMASNPPFLDADTLTDINTVFLMCNEGSVLFFFSVIGYMTALALFSFIAAFLAKDFPDRFNEAKNITFSMLGFCSVWGAFVPAYLSSKGSRMVAVEIFAILSSSAGLLACIFVPKCYMIFFKPEQNKRKM-------
>Western_clawed_frog.locus755.1
CFRCSEYEKSNKQRTGCVPKEINYLSYTDTLGASLTSIALVLFIAASVVLGIFVRYWETPIVRANNQHLSFLLLISLMLCFLCTLLFIGRPTQICCLLRQVTFGIVFTISVSSVLAKTLTVIIAFNATKPGSKLKKYVGTQLATILVIV
CCLGEMMISAVWMASNPPFLDADTLTDINTVFLMCNEGSVLFFFSVIGYMTALALFSFIAAFLAKDFPDRFNEAKNITFSMLGFCSVWGAFVPAYLSSKGSRMVAVELFAILSSSAGLLACIFVPKCYIIFFKPEQNRRKM-------
>Western_clawed_frog.locus772.1
CFRCSEYEKSNKQRTGCVPKEINYLSYTDTLGASLTSIALVLFIAASVVLGIFVRYWETPIVRANNQHLSFLLLISLMLCFLCTLLFIGRPTQICCLLRQVTFGIVFTISVSSVLAKTLTVIIAFNATKPGSKLKKYVGTQLATILVIV
CCLGEMMISAVWMASNPPFLDADTLTDINTVFLMCNEGSVLFFFSVIGYMTALALFSFIAAFLAKDFPDRFNEAKNITFSMLGFCSVWGAFVPAYLSSKGSRMVAVEIFAILSSSAGLLACIFVPKCYIIFLKPEQNRRRM-------
>Western_clawed_frog.locus754.1
CFRCSEYEKSNKQRTGCVPKEINYLSYTDTLGASLTSIALVLFIAASVVLGIFVRYWETPIVRANNQHLSFLLLISLMLCFLCTLLFIGRPTQICCLLRQVTFGIVFTISVSSVLAKTLTVIIAFNATKPGSKLKKYVGTQLATILVTV
CCLGEMMISAVWMASNPPFLDADTLTDINTVFLMCNEGSVLFFFSVIGYMTALALFSFIAAFLAKDFPDRFNEAKNITFSMLGFCSVWGAFVPAYLSSKGSRMVAVEIFAILSSSAGFLACIFVPKCYIIFFKPEQNRRRM-------
>Western_clawed_frog.locus763.1
CFKCSDYQRSNKQRTGCVPKEINYLSYTDTLGASLTSIALVLFIAASVVLGIFVRYWETPIVRANNQNLSILLLISLMLCFLCTLLFIGRPTQICCLLRQVTFGIVFTISVSSVLAKTLTVIIAFNATKPGSKLKKYVGTQLATILVTV
CCLGEMMISAVWMASNPPFLDADTLTDINTVFLMCNEGSVLFFFSVIGYMTALALFSFIAAFLAKDFPDRFNEAKNITFSMLGFCSVWGAFVPAYLSSKGSRMVAVEIFAILSSSAGLLACIFVPKCYIIFFKPEQNRRKM-------
>Western_clawed_frog.locus769.1
CFRCSEYEKSNKQRTGCVPKEINYLSYTDTLGASLTSIALVLFIAASVVLGIFVRYWETPIVRANNQNLSFLLLISLMLCFFCTLLFIGRPTQICCLLRQVTFGIVFTISVSSVLAKTLTVIIAFNATKPGSKLKKYVGTQLATILVTV
CCLGEMMISAVWMASNPPFLDADTLTDINTVFLMCNEGSVLFFFSVIGYMTALALFSFIAAFLAKDFPDRFNEAKNITFSMLGFCSVWGAFVPAYLSSKGSRMVAAEIFAILSSSAGLLACIFVPKCYIIFLKPEQNRRRM-------
>Western_clawed_frog.locus770.1
CFTCSEYEKSNKQRTGCVPKEINYLSYTDTLGASLTSIALVLFIAASVVLGIFVRYWETPIVRANNQNLSFLLLISLMLCFLCTLLFIGRPTQICCLLRQVTFGIVFTISVSSVLAKTLTVIIAFNATKPGSKLKKYVGTQLAIILVIV
CCLGEMMISAVWMASNPPFLDADTLTDINTVFLMCNEGSVLFFFSVIGYMTALALFSFIAAFLAKDFPDRFNEAKNITFSMLGFCSVWGAFVPAYLSSKGSRMVAVELFAILSSSAGLLACIFVPKCYIIFFKPEQNRRRM-------
>Western_clawed_frog.locus780.1
CLHCSDYEKSNKQRTGCVPKEINYLSYTDTLGASLTSIALVLFIAASVVLGIFVRYWETPIVRANNQHLSFLLLISLMLCFLCTLLFIGRPTQICCLLRQVTFGIVFTTSVSSVMAKTLTVIIAFNATKPGSKLKKYVGTQLATILVIV
CCLGEMMISAVWMASNPPFLDADTLTDINTVFLMCNEGSVLFFFSVIGYMTALALFSFIAAFLAKDFPDRFNEAKNITFSMLGFCSVWGAFVPAYLSSKGSRMVAAEIFAILSSSAGLLACIFVPKCYIIIFIPEQNRRRM-------
>Western_clawed_frog.locus779.1
CLHCSEYEKSNKQRTGCVPKEINYLSYTDTLGASLTSIALVLFIAASVVLGIFVRYWETPIVRANNQHLSFLLLISLMLCFLCTLLFIGRPTQICCLLRQVTFGIVFTTSVSSVMAKTLTVIIAFNATKPGSKLKKYVGTQLATILVTV
CCLGEMMISAVWMASNPPFLDADTLTDINTVFLMCNEGSVLFFFSVIGYMTALALFSFIAAFLAKDFPDRFNEAKNITFSMLGFCSVWGAFVPAYLSSKGSRMVAAEIFAILSSSAGLLACIFVPKCYIIFFIPEQNRRRM-------
>Western_clawed_frog.locus766.1
CLHCSDYEKSNKQRTGCVPKEINYLSYTDTLGASLTSIALVLFIAASVVLGIFVRYWETPIVRANNQHLSFLLLISLMLCFLCTLLFIGRPTQICCLLRQVTFGIVFTISVSSVLAKTLTVIIAFNATKPGSKLKKYVGTQLATILVIV
CCLGEMMISAVWMASNPPFLDADTLNDINTVFLMCNEGSVLFFFSVIGYMTALALFSFIAAFLAKDFPDRFNEAKNITFSMLGFCSVWGAFVPAYLSSKGSRMVAAEIFAILSSSAGLLACIFVPKCYIIFFKPEQNKRKI-------
>Western_clawed_frog.locus767.1
CLHCSDYEKSNKQRTGCVPKEINYLSYTDTLGASLTSIALVLFIAASVVLGIFVRYWETPIVRANNQHLSFLLLISLMLCFLCTLLFIGRPTQICCLLRQLTFGTVFTISVSSVLAKTLTVIIAFNATKPGSKLKKYVGTQLATILVIV
CCLGEMMISAVWMASNPPFLDADTLTDINTVFLMCNEGSVLFFFSVIGYMTALALFSFIAAFLAKDFPDRFNEAKNITFSMLGFCSVWGAFVPAYLSSKGSRMVAVEIFAILSSSAGLLACIFVPKCYIIFFKPEQNRRRM-------
>Western_clawed_frog.locus774.1
CLHCSEYEKSNKQRTGCVPKEINYLSYTDTLGASLTSIALVLFIAASVVLGIFVRYWETPIVRANNQHLSFLLLISLMLCFLCTLLFIGRPTQICCLLRQVTFGIVFTISVSSVLAKTLTVIIAFNATKPGSKLKKYVGTQLATILVTV
CCLGEMMISAVWMASNPPFLDADTLTDINTVFLMCNEGSVLFFFSVIGYMTALALFSFIAAFLAKDFPDRFNEAKNITFSMLGFCSVWGAFVPAYLSSKGSRMVAVEIFAILSSSAGLLACIFVPKCYIIFFKPEQNRRRM-------
>Western_clawed_frog.locus771.1
CLHCSEYEKSNKQRTGCVPKEINYLSYTDTLGASLTSIALVLFIAASVVLGIFVRYWETPIVRTNNQHLSFLLLISLMLCFLCTLLFIGRPTQICCLLRQVTFGIVFTISVSSVLAKTLTVIIAFNATKPGSKLKKYVGTQLATILVTV
CCLGEMMISAVWMASNPPFLDADTLTDINTVFLMCNEGSVLFFFSVIGYMTALALFSFIAAFLAKDFPDRFNEAKNITFSMLGFCSVWGAFVPAYLSSKGSRMVAVEIFAILSSSAGLLACIFVPKCYIIFLKPEQNRRRM-------
>Western_clawed_frog.locus776.1
CLHCSEYEKSNKQRTGCVPKEINYLSYTDTLGASLTSIALVLFIAASVVLGIFVRYWETPIVRANNQHLSFLLLISLMLCFLCTLLFIGRPTQICCLLRQVTFGIVFTISVSSVLAKTLTVIIAFNATKPGSKLKKYVGTQLATILVIV
CCLGEMMISAVWMASNPPFLDADTLIDINTVFLMCNEGSVLFFFSVIGYMTALALFSFIAAFLAKDFPDRFNEAKNITFSMLGFCSVWGAFVPAYLSSKGSRMVAVEIFAILSSSAGLLACIFVPKCYIIFFKPEQNRRRM-------
>Western_clawed_frog.locus773.1
CLHCSEYEKSNKQRTGCVPKEINYLSYTDTLGASLTSIALVLFIAASVVLGIFVRYWETPIVRANNQNLSFLLLISLMLCFLCTLLFIGRPTQICCLLRQVIFGIVFTISVSSVLAKTLTVIIAFNATKPGSKLKKYVGTQLAIILVIV
CCLGEMMISTVWMASNPPFLDADTLTDINTVFLMCNEGSVLFFFSVIGYMTALALFSFIAAFLAKDFPDRFNEAKNITFSMLGFCSVWGAFVPAYLSSKGSRMVAVEIFAILSSSAGLLACIFVPKCYIIFLKPEQNRRRM-------
>Western_clawed_frog.locus768.1
CLHCSEYEKSNKQRTGCVPKEINYLSYTDTLGASLTSIALVLFIAASVVLGIFVRYWETPIVRANNQNLSFLLLISLMLCFLCTLLFIGRPTQICCLLRQLTFGIVFTISVSSVLAKTLTVIIAFNATKPGSKLKKYVGTQLATILVIV
CCLGEMMISTVWMASNPPFLDADTLTDINTVFLMCNEGSVLFFFSVIGYMTALALFSFIAAFLAKDFPDRFNEAKNITFSMLGFCSVWGAFVPAYLSSKGSRMVAVEIFAILSSSAGLLACIFVPKCYIIFLKPEQNRKRM-------
>Western_clawed_frog.locus778.1
CLHCSEYEKSNKQRTGCVPKEINYLSYTDTLGASLTSIALVLFIAASVVLGIFVRYWETPIVRANNQNLSFLLLISLMLCFLCTLLFIGRPTQICCLLRQVTFGIVFTISVSSVLAKTLTVIIAFNATKPGSKLKKYVGTQLATILVTV
CCLGEMMISAVWMASNPPFLDADTLTDINTVFLMCNEGSVLFFFSVIGYMTALALFSFIAAFLAKDFPDRFNEAKNITFSMLGFCSVWGAFVPAYLSSKGSRMVAVEIFAILSSSAGLLACIFVPKCYIIFFIPEQNRRRM-------
>Western_clawed_frog.locus764.1
CLHCSEYEKSNKQRTGCVPKEINYLSYTDTLGASLTSIALVLFIAASVVLGIFVRYWETPIVRANNQNLSFLLLISLMLCFLCTLLFIGRPTQICCLLRQVTFGIVFTISVSSVLAKTLTVIIAFNATKPGSKLKKYVGTQLAIILVIV
CCLGEMMISAVWMASNPPFLDADTLTDINTVFLMCNEGSVLFFFSVIGYMTALALFSFIAAFLAKDFPDRFNEAKNITFSMLGFCSVWGAFVPAYLSSKGSRMVAVEIFAILSSSAGLLACIFVPKCYIIFFRPEQNSKKM-------
>Western_clawed_frog.locus758.1
CLHCSDYEKSNKQRTGCVPKEINYLSYTDTLGASLTSIALVLFIAASVVLGIFVRYWETPIVRANNQNLSCLLLISLMLCFLCTLLFIGRPTQICCLLRQVTFGIVFTISVSSVLAKTLTVIIAFNATKPGSKLKKYVGTQLATILVIV
YCLGEMMISAVWMASNPPFLDADTLTDINTVFLMCNEGSVLFFFCIIGYIGTLALLSFIAAFLAKDFPDRFNEAKNITFSMLGFCSVWGAFVPAYLSSKGSRMVAVEIFAILSSSAGLLACIFVPKCYIIIFRPEQNRRRM-------
>Western_clawed_frog.locus756.1
CFRCSEYEKSNKQRTGCVPKEINYLSYTDTLGASLTSIALVLFITTSVVLGIFVRYWETPIVRANNQNLSFLLLISLMLCFLCTLLFIGRPTQICCLLRQVTFGIVFTISVSSVLAKTLTVIIAFNATKPGSKLKKYVGTQLAIVLVTV
CCLGEIVISAVWLASNPPFPKADTLSDPDYIILLCNEGSGFFFFCIIGYIGTLALLSFIAAFLAKDFPDRFNEAKNITFSMLGFCSVWGAFVPAYLSSKGSRMVAVEIFAILSSSAGLLACIFVPKCYIIFFRPEQNRRKM-------
```

```
>Western_clawed_frog.locus743.1
CTKCSKYEKSNSGRTSCIPRDINYLSYDEHLGSTLSSISVTFSITCAVILGIFIKYRETPIVRANNRYLSCLLLISLMLCFLCTLLFIGRPTQICCLLRQVTFGIVFTISVSSVLAKTLTVIIAFNATKPGSKLKKYVGTQLAILLVIV
CSLGEIIISMVWLASNPPFPEDDMLSDADNIILLCNEGSDCFFFCIIGYIGTLALLSLIAAFLAKDFPDRFNEAKNITFSMLGFCSVWGAFVPAYLSSKGSRMVAVEIFAILSSSAGLLGCIFIPKCYILFLRPELNTKAN--RII--
>Western_clawed_frog.locus611.1
CTKCSKYEKSNSGRTSCIPRDINYLSYDDHLGSSFSSISVTFSISCAVILGIFIKYRETPIVRANNRYLSCLLLISLMLCFLCTLLFIGRPTQICCLLRQVTFGIVFTISVSSVLAKTLTVIIAFNATKPGSKLKKYVGTQLAIILVIV
CCLGEIIISIVWLVSNPPFPEDDTLSDADIIILLCNEGSVIFFFCIIGYIGTLALLSFIAAFLAKDFPDRFNEAKNITFSMLGFCSVWGAFVPAYLSSTGAKVVAVEIFAILSSAAGLLGCIFFPKCYIIFLRPQLNTRDYIMHK---
>Western_clawed_frog.locus713.1
CTKCSKYEKSNSGRTSCIPRDINYLSYDDHLGSTFSSISVTFSISCAVILGIFIKYRETPIVRANNRYLSCLLLIFLMLCFLCTLLFIGRPTQICCLLRQVTFGIVFSISVSSLLAKTFTVIIAFNATKPGSKMKKYVGTQLAIVLVIV
CCLGEIIISIVWLASNPPFPEDDTLSDADIIILLCNEGSVVFFFCIIGYIGTLALLSFIAAFLAKDFPDRFNEAKNITFSMLGFCSVWGAFVPAYLSSTGTKVVAVEIFAILSSAAGLLGCIFFPKCYIIFLRPQLNTRDCIMHK---
>Western_clawed_frog.locus749.1
CTKCSKYEKSNSGRTSCIPRDINYLSYDDHLGSTLSSISVTFSISCAVILGIFIKYRETPIVRANNRYLSCLLLISLMLCFLCTLLFIGRPTQICCLLRQVTFGIVFTISVSSVLAKTLTVIIAFNATKPGSKLKKYVGTQLAIVLVIV
CCLGEIIISIVWLASNPPFPEDDTLSDADTIILLCNEGSGCFFFCIIGYIGTLALLSFIAAFLAKDFPDRFNEAKNITFSMLGFCSVWGAFVPAYLSSKGSRMVAVEIFAILSSSAGLLGCIFIPKCYIIFLRPELNTKAN--RII--
>Western_clawed_frog.locus748.1
CTKCSKYEKPNVGRTSCIPRDINYLSYGDQLGATLSSISVIFSITCAVILGIFIKYRETPIVRANNRYLSCLLLISLMLCFLCTLLFIGRPTQICCLLRQVTFGIVFTISVSSVLAKTLTVIIAFNATKPGSKLKKYVGTQLATILVIV
CSLGEMIISIVWLVSSPPFPEDDTLSDADTIILLCNEGSGYFFFCIIGYMGTLALLSFIAAFLAKDFPDRFNEAKNITFSMLGFCSVWGAFVPAYLSSKGSRMVAVEIFAILSSSAGLLGWIFIPKCYIIFLRPELNTKAN--RII--
>Western_clawed_frog.locus751.1
-TKCSKYERSNSGRTSCIPRDINYLSYDDHLGSTFSSISVTFSISCAVILGIFIKYRNTPIVRANNRYLSCLLLISLMLCFLCTLLFIGRPTQICCLLRQITFGIVFTISVSSVLAKTLTVIIAFNATKPGSKLKKYVGTQLAIILVIV
CSLGEMIISIVWMVSYPPFLEDDTLSDADTIILLCNDGSGYFFFCIIGYIGTLALLSFIAAFLAKDFPDRFNEAKNITFSMLGFCSVWGAFVPAYLSSKGSRMVAVEIFAILSSSAGLLGCIFIPKCYIIFLRPELNTKAN--RII--
>Western_clawed_frog.locus745.1
CKECSEYEKSNRGRTSCIPRDINYLSYDDHLGSTFSSISVTFSISCAVILGIFIKYRETPIVRANNRYLSCLLLISLMLCFLCTLLFIGRPTQICCLLRQVTFGIVFTISVSSVLAKTLTVIIAFNATKPGSKLKKYVGTQLPIILVIV
CSLGEIIISIVWLASNPPFPEVNTLSDADTIILLCNEGSGFFFFCIIGYIGTLALLSFIAAFLSKDFPDRFNEAKNITFSMLGFCSVWGAFVPAYLSSKGSRMVAVEIFAILSSSAGLLACIFIPKCYIIFLRPELNTKAN--RII--
>Western_clawed_frog.locus747.1
CTKCSKYEKSNRGRTSCIPRDINYLSYDDHLGSTLSSISVTFSISCAVILGIFIKYHETPIVRANNRYLSCLLLISLMLCFLCTLLFIGRPTQICCLLRQVTFGIVFTISVSSVLAKTLTVIIAFNATKPGSKLKKYVGTQLATILVIV
CSLGESIISIVWLASNPPFSETDFFSDPYNIILQCNEGSGSFFFCIIGYIGTLALLSFIAAFLAKDFPDRFNEAKNITFSMLGFCSVWGAFVPAYLSSKGSSMVAVEIFAILSSSAGLLACIFIPKCYIIFIRPELNTKAN--RII--
>Western_clawed_frog.locus752.1
CTKCSKYERSNRGRTSCIPRDINYLSYDDHLGSTLSSISVTFSISCAVILGIFIKYRETPIVRANNRYLSCLLLISLMLCFLCTLLFIGRPTQICCLLRQVTFGIVFTISVSSVLAKTLTVIIAFNATKPGSKLKKYVGTQLAIILVIV
CSSGEIIISIVWLVSNPPFPEADTLSDPDYIILLCNEGSGFFFFCIIGYIGTLALLSFIAAFLAKDFPDRFNEAKNITFSMLGFCSVWGAFVPAYLSSKGSRMVAVEIFAILSSSAGLLGCIFIPKCYIIFLRPELNTKAN--RII--
>Western_clawed_frog.locus744.1
CTKCSKYEKSNSGRTSCIPRDINYLSYDEHLGSTLSSISVTFSITCAVILGIFIKYRETPIVRANNRYLSCLLLISLMLCFLCTLLFIGRPTQICCLLRQVTFGIVFTISVSSVLAKTLTVIIAFNATKPGSKLKKYVGTQLAIILVIV
CSSGEIVISIVWLASNPPFPEADTLSDPDYIILLCNDGSELFFFCIIGYIGTLALLSFIAAFLAKDFPDRFNEAKNITFSMLGFCSVWGAFVPAYLSSKGSRMVAVEIFAILSSSAGLLGCIFIPKCYILFLRPELNRKAN--RII--
>Western_clawed_frog.locus742.1
CTKCSKYEKSNSGRTSCIPRDINYLSYDEQLGSTLSSISVTFSISCAVILGIFIKYRDTPIVRANNRYLSCLLLISLMLCFLCTLLFIGRPTQICCLLRQVTFGVVFTISVSSVLAKTLTVIIAFNATKPGSKLKKYVGTQLAIVLVIV
CSLGEIVISAVWLASNPPFPEADTLSDPDYIILLCNEGSGFFFFCIIGYIGTLALLSFIAAFLAKDFPDRFNEAKNITFSMLGFCSVWGVFVPAYLSSKGSRMVAVEIFAILSSSAGLLGCVFIPKCYIIFLWPELNTRVTVIRTY--
>Western_clawed_frog.locus753.1
CTKCSKYEKSNSGRTSCIPRDINYLSYDDHLGSTLSSISVTFSISCAVILGIFIKYRETPIVRANNRYLSCLLLISLMLCFLCTLLFIGRPTQICCLLRQVTFGIVFTISVSSVLAKTLTVIIAFNATKPGSKLKKYVGTQLATILVIV
CSLGEIVVSAVWMASNPPFPEADTLSDPDYIILLCNEGSGFFFFCIIGYMGTLALLSFIAAFLAKDFPDRFNEAKNITFSMLGFCSVWGAFVPAYLSSKGSRMVAVEIFAILSSSAGLLGCIFIPKCYIIFLWPELNTRVTVIRTY--
>Western_clawed_frog.locus659.1
CTKCSKYEKSNSGRTSCIPRDINYLSYDDHLGSTFSSISVTFSISCAVILGIFIKYRETPIVRANNRYLSCLLLISLMLCFLCTLLFIGRPTQICCLLRQVTFGVVFTISVSSVLAKTLTVIIAFNATKPGSKLKKYVGTQLAIILVIV
CSLVVIAISAVWMVYNPPFPEADTLSDPDYIILLCNEGSGFFFFCIIGYIGTLALLSFIAAFLAKDFPDRFNEAKNITFSMLGFCSVWGAFVPAYLSSKGNRMVAVEIFAILSSSAGLLACIFIPKCYIIFLRPELNTKAN--RII--
>Western_clawed_frog.locus666.1
-MKCSKYEKSNSVRTSCFPRDINYLSYGDQLGATLSSISVTFSISCAVILGIFIKYRKNPIVRANNRYLSCLLLISLMLCFLCTLLFIGRPTQICCLLRQVTFGIVFTISVSSVLAKTLTVIIAFNATKPGSKLKKYVGTQLAIVLVIV
CSLVEIVISAMWMASNPPFHEADTLSDPDYIILLCNEGSGFFLFCIIGYIGTLALLSFIAAFLAKDFPDRFNEAKNITFSMLGFCSVWGAFVPAYLSSKGSRMVAVEIFAILSSSAGLLGCIFIPKCYIIFLRPELNRKDNIVRKQ--
>Western_clawed_frog.locus665.1
-MKCSKYEKSNSVRTSCFPRDINYLSYGDQLGATLSSISVTFSISCAVILGIFIKYRKNPIVRANNRYLSCLLLISLMLCFLCTLLFIGRPTQICCLLRQVTFGIVFTISVSSVLAKTLTVIIAFNATKPGSKLKKYVGTQLAIILVIV
CSLVEIVISAMWMASNPPFHEADTLSDPDYIILLCNEGSGFFLFCIIGYIGTLALLSFIAAFLAKDFPDRFNEAKNITFSMLGFCSVWGAFVPAYLSSKGSRMVAVEIFAILSSSAGLLGCIFIPKCYIIFLRPELNRKDNIVRKQ--
>Western_clawed_frog.locus664.1
-MKCSKYEKSNSVRNSCFPRNINYLSYGDQLGATLSSISVTFSISCAVILGIFIKYRETPIVRDNNRYLSCLLLISLMLCFLCTLLFIGRPTQICCLLRQVTFGVVFTISVSSVLAKTLTVIIAFNATKPGSKLKKYVGTQLAIILVIV
CSLVEIVISAMWMASNPPFPEADTLSDPDYIILLCNEGSGFFLFCIFGYIGTLALLSFIAAFLAKDFPDRFNEAKNITFSMLGFCSVWGAFVPAYLSSKGSRMVAVEIFAILSSSAGLLGCIFIPKCYIIFLRPELNRKDNVVRKQ--
>Western_clawed_frog.locus667.1
-MKCSKYEKSNSVRTSCIPKDINYLSYEDQLGSTLSSISVTFSISCAVILGIFIKYRKTPIVRANNRYLSCLLLISLMLCFLCTLLFIGRPTQICCLLRQVTFGVVFTISVSSVLAKTLTVIIAFNATKPGSKLKKYVGTQLAIILVIV
CCLVEIVISAVWLASNPPFPEADTLSDPDYIILLCNEGSGFFLFCIFGYIGTLALLSFIAAFLAKDFPDRFNEAKNITFSMLGFCSVWGAFVPAYLSSKGSRMVAVEIFAILSSSAGLLACIFIPKCYIIFLRPELNRKDNIVRKQ--
>Western_clawed_frog.locus653.1
-MKCSKYEKSNSVRTSCIPRDINYLSYGDQLGATLSSISVTFSISCAVILGIFIKYRETPIVRANNRYLSCLLLISLMLCFLCTLLFIGRPTQICCLLRQVTFGIVFTTSVSSVLAKTLTVIIAFNATKPGSKLKKYVGTQLAIILVIG
CSLVEIVISAVWLASNPPFPEADTLSDPDYIILLCNEGSGFFLFCIFGYIGTLALLSFIAAFLAKDFPDRFNEAKNITFSMLGFCSVWGAFVPAYLSSKGSRMVAVEIFAILSSSAGLLGCIFIPKCYIIFLRPELNRKDNIVRKQ--
>Western_clawed_frog.locus668.1
-MKCSKYEKSNTGRNSCIKRNVNYLSYGDQLGATLSSMSIMFSITCAVILGIFIKYRETPIVRANNRYLSCLLLISLMLCFLCTLLFIGRPTQICCLLRQVTFGVVFTISVSSVLAKTLTVIIAFNATKPGSKLKKYVGTQLAIILVIV
CSLVEIVISAVWLASNPPFPEADTLSDPDYIILLCNEGSGFFFFCIIGNIGTLALLSFIAAFLAKDFPDRFNEAKNITFSMLGFCSVWGAFVPAYLSSKGSRMVAVEIFAILSSSAGLLGCIFIPKCYIIFLRPELNRKDNIVRKQ--
>Western_clawed_frog.locus649.1
-MKCSKYEKSNTGRTSCIPRDINYLSYGDQLGATLSSISVIFSITCAVILGIFIKYRETPIVRANNRYLSCLLLISLMLCFLCTLLFIGRPTQICCLLRQVTFGIVFTIPVSSVLAKTLTVIIAFNATKPGSKLKKYVGTQLATILVIV
CSLVELVISAVWLASNPPFPEADTLSDPDYIILLCNERSGFFFFCIIGYIGTLALLSFIAAFLAKDFPDRFNEAKNITFSMLGFCSVWGAFVPAYLSSKGSRMVAVEIFAILSSSAGLLGCIFIPKCYIIFLRPELNRKDNIVRKQ--
>Western_clawed_frog.locus654.1
CLKCSKYKKSNTGKTSCIPRDINYLSYEDQLGTTLSSISVTFSISCAVILGIFIKYRETPIVRANNRYLSCLLLISLMLCFLCTLLFIGRPTQICCLLRQVTFGIVFTISVSSVLAKTLTVIIAFNATKPGSKLKKYVGTQLAIILVIV
CSLVEIVISAVWLASNPPFPEADTLSDPNYIILLCNEGSGFFFFCIIGYIGTLALLSFIAAFLAKDFPDRFNEAKNITFSMLGFCSVWGAFVPAYLSSKGSRMVAVEIFAILSSSAGLLGCIFIPKCYIIFLRPELNRKDNIVRKQ--
>Western_clawed_frog.locus640.1
-MKCSKYEKSNTGRTSCIPRDINYLSYGDQLGATFSSISVTFSISCAVILGIFIKYRETPIVRANNRYLSCLLLISLMLCFLCTLLFIGRPTQICCLLRQVTFGIVFTISVSSVLAKTLTVIIAFNATKPGSKLKKYVGTQLAIILVIV
CSLVEIVISAVWLAFNPPFPEADTLSDPDYIILLCNECSDFFFFCIIGYIGTLALLSFIAAFLAKDFPDRFNEAKNITFSMLGFCSVWGAFVPAYLSSKGSRMVAVEIFAILSSSAGLLGCIFIPKCYIIFLRPELNTKDPIVRK---
>Western_clawed_frog.locus655.1
-MKCSKYKKSNTGRTSCILRDINYLSYEDQLGATLSSISIMFFISCAVILGIFIKYRETPIVRANNRYLSCLLLISLMLCFLCTLLFIGRPTQICCLLRQVTFGIVFTISVSSVLAKTLTVIIAFNATKPGSKLKKYVGTQLAIILVIV
CSLVEIVISAVWLASNPPFPEADTLSDPDYIILLCNEGSGSFFFCIIGYIGTLALLSFIAAFLAKDFPDRFNEAKNITFSMLGFCSVWGAFVPAYLSSKGSRMVAVEIFAILSSSAGLLACIFIPKCYIIFLKPELNTKDPIVRK---
>Western_clawed_frog.locus651.1
-MKCSKYKKSNTGRTSCIPRNINYLSYGDQLGTTLSSISVTFSISCAVILGIFIKYRETPIVRANNRYLSCLLLISLMLCFLCTLLFIGRPTQICCLLRQVTFGIVFTISVSSVLAKTLTVIIAFNATKPGSKLKKYVGTQLSIVLVIV
CSLGEIVISAVWLASNPPFPEADTLSDPDYIILLCNEGSGFFFFCIIGYIGTLALLSFIVAFLAKDFPDRFNEAKNITFSMLGFCSMWGAFVPAYLSSKGSRMVAVEIFAILSSSAGLLGCIFIPKCYIIFLRPELNTKDPTVRK---
>Western_clawed_frog.locus675.1
-MKCSKYEKSNSGRTSCIPRNINYLSYGDQLGSILSSISVMFFITCAVILRNFIKYHETPIVRANNRYLSCLLLISLMLCFLCTLLFIGRPTQICCLLRQVTFGIVFTISVSSVLAKTLTVIIAFNATKPGSKLKKYVGTQLAILLVIV
CSLVEIVISAVWLASNPPFPEADTFSDPDYIILLCNEGSELFFFCIIGYIGTLALLSFIAAFLAKDFPDRFNEAKNITFSMLGFCSVWGAFVPAYLSSKGSRMVAVEIFAILSSSAGLLGCIFIPKCYIIFLRPELNTKDPLVRTL--
>Western_clawed_frog.locus676.1
-MKCSKYEKSNSGRTSCIPRNINYLSYGDQLGSTLSSISIMFSITCAVILRNFIKYRETPIVRANNQYLSCLLLISLMLCFLCNLLFIGRPTQICCLLRQVTFGIVFTISVSSVLAKTLTVIIAFNATKPGSKLKKYVGTQLAIVLVIV
CSLVEVVISAVWLASNPPFPEVDTVSDPDYIILLCNEGSELFFFCIIGYIGTLALLSFIAAFLAKDFPDRFNEAKNITFSMLGFCSVWGAFVPAYLSSKGSRMVAVEIFAILSSSAGLLGCIFIPKCYIIFLRPELNTKDPFVRKS--
>Western_clawed_frog.locus677.1
-MKCSKYEKSNSERNICIPRNINYLSYGDQLGSTLSSISIMFSITCPVILRIFIKYRETPIVRANNQYLSCLLLISLMLCFLCTLLFIERPTQICCLLRQVTFGVVFTISVSSVLAKTLTVIIAFNATKPGSKLKKYVGTQLAIILVTV
CSFVEIVISAVWLASNPPFPEDDTLSDPENMILLCNEGSDFFFFCIIGYIGTLALLSFIAAFLAKDFPDRFNEAKNITFSMLGFCSVWGAFVPAYLSSKGSRMVAVEIFAILSSSAGLLGCIFIPKCYIIFLRPELNTKDPFVRK---
>Western_clawed_frog.locus647.1
-MKCSKYEKSNSGRNSCIPRNINYLSYEDQLGSILASISITFSITCAVILRNFIKYRETPIVRANNQNLSCLLLISLMLCFLCTLLFIGRPTQICCLLRQVTFGIVFTISVSSVLAKTLTVIIAFNATKPGSKLKKYVGTQLAIVLVIV
CSLVEIVISAVWLASNPPFPEADTLSDPDYIILLCNEGSGFFFFCIIGYIGTLALLSFIAAFLAKDFPDRFNEAKNITFSMLGFCSVWGAFVPAYLSSKGSRMVAVEIFAILSSSAGLLACIFIPKCYIIFRRPELNTKDPLVRKL--
>Western_clawed_frog.locus681.1
-MKCSKYEKSNSERNSCIPRNINYLSYEDQLGTTLSSISVTFSVTCAVILGIFIKYRETPIVRATVSSL------
SLMLCFLCTLLFIGRPTQICCLLRQVTFGIVFTISVSSVLAKTLTVIIAFNATKPGSKLKKYVGTQLATILVIVCSLVEIVISAVWLASNPPFPEADTLSDPENIILLCNEGSGFFLFCIIGYIGTLALLSFIAAFLAKDFPDRFNEAK
NITFSMLGFCSVWGAFVPAYLSSKGSRMVAVEIFAILSSSAGLLGCIFIPKCYIIFLRPELNTKDPFVRK---
>Western_clawed_frog.locus679.1
-MKCSKYEKSNSERNSCIPRNINYLSYEDQLGTTLSSISVTFSVTCAVILGIFIKYRETPIVRANNRYLSCLLLISLMLCFLCTLLFIGRPTQICCLLRQVTFGIVFTISVSSVLAKTLTVIIAFNATKPGSKLKKYVGTQLATILVIV
CSLVEIVISAVWLASNPPFPEADTLSDPENIILLCNEGSGFFLFCIIGYIGTLALLSFIAAFLAKDFPDRFNEAKNITFSMLGFCSVWGAFVPAYLSSKGSRMVAVEIFAILSSSAGLLGCIFIPKCYIIFLRPELNTKDPFVRK---
>Western_clawed_frog.locus678.1
CTKCSKYEKSNSGRTSCIPRNINYLSYEDQLGSTFSSISVILSITCAVILGIFIKYRETPIVRANNRYLSCLLLISLMLCFLCTLLFIGRPTQICCLLRQVTFGIVFTTSVSSVLAKTLTVIIAFNATKPGRKLKKYVGTQLAIILVIV
CSLGEIVISAVWLASNPPFPEADTLSGPDYIILLCNEGSGFFFFCIIGYIGTLALLSFIAAFLAKDFPDRFNEAKNITFSMLGFCSVWGAFVPAYLSSKGSRMVAVEIFAILSSSAGLLGCIFIPKCYIIFLRPELNTKDPFVRK---
>Western_clawed_frog.locus680.1
CTKCSKYEKSNSGRTSCIPRNINYLSYEDQLGSTFSSISVILSITCAVILGIFIKYRETPIVRANNRYLSCLLLISLMLCFLCTLLFIGRPTQICCLLRQVTFGIVFTTSVSSVLAKTLTVIIAFNATKPGRKLKKYVGTQLAIILVIV
CSLGEIVISAVWLASNPPFPEADTLSGPDYIILLCNEGSGFFFFCIIGYIGTLALLSFIAAFLAKDFPDRFNEAKNITFSMLGFCSVWGAFVPAYLSSKGSRMVAVEIFAILSSSAGLLGCIFIPKCYIIFLRPELNTKDPFVRK---
>Western_clawed_frog.locus646.1
CTKCSKYEKSNSGRTSCIPRNINYLSYEDQLGSTFSSIAVILSISCAVILGIFIKYRETPIVRANNRYLSCLLLISLMLCFLCTLLFIGRPTQICCLLRQVTFGIVFTISVSSVLAKTLTVIIAFNATKPGSKLKKYVGTQLAIILVIV
CSLVEIVVSAVWLASNPPFPEADTLSDPDYIILLCNEGSGFFFFCIIGYIGTLALLSFIAAFLAKDFPDRFNEAKNITFSMLGFCSVWGAFVPAYLSSKGSRMMAVEIFAILSSSAGLLGCIFIPKCYIIFLRPELNTKDPFVRK---
>Western_clawed_frog.locus685.1
-MKCSKYEKSNTGRNGCILRDINYLSYEDHLGFTLSSISVILSVTCAVILGIFIKYRETPIVRANNRYLSCLLLISLMLCFLCTLLFIGRPTQICCLFQQVTFGVVFTISVSSLLAKTLTVIIAFNATKPGSKLKKYVGTQLAIILVIV
CSLVDIVISAVWLASNPPFPEADTLSDPDYIILLCNEGSVTFFFCIIGYIGTLALLSFTAAFLAKDFPDRFNEAKNITFSMLGFCSVWGAFVPAYLSSKGSRMVAVEIFAILSSSAGLLGCIFIPKCYIIFLRPELNTKDPIVRKFKT
>Western_clawed_frog.locus641.1
-MKCSKYEKSNTGRTSCILRDINYLSYEDHLGFTLSSISVILSVTCAVILGIFIKYRETPIVRANNRYLSCLLLISLMLCFLCTLLFIGRPTQICCLFQQVTFGIVFTISVSSLLAKTLTVIIAFNATKPGSKLKKYVGTQLAIILVIV
CSLVDIVISAVWLASNPPFPEANTLSDPDYIILLCNEGSVTFFFCIIGYIGTLALLSFTAAFLAKDFPDRFNEAKNITFSMLGFCSVWGAFVPAYLSSKGSRMVAVEIFAILSSSAGLLACIFIPKCYIIFLRPELNTKDPIVRKH--
>Western_clawed_frog.locus686.1
-MKCSKSKKSNTGRNGCILRDINYLSYGDQLGSTLSSISVILSISCAVILGIFIKYRETPIVRANNRYLSCLLLISLMLCFLCTLLFIGRPTQICCLLRQVTFGIVFTISVSSLLAKTLTVIIAFNATKPGSKLKKYVGTQLAFILVIV
CSLVEIVISAVWLASNPPFPEADTLSDPDYIILLCNEGSDFFFFCIIGYIGTLALLSFIAAFLAKDFPDRFNEAKNITFSMLGFCSVWGAFVPAYLSSKGSRMVAVEIFAILSSSAGLLGCIFIPKCYIIFLRPELNTKDPIVRK---
>Western_clawed_frog.locus687.1
CKKCSKYEKSNTGRTSCIPRDINYLSYGDQLGATLSSISVILSITCAVILGIFIKYRETPIVRANNRYLSCLLLISLMLCFLCTLLFIGCPTQICCLLRQVTFGVVFTISVSSVLAKTLTVIIAFNATKPGSKLKKYVGTQLPIVLVIV
CSLVEIVISAVWLASNPPFPEADTLSDPDYIILLCNEGSGFFFFCIIGYIGTLALLSFIAAFLAKDFPDRFNEAKNITFSMLGFCSVWGAFVPAYLSSKGSRMVAVEIFAILSSSAGLLACIFIPKCYIIFLRPELNTKDPIVRK---
>Western_clawed_frog.locus688.1
CTKCSKYEKSNTGRDNCIPRDINCLSYGDQLGATLSSISVIFSVTCAVILGIFIKYRETPIVRANNRYLSCLLLISLMMCFLCTLLFIGRPTQICCLLQQVTFGIVFTISVSSVLAKTLTVIIAFNATKPGSKLKKYVGTQLAIILVIV
CSFGEIIISAVWLASNPPFPEADTLSDPDYIILLCNEGSGFFFFCIIGYIGTLALLSFIAAFLAKDFPDRFNEAKNITFSMLGFCSVWGAFVPAYLSSKGSRMVAVEIFAILSSSAGLLGCIFIPKCYIIFLRPELNTKDPIIKKC--
>Western_clawed_frog.locus639.1
-MKCSIYEKSNTGRTSCIPRDINCLSYGDQLGATLSSISVILSLTCAVILGIFIKYRETPIVRANNRYLSCLLLISLMLCFLCTLLFIGRPTQICCLLRQVTFGIVFTISVSSVLAKTLTVIIAFNATKPGSKLKKYVGTQLAIILVIV
CSLGEIVISAVWLASNPPFPEADTLSDPDYIILLCNEGSGYFFFCIIGYIGTLALLSFIAAFLAKDFPDRFNEAKNITFSMLGFCSVWGAFVPAYLSSKGSRMVAVEIFAILSSSAGLLGCIFIPKCYIIFLRPELNTKDPIIKNVRN
```

```
>Western_clawed_frog.locus669.1
-MKCSKHEKSNTGRTSCILRNINYLSYGDQLGTTLSSISIMFSITCAVILGIFIKYRETPIVRANNRYLSCLLLISLMLCFLCTLLFIGRPTQICCLLRQVTFGIVFTISVSSVLAKTLTVIIAFNATKPGSKLKKYVGTQLSIILVIV
CSLVEIVISAVWLASNPPFPEADSLSDPDYIILLCNEGSGFFFFCIIGYIGTLAQLSFIAAFLAKDFPDRFNEAKNITFSMLGFCSVWGAFVPAYLSSKGSRMVAVEIFAILCSSAGLLACIFIPKCYILFIRSELNTKETIIRKQ--
>Western_clawed_frog.locus671.1
-MKCSKYEKSNTGRTSCIPRDINYLSYGDQLGATLSSISVTLSITCAVILGIFIKYRETPIVRANNRYLSCLLLISLMLCFLCTLLFNGRPTQICCVLRQVTFGIVFTTSVSSVLAKTLTVIIAFNATKPGSKLKKYVGTQLAIILVIV
CSLVEVVISAVWLASNPPFPEADTLSDPDYIILLCNEGSGFFLFCIIGYIGTLALLSFIAAFLAKDFPDRFNEAKNITFSMLGFCSVWGAFVPAYLSSKGSRMVAVEIFAILSSSAGLLGCIFIPKCYIIFLRSELNTKETITRK---
>Western_clawed_frog.locus672.1
-MKCSKYEKSNTGRNHCIPRDINYLSYGDQLGATLSSISVILSVTCAVILGIFIKYRETPIVRANNRYLSCLLLISLMLCFLCTLLFIGRPTQICCLLRQVTFGVVFTISVSSVLAKTLTVIIAFNATKPGSKLKKYVGTQLAIILVII
CSLVEIVVSAVWLASNPPFPEADTLSDPDYIILLCNEGSGSFFFCIIGYMGTLALLSFIAAFLAKDFPDRFNEAKNITFSMLGFCSVWGAFVPAYLSSKGSRMVAVEIFAILSSSAGLLGCIFIPKCYILFIRSELNTKETITRK---
>Western_clawed_frog.locus674.1
CMKCSKYEKSNRRRNSCIPRDINCLSYGDQLGATLSSISVILSLTCAVILGIFIKYHETPIVRANNQYLSCLLLISLMLCFLCTLLFIGRPTQICCLLRQVTFGIVFTISVSSVLAKTLTVIIAFNATKPGSKLKKYVGTQLAIILVIV
CSLVEIVISAVWMASNPPFPEADTLSDPDYIILLCNEGSGCFFFCLIGYMGTLALLSFIAAFLAKDFPDRFNEAKNITFSMLGFCSVWGAFVPAYLSSKGSRMVAVEIFAILSSSAGLLGCIFIPKCYILFLRPELNTKETIIRKQEN
>Western_clawed_frog.locus746.1
-MKCSKYAKSNVGRNGCIPRNINYLSYGDQLGSTLSSISVILSVTCAVILGIFIKYRETPIVRANNRYLSCLLLISLMLCFLCTLLFIGRPTQICCLLRQVTFGIVFTISVSSVLAKTLTVIIAFNATKPGSKLKKYVGTQLAIILVIV
CSLVEIVISSVWLASNPPFPEADTLSDPDYIILMCNEGSDFFFFCIIGYIGTLALLSFIAAFLAKDFPDRFNEAKNITFSMLGFCSVWGAFVPAYLSSKGSRMVAVEIFAILSSSAGLLGCIFIPKCYILFLRPELNTKANVIRK---
>Western_clawed_frog.locus662.1
-MKCSKYAKSNVGRNSCIPRDINYLSYGDQLGASLSSMSLILSISCAVILGIFIKYRETPIVRANNRYLSCLLLISLMLCFLCTLLFIGRPTQICCLLRQVTFGIVFTISVSSVLAKTLTVIIAFNATKPGSKLKKYVGTQLAIILVIV
CSLVEIVISAVWLASNPPFPEADTLSDPDYIILLCNEGSGYFFFCIIGYIGTLALLSFIAAFLAKDFPDRFNEAKNITFSMLGFCSVWGAFVPAYLSSKGSRMVAVEIFAILSSSAGLLACIFIPKCYIIFIRPELNTKANVINVT--
>Western_clawed_frog.locus663.1
-MKCSKYEKSNSGRNGCIKRNVNYLSYGDQLGATLSSISVILSVTCAVILGIFIKYRKTPIVRANNRYLSCLLLISLMLCFLCTLLFIGCPTQICCLLRQVTFGIVFTISVSSVLAKTLTVIIAFNATKPGSKLKKYVGTQLAIILVIV
CSLGEIVISAVWLASNPPFPEADTLSDPDYIILLCNEGSGFFFFCIIGYMGTLALLSFIAAFLAKDFPDRFNEAKNITFSMLGFCSVWGAFVPAYLSSKGSRMVAVEIFAILSSSAGLLACIFIPKCYIIFIRSELNTKETIIRKQ--
>Western_clawed_frog.locus738.1
-MKCSKYEKSNSVRNRCIPRNIDYLSYGDQLGSTLSSISVILSVTCAVILGIFIKYRETPIVRANNRYLSCLLLISLMLCFLCTLLFIGRPTQICCLLRQVTFGIVFTISVSSVLAKTLTVIIAFNATKPGSKLKKYVGTQLAIILVIA
CSLVEIVMSAVWMASNPPFPEADTLSDPDYIILLCNEGSVTFFFCIIGYMGTLALLSFIAAFLAKDFPDRFNEAKNITFSMLGFCSVWVAFVPAYLSSKGSRMVAVEIFAILSSSAGLLACIFIPKCYIIFLKPELNTKETIIRKQ--
>Western_clawed_frog.locus670.1
-MKCSKYEKSNSVRNSCIPRNIDYLSYDDHLGSTLSSISVILSVACAVILGIFIKYRETPIVRANNRYLSCLLLISLMLCFLCTLLFIGRPTQICCLLRQVTFGIVFTISVSSVLAKTLTVIIAFNATKPGSKLKKYVGTQLAIILVIV
CSLVEIVISALWLASIPPFPEADTLSDPDYIILLCNEGSGFFFFCIIGYIGTLALLSFIAAFLAKDFPDRFNEAKNITFSMLGFCSVWGAFVPAYLSSKGSRMVAVEIFAILSSSAGLLGCIFIPKCYILFLKPELNTKDSIIRNKEY
>Western_clawed_frog.locus650.1
-MKCSKYEKSNSVRNGCIPRDINYLSYEDHLGCTLSSISVIFSIVCAVILGIFIKYRETPIVRANNRYLSCLLLISLMLCFLCTLLFIGRPTQICCLLRQVTFGIVFTISVSSVLAKTLTVIIAFNATKPGSKLKKYVGMQLSIILVIV
CSLVEIVISAVWLASNPPFPEADTLSDPYYIILLCNEGSDFFFFCIIGYIGTLALLSFIAAFLAKDFPDRFNEAKNITFSMLGFCSVWGAFVPAYLSSKGSRMVAVEIFAILSSSAGLLGCIFISKCYIIFLKPELNTKETIITKQ--
>Western_clawed_frog.locus657.1
-MKCSKYEKSNSVRNGCIPRDIDYLSYEDHLGCTLSSISVIFSIICAVILGIFIKYRETPIVRANNQYLSCLLLISLMLCFLCTLLFIGRPTQICCLLRQVTFGVVFTISVSSVLAKTLTVIIAFNATKPGSKLKKYVGTQLAIILVIV
CSLGEIVISAVWLASNPPFPATDTLTDPDYIILLCNEGSVLFFFSVIGYMTALALFSFIAAFLAKDFPDRFNEAKNITFSMLGFCSVWGAFVPAYLSSKGSRMVAVEIFAILSSSAGLLGCIFIPKCYILFLRPELNTKETIIRKQ--
>Western_clawed_frog.locus656.1
-MKCSKYEKSNSVRNGCIPRDIDCLSYEDHLGSTLSSISVIFSITCAVILGIFIKYRETPIVRANNRYLSCLLLISLMLCFLCTLLFIGRPTQICCLLRQVTFGIVFTISVSSVLAKTLTVIIAFNATKPGSKLKKYVGTQLATILVIV
CSLGEIVISAVWLASNPPFPETDTLTDPDYIILLCNEGSVLFFFSVIGYMTALALFSFIAAFLAKDFPDRFNEAKNITFSMLGFCSVWGAFVPAYLSSKGSRMVAVEIFAILSSSAGLLGCIFIPKCYILFLRPELNTKETIIRKQ--
>Western_clawed_frog.locus673.1
-MKCSKYEKLNSIRNGCILRNIDYLSYEDQLGSTLSSISVTFSITCAVILGIFIKYRETPIVRANNQYLSCLLLISLMLCFLCTLLFIGRPTQICCLLRQVTFGIVFTISVSSVLAKTLTVIIAFNATKPGSKLKKYVGTQLAIVLVIV
CCLGEIIISIVWLASSPPFPEADTLSDPNYIILLCNEGSGFFFFCIIGYIGTLALLSFIAAFLAKDFPDRFNEAKNITFSMLGFCSVWGAFVPAYLSSKGSRMVAVEIFAILSSSAGLLGCIFIPKCYILFLRPELNTKETIITKQ--
>Western_clawed_frog.locus648.1
-MKCSKYEKLNSIRNGCILRNIDYLSYEDQLGCTLSSISVTFSISCAVILGIFIKYRETPIVRANNRYLSCLLLISLMLCFLCTLLFIGRPTQICCLLRQVTFGVVFTISVSSVLAKTLTVIIAFNATKPGSKLKKYVGTQLAIVLVIV
CCLDEIIISIVWLASSPPFPEADTLSDPNYIILLCNEGSGCFFFCIIGYIGTLALLSFIAAFLAKDFPDRFNEAKNITFSMLGFCSVWVAFVPAYLSSKGSRMVAVEIFAILSSSAGLLGCIFIPKCYIIFLKPELNTKETIITKQ--
>Western_clawed_frog.locus645.1
CIKCSKKEKSNSVKNSCIPRNINYLSYEDQLGAAFSSISVIFFVTCAVILGIFIKYSETPIVRANNRYLSCLLLISLMLCFLCTLLFIGRPTQICCLLRQVTFGIVFTISVSSVLAKTLTVIIAFNATKPGSKLKKYVGTQLAIVLIIV
CSLGEIVVSAVWLASNPPFPEADTLSDPDYIILLCNEGSGCFFFCIIGYIGTLALLSFIAAFLSKDFPDRFNEAKNITFSMLGFCSMWGAFVPAYLSSKGSRMVAVEIFAILSTSAGLLGCIFIPKCYIIFLRPELNTKEIIIRKQ--
>Western_clawed_frog.locus692.1
CIKCSKYEKSNKERNVCIPKNINFLSYEDILGSALSSIAVIYFIICAVILGIFIKYRETPIVRANNRYLSCLLLISLMLCFLCTLLFIGRPTQICCLLRQVTFGIVFTISVSSVLAKTLTVIIAFNATKPGSKLKKYVGTQLAIVLVII
CSLGEIVISAVWLASNPPFPEVDTFSDPNYIIIICNEGSGFFFFCIIGYIGTLALLSFIAAFLAKDFPDRFNEAKNITFSMLGFCSVWGAFVPAYLSSKGSRMVAVEIFAILSSSSGILGCIFIPKCYIVFVRPELNTKDTIVRKQ--
>Western_clawed_frog.locus635.1
CIKCSKYEKSNKERNVCIPKNINFLSYEDILGSALSSIAVIYFIICAVILGIFIKYRETPIVRANNRYLSCLLLISLMLCFLCTLLFIGRPTQICCLLRQVTFGIVFTISVSSVLAKTLTVIIAFNATKPGSKLKKYVGTQLAIILVIV
CSLGEIVISAVWLASNPPFPEVDTFSDPNYIIIICNEGSGFFFFCIIGYIGTLALLSFIAAFLAKDFPDRFNEAKNITFSMLGFCSVWGAFVPAYLSSKGSRMVAVEIFAILSSSSGILGCIFIPKCYIVFVRPELNTKDTIVRKQ--
>Western_clawed_frog.locus644.1
CIKCSKYEKPNNEKTECIPRNINFLSYEDTLGATLSFITLILFGICPVILGIFIKYRKTPIVRANNQYLSCLLLISLMLCFLCTLLFIGRPTQICCLLRQVTFGIVFTISVSSVLAKTLTVIIAFNATKPGSKLKKYVGTQLAIILVIV
CSLVEVVISAVWLASNPPFPEADTLSDPDYIILLCNEGSELFFFCIIGYIGTLALLSFIAAFLAKDFPDRFNEAKNITFSMLGFCSVWGAFVPAYLSSKGSRMVAVEIFAILSSSAGLLGCIFIPKCYIIFLRPELNTKDTVVRKL--
>Western_clawed_frog.locus682.1
CIKCSKYEKPNNEKTECIPRNINFLSYEDTLGATLSFITLILFGICAGILGIFIKYRETPIVRANNRHLSCLLLISLMLCFLCTLLFIGRPTQICCLLRQVTFGIVFTISVSSVLAKTLTVIIAFNATKPGSKLKKYVGTQLAIILVIV
CSFGEIVVSAVWLASNPPFPEADTLSDPDYIILLCNEGSGCFFFCIIGYIGTLALLSFIAAFLAKDFPDRFNEAKNITFSMLGFCSVWGAFVPAYLSSKGSRMVAVEIFAILSSSAGLLGCIFIPKCYIIFIRSELNTKETIIRKQ--
>Western_clawed_frog.locus642.1
CIRCTEYEKSNKEKNSCIPKNIDFLSYKDTLGVTLTSITVICSIICAVILGIFIKYRETPIVRANNRYLSCLLLISLMLCFLCTLLFIGRPTQICCLLRQVTFGVVFTISVSSVLAKTLTVIIAFNATKPGSKLKKYVGTKLSIILVIV
CSLGESIISIVWLASNPPFSETDFFSDPYNIILQCNEGSGSSFFCIIGYIGTLALLSFIAAFLAKDFPDRFNEAKNITFSMLGFCSVWGAFVPAYLSSKGSRMVAVEIFAILSSSAGLLGCIFIPKCYIIFLRPELNTKDNLVRKQYK
>Western_clawed_frog.locus684.1
CIRCTEYEKSNKEKNSCIPKNIDFLSYKDTLGVTLTSITVICSIICAVILGIFIKYRETPIVRANNRYLSCLLLISLMLCFLCTFLFIGRPTQICCLLRQVTFGVVFTISVSSVLAKTLTVIIAFNATKPGSKLKKYVGTQLAIILVIV
CSLGESIISIVWLASNPPFSETDFFSDPYNIILQCNEGSGSFFFCIIGYIGTLALLSFIAAFLAKDFPDRFNEAKNITFSMLGFCSVWGAFVPAYLSSKGSRMVAVEIFAILSSSAGLLGCIFIPKCYIIFLRPELNTKDNLVRKQYK
>Western_clawed_frog.locus683.1
--------
MSNKEKNGCISKNINVLSYEDMLGATLSFISVICSIACAVILGIFIKYRETPIVRANNRYLSCLLLISLMLCFLCTLLFIGRPTQICCLLRQVTFGIVFTISVSSVLAKTLTVIIAFNATKPGSKLKKYVGTQLSIILVIVCSLGESII
SIVWLVSNPPFSETEFFSDPYYIILQCNEGSGSFFFCIIGYIGTLALLSFIAAFLAKDFPDRFNEAKNITFSMLGFCSVWGAFVPAYLSSKGSRMVAVEIFAILSSSAGLLGCIFIPKCYIIFLRPDINSKHTIVKK---
>Western_clawed_frog.locus643.1
--------
MSNKEKNGCIPKNINFLSYEDMLGATLSFISVICSIACAVILGIFIKYRETPIVRANNRYLSCLLLISLMLCFLCTLLFIGRPTQICCLLRHVTFGIVFTISVSSVLAKTLTVIIAFNATKPGSKLKKYVGTQLATILVIVCSLGESII
SIVWLASNPPFSETDFFSDPYYIILQCNEGSGSFFFCIIEYIGTLALLSFIAAFLAKDFPDRFNEAKNITFSMLGFCSVWGAFVPAYLSSKGSRMV-----AILSSSAGLLGCIFIPKCYIIFLRPDINSKHTIVKK---
>Western_clawed_frog.locus691.1
CIKCSEYEKCNEKRIVCIPKNINFLSYSDPLGATLSSISIIFSIGCSAILGIFIKYRETPIVRANNRYLSCLLLISLMLCFLCTLLFIGRPTQICCLLRQVIFGIVFTISVSSVLAKTLTVIIAFNATKPGSKLKKYVGTQLAIILVFV
NSLGEIIISIVWLASNPPFREADTLSDPNYIILLCNEGSGCFFFCIIGYIGTLALLSFIAAFLAKDFPDRFNEAKNITFSMLGFCSVWGAFVPAYLSSKGSRMVAVEIFAILSSSAGLLGCIFIPKCYIIFLRPELNRKDNIVRKQ--
>Western_clawed_frog.locus636.1
CIKCSEYEKCNEKRIVCIPKNINFLSYSDPLGATLSSISIIFSIGCSAILGIFIKYHETPIVRANNRYLSCLLLISLMLCFLCTLLFIGRPTQICCLLRQVTFGVVFTISVSSVLAKTLTVIIAFNATKPGSKLKKYVGTQLAIILVFV
NSLGEIIISIVWLASNPPFREADTLSDPNYIILLCNEGSGCFFFCIIGYIGTLALLSFIAAFLAKDFPDRFNEAKNITFSMLGFCSVWGAFVPAYLSSKGSRMVAVEIFAILSSSAGLLGCIFIPKCYIIFLRPELNRKDNIVRKQ--
>Western_clawed_frog.locus637.1
CIKCSENEKSNKEQNGCIPKNINFLSYEDTLGATLSFISFLCTVTCTVILGIFIKYRETPIVRANNRYLSCLLLISLMLCFLCTLLFIGRPTQICCLLRQVTFGIVFTISVSSVLAKTLTVIIAFNATKPGSKLKKYVGTQLAIILVIV
CSLVEIVISAVWLASNPPFPEADTLSDPDYIILLCNEGSGFFFFCIIGYIGTLALLSFIAAFLAKDFPDRFNEAKNITFSMLGFCSVWGAFVPAYLSSKGSRMVAVEIFAILSSSAGLLGCIFVPKCYIIFLRPELNIRDTIVRKQ--
>Western_clawed_frog.locus690.1
CIKCSENEKSNKEQNGCIPKNINFLSYEDTLGATLSFISILCTVTCTVILGIFIKYRKTPIVRANNRYLSCLLLISLMLCFLCTLLFIGRPTQICCLLRQVTFGIVFTISVSSVLAKTLTVIIAFNATKPGSKLKKYVGTQLAIILVIV
CSLFVIVISAVWLASNPPFPEADTLSDPDYIILLCNEGSDFFFFCIIGYIGTLALLSFIAAFLAKDFPDRFNEAKNITFSMLGFCSVWGAFVPAYLSSKGSRMVAVEIFAILSSSAGLLGCIFVPKCYIIFLRPELNIRDTIVRKQ--
>Western_clawed_frog.locus613.1
CFKCPASQRSNSQRDGCVPKYIKCLSYEEPLGTSLASAAFLLSITCAVIQGIFIKYRETPIVRANNRYLSCLLLISLMLCFLCTLLFIGRPTQICCLLRQVTFGIVFTISVSSVLAKTLTVIIAFNATKPGSKLKKYVGTQLAIVLVII
CSLGSTVISAVWMVSNPPFSDTDDVSYVDSVILLCNEGSIIYFFSVIGYMGTLALLSFIAAFLAKDFPDRFNEAKNITFSMLGFGSVWGAFVPAYLSSTGTKVVAVEIFAILSSAAGLLGCIFFPKCYIIFLRPELNTRDSIMHK---
>Western_clawed_frog.locus711.1
CFKCPASQRSNSQRDGCVPKYIKCLSYEEPLGTSLASAAFLLSITCAVIQGIFIKYRETPIVRANNRYLSCLLLISLMLCFLCTLLFIGRPTQICCLLRQVTFGIVFTISVSSVLAKTLTVIIAFNATKPGSKLKKYVGTQLPIVLVII
CSLGSTVISAVWMVSNPPFSDTDDVSYVDSVILLCNEGSIIYFFSVIGYMGTLALLSFIAAFLAKDFPDRFNEAKNITFSMLGFGSVWGAFVPAYLSSTGTKVVAVEIFAILSSAAGLLGCIFFPKCYIIFLRPELNTRDSIMHK---
>Western_clawed_frog.locus634.1
CFKCPAFQRSNRKRDGCVPKYEKFLSHEEPLGTSLASAALILSLMCVVILGIFIKYRETPIVRANNRYLSCLLLISLMLSFLCTLLFIGRPTQTCCLLRQVTFGIVFTTSVSTVLVKTLTVIIAFNAAKPVSKLKKYVGTQLAIILVIV
CSLGETVISAVWIVSNPPFIDAD------DVILLCNEGSIIYFFSIVGYMGALALLSFIAAFLAKDFPDRFNEAKNITFSMLIFCSVWGGFVPAYLSSTGTKVVAVEIFAILSSAAGLLGCVFFPKCYIIFLRSELNTRETLIRK---
>Western_clawed_frog.locus712.1
CFKCPAFQRSNSQRDGCVPKYVKCLSYEEPLGTSLTSAALILSVTCAVIQGIFIKYRETPIVRANNRYLSCLLLISLMLCFLCTLLFLGRPTQICCLLRQVTFGIVFTISVSSVLAKTLTVIIAFNATKPGSKLKKYVGTQLSIILVIV
CSLGETVISIVWMASNPPFADTDDVSNVDSVILLCNEGSINYFFCIIGYMGTLALLSFIAAFLAKDFPDRFNEAKNITFSMLGFCSVWGAFVPAYLSSTGTKVVAVEIFAILSSAAGLLGCIFFPKCYIIFLRPQLNTRDYIMHK---
>Western_clawed_frog.locus612.1
CFKCPAFQRSNSQRDGCVPKYVKCLSYEEPLGTSLTSAALILSVTCAVIQGIFIKYRETPIVRANNRYLSCLLLISLMLCFLCTLLFIGRPTQICCLLRQVTFGIVFTISVSSVLAKTLTVIIAFNATKPGSKLKKYVGTQLAIILVIV
CSLGETVISIVWMASNPPFADTDDVSNVDSVILLCNEGSINYFFCIIGYMGTLALLSFIAAFLAKDFPDRFNEAKNITFSMLGFCSVWGAFVPAYLSSTGTKVVAVEIFAILSSGAGLLGCIFFPKCYIIFLRPQLNTRDYIMHK---
>Western_clawed_frog.locus714.1
CFKCPAFQRSNSQRDGCVPEYVKCLSYEEPLGASLASAALLLSVTCAVIQGIFIKYRETPIVRANNRYLSCLLLISLMLCFLCTLLFIGRPTQICCLLRQVTFGIVFTISVSSVLAKTLTVIIAFNATKPGSKLKKYVGTQLAIILVIV
CSLGETVISIVWMASNPPFPDTDDVSNVDSVILLCNEGSIIYFFSVISYMGTLALLSFIAAFLAKDFPDRFNEAKNITFSMLGFCSVWGAFVPAYLSSKGSRMVAVEIFAILSSSAGLLACIFIPKCYIIFLRPELNTKANRII----
>Western_clawed_frog.locus781.1
CLACPEDHWSNQGRDQCIPRIRDFLSYEDPLSAALVCVALVLTLITVLVLGVFILYRDTPIVRANNRNLSYMLLLFLIMSFLCSLLFMGPPDNLTCQLRQVSFGIVFTGSVSSVLAKTITVILAFKATKPGGKLRKWLGNRMSFSLVLI
CSLVQALLCTTWLIRSPPFPDYDTWSVKGQIILQCNEGSVPAFYMVVGYMGFLAALSFLVAFLVRKVPDRYNEAQLITFSMLGFCSVWVSFIPAHLSTKGKYTVAVEIFAILASGAGLLACIFVPKCYIILIRPELNAREKPMIIRGD
>Western_clawed_frog.locus786.1
CMKCPDDQWPNEGRDTCIVKRMEFLSYEDPLGFSLAILAAIVSSLAAGTLGIFIKHKDTPVVKANNQTLSFILLLSLMFCSLCPLLFIGRPTEISCLLCYISFGTFFTVAISTLLAKTFIVTLAFRVVKPDRKVRGWLWKNMFMIVVLL
CFCGEGLIGTTWLAHSPPFPDYNTKLEADKIILRCNEGSVIAFYVQIGYMGLLASFSFIVAFMARKLPDAFNEAQYITFSMLGFCSVWVSFIPAHLGTKGKYMVAVECFAILASSLGLLSCIFIPKCYIILIRPELNIRGSLIVKKQD
>Western_clawed_frog.locus985.1
CVKCAEDQWPNPARDQCIIRVIDFLSQEDHLGNILSGSASVFTVSTAAVCLVFIKHRRTPIVRANNQNISYILLMALLMSFLCSFMFIGQPTGVTCMLRQTTVMFVLSIAISSILGKTLMVLAAFKAAKMEKMRRKLGRINISVGVVFL
CSFGELVICVIWLSLYPPHVESDNKTVPGKIILQCNEGSIISFYLAVSYIGVLSLISFAVAFIARKLPDRFNEAQHITFSMLVFCSVWASFIPTYLSTKGKHMVAVEIFAIQASAAGLLMCIFTPKCYIILLKPELNVKGKPTAKIQS
>Western_clawed_frog.locus984.1
CVKCAEDHWPNPTRDQCIIRVIDFLSQEDHLGNILSGSASVFTVSTAAVCLVFIKHRRTPIVCANNQNISYILLMALLMSFLCTFIFIGQPTGVTCMLRQTTVMFVLSIAISSILGKTLMVLAAFKATQVHKKFRKWIRINISVGVVFL
CSFGEFVLCVIWLSLYPPHVEID-KTIPGKLILQCNEGSIISFYLAVSYIGVLSLISFAVAFIARKLPDRFNEAQHITFSMLVFCSVWASFIPTYLSTKGKHMVAVEIFAIQASAAGLLLCIFTPKCYIILLKPELNVKGKPTAKIQS
>Western_clawed_frog.locus987.1
CVKCAEDQWPNPTRDQCIIRVIEYLSYENLLGYILSGCASVFIVLTAAVCLVFIKHRRTPIVRANNQNISYILLMALLMSFLCSFMFIGQPTGVTCIVRQTTVMFVLSIAISSILGKTLMVLAAFKAAKMEKMFRKLGGINISVGVVFL
CSFGELVICVIWLSLYPPHVESDNKTIPGKIIQQCNEGSIISFYLAVSYIGVLSLISFAVAFIARKLPDRFNEAQHITFSMLVFCSVWASFIPTYLSTKGKHMVAVEIFAIQASAAGLLMCIFTPKCYIILLKPELNVKGKPTAKIQS
>Western_clawed_frog.locus989.1
CVKCAEDQWPNPTRDQCIIRVIDYLSYEDLLGYILSGCASVFTVLNSAVLLVFIKHRRTPIVRANNQNISYILLMALLMSFLCTFMFIGQPTGVTCMLRQTTVMFVLSIAISSMLGKTLMVLAAFQATKMEKMFRKLGRINISVGVVFL
```

CSFVELIICVIWLSLYPPHVESDNKTVPGKIILQCNEGSIISFYLAVSYIGVLSLISFAVAFIARKLPDRFNEAQHITFSMLVFCSVWVSFIPTYLSTKGKHMVAVEIFAIQASAAGLLMCIFTPKCYIILLKPELNVKGKPRVTH--
>Western_clawed_frog.locus986.1
CVKCAEDQWPNPTRDQCIIRVIEYLSYEDLLGYILSGCASVFTVLTSAVFVVFIKHRRTPIVRANNQNISYILLMALLMSFLCTFMFIGQPTGVTCMLRQTTVMFVLSIAISSILGKTLMVLAAFKAAKMEKMFRKLGRINISVGVVFL
CSFGEFVLCVIWLSLYPPHVESDNKTIPGKIILQCNEGSIISFYLAVSYIGVLSLISFAVAFIARKLPDRFNEAQHITFSMLVFCSVWASFIPTYLSTKGKHMVAVEIFAIQASAAGLLMCIFTPKCYIILLKPELNVKGKPTAKIQS
>Western_clawed_frog.locus983.1
CVKCAEAQWPNPAKDQCILRIIDFLSYQDILGHILSGCALVFIVLTAAVFLVFIKHRRTPIVRANNQNISYILLMALLMSFLCSFIFIGQPTGVTCMLRQTTVLLVLSIAISSILGKTTMVLAAFKATKMERTFRKWGRINISVVVVFL
CSFGELVICVIWLSLYPPHVESD-KTIPGKLVLQCNEGSIISFYLAVSYIGVLSLISFAVAFIARKLPDRFNEAQHITFSMLVFCSVWASFIPTYLSTKGKHMVAVEIFAIQASAAGLLMCIFTPKCYIILLKPELNVKGKPTAKIQL
>Western_clawed_frog.locus982.1
CVKCAEDQWPNPARDQCIKRTIDFLSYKDLLGYILSGCASVFIVLTAAVLFVFIKHRRTPIVRANNQNISYILLMALLMSFLCSFIFIGQPTGVTCMLRQALFLIAFSVAISSILGKTSMVLAAFKATKMKKGFRECGRINISVGVVFL
FSLGEFVICVIWLILYPPHVESDNKTLPEKTILQCNEGSIISFYLAVSYIGVLSLISFAVAFIARKLPDRFNEAQHITFSMLVFCSVWASFIPTYLSTKGKHMVAVEIFAIQASAAGLLMCIFTPKCYIILLKPELNVKEKPTAKIQS
>Western_clawed_frog.locus906.1
CVKCAEDQWPNPAKDQCIKRVIDFLSYGDLLGYILSGCASVFTVLTAAVFLVFIKHRRTPIVRANNQNISYILLMALLMSFLCSFIFIGQPTGVTCLLRQTTFVFVFSVAISSVLGKTIMVLIAFKATKIEKKFRMLGGINISLGLVFI
CSFGEFVLCVIWLIINPPHVESDNKTVPGKTILQCNEGSIISFYLAVSYIGVLSLISFAVAFIARKLPDRFNEAQHITFSMLVFCSVWASFIPTYLSTKGKHMVAVEIFAIQASAAGMLMCIFTPKCYIILLQPELNVRGKPTAKILS
>Western_clawed_frog.locus990.1
CEGCAEDQWPNPAKDQCIKRVIDFLSYGDLLGYILSGCASVFIVLTAAVFLVFIKHRRTPIVRANNQNISYILLMALLMSFLCSFIFIGQPTGVTCMLRQTTFVFVFSVAISSILGKTIMVLIAFKATKIEKIFRMLGGINISVGVVFI
CSFGEFVLCVIWLILNPPHVESDNKTVPGRTILQCNEGSIISFYLAVSYIGVLSLISFAVAFIARKLPDRFNEAQHITFSMLVFCSVWASFIPTYLSTKGKHMVAVEIFAIQASAAGLLMCIFTPKCYIILLKPELNVKGKPTAKIQS
>Western_clawed_frog.locus992.1
CVKCAEDQWPNPARDQCIKRVIDFLSYGDLLGYILSGCASVFIVLTAAVCLVFIKHRRTPIVRANNQNISYILLMALLMSFLCSFIFIGQPTGVTCMLRQTTFVFVFSVAISSVLGKTIMVLIAFKATKIEKMFRMLGRMNISVGVVFI
CSFGEFVLCVIWLILNPPHVESDNKTVPGKTILQCNEGSIISFYLAVSYIGVLSLISFAVAFIARKLPDRFNEAQHITFSMLVFCSVWASFIPTYLSTKGKHMVAVEIFAIQASAAGLLLCIFTPKCYIILLKPDLNVKGKPTAKIQS
>Western_clawed_frog.locus991.1
CMKCAEDEWPNPTRDQCIIRVIDFLSYEDLLGYILSGSASVFIVLTAAVFLVFIKHRRTPIVRANNQNISYILLMALLMSFLCSFIFIGQPTGVTCMLRQALFIFSFSVAISSVLGKTITVLIAFKATKMERSFRKWGRINISVVVVFI
CSFGEFVLCVIWLSLYPPHVESD-KTIPGKLVLQCNEGSIISFYLAVSYIGVLSLISFAVAFIARKLPDRFNEAQHITFSMLVFCSVWASFIPTYLSTKGKHMVAVEIFAIQASAAGLLMCIFTPKCYIILLKPELNVKGKLTAKIQS
>Western_clawed_frog.locus988.1
CVKCAEDHWPNPTRDQCIIRVIEYLSYEDLLGYILSGSASVFIVLTSAVCLVFIKHRRTPIVRANNQNISYILLMALLMSFLCTFIFIGQPTGVTCMLRQALFIFSFSVAISSVLGKTITVLIAFKATKMQRTFRKWGRINISVVVVFI
CSFGEFVLCVIWLSLYPPHVESDNKTVPGKTILQCNEGSIISFYLAVSYIGVLSLISFAVAFIARKLPDRFNEAQHITFSMLVFCSVWASFIPTYLSTKGKHMVAVEIFAIQASAAGLLMCIFTPKCYIILLKPELNVKGKPTANIQS
>Western_clawed_frog.locus981.1
CVKCAEDQWPNPTRDQCIKRTIDFLSWDDLLGYILSGCALVFIILTAAVCLVFIKHRRTPIVRANNQNISYILLMALLMSFLCSFMFIGQPTRVTCMLRQITFVFAFSVAISSILGKTMTVLVAFKSTKTSGNFRWWGKMHFSISAVFL
FSFGEFVICVIWLILFPPFVESDNQTIPGNTILQCNEGSIISFYLAVSYIGVLSLISFAVAFIARKLPDRFNEAQHITFSMLVFCSVWASFIPTYLSTKGKHMVAVEIFAIQASAAGLLMCIFTPKCYIILLKPDLNVKGKPTAK---
>Western_clawed_frog.locus993.1
CVKCPDDHWSNPTRNRCIKRITDFLSYEDLLGYILSGCASLFIVNTATVLLVFIKHRRTPIVRANNQNISYILLMALLMSFLCTFMFIGQPTGVTCLLRQALFVCVFSVAISSILGKTMTVLVAFKSAKMANRFRKWGKINISVVIVFV
CSFVELVICVIWLTMSPPYVESDIKTTPGKIILQCNEGSTISFYLAVSYIGVLSLISFAVAFIARKLPDRFNEAQHITFSMLVFCSVWASFIPTYLSTKGKHMVAVEIFAIQASAAGLLMCIFTPKCYIILLKPELNVKANLTFKINP
>Western_clawed_frog.locus905.1
CVKCPDDHWSNPTRNRCIKRIIDFLSYEDLLGYILSACASLFIANTAMVLLVLLKHRRTPIVRANNQKISYILLMALLMSFLCTFMFIGRPTGVTCLLRQALFVCVFSVAISSILGKTMTVLFAFKSTETANRFRKWGKMNISMVIVFL
CSFVEFVICVIWLTTSPPYVESDIKAIPGKIILQCNEGSVISFYLAVSYIGVLSLISFAVAFIARKLPDRFNEAQHITFSMLVFCSVWASFIPTYLSTKGKHMVAVEIFAIQASAAGMLMCIFTPKCYIILLKPELNVKANLTFKRNP
>Western_clawed_frog.locus980.1
CVKCPEDQWSNPTRDQCIQKTIDYLSYEDLLGYILSSCASVFIVFTAVVFLIFIKHRRTPIVRANNQNISYILLMALLMSFLCTFMFIGQPTGVTCMLRQTTVMFVLSIAISSILGKTVTVLVAFKSTKTSSNIRRWGKIYFSMNVVFL
FSFGEFLICIIWLTLSPPYVEVDIKIIPGTIILQCNEGSTISFYLAVSYIGVLSLISFAVAFIARKLPDRFNEAQHITFSMLVFCSVWASFIPTYLSTKGKHMVALEIFAIQASAAGLLMCIFTPKCYIILLKPELNVKGKFTFTIKP
>Western_clawed_frog.locus945.1

MVLATAALLFSVCTGAVLWLFIKNRSSQIVKANNRNLSYILLVSLFLSFCCCFLFIGRPVPITCILRQTAFLFLYTVAISSLLGKTLTVVIVFHATKPGTRLRKFVGSRVSISLVLLCSLGKLAICSTWLIWATPFVALDTKTTQKTMT
LWCNEGSIAAFYVAVAYTAALALLSFIVAFMARKLPDRYNEAQHITFSMLVFFSVWASFIPTYLSTKGKYMVAVEIFAILASTAGLLSCVFVPKCYIILLNPKQITTKTNILIL--
>Western_clawed_frog.locus946.1
CLQCPEDHWSNPSRDSCLKKAVDFLSYGNCLGMVLATAALLFSVCTGAVLWLFIKNRSSQIVKANNRNLSYILLVSLFLSFCCCFLFIGRPVPITCILRQTAFLFLYTVAISSLLGKTLTVVIAFHATKPGTRLRKFVGSRVSISLVLL
CSLGKLAICSTWLIWATPFVALGTKTTQQTMTLWCNEGSIAAFCVAVSYTAVLALLSFIVAFMARKLPDRYNEAQHITFSMLVFFSVWVSFIPTYLSTKGKYMVAVEIFAILASTAGLLFCVFVPKCYSLLLMPKQIIIKTNILLL--
>Western_clawed_frog.locus947.1
CRQCPEDHWSNPSRDFCLRKAVDFLSYGNCLGMVLATAALLFSVCTGAVLWLFIKNRSSQIVKANNRNLSYILLVSLFLSFCCCFLFIGRPVPITCILRQTAFLFLYTVAISSLLGKTLTVIIAFHATKPGTRLRKFVGSRVSISLVLL
CSLGKLAICSTWLIWATPFVALGTKTTQQTMTLWCNEGSIAAFCVAVSYTAVLALLSFIVAFMARKLPDRYNEAQHITFSMLVFFSVWVSFIPTYLSTKGKYMVAVEIFAILASTAGLLSCVFVPKCYIILLKPKQMTTKHNIILL--
>Western_clawed_frog.locus944.1

MVLATAALLFSVCTGAVLWLFIKNRSSQIVKANNRNLSYILLVSLFLSFCCCFLFIGRPVPITCILRQTAFLFLYTVAISSLLGKTLTVIIAFHATKPGTRLRKFVGSRVSISLVLLCSLGKLAICSTWLIWATPFVALGTKTTQQTMT
LWCNEGSIAAFCVAVSYTAVLALLSFIVAFMARKLPDRYNEAQHITFSMLVFFSVWVSFIPTYLSTKGKYMVAVETFAILASTAGLLSCVFVPKCYSIFFKVNLNTEKR-------
>Western_clawed_frog.locus943.1
CLQCPEDHWSDPSRNFCLRKAVDFLSYGNCLGMVLATAALLFSVCTGAVLWLFIKNRSSQIVKANNRNLSYILLVSLFLSFCCCFLFIGRPVPITCILRQTAFLFLYTVAISSLLAKTLTVIIAFHATKPGTRLRKFVGSRVSISLVLL
CSLGKLAICSTWLIWATPFVALGTKTTQKTMTLWCNEGSIAAFYAAVSYTALLALLSFIVAFMARKLPDRYNEAQHITFSMLVFCSVWASFIPTYLSTKGKYMVAVEIFAILASTAGLLSCVFVPKCYIILLKGNLTTQKVKLKIMQQ
>Western_clawed_frog.locus940.1
CVKCPEDHWSNPSRDLCIKRTIDFLSYTEPLGTILTTISVTFCLITTAVLWLFLRHRHTPIVKANNCHLSYILLVSIILSLLCSFLFIGRPTSMSCLLRQITSVLMFSISLSSLLGKTVTVVMAFNATKPRSKFKKWFGSKTSIVMTCI
CSSGELVICIFWLVWDPPFVHYDTKSTHDKITLQCREQSITAFSLAISYVGSLAISCFIVAFLARKLPDRFNEAKHITFSMLVFFSVWVSFIPTYLSVKGKHMVAVEIFTILASNIGLVFCIFMPKCYIIILKPELNTKNQLKYMKFQ
>Western_clawed_frog.locus941.1
CVKCPEDHWSNPSKDLCIKRTIDFLSYTDSLGSTLTAVTVTLSLTTTAVLWLFVRHRNTPIVKANNCHLSYILLVSIILSLLCSFLFIGRPTSMSCLLRQITSVLMFSISLSSLLEKTVTVVMAFNDTKPRSKFRKWFGSKTSVIMTFI
CSSGEFVICVWWMVWAPPYVYYDTKSTHNKITLQCREQSITPFILAISYVGSLAFSCFIVAFLARKLPDRFNEAKHISFSMLVFCCVWVSFIPTYLSTKGKYMVAVEIFTILASNAGLIFCIFMPKCYIIILKPELNTKTHLKYMKSQ
>Western_clawed_frog.locus999.1
CWSCPEDRWSNINKDKCIQKTIDFLSYGDILGVTSSCIAFIFVVITAAVLFVFVKFRRSPIVRANDRNLSFILLVSIMLSFLCSFLFIGYPVELTCMLRQAAFGFIFTVAVSAVLGKTVTVIIAFNATKPNSTFRKWIGTRVSISLVLL
FSFGELFICIIWLICSPPFVDTDTKTVVGTMIIQCNEGSFTAFYVVISYIGLLALFSFILAFLVRKLPDRFNDAQYITFSMLVFCSVWISFIPIYLSSKGKYVVAVEIFTILSSTGGLLLCIFAPKCYIILVKPELNSRKHLLKPK--
>Western_clawed_frog.locus994.1
CLRCPEDKWSNFLKDDCIERTKDFLSYGDILGVTSSCIAFIFVVITAAVLFVFVKFRRSPIVRANDRNLSYILLVSIMLSFLCSFLFIGYPVELTCMLRQAAFGFIFTVAVSAVLGKTVTVIIAFNATKPNSTFRKWIGTRISIGLVLF
FSLGELFICIIWLICSPPFVDTDTKTIPGTIIIQCNEGSFAAFYVVISYIGLLALFSFIVAFLVRKLPDRFNDAQYITFSMLVFCSVWISFIPIYLSSKGKYVVAVEIFTILSSTGGLLLCIFAPKCYIILVKPELNSRKHILKPK--
>Western_clawed_frog.locus998.1
CWSCPEDKWSNLNKDKCIERNIDFLSYGSMLGDTLCSIASIFLVTTAAVLFVFVKFRRSPIVRANDRNLSFILLMSIMLSFLCAFLFIGYPVELTCMLRRAAFGFIFTVAVSAVLGKTVTVIIAFNATKPNSTFRKWIGTRISIGLVLL
FSFGELFICIAWLICSPPFVDTDTKTIPGTIIIQCNEGSFAAFYVVISYIGLLALFSFILAFIVRKLPASFNDAQYITFSMVVFCSVWISFIPIYLSSKGKYVVAVEIFTILVSTGGLLLCIFAPKCYIILAKPELNSKKHLLKPR--
>Western_clawed_frog.locus995.1
CWSCPEDKWSNPNKDKCIQRTKDFLSYRCILGATLCSIASIFLVITAAVLFVFVKFRRSPIVRANNRNLSFILLVSIMLSFLCSFLFIGYPVELTCMLRQAAFGFIFTVAVSSVLGKTVTVIIAFAATKPNSTFRKWVGTRVSISLVLL
FSFGELFICIIWLICSPPFVDTDSKTIPGTIIIQCNEGSFTAFYVVISYIGLLALFSFIVAFLVRKLPDRFNDAQYITFSMVVFCCVWISFIPIYLSSKGKYVVAVELFAILSSTGGLLLCIFAPKCYIILVKPELNSRKHLLKPK--
>Western_clawed_frog.locus507.1
CMSCPEEEWPNSKKDTCVRKKIDFMSSQDVLGATLEAFALLLIFMTLAVLMIFIIYRDTPIVRANNRDLSYTLLICLMLSFLCTFSFIGPPTTEICLLRQVAFSLIFTVAVSSVVAKTITVLIAFRSTKPGSNLSKWTGKKLSAFIIIL
CTLVELGICTWSLVQFPPFPNRDTKTYTDRIILQCIAASSMGFYTTHGYILTLSLFNFALAFLVRKLPDRFNEAHYITFSMLLFCSVWISFIPAYLSAKGKYMVAVEVFAILASSAGLFCCIFIPKCCTILLKPQLNTRVKLSVSNKP
>Western_clawed_frog.locus508.1
CMSCPEEEWANSKKDTCVRKNIDFLSSQDALGATLVAFALLLIFMTLSVLVIFIIYRDTPIVRANNRDLSYTLLICLMLSFLCTFSFIGHPTTAICLLRQVAFSLIFTVAISSIVAKTITVLIAFRSTKPGSKLSKWTGKKLSAFIIIL
CTLVELGICTWSLVQFPPFPNRDTKTYTDRIILQCIATSSMGFYTTHGYILTLSLFNFALAFLVRKLPDRFNEAHYITFSMLLFCSVWISFIPAYLSAKGKYMVAVEVFAILASSAGLFCCIFIPKCCTILLKPHLNSRIKLSVYK--
>Western_clawed_frog.locus506.1
CITCPEEEWPNSKKDTCVRKNIDFLSSQDVLGATLEAFALLLIFMTLSVLVILIIYRDTPIVRANNRDLSYTLLISLKLSFLCPFFFIGCPTTEICLLRQVVFSLIFTVAVSSVVAKTITVLIAFRSTKPGSKLSKWTGKKLSAFVVIL
CTLVELGICTWSLVQFPPFSNRDTKTYTDRIILQCIAASSMGFYTTHGYILTLSLFNFALAFLVRKLPDRFNEAHYITFSMLLFCSVWISFIPAYLSAKGKYMVAVEVFAILASSAGLFYCIFIPKCCTILLKPHLNNRVKLSVSNKP
>Western_clawed_frog.locus505.1
CMTCPEEEWPNSKKDTCVRKNIDFLSSQDVLGATLEAFALLLTFMTLSVLGIFIIYRDTPIVRANNRDLSYTLLISLMLSFLCPFLFIGRPTTAICLLRQVVFSLIFTVAVSSVVAKTITVLIAFRSTKPGSKLSKWTGKKLSAFVVIL
CTLVELGICTWSLVQFPPFPNRDTKTYTDRIILQCIAASSIGFYTTHGYILTLSLFNFALAFLVRKLPDRFNEAHYITFSMLLFCSVWISFIPAYLSAKGKYMVAVEVFAILASSAGLFCCIFIPKCCTILLKPQLNTRVKLSVSNKP
>Western_clawed_frog.locus928.1
CIRCPEDQWSNERRDKCIMRLVEFLSYKEPLGVALTTTALVLSFCSAAVFCLFLKHKKSPIVKANNQELSYILLLSLMLSFLCSLLFIGRPTKATCLLRQAVFGIIFAICISSILGKTITVIIAFTATRPGSRLRNYVGTRVPKYILLL
CTLPEVFICALWLIISPPFPDYDTHSATGKIILQCNEGSPSAFYIMVGYIALLAFVSFFVAYLARKLPDIFNEAQYITFSMLLFCSVWISFILAYVSTTGKYLAAVEIFAIFVSSAGLLGLIFVPKCYIIFFKTPVSTRAHLI-----
>Western_clawed_frog.locus927.1
CIRCPEDQWSNERRDKCIIRLVEFLSYKEPLGVALTTTALVLSFCSAAVFCLFLKYKKSPIVKANNQELSYILLLSLMLSFLCSLLFIRRPTKVTCLLRQAVFGIIFAICISSILGKTITVIIAFTATRPGSRLRNYVGTRVPKYILLL
CTLPEVFICALWLIIPPPFPDYDTHSATGKIILQCNEGSPSAFYIMVGYIALLAFVSFFVAYLARKLPDIFNEAQYITFSMLLFCSVWISFILAYVSTTGKYLAAVEIFAILVSSAGLLGLIFIPKCYIIFFKTPVSTRAHLI-----
>Western_clawed_frog.locus929.1
CIRCPEDQWSNESRDKCIMRLVEFLSYKEPLGVALTTTALVLSFCSAAVFCIFLKHKKSPIVKANNQELSYILLLSLMLSFLCSLLFIGRPTKVTCLLRQAVFGIIFAICISSILGKTITVIIAFTATRPGSRLRNYVGTRVPKYILLL
CTLPEVFICALWLIISPPFPDYDTHSATGKIILQCNEGSPSAFYIMVGYIALLAFVSFFVAYLARKLPDIFNEAQYITFSMLLFCSVWISFILAYVSTTGKYLAAVEIFAILVSSAGLLGLIFIPKCYFIILKPSVSLRAHLI-----
>Western_clawed_frog.locus933.1
CIKCPEDQWSNERRDKCIMRTIDFLSYKEPLGVALTTTALVLSICSAAVFFLFLKHKKSPIVKANNQELSYILLLSLMLSFLCSLLFIGRPTKATCFLRQVVFGIIFAICISSILGKTITVIIAFNATRPGSRLRNYVGTRVPKYILLL
CTLPEVFICALWLIISPPFPDYDTHSATGKIILQCNEGSPSAFYIMVGYIALLAFVSFFVAYLARKLPDIFNEAQYITFSMLLFCSVWISFILAYVSTTGKYLAAVEIFAILVSSAGLLGLIFIPKCYFILIKQPVITRHN-------
>Western_clawed_frog.locus926.1
CMKCPEDQWSNASRDKCTMKAIDFLSYKDLLGVALTTTALVLSFCSAAVFCIFLKYKKSPIVKANNQELSYILLLSLMLSFLCSLLFIGRPTKVICLLRQAVFGIIFTICISSILGKTITVIIAFTATRPGSLLRNYVGTRVPKYILLL
CTLPEVFICALWLIISPPFPDYDTHSATGKIILQCNEGSPSAFYIMVGYIALLAFVSFFVAYLARKLPDIFNEAQYITFSMLLFCSVWISFILAYVSTTGKYLAAVEIFAILVSSAGLLGLIFVPKCYIISFKTPVSTRAHLI-----
>Western_clawed_frog.locus930.1
CMKCPEDQWSNESRDKCTMRTIDFLSYKDLLGVALTTTALVLSFCSAAVFCLFLKYKKSPIVKANNQELSYILLLSLMLSFLCSLLFIGRPTKVTCLLRQAVFGIIFAICISSILGKTITVIIAFNATRPGSRLRNYVGTRVPKYILLL
CTLPEVFICALWLIISPPFPDYDTHSATGKIILQCNEGSPSAFYIMVGYIALLAFVSFFVAYLARKLPDIFNEAQYITFSMLLFCSVWISFILAYVSTTGKYLAAVEIFAILVSSAGLLGLIFVPKCYIIFFKPSVSTRGHLM-----
>Western_clawed_frog.locus932.1
CLRCPEDQWSNESRDKCIMRAIDFLSYKDIFGVALTTTALVLSFCTSAVFCLFLKHKKSPIVKANNQELSYILLLSLMLSFLCSLLFIGRPKKVTCLLRHTSFGIIFAICISSILGKTITVIIAFNATRPGSRLRNYVGTRVPKYILLL
CTLPEVFICALWLIISPPFPDYDIHSATGKIILQCNEGSPSAFYIMVGYIALLAFVSFFVAYLARKLPDIFNEAQYITFSMLLFCSVWISFILAYVSTTGKYLGAVEIFAILVSSAGLLGLIFIPKCYFIIFKPSVSPRGHLL-----
>Western_clawed_frog.locus931.1
CMRCPEDQWSNESRDKCIMRAIDFLSYKDLFGVALTTTALVLSCFTSAVFCIFLKYKKSPIVKANNQELSYLLLLSLMLSFLCSLLFIGRPTKVTCLLRQTSFGIIFAICISSILGKTITVIIAFTATRPGSRLRNYVGTRVPKYILLL
CTLPEVFICALWLIISPPFPDYDTHSATGKIILQCNEGSPSAFYIMVGYIALLAFVSFFVAYLARKLPDIFNEAQYITFSMLLFCSVWISFILAYVSTTGKYLAAVEIFAILVSSAGLLGLIFIPKCYFIFFKPPVSTRGHLI-----
>Western_clawed_frog.locus934.1
CMRCLEDQWSNEKRTKCIMRATDFLSYEDPLGVTLTATALFLTFCTAAVFCIFIKYKKSPIVKANNQELSYLLLISLFLSFLCSLLFIGRPTKVTCLLRQTLFGIIFAICISSILGKTITVIIAFTATRPGSRLRNYVGTRVPKYILLL
CTLPEVFICALWLIISPPFPDYDTHSATGKIILQCNEGSPSAFYIMVGYIALLAFVSFFVAYLARKLPDIFNEAQYITFSMLLFCSVWISFILAYVSTTGKYLAAVEIFAILVSSAGLLGLIFIPKCYFILFKPPVSTRGHLVEQKL-
>Western_clawed_frog.locus935.1
CIKCPEDQWSNEKRSKCIMRTIDFLSYEDLLGIVLTTTALVLSSCTSAVFYIFIKYKKSPIVKANNQELSYILLLSLLVSFLCSLLFIGRPTKVTCLLRQAVFGIDFAICVSSILGKTVTVIIAFKATRPGSRLRNYVGVRVSKYILLL
CTLPEVFICALWLIISPPFPDYDTHSATGKIILQCNEGSPSAFYIMVGYIALLAFVSFFVAYLARKLPDIFNEAQYITFSMLLFCSVWISFLLAYVSTTGKYLAAVEIFAILVSSAGLLGLIFIPKCYIIIIKPAVSTRCQLLE----
>Western_clawed_frog.locus937.1
CVRCPKTQWSNEQRDKCIMRALDFLSFEDPLGMFLAFLAISLSILSTAVLIIFIKYKYTTIVRANNRELSYYLLVSLILSFLCSLLFIGWPMKGTCLLRQVVFAVTFTFSISSVLGKTLTVVIAFNATRPGSKLRGWVGTRIPKCLVLL
CTLVEIFICSLWLIISPPFPEYDTESHTATMILQCNEGSALAFYSVIGYIALLSFICLFVAYLSRKLPDIFNEAQYITFSMLVFCSVWVSFIPAYLSTKGKYLTSVEIFAILASGSGLLGLIFVPKCYIMLLKPQQNTNMYLVQLKHK
>Western_clawed_frog.locus936.1

```
CVRCPKIQWSNEQRDKCIMKTLDFLSFEDPLGLSLAFMAIFLSILTTAVLIIFIKYKYTTIVRANNRELSYYLLISLILSFLCSLLFIGWPMKGTCLLRQVVFAVTFTFSISSVLGKTLTVVIAFSATRPGSKLRGWVGTRIPKCLVLL
CTLVEIFICSLWLIISPPFPEYDTESHTATMILQCNEGSAFAFYTVVGYIALLAFICLFVAYLSRNLPDIFNEAQYITFSMLVFCSVWVSFIPAYLSTKGKYVTSVEIFAILASGSGLLALIFVPKCYIILFKPELKPRLGLIQLKHM
>Western_clawed_frog.locus938.1
CVRCPKIQWSNEQRDKCIMRALDFLAFEDPLGLSLSFMAIFLSILTTAVLIIFIKYKYTTIVRANNRELSYYLLISLILSFLCSLLFIGWPMKGTCLLRQVVFAVTFTFSISSVLGKTLTVVIAFNATRPGSKLRVWVGTRIPKCIVLL
CTLVEIFICSLWLIISPPFPEYDTESHTATMILQCNEGSAFAFYTVVGYIALLAFICLFVAYLSRKLPDIFNEAQYITFSMLVFCSVWVSFIPAYLSTKGKYLTSVEIFAILASGSGLLALIFVPKCYIILLKPEKNTRIYLIQLKNT
>Western_clawed_frog.locus939.1
CVRCPKIQWSNEQRDKCIMKTLDFLSFEDPLGLSLAFMAIFLSILATAVLIIFIKYKHTTIVRANNRELSYYLLISLILSFLCSLLFIGWPMKGTCLLRQVVFAITFTFSISSVLGKTLTVVIAFNATRPGSKLRVWVGTRIPKCLVLL
CTLVEIFICSLWLIISPPFPEYDTESHTATMILQCNEGSAFAFYTVVGYIAFLAFICLFVAYLSRKLPDIFNEAQYITFSMLVFCSVWVSFIPAYLSTKGKYLTSVEIFAILASGSGLLALIFVPKCYIILLKPEQNTRIYLIQLKNK
>Western_clawed_frog.locus912.1
CIRCQEDEWSNEKKNKCIKKTIDFLSYAEPLGLSLAILAILLSITASLVLGIFIKNRHSQIVKTNNRELSYTLLLTLILSFLCTLLFIGQPMKVTCMLRQVTFAVIFTTSISSVLGKTVTVLIAFHAITPGSMLKIWVGKRSPRSLVLL
LSMGEVIICIMWLLHCPPFPDYDTRSQPSVMTLQCNEGSAVAFYIAVGYIGMLASVSFITAYLARSLPDIFNEASHITFSMLVFCCVWIAFIPTYLSSKGKYMVAVEIFAILSSSSGLLGCIFIPKCYIILLKPTKSIREKATSKKNK
>Western_clawed_frog.locus911.1
CIRCQEDEWSNEKKNKCIKKTIDFLSYAEPLGLSLAILAILLSITASLVLGIFIKNRHSQIVKANNRELSYTLLLTLILSFLCTLLFIGQPMKVTCMFRQVTFAVIFTTSISSVLGKTVTVLIAFHAITPGSMLKIWVGNRVPRCLVLL
LSLGEVIICIMWFLHCPPFPDYDTRSQPSVMTLQCNEGSAVAFYIAVGYIGMLASVSFITAYLARRLPDIFNEASHITFSMLVFCCVWIAFIPTYLSSKGKYMVAVEIFAILSSSSGLLGCIFIPKCYIILLKPKKSVMEKATSKKK-
>Western_clawed_frog.locus910.1
CIRCQEDEWSNENRIKCIKKTIDFLSYAEPLGLSLAILAILLSITASLVLGIFIRNRHSQIVKANNRELSYSLLLTLILSFLCTLFFIGRPLKVTCMLRQVTFAVIFTISISSVLGKTVTVLIAFHAITPGSMLKIWVGKRVPRCLVLL
LSLGEVIICITWFLHCPPFPDYDTRSQPSVMTLQCNEGSAVAFYIAVGYIGMLASVSFITAYLARGLPDIFNEASHITFSMLVFCCVWIAFIPTYLSSKGKYMVAVEIFAILSSSSGLLGCIFIPKCYIILLKPTKSVKEKATSKKNK
>Western_clawed_frog.locus903.1
CIKCPEDEWSNENRNKCIKKTIDFLSYAEPLGLSLAILAILLSITASLVLGIFVRNRHSQIVKANNQELSYSLLLTLILSFLCTLLFIGKPMKVTCMFRQVTFAVIFTISISSVLGKTVTVLIAFHAITPGSMLKIWVGKRAPRCLVLL
LSLGEVIICIMWFLHCPPFPDYDTRSQPSVMTLQCNEGSAVAFYIAVGYIGMLASVSFITAYLARGLPDIFNEASHITFSMLVFCCVWIAFIPTYLSSKGKYMVAVEIFAILSSSSGLLGFIFIPKCYIILFKPKNTVKEKATSKKNK
>Western_clawed_frog.locus909.1
CIKCPEDEWSNENRNKCIKKTIDFLSYAEPLGLSLAILAILLSITASLVLGIVIKNRHSQIVKANNQELSYSLLLTLILSFLCTLLFLGKPMKVTCMFRQVTFAVIFTTSISSVLGKTVTVLIAFHAITPGSMLKIWVGKRVPRCLVLL
LSLGEVIICIMWFLHCPPFPDYDTRSQPSVMTLQCNEGSAVAFYIAVGYIGMLASVSFITAYLARGLPDIFNEASHITFSMLVFCCVWIAFIPTYLSSKGKYMVAVEIFAILSSSSGLLGCIFIPKCYIILLKPKKRVKEKATSKKK-
>Western_clawed_frog.locus914.1
CITCPEDEWSNENRNKCIKKTITFLSYAEPLGLILANLAVLLSITASLILGILIKNRHSQIVKANNEELSYSLLLTLILSFLCTLLFIGKPMKVTCMFRQVTFAVIFTISISSVLGKTVTVLIAFHAITPGSMLKIWVGKRAPRCLVLL
LSLGEVIICFMWFLHCPPFPDYDTRSQPSVMTLQCNEGSAVAFYIAVGYIGMLASVSFITAYLARRLPDIFNEASHITFSMLVFCCVWVTFIPAYISSKGKYMVAVEIFAILSSSSGLLGCIFFPKCYIILLKPERNISWKATSTKKS
>Western_clawed_frog.locus913.1
CIRCQEDEWSNEKKNKCIKKTIDFLSYAEPLGLSLAMLAILLSITASIVLGIFIRNRQSQIVKANNQELSYSLLLTIILSFLCTLLFIGKPKKVTCLLRQITFAVIFTISISSVLVKTMTVLIAFHAITPGSMLKILVRKSVSRCFILV
FSLGEVIICIMWFLHCPPFPDYDTRS--SVMTLQCNEGSAVAFYIAVGYIGMLASVSFITAYLARGLPDIFNEASHITFSMLVFCCVWITFIPAYISSKGKYMVAVEIFAILSSSSGLLGFIFLPKCYIILLKPEMNVKGKGTSKKK-
>Western_clawed_frog.locus917.1
CIKCKEHEWTDERRDTCIQRETEYLSFHDYLGIFLTATSLFLCVITTGIYSIFYRHRATCIVRANNLHLSYILLFSLTLSFLSSLLFIGRPKQVTCLVRQVTFGIIFATELSTLIGKTITVIIAFSATKPGSKLAKWMKTQITYRILLL
LLIGQVIICSVWLVCSPPFPDTDTKSKTGMIIVLCNEGSVVAFYIMIGYIGILAIVSFLLAYYARRLPDSFNESQLITFSMLVFCSVWVSFIPAYINTKGRSVVAVEVFAILTSNAGLLGFIFTPKCYIILFRPELNNKEHLMRKI--
>Western_clawed_frog.locus918.1
CIKCKAHEWTDDRRDKCIQRETEYLSFHDYLGICLTAICLFLCVITTGIYSIFYRHRATCIVRANNLHLSYILLFSLTLSFLSSLLFIGRPKQVTCLVRQVTFGIIFATELSTLIGKTITVIIAFSATKPGSKLAKWMKTQITYRILLL
LLIGQVIICSVWLVCSPPFPDTDTKSKTGMIILLCNEGSVVAFYIMIGYIGILAIVSFLLAYYARRLPDSFNESQLITFSMLVFCSVWVSFIPAYINTKGRSVVAVEVFAILTSNAGLLGFIFIPKCYIILFRPELNTKEHLMRKI--
>Western_clawed_frog.locus920.1
CIKCKEHEWTDERRDTCIQRETEYLSFHDYLGIFLTATSLFLCVITTGIYSIFHRHRATCIVRANNLHLSYILLFSLTLSFLSSLLFIGRPNEVTCLVRQVTFGIIFATELSTLIGKTITVIIAFSATKPGSKLAKWMKTQITYSIVLL
LSIGQVVICSVWLICSPPFPDTDTKSKTGMIIVLCNEGSVVAFYIMIGYIGILAIVSFLLAYYARRLPDSFNESQLITFSMLVFCSVWVSFIPAYINTKGRSVVAVEVFAILTSNAGLLGFIFIPKCYIILLRPELNNKEHLMRKM--
>Western_clawed_frog.locus919.1
CIKCKEHEWTDERRDTCIQREEEYLSFHDYIGIFWMATSLLLCAITTGIYSIFYRHRATCIVRANNLHLSYILLFSLTLSFLSSLLFIGRPKKVTCLVRQVTFGIIFATELSTLIGKTITVIIAFSATKPGSKLAKWLKTQITYRIVLL
LSFGQVIICSVWLICSPPFPDTDTKSKTGMIIVLCNEGSVVAFYIMIGYIGILAIVSFLLAYYARRLPDSFNESQLITFSMLVFCSVWVSFIPAYINTKGRSVVAVEVFAILTSNAGLLGFIFIPKCYIILFRPELNNKDHLMRKM--
>Western_clawed_frog.locus922.1
CLKCNEDQWTDERRDKCIQRETEYLSFHDYLGSILMGISLCLCATATLIYLMFYRHRKTCIVRANNLVLSYILLFSLTLSFLSSLLFIGRPRNVTCLVRQVTFGVIFATAISAIIGKTITVIIAFSATKPGSKLAKWTKTQITYRIVLL
LTNGQVIICLIWLICSPPFPDTDTKSMAGKIILLCNEGSVVAFYIMIGYIGILAIVSFLLAYYARRLPDSFNESQLITFSMLVFCSVWVSFIPAYINTKGRSVVAVEVFAILTSNAGLLGFIFIPKCYIILFRPELNNKKHLMRKI--
>Western_clawed_frog.locus923.1
CLKCNEDQWTDERRDKCIQRETEYLSFHDYLGSILMGISLCLCATATLIYLMFYRHRTTCIVRANNLVLSYILLFSLTVSFLSSLLFIGRPRNVTCLVRQVTFGVIFATALSAIIGKTITVIIAFSATKPGSKLAKWTKTQITYRIVLL
LTNGQVVICSIWLICSPPFPDTDTKSKTGMIIVLCNEGSVVAFYIMIGYIGILAIVSFLLAYYARRLPDSFNESQLITFSMLVFCSVWVSFIPAYINTKGRSVVAVEVFAILTSNAGLLGFIFIPKCYIILFRPELNNKKYLMRKI--
>Western_clawed_frog.locus924.1
CLKCNEDQWTDERRDKCIQRETEYLSFHDYLGSILMGISLCLCATATLIYLIFYRHRKTCIVRANNLVLSYILLFSLTVSFLSSLLFIGRPRNVTCLVRQVTFGVIFATALSTIIGKTITVIIAFSATKPGSKLAKWMKTQITYRIVLL
LTNGQVVICLFWLICSPPFPDTDTKSKTGQIILLCNEGSVVAFYIMIGYIGILAIVSFLLAYYARRLPDSFNESQLITFSMLVFCSVWVSFIPAYINTKGRSVVAVEVFAILTSNAGLLGFIFFPKCYIILIRPQLNNKENLMRKTKI
>Western_clawed_frog.locus916.1
CIKCNEHQWTDERRDKCIQRETEYLSFHDYLGSILMGISLLLCMMSTVIYLIFYRHRTTCIVKANNLFLSYILLFSLTLSFLSSLLFIGHPRNVTCLVRQMTFGIIFATALSTLIGKTMTVVIAFSATKPGSKLAKWVGSQITNISVLL
LSLGQVTICLFWLICCPPFPDTDTKSMAGKMIVLCNEGSVVAFYIMIGYIGILAIVSFLLAYYARRLPDSFNESQLITFSMLVFCSVWVSFIPAYINTKGRSVVAVEVFAILTSNAGLLGFIFIPKCYIILLRPELNKKEHLMRKT--
>Western_clawed_frog.locus915.1
CIKCNEHQWTDERRDKCIQRETEYLSFHDYLGSILMGTSLLLCMMSTVIYLIFYRHWTTCIVKANNLLLSYILLFSLTLSFLSSLLFIGHPKKGTCLIRQMTFGIIFATALSTLIGKTMTVVIAFSATKPGSKLAKWMGSQITNISVLL
LSLGQVTICLFWLICCPPFPDTDTKSITGKMILLCNEGSVVAFYIMIGYIGILAIVSFLLAYYARRLPDSFNESQLITFSMLVFCSVWVSFIPAYINTKGRSVVAVEVFAILTSNAGLLGFIFIPKCYIILLRPELNKKEHLMRKT--
>Western_clawed_frog.locus850.1
CITCAEDQWSNENRTMCISKTIDFLSYEDALGIALSVTAIFLSIVPLGVLGIFLKHSETAIVKANNRELSYILLLSLSMCSLCSLLFIGRPVALSCFFRQAAFGIIFTVAVSSILSKTITVVIAFNATKPGSQARKWVGSRVSTYLVSL
GAFGEAVICLVWFLCSPPFPDFDTSENSAKMTLKCNEGSEIAFYCVTGYVGLLAALSFVVAFLARNLPDNFNEATYITFSMLVFFSVWVSFIPAYLSTKGKYMVAVEIFAILASTAGLVSCIFAPKCYIIVIRPELNTKEHLIGKKVN
>Western_clawed_frog.locus848.1
CMACSEDHWSDEKRTMCISRTIDFLSFSDVLGITLAFVAIFLCLISLGVLSIFLKYRNTAIVKANNRELSYILLLSLIMCFLCCFLFIGRPEASTCFFRQAAFGIIFAVAVSAILAKTITVVIAFNATKPGSRARKWVGSNISAYLVSL
GAFGEAVICLVWFLCSPPFPDFDTTEKSAKMTLKCNEGSEVAFYCVVGYVGLLAALSFVVAFLARKLPDTFNEATHITFSMLVFCIVWVSFIPAHVSAKGKYTVAVEIFAILASTAGLLGCIFIPKCHIIVIRPDLNTKEHLIGRKVK
>Caecillan.locus22632.1
CKKCPQDQWSNEKRDKCIPRIIDFLSYEEPLGVTLASIAVFFSIVTSGVLGIFLKYKDTPIVKANNRELSYIFLLSLILSFLCSLIFIGRPSNVTCLLRQVAFGIIFAIAVSSVLAKTITVVIAFNATKPGSNLKKWIGTRVSGSIVLL
CSLGEVMICIIWLSHSPPFPDKDVQSEIGKMILQCNEGSVIAFYTVVGYMGFLALLSFTVAFLARKLPDSFNETQLITFSMLVFCSVWVAFIPAYLSTKGKYMVAVEIFAILTSSAGLLACIFIPKCYIILLRPDINTKEHLIGKKTN
>Western_clawed_frog.locus385.1
CIQCPEDQWPNDKRTICIQKITEFLSYEDPLGQSLAAVSALSSLTVILVFLIFLKYHKTPVVKANNQTLSYSLLLSLTLSFLCCFLFIGRPQKVTCLLRQVTFGINFTLSVSCVLAKTVTVAIAFNATKPGSKIKKWVGTRVSLCLVLL
CSLLQVGICLVWLISSPPFPDYDTHTYTGKMILQCNEGSVTAFYTVIGYLGFLSGLSFIVAFLVRKLPASFNEAQLITFSMLVFCSVWVSFIPAYLSTKGKYMVAVEIFAILASSAGLLGCIFIPKCYIILFRPEQNTRRGLTGKHLQ
>Western_clawed_frog.locus380.1
CIQCPEDQWPNDNRTICIQKITEFLSYEDPLGQSLAAVSVLSSLTVISVLLIFIKYHKTPVVKANNQTLSYSLLLSLTLSFLCCFLFIGRPQKVTCLLRQVTFGINFTLSVSCVLAKTVTVAIAFNATKPGSKIKKWVGTRVSLCLVLL
CSLLQVGICLVWLISSPPFPDYDTHTYTGKMILQCNEGSVTAFYTVIGYLGFLSGLSFIVAFLVRKLPASFNEAQLITFSMLVFCSVWVSFIPAYLSTKGKYMVAVEIFAILASSAGLLGCIFIPKCYIILFRPEQNTRRGLTGKHLQ
>Western_clawed_frog.locus382.1
CIQCPEDQWPNDNRTICIQKITEFLSYEDPLGQSLAAVSVLSSLTVILVFLIFIKYHKTPVVKANNQTLSYSLLLSLTLSFLCCFLFIGRPQKVTCLLRQVTFGINFTLSVSCVLAKTVTVAIAFNATKPGSKIKKWVGTRVSLCLVLL
CSLLQVGICLVWLISSPPFPDYDTHTYTGKMILQCNEGSVSAFYTVIGYMGFLSGLSFIVAFLVRQLPASFNEAQLITFSMLVFCSVWVSFIPAYLSTKGKYMVAVEIFAILASSAGLLGCIFIPKCYIILFRPEQNTRRGLTGKHLQ
>Western_clawed_frog.locus379.1
CIQCPEDQWPNDNRTICIQKITEFLSYEDPLGQSLAAVSVLSSLTVISVLLIFIKYHKTPVVKANNQTLSYSLLLSLTLSFLCCFLFIGRPQKVTCLLRQVTFGINFTVSVSCVLAKTVTVAIAFNATKPGSKIKKWVGTRVSLCLVLL
CSLLQVGICLVWLISSPPFPDYDTHTYTGTMILQCNEGSVSAFYTVIGYMGFLSGLSFIVAFLVRKLPASFNEAQLITFSMLVFCSVWVSFIPAYLSTKGKYMVAVEIFAILASSAGLLGCIFIPKCYIILFRPEQNTRRGLTGKHLQ
>Western_clawed_frog.locus383.1
CIQCPEDQWPNDKRTICIQKITEFLSYEDPLGQSLAAVSVLSSLTVISVLLIFIKYHKTPVVKANNQTLSYSLLLSLTLSFLCCFLFIGRPQKVTCLLRQVTFGINFTLSVSCVLAKTVTVAIAFNATKPGSKIKKWVGTRVSLCLVLL
CSLLQVGICLVWLISSPPFPDYDTHTYTGKMILQCNEGSVSAFYTVIGYMGFLSGLSFIVAFLVRKLPASFNEAQLITFSMLVFCSVWVSFIPAYLSTKGKYMVAVEIFAILASSAGLLGCIFIPKCYIILFRPEQNTRRGLTGKHLQ
>Western_clawed_frog.locus381.1
CIQCPEDQWPNDKRTICIQKITEFLSYEDPLGQSLAAVSVLSSLTVISVLLIFIKYHKTPVVKANNQTLSYSLLLSLTLSFLCCFLFIGCPQKVTCLLRQVTFGINFTLSVSCVLAKTVTVVIAFNATKPGSKIKKWVGTRVSLCLVLL
CSLLQVGICLVWLISSPPFPDYDTHTYTGKMILQCNEGSVSAFYTVIGYMGFLSGLSFIVAFLVRKLPASFNEAQLITFSLLVFCSVWVSFIPAYLSTKGKYMVAVEIFAILASSAGLLGCIFIPKCYIILFRPEQNTRRGLTGKHLQ
>Western_clawed_frog.locus388.1
CIQCPEDQWPNDKRTICIQKITEFLSYEDPLGQSLAAVSVLSSLTVILVFLIFMKYHKTPVVKANNQTLSYSLLLSLTLSFLCCFLFIGRPQKVTCLLRQVTFGINFTVSVSCVLAKTVTVVIAFNATKPGSKIKKWVGTRVSLCLVLL
CSLLQVGICLVWLISSPPFPDYDTHTYTGKMILQCNEGSVSAFYTVIGYMGFLSGLSFIVAFLVRKLPASFNEAQLITFSMLVFCSVWVSFIPAYLSTKGKYMVAVEIFAILASSAGLLGCIFIPKCYIILFRPEQNTRRGLTGKHLQ
>Western_clawed_frog.locus386.1
CIQCPEDQWPNDKRTICIQKITEFLSYEDPLGQSLAAVSVLSSLTVILVFLIFLKYHKTPVVKANNQTLSYSLLLSLTLSFLCCFLFIGRPQKVTCLLRQVTFGINFTVSVSCVLAKTVTVVIAFNATKPGSKIKKWVGTRVSLCLVLL
CSLLQVGICLVWLISSPPFPDYDTHTYTGKMILQCNEGSVSAFYTVIGYIGFLSGLSFIVAFLVRKLPASFNEAQLITFSMLVFCSVWVSFIPAYLSTKGKYMVAVEIFAILASSAGLLGCIFIPKCYIILFRPEQNTRRGLTGKRLQ
>Western_clawed_frog.locus389.1
CIQCPEDQWPNDNRTICIQKITEFLSYEDPLGQSLAAVSVLSSLTVISVFMIFIKYHKTPVVKANNQTLSYSLLLSLTLSFLCCFLFIGRPQKVTCLLRQVTFGINFTVSVSCVLAKTVTVVIAFNATKPGSKIKKWVGTRVSLCLVLL
CSLLQVGICLVWLISSPPFPDYDTHTYTGKMILQCNEGSVSAFYTVIGYMGFLSGLSFIVAFLVRKLPASFNEAQLITFSMLVFCSVWVSFIPAYLSTKGKYMVAVEIFAILASSAGLLGCIFIPKCYIILFHPEQNTRRCLTGKH--
>Caecillan.locus36895.1
CLKCPDDQWPTKKQDGCIPRTIEFLSYEDPLGAALASLAIVLCAITAGTLGIFLKYRETAVVKANNRDLSFILLLSLMLSFLCSLLFIGRPEKLTCLLRQTAFGIIFSVSVSAVLAKTITVVIAFKATKPGSRLREWVGSRVLSFLVLL
CSLGEVLICTVWLLISPPFPDYDIQTDLGRMILICNEGSLFAFYSVIGYMGLLAILSFIVAFLARRLPNSFNEAQLITFSMLVFCSVWVSFIPVYLSTKDKYMVTVEIFAMLLSSIGLLSCIFIPKCYIILLRPEMNTREHLIGKQST
>Caecillan.locus36885.1
CLKCPDDQWPTKKQDGCIPRTIEFLSYEDPLGAALASLAIVLCAITAGTLGIFLKYRETAVVKANNRDLSFILLLSLMLSFLCSLLFIGRPEKLTCLLRQTAFGIIFSVSVSAVLAKTITVVIAFKATKPGSRLREWVGSRVLSFLVLL
CSLGEVLICTVWLLISPPFPDYDIQTDLGRMILICNEGSLFAFYSVIGYMGLLAILSFIVAFLARRLPNSFNEAQLITFSMLVFCSVWVSFIPVYLSTKDKYMVTVEIFAMLLSSIGLLSCIFIPKCYIILLRPEMNTREHLIGKQST
>Caecillan.locus36898.1
CLKCPDNQWPTKKRDGCIPRTIEFLSYEDPLGAALASLAIVLCAITAGTLGIFLKYRETAVVKANNRDLSFILLLSLMLSFLCSLLFIGRPEKLTCLLQQTAFGIIFSVSVSAVLAKTITVVIAFKATKPGSRLREWVGSRALSFLVLL
CSLGEVLICTVWLLISPPFPDYDIQTDPGKMILICNEGSLFAFYSVIGYMGLLAILSFIVAFLARRLPNSFNEAQLITFSMLVFCSVWVSFIPVYLSTKDKYMVTVEIFAMLLSSTGLLSCIFIPKCYIILLRPEMNTREHLIGKQST
>Caecillan.locus36884.1
CLKCPDDQWPTKKRDGCIPRTIEFLSYEDPLGASLASLAIVLCAITAGTLGIFLKYRETAVVKANNRDLSFILLLSLMLSFLCSLLFIGRPEKLTCLLRQTAFGIIFSVSVSAVLAKTITVVIAFKATKPGSRLREWVGSRALSFLVLL
CSLGEVLICTAWLLISPPFPDYDIQTDPGRMILICNEGSLFAFYSVIGYMGLLAILSFIVAFLARRLPNSFNEAQLITFSMLVFCSVWVSFIPVYLSTKDKYMVTVEIFAMLLSSTGLLSCIFIPKCYIILLRPEMNTREHLIGKQST
>Caecillan.locus36883.1
CLKCPDDQWPTKKRDGCIPRTIEFLSYEDPLGASLASLAIVLCAITAGTLGIFLKYRETAVVKANNRDLSFILLLSLMLSFLCSLLFIGRPEKLTCLLRQTAFGIIFSVSVSAVLAKTITVVIAFKATKPGSRLREWVGSRALSFLVLL
CSLGEVLICTAWLLISPPFPDYDIQTDPGRMILICNEGSLFAFYSVIGYMGLLAILSFIVAFLARRLPNSFNEAQLITFSMLVFCSVWVSFIPVYLSTKDKYMVTVEIFAMLLSSTGLLSCIFIPKCYIILLRPEMNTREHLIGKQST
>Caecillan.locus36892.1
CLKCPDDQWPMKKRDGCIKRTIEFLSYEDPLGAALASLAIVLCAITAGTLGIFLKYRETAVVKANNRDLSYILLLSLMLSFLCSLLFIGRPEKLTCLLRQTAFGIIFSVSVSAVLAKTITVVIAFKATKPGSRLREWVGSRALSFLVLL
CSLGEVLICTAWLLISPPFPDYDIQIDPGRMILICNEGSLFAFYSVIGYMGLLAILSFIVAFLARRLPNSFNEAQLITFSMLVFCSVWVSFIPVYLSTKDKYMVTVEIFAMLLSSTGLLSCIFIPKCYIILLRPEMNTREHLIGKQST
>Caecillan.locus36893.1
CLKCPDDQWPMKKRDGCIKRTIEFLSYEDPLGAALASLAIVLCAITAGTLGIFLKYRETAVVKANNRDLSYILLLSLMLSFLCSLLFIGRPEKLTCLLRQTAFGIIFSVSVSAVLAKTITVVIAFKATKPGSRLREWVGSRALSFLVLL
CSLGEVLICTAWLLISPPFPDYDIQIDPGRMILICNEGSLFAFYSVIGYMGLLAILSFIVAFLARRLPNSFNEAQLITFSMLVFCSVWVSFIPVYLSTKDKYMVTVEIFAMLLSSTGLLSCIFIPKCYIILLRPEMNTREHLIGKQST
>Caecillan.locus36891.1
CLKCPDDQWPMKKRDGCIPRTIEFLSYEDPLGAALAFLAIVLCAITAGTLGIFLKYRETAVVKANNRDLSYILLLSLMLSFLCSLLFIGRPEKLTCLLRQTAFGIIFSVSVSAVLAKTITVVIAFKATKPGSRLREWVGSRVLSFLVLL
CSLGEVLICTAWLLISPPFPDYDIQTDAGKMILICNEGSLFAFYSVIGYMGLLAILSFIVAFLARRLPNSFNEAQLITFSMLVFCSVWVSFIPVYLSTKDKYMVTVEIFAMLLSSTGLLSCIFIPKCYIILLRPEMNTREHLIGKQSP
>Caecillan.locus36886.1
CLKCPDDQWPMKKRDGCIPRTIEFLSYEDPLGAALAFLAIVLCAITAGTLGIFLKYRETAIVKANNRDLSYILLLSLMLSFLCSLLFIGRPEKLTCLLRQTAFGIIFSVSVSAVLAKTITVVIAFKATKPGSRLREWVGSRVLSFLVLL
```

```
CSLGEVLICTAWLLISPPFPDYDIQTDPGKMILICNEGSLFAFYSVIGYMGLLAILSFIVAFLARRLPNSFNEAQLITFSMLVFCSVWVSFIPVYLSTKDKYMVTVEIFAMLLSSTGLLSCIFIPKCYIILLRPEMNTREHLIGKQSP
>Caecillan.locus36896.1
CLKCPDDQWPKKKRDGCIPRTIEFLSYEDPLGAALASLAIVLCAITAGTLGIFLKYRETAVVKANNRDLSYILLLSLMLSFLCSLLFIGRPEKLTCLLRQTAFGIIFSVSVSAVLAKTITVVIAFKATKPGSRLREWVGSRALSFLVLL
CSLGEVLICTAWLLISPPFPDYDIQTDPSRMILICNEGSLFAFYSVIGYMGLLAILSFIVAFLARRLPNSFNEAQLITFSMLVFCSVWVSFIPVYLSTKDKYMVTVEIFAMLLSSTGLLSCIFIPKCYIILLRPEMNIREHLIGKPCK
>Caecillan.locus36888.1
CLKCPDDQWPKKKRDGCIPRTIEFLSYEDPLGAALASLAIVLCAITAGTLGIFLKYRETAVVKANNRDLSFILLLSLMLSFLCSLLFIGRPEKLTCLLRQTAFGIIFSVSVSAVLAKTITVVIAFKATKPGSRLREWVGSRVLNFLVLL
CSLGEVLICTAWLLISPPFPDYDIQTDPGRMILICNEGSLFAFYSVIGYMGFLAILSFIVAFLARRLPNSFNEAQLITFSMLVFCSVWVSFIPVYLSTKDKYMVTVEIFAMLLSSTGLLSCIFIPKCYIILLRPEMNIREHLIGKPCK
>Caecillan.locus36882.1
CLKCPDDQWPKKKQDGCIPRTIEFLSYEDPLGAALASLAIVLCAITAGTLGIFLKYQETAVVKANNRDLSYILLLSLMLSFLCSLLFIGRPEKLTCLLRQTAFGIIFSVSVSAVLAKTITVVIAFKATKPGSRLREWVGSRALSFLVLL
CSLGEVLICTAWLLISPPFPDYDIQTDPGKMILICNEGSLFAFYSVIGYMGFLAILSFIVAFLARRLPNSFNEAQLITFSMLVFCSVWVSFIPVYLSTKDKYMVTVEIFAMLLSSTGLLSCIFIPKCYIILLRPEMNTREHLIGKQST
>Caecillan.locus36900.1
CLKCPDDQWPTKKRDGCIPRTREFLSYEDPLGAALASLAIVLCAITAGTLGIFLKYRETAVVKANNRDLSYILLLSLMLSFLCSLLFIGRPEKLTCLLRQTAFGIIFSVSVSAVLAKTITVVIAFKATKPGSRLKEWVGSRVLSFLVLL
CSLGEVLICTAWLLISPPFPDYDIQTDPGKMILICNEGSLFAFYSVIGYMGFLAILSFIVAFLARRLPNSFNEAQLITFSMLVFCSVWVSFIPVYLSTKDKYMVTVEIFAMLLSSTGLLSCIFIPKCYIILLRPEMNTREHLIGKKST
>Caecillan.locus44251.1
CMRCPEDQWPSERKDECLPRVIDFLSYDEPLGKALSSFSIILAIITTLVLGIFIKYQETPVVKANNRNLSYILLISLLLSFLCILIFIGHPGAVTCLVRQSAFGIIFSTAVSSVLAKTVTVVIAFGATKPSSKLRKWVGTRVSSYIVLL
CTSGETVLCMVWLLISPPFPEYDTQSETGKMIPQCNEGSIIAFYSMIGYMGFLALLSFIVAFLVRKAPDSFNEAQFITFSMLVFCSVWVSFIPAYLSTKGKYTVAVEIFAILTSSAGLLGCIFIPKCYIILLRPDLNTRGHLIGKQHP
>Caecillan.locus44250.1
CMRCPEDQWPSEKKDECLPRVIDFLFYDKPLGKALSSFSIILAIITTLVLGIFIKYQETPVVKANNRNLSYILLISLLLSFLCILIFIGHPGAVTCLVRQSAFGIIFSTAVSSVLAKTVTVVIAFGATKPSSKLRKWVGTRVSSYIVLL
CTSGETVLCMVWLLISPPFPEYDTQTETGKMISQCNEGSIIAFYSMIGYMGFLALLSFTVAFLVRKAPDSFNEAQFITFSMLVFCSVWVSFIPAFLSTKGKYTVAVEIFAILSSSAGLLGCIFIPKCYIILLRPDLNTRGH-------
>Caecillan.locus44450.1
CMRCPEDQWPSEKKDECLPRVIDFLSYDEPLGKALSSFSIILAIITALVLGIFIKYQETPVVKANNRNLSYILLISLLLSFLCIFIFIGHPGAVTCLVRQSAFGIIFSTAVSSVLAKTVTVVIAFGATKPSSKLRKWVGTRVSSYIVLL
CTSGETVLCTVWLLISPPFPEYDTQSETGKMIPQCNEGSIIAFYSMIGYMGFLALLSFTVAFLVRKAPDSFNEAQFITFSMLVFCSVWVSFIPAYLSTKGKYTVAVEIFAILTSSAGLLGCIFIPKCYIILLRPDLNTRGHLIGKQHP
>Caecillan.locus44258.1
CKKCPEDQWPNEKKDGCLPRVIDFLSYDDPLGIALSTISFLLFSITAVLLGIFIKYRKTAVVKANNRNLSYILLISLMLSFLSTLLFIGQPGTVTCLLRQSAFGIIFTIAVSSVLAKTVIVVIAFSATKPNSRLRKWVGTRVSSYVVSL
CTFGEVLICTIWLFISPPFPEYDTQSEKGKMIPQCNEGSAIAFYTVIGYMGFLALLSLIVAFLVRKVPDSFNEAQFITFSMLVYCSVWVSFIPVYLSTKGKYIAAVEIFAILTSSGGLLGCIFTPKCYIILLRPDLNTKDHLIGKQHP
>Caecillan.locus44261.1
CMKCPEDQWPSEKKDECLPRVIEFLSYYDPLGIALSSISILFTIITALVLGIFIKYRDTPMVKANNRNLSYTLLISLMLSFFCILIFIGHPGTVTCLLRQSAFGIIFSIAVSSVLAKTVTVVIAFSATNPTSKLRKWVGSRVSCYLVLL
CTSGEVLMCTIWLLSSTPFPEYDTQSVTGKMLLRCNEGSTIAFYSVIGYMGFLAFSSFIVAFLVRKVPDSFNEAQHITFSMLVFCSVWVSFIPAYLSVKGKYMVAVEIFAILTSSAGLLGCIFIPKCYIILFRPDLNKREQLTGKQYP
>Caecillan.locus44442.1
-MKCPEDQWPNEKKDKCNPKVIEFLSYGEPLGIALSSVSILLSIITALILGIFIKYRNTPLVKANNLNLSYVLLVSLTLSFLCALMFIGRPGAVSCLLRQSAFGIIFSTAVSSVLAKTITVVIAFSATKPSSKLSKWVGTRVSSYLILL
CTSGEIVICILWLLISPPYPDYDTQSETGKMILQCNEGSTFAFYLVIGYMGFLALLSFIVAFLVRKLPDTFNEAQFITFSMLVFCSVWVSFIPAYLSIKGKYMVVVELFAILASSAGLLGCIFIPKCYIVLFRPDLNTREHLIEK---
>Caecillan.locus44440.1
CMKCPEDQWPNEKKDKCNPKVIEFLSYGEPLGIALSSVSILLSIITALILGIFIKYRNTPLVKANNLNLSYVLLVSLTLSFLCALMFIGRPGAVSCLLRQSAFGIIFSTAVSSVLAKTVTVVIAFSATKPSSKLSKWVGTRVSSYLILL
CTSGEIVICILWLLISPPYPDYDTQSDTGKMILQCNEGSTFAFYLVIGYMGFLALLSFIVAFLVRKLPDSFNEAQFITFSMLVFCSVWVSFIPAYLSIKGKYMVVVELFAILASSAGLLGCIFIPKCYIVLFRPDLNTREHLIEK---
>Caecillan.locus44444.1
CMKCPEDQWPNEKKDKCNPKVIEFLSYGEPLGIALSSVSILLSIITALILGIFINYRNTPLVKANNLNLSYVLLVSLMLSFLCTLMFIGRPGEVSCLLRQSAFGIIFSTAVSSVLAKTVTVVIAFSATKPSSKLSKWVGTRVSSYLILL
CTSGEIVICILWLLISPPYPDYDTQSETGKMILQCNEGSTFAFYLVIGYMGFLALLSFIVAFLVRKLPDSFNEAQFITFSMLVFCSVWVSFIPAYLSIKGKYMVVVELFAILASSAGLLGCIFIPKCYIVLFRPDLNTREHLIEK---
>Caecillan.locus44437.1
CMKCPEDQWPNEKKDGCLPRIIEFLSYDDSLGIALSSISISFLIIINALVLGIFIKYRDTPVVKANNRNLSYILLISLKMSFLCSFVFINSPGSVTCLLRQTAFAIIFTVAVSSVLAKTITVAVAFNAAKPSSKLRKWVGTRVSSYLVLL
CSSGEFVICIVWLFISPPYPEYDTQSEIGKMILQCNEGSTIAFYSVIGYIGFLAILSFVVAFLVRKLPDSFNEAQFITFSMLVFCSVWVSFIPAYLSTKGKYMVAVEIFAILSSSAGLLGCIFIPKCYIILLRPDLNTRGHVIGKQYP
>Caecillan.locus44439.1
CMKCPEDQWPNEKKDGCLPRIIEFLSYDDSLGIALSSISISFLIIINALVLGIFIKYRDTPVVKANNRNLSYILLISLKMSFLCSFVFINSPGSVTCLLRQTAFAIIFTVAVSSVLAKTITVAVAFKAAKPSSKLRKWVGTRVSSYLVLL
CSSGEFVICIVWLFISPPYPEYDTQSEIGKMILQCNEGSTIAFYSVIGYIGFLAILSFVVAFLVRKLPDSFNEAQFITFSMLVFCSVWVSFIPAYLSTKGKYMVAVEIFAILSSSAGLLGCIFIPKCYIILLRPDLNTRGHVIGKQYP
>Caecillan.locus44252.1
CMKCPEDHWPNENQNECLQRAIIFLSYDDLLGAVLASIAIIFSLISALVLGIFIKFRDTPLVKANNRDLSYILLFSLMLTFLCSLVFIGHAGRVTCLLRQAAFAIIFTIAVSSVLAKTVTVVIAFSATKPSSKLSKWVGTRISSYLVLL
CSSGEIVICILWLLISPPFPEYDTKTETGQMILQCNEGSTIAFYSVIGYIGFLTFLSFIVAFLVRKLPDSFNEAQHITFSMLVFCSVWVSFIPAYLSTKGKYMVAVEIFAILASSAGLLGCIFIPKCYIILVRPDLNTRKHLTGKQHL
>Caecillan.locus44452.1
-MKCPEDHWPNEKQDDCLPRTITFLSYDDLLGAVLASIAIFFFLISALVLGIFIKFRDTPLVKANNRDLSYILLFSLMLTFLCSLVFIGHAGRVTCLLRQAAFGIIFTIAVSSVLAKAVTVVIAFSATKPSSKLSKWVGTRVSSYLVLL
CSSGEIVICILWLLISPPFPEYDTKTVTGQMILQCNEGSTIAFYSVIGYIGFLAFLSFIVAFLVRKLPDCFNEAQHITFSMLVFCSVWVSFIPAYLSTKGKYMVAVEIFAILASSAGLLGCIFIPKCYTILLRPDLNTRMHLTGKHHL
>Caecillan.locus44451.1
-MRCPEDHWPNENHNDCLPRTIIFLSYEDPLGAVLASVAIFFSIISALVLGIFITFRDTPLVKANNRDLSYILLFSLMLTFLCSLVFIGQAGRVTCLLRQAAFGIIFTIAVSSVLAKAVTVVIAFSATKPSGKLSKWVGTRVSSYLVLL
CSSGEIVICILWLLISPPFPEYDTKTVTGQMILQCNEGSTIAFYSVIGYIGFLAFLSFIVAFLVRKLPDCFNEAQFITFSMLVFCSVWVSFIPAYLSTKGKYMVAVEIFAILASSAGLLGCIFIPKCYIILLRPDLNTRMHLTGKQHL
>Caecillan.locus44253.1
CMRCPEDHWPNENHNDCLPRTIIFLSSDDPLGAVLASVAIFFSIISALVLGIFITFRDTPLVKANNRDLSFILLFSLMLAFLCSLMFIGHAGRVTCLLRQAAFGIIFTIAVSSVLAKTVTVVIAFSATKPSGKLSMWVGTRVSSYLVLL
CSSGEIVICILWLLISPPFPEYDIKTETGQMILQCNEGSTIAFYSVIGYIGFLAFLSFIVAFLLRKLPNSFNEAQFITFSMLVFCSVWVSFIPAYLSTKGKYMVAVEIFAILASSAGLLGCIFIPKCYIILVRPDLNTRMNLIGKQHF
>Caecillan.locus44300.1
-MKCPEDQWPSEKKDQCLPRVIEFLSYDDLLGAAVASIAVLFSIITATILGIFIKYRDTPVVKANNRDLSYILLTFLILSFLCPLLFIGRTGRVNCLLRQVSFGIIFGTAISSVLAKTITVLLAFKTTKPNSKLSKWVGIRVSNYVVLL
CSSGEIVICTAWLLISPPFPDYDTQSEKGKMILQCNEGSTIAFYSVIGYVGFLALLSFIVAFLARKLPDGFNEAQHITFSMLVFCSVWVSFIPAYLSTKGKYMVAVEIFAILASSAGLLGCIFLPKCYIIVLRPDLNTKGHLTGKKKK
>Caecillan.locus44297.1
CFNCPEDQWPSEKKDQCLPRVIEFLSYDDLLGAAVAFIAVLFSIITAIILGIFIKYRDTPVIKANNRDLSYILLTFLILSFLCPLLFIGRAGRVNCLLRQVSFGIIFGTAISSVLAKTITVLLAFKATKPNSKLSKWVGTRVSSYVVLL
CSSGEIVICIAWLLISPPFPDYDTQSEKGKMILQCNEGSAIAFYSVIGYVGFLALLSFIVAFLARKLPDGFNEAQHITFSMLVFCSVWVSFIPAYLSTKGKYMVAVEIFAILASSAGLLGCIFLPKCYIIVLRPDLNTKGHLTGKKKI
>Caecillan.locus46850.1
----------------------------------
MASIAVLFSIITATMLGIFIKYRDTPVVKANNRDLSYILLTSLILSFLCPMLFIGHTGRVNCLLRQVSFGIIFGTAISSVLAKTITVLLAFKATKPNSKLSKWVGARVSNYVVLLCSSGEIVICTAWLLISPPFPDYDTQSEKGKMILQ
CKEGSTIAFYSVIGYVGFLALLSFIVAFLARKLPDGFNEAQHITFSMLVFCSVWVSFILAYLSTKGKYMVAVEIFAILASSAGLLGCIFLPKCYIIVLRPDLNTKGHLTGKKFF
>Caecillan.locus44290.1
CVKCPEDQWPSEKKEQCLPRVIEFLSYNDLLGAAVASIAVFFSIITATILGIFIKYRDTPVVKANNRDLSYILLTSLILSFLCPLLFIGRTGRVNCLLRQVSFGIIFGTAISSVLAKTITVLLAFKATKPNSKLSKWVGTRVSNYVVLL
CSSGEIVICTAWLLISPPFPDYDIQSEKGKMILQCNEGSTIAFYSVIGYVGFLALLSFIVAFLARKLPDSFNEAQLITFSMLVFCSVWVSFIPAYLSTKGKYMVAVEIFAILASSAGLLGCIFLPKCYIIVLRPDLNTKDHLTGKKFS
>Caecillan.locus44273.1
----------------------------------
MASIAVLFSIITATILGIFIKYRDTPVVKANNRDLSYILLASLILSFLCPMLFIGRTGKVNCLLRQVSFGIIFGTAISSVLAKTITVLLAFKATKPNSKLSKWVGTRVSNYLVLLCSSGEIVICTAWLLISPPFPDYDTQSEKGKMILQ
CNEGSTIAFYSVIGYVGFLALLSFIVAFLARKLPDSFNEAQLITFSMLVFCSVWVSFIPAYLSTKGKYTVAVEIFAILASSAGLLGCIFLPKCYIIMLRPDLNTKGHLTGKKFS
>Western_clawed_frog.locus378.1
CMKCPKDQWPNKRRDRCLQKLLEFLSYQDPLGAGLGGASIGLSACAITILGVFIKFRATPVVRANNTNISYTFLVSLCLSFLSCLLFIGQPKPLACMVRQAAFGLAFSIAESSILAKAVTVAVAFRATSPDSQLRRWVGPRLPIYIVIG
CSVGQAVICLAWMVLSPPFPDYDIQSEKGKMILICNEGSAILLYVEISYLGILALLSFTVAFLVRNLPDCFNEAKYITFSMLVFLSVWVSFIPSYLSTKGKYMVAVEIFAILGSSTGLLACIFIPKMCLILLKRGLSSKVHVFSYPKL
>Western_clawed_frog.locus374.1
CMKCPKDQWPNKRRDRCLQRLVEFLSYQDPLGAGLGGASIGLSACAITILGVFIKFRATPVVRANNTNISYTLLVSLCLSFLSCLLFIGQPKPLACMVRQAAFGLAFSIAESSILAKAVTVAVAFRATSPDSQLRRWVGPRLPIYIVIG
CSVGQAAICLAWMVLSPPFPDYDIQSEKGKMILICNEGSAVLLYVEISYLGVLALLSFTVAFLVRNLPDRFNEAKYITFSMLVFLSVWVSFIPSYLSTKGKYMVAVEIFAILGSSTGLLACIFIPKMCLILLKRGLSSKVHVTGMDNK
>Western_clawed_frog.locus375.1
CMKCPEDQWPNKRRDRCLQRLVEFLSYQDPLGAGLGGASIGLSACAITILGVFIKFRATPVVRANNTNISYTLLVSLCLSFLSCLLFIGQPKPLACMVRQAAFGLAFSIAESSILAKAVTVAVAFRATSPDSQLRRWVGPRLPIYIVIG
CSVGQAVICLAWMVLSPPFPDYDIQSEKGKMILICNEGSAVLLYVEISYLGVLALLSFTVAFLVRNLPDRFNEAKYITFSMLVFLSVWVSFIPSYLSTKGKYMVAVEIFAILGSSTGLLACIFIPKMCLILLKRGLSSKVHVTGMDNK
>Western_clawed_frog.locus376.1
CMKCPEDQWPNKRRDRCLQRLVEFLSYQDPLGAGLGGASIGLSACAITILGVFIKFRATPVVRANNTNISYTLLVSLCLSFLSCLLFIGQPKPLACMVRQAAFGLAFSIAESSILAKAITVAVAFRATSPDSQLRRWVGPRLPIYIVIG
CSVGQAVICLAWMVLSPPFPDYDIQSEKGKMILICNEGSAVLLYVEISYLGVLALLSFTVAFLVRNLPDRFNEAKYITFSMLVFLSVWVSFIPSYLSTKGKYMVAVEIFAILGSSTGLLACIFIPKMCLILLKRGLSSKVHVTGMDNK
>Western_clawed_frog.locus377.1
CMKCPEDQWPNKRRDRCLQRLVEFLSYQDPLGAGLGGASIGLSACAITILAVFIKFRATPVVRANNTNISYTLLVSLCLSFLSCLLFIGQPKPLACMVRQAAFGLAFSIAESSILAKAITVAVAFRATSPDSQLRRWVGPRLPIYIVIG
CSVGQVAICLAWMVLSPPFPDYDIQSEKGKMILICNEGSAVLLYVEISYLGVLALLSFTVAFLVRNLPDRFNEAKYITFSMLVFLSVWVSFIPSYLSTKGKYMVAVEIFAILGSSTGLLACIFIPKMCLILLKRGLSSKVHFTGMDNK
>Small-eyed_rabbitfish.locus500.1
CLTCPLEYRSNDKRDHCLLKEIEFLSFEETLGIVLVTLALLGVCLTISVFTAFYVHRDTPIVKANNSELSFLLLVALALCFLCSVAFIGEPSVWSCRLRHMAFGITFVLCISCVLGKTIVVVMAFNANLPNKNMMKWFGPTQQRLSVFI
LALIQCLICAVWLSLSPPYPLKNSEYFTERIVFECHVGSAVAFYCVLGYFGLLSCVCFGVAFLARRLPDNFNEAKHITFSMLIFCAVWITFIPAYVSSPGKHTVAVEIFAILASSFGLLACIFAPKCYIIL-----------------
>Small-eyed_rabbitfish.locus322.1
CLTCPLEYRSNDKRDHCLLKEIEFLSFEETLEIVLVTLALLGVCLT------
FYVHRDTPIVKANNSELSFLLLVALALCFLCSVTFIGEPSVWSCRLRHTAFGITFVLCISCVLGKTIVVVMAFNASLPSNNMMKWFGPTQQRLSVFIRTLIQCLICTVWLSLSPPYPLKNSEYFTERIVFECHVGSAVAFYCVLGYIGL
FSCVCFGVAFLARRLPDNFNDVKHITFSMLIFCAVWITFIPVYVSSPGKHIVVGEIFAILASSFGLLACIFAPKCYIILLKKEENSKKHMMGIMAS
>Small-eyed_rabbitfish.locus332.1
CLTCPLEYQSNDKRDHCLLKEIEFFSFEETTGIVLVTRALLGVCLTISVFTAFYVHRDTPIVKANNSELSFLLLVVLALCFLRSVTFFGEPSVWSCRLCHMAFGITFVLCISCVLGKTIVVVMAFNATLPSNNMMKWFGPTQQRLSVFM
LTLIQCLICTVWLSLSPPYPLKNSEYFTERIVFECQVGSAVAFYCVLGYIGLLSCVCFGIAFLARRLPDNFNAAKHLTFSMLIFCAVWITFIPPYVIYPGKHTVAVEIFAILASSFVLLACIFAPKCYIILLKKELNSKKHMMGKVAS
>Small-eyed_rabbitfish.locus355.1
CLTCPLEYQSNDKRDHCLLKEIEFLSFEETLGIVLVTLALLGVCLTISVFTAFYIDRDTPIVKANNSELIFLLLVALDLCFLCSVTFIGEPSVWSCRLRHMAFGITFVPCISCVLGKTIVVVLAFNATLPSNNMMKWFGPTQQRLSVFI
LTLIQCLICTVWLSLSPPYPLKNSEYFAERIVFECHLGSAVAFYCVLGYIGLLSCVCFGVALLARRLPDNFNEAKHITFSMLIFCAVWITFIPSYVSSPGKHTVAVEIFAILASSFGLLAFIFAPKCYIILLKKEENTKKHMMGKVAS
>Small-eyed_rabbitfish.locus69.1
CLPCPLEYRSNDKRDHCLLKEIEFLSFEETLGIVLVTLALLGVCLTISVFTAFYVHRDTPIVKANNSELSFLLLVALALCFLCSVTFIGEPSVWSCRLRHMAFGITFVLCTSCVLGKTIVAVMEFNATLPSNNMMKWFGPTQQRLSVFI
LTLIQCLIRTVWLSLSPAYPLKNSEYFTERIVFECHVGSAVAFYFVLGYIGLLSCVCFGVAFLASRLPDNFNEAKHVTFSMLIFCAVWITFIPAYVSSPGKYTVAVEIFAILTSSFELLACIFAPKCYIILLKKEENSKKHMMGKVAS
>Small-eyed_rabbitfish.locus376.1
CLTCPLEYRSNDKRDHCLLKEIEFLSFEETLGIVLVTLALLGVCLTISVFAAFYVHRDTPIVKANNSELSFLLLVALALCFLCSVTFIGEPSVWSCRLRHVSFGITFVLCISCILGKTIVVVMAFNATLPSNNMMKWFGPTQQRLSVFI
LTLIQCLICTVWLSLSPPYPLKNSEYFTERIVFECHVGSAVAFYCVLGYIGLLSCVCFGVAFLARRLPDNFNEAKHITFSMLIFCAVWITFIPAYISSPGKYTVAVEIFAILASSFGLLACIFAPKYYIILLKKEENTKKHMMGKVAS
>Small-eyed_rabbitfish.locus651.1
-------------------
KEIEFLSFEETLGIVLVTLALLGVCLTISVFTAFYVHRDTPIVKANNSELSFLLLVAIALCFLCSVTFIGEPSVWSCRLRHMAFGITFVLCISCILGKTVVVVMAFNATLPSNNMMKWFGPTQQRLSVFILTLIQCLICTVWLSLSPPY
PLKNSEYFTERIVFECHVGSAVAFYCVLGYIGLLSCVCFGVAFLARRLPDNFNEAKHITFSMLIFCAVWITFIPAYVSSPGKYTVAVEIF---------------------------------------
>Spotted_gar.locus28.1
CIKCPWDYWPNSEKTACFPKQMEFLSFGEIMGSALTALGIFGACLTLFVTAVFFRFRSTPVVRANNSEISFLLLVALVFCFLCPLLFVGEPSPWSCMVKHAAFGIIFVLCVACVLGKTFVVLMAFRSTLPGSNTMKWFGPVQQRFTILL
FASIQIMICALWLGISPPFPQKNASLFNDKIILECNLGSVTAFCCVLGYIGVLAVMCFIFAFLARKLPDSFNEAKYITFSMLIFCAVWIAFIPAYVSSPGKYTVAVEIFAILSSSFAVLLCIFAPKCYIILLKPEKNTKRNIMGRSNI
>Japanese_eel.locus277.1
----------------------------------------------
MAVAVVFLMNVSTPLGKVNDPALTLSLLFSLVVTFLCSIVFLGRPQHWSCMTSQVALALGFALCLSSIMGKSIVLMLRARVLKEAKEARDPIQPIHQRALVVVCTLIQAVACTVWLILLPPHPIKNTAAQNIKIILECDEGSIVFICCI
FAYDILLALLAFVFAFVARKLEDHFGEAKCITFGMLVFFIVWISFVPAYLSTRGKFMVAVQIFAILASSFGLLCCIFLPKCYVFLVKPDRNLEELLKPRPKI
>Western_clawed_frog.locus867.1
```

```
-------
MWPTERHDGCRPRSLEYLAYGDPLGGTLASLSIVGSLIPLSILGIFLRNSKTPVVKANNRNLSYLLLFALFLCFLCSLMFIGAPVNLICVLRQITFGISFVLCVSCVLGKTIMVVIAFNATQPKSSRRMWLNSRIPNTLVIVCMTIQLI
ICSVWIMYSPPFKNDDITSKLGVTILECDEGSPVAFWCMI------------------KLPGSFNEAKLITFSMLIFGAVWISFIPTYLSTRGKESVAVEIFAILCSCSGLLALLFFPKCYIIILRPEMNNKELLTGKRGF
>Caecillan.locus40460.1
CMRCPEDKWPNEKRDVCIPKIISFLSHEEPLGIVLILLSLFFFLITTVILGIFIKYRDTPIVRSNNTNLSYILLVSLMLCFFCSLIFIGHPEEVACVLRQTAFGITFSISLSSVLAKTITVVMAFQSTKPGSKLRKWMGSRVSYSVVLF
FSFLQVLLCLVWLVTAPPFPHLNMQAEDGTIQIECNEGSAIAFYCVLGYLGFLAGISFIVAFLARNLPGSFNEAKHITFSMLVFCSVWVSFIPTYLSTRGKYMVAVEIFAILASSSGILGCIFVPKCYIILLKPHRNNRKYITKK---
>Caecillan.locus40458.1
CMRCPEDKWPNEKRDVCIPKIISFLSHEEPLGIVLILLSLFFFLITTVILGIFIKYRDTPIVRSNNTNLSYILLVSLMLCFLCSLIFIGHPEEVACILRQTAFGITFSISLSSVLAKTITVVMAFQSTKPGSKLRKWMGSRVSYSVVFS
FSLFQVLLCLVWLVTAPPFPHLNMQAEDGTIQIECNEGSAIAFYCVLGYLGFLAGISFVVAFLARNLPGSFNEAKHITFSMLVFCSVWVSFIPTYLSTRGKYMVAVEIFAILASSSGILGCIFVPKCYIILLKPHRNNRKYITKK---
>Caecillan.locus40462.1
CMRCPEDQWPNEKRDVCIPKIISFLSHKEPLGIVLMLLSLFFFLITTVILGIFIKYRDTPIVRSNNRNLSYILLVSLILCFLCSLIFIGHPEEVTCFLQQTAFGITFSISLSSILAKTITVVMAFQSTKPGSKLRKWMGSRVSYSVVLS
FPLLQVLLCLVWLVTAPPFPHLNMQVEDGTIQIECNEGSAIAFYCVLGYLGFLAGISFIVAFLARNLPGSFNEAKHITFSMLVFCSVWVSFIPTYLSTRGKYMVAVEIFAILASSSGILGCIFVPKCYIILLKPHRNNRKYITKK---
>Western_clawed_frog.locus866.1
-------
MWPTEQHDGCRPRSLEYLAYEDPLGGTLASLSVVGSLIPLSILGIFLRNSKTPVVKANNRNLSYLLLFALFLCFLCSLMFIGAPVHLICVLRQITFGVSFVLCVSCVLGKTIMVVIAFNATQPKSSRRMWLNSRIPNTLIIVCMTIQLI
ICSVWVMHSPSFKNDDITSKLGVTILECVEGSPVAFWCMI------------------NLPGSFNEAKMITFSMLIFGAVWISFISTYLSTRGKESVAVEIFAILCSCSGLLALLFFPKCYIIILRPEMNNKEFLTGKRGF
>Caecillan.locus36735.1
CTTCPDDQWSNQKRDACIPKVITFLSFEEPLGIALTFFSMFFFLITNVILGIFIYYRDTPIVRANNRHISYILLISLILCFLCSLLFIGHPEDVTCILRQTTFGITFSIALSSILAKTITVVTAFQATKPGSKLHKWMGSRVSNSIVFS
CSLIQTVLCLVWLCTAPPFAFLNRRSEIGTILIECNEGSVIAFYCVLGYLGFLAGISFIIAFLARNLPDSFNEAKNITFSLLVVCSIWISFIPTYLTSKGKYMVAVEIFAILASSAGLLCCIFIPKCYIILLRPERNNKKDLRKI---
>Caecillan.locus36946.1
CTTCPDDQWSNQKRDACIPKVITFLSFEEPLGIALTFFSMFFFLITNVILGIFIYYRDTPIVRANNRHISYILLISLILCFLCSLLFIGHPEDVTCILRQTTFGITFSIALSSILAKTITVVTAFQATKPGSKLHKWMGSRVSNSIVFS
CSLIQTVLCLVWLCTAPPFAFLNRRSEIGTILIECNEGSVIAFYCVLGYLGFLAGISFIIAFLARNLPDSFNEAKNITFSLLVVCSVWISFIPTYLSSKGKYIVAVEIFAILASSAGLLCCIFIPKCYIILLRPERNNKKDLRKI---
>Caecillan.locus36947.1
CTTCPDDQWSNQKRDACIPKVITFLSFEEPLGIALTFFSMFFFLITNVILGIFIYYRDTPIVRANNRHISYILLISLILCFLCSLLFIGHPEDVTCILRQTTFGITFSIALSSILAKTITVVTAFQATKPGSKLHKWMGSRVSNSIVFS
CSLIQTVLCLFWLCTAPPFAFLNRRSEIGTILIECNEGSVIAFYCVLGYLGFLAGISFIIAFLARNLPDSFNEAKNITFSLLVVCSVWISFIPTYLSSKGKYMVAVEIFAILASSAGLLCCIFIPKCYIILLRPERNNKKDLRKI---
>Caecillan.locus36948.1
CTTCPDDQWSNQKRDACIPKVITFLSFEEPLGIALTFFSMFFFLITNVILGIFIYYRDTPIVRANNRHISYILLISLILCFLCSLLFIGHPEDVTCILRQTTFGITFSIALSSILAKTITVVTAFQATKPGSKLHKWMGSRVSNSIVLS
CSLIQTVLCLAWLFTAPPFAFLNMRSEIGTILIECNEGSVIAFYCVLGYLGFLAGISFIIAFLARNLPDSFNEAKNITFSLLVVCSVWISFIPTYLSSKGKYMVAVEIFAILASSAGLLCCIFIPKCYIILLRPERNNKKDLRKI---
>Caecillan.locus36733.1
CTTCPDDQWSNLKRDACIPKMITFLSFEEPLGIALTSISMFFFLITTAILGIFIYYRDTPIVRANNRHISYILLISLILCFLCSLLFIGHPEDVTCILRQTTFGITFSIALSSILAKTITVVTAFQATKPGSKLHKWMGSRVSNSIVLS
CSLIQTVLCLAWLFIAPPFAFLNRRSEIGTILIECNEGSVIAFYCVLGYLGFLAGISFIIAFLARNLPDSFNEAKNITFSLLVVCSVWISFIPTYLSSKGKYMLAVEIFAILASSAGLLYCIFIPKCYIILLRPERNNKKDLRKI---
>Caecillan.locus36736.1
CTTCPDDQWSNQKRDACIPKMITFLSFEEPLGIALTSISMFFFLITTAILGIFIYYRDTPIVRANNQHISYILLISLILCFLCSLLFIGHPEDVTCILRQSTFGITFSIALSSILAKTITVVTAFQATKPGSKLHKWMGSRVSNSIVLS
CSLIQTVLCLAWLFTAPPFAFLNMRSEIGTILIECNEGSVIAFYCVLGYLGFLAGISFIIAFLARNLPDSFNEAKNITFSLLVVCSVWISFIPTYLSSKGKYMLAVEIFAILASSAGLLCCIFIPKCYIILLRPERNNKKDLRKI---
>Caecillan.locus36732.1
CTTCPDDQWSNQKRDACIPKMITFLSFEEPLGIALTSISMFFFLITTAILGIFIYYRDTPIVRANNQHISYILLISLILCFLCSLLFIGHPEDVTCILRQSTFGITFSIALSSILAKTITVVTAFQATKPGSKLHKWMGSRVSNSVVLS
CSLIQTVLCLAWLFTAPPFAFLNMRSEIRTILIECNEGSVIAFYCVLCYLGFLAGISFIIAFLARNLPDSFNEAKNITFSLLVVCSVWISFIPTYLSSKGKYMVAVEIFAILASSAGLLCCIFIPKCYIILLRPERNNKKDLRKI---
>Caecillan.locus36944.1
CITCPDDQWSNQKRDTCIPKVITFLSFEEPLGIALTSFSMFFFLITAAILGIFIYYRDTPIVRANNQHISYILLISLILCFLCSLLFIGHPEDVTCILRQTTFGITFSIAFSSILAKTITVVTAFQATKPGSKLHKWMGSRVSNSIVFS
CSLIQTVLCLAWLFTAPPFAYVNLRSEIGTILIECNEGSVIAFYCVLGYLGFLAGISFIIAFLARNLPDNFNEAKNITFSLLVVCSVWISFIPTYLSSKGKYMVAVEIFAILASSAGLLCCIFIPKCYIILLRPERNNKKDLRKI---
>Caecillan.locus36734.1
CTTCPDDQWSNQKRDACIPKVITFLSFEEPLGIALTSISMFFFLITTAILGIFIYYRDTPIVRANNRHISYILLISLILCFLCSLLFIGHPEDVTCILRQTTFGITFSIALSSILAKTITVVTAFQATKPGSKLHKWMGSRVSNFIVLS
CSLIQTVLCLIWLCTAPPFAYLNMRSEIGTILIECNEGSVIAFYCVLGYLGFLAGISFIIAFLARNLPDSFNEAKNITFSLLIVCSVWISFIPTYLSSKGKCMVAVEIFAILASTAGLLGCIFIPKCYIILLRPERNNKKDLRKI---
>Caecillan.locus36743.1
CTTCPDDQWSNQKRDACIPKVITFLSFEDPLGIALTSISMFFFLITTAILGIFIYYRDTPIVRANNRHISYILLISLILCFLCSLLFIGHPEDVTCILRQTTFGITFSIALSSILAKTITVVTAFQATKPGSKLHKWMGSRVSNFIVLS
CSLIQTVLCLIWLCTAPPFAYLNMRSEIGTILIECNEGSVIAFYCVLGYLGFLAGISFIIAFLARNLPDSFNEAKNITFSLLIVCSVWISFIPTYLSSKGKYMVAVEIFAILASTAGLLGCIFIPKCYIILLRPERNNKKDLRKI---
>Caecillan.locus36950.1
CTTCPDDQWSNQKRDACIPKVITFLSFEEPLGIALTSISMFFFLITTAILGIFIYYRDTPIVRANNRHISYILLISLILCFLCSLLFIGHPEDVTCILRQTTFGITFSIALSSILAKTITVVTAFQATKPGSKLHKWMGSRVSNFIVLS
CSLIQTVLCLIWLCTAPPFAFLNMRSEIGTILIECNEGSVIAFYCVLGYLGFLAGISFIIAFLARNLPDSFNEAKNITFSLLIVCSVWISFIPTYLSSKGKYMVAVEIFAILASTAGLLGCIFIPKCYIILLRPERNNKKDLRKI---
>Caecillan.locus36949.1
--------------------
MITFLSFEEPLGIALTSISMFFFLITTAILGIFIYYRDTPIVRANNRHISYILLISLTLCFLCSLLFIGHPEDVTCILRQTTFGITFSIALSSILAKTITVVTAFQATKPGSKLRKWMGSRVSNSIVLSCSLIQTVLCLAWLFTTPPFA
YLNMRSELGTILIECNEGSVIAFYCVLGYLGFLAGVSFIIAFLARNLPDSFNEAKNITFSLLVVCSVWISFIPTYLSSKGKYMVAVEIFAILASSAGLLGCIFIPKCYIILLRPERNSKKDLRKM---
>Caecillan.locus36738.1
---------KFENKNIMEKKK----
KKEEPLGIALTSISMFFFLITTAILGIFIYYRDTPIVRANNRHISYILLISLILCFLCSLLFIGHPEDVTCILRQTTFGITFSIALSSILAKTITVVTAFQATKPGSKLRKWMGSKVSNSIVLSCSLIQTVLCLAWLFTTPPFAYLNMR
SELGTILIECNEGSVIAFYCVLGYLGFLSGVSFIIAFLARNLPDSFNEANNITFSLLVFCSVWISFIPTYLSSKGKYMVAVEIFAILASSAGLLGCIFIPKC---------------------
>Caecillan.locus36951.1
CTTCPDDQWSNQKRDACISKMITFLSFEEPLGIILTSISMFFFLITTAILGIFIYYRDTPIVRANNRHISYILLISLILCFLCSLIFIGQPNEVSCILRHATFGITFSIALSSILAKTITVVIAFQATKPGSKLRKWMGSRVSNSIVLS
CSLIQTVLCLAWLFTAPPFAYLNMRSEIGTILIECNEGSVIAFYCVLGYLGFLAGVSFIIAFLARNLPDSFNEAKNITFSLLVVCSVWISFIPTYLSSKGKYMVAVEIFAILASSAGLLVCIFIPKCYIILLRPERNNKKDLRKI---
>Caecillan.locus36744.1
CTTCPDDQWSNQKRDACISKMITFLSFEEPLGIILTSISMFFFLITTAILGIFIYYRDTPIVRANNRHISYILLISLILCFLCSLIFIGQPNEVSCILRHATFGITFSIALSSILAKTITVVIAFQATKPGSKLRKWMGSRVSNSIVLS
CSLIQTVLCLAWLFTAPPFAYLNMRSEIGTILIECNEGSVIAFYCVLGYLGFLAGVSFIIAFLARNLPDSFNEAKNITFSLLVVCSVWISFIPTYLSSKGKYMVAVEIFAILASSAGLLGCIFIPKCYIILLRPERNNKKDLRKI---
>Caecillan.locus36943.1
CTTCPDDQWSNQKRDACIPKMITFLSFEEPLGIALTSISMFFFLITIAILGIFIYYRDTPIVRANNRHISYILLISLTLCFLCSLLFIGHPEDVTCILRQTTFGITFSIALSSILAKTIIVVTAFQATKPGSKLHKWMGSRVSNSIVFS
CSLIQTVLCLVWLCTAPPFAFLNMRSEIGTILIECNEGSVIAFYCVLGYLGFLAGISFIIAFLARNLPDSFNEAKYITFSMLVFCSVWISFIPTYLSSKGKYMVAVEIFAILASSAGLLGCIFFPKCYIILLRPERNNKKDLRKI---
>Caecillan.locus36742.1
CTTCPDDQWSNLKRDACIPKMITFLSFEEPLGIALTSISMFFFLITTAILGIFIYYRDTPIMRANNRHISYILLISLILCFLCSLLFIGHPEDVTCILRQTTFGITFSIALSSILAKTITVVTAFQATKPGSKLRKWMGSRVSNSIVLS
CSLIQTVLCLAWLFTAPPFAYLNMRSELGTILIECNEGSVIAFYCVLGYLGFLAVISFIIAFLARNLPDSFNEAKYITFSMLVFCSVWISFIPTYLSTKGKYMVAVEIFAILASSAGLLGCIFFPKCYIILLRPERNSKKDLRKM---
>Caecillan.locus36740.1
CTTCPDDQWSNLKRDACIPKMITFLSFEEPLGIALTSISMFFFLITTAILGIFIYYRDTPIMRANNRHISYILLISLILCFLCSLLFIGHPEDVTCILRQTTFGITFSIALSSILAKTITVVTAFQATKPGSKLRKWMGSRVSNSIVLS
CSLIHTVLCLAWLFTAPPFAYLNMRSELGTILIECNEGSVIAFYCVLGYLGFLAVISFIIAFLARNLPDSFNEAKYITFSMLVFCSVWISFIPTYLSTKGKYMVAVEIFAILASSAGLLGCIFFPKCYIILLRPERNSKKDLRKM---
>Caecillan.locus40308.1
CISCPDDQWSNQKRDACIPKVINFLSFEEPLGIVLTSISMFFFFITTVILGIFIYYRDTPIVRANNRHISYILLISLILCFLCSLLFIGYPENVTCILQQTTFGITFSIALSSILAKTITVIIAFQATKPGSKLHKWMGSRVSNFIVLS
CSLIQIVLCLAWLSTAPPFAYLNMRSEIGTILIECNEGSVIAFYCVLGYLGFLASISFIIAFLARNLPDSFNEAKYITFSMLIFCSVWITFIPTYLSSKGKYIVAVEIFAILTSSTGLLGCIFFPKCYIILLRPERNNKKDLRKMKGP
>Caecillan.locus40310.1
CITCPDDQWSNQKRDACIPKVINFLSFEEPLGIVLTSISMFFFFITTVILGIFIYYRDTPIVRANNRHISYILLISLILCFLCSLLFIGYPENATCILQQTTFGITFSIALSSILAKTITVIIAFQATKPGSKLHKWMGSTVSNFIVLS
CSLIQIVLCLAWLSIAPPFAYLNMRSEIGTILIECNEGSVIAFYCVLGYLGFLASISFIIAFLARNLPDSFNEAKYITFSMLIFCSVWITFIPTYLSSKGKYIVAVEIFAILTSSTGLLGCIFFPKCYIILLRPERNNKKDLRKMKGP
>Caecillan.locus40309.1
CITCPDDQWSNQKRDACIPKVINFLSFEEPLGIVLTSISMFFFFITTVILGIFIYYRDTPIVRANNRHISYILLISLILCFLCSLLFIGYPENVTCILQQTTFGITFSIALSSILAKTITVIIAFQATKPGSKLHKWMGSRVSNFIVLS
CSLIQIVLCLAWLSTAPPFAYLNMRSEIGTILIECNEGSEIAFYCVLGYLGFLASISFIIAFLARNLPDSFNEAKYITFSMLIFCSVWITFIPTYLSSKGKYIVAVEIFAILTSSTGLLGCIFFPKCYIILLRPERNNKKDLRKMKGP
>Caecillan.locus40311.1
CITCPDDQWSNQKRDACIPKVINFLSFEEPLGIVLTSISMFFFFITTVILGIFIYYRDTPIVRANNRHISYILLISLILCFLCSLLFIGYPENVTCILQQTTFGITFSIALSSILAKTITVIIAFQATKPGSKLHKWMGSRVSNFIVLS
CSLIQIVLCLAWLSTAPPFAYLNMRSEIGTILIECNEGSVIAFYCVLGYLGFLASISFIIAFLARNLPDSFNEAKYITFSMLIFCSVWITFIPTYLSSKGKYIVAVEIFAILTSSTGLLGCIFFPKCYIILLRPERNNKKDLRKMKGP
>Caecillan.locus40307.1
CITCPDDQWSNQKRDACIPKVINFLSFEEPLGIVLTSISMFFFFITTVILGIFIYYRDTPIVRANNRHISYILLISLILCFLCSLLFIGYPENVTCILQQTTFGITFSIALSSILAKTITVITAFQATKPGSKLHKWMGSRVSNFIVLS
CSLIQIVLCLAWLSTAPPFAYLNMRSEIGTILIECNEGSVIAFYCVLGYLGFLASISFIIAFLARNLPDSFNEAKYITFSMLIFCSVWITFIPTYLSSKGKYIVAVEIFAILTSSTGLLGCIFFPKCYIILLRPERNNKKDLRKMKGP
>Caecillan.locus40315.1
CTTCPDDQWSNQKRDACIPKVIIFLSFEETLGIALTSISMFFFLITVVILGIFIYYRDTPIVRANNRHISYILLISLKLCFLCSLLFIGHPEDVTCILRHTTFGITFSIALSSILAKTITVVTAFQATKPGSKLHKWIGSRVSNFIVLS
CSLIQIVLCLAWLSTAPPFAYLNMRSEIGTMLIECNEGSVIAFYCVLGYLGFLAGISFIIAFLARNLPDSFNEAKYITFSMLLFCSVWISFIPTYLSSKGKYMVAVEIFAILASSAGLLGCIFFPKCYIILLRPERNNKKNLRKM---
>Caecillan.locus40470.1
CTTCPDDQWSNKKRDACIPKVITFLSFEEPLGVALTSISVFFFFITTVILGIFIYYRDTPIVRANNQHISYILLISLILCFLCSLLFIGHPEDVTCILRHTTFGITFSIALSSILAKTITVVTAFQATKPGSKLHKWMGSRVSNSIVIS
CSLIQTFLCFAWLFTAPPFAYFNMRSEIGTILIECNEGSVIAIYCVLGYLGFLACISFIIAFLARNLPDSFNEAKYITFSMLVFCSVWISFIPSYLSSKGKYMVAVEIFAILASSTGLLGCIFFPKCYIILLRPERNNKKDLRKL---
>Caecillan.locus40473.1
CTTCPDDQWSNKKRDACIPKVITFLSFEEPLGVALTSISVFFFLITTVILGIFIYYKDTPIVKANNQHTSYILLISLILCFLCSLLFIGHPEDVTCILRHTTFGITFSIALSSILAKTITVVTAFQATKPGSKLHKWMGSRVSNSIVIS
CSLIQTFLCLAWFFTAPPFAYFNMRSEIGTILIECNEGSVIAIYCVLGYLGFLACISFIIAFLARNLPDSFNEAKYITFSMLVFCSVWISFIPTFLSNKGKYMVAVEIFAILASSTGLLGCIFFPKCYIILLRPERNNRKDLRKL---
>Caecillan.locus40476.1
CTTCQDDKWSNKKRDACIPKVITFLSFEEPLGIALTSISMFFFLITAVILGIFIYYRDTPIVRANNQQLSYILLISLILCFLCSLLFIGHPEDVTCILRHTTFGITFSIALSSILAKTITVVTAFQAIKPGSKLHKLMGSRVSNSIVIS
CSLFQIFLCLAWLFTTPPFAYLNMRSEIGTILIECNEGSIIAFYCVLGYLGFLACISFIIAFLARNLPDTFNEAKYITFSMVVFCSVWISFIPTYLSSKGTNMVAVEIFAILASSTGLLGCIFFPKCYIILLRPERNSKKDLRKM---
>Caecillan.locus40406.1
CITCPDDQWSNQKRDACIPKVIHFLSFQEPLGIILTSISMFFFLITSVILGIFIYYRDTPIVRANNRHISYILLISLIFCFLCSLLFIGHPEDATCILRQTTFGITFSIALSSILAKTITVVTAFQATKPGSKLHKWMGSRVSNSIILS
CTLIQTILCLVWLFTAPPFAYLNMRSETGTILIECNEGLVIAFYCVLGYLGFLAAVSFFIAFLARNLPNSLNEAKYITFSMLVFCSVWISFIPTYLSSKGKYMVTVEIFAILASSTGLLGCIFFPKCYIILLRPERNIKKDIRKM---
>Caecillan.locus40477.1
CTTCPDDQWPNQKRDTCIPKIITFLSFEELLGIALTSMSLFFFLITTVILGIFIYYRDTPIVRANNRHISYILLISLKLCFLCSLLFIGHPEDVTCILRQTTFGITFSIALSSVLAKTITVVTAFQAAKPGSKLHKWMGSRVSNSIVLS
GSLIQTFLCLAWLYTAPPFAFLNMRSEIGIIIIECNEGSVIAFYCVLGYLGFLAGISFIIAFLARNLPDSFNEAKNITFSILVFCSVWISFIPTYMSSKGKYMVAVEIFAILTSSTGLLGCIFFPKCYIILLRPERNNKKDLRKI---
>Caecillan.locus40480.1
CTTCTDDQWPNQKRDACIPKMITFLSFEEPLGIALTSISMFFFLITTVILGIFIYYRDTPIVRANNRHISYILLISLMLCFPCSLLFIGHPEDVTCILRQTTFGITFSIALSSVMAKTITVVTAFQAAKPGSKLHKWMGSRVSNSIVLS
SSLIQTFMCLAWLYTAPPFAFLNMRSEIGIILIECNEGSVIAFYCVLGYLGFLAGISFIIGFLARNLPDSFNEAKNITFSMLVFCSVWISFIPTYLSAKGKYMVAVEIFAILTSCTGLLSFIFFPKCYIILLRPERNNKKDLRKI---
>Caecillan.locus40321.1
CTTCSDDKWSNKKRDACIPKVITFLSFEEPLGIALTSISMFFFLITAVILGIFIYYRDTPIVRANNRHISYILLISLKLCFLCSLLFIGHPEDVTCILRQTTFGITFSIALSSILAKTITVVIAFQATKPGSKFHKWMGSRVSNSIVLS
CSLIQTILCLAWLFSAPPFPYLNKRSEIGKILIECNEGSVIAFYCVLSYLGFLAGISFIIAFLARNLPDSFNEAKYITFSMLVFCSVWISFIPTYLSNKGKNMVAVEIFAILASSTGLLGCIFFPKCYIILLRPERNNKKDLRKT---
>Caecillan.locus40384.1
CTTCPDDQWSNKKRDACIPKVITFLSFEEHLGIALTSISMFFFLITAVILGVFIYYRDTPIVRANNRDLSYIILISLMFCFLCSLLFIGHPVDVICILRQTIFGITFSIALSSILAKTITVVTAFQAAKPGSKLRKWMGSRVSICIILS
CSLIQTILCLAWLFTAPPFIFLNLKSEIGIILIECNEGSVIAFYCVLGYLGFLAGISFIIAFLARNLPGSFNEAKNITFSMLVFCSVWISFIPTYLSSKGKYMVAVEIFAILASSSGLLGCIFFPKCYIILLRPERNNKKDLRKI---
>Caecillan.locus40370.1
CTTCPDDQWSNKKRDACIPKVITFLSFEEHLGIALTSISMFFFLITAVILGVFIYYRDTPIVRANNRDLSYIILISLMFCFLCSLLFIGHPVDVICILRQTIFGITFSIALSSILAKTITVVTAFQAAKPGSKLRKWMGSRVSICIILS
CSVIQTILCLAWLFTAPPFIFLNLKSEIGIILIECNEGSVIAFYCVLGYLGFLAGISFIIAFLARNLPGSFNEAKNITFSMLVFCSVWISFIPTYLSSKGKYMVAVEIFAILASSSGLLGCIFFPKCYIILLRPERNNKKDLRKI---
>Caecillan.locus40379.1
```

CTTCPDDQWSNKKRDACIPKVITFLSFEEHLGIALTSISMFFFLITAVILGVFIYYRDTPIVRANNRDLSYIILISLMFCFLSSLLFIGHPVDVICILRQTIFGITFSIALSSILAKTITVVTAFQAAKPGSKLRKWMGSRVSICVILS
CSLIQTILCLAWLFTAPPFIFLNLKSEIGIILIECNEGSVIAFYCVLGYLGFLAGISFIIAFLARNLPGSFNEAKNITFSMLVFCSVWISFIPTYLSSKGKYMVAVEIFAILASSTGLLGCIFFPKCYIILLRPERNNKKDLRKI---
>Caecillan.locus40382.1
CTTCPDDQWSNKKRDACIPKVITFLSFEEHLGIALTSISMLFFLITAVILGIFIYYRDTPIVRANNRDLSYILLISLMFCFLSSLLFIGHPDDVICILRQTLFGITFSIALSSILAKTITVVTAFQAAKPGSKLRKWMGSRVSNCIILS
CSFIQIILCLAWLFTAPPFIFLNLKSEIGIILIECNEGSVIAFYCVLGYLGFLAGISFIIAFLARNLPGSFNEAKNITFSMLVFCSVWISFIPTYLSSKGKYMVAVEIFAILASSTGLLGCIFFPKCYIILLRPERNNKKDLRKI---
>Caecillan.locus40377.1
CTTCPDDQWSNKKRDACIPKVITFLSFEEPLGIALTSISMLFFLITAVILGIFIYYRDTPIVRANNRDLSYILLISLMFCFLSSLLFIGHPDDVICILRQTIFGITFSIALSSILAKTITVVTAFQAAKPGSKLRKWMGSRVSNCIILS
CSFIQIILCLAWLFTTPPFIFLNLKSEIGIILIECNEGSVIAFYCVLSYLGFLAGISFIIAFLARNLPGSFNEAKNITFSMLVFCSVWISFIPTYLSSKGKYMVAVEIFAILASSTGLLGCIFFPKCYIILLRPERNNKKDLRKI---
>Caecillan.locus40369.1
CTTCPDDQWSNKKRDACIPKVITFLSFEEPLGIALTSISMLFFLITAVILGIFIYYRDTPIVRANNRDLSYILLISLMFCFLSSLLFIGHPDDVICILRQTIFGITFSIALSSILAKTITVVTAFQAAKPGSKLRKWMGSRVSNCIILS
CSFIQIILCLAWLFTTPPFIFLNLKSEIGIILIECNEGSVIAFYCVLIYLGFLAGISFIIAFLARNLPGSFNEAKNITFSMLVFCSVWISFIPTYLSSKGKYMVAVEIFAILASSTGLLGCIFFPKCYIILLRPERNNKKDLRKYRCP
>Caecillan.locus40376.1
CTTCPDDQWSNKKRDACIPKVITFLSFEEPLGIALTSISMLFFLITAVILGIFIYYRDTPIVRANNRDLSYILLISLMFCFLSSLLFIGHPDDVICILRQTIFGITFSIALSSILAKTITVVTAFQAAKPGSKLRKWMGSRVSNCIILS
CSFIQIILCLAWLFTTPPFIFLNLKSEIGIILIECNEGSVIAFYCVLGYLGFLAGISFIIAFLARNLPGSFNEAKNITFSMLVFCSVWISFIPTYLSSKGKYMVAVEIFAILASSTGLLGCIFFPKCYIILLRPERNNKKDLRKI---
>Caecillan.locus40404.1
CTTCPDDQWSNQKRDACIPKVITFLSIEEPLGITLTSISMFYFLITAVILGIFIYYRDTPIVRANNRDLSYILLISLMLCFLCSLLFIGQPKETSCILRQTTFGITFSIALSSILAKTITVVTAFQATKPGSKLRKWMGSKVSNSIVLS
CSFIQIVLCLAWLFTAPPFPYLNMKSEIGKILIECNEGSIIAFYCVLGYLGFLAGISFIIAFLARNLPDSFNEAKNITFSMLVFCSVWISFIPTYLSTKGKYMVAVEIFAILASSTGLLGCIFVPKCYIILLRPESNNKKDIRKM---
>Caecillan.locus40402.1
CTTCPDDQWSNKKRDACIPKVITFLSFEEPLGIVLTSISMLLFLITAVILGVFIYYRDTPIVRANNRQLSYILLISLMLCFLCSLLFIGHPMDITCIFRQTTFGIIFSIALSSILAKTITVVTAFQAANPQSKLHKWMGSRVSNSIVLS
CSLIQTVMCLAWLFTAPPFPFLNTRSEIGTILIECNEGSVIVFYCVLGYLGFLAGISFIIAFLARNLPDSFNEAKNITFSMLVFCSVWITFIPTYLSAKGKYMVAVEIFAILASSTGLLGCIFIPKCYIILLRPERN-KKNLRKI---
>Caecillan.locus40403.1
CTTCPDDQWSNLKRDACIPKVITFLSFEEPLGIALTSISMFFFLITAVILGIFIYYRDTPIVRANNRQLSYILLISLMLCFLCSLLFIGHPENVTCFLRQTTFGITFSIALSSILSKTITVITAFQATKPGSKLHKWMGSKVSNSIVLS
CSLIQIVQCFAWLFTAPPFPYLNMRSEIGTILIECNEGSVIAFYCVLGYLGFLAGVSFIIAFLARNLPDSFNEAKYITFSMLVFCSVWISFIPTYLSSKGKYMVAVEIFAILASSTGLLGCIFIPKCYIILLRPERNNKKNLRKI---
>Caecillan.locus40407.1
CTTCPDDQWSNQKRDACIPKVINFLSFKEPLGITLTSIIMLLLLITAVILGIFIYYRDTPIVRANNRQLSYILLVSLILCFLCSLLFIGHPEDVTCILRQTTFGITFSIILSSILAKTFTVVTAFHATKPGSKLHKWMGSKVSNFIVIS
CSLIQIVLCLAWVFTAPPFAYLNMRSEIGTILIECNEGSVIAFYCVLGYLGLLACISFIIAFLARNLPDSFNEAKYITFSMLVFCSVWISFVPTYLSSKGKYTVAVEIFAILISNTGLLGCIFSPKCYIILLRPERNNKKNLRKT---
>Caecillan.locus40416.1
CTTCPDDQWSNQKRDACIPKVITFLSFEEPLGITLTSISMLLLLITAVILGIFIHYRDTPIVRANNRQLSYILLVSLILCFLCSLLFIGHPEDVTCILRQTTFGITFSIVLSSILAKTFTVVTAFHATKPGSKLHKWMGSKVSNFIVIS
CSLIPIVLCLAWVFTAPPFAYLNMRSEIGTILIECNEGSVIAFYCVLGYLGLLAGVSFIIAFLARNLPDSFNEAKYITFSMLVFCSVWISFVPTYLSSKGKYMVAVEIFAILASSTGLLGCIFFPKCYIILLRPERNINKGLRKM---
>Caecillan.locus40408.1
CTTCPDDQWSNQKRDACIPKVITFLSFEEPLGITLTSISMLLLLITAVILGIFIHYRDTPIVRANNRQLSYILLISLILCFLCSLLFIGHPEDVTCILRQTTFGITFSIVLSSILAKTFTVVTAFQATKPGSKLHKWMGSKVSNFIVIS
CSLIQIVLCLAWVFTAPPFAYLNMRSEIGTILIECNEGSVIAFYCVLGYLGLLACISFIIAFLARNLPDSFNEAKYITFSMLVFCSVWISFIPTYLSSKGKYMVAVEIFAILASSTGLLGCIFFPKCYIILLRPERNINKGLRKM---
>Caecillan.locus40481.1
CTTCPEYQWPNQKRDACIPKVINFLSFEEPLGIILTSISMLFFLITAVILGIFIYYQNTPIVRANNRQLTYILLISLMLCFLCSLVFIGHPEDVTCILRQTTFGITFSIALSSILAKTFTVVTAFQATKPGSKLQKWMGARVSNSIVLS
CSLIQIVLCLAWLFTAPPFVHLNMRSEIGTILIECNEGSVIAFYCVLGYLGFLAGISFIIAFLARNLPDSFNEAKYITFSMLVFCSVWISFIPTFLSSKGKYMVAVEIFAILASSTGLLGCIFSPKCYIILLRPERNNKKDLRKM---
>Caecillan.locus40397.1
CTTCPEYQWSNQKRDACIPKVINFLSFEEPLGIILTSISIFFFLITAAILGIFIYYRDTPIVKANNRQLSYILLISLMLCFLCSLVFIGHPEDVTCILRQTTFGITFSIALSSVLAKTFTVVIAFQATKPGSKLQKWMGARVSNYIVLS
CSLIQIVLCLAWLFTAPPFAYLNMRSEIGIILIECNEGSVIAFYCIMGYLGFLAGISLIIAFLARNLPDSFNEAKYITFSMLVFCSTWISFIPTYLSSKGKYIVAVEIFAILASSTGLLGCIFFPKCYIILLRPERNNKKDLRKM---
>Caecillan.locus40325.1
CTTCLDYQWSNQKRDACIPKVINFLSFEEPLGIALTSISMFFFLITAVILGIFIYYRDTPIVRANNRKLSYILLISLMLCFLCSLLFIGHPEDVTCILRQTTFGITFSIALSSVLAKTFTVVAAFQATKPGSKLQKWMGSRFSNFIVLS
CSLIQIVLCLAWLFTAPSFAYLNMRSEIGTILIECNEGSVIAFYCVLCYLGFLAGISFIIAFLARNLPDSFNEAKYITFSMLVFCSVWISFIPTYLSTKGKYMVAVEIFAILASSTGLLGCIFFPKCYIILLRPERNNKKDLRKM---
>Caecillan.locus40323.1
CTTCPDDQWPNQKRDACIPKVITFLSFEEPLGIALTSISMFLFLITSVILGIFIYYRDTPIVRANNRQLSYILLISLMLCFLCSLLFIGHPEDVTCILRQTTFGITFSIALSSILAKTITVVTAFQATKPRSKLHKWMGSRVSNSIVLS
CSLIQIVLCLAWLFTAPPFAYLNMRSEIGTILIECNEGSVIAFYCVLGYLGFLAGISFIIAFLARNLPDSFNEAKYITFSMLVFCSVWISFIPTYLSSKGKNMVAVEIFAILASSTGLLGCIFFPKCYIILLRPERNNKKDLRKM---
>Caecillan.locus40401.1
CTTCPDDQWTNQKRDACIPKVITFLSFEEPLGIALTSISIFFFLITAVILGIFIYYRDTPIVRANNQYLSYILLISLMFCFLWSLLFIGHPEDVTCILRHTIFGITFSIALSSVLAKTITVVTAFQATKPGSKIHKWMGSRVSNSIVLS
CSLIQTVLCVAWLYTAPPFTFLNMRSEIGIILIECNEGSVIAFYCVLGYLGFLASISFIIAFLARNLPDSFNEAKNITFSMLVVCNVWISFIPTYLSTKGKYTVAVEIFAILASSTGLLGCIFFSKCYIILLRPERNNKKDLRKI---
>Caecillan.locus40317.1
CITCPDDQWSNQKRDACIPKVITFLSFEEPLGIALTSISMFFFLITAVILGIFIYYGNTPIVRANNQQLSYILLISLILCFLCSLLFIGHPEDVTCILRQTTFGITFSIALSSVLAKTITVVTAFQATKPRSKLHKWMGSRVSNSIVLS
CSLIQTVLCLVWLCTAPPFAFLNMRSEIGTILIECNEGSVIAFYCVLGYLGFLAGISFIIAFLARNLPDSFNEAKYITFSMLVVCNVWISFIPTYLSTKGKYTVAVEIFAILASSTGLLGCIFIPKCYIILLRPERNNKKDLRKI---
>Caecillan.locus40318.1
CTICPDDQWSNQKRDACIPKVITFLSFEEPLGIALTSISMFFFLITAIILRIFIYYRDTPIVRANNQHISYMLLISLILCFLCSLLFIGHPEDVTCILRQTTFGITFSIALSSILAKTITVVTAFQATKPGSKLHKWMGSKVSNSIVIS
CSLIQTILCLAWLFTAPPFPYLNMRSEIGTILIECNEGSVIAFYCVLGYLGFLAGISFIIAFLARNLPDRFNEAKYITFSMLVFCSVWISFIPTYLSTKGKYTVAVEIFAILASSIGLLGSIFIPKCYIILLRPEKNNRKDLRKI---
>Caecillan.locus40313.1
CTTCLDDQWSNQKRDACIPKVITFLSFEESLGITLTSISMFLFLITVVILVIFIYYRNTPIVRANNRQLSYILLISLILLFLCSLLFIGHPEDVTCILRQTTFGITFSIALSSILAKTITVITAFQATKPGSKLHKWMGSRVSNSIVLF
CSLIQTILCLTWLFTAPPFSYLNMRSEIGTILIECNEGSVIAFYCVLGYLGFLAGISFIIAFLARNLPDRFNEAKYITFSMLVFCSVWISFIPTYLSTKGKYMVAVEIFAILASSTGLLGCIFLPKCYIILLRPERNSKKDLRKL---
>Caecillan.locus40322.1
CITCPDDEWPNQKRDACIPKVVTFLSFEEPLGIALTSISMFFFLITAVILGIFIHHRDTPIVRANNQYLSYILLISLMLCFLCSLIFIGHPQEVNCILRQTTFGITFSITLSSILAKTITVVMAFQATKPGSNLRKWMGSRVSVSIVLC
FSLLQTVLCLAWLFTAPPFPHLNMKSLTGTILIECNEGSIVAFYCVLCYLGFMAGISFSVAFLARNLPDSFNEAKYITFSMLVFCSVWISFIPTYLSTKGKYMVAVEIFAILASSGGILSCIFLPKCYIILLRPERNNRKELRKL---
>Caecillan.locus40314.1
CTTCPDDEWSNQNRDACIPKVITFLSFEEPLGVVLTSISMFFFLITAVILGIFIHYRDTPIVRANNQYLSYILLISLMLCFLCSLIFIGHPQEVNCILRQTTFGITFSITLSSILAKTITVVMAFQATKPGSKLRKWMGSRVSVSIVLC
CSLLQTVLCLAWLFIAPPFPYLNMRSFTGTILIECNEGSIVAFYCVLGYLGFLAGISFIVAFLARNLPDSFNEAKYITFSMLVFCSVWITFIPTYLSTRGKYMVAVEIFAILSSSGGILGCIFLPKCYIILLRPERNNRKELIKM---
>Caecillan.locus40306.1
CTTCPDDQWSNQKRDACIPKVINFLSFEEPLGIALTSISMFLFLVTAAILGIFIYYRDTPIVRANNQHLSYILLISLMLCFLCSLIFIGHPQEINCILRQPTFGITFSITLSSILAKTITVVTAFQAKKPGSKLQKWMGSRVSISIVLS
CSLIQTVLCLAWLFIAPPFPYLNMRSGTGMILIECNEGSIVAFYCILGYLGFLTGISFIVAFLARNLPDSFNETKYITFSMLVFCSVWISFIPTYLSTKGKYMVAVEIFAILASSGGILGCIFLPKSYIILLRPERNSKKDLRKM---
>Caecillan.locus40281.1
CTTCPDDQWSNQKRDACIPKVITFLSFQEPLGIVLSSISMIFFFITAVILEIFIHYQKTPIVRANNQHLSYILLISLMLCFLCSFIFIGHPQEVNCILRQTTFGITFSITLSSILAKTITVVTAFQATKPGSKLRKWMGYRVSLSIVLS
CSLIQIVLCLAWLFTAPPFPYVNMRLEIGTILIECNEGSVIAFYCVLGYLGFLAGISFIIAFLARKLPDSFNEAKYITFSMLVFCSVWISFIPTYLSTKGKYMVAVEIFAILASSVGILVCIFLPKCYIILLRPERNSKKELRKI---
>Caecillan.locus40348.1
CTTCPEDQRSNQKRDACIPKVITFLSFEESLGIVSTSISMLFFLVTAVILGIFIHYRDTPIVRANNQNLSYILLISLMMCFLCSLIFIGRPHEVNCILRQTTFGIAFSIALSSILAKTITVVTAFQATKPKSKLHKWMGSRVSNSVVLS
CSLIQTVLCLVWLTNAPPFAYLNTRSEIGTILIECNEGSVFAFYCVLGYLGLLAGISFIIAFLARNLPNSFNEAKYITFSMLVFCSVWISFIPTYLSSKGKYMVAVEIFAILASSAGLLGCIFFPKCYIILLRPERNNKKDLRKM---
>Caecillan.locus40283.1
CTTCPDDQRSNQIRDACIPKVITFLSFEEPLGIVLTSISMFFFLVTAVILGIFIHYRDTPIVRANNRHLSYIILISLMFCFFCPLIFIGRPHEVNCILRQTTFGIVFSIALSSILAKTITVVTAFQATKPGSKLHKWIGSRVSNSIILS
CSLIQTVLCLAWLFTAPPFPYLNMRSEIGTILIECKEGSVIAFYCVLGYLGFLAGISFIIAFLARNLPDSFNEAKYITFSMLIFCNVWISFIPTYLSSKGKYMVAVEIFAILASSAGLLGCIFSPKCYIILLRPERNNKKDLRKM---
>Caecillan.locus40278.1
CITCPDDQWSNQKRDACIPKVITFLTFEEPLGIVLTFISMFFFLVTAVILGIFIHYRHTPIVRANNRHLSYILLISLMLCFLCSLLFIGYPEDVTCILRHTTFGIAFSIALSSILAKTITVVTAFQATKPGSKLRKWMGSRVSVSVVLC
CSLIQIVFCFLWFFIAPPFPYLNMRSEIRSILIECNEGSIVAFYCVLGYLGFLAIISFIVAFLARNLPDSFNEAKYITFSMLVFCSVWISFIPTYLSTKGKYMVVVEIFAILASSAGILGCIFLPKCYIILLRPQRNSKKELKKM---
>Caecillan.locus40280.1
CITCPDDQWSNQKRDACIPKVITFLTFEEPLGIILTFISMFFFLVTAVILGIFIHYRHTPIVRANNRHLSYILLISLMLCFLCSLLFIGYPEDVTCILRHTTFGIAFSIALSSILAKTITVVTAFQATKPGSKLRKWMGSRVSVSVVLC
CSLIQIVFCFLWFLIAPPFPFLNMRSEIGSILIECNEGSIVAFYCVLGYLGFLAIISFIVAFLARNLPDSFNEAKYITFSMLVFCSVWISFIPTYLSTKGKYMVVVEIFAILASSAGILGCIFLPKCYIILLRPQRNSKKELKKM---
>Caecillan.locus40429.1
CTTCPDDQRSNQKRDACIPKVITFLSFEEPLGIVLTSISMFFFLITAVFLRIFIHYRDTPIVRANNQNLSYILLISLMLCFLCSLLFIGHPEDVTCIFRKTTFGIAFSIALSSILAKTITVVTAFHATKPGSKLRKWMGSRIANSLVLS
CSLIQIILCLAWLLTAPPFAYLNMRSEIGTILIECNEGSVIAFYCILGYLGLLAGISFIIAFLARNLPNSFNEAKYITFSMLVFCSVWIFFIPTYLSSKGKYMVAVEIFAILASSAGLLGCIFFPKCYIILLRPERNNKNELRKM---
>Caecillan.locus40312.1
CKTCPDDQWSNQKRDACIPKVITFLSFEEPLGIVLTSISIFFFLVILVILGIFIHYRDTPIVKANNRYLSCILLISLMFCSLCSLLFIGHPVDVTCILRQTAFGITFSIALSSILAKTITVVTAFQVTKPGSKLRKWMGSRISLSIVLF
CSLIETVLCLAWLFTAPPFAYLNMRSEIGMIIIECNEGSAIAFYCVLGYLGFLAGISFVIAFLARNLPNTFNEAKYITFSMLVFCSVWISFIPTYLSTKGKYMVVVEIFAILASSAGLLGCIFFPKCYIILLRPEKNMKG-LKKM---
>Caecillan.locus40400.1
CTTCPDDQWSNQKKDACIPKVITFLSFEEPLGIVLTFISMFFFLITAAILGIFIYFRDTPIVRANNRHISYILLISLMFCFLCSLLFIGHPEDITCILRQTTFGITFSIALSSILAKTITVITAFQTTKPGSKLRKWMGSRVSNSIVLS
CSLIQTFMCLAWFFSAPPFAYLNMRSEIGTILIECNEGSVIAFYCVLGYLGFLAGISFIIAFLARNLPDSFNEAKYITFSMLIFCSVWISFIPTYLSTKGKYMVAVEIFAILASSTGLLGCIFLPKCYIILLRPERNNKKDLRKT---
>Caecillan.locus40286.1
CTTCPDDQWSNQKRDACIPKVITFLSFEEPLGIVLTSINMLFFLITAVILGIFIYYRDTPIVRANNRHISYILLISLMLCFLCSLLFIGHPEDITCILRQSTFGITFSIALSSILAKTITVVTAFQATKIGSKLQKWMGSRVSNSIVLS
CSLIQTVLCLAWLCTAPPFAYSNMRSEIGTILIECNEGSVIAFYCVLCYLGFLAGVSFIIAFLARNLPDSFNEAKYITFSMLVFCSVWISFIPTYLSAKGKYMVAVEIFAILASSTGLLGCIFFPKCYIILLRPEKNSKKYVTKNVIT
>Caecillan.locus36911.1
CTTCPEDQWPNQKRDACIPKVIIFLSYEEPLGIALTSISLFFLLVTVVILGIFIYYRDTPIVRANNRDLSYIFLISLMLCFLCSLLFIGHPEDVTCILRQTTFGIIFSVTLSSILAKTITVVMAFHATKPGSKLRKWMGSKVSNSIVLS
CSLIQIVLCLAWLFTAPPFAYLNMRSEIGTILIECNEGSVIAFYCNLGYLGFLAGISFIIAFLARNLPDSFNEAKYITFSMLVFCSVWISFIPTYLSTKGKYMVAVEIFAILASSTGLLGCIFFPKCYIILLRPEKNSKKILTKNIMT
>Caecillan.locus36912.1
CTTCPEDQWPNQKRDACIPKVIIFLSYEEPLGIALTSISLFFFLVTVVILGIFIYYRDTPIVRANNRDLSYIFLISLMLCFLCSLLFIGHPLDIICILRQTTFGITFSITLSSVLAKTITVVMAFHATKPKSKLRKWMGSRVSNSIVLS
CSLIQIVLCVAWLFTAPPFAYVNMRSEIGTILIECNEGSVFAFYCVLGYLGFLAGVSFIIAFLARNLPDSFNEAKYITFSMLVFCSVWISFIPTYLSTKGKYMVAVEIFAILASSAGLLGCIFFPKCYIILLRPEKNLKKYLTKNKMT
>Caecillan.locus36907.1
CTICPEDQWPNQKRDACIPKAIIFLSYEEPLGIALTSISLFFFLVTAVILGIFIYYRDTPIVRANNQDLSYIFLISLILCFLCSLVFIGHPEDVTCILRQTTFGISFSVTLSSILAKTITVVMAFHATKPGSKLRKWMGSRVSNSIVLS
CSLIQIVLCFAWLFTAPPFAYLNMRSEIGTILIECNEGSIIVFYCVLGYLGFLAGVSFIIAFLARNLPDSFNEAKYITFSMLVFCSVWISFIPTYLSTKGKYMVAVEIFAILASSAGLLGCIFFPKCYIILLRPERNSKKYLTKT---
>Caecillan.locus36909.1
CTICLEDQWPNQKRDACIPKVIIFLSYEEPLGIALTSISLFFLLVTVVILGIFIYYRDTPIVRANNRDLSYIFLISLMLCFLCSLAFIGHPEDVTCILRQTTFGITFSITLSSILAKTITVVMAFHATKPGSKLRKWMGSRVSNSIVLS
CSLIQTILCLSWLFTAPPFAYLNMRSEIGTILIECNEGSAIAFYCVLGYLGFLAGVSFIIAFLARNLPDSFNEAKYITFSMLVFCSVWISFIPTYLSTKGKYMVAVEIFAILASSAGLLGCIFFPKCYIILLRPERNSKKYLTKTILT
>Caecillan.locus36910.1
CTTCPEDQWPNQKRDACIPKVIIFLSYEEPLGIALTSISLFFLLVTVVILGIFIYYRDTPIVRANNRDLSYIFLISLMLCFLCSLLFIGHPEDITCILRQTTFGITFSITLSSILAKTITVVMAFHATKPGSKLRKWMGSRVSNSIVLS
CSLIQTILCLSWLFTAPPFAYLNMRSEIGTILIECNEGSGIAFYCILGYLGFLAGVSFIIAFLARNLPDSFNEAKYITFSMLVFCSVWISFIPTYLSTKGKYMVAVEIFAILASSAGLLGCIFFPKCYIILLRPERNIKKYLTKT---
>Caecillan.locus36913.1
CTTCREYQWPNQKRDACIPKVIIFLSYEEPLGIALTSISLFFFLVTAIILAIFIYYRDTPIVRANNRDLSYIFLISHMLCFLCSLVFIGYPKDVTCILRQTIFGITFSITLSSVLAKTITVVMAFHATKPGSRLRKWMGSRVSNSIVLS
CSLIQTILCLSWLFTAPPFAYLNMRSEIGTILIECNEGSVIAFYCVLGYLGFLAGVSFIIAFLARNLPDSFNEAKYITFSMLVFCSVWISFIPTYLSTKGKYMVAVEIFAILASSTGLLGCIFFPKCYIILLRPEKNSKKYLSKM---
>Caecillan.locus36915.1
CTICPEDRWPSQKRDACIPKVIIFLTYEEPLGLALTSISLFCFLVTAVILGIFIYYLDTPIVRANNRDLSYIFLISLMLCFLCSLLFIGHPEDITCILRQTTFGITFSITLSSILAKTITVVMAFHATKPGSKLRKWMGSRVSNSIVLS
CSLIQTALCLAWLFTAPPFAYLNMRSEIGTILIECNEGSVIAFYCVLGYLGFLAGVSFIIAFLARNLPDSFNEAKYITFSMLVFCSVWISFIPTYLSTKGKYMVAVEIFAILASSTGLLGCIFFPKCYIILLRPERNSKKYLTKT---
>Caecillan.locus36954.1
CTTCPKDQWPNQKRDACIPKLKTFLSYEEPLGIALTSISLFFFLVTAVILGIFIYYRDTPIVRANNRGLSYMFLISLMLCFLCSLLFMGYPDDVTCILRQTTFGIIFSITLSSILAKTITVVMAFHATKPGSKLRKWMGSRVSNSIVLS
CSIIQTVLCLAWLFTAPPFPYLNMRSEIGTILIECNEGSVIAFYCVLGYLGFLAGVSFIIAFLARNLPDSFNEAKYITFSMLVFCSVWISFIPTYLSTRGKYMVAVEIFAILASSVGLLGCIFFPKCYIILLRPERNSKKYLTKT---
>Caecillan.locus36908.1
CTTCPEDQWPNQKRNACIPKVRTFLSYEEPLGIALTSIILFFFLVTAVILVIFIYYRDTPIVRANNRDLSYIFLISLMLCFFCSLAFIGHPEDVTCILRQTTFGITFSITLSSILAKTITVVMAFHATKPGSKLRKWMGSRVSNSIVLS

CSLIQTVLCLAWLFIAPPFVYLNMRSEIGIILIECNEGSVAAFYCVLGYLGFLAGVSFIIAFLARNLPDSFSEAKYITFSMLVFCSVWISFIPTYLSTKGKYMVAVEIFAILASSAGLLGCIFFPKCYIILLRPERNSKKYLTKT---
>Caecillan.locus36959.1
CTTCPEDQWPNKKRDACIPKVIIFLSYEEPVGIALTLISIFFFLITAAILGIFIYYRDTPIMRANNRDLSYILLIFLMLCFLCSLIFICQPNEASCILQHITFGITFSIALSSILAKTITVVTAFHATKPGSKLRKWMGSRVSNSIVLS
CFLIQTVLCLVWLFTAPPFAFVNMRSEIGTILIECNEGSVIAFYCVLGYLGFLAGVSFIIAFLARNLPDSFNEAKYITFSMLVFCSVWISFIPTYLSSKGKYMVAVEIFAILASSAGLLGCIFFPKCYIILLRPERNSKKYLTKT---
>Caecillan.locus40302.1
CTICPEDQWHNKKRDACIPKVKTFLSYEEPLGIALTSISLFFFLITVAILGIFIYYRDTPIVRANNRDLSYILLIFLMLCFLCSLIFIGQPNEASCILRHTTFGITFSIALSSILAKTITVVTAFQVTKPGSKLRKWLGYKVSNSIVIS
CSLIQTVLCLAWLFTAPPFPYLNMRSEIGTILIECNEGSVIAFYSVLGYLGFLAGVSFIIAFLARNLPDSFNEAKYITFSMLVFCSVWISFIPTYLSTKGKYMVAVEIFAILASSAGLLSCIFFPKCYIILLRPERNSKKYLSKT---
>Caecillan.locus36962.1
CTTCPEDQWTNKKRDACIPKVIIFLSYEEPIGIALILISIFFFLITATILGIFIYYRDTPIVRANNRDLSYILLISLMLCFLCSLMFIGQPNEASCILRHTTFGNTFSIALSSILAKTITVVTAFQATKPGSKLRKWMGSRVSNSIVLS
CSLIQTVLCLAWLFTAPPFPYLNMRSEIGTILIECNEGSVIAFYCVLGYLGFLAGVSFIIAFLARNLPDSFNEAKYITFSMLVFCSVWISFIPTYLSTKGKYMVAVEIFAILASSAGLLGCIFFPKCYIILLRPERNNKKYLSKT---
>Caecillan.locus36960.1
CTTCPEDQWPNKKRDACIPKVKTFLSYEEPLGIVLTLISIFFFLITAAILGIFIYYRDTPIVRANNRDLSYILLISLMLCFLCALIFIGQPKEATCILRQTTFGITFSIALSSILAKTITVVTAFQATKPGSKLRKWMGSRVSNSIVLS
CSLIQTVLCLAWLFIAPPFSYFNMKSEIGTILIECNEGSVIAFYCVLGYLGFLAGVSFIIAFLARNLPDSFNEAKYITFSMLVFCSVWISFIPTYLSTKNKYMVAVEIFAILASTVGLLGCIFIPKCYVILLRPDMNIRKYL-KN---
>Caecillan.locus40438.1
CTTCPEDQWSSSNRDTCIPKIITFLSYEEPLGLALTLISNVFFLITAVILGIFIYYRDTPIVKANNRNLSYILLISLMLSFLCTLVFIGRPKKVTCLLRQTIFGITFSISLSSILAKTITVVTAFQATKPGSKFRKWMGSRVSNSIILF
CFLIQTILCVVWLFTAPPFPYLNMRSEIGIILIECNEGSIVAFYCVLGYLGFLAGVSFIVAFLARNLPDSFNEAKYITFSMLVFCSVWISFIPTYLSSKGKYTVAVEIFAILASSAGLLGCIFMPKCYIILLKPDRNNRKYLTKN---
>Caecillan.locus40440.1
CTTCPEDQWSSSNRDTCIPKIITFLSYEEPLGLALT-------
LITAVILGIFIYYRDTPIVKANNRNLSYILLISLMLSFLCTLVFIGRPKKVTCLLRQSIFGITFSISLSSILAKTITVVTAFQATKPGSKLRKWMDSSVSNSIILFCFLIQTLLCLVWLFIAPPFPYLNMRSEIGLILIECNEGSIVAF
YCVLGYLGFLAGVSFIVAFLAKNLPNSFNEAKYITFSMLVFCSVWISFIPTYLSTKGKYTVEVEIFAILASSAGLLGCIFMPKCYIILLKPDRNNRKYLTKN---
>Caecillan.locus36934.1
CTKCPEDQWSNLNRDACIPKIITFLSYEEPLGIALTLISIGFFLITAVILGIFIYFRDTPIVKANNRNLSYILLISLMLSFLCTLVFIGRPEKVTCLLRQTVFAIIFSISLSSILAKTFTVVTAFQATKPGSKLRKWMGSRVSNSIILF
CFLIQTLLCVIWLFTAPPFPYLNMRSEIGLILIECNEGSIVAFYCVLGYLGFLAGVSFIVAFLARNLPDSFNEAKYITFSMLVFCSVWISFIPTYLSTKGKYTVAVEIFAILASSAGLLGSIFMPKCYIILLKPEKNSRKYLTKN---
>Caecillan.locus36926.1
CRTCPEDQWSNLNRDACIPKIITFLSYEEPLGITLTLISIVFFSNTAVILGIFIYYRDTPIVKANNRNLSYILLISLMLSFLCTLVFIGRPEKVTCLLRQTVFAITFSISLSSILAKTITVVIAFQATKPGSKLRKWMSSKVSNSIILF
CFLIQTLLCVIWLFTAPPFPYLNMRSEIGLILIECNEGSIVAFYCVLGYLGFLAGVSFIVAFLARNLPDSFNEAKYITFSMLVFCSVWVSFIPTYLSTKGKYTVAVEIFAILASSAGLLGCIFMPKCYIILLKPDRNSRKYLTKY---
>Caecillan.locus40344.1
CTTCPEDQWSNSNRDACIPKIVTFLSYGELLGIALTLISIIFFLIAAAILGIFIYYHDTPIVKANNRNLSYILLISLMLSFLCTLVFIGRPDKVTCLLRQTVFAIIFSISLSSILAKTITVVIAFQATKPGSKLRKWMGSRVSNSIIFF
CFLIQTLLCVVWLFTAPPFPYLNMRSEIGLILIECNEGSIVAFYCVLGYLGFLAGVSFNVAFLARNLPDSFNEAKYITFSMLVFCSVWVSFIPTYLSTKGKYMVAVEIFAILASSAGLLGCIFMPKCYIILLKPDRNSRKYLTKN---
>Caecillan.locus36931.1
CTTCPEDQWSNSDRDTCIPKIITFLSYEEPLGIALTLISIVFFLITAVILSFFIYYRDTPIVRANNRNLSYILLMSLMLSFLCTLVFIGRPEKVTCLLRQTVFAIIFSISLSSILAKTITVVIAFQATKPGSKLRKWMGSRISNSIIVF
CFLIQTLLCVVWLSTAPPFPYFNMRSEIGLILIECNEGSIVAFYCVLGYLGFLAGVSFIVAFLARNLPDSFNEAKYITFSMLVFCSVWISFIPTYLSTKGKYMVAVEIFTILASSAGLLGCIFMPKCYIILLKPEKNSRKYLTKN---
>Caecillan.locus36928.1
CTTCPEDQWSNSDRDTCIPKIITFLSYEEPLGIALTLISIVFFLITAVILRIFIYYRDTPIVRANNRNLSYILLMSLMLSFLCTLVFIGRPEKVTCLLRQTVFAIIFSISLSSILAKTITVVIAFQATKPGSKLRKWMGSRISNSIIVF
CFLIQTLLCVVWLSTAPPFPYFNMRSEIGLILIECNEGSIVAFYCVLGYLGFLAGVSFIVAFLARNLPDSFNEAKYITFSMLVFCSVWISFIPTYLSTKGKYMVAVEIFTILASSAGLLGCIFMPKCYIILLKPEKNSRKYLTKN---
>Caecillan.locus40343.1
CITCPEDQWSNLNRDACIPKIITFLSYEEPLGIVLTLINIVFFLITAVILGIFIYYRDTPIVRANNRNLSYILLISLMLSFLCTLVFIGRPDKVTCLLQQTVFAIIFSISLSSILAKTITVVMAFHATKPGSKLRKWMGSKISNSIILF
CFLVQTLLCVSWLFTAPPFPNLNMKSEIGLILIECNEGSIVAFYCVLGYLGFLAGVSFIVAFLARNLPDSFNEAKYITFSMLVFCSVWISFIPTYLSAKGKYMVAVEIFAILTSSAGLLGCIFMPKCYIILLKPDRNSRKYLTKN---
>Caecillan.locus40441.1
CTTCPEDQWSNSNRDACILKIITFLSYEEPLGITLTLINIVFFLITAVILGIFIYYRDTPIVRANNRNLSYILLISLMLSLLCTLVFIGRPDKVTCLLQQTVFAIIFSISLSSILAKTITVVMAFHATKPGSKLRKWMGSRVSNSIILF
CILVQTLLCVSWLFTAPPFLYLNMKSEIGLILIECNEGSIVAFYCVLGYLGFLAGVSLIVAFLARNLPDSFNEAKYITFSMLVFCSVWVSFIPTYLSTKGKYMVAVEIFAIMTSSAGLLGCIFMPKCYIILLKPDRNNKKYLTKN---
>Caecillan.locus36937.1
CTTCPEDQMSIPNRDACIPKMITFLSYGEPLGIALTLISIVFFLITAVILGIFIYYRDTPIVTANNRNLNYILLIALMLSFLCTLVFIGRPEKVTCLLRQTVFAITFSISLSSILAKTITVVTAFQATKPGSKLREWMGSRVSVTIVLC
CSLIQTVLCLAWLSTAPPFPYLNMRSEIGLILIECDEGSIVAFYCVLGYLGFLAGVSLIVAFLAINQPDSFNEAKYITFSMLVFCSVWVYFIPMYLSTKGKHMVAVEIFAILASSAGLLGCIFMPKCYIILLKPDTNIRKYLTIN---
>Caecillan.locus36860.1
CTTCPEDQWSNSNRDACIPKMITFLSNEEPLGIALTLISIVFFLITAVILGIFIYYRDTPIVRANNRNLSYILLISLMLSFLCTLLFIGRPEKVTCLLRQTVFAITFSISLSSILAKTITVVTAFQATKPGSKLRKWMGSRVSVTIVLC
CSFIQTVLCLAWLFTSPPFLYLNMKSEIGLILIECNEGSIVAFYCVLGYLGFLAGVSFIVAFLARNLPDSFNEAKYITFSMLVFCSVWVSFIPTYLSTKGKYMVAVEIFAILASNAGLLGCIFMPKCYITLLKPDRNSRKYLTKN---
>Caecillan.locus36940.1
CTICPEDQWSNPNRNACISKMITFLSYEELLGIALSLISIVFFLITGVILGIFIYYRDTPIVRANNRNLSYILLISLMLSFLCTLVFIGRPEKVTCLLRQTVFAITFSISLSSILAKTITVVMAFHATKPGSKLRKWMGSRVSVNIVLC
CSLIQTVLCLAWLFTAPPFSYLNMRSEIGLILIECNEGSIIAFYCVLSYLGFLACVSFIVAFLARNLPDSFNEAKYITFSMLVFCSVWISFIPTYLSTKGKYMVAVEIFAILASSAGLLGCIFMPKCYIILLKPDRNSRKYLIKT---
>Caecillan.locus36942.1
CTICPEDQWSNPNRNACISKMITFLSYEELLGIALSLISIVFFLITAVILGIFIYYRDTPIVRANNRNLSYILLISLMLSFLCTLVFIGRPEKVTCLLRQTVFAITFSISLSSILAKTITVVMAFHATKPGSKLRKWMGSRVSVTIVLF
CSLIQIVLCLAWLFTAPPFSYLNMKSEIGLILIECNEGSIIAFYCVLSYLGFLACVSFIVAFLARNLPDSFNEAKYITFSMLVFCSVWISFIPTYLSTKGKYMVAVEIFAILASSAGLLGCIFMPKCYIILLKPDRNSRKYLTKT---
>Caecillan.locus36939.1
CTTCPEDQWPNPNRDACIPRMITFLSYGESLGIALTLISIVFFLITAVILGIFIYYRDTPIVRANNRNLSYILLISLMLSFLCTLIFIGRPDKVTCLLQQTVFAITFSISLSSVLAKTITVVMAFHATKPGSKLRKWMGSRVSVTIVLC
CSLIQTVLCLAWLFTTPPFPYHNMRSEIGLILIECNEGSIVAFYCVLGYLGFLAGVSFIVAFLARNLPDSFNEAKYITFSMLVFCSVWISFIPTYLSTKGKYMVAVEIFAILASSAGLLGCIFMPKCYIILLNPDRNSRKYLTKN---
>Caecillan.locus40345.1
CIICSEDQWSNSDRDACIPKIITFLSYEEPLGIALTLISIIFFLIAAAILGIFIYYHDTPIVKANNRNLSYILLISLMLSFLCTLEFIGRPEKMTCLLQQTVFAITFSIALSSVLAKTITVVMAFHATKPGSKLRKWMGSRFSNSIVFS
CSLIQIVLCLSWLFTAPPFLYHNMRSEIGLILIECNEGSVISFYCVLGYLGFLAGVSFIVAFLARNLPDSFNEAKYITFSMLVFCSVWVSFIPTYLSTKGKYMVAVEIFAILASSAGLLGWIFIPKCYIILLKPDRNSRKYLTKN---
>Caecillan.locus40433.1
CMKCPEDQWPNQKRDACIPKVITFLTYEEPLGLTLTFICLFLFFINTVILGIFIHYKDTPIVKANNRDLSYILIISLMLCFLCSLVFIGQPDKLTCVLRQTAFGIMFSIALSTILAKTITVVMAFHATEPGSNLRKWMGTRISVIIVLC
CSLIQTVMCIAWLFIAPPFPYLNMKSEIGTILSECNEGSIVAFYCVLGYLGFLAGISFIVAFLARNLPDRFNEAKYITFSMLVFSNVWIYFIPTYLSTKGKYMVVVEIFAILSSSAGLLGCIFFPKCYVILLRPERNIRKLV------
>Caecillan.locus40274.1

PNQKRDACIPKVITFLTYEDPLGLTLTLICLLLFLINIVILGIFIHYKNTPIVKANNRDLSYILLISLMLCFLCTLVFIGQPNKLTCTLRQTAFAITFSIALSSILAKTIIVVMAFHATKPGSKLRKWMDTRVSVSIVLSCSIIQTAVC
LVWLSTAPPFPYLNMRSEIGTILSECNEGSVVAFYSVLGYLGFLAGISFIIAFLARNLPDRFNEAKYITFSMLVFCNVWISFIPTYLSTKGKYMVAVEIFAILASSAGLLGCIFFPKCYAILLIPERNSRKGWIV----
>Caecillan.locus40276.1
CIKCPEDQWPNQKRDACILKVINFLTYEEPLGLILIFICLFLFLINIVILGIFIHYKDTPIVKANNRDLSYILLISLMLCFLCSLVFIGQPDKLTCILRQTAFGITFSIALSSILAKTITVVMAFHATKPGSKLRKWMGTRVSVSIVLS
CSLIQTVICLAWLLIAPPFPYINMRSEIGTILIECNEGSIVAFYCVLGYLGFLAGISFIVAFLARNLPDRFNEAKYITFSMLVFCNVWISFIPTYLSTKGKYMVVVEIFAILASSAGLLGCIFFPKCYVILLRPERNNRKCL------
>Caecillan.locus40432.1
CTKCPDDQWPNQKRDACIPKLIIFLTYKEPLGLTLIFICLFLFLINIIILGIFIHYKDTPIVKANNRDLSYILLISLMLCFLCSMVFIGQPDKLTCILRQTAFGITFSIALSSILAKTITVVMVFQATKPGSKLQKWMGSRVSVSIVLS
CSLIQTVMCLVWLFVASPFPYINKRSEIGTILIECNEGSIVAFYCVLGYLGILAGISFIVAFLARNLPDRFNEAKYITFSMLVFCNVWISFIPTYLSSKGKYMVAVEIFAIMASSAGLLGCIFFPKCYVILLRPERNSRKCLKE----
>Caecillan.locus40341.1
CIKCPEDQWPNQKRDACIPKVITYLSYEEPLGIILSFICLFFFLINTVILGIFIHYRDTPIVKANNRHLSYILLISLMLCFICSLIFIGHPQEVSCILRQTTFGITFSIALSSILAKTITVVVAFHATKPGSKLQKWMGSKVSISIVTS
CSLIQTAMCLVWLSTAPPFPYLNMRSEIGIIIIECNEGSIVAFYYVLGYLGFLAGISFIVAFLARNLPDRFNEAKYITFSMLVFCNVWISFIPTYLSTKGKYMVAVEIFAILASSAGLLGCIFFPNCYIILLKPERNSRKGGFYN---
>Caecillan.locus40342.1
CVKCQEDQWPNQKKDACLPKLLVFLSFEEPLGIALMLISIILFFINAIILGIFIHNRDTPIVRANNRNLSYLLLISLMLCFLCALVFIGYPDKVTCILRHIAFGIIFSISLSSILAKTLTVVMAFQATKPESKLRKWMGSRVSNSIVIF
CSLVQTLLCVIWLFTASPFPYLNMHSEIGMILIECNEGSIVAFYCVLGYLGFLACISFIIAFLARNLPDSFNEAKYITFSMLVFCSVWISFIPTYLSTKGKYMVVVEIFAILTSSAGLLGCIFFPKCYIILLKPERNKRKYLTKN---
>Caecillan.locus36920.1
CIKCQNDQWPNQERDACIPKVITFLSYEEPLGITLTLLCIFFFLITAVILGIFIHYRDTPIVRANNRDISYIILISLMFCFLCSLVFIGHPEKITCILRQTSFGITFSISLSSVLAKTITVVTAFQATKPGSKLRKWMGSRVSNSIVLF
CSFLQALICLIWLFTAPPFPYLNMRSEAGTILIECNEGSIIAFYCVLGYLGCLAGISFIVAFLARNLPDRFNEAKYITFSMLVFFSVWVSFIPTYLSTKGKYMVAVEIFAILSSSAGLLGCIFFPKCYIILLRPDRNKRKHLTEN---
>Caecillan.locus36921.1
CIKCPADQWPNQERDACIPKVITFLSYEEPLGITLTLLCIFFFLITAVILGIFIHYRDTPIVRANNRDISYIILISLMFCFLCSLVFIGHPEKITCILRQTSFGITFSISLSSVLAKTITVVTAFQATKPGSKLRKWMGSRVSNSIVLS
CSFLQALLCLIWLFTAPPFPYLNMRSEAGTILIECNEGSIIAFYCVLGYLGCLAGISFIVAFLARNLPDRFNEAKYITFSMLVFFSVWVSFIPTYLSTKGKYMVAVEIFAILSSSAGLLGCIFFPKCYIILLRPDRNKKKYLTKN---
>Caecillan.locus36726.1
CIRCPEDQWPNQKRDACIPKVIIFLTYEESLGITLTFIIIFFFLITALILRIFINYRDTPIVKANNRHLSYLLLISLMLSFLCSLIFIGRPEKVTCILRQIVFGITFSISLSSVLAKTITVVTVFQATKPGSKLWKWMGSKVSNSIVLS
CSLLQVIFCLIWLSIAPPFPYLNMRSETGTILIECNEGSMAAFYCVLGYLGFLAGISFIVAFLARNLPDSFNEAKYITFSMLVFCSVWISFIPTYLSIKGKYMVAVEIFAILTSSAGLLGCIFFPKCYIILLRPEKNIRKYLNKKKLD
>Caecillan.locus36727.1
CIRCPEDQWPNQKRDACIPKVIIFLTYKEPLGITLIFIIIFFFLITALILGIFINYRDTPIVKANNRHLSYLLLISLMLSFLCSLIFIGRPKKVTCILRQIVFGITFSISLSSVLAKTITVVTVFQATKPGSKLWKWMGSKVSNSIVLS
CSLLQVIFCLIWLSIAPPFPFLNMRSETGTILIECNEGSMAAFYCVLGYLGFLAGISFIVAFLARNLPDSFNEAKYITFSMLVFCSVWISFIPTYLSTKGKYMVTVEIFAILASSAGLLGCIFFPKCYIILLRPEKNIRKYLNKKKLD
>Caecillan.locus40434.1
CIRCPEDQWPNQMRDACIPKVIIFLSYKEPLGITLTFIIIFFFLITTFILGIFINYRDTPIVKANNRHLSYLLLISLMFSFLCSFIFIGHPKKVTCILQKIVFGITFSISLSSILAKTITVVTVFQATKPGSKLWKWMGSKVSNSIVLF
CSLLQVIFCLTWLLIAPPFPYLNMRSETGTILIECNEGSIVAFYCVLGYLGFLAGISFIVAFLARNLPDSFNEAKYITFSMLVFCSVWISFIPTYLSTKGKNMVAVEIFAILASSAGLLGCIFFPKCYIILLRPEKNIRKHLNRKI--
>Caecillan.locus40435.1
CIRCPEDQWPNQKKDACIPKVIIFLSYKEPFGIALTFIIIFFFLITALILAIFINCRDTPIVKANNRHLSYLLLISLMLSFLCSLIFIGRPEKVTCILQQIAFGIIFSISLSSVLAKTITVVTAFQATKPGSKLETWMGSKVSNSIVLF
CSLLQTLVCLIWLLIAPPFPYLNMRSETGTILIECNEGSRVAFYCVLGYLGFLAAVSFIVAFLARNLPDSFNEAKYITFSMLVFCSVWVSFIPTYLSTKGKYMVVVEIFAILASSAGLLCCIFFPKCYIILLKPEKNSRKYLKKK---
>Caecillan.locus36728.1
CIRCPEDQWPNQKRDSCIPKEIIFLSYEEPLGIALTFIIIFFFLITALILGIFINYQGTPIVKANNRHLSYLLLISLMLSFLCSLLFIGRPEKVTCILRQIVFGITFSISLSSVLAKTITVVIIFQATKPGSKLQKWMGSKVSNSIILF
CSLLQTLFCLIWLLIAPPFPYLNMRSETGTMLIECNEGSLVAFYCVLGYLGFLAGISFIVAFIARNLPDSFNEAKYITFSMLVFCSVWISFIPSYLSTRGKYMVAVEIFAILASSAGLLGCIFFPKCYIILLRPEKNSRKYLNKNR--
>Caecillan.locus40541.1
CITCPEDQWPNQKRDTCNPKEIIFLSYKEPLGLTLTFIIIFFFLITAVILGIFINYRDTPIVRANNRDLSYILLISLMLCFLCSLVFIGRPEKVTCILRQMVFGITFSISLSSVLAKTITVVMAFQATKPGSKLRKWMGSKVSNSIVIS
CSLLQALFCLIWLLIAPPFPYFNTRSETGTILTECNEGSIVAFYCVLGYLGFLAGISFIVALLARNLPDCFNEAKYITFSMLVFCSVWISFIPTYLSARGKYTVAVEIFAILASSAGLLGCIFFPKCYIILLRPERNNRKYLTKTRND
>Caecillan.locus36723.1
CTRCPEDQWSNQKRDACIPKVITYLSYEEPLGITLTLIGILFFLITAFTLGVFLNYRDTPIVKANNRDLSYILLISLMLCFLCSLIFIGRPKKMTCVLRQTVFGIIFSISLSSVLAKTITVVTAFQATKPGNKIRKWMGSKVSYSIIIL
CSLLQALLCLAWLFTAPPFPYLNTRSEAGIILIECDEGSIVAFYSVLGYLGFLAGVSFIVAFLARNLPDSFNEAKYITFSMLVFCSVWVSFIPTYLSTRGKYMVAVEIFAILASSAGLLGCIFFPKCYIILLKPERNHKKYLTKNFND
>Caecillan.locus40513.1
CTRCPEDQWPNKERHACIPKVRNYLSYEEPLGIALSLISGIIFIIIVVILGIFIYHRDTPIVKANNRDLSYIILVSLMLCGFCPFIFIGHPNEMTCVLRQTAFGITFSISLSSILAKTFTVVIAFKATKPGSKLRKWMGSRLSSSIVLS
CSLLQAFFCLIWLLTSPPFPYLNMQSEIGTILIECKEGSVIAFYCVLGYLGILSGTSFIVAFLARNLPDSFNEAKHITFSMLVFCSVWVAFIPTYLSTKGKYMVGVEIFAILASSTGLLGCIFFPKCYIILIKPERNRKMYLTKNYND
>Caecillan.locus40508.1
CTRCPEDQWPNKERYSCIPKVRNYLSYEEPLGIALSLISVIIFIIIVVILGIFIYHRDTPIVKANNRDLSYIILVSLMLSCLCPFIFIGHPNEMICVLRQTAFGITFSISLSSILAKTFTVVIAFKATKPGSKLRKWMGSRISSSIVFS
CSIVQAVFCLIWLLTSPPFPHLNIQSEIGTILIECKEGSVIAFYCVLGYLGILSGTSFIVAFLARNLPDSFNEAKHITFSMLVFCSVWVSFIPTYLSTKGKYMVAVEIFAILASSTGLLGCIFFPKCYIILIRPERNSRMYLTKN---
>Caecillan.locus40509.1
CTRCPEDQWPNKERISCIPKVINYLSYEESLGITLSLISVIIFTNIVVILGIFIYHRDTPIVKANNRDLSYIILVSLMLSCLCPFIFIGHPNKMTCVLRQTAFGITFSISLSSILAKTVTVVMAFKATKPGSKFRKWMGSRISSSIVLS
CSLFQAIFCLVWLLTSPPSPYLNMQSEIGAILIECKEGSVIAFYSVLGYLGILSGTSFIVAFLARNLPDSFNEAKHITFSMLVFCSVWVSFIPTYLSTKGKYMVAVEIFAILASSTGLLSCIFIPKCYIILIRPERNNRMYLTKN---
>Caecillan.locus40511.1
CTRCPEDQWPNKERNSCIPKVRNYLSYEEPLGIALSLIIVIIFIIIAVILGIFIYHRDTPIVKANNRDLSYIILVSLMLCCLCSFIFIGHPNEMTCVLRQTAFGITFSISLSSILAKTVTVVMAFKATKPGSKLRKWMGSRLSSSIVLS
CSLFQAVFCLLWLLTSPPSAYLNMQSEIGTILIECKEGSVIAFYCVLGYLGILSGTSFIVAFLARNLPDSFNEAKHITFSMLLFCSVWVSFIPTYLSTKGKYMVAVEIFAILASSAGLLGCIFFPKCYIILIRPERNSKMYLTKNYND
>Caecillan.locus40506.1

CTRCPEDEWPNKERNSCIPKVRNYLSYEEPLGIALSLIIVIIFIIIVVIFGIFIYHRDTPIVKANNRDLSYIILVSLMFSCLCSLIFIGHPNKMACLLRQTAFGITFSISLSSILAKTVTVVMAFEGNKPGSKLRKWMGYRISNSIVLF
CSLLQAVFCLVWLLTFPPSPHLNMQSEIGTILIECKEGSVIAFYCVLGYLGFLSGISFIVAFLARNLPDSFNEAKHITFSMLVFFSVWVSFIPTYLSTRGKYMVAVEIFAILASSAGLLGCIFIPKCYIILFRPERNSRKHITKK---
>Caecillan.locus40447.1
CIKCPETQWSNQNRDACIPKMIIFLSYEEPLGISLTVISMVFCLLTALILGIFIKYQHTPIVKANNCSLSYALLISLMISFLCSLLFIGHPNKLTCIIRQIAFGINFSIALSSVLAKTITVVIAFQATKPKSKLRNWMGSSLSNSIVLC
CSLLQVLLCLVWMCTFPPFPHHNTESEIGTIVIECNEGSIIAFYCVMGYLGLLAGISFVVAFLARNLPDSFNEAKHITFSMVVFCSVWVSFIPTYLSARGKYTVAVEIFAILTSSAGLLGCIFVPKCYMILLKPEKNNKKYLTRYVKT
>Caecillan.locus40328.1
CIKCPEIQWSNQNRDACIPKMIIFLSYEEPLGISLTVISMFFCLITALILGIFIKYQHTPIVKANNCSLSYVLLISLMISFLYSLLFIGYPNKLTCILRQISFGITFSIALSSVLAKTITVVIAFQATKPKSKLRKWMGSSLSNSIVLC
CFLLQILICFVWMCTAPPFPYQNSELEIGTIVIECNEGSIIAFYCVMGYLGLLAGVSFVVAFLAKNLPDSFNEAKNITFSMLVFCSVWLSFIPTYMSTTGKYTVAVEIFAILASSAGLLGCIFVPKCYMILLRPEKNNKKYLTRYVKT
>Caecillan.locus40327.1
CIKCPENQWSNQNRDACIPKMITFLSYEEPLGISLTVVSMVFCLISAFIFRIFIKYRHTPIVKANNCSLSYVLLISLMFCFSCSLIFIGHPDKLTCILRHIAFGIIFTIALSSVLAKTITVVIAFQATKPKSKLRNWMGPILSNSIVLC
CSLLQVLLCLIWICTSPPFPYHNIKSVIGTIVIECNEGSIIAFYCVMGYLGLLSGISFLFAFFARNLPDTFNEAKHITFSMMVFCSVWVAFIPTYLSTRGKYMVAVEIFAILASSAGLLGCIFTPKCYMILMRPEKNNKKYLTRHIKS
>Caecillan.locus40392.1
CIKCPENQWSNQKRDACIPKMIIFLSYEEPLGIFLTVVSMVFCLISALILGIFIKYQHTPIVKANNRSLSYIFLISLMLCFLCSLIFIGHPDKLTCILRHIAFGITFSIALSSVLAKTITVVIAFQATKPKSKLRNWMGSSLSNSIVLC
CSLLQVLLCLVWICTAPPFPYHNTESEIGTIVIECNEGSIIAFYCVMGYLGLLAGISFVFAFLARNLPDTFNEAKHITFSMVVFCSVWVAFIPTYLSTRGKYMVAVEIFAILASCAGLLGCIFIPKCYMILLRPEKNNKKYLTRCVKT
>Caecillan.locus40490.1
CIKCPEDQWSNQKRDACIPKMITFLSYKEPLGISLIVVSMVFCFISAFILGIFIKYRHTPIVKANNRSLSYVLLISLMLCFLCSLIFIGRPNKLTCILRQVTFGITFSISLSSVLAKTITVVIAFQATKPKSKLRNWMGSSLSNSIVLS
CSLLQTLLCVVWMCTAPPFPYHNTESEIGTIAIECNEGSTIAFYCVMGYLGLLAGISFVVAFLARNLPDTFNETKHITFSMMIFCSVWVTFIPTYLSTKGKFMVAVEIFAILASSAGLLGCIFISKCYMILLRPEKNNKKHLTRHIKT
>Caecillan.locus40303.1
CIKCSENQWSNKKRDACIPKMITFLSYEEPLGISLTFTSMVFCFISAFILGIFIKYRHTPIVKANNRSLSYVLLISLMLCFLSSLIFIGRPDKLTCILRQIAFGITFSISLSSVLAKTITVVIAFQATKPQSKLRNWMGSSLSNSIVFS
CSLLQTLLCLVWMCTSPPFPYYNTESEIETIIIECNEGSTIAFYCVMGYLGLLAGISFVVAFLARNLPDTFNEAKYITFSMVVFCSVWVTFIPTYLSTRGKYLVAVEIFAILASSAGLLGCIFISKCYMILLRPEKNNKKNLTRHVKT
>Caecillan.locus40446.1
CIKCPENQWSNHKRDACIPKMIIFLSYEEPLGMFLTVVGMVFCFISAIILGIFIKYRQTPIVKANNRSLSYILLISLMLCFLCSLIFIGRPDKLTCILRQVAFGITFSISLSSVLAKTITVVIAFQATKPQSKLRNWIGSNLSNSIVLG
CSLLQMLMCLVWMCTSPPFPYHNTESEIGKIVIECNEGSIIAFYCVMGYLGLLSGISLVVAFLARNLPDTFNEAKHITFSMMVFCSVWVAFIPTYLSTRGKYMVAVEIFAILASSAGLLGCIFISKCYMVILRPDKNTRNYLTRQVKT
>Caecillan.locus40360.1
CIKCPENQWSNKKRDACIPKMITFLSYEEPLGISLTVTSMVFCFISALILGIFIKFRNTPMVKANNRNLSYILLISLILCFLCSLLFIGRPVKLTCILRQIAFGITYTISLSSVLAKTITVVIAFQATKPKSKLRNWMGSSLTNSIVLS
CSLLQTLLCLIWMCTAPPFPYHNTELEIGTIVIECNEGSIIAFYCVMGYLGLLAGISFVVAFLARNLPDTFNEAKHITFSMVVFCSVWVAFIPTYLSTRGKYMVAVEIFAILASCTGLLGCIFIPKCYIILLSPEKNHKRYLTRHVKT
>Caecillan.locus40467.1
CKKCPENQWSNWKRDACIPKVIIFLSYKEPLGISLTVVSMFLCFINSLIMGIFIKYRHTPIVKANNRSLSYILLISLMLCFLCSLIFISQPDKLTCILRQIAFGITFSISLSSVLAKTITVVIAFQATKPKSKLRSWMNSSVSNSIVLC
CSLLQALLCLVWLCTAPPFPYHNTESEIGTIVIECNEGSIIGFYCVLGYLGLLAGISFVVAFLARNLPDTFNEAKHITFSMVVFCSVWVAFIPTYLSTRGKYMVAVEIFAILASSAGLLGCIFIPKCFVVLLKPEKNSKKYLTRYVKT
>Caecillan.locus40331.1
CKKCPENQWSNRKRDACIPKVIIFLSYKEPLGISLTLISIFLCFINILILGIFIKYQHTPIVKANNRSLSYVLLISLMLCFLCSLIFIGQPDKLTCILRQIAFGITFSISLSSVLAKTITVVIAFQATKPKSKLRSWMSSSVSNSIVLC
CSLFQALLCLVWLCTAPPFPYQNNESEIGTIVIECNEGSIIAFYCVLGYLGLLAGISFIVAFLARNLPDTFNEAKHITFSMVVFCSVWVSFIPTYLSTRGKYMVAVEIFAILASSAGLLGCIFIPKCFVVLLKPEKNSKKYLTRYVKT
>Caecillan.locus40394.1
CIKCPENQWSNQKKDACIPRVITFLSYKEPLGIFLTFISMLFCYINIFILGIFIKYRHTPIVKANNCSLSYVLLVSLMLCFLCSLIFIGQPDKLTCILRQVAFGITFSISLSSVLAKTIIVVLAFQATKPQSKLRNWMSSSVSNSIVLC
CSLLQVLLCLVWLCTAPPFPYHNTESEIGTIVIECNEGSIIAFYCVLGYLGLLAAISFVVAFLTRNLPDTFNEAKHITFSMVVFCSVWVSFIPTYLSTRGKYMVAVEIFAILASSAGLLGCIFIPKCFVVLLKPEKNSKKYLTRYVKT
>Caecillan.locus40296.1
CTKCPEDQWSNQKRDACIPKVITFLSYEEPLGISLTLISTFFCFLSVLILGIFIKYRQTPIVKANNRSLSYILLISLMLCFLCSLIFIGHPGNLNCILRQITFGIIFSISLSSVLAKTITVVIAFQATKPKSKLRNWISSRVSNSIVLC
CFLLQVLLCIIWLCTSPPFPYYNTESEIGTILIECNEGSMIAYYCVMGYLGFLASISIVVAFLARNLPDTFNEAKHITFSMVVFCSVWVSFIPTYLSTKGKYMVAVEIFAILASCAGLLGCIFIPKCYIVLLRPENNNKKYLTRYIKT
>Caecillan.locus40393.1
CEKCRKDQWSNQKRDACIPRKITFLSYEEPLGISLTLISIFICFICALILGIFIKYRQTPIVKANNRSLSYVLLISLMLCFLCSLIFIGHPDKLTCILRQITFGIMFSISLSSVLAKTITVVIAFQATKPKSKLRNWMSPNVSNSIVIY
CSLLQAFLCLVWMCTAPPFPYQNTESEIGTIVIECNEGSMIAFYCVMGYLGFLASISFVVAFLARNLPDTFNEAKHITFSLVVFCSVWVAFIPTYLSTRGKYMVVVEIFAILASSAGLLSCIFIPKCYIILLRPDMNNKKYLTKYVKT
>Caecillan.locus40489.1
CIKCPEDQWSTQKRDACIPKVITFLSYEDPLGISLTLVSMFFCTVSALILGIFIKYRHTPIVKANNHSLSYSLLISLMLCFLCSLLFIGRPDKLTCILRQIAFGITFSISLSSVLAKTITVIIAFQATKPKSKLRNWMSSSFSNSIVLC
CSLLQALLCLVWICTAPPFPYHNTESEIGAIVIECNEGSIIAFYCVLGYLGFLACLSFTVAFLSRNLPDSFNEAKYITFSMVAFCSVWMAFIPTYLSTRGKSMIAVEIFAILASSAGLLGCIFIPKCYMILLRPEMNNKKYLTRHVKT
>Caecillan.locus40487.1
CIKCPEDNWSNQKRDACIPKVITFLSYEEPLGISLTLVSIFFCFIGAFILGIFIKHRHTPIVKANNRILSYILLISLMLCFLCSLLFIGQPVKFTCILRHIAFGITFSISLSSIFAKTITVVIAFQATKPKSKLRNWMGSSLSNSIVLS
CSLLQTLLCLVWMCTAPPFPYHNTESEIGAIVIECNEGSIIALYCVLGYLGLLACLSFTVAFLSRNLPDSFNEAKYITFSMVVFCSVWMAFIPTYLSTRGKYMVAVEIFAILASSAGLLGCIFIPKCYMILLRPEKNNKKHLTRHVKT
>Caecillan.locus40491.1
CIKCPEDNWSNQNRDACILKAKTFLSYEDPLGISLTLISTFFCFISALILGIFIKYRHTPIVKANNRSLSYILLISLMLCFLCSLIFIGRPDKLTCILRQIAFGITFSISLSSILAKTITVVIAFQATKPKSKLRNWMGSSLSNSIVLC
CSLLQVLLCLVWLCTSPPFPYHNTESEIGTIVIECSEGSIIAFYCALGYLGFLTGISFTVAFLSRNLPDSFNEAKYITFSMVVFCSVWISFIPTYLSTKGKYMVAVEIFAILASSAGLLGCIFIPKCYIILLRPEKNSKRNLTRHLKT
>Caecillan.locus40297.1
CIKCPEENWSNQKRDACIPKVITFLSYEEPLGISLMVISVVFCFISALILGIFIKYRHTPIVKANNHSLSFILLISLMFCFLCSLIFIGRPDKLTCILRQIAFSITFSLSLSSILAKTITVVMAFQATKPTSKLRNWMGSSLSNSIVLC
CSLLQVLLCLVWLCTAPPFPYYNTESEIGTIVIECNEGSIIAFYCVLGYLGFLVGISFTVAFFSRNLPDSFNEAKYITFSMVVFCSVWVSFIPTYLSTKGKYTVALEIFAILASSAGLLGCIFIPKCYMILLRPEKNNKSYLTRHVKT
>Caecillan.locus40332.1
CTKCPDDQWSNQKRDACIPKAIIFLTYEDPLGISLTLVSMIFCLVTAIILGIFIKYHHTPIVKANNRSLSYVLLISLMFCFLCSLIFIGHPDRLTCILRQIVFGTMFSISLSSILAKTITVVIAFQATNPKSKLRNWMSSSVSNSIVLC
CSLLQALLCLVWLCTAPPFPYQNTKSEIGIIVIECNEGSIIAFYCLLGYLGLLSGISFTLAFLSRNLPDSFNEAKYITFSMIVFCSVWLSFIPTYLSTRGKYMVAVEIFAILASSAGLLGCIFIPKCYIILLKPEKNNRKYLTRYIKT
>Caecillan.locus40581.1
CISCPEDQWPNENRDACVPKIISFLSHEEPWGIILTLISIFFFFITAVTLRIFIYYQATPIVKSNNKNLSYILLVSLMLCFLCSLIFIGRPDKVTCVLRQAAFGITFSISLSSVLAKTITVVMAFQSTKPGSKLRKWMGSRVSYSIVLS
CSLLQVFLCLIWLGAAPPFPYLNMEGEDGTIQIECNEGSAIAFYCVLGYLGFLAGISFIVAFLARNLPGSFNEAKHITFSMLVFCSVWVSFIPTYLSTRGKYMVAVEIFAILTSGSGILGCIFIPKCYIILLRPERNNRKYITKK---
>Caecillan.locus40571.1
CIRCPEDQWPNENRDACVPKIISFLSHEEPWGIILTLISIFFFFITAVILGIFIYYQATPIVKSNNKNLSYILLVSLMLCFLCSLIFIGRPDKVTCVLRQAAFGITFSISLSSVLAKTITVVMAFQSTKPGSKLQKWMGSRVSYSIVLS
CSLLQVFLCLIWLGAAPPFPHLNMEGEDGTIQIECNEGSAIAFYCVLGYLGFLAGISLIVAFLARNLPGSFNEAKHITFSMLVFCSVWVSFIPTYLSTRGKYMVAVEIFAILTSGSGILGCIFIPKCYIILLRPERNNRKYITKK---
>Caecillan.locus40572.1
CIRCPEDQWPNEKRDACVPKIISFLSHEEPWGIILTLISIFFFFITAVILGIFIYYQATPIVKSNNKNLSYIILVSLMLCFLCSLIFIGHPDKVTCVLRQAAFGITFSISLSSVLAKTITVVMAFQSTKPGRKLRKWMGSRVSYSIVLS
CSLLQVFLCLIWLGAAPPFPHLNMEGEDGTIQIECNEGSAIAFYCVLGYLGFLAGISLIVAFLARNLPGSFNEAKHITFSMLVFCSVWVSFIPTYLSTRGKYMVAVEIFAILASGSGILGCIFIPKCYIILLRPERNNRKYIAKK---
>Caecillan.locus40584.1
CIRCPEDQWPNEKRDACVPKIISFLSHEEPWGIILTLISIFFFFITAVILGIFIYYQATPIVKSNNKNLSYILLVSLMLCFLCSLIFIGHPDKVTCILRQAAFGITFSISLSSVLAKTITVVMAFQSTKPGSKLRKWMGSRVSYSIVLS
CSLLQVFLCLIWLGIAPPFPHLNMEGEDGTIQIECNEGSVIAFYCVLVYLGFLAGISFIVAFLARNLPGSFNEAKHITFSMLVFCSVWVSFIPTYLSTRGKYMVAVEIFAILASGFGILGCIFIPKCYIILLRPERNNRKYIAKK---
>Caecillan.locus40580.1
CISCPEDQWPNENRDACVPKIISFLSHEEPLGIILTLISIFFFFITAVILRIFIYYQATPIVKSNNKNLSYILLISLMLCFLCSLIFIGHPDKVTCVLRQAAFGITFSISLSSVLAKTITVVMAFQSTKPGSKLCKWMGSRVSYSIVLS
CSLLQVFLCLIWLGAAPPFPHLNMEGEDGTIEIECNEGSAIAFYCVLGYLGFLAGISFIVAFLARNLPGSFNEAKHITFSMLVFCSVWVSFIPTYLSTRGKYMVAVEIFAILASSSGIMGCIFIPKCYIILLRPERNNRKYIKKK---
>Caecillan.locus40579.1
CIRCPEDQWPNENRDACFPKIISFLSHEEPLGIILTLISIFFFFITAVILGIFIYYQATPIVKSNNRNLSYILLVSLMLCFLCSLIFIGHPDKVTCVLRQAAFGITFSISLSSVLAKTITVVMAFHSTKPGSKLRKWMGSRVSYSIVLS
CSLFQVFLCLIWLGAAPPFPHLNMEGEDGTIQIECNEGSAIAFYCVLGYLGFLAGISFIVAFLARNLPGSFNEAKHITFSMLVFCSVWVSFIPTYLSTRGKYMVAVEIFAILASGSGILGCIFIPKCYIILLRPERNNRKYIAKK---
>Caecillan.locus40585.1
CIRCPEDQWPNEKRDACVPKIISFLSHEEPLGIILTLISIFFFFITAVILGIFIYYQATPIVKSNNRNLSYILLIFLMLCFLCSLIFIGHPDKVTCVLRQAAFGITFSISLSSVLAKTITVVMAFQSTKPGSKLRKWMGSRVSYSIVLS
CSLLQVFLCLIWLGTAPPFPHLNMEGEDGTIQIECNEGSAIAFYCVLGYLGFLAGISFIVAFLARNLPGSFNEAKHITFSMLVFCSVWVSFIPTYLSTRGKYMVAVEIFAILTSGSGILGCIFIPKCYIILLRPERNNRKYIAKK---
>Caecillan.locus40578.1
CIRCPEDQWPNEKRDACVPKPISFLSHEETLGIILTLISIFFFFITAVILGIFIYYQATPIVKSNNKNLSYILLVSLMLCFLCSLIFIGHPDKVTCVLRQAAFGITFSISLSSVLAKTITVVMAFHSTKPGSKLRKWMGSRVSYSIVLS
CSLLQVFLCLIWLVAAPPFPHLNMEGEDGTIQIECNEGSAIAFYCVLGYLGFLAGISFIVAFLARNLPGSFNEAKHITFSMLVFCSVWVSFIPTYLSTRGKYMVAVEIFAILASGFGILGCIFIPKCYIILLRPERNNRKYIKKK---
>Caecillan.locus40566.1
CIRCPEDQWPNEKRDACVPKPIRFLSHEETLGIILTLISIFFFFITAVILGIFIYYQATPIVKSNNRNLSYIILVSLMLCFLCSLIFIGHPDKVTCVLRQAAFGITFSISLSSVLAKTITVVMAFQSTKPGSKLRKWMGSRVSYSIVLS
CSLLQVFLCLVWLGAAPPFPHFNMEGEDGTIQIECNEGSAIAFYCVLGYLGFLAGISFIVAFLARNLPGSFNEAKHITFSMLVFCSVWVSFIPTYLSTRGKYMVAVEIFAILASGFGILGCIFIPKCYIILLKPERNNRTYITKK---
>Caecillan.locus40582.1
CIRCPEDQWPNEKRDACVPKPISFLSHEETLGIILTLISIFFFFITAVILRIFIYYQATPIVKSNNRNLSYILLFSLMLCFLCSLIFIGRPDKVACILRQAAFGITFSISLSSVLAKTITVVMAFQSTKPGSKLRKWMGSRVSYSIVLS
CSLLQVFLCLIWLGAAPPFPHLNMEGEDGTIQIECNEGSAIAFYCVLGYLGFLAGISFIVAFLARNLPGSFNEAKHITFSMLVFCSVWVSFIPTYLSTRGKYMVAVEIFAILASGSGILGCIFIPKCYIILLRPERNNRKYIAKK---
>Caecillan.locus40568.1
CIRCPEDQWPNEKRDACVPKIISFLSHEEPLGIILTLISIFFFFITAVILRIFIYYQTTPIVKSNNRNLSYILLVSLMLCFLCSLIFIGHPDKVTCVLRQASFGITFSISLSSVLAKTITVVMAFQSTKPGSKLRKWMGSRVSYSIVLS
CSLLQIFLCLIWLGAAPPFPHLNMEGEDGTIQIECNEGSAIAFYCVLGYLGFLAGISFIVAFLARNLPGSFNEAKHITFSMLVFCSVWVSFIPTYLSTRGKYMVAVEIFAILASGFGILGCIFIPKCYIILLRPERNNRKYIAKK---
>Caecillan.locus40573.1
CIRCPEDQWPNEKRDACVPKIISFLSHEETLGIILTLISIFIFFITAIILGIFIYYQATPIVKSNNRNLSYILLVSLMLCFLCSLIFIGHPDKVTCVLRQAAFGITFSISLSSVLAKTITVVMAFQSTKPGSKLRKWMGSRISCSIVLS
CSLLQVFLCLIWLGAAPPFPHLNMEGEDGTIQIECNEGSAIAFYCVLGYLGFLAGISFIVAFLARNLPGSFNEAKHITFSMLVFCSVWVSFIPTYLSTRGKYMVAVEIFAILASGFGILGCIFIPKCYIILLRPERNNRKYIAKK---
>Caecillan.locus40574.1
CIRCPEDQWPNEKRDACVPKIINFLSHEETLGIILTLISIFFFFITAVILGIFIYYQATPIVKSNNRNLSYILLVSLMLCFLCSLIFIGNPDKMTCILRQAAFGITFSISLSSVLAKTITVVMAFQSTKPGSKLRKWMGSRVSYSIVLS
CSLLQVFLCLIWLGAAPPFPHLNMEGEDGTIQIECKEGSAIAFYCVLGYLGFLAGISFIVAFLARNLPGSFNEAKHITFSMLVFCSVWVSFIPTYLSTRGKYMVAVEIFAILASGSGIMGCIFIPKCYIILLRPERNNRKYIAKK---
>Caecillan.locus40570.1
CIRCPEDQWPNEKRDACVPKIISFLSHEETLGIILTLISIFFFFITAVILGIFIYYQATPIVKSNNRNLSYILLVSLMLCFLCSLIFIGHPDKVTCVLRQATFGITFSISLSSVLAKTITVVMAFQSTKPGSKLRKWMGSRVSYSIVLS
CSLLQVFLCLIWLGAAPPFPHLNMEGEDGRIQIECNEGSAIAFYCVLGYLGFLAGISFIVAFLARNLPGSFNEAKHITFSMMVFYSVWVSFIPTYLSTRGKYMVAVEIFAILASGSGILGCIFIPKCYIILLRPERNNRKYITKK---
>Caecillan.locus40565.1
CIRCPEDQWPNEKRDACIPK-
ISFLSHEETLGIILTLISIFFFFITAVILGIFIYYQATPIVKSNNRNLSYILLVSLMLCFLCSLIFIGHPDKVTCVLRQAAFGITFSISLSSVLAKTITVVMAFQSTKPGSKLRKWMGSRVSYSIVLSCSLLQIFLCLIWLGAAPPFPH
LNMEGEDGTIQIECNEGSAIAFYCVLGYLGFLAGISFIVAFLARNLPGSFNEAKHITFSMLVFCSVWVSFIPTYLSTRGKYMVAVEIFAILASGSGILGCIFIPKCYIILLRSDRNNRKYITKK---
>Caecillan.locus40426.1
CIRCPEDQWPNEKRYVCVPKIISFLSHEETLGIILTLISILFFFITAVIMGIFIYYRDTPIVKSNNRNLSYILLVSLMLCFLCSLIFIGRPDKVTCILRQTAFGITFSISLSSVLAKTITVVMAFQSTKPGSKFRKWMGSRVSYSIVLS
CSLLQVFLCLVWLGAVPPFPHVNMQAEDGTIQIECNEGSTIAFYCVLGYLGFLAGISFIVAFLARNLPGSFNEAKHITFSMLVFCSVWVSFIPTYLSTRGKYMVAVEIFAILASSSGILGCIFIPKCYIILLRPDRNNRKYITKK---
>Caecillan.locus40424.1
CIRCPEDQWPNEKRYVCVPKIISFLSHEETLGIILTLISILFFFITAVIMGIFIYYRDTPIVKSNNRNLSYILLVSLMLCFLCSLIFIGRPDKVTCILRQTAFGITFSISLSSVLAKTITVVMAFQSTKPGSKFRKWMGSRVSYSIVLS
CSLLQVFLCLVWLGAAPPFPHVNMQAEDGTIQIECNEGSTIAFYCVLGYLGFLAGISFIVAFLARNLPGSFNEAKHITFSMLVFCSVWVSFIPTYLSTRGKYMVAVEIFAILASSSGILGCIFIPKCYIILLRPDRNNRKYITKKNKF
>Caecillan.locus40395.1
CIRCPEDQWPNEKRDACVPKIISFLSHEESLGIILTLISILFFFITAVILGTFIYYQDTPIVKSNNRNLSYILLVSLMLCFLCSLIFIGHPDKVTCILRQTAFGITFSISLSSVLAKTITVVMAFQSTKPGSKFRKWMGSRVSYSIVLS
CSFLQILLCLVWLGAAPPFLHVNMQAEDGTIQIECNEGSIIALYCVLGYLGFLAGISFIVAFLARNLPGSFNEAKHITFSMLVFCSAWVAFIPTYLSTKGKYMVAVEVFAILASGSGILGCIFIPKCYIILLRPDRNNKKYITKK---
>Caecillan.locus40586.1
CIRCPEDQWPNDKRDVCVPKLISFLSFEEPLGIILIHISIFFFIITAIILGIFINYRDTPIVKSNNRNLSYILLVSLMLCFLCSLIFIGRPNEVTCVLRQTAFGINFSISLSSILAKTITVVMAFQSTKPGSKLRKWMGSRVSYSIVLS
CSLFQVLICLVWLAAVPPFPHLNMQGEDGIIQIECNEGSAIAFYYVLGYLGFLAGISFIVAFLARNLPDSFNEAKHITFSMLVFCSVWVSFIPTYLSTRGKYMVAVEIFAILASGSGILGCIFIPKCYIILLWPDRNNRKYITKK---
>Caecillan.locus40355.1
CIRCPEDQWPNEKKDACVPKMISFLSHEEPLGIILMLISIFFLFITVIILGIFINYRDTPIVKSNNTNLSYILLVSHMFCFLCSLIFIGRPDEVTCVLRQTAFGITFSISLASVLAKTITVVMAFQSTKPGSNLRKWMGSRVSYSIVLS
CSLFQVFLCLVWLGAAPPFPHINMQAEDKTIQIECNEGSAIAFYCVLGYLGFLAGISFIVAFLARNLPDSFNEAKHITFSMLVFCSVWVSFIPTYLSTRGKYMVAVEIFAILASSSGILGCIFFPKCYIILLKPDRNNRKYITKK---
>Caecillan.locus40354.1

CIRCSEDQWPNDKGNVCIPKIVSFLSHEEPLGTILMLISIFFLFITVIILRIFINYRDTPIVKSNNTNLSYILLVSLMLCFLCSLIFIGRPNEVTCVFRQTAFGITFSISLACVLAKTITVVMAFQSTKPGSNLRKWMGSRVSYSIVLS
CSLFQVLLCLVWLGAAPPFPHVNMQAEDKTIQIECNEGSAIAFYCVLGYLGFLAGISFIVAFLARNLPDSFNEAKHITFSMLVFCSVWVSFIPTYLSTRGKYMVAVEIFAILASSTGILGCIFLPKCYIILLKPDRNNRKYITKK---
>Caecillan.locus40349.1
CIKCPEDQWPNEKGDVCIPKIISFLSHEEPLGFVLILISIFFLFITVIILGIFINYRDTPIVKSNNTNLSYILLVSLMLCFLSSLIFIGHPDKVMCILRQTAFGITFSISLSSVLAKTITVVMAFQTTKPGSKLRKWMGSRVTYSIVLS
CSFLQVLLCLVWLGASPPFPYVNKEGEVGTIQIECNEGSAIAFYCVLGYLGCLSAISFIVAFLARNLPDSFNEAKHITFSMLMFCSVWVSFIPTYLSTRGKYMVAVEIFAILASSSGILGCIFFPKCYIIVLKPDRNNRKYITKNKLT
>Caecillan.locus40294.1
CIRCPEDQWPNEKGNVCIPKIISFLSHQEPLGIVLILISIFFLIITAIILGIFINYRDTPIVKSNNSNLSYILLVSLLLCFLCSLIFIGHPDEVTCVLQQTVFGITYSISLSSVLAKTIIVVMAFQSTKPGSKLHKWMGSRVSYSIVLS
CSIFQVLLCLVWLGTAPPFPYLNMQAEDGTIQIECNEGSAIALYCVLGYLGFLAGISFIVAFLARNLPDSFNEAKHITFSMLVFCSVWVSFFPTYLSTRGKYMVAVEIFAILASSSGILGCIFFPKCYIILLKPDRNNRKYITKK---
>Caecillan.locus40288.1
CIQCPEDQWPNEKRDACVPKRISFLSYQEPLGIILMFISIFFFLITVVILGIFIHYGDTPIVRSNNRNLSYILLVSVMLCFLCSLIFIGYPQKVTCFLRQIAFGITFSISLSSVLAKTITVVMAFYTTKPGSKLQKWMGSRVSYSIVFS
CPLLQVILCIIWLDIAPPFPHLNMQAE--TIQIECNEGSAIAFYCVLGYLGFLAGISFIVAFLARNLPDGFNEAKHITFSMLVFCSVWVSFIPTYLSTRGKYMVAVEVFAILASTSGILGCIFFPKCYIILLRPHMNNRKYITKK---
>Caecillan.locus40336.1
CMKCPEEQWPNEKRDVCVPKLISFLSHEEPLGIILTLISIFFFLITAVILGIFIKYQDTPIVRSNNRNLSYILLVSLMLCFLSSLIFIGHPEEVTCVFRQTVFGITFSISLSSVLAKTVTVVMAFQATKPGSKLRKWMGSRVSYSIVFS
CSLLQVLICLVWLGTATPFPYQNFQSEIGTILIECNEGSAIAFYCVLGYLGFLAGISFIVAFLARNLPGSFNEAKHITFSMLMFCSVWVSFIPTYLSTRGKYMVAVEIFGILVSSSGILGCIFIPKCHIILLRPDRNNRKYIITK---
>Caecillan.locus40337.1
CMKCPEEQWPNEKRDVCVPKLISFLSHEEPLGIILTLISIFFFLITAVILGIFIKYQDTPIVRSNNRNLSYILLVSLMLCFLCSLIFIGHPEEVTCILRQTVFGITFSISLSSVLAKTVTVVMAFQATKPGSKLRKWMGSRVSYSIVFS
CSLLQVIICLVWLGTATPFPHQNIQSEIGTILIECNEGSAIAFYCVLGYLGFLAGISFIVAFLARNLPGSFNEAKHITFSMLMFCSVWVSFIPTYLSTRGKYMVAVEIFGILVSSSGILGCIFIPKCYIILLRPDRNNRKYIITK---
>Caecillan.locus40448.1
CMKCPEDQWSNEKRDVCVPKMISFLSYEETLGIILTFISIFFFLITAVILRIFIKYRDTPIVRSNNRNLSYILLVSLMLCFLCSLIFISHPNEVICVLRQSVFGIAFSISLSSVLAKTITVVMAFQATKPGSKLQKWMGSRFSYSIGIS
CSLLQVLICLVWLGTATPFPYQNIQSEIGTILIECNEGSTIAFYCVLGYLGFLAGISFIVAFLARNLPGSFNEAKHITFSMLVFCSVWASFIPTYLSTRGKYMVAVEIFAILASSSGILGCIFIPKCYIILLRPDRNNRKYIVTK---
>Caecillan.locus40387.1
CMKCPEDQWPNEKRDVCVPKLINFLSNEEPLGIILTLISIFFFVITAVILGIFIKYQDTPIVRSNNRNLSYILLVSLMLCFLCSLIFIGHPDEVTCVFRQTVFGITFSISLSSVLAKTITVVMAFQATKPGSKLRKWMGSRVSYSIVFS
CSLLQVLICLVWLGIATPFSHQNIQSEIGTILIECNEGSAIAFYCVLGYLGFLAGISFFVAFLARNLPGSFNEAKHITFSMLMFCSVWISFIPTYLSTRGKYMVAVEIFAIMASSSGILGCIFIPKCYIILLRPDRNNRKYIITK---
>Caecillan.locus40390.1
CMKCPEDQWPNEKRDVCVPKMIIFLSHEEPLGIILTLIIIFFFLITAVILGIFIKYQDTPIVRSNNRNLSYILLVSLMLCFLCALIFIGHPEEVTCIFRQTAFGITFSISLSSVLAKTITVVIAFQATKPGSKLRKWMGSRVSYFIILS
CSLLQVLICLVWLGTATPFPHQNIQSEIGTILIECNEGSAIAFYCVLGYLGFLASISFIVAFLARNLPDSFNEAKHITFSMLMFCSVWVSFIPTYLSTKGKYMVAVEIFAILASSSGILGCIFIPKCHIILLRPDRNNRKYIITK---
>Caecillan.locus40450.1
CMKCPEDQWPNEKRDVCVPKMIIFLSHKEPLGIILTYISIFFFLITAVILGIFIKYKDTPIVRSNNRNLSYILLVSLMLCFLCSLIFIGHPDKVTCVFRQTAFGIIFSISLSSVLAKTITVVIAFQATKPGSKLRKWMGSRVSYSIIFS
CSLLQVLICLVWLGTAAPFPYQNIQSEIGTILIECNEGSSIAFYCVLGYLGFLAGISFIVAFLARNLPGSFNEAKHITFSMLMFSSVWISFIPTYLSTKGKYMVAVEIFAILASSSGILGCIFIPKCHIILLRPDRNNRKYIITK---
>Caecillan.locus40385.1
CMKCPEDKWPNEKRDVCVPKFISFLSHEEPLGIILTLISIFFILITAVLLGIFIKYHDTPIVRSNNRNLSYILLVSLMLCFLCSLIFIGHPDEVTCVFRQTAFGIIFSISLSTVLAKTITVVMAFQATKPGSKLRKWMGSRVSYSIIFS
CSLLQVLICLVWLGTATPFPHQNIHSEIGTILIECNEGSAIAFYCVLGYLGFLAGISFIVAFLARNLPGSFNEAKHITFSMLMFCSVWVSFIPTYLSTRGKYMVAVEIFAILASSSGILGCIFIPKCHIILLRPDRNNRKYIITK---
>Caecillan.locus40338.1
CMKCPEDQWPNEKRDVCVPKLISFLSYEETLGIILTLISIFFFLITAFLLGIFIKYQDTPIVRSNNRNLSYILLVSLMLCFLSSLIFIGHPDEMTCVLRQTAFGITFSISLSSVLAKTVTVVMAFQATKPGNKLRKWMGSRVSYFIVLS
CSLLQVLLCLVWLGTATPFPHQNIQSEVGTILIECNEGSAFAFYSVLGYLGLLAGISFIVAFLARNLPGSFNEAKHITFSMLVFCSVWISFIPTYLSTKGKYMVAVEIFAILASSSGILGCIFIPKCHIILLRPDRNNRKYIITK---
>Caecillan.locus40365.1
CMKCPEDQWPNEKRDVCVPKMISFLSHEEPLGIILTLISIFFFLITAIILGIFIKYQDTPIVRSNNRNLTYILLVSLMFCFLSSLIFIGHPDEVTCVLRQIVFGIIFSISLSAVLAKTITVVMAFQATKPGSKFRKWMGSRVSYSIVFS
CSLLQVLLCLVWLGTTIPFPHQNIHSEIGIILIECNEGSAIAFYCVLGYLGFLAGISFIVAFLARNLPGSFNEAKHITFSMLMFCSVWVSFIPTYLSTRGKYMVAVEIFAILASSSGILGCIFIPKCHIILLRPNRNNRKYII-K---
>Caecillan.locus40371.1
CMKCPEDQWPNEKRDVCVPKMISFLSHEEPLGIILTLISIFFFLITAIILGIFIKYQDTPIVRSNNRNLTYILLVSLMFCFLSSLIFIGHPDEVTCVLRQIVFGIIFSISLSAVLAKTITVVMAFQATKPGSKLRKWMGSRVSYSIVFS
CSLLQVLLCLVWLGTTTPFPHQNIHSEIGIILIECNEGSAIAFYCVLGYLGFLAGISFIVAFLARNLPGSFNEAKHITFSMLMFCSVWVSFIPTYLSTRGKYMVAVEIFAILASSSGILGCIFIPKCHIILLRPNRNNRKYII-K---
>Caecillan.locus40364.1
CMKCPEEQWPNEKRDVCVPKMISFLSHEEPLGIILTLISIFFFLITAVILGIFIKYQDTPIVRSNNRNLTYILLVSLMFCFLSSLIFIGHPDEVTCVLRQTVFGITFSISLSSVLAKTITVVMAFQATKPGSKLRKWMGSRVSYSIVFS
CSLLQVLLCLVWLGTTTPFPHQNIHSEIGIILIECNEGSAIAFYCVLGYLGFLAGISFIVAFLARNLPGSFNEAKHITFSMLMFCSVWVSFIPTYLSTRGKYMVAVEIFAILASSSGILGCILIPKCHIILLRPDKNNRKYIITK---
>Caecillan.locus40339.1
CMKCPEEQWPNEKRDVCVPKLISFLSHKELLGITLTLISIFFFLITAVILGIFIKYQDTPIVRSNNRNLTYILLVSLMFCFLCSLIFIGHPDEVTCVLRQTAFGITFSISLSSVLAKTITVVMAFQATKPGSKLRKWMGSRVSYSIVLS
CSLLQVLLCLVWLGTATPFPHQNIQSEIGIILIECNEGSAIAFYCVLGYLGFLAGISFIVAFLARNLPDSFNEAKHITFSMLIFCSVWISFIPTYLSTRGKYMVAVEIFAILASSSGILGCIFIPKCHIILLRPDRNNRKYIITK---
>Caecillan.locus40362.1
CMKCPEDQWPNEKRDGCVPKLISFLSHEEPLGIILTLISIFFFLITAVILGIFIKYQDTPIVRSNNRNLTYILLVSLMFCFLCSLIFIGHPEEVTCILRQSAFGITFSISLSSVLAKTITVVMAFQATKPASKLRKWMGSRVSYSIVFS
CSLLQVLICLVWLGTAPPFPHQNIQSEIGTILIECNEGSAIAFYCVLGYLGFLAGISFIVAFLARNLPDSFNEAKHITFSMLMFCSVWVSFIPTYLSTRGKYMVAVEIFAILASSSGILGCIFITKCHIILLRPDRNNRKFLTTKIN-
>Caecillan.locus40361.1
CMKCPEDQWPNEKRDGCVPKFISFLSHEEPLGIILTLISIFFFLITAVILGIFIKYQDTPIVRSNNRNLTYILLVSLMLCFLCSLIFIGHPEEVTCILRQSAFGITFSISLSSVLAKTITVVMAFQATKPASKLRKWMGSRVSYSIVFS
CSLLQVLICLVWLGIAPPFPHQNIQSEIGAILIECNEGSAIAFYCVLGYLGFLAGISFIVAFLARNLPDSFNEAKHITFSMLMFCSVWVSFIPTYLSTRGKYMVAVEIFAILASSSGILGCIFITKCHIILLRPDRNNRKFLTTKIN-
>Caecillan.locus40363.1
CVKCLEDQWPNEKRDVCVPKLISFLSHDEPLGIILTLISIFFFLITAVILGIFIKYQDTPIVRSNNRNLTYILLVSLMLCFLCSLIFIGYPEEVTCILRQSAFGITFSIALSSVLAKTITVVMAFHASKPGSKLRKWMGSRVSYSIVLS
CSLLQVLLCLIWLGTATPFPHQNIQSEIGTILIECNEGSAIAFYCVLGYLGFLAGISFIVAFLARNLPSSFNEAKHITFSMLVFCSVWVSFIPTYLSTRGKFMVAVEIFAILASSSGILGCIFIPKCHIILLRPDRNNKKYIITK---
>Caecillan.locus40334.1
CIRCPEDQWPNEKRDVCVSKIISFLSHEETLGIILTLISILFFFITAVILGIFIKYQDTPIVKSNNRNLSYILLGSLMLCFLCSLIFIGRPNEVTCVLRQTAFGIIFSISLSSVLAKTITVIMAFQTTKPGSKLRKWMDPRVSYSIVLS
CSLFQVILCLVWLSTASPFPHHNIHSEIGKILIECNEGSVIALYCVLGYLGFLAGISFIVAFLAKNLPDSFNEAKHITFSMLMFCSVWVSFIPTYLSTRGKYMVAVEVFAILASGSGILGCIFIPKCYIILLKPDRNNRNYITKK---
>Caecillan.locus40466.1
CMRCPEDQWPNEKRDVCVPKIISFLSHEEPLGIVLMLLSLFFFLITTVILGIFIKYRDTPIVRSNNTNLSYILLVSLMLCFLCSMIFIGHPDKVTCVLRQTAFGISFSISLSSVLAKTITVVMAFQTTKPGSKLQKWMGLRVSYSIVLS
CSLLQVLLCLVWLVTATPFPHYNIKSEIGTILIECNEGSAIAFYCVLGYLGFLAGISFVVAFLARNLPGSFNEAKHITFSMLMFCSVWVSFIPTYLSTRGKYTGAVEIFAILGSSSGILGCIFVPKCYIILLKPHRNNRKYITKNINE
>Caecillan.locus40464.1
CMRCPEDQWPNEKRDVCVPKIISFLSHEEPLGIVFIFISLFFFLINAVILGIFIKYRDTPIVRSNNRNLSYILLVSLILCFVCSLIFIGHPEEVICIFRQTAFGISFSISLSSVLAKTITVVMAFQSTKLGSKLRKWMGPRVSYSIILS
CSLLQVFICLVWLGTAPPFPYHNIHSEIGTIFIECNEGSAIAFYCVLGYLGFLAGISFIVAFLARNLPDSFNEAKHITFSMLVFCSVWVSFIPTYLSTRGKYMVAVEIFAILASSSGILGCIFIPKCYIILFKPHRNNRKYITKK---
>Caecillan.locus40463.1
CMRCPEDKWPNEKRDVCIPKMISFLSHEEPLGIVLILLCLFFFLINAVILGIFIKYRDTPIVRSNNRNLSYILLVSLILCFVCSFIFIGHPEEVTCILRQTAFGISFSISLSSVLAKTITVVMAFQSTKPGSKLRKWMGSRVSYSIVLS
FPLLQVLLCLVWLGTAPPFPHHNIQSEIGTILIECNEGSAIAFYCVLGYLGFLAGISFIVAFLARNLPGSFNEAKHITFSMLVFCSVWVSFIPTYLSTKGKYTIAVEIFAILASGSGILGCIFIPKCFIILLRPERNNRKHITKK---
>Caecillan.locus40335.1
CMRCPEDQWPNEKRDVCIPKMISFLSHEEPLGIVLIFLSLFFFLITAVILGIFIKYRDTPIVRSNNRNLSYILLVSLILCFVCSLIFVGHPVEVTCILQQTAFGITFSISLSSVLAKTITVVMAFQTTKPGSKLRKWLGSKVSYSIVLS
CSLLQVLLCLVWLGTAPPFPHHNIQSEIGTILIECNEGSTIAFYCVLGYLGFLAGISFIVAFLARNLPNSFNEAKHITFSMLVFCSVWVSFIPTYLSTRGKYMVAVEIFAILASGSGILGCIFIPKCYIILLRPDRNNRKYITKI---
>Caecillan.locus40455.1
CMRCPEDQWPNEKRDVCIPKMISFLSHEEPLGIVFIFISLFFFLITAVILGIFIKYRDTPIVRSNNRNLSYILLVSLILCFLCSLIFIGHPEKVTCFLRQTAFGITFSISLSSVLAKTITVVMAFQTTKPGSKLRKWMGSRVSYSIVLS
CSLLQVFICFVWLGTAPPFPHHNIQSEIGTILIECNEGSAVAFYCVLCYLGFLAGISFIVAFLARNLPDSFNEAKHITFSMLVFCSVWISFIPTYLSTRGKYMVAVEIFAILASGSGILGCIFIPKCYIILLRPDRNNRKYITKN---
>Caecillan.locus40291.1
-MRCPEDQWPNEKRDVCIPKMISFLSHEEPLGIVLMLISIFFFLITAVILGIFIKYRDTPIVRSNNRNLSYILLVSLMLCFLCSLIFIGHPKEVTCILRQTAFGITFSISLSSVLAKTITVVMAFQTTKPGSKFRKWMGSRVSYSIVLF
CSLLQVLLCLVWLGTAPPFPQQNIQSKIGTILIECNEGSAIAFYCILGYLGFLAGISFIVAFLARNLPNSFNEAKHITFSMLVFCSVWVSFIPTYLSTRGKYMVAVEIFAILASSSGLLGCIFIPKCYIILLRPDRNNKKYITKK---
>Caecillan.locus40356.1
CVACPEDQWPNEKRDVCIPKIISYLSYEEPLGIILIHISIF---
ITAVILGIFINYRDTPIVKSNNTNLSYILLVSLMLCFLCSLIFIGRPEELTCVLRQIAFGITFSISLSSVLAKTITVVMAFQSTKPGSKLRKWMGSRVSYSIVFSCSLLQALLCLVWLCTAPPFPYYNIQSEIGTILIECKEGSAVAFY
CVLGYLAFLVCISFIVAFLARNLPDSFNETKHITFSMLLFCSVWVSFIPTYLSTRGKYMVAVEIFAILASSSGILGCIFIPKCYIILLRPEKNNRMYITKNKLT
>Caecillan.locus40514.1
CMACPEDQWPNQKRDACIPKMKAFLSHEDPLGIALTLISILFFLFTAVTLGIFIYYRNTPIVKANNRELSYILLISLMLCFLCSLVFIGQPTDITCILRQTVFGITFSIALSAILAKTITVVMAFQAIKPGNKLQKWIGPWFSNSIVLS
CLLIQTVLCFVWLGTAPPFSYLNMHSEIGTILIECNTGSMVAFFCVHGFLGILASISFTVAFLARNLPDSFNEAKHITFSMLVFYSVWVSFIPTYLSTRGKYMAAVEIFAILASASGILGCIFIPKCYIIMMRPDKNNRKNLTKKGND
>Zebrafish.locus535.1
CFLCPDEFWSNQYNNHCVPKEVEFLSYEDPLGISLTTASLLGTCFCALVIFVFALHRNTPIVRANNSELSFLLLLSLKLCFLCVLLFIGRPQLWTCQLRHVVFGISFVLCMSSILVKTMVVIAVFKSSRPGKGAIKWFGAVQQRSTVVV
LTVLQVLICAVWLSTASPTPYKNNQYIRSKIVYECAIGSVVGFSMLLSYIGFLAAASFLLAFLARNLPDNFNEAKFITFSMLIFSAVWIAFVPAYVSSPGKYTVAVEIFAILASTFGLLIAIFAPKCYIILLHPERNTKNVIMGRETQ
>marker.zebrafish_16_16_F

CPDEFWSNQYNNHCVPKEVEFLSYEDPLGISLTTASLLGTCFCALVIFVFALHRNTPIVRANNSELSFLLLLSLKLCFLCVLLFIGRPQLWTCQLRHVVFGISFVLCMSSILVKTMVVIAVFKSSRPGKGAIKWFGAVQQRSTVVVLTV
LQVLICAVWLSTASPTPYKNNQYIRSKIVYECAIGSVVGFSMLLSYIGFLAAASFLLAFLARNLPDNFNEAKFITFSMLIFSAVWIAFVPAYVSSPGKYTVAVEIFAILASTFGLLIAIFAPKCYIILLHPERNTKNVIMGRE--
>Zebrafish.locus536.1
--
MCPDEFWSNLDKDQCIPKEVEFLSYEDPLGISLTTASLLGTCFCVLVMFVFAVHRNTPIVRANNSELSFLLLLSLKMCFLCALLFIGQPQLWTCQLRHVVFGISFVLCISSILVKTMVVIAVFKSSRPSKGAMKWFGAVQQRCTVLVLT
AIQVVICAVWLSTASPAPHKNNQYIRSKIVYECAIGSVAGFSMLLAYIGLLAAVSFLLAFLARNLPDNFNEAKFITFSMLIFCAVWIAFVPAYVSSPGKYAVAVEIFAILASSFGLLLAIFAPKCYIILLHPERNTKKAIMGKEAQ
>marker.zebrafish_16_17_F

CPDEFWSNLDKDQCIPKEVEFLSYEDPLGISLTTASLLGTCFCVLVMFVFAVHRNTPIVRANNSELSFLLLLSLKMCFLCALLFIGQPQLWTCQLRHVVFGISFVLCISSILVKTMVVIAVFKSSRPSKGAMKWFGAVQQRCTVLVLTA
IQVVICAVWLSTASPAPHKNNQYIRSKIVYECAIGSVAGFSMLLAYIGLLAAVSFLLAFLARNLPDNFNEAKFITFSMLIFCAVWIAFVPAYVSSPGKYAVAVEIFAILASSFGLLLAIFAPKCYIILLHPERNTKKAIMGKE--
>Zebrafish.locus814.1
-MLCPDEFWSSPDKDQCVPKEVEFLSYEDPLGISLTTASLLGSCFCVLVMIIFGLHRNTPIVRANNSELSFMLLLSLKMCFLCVLLFIGRPQLWTCQLRHAVFGISFVLCISSILVKTMVVIAVFKSSRPGKGAMKWFGAAQQRCTVLV
LTVLQVVICSVWLSTASPTPYKNNQYIRSKIVYECAIGSVAGFSLLLGYIGLLAAVSFLLAFLARNLPDNFNEAKFITFSMLIFCAVWIAFVPAYVSSPGKYAVAVEIFAILASSFGLLVAIFAPKCYIILLHPERNTKNAIMGRETQ
>Zebrafish.locus538.1
-MLCPDEFWSSPDKDQCVPKEVEFLSYEDPLGISLTTASLLGSCFCVLVMIIFGLHRNTPIVRANNSELSFMLLLSLKMCFLCVLLFIGRPQLWTCQLRHAVFGISFVLCISSILVKTMVVIAVFKSSRPGKGAMKWFGAAQQRCTVLV
LTVLQVVICSVWLSTASPTPYKNNQYIRSKIVYECAIGSVAGFSLLLGYIGLLAAVSFLLAFLARNLPDNFNEAKFITFSMLIFCAVWIAFVPAYVSSPGKYAVAVEIFAILASSFGLLVAIFAPKCYIILLHPERNTKNAIMGRETQ
>Zebrafish.locus534.1
CMMCPDEFWSSPDKDQCVPKEVEFLSYEDPLGISLTTASLLGTCFCALVMIIFALHHNTPIVRANNSELSFLLLLSLKLCFLCVLLFIGRPQLWTCQLRHAVFGISFVLCVSSILVKTMVVIAVFKSSRPGKGAMKWFGASQQRCTVLV
LTALQVVICAVWLSTASPTPHKNNHYFRSIIVYECAIGSVAGFSLLLGYIGLLAAISFLLAFLARNLPDNFNEAKFITFSMLIFCAVWIAFVPAYVSSPGKYAVAVEIFAILASSFALLLAIFAPKCYIILLHPERNTKNAIMGRETQ
>Zebrafish.locus533.1
CTVCPDEFWSNPEKDQCVPKEVEFLSYEDPLGISLTTASLLGTCFCALVMIIFALHRNTPIVRANNSELSFLLLLSLKMCFLCVLLFIGRPQLWTCQLRHAVFGISFVLCISSILVKTMVVIAVFKASRPGKGAMKWFGATQQRCTVLV
LTALQVVICVVWLLTASPTPHKNNQYIRSKIVYECAIGSVAGFSLLLGYIGLLAAISFLLAFLARNLPDNFNEAKFITFSMLIFCAVWIAFVPAYVSSPGKYAVAVEIFAILASSFGLLVAIFAPKCYIILLHPERNTRNAIMGREMQ
>Zebrafish.locus520.1
CTVCPDEFWSNLNKDQCVPKEIDFLSYEDPLGISLTTTSLLGTCFCALVMIIFTFHRNTPIVRANNSELSFLLLLSLKLCFLCVLLFIGQPQLWTCQLRHAVFGISFVLCISSILVKTMVVIAVFKSSRPGSGAMKWFGTAQQRCTVLV
LTALQIVICAVWLSTSSPTPYKNNQSIRSKIVYECAIGSLAGFSLLLGYIGLLAAISFLLAFLARNLPDNFNEAKFITFSMLIFCAVWVAFVPAYMSSSGKYAVAMEIFAILASSFGVLVAIFAPKCYIIILHPERNTKKAIMGRENK
>marker.zebrafish_16_2_F

CPDEFWSNLNKDQCVPKEIDFLSYEDPLGISLTTTSLLGTCFCALVMIIFTFHRNTPIVRANNSELSFLLLLSLKLCFLCVLLFIGQPQLWTCQLRHAVFGISFVLCISSILVKTMVVIAVFKSSRPGSGAMKWFGTAQQRCTVLVLTA
LQIVICAVWLSTSSPTPYKNNQSIRSKIVYECAIGSLAGFSLLLGYIGLLAAISFLLAFLARNLPDNFNEAKFITFSMLIFCAVWVAFVPAYMSSSGKYAVAMEIFAILASSFGVLVAIFAPKCYIIILHPERNTKKAIMGRE--

```
>Zebrafish.locus521.1
CTVCPDEFWSNLNKDRCVPKEIDFLSYEDPLGISLTTTSLLGTCFCALVMIIFTFHRNTPIVRANNSELSFLLLLSLKLCFLCVLLFIGQPQLWTCQLRHAVFGISFVLCISSILVKTMVVIAVFKSSRPGSGAMKWFGTAQQRCTVLV
LTALQIVICAVWLSTSSPTPYKNNQSIRSKIVYECAIGSVAGFSLLLGYIGLLAAISFLLAFLARNLPDNFNEAKFITFSMLIFCAVWVAFVPAYMSSSGKYAVAMEIFAILASSFGVLVAIFAPKCYIIILHPERNTKKAIMGRENK
>Zebrafish.locus526.1
CTVCPDEFWSNFNKDQCVPKEIEFLSYEDPLGISLTTASLLGTCFCALVMIIFTFHRNTPIVRANNSELSFLLLLSLKLCFLCVLLFIGQPQLWTCQLRHAVFGISFVLCISSILVKTMVVIAVFNSSRPGTGAMKWFGAAQQRCTVLV
LTALQVVICAVWLSTSPPTPHKNSQYVRSKIVYECAIGSVAGFSLLLGYIGLLAAISFLLAFLARNLPDNFNEAKFITFSMLIFCAVWIAFVPAYVSSPGKYAVAVEIFAIVASSFGVLVAIFAPKCYIIILHPERNTKKAIMGRETK
>Zebrafish.locus525.1
CTVCPDEFWSNFNKDQCVPKEIEFLSYEDPLGISLTTASLLGTCFCALVMIIFAFHHNTPIVRANNSELSFLLLLSLKLCFLCVLLFIGQPQLWTCQLRHAVFGISFVLCISSILVKTMVVIAVFNSSRPGKGAMQWFGAAQQRCTVLV
LTVLQVVICAVWLSTSSPTPHKNNQYVRSKIVYECAIGSVAGFSLLLGYIGLLAAISFLLAFLARNLPDNFNEAKFITFSMLIFCAVWIAFVPAYVSSPGKYAVAVEIFAILASSFGVLVAIFAPKCYIIILHPERNTKKAIMGRETK
>Zebrafish.locus532.1
CVTCPDEFWSNFDKNQCVPKEVEFLSYEDPLGISLTTASLLGTCFCALVMIIFALHLNTPIVRANNSELSFLLLLSLKLCFLCVLLFIGQPQLWTCQLRHVVFGISFVLCISSILVKTMVVIAVFKSSRPGKGTMKWFGAAQQRCTVLV
LTALQVVICAVWLSNASPTPIKNNQYIRSKIVYECAIGSVAGFSLLLGYIGLLAAISFLLAFLARNLPDNFNEAKFITFSMLIFCAVWIAFVPAYVSSPGKYAVAVEIFAILASSFGLLVAIFAPKCYIILLHPERNTKKAIIRRETK
>Zebrafish.locus522.1
CTVCPDDFWSNLYKDQCIPKEVEFLSYNDPLGISLTTASLLGTCFCAFVMVIFVHHHNTPIVRANNSELSFLLLFSLKLCFLCVLLFIGRPQLWTCQLRHAVFGISFVLCISSILVKTMVVIAVFKSSRPGKMAMKWFGAAQQRCTILA
LTAIQVVICVVWLSTASPTPHKNNLYIRSIIVYECTIGSVTGFSMLLGYIGLLAAVSFLIAFLARNLPDNFNEAKFITFSMLIFCAVWITFVPAYVSSPGKYSVAVEIFAILASSFGLLVSIFAPKCYIILLHPERNNKKAIMGRETH
>marker.zebrafish_16_6_F
---
CPDDFWSNLYKDQCIPKEVEFLSYNDPLGISLTTASLLGTCFCAFVMVIFVHHHNTPIVRANNSELSFLLLFSLKLCFLCVLLFIGRPQLWTCQLRHAVFGISFVLCISSILVKTMVVIAVFKSSRPGKMAMKWFGAAQQRCTILALTA
IQVVICVVWLSTASPTPHKNNLYIRSIIVYECTIGSVTGFSMLLGYIGLLAAVSFLIAFLARNLPDNFNEAKFITFSMLIFCAVWITFVPAYVSSPGKYSVAVEIFAILASSFGLLVSIFAPKCYIILLHPERNNKKAIMGRE--
>Zebrafish.locus524.1
CTVCPDDFWSNLYKDQCIPKEVEFLSYYDPLGISLTTASLLGTCFCAFVMVIFVHHHNTPIVRANNSELSFLLLFSLKLCFLCVLLFIGRPQLWTCQLRHAVFGISFVLCISSILVKTMVVIAVFKSSRPGKKAMKWFGAAQQRCTILV
LTAIQVVICAVWLSTASPTPHKNNLYIRSIIVYECTIGSVTGFSMLLGYIGLLAAVSFLIAFLARNLPDNFNEAKFITFSMLIFCAVWITFVPAYVSSPGKYSVAVEIFAILASSFGLLVAIFAPKCYIILLHPERNNKKTIMGRETH
>Cloudy_catshark.locus87.1
CFQCPEDYWSNKKKDMCVPKEVEFLSFHEPLAGVLLFFSILGIIISVAIIVIFIKFMNTPIVRANNYELSFLLLFSLTLCFLTSVTFIGKPSAEFCVLRQMGFGTSFSLSLSCILVKTIVVVLAFTMTTPHQNVLKRFRPMHHRMIVAA
ITLIQISICVGLLLSSSLSVEKNTMAVVGKIILECSNGSEHAYFGNLGYVGLLALLCFVLAFLARKLPNNFNEAKFITFSLLIFFAVWISFLPAYFSTKGKYLVAIEIFAILASSFGLLACIFFPKCYVVLLRPELNTKRGLMGRAPQ
>Great_white_shark.locus10.1
CFQCPEDYWSNQKKDKCVPKEVEFLSFYEPLGNVLTFFSILGVTISVAIIVIFIKFMNTPLVRANNYELSFLLLFSLMLCFLISLIFIGKPSVKVCVLRQIGFGTSFSLSLSCILVKTTVVVLTFAMTTPNQNVLKHFRPMHHRMSVAA
ITLIQIGICIGFLLSSPLSVEKNTMAIGGKIILQCSKGSEQALFGILGYVGLLAVSCFVLAFLARKLPNNFNEAKFITFSLLIFFAVWISFLPAYFSSKGKYLVAIEIFAILSSSFGLLACIFFPKCYVVLLRPELNTKRGLMGRAEK
>Brownbanded_bambooshark.locus519.1
CFQCPEDYWSNINKDQCVPKEVEFLSFYEPLGSILTFFSILGVTTSAAVILIFIKFMNTPIVRANNYELSFLLLFSLMLCFLTSFAFIGQPSVEFCVLRQIGFGISFTLCLSCILVKTVVVVLAFKMTTPNQNVLKHFQPIHHRLVVTA
TMLIQICICVGFLFSLPIAVQKNTKAAVGKIILECSKGSEHAFFGNLGYIGFLSVLGFVLAFLARKLPNNFNEAKFITFSLFIFFAVWVSFLPAYFSIKGKYLVAIEIFAILSSSFGLLVCIFFPKCYVLLLRPELNTKRGLMDRAHL
>Thorny_skate.locus24.1
CFECPEDYWSNKQKDTCVLKEVEFLSFDEPLGIVLTFFSILGIAISAAILAIFIKFMNTPIVRANNYELSFLLLFSLTLCFLASLAFLGRPTVKFCILRQIGFGIGFSLCLSCILVKTIVVVLAFTMTKPNQNALKRFRPMYHRVIVAS
ITFIQICICIGFLLSSPISVQKDATVAVGKIILECSQGAEQTLFAILIYIGLLAILCFILAFLARRLPNNFNEAKFISFSLLIFFVVWVSFIPAYFSSKGKYIVAVEVFAILASTFGLLICIFFPKCYIILLKPELNTKREMMGRAPA
>Little_skate.locus5.1
CFECPEDYWSNKQKDTCLLKEVEFLSFDEPLGIVLTFFSVLGITISAAILAIFMKFMNTPIVRANNYELSFLLLFSLILCFLASLAFLGRPTVKFCILRQIGFGIGFSLCLSCILVKTIVVVLAFTMTKPNQNALKRFRPMYHRVIVAS
ITFIQICICIGFLLSSPISVQKDATVAVGKIILECSQGAEQTLFAILIYIGLLAILCFILAFLARKLPNNFNEAKFISFSLLIFFVVWVSFIPAYFSSKGKYIVAVEVFAILASTFGLLICIFFPKCYIILLKPELNTKREMMGRAPA
>Smalltooth_sawfish.locus20.1
CFQCPVDYWSNQQKDKCILKEVEFLSFEEPLGIVLTFFSIFGITISVAILVIFIRFMNTPIVRANNYELSFLLLFSLILCFLASVAFIGEPTVELCVLRQIGFGIGFSLCLSCILVKTIVVVLAFTMTKPNQTVLKHFRPMHHRVIVVA
ITFIQICICIGFLTASPLSVQKDTTVAVGKIILECSQAAEQTLFAILVFIGLLAILCFILAFLARKLPNNFNEARFITFSILIFFVVWFSFIPAYFSSKGKYIVAVEVFAILSSSFGLLICIFFPKCYVILFRPELNKKRELMGRTSA
>marker.fugu_15_1_F
---
CPPEFWSNDGRTACVPRQLDFLSFNETLGVALTAVAVSGAVVTTAVFVVFLHYRHTPMVRANNSELSFLLLLSLKLCFLCSLVFIGRPSVWSCRFQQAAFGISFVLCVSCLQVKTIVVLAAFRSARPGAALMKWFGPSQQRGSVCIFTC
VQVIICIVWLSLSPPVPQADLDVPGLQVTLECAMASVVGFSLVLGYIGLLACTCLLLAFLARKLPDNFNEAKLITFSMLIFCAVWVAFVPAYISSPGKYSVAVEIFAILASSYGLLFCIFAPKCFIILLRPEKNTKKHLMMR---
>Reedfish.locus27554.1
CMSCPLDYNSNEQKDHCVLKVIEFLSFGEIMGIILVTCSLFGVTLTALVASIFFYYKETPVVKANNSELSFLLLLSLMLCFSCSLTFIGEPSQWSCTIRHTTFGITFVLCISCILAKTIVVLMAFRATLPGNNIMKWFGPTQQRLSVLA
FTLIQVLICALWLIISPPFPHKNMVLYKEKIIFECDLGSPTAFYAVLGYIGFLSAMCFSLAFLARKLPDNFNEAKFITFSMLLFCAVWISFIPAYISSPGKYTVAVEIFAILTSSFGLLLCIFAPKCYIILLKPEENTKKHLMGKALT
>Reedfish.locus40520.1
CLSCPLDYNSNEQKDHCVLKVIEFLSFGEIMGIILVTCSLFGVTLTALVASIFFYYKETPVVKANNSELSFLLLLSLMLCFSCSLTFIGEPSQWSCTIRHTTFGITFVLCISCILAKTIVVLMAFRATLPGNNIMKWFGPTQQRLSVLA
FTLIQVLICALWLIISPPFPHKNMVLYKEKIIFECDLGSPTAFYAVLGYIGFLSAMCFILAFLARKLPDNFNEAKFITFSMLLFCAVWISFIPAYISSPGKYIVAVEIFAILTSSFGLLLCIFAPKCYIILLKPEENTKKHLMGKALT
>Whale_shark.locus79.1
------------------------------
MGIILVILAFLGASTTIGVFVLFYVHRNTPIVKANNSELSFLLLCALTLCFLCSMTFLGEPSTWSCMLRHTAFGVIFVLCISCVLSKTVVVVMAFNAVLPSNNTMRWFGPTQQRLSVCVLTLVQCLICAVWLRTSPPYPSKNSNYFSEI
IVFECNVGSPIAFYCVLGYIGFLSCVCFVVAFLARQLPNNFNEAKHITFSMLIFCAVWITFIPAYISSPGKYAVAVQVFAILASSYGLIFCIFAPKCFIILLRKDXNTKKHMMVK---
>Whale_shark.locus77.1
CIKCPWEYRSNWRRDECILKVIEFLSFGDLMGIVLVTLAFLGACITIGVFVLFYVHRNTPIVKVNNSELSFLLLCALTLCFLCSVTFLGEPSTWSCILRHTAFGVIFVLCISCVLSKTVVVVMAFNAVLPSNNMMRWFGPTQQRLSVCI
LTLVQCLICAVWLRTSPPHPSKNSNYSSEIIVFECNVGSPIAFYCVLGYIGFLSCVCFVVAFLARQLPNNFNEAKHITFSLLIFCAVWITFIPAYISSPGKYAVAVEIFAILASSYGLVFCIFAPKCYTILLRKDRNTKKHMMVK---
>Whale_shark.locus61.1
-----------------------------
MMGIIFVTLALLGVSITIGVFALFYVHRNTPIVKANNSELSFLLLCALTLCFLCSMTFLGEPSTWSCILRHTTFGVIFVLCISCVLSKTVVVVIAFKAVLPGSNMMRWFGPTQQRLSVCFLTLMQCLICLVWLRTSPPYPFKNSDYFSK
IIVFECNVGSSIAFYCVLGYIGFLSCVCFVVAFLARQLPNNFNEAKHITFSMLIFCAVWITFIPAYISSPGKYAVAVEVFAILASSFGLVFCIFAPKCYIILLRKDRNTKKNMMDKLAS
>Cloudy_catshark.locus43.1
-MKCPWDYRSNQQRDKCILKEVEFLSFAELMGIILVTLALFGASLTIGVFVLFHVHRDTPIVKANNSELSFLLLLALILCFLCSITFIGEPSTWSCGLRHTAFGIIFVLCISCVLSKTIVVVMAFRAVLPGSNKMRWFGPVQQRLCVCA
LTLVQCLICTIWLRISPPYPLKNSAYFKDIIVFECNVGSPIAFYCVLGYIGFLSCVSFVVAFLARQLPNNFNEAKYITFSMIIFCAVWITFIPAYVSSPGKYTVAVEVFAILASSFGLVFCIFAPKCYIILLKKEMNTKKHMMDKMAS
>Great_white_shark.locus29.1
CINCPLEYRSNQKRDRCIPKEIEFLSFGETMGIILVVVALLGACITLGIFSTFYMYKDTPVVKANNSELSFLLLVALMLCFLCSITFIGEPSVWSCILRHTAFAVIFVLCISCVLSKTVVVVMAFNAMHPNHNIMKWFGPTQQRLTVIL
LTMVQCLICTVWLSTSPPYPRKSSDYAKDIITFECNVGSVINFYCALGYIGFLSCVCFVVAFLARQLPNNFNEAKHITFSMLIFCAVWITFIPAYISSPGKYAVAVEVFAILASSFGLLLCIFAPKCYIIMFKQAENTKRHIMGNKGS
>Smalltooth_sawfish.locus36.1
------------------------------
MGITLLVVALLGASITMGVFTTFYFYRDTPIVKANNSELSFLLLLALILCFMCSVTFIGEPSVWSCILRHTAFAVIFVLCVSCVLSKTIVVVMAFNATRPNGNIMKWFSPTYQRLTVVLLTMVQCFICTVWLITLPPYPRKNVEYANDI
IVFECSVGSAVNFYCTLGYIGLLSCVCFVVAFLARQLPSNFNEAKHITFSMLIFCAVWLSFIPAYISSPGKYAVAVEVFAILASSFGLLLCIFIPKCYVILLKQEENTKRHIKGNKGS
>Caecillan.locus40504.1
CVSCSEDQRPNHERNSCIPKLITFLSYEDSLGNALTISSIFFFLINAVILRIFITYRDTPIVRANNRDISFILLTSLMSCFLCSLMFIGRPQVTTCILRQAAFGIIFSISLSSILAKTVTVVTAFHATKPGSRFQKWMSSRVSFSIILS
CFLLQVLICLVWLCTAPPFPYLNMHVDTGIIVVECNEGSAIAFYSLLGFLGFLAAISFIIAFLSRNLPDGFNEAKNITFSMLVFCSVWLTFIPAYLSTRGKYTVAVEIFAIQASSAGLLGCIFIPKCYIIILRPDMNSRKCLK-----
>Caecillan.locus40497.1
CVSCREDQRPNHERNTCIPKLITFLSYEDSLGNALTISSIFFFLINAVILRIFITYRDTPIVRANNRDISFILLTSLMSCFLSSLMFIGRPQVTTCILRQAAFGIIFSISLSSILAKTITVVTAFHATKPGSRFQKWMSSRVSYSIILS
CFLLQVLICLVWLCTAPPFPYLNMHVDTGIIVVECNEGSAIAFYSLLGFLGFLAAISFIIAFLSRNLPDGFNEAKNITFSMLVFCSVWLTFIPAYLSTRGKYTVAVEIFAIQASSAGLLGCIFIPKCYIILLRPDMNSRKCLK-----
>Caecillan.locus40502.1
CVSCSENQRPNHERNSCIPKLITFLSYEDSLGNALTISSIFFFLINAVILRIFITYRDTPIVRANNRDISFILLTSLMSCFLCSLMFIGRPQVTTCILRQAAFGIIFTISLSSILAKTVTVVTAFHATKPGSRFQKWMSYRVSFSIILS
CFLLQVLICLVWLCTAPPFPYLNMHVDTGIIVVECNEGSAIAFYCVLGFLAFLAAISFIIAFLSRNLPDGFNEAKNITFSMLVFCSVWLTFIPAYLSTRGKYTVAVEIFAIQASSAGLLGCIFIPKCYIILLRPDMNSRKCLK-----
>Caecillan.locus40494.1
CVSCREDQRPNHERNTCIPKLITFLSYEDSLGNALTTSSIFFFLINAVILRIFITYRDTPIVRANNRDISFILLTSLMSCFLCSLMFIGRPQVTTCILRQAAFGIIFSISLSSILAKTITVVTAFHATKPGSRFQKWMSSRVSISIILS
CFLLQVLICLVWLCTAPPFPYLNMNVDTGIIVVECNEGSAIAFYCVLGFLAFLAAISFIIAFLSRNLPDGFNEAKNITFSMLVFCSVWLTFIPAYLSTRGKYTVAVEIFAIQASSAGLLGCIFIPKCYIILLRPDMNSRKCLK-----
>Caecillan.locus40499.1
CMPCREDQRPNHKRDTCIPKLITFLSYEDSLGKALTTSSIFFFLINVIILRIFINYRDTPIVRANNQDISFILLTSLMSCFLCSLMFIGRPQVMTCILRQASFGITFSISLSSILAKTITVVTAFHATKPGSRFQIWMSSRVSFSIILS
CFLLQVLICLVWLCTAPPFPYLNMNVDTGIIVVECNEGSAIAFYCVLGFLAFLAAISFIIAFLSRNLPDSFNEAKNITFSMLVFCSVWLTFIPAYLSTRGKYTVAVEIFAIQASSAGLLGCIFIPKCYIILLRPDMNSRKCLK-----
>Caecillan.locus40498.1
CMPCREDQRPNHKRDTCIPKLIAFLSFEDFLGKALTTSSIFFFLINVIILRIFINYRDTPIVSANNQDISFILLTSLMSCFLCSLMFIGRPQVMTCILRQASFGITFSISLSSILAETITLVTAFHATKPGSRFQKWMSSRVSFSIILS
CFFLQVLICLVWLCTAPPFPYLNMHVDTGIIVVECSEGSAIAFYCVLGFLAFLAAISFIIAFLSRNLTDGFNEAKNITFSMLVFCSVWLTFIPAYLSTRGKYTVAVEIFAIQASSAGLQGCIFIPKCYIILLRPDMNSRKCLK-----
>Caecillan.locus40492.1
CIKCPEDQWSNQKRDACIPKMIHFLSYEEPLGIALTLISIFFFLSDIVVLGIFIYYRDTPIVKANNRTLSYILLISLMLCFLCSLMFIGHPNHATCVVRQTAFAMTFSVSLSSILAKTITVVIAFHATKPESMYKKWMGSHLSSSIVFF
CTFFQGVTCFVWLGTAPPFPFLNMQAETGAIIIECNEGSLAAFYFVLGYLGFLASISFIIAFLARNLPESFNEARNISFSMLVFCSVWVFFIPTYLSTRGKYTVVVEIFAILTSSAGLLGCIFIPKCYIILLRPDRNNRKYLTKNWND
>Spotted_gar.locus147.1
CFQCPLDSWPNPSRDQCVLKEIEFLSYEETLGISLTTISLFGGCISAAVLAVFIYYRNTPIVRANNSELSFLLLLSLILCFLCSLLFIGQPLHLTCMLRHVVFGISFVLCISCILVKTIVVIMAFRATLPDNNVMKRFGAAQQRGTVFV
FTVIQAVICTVWLSTASPVPFKNMQYQNSKIIFECYIGSITGFSFLLGYIGLLASVCFVLAFLARNLPDNFNEAKFITFSMLIFCAVWIAFIPAYVSSPGKYSDAVEIFAILASSFGLLISIFAPKCYIILIHPEKNTKKVLMGKAIS
>Spotted_gar.locus148.1
CFKCSPDFWPNSSRDQCVLKEIEFLSYEETLGISLTTISVFGACISAGVLAVFIYYRNTPIVRANNSELSFLLLLSLILCFLCSLLFIGQPLHLTCMLRHVVFGISFVLCISCILVKTIVVIMAFRATLPDNNVMKWFGAAQQRGTVFV
FTVIQAMICIIWLSTASPVPFKNTQYQNSKIIFECYIGSITGFSCLLGYIGLLASVCFLLAFLARNLPDNFNEAKFITFSMLIFCAVWIAFIPAYVSSPGKYSDAVEIFAILASSFGLLLSIFAPKCYIILLHPEKNTKKALMSRADP
>Spotted_gar.locus152.1
CFQCPLYSWSNPSRDQCVLKEIEFLSYEDVLGISLSVVSVFGACISAAVLAVFIYYRNTPVVRANNSELSFLLLLSLILCFLCSLLFIGQPLHLTCMLRHVVFGISFVLCISCILVKTIVVIMAFRATLPDNNVMKWFGAAQQRGTVFV
FTVIQALICIIWLSTASPVPFKNTQYQNSKIIFQCNVGSITGFSCLLGYIGLLASVCFLLAFLARNLPDNFNEAKFITFSMLIFCAVWIAFIPAYVSSPGKYSDAVEIFAILASSFGLLTAIFAPKCYIILLCPEKNTKKALMRRAAL
>Western_clawed_frog.locus1009.1
CFKCPINQWPNLNRTICTDKVIIYLSYHEPLGASLALLSVLFCIMTCLVLMIFTKYRNTAVVKANNRDLSYILLVSLKICFLCNLMFIGHPRHVTCILRQTVFGVIFSIVISSVLAKTVTVVIAFNATRPGSKLKNYLGPRVSNSIVIF
CSLIQVIICTSGLINSPPFPYYNTDDIVGVIVAECHEHSRYGFYCILGFMGLLAALSFIIAFFARTLPDIFNEAKFITFSMLLFCSLWVSFIPAYLSTKGKYVVAVEIFAILSSSSGILGCIFIPKCYIILFRADKNKTVLKKRTQ--
>Western_clawed_frog.locus1007.1
CFKCPINQWPNLNRTICTDKVIIYLSYHEPLGASLALLSVLFCIMTCLVLMIFTKYRNTAVVKANNRDLSYILLVSLKICFLCNLMFIGHPRHVTCILRQTVFGVIFSIVISSVLAKTVTVVIAFNATRPGSKLKNYLGPRVSNSIVIF
CSLMQVIICTSGLINSPPFPYYNTDDIVGMIVAECHEHSSYGFYCILGFMGLLAALSFIIAFFARTLPDIFNEAKFITFSMLLFCSVWVSFIPAYLSTKGKYVVAVEIFAILSSSSGILGCIFIPKCYIILFRADKNKTVLKKRTQ--
>Western_clawed_frog.locus997.1
CFKCPINQWPNLNRTICTDKVIIYLSYHEPLGASLALLSVLFCIMTCLVLMIFTKYRNTAVVKANNRDLSYILLVSLKICFLCNLMFIGHPRHVTCILRQTVFGVIFSIVISSVLAKTVTVVIAFNATRPGSKLKNYLGPRVSNSIVIF
CSLMQVIICTSGLINSPPFPYYNTDDIVGMIVAECHEHSSYGFYCILGFMGLLAALSFIIAFFARTLPDIFNEAKFITFSMLLFCSVWVSFIPAYLSTKGKYVVAVEIFAILSSSSGILGCIFIPKS---------------------
>Western_clawed_frog.locus1014.1
CFKCPINQWPNLNRTICTDKVIIYLSYHEPLGASLALLSVLFCIMTCLVLMIFTKYRNTAVVKANNRDLSYILLVSLKICFLCNLMFIGHPRHVTCILRQTVFGVIFSIVISSVLAKTVTVVIAFNATRPGSKLKNYLGPRVSNSIVIF
CSLMQVIICTSGLINSPPFPYYNTDDIVGMIVAECHEHSSYGFYCILGFMGLLAALSFIIAFFARTLPDIFNEAKFITFSMLLFCSVWVSFIPTYLSTKGKYVVAVEIFAILSSSSGILGCIFIPKCYIILFRTDKNKTDLKKRTH--
>Western_clawed_frog.locus908.1
CFKCPINQWPNLNRTICTDKVIIHLSFHQPLGASLALLSVLFFIMTCLVLMIFTKYRNTAVVKANNRDLSYILLVSLKICFLCNLMFIGHPRHVTCILRQTVFGVIFSIVISSVLAKTVTVVIAFNATRPGSKLRNYLGPRVPNSIVIF
CSLTQVIICTSGLINSPPFPYYNTDDIVGVIVAECHEHSSFGFYCILGFMGLLAALCFIIAFFARTLPDIFNEAKFITFSMLLFCSVWVTFIPAYLSTKGKYVVAVEIFAILSSSSGILGCIFIPKCYIILFRIDKNKTDFKKKNS--
>Western_clawed_frog.locus1011.1
CLKCPINQWPNLNRTICTDKVIIYLSYHEPLGASLALLSVLFCIMTCLVLMIFTKYRNTAVVKANNRDLSYILLVSLKICFLCNLMFIGHPRHVTCILRQTVFGVAFSIVISSVLAKTVTVVIAFNATRPGSKLKNYLGPRVSNSIVIF
```

```
CSLMQVIICTSGLINSPPFPYYNMEDIVGVIVAECHEQS-VGFYCILGFMGLLAALSFIIAFFARTLPDIFNEAKFITFSMLLFCSVWVSFIPAYLSTKGKYVVAVEIFAILSSSSGILGCIFIPKCYIILFRADKNKKNYITKDSSH
>Western_clawed_frog.locus1000.1
CFQCQENQWPNTKGNACVFKEIIYLSYGEPLGMSLALISILFFLLTSLTLLIFTIYRSTAIVKANNRDLSYLLLLSLKTCFLCNFIFMGHPIHMTCILRQTVFGVTFSISLSTILAKTVTVIIAFHATKPRTRLRTFLGPYVAYFIVIF
CSLIQVLISASWLGTYPPFPQYNMVDEVGKIIAECNEGSKLGFYSVLGFMGLLACISFIIAFFARNLPDNFNEAKLITFSMLVFCSVWVSFIPAYMSTKGKYVVATEIFAIIASSMALLVCIFIPKCYIILLRPERNTKVCIRN----
>Western_clawed_frog.locus1001.1
CVKCPENQWSNTNRDTCVYKVIIYLSYEEPLGTSLALLSIIFFLLTCFVSLIFTKYRKTPIVRSNNRDLSYILLFSLKTCFLCNLLFLGHPIRVTCVLRQTVFGVTFSISLSSILAKTITVIIAFSVTKPASKCRNWVGSRVPNSVIFI
CSILQVTICACWLGTSPPFPYYNMEDEVGMIIAQCNEGSVFGFYCVLGFTGILACVCFIIAFLARNLPNSFNEAKFITFSMLVFCSVWVSFIPAYMSTKGKYVVAVEIFAILASSLALLGCIFIPKCYIILVKPECNTKVCLKKV---
>Western_clawed_frog.locus1006.1
CIKCHQTQWHNEKKEKCVNKYIIYLSYGEALGTSMTSISVFLFVLTCLVMGVFTKYQNTPIVKANNRNLSYVLLFSLKMCFLCSLIFIGQPIKLTCMTRQTVFAITFSISLSSILAKTITVVIVFHATKPGSKLNNYIGSKGSVSFIGF
CSLVQVVICACWLGISPPFPQYNMEDEVGKIIAECNEGSLIGFYCVLGYLGVLASVSFIIAFLARDLPDTFNEAKFITFSMLVFCSVWVSFIPAYMSAKGKYVVAAEIFAILASSLALLGCIFIPKCYIILVKPECNTRDFIKKKDKN
>Western_clawed_frog.locus1005.1
CIKCHQTQWHNEKKEKCVNKYIIYLSYGEALGTSMTSVSVFLFVLTCLVMGVFTKYQNTPIVKANNRNLSYVLLFSLKMCFLCSLIFIGQPIKLTCMTRQTVFAITFSISLSSILAKTITVVIVFHATKPGSKLNNYIGSKGSVSFIGF
CSLVQVVICACWLGISPPFPQYNIEDEVGKIIAECNEGSLIGFYCVLGYLGVLASVSFIIAFLARDLPDTFNEAKFITFSMLVFCSVWVSFIPAYMSAKGKYVVAVEIFAILASSLALLGCIFIPKCYIILVKPECNTRDFIKKKDKN
>Western_clawed_frog.locus1004.1
CITCPITQWPNAKKDTCLDKVIIYLSYEEELAMSISFSSIFFFFLTCLVMAVFNKYRTTPIVKANNQNLSYVLLFSLKMCFLCPLIFIGQPIKLACMTRQTVFSIIFSISLSSILAKTITVVIVFHATKPGSKLKNLMGSKVSVSIVIF
CSFVQVVICACWLGISPPFPQYNMEDEVGKIIAECNEGSLIGFYCVLGYLGVLASVSFIIAFLARDLPDTFNEAKFITFSMLVFCSVWVSFIPAYMSAKGKYVVAVEIFAILASSLALLGCIFIPKCYIILVKPECNTRDFVKRGIA-
>Western_clawed_frog.locus1003.1
CITCPITQWPNAKKDTCLDKVIIYLSYEEELGIPIAFSSIFFFFLTCLVMTVFNKYRTTPIVKANNQNLSYVLLFSLKMCFLCPLIFIGQPIKLTCMTRQTVFSITFSISLSSILAKTITVVIVFHATRPGSKLKNLMGSKVSVSIVIF
CSFVQVVICACWLGISPPFPQYNMEDEVGKIIAECNEGSLIGFYCVLGYLGVLASVSFIIAFLARDLPDTFNEAKFITFSMLVFCSVWVSFIPAYMSAKGKYIVAVEIFAILASSLALLGCIFIPKCYVILVKPECNTRDFVKRGIA-
>Western_clawed_frog.locus942.1
CIKCGEDQWPNANKDKCINKVITFLSYEEPLGISLVLISILFFIFTCVVLTIFTLYRNTPIVKANNRDLSYILLFSLMMCFLCSLLFIGHPLRVTCILRQTVFAIAFAISLSSTLAKTVTVIVAFNATRPGNKLRDWMGPSVFNTLVLL
GGLVQVFICAGWVGISPPFPYYNLEDDIVVILAECKEGSLLGFYCVLCYLGLLASISFVIAFLARRLPDAFNEAKLITFSMLVFCSVWISFIPAYLSTKGKYTVAVEIFAILSSGAGLLCCIFLPKCYIILRRPEQNTKVFITQKG--
>Western_clawed_frog.locus890.1
CTKCPENQWPNKQKTSCSPMDITFLSYSDPIGITLTVIAIICLFASFWVLGIFMKYQNTPIVKANNRDLSYLLLISLMCSFLCCLIFIGPPEQVTCFLQQAIFGITFTISVSSLLAKTFIVVVAFNATKPGNNLRKWVGAKIPKYIVIT
FSVIQVCICFLWFVISPPYSYYNKDSQPGIIIVVCNEGSVTAFYTILGYLCLLASVCFVVAFLARKLPDTFNDAKLITFSMLVFFSVWVFFIFTSRSAKGTSTVAVEVFAILGSSSGLLACMFAPKCYIILIKPEQNMKKNLVRDSAA
>Spotted_gar.locus150.1
CFKCQPDYWSNTRRDQCVPKEIEFLSHDETLGISLTTVSVCGGCISATVLAIFIHYRNTPVVRANNSELSFLLLLSLILCFLCSLLFIGQPLHLTCMLRHVVFGISFVLCISCILVKTIVVIMAFRATLPDNNVMKWFGAAQQRGTVFV
FTVIQALICIIWLSTASPVPFKNTQHLNSKIIFECNIGSITGFSFLLGYIGLLASVCFVLAFLARNLPDNFNEAKFITFSMLIFCAVWIAFIPAYVSSPGKYSDAVEIFAILASSFGLLIAIFAPKCYIILLHPEKNTKKALLGKPGL
>Mouse.locus426.1
--------------------------------
MVLASIAICLSALTVFVIGIFMKHRDTPIVKANNRALSYILLITLTFCFLCSLNFIGKPTTASCILQQTTFAVAFTMALATVLAKAITVVLAFKVSFPGRMVRWLMISRGPNYIIPICTLIQLLICGIWMATSPPFIDQDAHTEYGHII
ILCNKGSAVAFHSVLGYLCLMALGSYAMAFLSRNLPDTFNESKFLSFSMLVFFCVWVTFLPVYHSTKGKVMVAMEVFSILASSTALLIFIFGPKCYIILLRPDKNSFNHIRKKTPT
>Mouse.locus430.1
--------------------------------
MAVASIALCLSALTAFVIGIFVKHRDTPIVKANNRALSYILLITLTFCFLCSLNFIGKPTTASCILQQTTFAVAFTMALGTVLAKAITVVLAFKISFPGRMVRWLMISRGPNYIIPISTLIQLIICGIWMATSPPFIDQDAHTEYGHIL
ILCNKGSAVAFHSVLGYLCFMALGSYAMAFLSRNLPDTFNESKFLSFSMLVFFCVWVTFLPVYHSTKGKVMVAMEVFSILASSTALLVFIFGPKCYIILLRPDKNSFNHIRKKTPS
>Mouse.locus421.1
--------------------------------
MALASIALCLCALTAFVIGIFVKHRDTPIVKANNRALSYILLITLTFCFLCSLNFIGKPTTASCILQQTTFAVAFTMALATVLAKAITVVLAFKVSFPGRIVRWLMISRGPNYIIPICTLIQLLICGIWMAASPPFIDQDAHTEYRHII
ILCNKGSAVAFHSVLGYLCFMALGSYAMAFLSRNLPNTFNESKFLSFSMLVFFCVWVTFLPVYHSTKGKVMVAMEVFSILASSTALLAFIFGPKCYIILLRPDKNTFNHIRKKTPS
>Mouse.locus419.1
--------------------------------
MALASIALCLSVLTAFVIGIFVKHRDTPIVKANNRALSYILLITLTFCFLCSLNFIGKPTTVSCILQQTTFAVAFTMALATVLAKAITVVLAFKVSFPGRMIRWLMISRGPNYIIPICTLIQLLICGIWMATSPPFIDQDAHTEYGHII
ILCNKGSSVAFHSVLGYLCFMALGSYAMAFLSRNLPDTFNESKFLSFSMLVFFCVWVTFLPVYHSTKGKVMVAMEVFSILASSTALLAFIFGPKCYIILLRPDKNSFNYIRKKTPS
>Mouse.locus415.1
--------------------------------
MVLVSIALCLSTLTVFVIGIFVKHRETPIVKANNRALSYILLVTLTFCFLCSLNFIGKPTTAACILQQTTFAVAFTVALATVLAKAITVVLAFKVSFPGRMVRWLMISRGPNYIIPICTLIQLLLCGIWMATSPPFIDQDAHTEYGHII
ILCNKGSAVAFHSVLGYLCFMALGSYAMAFLSRNLPDTFNESKFLSFSMLVFFCVWITFLPVYHSTKGKVMVAMEIFSILASSTALLAFIFGPKCYIILLRPDKNSFNHIRK----
>Mouse.locus432.1
--------------------------------
MALASIALCLSVLTAFVIGIFVKHRDTPIVKANNQALSYILLITLTFCFLCSLNFIGKPTTAACILQQTTFAVAFTMALATVLAKAITVVLAFKINFPGRMLRWLMISRGPNYIIPICKVIQLFICGIWMTTSPPFLDQDAHTEYGHII
ILCNKGSAVAFHSVLGYLCFLALGSYAMAFLSRNLPDTFNESKFLSFSMLVFFCVWVTFLPVYHSTKGKAMVAMQVFSILASSTALLAFIFAPKCYIILLKPDKNSFLHIRKETPS
>Mouse.locus436.1
--------------------------------
MALASIALCLSALTAFVIGIFVKHRDTPIVKANNRALSYILLITLTFCFLCSLNFIGQPNTAACILQQTTFAVAFTIALATVLAKAITVVLAFKVSFPGRMLRWLMISRGPNYIIPVCTLIQLLICGIWMATSPPFIDQDPQTEYGHII
ILCNKGSAVAFHSVLGYLCFMALGSYAMAFLSRNLPDTFNESKFLSFSMLVFFCVWVTFLPVYHSTKGKVMVAMEVFSILASSTALLVFIFGPKCYIILLRPDKNSFNHIRKKTLS
>Mouse.locus434.1
CVKCPESHYANTVKSNCFQKSVSFLAYEDPLGLALASIALCLSALTAFVIGIFVKHRDTPIVKANNRALSYVLLINLTFCFLCSLNFIGKPNTAACILQQTTFAVAFTMALATVLAKAIAVVLAFKVSFPGRMVRWLMISRGPNYIIPI
CTLIQLLICGIWMATSPPFIDQDAHTEHGHIIILCSKGSVVAFHSVLGYLCFLAFGSYAMAFLSRNLPDTFNESKFVSFSMLVFFCVWVTFLPVYHSTKGKIMVAMEVFSILASSTALLAFIFGPKCYIILLKPDKNSFNHIKKKTPS
>Mouse.locus440.1
----------------
MCYQKSVSFLAYEDPLGMALASIALCLSALTAFVIGIFVKYRDTPIVKANNRALSYILLITLTFCFLCPLNFIGQPNIAACILQQTTFAVAFTMALATVLAKAITVVLAFKVSFPGRMVRWLMISKGPNYIIPICTLIQLLICGIWMAT
SPPFIDQDAHTEHGHIIILCNKGSAVAFHSVLGYLCLLALGSYTMAFLSRNLPDTFNESKFVSFSMLVFFCVWVTFLPVYHSTKGKIMVAMEVFSILVSSTALLAFIFGPKCYIILLRPDKNSFNHIKKKTSH
>Mouse.locus453.1
--------------------------------
MALASIALCLSALTVFVIGIFVKNRDTPIVKANNRTLSYILLITLTFCFLCSLNFIGQPNTAACILQQTTFAVAFTMALATVLAKAITVVLAFKISFPGRMLRWLMISRGPRYIIPICTLIQLLLCGIWMATSPPFIDQDVNTEDGYII
LLCNKGSAVAFHSVLGYLCFLALGSYTMAFLSRNLPDTFNESKFLSFSMLVFFCVWVTFLPVYHSTKGKVMVAMEVFSILASSTALLAFIFGPKCYIILLRPEKNSFNHIRKKTQS
>Mouse.locus452.1
-MKCPESHYANTEKNYCFPKSVSFLAYEDPLGMALTSIALCLSTLTVFVIGIFVKNRDTPIVKANNQALSYILLITLTFCFLCSLNFIGQPNTAACILQQTTFAVAFTMALATVLAKAITVVLAFKISFPGRMLRWLMISRGLRYIIPI
CTLIQLLVCGIWMATSPPFIDQDTHAEHGYIILLCNKGSAVAFHSVLGYLCILALGSYTMAFLSRNLPDTFNESKFLSFSMLVFFCVWVTFLPVYHSTKGKVMVAMEVFSILASSTALLVFIFGPKCYIILLRPEKNSFTHIRKKTHS
>Mouse.locus410.1
--------------------------------
MALGSIALCLSAFTAFVIGIFVKHRDTPIVKANNRALSYILLITLTFCFLCSLNFIGKPNTVACILQQTTFAIAFTVALATVLAKAITVVLAFKVSFPGRMVRWLVISWGPRYIIPICTLIQLLICGIWMTTSPPFIDQDAHTEHGHII
LLCNKGSAVAFHSVLGYLCILALGSYTMAFFSRNLPDTFNESKFLSFSMLVFFCVWVTFLPVYHSTKGKVMVAMEVFSILASSTALLAFIFGPKCYIILLRPEKNSFNHIKKKTRS
>Mouse.locus413.1
--------------------------------
MALASIALCLSTITVFVIGIFLRHRDTPIVKANNQGLSFILLITLTFCFLCSLNFIGQPNTSACILQQTTFAVAFTMALATVLAKAITVVLAFKVSFPGRMVRWLMISRGPNYIIPICTLIQLLLCGIWMATYPPFIDQDAHTEHGHII
LLCNKGSAVAFHSVLGYLCFLALGSYTMAFLSRNLPDTFNESKFLSFSMLVFFCVWVTFLPVYHSTNGKVLVAMEVFSILASSSALLAFIFGPKCYIILLRPDKNSFNHIRRKQHT
>Mouse.locus409.1
--------------------------------
MALASIALCLSALTVFVIGIFVKHRDTPIVKANNRVLSYILLITLTFCFLCSLNFIGQPNTAACILQQTTFAVAFTVALATVLAKTITVVLAFKVSFPGRIVRWLMISRGPNYIIPICTLIQLLLCGIWMATSPPFIDQDAHSEYGHII
ILCNKGSAVAFHSVLGYLCFLALVSYTMAFLSRNLPDTFNESKFLSFSMLVFFCVWVTFLPVYHSTKGKVMMAMEVFSILASSTALLAFIFGPKCYIILLRPEKNSFSHTRKKTHS
>Mouse.locus448.1
CVKCPEGHYANTERNNCFQKSVIFLAYEDPLGMTLASVALCLSALTVFVIGIFVKHRDTPIVKANNRALSYILLLTLTFCFLCSLNFIGQPNTATCILQQTTFAVTFTMALATVLAKAITVVLAFKVSFPGKIVRWLMLSRGPNYIIPI
CTLIQLLICGIWMTTSPPFIDQDAHTEHGHIIILCNKGSAVAFHSVLGYLCFLALGSYTMAFLSRNLPDTFNESKYLSFSMLVFFCVWVTFLPVFHSTKGKIMVSMEVFSILASSIALLAFIFGPKCYIILLKPENNSFTHVRKKTYS
>Mouse.locus462.1
--------------------------------
MALASIALCLSALTAFVIGIFVKHKDTPIVKANNQALSYTLLITLKFCFLCSLNFIGQPNTVACILQQTTFAVAFTMALATVLAKAITVVLAFKVSFPGRMVRWLMISRGPNYIIPICTLIQLLLCGIWMAISPPYIDQDAHIEHGHII
ILCNKGSAVAFHSVLGYLCFLALGSYTMAFLSRNLPDTFNESKFISLSMLVFFCVWITFLPVYHSTKGKVMVAMEVFSILASSTALLAFIFGPKCYIILLRPEKNSFHHIREKTHS
>Mouse.locus465.1
CIKCPESHYASIEKNRCFQKALSFLSYKDPLGVTLANISLCLSALTAFVIGVFVKYRDTPIVKANNRGLSYTLLITITFCLLCSLNFIGQPNTASCILQQTIFGVSFTVALATVLAKAITVVIAFKATFPGRMVRWLMTSRAPNYIIPI
CTLIQLLLCGIWMATSPPFIDQDVHAEHGQILLFCNKGSSVAFHCVLGYLCSLALGGYTMAFLSRNLPDTFNESKFLSFSMLVFFCVWVTFLPVYHSTKGKVMVAMEVFSILASSAALLGFIFAPKCYIILLRPEKNSFHHIRDKTHS
>Mouse.locus467.1
CVKCPESHYANTEKNHCFQKTVSFLAYEEPLGITLASITLCLSALTAFVIGVFVKYRDTPIVKANNRGLSYTLLITITFCLLCSLNFIGQPNTASCILQQNIFGVAFTVALATVLAKAITVVIAFKATFPGRMVRWLMTSRAPNYIIPI
CTLIQLLLCGIWMATSPPFIDQDVHAEHGQILLFCNKGSSVAFHCVLGYLCSLALGGYTMAFLSRNLPDTFNESKFLSFSMLVFFCVWVTFLPVYHSTKGKVMVAMEVFSILASSAALLGFIFAPKCYIILFRPDRNSFHHIRDKTHS
>Mouse.locus458.1
CVKCPESHYSNTEKNHCFQKTVIFLAYEDPLGMTLACIALCLSALTAFVIGIFVKYRDTPIVKANNRALSYTLLITLTFCFLCSLNFIGQPNLTTCILQQTTFGVAFTMALATVLAKAITVVIAFRATFPGRMVRWLMMSRVPNYIIPI
CTLIQLLLCGIWMAISPPFIDNDVHTEHGYIIIFCNKGSSFAFHCVLGYLCSLAFGGYTMAFLSRNLPGTFNESKFLSFSMLVFFCVWVTFLSVYHSTKGKFMVAMEVFAILASSSALLGFIFGPKCYIILIRPDRNSFHHIRDKTHS
>Mouse.locus456.1
-MKCPDSHYANTEKNYCLKKAVSFLAYEDLLGMILASIALCLSALTVFVIGIFVKNRHTPIVKANNQALSYILLITLTFCFLCSLTFIGQPNKNTCILQQTTFGVAFTAALATVLAKAITVVIAFKATFPGRIVRWLMISRAPNYIIPI
CTLIQVGFCGTWMATSPPFIDQDAHTEHGHIIIFCNKGSSVAFHCVLGYLCFLALCGYTVAFLSRTLPDTFNESKFLSFSLLVFFCVWVTFLPVYHSTKGKFMVATEIFSILASSTALLGFIFGPKCYIVLLRPDENTFNYIRNKIHS
>Mouse.locus231.1
CMKCPESHYANSEKSHCLQKSVSFLAYDEPLGMALIITALCFSLLIAAVLVVFVKHRDTPIVKANNRTLSYILLLTLFICLLSSFLFIGQPNTTTCILQQTAFGILFTVALSTVLAKAITVVIAFKATVPARMVRWLMVSRAPNFIIPI
CTLIQLIICGIWLVTSPPFIDQDTQTEHGHIIIVCNMGSTVAFHTVLGYLCSLALGSYTMAFLSRNLPDTFNEAKFLSFSMQVFFCVWITFLPVYHSSKGKVMVVMEVFSILASSVALFILIFAPKCYIILLRPEKNSCHNVRHKKHS
>Mouse.locus229.1
CVKCPESHYANSEKSHCLQKSVSFLAYDEPLGMALTITALCFSLLIAAVLVVFVKHRDTPIVKANNRTLSYILLLTLFICLLSSFLFIGQPNTTTCILQQTAFGILFTVALSTVLAKAITVVIAFKATVPARMVRWLMVSRAPNFIIPI
CTLIQLIICGIWLVTSPPFIDQDTQVEHGHIIIVCNMGSTVAFHTVLGYLCSLALGSYTMAFLSRNLPDTFNEAKFLSFSMQVFFCVWITFLPVYHSTKGKIMVAMEVFSILASSVALFGLIFAPKCYIILLRPEKNSCHNVRHKKHS
>Mouse.locus227.1
CLKCPESHYANSEKNHCLQKSVSFLAYKEPLGMALTITALCFSLLIAAVLVVFVKHRDTPIVKANNRTLSYILLLTLFICLLSSFLFIGQPNTTTCILQQTAFGILFTVALSTVLAKAITVVIAFKATVPAQIVRWLMVSRAPNFIIPI
CTLIQLIICGIWLVTSLPFIDQDTQVEHGHIIIVYNMGSTVAFHTVLGYLCSLALGSYTMAFLSRNLPDTFNEAKFLSFSMQVFFCVWITFLPVYHSTKGKVMVAMEVFSILTSNTALLGFIFAPKCYIILFRPAKDSF-GFRQKTIQ
>Mouse.locus232.1
CVKCPESHYANSEKNYCLQKSVSFLAYEEPLGMALTTTAMCFSLLTVVVLVVFVKHRDTPIVKANNRTLSYILLLTLLICLFSSLLFIGQPNTTTCILQQTAFGILFTVALSTVLAKAITVVIAFKATVPARMVRWLMISRAPNFIIPI
CTLIQLIICGIWVVTSPPFIDQDTQAEHGHIIIVCNMGSTVAFHTVLGYLCSLALGSYTMAFLSRNLPDTFNEAKFLSFSMQVFFCVWITFLPVYHSTKGKIMMAMEVFSILASSIALLGLIFAPKCYIILLRPEKNSCHTVRHKKHS
>Mouse.locus243.1
CMKCPESHYANSEKNHCLQKSVSFLAYEEPLGMALTITALCFSLLTAVVLVVFVKHGDTPLVKANNRTLSYILLLTLFISFLSSLLFIGQPNTTTCILQQTAFGILFTVALSTVLAKAITVVIAFKATVPARMVRWLMVSRAPNFIIPI
CTLIQLIICGIWVVTSPPFIDQDTHAEHGHIIIVCNMGSTVAFHTVLGYLCSLALGSYTMAFLSRNLPDTFNEAKFLSFSMQVFFCVWITFLPVYHSTKGKVMVAMEIFCILASSIALLGLNFAPKCYIILLRPEKISCLGIRYKKNS
>Mouse.locus239.1
--------------------------------
```

```
MALTITSLCFSLLTAVVLVVFVKHRDTPIVKANNRTLSYILLLTLFISFLSSLLFVGQPNTTTCILQQTAFGILFTVSLSTVLAKAITVVIAFKAIVPARMVRWLMVSRAPNFIIPICTLIQLIICGIWILTSPPFIDQDTQAEHGHII
IVCNMGSTVAFHTVLGYLCSLALGSYTMAFLSRNLPDTFNEAKFLSFSMQVFFCVWITFLPVYHSTKGKVMVAMEVFSILASSIALLGLNFAPKCYIILLRPEKISCIDIRHTKYT
>Mouse.locus234.1
CVKCPESHYANSERNHCLQKSVSFLAYEEPLGMALTITAICFSLLTALVLAVFVKNRDTPIVKANNRALSYILLLTLFICLLSSLLFIGQPNTITCILQQTAFGILFTVALSTVLAKAITVVIAFKATVPARMMRWLMISRAPNFIILI
CTLIQVIICGIWVVTSPPFIDQDTQAEHGHIIIVCNMGSTVAFHTVLGYLCSLALGSYTMAFLSRNLPDTFNEAKFLSFSIQVFFCVWITFLPVYHSTKGKVMVAMEVFSILASSIALLGLIFGPKCYIILLRPEKISCLGIRHKKHS
>Mouse.locus237.1
CVQCPESHYANSEKNHCLQKSVSFLAYEEPLGMALTIIALCFTLLTAVVLVVFVKHRDTPIVKANNRTLSYILLLTLFISFLSSLLFIGQPNTTTCILQQTAVGILFTVALSTVLAKAITVVIAFKATVPARMVRWLMVSRAPNFIIPI
CTLIQLIICGIWLVTSPPFIDLDTHAEHGHIIIVCNMGSTVAFHTVLGYLCSLALGSYTMAFLSRNLPDTFNEAKFLSFSMQIFFCVWITFLPVYHSTKGKVMVAMEVFCILASNIALLGLIFTPKCYIILLRPEKNSYLGVRHKII-
>Mouse.locus244.1
CVQCPESHYANSEKNHCLQKSVSFLAYEEPLGMGLTITALDFSVLTAVVLVVFVKHRDTPIVKANNRVLSYTLLLTLIICFLSCLLYIGQPNTATCILQQTAFGTLFTVALSTVLAKAIVVVTAFKVTSPARMVRWLIVSGAPNSIIPI
CTLIQLIICGIWLATSPPFIDQNAHVEHGHIIIMCNKGSAVAFHCVLGYLCFLALGSYSMAFLSRNLPDTFNEAKYLSFSMQVFFCVWITFLPVYHSTKGKVMVAMEVFSVLASSIALLALIFAPKCYIILLRPDKNSRLDIRHKIHS
>Mouse.locus246.1
CVQCPESHYANSEKNHCLQKSVSFLAYEEPLGMALTITALDFSVLTAVVLVVFVKHRDTPIVKANNRVLSYTLLLTLIICFLSCLLYIGQPNTATCILQQTAFGTLFTVALSTVLAKAIVVVTAFKVTSPARMVKWLIVIGAPNLIIPI
CTLIQLIICGIWLATSPPFIDQNAHVEHGHIIIMCNKGSAVAFHCVLGYLCSLALGSYTMAFLSRNLPDTFNEAKCLSFSMQVFFCVWITFLPVYHSTKGKVMVAMEVLSVLASSITLLVLIFAPKCYIILLRPDKNSCLDIRHKIHS
>Mouse.locus224.1
CVQCPESHYANSEKNHCLQKSVSFLAYEEPLGIALTITALDFSVLTALVLVVFVKHRDTPIVKANNRVLSYILLLTLIICFLSCLLYIGQPNTATCILQQTAFGTLFTVALSTVLAKAIVVVTAFKVTSPSRMVRWLIVSRAPNLIIPI
CTLIQLIICGIWLATFPPFIDQNAHVEHGHIIIMCNKGSAVAFHCVLGYLCSLALGSYTMAFLSRNLPDTFNEAKCLSFSMQVFFCVWITFLPVYHSTKGKVMVAMEVFSILASSTSFLALIFVPKCYIILLRPDKNSCLDIRNKIHS
>Mouse.locus403.1
CVKCPESHYANSEKNHCLQKSVSFLAYEDSLGIALTTTALCLSVLTAVVLVVFVKHRHTPIVKANNRTLSYTLLLTLIMCFLSSLLFIGQPNTTTCILQQTAFGIFFTVALSTVLAKAITVVIAFKVTVPNRKVRRLMISRAPNFIIPI
CTLVQLLFCGIWLITSPPFIDQNAHAEHGNIIILCNKGSAVAFHCVLGYLCFLALVSYTLAFFSRNLPDTFNEAKFLSFSMQVFFCVWITFLPVYHSTKGKVMVAMEVFSMLASSTALLSLIFGPKCYIILLRPDKNVCLDIRHKSHS
>Mouse.locus90.1
-----------------------------------
MALGFFVLTAGVLGVFVKYHHTPIVKANNQVLTYILLVTLAFCFLCPLLFIGYPNTATCILQQSTFSLLFTVALSTVLAKTMTVVLAFKVTVPRRMVRWLMISKALNFIIPICTFIQFVLTGIWLSTFPPFIDSYAHSEHGHIIILCNK
GSVVAFHCVLGYLCCLALGSYILAYHSMKLPDTFNEAKFLTFSMLMFFSVWVTFLPVYHSTNGKVMVAMEAFSVFVSSAALLGCIFVPKYYIILLRPERNSLHCIRQNAHS
>Reedfish.locus40408.1
----------------------------
MGITLTFISIFGACVTIAVAIVFTIFRQTPIVRANNMELSFLLLLFLVLCFLCPLAFIGEPTNISCFVRHTLFGISFALCISCILGKTVVVLMAFRATLPGNNVMKWFGLAQQRASVFICTSIQIVICIIWLTTSPPYAIPNSKFHNSK
IILECSVGSAFGFWCVLSYIGFLASMCFVIAFLARKLPDNFNEAKFITFSMLIFFAVWITFIPAYISTPGKYTVAVELFAILASAFGLLFCIFAPKCYIILFKPEKNTKKHMMGKGNV
>Reedfish.locus40406.1
CLKCSLEKWSNEAKDQCIPKSIEFLSYQDLLGSTLAFISIFGACVTTAVAAVFTIFRKTPIVRANNMELSFLLLLFLVLCFLCPLAFIGEPTTISCFMRHTLFGISFALCISCILGKTVVVLMAFRATLPGSNVMKWFGLAQQRASVFL
CTSIQIVICIVWLTTSPPYAIQNTKYQSAKIILECSVGSNVGFWCVLGYIGFLASMCFIIAFLARKLPDNFNEAKFITFSMLIFFAVWITFIPAYVSTSGKYTVAVELFAILASAFGLLVCIFAPKCYIILLKPEKNTKKYMMGRNSH
>Japanese_eel.locus260.1
CYRCPVDFWSNQLRDRCVPKEIEFLSFQEPLGISLTTISVLGACICAVTLSVFARHSNTPVVKANNSELSFLLLLSLTLCFLCSLLFIGQPQWWTCQLRHAAFGISFALSISCILVKTIVVVMAFKAARPGGNTMKWFGVQQQRGTVLA
FTIIQAVICVAWLTAASPVPYKNTRLQNSKIIYECAVGSIAGFSALLGYIGLLAAISLLLAFLARNLPDNFNEAKFITFSMLIFCAVWIAFVPAYVSSPGKYSDAVEIFAILASSFGLLGAIFAPKCYIILLHPEKNTKKALMGRDAP
>Cloudy_catshark.locus91.1
CLKCPLEYWSNQQKDRCVLKKIEFLSFGEILGYVLVALALVGVGFTLATAAIFFQYRETPIVRANNSELSFLLLLGLMLCFLCSLTFIGEPTGWSCMLRRTAFGIVFVLSISCILGKTILVVIAFKATLPSNNLMSWFGPMQQRLGVFV
LTFLQALICTIWLSVSPPYPLKNMTYYRDVIILECDVGSLKAFYFVSCYIGLLSTVCFMLAFLARKLPDNFNDAKYITFSMLIFCAVWITFIPAYVSSPGKYTVAVEVFAIWASSFGLLVCIFAPKCYIILLKPEDNTKKNMMGK---
>Japanese_eel.locus82.1
------------------------------
MALAAMAILGALSTAAVAAIFLWHRRTPIVRANNSELSFLLLLSLTCCFLCVLTFLGRPSHWGCRLRRTSFGISFALCLSCLLSKTLVVLMAFQATLPGNSVARWFGPTQQRLGVLVCVAVQVGICGVWLARAPPYPIKNTWLYRDRII
LECHLGSVVLFGCVLGYIGCLAGFCCILAFLARKLPDNFNEAKFITFSMLIFCAVWITFIPAYVSSPGKFTVAVEIFAILASSFGVLLCIFAPKTFIIMFQPEKNSKRNLLSQRSA
>Western_clawed_frog.locus103.1
CIKCHIYEWPNEEKTKCIEKQTEFLSYDDPLTLVFIVLSFVSILLVTVILGIFISFRDTPVVKANNRNLSFILLVSIKLSFLSVFLFLGRPVDITCMLRQTSFGITFSIAMSSVLAKTIMVSIAFKASKPGSPWRKLLKTHLTHGIVVI
CSFIQTLINVIWLVLSPPFVELNILSDPGKIIIQCNEGSAIGFYIVLSYMGLLASVSFIVAFLARSLPDSFNEAKYITFSMLLFCSVWITMIPAYLSTKGKYMVAVEIFAIISSSCGLLFCIFLPKCYIIMLKPEMNTKQYLLRNN--
>Western_clawed_frog.locus102.1
CIKCHIYEWPNEEKTMCIEKQTEFLSYDDSLTLVFIVLSLVSVLLVTVILGIFISFRDTPVVRANNRNLSFILLIAIKLSFLSVFLFLGRPVDITCMLRQTCFGITFSIAVSCVLAKTMIVCIAFKASKPGSPWRKLLKIHLAHGIVVI
CSFIQTLINVIWLALSPPFMELNILSDPGKIIIQCNEGSVVAFYIVLSYMGLLASVSFIVAFLARSLPDSFNEAKYITFSMLLFCSVWITMIPAYLSTKGKYMVAVEIFAIISSSCGLLFCIFLPKFYIIMLKPEMNTKQYLLGNNK-
>Western_clawed_frog.locus101.1
CIKCPIYEWPNEEKTMCIEKQTEFLSYDDSLTLVFIVLSLVSVLLVTVILGIFISFRDTPVVRANNRDLSFILLVSIKLSFLSVFLFLGRPVDITCMLRQTSFGITFSIAMASVLAKTIMVCIAFKASKPGSPWRKFLKIHLAHCIVVI
CSFIQTLISVIWLALSPPFVELNILSDPRKIIIQCNEGSAVAFYIVLSYMGLLASVSFIVAFLARSLPDSFNEAKYITFSMLLFCSVWITMIPAYLSTKGKYMVAVEIFAIISSSCGLLFCIFLPKCYIIMLKPEMNTKQYLLGNNK-
>Western_clawed_frog.locus1018.1
-----------
MKERCLPKTIQFLSYDEPLGSSLACISVLFCLITFSAFCLFIAKRKTPIVKANNRELSYLLLISLMFGFLCSLAFIGRPNLKTCMIRQVLFAVIFSFCISVILAKTVTVIMIFSATNPDSKLKRLVAMRIPVYIVPVCTMGQIILCIVW
LARAAPFAEFNMVAEIGIIVIECNEGSRVLFACVLGYMGLLAAISLLVACLARKLPDTFNETKFITFSMLVFASVWLTFIPAYLSTKGKQTVAVEIFAILSSMAGLLVCIFIPKCYIILLHPEMNSKQYITGRNTR
>Western_clawed_frog.locus1020.1
CLKCPEDQWPDSMKERCLPKIIQFLSYDEPLGSSLACISVLFCLITFSAFCLFIAKRKTPIVKANNRELSYLLLISLMFGFLCSLAFIGRPNLKTCMIRQVLFAVIFSFCISVILAKTVTVIMIFSATNPDSKLKRLVAMRIPVYIVPV
CTMGQIILCIVWLARAAPFAEFNMAAEIGIIVIECNEGSRVLFACVLGYMGLLAAISLLVACLARKLPDTFNETKFITFSMLVFASVWLTFIPAYLSTKGKQTVAVEIFAILSSMAGLLVCIFIPKCYIILLHPEMNSKQYITGRNTR
>Anole_lizard.locus211.1
--------------------------------------------------------
MVKANNRDLTYMLLVSLLLSFLSSFLFIGQPRMATCLLRQTAFSTIFSVAVSSLLAKTITVVVAFMATKPGSRMRKWLGKSLANSIAISCSGVQVGICMVWLGVSPPFPDSDLNSQPGQIVLQCNEGSMVMFYGALGYMGFLAAICFTV
AFLARKLPGAFNEAKLITFSMLVFCSVWISFVPTYFSTKGKYMVAVQLFSILSSNLGLLGCIFIPKCYIILFRPHLNTKNFLVLKTQD
>Western_clawed_frog.locus444.1
-------
MWPNSARDRCLSKPIEFLSYEEPLAIILASTGVISSLIPISIFRLFIRYKSTPIVRANNYSVSCILLVSLSFCFLCALAFIGYPQPEKCLLRQAAFGLVFTLCVSCILAKTVIVVFAFMATKPGSRLKKWTTPRVPYMIITICTFIQLT
LCIFWLSISPPFPQYNIEAKPGIIVVECNENSQFAFWCMLGYLGFLASVSFTVAFLARRLPDSYNEAKFITFSMLAFLSVWVSFIPASLSAQGKYTVAMEIFAILTSTWALVFCMFLPKCFVILFRPNMNTKEKLMGKDRS
>Japanese_eel.locus272.1
CYRCPVDFWSNQLRDRCVPKEIEFLSFHEPLGISLTTISVLGACICTVTLSVFARHRNTPVVKANNSELSFLLLLSLTLCFLCSLLFIGQPQRWTCQLRHAAFGISFALSISCILVKTIVVVMAFKAARPGGNTMKWFGVQQQRGTVLA
FTIIQAVICVAWLTAASPVPYKNTRLQNSKIIYQCAVGSIAGFSALLGYIGLLAAISFLLAFLARNLPDNFNEAKFITFSMLIFCAVWISFVPAYVSSPGKYSDAVEIFAILASSFGLLGAIFAPKCFIILLHPEKNTKKALMGRDAP
>Western_clawed_frog.locus177.1
CLKCEDTEWPNQEKTLCIEKQIEFLSYGDPLTLISIISSVILFIIAAVILGIFISFRDTPVVRANNHTLSFILLVSIKLSFLSVFLFLGRPVDITCMLWQTSFGITFSIAVSCVLAKTLMVSIAFKATKPGSPWRKWVGVKLANGLVFI
CSLIQFLISVIWLVIAPPYVEHNTHSEPGKIIIQCNEGSAIGFYIVLSYMGLLASVSFIVAFLARSLPDSFNEAKYITFSMLLFCSVWITMIPAYLSTKGKYMVAVEIFAIISSSCGLLFCIFLPKCYIIIFKPE---RQWLYQAMEL
>Anole_lizard.locus183.1
CTKCPLDQHPNKDQNRCVPKNTTFLSYEESLGNILVSIALLLSITTCTVFGVFITNQETPIVKANNRDLSYVLLLSLLLSFLSSFLFIGQPSEATCLLQQIVFNVIFSVAVSSLLAKTITVVLAFLATSPGNRARRWLGKTLANAIVLS
CSSVQVLICSLWLGLSPPFPDSDMHSQPGEILLKCNPGSAAMFYAALGYLGFLAGVCFTVAFLARKLPGSFNEAQLITFSMLVFCSVWVSFVPTYLSTKGKYLVAVQVFSILASGAGLLVGIFLPKSYIILLRPDLNTKEHLAIKSKG
>Anole_lizard.locus184.1
CNKCPPDQHPNKDQSQCIPKDITFLSYEESLGNILVSIALLLSITTCIVLGVFITNQETPIVKANNRDLSYVLLVSLLLSFLSSFLFIGRPSEATCLLQQIAFNVIFSVAVSSLLAKTITVVLAFLATSPGNRARRWLGKTLANAIVLS
CSSVQVLICSLWLGLSPPFPDSDMHSQPGEILLKCNPGSPAMFYAALGYLGFLAGVCFTVAFLARKLPGSFNEAQLITFSMLVFCSVWVSFVPTYLSTKGKYLVAVQVFSILASGAGLLVGIFLPKSYIILLRPDLNTKEHLKN----
>Mouse.locus354.1
CVRCPDDKYANLEQSHCLQRTVSFLAYEDPLGMVLGSMALSSSAITILVLVTFMKYKDTPIVKANNCILSYILLISLVFCFLCSLLFIEHPNQVTCILQQTTFGLFFTVAVSTVLAKTITVVMAFKLTTPGKRMRG-
MMTGAPKLVIPICTLIQLVLCGIWLLTSPPFIDRDIQSEHGKTVIFCNKGSVIAFHVVLGYLGSLALGSFTVAFLARNLPDRFNEAKFLTFSMLVFCSVWITFLPVYHSTRGKIMVVVEVFSILASSAGLLGCIFVPKCYVILVRPDSN
FIQKYKGKLLY
>Reedfish.locus40372.1
CEKCQPYFWSNPERTQCVPKEVEFLSYGDAMGIALTAVSLSGAALSLAVLAVFIYYRETPVVKANNSELSFLLLISLILCFLCSLCFIGKLKNLNCFLRHVVFGISFVLCISCILVKTIIVIMAFKTTLRGKNIMKWFRSLQQRGTVFL
FTLIQALICIIWVVVSPPALSRNTKYQNAKIILECNIASITGFSCLMRYIGFLASICFVLAFLARKLPDNFNEAKFITFSMIIFCTVWLTFIPAYISTPGKYTVAVEIFTILASSF------------------------------
>Reedfish.locus40371.1
----------------------------
MGITLTAVSLSGAALSLAVLSVFIYYRETPVVKANNSELSFLLLISLILCFLCSLCFIGKPNNLNCILRHVVFGISFVLCISCILVKTIVVIMAFKATLPGKNIMKWFRSLQQRGTVFLFTLIQALICIIWVVISPPAPSRNTKYQNSK
IILECDIGSITGFSCLMGYIGFLASVCFVLAFLARKLPDNFNEAKFITFSMIIFCTVWLTFIPAYISTPGKYTVAVEIFTILASSFGIITTIFFPKCYIILIRPERNTKKFLMARA--
>Reedfish.locus40368.1
CDKCQPDSWSNPERTQCVPKEVEFLSHEDALGIALTAVSLSGAALSLAVLSVFIYYRETPVVKANNSELSFLLLISLILCFLCSLCFIGKPTYLTCILRHVVFGISFVLCISCILVKTIVVIMAFKATLPGKNIMKWFRSLQQRGTVFL
FTLIQALICIIWVVISPPAPSRNSKYQNSKIILECDIGSITGFSCLMGYIGFLASVCFVLAFLARKLPDNFNEAKFITFSMIIFCTVWLTFIPAYISTPGKYTVAVEIFTILASSFGIITTIFFPKCYIILIRPETNTKKFLMARADP
>Reedfish.locus27689.1
-MKCPLDYRSNKEKDACVLKDIEFLSYAEIISLVLEAFSFFGASTTTAVMVVFYKHKETPIVKANNSELSFLLLFALTLCFLCSLAFIGEPTQWSCMLRHTAFGISFVLCISCVLGKTIVVLMAFRATLPGNNIMKWFGPTQQRLTVCT
FTAIQVLICTLWLSISPPSPIRNMKYFTEKIILECDIGSGIAFYSVLGYIGFLSVLCFVLAFLARKLPDNFNEAKFITFSMLTFCAVWVTFIPAYISSPGKYTVAVEIFAILASSFGLLSCIFVPKCYIILFRPENNSKKHLMGKMAS
>Coelacanth.locus412.1
CLKCPDDQWSNEQQNECIPKTVEFLVYEDALGAALAIVSIFCALASTAVLCVFIKFRNTPIVKANNQELSYLLLIALLLCFLNSLVFIGQPKAWSCMLCHVAFGITFALSVSCVLAKTIMVVIAFNATKPNSNLKKGVGPKLANSIVLV
CTLIQIIIFIIWLASAPPFPTENMKSQTGKIVIECNEGSTTAFWCVLGYMGLLAIVSFIVAFLARNLPDSFNEAKFITFNMLVFVSVWLAFIPAYLSTRGKYMVAVEIFAILASGSWLLACIFFPKCYIILLRPEMNTRKSLIGKVKL
>Spotted_gar.locus110.1
----------------------------
MGILLTAFSSSGACFTTGVAFIFFRNRDTPIVKANNSELSFLLLFSLTLCFLCSLTFIGQPSDWSCMLRHTAFGITFVLCISCVLGKTIVVLMAFRATLPGSNIMKWFGPTQQRLSVLAFTLIQVLICVLWLTISPPFPNRNMKYYADR
IILECDLGSAGAFWAVLGYIGLLSAMCFVLAFLARNLPDNFNEAKFITFSMLIFCAVWITFIPAYISSPGKFTVAVEIFAILASSFGLLVCIFVPKCHIILLKPDLNTRKHLMGKMPS
>Spotted_gar.locus113.1
CVQCPLEYWSNEKRDECVLKIIEFLSFGEIMVILLIIFSVIGACLTIGIALIFFKNKDTPIVKANNSELSFLLLFSLTLCFLCSLTFIGQPSDWSCMLRHTAFGITFVLCISCVLGKTIVVLMAFRATLPGNNIMKWFGPTQQRLSVLG
FTLVQVLICVLWLTTSPPFPSKNMKYYADRIILECDLGSTGAFWAVLGYIGLLSAMCFVLAFLARKLPDNFNEAKFITFSMLIFCAVWITFIPAYISSPGKYTVAVEIFAILASSFGLLFCIFAPKCYIIILKPEMNTKKHLMGKMIA
>Spotted_gar.locus105.1
CIQCPLEYWSNDKRDKCALKITEFLSYGEIMGIFLMAFSLFGAFLTIGISLIFFKYKDTPIVKANNSELSFLLLFSLTLCFLCSLTFIGQPSDWSCMLRHTAFGITFVLCISCVLGKTIVVLMAFRATLPGNNIMKWFGPTQQRLSVFT
FTLIQVLICVIWLTTAPPFPNKNMKSYADRIILECDLGSAGAFCAVLGYIGFLSAMCFVLAFLARKLPDNFNEAKFITFSMLIFCAVWITFIPAYISTPGKYTVAVEIFAILASSFGLLLCIFTPKCYIILLKPERNTKKLLIGKMSS
>Spotted_gar.locus128.1
CIQCPLEYWSNEKQDKCILKTIEFLSFGEIMGILLIAFSFSGASLTLCIAFIFIKYKDSPIVKANNSELSFLLLFSLTLCFLCSLTFIGQPSDWSCMLRHTAFGITFVLCISCVLGKTIVVLMAFRATLPGSNIMKWFGPKQQRLSVLG
FTLVQVLICILWLTISRPFPHINTQYYKDKIILECDIGSVMGFCAVLGYIGFLSAMCFVLAFLARKLPDNFNEAKFITFSMLIFCAVWITFIPAYVSFPGKFTVAVEIFAILSSSFSLLFCIFAPKCYIILLKPELNTKKHMMGSMA-
>Cloudy_catshark.locus78.1
-----------------------------------------
MALNIAVIFFRYRETPLVKANNSELSFLLLLALMCCFLCSVTFIGEPSDWSCMLRHTAFGVTFVLCISCVLVKTILVVMAFKATHPSSNRMKWFGQKQQRLSVFSLTFVQGLICALWLIIAPPFPRRNTSHYREVIILECDAGSSAAFY
SASGYIALLSCLCFALAFLARKLPSNFNEAKCLTFSMLIFCAVWITFIPASVSSPGKYTVAVEVFAILASSYGLLLCIYAPKCYIIMITPEKNTKKNLMSKVIE
>Reedfish.locus40339.1
CFTCPPEYWSNHERSECIQKEIEFLSYDDAMGITLTATSALGACLSLSVLAIFIHHRHTPVVKANNSELSFLLLVSLTLCFLCVLCFIGKPSDITCTIRHVVFGLSFAVCISCILVKTIVVIMAFKATLPGNNMVKWFGTSQQRGTVFF
TAFIQCIICLVWLTTAPPVPNKNTKYQNSKIIFECHVGSLIGFSCLLGYIGLLACICFLLAFLARNLPDTFNEAKFITFSMLIFCAVWITFVPAYISSPGKHTVAVEIFAILASSFGLLFSIFAPKCYIILLRPELNTKKALMGRAGQ
>marker.zebrafish_16_1_F
```

```
---
CPDEFWSSPKKNQCVPKDVEFLTYGDPLGISLTTASLLGSCICSAVVVIFAHHRHTPVVRANNSELSFLLLVSLKLCFLCVLLFIGQPQLWTCRLRHAVFGISFVLCISSILVKTMVVIAVFKSSRPGKSAMKWFGSHQQRCTVLVLTA
LQVVICAVWLTNASPKPYKNNQYTSSKIVYECTIGSVVGFAMLLGYIGILAAVSFLLAFLARNLPDHFNEAKFITFSMLIFCAVWIAFVPAYVSSPGKYAVAVEIFAILASSFGLLAAIFAPKCYIIILHPERNTKKAIMGRA--
>Reedfish.locus40475.1
------------------------------
MGLLLVVLSLFGAFLTLLITLVFYLNRETPIVRANNSELSFLLLFSLTLCFLCSLTFIGEPSEWSCMLRHTAFGITFVMCISCVLGKTIVVLMAFRATIPGNNIMKWFGVMQQRLSVCSFTLIQILICLLWLLTSPPFPNKIMRHSTET
IILECNLGSPTAFYAVLGYIGILACLCFILAFLARKLPDNFNEAKFITFSILIFCAVWITFIPAYISSPGKYTVAVEIFAILASSFGLLFCIFIPKCYIILLRPERNTKKHLMGKIPS
>Reedfish.locus40481.1
CVKCPLEYKANKQKDQCILKEIEFLSFEENMGILLVTLALLGASLTIAIGVVFYRKRDTPIVKANNSELSFLLLFSLTLCFLCSLTFIGQPTEWSCMLRHTAFGITFVLCISCVLGKTIVVLMAFRATLPGNNVMKWFGATQQRLSVCM
FTLVQVIICTLWLMVSPPFPNKNMAHFTEIIILECDLGSKTAFYAVLGYIGFLAALCFVLAFLARKLPDNFNEAKFITFSILIFCAVWITFIPAYISSPGKYTVAVEIFAILASSFGLLFCIFLPKCYIILLKPEKNTKKNLMGKMKP
>Reedfish.locus40483.1
------------------------------
MGILLVTLALLGASLTIAIGFVFYLKRDTPIVKANNSELSFLLLFSLTLCFLCSLTFIGQPTEWSCMLRHTAFGITFVLCISCVLGKTIVVLMAFRATLPGSNVMKWFGVTQQRLSVCMFTLIQVIICTLWLLVSPPFPNKNMAHIAEI
IILECDLGSKTAFYAVLGYIGFLAALCFILAFLARKLPDNFNEAKFITFSILIFCAVWITFIPAYISSPGKYTVAVEIFAILASSFGLLSCIFLPKCYIILLKPEKNTKKNLMGKMKS
>Reedfish.locus40479.1
CLKCSLEYKANKQKDQCILKDIEFLSFEENMGILLVSLALLGASLTISIAFVFYLKRDTPIVKANNSELSFLLLFSLTLCFLCSLTFIGQPTEWSCMLRHTAFGITFVLCISCVLGKTIVVLMAFRATLPGNNVMKWFGAMQQRLSVCM
FTFIQVIICTLWLMVSPPYPNKN-THFTEILILECDVGSVTAFYAVLGYIGFLAALCFVLAFLARTLPDNFNEAKFITFSILIFCAVWITFIPAYISSPGKYTVAVEIFAILASSFALLFCIFLPKCYIILFKPEMNTKKNLMGKVKS
>Reedfish.locus40471.1
------------------------------
MGILLVALALLGASVTIAIAFVFYFRRDTPIVKANNSELSFLLLFSLTLCFLCSLTFIGQPTDWSCMLRHTAFGITFVLCISCVLGKTIVVLMAFRATLPGSNVMKWFGATQQRLSVCTLTLIQVVICLFWILISPPFPNKNFKHYSEI
IILECDLGSKTAFYTVLGYIGLLAALCFVLAFLARKLPDNFNEAKFITFSILIFCAVWITFIPAYISSPGKYTVAVEIFAILASSFGLVFCIFIPKCFVILFKPEKNTKKHLMGKAAS
>Reedfish.locus40478.1
------------------------------
MGILLVTLALLGAFLTIAIAFVFYFRRDTPIVRANNSELSFLLLFSLTLCFLCSLTFIGRPTEWSCMLRHTAFGITFVLCISCVLGKTIVVLMAFRATLPGNNVMKWFGATQQRLSVCTLTFIQVVICLFWLLISPPFPNRNLKHYSEI
IILECDLGSTTAFYAVLGYIGLLAALCFVLAFLARKLPDNFNEAKFITFSILIFCAVWITFIPAYISSPGKYTVAVEIFAILASSFGLLFCIFIPKCFVILFKPEQNTKKHLMGKMAS
>Reedfish.locus40513.1
------------------------------
MGILLVTLSLLGAFLTTSIAVIFYIHRDTPIVKANNSELSFLLLFSLTLCFLCSLTFIGQPSEWSCILRHTSFGITFVLCISCILGKTIVVLMAFRATLPGSNIMKWFGPSQQRLSVCAFTLIQVSICTVWLLTSPPFPNKNMKHSSEI
IILECDLGSVTAFYVVLGYIGFLASWCFVLAFLARKLPDNFNEAKFITFSILIFCAVWITFIPAYVSSPGKYTVAVEIFAILASSFGLLFCIFMPKCYIILIKPEQNTKKYLMGKMPS
>Reedfish.locus40492.1
------------------------------
MGILLATFSLLGAFLTTSIAFVFYLKRDTPIVKANNSELSFLLLFSLALCFLCSLTFIGQPSDWSCILRHTAFGITFVLCISCVLGKTIVVLMAFRATLPGSNIMKWFGPTQQRLSVCAFTLIQVVICLFWLLISPPFPNKNMKHSSEI
IILECDLGSATAFYAVLGYIGFLSGLCFILAFLARKLPDNFNEAKFITFSILIFCAVWITFIPAYISSPGKYTVAVEIFAILASSFGLLICIFIPKCYIILLKPEKNTKKYLMGKM--
>Reedfish.locus40486.1
--
MCYEEYKSNKQKDQCILKDIEFLSFEEKMGILMVTLSVLGASLTMSVALIFYMNRDTPIVKANNSELSFLLLFSLTLCFLCSLTFIGEPSYWSCMLRHTIFGITFVLCISCVLGKTIVVLMAFRATLPGNDIMKWFGPIQQRLSVCTFT
LVQVLICLLWLLISPPFPNRNLKHFTEIIIIECALGSQTAFSAVLGYIGFLALLCFILAFLARKLPDNFNEAKYITFSMLIFCAVWVTFIPAYISSPGKYTVAVEIFAILASSFGLLFCIFIPKCYIIILKPEQNTKKHIMGKIPS
>Reedfish.locus27495.1
------------------
MKQIEFLSYEETMGIILAASAIFGSCLSFGVLIVFVYNRHTPVVKANNSELSFLLLVSLSLCFLCSLCFIGQPSQLNCLLRHIMFGVSFVLCISCILVKTVVVIMAFKATLPGNNMMKWFGVAQQRGTVFIFTVIQSIICITWLITAPP
APSKNTKYQNAKIIFECDIGSVTGFSFLLGYIGLLSLVCFVLAFLARNLPDTFNEAKFITFSMLIFCSVWISFIPAYISSPGKYTVAVEIFAILASSFGLLLSIFAPKCYIILLKPEKNTKKALMGKSEK
>Mouse.locus176.1
CVNCPEYQYANTEQNKCIQKGVTFLSYEDPLGMALALMAFCFSAFTALVLCVFLKHHDTPIVKANNRSLSYILLISLMSCFLCSFFFIGHPNRATCVLQQITFGIVFTVAVSTVLAKTVTVVLAFKVTDPGRRLRNFLVSGTPNYIIPI
CSLLQCILSAIWLEVSPPFVDIDEHSQHGHIIIVCNKGSVTAFYCVLGYLACLALGSFTLAFLAKNLPDAFNEAKFLTFSMLVFFSVWVTFLPVYHSTKGKVMVAVEIFSILASSAGMLGCIFAPKIYIILMRPVRNSTQKIKEKSYF
>Mouse.locus208.1
CVNCPEYQYANTEQNKCIQKGVTFLSYEDPLGMALALMAFCFSAFTALVLCVFLKHHDTPIVKANNRSLSYILLISLMSCFLCSFFFIGHPNRATCVLQQITFGIVFTVAVSTVLAKTVTVVLAFKVTDPGRRLRNFLVSGTPNYIIPI
CSLLQCILSAIWLEVSPPFVDIDEHSQHGHIIIVCNKGSDTAFYCVLGYLACLALGSFTLAFLAKNLPDAFNEAKFLTFSMLVFFSVWVTFLPVYHSTKGKVMVAVEIFSILASSAGMLGCIFAPKIYIILMRPVRNSTQKIKEKSYF
>Mouse.locus150.1
CVNCPEYQYANTEQNKCIQKGVTFLSYEDPLGMALALMAFCFSAFTAVVLCVFLKHHDTPIVKADNRILSYILLISLMSCFLCSFFFIGHPNRATCVLQQITFGIVFTVAVSTVLAKTVTVVLAFKVTDPGRRLRNFLVSGTPNYIIPI
CSLLQCALSAIWLEVSPPFVDIDEHSQHGHIIIVCNKGSVTAFYCVLGYLACLALGSFTLAFLAKNLPDAFNEAKFLTFSMLVFFSVWVTFLPVYHSTKGKVMVAVEIFSILASSAGMLGCIFAPKIYIILMRPVRNSTQKIKEKSYF
>Mouse.locus159.1
CVNCPEYQYANTEQNKCIQKGVTFLSYEDPLGMALALMAFCFSAFTAVVLCVFVKHHDTPIVKANNRILSYLLLISLMSCFLCSFFYIGHPNRATCVLQQITFGIVFTVAVSTVLAKTVTVVLAFKVTDPGRRLRNFLVSGTPNYIIPI
CSLFQCVLCAIWLEVSPPFVDIDEHSQHGHIIIVCNKGSVTAFYCVLGYLACLALGSFTLAFLAKNLPDAFNEAKFLTFSMLVFFSVWVTFLPVYHSTKGKVMVAVEIFSILASSAGMLGCIFAPKIYIILMRPVRNSTQKIKEKSYF
>Mouse.locus126.1
CVNCPEYQYANTEQNKCIQKGVTFLSYEDPLGMALALMAFCFSAFTAVVLCVFVKHHDTPIVKANNRILSYLLLISLMSCFLCSFFYIGHPNRATCVLQQITFGIVFTVAVSTVLAKTVTVVLAFKVTDPGRRLRNFLVSGTPNYIIPI
CSLLQCVLCAIWIEVSPPFVDIDEHSQHGHIIIVCNKGSVTAFYCVLGYLACLALGSFTLAFLAKNLPDTFNEAKFLTFSMLVFFSVWVTFLPVYHSTKGKVMVAVEIFSILASSAGMLGCIFAPKIYIILMRPVRNSTQKIKEKSYF
>Mouse.locus133.1
CVNCPEYQYANTEQNKCIQKGVTFLSYEDPLGMALALMAFCFSAFTAVVLCVFVKHHDTPIVKANNRILSYILLISLMSCFLCSFFFIGHPNRATCVLQQITFGIVFTVAVSTVLAKTVTVVLAFKVTDPGRRLRNFLVSGTPNYIIPI
CSLLQCVLCAIWLEVSPPFVDIDEHSQHGHIIIVCNKGSVTAFYCVLGYLACLALGSFSLAFLAKNLPDTFNEAKFLTFSMLVFFSVWVTFLPVYHSTKGKVMVAVEIFSILASSAGMLGCIFAPKIYIILMRPVRNSTQKIKEKSYF
>Mouse.locus183.1
CVNCPEYQYANTEQNKCIQKGVTFLSYEDPLGMALALMAFCFSAFTAVVLCVFVKYHDTPIVKANNRILSYILLISLMSCFLCSFFFIGHPNRATCVLQQITFGIVFTVAVSTVLAKTVTVVLAFKVTDPGRRLRNFLVSGTPNYIIPI
CSLLQCVLCAIWLEVSPPFVDIDEHSQHGHIIIVCNKGSVTAFYCVLGFLACLALGSFSLAFLAKNLPDTFNEAKFLTFSMLVFFSVWVTFLPVYHSTKGKVMVAVEIFSILASSAGMLGCIFAPKIYIILMRPVRNSTQKIKEKSYF
>Western_clawed_frog.locus457.1
---------------------------------------
MISSPVPLFILGIFICYKKTPIVRANNYSLSCLLLLSLFLCFLCSLGFIGYPQPEKCLLRQVAFGMVFALCISCVLAKTITVVIAFNATKPGNRLRKWTGVRVSYCVIGICVLIQLFLCVIWLILSPPFPEYDTGTKPGIIIVVCNEGS
PTAFWCMLGYLGFLAMISFIVAFLARRLPDSFNEAKLITFSMLAFLSVWVSFIPAYLSARGMYTVAMEVFAILSSSWAVVGCIFVPKCYIVLFKPNVNSRKHILGK-RG
>Coelacanth.locus377.1
CQKCRDDEWSNERHDECIPKLIDYLSYEELMGITFAVISLSSTLFPALILYTFIKYRDTPLVKPNNREISYLLLVGLMLSFLCSLIFIGRPTRVTCMMRQTVFGIIFALCVSCVLAKTIMVVIAFNATKPNSMLKKWVGPKLSISIVVF
CTVIQISICIAWLSSAPPFPEKDMKSQIGMIIIGCNEGSTTAFWCVLGYMGLLAIISFIVAFLARNLPDSFNEASFITFSMLIFVSVWLAFIPAYVSTRGKYMVAVEIFAILASSTGLLTCIFFSKCYIILLMSDRNTKEYLLGKGTK
>Western_clawed_frog.locus494.1
CHPCSWDTWPNLQQDRCLPRPTEFLSYGDPLGYSLAAISIFSSLIPLGVLGVFIHQKKTPIVRANNYSLSCLLLLSLFLCFLCSLGFIGYPQPEKCLLRQVAFGMVFALCISCVLAKTITVVIAFNATKPGSRLRKWTGVKVSYCVIML
CALIQITDCALWLIFSPPFHELDVDTKPGVIIVNCNEGSPTAFWCMLGYLGLLASISFIVAFLARRLPDSFNEAKLITFSMLAFLSVWVSFIPAYLSARGMYTVAMEVFAILSSSWAVVGCIFVPKCYIILFRPNMNSREHLMGKDKN
>Western_clawed_frog.locus417.1
CFPCTAETWPNEDRTECLPKPVDFLSFEEPLGITITCTTIFSSFSTLTILCVFIRYKDTAIVKANNRDLTYVLLGSLSLSFLCPLLFIGQPHRFTCLFRQVTFGVIFVLSVSCVLAKTVMVVIAFRATKPGSNMRRWLGPIVPGLIVSA
CTNLQLFICIFWMLYCPPFPERNSRIKIGVIVFQCNECSDTLLWCMVGCMAFLSCVCFLVAFLARKLPDTFNEAKWITFSMLIFLSVWIAFAPAYLSTQGKNMAAVEVFGIICSSAGILVCIFLPKCYIILLRPDLNTRGNILNNRST
>Spotted_gar.locus137.1
------------------------------
MGIILSAVSLLGSCFTIAVASIFVYYRNTPIVKANNSELSFLILLSLVLCFLCSLAFIGQPTVWSCMLRHSVFSIAFVLCISCILGKTIVVLFAFKATLPGNNRMKWFGPSQQRIIIFLCTSVQILNCTVWLTVAPPSPKKSLTYESSK
IVLQCDVGSVLFFCLVLGYIGFLSVLCFLLAFLARKLPDNFNEAKFITFSMLIFFAVWIAFVPAYVSSPGKYTVATEIFAILSSSFGLLVCLFFPKCYIILLKPEKNSKKHIMGKTAS
>Mouse.locus191.1
CVNCPEYQYASTEQNKCIQKVVTFLSYEDPLGMALALIAFCFSAFTAVVLCVFVKHHDTPIVKANNRILSYILLLSLMSCFLCSFFFIGHPNRATCVLQQITFGIVFTVALSTVLAKTVTVVLAFKITDPGRRLRHFLVSGTPNYIIPI
CSLLQCVLCAIWLAVSPPFVDIDEHSQNGHIIIVCNKGSVTAFYCVLGYLACLALGSFTVAFLAKNLPDTFNEAKFLTFSMLVFFSVWVTFLPVYHSTKGKVMVAVEIFSILASIAGMLGCIFAPKIYIILMSPENNSTQKIKEKSYF
>Brownbanded_bambooshark.locus520.1
CIQCPEDYWSNENRDECVHKVIEYLGYNDPLGLALIALSIFGACVATAIAVAYLIRKDTALVKANNCGLSFVLLFSLVICFLSSVVFVGEPVAWSCMTRQVLLAISFATCLSCMLSKAVNLMLKARKTKPEDTTEKLFSPLQQRIIALI
FVSCHACLCAAWLIILPPYPVKNTQSQNIKIIMECNEGSVVFLCCVLGYDALLAAICFVFAFIARKLPDNFNEAKFMTFALLVFFIVWISFIPAYLSTRGKYMVAVEMFAILASSFGLVACLFVPKCYIILLKPERNTEELVSGKTDT
>Whale_shark.locus98.1
--------------------------------
MALIALSIFGACVAAAIAVAYLVRKDTALVKANNRGLSFVLLFSLVICFLSSVVFVGEPVAWSCMTRQVLLAIGFATCLSCMLSKAVNLMLKARETKPEGTTKKLFSPLQQRIIALIFVSCHACLCAAWLIILPPYPVKNTQSQNIKII
MECNEGSVVFLCCVLGYDALLAAICFVFAFIARKLPDNFNEAKFMTFALLVFFIVWISFIPAYLSTRGKYMVAVEMFAILASSFGLVACLFVPKCYIILLKPERNTEELVSGKTDT
>Great_white_shark.locus11.1
--------------------------------
MTLIALSIFGACTAAAIAVVYMVRKNTALVKANNRGLSFMLLFSLVICFLSAIIFVGLPVAWSCMTRQVLLAVSFATCLSCMLSKAVNLMLKAKATKPEGTETKLLGPFQQRIIALILVLCHACLCTAWLLILPPYPIKNTQSQNIKII
MECNEGSVVFLCCVLGYDALLAAICFVFAFIARKLPDNFNEAKFMTFALLVFFIVWISFIPAYLSTRGKYMVAVEMFAILASSFGLLACLFVPKCYIILLKPERNTEELVKGKTVT
>Cloudy_catshark.locus79.1
--------------------------------
MALIALSVFGACIAIAIAVAYMVRRDTPLVKANDRGLSFVLLFSLVICFLSSIVFVGLPVAWSCMTRQVLLAISFSTCLSCMLSKAVNLMLKARANKPEGTEKKPLSPFQQRMITLVFVLCHACLCAAWLLILPPHPIKNTQSQNIKII
MECNEGSVIFLCCVLGYDLLLAAICFVFAFIARKLPDNFNEAKFMTFALLVFFIVWISFIPAYLSTRGKYMVAVEMFAILASSFGLLACLFVPKCYIILLKPERNTEELVKGKNDT
>Thorny_skate.locus23.1
------------------------------------------------MAIVYIARKDTALVQANDCGLSLVLLFSLAICFLSSIVFVGLPVAWSCMTRQVLVAVSFAVCLSCMLSKAVNLMLIAKADR-
EGGGGKQLGPHQQRMLTLVSILCHACLCTAWLLIFPPYPIKNTLSQNIKIIMECNEGSVAFLCCVLGYDALLAGVCFFFAFVARQMPDNFNEAKFMTFALLVFFIVWISFIPAYLSTRGKYMVAVEMFAILASGFGLLACLFVPKCYII
VLKPERNTEELVKGVTSN
>Little_skate.locus49.1
CIQCPEDYWSNAKRDECVHKVIEYLGYNDPLGIALIALSVFGACISFAVAIVYIARKDTALVQANDWGLSLVLLFSLAICFLSSIVFVGLPVAWSCMTRQVLVAVSFAVCLSCMLSKAVNLMLIAKAER-
EGGRGKQLGPHQQRMLTLVSVLCHACLCTAWLLIFPPYPIKNTLSQNIKIIMECNEGSVAFLCCVLGYDALLAGVCFFFAFVARQIPDNFNEAKFMTFALLVFFIVWISFIPAYLSTRGKYMVAVEMFAILASGFGLLACLFVPKCYII
VLKPERNTEELAKGVTSN
>Smalltooth_sawfish.locus19.1
CIRCPEDYWSNANRDECVHKVIEYLGYNDPLGIALIALSIFGACVTIAIAVVYIVRKDTALVKANDRGLSLILLFSLVICFLSSIVFVGLPVAWSCMTRQVLLAISFATCLSCMLSKAVNLMLKAKAVKSEGTEKKRLRPFQQKIIALI
FTLCHACLCTAWLLIFPPYPVKNTLSQNIEIIMECNEGSVAFLCCVLGYDALLAGICFIFAFIARKLPDNFNEAKFMTFALLVFFIVWISFVPAYLSTRGKYMVAVEMFAILASSFGVLACLFVPKCYIIILKPERNTEELVQGITGT
>Elephant_shark.locus27.1
CIECLEDYWSNEKRDECLHKDIEYLGYNDPLGIALIVLSILGACVTAAVGAVYLVRRDTALVKSNDRGLSFLLLFSLVISFLSSVFFVGQPQNWSCMTRQVLLALSFSVCLSCMLAKAFALMLVARAAKPEKTEQKAAGHLQQRVIASI
FIFCHACLCLAWLLIFPPHPLKNTLSQNIKIILECSEGSVVFLCCVLGYDLLLAAICFIFAFIARKLPDNFNEAKFMTFALLVFFIVWISFIPAYLSTRGKYMVAVEIFAILASSFGMLGCLFLPKCYIILLKPERNTEELVTGKTNG
>Small-eyed_rabbitfish.locus110.1
CVECLEDYWSNEKRDECLLKEIEFLGYDDPLGITLIGLSILGASVAAAVGAVFLVRRDTALVKANDRELSLLLLFSLVISFLSSVVFIGQPRNWSCMTRQVLLGLSFGVCLSCMSAKAFALMLVARAAKPEKTGQKSAGHFQQRVIASA
FIMCQACLCLAWLLINPPKPVKNTLSQNIKIILECSEGSVIFLCCVLGYDLVLAGICFIFAFIARKLPNNFNEAKFMTFGLLVFFIVWISFIPAYLSTRGKYMVAVEIFAILASSFGMLGCLFLPKCYIILLKPERNTEELVTGKTEG
>Reedfish.locus27509.1
------------------------------
MGITLTATAASGVCLSVIVLSVFIHYRHTPVVKANNSELSFLLLVSLTLCFLCSLCFIGHPTELTCKLRHILFGISFVLCISCILVKTIVVIMAFKATLPGNNIMKWFGVIQQRSTVFFFTFIQGLICIIWLTASPPLPAKNIQYQNSK
IIFECDVGSVTGFSCLLGYIGLLTCISFLLAFLARNLPDTFNEAKFITFSMLIFCAVWITFIPAYISSPGKYTVAVEIFAILASSFGLLFAIFAPKCFIILIRPELNTKKALMGRDSE
```

```
>Reedfish.locus27507.1
------------------------------
MGITLTTVALLGVFISLGVLLVFFHYRHTPVVKANNSELSFLLLVSLTLCFLCALCFIGQPTDITCILRHIVFGISFVLSISCILVKTVVVIMAFKATLPGNNVMKFFGVTQQRSTVFLFTFIQSLVCIIWLSIAPPNPVKNTMYQNAK
IIFECDVGSLMGFSCLLGYIGILTCISFLLAFLARNLPDTFNEAKFITFSMLIFCAVWITFIPAYISSPGKYTVAVEIFAILASSFGLLFSIFAPKCYIILIRPELNTKKALMGRDSE
>Reedfish.locus27444.1
------------------------------
MGITLTTTASIGVFLSLGVLAIFINYRNTPVVKANNSELSFLLLGSLTLCFLCSLCFIGQPSDLTCRVRHVAFGISFVLCISCILVKTIVVIIAFKATLPGNNIMKWFGVTQQRGTILFFTIIQIIICIIWLNTAPPFSSRNTKNQNVK
IIFECDVGSVTGFSCLLGYIGLLSCVCFILAFLARNLPDTFNEAKFITFSMLIFCAVWITFIPAYVSSPGKYTVAVEVFAILASSFGLLVAIFAPKCYIILLRPDLNTKKALLGREQK
>Reedfish.locus27442.1
------------------------------
MGITLTTTASIGVFLSVGVLAIFVNYRNTPVVKANNSELSFLLLGSLTLCFLCSLCFIGQPSDLTCRVRHVAFGISFVLCISCILVKTIVVIIAFKATLPGNNIMKWFGVTQQRGTILFFTIIQIIICIIWLNTAPPFSSRNTKNQNVK
IIFECDVGSVTGFSCLLGYIGLLSCVCFILAFLARNLPDTFNEAKFITFSMLIFCAVWITFIPAYVSSPGKYTVAVEVFAILASSFGLLAAIFAPKCYIILLKPDLNTKKALLGREQK
>Reedfish.locus27411.1
------------------------------
MGCTLTTTALFGTCLSITVLAVFVLYRNTPVVKANNSELSFLLLIALTLCFLCALCFIGQPSDLTCRLRHVVFGISFVLCISCILVKTIVVIMAFKATLPGNNMMKWFGVTQQRGTVFFFTFIQALICIIWLATNPPFPAKNNKYQTAK
IIYECDVGSVTGFGCLLGYIGLLAGISFVLAFLARNLPDTFNEAKFITFSMLIFCAVWITFIPAYISSPGKYTVAVEIFAILASSFGLLFAIFAPKCYIILLRPEQNTKKALMGRGNA
>Reedfish.locus27424.1
------------------------------
MGITLTTTALLGGCSSVVILAIFIYFRKTPVVKANNSELSFLLLVSLMLCFLCSLCFIGEPTHLSCRLRHVAFGISFVLSISCILVKTIVVIMAFKATKPGKSVMKWFGATQQRGTVFFFTFIQALICIIWLTTAPPFPNKNTKYQNAK
IIFECDIGSVAGFSCLLGYIGLLACISFLLAFIARGLPDTFNEAKFITFSMLIFCAVWITFIPAYISSPGKHTVAVEIFAILASSFGLLFAIFAPKCYVILFKPEKNTKKALMGKDIG
>Reedfish.locus27423.1
------------------------------
MGITLTTTTLLGVCSSVVVLAIFIYFRNTPVVKANNSELSFLLLVSLTLCFLCSLCFIGEPTHWSCRLRHVAFGISFVLSISCILVKTIVVIMAFKATKPGKSVMKWFGATQQRGTVFFFTFIQALICIIWLTTAPPFPNKNTKYQNAK
IIFECDIGSVAGFSCLLGYIGLLACISFLLAFIARSLPDTFNEAKFITFSMLIFCAVWITFIPAYISSPGKHTVAVEIFAILASSFGLLFAIFVPKCYIILLRPERNTKKALMGKDIA
>Reedfish.locus27427.1
CDMCPLDSWSNPKRTVCLLKEIEFLSYEDAMGLTLTLIAFFGACSSVSVIVIFLVFRNTPVVKANNAELSFLLLFSLTLCFLCSLCFIGEPSNLTCMFRHVVFGISFVLCISCILVKTIVVIMAFKATVPGNNVTKWFGISRQRGTVFF
FTFIQALICLTWLMTAPPTPAKNTKYHHAKIILECNLGSVLGFSCLFGYIGLLACVSFLLAFLARRLPDNFNEAKFITFSMLIFCAVWIAFIPAYISSPGKHTVAVEIFAILSSSFGLLIAIFAPKCYVILIQPEYNTKKALLGKEFK
>Reedfish.locus27420.1
------------------------------
MGLTLTLISFFGACSSVSVIVIFLVFRNTPMVKANNSELSFLLLLSLTLCFLCSLCFIGEPSNLTCMFRHVVFGISFVLCISCILVKTVVVIMAFKATVPGNNITKRFGISRQRGTVFFFTFIQAFICLTWLMTAPPTPAKNTKYHHAK
IILECNLGSVLGFSCLFGYIGLLACVSFLLAFLARRLPDNFNEAKFITFSMLIFCAVWIAFIPAYISSPGKHTVAVEIFAILSSSFGLLIAIFAPKCYVILLRPEYNTKKALLGKEFK
>Reedfish.locus27418.1
------------------------------
MGVSLTVTALLGVFISVSVLVVFIRYRNTPVVKANNSELSFLLLVSLSLCFLCSLCFIGKPSDLTCMFRHALFGISFVLCISCILVKTIVVIMAFKAKLPGYNITKWFGISQQRGTVFFFTFIQALICIAWLTTAPPYPFKNTKHHPTK
VIFECDVGSVTGFSCLLGYIGMLAGISFLLAFLARHLPDKFNEAKFITFSMLIFCAVWITFIPAYISSPGKHTVAVEIFAILASSFGLLIAIFAPKCYIILFKPEQNTKKALMGRDIP
>Reedfish.locus27432.1
------------------------------
MGITLTATALSGVCLSAAALAVFIYYRRTPVVKANNSELSFLLLVSLILCFLCSLCFIGKPSPLTCMLRHVVFGISFVFCISCILVKTIVVLMAFKATLPGNNMMKWFGAAQQRGTVFALSFVQFLICIVWLVTSPPVPAKNTRYNNAK
IIFECDIGSVTGFSCLLGYIGLLACVSFLLAFLARRLPDTFNEAKFITFSMLIFCAVWITFIPAYVSSPGKHTVAVEIFAILASSFGVLVAIFGPKCYIILLRPELNTKKALMGRDIV
>Reedfish.locus27426.1
------------------------------
MGITLTATALSGVWLSFGVLAVFIYYRRTPVVKANNSELSFLLLVSLILCFLCSLCFIGKPSPLTCMLRHVVFGISFVLCISCILVKTIVVLMAFKATLPGNNMMKWFGATQQRGTVFALSFVQFLICIVWLVSAPPVPAKNTRYNNAK
IIFECDIGSVTGFSCLLGYIGLLACVSFLLAFLARRLPDTFNEAKFITFSMLIFCAVWITFIPAYVSSPGKHTVAVEIFAILASSFGVLVAIFGPKCYIILLRPELNTKKALMGRDIV
>Reedfish.locus27441.1
------------------------------
MGITLAATALTGFCLTVAVLAVFIHFRNTPVVKANNSELSYLLLVSLMLCFLCSLSFIGQPSHITCMLRHVVFGISFVMCISCILVKTIVVIMAFTATLPGKSTMKWFGVVQQRCTVFVFTALQALICIIWLITYPPSPTKNTKYQTAK
IIFECDIGSAAGFGCLLGYIWLLAGVSFLLAFLARRLPDTFNEARFITFSMLIFCAVWITFIPAYVSSPGKHTVAVEIFAILASSFGLLFAIFAPKCYIILLKPHKNTKKSLMCRSLQ
>Reedfish.locus27436.1
------------------------------
MGITLAATALTGFCLTVAVLAVFIHFRNTPVVKANNSELSYLLLVSLMLCFLCSLSFIGQPSHITCMLRHVVFGISFVMCISCILVKTIVVIMAFTATLPGKSTMKWFGVVQQRCTVFVFTALQALICIIWLITSPPSPTKNTKYQTAK
IIFECDIGSAAGFGCLLGYIWLLAGVSFLLAFLARRLPDTFNEARFITFSMLIFCAVWITFIPAYVSSPGKHTVAVEIFAILASSFGLLFAIFAPKCYIILLKPHKNTKKSLMCRSLQ
>Reedfish.locus27451.1
------------------------------
MGITLAATALSGVGLSLGVLAIFIRARNTPVVKANNSELSFLLLVSLTLCFLCALCFIGLPSPLTCSLRHVMFGISFVLCISCILVKTIVVMMAFKTKLPGDNMMKWFGTSQQRGTVFFFTLVQSLICLVWLITSPPGPVKNLKYQNIK
IIYECDVGTGIGFSCLLGYIGLLACVSFLLAFLVRNLPDTFNEAKFITFSMLIFCAVWITFIPAYISSPGKYTVAVEIFAILASSFGLLFAIFAPKCYIILFKPEQNTKKALMGRNTK
>Reedfish.locus40344.1
CFTCPPEYWSNHERSECIQKEIEFLSYDDAMGITLTATSALGACLSLSVLAIFIHHRHTPVVKANNSELSFLLLVSLTLCFLCVLCFIGKPSDITCTIRHVVFGLSFAVCISCILVKTIVVIMAFKATLPGNNMVKWFGTSQQRGTVFF
TAFIQCIICLVWLTTAPPVPNKNTKYQNSKIIFECHVGSLIGFSCLLGYIGLLACICFLLAFLARNLPDTFNEAKFITFSMLIFCAVWITFVPAYISSPGKHTVAVEIFAILASSFGLLFSIFAPKCYIILLRPELNTKKVLMGRAGQ
>Reedfish.locus40349.1
CFKCPPDYWSNLERSECIQKEIEFLSYDDAMGITLTATSALGACLSLSVLAIFIQHRHTPVVKANNSELSFLLLVSLTLCFLCVLCFIGKPSEITCTIRHVVFGLSFAVCISCILVKTIVVIMAFKATLPGNNMVKWFGTSQQRGTVFF
TAFIQSIICLIWLTTAPPLPNKNTKYQNSKIIFECHVGSLIGFSCLLGYIGLLACICFLLAFLARNLPDTFNEAKFITFSMLIFCAVWITFVPAYISSPGKHTVAVEIFAILASSFGLLFSIFAPKCYIILLRPELNTKKALMGRAGQ
>Reedfish.locus40350.1
CVKCSDDYWSNDRKTECILKEIDFLSYDDAMGATLSTISAFGACLSFGVLVIFIHYRHTPVVKANNSELSFLLLLSLTLCFLCALCFIGKPSELTCMIRHVVFGISFVVCISCILVKTIVVIMAFKATLPGNNMVKWFGTSQQRGTVFF
TAFIQSIICLVWLTTAPPVPNKNTKYQNSKIIFECHVGSLIGFSCLLGYVGLLACICFLLAFLARNLPDTFNEAKFITFSMLIFCMVWITFVPAYISSPGKHTVAVEIFAILASSFGVLLSIFAPKCYIILLRPELNTKKALMGKAGS
>Reedfish.locus27527.1
CVQCAPEFWSNQEKTQCILKDIEFLSYEDAMGITLTTVALSGAFLSTGVLTIFIHFRNSPVVKANNSELSFLLLVSLTMCFLCALCFIGKPTDLTCMVMHVAFGISFVLCIACILVKTIVVIVAFRATLPGNNMMKWFGTTQQRGTVFL
FTFIQSIICTIWLITAPPFPKKNTQYQNSKIIYECNIGSLVGFSCLLGYIGMLAAACFLLAFLARNLPDTFNEAKFITFSMLVFCAVWLTFIPAYISSPGKYTVAVEIFAILASSFGLLFAIFIPKCYIILLKPEKNTKKALLGRGLP
>Reedfish.locus40363.1
------------------------------
MGITLTTVASVGGLISVGILAIFIYHRNTPVVRANNSELSFLLLISLTLCFLCALCFIGKPSDLNCMLRHVMFGISFVLCISCILVKTIVVIMAFRATLPGNNTMKWFSVSKQRGTVFFFTFIQSIICIIWLASAPPYPYKNTKYQNAK
IIIECDVGSSAGFGCLLGYIGLLACICFLLAFLARNLPDTFNEAKFITFSMLIFCAVWITFIPAYISSPGKHTVAVEIFAILASSFGLLFAIFAPKCYIILLKPEQNTKKALMGRGTN
>Reedfish.locus27410.1
------------------------------
MGITLTATALLGASISVAVFAIFLCFRNTPVVKANNSELSFLLLLSLTLCFLCSLCFIGKPTDVTCMLRHVMFGISFVLCISCILVKTIVVIMAFKATLPGNNMMKWFGVSKQRSTVLLFTLLQALICLIWLITSPPAPSKNTKYQNLK
IILECDVGSVAGFACLLGYIGFLACLCFLLAFLARNLPDTFNEAKFITFSMLIFCAVWITFIPAYISSPGKYTVAVEVFAILASSFGLLVAIFAPKCYIILFKPEQNTKKALMGRSV-
>Reedfish.locus40334.1
------------------------------
MGIALSTTALSGSALSFIIFIVFFHYRNTPVVKANNSELSFLLLVSLALCFLCSLCFIGKPTSLNCMLRHVVFGISFVLAISCILVKTIVVIMAFKATLPGNNMMKWFGVAQQRTTVFLFTVIQAIICLIWLSLSPPVPNKNSKYQNGK
IIFECDIGSTAGFTCLLGYIGFLACVCFAIAFLARNLPDTFNEAKFITFSMLIFCTVWITFIPAYISSPGKHTVAVEVFAILASSFGLLFAIFAPKCIVILFKPELNTKKALMGKGTA
>Reedfish.locus27531.1
CFKCPNDHWSNLGKTQCVLKELEFLSYEDAMGITLTSVTLFGAFLSVVVLAVFIRYRSTPVVKANNSELSFLLLISLTLCFLCSLSFIGQPSYTTCMLRHVIFGISFVLCISCILVKTIVVIMAFRATLPGNNMMKWFGIVQQRGTVFF
FTFVQSLICITWLVTAPPAPAKNTKYFNSKIILECDLGSLIGFSLLLGYIGFLSCICFLLAFFARNLPDTFNEAKFITFSMLIFCAVWITFIPAYISSPGKHTVAVEVFAILASSFGVLLSIFVPKVYIIIFKPEMNSKKALMSRQNG
>Reedfish.locus27535.1
------------------------------
MGITLTAMTILGTCLSVAVLAVFIHYRSTPVVKANNSELSFLLLISLTLCFLCSLTFIGQPTHTTCMLRHVMFGISFVLCISCILVKTVVVIMAFRATLPGNNMMKWFGVSQQRGTVFFFTFVQSLICIIWLVTSPPTPAKNTKYFNSK
IILECDVGSVIGFSFLLGYIGFLACSCFLLAFFARHLPDNFNEAKFITFSMLIFCAVWITFIPAYISSPGKHTVAVEVFAILASSFGVLFAIFAPKCYIIIFRPERNTKRALMGKQNA
>Reedfish.locus27543.1
------------------------------
MGITLTTMASFGACLSVAVLAVFIHYRSTPVVKANNSELSFLLLISLTLCFICSLSFIGQPSTTTCMLRHVMFGISFVLCISCILVKTIVVIMAFRATLPGNNMMKWFGVAQQRGTVFCFTFIQSLICIIWLSTAPPVPAKNTKYQNAK
IILECDVGSLIGFSFLLGYIGLLACICFLLAFFSRNLPDTFNEAKFITFSMLVFCAVWITFIPAYISSPGKHTVAVEVFAILASSFGILLAIFAPKCYIIILKPERNIKRPLVVR---
>Reedfish.locus27530.1
------------------------------
MGITLTTTALVGAGLSICVLAIFIKYKNTPVVKANNSELSFLLLVSLTFCFFCALTFIGQPSTLTCILRHVVFGISFVLCVSCILVKTVVVIVAFRANMPGNNMMKWFGAAQQRGTIFFFTSVQSMICTIWLSVAAPVPAKNNKYQNSK
IILECEVGSVTGFSFLLGYIGLLASACFVLAFFARNLPDTFNEARFITFSMLIFCAVWITFIPAYISSPGKHTVAVEVFAILASSFGLLFSIFAPKCYIILLKPEQNTKKVLMTKGKI
>Reedfish.locus40382.1
------------------
MKEVEFLSYEDVMGIILTTIALIGASLSVGVLGIFIHHRNTPVVKANNAELSFLLLVSLTFCFLCSLCFIGKPSDLTCMLRHVIFGISFSFSVSCILVKTIVVIMAFKATLPGNNIMKWFGVAQQRSTVILFTFIQSVVCTVWLTISPS
VPIKNMNHQNSKVILECAIGSMTGFSFLLGYIFLLSVVCFLLAFLARNLPDTFNEARFITFSMLIFCAVWVAFIPAYVSTPGKYTVAVEVFAIITSSFGILFSIFTPKCYIILLKPDKNTKKSLLEWKKD
>Reedfish.locus27521.1
------------------------------
MGITLTAVSLSGFFISVGVLAVFIHYRHTPVVKTNNSELSFLLLASLSMCFLCSLCFIGQPSHISCMMMHAIFGISFVLCISCILVKTIVVIVVFKATQPGNNVIQWFGISQQRFTVFLCTFTQILICIVWLATAPPFPVKNTKYNMSK
IILECNIGSVTGFSFLLGYIGLLSGICFLLAFLARNLPDSFNEARFITFSMLIFCAVWITFIPAYVSSPGKYTVAVEIFAILASSFGILFAIFTPKCYIILLKPEQNTKKALMSR---
>Reedfish.locus40330.1
---------------------
MEFLSYEEPMGITLTTVAIVGACFSVAVLGLFIHFRSTPVVKANNSELSFFLLASLTLCFLCTLCFVGQPSKLTCMLRHVVFGISFVLCVSCILVKTIVVIMAFKSSLPGSNAMKWFGIAQQRSTVFFFTFVQSLICIVWLTTAPPIPA
KNTKYQAAKIILECDLGSLVGFSFILGYIGLLAAVCFVIAFLARNLPDNFNEARFITFSMLIFCAVWITFIPAYISSPGKHTVAVEIFAILASSFGVLFAIFTPKCYIIIFKPQQNTRRAVMRR---
>Reedfish.locus27467.1
------------------------------
MGITLTTTAIFGSCLSVSVLIVFIYYRNTPVVKANNSELSFLLLLSLTLCFLCSLCFIGEPSNATCMLRHVVFGISFVLSISCILVKTIVVIMAFKATLPGNNMMKWFGATQQRGTVFMFTFIQSLICAVWLTSAPPFSSKNIKHQNSK
IIFECDVGSFIGFSCLLGYIGLLAGISFVLAFLARNLPDTFNEAKFITFSMLIFCAVWITFIPAYISSPGKYTVAVEIFAILASSFGLLGAIFAPKCYIILLKPEKNTKKALMGR---
>Reedfish.locus27472.1
------------------------------
MGITLTATAIFGSCMSVGVLVVFIYYRNTPVVKANNSELSFLLLLSLTLCFLCSLCFIGEPSNANCTLRHVVFGISFVLSISCILVKTIVVIMAFKATLPGNNMMKWFGVTQQRGTVFMFTFIQSLICVVWLSSAPPFPSKNIKYHNSK
IIFECNVGSFIGFSCLLGYIGLLAGISFVLAFLARNLPDTFNEAKFITFSMLIFCAVWITFIPAYISSPGKYTVAVEIFAILASSFGLLGAIFAPKCYIILLKPEKNTKKALMGR---
>Reedfish.locus27474.1
------------------------------
MGITLATTAVFGSCMSISVLLIFLNFRNTPVVKANNSELSFLLLLSLTFCFLCSLCFIGEPSNATCMLRHVVFGISFVLSISCILVKTIVVIMAFKATLPGNNMMKWFGVTQQRGTVFMFSFIQSLVCVVWLSSAPPFPSKNTKHQNSK
VIFECDIGSLIGFSCLLGYIGLLACISFVLAFLARNLPDTFNEAKFITFSMLIFCAVWITFIPAYISSPGKYTVAVEIFAILASSFGLLSAIFAPKCYIILLKPERNTKRALMGR---
```

```
>Reedfish.locus27468.1
------------------------------
MGITLTATAIFGSCLSVSVLIVFIYYRNTPVVKANNSELSFLLLLSLTLCFLCSLCFIGEPSNASCTLRHVVFGISFVLSISCILVKTIVVIMAFKATLPGNNMMKWFGVTQQRGTVFMFTFIQSLICVAWLTSAPPFPSKNTKHQNSK
VILECDVGSLIGFSCLLGYIGLLACISFVLAFLARNLPDTFNEAKFITFSMLIFCAVWITFIPAYISSPGKYTVAVEIFAILASSFGLLGAIFAPKCYIILFKPERNTKKALMGR---
>Reedfish.locus27482.1
------------------
MKQIEFLSYEETMGIILAASAIFGSCLSFGVLIVFVYNRHTPVVKANNSELSFLLLVSLSLCFLCSLCFIGQPSQLNCLLRHIIFGVSFVLCISCILVKTVVVIMAFKATLPGNNMMKWFGVAQQRFTVFLFTFIQSIICIIWLITAPP
APSRNTKHQNAKIILECDIGSVTGFSFLLGYIGLLSLVCFVLAFLARNLPDTFNEAKFITFSMLIFCAVWLSFIPAYISSPGKYTVAVEIFAILASSFGLLMAIFAPKCYIILLKPEKNTKKVLMSR---
>Reedfish.locus27487.1
------------------
MKQIEFLSYEETMGIILAASAIFGSSLSFGVLIVFVYNRHTPVVKANNSELSFLLLVSLSLCFLCSLCFIGQPSQLNCLLRHIMFGVSFVLCISCILVKTVVVIMAFKATLPGNNMMKWFGVAQQRFTVFIFTVVQSIICIIWLITAPP
APSKNTKYQNAKIILECDIGSVTGFSFLLGYIGLLSLVCFVLAFLARNLPDTFNEAKFITFSMLIFCAVWLSFIPAYISSPGKYTVAVEVFAILASSFGLLMAIFAPKCYIILLKPEKNTKKVLMGRYDK
>Reedfish.locus27491.1
---------------
MCLMKQIEFLSYEETMGIILAASAIFGSSLSFAVLIVFVYNRHTPVVKANNSELSFLLLVSLSLCFLCSLCFIGQPSQLNCLLRHIMFGVSFVLCISCILVKTVVVIMAFKATLPGNNMMKWFGVAQQRGTVFIFTIIQSIICITWLIT
APPAPSKNTKYQNAKIILECDIGSVTGFSFLLGYIGLLSLVCFVLAFLARNLPDTFNEAKFITFSMLIFCAVWLSFIPAYISSPGKYTVAVEIFAILASSFGLLMAIFAPKCYIILLKPEKNTKKVLMGRFDK
>Reedfish.locus27518.1
------------------------------
MGIVLTSTTSLGVCLSVGVLLVFIRYRNTPVVKANNSELSFLLLVSLTLCFLCALCFIGPPSHFTCALRHVAFGISFVLCISCILVKTIVVIMAFKATLPGNNMMKWFGAAQQRGSVLFFTFIQSLICIVWLSTSPPIPSKNTKYQNAK
IIFECDVGSVTGFSCLLGYIGFLACFCFALAFLARNLPDTFNEAKFITFSMLIFCAVWVSFIPAYISSPGKYTVAVEVFAILASSFGLLFAIFAPKCYIILLKPEKNTKRALMGRQA-
>Reedfish.locus40381.1
------------------------------
MGIALTSLAALGMCVSVSVLAVFIHFRETAVVKANNSELSYFLLVSLALCFLCSLFFIGEPSHVTCRLRHVAFGISFVLCISCLLVKTVVVIIAFKATLPGNNRIKWFGAVQQRGTVFFFTSVETLICILWLVIAPPVPAKNTNNEHSK
IILECYVGSITGFSCVLGYIFFLACICFILAFLSRNLPDSFNEAKFITFSMLIFLAVWIAFIPAYVSSPGKYTIAVEIFAILASSFGLLTAIFTPKCYIILLKPELNNKRSLMGRLIN
>Reedfish.locus40378.1
------------------------------
MGITLTTIAALGICVSFSVLAVFIHFRETAVVKANNSELSYFLLVSLALCFLCSLFFIGEPSHVTCRLRHVAFGISFVLCISCLLVKTVVVIIAFKATVPGNNRIKWFGAVQQRGTVFFFTSVETLICILWLVISPPVPAKNTNSEHSK
IILECNVGSVTGFACVLGYIFFLACVCFVLAFLARNLPDSFNEAKFITFSMIIFLAVWIAFIPAYVSSPGKYTIAVEIFAILASSFGLLMAIFTPKCYIILLKPELNNKRSLMGRSTN
>Reedfish.locus27478.1
------------------------------
MGITLTTTALFGSCLSLSVLGVFLLYRNTPVVKANNSELSFLLLIALNLCFLCSLCFIGQPSQLTCGLRHVLFGISFALCISCILVKTLVVIMVFKATLPGNNTMKWFGAAQQRGTVFIFTCIQSLICIIWLTTAPPVPAKNTNYQNTK
IILECNVGSLMGFGFLLGYIGILACVCFVLAFLARNLPDNFNEAKFITFSMLIFCAVWITFIPAYISSPGKYTVAVEIFAILASSFGVLIAIFAPKCYIILFKPEENTKKALMGRAVT
>Reedfish.locus27457.1
-----
MEYWSNYERNKCVIREVEFLSYEDAMGITLTSTALFGACLSVGVLAVFIHHRNTPVVKANNSELSFLLLVSLTLCFLCSLCFIGKPSDLTCRLRHVMFGISFVLCISCILVKTIVVLMAFKATLPGNNMMKWFGVAQQRSTVFFCTFIQ
SLVCTVWLMTAPPVPAKIPTFQTAKLIFECNVGSLTGFSCLLGYIGLLASICFVLAFFARNLPDNFNEARFITFSMLIFCAVWITFIPAYISSPGKYTVAVEIFAILASSFGLLVAIFSPKCYIILFKPERNTKKAMMSRTLS
>Sterlet.locus293.1
------------------------------
MGISLMTIALFGAGLSVGVLFVFIFYRNTPVVKANNSELSFVLLVSLTLCFLCSLCFIGQPTNLTCLLRHVVFGISFVVCISCILVKTIVVIMAFKATLPGDNLMKWFGVAQQRGTVFLLTFIQVVICIVWLTTAPPLPTKNTKYQTAK
IIFECNAGSFTGYSCLLGYIGLLACTCFLFAFFARNLPDHFNEAKFITFSMLVFCAVWITFIPAYVSSPGKYTVAVEIFAILASSFGLLVAIFAPKCYIILLKPEKNTKKALMGRAGT
>marker.zebrafish_16_13_F
---
CPYEFWSNKEKDYCVPKQVEFLSYEDPLGISLTTASLLGICFCALVMVIFSHHHNTPIVRANNSELSFLLLVSLKLCFLCVLLFIGQPQLWTCQLRYAVFGISFVLSVCSILVKTMVVIAVFKSSRPGKDAMKWFGLLQQRCTILVLTT
IQVVICTVWISNASPTPHKNHQYIRSKIVFECAIGSVAGFSLLLGYIGLLAAISFLLAFLARNLPDNFNEAKFITFSMLIFCAVWIAFVPAYVSSPGKYAVAVEIFAILASSFGLLMAIFTPKCYIILLHPERNTKKAIMGR---
>Zebrafish.locus531.1
CLVCPYEFWSNKEKDYCVPKQVEFLSYEDPLGISLTTASLLGICFCALVMVIFSHHHNTPIVRANNSELSFLLLVSLKLCFLCVLLFIGQPQLWTCQLRYAVFGISFVLSVCSILVKTMVVIAVFKSSRPGKDAMKWFGLLQQRCTILV
LTTIQVVICTVWISNASPTPHKNHQYIRSKIVFECAIGSVAGFSLLLGYIGLLAAISFLLAFLARNLPDNFNEAKFITFSMLIFCAVWIAFVPAYVSSPGKYAVAVEIFAILASSFGLLMAIFTPKCYIILLHPERNTKKAIMGR---
>Zebrafish.locus530.1
CTVCPDEFWSNKDKDQCVPKEVEFLSYEDPLGISLTTACLLGTCFCALVMIIFCQHRNTPIVRANNSELSFLLLLSLKLCFLCVLLFIGRPQLWTCQLRHAVFGISFVLCVSSILVKTMVVIAVFKSSRPGKGSMKWFGTTQQRCTVLI
LTALQVVICIVWLSNSSPAPHKNSQHISSKIVYECAIGSLAGFSLLLGYIGLLAAVSFLLAFLARNLPDNFNEAKFITFSMLIFCAVWIAFVPAYVTSPGKYAVAVEIFAILASSFGLLGAIFAPKCYIILLHPEKNTKKAIMGRQIT
>marker.zebrafish_16_12_F
---
CPDEFWSNKDKDQCVPKEVEFLSYEDPLGISLTTACLLGTCFCALVMIIFCQHRNTPIVRANNSELSFLLLLSLKLCFLCVLLFIGRPQLWTCQLRHAVFGISFVLCVSSILVKTMVVIAVFKSSRPGKGSMKWFGTTQQRCTVLILTA
LQVVICIVWLSNSSPAPHKNSQHISSKIVYECAIGSLAGFSLLLGYIGLLAAVSFLLAFLARNLPDNFNEAKFITFSMLIFCAVWIAFVPAYVTSPGKYAVAVEIFAILASSFGLLGAIFAPKCYIILLHPEKNTKKAIMGRQ--
>Zebrafish.locus516.1
CVVCPDEFWSSPKKNQCVPKDVEFLTYGDPLGISLTTASLLGSCICSAVVVIFAHHRHTPVVRANNSELSFLLLVSLKLCFLCVLLFIGQPQLWTCRLRHAVFGISFVLCISSILVKTMVVIAVFKSSRPGKSAMKWFGSHQQRCTVLV
LTALQVVICAVWLTNASPKPYKNNQYTSSKIVYECTIGSVVGFAMLLGYIGILAAVSFLLAFLARNLPDHFNEAKFITFSMLIFCAVWIAFVPAYVSSPGKYAVAVEIFAILASSFGLLAAIFAPKCYIIILHPERNTKKAIMGRAT-
>Zebrafish.locus855.1
CVVCPDEFWSSPKKDQCVPKDVEFLTYGDPLGISLTTASLLGSCICSAVVVIFAHHRHTPVVRANNSELSFLLLVSLKLCFLCVLLFIGQPQLWTCRLRHAVFGISFVLCISSILVKTMVVIAVFKSSRPGKSAMKWFGSHQQRCTVLV
LTALQVVICAVWLTNASPKPYKNNQYTSSKIVYECTIGSVVGFAMLLGYIGILAAVSFLLAFLARNLPDHFNEAKFITFSMLIFCAVWIAFVPAYVSSPGKYAVAVEIFAILASSFGLLAAIFAPKCYIIILHPERNTKKAIMGRAT-
>marker.zebrafish_16_11_F
---
CPDELWPNPNKDQCVPKEEDFLSFEDPLGISLTTASLLGTCFCALVMVIFSHHRNTPVVRANNSELSFLLLLSLKLCFLCVLLFIGRPKLWTCQLRHAAFGISFVLCVSSILVKTMVVIAVFKSSRPSKSAMKWFGVAQQRGTVMALTT
LQIVICTVWLSMASPKPYKNSLYISSKVVYECDIGSVVGFSLLLGYIGLLAAVSFLFAFLARNLPDNFNEAKFITFSMLIFCAVWITFVPAYVSSPGKYAVAVEIFAILASSFGLLVAIFAPKCYMILFHPERNTKKSIMGRA--
>Zebrafish.locus528.1
CKICPDELWPNPNKDQCVPKEEDFLSFEDPLGISLTTASLLGTCFCALVMVIFSHHRNTPVVRANNSELSFLLLLSLKLCFLCVLLFIGRPKLWTCQLRHAAFGISFVLCVSSILVKTMVVIAVFKSSRPSKSAMKWFGVAQQRGTVMA
LTTLQIVICTVWLSMASPKPYKNSLYISSKVVYECDIGSVVGFSLLLGYIGLLAAVSFLFAFLARNLPDNFNEAKFITFSMLIFCAVWITFVPAYVSSPGKYAVAVEIFAILASSFGLLVAIFAPKCYMILFHPERNTKKSIMGRATQ
>Zebrafish.locus858.1
CTSCPNEFWSSPKKDQCVPKEVEFLSYEDPLGISLTTASLLGTCFCALVLAVFAHHHNTPVVRANNSELSFLLLLSLKLCFLCVLLFIGRPQLWTCQLRHAMFGISFVLCVSSILVKTMVVIAVFKSSRPGKSAVKWFGAVQQRGTVLV
LTAVQIVICVVWLSTASPTPHKNILYVRSKIVFECSIGSMAGFAILLGYIGILAAVSFLLAFLARNLPDNFNEAKFITFSMLIFSAVWIAFIPAYVSSPGKYSVAVEIFAILASSFGLLVAIFVPKCYIIILHPERNTKKAIMGRTAE
>Zebrafish.locus519.1
CTSCPNEFWSSPKKDQCVPKEVEFLSYEDPLGISLTTASLLGTCFCALVLAVFAHHHNTPVVRANNSELSFLLLLSLKLCFLCVLLFIGRPQLWTCQLRHAMFGISFVLCVSSILVKTMVVIAVFKSSRPGKSAVKWFGAVQQRGTVLV
LTAVQIVICVVWLSTASPTPHKNILYVRSKIVFECSIGSMAGFAILLGYIGILAAVSFLLAFLARNLPDNFNEAKFITFSMLIFSAVWIAFIPAYVSSPGKYSVAVEIFAILASSFGLLVAIFVPKCYIIILHPERNTKKAIMGRTAE
>Zebrafish.locus856.1
---------------------
MEFLSYDDPLGISLTTASLLGTCFCVLVLVIFAYHHNTPVVRANNSELSFLLLVSLKFCFLCVLLFIGRPQLWTCQLRHAVFGISFVLCISSILVKTMVVIAVFKSSRPGKTSIKWFGSAQQRGTVIALTSAQVAICTVWLSSASPTPH
KNNQYIHSKIVYECAIGSVAGFSLLLGYIGLLAAVSFLLAFMARKLPDNFNEAKFITFSMLIFCAVWIAFVPAYVSSPGKYAVAVEIFAILASSFGLLAAIFAPKCYIIILHPERNTKKAIMGRSTQ
>Zebrafish.locus517.1
---------------------
MEFLSYDDPLGISLTTASLLGTCFCVLVLVIFAYHHNTPVVRANNSELSFLLLVSLKFCFLCVLLFIGRPRLWTCQLRHAVFGISFVLCISSILVKTMVVIAVFKTSRPGKTSIKWFGSAQQRGTVIALTSAQVAICTVWLSSASPTPH
KNNQYIHSKIVYECAIGSVAGFSLLLGYIGLLAAVSFLLAFMARKLPDNFNEAKFITFSMLIFCAVWIAFVPAYASSPGKYAVAVEIFAILASSFGLLAAIFAPKCYIIILHPERNTKKAIMGRSTQ
>marker.zebrafish_16_10_F
---
CPNDFWSNPEKDQCIPKEVEFLSYEDPLGISLTTAAMLGTFICALVMTIFAHYRNTPVVRSNNSELSFLLLLSLKLSFLCVLLFIGQPQLWTCQLRHAVFGISFVLCVSSILVKTMVVIAVFKSSRPGKNAMKWFGAAQQRGTVLILTA
LQVLICAVWLSTASPTPHKNSRYIRSIIVYECAIGSVAGFSLLLGYIGLLAAVSFLLAFLARNLPDSFNEAKFITFSMLIFCAVWIAFVPAYVSSPGKYAVAVEIFAILASSFGLLLAIFAPKCYIIILHPERNTKKAIMGKG--
>Zebrafish.locus527.1
---------------------------------------
MLGTFICALVMTIFAHYRNTPVVRSNNSELSFLLLLSLKLSFLCVLLFIGQPQLWTCQLRHAVFGISFVLCVSSILVKTMVVIAVFKSSRPGKNAMKWFGAAQQRGTVLILTALQVLICAVWLSTASPTPHKNSRYIRSIIVYECAIGS
VAGFSLLLGYIGLLAAVSFLLAFLARNLPDSFNEAKFITFSMLIFCAVWIAFVPAYVSSPGKYAVAVEIFAILASSFGLLLAIFAPKCYIIILHPERNTKKAIMGKGTK
>marker.cichlid_16-10aa
---
CPEDFWSNPQRDHCVPKKTEFLSYHEPLGICLAATSLLGTLICAVVLGIFFYHRRTPIIRANNSELSFQLLLSLKLCFLCSLLFIGRPRLWTCQLRHAAFGISFVLCVSCILVKTMVVLAVFKASKPGGTSLKWFGVTQQRGTVLFLTS
IQAAICTTWLVSSSLTPHKNIQYQNDKIVYECAFGSTVGFAVLLGYIGVLAFLSFLIAFLARNLPDSFNEAKLITFSMLIFCAVWVAFVPVYISSPGNYADAVEVFAILASSFGLLITLFGPKCYIIIMRPELNTKKAIMCRG--
>marker.cichlid_16-8aa
---
CPEDFWSNPQRDHCVPKKTEFLSYHEPLGICLTATSLLGTCTCAVVLGIFIYHHKTPIVRANNSELSFQVLLSLKLCFLCSLLFIGRPRLWTCQLRHAAFGISFVLCVSCILVKTMVVLAVFKASKPGVTSLKWFGAMQQRGTVLCLAS
VQAAICTAWLVSASPSPHKNTKYHNDMIVYECAVGSPVGFAVLLGYIGMLAVLSFLIAFLVRNLPDSFNEAKLITFSMLIFCAVWVAFVPVYISSPGNYADAVEAFAILASSFGLLITLFGPKCYIILLKPELNTKKAIMGRG--
>marker.cichlid_16-4aa
---
CPEDFWSNHQRDHCVPKKTEFLSYDEPLGFCLTATSLLGTIICAVVIGIFFYHRRTPIVLANNSELSFQLLLSLKLCFLCSLLFIGRPRLWTCQLRHAAFGISFVLCVSCILVKTMVVLAVFKASKPGGASLKWFGAMQQRGTILFLTS
IQVVICTAWLVSASPTPNKSTQYHNDKIIYECSVGSSVGFGVLLGYIGMLAFLSFLIAFLARNLPDSFNEAKLITFSMLIFCAVWVAFVPAYISSPGNHADAVEVFAILASSFGLLVALFGPKCYIILLRPEMNTKKVIMGRG--
>marker.medaka_16_2_F
---
CPEDFWSNSRRDLCVPKKTEFLSYHEPLGICLTTTSLLGTFICTVVLAIFAFHRRSPIVRANNSELSFLLLVALKLCFLCSLLFIGQPRLWTCQLRHAVFGISFVLCISCILVKTMVVLAVFKASEPGGGNLKWFGPKQQRGTVFSLTC
IQAAICAAWLVSSSPAPRKNTQYHNDKIVYECEVGSTIGFAVILGYIGMLAILSFFLAFLARNLPNNFNEAKLITFSMLIFCAVWMAFVPAYINSPGRYADAVEVFAILASSFGLLVPLFGPKCYIILLKPEKNTKKAIMGR---
>marker.cichlid_16-3aa
---
CPEDFWSNPQNDHCVPKKTEFLSYHEPLGICLTTASLLGTFICAVVLGIFFYHRRTPMVRANNSELSFLLLVSLKLCFLCSLLFIGRPRVWTCQLRHAAFGISFVLCVSCILVKTMVVLAVFKASKPGGTILKWFGSMQQRGTVLALTC
IQAGICTAWLVSSSPAPHKNMQYQNDKIVYECAVGSTIGFAVLLGYIGVLAVLSFLLAFLARNLPDNFNEARLITFSMLSFCAVWVAFVPAYVNSPGKYADAVEVFAILASSFGLLVALFGPKCYIILLKPERNTKKAIMGRL--
>marker.cichlid_16-1aa
---
CPEDFWSSPQRDRCVPKKTEFLSYHEPLGICLTTASLLGTFICAVVLGIFTHYRSTPIVRANNSELSFLLLVSLKLCFLCSLLFIGRPRLWTCQLRHVAFGISFVLCVSCILVKTMVVVAVFKASKPGGTNIKWFGSVQQRGTVLVLTS
IQAAICTAWLISSSPVPYKNTQYYNDKIVFECVVGSTIGFAVLLGYIGVLAILSFLLAFLARNLPDNFNEAKLITFSMLIFCAVWVAFVPAYVNSPGKYADAVEVFAILASSFGLLVALFGPKCYIILLKPERNTKKEIIGRG--
>marker.pufferfish_16_3_Y
---
CPEDFWSSSQRDHCVPKKTEFLSYHDPLGICLTVASLLGTVISAVVLGIFIHHRSTPVVRANNSELSFLLLVSIKLCFLCSLLFIGRPRLWTCQLRHAAFGISFVLCVSCILVKTMVVLAVFSASKPGVAILKWFGAVQQRMTATILTF
IQVAICILWILLASPFPRKNTQYSNEKIVYECAIGSTVGFSLLLSYIGFLAILSCLIAFYSRNLPDSFNEAKLIAFSMLIFSAVWVVFVPAYISSPGKYADAVEVFAILASSFGLLVTLFGPKCYIILLRPERNTKKAIMGRV--
>marker.fugu_16_1_F
```

```
---
CPEDFWSSPQRDHCVPKKTEFLSYHEPLGICLTAASLLGTVISAVVLGIFIHHRSTPVVRANNSELSYQILVSLKLCFLCSLLFIGRPRLWTCQLRHAAFGISFVLCVSCILVKTMVVLAVFRASKPGGATLKWFGAVQQRMTVIILTS
IQAAICLSWILLASPFPHKNTQYSNERIIYECAVGSTVGFILLLSYIGLLATLSFLIAFYSRKLPDSFNEAKLITFSMLIFCAVWVAFVPAYISSPGKYADAVEVFAILASSFGLLAALFGPKCYIVLLRPEKNTKNAIMGRV--
>marker.fugu_16_4_FP
---
CPEDFWSSPQRDHCVPKKTEFLSYHEPLGICLTAASLLGTVISAVVLGIFIHHRSTPVVRANNSELSFLLLVSLKLCFLCSLLFIGRPRLWTCQLRHAAFGISFVLCVSCILVKTMVVLAVFRASKPGGTTLKWFGAVQQRGTVLGLTS
VQAAICTAWLVSYSPEPHKNTQYYNYKIVYECVVGSPIGFSVLMGYIGLLAVLSFQLAFLARNLPDNFNEAKLITFSMLIFCAVWVAFVPAYINSPGKYADAVEVFAILASSFGLLVALFGPKCYIILLRPERNTKKAIMDRD--
>marker.medaka_16_1_F
---
CPEDFWSSKQNDHCVPKEIEFLSYDDPLGICLTTASLLGTFICSIVFAIFIYHRSTPVVRANNSELSYLILVSLKLCFLCSLLFIGQPQLWTCQLRHAVFGISFVLCVSCILVKTMVVLAVFRSSKPGGSKLKWFGSVQQRGTVLALTC
VQAVVCTVWIISASPTPHANTRYHNDKIIYECVVGSTAGFAVLLGYISLLAIISFLLAFLARNLPDNFNEAKFITFSMLIFCAVWVAFVPAYVNSPGKYADAVEVFAILASSFGLLVALFGPKCYIILLKPEKNTRKAIMGRD--
>Japanese_eel.locus262.1
CYRCPVDFWSNQLRDRCVLKEIEFLSFQEPLGISLTTISVLGACICTVTVSIFARHRNTPVVKANNSELSFLLLLSLTLCFLCSLLFIGQPQRWTCQLRHAAFGISFALSISCILVKTIVVVMAFKAARPGGNTMKWFGVQQQRGTVLA
FTIIQAVICVAWLTAASPVPYKNTRLQNSKIIYECAVGSIAGFSALLGYIGLLAAISFLLAFLARNLPDNFNEAKFITFSMLIFCAVWISFVPAYVSSPGKYSDAVEIFAILASSFGLLGAIFAPKCYIILLHPEKNTKKALMGRDAP
>Japanese_eel.locus274.1
CYRCPVDFWSNQLRDHCVPKEIEFLSFQEPLGISLTTISVLGACICTVTLSVFARHSNTPVVKANNSELSFLLLLSLTLCFLCSLLFIGQPQRWTCRLRHAAFGISFALSISCILVKTIVVVMAFKAARPGGNTIKWFGVQQQRGTVLA
FTIIQAVICVAWLTTASPVPYKNTRLQNSKIIYQCTVGSIAGFGALLGYIGLLAASSFLLAFLARNLPDNFNEAKFITFSMLIFCAVWIAFVPAYVSSPGKYSDAVEIFAILASSFGLLGAIFAPKCYIILLHPEKNTKKALMGRDAP
>Japanese_eel.locus270.1
CYKCPVDFWSNELRDHCVPKEIEFLSFQEPLGISLTTISVLGACICAVTLSVFARHRNTPVVKANNSELSFLLLLSLKLCFLCSLLFIGQPQRWTCQLRHAAFGISFALSISCILVKTIVVVMAFKAARPGGNTIKWFGMQQQRGTVLA
FTIIQAVICVAWLTAASPVPYKNTRLQNSKIIYECAVGSIAGFSALLGYIGLLAAISLLLAFLARNLPDNFNEAKFITFSMLIFCAVWIAFVPAYVSSPGKYTVAVEIFAILASSFGLLGAIFAPKCFIILLHPEKNTKKALMGRDAP
>Spotted_gar.locus161.1
CFKCPQEYWSNPSRDQCVLKEIEFLSYGETLGISLTTVSVFGAFISASVLAVFIYYRNTPVVRANNSELSFLLLLSLILCFLCSLLFIGQPLHLTCMLRHVVFGISFVLCISCILVKTIVVIMAFRATLPDNNVMKWFGAAQQRGTVFV
FTVIQALICIIWLSTASPVPFKNTQYQNSKIIFECNVGSITGFSFLLGYIGLLASVCFLLAFLARNLPDNFNEAKFITFSMLIFCAVWIAFIPAYVSSPGKYSDAVEIFAILASSFGLLFAIFAPKCYIILLHPEKNTKKALMGRAAP
>Spotted_gar.locus163.1
CLKCPPDFWSNPSRDQCVPKEIEFLSYEEPLGISLTTVSVFGAFISAGVLAVFIYYRNTPVVRANNSELSFLLLLSLILCFLCSLLFIGQPLHLTCMLRHVVFGISFVLCISCILVKTIVVIMAFRATLPDNNVMKWFGAAQQRGTVFV
FTVIQALICIIWLSTASPVPFKNTQYQNSKIIFECNVGSITGFSFLLGYIGLLASVCFLLAFLARNLPDNFNEAKFITFSMLIFCAVWIAFIPAYVSSPGKYSDAVEIFAILASSFGLLIAIFVPKCYIILLHPEKNTKKSLMGRAAP
>Spotted_gar.locus154.1
CFKCPPDFWSNPSRDQCVPKEIEFLSYKEPLGISLTTVSVFGGCISAAILFVFIYYRNTPVVRANNSELSFLLLLSLILCFLCSLLFIGQPLHLTCMLRHVVFGISFVLCISSILVKTIVVIMAFRATLPDNNVMKWFGAAQQRGTVFV
FTVTQALICIVWLTVAPPLPLKNTQYQNSKIIFECNVGSITGFSCLLGYIGLLASVCFLLAFLARNLPDNFNEAKFITFSMLIFCAVWIAFIPAYVSSPGKYSDAVEIFAILASSFGLLIAIFMPKCYIILLQPEKNTKKALMGRAGL
>Spotted_gar.locus166.1
-----
MDYWSSPGRDQCVLKEIEFLSYHDPLGISLTTISIFGACTSVGVLIIFFYYRNTPVVRANNSELSFLLLLSLILCFLCSLLFIGQPQHVTCMLRHIVFGISFVLCISCILVKTIVVIMAFRATLPDNNVMKWFGAAQQRGTVFVFTVIQ
ALICIVWLTVAPPLPFKNTQYQNSKIIFECNVGSITGFSFLLGYIGLLASVCFLLAFLARNLPDNFNEAKFITFSMLIFCAVWIAFIPAYVSSPGKYSDAVEIFAILASSFGLLIAIFAPKCYIILLHPEKNTKKALMGRAAP
>Reedfish.locus40365.1
------------------------------
MGIALTAVSLSGAAMSLAVLAVFIYYRETPVVKANNSELSFLLLISLILCFLCSLCFIGKPTYLTCILRHVVFGISFVLCISCILVKTIVVIMAFKATLPGKNIMKWFRSIQQRGTVFLLTLIQALICIVWLVIAPPTPSRNTKYQNAK
IILECDIGSITGFSCLMGYIGFLASICFVLAFLARKLPDNFNEAKFITFSMIIFCAVWLTFIPAYISTPGKYTVAVEIFTILASSFGIIAMIFFPKCYIILIRPERNTKKFLMAKADP
>Mouse.locus124.1
CVNCPEYQYANTEQNKCIQKGVTFLSYEDPLGMALALMAFCFSAFTAVVLCVFVKHHDTPIVKANNRSLSYLLLMSLMFCFLCSFFFIGLPNRAICVLQQITFGIVFTVAVSTVLAKTVTVVLAFKVTDPGRRLRNFLVSGTPNYIIPI
CSLLQCVLCAIWLAVSPPFVDIDEHTLHGHIIIVCNKGSDTAFYCILGYLACLALGSFSLAFLAKNLPDTFNEAKFLTFSMLVFCSVWVTFLPVYHSTKGKHMVAVEIFSILASSAGILGCIFVPKIYIILMRPERNSTQKIREKSYF
>Western_clawed_frog.locus847.1
CTKCPEDMWANEMHDGCRDKTLEFLSFEEALGGTLAAFSVVGSLFPLSILFIFLRNADTPVVKANNRNLSYILLVSLFFCYFCALTFIGRPITITCILRQFIFGISFVMCISCVLGKTMMVVIAFNLTQPRSSRRMWLNSRVTNTLVLV
CTAIQVIICAGWLAHSPPFQYTDNKSKAGTIIVECNKGSPIAYSCTI------------------KLPGSFNEAKLITFSMLIFGAVWISFIPAYLSTTGKYMVAVEVFAILSSSSGLLACIFLPKVYIIILTPEINKIKIKTTNKLK
>Western_clawed_frog.locus849.1
CTECPEDMWANEMHDGCRDKTLEFLSFEEILGGTLAAFSIVGSLLPLSILFIFLKNADTPVVKANNRNLSYTLLVSLFFCYLCALMFIGRPITITCILRQFIFGISFVMCISCVLGKTMMVVIAFNLTQPRSSRRMWLNSRVTNTLVLV
CTAIQVIICAGWLAHSSPFQYTDNKSKSGTIIVECNKGSSIAYSCTI------------------KLPGSFNEAKLITFSMLIFGAVWISFIPAYLSTTGKYMVAVEVFAILSSSSGLLACIFLPKVYIIILSPEMNKTKIKTTNKL-
>Elephant_shark.locus31.1
CTKCPLDYWSNEQRDSCIPKEIEFLSFEDDLGIVLSVVALSGTFTTIAVAAVFFYHRNTPIVRANNSELSFLLLLSLCFCFLCSLTFIGEPLVWSCMLRHTAFGISFALCISCILGKTIVVLMAFKATLPNNNRMKWLGPTQQRSIVFV
STGIQVIICTLWLATSPPFPTQS-KFRSAKLLLECDVGSVTAFSCMLGYIGLLACLCFVLAFFARKLPDNFNEAKFITFSMIIFCAVWLTFIPAYVSTPGKYTVAVEIFAILSSSTGLLGCIFVPKCYIIMLKPEQNTKKHLMGK---
>Sterlet.locus277.1
CIPCLLEYWSNKERNSCIPKEIEYLSFADIVGIISVTISILGAVLTTAVAAIFLKNKNTPIVRANNSELSFLLLISLVFCFLCAITFVGQPTAWACKLRRTSFSISFVLCLSCVLSKTVVVLMAFKATLPGNNVMKWFGPTQQRFSVFL
CTGVQVVICIVWLTVSPPFAFKNTRHYNNRIILECDLGSVTLFCCVLGYIGFLACVCFALAFLARKLPDNFNEAKFITFSMLIFCAVWITFIPAYISSPGKYTVAVEIFAILSSSFGLLLCIFTPKCYIILFKPENNSRKYLMSRAN-
>Japanese_eel.locus160.1
------------------------------
MGIILVIFSLLGVCVTVLVAVLFFIKKDTPIVKANNSELSFLLLFSLKLCFLCSLTFIGRPFDWSCMLRHTAFGITFVLCISCVLGKTIVVLMAFRATLPGSNVMKWFGPLQQRLSVLAFTLIQVLICVLWLTISPPFPNKNMKHYKEK
IILECDVGSAVGFWAVLGYIGLLSILCFVLAFLARKLPDNFNEAKFITFSMLIFCAVWITFIPAYVSSPGKFTVAVEIFAILASSFGLLFCIFAPKCYIILLKPELNSRRHVMGKMLS
>Spotted_gar.locus168.1
---------------------
MEFLEFREPLGIVLTVLAVLGAVLAVSITGAFVKHRDTPLVRANNRELSFLLLGSLALCFLCALTFIGEPSGGVCVLRQVGFAVGFVLCISCILVKTIVVLLAFRMTAPNRNVMKRFRPVHQRAVVMVTTCVQIAICVGFLLSSPPSVH
KNREAVIPKIFVECDAGSPLALFCVLAYTGFLAVINFGLAFLARNLPDNFNEAKFITFSLLVFVVVWITFIPAYISTKGKYLTAVEIFAILSSSFGLLVCIFFPKCYVILIRPELNTKNKLIGRDSK
>Reedfish.locus27375.1
--
MCPEDFWSNARRDHCVPKDSEFLSFGESLGIILTVLALIGTLVTFSIIMIFLKYMKTPIVRANNLELSFLLLFSLLLCFLCALTFMGEPSMQMCIMRQTGFAISFVLSISCILVKTLVVLLAFKMTLPNQNAMKRFRPLHQRLIIITTT
LVQIGICVGFMVSSPPSVNRNRDTVISKIILECNEGPELALFCILGYIGFLAVINFVLAFLARNLPDNFNEAKFITFSLLVFVVVWISFIPGYISTKGKYLIAVEIFAILSSSFGLLVCIFFPKCYIILLKPEMNTKNSLMGRPNK
>Japanese_eel.locus165.1
CIQCPEEYWSNAKRDQCVLKTVEFLSFQEIMGIVLVLFSLFGTCITVLVAVLFFIQKDTPIVKANNSELSFLLLFSLKLCFLCSLTFIGRPSDWSCMLRHIAFGITFVLCISCVLGKTIVVLMAFRATLPGSNVMKWFGPLQQRLSVLA
FTLIQVLICVLWLTISPPFPNKNMKHYKEKIILECDVGSAVGFWAVLGYIGLLSLLCFVLAFLARKLPDNFNEAKFITFSMLIFCAVWITFLPAYVSSPGKFTVAVEIFAILASSFGLLFCIFAPKCYIILLKPELNSRKHVMGKMPS
>Western_clawed_frog.locus431.1
-------
MWPNSKRDRCFLKPTEFLSYEDYLGITLATISTFSSMVPISVFRLFIQYKSTPIVRANNYSVSCILLVSLSFCFLCALAFIGYPQPEKCLLRQAAFGLVFTLCVSCILAKTVIVVFAFMATKPGSSLKKWTTPRVPYMIITICTFIQLT
LCLFWLTISPPFLQFNIEAKPGIIVVECNENSQFAFWCMLGYLGILASVSFTVAFLARRLPDNFNEAKLITFSMLAFLSVWVYFIPASLSAQGKYTVAMEIFAILTSSWALVFCMFLPKCFIILFRPDMNSRDNFMRKEGG
>Western_clawed_frog.locus432.1
-------
MWPNSKRDRCFLKPIEFLSYEDYLGITLATISTFSSMVPISVFRLFIQYKSTPIVRANNYSVSCILLVSLSFCFLCALAFIGYPQPEKCLLRQAAFGLVFTLCVSCILAKTVIVVFAFMATKPGSSLKKWTTPRVPYMIIICCTFIQLT
LCVFWLTISPPFLQFNIEAKPGIIVVECNENSPFAFWCMLGYLGFLATVSFTVAFLARRLPDNFNEAKLITFSMLAFLSVWVYFIPASLSAQGKYTVAMEIFAILTSSWALVFCMFLPKCFIILFRPDMNSRDNFMRKEGG
>Western_clawed_frog.locus429.1
-------
MWPNSKRDRCFLKPIEFLSYEDYLGITLATISTFSSMVPISVFRLFIHYKSTPIVRANNYSVSCILLVSLSFCFLCALAFIGYPQPEKCLLRQAAFGLVFTLCVSCILAKTVIVVFAFMATKPGSSLKKWTTPRVPYMIITICTFIQLT
LCIFWLSISPPFFQFNIEAKPGIIVVECNENSQFAFWCMLGYLGMLATVSFTVAFLARRLPDNFNEAKLITFSMLAFLSVWVYFIPASLSAQGKYTVAMEIFAIVTSTWALVFCMFLPKCFIILFRPNINSRENFMRKEGG
>Western_clawed_frog.locus433.1
CWKCPWDMWPNSARDRCLTKPIEFLSYEDYLGITLATISTFSSMVPIFIFRLFVHYKSTPIVRANNYSVSCILLVSLSFCFLCALAFIGYPQPEKCLLRQAAFGLVFTLCVSCILAKTVIVVFAFMATKPGSSLKKWTTPRVPYMIITI
CTFIQLTLCVFWLTISPPFLQFNIEAKPGIIVVECNENSQFAFWCMLGYLGMLATVSFTVAFLARRLPDNFNEAKLITFSMLAFLSVWVYFIPASLSAQGKYTVAMEIFAILTSSWALVFCMFLPKCFIILFRPDMNSRENLMRKEGG
>Western_clawed_frog.locus436.1
-------
MWPNSARDRCFPKLIEFLSYEDPLGITLASTSVLSSMVPISIFYLFIQYKSTPIVRANNYSVSCILLVSLSFCFLCALAFIGYPQPEKCLLRQVEFGLVFTLCVSCILAKTVIVVFAFMATKPGSSLKKWTTPRVPYMIITICTFIQLT
LCVFWLTIFPPFLQFNIEAKPGIIVVECNENSQFAFWCMLGYLGILASVSFTVAFLARRLPDNFNEAKLITFSMLAFLSVWVYFIPASLSAQGKYTVAMEIFAILTSSWALVFCMFLPKCFIILFRPDMNLRENLMVKTGV
>Western_clawed_frog.locus435.1
-------
MWPNSARNKCFLKLTEFLSYEDPLGLILAAVSAFSSMVPMSIFRLFFQYKSTPIVRANNYSVSCILLVSLSFCFLCALAFIGYPQPEKCLLRQAAFGLVFTLCVSCILAKTVIVVFAFMATKPGSSLKKWTTPRVPYMIITICTFIQLT
LCVFWLTIFPPFLQFNIEAKPGIIIVECNENSQFAFWCMLGYLGILASVSFTVAFLARRLPDNFNEAKLITFSMLAFLSVWVYFIPASLSAQGKYTVAMEIFAILTSSWALVFCMFLPKCFIILFRPDMNSREKLMGK---
>Western_clawed_frog.locus434.1
-------
MWPNSKRDRCFRKPIEFLSYEDYLGITLATISTFSSMVPIFIFRLFIHYKSTPTVRANNYSVSCILLVSLSFCFLCALAFIGYPQPEKCLLRQAAFGLVFTLCVSCILAKTVIVVFAFMATKPGSSLKKWTTPRVPYMIITICTFIQLT
LCIFWLSISPPFPQYNIEAKPGIIVVECNENSQFAFWCMLGYLGLLASISFIVAFLARRLPDSFNEAKLITFSMLAFLSVWVYFIPASLSAQGKYTVAMEIFAILTSSWALMFCIFLPKCFIILFRPNMNSRENILAGT--
>Western_clawed_frog.locus437.1
-------
MWPNSARDRCIVKPIEFLSYEDPLGIILATISALSSMVPIFIFRLFIHYKSTPIVRANNYSVSCILLVSLSFCFLCALAFIGYPQPEKCLLRQAAFGLVFTLCVSCILAKTVIVVFAFMATKPGSSLKKWTTPRVPYMIITICTFIQLT
LCIFWLSISPPFPQYNIEAKPGIIVVECNENSQFAFWCMLGYLGLLASVSFTVAFLARRLPDNFNEAKLITFSMLAFLSVWVYFIPASLSAQGKYTVAMEIFAILTSSWALVFCMFLPKCFIILFRPDMNLRENLMVKTGV
>Western_clawed_frog.locus438.1
-------
MWPNSARDRCILKPTEFLSYEDPLGIILAATSTLSSMVPIFIFRLFIHYKSTPIVRANNYSVSCILLVSLSFCFLCALAFIGYPQPEKCLLRQAAFGLVFTLCVSCILAKTVIVVFAFMATKPGSSLKKWTTPRVPYMIITICTFIQLT
LCIFWLSISPPFPQYNIEAKPGIIVVECNENSPFAFWCMLGYLGLLATVSFTVAFLARRLPDNFNEAKLITFSMLAFLSVWVYFIPASLSAQGKYTVAMEIFAILTSTWALVFCMFLPKCFIILFRPDMNLRENLMVKTGV
>Western_clawed_frog.locus439.1
-------
MWPNSARDRCIVKPIEFLSYEDPLGIILAATSTLSSMVPISVFRLFIHYKSTPIVRANNYSVSCILLVSLSFCFLCALAFIGYPQPENCLLRQAAFGLVFTLCVSCILAKTVIVVFAFMATKPGSSLKKWTTPRVPYMIITICTFIQLT
LCIFWLSISPPFPQYNIEAKPGIIVVECNENSPFAFWCMLGYLGLLATVSFTVAFLARRLPDNFNEAKLITFSMLAFLSVWVYFIPASLSAQGKYTVAMEIFAILTSSWALVFCMFLPKCFIILFRPDMNSRDNLMGKDRS
>Western_clawed_frog.locus441.1
-------
MWPNSARDRCIVKPIEFLSYEDPLGIILAATSFLSSMVPIFIFRLFIHYKSTPIVRANNYSVSCILLVSLSFCFLCALAFIGYPQPEKCLLRQAAFGLVFTLCVSCILAKTVIVVFAFMATKPGSSLKKWTTPRVPYMIITICTFIQLT
LCIFWLSISPPFPQYNIEAKPGIIVVECNENSQFAFWCMLGYLGFLATVSFTVAFLARRLPDNFNEAKLITFSMLAFLSVWVYFIPASLSAQGKYTVAMEIFAILTSSWALVFCMFLPKCFIILFRPDMNSRDNLMGKDRS
>Western_clawed_frog.locus443.1
-------
MWPSGARDRCISKPVEFLSYVEPLGITLAATSVFSSLVPISIFYLFIHYKSTPIVRANNYSVSCILLVSLSFCFLCALAFIGYPQPEKCLLRQAAFGLVFTLCVSCILAKTVIVVFAFMATKPGSSLKKWTTPRVPYMIITICTFVQFL
LCVIWLSASPPFPQYNIEAKPGIIVVECNENSQFAFWCMLGYLGFLASVSFTVAFLARRLPDNFNEAKLITFSMLAFLSVWVYFIPASLSAQGMYTVAMEIFAVLTSSWALVVCMFLPKCFIILFRPNMNSRENIMSKSTD
>Western_clawed_frog.locus442.1
-------
MWPSGARDRCISKPVEFLSYVEPLGITLAATSVFSSLVPISIFYLFIHYKSTPIVRANNYSVSCILLVSLSFCFLCALAFIGYPQPEKCLLRQAAFGLVFTLCVSCILAKTVIVVFAFMATKPGSSLKKWTTPRVPYMIITICTFIQLT
LCIFWLSISPPFPQYNIEAKPGIIVVECNENSPLAFWCMLGYLGFLASVSFTVAFLARRLPDNFNEAKLITFSMLAFLSVWVYFIPASLSAQGKYTVAMEIFAILTSSWALVFCMFLPKCFIILFRPNMNSRDNLMGKDRS
```

```
>Thorny_skate.locus26.1
-MKCPIDYWSNDRRDACVPKQIEFLSFGESMGVTLMAISVCGACVTGGVAAVFLRFINTPIVRANNSELSFLLLISLMLCFLCSIAFIGQPTPWSCAMRHTVFGISFVLCISCILSKTLVVLMAFKATLPSSNVMRWFGPAQQRSIVFI
STLIQVIVCAVWLATSPPFPTQNTQYQSAKIILECAVGSTLAFCCAFGYIGLLACICLVLAFLGRALPDKFNEAKFITFSMLIFFAVWLTFVPAYVSTPGKYTVAVEIFAILASSFGLLSCIFIPKCYIILIKPEENTKKQMMGRAER
>Little_skate.locus54.1
-MKCPIDYWSNDRRDACVPKQIEFLSFGESMGVTLTAISVCGACVTGGVAAVFLRFINTPIVRANNSELSFLLLISLMLCFLCSIAFIGQPTPWSCAMRHTVFGISFVLCISCILSKTLVVLMAFKATLPSSNVMRWFGPAQQRSIVFI
STLIQVIVCAVWLATSPPFPTQNTQYQSAKIILECAVGSTLAFCCAFGYIGLLACICLVLAFLGRALPDKFNEAKFITFSMLIFFAVWLTFVPAYVSTPGKYTVAVEIFAILASSFGLLSCIFIPKCYIILIKPEENTKKQMMGRAER
>Mouse.locus110.1
CIKCPEDQYPNKQKNNCLPKIKIFLSHEDTLGAVLVSLAISLSAFSAMILGLFIHYRDTPIVRANNRNLSYVLLVSLMLCFFCSLIFIGQPSTVTCVLRQMIFGVVFSVAVSAILAKTFIVVVAFTAIKPGSTLQMWMVTRLSNAIVCC
GSIIQVCICAVWLGTYPPFPDADMHSEFGQIILWCNEGSTLAFYCVLGYLGFLSSLSLLIAFLARRLPESFNEAKTITFSMLVFCTVWITFVPTYLSSKGKTMVAVEILSILASSSILLLCIFLPKCYVILLRSGGHSRKKFFK----
>marker.fugu_4_3_FP
---
CSEEFWPNPERNACFLKPVEFLSFNEVLGIILAVFSVGGACLAVITAAVFFHHRASPIVRANNSELSFLLLFSLTLCFLCSLTFIGAPSQLSCMLRHTAFGITFVLCISCILGKTVVVLMAFRATLPGSNVMKWFGPPQQRMTVVTFTS
IQVLICIVWLVVSPPFPVRNLTTYKERIILECALGSSVGFWAVLGYIGLLAAVCLVLAVLARKLPDNFNEAKMITFSMLIFCAVWITFIPAYVSSPGKFTVAVEIFAILASSFGLILCIFAPKCFIILFKPEKNSKKHLMNKK--
>Western_clawed_frog.locus965.1
CLNCPEDKWPDSRKEECLPKLIQFLSYEEILGSALACISVLFCLLTFSVFCLFIIKRKTPIVKANNRDLSYLLLISLMFGFMCSLAFIGRPNQIMCMIRQVMFAVIFSLCVSTILAKTITVVMIFSATNPDSKLKKLVGLRIPIYIVPV
CTMVQVILCIVWLTTDAPFAEFNMEAEIGIIVIECNEGSRVLFASVLGYMGLLASVSLFVAFLARKLPDTFNETKFIAFSMLVFASVWVTFIPAYLSTKGKQTVAVEIFAILSSSAGCLFCIFSPKCYTILLHPEMNNKENITGRNTQ
>Sterlet.locus189.1
------------------------------
MGVTLTVIALFGACLTIGVLAVFLHYKNTPIVRVNNSELSFFILLSLALCFLSSIAFIGEPANWSCMFRHTVFSITFSLCISCILGKTVVVLMVFKATQPGSNIMKWFGPIQQRIMISACTSVQIIICAIWLIAMPPFPSKNTKYQSSK
IILECDVGSTLAFWCVLGYIGVLACMCFVLAFLARKLPGNFNEAKYITFSMLIFCAVWIAFIPAYVSSPGKYTVAVEIFAILSSSFGLLFCIFAPKCYIILLKPEKNTKQYIMGK---
>Sterlet.locus184.1
------------------------------
MGVTLTVIALFGACLTIGVLAVFLHYKNTPMVRVNNSELSFFILLSLALCFLSSIAFIGEPANWSCMFRHTVFSITFSLCISCILGKTVVVLMVFKATQPGSNIMKWFGPIQQRIMISACTSVQIIICAIWLIAMPPFPSKNTKYQSSK
IILECDVGSTLAFWCVLGYIGVLACMCFVLAFLARKLPGNFNEAKYITFSMLIFCAVWIAFIPAYVSSPGKYTVAVEIFAILSSSFGLLFCIFAPKCYIILLKPEKNTKQYIMGK---
>Sterlet.locus188.1
CIQCPVDFWSNAERTECIPKEIEYLTY-
DEMGVTLTVIALFGACLTIGVLAVFLHYKNTPMVRVNNSELSFFILLSLALCFLSSIAFIGEPANWSCMFRHTVFSITFSLCISCILGKTVVVLMVFKATQPGSNIMKWFGPIQQRIMISACTSVQIIICAIWLIAMPPFPSKNTKYQS
SKIILECDVGSTLAFWCVLGYIGVLACMCFVLAFLARKLPGNFNEAKYITFSMLIFCAVWIAFIPAYVSSPGKYTVAVEIFAILSSSFGLLFCIFAPKCYIILLKPEKNTKQYIMGK---
>Sterlet.locus183.1
CIQCPVDFWSNAERTECIPKEIEYLTY-
DEMGVTLTVIALFGACLTIGVLAVFLHYKNTPMVRVNNSELSFFILLSLALCFLSSIAFIGEPANWSCMFRHTVFSITFSLCISCILGKTVVVLMAFKATQPGSNIMKWFGPIQQRIMISACTSVQIIICAIWLIAMPPFPSKNTKYQS
SKIILECDVGSSLAFWCVLGYIGVLACMCFVLAFLARKLPGNFNEAKYITFSMLIFCAVWIAFIPAYVSSPGKYTVAVEIFAILSSSFGLLFCIFAPKCYIILLKPEKNTKQYIMGK---
>Sterlet.locus185.1
------------------------------
MGVTLTVIALFGACLTIGVLAVFLHYKNTPMVRVNNSELSFFILLSLALCFLSSIAFIGEPANWSCMFRHTVFSITFSLCISCILGKTVVVLMAFKATQPGSNIMKWFGPIQQRIMISACTSVQIIICAIWLTAMPPFPSKNTKYQSSK
IILECDVGSTLAFWCVLGYIGVLACMCFVLAFLARKLPGNFNEAKYITFSMLIFCAVWIAFIPAYVSSPGKYTVAVEIFAILSSSFGLLFCIFAPKCFTILLKPEKNTKQYIMGK---
>Sterlet.locus187.1
CIQCPVDFWSNAERTECIPKEIEYLTY-
DEMGVTLTVIALFGACLTIGVLAVFLHYKNTPMVRVNNSELSFFILLSLALCFLSSIAFIGEPANWSCMFRHTVFSITFSLCISCILGKTVVVLMAFKATQPGSNIMKWFGPIQQRIMISACTSVQIIICAIWLIAMPPFPSKNTKYQS
SKIILECDVGSTLAFWCVLGYIGVLACMCFVLAFLARKLPGNFNEAKYITFSMLIFCAVWIAFIPAYVSSPGKYTVAVEIFAILSSSFGLLFCIFAPKCYTFSFY---------------
>Sterlet.locus182.1
------------------------------
MGVTLTVIALFGACLTIGVLAVFLHYKNTPMVRVNNSELSFFILLSLALCFLSSIAFIGEPANWSCMFRHTVFSITFSLCISCILGKTVVVLMAFKATQPGSNIMKWFGPIQQRIMISACTSVQIIICAIWLIAMPPFPSKNTKYQSSK
IILECDVGSTLAFWCVLGYIGVLACMCFVLAFLARKLPGNFNEAKYITFSMLIFCAVWIAFIPAYVSSPGKYTVAVEIFAILSSSFGLLFCIFAPKCYIILLKPEKNTKQYIMGK---
>Mouse.locus78.1
CVRCPDDKYANLEKTHCLQRAVSFLAYEDPLGIALGCMALFLSALTVLVLVTFVKYKDTPIVKANNRILSYILLISLVFCFLCSLLFIGHPNQATCILQQTTFGVFFTVAISTVLAKTITVLMAFKLTTPGRRMRGMLASGAPNLVIPI
CTLIQLVFCGIWLVTSPPFIDRDTQSEYGKTIIICNKGSVIAFHFVLGYLGSLALGSFTVAFLARNLPDRFNEAKFLTFSMLVFCSVWITFLPVYHSTRGTVMVVVEVFSILASSAGLLGCIFLPKCCVILVRLDSNFLQKYEHKLPS
>Mouse.locus75.1
CVSCTGDKYANLEKTHCLQRAVSFLAYEDPLGMSLGCIALFLSALTSLVLVTFVKYKDTPIVKANNRILSYILLISLVFCFLCSLLFIGHPNQATCILQQTTFGVFFTVAISTVLAKTITVLMAFKLTTPGRRMRGMLASGAPNLVIPI
CTLIQLVFCGIWLVTSPPFIDRDTQSEYGKTIIICNKGSVIAFHFVLGYLGSLALGSFTVAFLARNLPDRFNEAKFLTFSMLVFCSVWITFLPVYHSTRGTVMVVVEVFSILASSAGLLGCIFLPKCCAILVRLDSNFLQKYKDKLPS
>Mouse.locus74.1
CVRCPDDKYANLEKTHCLQRAVSFLAYEDPWGMALGCMALFLSALTVLVLVTFVKYKDTPIVKANNRILSYILLISLVFCFLCSLLFIGHPNQATCILQQTTFGVFFTVAISTVLAKTITVLMAFKLTTPGRRMRGMLASGAPNLVIPI
CTLIQLFLCGIWLVTSPPFIDRETQSEYGKTIIICNKGSVIAFHFVLGYLGSLALGSFTVAFLARNLPDRFNEAKFLTFSMLVFCSVWITFLPVYHSTRGTVMVVVEVFSILASSAGLLGCIFLPKCCVILVRLDSNFLQKYKDKLPS
>Mouse.locus79.1
CVRCPDDKYANLEKTHCLQRAVSFLAYEDPLGMALGCMALFLSALMVLVLVTFVKYKDTPIVKANNRILSYILLISLVFCFLCPLLFIGHPNLATCILQQTTFGVFFTVAISTVLAKTITVLMAFKLTTPGRRMRGMLASGAPNLVIPI
CTLIQLVLCGIWLVTSPPFIDRDTQSEYGKTIIICNKGSVIAFHFVLGYLGALALGSFTVAFLARNLPDRFNEAKFLTFSMLVYCSVWITFLPVYHSTRGAVMVVVEVFSILASSAGLLGCIFLPKCCVILLD---------------
>Mouse.locus83.1
CVRCPDDKYANLEKTQCLQRVVSFLAYEDPLGMTLGCMALTFSALTVLVLVIFVKYKDTPIVKANNRIVSYILLISLVFCFLCSLLFIGHPTQATCILQQTTFGVFFTVAISTVLAKTITVLMAFKLTTPGGRMRGMLASGAPNLVIPI
CTLIQLVLCGIWLVTSPPFIDRDIQSEHGKTIIICNKGSVIAFHFVLGYLGALALGSFTVAFLARNLPDRFNEAKFLTFSMLVFCSVWITFLPVYHSTRGTVMVVVEVFSILASSAGLLGCIFLPKCCVILLRPDLTFLQKYKDKLPS
>Mouse.locus77.1
CMRCPDDKYANLEKTHCLQRAVSFLAYEDPLGMALGCTALSFSALTVLVLVTFVKYKDTPIVKANNRILSYILLISLVFCFLCSLLFIGHPNQATCILQQTTFGVFFTVAISTVLAKTITVLMAFKLTTPGRRMRGMLASGAPNIVIPI
CTLIQLVLCGIWLVTSPPFIDRDMQSEHGKTIIICNKGSVIAFHFVLGYLGALALGSFTVAFLARNLPDRFNEAKFLTFSMLVFCSVWITFLPVYHSTQGTVMVVVEVFSILASSAGLLGCIFLPKCCVLLRIQNSNFLHKYKHELHS
>Anole_lizard.locus102.1
CFQCPEDHYPNKNRDFCIPKSLTFLTFEEALGTSLASSALLLFCVTSVVLGIFLKHRDTPIVKANNRTLTYTLLLSLLLSFLCALLFIGKPQKMTCLFRQTAFGIIFSVAISSVLAKTITVILAFQATKPESKLRKWLGKRMATSIVLS
SLLIQATICTVWLSTFPPFPDLDVNSMAEEVILKCNEGSITMFYCLLGFMGFLSICSFTSAFFARKLPDSFNEAKFITFSMLVFCSVWISFVPTYLSSKGKYMVAVEIFSILASSAGLLGFIFFPKCYIIILRPDMNTKEQVMKRKH-
>Mouse.locus307.1
CMKCPVDEYANTDQTNCFKKVVSFLDYEEPLGMTLAVLAVFFSSLTVIILCIFLKHRDTPIVKANNETLSYVLLTSLIFCFICSLLYIGYPTMVSCILQQTTFAIVFTVAASCVLAKTITVVLAFKITVPGRRLRWLLLSGAPNYIIPI
CTTIQMILCGIWLGTSPPFVDVDLHMIHGHIIIVCNKGSVIAFYCVLGYMGSLALSSFTVAFLARNLPDTFNEAKLLTFSMLVFCSVWITFLPVYHSTKGKTMVAVEVFCILASSAGMLICIFLPKCYIILLRPQINSFHEFRKISAK
>Reedfish.locus27727.1
------------------------------
MGVVLTIMSISGASITLFVITVFLYYKDTAIVKANNSELSFLLLFSLTLCFLCSLTFIGQPLSWSCMLRHTAFGISFVLCISCVLGKTIVVLMAFRATLPGKNIMKYFGPLQQRAGIFLCTLVQILICILWLILSPPFPTKTS-
HYSGRVILECNVGSVLGFYSVLGYIGFLACMCFFLAFLGRKLPDNFNEAKFITFSMLIFCAVWITFIPAYVSSPGKYTVAVEIFAIWSSSFSLLVCIFAPKCYIILVRPEKNTKKHLMEKPAA
>Reedfish.locus40470.1
CMSCPISYRSNKEKNQCILKDIEFLSFEEIMGSLLVLFALLGALMTVCVAVIFILYIDTPVVRANNSELSFLLLFSLILCFLCSLTFIGQPSDWSCMLRHTIFGITFALCISCILAKTVVVLMAFRATLPGSNVMKWFGPCQQRMSVAS
FTLTQVIICTFWLFLSPPFPHENFMSYKEKIILECEVGSMIAFCVVLGYIGFLSAVCFVLAFLARKLPDNFNEAQLITFSMLIFCSVWITFIPAYISSPGKYTVAVEIFAILASSFGLLFCIFFPKCYVILLKPEQNNRKHIMGKMPS
>Reedfish.locus40462.1
CMSCPISYRSNQEKNQCILKDTEFLSFEEIMGSLLVLFALLGALMTVCVAVIFILYIDTPVVRANNSELSFLLLFSLILCFLCSLTFIGQPSDWSCMLRHTIFGITFALCISCILAKTVVVLMAFRATLPGSNVMKWFGPCQQRMSVAS
FTLTQVIMCTFWLFLSPPFPHENFMSYKEKIILECEVGSMIAFCVVLGYIGFLSAVCFVLAFLARKLPDNFNEAQLITFSMLIFCSVWITFIPAYISSPGKYTVAVEIFAILASSFGLLFCIFFPKCYVILLKPEQNTRKHIMGKMPS
>Reedfish.locus40460.1
-MSCPISYRSSKEKNQCILKDTEFLSFEEIMGSLLVLFSLLGALMTVCVAVIFILYIDTPVVRANNSELSFLLLFSLILCFLCSLTFIGQPSEWSCMLRHTIFGITFALCISCILAKTVVVLMAFRATLPGSNVMKWFGPCQQRMSVAS
FTLTQVIICTFWLFISPPFPHENFTSYKEKIILECEVGSIVAFCAMLGYIGFLSAVCFVLAFLARKLPDNFNEAQLITFSMLIFCSVWITFIPAYISSPGKYTVAVEIFAILASSFGLLFCIFVPKCYVILLKPEQNTRKHLMGKMPS
>Reedfish.locus40468.1
-MSCPISYRSSKEKNQCILKDTEFLSFEEIMGSLLVLFALLGALMTVCVAVIFIFYIDTPVVRANNSELSFLLLFSLILCFLCSLTFIGQPSEWSCMLRHTIFGITFALCISCILAKTVVVLMAFRATLPGSNVMKWFGPCQQRMSVAS
FTLTQVIICTFWLFLSPPFPHENFTSYKEKIILECEVGSIVAFCVVLGYIGFLSAVCFVLAFLARKLPDNFNEAQLITFSMLIFCSVWITFIPAYISSPGKYTVAVEIFAILASIF--------------------------------
>Reedfish.locus40458.1
CMSCPIRYRSSKEKNQCILKDTEFLSFEEIMGSLLVLFALLGALITISVAVIFILYNETPVVRANNSELSFLLLFSLILCFLCSLTFIGQPSDWSCMLRHTIFGITFALCISCILAKTVVVLMAFRATLPGSNVMKWFGPCQQRLSVVS
FTLTQVLICTFWLFLSPPFPHENFASFKEKIILECEVGSMVAFSAMLGYIGFLSAVCFILAFLARKLPDNFNEAQLITFSMLIFCSVWITFIPAYISSPGKYTVAVEIFAILASSFGLLFCIFFPKCYVILLKPEQNTRKHIMGKMPS
>Reedfish.locus40464.1
CMSCPISYRSNQEKNQCILKDTEFLSFEEIMGSLLVLFALLGALMTISVAVIFILYIDTPVVRANNSELSFLLLFSLILCFLCSLTFIGQPSEWSCMLRHTIFGITFALCISCILAKTVVVLMAFRATLPGSNVMKWFGPCQQRMSVAS
FTLTQVIICTFWLFLSPPFPHKNFTSYKDKIILECEVGSIVAFCAVLGYIGFLSAVCFILAFLARKLPDNFNEAQLITFSMLIFCSVWITFIPAYISSPGKYTVAVEIFAILASSFGLLFCIFFPKCYVILLKPEKNTRKHLMRKMPS
>Reedfish.locus40437.1
-MSCPLDYRSNQDKNQCILKDTEFLSFEEIMGSLLILFSLLGALMTISVAVIFILYIDTPVVRANNSELSFLLLFSLILCFLCSLAFIGQPSDWSCMLRHTIFGITFALCISCILAKTFVVLMAFRATLPGSNVMKWFGPCQQRMSVAS
FTIIQVIICTFWLFLSPPFPHKNFTSYKEKIILECEVGSMVAFCVMLGYIGFLSAVCFILAFLARKLPDNFNEAQLITFSMLIFCSVWITFIPAYISSPGKYTVAVEIFAILASSFGLLFCIFVPKCYVILLKPEQNTRKHLMGKVPS
>Reedfish.locus40443.1
-MSCPLDYRSNQDKNQCILKDTEFLSFEEIMGSLLILFSLVGALMTISVTVIFILYVDTPVVRANNSELSFLLLFSLILCFLCSLAFIGQPSDWSCMLRHTIFGITFALCISCILAKTFVVLMAFRATLPGSKVMKWFGPCQQRMSVAS
FTIIQVIICTFWLFLSPPFPHKNFTSYKEKIILECEVGSMVAFCVMLGYIGFLSAVCFILAFLARKLPDNFNEAQLITFSMLIFCSVWITFIPAYISSPGKYTVAVEIFAILASSFGLLFCIFVPKCYVILLKPEQNTRKHLMGKVPS
>Reedfish.locus40448.1
CMSCPLDYRSSQDKNQCILKDTEFLSFEEIMGSLLVLFSLLGTITTICVSVIFILYRDTPVVRANNSELSFLLLFSLILCFLCSLTFIGQPSNWSCMLRHTVFGVTFALCISCILAKTVVVLMAFRATLPGSNVMKWFGPFQQRMSVAS
FTLTQVIICMFWLVLSPPFPHKNFTSYKEKIILECEVGSIVAFCTVLGYIGFLSSVCFGLAFLARKLPDNFNEAQLITFSMLIFCSVWITFIPAYISSPGKYTVAVEIFAILASSFGLLFCIFFPKCYVILLKPEENTRKHLMGKMPS
>Reedfish.locus40523.1
-MSCPLDFRSSQEKTQCILKDTEFLSFEDIMGSLLVFLSFLGTLMTFCVVIIFILYHDTPVVRANNSELSFLLLFSLILCFLCSLTFIGQPSEWSCMLRHTVFGITFALCISCILAKTVVVLIAFRATLPGSNVMKWFGPCQQRMSVAS
FTLTQGLICMLWLLLSPPLPHKNVKSYKEKIILECEVGSMVAFCVVLGYIGFLSAVCFILAFLARKLPDNFNEAQLITFSMLIFCSVWITFIPAYISSPGKYIVAVEIFAILPSIFGLLFCIFVPKCYVIIFKPDQNTRKHIMGKTLS
>Reedfish.locus27552.1
-MSCPLDFRSSQEKTQCILKDTEFLSFEDIMGSLLVFLSFLGTLMTFCVVIIFILYHDTPVVRANNSELSFLLLFSLILCFLCSLTFIGQPSEWSCMLRHTVFGITFALCISCILAKTVVVLMAFRATLPGSNVMKWFGPCQQRMSVAS
FTLTQGLICMLWLLLSPPLPHKNVKSYKEKIILECEVGSMVAFCVVLGYIGFLSAVCFILAFLARKLPDNFNEAQLITFSMLIFCSVWITFIPAYISSPGKYTVAVEIFAILASSFGLLFCIFVPKCYVIIFKPDQNTRKHIMGKTLS
>Reedfish.locus40451.1
CMSCPLDYRSSQEKTQCILKDTEFLSFEEIMGSLLVLFSLLGTLMTISVAVIFILYIDTPVVRANNSELSFLLLFSLILCFLCSLTFIGQPSDWSCMLRHTIFGITFALCISCILAKTVVVLMAFRATLPGSNVMKWFGPCQQRMSVVS
FTLTQGLICMFWLLLSPPLPHKNFTSYKEKIILECEVGSMVAFCTVLGYIGFLSAVCFILAFLARNLPDNFNEAKLITFSMLIFCSVWITFIPAYISSPGKYTVAVEIFAILASSFGLLFCIFFPKCYVILVKPEQNTRKHIMGKMPA
>Reedfish.locus27546.1
-MSCPLDYRSSQEKSQCILKDTEFLSFEEIMGSLLVLFSLLGAFMTFCVAAIFILYIDTPVVRANNSELSFLLLFSLILCFLCSLMFIGQPSDWSCMLRHTIFGITFALCISCILAKTVVVLMAFRATLPGSNVMKWFGPCQQRMSVAS
FTLTQVIICLLWLLLSPPFPHKNFTSYKEKIILECEVGSMVAFCAVLGYIGLLSTVCFILAFLARKLPDNFNEAQLITFSMLIFCSVWITFIPAYISSPGKYTVAVEIFAILASSFGLLFCIFVPKCYVILLKPEQNTRRHIMGKMPS
>Reedfish.locus27548.1
CMSCPLDYRSSQDKTQCILKDIEFLSFEEIMGSLLVLFALLGALMTVCVAVIFILYIDTPVVRANNSELSFLLLFSLILCFLCSLTFIGQPSDWSCMLRHTIFGITFALCISCILGKTVVVLMAFRATLPGSNVMKWFGPCQQRMSVAS
FTFTQVLICMFWLLLSPPFPNKNFKSYKEKIILECEVGSMVAFSAVLGYIGFLSTVCFILAFLARKLPDNFNEAQLITFSMLIFCSVWITFIPAYISSPGKYTVAVEIFTILASSFGLLFCLFVPKCYVILLKPEQNTRKHLMGKMPS
>Reedfish.locus27551.1
------------------------------
MGSLLVLFSLLGTFMTICVSVIFILYRDTPVVKANNSELSFLLLFSLILCFLCSLPFIGQPSDWSCMLRHIGFGITFALCISCILAKTVVVLMAFRATLPGSNVMKWFGPSQQRMSVASFTLIQVLICMFWLLFSPPIPHKNFKSYKEK
IILECEVGSVLAFCAVLGYIGFLSAVCFILAFLARKLPDNFNEAQLITFSMLIFCSVWITFIPAYISSPGKYTVAVEIFAILASSFGLLFCIFVPKCYVILLKPEQNNRKHLMGKMPS
```

```
>Reedfish.locus40436.1
CFSCPLDYRTNLARTQCILKDIEFLSFEEVMGSLLVLFSLLGAFMTIFVAVIFILYRDTPVVRANNSELSFLLLFSLILCFLCSLPFIGQPSDWSCMLRHTAFGITFALCISCILAKTVVVLMAFRATLPGSNVMKWFGPPQQRMSVAS
FTLTQVIICTVWLFLSPPFPHRNVKSFKEKIILECEVGSMVAFCTVLGYIGFLSAVCFILAFLARKLPDNFNEAQLITFSMLIFCSVWITFIPAYISSPGKYTVAVEIFAILASSFGLLFCIFVPKCYVILLKPDQNTRKHIMGKMPS
>Caecillan.locus34702.1
--------------------------------
MTLIALSVFGVLVVIAVTVVYILHRDTPLVMANDRELSFVIQFSLVITLLTSILFVGKPEEWSCMARQTTLSLGFSLCLSCVFGKTIVLMLIARAAKPEKTGEIIIRPIHQKCIALIGLTLEIGICIAYLILIPPSVYKNMESQNVKII
FECNEGSIEFLCSMFGFDIFLALLAFLAAFLARKLPDNFNEAKFVTFGMLVFFIVWISFVPAYLSTRGKFKVAVEIFAILASSFGLLGCIFAPKCYIILLKPERNTEEIVGGRARM
>Coelacanth.locus251.1
CTECPGDQWSNEKRDKCIPKPIEFLTYEETLGATLASTSILFSLIPGIILSILIWYHDTPIVKANNRELSYLLLIALMLCFLCSLVFIGQPLTVTCMLRQTAFGIIFAFCVSCVLAKTIMVVVAFNATKPNSSLKNWVGYKLPNTIVLV
CTLFQVIICLAWLCSSPPFPEQNMKSEPEKIIIECNEGSTVAFWCMLGYMGLLATVSFLMAFLARNLPDSFNEAKFITFSMLVFVSVWLSFIPAYLSTRGKYMVAVEVFAILASSVGLLACIFFPKCYIIILRPDMNSRDYLMGKGTF
>Brownbanded_bambooshark.locus1585.1
----------------------
MFLSFFETMGIILVTLALFGACTTVGVFTIFYIYRHTPIVKANNSELSFLLLFALTLCFLCSITFIGTPSVWSCMLCHVAFGISFVLCISCVLSKTIVVVMAFKATFPSNNMMKWFGPRQQRLMVYILTLVQCVICTTWLIVFPPYPLK
STSYSPEIIMFECNVGSSLAFYSVLGYIGFLSCACFVVAFLARQLPDNFNEAKHITFSMLIFCAVWITFIPAYISSPGKYAVVVEVFAILASSFGLLICIFVPKCYIILLKKENNIKRNMMGSVTS
>Brownbanded_bambooshark.locus1588.1
------------------------------
MGIILVTLALTGACTTVAVLAIFYIYRHTPIVKANNSELSFLLLFALTLCFLCSITFIGTPSVWSCMLCHVVFGISFVLCISCVLSKTIVVVMAFKATFPSNNMMKWFGPRQQRLIVYILTLVQCAICTTWLIAFPPYPLKNTSHSHEI
IVFECNIGSSLAFYSVLGYIGFLSCVCFVVAFLARQLPDNFNEAKHITFSMLIFCAVWITFIPAYISSPGKYAVVVEVFAILASSFGLLICIFTPKCYIILLKKENNTKRNMMGSVAS
>Brownbanded_bambooshark.locus1564.1
CIKCPLEFKSNERQDMCIPKDIEFLSFFETMGIILVTLALFGACTTVGVFAIFYIYRHTPIVKANNSELSFLLLFALTLCFLSSITFIGTPSVWSCMLCHVAFGISFVLCISCVLSKTIVVVMAFKATFPGNNMMKWFGPRQQRLMVYL
LTLVQCAICTTWLIVFPTYPLKNTSHSHKIIVFECNVGSSLAFCCVLGYIGFLSCVCFVVAFLARKLPDNFNEAKHITFSMLIFCAVWITFIPAYISSPGKYAVAVEVFAILASSFGLLICIFAPKCYIILLKKENNTKRNMVGSVAP
>Brownbanded_bambooshark.locus1570.1
CIKCPLEFKTNERQDQCVPKEIEFLSFFETLGIILVILALLGGCTTIGTFAIFYIYRHTPIVKANNSELSFLLLFALTLCFLSSITFIGEPSIWSCMLCHVAFGISFVLCISCVLSKTIVVVMAFKSTLPGNNMMKWFGPRQQRLMVYI
LTLVQCAICTTWLIVFPPYPMKNTSHSNEIIVLECNVGSSSAFYCVLGYIGFLSGVCFVVAFIARQLPDNFNEAKHITFSMLIFCAVWITFIPAYISSPGKYAIAVEVFAILASSFGLLICIFAPKCYIILLKKEDNTKRNMMGSVAS
>Brownbanded_bambooshark.locus1562.1
CIKCPLEFKSNERRDQCIPKDIEFLSFFETLGIILVTLALFGACTTVGVCAIFYVYRCTPIVKANNSELSFLLLFALTLCFLCSITFIGEPSVWSCMLCHVAFGISFVLCISCVLSKTIVVVMVFKATLPGNNMMKWFGPRQQRLIVYI
LTLVQCAICTTWLIVFPPYPLKNTSHSNEIIVFECNVGSSLAFYCVLGYIGFLSCVCFVVAFLARKLPDNFNEAKHITFSMLIFCAVWITFIPAYISSPGKYAVAVEVFAILASSFGLLICIFVPKCYIILLKKENNTKRCIMGSVVS
>marker.cichlid_3-2aa
---
CPKEYWSNERKDKCIPRTIEFLKYQEPMGIAVTFVSLLGASVSLATMVVFVHNKETPVMKASNFELSSLLLFSLFLSFLCPLTFLGKPTVWSCMLRHTTFGVTFALCISCVLGKTIVVVTAFKATFPGNKVAEKFGHAQQRIIVCSCTL
IQVVICALWLTSNPPHPHKLFTSSNNMIVLECNTGSEFAFYAVLGYIGILAIICLILAFMARKLPDNFNEAKLITFSLLIFCAVWITFIPAYISSPGKFTVAVEIFAILSSAFGLLSSIFAPKCYIIFLKPEKNTKKHIMGKT--
>Coelacanth.locus361.1
CMKCPDDYWSNERKDECIPKTIEFLSYEEPMGATLVAISISWAIIYTTVLCMFIKYRDTPVVKANNRDLSYLLLIALVLCFLSSLIFIGQPKVVTCMLRQIAFGIIFALCVSCVLAKTILVVIAFNATKPNSNLKKWVGNKLPNSIVF-
-------ICIAWLTTSSPFPEENTKSQNGVIILECNVGSTAAFWCVLGYMGLLAIVSFIVAFLARNLPDSFNEAQFITFSMVVFVSVWLAFIPSYLSTRGKYMVAVEIFAILASSAGLLYCIFFPKCYIIILRPDMNTKEYLMGKGKP
>Coelacanth.locus362.1
-MKCPDYYWSNERQDECIPRTTEFLSYEEPMGAALVAISIAWAVISTTVLCIFIKYKNTPIVKANNRDLSYLLLIALLLCFLSSLIFIGQPKVVTCMLRQTVFGIIFALCVSCVLAKTILVVIAFNATKPNSNLKKWVGNKLPNGIVFI
CTAIQVIICIAWLTISSPFPEENIKSKNGVIILECNEGSTAAFWCVLGYMGLLAIVSFIVAFLARNLPDSFNETQFITFSMVVFVSVWLAFIPAYLSTRGKYMVAVEIFAILASSAGLLGFIFFPKCYIIVLKPDMNTKEYLMGKGKC
>Coelacanth.locus370.1
-MTCPDDYWPNERQDECIPKTIEFLSYEEPMGATLAAISITWAITSATVLCIIIKYRDTPIVKANNRDLSYLLLIALVLCFLSSLIFIGQPKVVTCMLRQTAFGIIFALCVSCVLAKTILVVITFNATKPNSNLKKWVGNKLPNGIVFI
CTTTQVIICIAWLTTSSPFPEENTTSQDGVIILECNEGSTTAFWCVLGYMGLLAIVSFIVAFLARNLPDSFNEAQFITFSMVVFVSVWLAFIPAYLSTRGKYMVAVEIFAILASSAGLLGCIFFPKCYIIILRPEVNNRNISWEKVNL
>Coelacanth.locus199.1
-MKCPDGYWPNEKQDECIPKIIEFLSYEEPMGAILVATSISWAIISTTVLCIFIKYRDTPIVKANNRDLSYLLLIALALCFLSSLIFIGQPNTVTCMLRQTIFGIIFALCVSCVLAKTILVVIAFNATKPNSNLKKWVGNKLPNSIVFI
CTAIQVIICIAWLTTSSPFPEENTKSKKGVIILECNEGSTTAFWCVLGYMGLLAIVSFIVAFLARNLPDSFNEAQFITFSMVVFVSVWLAFIPAYLSTRGKYMVAVEIFALLASSAGLLGCIFFPKCYIIILRPEVNTREYLIGKGKS
>Coelacanth.locus319.1
------------------------------
MGATLAAISISCVVTSITVLCIFIKNKDTPIVKANNRELSYLLLLALALCFLSSLIFIGQPKTVTCMLRQAAFGIIFALCVSCVLAKTIMVAIAFNATKPDSNLKKWVGAKLPNTIVFVCTGIQIIICIAWLATSPPFPVQNMKSQIGV
IIIECNEGSATAFWCVLGYMGLLAILSFTVAFLARNLPNSFNEAKFITFSMLIFVSVWLAFIPAYLSTQGKYMAAVEIFAILASSAGLLGCIFFPKCYIIILRPDINTKEYLMGKVTL
>marker.medaka_10_1_F
---CPLGYWSNEDHNKCVPKVIEFLSYEETMGTLLTAFSLFGAGLTLVLLFVFFWFRHTPLVKASNSELSFLLLFSLTLCFLCSLTFIGRPTDWSCMLRHVAFGIAFALCISCILTKTITVVIAFKARTPGNRVPQCLAS-
HQRMGVVGGTFLQVLVCVAWLAQAPPFPHKNTIYALERIILECNLGSSIGFWVVLGYIGLLAVLCLILAFLARKLPDNFNEAKFITFSMLIFSAVWVTFIPAYVSSPGKFTVAVEIFAILASSFGLLFCIFAPKCYILLLKPERNTKSH
IMGRD--
>marker.fugu_10_1_F
---CPLEFWSNEDHSQCVPKVIEFLSFEETMGALLAAVSLFGAALTSLVFCVFFRFRHTPLVKASNSELSFLLLFSLTLCFLCSLTFIGRPSRWSCVLRHTAFGITFALCMSCVLAKTVAVLFAFTAKRPGNTVFYCSVP-
LQRTSVFACITLQVIICVLWLTLAPPHPHKNTAHAKERIILECNLGSPVWFWVVLGYIGLLAVICFILAFLARKLPDNFNEAKFITFSMLIFCAVWVTFIPAYVSSPGKFTVAVEIFAILASSFGLLFCIFAPKCYILILKPEKNTKKH
MMGRN--
>marker.cichlid_10-1aa
---CPLECWSDEDRSHCVPKVIEFLSFGETMGGLLAAFSLFGASLTLAVSCVFFRFRHTPLVKASNSELSFLLLFSLTLCFLCSLTFIGQPTEWSCMLRHTAFGASFALCMSCILAKTITVVIAFKATRPAKTVPQC-
SAALQRTSVLSFTLLQLLICVLWLIFAPPFPYKNTVHATEKIILECNLGSPLGFWAVLGYIGLLSLLCFILAFLSRKLPDNFNEAKFITFSMLIFCAVWVTFIPAYVSSPGKFTVAVEIFAILASSFGLFFSIFAPKCYILLVKPERNT
KRHMMGRN--
>Zebrafish.locus489.1
------------------------------MGILLTAFSLTGVTLTIAVAIVFYKFIDTPLVKASNTELSFLLLFSLSLCFLCSLTFIGRPTEGSCMLRHSSFGVTFALCMSCVLTRTIAVVMAFKTTVPGSGLPHCSLP-
LQRISVFCCTVFQVMICILWLALARPMPYKNSMYSLDKVILECDLGSAIGFWAVLGYIGLLSVLCFFLAFLARKLPDNFNEAKFITFSMVIFCAVWITFIPAYISSPGKLTVAVEIFAILASTFGLLFCIFTPKCYIIIFKPEQNTRKH
IMGKT--
>marker.zebrafish_10_1_F
---CPLEYWSNKNHSICVLKKVEFLSFEENMGILLTAFSLTGVTLTIAVAIVFYKFIDTPLVKASNTELSFLLLFSLSLCFLCSLTFIGRPTEGSCMLRHSSFGVTFALCMSCVLTRTIAVVMAFKTTVPGSGLPHCSLP-
LQRISVFCCTVFQVMICILWLALARPMPYKNSMYSLDKVILECDLGSAIGFWAVLGYIGLLSVLCFFLAFLARKLPDNFNEAKFITFSMVIFCAVWITFIPAYISSPGKLTVAVEIFAILASTFGLLFCIFTPKCYIIIFKPEQNTRKH
IMGKT--
>Japanese_eel.locus400.1
------------------------------MGIVLTVFSVTGACLTSGIGLIFFLFKKTPIVKANNSELSFLLLFSLKLCFLCSLTFIGRPSEWSCMLRHTAFGITFALCISCILAKTIVVVMAFKATVPGSNVSQCTVPL-
QRFSVFGSTLPQVVICTLWIALAPPVPHSR---
FTEKIILECDVGSAVGFWAVLGYIGLLSILCFVLAFLARKLPDNFNEAKFITFSMLIFCAVWITFIPAYVSSPGKFTVAVEIFAILASSFGLLFCIFLPKCYIILFRPEQNTRRHLMGKKGM
>Cloudy_catshark.locus76.1
CIKCPLEYWSNPNKDKCFPKEIEFLSFEETLGIVLVALALGGICGTLAIAGVFLQYKETPIVKANNSELSFLLLFALTLCFLCSLTFIGQPSFWSCMLQRVSFGITFVLCISCVLTKTILVLMAFTATLPNNNLMRWFGPTQQRFGVFG
LTFIQGFICTIWLSIAPPFPMKNTSYYREIIILECHVGSTMAFYCVSGYIALLSCVCFVLAFLARKLPDNFNEAQCITFSMLIFCVVWIAFIPAYVSSPGKYTVAVEVFAILASSFALLLCIFVPKCTIILLKPETNTRKHVMSKLPS
>marker.cichlid_15-3aa
---
CPSEFWSNVERIACIPRQLDFLSFNETLGITLTTAAVSGVSVTTTVFVVFLFYRQTPMVRANNSELSFLLLLSLKLCFLCSLVFIGRPSVWSCRFQQAAFGISFVLCVSCLLVKTIVVLAVFRSARPGATLMKWFGPGQQRGSVCFFTC
IQVIICVIWLSLSPPRPQSDFGFQGSKVTLECTMESVIGFSLVLGYIGLLACTCLLLAFLARKLPDNFNEAKLITFSMLIFCTVWVSFVPAYISSPGKYVVAVEVFAILASSYGLLLCIFAPKCFIILLRPEKNTKKYLMAR---
>Mouse.locus177.1
CMNCPEYQYANTEQNKCIQKGVTFLSYEDPLGMALALMAFCFSAFTALVLCVFVKHHDTPIVKANNRSLSYLLLMSLMFCFLCSFFFIGLPNRAICVLQQITFGIVFTVAVSTVLAKTVIVVLAFKVTDPGRRLRNFLVSGTPNYIIPI
CSLLQCVLCAIWLAVSPPFVDIDEHTLHGHIIIVCNKGSDTAFYCILGYLACMALGSFSLAFLAKNLPDTFNEAKFLTFSMLVFCSVWVTFLPVYHSTKGKHMVAVEIFSILASSAGILGCIFVPKIYIILMRPERNSTQKIREKSYF
>Mouse.locus151.1
CMNCPEYQYANTEQNKCIQKGVTFLSYENPLGMALALMAFCFSAFTALVLCVFVKHHDTPIVKANNRSLSYLLLMSLMFCFLCSFFFIGLPNRAICVLQQITFGIVFTVAVSTVLAKTVIVVLAFKVTDPGRRLRNFLVSGTPNYIIPI
CSLLQCVLCAIWLAVSPPFVDIDEHTLHGHIIIVCNKGSDTAFYCILGYLACMALGSFSLAFLAKNLPDTFNEAKFLTFSMLVFCSVWVTFLPVYHSTKGKHMVAVEIFSILASSAGILGCIFVPKIYIILMRPERNSTQKIREKSYF
>Mouse.locus128.1
CMNCPEYQYANTEQNKCIQKGVTFLSYEDPLGMALALMAFCFSAFTALVLCVFVKHHDTPIVKANNRSLSYLLLMSLMFCFLCSFFFIGLPNRAICVLQQVTFGIVFTVAVSTVLAKTVTVVLAFKVTDPGRRLRNFLLSGTPNYIIPI
CSLLQCVLCAIWLAVSPPFVDIDEHTLHGHIIIVCNKGSDTAFYCILGYLACMALGSFSLAFLAKNLPDTFNEAKFLTFSMLVFCSVWVTFLPVYHSTKGKHMVAVEIFSILASSAGILGCIFVPKIYIILMRPERNSTQKIREKSYF
>Mouse.locus174.1
CVNCPEYQYANTEQNKCIQKGVTFLSYEDPLGMALALMAFCFSAFTAVVLCVFVKHHDTPIVKANNRSLSYLLLMSLMFCFLCSFFFIGLPNRAICVLQQITFGIVFTVAVSTVLAKTVTVVLAFKVTDPGRRLRNFLVSGTPNYIIPI
CSLLQCVLCAIWLAVSPPFVDIDEHTLHGHIIIVCNKGSDTAFYCILGYLACMALGSFSLAFLAKNLPDTFNEAKFLTFSMLVFCSVWVTFLPVYHSTKGKHMVAVEIFSILASSAGILGCIFVPKIYIIFMRPERNSTQKIREKSYF
>Mouse.locus198.1
CVNCPEYQYANTEQNKCIQKGVTFLSYEDPLGMALALMAFCFSAFTAVVLCVFVKHHDTPIVKANNRSLSYLLLMSLMFCFLCSFFFIGLPNRAICVLQQITFGIVFTVAVSTVLAKTVTVVLAFKVTDPGRRLRNFLVSGTPNYIIPI
CSLLQCVLCAIWLAVSPPFVDIDEHTLHGHIIIVCNKGSDTAFYCILGYLACMALGSFSLAFLAKNLPDTFNEAKFLTFSMLVFCSVWVTFLPVYHSTKGKHMVAVEIFSILASSAGILGCIFVPKIYIIFMRPERNSTQKIREKSYF
>Mouse.locus206.1
CVNCPEYQYANTEQNKCIQKGVTFLSYEDPLGMALALMAFCFSAFTAVVLCVFVKHHDTPIVKANNRSLSYLLLMSLMFCFLCSFFFIGLPNRAICVLQQITFGIVFTVAVSTVLAKTVTVVLAFKVTDPGRRLRNFLVSGTPNYIIPI
CSLLQCVLCAIWLAVSPPFVDIDEHTLHGHIIIVCNKGSDTAFYCILGYLACMALGSFSLAFLAKNLPDTFNEAKFLTFSMLVFCSVWVTFLPVYHSTKGKHMVAVEIFSILASSAGILGCIFVPKIYIIFMRPERNSTQKIREKSYF
>Mouse.locus170.1
CMNCPEYQYANTEQNKCIQKGVTFLSYEDPLGMALALMAFCFSAFTAVVLCVFVKHHDTPIVKANNRSLSYLLLMSLMFCFLCSFFFIGLPNRAICVLQQITFGIVFTVAVSTVLAKTVTVVLAFKVTDPGRRLRNFLVSGTPNYIIPI
CSLLQCVLCAIWLAVSPPFVDIDEHTLHGHIIIVCNKGSDTAFYCILGYLACLALGSFSLAFLAKNLPDTFNEAKILTFSMLVFCSVWVTFLPVYHSTKGKHMVAVEIFSILASSAGILGCIFVPKIYIILMLPERNSTQKIREKSYF
>Mouse.locus143.1
CMNCPEYQYANTEQNKCIQKGVTFLSYEDPLGMALALMAFCFSAFTAVVLCVFVKHHDTPIVKANNRSLSYLLLMSLMFCFLCSFFFIGLPNRAICVLQQITFGIVFTVAVSTVLAKTVTVVLAFKVTDPGRRLRNFLVSGTPNYIIPI
CSLLQCVLCAIWLAVSPPFVDIDEHTLHGHIIIVCNKGSDTAFYCILGYLACLALGSFSLAFLAKNLPDTFNEAKFLTFSMLVFCSVWVTFLPVYHSTKGKHMVAVEIFSILASSAGILGCIFVPKIYIILMLPERNSTQKIREKSYF
>Mouse.locus161.1
CMNCPEYQYANTEQNKCIQKGVTFLSYEDPLGMALALMAFCFSAFTAVVLCVFVKHHDTPIVKANNRSLNYLLLMSLMFCFLCSFFFIGLPNRAICVLQQITFGIVFTVAVSTVLAKTVIVVLAFKVTDPGRRLRNFLVSGTPNYIIPI
CSLLQCALCAIWLAVSPPFVDIDEHTLHGHIIIVCNKGSDTAFYCILGYLACLALGSFSLAFLAKNLPDTFNEAKFLTFSMLVFCSVWVTFLPVYHSTKGKHMVAVEIFSILVSSAGILGCIFVPKIYIILMLPERNSTQKIREKSYF
>Mouse.locus212.1
CVNCPEYQYANTEQNKCIQKGVTFLSYEDPLGMALALMAFCFSAFTAVVLCVFVKHHDTPIVKANNRSLSYLLLMSLMFCFLCSFFFIGLPNKVICVLQQITFGIVFTVAVSTVLAKTVTVVLAFKVTDPGRRLRYFLVSGTLNYIIPI
CSLLQCELCAIWLAVSPPFVDIDEHSQHGHIIIVCNKGSVTAFYCVLGYLACLALGSFTLAFLAKNLPDAFNEAKFLTFSMLVFCSVWVTFLPVYHSTKGKHMVAVEIFSILASSAGMLGCIFVPKIYIILMRPERNSTQKIREKSYF
>Mouse.locus153.1
CVNCPEYQYANTEQNKCIQKGVTFLSYEDPLGMALALMAFCFSAFTAVVLCVFVKHHDTPIVKANNRSLSYLLLMSLMFCFLCSFFFIGLPNKVICVLQQITFGIVFTVAVSTVLAKTVTVVLAFKVTVPGRRLRYFLVSGTLNYIIPI
CSLLQCVLCAIWLAVSPPFVDIDEHSQHGHIIIVCNKGSVTAFYCVLGYLACLALGSFTLAFLAKNLPDAFNEAKFLTFSMLVFCSVWVTFLPVYHSTKGKHMVAVEIFSILASSAGMLGCIFVPKIYIILMRPERNSTQKIREKSYF
>Mouse.locus130.1
CVNCPEYQYANTEQNKCIQKGVTFLSYEDPLGMALALMAFCFSAFTAVVLCVFVKHHDTPIVKANNRSLSYLLLMSLMFCFLCSFFFIGLPNKVICVLQQITFGIVFTVAVSTVLAKTVTVVLAFKVTDPGRRLRYFLVSGTLNYIIPI
CSLLQCVLCAIWLAVSPPFVDIDEHSQHGHIIIVCNKGSVTAFYCVLGYLACLALGSFTLAFLAKNLPDAFNEAKFLTFSMLVFCSVWVTFLPVYHSTKGKHMVAVEIFSILASSAGMLECIFVPKIYIILMRPERNSTQKIREKSYF
>Mouse.locus210.1
CMNCPEYQYANTEQNKCIQKGVTFLSYEDPLGMALALMAFCFSAFTALVLCVFVKHHDTPIVKANNRSLSYLLLMSLMFCFLCSFFFIGLPNRVICVLQQITFGIVFTVAVSTVLAKTVIVVLAFKVTDPGRRLRYFLVSGTPNYIIPI
CSLLQCVLCAIWLAVSPPFVDIDEHTLHGHIIIVCNKGSDTAFYCILGYLACLALGSFSVAFLAKNLPDTFNEAKFLTFSMLVFCSVWVTFLPVYHSTKGKHMVAVEIFSILASSAGMLGCIFVPKIYIILMRPERNSTQKIREKSYF
>Mouse.locus216.1
```

CVNCPEYQYANTEQNKCIQKGVTFLSYEEPLGMALALMAFCFSAFTALVLCVFVKHHDTPIVKANNRSLSYLLLMSLMFCFLCSFFFIGLPNRVICVLQQITFGIVFTVAVSTVLAKTVIVVLAFKVTDPGRRLRYFLVSGTPNYIIPI
CSLLQCVLCAIWLAVSPPFVDIDEHTLHGHIIIVCNKGSDTAFYCILGYLACLALGSFSVAFLAKNLPDTFNEAKFLTFSMLVFCSVWVTFLPVYHSTKGKHMVAVEIFSILASSAGMLGCIFVPKIYIILMRPERNSTQKIREKSYF
>Mouse.locus163.1
CMNCPEYQYANTEQNKCIQKGVTFLSYEDPLGMALALMAFCFSAFTAVVLCVFVKHHDTPIVKANNRSLSYLLLMSLMFCFLCSFFFIGLPNRAICVLQQITFGIVFTVALSTVLAKTVTVVLAFKVTDPGRRLRNFLVSGTPNYIIPI
CSLLQCVLCAIWLAVSPPFVDIDEHTLHGHIIIVCNKGSVTAFYCILGYLACLALGSFSVAFLAKNLPDAFNEAKFLTFSMLVFCSVWVTFLPVYHSTKGKHMVAVEIFSILASSAGILGCIFVPKIYIILTRPERNSTQKIREKSYF
>Mouse.locus136.1
CVNCPEYQYANTEQNKCIQKGVTFLSYEDPLGMALALMAFCFSAFTAVVLCVFVKHHDTPIVKANNRSLSYLLLMSLMSCFLCSFLFIGLPNRAICVLQQITFGIVFTVAVSTVLAKTVTVVLAFKVTDPGRRLRNFLVSGTPNYIIPI
CSLLQCVLCAIWLAVSPPFVDIDEHTLHGHIIIVCNKGSVTAFYCILGYLACLALGNFSVAFLAKNLPDTFNEAKFLTFSMLVFCSVWVTFLPVYHSTKGKHMVAVEIFSILASSAGILGCIFVPKIYIILMRPERNSTQKIREKSYF
>Mouse.locus187.1
CVNCPEYQYANTEQNKCIQKGVTFLSYEDPLGMALALMAFCFSAFTAVVLCVFVKHHDTPIVKANNRSLSYLLLMSLMSCFLCSFFFIGLPNRAICVLQQITFGIVFTMAVSTVLAKTVTVVLAFKVTDPGRRLRNFLVSGTPNYIIPI
CSLLQCVLCAIWLAVSPPFVDIDEHTLHGHIIIVCNKGSVTAFYCILGYLACLALGNFSVAFLAKNLPDTFNEAKFLTFSMLVFCSVWVTFLPVYHSTKGKHMVAVEIFSILASSAGILGCIFVPKIYIILMRPERNSTQKIREKSYF
>Mouse.locus200.1
CVNCPEYQYANTEQNKCIQKGVTFLSYEDPLGMALALMAFCFSAFTAVVLCVFVKHHDTPIVKANNRILSYILLISLMSCFLCSFFFIGHPNRATCVLQQITFGIVFTVAVSKVLAKTVTVVLAFKVTDPGRRLRNFLVSGTPNYIIPI
CSLLQCVLCAIWLAVSPPFVDIDEHTLHGHIIIVFYKGSVTAFYCILGYLACLALGSFSLAFLAKNLPDTFNEVKFLTFSMLVFCSVWVNFLPVYHSTKGKHMVAVEIFSILASSAGILGCIFVPKIYIILMRPERNSTQKIREKSYF
>Mouse.locus192.1
CVNCPEYQYANTEQNKCIQKGVTFLSYEDPLGMALALMAFCFSAFTAVVLCVFVKHHDTPIVKANNRILSYILLISLMSCFLCSFFFIGHPNRATCVLQQITFGIVFTVAVSKVLAKTVTVVLAFKVTDPGRRLRNFLVSGTPNYIIPI
CSLLQCVLCAIWLAVSPPFVDIDEHTLHGHIIIVFYKGSVTAFYCILGYLACLALGSFSLAFLAKNLPDTFNEVKFLTFSMLVFCSVWVNFLPVYHSTKGKHMVAVEIFSILASSAGILGCIFVPKIYIILMRPERNSTQKIREKSYF
>Mouse.locus157.1
CVNCPEYQYANTEQNKCIQKGVTFLSYEDPLGMALALMAFCFSAFTAVVLCVFVKHHDTPIVKANNRILSYILLISLMSCFLCSFFFIGHPNRATCVLQQITFGIVFTVAVSTVLAKTVTVVLAFKVTDPGRRLRNFLVSGTPNYIIPI
CSLLQCVLCAIWLAVSPPFVDIDEHTLHSHIIIVCNKGSVTAFYCVLGYLACLALGSFTLAFLAKNLPDTFNEAKFLTFSMLVFFSVWVTFLPVYHSTKGKVMVAVEIFSILASSAGILGCIFVPKIYIILMRPERNSTQKIKEKSYF
>Mouse.locus172.1
CVNCPEYQYANTEQNKCIQKGVTFLSYEDPLGMALALMDFCFSAFTAVVLCVFVKHHDTPIVKANNRILSYILLISLMSCFLCSFFFIGHPNRATCVLQQITFGIVFTVAVSTVLAKTVTVVLAFKVTDPGRRLRNFVVSGTPNYIIPI
CSLLQCVLCAIWLAVSPPFVDIDEHTLHGHIIIVCNKGSVTAFYCVLGYLACLALGSFTLAFLAKNLPDTFNEAKFLTFSMLVFFSVWVTFLPVYHSTKGKVMVAVEIFSILASSAGMLGCIFAPKIYIILMRPERNSTQKIKEKSYF
>Mouse.locus145.1
CVNCPEYQYANTEQNKCIQKGVTFLSYEDPLGMALALMAFCFSAFTAVVLCVFVKHHDTPIVKANNRILSYILLISLMSCFLCSFFFICHPNRATCVLQQITFGIVFTVAVSTVLAKTVTVVLAFKVTDPGRRLRNFVVSGTPNYIIPI
CSLLQCVLCAIWLAVSPPFVDIDEHTLHGHIIIVCNKGSVTAFYCVLGYLACLALGSFTLAFLAKNLPDTFNEAKFLTFSMLVFFSVWVTFLPVYHSTKGKVMVAVEIFSILASSAGMLGCIFAPKIYIILMRPERNSTQKIKEKSYF
>Mouse.locus122.1
CVNCPEYQYANTEQNKCIQKGVTFLSYEDPLGMALALMAFCFSAFTAVVLCVFVKHHDTPIVKANNRILSYILLISLMSCFLCSFFFIGHPNRATCVLQQITFGIVFTVAVSTVLAKTVTVVLAFKVTDPGRRLRNFLVSGTPNYIIPI
CSLLQCVLCAIWLAVSPPFVDIDEHTLHGHIIIVCNKGSVTAFYCVLGYLACLALGSFTLAFLAKNLPDTFNEAKFLTFSMLVFFSVWVTFLPVYHSTKGKVMVAVEIFSILASSAGMLGCIFAPKIYIILMRPERNSTQKIKEKSYF
>Mouse.locus185.1
CVNCPEHQYANTEQNKCIQKGVTFLSYEDPLGMALALMAFCFSAFTAVVLCVFVKHHDTPIVKANNRILSYILLISLMSCFLCSFFFIGHPNRATCVLQQITFGIVFTVAVSTVLAKTVTVVLAFKVTDPGRRLRNFLVSGTPNYIIPI
CSLLQCILCAIWLAVSPPFVDIDEHTLHGHIIIVCNKGSVTAFYCVLEYLACLALGSFTLAFLAKNLPDTFNEAKFLTFSMLVFFSVWVTFLSVYHSTKGKVMVAVEIFSILASSAGMLGCIFAPKNCIILMRPERNSTQKIKEKSYF
>Mouse.locus134.1
CVNCPEHQYANTEQNKCIQKGVTFLSYEDPLGMALALMAFCFSAFTAVVLCVFVKHHDTPIVKANNRSLSYLLLMSLMSCFLCSFLFIGLPNRAICVLQQITFGIVFTVAVSTVLAKTVTVVLAFKVTDPGRRLRNFLVSGTPNYIIPI
CSLLQCILCAIWLAVSPPFVDIDEHTLHGHIIIVCNKGSVTAFYCVLEYLARLALGSFTLAFLAKNLPDTFNEAKFLTFSMLVFFSVWVTFLSVYHSTKGKVMVAVEIFSILASSAGMLGCIFAPKICIILMRPERNSTQKIKEKSYF
>Mouse.locus155.1
CVNCPEYQYANTEQNKCIKKGVTFLSYEDPLGMVLALMAFCFSAFTAVVLCVFVKHHDTPIVKANNRILSYILLLSLMSCFLCSFFFIGHPNRATCVLQQITFGIVFTVAVSTVLAKTVTVVLAFKVTDPGRRLRHFLVSGTPNYIIPI
CSLFQCVLCAIWLAVSPPFVDIDEHSQHGHIIIVCNKGSVTAFYCVLGYLACLALGSFIVAFLAKNLPDTFNEAKFLTFSMLVFFSVWVTFLPVYHSTKGKVMVAVEIFSILASSAGMLGCIFAPKIYIILMRPESNSTQKIKEKSNS
>Mouse.locus181.1
CVNCPEYQYANTEQNKCIKKGVTFLSYEDPLGMVLALMAFCFSAFTAVVLCVFVKHHDTPIVKANNRILSYILLLSLMSCFLCSFFFIGHPNRATCVLQQITFGIVFTVAVSTVLAKTVTVVLAFKVTDPGRRLRHFLVSGTPNYIIPI
CSLFQCVLCAIWLAVSPPFVDIDEHSQHGHIIIVCNKGSVTAFYCVLGYLACLALGSFIVAFLAKNLPDTFNEAKFLTFSMLVFFSVCVTFLPVYHSTKGKVMVAVEIFSILASSAGMLGCIFAPKIYIILMRPESNSTQKIKEKSNS
>Mouse.locus214.1
CVNCPEYQYANTEQNKCIKKGVTFLSYEDPLGMVLALMAFCFSAFTALVLCVFVKHHDTPIVKANNRSLSYILLLSLMSCFLCSFFFIGHPNRATCVLQQITFGIVFTVAVSTVLAKTVTVVLAFKVTDPGRRLRQFLVSGTPNYIIPI
CSLFQCVLCAIWLAVSPPFVDIDEHSQHGHIIIVCNKGSVTAFYCVLGYLACLALGSFTLAFLAKNLPDTFNEAKFLTFSILVFFSVWVTFLPVYHSTKGKVMVAVEIFSILASSAGMLGCIFAPKIYIILMRPESNSTQKIKEKSNF
>Mouse.locus140.1
CVNCPEYQYASTEQNKCIQKVVTFLSYEDPLGMALALIAFCFSAFTAVVLCVFVKHHDTPIVKANNRILSYILLLSLMSCFLCSFFFIGHPNRATCVLQQITFGIVFTVALSTVLAKTVTVVLAFKITDPGRRLRHFLVSGTPNYIIPI
CSLLQCVLCAIWLAVSPPFVDIDEHSQNGHIIIVCNKGSVTAFYCLLGYLACLALGSFTVAFLAKNLPDTFNEAKFLTFSMLVFFSVWVTFLPVYHSTKGKVMVAVEIFSILASIAGMLGCIFAPKIYIILMSPENNSTQKIKEKSYF
>Mouse.locus218.1
CVNCPEYQYANTEQNKCMQKAVIFLRYEDPLGMALALMAFCFSAFTALVLWAFVKHHDTPIVKANNRILSYLLLMSLMFCFLCTFFFIGHPNRVTCVLQQITFGIVFTVAVSTVLAKTVTVILAFKFTGPGRRLRNILVSGTPNYIIPI
CCLFQCVLCAIWLAVSPPFVDIDEHSEHGNIILVCNKGSVTAFYCVLGYLACLALGSFTVAFLARNLPDTFNEAKFLTFSMLVFCSVWVTFLPVYHSTKGKVMVAVEIFSILASSAGILGCIFAPKIYIIFLRPERNSIKKIREKSHF
>Mouse.locus120.1
CVNCPEYQYANTEQNKCIQKAVIFLSYEDPLGMALALMAFGFSAFTALVLWAFVKHHDTPIVKANNRILSYLLLMSLMFCFLCTFFFIGHPNRVTCVLQQITFGIVFTVAVSTVLAKTITVILAFKFTGPGRTLKNILVSGTPNYIIPI
CSLFQCILCAIWLAVSPPFVDIDEHSEHGNIILVCNKGSVTAFYCVLGYLACLALGSFTVAFLARNLPDAFNEAKFLTFSMLVFCSVWVTFLPVYHSTKGKVMVAVEIFSILASSSGILGCIFAPKIYIIFLRPERNSIKKIREKSHF
>Mouse.locus481.1
CVQCLEDQYANTEQNHCIRKTVVFLSYEEPLGVALSLISLFFSAVTIVVLGVIVKHHSTPVVKANNRTLTYILLISLIFCFLCPLLFIGHPNAATCILQQITFGVVFTVSVSTVLAKTITVVLAFKITASQRMMKYFLVSGAINYIIPF
CILIQVIVCAVWLGASPPSVDIDAHSEHGQIIIVCHKGSVNAFYCVLGYLATLAIGSFTLAFFSRNLPGAFNEAKSITFSMLVFCSVWVTFIPVYHSTKGRVMVAVEIFSILASSAGMLGCIFVPKCYTILFRPDQNSLEMIRAKSSS
>Mouse.locus484.1
CVQCLEDQYANTEQNHCIRKIVVFLSYEEPLGVALSLISLFFSAFTIVVLGVIVKHHSTPVVKANNRTLTYILLISLIFCFLCPLLFIGHPNAATCILQQITFGIVFTVSVSTVLAKTITVVLAFKIIASQRMMKYFLVSGAINYIIPV
CILIQVIVCAVWLGASPPSVDIDGQSEHGQIIIVCHKGSVNAFYCVLGYLATLAIGSFTLAFFSRNLPGAFNEAKSITFSMLVFCSVWVTFIPVYHSTKGKVMVAVEIFSTLASSAGMLGCIFVPKCYTILFRPDQNSLEMIRVKSSS
>Mouse.locus489.1

MALSLLSLCFSAFTTVVLGVMVKHHSTPIVKANNRTLTYILLISLIFCFLCPLLFIGHPNAATCILQQITFGVVFTVSVSTVLAKTITVVLAFKITASQRMMKYFLVSGAINYIIPICILIQVIVCAVWLRASPPSVDIDAHSEHGQII
IVCHKGSVNAFYCVLGYLATLAIGSFTLAFFSRNLPGAFNEAKSITFSMLVFCSVWVTFIPVYYSTKGKVMVAVEIFSILASSAGMLGCIFVPKCYTILFRPDQNSLEMIRVKSSS
>Mouse.locus487.1

MALSLLSLCFSAFTTVVLGVMVKHHSTPIVKANNRTLTYILLISLIFCFLCPLLFIGHLNSATCILQQITFGVVFTVSVSTVLAKTITVVLAFKITASQRMMKYFLVSGAINYIIPICILIQVIVCAVWLGASPPFVDIDAHSEHGQII
IVCHKGSVNAFYCVLGYLAILAIGGFTLAFLSRNLPGAFNEAKSITFSMLVFCSVWVTFIPVYYSTKGKVMVAVEIFSILASSSGMLGCIFVPKCYTILFRPDQHSLEMIRVKSSS
>Mouse.locus486.1
CVQCPEDQYANTEQNHCIRKAVVFLSYEEPLGVALSLLSLCFSAFTTVVLGIFVKHHNTPIVKANNRTLTYLLLISLIFCFLCPLLFIGHPNSATCILQQLTFGVVFTVSLSTVLAKTITVVLAFKIIASQRMMKYFLISGAINYIIPI
CILIQVIVCAVWLRASPPSVDIDAHSEHGQIIIVCHKGSVNAFYCVLGYLAILAFGSFTLAFLSRNLPGAFNEAKSITFSMLVFCSVWVTFIPVYHSTKGKVMVAVEIFSTLASSAGMLGCIFVPKCYTILFRQDQNSLEMIRVKSSS
>Mouse.locus469.1

MALSLFSLCFSAFTTVILGVIVKHHNTPIVKANNRTLTYILLISLIFCFLCPLLFIGHPNSVTCILQQITFGVVFTVALSTVLAKTIVVVLAFKITASQRMMKYFLVSGASNYIILICTLIQVIVCAVWLGASPPSVDIDAQSEHGHII
IVCHKGSVNAFYSILGYLALLSFGSFTLAFLSRNLPGAFNEAKSITFSMLVFCSVWVTFIPVYHSTKGKVMVAVEIFSTLASSAGMLGCIFVSKCYTILFRPDRNSVEMTRVKSSF
>Mouse.locus472.1

MALSLLSLFFSAFTTVILGVIVKHHNTPIVKANNRTLTYILLISLIFCFLCPLLFIGHPNSVTCFLQQITFGVVFTVALSTVLAKTITVVLAFKITASQRMMKYFLVSGASNYIILICTLIQVIVCAVWLGASPPFVDIDAQSEHGHII
IVCHKGSVNAFYSILGYLALLSFGSFTLAFLSRNLPGAFNEAKSITFSMLVFCSVWVTFIPVYHSTKGKVMVAVEIFSTLASSAGILGCIFVSKCYTILFRPDRNSVEMTRVKSSF
>Mouse.locus474.1

MALSLMALCFSAFTAVVLLVFVKHHSTPIVKANNRTLTYILLISLIFCFLCPLLFIGHPNSVTCILQQVTFGVAFTVAVSTVLAKTITILLAFRVTAPQRMMKYFLVSGTYIYIIPICTLIQVIVCAVWLGAAPPSVDIDARSEHGHII
IVCNKGSVSAFYCVLGYLACLAFGSFTLAFLSRNLPSTFNEAKSITFSMLVFCSVWVTFLPVYHSTKGKVMVAVEIFSTLASGAGMLICIFVPKCYIILFMPDRNSLQMIRKKSSS
>Mouse.locus476.1

MALSLMALCFSAFTAVVLLVFVKHHSTPIVKANNRTLTYILLISLIFCFLCPLLFIGHPNSVTCVLQQVTFGVAFTVAVPTVLAKTITIVLAFRVTAPQRMMKYFLVSGASIYIIPICTLIQVIVCAVWLGAAPPSVDIDARSEHGHII
IVCNKGSVSAFYCVLGYLACLAFGSFTLAFLSRNLPSTFNEAKSITFSMLVFCSVWVTFLPVYHSTKGKVMVAVEIFSTLASGAGMLICIFVPKCYIILFMPDRNSLQMIRKKSSS
>Mouse.locus477.1

MALSLMALCFSAFTAVVLLVFVKHHSTPIVKANNRTLTYILLISLIFCFLCPLLFIGHPNSATCVLQQVTFGVAFTVAVSTVLAKTITIVLAFRVTAPQRMMKYFLVSGASIYIIPICTLIQVIVCAVWLGAAPPSVDIDARSEHGHII
IVCNKGSVSAFYCVLGYLACLAFGSFTLAFLSRNLPSTFNEAKSIKFSMMVFFSICVTFLPVYHSTKGKAMVAIEIFSTLASGAGMLICIFFPKYYTILFVPDRNSLQVIRKKSSS
>Mouse.locus494.1
CIKCPDDQYANTEQTHCIPKAVTFLAYEDPLGMALACMALCFSALTALVLGVFVKHHETPIVKANNRTLSYILLISLILCFLCSLPFIGLPNMATCILQQMSFGVVFTIAVSTVLAKTVTVVLAFRSTVPGRRMRWILVS--
PNLIIPICTLIQMVLCAIWLGASAPFVDINAHNERGHITIMCNKGSATAFYCVLGYLGFLAMGTLTVAFWARNLPDIFNEAKFLTFSMLVFCSVWVTFLPVYHSTKGKVMVAVEVFSILASSAGLLGCIFVPKCYIILIRPKNGSFKGL
RGTRVC
>Small-eyed_rabbitfish.locus629.1

VGVSTTISIATVFFTHRDTPIVKANNSELSYLLLFALTLCFLCSVTFIGEPSVWACKLCHTAFAIAFVLSISCILIKTILVLLAFKATLPNNNMAKLFGPAQQRLSILLLTSVQCLICTLWLTISHPFPMRNTEYYREIIILECDLGSV
TAFYSMAGYIAFLAGVSFVLAFLARKLPDKFNEAQCITFSMLIFYAVWITFIPVYVSSPGKYTVAVEVFAILASSFGLMFCIFVPKCYIILIKPEMNT----------
>Smalltooth_sawfish.locus38.1

MGIILMIVALFGTFITLVMLSVFLYCKDTPIVKGNNSELSFLLLFSLLLCFLCSLTFIGKPSVWSCVLRHTLFGIAFVLCVSCILVKTIVVLKAFKATVPQKSTMKWFGAAQQRSIVCACTLIQVLICVTWLAMAPPFPAKNMRHHNAK
VILECDVGSPIAFYCALGYIGVLSGLCFVLAFLARKLPGNFNEAKLITFSMLIFCAVWLTFIPAYVSSPGKYTVAVETFAILSSSFGLLFCIFVPKCYIILFKPENNTKKSLMGKGPS
>Elephant_shark.locus29.1
CLKCPIDYWSNQGRDQCVLKEVEFLSFEGTLGIILASVALIGACITICVFAIFLHYRNTPIVRANNMELSFLLLGSLVLCFLCSLFYIGRPSLWSCVLRHTVFGVSFALCISCILGKTIVVLMAFSATLPNNNMMKYFGPLQQAASVLA
CTILQVIICVIWLITSPPFPTKNTEHQKAKVILECNVGSVAAFCCVLGYIGLLASVSFVLAFLARKLPDNFNEAKFITFSMLIFCAVWITFIPAYISSPGKYTVAVEVFAILSSSFGLIACNFAPKCYVILLRPEENTKKHLMGRGRS
>Elephant_shark.locus30.1
CLKCPIDYWSNQGRDQCVLKEVEFLSFEGTLGIILASVALIGACITICVFAIFLHYRNTPIVRANNMELSFLLLGSLVLCFLCSLFYIGRPSLWSCVLRHTVFGVSFALCISCILGKTIVVLMAFSATLPNNNMMKYFGPLQQTASVLA
CTILQVIICVIWLITSPPFPTKNTEHQKAKVILECNVGSVAAFCCVLGYIGLLASVSFVLAFLARKLPDNFNEAKFITFSMLIFCAVWITFIPAYISSPGKYTVAVEVFAILSSSFGLIACNFAPKCYVILLRPEENTKKHLMGRAGP
>Coelacanth.locus252.1
CLECPDDYWSNDRRDRCIPKSIEFLSYKDPLGAVLASFSIFSSLIPVSILCIFIWYRNTPIVKANNRELSYVLLLSLILCFLCSLIFIGRPSIETCMLRQTAFGIIFAFSVACVLAKTIMVVIAFNATKPNSNLKKWVGPKLPNTIVVT
CSLLQIIICIAWLASLPPFPEKNIKSEPGKIIIECNEGSTVAFWCMLGYMGLLAIVSFIVAFLARNLPDSFNEAKFITFSMLVFVTVWLSFIPAYLSTKGKYMVAVEIFAILASSAGLLVCIFFPKCYIILLRPGFNTKEYLMGKGTH
>Coelacanth.locus250.1
CLECPDDHWSNDRRDKCIPKSIEFLSYRDILGATLASISIFCSLIPCTLLSIFIWYHDTPIVKANNRELSYLLLIALMLCCLTSLIFIGQPLMVTCLLRQAAFGIIFALCVSCVLAKTIMVVIAFNATKPSTNLKKWVGHKLPNTIVFF
CTLFQVILCIAWLTASPPFPEESMKSEPGKIIIECNEGSAVAFWCMLGYMGLLAVTSFIVAFLARNLPDTFNEAKFITFSMLVFVTVWLAFIPAYLSTKGKYMVAVEIFAILASSVGLLACIFFPKCYIILLRPDVNTKQYLLGKGTF
>Coelacanth.locus253.1
CLECPDDHWSNDRGDKCIPKSIEFLSYTEPLGIILTCIAIFCSIIPAAILFTFIWYCDTWIVKANNRELSYLLLLGLVLCFLCCLIFIGQSLTATCMLRQATFGVAFAICVSCVLAKTIMVVIAFNATKPNSNLKNWVGPKLPNTIVFV
CSLFQVIICLAWLTGSPPFPEPNMKLELGRIIIECNEGSTVAFWCMLGYMGLFAVTSFIVAFLARNLPDSFNEAKFITFSMLVFVTVWLSFIPAYLSTRGKYMVAVEIFAILASSAGLLACIFFPKCYIIFLRPEMNSREYLMGKGIF
>Coelacanth.locus309.1

CLECPEDHWSNDRRDKCILTLIEFLSYEEPLGAILASISIFSSLIPAAVLCSFIRYHDTPIVKANNRELSYLLLLALMLCFLCSLIFIGQPLVATCMLRQTAFGIIFALCVSCVLAKTIMVVIAFNTTKPNSNLKKWVGPKLANSIVFV
CTLFQVIICITWLSSSPPFPEQNMKSQPGTIIIECNEGSTVAFWCMLGYMGLLAIISFIVAFLARNLPDSFNEAKFITFSMLVFVIVWLSFIPAYLSTRGKYMVAVEIFAILASSAGLLACIFFPKCYIILLRPDKNTREYLMGKGTL
>Coelacanth.locus304.1
CLKCPNDHWSNSARDKCIPKVIEFLSYKEPLGATLASISSLSSVIPVAILCTFIKYQDTPIVKANNREVSYLLLLALVLCFLCSLVFVGYPMMITCMLRQAAFGIIFALCVSCVLAKTIMVVIAFNATKPNSNLQKWVGAKLPNSVVFI
CTMLQVILCIAWLTSSPPFPEENMKSQPGKIIIECNEGSTVAFWCMLGYMGLLAIISFIVAFLARNLPDSFNEAKFITFSMLVFVSVWLAFIPAYLSTSGKYTVAVEIFAILASSAGLLACIFFPKCYIILLRPHMNTKGYLMGKGTF
>Coelacanth.locus305.1
CLKCPDDHWSNQRRDECIPKTIEFLSYEEPLGATLASISVVSSLLPVAILSTFIKYRDTPVVKANNRELSYILLLAMIFCFLCSLIFIGYPKRVTCMLRQAAFGIIFALCVSCVLAKTIMVVIAFNATKPSSNLKKWVGHKLPNTIVFV
CTLLQVIICIAWLTSSPPFPEQNMKSQPGKIIIECNEGSTIAFWCVLGYMGLLATVSFTVAFLSRNLPDSFNEATYITFSMVVFVSVWLAFIPAYLSTRGKYMVAVEIFAILASSAGLLSCIFFPKCYIILLRPDMNSKEYLLGKGVF
>Coelacanth.locus308.1
CLKCPDDHWSNQRRDECIPKTIEFLSYEEPLGATLASISVASSLLPVTILSTFIKYRDTPIVKANNRELSYVLLLAMIFSFLCSLIFIGYPKRVTCMLRQAAFGIIFALCVSCVLAKTIMVVIAFNATKPSSNLKKWVGHKLPNTIVFV
CTLLQVIICIAWLTSSPPFPEQNMKSQPGKIIIECNEGSTIAFWCVLGYMGLLATVSFTVAFLSRNLPDSFNEAMYITFSMLVFVSVWLAFIPAYLSTRGKYMVAVEIFAILASGAGLLSCIFFPKCYIILLRPDMNSKEYILGKGVF
>Coelacanth.locus306.1
CLKCPDDHWSNQRRDECIPKTVEFLSYEEPLGATLASISIAFSLIPVAILSTFIRYRDTPVVKANNRELSYILLFAMTLCFLCSLIFIGYPKRVTCMLRQAAFGIIFALCVSCVLAKTIMVVIAFSATKPSSNLKKWVGHKLPNTIVFV
CTLLQVIICIAWLTGSPPFPEQNMKSQPGRIVIECNEGSTIAFWCVLGYMGLLASISFIVAFLSRNLPDSFNEAIYITFSMLVFVSVWLAFIPAYLSTRGKYMVAVEIFAILASSAGLLSCIFFPKCYIILLRPDMNTKEHLLGKGVF
>Coelacanth.locus352.1
CLKCPDDQWSNDRRDTCIPKPTEFLSYKEPLGATLGAITIFSALIPSTILCVFIRYRNTPIVKANNRELSYLLLLALVFCFLCSLIFIGYPTGVTCMLRHVAFGIIFALCVSCMLAKTIMVVIAFNATKPNSKLRNWVGPKLPNGLVFV
CTLIQVIICIAWLISSPPFSEQNMKSQPGKIIFECNEGSPVAFWCLLVYMGLLALVCLIVAFFARNLPNSFNEAKFISFSMLVFVSVWLAFIPAYLSTRGKYMVAVEIFAILTSSTGLLACIFFPKCYIILLRPDMNTREYLMGKVPK
>Coelacanth.locus261.1
CMKCPDDQWSNDRRDTCIPKSIEFLSYKEPLGATLGVITIFSALIPSAILCIFIRYRDTPVVKANNRELSYLLLLALVFCFLCSSIFIGYPMRVTCMLRHVAFGIIFALCVSCMLAKTIMVVIAFNATKPSSNLKNWVGPKLPNGLVFV
CTLIQVIICIVWLISSSPFSEQNMKSQPGKIIFECNEGSPVAFWCMLGYMGLLALVCFIVAFFAKNLPDSFNEAKFISFSMLVFVSVWLAFIPAYLSTRGKYMVAVEIFAILASSAGLLACIFFPKCYIILLRPDMNTREYLMGKVPK
>Coelacanth.locus395.1
CMKCPDNQWSNERRDKCIPKTIEFLSYKDPLGATLAAISVFFSLIPTTILCIFYRYQTTPIVKANNRQLSYLLLVALVLCFLCSLLFIGYPVRVTCMLRQAAFGIIFALCVSCVLAKTIMVVIAFNATKPNSNLKTWVGPKLPNSIVLV
CTAVQIIICITWLMSSPPFSEQNMKSQLGKIIFECNQGSTTAFWCLLGYMGLLAIISFIVAFLARNLPDSFNEAKFITFSMLVFVTVWLAFIPAYLSTRGKYMVAVEIFAILASSAGLLGCIFFPKCYIILLRPEMNTKEYLMGKVAK
>Coelacanth.locus274.1
CMKCPDDQWSNDRQDMCIQKTFEFLSYKDPMGATLTVLASFFAVIPITILCIFIKYRDTPIVKANNQELSYLLLLALVLCFLCSLIFIDKPRIWTCMVRQAAFGIVFALCVSCVLAKTIMVVIAFNATKPNSNLKKWVGPKLANSIVLV
CAVIQVIICVAWLATFPPFPEYNMKSQIGIIIIECNQGSSTAFWCVLGYMGLLAIICFIVAFLARNLPGSSNEAKFITFSMLVFVSVWLAFIPAYLSTKGKYMVAVEIFAILASSAGLLACIFFPKCYIIVLRPDVNTKEYLMGKETF
>Coelacanth.locus334.1
CIQCSLDYWPNGKKDKCILKEIEFLSFHDTLGITLTIVSLVGALTTLTVASVFLQYKTTPIVRANNSELSFLLLFSLILCFLCSLSFIGRPTVWSCMLRHTLFGISFVLCISCVLGKTIVVLMAFKATLPGNNVMQYFGPTQQRLIVSL
CTLIQVTFCIVWVTVLPPIPYKNSSHYSYKIILECALGSLALYGCVMGYIGFLSCICFILAFFARKLPDNFNEAKFITFSLIIFCAVWITFIPAYISSPGKYTVAVEVFAILSSSFGLLLCIFAPKCYVILLKPEKNTKKHMMGKVVS
>Mouse.locus23.1
CDPCGEDDWSNAEKSKCVPKLVEFLAYGEALGFTLVILSIFGALVVLAVTVVYVIHRHTPLVKANDRELSFLIQMSLVITVLSSLLFIGKPCNWSCMARQITLALGFCLCLSSILGKTISLFFAYRISVSKTRLI-
SMHPIFRKLIVLVCVVGEIGVCAAYLVLEPPRMFKNIEIQNVKIIFECNEGSVEFLCSIFGFDVLLALLCFLTTFVARQLPDNYYEGKCITFGMLVFFIVWISFVPAYLSTKGKFKVAVEIFAILASSYGLLGCLFLPKCFIILLRPKR
NTDETVGGRVPT
>Mouse.locus22.1
CDPCGEDDWSNAEKSKCVPKLVEFLAYEEALGFTLVILSIFGALVLLAVTVVYVIHRHTPLVKANDRELSFLIQMSLVITVLSSLLFIGKPCNWSCMARQITLALGFCLCLSSILGKTISLFFAYRISVSKTRLI-
SMHPIFRKLIVLICVVGEIGICAAYLVLEPPRMFKNIEIQNVKIIFECNEGSIEFLCSIFGFDVLLALLCFLTTFVARQLPDNYYEGKCITFGMLVFFIVWISFVPAYLSTKGKFKVAVEIFAILASSYGLLGCLFLPKCFIILLRPKR
NTDETVGGRVPT
>Coelacanth.locus327.1

MGILLAVFSLIGFFITIAVVIIFFYYRHTPVVKANNSELSFLLLFSLVLCFLCSLTFIGQPSVWSCMLRHTAFGIAFVLSISCILGKTVIVIMAFKATLPGSKLMKWFGPTLQRLAVFTLTLIQGLICILWLSLSPPLPSQNTNYYKER
IILECDLGSVSAFYCVLGYIGILSILSFVLAFFARKLPDNFNEAKFITFSMLIFCAVWISFIPAYISSPGKYTVAVEIFAILASSFGLLFCIFAPKCYIILVNPEKNTRKHLMGKVPS
>Coelacanth.locus329.1
CTKCPLEYWSNQNRDQCILKDTEFLAFRETMGTLLATISLTGSFMIIVVVVIFFYHRDTPVVKANNSELSFLLLFSLALCFLCSFSFIGQPTTWSCMLRHSTFGISFALCISCVLGKTIVVVMAFKANLPGTKVMKWFGPTCQRLGVFN
LTLIQGLICTLWLSLSPPYASKNMKHYKDRIILECNLGSVTAFYCVLGYIGVLSILCFVLAFLARKLPDNFNEAKYITFSMLIFCLVWLTFIPVYISSPGKYTVAVEIFAILSSSFGLLVCIFAPKCYIILCKPEKNTHKHLMGKTVS
>Coelacanth.locus330.1

MGILLVVLSLTGSFTTIAILIIFFYYRHTPIVKANNSELSFLLLFSLLLCFLCSITFIGQPSPWTCSLRHAAFGITFVLCISCILGKTIVVLMAFKTTLPGANTMKWFGPTQQRLTVFAFTLVQGLMCTLWLSLSPPFPSQNMNYYKDR
IILECNLGSVTAFYCVLGYIGVLSILCFVLAFLARKLPDNFNEAKHITFSILIFCTVWLAFIPAYISSPGKYTVALEIFAILASSFALLFCIFAPKCYIILFKPENNTRKHLMGKKTS
>Reedfish.locus27623.1
CLKCPPEYWPNERRDQCILKKTEFLSLEETMGLLLATFALVGAAITISVAAIFFYFGDTPVVKANNSELSFLLLFSLTLCFLCSLTFIGQPSDWSCMLRHTTFGITFVMCISCVLGKTIVVVMAFTATLPGSNVMKWFGPAQQRLTVSV
STLIQVCICAVWLMISPPFSSKNLIYYKDKIILECNPGSVAAFYIVLGYIGLLSILCFFLAFLARKLPDNFNEAKFITFSMLIFCAVWITFIPVYISSPGKFTVTIEIFAILCSSYGLLFCIFVPKCYIILLKPEKNTRKLLMGKRR-
>Reedfish.locus27713.1

MEYWSNAKKDKCIFKETEHLSFEEILGILLTAVSLMGVCITLVTAFIFFLYKHTPIVKANNSELSFLLLFSLTLCFLCSVTFIGRPSDWSCMLRHTAFGITFVLCISCVLGKTIVVLMAFRATQPGSSVMKWFGASQQRLCVFVITSLQ
SLICFLWLLIAPPSADRNFSYYRDKIILECNVGSQTAFYAVLGYIGLLSSLCFILAFKARTLPDNFNEAKFITFSMLIFCAVWITFIPVYISSPGKYIVVVEIFAILASSFGLLFCIFTPKCYIILFKPHRNTKKYLMGKTC-
>Reedfish.locus27629.1

MGILLVILSLLGIFLTIIIATIFYYYRHTPVVKANNSELSFLLLFSLALCFLCSLTFIGQPSEWSCKLRHTSFGITFVLCISCVLGKTIVVLMAFRATLPGNNLMKWFGQTQQRLSVFILTLAQIIICAVWLISSPPIPIQNLKYYKEK
IILECNVGSDVAFYAVLGYIGGLSLLCFVLAFLARKLPDNFNEAKYITFSMLIFCAVWITFIPAYISSPGKFTVAVEIFAILASSYSLLFCIFSPKCYIILLQPEKNTKKHLMGKTP-
>Reedfish.locus40391.1
CFPCPWKYWPNTGKSQCILKKIDFLSYNEILGTILVVLPVSGAFLTFSVAIIFFHHRRTPIVKANNSELSFLLLFSLTLCFLCSLTFIGEPSDWSCILRHIAFGITFVLCISCILVKTIVVLMAFKASQPGNKIKKWFGPVQQKLSVAA
STFVQIVICTLWLTLSPPFLYHNTKLFKDTIILECDVGSPIAFYLVLGYIGLLSGMCFVFAFLARQLPDNFNEAKHITFSMLIFCAVWLTFIPAYISSPGKYIVAVETFAILSSSFGLLLCIFAPKCYIILLNPEKNTRKHLMGKNVS
>Reedfish.locus40393.1
-MLCPPQYWPNDQRNQCLPKEIEFLSFDENLGIVLVLFSLCGASLTVAIVVVFFHHRRTPIVKANNSGLSFLLLFSLTLCFLSSISFIGKPSQWSCMFRHTVFGITFVLSISSVLGKTIVVLMAFKATVPGNNTLKWFGPKHQRLGVAA
CTLIQAIICVLWLITSPPYPYQNMKYYNDIIILECALGSSAAFFSVLGYIGLLSFVCFIFAFLARHLPDNFSEAKYITFSMLIFCTVWLTFIPVYISTPGKYTVAVEIFAILASSFGLLFCIFAPKCYIILLYPERNTKKHMMGKMYS
>Reedfish.locus40385.1

MAKDQCNEKIFEFLSYEDVLGIILVLFSLSGASITVAVALIFFQHRHTPIVKANNSELSFLLLLSLTLCFLCSITFIGKPSSWSCMLRHTAFGITFVLCLSCVLGKTIVVLMAFRATVPGTNIMKWFGPFQQRMSVFACTFLQVLICIG
WLTVSSPYPDRNMEYSKDKIILECNVGSSVAFYVVLGYIGILSTFCFILAFLARKLPDNFNEAKFITFSMLIFCAVWITFIPAYISSPGKYTVAVEIFAILASSFGLLFCIFAPKCYIVLLKPEKNTKKFMMEKMPQ
>Western_clawed_frog.locus480.1

MLSSVIPLVILGIFIHYKNTPIVEANNYSLSYLLLLSLFLCFLCSLGFIGYPQPEKCLLRQVAFGMVFALCISCVLAKTITVVIAFNATKPGSRLRKWTGVKVSYCAIMLCALIQIIDCALWLIFSPPFHELDTDTQPGVIIANCNEGS
PTAFWCMLGYLGLLASISFIVAFLARRLPDSFNEAKLITFSMLAFLSVWVSFIPAYLSARGMYTVAMEVFAILSSSWAVVGCIFVPKCYIILFRPNLNSREHLIGKRT-
>Western_clawed_frog.locus479.1

MWPNLQQDRCLPRPTEFLSYENPLGYSLAAISMLSSMIPLVILGIFIHYKNTPIVKANNYSLSYFLLLSLFLCFLCSLGFIGYQQPEKCLLRQVAFGLVFALCISCVLAKTITVVIAFNATKPGSRLRKWTGVKVSYCVIMLCALIQMI
DCVLWLIFSPPFHELDTDTQPGVIIANCNEGSPTAFWCMMGYLGLLASISFIVAFLARRLPDSFNEAKLITFSMLAFLSVWVSFIPAYLSARGMYTVAMEVFAILSSSWAVVGCIFVPKCYIILFRPNLNSREHLIGKRT-
>Western_clawed_frog.locus476.1

MWPNLQQDRCLPRITEFLSYENPLGYSLAAISMLSSMIPLVILGIFIHYKNTPIVKANNYSLSYLLLLSLFLCFLCSLGFIGYPQPEKCLLRQVAFGMVFALCISCVLAKTITVVIAFNATKPGSRLRKWTGVKVSYCVIMLCALIQII
DCALWLIFSPPFHELDTDTKPGVIIVYCNEGSPTAFWCMLGYLGLLASISFIVAFLARRLPDSFNEAKLITFSMLAFLSLWVSFIPAYLSARGMYTVAMEVFAILSSSWAVVGCIFVPKCFIILFRPNLNSRENLIGKRT-
>Western_clawed_frog.locus482.1

MIPLVILGIFIHFKTTPIVKANNYSLSCLLLLSLFLCFLCSLGFIGYPQPEKCLLRQVAFGMVFALCISCVLAKTITVVIAFNATKPGSRLRKWTGVKVSYCVIMLCALIQIIDCALWLIFSPPFHEVDTDTKPGVIIVNCNEGSPTAF
WCMLGYLGLLASISFIVAFLARRLPDSFNEAKLITFSMLAFLSVWVSFIPAYLSARGMYTVAMEVFAILSSSWAVVGCIFVPKCYIILLRPNLNSREHLIGKRT-
>Western_clawed_frog.locus483.1
CHPCSWDTWPNLQQDRCLPRPTEFLSYGDPLGYSLAAISILSSLIPLTILGIFIRFKNTPIVKANNYSLSCLLLLSLLLCFLCSLGFIGYPQPEKCLLRQVAFGMVFALCISCVLAKTITVVIAFNATKPGSRLRKWTGVKVSYCVIML
CALIQIIDCALWLIFSPPFHELDTDTQPGVIIVNCNEGSPPAFWCMLGYLGLLATISFIVAFLARRLPDSFNEAKLITFSMLAFLSVWVSFIPAYLSARGMYTVAMEVFAILSSSWAVVGCIFVPKCFIILFRPNLNSRENLIGKR--
>Western_clawed_frog.locus477.1
CHPCSWDTWPNLQQDRCLPRPTEFLSYGDPLGYSLAAISIFSSLIPLGVLGVFIHQKKTPIVRANNYSLSCLLLLSLFLCFLCSLGFIGYPQPEKCLLRQVAFGMVFALCISCVLAKTITVVIAFNATKPGSRLRKWTGVKVSYCVIGF
CIFVQIIDCALWLIFSPPFHELDTDTQPGVIIANCNEGSPTAFWCMLGYLGLLASISFIVAFLARRLPDSFNEAKLITFSMLAFLSVWVSFIPAYLSARGMYTVAMEVFAILSSSWAVVGCIFVPKCYIVLFRPNLNSRENLIGKRTT
>Western_clawed_frog.locus474.1
CHPCSWDTWPNLQQDRCLPRPIEFLSYGDPLGYSLAVISSFSSLTPITILGVFIRYNKTPIVRANNYSLSCLLLLSLFLCFLCSLGFIGYPQPEKCLLRQVAFGMVFALCISCVLAKTITVVIAFNATKPSSRLRKWTGVKVSYCVIGF
CIFVQITDCALWLIFSPPFHELDTDTQPGVIIANCNEGSPTAFWCMLGYLGLLATISFIVAFLARRLPDSFNEAKLITFSMLAFLSVWVSFIPAYLSARGMYTVAMEVFAILSSSWALVVCIFMPKCYIVLFRPNLNSREHLMGKGRG
>Western_clawed_frog.locus481.1
CHPCSWDTWPNLQQDRCLPRPTEFLSYEQPLGYSLAAISIFSSFTPITILGVLIRYNKTPIVRANNYSLSCLLLLSLFLCFLCSLGFIGYPQPEKCLLRQVAFGMVFALCISCVLAKTITVVIAFNATKPGSRLRKWTGVKVSYCVIGF
CALIQILDCALWLIFSPPFHELDTDIQPGVIIANCNEGSPTAFWCMLGYLGLLASISFIVAFLARRLPDSFNEAKLITFSMLAFLSVWVSFIPAYLSARGMYTVAMEVFAILSSSWAVVGCIFLPKCFIILFRPNLNSREHLIGKRT-
>Western_clawed_frog.locus454.1

MFSSLTPIAILGIFIHYKTTPIVRANNYSLSCLLLLSLFLCFLCSLGFIGYPQPEKCLLRQVAFGMVFALCISCVLAKTITVVIAFNATKPGSRLRKWTGVKVSYCVIMLCALIQTIDCALWLIFSPPFHELDTDTQPGVIIANCNEGS
PTAFWCMLGYLGLLASISFIVAFLARRLPDSFNEAKLITFSMLAFLSVWVSFIPAYLSARGMYTVAMEVFAILSSSWAVVGCIFVPKCYIILFRPNMNSRIHLSFKGK-
>Western_clawed_frog.locus484.1
CHPCSWDTWPNLQQDRCLPRPTEFLSYGDPLGYSLAAASIFSALDPLVILGVFIRYKKTPIVRANNYSLSCLLLLSLFLCFLCSLGFIGYPQPEKCLLRQVAFGMVFALCISCVLAKTITVVIAFNATKPGSRLRKWTGVKVSYCVIVL
CALIQTIDCALWLIFSPPFHELDTDTKPGVIIVNCNEGSPTAFWCMLGYLGFLASISFIVAFLARRLPDSFNEAKLITFSMLAFLSVWVSFIPAYLSARGMYTVAMEVFAILSSSWAVVGCIFVPKCYIILFRPNMNSREHLMGKKKG
>Mouse.locus347.1
CVHCPETHYASAEKRHCLKKTITFLSYNDPLGKGITLLSIGFSTLTALVIWVFVEHRGTPIIKANNRSLSYILLITLILCFLCPLLFIGLPNTATCIMQQYMFGLLFTVALSTVLAKTITVVMAFKITAPGRKIRWLLTSQVPKFVIPV
CILIQVLLSGIWLGTSPPFIDKDYQSEHGHIIILCSKGSYIYFYCTLAYLGVMAFGSYLLAFLSRRLPDRFNESKSLAFSMLVFCSVWVMFLPVCHSTSGKVMVAMEMFSILASSASILIIVFAPKCYIVLFRPELNTLTHNRDKRHH
>Mouse.locus345.1
CVHCPETHYANGEKRYCLKKRVTFLSYDDPLGKGITLLSIGFSTLTALFIWVFVDHRGTPIIKANNRSLSYILLITLILCFLCPLLFIGLPNTAMCIMQQYIFGLLFTVALSTVLAKTITVVMAFKITAPGRKIRWLLTSQVPKFVIPV
CTLIQILLSGIWLGTSPPFIDKDCHSEHGHIIILCSKGSYIYFYCTLAYLGVMAFGSYLLAFLSRRLPDRFNESKSLAFSMLVFCSVWVMFLPVYHSTSGKVMVAMEIFSTLASSASILIIVFAPKCYIVLFRPEVNTLTHNRDKRQH
>Mouse.locus346.1
CVHCPETHYANAEKRHCLKKTVTFLYYNDPLGKGITLLSIGFSTLTALFIWVFVDHRGTPIIKANNRSLSYILLITLILCFLCPLLFIGLPNTATCIMQQYMFGLLFTVALSTVLAKTITVVMAFKITAPGRKIRWLLTSQVPKFAIPI
CTLIQVLLSGIWLGTSPPFIDKDYHSEHGHIIILCNKGSYIYFYCTLAYLGVMAFGSYLLAFLSRRLPDRFNESKSLAFSMLVFCSVWVMFLPVYHSTSGKVMVAMEMFSILASSASILIIVFAPKCYIVLFRPELNILTHNRDKRHH
>Mouse.locus348.1

MSLGFSALTTLIIGVFVNHRDTPIVKANNRSLTYILLITLALCFLCPLLFIGHPNTVTCIMQQNLFGLLFTVVLSTVLAKTITVVMAFKIAVPGRKLRLLLISQVPNFIIPVCTLIQVCLSGIWLGTFPPFIDMDAHSEYGHIIILCNK
GSAIAFYCTLAYLGVMAIGSYLMAFLSRSLPDTFNEAKFLAFSMLVFCAVWVTFLPVYHSTTGKVMVAMEMFSILASSSSLLILIFVPKCYIILFRPERNTVHHIRDERHD
>Mouse.locus63.1

```
CVRCPDDKYANIEQTHCLSRAVSFLAYEDPLGMALGCMALSFSAITILVLVTFVKHNDTPIVKANNRILSYILLISLVFCFLCSLLFIGPPDQVTCILQQTTFGVFFTVSVSTVLAKTITVVMAFKLTTPGRRMRGMMMTGAPKLVIPI
CTLIQLVLCGIWLVTSPPFIDRDIQSEHGKIVILCNKGSVIAFHVVLGYLGSLALGSFTLAFLARNLPDTFNEAKFLTFSMLVFCSVWITFLPVYHSTRGRVMVVVEVFSILASSAGLLMCIFVPKCYVILIRPDSNIIKKHKGKVLN
>Mouse.locus508.1
CVRCPDDKYANIEQTHCLSRAVSFLAYEDPLGMALGCMALSFSAITILVLVTFVKYKDTPIVKANNRILSYILLISLVFCFLCSLLFIGHPNQVSCILQQTIFGVFFTVSVSTVLAKTITVVMAFKLTTPGRRIRGMMMTGAPKLVIPI
CTLIQLVLCGIWLVTSPPFIDRDIQSEHGKTVILCNKGSVIAFHVVLGYLGSLALGSFTLAFLARNLPDTFNEAKFLTFSMLVFCSVWITFLPVYHSTRGRVMVVVEVFSILASSAGLLMCIFVPKCYVILIRPDSNFIKKHKGKLLN
>Mouse.locus391.1
----------------------------------
MALGCMALSFSAITILVLVTFVKYKNTPIVKANNRILSYILLISLVFCFLCSLLFIGHPDQVTCILQQTTFGVFFTVSVSTVLAKTITVVMAFKFTTPGRRMRGMLVTGAPKLVIPICTLIQLVLCGIWLVTSPPFIDRDIQSEHGKVV
ILCNKGSVIAFHIVLGYLGSLALGSFTLAFLARNLPDTFNEAKFLTFSMLVFCSVWITFLPVYHSTRGKVMVVVEVFSILASSAGLLMCIFVPKCYVILVRPDSNFTKNRKGKLLY
>Mouse.locus389.1
CVKCPYDKYANIEKTHCLSRAVSFLAYEDPLGIALGCIALSFSAITILVLITFLKYKDTPIVKANNRILSYILLISLVFCFLCSLLFIGHPNQVSCVLQQTTFGVFFTVSVSTVLAKTITVVMAFKLTTPGRRMREMLVTGAPKLVIPI
CTLIQFVLCGIWLITSPPFIDRDIQSEHGKIVILCNKGSVIAFHVVLGYLGSLALGSFTLAFLARNLPDTFNEAKFLTFSMLVFCSVWITFLPVYHSTRGKVMVVVEVFSILASSAGLLMCIFVPKCYVILVRPDSNFIRKYKDKFRY
>Mouse.locus755.1
CVSCPDDKYANIEKTHCLPRAVSFLAYEDPLGMALGCMALSFSAITILVLVTFVKYKDTPIVKANNRILSYILLIALVFCFLCSLLFIGHPNQVSCILQQTTFGVFFTVAISTVLAKTITVVMAFKLTTPGRRMRGVMMTGAPKLVIPI
CTLIQLILCGIWLVTSPPFIERDIKSEHGKIVILCNKGSVIAFHVVLGYLGSLALGSFTMAFLARNLPDRFNEAKFLTFSMLVFCSVWITFLPVYHSTRGTVMVVVEVFSILASSAGLLGCIFVPKCCVILVRPDSNFIQKYKDKLLY
>Mouse.locus338.1
CVSCPDDKYANIEKTHCLPRAVSFLAYEDPLGMALGCMALSFSAITILVLVTFVKYKDTPIVKANNRILSYILLIALVFCFLCSLLFIGHPNQVSCILQQTTFGVFFTVAISTVLAKTITVVMAFKLTTPGRRMRGVMMTGAPKLVIPI
CTLIQLILCGIWLVTSPPFIERDIKSEHGKIVILCNKGSVIAFHVVLGYLGSLALGSFTMAFLARNLPDRFNEAKFLTFSMLVFCSVWITFLPVYHSTRGTVMVVVEVFSILASSAGLLGCIFVPKCCVILVRPDSNFIQKYKDKLLY
>Mouse.locus87.1
------
IEKTNIEKTHYLPRAVSFLAYEDPLGMALGCMALSFSAITNIVLVTFVKYKDTPIVKANNRILSYILLISLVFCYLCSLLFIGHPNQATCILQQTTFGLFFHCGCFYSVGQNNNHGPGFQAHYSRKKDERDAASGTPNLVIPICILIQL
VLCGIWLVTSPPFIERDIKSEYGKIIITCNKGSIIAFHAVLGYLGSLAPGSFTVAFLARNLPDRFNEATFLTFSMLVFCSVWITFLPVYHSTRGTVMVVVEVFSILVSSAGLLGCIFVPKCYVILVRPDSNFIQNYKDKLLY
>Mouse.locus353.1
CVRCPDDKYANLEQMHCLQRAVSFLAYEDPLGMALGCMALSFTAITILVLVTFVKYKDTPIVKANNCILSYILLISLVFCFLCSLLFIGHPNQVTCILQQTTFGLFFTVAVSTVLAKTITVVMAFKLTTPGKRMRG-
MMTGAPKLVIPICTLIQLVLCGIWLLTSPPFIERDIQSEHGKTVIFCNKGSVLSFHVVLGYLGSLALGSFIVAFLARNLPDRFNEAKFLTFSMLVFCSVWITFLPVYHSTRGKVMVVVEVFSILASSAGLLGCIFVPKCYVILVRPDSN
YIQKFKGKLLY
>Mouse.locus352.1
CVRCPDDKYANIEQTHCLQRAVSFLAYEDPLGMALGCMALSFTAITILVLVTFVKYKDTPIVKANNCILSYILLISLVFCFLCSLLFIGHPNQVTCILQQTTFGLFFTVAVSTVLAKTITVVMAFKLTTPGRRMRGMMMTGAPKLVIPI
CTLIQLVLCGIWLLTSPPFIERDIQSEHGKTVIFCNTGSVLSFHVVLGYLGSLALGSFTVAFLARNLPDRFNEAKFLTFSMLVFCSVWITFLPVYHSTRGKVMVVVEVFSILASSAGLLGCIFVPKCYVILVRPDSHFIQKYKGKLHY
>Mouse.locus351.1
CVRCPDDQYANLEQTHCLQRAVSFLAYEDPLGMALGCMALSFTAITILVLVTFVKYRDTPIVKANNCILSYILLISLVFCFLCSLLFIGHPNQVTCILQQTTFGLFFTVAVSTVLAKTITVVMAFKLTTPGGRMRGMMMTGAPKLVIPI
CTLIQLVLCGIWLLTSPPFIDRDIQSEHGKTVIFCNTGSVLAFHVVLGYLGSLALGSFTVAFLARNLPDRFNEAKFLTFSMLVFCSVWITFLPVYHSTRGKVMVVVEVFSILASSAGLLGCIFVPKCYAILVRPDSNCIQKYKGKLLY
>Mouse.locus17.1
CVMCPDDKYANIEQTHCLQRTVSFLAYEDPLGMALGCMALSFSAITTLVLVTFVKYKDTPIVKANNRILSYILLISLDFCFLCSLLFIGHPTQVTCILQQTTFGVFFTVAVSTVLAKTITVVMAFKLTTPGRRMRGMMMTGAPKLVIPI
CTLIQLVLCGIWLVTSPPFIDRDIKSEHGKTVILCNKGSVIAFHFVLGYLGFLALGSFTVAFLARNLPDRFNEAKFLTFSMLMFCSVWITFLPVYHSTRGKVMVAVEVFSILASCAGLVGCIFFPKCYVILIRSDSIFIQKYKDELHY
>Mouse.locus80.1
CVRCPDDKYANVEKTHCLQRVVSFLAYEDPLGMALGCMALSFSALTVLILVTFVKYKDTPIVKANNRILSYILLISLVFCFLCSLLFIGHPNQATCILQQITFGVFFTVAISTVLAKTITVVLAFKLTTPGRRMRGMLASGAPNLAIPI
CTLIQLVLSGIWLVKSPPFIDRDTQSEHGKTIIICNKGSVIAFHFVLGYLGSLALGSFTVAFLARNLPDRFNEAKFLTFSMLVFCSVWITFLPVYHSTRGTVMVVVEVFSILASSAGLLGCIFLPKCCVILD-----------------
>Mouse.locus82.1
CVRCPDDKYANLEKTHCLQRAVSFLAYEDPLGMALGCMALTFSALTVLVLVTFVKYKDTPIVKANNRILSNILLISLVFCFLCPLLFIGHPNKATCILQQTTFGVFFTVAISTVLAKTITVLMAFKLTSPGGKMRGMLASGAPNLVIPI
CTLVQLVLCGIWLVTSPPFIDRDTQSEHGKTIIICNKGSVIAFHFVLGYLGSLALGSFTVAFLARNLPDRFNEAKFLTFSMLVFCSVWITFLPVYHSTRGTVMVVVEVFSILASSAGLLGCIFVPKCFVILVRPDSNFLQKYKDKLFY
>Mouse.locus85.1
CVRCPDDKYANLEKTHCLQRAVSFLAYEDPLGMALGSMTMCFSALTVLVLVTFVKYKDTPIVKANNRILSYILLISLILCFLCSLFFIGQPNHATCILQQTTFGVFFTVAISTVLAKTITVVMAFKLTTPGRRMRGMLASGTPNLVIPI
CSLVQLVLCGIWLVTSPPFIDRDTQSEHGKTIIICNKGSVIAFHFVLGYLGSLALGSFTVAFLARNLPDRFNEAKFLTFSMLVFCSVWITFLPVYHSTRGTVMVVVEVFSILASSAGLLGCIFVPKCFVILVRPDSNFLQKYKDKLFY
>Mouse.locus86.1
CVRCPDDKYANLEQTHCLQRAVSFLAYEDPLGMALGCMALCFSALTLLVFFTFVKYKDTPIVKANNRILSYILLISLVFCFLCSLLFIGHPNQATCILQQTTFGVFFTVAISTVLAKTITVVMAFKLTAPGRRVRGMLVAGAPNLAIPI
CTLIQIVLCGIWLVTSPPFIGRDTKSEHGKTILICNKGSVIAFHFVLGYLGSLALGSFTVAFLARNLPDRFNEAKFLTFSMLMFCSVWITFLPVYHSTRGKAMVVVEVFSILASSAALLGFIFVPKCYVILVKSDSNFREKYKNKFHY
>Mouse.locus271.1
CVKCPENQYANEDHTLCLEKVVAILDYKDPLGKALAGFALCFSVLTSVVLGIFLKNRDTPIVKANNQSLSFVLLISLIFCFICSLLYIGHPTMVICILQQTTFAIVFTVATSTILAKTVIVVLAFKITVPGRRMRWLLEIGAPKYIILI
CTIIQLILCGIWLGTSPPFVDADVHMVHGHIIIVCNKGSVIAFYCVLGYMGSVALASFTVAFLARNLPDTFNEAKLLTFSMLVFCSVWITFIPVYHSTKGKTMVAVEVFSILASSAGLLLCIFAPKCYIILLKPQKNSFQKFRKPHAI
>Mouse.locus289.1
CVKCPEDQYANEDHTVCLQKVVTILDYRDPLGKALAGFALCFSVLTSLVLVVFLKHRDTPIVKANNQTLSFVLLISLIFCFICSLLYIGHPTMAICILQQTTFAIVFTVATSTILSKTVIVILAFKITVPGRRMRWLLESGTPKYIIPI
CTIIQLILCGIWLGTSPPFVDADLHMVHGHILIVCNKGSVIAFYCVLGYMGSVALASFTVAFLARNLPDTFNEAKLLTFSMLVFCSVWITFIPVYHSTKGKTMVAVEVFCILASSAGLLLCIFAPKCYIILLRPQKNYFYKFRK-RRE
>Mouse.locus276.1
CVKCLEDQYANEDHTLCLQKVVVIFDYRDPLGKALAGFALCFSVLTSVVLFVFFKHRDTPIVKANNQTLSFVLLISLIFCFICSLLYIGHPTMVTCIVQQITFAIGFTVATSTILAKTVIVVLAFKITVPGRRMRWMPESGAPKYIILI
CTMIQLILCGIWLGTSPPFVDADVQMVHGHIIIICNKGSVIAFYCVLGYMGSLALASFTVAFLARNLPDTFNEAKLLTFSMLVFCSVWITFIPVYHSTKGKIMAVVEVFCILASSAGLLLCIFAPKCYIILLRPQTNYFHKFWKSHAN
>Mouse.locus268.1
CVKCLEDQYANEDHTLCLQKVVAILDYRDPLGKALAGFALCFSLLTSVVLFVFLKHRDTPIVKANNQTLSIVLLISLIFCFICSLLYIGHPTMVICILQQTTFAIAFTVATSTILAKTITVVLAFKITVPGRRMRWLLVSGAPKYIILI
CTMIQLILCGIWLGTSPPFVDADVQMVHGHIIIVCNKGSVIAFYCVLGYMGSVALAGFTVAFLARNLPDTFNEAKLLTFSMLVFCSVWMTFIPVYHSTKGKTMVAVEVFCILASSAGLLLCIFAPKCYFI-LRPQTNSFHKFRKPHAK
>Mouse.locus261.1
CVKCQEDQYANEDHTLCLQKVVSILDYRDPLGKALAGIALCFSVLTSGILCIFLKHRETPIVKANNQTLSYVLLISLVFCFICSLLYIGHPTTVICILQPITFAIAFTVATSTILAKTITVLLAFKITIPGRRMRWLLLSGVPKYIVLI
CTMIQLILCGIWLGTSPPFVDADVYMVHGHIIIVCNKGSVIAFYCVLGYMGSVALASFTVAFLARNLPDTFNEAKLLTFSMLVFCSVWITFIPVYHSTKGKTMVAVEVFSILASSAGLLLCIFAPKCYIILLRPQTNSFHKFRKTHSK
>Mouse.locus264.1
CVKCREDQYANEHHTLCLQKVVSILDYRDPLGKALAGIALCFSFLTSVVLCIFLKHRETPIVKANNQTLSYVLLISLVFCFICSLLYIGHPTAVICILQQTTFVIAFTVATSTILAKTITVLLAFKITIPGRRMRWLLVSGAPKYIILI
CTMIQLILCGIWLGTSPPFVDADAHMVHGHIIIVCNKGSVIAFYCVLGYMGSVALASFTVAFLARNLPDTFNEAKLLTFSMLVFCSVWITFIPVYHSTKGKTMVAVEVFCILASSAGLLLCIFAPKCYIILLRPDINSFQKFRKPHAN
>Mouse.locus280.1
CVKCREDQYANEDHTLFLQKVVSILDYREPLGKALAGFALCFSVLTSGIFCVFLKHRETPIVKANNQTLSYVLLISLIFCFICSLLYIGHPTTVICILQQTTFAIAFTVATSTILAKTITVLLAFKITIPGRRMRWLLVSGAPKYIILI
CTMIQLILCGIWLGTSPPYVDADVHMVHGHIIIVCNKGSVIAFYCVLGYMGSFALTSFTVAFLARNLPDTFNEAKLLTFSMLVFCSVWITFIPVYQSTKGKTMVAVEVFCILASSAGLLLCIFAPKCYIILLRPHINSFRNFRKPHAK
>Mouse.locus286.1
CVKCPEDQYANEDHTLCLQKIVAILDYRDPLGKALAGFALCFCVITSGVLCIFLKHRETPIVKANNQTLSYVLLISLIFCFICSLLYIGHPTIVICVLQQSTFAISFTVAASTILAKTITVVLAFKVTVPG-
RMRWLLVAGAPKYIILICTMIQLILCGVWLGTSPPFVDADLHMVHGHIIIVCNKGSVIAFYFVLGYMGSIALASFTVAFLARNLPDTFNEAKLLTFSMLVFCSVWVTFIPVYYSTKGKTMVAVEVFCILASSAGLLLCIFAPKCYNILL
RPGKNSSHMFKKLHAK
>Mouse.locus298.1
CVKCPEDQYANEDQTLCLQKVVDVLDYRDPLGKSLAGFALCFSVLTSIVLCVFLKHRESPIVKANNQTLSYVLLISLIFCFICSLLYIGHPTMFICILQQTAFAIAFTVAASTVLAKTITVILAFKITVPG-
RMRWLLVSGAPKYIIFVCTMIQLIFCGIWLGTSPPFVETDVHMTHGHIIIVCNKGSVIAFYCVLGYMGSVALASFTVAFLSRKLPDTFNEAKLLTFSMLVFCSVWITFIPVYHSTKGKTMVAVEVFCILASSAGLLLCIYAPKCYIILL
RPQKNSFYKFRKPHSK
>Mouse.locus255.1
CVKCPEDQYANEDQTLCLQKVVAVLDYRDPLGKALAGFALCFSVLTSVVICVFLKHRETPIVKANNQTLSYVLLISLIFCFMSSLLYIGHPTMVICILQQTTFAIVFTVAASTILAKTITVVLAFKATVPGRRMRWLLVSGAPKYIILI
CTMVQLILCGIWLRISPPFVDADIHMLHGQIIIVCNKGSVVAFYCVLGYMGSIALASFTVAFLARNLPHTFNEAKLLTFSMLVFCSVWITFIPVYHSTKGKTTVAVEVFCILASSAGLLLCIFAPKCYLILLRPQTNSFYKFRRPHAK
>Mouse.locus309.1
CVKCPEDEYANTDQTNCLKKVVTFLDYKEPLGMALAVLAVFFSSLTFIILCVFLKHRDTPIVKANNETLSYVLLTSLIFCFICSLLYIGYPTMVSCILQQTTFALVFTVAASCVLAKTITVVLAFKITVPGRRLRWLLLSGAPNYIIPI
CTTIQMILCGIWLGTSPPFVDADLHMIHEHIIIVCNKGSVTAFYCVLGYMGSLALASFTVAFLARNLPDTFNEAKLLTFSMLVFCSVWVTFLPVYHSTKGKAMVAVEVFCILASSAGMLLCIFVPKCYLILLRPQINSFHEFRKIHAK
>Mouse.locus311.1
CLKCPEDEYANTDQTNCLKKVVTFLDYKEPLGMALTGLAVLFSSLTVVVLCVFLKHRDTPIVKANNETLSYVLLISLIFCFICSLLYIGYPSMVSCILQQTTFAMVFTVSASCVLAKTITVVLAFKITVPGRRLRWLLLSGAPNYIIPI
CTTIQMMLCGIWLGISPPFVDADLHMVHGHIIIVCNKGSVIAFYCVLGYMGSLALASFTVAFLARNLPDTFNEAKLLTFSMLVFCSVWITFLPVYHSTKGKAMVAVEVFCILASSAGMLLCIFVPKCYLILWRPQINSFHEFRKMHVK
>Mouse.locus305.1
CVKCPEDEYANTNQTKCFKKVVTFLAYEDPSGMALAVLAVCFSVLTSVVLCVFLKHRNTPIVKANNETLSYVLLTSLIFCFICSLLYIGYPTMVSCILQQTTFAIVFTVAASCVLAKTITVVLAFKITVPGRRLRWLLLSGAPNYIIPI
CTMIQMILCGIWLGTSPPFVDADLHMVHGHIIIVCNKGSVIAFYCVLGYMGSLALSSFTVAFLARNLPDTFNEAKLLTFSMLVFCSVWITFLPVYHSTKGKAMVAVEVFCILASSAGMLVCIFLPKCYIILLRPQINSFHDFRKIHVK
>Mouse.locus302.1
CFKCPDNQYANPEGTHCLSKIVTFLSYEDPMGVVLACLALGFSALTAAVFGIFLRNQDTPIVKANNRALSYCLLISLIYCFLCSLLFIGQPHILTCIMQQTIFAVVFTVAISTVLTKTITVVLAFRFTLPSKRMRWLMTSGASILIIPI
CTMIQLIICGIWLGTSPPFVDSDGHIEHGHILVVCNKGSVIAFYCVLGYLGSIALTSFTVAFLARNLPDTFNEAKFLTFSMLVFCSVWLTFLPVYHSTNGKALVAVEVFCILTSTAGIFLCIFAPKCYIILLKPENKSFHKFRHKNAN
>Mouse.locus254.1
CVKCPNDQYANIKQTHCLKKVVTFLAYEDPLGMTLTCLALLFSALTTVILSILLKHQDNPIVKANNRLLSYILLISLIFCFLCSLLYIGHPHMATCILQQTTFAVVFTVAVSTILAKTITVVMAFKITDPRKKIRQMLVTRAPNYIIPI
CTMIQLILCGTWIGTSPPFVDSDPHFEHGHIIIVCNKGSVIAFYCVLGYLGLLALGSFTVAFLARNLPDRFNEAKFLTFSMLVFCSVWITFLPVYHSTKGKAMVVVEIFSILTSSAGLLVCIFFPKCFTILLKQKVNFTQKFSDTHSK
>Mouse.locus758.1
CVKCPDGQYANIERNHCFPKVVTYLAYADPLGIVLACMALFLSMLSVLVLGIFVKNQYTPIVKANNRTLSYILLISLTLCFLCSLFFIGHPSTARCLLQQITFGILFTVAVSAVLAKTITVLLAFSITIPGRRVKWLLLFGTHKVIIPI
CSLFQLIICGFWLGLSPPFVERDTHTEHGHIIIMCNKGSLIAFYSVLGYLGCLALASFIMAFLVRNLPDTFNEAKFLTFSMLVFCSVWVTFLPVHHSTKGKAMVAVEVFSILASSAGLLGCIFIPKCYIIVLRPDRNFIQRSKGKNHS
>Mouse.locus493.1
----------------------------------------
MALFLSMLSVLVLGIFVKNQYTPIVKANNRTLSYILLISLTLCFLCSLFFIGHPSTARCLLQQITFGILFTVAVSAVLAKTITVLLAFSITIPGRRVKWLLLFGTHKVIIPICSLFQLIICGFWLGLSPPFVERDTHTEHGHIIIMCNK
GSLIAFYSVLGYLGCLALASFIMAFLVRNLPDTFNEAKFLTFSMLVFCSVWVTFLPVHHSTKGKAMVAVEVFSILASSAGLLGCIFIPKCYIIVLRPDRNFIQRSKGKNHS
>Mouse.locus93.1
----------------------------------
MSLACMALGFSVSTAAVLGIFLKYQDSPIIKANNLTLSYILLISLLLCFLCSFLFIGHPNTVSCILQQITFALVFTLALSTVLAKTITVILAFRALKPGRTMRRLFVLGIYNAVIPFCVLIQLITSGIWLATSPPYIDTDSYSEHAHII
ILCNNGSVTAFYCMLVYLGTLALGSFTVAFLARNLPDTFNEAKFLTFSMVVFCSVWVTFVPVYQSTKAKAMVAVEVFSILASSAGLLACIFFPKCYIILREPDKRNLKCFKNKTRI
>Mouse.locus92.1
-------
EYPNPERNHCLPKSVSFLSFEDPLGISLICTALGFSLSTAAVLGIFLKYQDSPIIKANNLTLSYILLISLLLCFLCSFLFIGHPNTVSCILQQITFALVFTLALSTVLAKTITVILAFRALKPGRTMRRLFVLGIYNAVIPICFLIQLI
ISGVWLATIPPYIGTDAYSEHAHIIISCNKGSVTAFYCVLAYLGILALGSFTVAFMSRNLPDTFNEAKFLTFSMMVFFSVWVTFVPVYQSTKAKAMVAVEIFSILVSSAGLLGCIFFPKCYIILREPDKNNLRCFKNKRHC
>Mouse.locus317.1
-------
EYPNPKRNHCLPKSLTFLSFKDPLGMSLACTALGFSVRSAAVLGIFPKHQDSPIIMANNLTLSYILLISLLLCFLCSFLFIGHPNTVSCILQQITFAHVFTLALSTVLAKTITVILAFRALKAGRTMKKLFVLSAYNAVIPICVLIQLI
ISGIWLATSPPYINTDAYSEHAHIIILCNKGSVTAFYCMLAYLGTLALGSFTVAFLARNLPDTFNEAKFLTFSMVLFCSVWVTFAPVYQST--KAMVAVEVFSILASSAGLLGCIFFPMCYIILREPDKNTLRCFKNKRHC
>Mouse.locus220.1
CLLCPKEQYSSHTRDHCLPRTEIFLAFEEPLGFILALVALLLAGLAVLVLGVFLKHRDTPVVRANNRTLSYFLLISLSLCALCALLFLGRPTVTTCLLRQTIFAVVFTVAVSSVLAKTLTVVLAFRVTRPRSRIQVCLSSGASTSVVLI
ASLIQVVLCGIWLITSPPFPDRDMISEPRHTVIQCHEGSGANFFCVLGFLGFLAGGTFSVAFLARGLPDVFNETKFLTFSMLLFCSVWTAFLPLYHSARGKSTVAVEIFSILASTAGLLGGIFIPKCYIILLKPEKNTPAWLRQGHQV
>Mouse.locus221.1
CLLCPKEQYSSHTRDHCLPRTEIFLAFEEPLGFVLALVALLLAGLAVLVLGVFLKHRDTPVVRANNRTLSYFLLISLSLCALCALLFLGRPTVTTCLLRQTTFAVVFTVAVSSVLAKTLTVVLAFRVTRPRSRIQICLSPGTSTFVVLI
```

```
ASLIQVVLCGVWLATSPPFPDKDMISEPQHIVIQCQEGSGATFFCVLGYLGFLAGGTFSVAFLARGLPDIFNETKFLTFSMLLFCSVWTAFLPLYHSARGKSTVAVEIFSILASTAGLLGGIFIPKCYIILLKPEKNTPAWLKQGYCA
>Mouse.locus223.1
CLLCPKEQYSSHTRDHCLPRTEIFLAFEEPLGFILALVALLLAGLAVLVLGVFLKHRDTPVVRANNRTLSYFLLISLSLCALCALLFLGRPTVTTCLLRQTTFAVVFTVAVSSVLAKTLTVVLAFKVTRPRSRIQICLSPGTSTLVVLI
ASLIQVVLCGVWLATFPPFPDKDMLSEPQHIVIQCHDGSGATFFCVLGYLGFLAGGTFSVAFLARDLPDVFNETKFLTFSMLLFCSVWTAFLPLYYSARGKSTVAVEIFSILASTAGLLGGIFIPKCCIILLKPEKNTSSWLRQGHHV
>Mouse.locus222.1
CLLCPKEQYSSHTRDHCLPRTEIFLAFEEPLGFILALVALLLAGLAVLVLGVFLKHRDTPVVRANNRTLSYFLLISLSLCALCALLFLGRPTVTTCLLRQTTFAVVFTVAVSSVLAKTLTVVLAFKVTRPRSRIQICLSPGTSTLVVLI
ASLIQVVLCGVWLATFPPFPDKDMLSEPQHIVIQCHDGSGATFFCVLGYLGFLAGGTFSVAFLARDLPDVFNETKFLTFSMLLFCSVWTAFLPLYYSARGKSTVAVEIFSILASTAGLLGGIFIPKCYIILLKPEKNTPSWLKQGHHI
>Mouse.locus108.1
CIKCPEDQYPNKQKNNCLPKIKIFLSHEDTLGAVLVSLAISLSAFSAMILGLFIHYRDTPIVRANNRNLSYVLLVSLMLCFFCSLIFIGQPSTVTCVLRQMIFGVVFSVAVSAILAKTFIVVVAFTAIKPGSTLQMWMVTRLSNAIVCC
GSIIQVCICAVWLGTYPPFPDADMHSEFGQIILWCNEGSTLAFYCVLGYLGFLSSLSLLIAFLARRLPESFNEAKTITFSMLVFCTVWITFVPTYLSSKGKTMVAVEILSILASSSILLLCIFLPKCYVILLRSGGHSRKKFFK----
>Mouse.locus106.1
CIKCPEDQYPNKQKNNCLPKIKIFLSHEDTLGAVLVSLAISLSAFSAMILGLFIHYRDTPIVRANNRNLSYVLLVSLMLCFFCSLIFIGQPSTVTCVLRQMIFGVVFSVAVSAILAKTFIVVVAFTAIKPGSTLQMWMVTRLSNAIVCC
GSIIQVCICAVWLGTSPPFPDVDMHSEFGQIILWCNEGSTLAFYCVLGYLGFLSSLSLLIAFLARRLPESFNEAKTITFSMLVFCTVWITFVPTYLSSKGKTMVAVEILSILASSSILLLCIFLPKCYVILLRSGGHSRKKFFK----
>Mouse.locus105.1
CIKCPEDQYPNKQKNYCLPKITTYLSHGDTLGAVLGSVAISLSIFSAMILGLFIHYRDTPIVRANNRNLSYLLLVSLMLCFFCSLIFIGQTRTVTCVLRQMIFGVVFSVTISAILAKTFIVVVAFNAIKPGSRLQMWMVIRLSYAIVCC
GSIIQVCICAVWLGTYPPFPDVDMHSEFGQIILWCNEGSTFAFYFVLGYLSFLSSLSLLIAFLARRLPDSFNEAKTITFSMLVFCSVWISFVPTYLSSKGKTMVAVEIVSILVSSSILLLCIFLPKCYVILLRSGGHSRKKFFK----
>Mouse.locus115.1
CIKCPEDQYPNKQRNQCLPKIAAFLSHEDILGAILVSMATSFGTFSAMILGLFIHYRDTPIVRANNRNLSYLLLISLMLCFFCSLMFTGDPRRVTCVLRQMIFGVVFSIALSAILAKTFIVVVAFKTIKPGSILKMGIVTRLSNAIVCC
GSIIQVCICAVWLGTYPPFPDVDMHSEFGQIILLCNEGSTFAFYCVLGYLGFLASLILLIAFLVRRLPDSFNEAKTITFSMLVVCSVWICFIPTYLSSTGKTMVAVEIVSILASSASLLSFIFVPKCYVILLRSGGQSRKEFFK----
>Mouse.locus116.1
CIKCPEDQYPNKKRSQCLPKITAFLSHEDTLGAILVSVAISFAAISAMIFGLFIHYRDTPIVRANNRNLSYVLLISLILCFFCSLMFIGEPRRVTCVLRQMTFGVVFSIALSAILAKTFIVVVAFKTIKPGSILKMWMATRISNAIVCC
GSIIQVCICAVWLGTYPPFPDVDMQSEFGQIILLCNEGSTLAFYCVLGYLGFLASLSLFIVFLVRRLPDSFNEAKTITLSMLIFCSVWISFLPTYLSSKAKAMVAVEIFSILVSSASLLLFIFVPKCYVILLKSGGHSRKKFFK----
>Mouse.locus117.1
CIKCPEDQYPNEQRNQCLPKITAFLSHEDTLGAVLGSMAISFAAFSAMILGLFIHYRDTPIVRANNRNLSYLLLVSLKLCFFCSLMFIGEPRRVTCLLRQMIFGVVFSVALSAVLAKTFIVVVAFKVIKPGSTLQMRMATQISNAIVCC
GSIIQVCICAVWLGTYPPFPDVDMHSEFGQIILWYNEGSTLAFYCVLGYLGFLASLSLFIAFLVRSLPDSFNEAKTITFSMLVFCSVWISFVFTYLRSKAKTMVAVEIVSILASSAGLLSFIFLPKCYVILLRSGGYSRKKFFKSVSP
>Mouse.locus113.1
CIKCPEDQYPNKQRNQCLPKIMVFLAHEDPLGTVLVSLAISLSAFSAMILGLFICYRETPIVRANNRNLSYLLLISLKLCFFCSLIFIGQPRTVTCVLRQIIFGIVFSIVISAILAKTFIVVVAFKAIKPGSILKMGMVTRLSNAIVCC
GSIIQVCICAVWLGTYPPFPDVDMQSEFGQVILWCNEGSTLAFYCVLGYLGFLASLSLLIAFLARRLPDSFNEAKTITFSMLGFCSVWISFIPTYLSSKGKTMVAVEILSILASSAGLLGCIFLPKCYVILLKSGGHSRKKFFK----
>Mouse.locus114.1
CIKCPEDQYPNKQRNQCLPKIMVFLAHEDPLGTVLVSLAISLSAFSAMILGLFICYRETPIVRANNRNLSYLLLISLKLCFSCSLMFIGQPRTVTCVLRQIIFGIVFSIVISAILAKTFIVVMAFKAIKPGSILKMGMVTRLSNAIVCC
GSIIQVCICAVWLGTYPPFPDVDMHSEFGQIILWCNEGSTLAFYCVLGYLGFLASLSLLIAFLARRLPDSFNEAKTITFSMLVFCSVWISFVPAYLSSKGKTMVAVEILSILASSAGLLGCIFLPKCYVILLKSGGHSRKKFFK----
>Mouse.locus111.1
CIKCPEDQYPNKQRNQCLPKIMAFLSHEDTLGALLVCTAISLSAFAAMILGLFIHYRDTPIVRANNRNLSYLLLVSLMLCFFCSLIFIGQPGTVTCVLRQMIFGVVFSIAVSAILAKTFIVVVAFKSIKPRSTLQMWIVTRLSITIVCC
GSIIQVCICAVWLGTYPPFPDVDMHSEFGHIILCCNEGSTLAFYCVLGYLGFLASLSLLIAFLVRRLPDSFNEAKTITFSMLVFCSVWISFIPTYLSSTGKTMVAVEIFCILASSAGLLGCMFLPKCYVILLRSGDHSRKKVFK----
>Mouse.locus112.1
CIKCPEDQYPNKQRNQCLPKIMVFLSHEDTLGAVLVSLAISLSAFSAMILGLFVCYRDTSIVRANNRNLSYLLLISLMFCFFCSLIFIGQPSTVTCVLRQMIFGVVFSVAVSAILAKTFIVVVAFKAIKPGSRLQMWMVMRLSNVIVCC
GSIIQVCICAVWLGTYPPFPDVDMHSEFGHIILCCNEGSTLAFYCVLGYLGFLASLSLLIAFLVRRLPDSFNEAKTITFSMLVFCSVWISFIPTYLSSTGKTMVAVEIFSILASSAGLLGCMFLPKCYVILLRSADLSRKKFFK----
>Anole_lizard.locus187.1
CMKCKKDFYPNKARNKCILKSITFLSYTEPLGIVSASVALFFSLLSALVLAMFIKHSESAIVKANNQHLSYILLSSLMLCFLCPLLFIGRPRKITCIFRQTAFGIIFSVSLAAVLAKTITVVLAFKATKPGSGTRKWLRPRLSYFFVIV
CSLIQGVICIVWLGTSPPFPDADFYSEPGHIILECNEGSPIAFYSILGYMGLLALVSFIVAFLARKLPDIFNEAKFITFSMLVFCTVWIAFIPTYLSTKGKYMVSVEIFSILTSGAGLLGCIFLPKCYVLLLHLDKKQKVHITSKYFH
>Anole_lizard.locus188.1
CMKCKEDFYPNKARKKCIPKHIVFLSYAEPLGIVSASIALYFSFLSALVLGIFIKHRDSPTIKANNRHLSYILLSSLILCFLCSLLFIGRPRKITCILRQTAFGIIFSVSLATILAKTITVVLAFKATKPGSSAQKWLRPKFSYFFVMV
CFLIQAVICMVWLGTSPPFPDADFYSEPGHIILECNEGSSIAFYSILGYMGLLALVSFIVAFLARKLPNVFNEAKFITFSMLVFCVVWISFIPAYLSTKGKYIMAVEIFSILASSAGLLGCIFLPKCYVLLLHLDKNQKVHITRKYFD
>Anole_lizard.locus186.1
CVVCEEHLYSNEARNACIPKNIIYLSYEEPLGIISASTSFSFSVFAALVLGTFIKFRERPTVKANNRTLSYILLSTLILCFLCCLLFIGRPRKMTCLFQQPVFGIIFSVSLATVLAKTITVVLAFKATKPGSGARKWLRPRLSYFFVMV
CSLIQVVICMVWLGTSPPFPDADFYSEPGHIILECNEGSPIAFYSILGFMGFLALVSFIVAFLARKLPDIFNEAKFITFSMLVFCTVWVTFIPAYLSTKGKYTVTVEIFSILASNAGLLGCIFLPKCYVILLHLDKNQKVQINNKYFH
>Anole_lizard.locus190.1
CVDCKKDRYPNKGRNKCIPKNITFLSYEEPLGIALAFIAVSFSFLSALILGNVIRYRESPIVKANNRQLSYVLLSSLILSFLCSLLFIGRPSNRTCFLRQTVFGIIFSVSLATVLAKTITVVLAFKATKPGSGTRKWLRPRVSYFFVMV
CSLIQVVICMIWLGTSPPFPEADFYSEPGHIILECNEGSPSAFYSNLAYMSVLALVSFIVAFLARKLPDSFNEAKFITFSMLIFCTVWTSFIPAYLSTKGKYTVAVEIFSILTSSAGLLGCIFFPKCYIILLYPNKDQKAHIISKHFQ
>Anole_lizard.locus117.1
CFACPGYQYPNNDKDGCLPKEITFLSFKEPLGIVLAVLALTFSLITVFILGIFIKYRDTPIVKANNKNLTYILLASLLLSFLCALLFIGQPRKATCLLQQTAFGVIFSVALSSILAKTITVILAFVATKPGSRIRQWMGKRLTYSIVLS
CSLTQVVINMAWLATSPPFPDLDMYSVSQKIVLKCNEDSGAMLYSLLGFQGFLALVSLTLAFLARKLPDSFNEAKFITFSMLVFCSVWLCFIPTYLSTKGKNMVAVEIFSILASSFGLLGCIFSPKCYIILLKPELNSKEQLTKRRN-
>Anole_lizard.locus115.1
---------------------
MFLSFEEPLGIVLAVFALKFSLITVFILGIFIKYRDTPIVKANNKNLTYILLTSLLLSFLCALLFIGQPGKVACLLQQTAFGVIFSVALSSILAKTITVILAFVATKPGSRIRQWMGKRLAYSIVLSCSLTQAAVSIAWLAISPPFPDL
DMYSVSQKIVLKCNEDSGTMLYSLLGFLGFLALVSLTLAFLARKLPDSFNEAKFITFSMLVFCSVWLCFIPTYLSTRAKSMVAVEIFSILASSFGLLICIFSPKCYVILLKPELNSKEQLIKRI--
>Anole_lizard.locus124.1
CFECPRNQYPNNDKDGCLPKEITFLSFGEPLGIILTVFALKFSLITVFILGIFMKYRDTPIVKANNKNLTYILLASLLLSFLCALLFIDQPRKATCLLQQTAFGVIFSAALSSILAKTITVILAFVATKPGSRVRQWMGKRLAYSIVLS
CSLTQAAVSIAWLAISPPFPDLDMYSVSKKIVLKCNEGSGSMLYALLGFLGFLALVSLTLAFLARKLPDSFNEAKFITFSMLVFCSVLLCFIPTYLSTRGKSMVAVEIFSILASSFGLLGCIFSPKCYIILLKPELNSKEQLIKRRNK
>Anole_lizard.locus119.1
CFECPGYQYPNNDKDGCLAKEIIFLTFEEPLGIILVVFALKFSLITVFIFGNFMKYRDTPIVKANNRNLTYILLASLLLSFLCALLFIVRPGKVVCLLQQTAFGMVFSVALSSILAKTIIVLLAFITTKPGSRVRRWMGKRLAYSIVLS
CSLTQAAVSIAWLAISPPFPDLDMYSVSQKIVLKCNEGSGSMLYALLGFLGFLALVSFTLAFLARKLPDSFNEAKFITFSMLVFCSVWLCFIPMYLSTKGKNMVAVEIFSILASSFGLLSCIFTPKCYIILVKPELNSKKQLIKRRN-
>Anole_lizard.locus113.1
CFDCPGYQYPNDDKDGCLPKEITFLSFGEPLGIIVTVFALKFSLITVFILGIFMKYKDTPIVKANNKDITYILLVSLLLSFLCVLLFIGRPGKVACLLQLTAFGMIFSVALSSILAKTITVILAFVATKPGSRVRQWMGKRLAYSIVLS
CSFFQATICSIWLITSPPFPDLDMYSVAQTIVFKCNEGSSGMLYALLGFLGFLALVSLTLAFLARKLPDSFNEAKFITFSMLVFCSVWLCFIPTYLSTKGKNMVAVEIFSILASSFGLLGCIFSPKCYIILMKPELNTKEQLTKRRN-
>Anole_lizard.locus112.1
CFDCPGYQYPNDNKDGCLAKEITFLSFGEPLGIILTVFALKFSLITVFILDIFMKYKDTPIVKANNKNLTYILLSSLLLSFLCVLLFIGRPGKAACLLQQTAFGVIFSVALSSILAKTITVILAFVATKPGSRVRQWMGKRLAYSIVLS
CSFFQATICSIWLITSPPFPDFDMYSVAQTIVLKCNEGSSGMLYALLGFLGFLALVSLTLAFLARKLPDSFNEAKFITFSMLVFCSVWLCFIPTYLSTRGKSMVAVEIFSILASSFGLLGCIFSPKCYIILVKPELNTKEQLIKRRN-
>Anole_lizard.locus107.1
CFECPKGQYPSNDQHECIPKHIIFLSYHEPLGVSLTILSLTFVFITALVLGIFFKYQDTAIVKANNRSLSYVLLISLLLVFSCVFLFIGQPNHLICLLRDCLFSVLFTVALSCVLAKTITVILVFKATTPESKMKKWVSKPLVNSIVFS
CPFIKLIICMVRLIIFPPFLDSDMELMAKYIVLYCNPGSHLMLYSCLSTFGLLIILSFSMAFLARKLPDIFNEAKFITFSMLAFSSVWLTFIPTYLSTKGINMVTVEIFCILASGAALLICIFSPKIYIILLKPELNNKKLLIRTKT-
>Anole_lizard.locus120.1
CFYCLEDHYPNHDQDLCIQKNIIFLSYEEPLGICLAILALFLSFMAVFMLGIFIKHQDTPIVKANNRTLTYILLTSLLLSFLCVFLFLGQPNRIICLLRQTTFGVIFSLALSSVLAKTILVILAFMSTMPRSWMRKWMGKHLAVSIVLC
CSFIQATICIIWLTTFPPFPDFDMQSMAEEIVLVCNEGSPAMFYSVLSFLGFLATVSFIVAFLARKLPENFNEAKFITFSMLIFCTVWLSFVPTYLSTKGKYMVVVEIFSILGSGAGLLGCIFSPKCYVILLKPELNHKKQFIRKKNL
>Anole_lizard.locus135.1
CFKCSDDQYANLDQNSCFPKTITFLSYEEPLGISLASCAVLFSFTVAVILRIFMKHHNTPIVKANNQSLTYILLISLLLSFLCTLIFIGQPKKITCYLRQSVFGFIYNIAVSTVFAKNVTVILAFKATQPGSRIKKWMGRKLTISIIFS
SSIIQTGIWLVYMTIYSPSPETDMHSLREEIVLQCHEPPSVVLCYLLVFMGILATGSFSVAFFAHKVPDSFNESKYITISMLLFCSVWLIFIPSHMSTKGKTKVVVEIFAMLASSAGLLGCLFFPKCYIILLKPELNKKEIFFKKSTE
>Anole_lizard.locus111.1
CFQCPGDHYPNKFKDFCIPKSLTFLTFEDALGTGLASSALLLFCVTAGVLGIFIKHRDTPIVKANNRNLTYTLLISLLLSFLCALLFIGKPQKMTCLFRQTAFGIIFSVAVSSVLAKTITVILAFQATKPESTLRKWMGKRTATAIILF
CFLIQATICTVWLTTFPPFPDFDVNSMAEEVILECNEGSVFMFYCLLGFMGFLSICSFTAAFFARKLPDSFNEAKFITFSMLVFCSVWISFVPTYLSTKGKNMVAVEIFSILASSTGLLGFIFFPKCYIIVLRPEMNRKEQLIKRRH-
>Anole_lizard.locus114.1
CFNCPKDHYPNKNRDACLPKDISYLTYEEPLGISLVIIALTLFFITAWVFWIFRKHRNTPIVKANNQNLTFTLLISLLLSFLCIFLFIGHPKKVTCLLRQMAFGLIFSVAVSSILAKTTLVIVAFMATKPGSRIRKWVGRWLSSSIVFS
CSLIQATICSVWLAISPPFPDVDMHSVTEEIVLECNEGSSTMFYCVLGFLGFLASFGFTVAFLARKLPDSFNEAKFITFSMLMFCCVWLTFVPSYLSTKGKYMVAVEIFSILTSSAGLLGCIFSPKCYVILLRPDLNRREQLKKRNHQ
>Anole_lizard.locus155.1
CYKCTDESYPNKKQDMCIPKDIIFLSYEEPLGLSLAILALFCSVITALVLGTFMKHHRTPIVRANNRTLTYTLLIFLLLCFLSAFLFIGRPQKLTCLLQQTAFGIIFSTAISSVLAKTITVILAFMATMPGSSTRKWMGKRLSISTVIS
CSLVQVAICTAWLVSSPPFPDADMHSIPGKIVLKCNEGSITMFCCVLGYMGLLAIVCFSVAFFVRKLPDTFNEAKFITFSMLIFCSVWLSFVPTYISSKGKYMVAVEIFSILASSAGLLGCIFSPKCYIIILRPQLNKRERLIRRKT-
>Anole_lizard.locus144.1
CIECSEDYYPNKRQDSCIPKFITFLSYGEPLGIGLAISALFFSLITVLVLVTFVKHKDTPIVIANNRSLTYTLLLSLLLCFLCTLLFIGQPQMVTCLLRQTAFGIVFSVAISCVLAKTIMVVWAFAATKPRTRMRGWVGKSLANYIVLS
CSIFQAGICMIWLVTSPPFPHFDMHLDVEEILLECNEGSTFTFYCVLAYLGFLALLSFFVAFLARKLPDSFNEAKFITFSMLVFCSVWLSFVPSYLSTKGKYMVAVEIFSILASSAGLLGCIFFPKVYIIVVKPELNSREQLRKVFKG
>Anole_lizard.locus152.1
CYTCRDEYYPNKKHDLCIPRDISYLSYEEPLGIALAFLALLLSFITCFILVIFLKYQNTPIIIANNRNLTYILLIALLFCFLCALLFIGQPQKVTCLLQQTAFGIIFSVAVSCVLAKTITVLLAFMATKPGSKMRKWVGKTLTYCIVIS
CPLIQTGICVVWLATFPPFPSPNTNSVREEIILECNNNSATMFYCVLAYMGFLAAASFVVAFLSRELPDTFNEARFISFSMLVFCSVWISFIPSYLSTKGKSMVAVEIFSILISSTGLLGCITFPKCYIILFRPNLNNRETLMKKKLR
>Anole_lizard.locus109.1
CFHCPSDQYANNAQDACILKKLSFLSYEEPLGISLAIFALLFSFITVLVLGTFVKHRNTPIVKANNESLSYVLLVSLLLSFLCVFLFIGQPGKLTCLLQQTAFGIIFSVCVSCVLAKTILVVLAFKANKPGSKMKKWVGKRLAASIVIF
CSLIQATICAAWLASSPPFLDLDMYSVTENIIVECNQGSFTMFYCVLSYMGFLAIVSFGVAFLARRLPDSFHEAKSITFSMLVFCSVWLSFVPTYLSIRGKYVVAVEIFSILASSAGLLGFIFFPKCCIIVLRPELNKRRNLGRI---
>Anole_lizard.locus150.1
CFQCHEDRYPNKYRNACIPKVVTFLSFEEPLGIGLTSLTLSFLLMTILVFGIFLKHRSTPIVKANNETLTYNLLFCLFLCFLSALLFIGHPRKMTCLLQQAAFGIVFSVAISCVLAKNLTVLLAFMATKPGSRLTKWIGKKLANAIILS
CSLIQTGLCAVWLATDPPFPDLDMHSVAEEIVLECNEGSVTMFYCVLSYMVFLAIASFTVAFLVRKLPNNFNEAKFITFSMLIFCSVWLSFAPVYLSTKGKYVVAVEIFSILTSSGGLLSFIFFPKCYIIVLRPELNNKEQLIKRKVP
>Anole_lizard.locus146.1
CSKCPDDQYTNKNHDQCLPKSIHFLSSTEPLGITLTCLALSLSL--
ILVLKIFIINKNTPIVKANNRNLTYSLLVSLLLCSLCTFLFIGQPKTVTCYLRQTIFGIVFSVAVSCVLAKTLTVVVAFMATKPGSRMRKWVGKGLANAVVLNCSLIQASMCAVWLCIAPPFPNFDMHTLAKEIILECNEGSVTMFYCI
MAYLGFLAMVSFTVAFFARKLPDSFNEAKFITFSMVVFCSVWLCFVPSYLSTKGKYMVAVEIFSILASSAALLGFIFFPKCFIIVLKPELNCKDYLIRKK--
>Anole_lizard.locus149.1
CSKCPDDQYTNKNHDQCLPKSIHFLSFTEPLGITLTCLALSLSLITILVLKIFIINKNTPIVKANNRNLTYVLLVSLLLCSLCTFLFIGQPKTVTCYLRQTIFGTVFSLAVSCILAKTLTVVVAFMATKPGSRMRKWVGKGLANAVVFN
CSLIQASMCAVWLCIAPPFPNFDMHTLAEEIILECNEGSVTMFYCIMAYLGFLAMVSFTVAFFARKLPDSFNEAKFITFSMVVFCSCWLCFVPSYLSTKGKYMVVVEIFSILASSAALLGFIFFPKCFIIVLKPGLNCKDYLIRKK--
>Anole_lizard.locus148.1
CITCPEGQYPNKKQDQCNLKTLNFLSFSEPLGIISTVLSLFFSLVTAIVLVIFTKNKSTPIVKANNVDLSFTLLVSLFFCFLCSLLFIGVPHTVSCYLQQIGFVVTFTVAISCILAKTIIVVVAFMATKPGSRMKNWVRKRLANSILLG
CSSIQVIICTLWLLIFPPFPDVDMHSLSKEIILECNEGSVIMFYCGLSYLGFLALASLFMAFIARKLPATFNEAKLITFGMLVFCSVLLCFLSAYSSIKGKHFAIVEIFSILAVSVGFLVCIFFPKCYILIMRSELNCKGQLVRKISK
>Anole_lizard.locus147.1
------------------------------
MTSAILSLSFALITVFVLGIFIKYKNTPVVKANNRNLTYCLLISLLLCFLCALLFIGKPLQINCYLRQPAFGVIFSVAVSCMLAKTITVVVAFMATKPGTKISKWVGKGLAKCILLSCPLIQVSICVVWLCTAPPFPNFDMYSQAEEII
VECNEGSVTMFYCVLGYMGLLAFITFTVAFLARKLPDGFNEAKFITFSMVMFCSVWLSFILSYLSTKGKYIIAVEIFSILTSSFGLLSCIFFPKCYIIMLRPELNSKDQLLRKK--
>Anole_lizard.locus141.1
CTPCPEDQYPSSNQDHCIPKALNCLLYAETLSIIIIFMTFSFSMITALVLGIFIKYKNTPIVKANNQDLTYTLLISLFLCFLCPLLFIGQPHKIKCLLRQTTFGIIFSVAISSVLAKTITVVLAFITTRPGSKIRKWMGKWMAICIVLS
CSLIQTGICTVWMSIAPPFPDFDKYSSPKEIVVQCNEGSTFMFYCVLGYLGLLAVVSFVVAFFARKLPDAFNEAKFITFSMLVFCSVWLSFVPSYLSTKGKYMVAVEIFSILFSSVGLLGCIFFPKCFIIILKPELNSKEYLTRKNRA
>Anole_lizard.locus99.1
CFACPEDHYPNKERNGCLPKNLNFLSYDQPLGIVLAILALSFSVITVCVFGIFVKHRNTAIVRANNRNLSYTLLISLFLCFLCSLLFIGHPNVLTCQLRQTAFGIVFSVAVSSVLAKTTTVVLAFMITKPGSKLSKWVGTKLATSVVLS
CSSIQIILCSIWLCTSPPFPSLEMNALPKEIVVRCNEGSANMFYLILGYMAFLAFVSFIVAFFARKLPDTFNEAKFITFSMLVFCSVWVSFAPSYLSTKGKYMVAVEIFSMLTSSAAILGFIFSPKCYIIVLRPQLNSRNQLKRKNN-
```

```
>Anole_lizard.locus180.1
CFKCAEDQYSNEHKNKCMNKTIRFLAFEEPLGIGLASIAVSFSLITALVLGVFVKNKDTPIVKANNRDLTYGLLVSLLLCFLSSLLFLGKPSKVTCLLRQPTFGIIFSVAVSCILAKTTLVSLAFLATKPGSRKKKWIGKKLARDIVLP
CSLLQTSICILWLLSFPSFPELDMYSLPEESIVQCNEGSVIMFYSILGYLGLLAIASFAVAFQVRNLPKGFNEAKFITFSMLIFCSVWASFVPTHLSSKGKGMVAVEIFSIVFSSAGILGFIFAPKCYIIVLRPDLNSRELLIQRNR-
>Anole_lizard.locus179.1
CFRCPEDHYPSNDRNMCIPKKTSFLSYGDPLGTSLAVIAASFAFITALVLGVFIKHKDTPLVKANNQNLTYILLASLMLCFLSSFLFLGEPREVTCLLRQSIFGLTFSVAISCILAKTTIVSLAFLATRPGSRMMKWVGNRLVHFIIFP
CSLLQACICLLWLTYSPPYPDVDMKSITKEIIIQCNEGSVVMFYCVIGYIGLLAMASFIVAFQVRKLPDSFNEAKFITFSMLVFCSVWICFVPTYLSTKGKDMVAVEIFSILFSSAALLGFIFAPKCYIIALRPDLNNREQLIKRKN-
>Anole_lizard.locus177.1
CFRCPEDHYPSNGRNTCIPKNITFLSYKDPLEISLSAIAVSFVLITILVLQIFIKHKDTPIVKANNRNLSYILLISLLLCFLSPFLFLGQPEEVRCLLRQPTFGILFSVAVSCVLAKTMIVSLAFLATKPGSWMNKWAGRRLPYSIAVS
SCLIQASICIAWLATSPPFPDVDMHSAHEEIILQCNEGSVTMFYCVLSYMGFLALASFIVAFLVRKLPDRFNEAKFITFSMLAFCSVWISFVPAYLSATGKAMVAVEIFSILTSGASLLGCIFFPKCYIIVLRPGLNNRKHLLRQF--
>Anole_lizard.locus178.1
CFRCPEDHYPSNGRNTCIPKNITFLSYKDPLGISLSAIAVSFVLITILVLQIFIKHKDTPIVKANNRNLSYILLISLLLCFLSPFLFLGQPEEVRCLLRQPTFGILFSVAVSCVLAKTMIVSLAFLATKPGSWMNKWAGRRLPYSIAVS
SCLIQASICIAWLATSPPFPDVDMHSAHEEIILQCNEGSVTMFYCVLSYMGFLALASFIVAFLVRKLPDRFNEAKFITFSMLAFCSVWISFVPTYLSATGKAMVAVEIFSILTSGASLLGCIFFPKCYIIVLRPGLNNRKHLLRQF--
>Anole_lizard.locus181.1
CFECPEDQYPSKSKNKCVPKDISFLSYDDPLGISAASVAFSFFVITALVQVIFIKHQDTPIVKANNRDLSYILLVSLLLCFLSSFLFLGRPGKVSCFLRQAAFGIIFSVAVSCILAKTITVVVAFMATKPGSNMRKWVGKRLANSIVFF
GTSIQVVICTVWLGISPPFPDLDMHSLTGLIVVECNEGSFIMFYLVLGYMGLLSVISFTVAFLARKLPDSFNEAKFITFSMVIFCSVWLSFVPTYLSTKGKYIVAVEIFSILASSAGLLACIFFPKCYVILLRPELNIKDNLIKRKN-
>Anole_lizard.locus182.1
CFECPEDQYPSKSKNKCVPKDISFLSYDDPLAISAASVAFSFFLITALVLAIFIKHQDTPIVKANNRDLSYILLVSLLLCFLSSFLFLGRPGKVSCFLRQAAFGIIFSVAVSCVLAKTITVVVAFMATKPGSNMRKWVGKRLANSIVLF
CTSIQVVICTVWLGISPPFPDLDMHSLTGLIVVECNEGSFIMFYLVLGYMGLLSVISFTVAFLARKLPDSFNEAKFITFSMVIFCSVWLSFVPTYLSTKGKYIVAVEIFSILASSAGLLACIFFPKCYVILLRPELNNKDHLIKRKN-
>Anole_lizard.locus127.1
CIKCAEDQYPNLDQNGCIAKGVSFLSYEDPLGITITLCALLFSFITSLVLGTFIKHKDTPIVKANNRSLSYTLLICLLLCFLCPLLFLGQPGKVTCLLRQVSFGITFSVALSCILAKTILVVLAFLVTKPGSRKSKWVGKRLAAIIVLS
CSFIQTGISAVWLGFSPPFPEVDMRSFKEAIILECNEGSVLMFYCVLGLMGCLSAVSFTVAFLSRKLPDSFNEAKFIAFSMLVFCSVWVSFIPTYLSTKGKYMVAVEIFSILASSAGLLACIFFPKCYIILLKPEMNHKEYLIKGKKR
>Anole_lizard.locus98.1
-------------------------------------------
MLTAFMLAIFIKQRHTPIVKANNRGLSYLLLTSLCLCFLSSLLFIGKPNHLTCLLRQMAFGIVFSIAVSSVLAKTTVVVVAFMGSQSRNIFRKWIGKRMAYSVVLGCSLVQVSICAAWLAYSPPFPDFNKHLITEEIVVECNEGSVTMF
YCVLSYMGFLSFVSFTVAFLARKLPDSFNEAKFITFSMLVFCSVWVSFVPTYLSTKGKYMVAVEVFSILSSGAGILGCIFSPKCYIILLKSNLNNREQLIRRRQN
>Anole_lizard.locus96.1
CISCPEDDYPNRGRDHCIPKVQTFLSIEESLGLTLASLSLFLSVLTAFVLAIFIKQQHTPIVKANNRGLSYLLLTSLCLCFLSSLLFIGKPNHLTCLLRQMAFGIVFSIAVSSVLAKTTVVVVAFMGSQSRNIFRKWIGKRMAYSVVLG
CSLVQVSICAAWLAYSPPFPDFNKHLITEEIVVECNEGSVTMFYCVLSYMGFLSFVSFTVAFLARKLPDSFNEAKFITFSMLVFCSVWVSFVPTYLSTKGKYMVAVEVFSILSSGAGILGCIFSPKCYIILLRSNLNNRMHLIRRKHN
>Anole_lizard.locus97.1
CISCPEDDYPNRGRDHCIPKVQTFLSIEESLGLTLASLSLFLSVLTAFVLAIFIKQRHTPIVKANNRGLSYLLLTSLCLCFLSSLLFIGKPNHLTCLLRQMAFGIIFSIAVSSVLAKTTVVVVAFMGSQSRNIFRKWIGKRMAYSVVLG
CSLVQVGICAAWLAYSPPFPDFNKHLITEEIVVECNEGSVTMFYCVLSYMGFLSFVSFTVAFLARKLPDSFNEAKFITFSMLVFCSVWVSFVPTYLSTKGKYMVAVEVFSILSSGAGLLVCIFSPKCYIILLKSDLNNREQLIRRKQN
>Anole_lizard.locus145.1
CKSCPDYQYANLYRNQCIPKIINYLSYEEPLGIGITLSAIILALISALVLGTFMKHWDTPIVKANNRTLSITLLISLFLCFLCSLLFIGQPTKMTCLLRQTTFGIIFTVSVSSILAKTITVILVFMATKPGSNVRKWVGTMLANAIVLS
CTFIQAGICIIWLGTYPPFPDTNTHSLNLSIIMECNEGSTFMFYCVLGYMGLLAVVSFTVAFLARKLPDSFNEAKFITFSMLVFCSVWLCFVPAYLSTKGKQMVVVEIFSILSSSVGLLTCIFFPKCFVILLRPDLNNREKIMIKKST
>Anole_lizard.locus129.1
CERCPEDQYASKDQVQCIPKVITYLSFDKPLGIALAVSAAFFSLVTLLVMGSFIQHRNTPIVKADNWNITRIILCSLLFCFLSIFLFFGQPGKALCFLRHTVYDILISSTLSCLLGKTLTVLFAFRVTKPGNKIWKWVVKTLGISVPIV
CCLIQTGLFAVWLTTSPPFPEFDKQSQIEQMAVYCKEGSTVFFYTANGYPCLLAIISLTTSFFARRLPNVFNDTKRIVFSMMMHFSILASFVAACLNAARKYMAPVAAFSILIFNSGILAFIFLPKVYIIVLRPELNTKAQLVRKRKY
>Anole_lizard.locus130.1
CVKCQEDHYPNRKQDQCLPKRTTSLYYTEPLGAILASCALVFSVITILVMGTFIRYWNTPIVKANNWTITCILLVSLLLCFLCSFLFLGTPGKVSCLLRQTVFGIVFCIAVSCMLAKTITVVLAFMATKPRNQMKKWIGKKLAILVIVF
CAFIQAGLCTVWLSTSAPFPEFDMRSQLAEIVLQCNEGSDTMFYLVLGYMGLLSFISFTVAFFARKLPSSFNEAKLITFSMLVFCSVWVAFVPSYLSTKGKNMVAVEVFSILVSSFALLGCIFFPKCYIILVRPELNIREKLVKKKN-
>Anole_lizard.locus143.1
CNSCPADHYPNKLRDECIPKIITFLSYEEPLGQAFSSFALLLFFMTFGIWGIFIKYQNTPIVKANNREVSHVLLTSLMLCSVSSFLFIGRPRKLSCLLRQTVFGIVFVVAISCVLAKTITVVLAFLATKPGSKMKERMGKWLSVFIVLF
CFLTQLCIIVVWLGNSPPFPEFDMHSQSGKIIAQCNEGSTAMFYVVLTYIGLLALTSFIVAFFARKLPDSFNEAKFITFSMVVFCSVWVSFVPTYLSSKGLHMVAVEIFSILASTAGLLGFLFLPKCYIIILKPWLNTKEQLIIRKTI
>Anole_lizard.locus140.1
CFRCPEDRYSSKNHDECIPKIVNFLSYEEPLGISLASFAISFSLITVLVLAVFIKHGDTPIVKANNRDITFILLISILFCFLCSLLFLGKPKNVTCILQQSAFGTVFSMAVSSVLAKTTTVVLAFMATKPGSNIRKWVGKRLTGVIVFF
CSLVQGIICVVWLGIAPPFPDVDVESFSEEIIVKCNEGSVNMFYIVMGYMGFLSILSFIVAFTARKLPDSFNETKFISFSMLMFCSVWVSFVPTYLSTKGKYMVAVEIFSILVSGAGLLGCIFFPKCYIILVRPELNSRDQLIRKWK-
>Anole_lizard.locus137.1
CYKCPEEECPNKKRNRCVPKVITFLTYDGPLGICLVSAAVFLSLLTASVFGIFIRHRNTPIVKATNRDLTYRLLISIFLCFLCSLIFIGQPSTVTCLLRQVAFGNIFTFALSCVLAKTITVVIAFIATNPGGKIIKWVGKKLSNYIIFF
CSAIQAGICAIWLGTSPPFP--DKVSQSAEIVAQCNEGSSTMFYIVLGYLGFLASVSFTLAYLTRKLPDTFNEAKFITFSMLVFCSVWISFIPTYLSTKGKYMVAVEIFSILASGVGLLSFIFAPKVYIILLKPNMNIKDHLFKKGKS
>Anole_lizard.locus139.1
CEKCPEDQYPSDSRDKCIAKLIVFLSYNEPLGAILAFISGFHSLITVLILGIFFKFQDTPIVKANNRDLTFVLLFSLLLCFLCSLMFIGQPSKVSCFLRQTFFGIVFSIAVSSILAKTITVILAFLIKNPGSTWQHWTGKRLTNSIVFF
CSLIQVVICGIWLVTSPPFPDMEKPSGHDHIILLCKEGSISMFYVVLGYMGFLAIVSFIVAFFARKLPGVFNETKLITFSMLVFCSVWLSFVSTYISNTGKSMVVVEIFAILGSSSALLGFIFFPKCYIIVLRPEWNRKTNILGKANS
>Anole_lizard.locus204.1
CTKCPEEQHPNKDRDQCVPKVIIFLSYGEPLGVILVSFSIFLFLISGVVLGIFIKFQETPLVKANNRDLSYVLLVSLLLSFLSSFLFIGQPKKATCLLQQTSFNVIFSVAVSSVLAKTTTVVMAFLATRPGKTLRRWLRKGLANTIVIS
CSSLQLLICILWLGLFPPFPDSDMHSQPGMILQQCNEGSVAMFYAALGYMGFLAIVCFVVAFLARNLPGTFNETKMITFSMLVFCSVWVTFVPTYLSTKGKYMVVVQIFSILVSSAALLGCIFIPKCYIIVLRPNLNTKAHVVKRKCG
>Anole_lizard.locus37.1
CIKCPDEDYSNKDHTECLAKSISYLSYKDPLGISIAFFALFFSLITGFVLGIFLKYQETAMVKANNRDLTYMLLVSLLLSFLSSFLFIGQPQMATCLLRQTAFITIFSVAVSSLLAKTITVVVAFMATKPGSRMRKWLRKSLANSIVIS
CSGAQVGICMVWLGVSPPFPDADMHSQPEQIILQCNEGSMVMFYSALGYMGFLAAVCFTVAFLARKLPGAFNEAKLITFSMLVFCSVWISFVPTYLSTKGKYMVAVQLFSMLSSSLGLLGCIFIPKCYIILFRPHLNTKNFLVLKTQD
>Anole_lizard.locus175.1
CQECSEDQYSNKNRDQCISKIIDFLSFGEVLGIVLVFFSLFLCVATVLILVIFLRHRNTPIVKANNRNLTYILLVSILLACLSSFLFIGQPEKVTCLLRQTTFSVIFSIAISSVLAKTITVILVFMATQPGHRMQKWMGQNLANAIVFF
GSIIQFGICIVWMGTSPPYPELVMNSHVRQIILQCNEGSAIMFYSALGFMGILTTTSFIVAFLARKLPGGFNEAKFITFSMLIVCSVWISFVPTYLSTKGKYMVAVQIFSILASNTGLLGFIFLPKCYIIVLKPHLNTKEQLMAKRNK
>Anole_lizard.locus142.1
CVKCPEDQYSNKNRHQCIPKVIVFLSFQEPLGIILVFLSIFLSVITCLVVQTFLKSWNTPIVKANNRSLTCVLLISILLCYLSAILFIGKPGKVTCLLQQMTFGIIFTVAVSCVLAKTIIVVLAFLATQPGNRVRKWLGQKVATTIVIS
CSLIQVCICAAWLSTSPPFPNADMHSESGKIVIHCNEGSLIMFYCFLGYMGLLAIISFTVAFLARKLPDTFNESKLITFSMLVFCSVWILFIPSYMSTMGKYIVAVEVFAILASNTGLLALIFFPKCYIIIIRADMNSKKLLTEKRH-
>Western_clawed_frog.locus141.1
CYKCEDNEWPNQEKTLCIEKQIEFLSYDDPLTLISIISSVILFIIAAVILGIFISFRDTPVVRANNHTLSFLLLVSIKLSFLSVFLFLGRPVDITCMLRQTSFGITFSIAVSCVLAKTLMVSIAFKATKPGSPWRKWVGVKLANGLVFN
LSLIQFLISVIWLVIAPPYVEHNTHSEPRKIIIQCNEGSVVAFYIVLSYMGLLASVSFIVAFLARSLPDSFNEAKYITFSMLLFCSVWITMIPACLSTKGKYMVAVEIFAIISSSCGLLFCIFLPKCYIILFKQEMNSKQYLLGKCNT
>Western_clawed_frog.locus142.1
CLKCEDNKWPNQEKTLCIEKQIEFLSYDDPLTLIFIIISMMLFIIAAVILGIFISFRDTPVVRANNHTLSFILLVSIKLSFLSVFLFLGRPVNITCMLRQTSFGITFSIAVSCVLAKTLMVCIAFKATKPGSRWQKWVGVKLADWLVFI
CSLIQFLISVIWLVIAPPYVEHNTHSEPGKIIIQCNEGSAIGFYIVLSYMGLLASVSFIVAFLARSLPDSFNEAKYITFSMLLFCSVWITMIPAYLSTKGKYMVAVEIFAIISSSCGLLFCIFLPKCYIILFKPQMNSKQYLLGKGYR
>Western_clawed_frog.locus172.1
CLKCEDNKWPNQEKTLCTEKQIEFLSYDDPLTVIFIIISMMLFIIAAVILGIFISFRDTPVVKANNHTLSIVLLVSIKLSFLSVFLFLGCPVDITCMLRQISFGITFSIAVSCVLAKTLMVSIAFKATKPGSPWQKWVGVKLADWLVFI
CSLIQFLISVIWLVISPPYVEHNTHSEPEKIIIQCNEGSVVAFCIVLSYMGLLASVSFIVAFLARSLPDSFNEAKYITFSMLLFCSVWITMIPAYLSTKGKYMVAVEIFAIISSSCGLLFCIFLPKCYIILFKPEMNTKQYLLGKCYR
>Western_clawed_frog.locus242.1
INECCFDTLFNQSFNLLQRSKLSFFH----LTLVIIIITVFLFILAAIILGIFISYRDTPVVKANNHSLSFILLVSIKLSFLSVF----------CSLD--------------------
AQLIAFKATKPGSSWRKWVGEKLANWLLFICKLIQSLIISIIIWS------------
TTYILNLCNEGSVVAFYIVLSYMGLLASVSFIVAFLARSLPDSFNEAKYITFSMLLFCSVWITMIPAYLSTKGKYTVAVEIFAIISLSFGLI-----------------------------
>Western_clawed_frog.locus178.1
CFKCEDTEWPNQEKTLCIERQIEFLSYGDPLTLIFIISSVILFIIAAVILGIFISFRDTPVVRANNHTLSFILLVSIKLSFLSVFLFLGRPVDITCMLRQTSFGITFSIAVSCVLAKTLMVSIAFKATKPGSPWRKWVGVKLANGIVFI
FSLIQSLISVIWLVIAPPFVEQNTHSEPGKIIIQCNEGSVVAFYIVLSYMGLLASVSFIVAFLARSLPDSFNEAKYITFSMLLFCSVWITMIPAYLSTKGKYMVAVEIFAIISSSCGLLFCIFLPKCYIILFKPEMNTKQYLLGKCNT
>Western_clawed_frog.locus129.1
CLKCEDNEWPNQEKTLCIEKQTEFLSYGDPLTQIFIISSVILFIIAAVILGIFISFRDTPVVRANNHTLSFILLVSIKLSFLSVFLFLGRPVDITCMLRQTSFGITFSIAVSCVLAKTLMVSIAFKATKPGNPWRKWVGVKLANGLVFI
FSLIQSLISVIWLVIAPPYVEHNTHSEPGQIIIQCNEGSAIGFYIVLSYMGLLASVSFIVAFLARSLPDSFNEAKYITFSMLLFCSVWITMIPAYLSTKGKYMVAVEIFAIISSSCGLLFCIFLPKCYIILFKPEMNTKQYLLGKCNT
>Western_clawed_frog.locus140.1
CFKCEDNEWPNQEKTLCIEKQIEFLSYGDPLTLISIISSVILFIIAAVILGIFISFRDTPVVRANNHTLSFILLVSIKLSFLSVFLFLGRPVDITCMLRQTSFGITFSIAVSCVLAKTLMVSIAFKATKPGSPWRKWVGVKLANGLVFN
FSLIQFMISVIWLVIAPPYVEHNTHSEPGKIIIQCNEGSAIGFYIVLSYMGLLASVSFIVAFLARSLPDSFNEAKYITFSMLLFCSVWITMIPAYLSTKGKYMVAVEIFAIISSSCGLLFCIFLPKCYIILFKPEMNSKQYLLGKCNT
>Western_clawed_frog.locus138.1
CFKCEDTEWPNQEKTLCIEKQTEFLSYGDPLTLIFIISSVILFIIAAVILGIFISFRDTPVVRANNHTLSFILLVSIKLSFLSVFLFLGRPVDITCMLRQTSFGITFSIAVSCVLAKTLMVSIAFKATKPGSPWRKWVGVKLANGLVFI
CSLIQFLISVIWLVIAPPYVEHNTHSEPGKIIIQCNEGSAIGFYIVLSYMGLLASVSFIVAFLARSLPDSFNEAKYITFSMLLFCSVWITMIPAYLSTKGKYMVAVEIFAIISSSCGLLFCIFLPKCYIILFKQEMNSKQYLLGKCNT
>Western_clawed_frog.locus174.1
CLKCEDTEWPNQEKTLCIEKQIEFLSYGDPLTLISIISSVILFIIAAVILGMFISFRDTPVVRANNHTLSFILLVSIKLSFLSVFLFLGRPVDITCMLRQTSFGITFSIAVSCVLAKTLMVSIAFKATKPGSPWRKWVGVKLANGLVFI
CSLIQFLISAIWLVIAPPYVEQNTHSEPGKIIIQCNEGSAIGFYIVLSYMGLLASVSFIVAFLARSLPDSFNEAKYITFSMLLFCSVWITMIPAYLSTKGKYMVAVEIFAIISSSCGLLFCIFLPKCYIILFKPEMNSKQYLLGKYNT
>Western_clawed_frog.locus176.1
CLKCEDTEWPNQEKTLCIEKQIEFLSYGDPLTLISIISSIILFIIAAVILGIFISFRDTPVVRANNHTLSFILLVSIKLSFLSVFLFLGRPVDITCMLRQTSFGITFSIAVSCVLAKTLMVSIAFKATKPGSPWRKWVGVKLANGHVFI
CSLIQFLISVIWLVIAPPYVEKNFRSEPGKIIIQCNEGSAIAFYIVLSYMGLLASVSFIVAFLARSLPDSFNEAKYITFSMLLFCSVWITMIPAYLSTKGKYMVAVEIFAIISSSCGLLFCIFLPKCYIILFKPEMNSKQYLLGKCNT
>Western_clawed_frog.locus173.1
CLKCEDTEWPNQEKTLCIEKQIEFLSYGDPLTLIFIISSVILFIIAAVILGIFISFRDTPVVRANNHTLSFILLVSIKLSFLSVFLFLGRPVDITCMLRQTSFGITFSITVSCVLAKTLMVSIAFKATKPGSPWRKWVGVKLANGLVFI
CSLIQFLISVIWLVIAPPYVEKNFRSEPGKIIIQCNEGSAIGFYIVLSYMGLLASVSFIVAFLALSLPDSFNEAKYITFSMLLFCSVWITIIPAYLSTKGKYMVAVEIFAIISSSCGLLFCIFLPKCYIILFKPEMNSKQYLLGKCNT
>Western_clawed_frog.locus180.1
CYKCEDTEWPNQEKTLCIEKQIEFLSYGDPLTLIFIISSVILFIIAAVILGIFISFRDTPVVRANNHTLSFILLVSIKLSFLSVFLFLGRPVDITCMLRQTSFGITFSIAVSCVLAKTIMVSIAFKATKPGSPWRKWVGVKLANGLVFI
CSLIQFLIIVFWLVIAPPFVEKNFHSEPGKIIIQCNEGSAIGFYLVLSYMGLLASVSFIVAFLARSLPDSFNEAKYITFSMLLFCSVWITMIPAYLSTKGKYMVAVEIFAIISSSCGLLFCIFLPKCYIILFKPEMNSKQYLLGKCNT
>Western_clawed_frog.locus126.1
CLKCQDTEWPNQEKTLCIERQIEFLSYGDPLTLIFIISSVILFIIAAVILGIFISFRDTPVVRANNHTLSFILLVSIKLSFLSVFLFLGRPVDITCMLRQTSFGITFSIAVSCVLAKTLMVSIAFKATKPGSPWRKWVGVKLANGLVFI
CSLIQSLINVIWLVIAPPYVEQNFHSEPGKIIIQCNEGSAIGFYIVLSYMGLLASVSFIVAFLARSLPDSFNEAKYITFSMLLFCSVWITMIPAYLSTKGKYMVAVEIFAIISSSCGLLFCIFLPKCYIILFKPEINSKQYLLGKCNK
>Western_clawed_frog.locus184.1
CLKCQDNEWPNQEKTLCIERQIEFLSYGDPLTLISIISSVILFIIAAVILGIFISFRDTPVVRANNHTLSFILLVSIKLSFLSVFLFLGRPVDITCMLRQTSFGITFSIAVSCVLAKTLMVSIAFKATKPGSPWRKWVGVKLANGLVFI
CSLIQFLISVIWLVIAPPYVEQNFHSEPGKIIIQCNEGSLVAFWFVLSYMGLLASVSFIVAFLARSLPDSFNEAKYITFSMLLFCSVWITMIPAYLSTKGKYMVAVEIFAIISSSCGLLFCIFLPKCYIILFKPEMNSKQYLLGKCNK
>Western_clawed_frog.locus183.1
CLKCQENEWPNQEKTLCIEKQTEFLSYGDPLTLVIIISSVILFIIAAVILGIFISFRDTPVVRANNHTLSFILLVSIKLSFLSVFLFLGRPVDITCMLRQTSFGITFSIAVSCVLAKTLMVSIAFKATKPGSPWRKWVGVKLANGLVFI
CSLIQFLISVIWLVVAPPYVEKNFHSEPGKIIIQCNEGSAIGFYIVLSYMGLLASVSFIVAFLARSLPDSFNEAKYITFSMLLFCSVWITMIPAYLSTKGKYMVAVEIFAIISSSCGLLFCIFLPKCYIIIFKPEMNTKQCLLGKCNK
>Western_clawed_frog.locus97.1
CIKCQDNEWPNQEKTMCIEKQTEFLSYNDSLTLVFIVLSMVLILITTLILGIFISFRDTPVVRAHNHTLSFILLVSIKLSFLSVFLFLGRPVDITCMLRQTSFGITFSIAVSCVLAKTLMVSIAFKATKPGSPWRKWVGVKLANGLVFI
CSLIQFLISVIWLVIAPPYVEKNFHFEPGKIIIQCNEGSVVAFYIVLSYMGLLASVSFIVAFLARSLPDSFNEAKYITFSMLLFCSVWITMIPAYLSTKGKYMVAVEIFAIISSSCGLLFCIFLPKCYIILFKPEMNSKQYLLGNSKQ
>Western_clawed_frog.locus154.1
---------------------
MEFLSYDDSLTVAFILLSLVFFIIAAVILGIFISFRDTPVVRANNHTLSFILLVSIKLSFLSVFLFLGRPVDITCMLRQTSFGITFSIAVSCVLAKTLMVSIAFKATKPGSPWRKWVGVKLANGLVFICSLIQFLISVFWLVIAPPFVE
QNTHSEPGKIIIQCNEGSAIGFYIVLSYMGLLASVSFIVAFLARSLPDSFNEAKYITFSMLLFCSVWITMIPAYLSTKGKYMVAVEIFAIISSSCGLLFCIFLPKCYIILFKPEMNTKQYLLRNNK-
>Western_clawed_frog.locus153.1
```

```
---------------------
MEFLSYDDSLTVAFILLSLVFFIIAAVILGILISFQDTPVVRANNHTLSFLLLVSIKLSFLSVFLFLGRPVDITCMLRQTSFGITFSIAVSCVLAKTLMVSIAFKATKPGSPWRKWVGVKLANGLVFICSLIQFLINVIWLVIAPPYVE
HNTHSEPGKIIIQCNEGSAIAFYIVLSYMGLLASVSFIVAFLARSLPDSFNEAKYITFSMLLFCSVWITMIPAYLSTKGKYMVAVEIFAIISSSCGLLFCIFLPKCYIIILKPEMNSKQYLLRNNK-
>Western_clawed_frog.locus108.1
CLKCGEYEWPNQEKTHCIQKQNEFLSYDDSLTVAFILLSLVFLIIAAVILGIFISFRDTPVVRANNHTLSFILLVSIKLSFLSVFLFLGRPVDITCMLRQTSFGITFSIAVSCVLAKTLMVSITFKATKPGSPWRKWVGVKLENGLVFI
CSLIQFLISVIWLVIAPPYVEHNTHSEPGKIIIQCNEGSAIGFYIVLSYMGLLASVSFIVAFLARSLPDSFNEAKYITFSMLLFCSVWITMIPAYLSTKGKYMVAVEIFAIISSSCGLLFCIFLPKCYIILLKPEMNTKQYLLGNNKE
>Western_clawed_frog.locus148.1
CLKCGDYEWSNPEKTVCIEKQIQFLSYDDSLTLVFIVHSLVFFIIAAVILGIFISFRDTPVVRANNHTLSFILLVSIKLSFLSVFLFLGRPVDITCMLRQTSFGITFSIAVSCVLAKTIMVCFAFKATKPGSPWRKWVGVKVAYCIVLF
CSIIQILISVIWLTISPPFLELNFLSEPGQIIIQCNEGSAIAFWFVLSYMGLLASVSFIVAFLARSLPDSFNEAKYITFSMLLFCSVWITMIPAYLSTKGKYMVAVEIFAIISSSCGLLFCIFLPKCYIILFKPEMNSKQYLLGNNK-
>Western_clawed_frog.locus149.1
CLKCGDYEWPNPEKTVCIEKQIQFLSYDDSLTLVFIVHSLVFFIIAAVILGIFISFRDTPVVRANNHTLSFILLVSIKLSFLSVFLFLGRPVDITCMLRQTSFGITFSIAVSCVLAKTIMVCVAFKATKPGSPWRKWVGVKVAYCIVLF
CSIIQILISVIWLTISPPFLELNFLSEPGQIIIQCNEGSVVAFWFVLSYMGLLASVSFIVAFLARSLPDSFNEAKYITFSMLLFCSVWITMIPAYLSTKGKYMVAVEIFAIISSSCGLLFCIFLPKCYIILFKPEMNSKQYLLGNNK-
>Western_clawed_frog.locus168.1
CLKCGDYEWPNPEKTVCIEKQIQFLSYDDSLTLVFIVHSLVFFIIAAVILGIFISFRDTPVVRANNHTLSFILLVSIKLSFLSVFLFLGRPVDITCMLQQTSFGITFSIAVSCILAKTIMVCVAFKATKPGSPWRNWVGVKVAYCIVIF
CSIIQILISVIWLTISPPFLELNFLSEPGQIIIQCNEGSVIAFYIVLSYMGLLASVSFIVAFLARSLPDSFNEAKYITFSMLLFCSVWITMIPAYLSTKGKYMVAVEIFAIISSSCGLLFCIFLPKCYIILFKPEMNSKQYLLGNNK-
>Western_clawed_frog.locus150.1
CLKCGDYEWPNPEKTVCIEKQLQFLSYDDSLTLVFIVHSLVFFIIAAVILGIFISFRDTPVVRANNHTLSFILLVSIKLSFLSVFLFLGRPVDITCMLRQTSFGITFSIAVSCVLAKTIMVCFAFKATKPGSPWRKWVGVKVAYCIVLF
CSIIQILISVIWLTISPPFLELNFLSEPGHIIIQCNEGSAIAFYIVLSYMGLLASVSFIVAFLARSLPDSFNEAKYITFSMLLFCSVWITMIPAYLSTKGKYMVAVEIFAIISSSCGLLFCIFLPKCYIILFKPEMNSKQCLLGNNK-
>Western_clawed_frog.locus133.1
CLKCGDYEWPNPEKTVCIEKQLQFLSFDDCLTLIFIISSVILFIIAAVILRIFISFRDTPVVRANNHTLSFILLVSIKLSFLSVFLFLGRPVDITCMLRQTSFGITFSIAVSCVLAKTLMVSIAFKATKPGSPWRKWVGVKVAYCIVLF
CSIIQILISVIWLTISPPFLELNFLFEPGQIIIQCNEGSVIAFYIVLSYMGLLASVSFIVAFLARSLPDSFNEAKYITFSMLLFCSVWITMIPAYLSTKGKYMVAVEIFAIISSSCGLLFCIFLPKCYIILFKPEMNSKRYLLGNNK-
>Western_clawed_frog.locus130.1
CLKCGDYKWPNPEKTVCIEKQLQFLSFDDPLTLIFIISSVILFVIAAVILGIFISFRDTPVVRANNHTLSFILLVSIKLSFLSVFLFLGRPVDITCMLRQTSFGITFSIAVSCVLAKTLMVCFAFKATKPGSPWRKWVGVKVAYCIVLS
CSIIQILISVIWLTISPPFLELNFLFEPGQIIIQCNEGSVVAFYIVLSYMGLLASVSFIVAFLARSLPDSFNEAKYITFSMLLFCSVWITMIPAYLSTKGKYMVAVEIFAIISSSCGLLFCIFLPKCYIILFKPEMNSKQYLLGNNR-
>Western_clawed_frog.locus132.1
CLKCGDYEWPNPEKTVCIEKQLQFLSFEDCLTLIFIVLSLVFFIIAAVILGIFISFRDTPVVRANNHTLSFILLVSIKLSFLSVFLFLGRPVDITCMLRQTSFGITFSIAVSCVLAKTLMVCFAFKATKPGSPWRKWVGVKVAYCIVLS
CSIIQILISVIWLTISPPFLELNFLSEPGQIIIQCNEGSAIGFYIVLSYMGLLASVSFIVAFLARSLPDSFNEAKYITFSMLLFCSVWITMIPAYLSTKGKYMVAVEIFAIISSSCGLLFCIFLPKCYIILFKPEMNSKQYLLGNNK-
>Western_clawed_frog.locus131.1
CLKCGDYEWPNPEKTLCIEKQLQFLSYDDSLTLVFIVLSLVFFIIAAVILGIFISFRDTPVVRANNHTLSFILLVSIKLSFLSVFLFLGHPVDITCMLRQTSFGITFSIAVSCVLAKTLMVSIAFKATKPGSPWRKWVGVKVAYCIVLS
CSIIQILISVIWLTISPPFLELNFLFEPGQIIIQCNEGSAIGFYIVLSYMGLLASVSFIVAFLARSLPDSFNEAKYITFSMLLFCSVWITMIPAYLSTKGKYMVAVEIFAIISSSCGLLFCIFLPKCYIILFKPEMNSKQYLLGNNR-
>Western_clawed_frog.locus68.1
CIKCHIYEWPNKEKTMCIEKQTEFLSYDDSLTLVFIVLSLVSVLLATVILGIFISFRDTPVVRANNRNLSFILLVSIKLSFLSVFLFLGHPVDITCVLQKTTYGITFSIAMSSVLAKTILVWIAFKASKPGSPWRKLLKGNLGHYIVVI
CSFIQTLICFIWLALSPPFVELNILSDPGKIIIQCNEGSVVAFYIVLSYMGLLASVSFIVAFLARSLPDSFNEAKYITFSMLLFCSVWITMIPAYLSTKGKYMVAVEIFAIISSSCGLLFCIFLPKCYIIMLKPEMNTKQYLLGYKK-
>Western_clawed_frog.locus67.1
CIKCHIYEWPNEEKTMCIEKQTEFLSYDDSLTLVFIVLSFVSILLVTVILGIFISFRDTPVVRANNRNLSFILLVSIKLSFLSVFLFLGRPVDITCMLRKTSYGITFSIAMSSVLAKTILVWIAFKASKPGSPWRKLLKGNLGHYIVVI
CSFIQTLICFIWLALSPPFVELNILSDPGKIIIQCNEGSVVAFYIVLSYMGLLASVSFIVAFLARSLPDSFNEAKYITFSMLLFCSVWITMIPAYLSTKGKYMVAVEIFAIISSSCGLLFCIFLPKCYIIMLKPELNTKQYLLGNNK-
>Western_clawed_frog.locus202.1
CLKCHIYEWPNQEKTLCIKKQTEFLSYEDCLSLAFIVLSVVFIFIAAVILGIFILFQDTPVVKANNRNLSFILLVSIKLSFLCVFLFLGRPVDITCMLRQTSFGITFSLAMSSVLGKTIMVCVAFKATKPGSSWRKWLGVKLSYFTVVI
CSFIQFLISIVWLVISPPFVELSSLSDPGKIFIQCNEGSVIPFYIVLSYMGFLASVSFIVAFLARSLPDSFNEAKYITFSMLLFCSVWITMIPAYLSTKGKNTVCVEIFAILTSSAGLLACIFLPKCYIILLKPEMNTKQYLMGTNKQ
>Western_clawed_frog.locus98.1
CLKCNDFDWPDQKKIKCIKKKTEFLSYEDPLTLAIIVLSVVFILKADIILVILISFRDTPLVKANNRILSFILLVSIKMSILSVFLFLGQPINMTCILQKASFGITFSIAMSSVLAKTVMVCIAFKASKPDSPWRNWMGAKVPYCMVLV
CSLIQILITVTWLASSPPFVEHDIHSEPGKRIIKCNEGSAIAFYIVLSYMGLLASVSFIVAFLARSLPDSFNEAKYITFSMLLFCSVWITMIPAYLSTKGKYMVAVEIFAIISSSCGLLFCIFLPKCYIILFKPEMNSKQYLLGNNHK
>Western_clawed_frog.locus279.1
CLKCQDYEWPNQEKTMCIEKQTEFLSYDDPLTLAFIVLSVVFILITTVILGIFISFRDTPVVKANNRNLSFILLVSIKLSVLSVFLFLGRPMDITCMLRQTSFGITFSIAMSSVLSKTIMVCMAFKASKPDSPWRKYVSAKVAYWIVFV
CSVIQILISVIWLASSPPFVEHNIHSEPGKILILCNEGSVVAFYIVLSYMGLLASVSFIVAFLARSLPDSFNEAKYITFSMLLFCSVWITMIPAYLSTKGKYMVAVEIFAIISSSCGLLFCIFLPKCYIILFKPEMNTKQYLLGSSK-
>Western_clawed_frog.locus283.1
CLTCQDNKWPNHEKTMCIEKQVEFISFTDYFAIFFTINSLILFTVGAVILGIFISFRDTPVVKANNRNLSFILIVSIKLSILSVFLFLGRPTDIICTLRQSSFGITFSIAVSCVLAKTIMVYIVFKATKPGRSWSKWVGVKLAHCIVLV
CSVIQILITSLWLSISPPFVEYNILSEPGKIIIQCNEGSVVAFYIVLSYMGLLASMSFIVAFLARSLPDSFNEAKYITFSMLLFCSVWITMIPAYLSTKGKPMVAVEIFAIITSSCGLLFCIFLPKCYIILLKPEWNTKHCLLGNPNK
>Western_clawed_frog.locus265.1
CVTCLDYEWPNNGKTRCIEKPTDFLSYRDSLTLVFNVITLILFVIALSILGTFILYRDTPVVKANNRSLSFILLVSIKLSFLSVFLFLGRPVDITCMLRQTSFGITFSIAISCVLAKTIMVSVAFKATKPDSSWRKLAGAKLANTIVLV
SSIIQVVISVIWLAISPPFAEQNIHSEPGKIIIQCNEGSVVAFYIVLSYMGLLASVSFIVAFLARSLPDSFNEAKYITFSMLLFCSVWITMIPAYLSTKGKYTVAVEIFAIISSSCGLLFCIFLPKCYIILLKPEMNTKLNLLGNKKK
>Western_clawed_frog.locus266.1
CVTCLDYEWPNNGKTRCIEKPTDFLSYRDSLTLVFNVITLILFVIALSILGTFISYRDTPVVKANNRSLSYILLVSIKLSFLSVFLFLGRPVDITCMLRQTSFGITFSIAISCVLAKTIMVSVAFKATKPDSSWRKLAGAKLANTIVLV
SSMIQVVISVIWLAISPPFAEQNIHSEPGKIIIQCNEGSVVAFYIVLSYMGLLASVSFIVAFLARSLPDSFNEAKYITFSMLLFCSVWITMIPAYLSTKGKYTVAVEIFAIISSSCGLLFCIFLPKCYIILLKPEMNTKLNLLGNKKK
>Western_clawed_frog.locus268.1
CVTCLDYEWPDNGKTRCIEKPTDFLSYRDSLTLVFNVITLIFFVIALSILGTFILYRDTPVVKANNRSLSFILLVSIKLSFLSVFLFLGRPVDITCMLRQTSFGITFSIAISCVLAKTIMVSVAFKATKPGSSWRKLVGARLANTIVLV
SSIIQVVISVIWLAISPPFAEHNIHSEPGKIIIQCNEGSVVAFYIVLSYMGLLASVSFIVAFLARSLPDSFNEAKYITFSMLLFCSVWITMIPAYLSTKGKYTVAVEIFAIISSSCGLLFCIFFPKCYIILLKPEMNTKLNLLGNKKK
>Western_clawed_frog.locus192.1
CQPCPEDKWPNQKKDQCLPRSSEFLSYQSDWKTLLSIVSILLSGVTAIIVGIFVAFRHSPIVKANNQRLSFLLLGSIMMSFLCVFLFLGRPLHVTCMLRQTCMGIIFSVVVSSVLAKTIMVCIVFKASKPGNYWKKCVGAKIPNGVVVA
CSLLQVSISIAWLSISPPFMELN-RSFPGKILIQCNEGSPVAFYAVLGYLGLLAAVSFIVAFLARKLPDSFNEAKYITFSMLVFCSVWVSFIPAHLSVTGKNTVVVEIFAIMASSFGVLASIFFPKCYIILIKPEQNTKRNLLGRSPN
>Western_clawed_frog.locus966.1
CLKCPEDKWPDSRKEECLPKLIQFLSYEETLGSALACISVLFCLLTYSVFCLFIIKRKTPIVKANNRDLSYLLLISLMFGFMCSLAFIGRPNRIMCMIRQVMFAVIFSLCVSTILAKTITVVMIFSATNPDSKLKKLVGLRIPIYIVPV
CTMVQVILCIVWLTTDAPFAEFNMEAEIGIIVIECNEGSRVLFASVLGYMGLLASVSLFVAFLARKLPDTFNETKFIAFSMLVFASVWVTFIPAYLSTKGKQTVAVEIFAILSSSAGCLFCIFSPKCYTILLHPEMNSKENITGRNTR
>Western_clawed_frog.locus963.1
CLKCPEDKWPDSRKEECLPKPIQFLSYEETLGSALACISVLFCLLTFSVFCLFIIKRKTPIVKANNRDLSYLLLISLMFGFMCSLAFIGRPNRIMCMIRQVMFAVIFSLCVSTILAKTITVIMIFSATNPDSKLKKLVGLRIPIYIVPV
CTMVQIILCIVWLTTDAPFAEFNMAAEIGIIVIECNEGSRVLFASVLGYMGLLASVSLFVAFLARKLPDTFNETKFITFSMLVFASVWVTFIPAYLSTKGKQTVAVEIFAILSSSAGCLFCIFSPKCYTILLHPEMNSKENITGRNTR
>Western_clawed_frog.locus967.1
CLKCPEDKWPDSRKEECLPKSIKFLSYEETLGSILACISVLFCLLTFSVFCLFIIKRKTPIVKANNRDLSYLLLISLMFGFMCSLAFIGRPNRIMCMIRQVMFAVIFSLCVSTILAKTITVVMIFSATNPDSKLKKLVGLRIPIYIVPV
CTMVQIILCIVWLTTESPFVEFNMAAEIGIIVIECNEGSRVLFACVLGYMGLLASVSLFVAFLARKLPDTFNETKFITFSMLVFASVWVTFIPAYLSTKGKQTVAVEIFAILSSSAGCLFCIFSPKCYIILLHPEMNSKENITGRNTR
>Western_clawed_frog.locus957.1
CLKCPEDKWPDSRKEECLPKPIQFLSYEETLGSTLACISVLFCLLTFSAFCLFIIKRKTPIVKANNRDLSYLLLISLMFGFMCSLAFIGRPNRIMCMIRQVMFAVIFSLCVSAILAKTITVVMIFSATNPDSKLKKLVGLRIPIYIVPV
CTMIQIILCIVWLTTDAPFAEFNMAAEIGIIVIECNEGSRVLFACVLGYMGLLASVSLFVAFLARKLPDTFNETKFITFSMLVFASVWVTFIPAYLSTKGKQTVAVEIFAILSSSAGCLFCIFSPKCYTILLHPEMNSKQYITGRNAR
>Western_clawed_frog.locus960.1
CLKCPEDKWPDSRKEECLSKLIQFLSYEETLGSALACISVLFCLLTFSVFCLFIIKRKTPIVKANNRELSYLLLISLMFGFMCSLAFIGRPNRIMCMIRQVMFAVIFSLCVSTILAKTITVVMIFSATNPDSKLKKLVGLRIPIYIVPV
CTMVQIILCIVWLTTDAPFAEFNMAAEIGIIVIECNEGSRVLFACVLGYMGLLASVSLFVAFLARKLPDTFNETKFITFSMLVFASVWVTFIPAYLSTKGKQTVAVEIFAILSSSAGCLFCIFSPKCYTILLHPEMNTRQYITGRNAR
>Western_clawed_frog.locus971.1
CLKCPEDKWPDSRKEECLPKLIQFLSYEEALGSALACISVLFCLLTFSVFCLFIIKRNTPIVKANNRDLSYLLLISLMFGFMCSLAFIGRPNRIMCMIRQVMFAVIFSLCVSTILAKTITVIMIFSATNPDSKLKKLVGLRIPIYIVPV
CTMIQVILCIIWLTTDAPFAEFNMAAEIGIIVIDCNEGSRELFACVLGYMGLLASISLFVAFMARKLPDTFNETKFITFSMLVFASVWVTFIPAYLSTKGKQTVAVEIFAILSSSAGCLFCIFSPKCYTILLHPEMNSRQYITGRNAR
>Western_clawed_frog.locus953.1
CLKCPEDKWPDSRKEECLPKLIQFLSYEETLGSALACISILFCLLTFSVFCLFIIKRNTPIVKANNRDLSYLLLISLMFGFMCSLAFIGRPNRIMCMIRQVMFAVIFSLCVSTILAKTITVVMIFSATNPNSKLKKLVGLRIPIYIVPG
CTMVQIILCIVWLTTDAPFAEFNMAAEIGIIVIECNEGSKVLFASVLGFMGLLASVSLFVAFMARKLPDTFNETKFITFSMLVFASVWVTFIPAYLSTKGKQTVAVEIFAILSSSAGCLFCIFSPKCYTILLHPEMNSRQYITGRNTR
>Western_clawed_frog.locus969.1
CLKCPEDKWPDSRKEECLPKLIQFLSYEETLGSALACISVLFCLLTFSVFCLFIIKRKTPIVKANNRDLSYLLLISLMFGFMCSLAFIGRPNRIMCMIRQVMFAVIFSLCVSTILAKTITVVMIFSATNPDSKLKKLVGLRIPIYIVPV
CTMVQITLCIVWLTTDAPFAEYNMAAEIGIIVIECNEGSRVLFACVLGYMGLLASISLFVAFMARKLPDTFNETKFITFSMLVFASVWVTFIPAYLSTKGKQTVAVEIFAILSSSAGCLFCIFSPKCYTILLHPEMNSRQYITGRNAR
>Western_clawed_frog.locus972.1
CLKCPEDKWPDSRKEECLPKLIQFLSYDETLGSALACISVLFCLLTFSVFCLFIIKRETPIVKANNRDLSYLLLISLMFGFMCPLAFIGRPNRIMCMIRQVMFAVIFSLCVSTILAKTITVIMIFSATNPDSKLKKLVVLRIPIYIVPV
CTMVQIILCIVWLTTDAPFAEYNMAAEIGIIVIECNEGSRVLFACVLGYMGLLASVSLFVAFLARKLPDTFNETKFITFSMLVFATVWVTFIPAYLSTKGKQIVAVEIFAILSSSTGCLFCIFSPKCYTILLHPEMNSRQYITGRNAR
>Western_clawed_frog.locus952.1
CLKCPEDKWPDSRKEECLPKPIQFLSYEETLGSVLACISVLFCLLTFSVFCLFIIKRNTPIVKANNRDLSYLLLISLMFGFMCSLAFIGRPNRIMCMIRQVMFAVIFSLCVSTILAKTITVVMIFRATNPDSKLKKLVGLRIPIYIVPV
CTMVQIILCIVWLTTDAPFAEFNMAAEIGIIVIECNEGSRVLFVSVLGYMGLLASVSLFVAFLARKLPDTFNETKFITFSMLVFASVWVTFIPAYLSTKGKQTVAVEIFAILSSSAGCLFCIFSPKCYIILLHPEMNSRQYITGRNAR
>Western_clawed_frog.locus976.1
CLKCPGDEWPDSKKEKCLPKSIQYLSYEETLGSVLACISVLFCLLTFSVFCLFIIKRKTPIVKANNRDLSYLLLISLMFGFMCSLAFIGRPNRIMCMIRQVMFAVIFSLCVSTILAKTITVVMIFSATNPDSKLKKLVGMRLPDYIVPV
CTMVQIILCIVWLTTDAPFAEFNMAAEIGIIVIECNEGSRVLFASVLGYMGLLASISLLVAFLARKLPDTFNETKFITFSMLVFASVWVTFIPAYLSTKGKQTVAVEIFAILSSSAGCLFCIFFPKCYIILLHPEMNSKQYITGRNAR
>Western_clawed_frog.locus973.1
CLKCPEDKWPDSRKEECLLKLIQFLSYEETLGSTLACISVLFCLLTFSVFCLFIIKRKTPIVKANNRDLSYLLLISLMFGFMCPLAFIGRPNRIMCMIRQVMFAVIFSLCVSTILAKTITVVMIFSATNPDSKLKKLVGLRIPIYIVPV
CTMVQIILCIVWLNTDAPFAEFNMAAEIGIIVIECNEGSRVLFASVLGYMGLLASVSLFVAFLARKLPDTFNETKFIAFSMLVFASVWVTFIPAYLSTKGKQTVAVEIFAILSSSAGCLFCIFSPKCYIILLHPEMNSRQYITGRNAR
>Western_clawed_frog.locus975.1
CLKCPEDKWPDSRKEECLPKLIQFLSYEETLGSALACISVLFCLLTFSVFCLFIIKRNTPIVKANNRDLSYLLLISLMFGFMCSLAFIGRPNRIMCMIRQVMFAVIFSLCVSTILAKTITVVMIFSATNPDSKLKKLVVLRIPIYIVPV
CTMVQIILCIVWLTTDAPFPEFNMAAEIGIIVIECNEGSRVLFACVLGYMGLLASISLFVAFMARKLPDTFNETKFITFSMLVFASVWVTFIPAYLSTKGKQTVAVEIFAILSSSAGCLFCIFSPKCYIILLHPEMNNRQYITGRNAR
>Western_clawed_frog.locus964.1
CLKCPEDKWPDSGKEECLPKLIQFLSYEETLGSALACISVLFCLLTFSVFCLFIIKRKTPIVKANNRDLSYLLLISLMFGFMCSLAFIGRPNRIMCMIRQVMFAVIFSLCVSTILAKTITVVMIFSATNPNSKLKKLVVLRIPIYIVPV
CTMVQIILCIVWLTTDAPFPEFNMAAEIGIIVIECNEGSRVLFACVLGYMGLLASVSLFVAFLARMLPDTFNETKFITFSMLVFASVWVTFIPAYLSTKGKQTVAVEIFAILSSSAGCLFCIFSPKCYIILLHPEMNSKQYITGRNTR
>Western_clawed_frog.locus950.1
CLKCPEDKWPDSRKEECLPKPIKFLSYEETLGSILACISVLFCLVTFSVFCLFIIKRKTPIVKANNRDLSYLLLISLMFGFMCSLAFIGRPNRIMCMIRQVMFAVIFSLCVSTILAKTITVVMIFSATNPDSKLKKLVGLRIPIYIVPV
CTMVQIILCIVWLTTDAPFAEYNMAAEIGIIVIECNEGSRVLFASVLGYMGLLASVSLFVAFLARKLPDTFNETKFITFSMLVFASVWVTFIPAYLSTKGKQTVAVEIFAILSSSAGCLFCIFSPKCYIILLHPEMNSRQYITGRNTR
>Western_clawed_frog.locus962.1
CLKCPEDKWPDSRKEECLPKPIKFLSYEETLGSTLACISVLFCLLTLSVFCLFIIKRKTPIVKANNRDLSYLLLISLMFGFMCSLAFIGRPNQIMCMIRQVMFAVIFSLCVSTILAKTITVVMIFSATNPDSKLKKLVGLRIPIYIVPV
CTMVQVILCIVWLTTDAPFAEYNMAAEIGIIVIECNEGSRVLFASVLGYMGLLASISLFVAFLARKLPDTFNETKFITFSMLVFASVWVTFIPAYLSTKGKQTVAVEIFAILSSSAGCLLCIFSPKCYSILLHPEMNSRQYITGRNAR
>Western_clawed_frog.locus951.1
CLKCPEDKWPDSRKEECLPKPIKFLSYDETLGSTLACISVLFCLLTFSVFCLFIIKRKTPIVKANNRDLSYLLLISLMFGFMCPLAFIARPNRIMCMIRQVMFAVIFSLCVSTILAKTITVIMIFSATNPDSKLKKLVGLRIPIYIVPV
CTMVQVILCIVWLTTEAPFAEFNMAAEIGIIVIECNEGSRVLFACVLGYMGLLASISLFVAFLARKLPDTFNETKFITFSMLVFASVWVTFIPAYLSTKGKQTVAVEIFAILSSSAGCLFCIFSPKCYLILLHPEMNSRQYITGRNTR
>Western_clawed_frog.locus961.1
CLKCPEDKWPDSRKEECLPKPIKFLSYEETLGSALACISVLFCLLTFSVFCLFIIKRMTPIVKANNRDLSYLLLISLMFGFMCSLAFIGRPNRIMCMIRQVMFAVIFSLCVSTILAKTITVIMIFSATNPDSKLKKLVGLRIPIYIVPV
CTMVQVIQCIVWLTTDAPFAEYNMAAEIGIIVIECNEGSRVLFACVLGYMGLLASVSLFVAFMARKLPDTFNETKFITFSMLVFASVWVTFIPAYLSTKGKQTVAVEIFAILSSSAGCLFCIFSPKCYIIFLHPEMNTKEYITGRNTR
>Western_clawed_frog.locus968.1
CLKCPEDKWPDSRKEECLSKLIQFLSYEETLGSALACISVLFCLLTFSVFCLFIIKRKTPIVKANNRDLSYLLLISLMFGFMCSLAFIGRPNRIMCMIRQVMFAVIFSLCVSTILAKTITVIMIFSATNPDSKLKKLVGLRIPIYIVPG
CTMVQVILCIVWLTTEAPFAEYNMAAEIGIIVIECNEGSRVLFACVLGYMGLLASVSLFVAFLARKLPDTFNETKFITFSMLVFASVWVTFIPAYLSTKGKQTVAVETFAILSSSAGCLFCIFSPKCYIILLHPEMNSRQYITGRNTR
>Western_clawed_frog.locus959.1
```

```
CLKCPEDKWPDSRKEECQPKLIQFLSYEETLGSILACISVLFCLLTFSVFCLFIIKRKTPIVKANNRDLSYLLLISLMFGFMCSLAFIGRPNRIMCMIRQVMFAVIFSLCVSTILAKTITVIMIFSATNPDSKLKKLVGLRIPIYIVPV
CTMVQVILCIVWLTTDAPFAEFNMAAEIGIIVIECNEGSRVLFAFVLGYMGLLASISLLVAFLARKLPDTFNETKFITFSMLVFASVWVTFIPAYLSTKGKQTVAVEIFAILSSSAGCLFCIFSPKCYTILLHPEMNSRQYITGRNAR
>Western_clawed_frog.locus974.1
CLKCPEDKWPDSRKEECLPKLIQFLSYEETLGSALACISVLFCLLTFSVFCLFIIKRKTPIVKANNRDLSYLLLISLMFGFMCSLAFIGRPNRIMCMIRQVMFAVIFSLCVSTILAKTITVIMIFSATNPDSKLKKLVGLRIPIYIVPV
CTMVQVILCIVWLTTDAPFSEFNMAAEIGIIVIECNEGSRVLFACVLGYMGLLASVSLFVAFLARKLPDTFNETKFITFSMLVFASVWVTFIPAYFSTKGKQTVAVEIFAILSSNAGCFFCIFSPKCYIILLHPKMNSRQYITGRNAR
>Western_clawed_frog.locus955.1
CLKCPEDKWPDSRKEECLPKPIKFLSYEEALGSALACISVLFCLLTFSVFCLFIIKRKTPIVKANNRDLSYLLLISLMVGFLCPLAFIGRPNQIMCMIRQVMFAVIFSLCVSAILAKTITVVMIFSATNPNSKMNKLVGFRIPVYIVPV
CTVIQIILCIVWLAQAAPFAEFNMAAEIGIIVIECNEGSRVLFASVLGYMGLLASVSLFVAFMARKLPDTFNETKFITFSMLVFASVWVTFIPAYLSTKGKQTVAVEIFAIVSSSAGLLVCVFSPKCYIILLHPEMNSKEYITRRNPR
>Western_clawed_frog.locus948.1
CLKCPEDKWPDSRKEECLPKLIQFLSYEETLGSALACISVLFCLLTFLVFCLFIIKQKTPIVKANNRELSYLLLISLIFGFLCPLAFIGRPNRIMCMIRQVMFAVIFSLCVSTILAKTITVIMIFSATNPNSKMNTLVGFRIPVCIVPV
CTVIQIILCIVWLAQAAPFAEFNMAAEIGIIVIECNEGSRVLFASVLGYMGLLASVSLFVAFMARKLPDTFNETKFITFSMLVFASVWVTFIPAYLSTKGKQTVAVEIFAILSSSAGLLVCVFFPKCYIILLHPEMNSREYITRRNAR
>Western_clawed_frog.locus958.1
CLKCPADKWPDSRKEECLPKLIQFLSYEETLGSALACISVLFCLLTFSVFCLFIIKRNTPIVKANNRDLSYLLLISLMFGFMCSLAFIGRPNRIMCMIRQVMFAVIFSLCVSTILAKTITVIMIFSATNPNSKLKTLVGFRIPIYIVPV
CAMVQIILCIVWLAQAAPFAESNMAAEIGIIVIECNEGSRVLFACVLGYMGLLASISLFVAFLARKLPDTFNETKFITFSMLVFASVWVTFIPAYLSTMGKQTVAVEIFAILSSSAGLLVCIFFPKCYIILLHPEMNSREYITRRNGK
>Western_clawed_frog.locus979.1
CIKCPEDKWPDSTKEKCLPKPIQFLSYDEILGSSLACISVLFCLFTFSVFCLFVVKRKTPIVKANNRELSYLLLISLMFGFLCSLAFIGRPNLITCMIRQVMFAVIFSVCISVILAKTITVVMIFSATNPDSKLKKLVGLRIPIYIVPV
CTMVQIILCIIWLTSAAPFAEFNMAAELGKIVIECNEGSKVLFSCVLGYMGVLAAISLLVAFLARKLPDTFNETKFITFSMLVFASVWVTFIPAYLSTKGKQTVAVEIFAILSSSAGLLVCIFFPKCYIILLHPEMNSKQYITGRNVR
>Western_clawed_frog.locus1022.1
CLKCPEDQWPDRMKERCLPKPIQFLSYDEPLGSSLACISVLFCLITFSAFCLFIAKRKTPIVKANNRELSYLLLISLMFGFLCSLAFIGRPNLKTCMIRQVLFAVIFSFCISVILAKTVTVIMIFSATNPDSKLKRLVAMRIPVYIVPV
CTMVQIILCIVWLARAAPFPEFNMAAEIGKIVTECNEGSRVLFACVLGYMGLLAAISLLVACLARKLPDTFNETKFITFSMLVFASVWLTFIPAYLSTKGKQTVAVEIFAILSSMAGLLVCIFFPKCYIILLHPEMNTKQYITGRNTR
>Western_clawed_frog.locus1019.1
CLKCPEDQWPDSMKERCLPKPIQFLSYDEPLGSSLACISVLFCLITFSAFCLFIAKRKTPIVKANNRELSYLLLISLMFGFLCSLAFIGRPNLKTCMIRQVLFAVIFSFCISVILAKTVTVIMIFSATNPDSKLKRLVAMRIPVYIVPV
CTMVQIILCIVWLARAAPFPEFNMAAEIGIIVTECNEGSRVLFACVLGYMGLLAAISLLVACLARKLPDTFNETKFITFSMLVFASVWLTFIPAYLSTKGKQTVAVEIFAILSSSAGLLVCIFIPKCYIILLHPEMNTKQYITGRNTR
>Western_clawed_frog.locus1017.1
------------
MKEKCLPKPIQFLSYDEPLGSSLACISVFFCLITFSAFCLFIAKRKTPIVKANNRELSYLLLISLMFGFLCSLAFIGRPNLKTCMIRQVLFAVIFSFCISVILAKTVTVIMIFSATNPDSKLKRLVAMRIPVYIVPVCTMVQIILCIVW
LARAAPFAEFNMAAEIGIIVIECNEGSRVLFACVLGYMGLLAAISLLVACLARKLPDTFNETKFITFSMLVFASVWLTFIPAYLSTKGKQTVAVEIFAILSSMAGLLVCIFIPKCYIILLHPEMNSKQYITGRNTR
>Western_clawed_frog.locus1021.1
CLKCPEDQWPDSMKERCLPKIIQFLSYDEPLGSSLACISVLFCLITFSAFCLFIAKRKTPIVKANNRELSYLLLISLMFGFLCSLAFIGRPNLKSCMIRQVLFAVIFSFCISVILAKTVTVIMIFSATNPDSKLKRLVAMRIPVYIVPV
CTMVQIILCIVWLARAAPFAEFNMAAEIGIIVIECNEGSRVLFACVLGYMGLLAAVSLLVACLARKLPDTFNETKFITFSMLVFASVWLTFIPAYLSTKGKQTVAVEIFAILSSSAGLLVCIFIPKCYIILLHPEMNSKQYITGRNTR
>Western_clawed_frog.locus1023.1
CLKCPEDQWPDGMKERCLPKIIQFLSYDEPLGSSLACISVLFCLITFSAFCLFITKRKTPIVKANNRELSYLLLISLMFGFLCSLAFIGRPNLKTCMIRQVLFAVIFSFCISVILAKTVTVIMIFSATNPDSKLKRLVGLRIPVYIVPV
CTMVQIILCIVWLARAAPFAEFNMAAEIGIIVIECNEGSRVLFACVLGYMGLLAAISLLVAFLARKLPDTFNETKFITFSMLVFASVWLTFIPAYLSTKGKQTVAVEIFAILSSSAGLLVCIFFPKCYIILLHPEMNTKQYITGRNTR
>Western_clawed_frog.locus977.1
-----------------------------
MLGTSLACISVLLCLLTFSVFCLFIAKHKTPIVKANNRELSYLLLISLMLCFLCSLVLIGRPNMITCMIRQVVFGVIFSVCLSVILAKTMTVILVFSATNPDSKIRRLVGLRIPIYIVPSCTVIQIVLCIVWLAIDPPFSEFNMEAEIG
TIVIECNEGSNVLFACVLGYMGLLASISLLFAFLARKLPDTFNEAKFITFSMLVFASVWVTFIPAYLSTKGKHMVAVEIFAIISSSAGLLVCIFFPKCYIILLHPEMNSKQFIKGANNK
>Western_clawed_frog.locus978.1
-----------------------------
MLGTSLACISVLLCLLTFSVFCLFIAKHKTPIVKANNRELSYLLLISLMLCFLCSLVFIGRPNMITCMIRQVVFGVIFSVCLSVILAKTMTVILVFSATNPDSKIRRLVGLRIPIYIVPFCAMVQIALCIAWLAIAAPFAEFNMEAEIG
TIVIQCNEGSNAFFTCVLGYMGLLASISLLVAFLARKLPDTFNEAKFITFSMLVFASVWVTFIPAYLSTKGKNMVAVEIFAIISSSAGLLVCIFFPKCYIILLHPEMNSKQFITGKNNR
>Caecillan.locus10029.1
CLKCPEDQWSNENLNKCIPKVIEFLAFEEPLGITLASLSSIFSLLTMSVLCIFIKYRDTAIVKANNRNVSYLLLLALLLCFLCSFLFIGHPWKITCLIRQAVFGIVFTLSLSCVLAKTITVVIAFNATKPNSSLKRWIGSYIPNAVILI
CSGLQVLICISWVTIYPSSPEPNRNVSKDRIIMECKEGSIVFFSVMLSFMGLLATITLVVAFLARKLPDGFNEAKCITFSMLVFTSVWISFILAYASTKGKYMVAVELFAILASSFGLLCCVFFPKCHIIILRPKMNSRDNLTGKKK-
>Western_clawed_frog.locus531.1
CMRCPEDQWSDETRIHCIPKVIEFLSYEESLGMALSSVSTVFSLLTVFVLCIFIRYRDTPLVKANNRSLSYLLLVALLLCFLCSFLFIGHPVLITCLIRQAIFGIIFSLCVSCIFAKTITVVIAFSATKPNSPLRKWVGSSIPNYMILG
GSSIQTVICALWVIIYPSSPELNTKASKGKITVECKEASPAFLYVMLGFLGFLALLTLTVAFLARKLPDGFNETKLIAFSMLVFTSVWITFIPAYVSTVGKYTVAVEIFAIVSSSFGLLVCIFAPKCHIIILRPHQNTRDFLTGKTGK
>Caecillan.locus36917.1
CRKCPEDQWPNESRDKCIPKIIEFLSYQEPLGVALAVVALILSVTTALILAIFFRFQNTPVVKANNRGLSYLLLCSLILCFLCSLTFIGLPAKLTCMIRQPAFGLTFTISVSCILAKTVTVVIAFKAIDPHNHLRRWIGQSISYFTAVL
CSLIQLLICLYWISNHPSLPENSSRSGSGKMIIECNDGSTILFYCMLGYVGFLAIISFIVAFLARTLPDSFNEAKFITFSMLVFVSVWLSFIPAYLSTHGKYMVAVEIFAILSSAAGLLFLIFFPKCYIILLRPEMNTKEHLVGKTIT
>Western_clawed_frog.locus783.1
---------------
MCVPKVIEFLSYQEPLGIFLAITVIIFFLITLAILFIFMKYRGTPIIRATNRELSFILLVSLTLCFLCCLIFIGSPSPMTCPLRQTLFMVVFSISISSVLAKTIMVIVAFKATKMHSPLRKWLGPKIPRTVVALCTTAQVGICGAWLLL
SPPFPQVNAEIEKHKLIRECHEGQSLFFYVTLGFMGFLAMVSFFAAFLARNLPGSYNEAKLITFSMLVFCSVWVSFIPAHLSTKGKYTVSVQIFAILASSAGLLACIFLPKCYIVLLRPDRNSRGRITKLVT-
>Western_clawed_frog.locus785.1
---------------
MCVPKVIEFLSYQEPLGIFLAVTVIIFFLITLAILFIFMKYRGTPIIKATNRELSFILLVSLTLCFLCCLIFIGSPSPMTCPLRQTLFMVVFSISISSVLAKTIMVIVAFKATKMHSPLRKWLGPKIPRTVVALCTTAQVGICGAWLLL
SPPFPQVNAEIEKHKLIRECHEGQSLFFYVTLGFMGFLAMVSFFAAFLARNLPGSYNEAKLITFSMLVFCSVWVSFIPAHLSTKGKYTVSVQIFAILASSAGLLACIFLPKCYIVLLRPDRNSRGRITKLVT-
>Western_clawed_frog.locus875.1
---------------------------------------
MTFSLMTVAVLCVFLRFRDTVIVKANNRSLSYVLLLSLTFCFLSSLTFIGSPTAVTCSLRQSIFGVIFSLCVSCILAKTIIVVMAFKATRPDSRLRQWVSSKTTVLVVSLSTMVQVILIALWLIISPPFLEHTVQSITGKVLLECNEGS
SPMFYSVLGYLGFLAFLSFVVAFLARSLPDNFNEAKYITFSMLIFLSVWVSFIPAHLSTKGKYIVATEVFAIIVSGSGLLGCIFCPKCFIILLRPSMNTKEFLVSSRSN
>Western_clawed_frog.locus496.1
CNPCSWDTWPNLQQDRCLPRPTEFLSYGDPLGYSLAAISIFSSLIPLIILAVFIHQKKTPIVRANNYSLSCLLLLSLFLCFLCSLGFIGYPQPEKCLLRQVAFGMVFALCISCVLAKTITVVIAFNATKPGSSLRKWTGIRVSYCLILL
CALNQLTICGIWLIFFPPFYELDTDTKPGIIILNCNEGSPFAFWCMLGYLGLLASISFIVAFLARRLPDSFNEAKLITFSMLAFLSVWVSFIPAYLSARGMYTVAMEVFAILSSSWAVVGCIFVPKCYIVLFRPNMNSREHLMVKGRG
>Western_clawed_frog.locus478.1
CHPCSWDTWPNLQQDRCLPRTVEFLTYTEPLGYSLAAITSFSTLTPITILGVFIYYRKTPIVRANNYSLSCLLLLSLFLCFLCSLGFIGYPQPEKCLLRQVAFGMVFALCISCVLAKTITVVIAFNATKPGSRLRKWTGVKVPYCVIVF
CALIQLFLCVMWLILSPPFHEYDTDTKPGVLIVNCNEGSTTAFWCMLGYLGLLASISFIVAFLARRLPDSFNEAKLITFSMLAFLSVWVSFIPAYLSARGMYTVAMEVFAILSSSWAVVGCIFVPKCYIILFRPNMNSREHLLGK---
>Western_clawed_frog.locus475.1
CHPCSWDTWPNLQQDRCLPRPTEFLTYTEPLGYSLAAITSFSTLTPITILGVFIYYRKTPIVRANNYSLSCLLLLSLFLCFLCSLGFIGYPQPEKCLLRQVAFGMVFALCISCVLAKTITVVIAFNATKPGSRLRKWTGAKVPYCVIVF
CALIQLFLCVMWLILSPPFHEYDTDTKPGVLIVNCNEGSTTAFWCMLGYLGLLASISFIVAFLARRLPDSFNEAKLITFSMLAFLSVWVSFIPAYLSARGMYTVAMEVFAILSSSWAVVGCIFVPKCFIVLFRPNLNSREHLLGK---
>Western_clawed_frog.locus455.1
CHPCSWDTWPNLQQDRCLPRPTEFLSYGDPLGYSLAAISIFSSLIPLIILAVFIHQKKTPIVRANNYSLSCLLLLSLLLCFLCSLGFIGYPQPEKCLLRQVAFGMVFALCISCVLAKTITVVIAFNATKPGSRLRKWTGVKVSYCLIMF
CVFIQLFVCIMWLILSPPFHELETDTKPGVIIVNCNEGSPTAFWCMLGYLGLLASISFIVAFLARRLPDSFNEAKLITFSMLAFLSVWVSFIPAYLSARGMYTVAMEVFAILSSSWAVVGCIFVPKCVIILFKPNLNTRDHIMFKGSN
>Western_clawed_frog.locus470.1
CHPCSWDTWPNLQQDRCLPRPTEFLSYGDPLGYSLAAISIFSSLIPLGVLGVFIHQKKTPIVRANNYSLSCLLLLSLFLCFLCSLGFIGYPQLQQCLLRQVAFGMVFALCISCVLAKTITVVIAFNATKPGSRLRKWTGVKVSYCLIMF
CVFIQLFVCLMWLILSPPFHELDTDTKPGVIIVNCNEGSPTAFWCMLGYLGLLASISFIVAFLARRLPDSFNEAKLITFSMLAFLSVWVSFIPAYLSARGMYTVAMEVFAILSSSWAVVGCIFVPKCFIILFKPNRNSREHLMGKGRN
>Western_clawed_frog.locus456.1
CHPCSWDTWPNLQQDRCLPRPTEFLSYGDPLGYSLAAISIFSSLIPLIILAVFIHQKKTPIVRANNYSLSCLLLLSLFLCFLCSLGFIGYPQPEKCLLRQVAFGMVFALCISCVLAKTITVVIAFNATKPGSRLRRWTGVKVSYSLIAL
CAFIQSILCLLWLVLSPPFPESDISTKPGIIIVNCNEGSPTAFWFMLGYLGLLASISFIVAFLARRLPDSFNEAKLITFSMLAFLSVWVSFIPAYLSARGMYTVAMEVFAILSSSWAVVVCIFMPKCFIILFKPEKNSREHLRAKGKG
>Western_clawed_frog.locus463.1
CQPCSWDSWPNLQHTKCLPRPTEFLSYEEPLGYSLAAIAIVSSLIPLNIFGVFIHYRKTPIVRANNYIVSCLILLSLFLCFLCSLGFIGYPQPEKCLLRQVAFGMVFALCISCVLAKTITVVIAFNATKPGSRLRKWTGVKVPYCLITL
CVSIQFFLCVMLLIYACPFPELDINTKPGIILVNCNEHSPIAFWCMLGYLGLLASISFIVAFLARRLPDSFNEAKLITFSMLAFLSVWVSFIPAYLSARGMYTVAMEVFAILSSSWAVVGCIFVPKCFIILLRPNMNSREYLMRKDKG
>Western_clawed_frog.locus469.1
CQPCSWDTWPNPQHTRCLPRPTEFLSYEEPLGYSLVVIAILSSLIPLVILGVFIRYNKTPIVRANNYSLSCLLLLSLFLCFLCSLGFIGYPQPEKCLLRQVAFGMVFALCISCVLAKTITVVIAFNATKPGSRLRKWTGVKVSYCLILF
CVFIQLFLCIMWLIFSPPFHELDTDTKPEVIVVCCNEGSPTAFWCMLGYLGLLASISFIVAFLARRLPDSFNEAKLITFTMLAFLSVWVSFIPAYLSARGMYTVAMEVFAILSSSWAVVGCIFVPKCYIILFKPNRNSRELLMGKGRN
>Western_clawed_frog.locus490.1
CHPCSWDTWPNLQQDRCLPRTREFLSYEEPLGYSLAAISILSSLIALVILGLFIQYKRTPIVRANNYSLSCLLLLSLFLCFLCSLGFIGYPQPEKCLLRQVAFGLVFALCISCVLAKTIIVVIAFKATKPGSRLRKWTRVRVSYWIIGF
GVFIQSFLCVLWLLFSPPFPETDTDTKPGVIILNCNEGSPISFWCMLGYLGLLAAISFIVAFLARRLPDSFNEAKFIIFSMLAFLSVWVSFIPAYLSARGMYTVAMEVFAILSSTWAVLVCIFMPKCYIVLFRPNLNSRKYLMSNGRS
>Western_clawed_frog.locus473.1
--------------------
MTEFLSYESPLGTSLAAIAILSSMGPLLILGVFKSYKQTPIVRANNYSLSCLLLLSLFLCFLCSLGFIGYPQPEKCLLRQVAFGMVFALCISCVLAKTITVVIAFNATKPGSRLRKWTGVKTSSVLIGFCVFIQLFVCVIWLIFFPPVP
EHDTDTKPGVIIVNCNEGSPTAFWCMLGYLGLLASISFIVAFLARRLPDSFNEAKLITFSMLAFLSVWVSFIPAYLSARGMYTVAMEVFAILSSSWAVVGCIFVPKFFIVLFRPHMNSREHFKVKNVK
>Western_clawed_frog.locus472.1
-------
MWPNLQQDRCLPRPTEFLSYGDPLGYSLAAISIFSSSIPLNILGVFIHFKKTPIVRANNYSLSCLLLLSLFLCFLCSLGFIGYPQPEKCLLRQVAFGMVFALCISCVLAKTITVVIAFNATKPGSRLRKWTGVKVSYCVIGFCIFIQLF
VCVIWLIFFPPVPEHDTDTKPGVIIVNCNEGSPTAFWCMLGYLGLLASISFIVAFLARRLPDSFNEAKLITFSMLAFLSVWVSFIPAYLSARGMYTVAMEVFAILSSSWAVVGCIFVPKCYIILFRPNMNSREHFKVKKKK
>Western_clawed_frog.locus451.1
CHPCSWDTWPNLQQDRFLPRPTEFLAYEEPLGYSLAAIAIFSSLFPLIILGVFIYYKKTPVVRANNYSLSCLLLLSLFFCFLCSLGFIGYPQPEKCLLRQVAFGMVFALCISCVLAKTITVVIAFNATKPGSRLRKWTGVKVPYCIIGF
CSSSQSCMSITRLIFSHPFAKLDTSTKPGVIIIYCTEGSPIAFWCMLGYLGLLASISFIVAFLARRLPDSFNEAKLITFSMLAFLSVWVSFIPAYLSARGMYTVAMEVFAILSSSWAVVGCIFVPKCYIVLFRPNMNTREQFKIKMKF
>Western_clawed_frog.locus453.1
CHPCSWDTWPNLQQDRCLPRPTEFLSYGDPLGYSLAAISILSSLIPLTIFGIFIRFKKTPIVRANNYSLSCLLLLSLFLCFLCSLGFIGYPQPEKCLLRQVAFGMVFALCISCVLAKTITVVIAFNATKPGSRLRKWTGVKVSYCFIAF
CLFFQLFLCIMWLIFSPPFTELDTITYPGVIIVNCNEGSPTAFWCMLGYLGLLASISFIVAFLARRLPDSFNEAKLITFSMLAFLSVWVSFIPAYLSARGMYTVAMEVFAILSSSWAVVGCIFVPKCYIVLFRPNMNSREHLTVKSKV
>Western_clawed_frog.locus471.1
CHPCSWDTWPNLQQDRCLPRPTEFLSYGDPLGYSLAAISIFSSLIPLGVLGVFIHQKKTPIVRANNYSLSCLLLLSLFLCFLCSLGFIGYPQPEKCLLRQVAFGMVFALCISCVLAKTITVVIAFNATKPGSRLRKWTGVKVSYCVIGF
CAFLQLVVCVLWLSLSSPFPELDTDTKPGVIIVNCNEGSPTAFWCMLGYLGLLASISFIVAFLARRLPDSFNEAKLITFSMLAFLSVWVSFIPAYLSARGMYTVAMEVFAILSSSWAVVGCIFVPKCYIVLFRPNMNSRENLMGKGRS
>Western_clawed_frog.locus445.1
CHPCSWNRWPNLQQDKCLPRPTEFLSYGDPLGYSLAVTSILSSLIPLIMFGIFLHYKKTPVVRANNYSLSCLLLLSLFLCFLCSLGFIGYPQPEKCLLRQVAFGMVFALCISCVLAKTIIVILAFNATKPGSSLKKWIGVKLSLCVIGF
CVSIQFFVCLMWLIVSSPFSELDTETKPGILIVNCNEGSSIAFWCMLGYLGLLASISFIVAFLARRLPDSFNEAKLITFSMLAFLSVWVSFIPAYLSARGMYTVAMEVFAILSSSWAVVACIFVPKCFIVLFRPTMNSRELLS-KGRG
>Western_clawed_frog.locus485.1
CQPCSWDTWPNLQQDRCLPRPTEFLSYEQPLGYSLAFSSVFLGLTPLVILGIFVCNNKTPIVRANNYSLSCLLLLSLFLCFLCSLGFIGYPQPEKCLLRQVAFGMVFALCISCVLAKTITVVIAFNATKPGSRLRKWTGVKVSYCVIGF
CIFVQLCVCGIWLILSPPFSELDAETKPGIIIINCNEGSATAFWCMLGYLGFLASISFIVAFLARCLPDSFNEAKLITFSMLAFLSVWVSFIPAYLSARGMYTVAMEVFAILSSSWAVVGCIFVPKCYIVLFRPNMNSREHLLGKSRI
>Western_clawed_frog.locus487.1
CQPCSWDTWPNLQQDRCLPRPTEFLSYEQPLGYSLAAISIFSTLAPLVILRIFVCSNKTPIVRANNYSLSCLLLLSLFLCFLCSLGFIGYPQPEKCLLRQVAFGMVFALCISCVLAKTITVVIAFNATKPGSRLRKWTGVKVSYCVIGF
CIFVQLCVCGIWLILSPPFSELDTETKPGIIIINCNEGSPTAFWCMLGYLGLLASISFIVAFLARRLPDSFNEAKLITFSMLAFLSVWVSFIPAYLSARGMYTVAMEVFAILSSSWAVVGCIFVPKCYIVLFRPNMNSREHLLGKSRI
>Western_clawed_frog.locus486.1
CQPCSWDTWPNLQQDRCLPRPTEFLSYEQPLGYSLAAISIFSTLAPLVVLRIFVCNNKTPIVRANNYSLSCLLLLSLFLCFLCSLGFIGYPQPEKCLLRQVAFGMVFALCISCVLAKTITVVIAFNATKPGSRLRQWTGVKVSYCVIGF
CIFVQLCVCGIWLILSPPFSEFDTETKPGIIIINCNEGSPTAFWCMLGYLGLLASISFIVAFLARRLPDSFNEAKLITFSMLAFLSVWVSFIPAYLSARGIYTVAMEVFAILSSSWAVVGCIFVPKCYIVLFRPNMNSREHLLGKSRI
```

>Western_clawed_frog.locus488.1
CHPCSWDTWPNLQQDRCLPRPTEFLSYGDPLGYSLAAISIFSTLAPLVVLRIFICNNKTPIVRANNYSLSCLLLLSLFLCFLCSLGFIGYPQPEKCLLRQVAFGMVFALCISCVLAKTITVVIAFNATQPGSRLMKWTGVKVSYCVIGF
CIFVQLCVCGIWLIFSPPFSEFDTETKPGIIIVNCNEGSPTDFWCMLGYLGLLATISLIVAFLARRLPDSFNEAKLITFSMLAFLSVWVSFIPAYLSARGMYTVAMEVFAILSSSWAVVGCIFVPKCYIVLFRPNMNSREHLLGKSRT
>Western_clawed_frog.locus491.1
CHPCSWDTWPNLQQDRCLPRPTEFLSYGDPLGYSLAAISISSTLAPLVVLRIFICNNKTPIVRANNYSLSCLLLLSLFLCFLCSLGFIGYPQPEKCLLRQVAFGMVFALCISCVLAKTITVVIAFNATKPGSRLRKWTGVKVSYNLLVF
CVFIQLCVCTVWLTLSPPFHELDTDTQPGIIIANCNEGSPAAFWCMLGYLGLLATISFIVAFLARRLPDSFNEAKYITFSMLAFLSVWVSFIPAYLSARGMYTVAMEVFAILSSSWALLGCIFVPKCFIVIFRPTKNSKEQVLGRVRG
>Western_clawed_frog.locus468.1
CHPCSWDTWPNLQQDRCLPRPTEFLSFGDPLGFSLTGTAIFSSLIPLLILRVFTWFKKTPIVKANNYSLSCLLLLSLFLCFLCSLGFIGYPQLQQCLLRQVAFGLVFALCISCVLAKTITVVIAFNATKPGSRLRKWTGVKVSYPFILI
CTLIQLCVCVLWLFVIPPFPELDTDTKPGVIIVNCNEGSPTAFWCMLGYLGLLASISFIVAFLARRLPDSFNEAKLITFSMLAFLSVWVSFIPAYLSARGMYTVAMEVFAILSSSWAVLGCIFVPKCYIVLFRPNMNTKGHLLGKSKS
>Western_clawed_frog.locus467.1
CHPCSWDTWPNLQQDRCLPRPTEFLSYGDPLGFSLACTAIFSSLIPLVILRVFVCYKETPIVRANNYSLSCLLLLSLFLCFLCSLGFIGYPQPEKCLLHQVAFGMVFALCISCVLAKTITVVIAFNATKPGSRLRQWTGVKVSYSVILT
CVSIQLFVCAIWLVILPPFLELDVDTKPGVMIVNCNEGSPTAFWCMLGYLGLLASISFIVAFLARRLPDSFNEAKLITFSMLAFLSVWVSFIPAYLSARGMYTVAMEVFAILSSSWAVVGCIFVPKCFIILLRPNMNSREHLMGKGRG
>Western_clawed_frog.locus448.1
CHPCSWDTWPNLQQDRCLPRPTEFLSYGDPLGYSLAVISIFSSWVPVIILGVFICYKKTPVVRANNYSLSCLLLLSLFLCFLCSLGFIGYPQPEKCLLRQVAFGMVFALCISCVLAKTITVVIAFNATKPGTILRKWTGLKVSYSVIVL
SAFIQLCVCLLWLIFSPPFSELNSEDQPGIIIINCNEGSPAAFWCMLGYLGLLATISFIVAFLARRLPDSFNEATFITFSMLAFLSVWVSFIPAYLSARGMYTVAMEVFAILSSTWAVVVCIFVPKCFVVLFRPTMNSKELLRRKDIG
>Western_clawed_frog.locus460.1
CHSCSWNKWPNLQRDKCLQRTTEFLSYKEPLGYGLSAISIFSSLIPLVILFVFNYYKTTPVVRANNYSLSCLLLLSLFLCFLCSLGFIGYPQPAQCLLRQVAFGIVFSLCISCVLAKTITVIIAFNATKPGSRLRQWAGVRVSYCVIVL
CTFTQLVICVIWLFFFPPFPELDNDTQPGIIIANCNEGSPTAFWCMLGYLGFLASISFIVAFMARRLPDSFNEAKFITFSMLAFLSVWVSFIPAYLSARGMYTVAMEVFAILSSSWTVMLCIFVPKCFIILFRPNMNSREHLMGKVRG
>Western_clawed_frog.locus459.1
CHSCSWDTWPNLQRDKCLLRTTEFLSYKEPLGYGLSAISIFSSLIPLVILFVFIYYKTTPVVRANNYSLSCLLLLSLFLCFLCSLGFIGYPQPAQCLLRQVAFGIAFSLCISCVLAKTITVVIAFNATKPSSRLRQWAGVRVSYCVIVL
CTFIQLVICGIWLFFFPPFPELDIDTQPGIIIANCNEGSPTAFWCMLGYLGFLATISFIVAFLARRLPDSFNEAKFITFSMLAFLSVWVSFIPAYLSARGMYTVAMEVFAILSSSWTVKLCIFVPKCFIILFRPNMNSREHLMGKEVK
>Western_clawed_frog.locus492.1
CLPCSWDTWPNLHQDRCLPRPTEFLSYRDPLGYSLAATAVFSSLITLVILGVFYFFKKTPIVRANNYSLSCLLLLSLFLCFLCSLGFIGYPKPAQCLLRQVAFGMVFALCISCVLAKTITVVIAFNATKPGSRLRKWSGIKVSYCVIVF
CVIIQIGICTVWLLFYPPFSELDNDNQQGVIIINCNEGSPTAFWCMLGYLGLLASISFIVAFLARRLPDSFNEAKLITFSMLAFLSVWVSFIPAYLSARGMYTVAMEVFAILSSSWAMVVCIFVPKCFIVIFRPNKNAREHLMSKGRS
>Western_clawed_frog.locus446.1
CHPCSWDTWPNLQQDRCLPRPTEFLSYEKPLGYSLAAISIFSSLIPLIMLAVFIYYKKTPIVRANNYSLSCLLLLSLFLCFLCSLGFIGYPQPEKCLLRQVAFGMVFALCISCVLAKTITVIIAFNATKPGSRLKQWIGLKVSYCVTVL
CVFVEFCMCMIWLIYFPPFPELDTNTKPGIIIVNCNEGSAAAFWCMLGYLGLLATISFIVAFLARRLPDSFNEAKLITFSMLAFLSVWVSFIPAYLSARGMYTVAMEVFAILSSSWAMVGCIFVPKMFILLFKPHLNSREHLIRNE--
>Western_clawed_frog.locus447.1
CHPCSWDTWPNLQQDRCLPRPTEFLSFQEPLGYSLAVAAIFSSLSPLTVLGVFIHYKNTAIIRANNYSLSCLLLLSLFLCFLCSLGFIGYPQPEKCLLRQVAFGMVFALCISCVLAKTITVVIAFNATKPGSRLRKWTGIKVSYCVIIF
CVFIQLWICILWLLFFPPFHELDLDTKPGFIIVTCNEGSPTAFWCMLGYLGLLASISFIVAFLARRLPDSFNEAKLITFSMLAFLSVWVSFIPAYLSARGMYTVAMEVFAILSSSWALVGCIFVPKCFIVLFRPHMNSRDYLMGKGRN
>Western_clawed_frog.locus452.1
CHPCSWDTWPNLQQDRCLPRPTEFLSYGDPLSYSLAATAIFSSLVPLVILGVFINYKETPIVRANNYPVSCLLLFSLFLCFLCSLGFIGYPQPEKCLLRQVAFGMVFALCISCVLAKTITVVIAFNATKPGSRLRKWTGVKVPYFLILC
CALIQLCICVIWLIFFQPFHELDTDTKPGIIIVNCNEGSSTAFWCMLGYLGLLATISFIVAFLARRLPDSFNEAKLITFSMLAFLSVWVSFIPAYLSARGMYTVAMEVFAILSSSWALVGCIFVPKCFIILFSPHINSREHFTIKPIN
>Western_clawed_frog.locus466.1

MAEFLSYETPLGFSLAAIASFSSLVPLIIWGVFMYYKETPIVRANNYSVSCLLLLSLFLCFLCSLGFIGYPQPEKCLLRQVAFGMVFALCISCVLAKTITVVIAFNATKPGSRLRKWTGVKVPYFLILCCALIQLCICVIWLIFFQPFH
ELDIDTKPGIIIVNCNEGSPTAFWCMLGYLGLLASMSFIIAFLARRLPDSFNEAKLITFSMLAFLSVWVSFIPAYLSARGMYTVAMEVFAILSSSWALMVCIFLPKCFIILFRPNMNSREHLMGKGQQ
>Western_clawed_frog.locus465.1

MTEFLSYESPLGTSLAAIAILSSMGPLLILGVFKSYKQTPIVRANNYSLSCLLLLSLFLCFLCSLGFIGYPQPEKCLLRQVAFGMVFALCISCVLSKTIIVVIAFNATKPGSRLRKWTGVKVPYFLILCCVLIQLCICLIWLIFFPPFH
ELDTDTKPGLIIVNCNEGSPTAFWCMLGYLGLLVTISFIVAFLARRLPDSFNEAKLITFSMLAFLSVWVSFIPAYLSAQGMYTVAMEVFAILSSSWALVVCIFIPKCFIILLRPNVNSRENIMGKRRG
>Western_clawed_frog.locus489.1
CQPCSWDTWPNLQQDRCLPRTIEFLSYEEPLGYSLAAITIFSSIIPLVILGLFIQYKETPIVRANNYSLSCLLLLSLFLCFLCSLGFIGYPQPEKCLLRQVAFGMVFALCISCVLAKTITVVIAFNATKPGSRLRKWTGVKVPYCVIIC
CVFIQLCVCVLWLTLSSPFPEQDTRTKPGLIIVNCNEGSPPAFWCMLGYLGLLASISFIVAFLARRLPDSFNEAKLITFSMLAFLSVWVSFIPAYLSARGMYTVAMEVFAILSSSWALVGCIFVPKCYIVLLRPDMNSREHLMGKRKD
>Western_clawed_frog.locus464.1
CNPCSWDTWPNQHRAKCIARTTEFLSYEDDLGLILAPVSILSSLVPLVFWGVFVHYKKTPIVRANNYSLSCLLLLSLFLCFLCSLGFIGYPQPEKCLLRQVAFGMVFALCISCVLAKTITVVIAFNATKPGSRLRKWTGVRLPYFVIIL
CTFIQFSLCMIWLIFYPPVPELNTETKPGIIIVSCNEGSPTAFWCMLGYLGLLATISFIVAFLARRLPDSFNEAKLITFSMLAFLSVWVSFIPAYLSARGMYTVAMEVFAILSSSWAVVGCIFVPKCFIVLFRPNMNSREHLMGKRNI
>Western_clawed_frog.locus498.1
CFRCPWDQWPNNQKDQCILKPVEFLSCDDPVGVTLITSSVISSIIPVALLGLFINFKTTPIVRANNYLLSCVLLVSLSLCFLCSLGFIGYPQPEKCLLRQVAFGMVFALCISCVLAKTITVVIAFKATKPGSKLRKWTGTKVTYLVTGL
GVLLQLGICIIWLSTFPSFPENDIQTQPGVIIAHCNEGFPTAFWCMLGYLGLLATISFVLAFLVRRLPDSFNEAKYITFSMLAFLSVWVSFIPAYLSARGKYTDAMEVFAILSSTWALVICIFLPKCFIIIFQPELNNKDSLMGRGKK
>Western_clawed_frog.locus428.1

MWPNSKRDRCFPKPIEFLSYDDSLGITLAAISVFSCVVPLAIFRLFIHYKSTPIVRANNHLVSCVLLVSLSFCFLCALAFIGYPQPEKCLLRQAAFGLVFTLCVSCILAKTIIVVFAFMATKPGSSLKKWTTPRVPYMIITICTFIQLT
LCVIWLTISPPFFPQYNSEAKPGIIFVECDENSPYAFWCMLGYLGFLASVSFTVAFLARRLPDNFNEAKLITFSMLAFLSVWVYFIPASLSAQGKYTVAMEIFAILTSTWALVICMFLPKCFVILFRPNMNSKENLMGKDRR
>Western_clawed_frog.locus425.1
CVTCTWDEWPNLQKTKCLQKNIEYLSYEDPLGASLAVTGIISSLVPVVILRLFIKYKSTPIVKANNYSLSCLLLLCLPLCFLCSLTFIGYPQPEKCLLRQAAFGLVFTLCISCILAKTIMVVFAFMAHQPGSKLRILVRPFVSYLIISI
CFLLQLILCLVSLVKLPSFTEYDTQTKPGIIIVQCNNGFSLAFWSMLAYLGLLATISFIVAFLARRLPDSFNEAKFITFSMLAFLSVWVSFILASLNAQGKYTEAVEVFAILSSSWALVACMFLPKCFIMLFRPALNSKKRLIKDNSH
>Western_clawed_frog.locus422.1
CFKCSWEEWPNLQNSKCIQKGIEYLSYEDPLGATLAAISILSSLVPVFILRLFIKHRTTPIVKANNYSLSCLLLLCLPLCFLCSLAFVGFPQPEKCLLCQAAFGLVFALCVSCILAKTIMVVLAFMATKPVSKLRRWASPLVSYMIISL
CFLLQFLLCTVWLLKAPPYTEYDTQTKPGLIIVQCNNGSTAAFWSMLGYLGLLATISFIVAFLSRGLPDTFNEAKYITFSMLAFLSVWASYTLASFSAQGKYTVATEIFAILSSSWALVVCMFASKCYIILFRPLLNSKKSLMKNDSR
>Western_clawed_frog.locus421.1
CYKCSWDEWPNLQKSKCLQKNIEYLSYEDPLGAGLAATGIASFWVPVLVLNVFIKYKSTPIVKANNYCLSCLLLLCLPLCFLCSLAFIGYPQPEKCLLRQAAFGLVFTLCISCILAKTFMVVFAFMATKPGSRIKKWTSPSVSYMIIFI
CFGLQFILCISWLLEAPPFPQYDIQTKPGVIIIECNNGSATAFWCMLGYLGFLAAISFAVAFLARRLPDNFNEAKFITFSMLAFLSVWVSFIPASLSAQGKYTEAMEIFAILSSTWSLVVCLFLPKCFIIVFRPNMNSKKELMKKTSA
>Western_clawed_frog.locus420.1

MQKNIEFLSYEDPLGAGLAATGITSSLVPVVILKLFMKYKTTPIVKANNYSLSCILLSCLPLCFLCSLAFIGYPQQEKCLLRQAAFGLVFALCVSCILAKTVMVVFAFMATKPGSRLRKWTSPIVSYMIIAICFALQCILCISWLLQAP
PFPQYDIQTKPGVIIIECNNGSATAFWCMLGYLGFLAAISFSVAFLARRLPDNFNEAKFITFSMLAFLSVWVSFIPASLSAQGKYTEAMEIFAILSSSWALVVCMFFPKCYIIKFKPNMNSKKQLMKKAGS
>Western_clawed_frog.locus423.1
-MKCSWDEWPNLRKSKCIEKNIEYLSYDDPLGAALTTTGIVSTLVPVLILRLFITYKETPIMKANNYSLSCLLLVCLSLCFLCSLAFIGYPQHEKCLLRQAAFGLVFTLCISCILAKTILVVLAFMATKPGSKLRKWTSRSVSYMFISI
CFFVQFLLCLVWLLRAHPFTEYNIQLRPDLIIVECNNGSPTAFWSVLGYLGLLATISFIVAFFARRLPGNFNEAKFITFSMLAFLSVWVSFIPAYLSAHGKYTEAMEIFAILSSSWALIGCMFIPKCYILLFRPNMNSKIQLIKRP--
>Western_clawed_frog.locus424.1
CAACSWDEWPNLQRSSCIPKTIEYLSYEDPLGAALAYTGSLSSLVPVLILRLFITYRTTPIVKANNYSLSCLLLLCLSLCFLCSLAFIGYPQPVKCLLRQVAFVLVFTLCVACILAKTILVVLAFMATKPGSKLKKWTSPYMPYVIISF
GFFVQCILCMVWLLMSPPFKEYNIQLKPGHIIVDCNNGSPTAFWSVLAYLGLLATISFIVAFLARRLPGNFNEAKFITFSMLAFLSVWVSFIPASLSAQGKYTEAMEIFAILSSSWALLGCMFIPKCFILLFRPNMNSKTQLIKKDSS
>Western_clawed_frog.locus426.1
CSKCPWNMWPNVDKDRCVPKATDFLSFEEPLGTTLTAATITSSLVPLSVLGLYYCYRSTPIVRANNYSLSCTLLVSLTLCFLCSLSFIGCPQQLKCFLRQVAFGMVFTLCVSCILAKTIMVVLAFMATKPGSSLKKWTGPRVSYIIVSI
CNILQLCLCICWLSLFPPFPEYNTDTQPEIIIIVCNEGSTIAFWCMLGYLGLLATISFIVAFLARHLPESFNEARFITFSMLSFLTVWISFIPASLSTRGQYIVAMEVFAILSSSWALLVCMFLPKCFIIVFRPNLNSREHLIRRARN
>Western_clawed_frog.locus427.1
CTKCSWNMWSNADKDRCIPKAIEFLSSEEPLGATLTITSIISSLVPLLVLGLFYHYKSTPIVRANNYSLSCTLLGSLSLCFLCSLPFIGCPQQLKCLLRQVAFGIVFSLCISCILAKTIMVVLAFMVTKPGSSLKKWTGPRVSYIIVSI
CNILQLCLCICWLSLFPPFPEYNTDTQPEIIIFICNEGSAIAFWCMLGYLGLLASISFIVAFLARRLPDSFNEAKLITFSMLAFLSVWVSFIPAYLSARGMYTVAMEVFAILSSSWAVVGCIFVPKCFIIVFRSSMNSREHLMRRARN
>Western_clawed_frog.locus419.1

MWPNIKRDSCLPKPREFFSYEDSLGETLATTSISSSLVPFAILVLFVHYKMTPIVRANNYLLSCLLLVSLSLCFLCSLFFIGYPHPEMCRLRQVTFGMVFALCISCILAKTFMVVVAFKATKPNSSLKKWTRPRISYLIVVLCILIQTC
LCTMWLLLSPPFFEENIRTKVGVVIVECNDGSSVAFWCMLGYLGLLASVSFFVAFLARRLPDSFNEAKFITFSMGTFLSVWISFIPATMSARGKYTVAMEIFAILSSTWALVVCMFLPKCFIILFRPNMNSREHLMGKEKR
>Western_clawed_frog.locus418.1

MWPNIKRDSCLPKPREFLSYEDSLGETLATTSISSSLVPFAILVLFVHYKITPIVRANNYLLSCLLLVSLSLCFLCSLFFIGFPHPEMCRLRQVTFGMVFALCISCILAKTFMVVVAFKATKPNSSLKKWTRPRISYLIVVLCILIQTC
LCTMWLLLSPPFFEENIRTKVGVVIVECNDGSSVAFWCMLGYLGLLASVSFFVAFLARRLPDSFNEAKFITFSMGTFLSVWISFIPATMSARGKYIVAMEIFAILSSTWALVVCMFVPKCFIILFRPNMNSREHLMGKEKR
>Caecillan.locus44365.1
CWKCSSDEWPNAKQNQCVQKSVVFLSYEEPLGATLAGASMIFSLFPVASLGLFICYRNTPIVKANNCNLSYLLLLALTFCFLCSLAFVGYPSPEKCLLRQAAFGISFALCISCVLAKTIMVVIAFNTIKPNSNFRRWVGPQLSYGVIGV
CTSIQILVCSSWLILSPPFLEYNTNTHPGMIVLECNEGSPIAFWGMLGYLWLLATFNFIVAFLARKLPDSFNEAKFITFSMLAFLTVWLSFIPAYLSTRGKYMVAMEIFAILSSSCALMFCIFFPKCYFILVKPEMNIKSHLMRKDPS
>Caecillan.locus44514.1
RDK-
QYDKWPNEKQNQCVQKSVVFFSYEEPLGATLAGASMIFSLFPVASLSLFICYRNTPIVKANNRNLSYLLLLALTLCFLCSLAFVGYPRPEKCLLRQAAFGISFALCISCVLAQTIMVVIAFDTIKPNGNFRRWVGPQLSYVVIGVCTSI
QILVCSSWLIFSPPFLEYNTNTHPGMIVLECNEGSPIAFWGMLGYLGLLATFNFIVAFLARKLPDSFNEAKFITFSMLAFLTVWLSFIPAYLSTRGKYMVAMEIFAILSPSCTLMFCIFFPKCYIILVKPEMKTKRYLMRKDPS
>Caecillan.locus44364.1
CTKCSWNQWPNARQDQCVPRIIEFLSYEEPLGAILAATSIIFSLIPLASLGLFIYHRDTPIVKANNRNISYLLLLALTLCFLCSLTFIGYPSTEKCLIRQAAFGISFSLCISCVLVKTIMVVMAFHATKPNSAFRRWVRPELSYMVISV
CILIQVLVCVSWLILSHPFSEYNTQVHPGKIIVECNEGSPLAFWCMLGYLGFLATISFIVAFLARNLPDSFNEAKFITFSMLAFLSVWMSFILAYLSARGKYMVAMEIFAILASSSALLSCIFFPKCYLILVRPELNSKENLMGRKEG
>Western_clawed_frog.locus855.1
-MKCPEDMWANERHDGCRDKTLEFLSFEEILGGTLAALSITGSLLPLSILFIFLRNADTPVVKANNRNLSYTLLVSLFFCYLCALMFIGRPITITCILRQFIFGISFVMCISCVLGKTMMVVIAFNLTQPRSSRRMWLNSRVTNTLVLV
CTAIQVIICAGWLAHSPPFQYTDNKSKAGTIIVECNKGSPTGYSCTI------------------KLPGSFNEAKLITFSMLIFGAVWISFIPAYLSTTGKYMVAVEVFAILSSSIGLVTCIFLPKVYIIILRPEMNKIGRKIIKE--
>Western_clawed_frog.locus856.1
-MKCPEDMWANERHDGCRDKTLEFLSFEEILGGTLAALSVMGSLLPLSILFIFLRNANTPVVKANNRNLSYTLLVSLFFCYLCALMFIGRPITITCILRQFIFGISFVMCISCVLGKTMMVVIAFNLTQPRSSRRMWLNSRVTNTLVLV
CTAIQVIICAGWLAHSPPFQYTDNKSKAGTIIVECNKGSPTGYSCTI------------------KLPGSFNEAKLITFSMLIFGAVWISFIPAYLSTTGKYMVAVEVFAILSSSIGLVACIFLPKVYIIILRPEMNKTGRKIIKE--
>Western_clawed_frog.locus853.1

MWANEMHDGCRDKTLEFLSFEGALGGILAALSITGSLLPLSILFIFLKNADTPVVKANNRNLSYTLLVSLCFCYLCALMFIGRPITITCILRQFIFGISFVMCISCVLGKTMMVVIAFNLTQPRSSRRMWLNSRVTNTLVLVCTAIQVI
ICAGWLAHSPPFQYTDNKSKAGTIIVECNKGSPVAYSCTI------------------KLPGSFNEAKLITFSMLIFGAVWISFIPAYLSTTGKYMVAVEVFAILSSSSGLVACIFLPKVYIIILRPEMNKIGRKIIKE--
>Western_clawed_frog.locus854.1
CMKCHEDMWANEMHDGCRDKTLEFLSSEGALGGTLAALSIMGSLLPLSILFIFLKNADTPVVKANNRNLSYTLLVSLFLCYLCALMFIGRPITITCILRQFIFGISFVMCISCVLGKTMMVVIAFNLTQPRSSRRMWLNSRVTNTLVLV
CTAIQVIICAGWLAHSPPFQYTDNKSKMGTINVECNKGSPIAYSCTI------------------KLPGSFNEAKLITFSMLIFGAVWISFIPAYLSTTGKYMVAVEVFAILSSSSGLVACIFLPKVYIIILRPEINKIGRKIIKE--
>Western_clawed_frog.locus852.1
-MKCPEDMWAKEMHDGCRNKTLEFLSFEEILGGTLAALSITGSLLPLSILFIFLKNANTRIVKANNRNLSYTLLVSLFFCFLCALMFIGRPITITCILRQFIFGISFVMCISCVLGKTMMVVIAFNLTQPRSSRRMWLNSRVTNTLVLV
CTAIQVIICAGWLAHSPPFQYTDNKSKMGTIIVECNKGSPIAYSCTI------------------KLPGSFNEAKLITFSMLIFGAVWISFIPAYLSTTGKYMVAVEVFAILSSSSGLVACIFLPKAYIIILRPEMNNIEMK--RT--
>Western_clawed_frog.locus863.1
CIRCPDNMWANEKHTACRLRTFEFLSYNEILGGMLATVSILGSLLPLSILAIFIRNSGTPVVKANNRNLSYLLLLSLCFCYLCALMFIGRPITITCILRQFIFGISFVMCISCVLGKTMMVVIAFNLTQPRSSRRMWLNSRVTNTLVLV
CTAIQVIICAGWLAHSPPFQYTDNKSKMGTIIMQCNKGSPIGYSCTI------------------KLPGSFNEAKLITFSMLIFGAVWISFIPAYLSTTGKYMVAAEVFAILSSSSGLLVCILLPKVYIIIFKPDMNKKDRKIFII--
>Western_clawed_frog.locus862.1
-MKCPEDMWANDKHNGCWMRSLEFLSFRETLGGTLTTISVLGSLLPLSILAIFIKNAETPVVKANNRNLSYLLLLSLCFCYLCALMFIGHPITITCILRQFIFGISFVMCISCVLGKTMMVVIAFNLTQPRSSRRMWLNSRVTNTLVLV

CTAIQVIICAGWLAHSPPFQYTDNKSKMDTIIMECNKGSPVAYSCTI------------------KLPGSFNEAKLITFSMLIFGAVWISFIPAHLSTTGKYMVAVEVFAILSSSSGLLVCILLPKVYIIILRPEMNKKDRKIFII--
>Western_clawed_frog.locus858.1
CMKCPEDMWANDKHNGCWMKSLEFLSFRETLGGTLTTISVLGSLFPLSILAVFIKNAETPVVKANNRNLSYILLLSLCFCYLCALMFIGRPITINCILRQFIFGISFVMCISCVLGKTMMVVIAFNLTQPRSSRRMWLNSRVTNTLVLV
CTAIQVIICAGWLAHSPPFQYTDNKSKMGTIIVECNKGSPIAYSCTI------------------KLPGSFNEAKLITFSMLIFGAVWISFIPAYLSTTGKYMVAVEIFAMLSSSSGLVTCIFLPKVYVIILKPEMNKKDRKMTRNSY
>Western_clawed_frog.locus860.1
CMKCTEDMWANDKHNGCWIRTLEFLSFKELLGGILATISVLGFLLPLSILAIFVKNSKTPVVKANNQNLSYLLLLSLCFCYLCALMFIGRPITITCILRQFIFGISFVMCISCVLGKTMMVVIAFNLTQPRSSRRMWLNSRVTNTLVLV
CTAIQVIICAGWLAHSPPFQYTDNKSKAGTIIVECNKGSPVAYSCTI------------------KLPGSFNEAKLITFSMLIFGAVWISFIPAYLSTTGKYMVAVEIFAMLSSSSGLVTCIFLPKVYIIILRPEMNTKDRKICIK--
>Western_clawed_frog.locus857.1

MWANEEHNACGMRTLELLTYNEALGGTLATLSVMGFLVPLTILTIFIKNSETPVVKANNRNLSYLLLLSLCFCYLCALMFIGRPITITCILRQFIFGISFVMCISCVLGKTMMVVIAFNLTQPRSSRRMWLNSRVTNTLVLVCTAIQVI
ICAGWLAHSPPFQYTDNKSKAGTIIVECNKGSPVAYSCTI------------------KLPGSFNEAKLITFSMLIFGAVWISFIPAYLSTTGKYMVAVEAFAILSSSSGLLACIFLPKVYIIILKPEKNKKERNILIT--
>Western_clawed_frog.locus865.1

MWPTEQHDGCRPRSLEYLAYEDPLGGTLASLSVVGSLIPLSILGIFLRNSKTPVVKANNRNLSYLLLFALFLCFLCSLMFIGAPVHLICVLRQITFGVSFVLCVSCVLGKTIMVVIAFNATQPKSSRRMWLNSRIPNTLIIVCMTIQLI
ICSVWVMHSPSFKNNDITSKLGVTILECVEGSPVAFWCMI------------------NLPGSFNEAKMITFSMLIFGAVWISFIPTYLSTRGKESVAVEIFAILCSCSGLLALLFFPKCYIIILRPEMNNKEFLTGKRGF
>Western_clawed_frog.locus864.1

MWPTERHDGCRPRSLEYLAYEDPLGGTLASLSVVGSLIPLSILVIFLRNSKTPIVKANNRNLSYLLLFALFLCFLCSLMFIGAPVNLICVLRQITFGVSFVLCVSCVLGKTIMVVIAFNATQPKSSRRMWLNSRIPNTLVIVCMTIQLI
ICSVWIMYSPPFKNNDITSKLGVTILECVEGSPVAFWCMI------------------KLPGSFNEAKMITFSMLIFGAVWISFIPTYLSTRGKESVAVEIFAILCSCSGLLALLFFPKCYIIILRPEMNNKEFLTGKRGF
>Anole_lizard.locus203.1

MCLPKKELFLAFHEPLGASLASASVCGSIMPIMILAIFIAHQETPLVRANSRELSYLLLIGLALSSLCCLLFLGQPSHTSCLLRQVVFGMTFALCLSCILAKTILVVAAFRAISPRGHSRVWLSSRMPCSVILLGCLLQFLLCAIWSMT
SPPYIKRN--SFPSAITLSCHEGSPTAFWGMLSYLGFLAGLSLTAAFLARNLPEAFNEASLICFSLSGSFGVWLAFVPAYLTSPEYYAAVTEAFAILASSLSLICGMFLPKCYILLFHPQLNNRRCLMRLEKP
>Western_clawed_frog.locus499.1
CYLCSDDEWPNSARNHCLSRAIELLSLSEPFGLSLGTTAVIGSLLPAAVLVIFIRNRDTPLVRANNRGLSFLLLAALLLSFLCPLLLLFPPGTFLCFIRQAAFGILFALCISCLLAKTVIVVLAFRANQLGKGLMLIMGPRSPILIALS
CTVFQLTLCLSWIIQSPPFPEQDIKSQVGTITIKCNEG--LGFWFMLGYLGLLSTICFVAAFLARKLPGAFNEATHITFSMVVFLCVWVSFVPAYLSTHGKLAVATEIFAILSSSAGLLFCIFSPKCYIILLKPQLNTRPLVSGHHRR
>Coelacanth.locus294.1
CLKCPDDQWSNELQDQCIPTTVEFLAYEDAVGATLAVFSIFCALLFFTVLCIFIKYRNTPIVKANNRELSYLLLLSLVLCFLSSLVFIGQPKTWSCMLRQTAFGIIFALSVSCVLAKTVMVVIAFNVTKPNSNLRKWVGPKLAISVVLV
CTVIQIIICIVWLASAPPFPTENMKSQIGTIILECNDRSTIAFWCVLGYMGLLATISFVVAFLARNLPDSFNETKYITFSMLVFVSVWLAFIPAYLSTRGKYMVTVEIFAILASGAGSLACIFFPKCYIILLRPDMNKRESLMGKSKL
>Coelacanth.locus295.1

MGATLAALSILCALLSKTILCIFIKYRNTPIVKANNRELSYVLLLALVLCFLSSLVFIGQPKTWSCMLRQAAFGIIFALSVSCVLAKTIMVVIAFNATKPNSNLKKWVGPKLSNSIVFVCTLIQIIICIVWLASTPPFSTKNMKSQIGT
IILECNDGSTTAFWCVLGYMGLLAIVSFIVAFLARNLPDSFNEAKFITFSMLVFVSVWLAFIPAYLSTKGKYMVAVEIFAILASSAGLLACIFFPKCYIILLRPDMNNRESLMGKSKL
>Coelacanth.locus292.1

MGATLAVLSIFCALVSMIVLCIFIKYRNTPIVKANNRELSYILLLSLVLCFLSSLLFIGKPKTWSCMLRQVAFGIIFALSVSCVLAKTIMVVIAFNATKPNSKLRKWVGAKLAISIVLICTIIQIIICIVWLARVPPFPTENMESQIGK
IILECNEGSTTAFWCMMGYMGLLAIVSFIEAFLARNLPGSFNEAKFITFSMLVFVTVWLAFIPAYLSTRGKYMVAVEIFAILASTAGLLACIFSPKCYIILLRPDMNNKESLMGKGKL
>Coelacanth.locus389.1

MGATLTALSIFCALVSITVLCIFIKYRNTPIVKANNRELSYLLLLSLALCFLSSLVFIGQPKTWSCMLRQAAFGIIFALSVSCVLAKTIMVVIAFNATKPNSNLKKWVGPKLANSIVLVCTLIQIIICIVWLASAPPFATENMKSQIGK
MILECNEGSSTAFWCMLGYMGLLAIISFVVAFLARNLPDSFNEAKFITFSMLVFVSVWLAFIPAYLSTKGKYMVAVEIFAILASSAGLLACIFFPKCYIILLKPDMNNRESLMGKGKL
>Coelacanth.locus290.1
CLKCPYDRWSNEQQDECIPKTIEFLAYEDAMGTTLATVSILCALVSTTVLCIFIKYRNTPIVKANNRELSYILLLSLVLCFLSSLVFIGQPKTWSCMLRQAAFGIIFALSVSCVLAKTMMVVIAFNATKPSSKLNKWVGSKISNSIVLV
CTVIQIIICIVWLASAPPFPTENMESHIGKIIIECNEGSTTAFWCVLGYMGLLAIVSFKVAFLARNLPDSFNEAKFLTFSMLLFVSVWLAFIPAYLSTRGKYMVAVEIFAILASSSGLLACIFFPKCYIILLRPDMNTRKSLMGKDKL
>Coelacanth.locus321.1

MGATLATVSILCALASTTVLCIFIKYRNTPIVKANNRELSYLLLLSLVLCFLSSLVFIGQPKSWSCMLRQTAFGIIFALSVSCVLAKTIMVVIAFNATKPNSNLKKWVGPKLSNSIVFVCTVIQVIICIAWLASAPPFHTENMKSQIGK
IIIECNEGSITAFWCVLGYLGLLAIVSFIVAFLARNLPDSFNEAKFITFSMLIFISVWLAFIPVYLSTKGKSIVAVEILAILVSSAGLLACIFFPKCYIILLRPDMNTRESLIGKGKL
>Coelacanth.locus320.1
CLKCPDDQWSNE-
QDKCISKTIEFLAYEDALGATLATASIFCALASTTVLCIFIKYRNTPIVKANNRELSYLLLLSLVLCFLSFLVFIGQPKTWSCMLHQAAFGIIFALSISRVLAKTIMVVIAFNATKPNSNLKKWVGSKLSNSIVLICTIIQIIICIVWL
ASAPPFPTENMKSQIGKIIIECNNGSTAAFWCVLGYMGLLAIVSFIVAFLARNLPDSFNEAKFITFSMLVFVSVWLAFIPAYLSTRGKYMVAVEIFAILFSSAGLLACIFFPKCYIILLRPDMNTRESLIGKGKL
>Coelacanth.locus346.1
CLKCPDDQWSNELQHECIPKTIEFLAYEDALGAALATLSILCALASTNVLCIFIKYRSTPIVKANNRELSYILLLALVLCFLTSLVFIGQPKTWSCMLRQAAFGIIFALSVSCVLAKTIMVVIAFNATKPNSNLNKWVGPKLLNCIVLG
CTVIQIIICIIWLASAPPFPTENTKSEIGMIIIECNEGSTTAFWCVLGYMGLLAIVSFIVAFLARNLPDSFNEAKFITFSMLVFVSVWLAFIPAYLSTKGKYMVAVEIFAILASSAGLLACIFFPKCYIILLRPDMNTRESVMGKGKL
>Coelacanth.locus322.1

MGSTLATVSIFCALTSTTVFCIFIKYRNTPIVKANNCELSYLLLLALVLCFLSSLVFIGKPRTWSCMLRQVVFGIIFALSVSCVLAKTIMVVIAFNATKPNSNLKKWVGPKLSNSIVFVCTVIQIIICIGWLASAPPFATENMKSQIGM
IIIECNEGSTIAFWCVLGYMGLLAIVSFIVAFLARNLPDSFNEAKFITFSMLVFVSVWLAFIPAYLSTKGKYMVAVEIFAILVSSAGLLACIFVPKCYIILLKPDMNTRESLMGKGKL
>Coelacanth.locus324.1
CLKCPDDQWSNELQDQCIPKIIEFLAYDDAMGTTLVAISIFCALTSTTVLCIFIKYRNTPIVKANNRELSYLLLLALVFCFLSSLVFIGQPRMWTCMLRQVAFGIIFALSVSCVLAKTIMVVIAFNATKPNSNLKKWVGPKLANSIVFV
CTAIQIIICIVWLASAPPFSTKNMKSQVGMIIIECNEGSTTAFWCVLGYMGLLAIVSFIVAFLARNLPDSFNEAKFITFSMLVFVSVWLAFIPAYLSTRGKYMVAVEIFAILASSAGLLACIFFPKCYIILLRSDMNTREYLMGKGKL
>Coelacanth.locus318.1
--
MCPDDQWSNERRDKCIPKLREFLSYEDPMGATLAAISVFCMLLSVTILCIFMKYRESPIVKANNRELSYLLLLALVLCFLCSLVFIGQPRTWTCMLRQPAFGIIFALCVSCVLAKTIMVVIAFNATKPNSNLRKWVGPKLPNIIVFVCT
GIQFIICVVWLANYPPFAEKNTKSQTGIIIIECNEGSAPAFWCMLGYMGLLAIICFIVAFLARNLPDSFNEAKFITFSMVVFVSVWLAFIPAYLSTRGKYMVAVEIFAILASSAGLLSCIFFPRCVIILMKPDMNTRAYLMGKSIK
>Coelacanth.locus268.1

MGATLAAISIFCAVICTTAFCIFIKFQDTPIVKANNRGLSYVLLLCLVLCFLCSLVFIGQPKTWTCMLRQTAFGITFALGVSCVLAKTIMVVIAFNATKPNSNLKKWVGSRLPNSIVLVCTTIQVIICIAWLATSPPSSEQNMKSQIGI
IIIECNEGSTTAFWCVLGYMGLLATVSFIVAFLARNLPDSFNEAKLITFSMFVFVSVWLSFIPAYLSTRGKYMVAVEIFAILASSAGLLACIFFPKCYIIILRPDRNTRECLMGKGIG
>Coelacanth.locus272.1

MALVLCFLCSLTFIGRPRTWTCMLRQTTFGIIFALCVSCVLAKTIMVVIAFNATKPNSNLKKWLGPKLPNTIVVVCTVIQIIICIAWLTSS-
PFPIQNMKSQIGKIIIECNEGSALAFWCVLGYMGLLAIISFVVAFLARNLPDSFNEAKFITFSMLVFVSVWLAFIPAYLSTKGKYMVAVEIFAILASSSGLLACIFFPKCYIILLKPEMNTKDYLMGKGTG
>Coelacanth.locus269.1

MGAILAVISVVCAIIPAMTFWIFIKYRHTPIVKANNRELSYLLLLALVLCFLCSLVFIGEPSKWTCMLRQTAFGIIFALCVSCVLAKTIMVVIAFNATKPNSNLRKWVGPKLPNSIVLVCTVIQVIICIIWLSSSPPSPEQNMKSQIGK
IIIECNEGSAIAFWCVLGYMGLLAIVSFIVAFLARNLPDSFNEAKFITFSMLVFVSVWLAFIPAYLSTKGKYMVAVEIFSILASSAGLLACIFSPKCYIILLRPDKNTKEYLMAKGAN
>Coelacanth.locus276.1
-MKCPDDQWSNDRQDMCIPKTFEFLSYKDPMGETLSVLASFFAVIPITILCIFIKYRDTPIVKANNRELSYLLLLALVLCFLCSLIFIDKPRIWTCMARQAAFGIIFALCVSCVLAKTIMVVIAFNATKPNSNLKKWVGPKLASSIVLV
CTVIQVIICVAWLATFPPFPEYNMKSQIGIIIIECNQGSSTAFWCVLGYMGLLAIICFIVAFLARNLPGSFNEAKFITFSMLVFVSVWLAFIPAYLSTKGKYMVAVEIFAILASSAGLLACIFFPKCYIIVLRPDMNTKEYLMGKETF
>Coelacanth.locus270.1

MGATLAAFSIFCSIIPAKALCIFIKYRDTPIVKANNRELSFHLFVVLGVCFLCSLVFIGQPRIWTCILRQIAFGIIFALCVSCVLAKTIVVVIAFNATKPNSNLRKWVGPKLPNSIVFVCTVIQVIICIAWLSISPPFPEHNMKSQIGL
LIIECNEGSATAFWCMLGYMGLLAIISFIVSFLARNLPDSFNETKFIIFSMLFFVTVWLAFIPAYLSTRGKYMVAVEIFAILASSSGLLGCIFFPKRYIILLRPEMNTKEFLLAKEKN
>Coelacanth.locus271.1
-MKCPDDHWSNERQDTCIPKPTEFLSYEDSMGTTLAAISIFCAVISVTVLYIFITYRDTPIVKANNRELSYFLLLALVLCFLCSLIFIGKPQTWTCMLRQTAFGIIFALCVSCVLAKTIMVVIAFNATKPNSNLRKWVGTKVPNTVAFI
CTIIQVIICISWLAISPPFPEQNMTSQTGMIIIECNEGSTIAFWCVLGYMGLLAVISFVVAFLARNLPDSFNEAKFITFSMLVFVSVWLAFIPAYLSTRGKYMVAVEIFAILASGAGLLTCIFSPKCYVIILRPDMNTKEHLMGRGTP
>Coelacanth.locus208.1
CMKCPDDHWSNERQDECIPKTIEFLSYEEPMGATLAVISISCAAISLTVLCIFIKYQDTPIVKANNRELSYLLLLALVLSFLCSLIFIGKPRAITCMLRQTAFGIIFALCISCVLAKTIMVVVAFNATKPNSNFKKWVGPKVSNTMAMI
CTIIQVIICIAWLSCSPPFPEQNMKSQIGVIIIECNEGSTTAFWCVLGYMGLLAMISFIVAFAARNLPDSFNEAKFITFSMLIFVSVWLAFIPAYLSTWGKYMVTVEIFAILASSAGLLTCIFFPKCYIILLRPNMNTKEHLMGKHTF
>Coelacanth.locus388.1
-MKCPDDHWSNERQDECIPKPIEFLSYEEPMGAILAVISISCTAISLTVLCIFIKYQDTPIVKANNRELSYLLLVALMLSFLCSLIFIGKPRAVTCMLRQAAFGIIFALCVSCVLAKTIMVVIAFNATKPNSNLKKWMGPKVSNTTAMI
CTIIQVIICIVWLASSPPFPEQNMKSQIGVIIIECNEGSTTAFWCVLGYMGLLAIISFIVAFLARNLPDSFNEAKFITFSMLVFVSVWLAFIPAYLSTRGKYMVTVELFAILASSAGLLVCIFFPKCYIILLRPDMNTREHLMGKHAT
>Coelacanth.locus206.1
-MKCPDDHWSNEKQDECIPKTIEFLSYEEPMGATIAVISISCAAISLTVLCIFIKYQDTPIVKANNRELSYLLLLALMLSFLCSLIFIGKPRAVTCMLRQTAFGIIFALCVSCVLAKTIMVVIAFNATKPNSNLKKWVGPKVSNTMAMI
CTIIQVIICIAWLSSSPPFPEQNMKSQIGVIIIECNEGSTTAFWCVLGYMGLLAIISFIVAFLARNLPDSFNEAKFITFSMLVFVSVWLAFIPAYLSTKGKFMVAVEIFAILASSAGLLACIFIPKCYIILLRPHMNTRGYLLGKHTS
>Coelacanth.locus205.1
CLKCPDDHWSNERQDTCIPKNIEFLSSEDPLGATLIAISNCCASISVTVFCIFIKYRDTPIVKANNCELSYLLLLALVLSFLCSLIFIGEPRAVTCMLHQAAFGIIFALCVSCVLAKTILVVIAFNATKPNSKLKKWVGPTLPNTTVFV
STVFQVVICIAWLASSPPFPVRNMKSQTGMIIIECNEGSAIAFWCVLVYMGILAIVSFIVAFLARNLPDSFNEAQFITFSMLVFAVWLAFIPAYLSTWGKFMVAVEVFAILASSAGLLCCIFFPKCYIILLRPEINTRDYLMGKSTI
>Coelacanth.locus203.1
CLKCPDDHWSNERQDTCIPKNIEFLSFEEPLGTTLIAISISCASLAATVLCIFINYRDTPIVKANNREISYLLLLALVLSFLCSLIFIGEPRAVTCMLRQAAFGIVFALCVSCVLAKTIMVVIAFNATKPNSNLKKWVGPKLPNTMVFA
CSVIQVIICIAWLSSSPPFPVRNMKSQSELIIIECNEGSTIAFWCVLGYMGILAIVSFIVAFLARNLPDSFNEAQFITFSMLVFAVWLAFIPAYLSTRGKFMVAVEVFAILASSSGLLCCIFFPKCYVILLRPEMNTRDYLMGKSTI
>Coelacanth.locus201.1

MGATLLAISVSCASLSTTVLCIFVKYRDTPIVKANNRELSYLLLLALILSFFCSLIFIGEPTVITCMLRQAAFGIIFALCVSCVLAKTIMVVIAFNATKPNSHLKKWVGPKLPKGIVFLCTIIQVIICNTWLTSSPPFPERNIKSKVGM
IIVECNEGSTIAFWCVLGYMGILAIVSFIVAFLARNLPGSFNEAKFMTFSMLVFVSVWLAFIPAYLSTRGKYMVAVEIFAILASSAGLLACIFFPKCYIILMRPEMNTKEYLMGKATV
>Coelacanth.locus200.1
CLKCPDDHWSNEKQDKCIPKTIEFLSFEEPMGAALLAITISCASITAIVLCIFVKYRDTPIVKANNRELSYLLLLAIMLSFLCSLIFIGEPRTETCMLRQSAFGIIFALCVSCVLAKTIMVVIAFNATKPNSKLKKWVGPKLSRCIVLA
GTLIQFIICIVWLTSSPPFPEQNMKSQTGMIIIQCNEASTIAFWFVLGYMGILAIVSFIVAFLARNLPDSFNDAKFITFSMLVFVTVWLVFIPAYLSTRGKYMVAVEIFAILASSAGLLACIFFPKCYIILLRPEENTKEHLIGKNKI
>Coelacanth.locus278.1
CLKCPDDHWSNEKRDTCIKKITEFLSYKESLGAALAAVSILCATVPAMVLCIFIKYRETPIVKANNRELSYCLLSALMLGFLCSLLFIGKPRRATCILRQTAFGVIFAFSVSCVLAKTIMVIIAFKATKPNSNLRKWVGPKLPNGIAFA
CTVIQVIICITWLAKSPPFPIENMKSHVGVIIIECNEGSTTTFWCVLGYMGLLAIVSFVVAFLARNLPDSFNETKFITFSMLVFVSVWLAFIPAYLSTRGKYMVAVEIFAILASSAGLLACIFFPKCYIILLRPDMNTREYLMGKGSH
>Coelacanth.locus277.1
CLKRSDDHWSDEKRDTCLKKITKFLSYKESLGAALAAVSILCATIPAMVLCIFVQYQETPIVKANNYELSYCLLSALMLHFPCSLLFIGKPR-
TKCILCQTAFGIIFAFSVSYVLAKTIMVIIAFKATKPNSNLMKWVGPKLPNSIVLACTVIQVVICVIWLAKSSPFPIENMKSHVGVIILECNEGSTTAFWCVLGYMGLLAIVSFIVAFLARNLLDNFNETKFITFNMLVFVSVWLAFIP
AYLSTRGLYMVAVEIFTILASSSYLLTH---------------------------
>Coelacanth.locus131.1

CLKCPDDHWSNEKQDACIEKTIEFLSYQESLGAALATISIFCATIPAVVLAIFIKYQETPIVKANNRELSYFLLSALMLGFLCSLMFIGKPRRATCILRQTAFGVIFAFSVSCVLAKTIMVVIAFKATKPNSNLRKWVGPKLPNSIVLA
CTVIQVVICVAWLVGSPPFPIDNMKSQVGVIIIECSEGSPIAFWCVLGYMGLLAIISFIVAFLARNLPDSFNEAKFITFSMLVFVSVWLAFIPAYLSTRGKYMVAVEIFAILASSAGPLVCIFFPKCYIILLKPDMNTKDYLMGKGSD
>Coelacanth.locus349.1
CLKCTDDEWSNEGQDKCIPKLIDYLSYEEPMGIILTVISISFTFFPFMILCLFIKYRDTPLVKANNREISYLLLVGLMLSFLCSLLFIGQPWSVNCMMRQTAFGIIFALCVSCVLAKTIMVVIAFNATKPNSKLKKWVGPKLPISIVFF
CTLIQIIICIAWLSSAPPFQEKDMKSEIGMIIIKCNEGSTTAFWCMLGYMGLLAIVSFIVAFLARSLPDSFNEAKFITFSMLVFVSVWLAFIPAYLSTQGKYMVAVEIFAILASSASLLACLFSPKCYIILLKPDKNTKEYLLGKGTK
>Coelacanth.locus347.1

MGTTLAVISISCALFPAVILHMCIKHRHTPLVKANNRELSYLLLFALVLAFLCSLIFIGQPRTWTCMMRQTAFGIIFALCVSCVLAKTIMVVIAFNATKPNSNLKKWVGSRLPNSIVFVCTVIQVIICITWLSSYPPFPEQDTKSQIGQ
IIIQCNEGSSTAFWCVLGYMGFLVIISFIVAFLARNLPDSFNEAKFITFSMLIFVSVWLAFIPAYLSTKGMYMVAVEIVAILASSIGLLARIFFPKCYIILLRPDMNTKEYLLGKGTF
>Coelacanth.locus136.1
--MCPDDHWSNLERDKCIPKTVEFLSYEDPMGVALTAIAIISSLLPAAIPCVFIKYRDTPVVKANNRELSYLLLLALVLCFLCSVLLIGHPTVVTCMFRQIAFGIIFTLSVSCVLAKTIMVVIAFNATKPNSNLKKW----
LPNTVALVCTLIQVIICTTWLCSSPPFPVQNMKSQIGTIIIECNDGSTTTFWYVLGYMGLLAIVSFIVAFLARNLPDSFNEAKFITFSMLVFVSVWLAFIPAYLSTQGKYMVAVEIFAILASSAGLLGCIFFPKRYIILLQPHMNTKEY
LMGKANK
>Coelacanth.locus209.1
--
MCPDDHWSNERRDECILKATEFLSYRDPMGATLAAISIFCALFPATILCIFIKYRDTPIVKANNRELSFLLLLALVLCFLCSLLFIGLPKTITCMFRQVAFGIIFALCVSCVLAKTIMVVIAFHATIPSSKLKRWVGPKLPNTIVFICT
VIQVIICTIWLYNSPPFPVQNMKSQIGMIIIECNDGSTIAFWLVLGYMGLLATISFVVAFLARNLPDSFNEAKFITFSKLVFVSVWLVFIPAYLSTKGKYVVAVEIFAILASSIGLLGCIFFPKCYIILLKPDMNTREYLMGKGTF
>Coelacanth.locus132.1

MGATLATISISIALTPVTILLLFIKHRDTAIVKANNRELSYLLLLALVLGFLCSLIFIGHPSTWTCVLRQVAFGIIFALCVSCVLAKTIMVVIAFNATKPNSNLKKWVGAKLPNSIVFVCTVIQVIICIAWLSSSPPFPEHNMQSQIGM
IIIECNQGSSTAFWCVLGYMGLLAIISFIVAFLARNLPDSFNEAKFITFSMLVFVSVWLAFIPAYLSTQGKYMVAVEIFAILASSAGLLGCIFFPKCYIILLRPDINTKEYLMGKGAF
>Coelacanth.locus375.1

MGVALAAISIFCALVPAFGLGTFIKFRDTPIVKANNRELSYLLLVALVLCFLCSLIFIGRPRKVTCMLRQVAFGIIFVLCVSCVLAKTIMVVIAFNATKPNSNLRKWVGPKLPNTIVFLCTIIQVVICIAWLTSSPPFPEQNMKSQIGM
IIIECNEGSVTAFWCVLGYMGLLAIISFIVAFLARKLPDSFNEAKFISFSMLVFVTVWLSFIPAYVSTRGKYMVAVEIFAILASSAGLLGCIFFPKCYIILLKPHMNTKEYLMGKGTY
>Coelacanth.locus301.1

MGVTLASISIFSALIPVTILYIFIKNRNTPLVKANNRELSYLLLLALMLCFLCSLNFIGNPSRVACMLRQVAFGIVFALCVSCVLAKTIMVVIAFNATKPNSNLKKWVGPKLPNAIVFVCTVIQVIICIVWLSISPPFPEHNMKSQIGV
IIKECNEGSTTAFWCMLGYMGLLAIVSFVVAFLAKNLPDSFNEAKFITFSMIVFVTVWLSFIPAFLSTRGKYMVAVEIFAILASTAGLLACIFLPKCYIILLRPHINTREYLMGKGSK
>Coelacanth.locus351.1

MGATLAAISLFAALITAATLCTFIKYQDTPIVKANNRELSYLLLLALMLCFLCSLIFIGYPMRVTCMLRQVAFGIIFTLSVSCVLAKTFMVIIAFNATNPNSNLRKWIGPKLPIAIVCVGTLLQVIICIVWLSSSPPFPEQNIKSQVAK
IIIECNEGSVIAFWCMLGYMGLLAIISFIVAFLARRLPGSFNEAQFITFSMLVFVTVWLGFIPAYLSTRGKYMAAVEIFAILASSAGLLACIFFPKCYIIILKPDMNTKEYLMGKESS
>Coelacanth.locus262.1
CLKCLDDYWANEAQDRCIPKSHEFLSFEDPMGATLTSVSIFGALITAATLCIFIKYRDTPIVKANNRELSYLLLLALMLCFLCSLIFIGYPMRVTCMLRQAAFGNVFTLSVSCVLAKTFMVIIAFNATNPNSNLRKWIGPKLPIAIVCV
GTLLQVIICIVWLSSSPPFPEQNKKSQVEKIIIECNEGSDLAFWCMLGYTGLLAIISFIVAFLARRLPGSFNEAQFITFSMLVFVTVWLGFIPAYLSTRGKYMVAVEIFAILASSAGLLACIFFPKCYIILLKADMNTKEYLMGKVSS
>Coelacanth.locus141.1

MGATLTSVSISVALITATTLYIFIKYRDTPIVKANSRELSYLLLLALMLCFLCSLIFIGYPMRVTCMLRQAAFGIVFTLSVSCVLAKTFMVIIAFNATNPNSNLRKWIGPKLPITIVCVGTLLQVIICIAWLSSSPPFPEQDMKSQVGK
IIIECNEGSVLAFWCMLGYMGLLAIISFIVAFLARKLPGSFNEAQFITFSMLVFVTVWLAFIPAYLSTRGKYMAAVEIFAILASSAGLLACIFFPKCYIIILKPHMNTKEYMMGKESS
>Coelacanth.locus374.1

MGATLTSISIFAALITAATLCIFIKYRDTPIVKANNRELSYLLLLALTLCFLCSLIFIGYPMRVTCMLRKAAFGIVFTLSVSCVLAKTFMVIIAFNATNPNSSLRKWIGPKLPIAIVCVGTLLQVIICIAWLSSSPPFPEQNMKSQVGK
IIIECNEGSVIAFWCMLGYMGLLAIISFIVAFLARKLPGSFNEAQFITFSMLVFVTVWLAFIPAYLSTRGKYMVAVEIFAILASSAGLLACIFFPKCYIILLQPDMNTKEYLMGKSAF
>Coelacanth.locus145.1
CQKCPDDYWSNEAQDRCIPKSIDFLSYEDPMGATLTSISIFAALITAATLCIFIKYRDTPIVKANNRELSYLLLLALMFCFLCSLIFIGYPMRVTCMLRQVAFGIIFTLSVSCVLAKTFMVIIAFNATNPNSRLRKWIGPKLPIAIVCV
GTLLQVILCIARLSSFPPFPEQNMKSQVGKIIIECNEGSNTAFWCMLGYMGLLALVCFTVAFFARKLPSSFNEAQFITFSMLVFVTVWLSFIPAYLSTKGKYMVVVEIFAILASSAGLLACIFFPKCYIILRRPDMNTKEYLMGKGAF
>Coelacanth.locus147.1
CNQCPDDHWSNEARDECIPKSIDFLSYEDPMGATLTSISIFATLITAATLYIFNKYQDTPIVKANNRELSYLLLLALMLCFLCSLIFIGYPTRVTCLLRQVAFGIVFTLSVSCVLAKTFMVIIAFSATNPNSDLRKWIGPKLPIAIVCV
GTLLQVVICIVWLSSSPPFPEQDMKSQVGKIIIECNAGSDIAFWCMLGYMGLLALVCFTVAFFARKLPGSFNDAQFITFSMLVFVTVWLAFIPAYLSTKGKYMVVVEIFAILASSAGLLVCIFFPKCYIILLRPDMNTKEYLMGKNAF
>Coelacanth.locus380.1
CLPCPDDHWSNEERDKCIPKSIEFLSYEDPMGATLTSVSVSAVLITAATFCIFIKYQDTPIVKANNRELSYLLLLALMLCFLCSLIFIGYPMRVTCMLRQAAFGIVFTLSVSCVLAKTFMVIIAFNATNPNSSLRKWIGPKLPITIVCV
GTLLQVIICIVWLSSSPPFPEQNMKSQVGKIIIECNVGSGIAFWCMLGYMGLLASISFIVAFFARKLPDRFNEAQFITFSMLVFISVWLAFIPAYLSTKGKYMVAVEIFAILASSTGLLASIFFSKCYIILLRPDMNTKVHLMGKGTF
>Coelacanth.locus341.1
CLKCPDDHWSNDRRDRCIPKPIEFLSYEEPLGATLAAISTFSSLLPAVILLIFIKYRETPIVKANNRGLSYLLLLALTLCFLCSLVFIGYPIKVTCMLRQTAFGIVFALGVSCVLAKTMMVVIAFHATKPNSNLKKWVGPKLPNGIVFV
CTVIQITICITWLSHSSPFPEKNMKSQIGVIIIECNEGSTTAFWCMLGYMGLLAIVSFIVAFLARNLPDSFNEAKLITFSMLFFLSVWLAFIPAYLSAQGKYMVAVEIFAILASSAGLMACIFFPKCYIILLHPDRNTRYYVNEKNNT
>Coelacanth.locus353.1
CLKCPDDHWSNDKRNKCIPKTIEFLSYEEPLGATLAAISTFSSLLPAAILFIFIKYQETPIVKANNRGLSYLLLLALMLCFLCSLIFIGYPTRITCMLRQSAFGIVFALCVSCVLAKTIMVVIAFNAMKPNSKLKKLVGPKLSNSIVLV
CTVIQVIICIAWLSSVPPFPVQNMKSQIGVIIIECNEGSAIAFWCMLGYMGLLAIVSFIVAVLARNLPDSFNEAKFITFSMLVFVSVWLAFIPAYLSARGKYMVAVEIFAILASSTGLMACLFFPKCYVILLHPDMNTREHLMGKGTF
>Coelacanth.locus302.1
CLKCPSDHWSSNGRDKCIPKTIEFLSYQEPLGATLASVSILFSLIPAAILCTFVKHRDTPIVKANNRELSYLLLLALMLCFLCSLIFIGQPKISTCMLRQVAFGIIFALCVSCVLAKTIMVVIAFNATKPNSKLKKWVGPKLPNAVVFV
CTVIQVIICIAWLSSSPPFPEQNMRSQIQKIIIECNDGSTTAFWLVLGYMGILAIVCFIVAFLARNLPDSFNEAKFITFSMLVFVTVWLSFIPAYLSTKGKYMVAVEIFAILASSTGLMACIFSPKCYIIILRPERNTREYLMGKKNG
>Caecillan.locus36976.1
CWKCPENYWPDERKQKCIEKSVEFLSYTEPLGIILAVTSVVTAVIPAVILVIFIKYRHTHIVKANNWQLSFLLLVSLVFCCLCSLIFIGHPRDVTCLVRQVAFGIIFALCISCVLAKTIMVVIAFKATKPDSSLKYWVRPQLSNAIVIT
STSIQVIICILWLSISPPFLHKNKNFQIGSIILECNEGSATAFWCMLGYIGFLASLSLVVAFLTRKLPDSFNEAKFITFSMLVFVSVWLSFIPAYLSTRGKQMVAVEVFAILSSSTGLMTCIFLPKCHIILFRPDMNTKEYLLRRTVG
>Caecillan.locus36981.1
CWKCPENYWPDERKQKCIEKSVEFLSYTEPLGIILAVTSVVTAVIPAVILVIFIKYRHTHIVKANNWQLSFLLLVSLVFCCLCSLIFIGHPRDVTCLVRQVAFGIIFALCISCVLAKTIMVVIAFKATKPDSSLKYWVRPQLSNAIVIT
STSIQVIICILWLSISPPFLHKNKNFQIGSIILECNEGSATAFWCMLGYIGFLASLSLVVAFLTRKLPDSFNEAKFITFSMLVFVSVWLSFIPAYLSTRGKQMVAVEVFAILSSSTGLMTCIFLPKCHIILFRPDMNTKEYLLRRTVG
>Caecillan.locus36974.1
CWKCPEDYWPDETKQKCIEKSIEFLSYTEPLGTVLAVTSVVTAVIPAVILVIFIKYRHTPLVKANNWQLSFLLLVSLVFCCLCSLIFIGHPRDVTCLVRQVAFGIIFALCISCVLAKTIMVVIAFKATKPDSSLKHWVGPQLSNSIVIT
STSIQVIICILWLSISPPFLHKNKNFQIGSIILECNEGSATAFWCMLGYIGFLASLSLVVAFLSRKLPDSFNEAKFITFSMLVFVSVWLSFIPAYLSTRGKQMVAVEVFAILSSSTGLMACIFLPKCHIILFRPDMNTKEYLLRRTVG
>Sterlet.locus438.1
CLRCPSDQWSNTRQDACVPRLVEFLSYEEPLGASLASLAVLASLLPASVLGVFLRHRHSPIVKANNRQLSYVLLGALALCPLCSLIFIGRPSPASCMLRQTAFAIVFTLGVSCVLAKTLMVVIAFKASIPGSGLRRWVGSRLPNTVASV
GTLVQVGICGAWLGLASPYPENNTTSHVARIVLECNEGSALAFWCMLGYMGVLASVSFVVAFLARRLPDSFNEAKFITFSMLVFVSVWLSFIPAYMSTGGKYMVAVEIFAILSSSMGLLACIFFPKCYVILLRPERNTRDHLMGKGAA
>Sterlet.locus416.1
CLRCPSDQWSNTRQDACVPRLVEFLSYEEPLGASLASLAVLASLLPASILGVFLRHRHSPIVKANNRQLSYVLLGALALCPLCSLIFIGRPSPASCMLRQTAFAIVFTLGVSCVLAKTLMVVIAFKASIPGSGLRRWVGSRLPNTVASV
GTLVQVGICGAWLGLASPYPENNTTSHVARIVLECNEGSALAFWCMLGYMGVLASVSFVVAFLARRLPDSFNEAKFITFSMLVFVSVWLSFIPAYMSTGGKYMVAVEIFAILSSSMGLLACIFFPKCYVILLRPERNTRDHLMGKGAA
>Sterlet.locus410.1

XLPASTLALFLWHHHTPIVKANNRQLSYVLLCALALCPLCSLIFIGQPSPASCMLRQTAFGIIFALSVSCVLAKTVLVVIAFKASIPGSSLHRWVGTRLPNSIACLGTLIQVGICGTWLGAAPPFPENNIKSQIGKVILECNEGSALAF
WCMLGYMGILASVSFVVAFLSRNLPDSFNEAKFITFSMLVFVSVWLSFIPAYLSTKGKYMVSVEIFAILSSSAGLQGCIFFPKCYIILLRPERNTRDYLMGKGPT
>Reedfish.locus56974.1

MVEFLSYSEPLGIVLAVLSIVISVVPKSILGLFLWYRHTPIVKANNRQLSYVLLCALTLCPLCSLIFIGKPSSASCILRQTMFAIIFALSVSCVLAKTIMVIIAFKASKPGSNLHRWVGSRLPNTIACLGTLIQAGICALWLGYAPPFP
QNNMNSQVGKIILECNEGSSLAFWCMMGYMGILAIVSFVIAFLARKLPNSFNEAKFITFSMLVFVIVWLSFIPSYLSTRGKYTVAVEIFAILSSSAGLLGCIFFPKCYVILLKPERNTRDFLMGKGSS
>Reedfish.locus56973.1

MVEFLSFDEPLGIVLCIMSMLISLVPKSILGLFIWYRHTPIVKANNRQLSYVLLGALTLCPLCSFIFIGKPSLASCILRQTTFGIIFALSVSCVLAKTVMVIIAFKASKPGSNLRKWVGSRLPNAVACLGTLIQAGICTVWLGYAPPFP
QNNMNSQVGKIILECNEGSSLAFWCMMGYMGVLAIISFMMAFLARKLPDSFNEAKFITFSMLVFVSVWLSFIPAYLSTRGKYMVAVEIFAILSSSAGLLACIFFPKCYIILLKPDRNTRDFLMGKGDP
>Reedfish.locus56889.1
CLRCPDDHWSNKRRNACIPKVVEFLSYEEPLGTTLASLSVLTSTVPSLILCVFMWHGDTPIVKANNRQLSYVLLCALTLCPLCSLIFIGKPSPASCLLRQTTFGIIFTLSVSCVLAKTIIVVIAFKASKPGSKLHKWVGSKLPNTIIFF
TTLIQVGICSVWLLCAPPFPETNMKAQVGKIILECNEGSTLALWCMMGYMGILASISFLVAFLARKLPDSFNETKYITFSMLLFVSVWLSFVPAYLSTRGKYMVAVEIFAILSSSTGLLGCFFFPKCYIILIKPEKNTREFIMRKRSS
>Western_clawed_frog.locus416.1
CFPCTDETWPNEGRTECLPKPVDFLSFEEPLGITITCTTIFSYISTVTILCVFIRYKDTAIVKANNRDLTYVLLGSLSLSFLCPLLFIGQPHRFTCLFRQVTFGVIFVLSVSCVLAKTIMVVIAFRATKPGSNMRRWLGPSVPGLKVSA
CTNLQLFICIFWMLYCPPFPERNTRIKIGVIVFQCNECSDTLLWCMVGCMAFLSCVCFLVAFLARKLPDTFNEAKWITFSMLIFLSVWIAFAPAYLSTQGKYMAAVEVFGIICSSAGILVCIFLPKCYIILLRPDLNTRGNILSNRST
>Zebrafish.locus509.1
CHKCQPDYWPNAEKIKCLPKPVEFLSWDEILGNTLAAFSIAGSLVALSMALVFYKNRVSPIVRANNSELSFLLLFSLTLCFLCSLTFIGQPTEWSCMLRHTAFGITFVLCISCVLGKTIVVLMAFKATLPGSNVMKWFGPPQQRLSVFG
FTLVQIIICVLWLTISPPFPYKNMQHYKDKIILECSLGSAVGFWAVLGYIGLLAFLCFVLAFLARKLPDNFNEAKFITFSMLIFCAVWITFVPAYVSSPGKFTVAVEIFAILASSFSLILLIFAPKCFVIVFRPEENTKRHLLGKVPP
>marker.zebrafish_4_1_F

CQPDYWPNAEKIKCLPKPVEFLSWDEILGNTLAAFSIAGSLVALSMALVFYKNRVSPIVRANNSELSFLLLFSLTLCFLCSLTFIGQPTEWSCMLRHTAFGITFVLCISCVLGKTIVVLMAFKATLPGSNVMKWFGPPQQRLSVFGFTL
VQIIICVLWLTISPPFPYKNMQHYKDKIILECSLGSAVGFWAVLGYIGLLAFLCFVLAFLARKLPDNFNEAKFITFSMLIFCAVWITFVPAYVSSPGKFTVAVEIFAILASSFSLILLIFAPKCFVIVFRPEENTKRHLLGKV--
>Zebrafish.locus848.1
CHKCQPDYWPNAEKIKCLPKPVEFLSWDEILGNTLAAFSIAGSLVALSMALVFYKNRASPIVRANNSELSFLLLFSLTLCFLCSLTFIGQPTEWSCMLRHTAFGITFVLCISCVLGKTIVVLMAFKATLPGSNVMKWFGPPQQRLSVFG
FTLVQIIICVLWLTISPPFPYKNMQHYKDKIILECSLGSAVGFWAVLGYIGLLAFLCFVLAFLARKLPDNFNEAKFITFSMLIFCAVWITFVPAYVSSPGKFTVAVEIFAILASSFSLILLIFAPKCFVIVFRPEENTKRHLLGKVPP
>Zebrafish.locus852.1
CHECLSDYWPNNEKNKCLRKPVEFLSWDEILGIILAAFSVAGSLVALSMALVFYKNRASPIVRANNSELSFLLLFSLTLCFLCSLTFIGQPTEWSCMLRHTAFGITFVLCISCVLGKTIVVLMAFKATLPGSNVMKWFGPSQQRLSVFG
FTLVQIIICVLWLTISPPFPYKNMQHYKDKIILECSLGSAVGFWAVLGYIGLLAFLCFVLAFLARKLPDNFNEAKFITFSMLIFCAVWITFVPAYVSSPGKFTVAVEIFAILASSFGLILCIFAPKFFIIIFRPERNTKKHIMGKVPS
>Zebrafish.locus513.1
CHECLSDYWPNNEKNKCLRKPVEFLSWDEILGIILAAFSVAGSLVALSMALVFYKNRASPIVRANNSELSFLLLFSLTLCFLCSLTFIGQPTEWSCMLRHTAFGITFVLCISCVLGKTIVVLMAFKATLPGSNVMKWFGPSQQRLSVFG
FTLVQIIICVLWLTISPPFPYKNMQHYKDKIILECSLGSAVGFWAVLGYIGLLAFLCFVLAFLARKLPDNFNEAKFITFSMLIFCAVWITFVPAYVSSPGKFTVAVEIFAILASSFGLILCIFAPKFFIIIFRPERNTKKHIMGKVPS
>Zebrafish.locus850.1
CDKCPPDYWPSTEKDKCLPKPVEFLSWDEILGIILAVLSVSGSLLALSMALVFYKNRASPIVRANNSELSFLLLFSLTLCFLCSLTFIGQPTEWSCMLRHTAFGITFVLCISCVLGKTIVVLMAFKATLPGSNVMKWFGPSQQRLSVFG
FTLVQIIICVLWLTISPPFPYKNMQHYKDKIILECSLGSAVGFWVVLGYIGLLASLCFVLAFLARKLPDNFNEAKFITFSMLIFSAVWITFIPAYVSSPGKFTVAVEIFAILASSFSLILCIFAPKFFIILFRPEENTKKHLMGKVQS
>Zebrafish.locus511.1
CDKCPPDYWPSTEKDKCLPKPVEFLSWDEILGIILAVLSVSGSLLALSMALVFYKNRASPIVRANNSELSFLLLFSLTLCFLCSLTFIGQPTEWSCMLRHTAFGITFVLCISCVLGKTIVVLMAFKATLPGSNVMKWFGPPQQRLSVFG
FTLVQIIICVLWLTISPPFPYKNMQHYKDKIILECSLGSAVGFWVVLGYIGLLASLCFVLAFLARKLPDNFNEAKFITFSMLIFSAVWITFIPAYVSSPGKFTVAVEIFAILASSFSLILCIFAPKFFIILFRPEENTKKHLMGKVQS
>marker.cichlid_4-10aa

CHTEFWPNAKRDTCIPKPVEYLSFQGILGIILATFSVVGAFLAIITAAVFFHHRTSPIVRANNSELSFLLLISLTLCFLCSLTFIGAPSEWSCMLRHTAFGITFVLCISCVLGKTIVVLMAFKATLPGSNVMKWFGPPQQRMTVVSFTF
IQVLICTIWLVVSPPFPIKNLTTYKEKIILECALGSAVGFWAVLGYIGILAAFCFVLAVLARKLPDNFNEAKLITFSMLIFCAVWITFIPAYVSSPGKFTVVVEIFAILASSFGLIMCIFAPKCFIILFKPEKNTKKYLMNKN--
>marker.medaka_4_1_F

CPIEFWPNPEKDSCHPKPVEFLSIDETLTIILAVVSVGGACLSILTAAVFFHHRTTPIVRANNSELSFLLLFSLTLCFLCSLTFIGAPTEWSCMLRHSAFGITFVLCISCVLGKTIVVLMAFKATLPGSNVIKWFGPLQQRLTVVFFTF
VQVLICIVWLVLRPPFPMKNFSTYKERIILECALGSAIGFWAVLGYIGLLAVFCFVLAVLARKLPDNFNEAKLITFSMLIFCAVWLSFIPAYVSSPGKFTVAVEIFAILASSFGLIMCIFAPKCFIILCQPEKNTKKYLMNKN--
>Japanese_eel.locus101.1
CITCPADYWSNVEKDECLPKPIEFLSFHEVLGIILAAFSVTGACLAIIVAAVFYRHRDTPIVKANNSEMSFLLLFSLKLCFLCSLTFIGRPSEWSCMLRHTAFGITFVLCFSCVLGKTIVVLIAFRATLPGSNVMKWFGPLQQRLSVLA
FTLIQVLICVLWLTISPPFPYKNMKHYKEKIILECDAGSAVGFWAVLGYIGLLSVLCFVLAFLARKLPDNFNEAKFITFSMLIFCAVWTTFIPAYVSSPGKFTVAVEIFAILASSFSLIVCIFVPKCFIILFWPEQNTKKYMMGKMPS
>Japanese_eel.locus223.1
CITCPADYWSNAEKDECLPKPVEFLSFHEILGIILAACSVTGACLAIMVAAVFYRHRDTPIVKANNSELSFLLLFSLKLCFLCSLTFIGRPSEWSCMLRHTAFGITFVLCISCVLGKTIVVLMAFRATLPGSNVMKWFGPLQQRLSVLA
FTLIQVLICVLWLTISPPFPYKNMKHYKEKIILECDVGSAVGFWAVLGYIGLLSVLCFALAFLARKLPDNFNEAKFITFSMLIFCAVWITFIPAYVSSPGKFTVAVEIFAILASSFGLIICIFVPKCFIILFRPEQNTKKHMMGKMPS
>Japanese_eel.locus217.1
CITCPADYWSNAEKDECLPKPVEFLSFHEVLGIILAACSVTGACLAIMVAAVFYRHRDTPIVKANNSELSFLLLFSLKLCFLCSLTFIGQPSEWSCMLRHTAFGITFVLCISCVLGKTIVVLMAFRATLPGSNMMKWFGPSQQRLSVLA
FTLIQVLICVLWLTISAPFPYKNTKDYKEKIILECDVGSAVGFWAVLGYIGLLSILCFGFAFLARKLPDNFNEAKFITFSMLIFCAVWITFIPAYVSSPGKFTV--
>Japanese_eel.locus99.1
CITCPADYWSNAEKDECLPKPVEFLSFHEVLGIILAACSVTGACLAIMVASVFYRHKDTPIVKANNSELSFLLLFSLKLCFLCSLTFIGQPSEWSCMLRHTAFGITFVLCISCVLGKTIVVLMAFRATLPGSNVMKWFGPSQQRLSVFA
FTLIQVLICVLWLTISPPFPYKNMKHYKEKIILECDVGSAVGFWAVLGYIGLLSILCFGFAFLARKLPDNFNEAKFITFSMLIFCAVWITFIPAYVSSPGKFTVAVEIFAILASSFSLIVCIFLPKCFIILFQPEQNTKKHMMGKIPS
>Japanese_eel.locus106.1
CITCPAESWSNAEKDECVPKPVEFLSFHEVLGIILAACSVTGACLAIMVAAVFYRHRDTPIVKANNSELSFLLLFSLKLCFLCSLTFIGRPSEWSCMLRHTAFGITFVLCISCVLGKTIVVLMAFRATLPGSNVMKWFGPSQQRLSVLA
FTLIQVLICVLWLTISPPFPYKNMKHYKEKIILECDVGSAVGFWAVLGYIGLLSILCFGFAFLARKLPDNFNEAKFITFSMLIFCAVWITFIPAYVSSPGKFTVAVEIFAILASSFSLIVCIFVPKCFIILFRPEQNTKKHMMGKMP-
>Japanese_eel.locus108.1
CITCPADYWSNAEKDKCVPKPVEFLSFHEVLGIILAACSVTGACLAIMVAAVFYRHRDTPIVKANNSELSFLLLFSLKLCFLCSLTFIGRPSVWSCMLRHTAFGITFVLCISCVLGKTIVVLMAFRATLPGSNVMKWFGPLQQRLSVLA
FTLIQVLICVLWLTISPPFPNKNMMHYKEKIILECDVGSAVGFWAVLGYIGLLSILCFGFAFLARKLPDNFNEAKFITFSMLIFCAVWITFIPAYVSSPGKFTVAVEIFAILASSFSLIVCIFVPKCFIILFRPEQNTKKHMMGKMPS
>Japanese_eel.locus219.1
CITCPADYWSNAENDECVPKPVEFLSFHEVLGIILAACSVTGACLAIMVAAVFYRHRDTPIVKANNSELSFLLLFSLKLCFLCSLTFIGRPSEWSCMLRHTAFGITFVLCISCVLGKTIVVLMAFRATLPGSKVMKWFGPSQQRLSVLA
FTLIQVLICVLWLRISPPFPNKNMKHYKEKIILECDVGSAVGFWAVLGYIGLLSILCFGFAFLARKLPDNFNEAKFITFSMLIFCAVWITFIPAYVSSPGKFTVAVEIFAILASSFGLIVCIFVPKCFIILFRPEQNTKKHMMGKMPS
>Japanese_eel.locus110.1
CITCPTDYWTNAEKDKCVPKPVEFLSFHEVLGIILAACSVTGACMAITVAAVFYRHRDTPIVKANNSELSFLLLFSLKLCFLCSLTFIGRPSEWSCMLRHSAFGITFVLCISCVLGKTVVVLMAFRATLPGRNIMKWFGPPQQRLSVLA
FTLIQVLICVLWLTISPPFPNKNMKHYKEKIILECDVGSAVGFWAVLGYIGLLSILCFGLAFLARKLPDNFNEANFITFSMLIFCAVWITFIPAYVSSPGKFTVAVEIFAILASSFGLIVCIFVPKCFIILFRPEQNTKKHMMGKMPS
>Japanese_eel.locus256.1

MILAACSVTGACMAITVAAVFYRHRDTPVVKANNSELSFLLLFSLKLCFLCSLTFIGQPSDWSCMLRHTAFGITFVLCISCVLGKTIVVLMAFRATLPGSNVMKWFGPPQQRLSVLLFTLVQAVICTLWLILSPPFPLRNMKHSKERII
LECDLGSESAFWAVLGYIGLLSILCFVLAFLARKLPDNFNEAKFITFSMLIFCAVWITFIPAYVSSPGKFTVAVEIFAILASSFGLITCIFVPKCFIILFRPEQNTRKHMMGKMPS
>Japanese_eel.locus236.1
CITCPADYWTNAERDECVPKPVEFLSFHEVLGIILAACSVTGACMAIMVAAVFYIHRDTPIVKANNSELSFLLVFSLKLCFLCSLTFIGQPSEWSCMLRHTAFGITFVLCISCVLGKTIVVLMAFRATLPGSNVMKLFGPPQQRLSVLA
FTLIQVLICVLWLTISPPFPSKNMIHYKEKIILECDVGSTVGFWAVLGYIGLLSILCFGFAFLARKLPDNFNEAKFITFSMLIFCAVWITFIPAYVSSPGKFTVAVEIFAILASSFGLIVCIFVPKCFIILFQPEKNTKKHMMGKMPS
>Japanese_eel.locus234.1
CITCAADYWSNAKKDECVPKPVEFLSFHEVLGIILTACSVTGACIAITVAAVFYRHRDTPIVKANNSELSFLLVFSLKLCFLCSLTFIGRPSEWSCMLRHTAFGITFVLCISCVLGKTIVVLMAFKATLPGCNVMKWFGPPQQRLSVLA
FTLIQVLICVLWLTISPPFPNENMIHYKEKIILECDVGSAVGFWAVLGYIGLLSILCFVLAFLARKLPDNFNEAKFITFSMLIFCAVWITFIPAYVSSPGKFTVAVEIFAILASSFGLIICIFVPKCFIILFQPEKNTKKHMMGKIPS
>Japanese_eel.locus250.1
CITCPVDYWTNTEKDECVPKPVEFLSFHEVLGIILAACSVTGACMAITVAAVFYRHRDTPIVKANNSELIFLLVFSLKLCFLCSLTFIGRPSDWSCMLRHTAFGITFVLCISCVLGKTIVVLMAFRATLPGSNVMKWFGPLQQRLSVLA
FTLIQVLICVLWLTISPPFPSKNMIHYKEKIILECDVGSTVGFWAVLGYIGLLSILCFVLAFLGRKLPDNFNEAKFITFSMLIFCAVWITFIPAYVSSPGKFTVAVEIFAILASSFGLTVCIFVPKCFIILFRPEQNTKKHMMGKMPS
>Japanese_eel.locus247.1
CITCPVDYWTNAEKDECVPKPVEFLSFHEILGIILTGCSVTGACMAITVAAVFYRHRDTPIVKANNSELSFLLVFSLKLCFLCSLTFIGQPSEWSCMLRHTAFGITFVLCISCVLGKTIVVLMAFRATLPGSNVMKWFGPTQQRLSVLA
FTLIQVLICGLWLTISPPFPSKNMKHYKEKIILECDVGSAVGFWAVLGYIGLLSILCFALAFLARKLPDNFNEAKFITFSMLIFCTVWITFIPAYVSSPGKFTVAVEIFAILASSFGLIICIFVPKCFIILFRPEKNTKKHMMGKTMS
>Japanese_eel.locus238.1
CITCPADYWPNAEKDACVLKPVEFLSFHEVLGIILAACSVTGACMAFMVAVVFYRHRDTPIVKANNSELSFMLVFSLKLCFLCSLTFIGRPSDWSCMLRHTAFGIAFVLCISCVLGKTLVVLMAFRATLPGSNVMKWFGPLHQRMSVLA
FTLIQVLICVLWLTTCPPFPNKNMKHYKEKIILECDVGSAVGFWAVLGYIGLLSILCFILAFLARKLPDNFNEAKFITFSMLIFCAVWITFIPAYVSSPGKFTVAVEIFAILASSFGLIVCIFFPKCFIILFRPEQNTKKHMMGKMPS
>Japanese_eel.locus227.1
CITCPADYWTNAEKDECVPKPIEFLSFHDVLGIILAACSVTGACIAITVAAVFYRHRDTPIVKANNSELSFLLLFSLKLCFLCSLTFIGRPSEWSCMLRHTAFGITFVLCISCVLGKTIVVLMAFRATLPGSNVMKWFGPLQQRLSVLA
FTLIQVLICVLWLTISPPFPTKNMKHYKEKIILECDVGSAVGFWAVLGYIGLLSTLCFVLAFLARKLPDNFNEAKFITFSMLIFCAVWITFIPAYVSSPGKFTVAVEIFAILASSFGLIFCIFVPKCFIILFRPEQNTKKHMMGKMPS
>Japanese_eel.locus202.1

EEYWPNAKRNECVLKAVEFLSFEEIMGIMFVVFSIFGVCFTAFVAVLFFIKKDTPIVKANNSELSFLLLFSLKLCFLCSLTFIGRPSEWSCMLRHTAFGITFVLCISCVLGKTIVVLMAFRATLPGSNVMKWFGPPQQRLSVLAFTLIQ
VLICVLWLTISPPLPNKNTKHYKEKIILECEIGSAVGFWAVLGYIGLLSLLCFVLAFLARKLPDKFNEAKFITFSMLIFCAVWITFIPAYVSSPGKFTVAVEIFAILASSFGLLFCIFLPKCYIMLFRPEQNTRKHIMGKTDV
>Japanese_eel.locus191.1

EEFWPNAKRNECVVKAVEFLSFEEIMGILFVVFSIFGACFTAFVAVLFFIKKDTPIVKANNSELSFLLLFSLKLCFLCSLTFIGRPTEWSCMLRHTAFGITFVLCISCVLGKTIVVLMAFRATLPGSNVMKWFGPPQQRLSVLAFTLIQ
VLICVLWLTISPPLPNKNMKHYKEKIILECEIGSPVGFWAVLGYIGLLSILCFVLAFLARKLPDKFNEAKFITFSMLIFCAVWITFIPAYVSSPGKFTVAVEIFAILASSFGMLFCIFLPKCYIMLFRPEQNTRKHIMGKTDV
>Japanese_eel.locus404.1

MGIILVIFSIFGACITAFVAVLFFIKKDTPIVKANNSELSFLLLFSLKLCFLCSLTFIGRPSEWSCMLRHTAFGITFVLCISCVLGKTIVVLMAFRATLPGSNIMKWFGPSQQRLSVLAFTLIQVLICVLWLTISPPFPNKNMKHYKEK
IILECNVGSAVGFWAVLGYIGLLSILCFVLAFLARNLPDNFNEAKFITFSMLIFCAVWITFIPGYVSSPGKFTVAVEIFAILASSFGLLFCIFLPKCYIILFKPEQNTRKHIMGKTDM
>Japanese_eel.locus189.1

MGIVLVIFSIFGACVTSFVAVWFFIKKDTPIVKSNNSELSFLLLFSLKLCFLCSLTFIGRPSEWSCMLRHTAFGITFVLCISCVLGKTIVVLMAFRATLPGSNVMKWFGPPQQRLSVLAFTLIQVLICVLWLTISPPFPNKNMKHYKEK
IILECDVGSAVGFWAVLGYIGLLSILCFVLAFLARKLPDNFNEAKYITFSMLIFCAVWITFIPAYVSSPGKFTVAVEIFAILASSFGLLFCIFLPKCYIILFRPEQNTRKHIMGKTDM
>Japanese_eel.locus199.1

MGIVLVLFSIFGACVTAFVAVWFYIKKDTPIVKANNSELSFLLLFSLKLCFLCSLTFIGRPSEWSCMLRHTAFGITFVLCISCVLGKTIVVLMAFRATLPGSNVMKWFGPPQQRLSVLAFTLIQVLICVLWLTIFPPFPNKNMKHYKEK
IILECDVGSAVGFWAVLGYIGLLSILCFVLAFLARKLPDNFNEAKFITFSMLIFCAVWITFIPAYVSSPGKFTVAVEIFAILASSFGLLFCIFLPKCYIILFRPEQNTRKHIMGKTDV
>Japanese_eel.locus186.1

EEYWPNTKRDQCVPKAAEFLSFHEILGIVLAIFSIFGACVTGFVTVQFFIKKDTPIVKANNSELSFLLLFSLKLCFLCSLTFIGRPSEWSCMLRHTAFGITFVLCISCVLGKTIVVLMAFRATLPGSNVMKWFGPLQQRVSVLAFTLIQ
VLICVLWLTISPPFPNKNMKHYKEKIILECDVGSAVWFWAVLGYIGLLSIVCFVLAFLARKLPDKFNEAKYITFSMLIFCAVWITFIPAYVSSPGKFTVAVEIFAILASSFGLLLCIFLPKCYIILFQPEQNTKKHIMGKTNV
>Japanese_eel.locus182.1

EEYWSNTEKDQCVPKAVEFLSFHEILGIVLVIFSIFGACVTGFVTVLFFINKDTPIVKANNSELSFLLLFSLKLCFLCSLTFIGLPSEWSCMLRHTAFGITFVQCISCVLGKTIVVLMAFRATLPGSNVMKWFGPLQQRLSVLAFTLIQ
VLICVLWLTISPPFPNKNMKHYKEKIIQECDVGSAVGFWAVLGYIGLLSILCFVLAFLARKLPDNFNEAKFITFSMLIFCAVWITFIPAYVSSPGKFTVAVEIFAILASSFGLLFCIFLPKCYIILFQPEQNTRKHIMGKTNV
>Japanese_eel.locus175.1

MLAIFSIFGACVTAFVAVLFFIKKDTPIVKANNSELSFLLLFSLKLCFLCSLTFIGQPSEWSCMLRHTAFGITFVLCISCVLGKTIVVLMAFRATLPGSNVMKWFGPLQQRLSVLAFTLIQVLICVLWLTISPPFPNKNMKHYKEKIIL
ECDVGSAVGFWAVLGYIGLLSVLCFVLAFLARKLPDNFNEAKFITFSMLIFCAVWITFIPAYVSSPGKFTVAVEIFAILASSFGLLFCIFLPKCYIILFRPEQNTRKHVMAKT--
>Japanese_eel.locus184.1

MGIMLVIFSIFGACVTVLVSVLFFIKKDTPIVKSNNSELSFLLLFSLKLCFLCSLIFIGRPSEWSCMLRHTAFGITFVLCISCVLGKTIVVLMAFRATLPGSNVMKWFGPPQQRLSVLAFTLIQVLICVLWLTISPPFPNKNMKHYKEK
IILECDVGSTVGFWAVLGYIGLLSILCFVLAFLARKLPDNFNEAKHITFSMLIFCAVWITFIPAYVSSPGKFTVAVEIFAILASSFGLLFCIFLPKCYIILFRPKENTKKHIMGKTNV
>Japanese_eel.locus197.1

MMGIVLVIFSIFGACVTVLVAVLFFIKKDTPIVKSNNSELSFLLLFSVKLCFLCSLTFIGRPSEWSCMLRHTAFGITFVLCISCVLGKTIVVLMAFRATIPGSNVMKWFGPLQQRLSVLAFTLIQVLICVLWLTMSPPFPNKNMEHYKE
KIILECDVGSAVGFWAVLGYIGLLSILCFVLAFLARKLPDNFNEAKYITFSMLIFCAVWITFIPAYVSSPGKFTVAVEIFAILASSFGLLFCIFLPKCYIILFRPEQNTRKHIMGKTDV
>Japanese_eel.locus162.1
CIQCPDEYWPNAKRDQCVLKAVEFLSFQEIMGSILVIFSLLGTSLTVLVAVLFFIQKDTPIVKANNSELSFLLLFSLKLCFLCSLTFIGRPSEWSCMLRHTAFGITFVLCISCVLGKTIVVLMAFRATLPGSNVMKWFGPLQQRLSVLA
FTLIQVLICVLWLTISPPFPNKNMKHYKEKIILECDVGSAVGFWAVLGYIGLLSLLCFILAFLARKLPDNFNEAKFITFSMLIFCAVWITFIPAYVSSPGKFTVAVEIFAILASSFGLLFCIFIPKCYIMLFRPEQNTRKHLMGKTHA
>Japanese_eel.locus166.1
CILCPNEYWSNTKRDQCVPKAVEFLSFKEIMGIVLAIFSLFGACVTVLVAVLFFIKKDTPIVKANNSELSFLLLFSLKLCFLCSLTFIGRPSEWSCMLRHTAFGITFVLCISCVLGKTIVVLMAFRATLPGSNVMKWFGPLQQRVSVLA
FTLIQVLICVLWLTISPPFPNKNMKHYKEKIILECDVGSAVGFWAVLGYIGLLSILCFVLSFLARKLPDNFNEAKFITFSMLIFCAVWITFLPAYVSSPGKFTVAVEIFAILASSFGLLFCIFAPKCYIILLKPELNSRKHMMGKMPS
>Japanese_eel.locus158.1

MGIMLVIFSLFGTCVTVLVAVLFFMYRQTPIVKANNSELSFLLLFSLKLCFLCSLTFIGRPSEWSCMLRHTAFGITFVLCISCVLGKTIVVLMAFRATLPGSNVMKWFGPLQQRLSVLAFTLIQVLICVLWLTISPPVPNKNMKHYKEK
IILECDVGSALGFWAVLGYIGLLSVLCFILAFLARKLPDNFNEAKFITFSMLIFCAVWITFIPAYVSSPGKFTVAVEIFAILASSFGLLFCIFAPKCYVILFRPEQNTKKNIMGKPPS
>Japanese_eel.locus143.1
-MKCPPEYWSNENRSQCVLKAIEYLSYEEIMGIFLVGFACLGAVLAFVASTVFLLFKDTPIVKANNSELSFLLLFSLKLCFLCSLTFIGRPSEWSCMLRHTAFGITFVLCISCVLGKTIVVLMAFRATLPGSNVMKWFGPLQQRLSVLA
FTLIQVLICVLWLTISPPFPNKNMKHYKEKIILECDVGSAVGFWAVLGYIGFLSILCFVLAFLARKLPDNFNEAKFITFSMLIFCAVWITFIPAYVSSPGKFTVAVEIFAILASSFGLLFCIFVPKCYIIIFKPEFNSRKHLMGKMPS
>Japanese_eel.locus159.1

MGILLVAFSVIGGCLTITIALIFFVYKDTPIVKANNSELSFLLLFSLKLCFLCSLTFIGRPSEWSCMLRHTAFGITFVLCISCVLGKTIVVLMAFRATLPGSNVMKWFGPLQQRLSVLAFTLIQVLICVLWLTISPPFPNKNMKHYKEK
IILECDVGSAVGFWAVLGYIGLLSILCFVLAFLARKLPDNFNEAKFITFSMLIFCAVWITFIPAYVSSPGKFTVAVEIFAILASSYGMLFCIFVPKCYIILLKPEKNTKRNMMGKTS-
>Japanese_eel.locus193.1

MGILLVAFSVIGGCLTITIALIFFVYKDTPIVRANNSELSFLLLFSLKLCFLCSLTFIGRPSKWSCMLRHTAFGITFVLCISCVLGKTIVVLMAFRATLPGSNVMKWFGPLQQRLSVLAFTLIQVLICVLWLTISPPFPNKNMKHYKEK
IILECDVGSAVGFWAVLGYIGLLSILCFVLAFLARKLPDNFNEAKLITFSMLIFCAVWITFIPAYVSSPGKFTVAVEIFAILASSYGMLFCIFVPKCYIILLKPEKNTKRNMMGKT--
>Japanese_eel.locus149.1

MGIVLLIFSLLGATLTVGVALIFFMHKDTPIVKANNSELSFLLLFSLKLCFLCSLTFIGRPSEWSCMLRHTAFGITFVLCISCVLGKTIVVLMAFRATLPGSNVMKWFGPTQQRLSVLAFTLIQVLICVLWLTISPPFPTKNMKHFKEK
IILECDVGSAVGFWAVLGYIGLLSILCFVLAFLARKLPDNFNEAKFITFSMLIFCAVWITFIPAYVSSPGKFTVAVEIFAILSSSYGMLFCIFAPKCYIILVTPEKNTKKHMMGKIPL

```
>marker.zebrafish_9_10_F
---
CPGEYWSNTERNRCVIKAVEFLSFSEVMGIVLVIFSLFGAGLTVLVAVLFYSKKDTPIVKANNSELSFLLLFSLTLCFLCSLTFIGQPTKWSCMLRHTAFGITFVLCISCVLGKTIVVLMAFKATLPGSNVMKWFGPVQQRLSVFAFTL
IQVLICVLWLTISPPFPYKNMKYYKEKIILECNLGNTIGFWAVLGYIGLLAALCFLLAFLARKLPDNFNEAKFITFSMLIFCAVWITFIPAYVSSPGKFTVAVEIFAILSSSFGLLVSIFAPKCFIILLKPEQNTKQHVMGKT--
>marker.zebrafish_9_12_F
---
CPGEYWSNAEKNKCVLKDVEFLSFTEIMGIVLVIFSLFGAVLTALMAILFYRKKDTPIIKANNSELSFLLLFSLILCFLCSLTFIGRPTEWSCMLRHTAFGITFVLCISCVLGKTIVVLMAFKAALPGNNIMKWFGPVQQRLSVFALTL
IQVLICVLWLTMSPPFPHKNLKYYQEKIVLECNLGSNIGFWAVLGYIGLLAVLCFILAFLARKLPDNFNEAKFITFSMLIFCAVWITFIPAYVSPPGKFTVAVEIFAILASSFGLLFCIFVPKCYIILCKPEKNTKQHLMGKV--
>Zebrafish.locus479.1
------------------------------
MGIVLVIFSLFGAVLTALMAILFYRKKDTPIIKANNSELSFLLLFSLILCFLCSLTFIGRPTEWSCMLRHTAFGITFVLCISCVLGKTIVVLMAFKAALPGNNIMKWFGPVQQRLSVFALTLIQVLICVLWLTMSPPFPHKNLKYYQEK
IVLECNLGSNIGFWAVLGYIGLLAVLCFILAFLARKLPDNFNEAKFITFSMLIFCAVWITFIPAYVSPPGKFTVAVEIFAILASSFGLLFCIFVPKCYIILCKPEKNTKQHLMGKVLM
>marker.zebrafish_9_2_F
---
CLEEYWSNAEKNKCVLKAVEFLSFTEIMGIILVFFSLFGVVLTTLVTVLFYRKKDTPIVKANNSELSFLLLFSLSLCFLCSLTFIGRPTEWSCMLRHTVFGITFVLCISCVLGKTIVVLMAFKATLPGSNVMKWFGPVQQRLSVLAFTL
IQIIICVVWITLSPPFPYKNMEYYKEKIIIECNLGSTLGFWAVLGYISLLSVLCFVLAFLARKLPDNFNEAKFITFSMLIFCAVWITFIPAYVSSPGKLTVAVEIFAILSSSFGLLFCIFAPKCYIILLKPEQNTKQHMMGKT--
>Zebrafish.locus485.1
CKQCLEEYWSNAEKNKCVLKAVEFLSFTEIMGIILVFFSLFGVVLTTLVTVLFYRKKDTPIVKANNSELSFLLLFSLSLCFLCSLTFIGRPTEWSCMLRHTVFGITFVLCISCVLGKTIVVLMAFKATLPGSNVMKWFGPVQQRLSVLA
FTLIQIIICVVWITLSPPFPYKNMEYYKEKIIIECNLGSTLGFWAVLGYISLLSVLCFVLAFLARKLPDNFNEAKFITFSMLIFCAVWITFIPAYVSSPGKLTVAVEIFAILSSSFGLLFCIFAPKCYIILLKPEQNTKQHMMGKTL-
>marker.medaka_9_1_F
---
CPEEFWSNQIRDACVPKNVEFLSYTEDMSKILMFFTLFGVVLTVTVAVLFLINRDTPLVKANNSELSFLLLFSLSLCFLCSLTFIGRPTEWSCMLRHTAFGITFVLCISCILGKTIVVLMAFKATLPSSNVMKWFGPVQQRLTVFSLTI
IQVIICILWLTINPPFPFKNMKIFREKIILECSLGSSLGFWAVLGYIGILALLCFVLAFLARKLPDNFNEAKFITFSMVIFSAVWITFIPAYVSSPGKLTVAVEIFAILASSYGILFCIFAPKCYILALKPELNTKKSMLGKS--
>marker.cichlid_9-1aa
---
CPGEYWSNQNRDACILKNVEFLSFTEFMGKIIVFFTFFGVLLNLIVAALFLIKKDTPLIKANNSELSFLLLISLTLCFLCSLTFIGQPSEWSCMLRHTAFGITFVLCISCILGKTIVVLMAFKATLPGSNVMKWFGPVQQRLSVLAFTL
IQVLICILWLTINPPFPYKNMNHYKEKIILECALGSPVGFWAVLGYIGLLAMLCFVLAFLARKLPDNFNEAKFITFSMLIFCAVWLTFIPAYVSSPGKFTVAVEIFAILASSYGLLFCIFAPKCYIIVIKPELNTKKHLMGKI--
>marker.pufferfish_9_1_F
---
CPEELWPNQNRDACVPKTVEFLYFTEFMGIVLVFFTLLGVFFTLTVTALFLIHKDTPLVRANNSELSFLLLFSLTLCFLCSLTFIGRPSEWSCMLRHTAFGITFVLCISCVLGKTMVVLMAFRATLPGSHVMKWFGPAQQRLSVLTFTL
VQVLICILWLTNDPPFPLRNTKHYKGKIILECALGSAIGFWAVLGYIGVLALLCFILAFLARQLPDNFNEAKFITFSMLIFCAVWITFIPAYVSSPGKFTVAVEIFAILASSYGLLFCIFAPKCFIIVLKPQLNTKKHMMGKT--
>Zebrafish.locus841.1
------------------------------
MGVGLCIFSFIGVLLTATVSFLFYFYKETPIVRANNSELSFLLLFSLTLCFLCSLTFIGRPTDWSCMLRHTAFGITFVLCISCVLGKTIVVLMAFRATLPGSNVMKWFGPLQQRLSVVSLTLIQVIICVLWLTMSPPFPFLNLSYYREK
IILECNLGSAFGFWAVLGYTGLLSTLCFVLAFFARKLPDNFNEAKFITFSMLIFCAVWLTFIPAYVSAPGKYTVAVQLFAILASSFGLLFCIFAPKCYIILLKPEKNTKKQMMGKS--
>Zebrafish.locus502.1
------------------------------
MGVGLCIFSFIGVLLTATVSFLFYFYKETPIVRANNSELSFLLLFSLTLCFLCSLTFIGRPTDWSCMLRHTAFGITFVLCISCVLGKTIVVLMAFRATLPGSNVMKWFGPLQQRLSVVSLTLIQVIICVLWLTMSPPFPFLNLSYYREK
IILECNLGSAFGFWAVLGYTGLLSTLCFVLAFFARKLPDNFNEAKFITFSMLIFCAVWLTFIPAYVSAPGKYTVAVQLFAILASSFGLLFCIFAPKCYIILLKPEKNTKKQMMGKS--
>Zebrafish.locus493.1
CFSCDLEYWSNEKKDRCILKVVEFLSYAEIMGIILCIVSFIGLLLTVMVTCLFYLHKETPIVRANNSELSFLLLFSLSLCFLCSLTFIGRPTQWSCMLRHTAFGITFILCISCVLGKTIVVLMAFRATLPGSNVMKWFGPLQQRLSVVS
LTLVQVIVCLLWLTLSPPYPYMNLSYYREKIILECNLGSAFGFWAVLGYTGLLSTLCFVLAFLARKLPDNFNEAKFITFSMLIFCAVWLTFIPAYVSSPGKYTVAVEIFAILVSSFGLLFCIFAPKCYIILLKPEKNTKKQMMGKL--
>Zebrafish.locus494.1
------------------------------
MGILLGLFSLIGAFFTIVVTLIFYIYRSTPIVRANNSELSFLLLFSLTLCFLCSLTFIGRPTEWSCMLRHTAFGITFVLCISCVLGKTLVVLMAFKATLPGSNVMKWFGPLQQRLSVVSFTFIQLLICALWLILSPPFPYMNMKHYQEK
VILECNVGSATGFWAVLGYIGLLAVLCFVLAFLGRKLPDNFNEAKFITFSMLIFCAVWITFIPAYISSPGKFIVAVEIFAILVSSFGLLFCIFLPKCYVILLRPEINTKKHVMAKINK
>Zebrafish.locus497.1
CLKCPPEFWSNKWRDTCVPKLVEFLSFEDVMGIVLIIFSLLGVSFTLGIAIIFFVHKDTPIVKANNSELSFLLLFSLTLCFLCSLTFIGRPTEWSCMLRHTAFGITFVLCISCVLGKTIVVLMAFKATLPGSNVMKWFGPLQQKLSVIT
FTLLQVVICVLWLTLSPPFPYMNMNYYQERIILECNLGSAFGFWAVLGYIGLLAILCFILAFLARKLPDKFNEAKFITFSMLIFCAVWITFIPAYVSSPGKFTVAVEIFAILASSFALLFCIFAPKCYIILLRPEENTKKHMMSKS--
>marker.zebrafish_5_1_F
---
CPPEFWSNKWRDTCVPKLVEFLSFEDVMGIVLIIFSLLGVSFTLGIAIIFFVHKDTPIVKANNSELSFLLLFSLTLCFLCSLTFIGRPTEWSCMLRHTAFGITFVLCISCVLGKTIVVLMAFKATLPGSNVMKWFGPLQQKLSVITFTL
LQVVICVLWLTLSPPFPYMNMNYYQERIILECNLGSAFGFWAVLGYIGLL-ILCFILAFLARKLPDKFNEAKFITFSMLIFCAVWITFIPAYVSSPGKFTVAVEIFAILASSFALLFCIFAPKCYIILLRPEENTKKHMMSKS--
>Zebrafish.locus498.1
CLKCPPEFWSNKWRDTCVPKLVEFLSFEDVMGIVLIIFSLLGVSFTLGIAIIFFVHKDTPIVKANNSELSFLLLFSLTLCFLCSLTFIGRPTEWSCMLRHTAFGITFVLCISCVLGKTIVVLMAFKATLPGSNVMKWFGPLQQKLSVIT
FTLLQVVICVLWLTLSPPFPYMNMNYYQERIILECNLGSAFGFWAVLGYIGLLAILCFILAFLARKLPDNFNEAKFITFSMLIFCAVWITFIPAYVSSPGKFTVAVEIFAILASSFGSLFCIFAPKCYIILLKPEENTKKHMMSKS--
>marker.cichlid_8-1aa
---
CPPEFWSNERRDTCIKKEAVFLSYEEIMGALLTAASLFGTCMTTGVALIFFKYRKTPIVRANNSELSFLLLFSLTLCFLCSLTFIGQPSEWSCMLRHVAFGITFVLCISCVLGKTMVVLMAFRATRPGSNVQKWFGPTQQRLCVTSFTL
IQVIICIIWLTVSPPFPFKNFKEFKDKITLECALGTSVGYWAVLGYIGLLSVLCFIFAFLARKLPDNFNEAKFITFSMLIFCAVWITFIPAYVSSPGKFSVAVEIFAILASSFGLLICIFFPKCYIILLKPEKNTKKNMMGKG--
>marker.cichlid_8-4aa
---
CLPEFWSNERRDSCIKKEAVFLSYEEIMGALLTAASLFGACMTIGVILIFFKYRKSPIVRANNSELSFLLLFSLILCFLCSLTFIGQPSDWSCMLRHVTFGITFVLCISCLLGKTIVVLMAFRATRPGSNVKKWFGQTQQRLCVTAFTL
IQVVICIIWLTTFPPFPSKNLLQFRDKIILECALGSAVGFWAVLGYIGILAILCFIFAFLARKLPDNFNEAKLITFSMLIFCAVWITFIPAYISSPGKFSVAVEIFAILASSFGLLICIFIPKCYIILLKPEKNTKRNMMGKK--
>marker.medaka_8_2_FP
---
CHPEFWSNERRDACVRKQEVFLSYEEIMGALLTAASLFGTCLTAIVAFIFFRHRKTPLVRANNSELSFLLLFSLILCFLCSLTFIGRPSEWTCQLRHTAFAITFVLCISCVLGKTIVVLMAFKARLPGGNMMKWFGPAQQRLSVLGLTL
IQVIICILWLTISPPFPFKNVQEFRDKIILECALGSELGFWAGLGYIGLLAVLCFISAFLARKLPDNFNEAKFITFSMLIFCAVWITFIPAYVSSPGKFSVAVEIFAILASSFGLLICIFIPKCYVILLKPEKNTRKYMMGKG--
>marker.fugu_8_2_F
---
CHSDFWSNERRDTCIKKKEEFLSYEEMMGALLTAASLLGTCLTAVVMFIFFRYRRTPIVRANNSELSFLLLFSLTLCFLCSLSFIGRPSGWSCMLRHAAFGITFVLCISCVLGKTMVVLMAFRATLPGSNVMKWFGPAQQRLSVLGFTL
IQVFICLLWLTISPPFPFKNIKDFKDRIILECALGSALGFWAVLGYIGLLAMFCFILAFLARKLPDNFNEAKFITFSMLIFCAVWVTFIPAYVSSPGKFSVAVEIFAILSSGFGLLICIFIPKCYIILLKPDRNNKKNIMGKA--
>Zebrafish.locus492.1
CLRCHQDFWSNLQKDQCIRKQTEFLSYEEIMGLLLTTISVAGAFLTMLIAVIFFKYKNTPIVKANNSELSFLLLFSLMLCFLCSLTFIGRPTEWSCMLRHTAFGITFVLCISCVLGKTIVVLMAFKATLPGSNVMKWFGPPQQRLSVLA
FTLIQVLICVLWLTISPPFPFKNVKYFKEKIILECNVGSVLGFWAVLGYVGLLAILCFFLAFLARKLPDNFNEAKFITFSMLIFCAVWVAFIPAYVSSPGKFTVAVEIFAILASTYGLLFCIFIPKCYVILLKPDKNSKKHLIGKGPP
>Zebrafish.locus495.1
------------------------------
MGILLTTISIVGAFVTMIIAVIFFRYKNTPIVKANNSELSFLLLFSLMCCFLCSLTFIGQPTEWSCMLRHTAFGITFVLCISCVLGKTIVVLMAFKATLPGSNVMKWFGPPQQRLSVLGFTLIQVLICVLWLTISPPFPFKNFNYFKEK
IILECHVGSSIGFWAVLGYIGCLALLCFILAFLARKLPDKFNEAKFITFSMLIFCAVWIGFIPAYVSSPGKFTVAVEIFAILASSFGLLFCIFLPKCYVILFKPDKNSKKHIMGKLTS
>marker.fugu_6_2_F
---
CPNLFWSNQERDACIPKSIEFLAHKELLGTLLVLFSLLGVFLTTFMFLIFYCHKETPIVRANNSELSFLLLFSLTLCFLCSLTFIGRPSGWSCMLRHTAFGITFVLCISCVLGKTMVVLMAFRATLPGSNMLKWFGPAQQRLCVVAFTF
VQVLICILWLTISPPYPFENMDYYNDRIILECALGSAVGFWAVLGYIGLLAMFCFILAFLARKLPNNFNEAKFITFSMLIFCAVWVTFIPAYVSSPGKFLVAVEIFAILASSYGLLFCIFLPKCFIILFRPELNTKKHIIGKT--
>marker.pufferfish_6_1_F
---
CPTLFWSSKERDACVPKTIEFLAHEELLGTLLVLCSLLGVFLTTIMFLVFYCHKETPLVRANNSELSFLLLFSLTLCFLCSLTFIGRPSEWSCMLRHTAFGITFVLCISCVLGKTVVVLLAFRATLPGSNVMKWFGPAQQRLSVLAFTF
VQVLTCTLWLTISPPYPFQNMEYYNDRIILECALGSAVGFWAVLGYIGVLAMFCFILAFLARNLPDNFNEAKFITFSMLIFCAVWITFIPAYVSSPGKFTVAVEIFAILASSFGLLFCIFLPKCYIILFRPELNTKKHIMGKA--
>marker.fugu_6_1_F
---
CPQELWPNDQKNLCLPKPSEYLSYREITGALLSGFGCLGVFLSLLTTLIFLVHKETPIVKANNSELSFLLLLSLKLCFLCSLTFIGRPSEWSCMLRHTAFGITFVLCISCVLGKTVVVLMAFRATLPGSKVMKWFGPKQQRLTVLTFTL
IQVVICILWLTTNPPFPMRNMTYYKDKIILECALGSAVGFWAVLSYIGVLAILCFILAFLARKLPDTFNEAKLITFSMLIFCAVWITFIPAYISSPGKFTVAVEIFAILASSFGLLLCIFFPKCYIIIFRPQKNCRKSLLGNP--
>marker.fugu_7_1_F
---
CPPEYKSNEERNNCNLKAIEFLTFRELMGILLVAFSVFGACLSTTIALIFFHFRQTPIVRANNSELSFLLLFSLTLCFLCSLTFIGRPSEWSCMLRHTAFGITFVLCISCILGKTMVVLMAFRATLPGSNMMKWFGAAQQRLSVLTFTL
VQVLICILWLTINPPFPFKNTKHYKDKIILECALGSALGFWAVLGYIGLLAVLCFVLAFLARKLPDNFNEAKFITFSMLIFCAVWITFIPAYASSPGKFTVAVEIFAILASSYGMLFCIFLPKCYVILLKPENNTKKKLMGKV--
>marker.pufferfish_7_1_PF
---
CLPEYKPNEERNKCHLKAIEFLTFRELMGTLLATFSVFGACLSMAMALIFFYFRQTPLVKANNSELSFLLLFSLTLCFLCSLTFIGRPSDWSCMLRHTAFGITFVLCISCVLGKTMVVLMAFRATLPGSHVMKWFGPAQQRLSVLTLTL
GQVLICILWLTIDPPFPFKNTKHYKEKIILECAMGSAVGYWAVLGYIGLLAVLCFILAFLARKLPDNFNEAKFITFSMLIFCAVWITFIPAYASSHGKFTVAVEIFAMLASSYGMLFCIYLPKGYIILLKPEYNTRKNLIGKV--
>marker.cichlid_7-1aa
---
CPPEFKSNEERTNCEMKAIEFLTFTELLGILLVSFSLFGACISMTVALIFFQYRHTPIVRANNSELSFLLLFSLTLCFLCSVTFIGRPSEWSCMLRHTAFGITFVLCISCMLGKTIVVVMAFKATLPGSNVKKWFGPVQQRLSVLAFTL
IQILICLVWLAINPPFPYKNMNYYKDKIILECALGSPLGFWTVLGYIGLLAMLCFVLAFLVRKLPDNFNEAKFITFSMLIFCAVWLTFIPAYVSSPGKFTVAVEIFAILASSYGMLFCIFLPKCYIILLKPENNTKKHLMSKG--
>marker.medaka_7_1_F
---
CHPEYKSSEERTSCDLKAIEFLTFTELMGILLVAFSIFGACLAMIVAVIFYHHRKTPMVKANNSELSFLLLFSLSLCFLCPLTFISRPTKWSCMLRHSTFGIIFVLCMSCVLGKTIVVLMAFNATVPGSNVIKWFGPVQQRVSVLTFTI
IQVLICIMWLTINPPYPHKNVKYYKDKVILECSHGSSLGFWAVLGYIGVLSLLCFGLAFMARKLPNNFNEAKFITFSMLIFCAVWLTFIPAYVSSPGKFTVAVEIFAILASSYGMLCCIFFPKCYIILFKPEKNTKKHLMGKE--
>marker.zebrafish_7_1_F
---
CPLEYKSNGNRTQCVLKNIEFLTFNEVMGNILVTFSMCGGCLTITVGLIFFYHRHTPIVRANNSELSFLLLFSLTLCFLCSLTFIGQPTEWSCMLRHTAFGITFVLCISCVLGKTLVVLMAFRATLPGSNVMKWFGPPQQRLSVFVFTF
IQLLICMLWLTMSPPFPNKTTNNYKDKIILECDSGSAVGFWAVLSYIGFLAILCFILAFLARKLPDNFNEAKFITFSMLIFCAVWITFIPAYISSPGKFTVAVEIFAILASSYGMLFCIFIPKCYIILLKPDLNSKKKIMGKV--
>Zebrafish.locus490.1
------------------------------
MGNILVTFSMCGGCLTITVGLIFFYHRHTPIVRANNSELSFLLLFSLTLCFLCSLTFIGQPTEWSCMLRHTAFGITFVLCISCVLGKTLVVLMAFRATLPGSNVMKWFGPPQQRLSVFVFTFIQLLICMLWLTMSPPFPNKTTNNYKDK
IILECDSGSAVGFWAVLSYIGFLAILCFILAFLARKLPDNFNEAKFITFSMLIFCAVWITFIPAYISSPGKFTVAVEIFAILASSYGMLFCIFIPKCYIILLKPDLNSKKKIMGKVSS
>Spotted_gar.locus130.1
------------------------------
MGIVLLIFSVSGVGVTIGIALIFFKYKDTPIVKANNSELSFLLLFSLTLCFLCSLTFIGQPSDWSCMLRHTAFGITFVLCISCVLGKTIVVLMAFRATLPGNNIMKWFGPTQQRLSVLAFTLVQVLICVLWLAISPPFPKKNMKYYREK
```

```
IILECDLGSAGAFWAVLGYIGFLSALCFVLAFLARKLPDNFNEAKFITFSMLIFCAVWITFIPAYISSPGKFTVAVEIFAILASSFGLFFCIFAPKCYILLLKPELNTKKHLMGKMPS
>Spotted_gar.locus120.1
------------------------------
MGVVLSIFSLVGTCLTLGIALIFFKFKHTPIVKANNSELSFLLLFSLTLCFLCSLTFIGQPSDWSCMLRHTAFGITFVLCISCVLGKTIVVLMAFRATQPGSNIMKWFGPKQQLLSVLSFTLLQALICTIWLTLSPPFPKQNMKYYKDR
IIL-CDLGSAGAFWAVLGYIGFLSVLCFVLAFLARKLPDNFNEAKFITFSMLIFCAVWLTFIPAYISSPGKFTVAVEIFAILASSFGLLFCIFAPKCYIIVLKPELNTRKHLMGKVLT
>Spotted_gar.locus70.1
------------------------------
MGILLIIFSLLGVLLTFGVALVFFQHKHTPIVKANNSELSFLLLFSLTLCFLCSLTFIGQPSDWSCMLRHTAFGITFVLCISCVLGKTIVVLMAFRATLPGNNIMKWFGPKQQRLSVLAFTLIQVLICVLWVTISPPFPYKNMKYYKEK
IILECDLGSAGAFWAVLGYIGFLSALCFVLAFLARKLPDNFNEAKFITFSMLIFCAVWITFIPAYISSPGKFTVAVEVFAILASSFGLLFCIFSPKCYIILLKPQYNTKKNMMGKP--
>Spotted_gar.locus100.1
------------------------------
MGILLAILSLLGSCLTIAMALVFIKFKDTPIVKANNSELSFLLLFSLTLCFLCSLTFIGQPSDWSCMLRHTAFGITFVLCISCVLGKTIVVLMAFRATLPGSNIMKWFGPTQQRLSVLAFTLVQVLICAFWLAISPPFPNKNMKHYREK
IILECDLGSAGAFWAVLGYIGFLSALCFVLAFLARKLPDNFNEAKFITFSMLIFCAVWITFIPAYVSSPGKFTVAVEIFAILASSFGLLFSIFTPKCYIILLKPELNTKKHMMGKMTL
>Spotted_gar.locus83.1
------------------------------
MGMLLVTCSGFGTSLTIAIAVVFFRYRYTPIVRANNSELSFLLLFSLTLCFLCSFTFIGQPSDWSCMLRHTAFGITFVLCISCVLGKTIVVLMAFRATLPGSNIMKWFGPTQQRLNVLAFTLVQVLICFLWLTTSPPFPNKNMKSYADR
IILECDLGSVGAFCAVLGYIGFLSAMCFVLAFLARKLPDNFNEAKFITFSMLIFFAVWITFIPAYISSPGKFTVAVEIFAILASSYGLLLCIFMPKCYIILFKPEENTKKHVMGKM--
>Spotted_gar.locus81.1
------------------------------
MGILLVTCSGFGTSLTIAIAFVFFRYRYTAIVKANNSELSFLLLFSLTLCFLCSLTFIGQPSDWSCMLRHTAFGITFVLCISCVLGKTIVVLMAFRATLPGNNIMKWFGPTQQRLSVLAFTLVQVLICFLWLKISPPFPNKNMKYYADR
IILECDLGSVGAFCAVLGYIGFLSAMCFVLAFLARKLPDNFNEAKCITFSMLIFCKVWITFIPAYVSSPGKFTVAVEIFAILASSYGLLFCIFMPKCYIILFKPEQNTKQHVMGKMPS
>Spotted_gar.locus87.1
------------------
MKNIEFLSYGELMGILLVTCSAFGTSLTIAIAVVFFRYRSTPIVRANNSELSFLLLFSLTLCFLCSLTFIGQPSDWSCMLRHTAFGITFVLCISCVLGKTIVVLMAFRATLPGNNIMKWFGPTQQRLSVLAFTLVQVLICGLWLIISPP
FPNTNMNYYADRIILECDLGSAGAFWAVLGYIGFLSAMCFVLAFLARKLPDNFNEAKFITFSMLIFCAVWITFIPAYVSSPGKFTVAVEIFAILASSFGLLFCIFMPKCFIILLKPEKNSKKHLMRKMPS
>Spotted_gar.locus78.1
------------------------------
MGVLLVTCSVLGSSITIVIALIFFHHRSTPIVKANNSELSFLLLFSLTLCFLCSLTFIGQPSDWSCMLRHTAFGITFVLCISCVLGKTIVVLMAFRATLSGNNIMKWFGPTQQRLSVLAFTIVQVLICVLWLIMSPPFPNKNMKYYAER
IILECDLGSAGAFWAVLGYIGFLSAMCFVLAFLARKLPDNFNEAKFITFSMLIFCAVWITFIPAYISSPGKFTVAVEIFAILASSYGLLFCIFMPKCYIILLKPEQNTKKHLMGKIVS
>Spotted_gar.locus77.1
CTACPLEYRSNEQRSQCVLKNIEFLSFGELMGVLLVTCSVLGSSITIVIALIFFHHRSTPIVKANNSELSFLLLFSLTLCFLCSLTFIGQPSDWSCMLRHTAFGITFVLCISCVLGKTIVVLMAFRATLPGNNIMKLFGPKQQRLSVLG
FTLIQVFICVLWLTITPPFPNKNVKYYKEKIILECDLGSAGAFWAVLGYIGFLSAMCFVLAFLARKLPDNFNEAKFITFSMLIFCAVWITFIPAYISSPGKFTVAVEIFAILASSYGLLFCVFLPKCYIILIKPEINTKKHLMSKG--
>Spotted_gar.locus92.1
------------------------------
MGILLVTFSLLGTFLTIAIAVIFFQYRFTPIVRANNSELSFLLLFSLTLCFLCSLTFIGQPSDWSCMLRHTAFGITFVLCISCVLGKTIVVLMAFRATLPGNNIMKWFGPTQQRLSVLAFTLIQVLICVLWLTISPPFPYKNMKYYTER
IILECDLGSAGAFCAVLGYIGFLSALCFVLAFLARKLPDNFNEAKFITFSMLIFCAVWITFIPAYISSPGKFTVAVEIFAILASSFGLLFCIFIPKCYIILLKPEKNTKKHLMSKMSS
>Spotted_gar.locus94.1
------------------------------
MGILLVTFSLLGTCLTIAIAVIFYQYRFTPIVRANNSELSFLLLLSLTLCFLCSLTFIGQPSDWSCMLRHTAFGITFVLCISCVLGKTIVVLMAFRSTLPGNNIMKWFGPRQQRLSVLAFTLVQVLICVLWLTISPPFPNKNMKYYTER
IILECDFGSNIGYYVALGYIGFLSALCFVLAFLARKLPDNFNEAKFITFSMLIFCAVWITFIPAYISSPGKFTVAVEIFAILASSFGLLFCIFLPKCYIILLNPEKNTKKHLMGKMPS
>Spotted_gar.locus73.1
------------------------------
MGILLTVFSLLGTSLSIAIAAIFFRYRFTPIVRANNSELSFLLLFSLTLCFLCSLTFIGQPSDWSCMLRHTAFGITFVLCISCVLGKTIVVLMAFRATLPGNNIMKWFGPKQQRLSVLAFTLVQVLICVLWLTTSPPFPNKNTKYYAER
IILECDLGSAGAFWAVLGYIGFLSAMCFVLAFLARNLPDNFNEAKFITFSMLIFCAVWITFIPAYISSPGKYTVAVEIFAILASSFGLLLCIFTPKCYIIILKPEKNTKKHMMGKVPS
>Spotted_gar.locus88.1
------------------------------
MGIILVTFSVFGACLTIIIANIFFKYRSTPIVRANNSELSFLLLFSLTLCFLCSLTFIGQPSDWSCMLRHTAFGITFVLCISCVLGKTIVVLMAFRATLPGNNIMKWFGPTQQRLSVLFFTLIQILICVLWLIMSPPFPNMNMKYYAER
IILECDLGSAGAFWAVLGYIGFLSAICFVLAFFARKLPDNFNEAKFITFSMLIFCAVWITFIPAYISSPGKFTVAVEIFAILASSFGLLFCIFMPKCYVILLKPERNTKKYLMSKMPS
>Spotted_gar.locus90.1
CAKCPLEYKSNEHRNQCVLKNIEFLSFGESMGKVLVTFSVFGACLTIIISHIFFRYRSTPIVRANNSELSFLLLFSLTLCFLCSLTFIGQPSDWSCMLRHTAFGITFVLCISCVLGKTIVVLMAFRATLPGNNIMKWFGPKQQRLSVLA
FTLVQVLICVLWLTISPPFPHKNTTYYKETIILECDVGSPVGFYAVLGYIGFLSAMCFVLAFLARNLPDNFNEAKFITFSMLIFCAVWITFIPAYISSPGKFTVAVEIFAILASSYGLVFCIFTPKCYIILLKPDKNSKKHLMGKMPS
>Spotted_gar.locus89.1
------------------------------
MGKLLVAFSIFGACLTIFIAHIFFRYRSTPIVRANNSELSFLLLFSLTLCFLCSLTFIGQPFDWSCMLRHTAFGITFVLCISCVLGKTIVVLMAFRATLPGNNIMKWFGPKQQRLSVLAFTLVQVLICVLWLTISPPFPHKNTTYYKET
IILECDVGSPVGFYAVLGYIGFLSAMCFVLAFLARNLPDNFNEAKFITFSMLIFCAVWITFIPAYISSPGKFTVAVEIFAILASSFGLLFCIFIPKCYIILLKPEKNTKKHLMSKVPS
>Spotted_gar.locus66.1
------------------------------
MGKVLVFFSLLGATITCSITIVFFRYRNTPIVKANNSELSFLLLFSLTLCFLCSLTFIGQPFDWSCMLRHTAFGITFVLCISCVLGKTIVVLMAFRATLPGNNIMKWFGPTQQRFSVLAFTLIQVLICTLWLTISPPFPYKNTKYYKEK
VILECDIGSLAGFCAVLGYIGFLSAMCFVLAFLARNLPDNFNEAKFITFSMLIFCAVWTTFIPAYISSPGKFTVAVEIFAILASSFGLLICIFMPKCYILLLKPEKNTKKYLMGKMPS
>Spotted_gar.locus96.1
CTLCPLEYRPAEQRNQCILKSVEFLSFRELMGVLLVTFALLGTCLTMVISVIFFQYRFTPIVRANNSELSFLLLFSLTLCFLCSLTFIGQPSDWSCMLRHTAFGITFVLCISCVLGKTIVVLMAFRATLPGNNIMKWFGPKQQRLSVLG
FTLVQVLICTFWLAISPPFPQNNTKYYKEKNILECNLGSTVGFGAVLGYIGFLSAMCFVLAFLARKLPDNFNEAKFITFSMLIFCAVWITFIPAYISSPGKFTVAVEIFAILASTYGLLFCIFMPKCYIILLQPEKNTKKHLMGKAPI
>Spotted_gar.locus71.1
------------------------------
MGILLTAFSVLGIVLTMVTAFIFFQYRSTPIVRANNSELSFLLLVSLTLCFLCSLTFIGQPSDWSCMFRHTAFGITFVLCISCVLGKTIVVLMAFRATLPGNNIMKWFRPTQQRLSVLAFTLVQVLICTLWLTISPPFPYKNTKYYKEK
IILECDIGSLAGFCAVLGYIGFLSAMCFMLAFLARKLPDNFNEAKFITFSMLIFCAVWIAFIPAYISSPGKFTVAVEIFAILASSYGLLSCIFMPKCYIILLKPEKNSKKHLMGKIPT
>Spotted_gar.locus76.1
------------------------------
MGIFLVTFSAFGSCLTIAVAFIFFRYRFTPIVRANNSELSFLLLFSLTLCFLCSLTFIGQPSDWSCMLRHTAFGITFVLCISCVLGKTIVVLMAFRATLPGNNIMKWFGPRQQRLSVLALTFIQVLICVFWLTKTPPSPKKNMSYYREK
IILECDLGSLAGFIAVLGYIGFLSAMCFVLSFLARKLPDNFNEAKFITFSMLIFCAVWITFIPAYISSPGKFTVAVEIFAILASSYGLLFCIFMPKCYIILLKPEKNTKKLLMGKVSS
>Spotted_gar.locus74.1
------------------------------
MGILLVAFSLLGTFLTTAIALIFFQYRSTPIVRANNSELSFLLLFSLTLCFLCSLTFIGQPSDWSCMLRHTAFGITFVLCISCVLGKTIVVLMAFRATLPGNNIMKWFGPKQQRLSVLAFTLIQVLICVLWLTISPPFPNKNMNYYREK
IILECDLGSTAGFCAVLGYIGFLSALCFVLAFLARKLPDNFNEAKFITFSMLIFCAVWITFIPAYISSPGKFTVAVEIFAILASSYGLLFCIFIPKCYIILLKPEQNTKKHLMSKMPS
>Spotted_gar.locus69.1
------------------------------
MGIILVFVSASGACLTIITAIIFYNNRDTPLVKANNSELSFLLLFSLTLCFLCSLTFIGQPSDWSCMLRHTAFGITFVLCISCVLGKTIVVLMAFRATLPGSNIMKWFGPTQQRLSVLAFTLIQVLICVLWVTISPPLPYKNMKYYKEK
IILECGLGSAGAFWAVLGYIGFLSAMCFVLAFLARNLPDNFNEAKFITFSMLIFCAVWITFIPAYISSPGKYTVAVEIFAILSSSYGLLLCIFTPKCYIILLKSEKNSRKNLMARMPS
>Spotted_gar.locus62.1
------------------------------
MGILLVLFSITGTCLTIITVIIFYCYRDTPLVRANNSELSFLLLFSLTLCFLCSLTFIGQPSDWSCMLRHTAFGITFVLCISCVLGKTIVVLMAFRATLPGNNIMKWFGPTQQRLSVLGFTLVQVLICVLWLTISPPFPNRNMKYYREK
IILECDLGSAGAFWAVLGYIGFLSAMCFVLAFLARNLPDNFNEAKFITFSMLIFCAVWITFIPAYISSPGKFTVAVEIFAILASSYGLLFCIFIPKCYVILLYPEKNTKRHLMGKI--
>Spotted_gar.locus48.1
------------------------------
MGILLAVFALLGTFLAVATAIVFFRFKDTPIVKANNSELSFLLLFSLTLCFLCSLTFIGQPSDWSCMLRHTAFGITFVLCISCVLGKTIVVLMAFRATLPGSNIMKWFGPTQQKLSVLAFTLVQALICTLWLILSPPFPNQNMKYYKDR
IILECDLGSAGAFWTVLGYVGLLSAMCFVLAFLARNLPDNFNEAKFITFSMLIFCAVWITFIPAYISSPGKFTVAVEIFAILASSYGLLFCIFSPKCYIILLKPARNTKKHMMSKMPS
>Spotted_gar.locus52.1
CTKCPLEYWSNDQRDQCILKDIEFLSYGEIMGILLAAFALLGTCLAFVTAAVFFRFKDTPIVKANNSELSFLLLFSLTLCFLCSLTFIGQPSDWSCMLRHTAFGITFVLCISCVLGKTIVVLMAFRATLPGSNIMKWFGPTQQRLSVLA
FTLVQVLICVLWLILSPPFPYKNTKYYKEKIILECDLGSTVGFCAVLGYIGFLSAMCFLLAFLARKLPDNFNEAKFITFSMLIFCAVWITFIPAYISSPGKFTVAVEIFAILASSFGLLFCIFAPKCYIIILNPDMNTKRHLMGKQPS
>Spotted_gar.locus55.1
CDKCPLEYWSNENRDQCILKDTEFLSYEETLGILLSIFSMLGACFTLAVAIILFHYKHTPIVKANNSELSFLLLFSLTLCFLCSLTFIGQPSDWSCMLRHTAFGITFVLCISCVLGKTIVVLMAFRATLPGNNIMKWFGPKQQRLSVLG
FTLVQVLICILWVTISPPFPNKNIKYYKDRIILECDLGSAGAFWAALGYIGFLSALCFVLAFLARKLPDNFNEAKFITFSMLIFCAVWITFIPAYISSPGKFTVAVEIFAILASSFGLLFCIFAPKCYIILLQPEKNTKKHLMGKMPN
>Reedfish.locus40420.1
------------------------------
MGILLVLLSVLGVCACFAIAVVFFWKRNTPLIRANNSEISFLLLFSLSLCFLCSLTFIGEPSDWSCMLRHTAFGITFVLCISCLLAKTIVVLMAFRSTLPGGNVMKWFGPTQQRIHIYTLTLIQSFICVLWLTISPPFPYRNITYYKDK
IILECNLGSPLAFYAVLGYIGFLSAMCFILAFLARKLPDNFNEAKFITFSMLIFCAVWITFIPAYISSPGKYTVAVEIFAILASSYGLLFCIFAPKYYIVILRPEKNTKKYLMNKVP-
>Reedfish.locus40429.1
------------------------------
MGSLLVVISLFGACITMCITVVFFYYRDTPVVRANNSEISFLLLFSLSLCFLCSLTFIGQPSDWSCMLRHTAFGITFALCISCVLAKTVVVLMAFRATLPGNNIMKWFGPSQQRLSVASFTLMQVLICSLWLIISPPFPYKNFKYYKER
IILECDLGSSVAFYSVLGYIGFLSSICFILAFLARNLPDNFNEAKFITFSMLIFCAVWISFIPAYISSPGKYIVAVEIFAILASSFGLLFCIFAPKCYVIIFKPYKNTKKHLMGKMPS
>Reedfish.locus40435.1
------------------------------
MGSLLVVISLFGACITMFIILVFFYYRDTPVVRANNSEISFLLLFSLSLCFLCSLTFIGQPSDWSCMLRHTAFGITFALCISCILAKTIVVLMAFRATLPGNNIMKWFGPSQQRLSVASFTLMQVLICLLWLILSPPFPYKNFKYYKER
IILECDLGSSVAFYSVLGYIGFLSAICFILAFLARNLPDNFNEAKFITFSMLIFCAVWISFIPAYISSPGKYTVAVEIFAILASSFGLLFCIFAPKCYVIIFKPYKNTKKHLMGKMPS
>Reedfish.locus27634.1
-------------------------
MFSEIMGSLLVAISLFGACIVTATAAIFYHYKDTPVVKANNSELSFLLLFSLALCFLCSLTFIGQPSDISCMLRHTAFGITFVLCISCILGKTIVVLMAFRATLPGNNVMKWFGPNQQRLTVFSFTSIQILICIMWLIISPPFPDKNTK
YYNDKVILECDLGSSIAFYIVLGYIGLLSAICFVLAFLARKLPDNFNEAKFITFSMLIFCSVWITFIPAYISSPGKYIVAVEVFAILVSSFGLLFCIFAPKCYIIILKPEKNTKKHLMAKTNV
>Reedfish.locus27636.1
-------------------------
MFNEIMGSLLVAISLFGACIVTATAAIFYHYKDTPVVKANNSELSFLLLFSLALCFLCSLTFIGQPSDISCMLRHTAFGITFVLCISCILGKTIVVLMAFRATLPGNNVMKWFGPNQQRLTVFSFTSIQILICIMWLIISPPFPDKNTK
YYNDKVILECDLGSSIAFYIVLGYIGLLSAICFVLAFLARKLPDNFNEAQFITFSMLIFCSVWITFIPAYISSPGKYIVAVEVFAILVSSFGLLFCIFAPKCYIIILKPEKNTKKHLMAKTNV
>Reedfish.locus40419.1
------------------------------
MGILLAAFSLFGACITAAIALVFFMNRDTPIVKANNSELSFLLLFSLTFCFLCSLTFIGEPTDWSCMLRHTVFGITFVLCISCILGKTIVVLMAFRATLPGRNVMKWFGTTQQRLSVYTLTLMQVLICVLWLIVSPPFPSRNTKYYKER
IIVECDLGSSVAFYSVLAYIGFLSSICFVLAFLARKLPDNFNEAKMITFSMLIFCAVWITFIPAYISSPGKYTVAVEIFAILASSFGLLFCIFFPKCYIILFKPEENTKKHIMGKMPS
>Reedfish.locus27690.1
CLKCPSEFWSNEERNHCLEKEIEFLSFAEIMGTLLMTISLAGATLSMTIALIFLYYKDTPIVKANNSELSFLLLISLTLCFLCSLTFIGQPSNWSCMLRHTTFGITFVLCISCVLGKTIVVLMAFRATLPGNNIMKWFGPTHQRISVCS
```

```
LTLIQILICIMWLILSPPFPNKNMKPYKEKIILECNMGSAIAFYAVIGYIGILSLLCFVLAFLARKLPDNFNEAKFITFSMLIFCAVWITFIPAYISSLGKYTVAVEIFAILASSFGLLFCIFAPKCYIILLKPDKNTRKHVMGK---
>Reedfish.locus27683.1
------------------------------
MGIFLITISLAGATLSITIAFIFFNYKDTPVVRANNSELSFLLLFSLTLCFLCSLTFIGQPSEWSCIMRHTTFGVTFVLCISCVLGKTIVVLMAFRATLPGNNIMKWFGPIQQRLSVCSLTLIQIFICIMWLILSPPFPNKNTSAYKEK
IILECNMGSTIAFYSVIGYIGVLSFLCFLLAFLARKLPDNFNEAKFITFSMLIFCAVWITFIPAYISSLGKYTVAVEIFAILASSFGLLFCIFAPKCYIILLKPEKNTRKHVMGK---
>Reedfish.locus27632.1
------------------------------
MGILLSIFSVAGACLSISIGIVFFQYRDTPVVKANNSELSFLLLFSLTLCFLCSLLFIGQPTEWSCMLRHTTFGITFALSITCILGKTIVVLVAFKATLPGYNIMKWLGATQQRLSVFSITLIQTIICTLWLILSPPFPYKNTNSYKDK
IILECNVGSAVAFYAVIGYVGFLSLACFILAFLARKLPDNFNEAKFITFSMLIFCSVWITFIPSYISSPGKYTVAVEIFAILASSFGLLTCIFAPKCYILLLRPEQNTKKHLMSKTPL
>Reedfish.locus40433.1
------------------------------
MGLLLTIFSLLGACLTIGIAVIFFHFKDTPVVRANNSELSFLLLISLTLCFLCSLTFIGQPSEWSCMLRHTAFGITFVLCISCVLAKTIVVLMAFRATLPGSNIMKWFGPTQQRLSVCVFAMPQILICTLWLILAPPFPNRNMKSFKEK
VIFECSLGSPLAFMAVLGYIGFLAIICFVLAFLARKLPDNFNEAKFITFSMLIFCAVWITFIPSYISSPGKYTVAVEIFAILASSFGLLFCIFAPKCFIILFKPEQNTKKHLMSKTPV
>Reedfish.locus27607.1
CLKCPLDFWPNEKKDLCLLKSMEFLSFGDIMGILLGTLSLLGASVTMFITLVFFYFRDTPVVRANNAELSFLLLFSLTLCFLCSLTFIGQPSEYSCMLRHTAFGITFVLCISCVLAKTVVVLMAFRATLPGNNIMKWFGVNQQRASVCT
FTLIQVLICSLWLATSPPFPTKNVKLYKEIIILECDLGSAVAFCTALGYIGILSLVCFFLAFLARNLPDNFNEAKFITFSMLIFCAVWITFIPAYISSPGKFTVAVEIFAILASSFALLFCIFIPKCYVILLKPESNTKKHLMGKIK-
>Reedfish.locus27626.1
--------------------
MEFLSFGDIMGILLGTLSLLGASVTMFITLVFFYFRDTPVVRANNAELSFLLLFSLTLCFLCSLTFIGQPSEYSCMLRHTAFGITFVLCISCVLAKTVVVLMAFRATLPGNNIMKWFGVNQQRASVCTFTLIQVLICSLWLATSPPFPT
KNVKLYKEIIILECDLGSAVAFCTALGYIGILSLVCFFLAFLARNLPDNFNEAKFITFSMLIFCAVWITFIPAYISSPGKFTVAVEIFAILASSFALLFCIFIPKCYVILLKPESNTKKHLMGKIK-
>Reedfish.locus27567.1
------------------------------
MGSLLVAFSLLGTCITVAIAITFFYFRDTPIVKANNSELSFLLLFSLTLCFLCSLTFIGQPSELSCMLRHTAFGIAFVLCISCVLGKTIVVLMAFKATFPGTNVMKWFGPSQQRLSVCSFTLIQVLICSLWLIISPPFPNKNMKSYKDK
IILECHVGSALAFYSVLGYIGFLSFICFILAFLARKLPDKFNEAKYITFSMLIFCAVWITFIPAYISSPGKYTVAVEIFAILASSFSLLFCIFIPKCYILLLKPEKNTKRHLMGKVRT
>Reedfish.locus27570.1
------------------------------
MGVVLVVCSLLGAFTTISIAAIFFLHRDTPIVRANNSELSFLLLFSLALCFLCSLTFIGQPSDWSCMLRHTAFGITFVLCISCVLGKTIVVLMAFRATLPGSNIMKWFGPTQQRFSVCTFTMIQVLICALWLLTSPPFPNKNMNSFKDK
IILECDLGLPAAFYAVLGYIGLLSVLCFVLSFLARKLPDNFNEAKFITFSMLIFCAVWITFIPAYISSPGKYTVAVEIFAILASSFGLLFCIFAPKCYVILLKPEKNTKKHLMGKMPS
>Sterlet.locus193.1
-MKCPLEYRSNNQKKQCILKAIEFLSFGEIMGMLLMIISLIGACLTTAIAIVFFLYRDTPIVRANNSELSFLLLFSLALCFLCSLTFIGQPSEWSCMLRHTSFGIIFVLCLSCVLGKTIVVLMAFRSTLPGNNMMKWFGPVQQRLTLFA
FTFIQALICTLWLIISPPFPNKNMNSFKDRIILECDLGSAAAFYAVLGYIGFLAAMCFVLAFLARNLPDNFNEAKYITFSMLIFCAVWITFIPAYISSPGKYTVAVEIFAILASSFGLLFCIFAPKCYIILFKPEKNTKKHLMGKGPS
>Sterlet.locus192.1
CMKCPLEYRSNNQKKQCILKAIEFLSFGEIMGMLLMILSLIGACLTTAIATVFFHFRDTPIVKANNSELSFLLLFSLALCFLCSLTFIGQPSEWSCMLRHTAFGITFVLCISCVLGKTIVVLMAFRSTLPGNNMMKWFGPVQQRLTLFV
FTFIQALICTLWLILSPPFPNKNVNSFKDRIILECDLGSAAAFYAVLGYIGFLAAMCFVLAFLARNLPDNFNEAKYITFSMLIFCAVWITFIPAYISSPGKYTVAVEIFAILASSFGLLFCIFAPKCYIILFKPEKNTKKHLMGKGPS
>Sterlet.locus195.1
-MTCPLEYRSNNQKNQCILKAIEFLSFGEIMGMLLMILSLIGACLTTAIATVFFHFRDTPIIRANNSELSFLLLFSLALCFLCSLTFIGQPSEWSCMLRHTAFGITFVLCISCVLGKTIVVLMAFRATLPGNNMMKWFGPVQQRLTLFA
FTFIQALICTLWLIISPPFPNKNMNSFKDRIILECDLGSAAAFYAVLGYIGFLAAMCFVLAFLARNLPDNFNEAKYITFSMLIFCAVWITFIPAYISSPGKYTVAVEIFAILASSFGLLFCIFAPKCYIILFKPEKNTKKHLMGKGPS
>Sterlet.locus199.1
-MKCPLEYRSNNQRNQCILKAIEFLSFGEIMGMLLMILSLIGACLTTAIATVFFRFRDTPIVRANNSELSFLLLFSLALCFLCSLTFIGQPSEWSCMLRHTAFGIAFVLCISCVLGKTIVVLMAFRATLPGNNMMKWFGPVQQRLTLFA
FTFIQALICTLWLIISPPFPSKNMNSFKDRIILECDLGSAAAFYAVLGYIGFLAAMCFVLAFLARNLPDNFNEAKYITFSMLIFCAVWITFIPAYISSPGKYTVAVEIFAILASSFGLLFCIFAPKCYIILFKPEKNTKKHLMGKGPS
>Sterlet.locus197.1
------------------------------
MGMLLMILSLIGACLTTAIAIVFFRFRDTPIVRANNSELSFLLLFSLALCFLCSLTFIGQPSEWSCMLRHTAFGITFVLCISCVLGKTIVVLMAFRSTLPGNNMMKWFGPAQQRLTLFAFTFIQALICTLWLIISPPSPNKNMRSYKDR
IILECDLGSAAAFYAVLGYIGLLAAMCFVLAFLARNLPDNFNEAKYITFSMLIFCAVWITFIPAYISSPGKYTVAVEIFAILASSFGLLFCIFAPKCYIILFKPEKNTKKHLMGKGPS
>Sterlet.locus194.1
-MKCPLEYMSNNQKNQCTLKAIEFLSFGEIMGMLLMILSLIGACLTTAIATVFFRFRDTPIVRANNSELSFLLLFSLALCFLCSLTFIGQPSEWSCMLRHTAFGITFVLCLSCVLGKTIVVLMAFRSTLPGNNMMKWFGPAQQRLTLFA
FTFIQALICTLWLIISPPFPNKNMNSFKDRIILECDLGSAAAFYAVLGYIGFLAAMCFVLAFLARNLPDNFNEAKYITFSMLIFCAVWITFIPAYISSPGKYTVAVEIFAILASSFGLLVCIFAPKCYIILFKPEKNTKKHLMGKGPS
>Sterlet.locus196.1
-MKCPLEYRSNNQKNQCILKAIEFLSFGEIMGMLLMILSLIGACLTTAIAIVFFRFRDTPIVRANNSELSFLLLFSLALCFLCSLTFIGQPSEWSCMLRHTAFGITFVLCISCVLGKTIVVLMAFRSTLPGNNIMKWFGPAQQRLTLFA
FTFIQSLICTLWLIISPPFPNKNMNSFKDRIILECDLGSAAAFYAVLGYIGFLAAMCFVLAFLARNLPDNFNEAKYITFSMLIFCAVWITFIPAYISSPGKYTVAVEIFAILASSFGLLFCIFAPKCYIILFKPEKNTKKHLMGKGPS
>Sterlet.locus284.1
CMKCPLEYRSNNQRNHCILKAIEFLSFGEIMGMLLMILSLIGACLTTAVAIVFFHYRDTPIVKANNSELSFLLLFSLALCFLCSLTFIGQPSEWSCMLRHTAFGITFVLCISCILGKTIVVLMAFRSTLPGNNMMKWFGPAQQRLTLFA
FTFIQALICTLWLIISPPFPNKNMNSFKDRIILECDLGSAAAFYAVLGYIGFLAAMCFVLAFLARNLPDNFNEAKYITFSMLIFCAVWITFIPAYISSPGKYTVAVEIFAILSSSFGLLFCIFAPKCYIILFKPEKNTKKHLMGKVPS
>Sterlet.locus285.1
-MKCPLEYRSNNQRNHCILKAIEFLSFAEIMGMLLMILSLIGACLTTAVAIVFFHYRDTPIVKANNSELSFLLLFSLALCFLCSLTFIGQPSEWSCMLRHTAFGITFVLCLSCVLGKTIVVLMAFRSTLPGNNMMKWFGPAQQRLTLFA
FTFIQALICTLWLTIAPPFPNKNMNSFKDRIILECDLGSAAAFYAVLGYIGFLAAMCFVLAFLARDLPDNFNEAKYITFSMLIFCAVWITFIPAYISSPGKYTVAVEIFAILSSSFGLLFCIFAPKCYIILFKPEKNTKKHLMGKGPS
>Sterlet.locus281.1
CVKCPLEYKSNEQRDRCILKDIEFLSFGEIMGILLVIFSLIGACLTTAIAIVFFLYRDTPMVKANNSELSFLLLFSLALCFLCSLTFIGQPSEWSCMLRHTAFGITFVLCLSCVLGKTIVVLMVFRSTLPGNNMMKWFGPVQQRLSVFS
FTLIQVLICTLWLILSPPFPNKNMHSFKDRIILECDLGSAAAFYTVLGYIGFLAAMCFVLAFLARKLPDNFNEGKLITFSMLIFCAVWITFIPAYISSPGKYTVAVEIFAILASSFGLLFCIFVPKCFIILLKPEINTKKHLLGKMPS
>Sterlet.locus200.1
--------
MSNEQRDRCILKDIEFLSFGEIMGILLVIFSLIGACLTTAIAIVFFLYRDTPIVRANNSELSFLLLFSLALCFLCSLTFIGQPSEWSCMLRHTAFGITFVLCLSCVLGKTIVVLMAFRATLPGNNMMKWFGPAQQRLSVFSFTLIQVLI
CTLWLILSPPFPNKNMHSFKDRIILECDLGSAAAFYSVLGYIGFLAAMCFVLAFLARNLPDNFNEGKFITFSMLIFCAVWITFIPAYISSPGKYTVAVEIFAILASSFGLLFCIFVPKCFIILLKPELNTKKHLLGKMPS
>Sterlet.locus282.1
------------------------------
MGILLATFSLIGACLTTAIAIAFFRYRDTPMVKANNSELSFLLLFSLALCFLCALTFIGQPSEWSCMLRHTAFGITFVLCLSCVLGKTIVVLMVFRSTLPGNNTMKWFGPVQQRLSVFAFTFIQALTCTLWLAMSPPLPHKNMSSYKDR
IILECDLGSAAAFYAVLGYIGFLAAMCFVLAFLARKLPDNFNEAKYITFSMLIFCAVWITFIPAYISSPGKYTVAVEIFAILASSFGLLFCIFAPKCYIIILKPEKNTKKHLMGKVPS
>Sterlet.locus283.1
--------
MSNEQREQCILKDTEFLSFGEIMGILLVIFSLIGACLTTAIAIVFFLYRNTPIVRANNSELSFLLLFSLALCFLCSLTFIGQPSEWSCMLRHTAFGITFVMCISCVLGKTIVVLMAFRSTLPGNNTMKWFGPAQQRLSVFAFTFIQALI
CTLWLAMSPPLPHKNMNSFKDRIILECDLGSAVAFYAVLGYIGFLAAMCFVLAFLARKLPDNFNEAKFITFSMLIFCAVWITFIPAYISSPGKYTVAVEIFAILASSFGLLFCIFAPKCYIILLKPEKNTKKHLMGKVPS
>Sterlet.locus280.1
------------------------------
MGIVLVIFSLAGAFVTTAVAVVFFRFRDTPIVRANNSELSFLLLFSLALCFLCALTFIGQPSEWSCMLRHTAFGITFVMCIACVLGKTIVVLMAFRSTLPGNNMMKWFGPAQQRLSVLALTFNQALICTIWLITYPPFPNKNMRSYKDR
IILECDLGSAAAFYAVLGYIGFLAAMCFVLAFLARNLPDNFNEAKYITFSMLIFCAVWITFIPAYVSSPGKYTVAVEIFAILASSFGLLFCIFAPKCYIILLKPEKNTKKHLMGKTST
>Sterlet.locus201.1
CLKCPLENWPNAQRDQCTLKDIEFLSFGEIMGIVLVIFSLAGAFVTTAIAVVFFRFRDTPIVRANNSELSFLLLFSLALCFLCALTFIGQPSEWSCMLRHTAFGITFVLCLSCVLGKTIVVLMAFRATLPGNNMMKWFGPAQQRLSVFA
FTFIQALICTLWLITCPPFPNQNMRSYKDRIILECEVGSAVAFYTVLGYIGFVAAMCFVLAFLARKLPDNFNEAKFITFSMLIFCAVWITFIPAYISSPGKYTVAVEIFAILASSFGLLFCIFAPKCYIILLKPERNTKKHLMGKT-S
>Sterlet.locus202.1
------------------------------
MGIVLVIFSLAGAFVTTAIAVVFFRFRDTPIVRANNSELSFLLLFSLALCFLCSLTFIGQPSEWSCMLRHTAFGITFVLCISCVLGKTIVVLMAFRSTLPGNNMMKWFGPAQQRLSVFAFTFIQALICTLWLITFPPFPTKNMRSYKDR
IILECDLGSAAAFYAVLGYIGFLAAMCFVLAFLARKLPDNFNEAKYITFSMLIFCAVWITFIPSYISSPGKYTVATEIFAILASSFGLLFCIFGPKCYVILLKPEKNTKKHLMGKVQT
>Sterlet.locus203.1
------------------------------
MGIVLVICSLVGTCVTIAIAVVFFCYRETPIVKANNSELSFLLLFSLALCFLCSLTFIGQPSEWSCMLRHTAFGITFVLCISCVLGKTIVVLMAFRATLPGDNIMKWFGPIQQRLSVFAFTFIQGLICTLWLAISPPFPNKNIKSHKDR
IILECDLGSAAAFYAVLGYIGFLAAMCFVLAFLARKLPDNFNEAKYITFSMLIFCAVWITFIPAYISSPGKYTVAVEIFAILASSFGLLFCIFGPKCYVILLKPEKNTKKHLMGKIQS
>Sterlet.locus204.1
CLKCPVEYWSNKQRDECILKDIEFLSFGEIMGIVLVICSLVGTCVTIAIAVVFFCYRETPIVRANNSELSFLLLFSLALCFLCSLTFIGQPSEWSCMLRHTAFGITFVLCISCVLGKTIVVLMAFRATLPGDNIMKWFGPIQQRLSVFT
LTFIQGLICTLWLAISPPFPNKNIKSHKDRIILECEVGSAAAFCAVLGYIGFLAAMCFVLAFLARKLPDNFNEAKFITFSMLIFCAVWITFIPAYISSPGKYTVAVEIFAILASSFGLLFCIFGPKCYVILLKPEQNTKKHLMGKVQ-
>Sterlet.locus290.1
--------------------------------
MILVIFSVCGACVTITVAVVFLHNRDTPIVKANNSELSFFLLFSLALCFLCALTFIGQPSEWSCMLRHTSFGITFVMCISCVLGKTIVVLMAFRSTLPGNNIMKWFGPIQQRLSVFAFTFIQALICTLWLAMSPPFPSKNTSTYKDRII
LECDLGSAAAFYSVLGYIGFLAAMCFVLAFLARKLPDNFNEAKYITFSMLIFCAVWITFIPAYISSPGKYTVAVEIFAILASSFGLLFCIFAPKCYIIVLKPEKNTRKHMMGKMPS
>Sterlet.locus206.1
-----
MEYWSNVKRDQCILKDIEFLSFGEIMGMLLVIFSLTGSCITIAVAIVFFYYIDTPIVKANNSELSFLLLFSLALCFLCSLTFIGQPSSWSCMLSHMTFGVTFVLCISCVLGKTIVVLMAFRATLPGKNVMKWFGPTQQRLSVFTSTFIQ
CLICTLWLLISPPFPNRNMRHYKDKIILECDLGSAIAFYAVLGYIGFLSAMCFVLAFLARNLPDNFNEAKYITFSMLIFCAVWITFIPAYVSSPGKYTVAVEIFAMLASSFGLLFCIFAPKCYIILLKPEKNTKKHLMGRTST
>Sterlet.locus205.1
-----
MEYWSNVKRDQCILKDIEFLSFGEIMGMLLVIFSLTGSCITIAVAIVFFYYIDTPIVKANNSELSFLLLFSLALCFLCSLTFIGQPSSWSCMLSHMTFGVTFVLCISCVLGKTIVVLMAFRATLPGKNVMKWFGPTQQRLSVFTTTFIQ
CLICTLWLLISPPFPNRNMRHYKDKIILECDLGSATAFYAVLGYIGFLSAMCFVLAFLARNLPDNFNEAKYITFSMLIFCAVWITFIPAYVSSPGKYTVAVEIFAMLASSFGLLFCIFAPKCYIILLKPEKNTKKHLMGRTST
>Spotted_gar.locus135.1
CTKCPLEYWSNEDQTQCILKETEFLSFGENMGIVLTMFSLAGACLNLAIGFIYFHFRATPLVKANNSELSFLLLFSLTLCFLCSLTFIGQPSDWSCMLRHTAFGITFALCISCVLGKTLVVLMAFKAKMPGNSVPKCSGPL-
QRLSVFTCTIIQVLLCILWLALSPPFPYKNMEYFKNKIILECDLGSTVAFYAVLGYIGFLSAMCFVLAFLARKLPDNFNEAKFITFSMLIFCAVWITFIPAYISSPGKYTVAVEIFAILASSFGLLFCIFLPKCYIMLLKPEKNTRKHL
IGKMHS
>Reedfish.locus40417.1
------------------------------MGLLLTVISLFGAITSIIVLLIFYWHKDTPIVKANNSELSFLLLLSLTFCFLCSLAFTGEPSNWSCMLRHTAFGITFVCCVSCLLGKTVVVLMAFRSTIPGNNVKKYSGPV-
QRLCVFICPGIQLILCIFWLLLSPPFPKKNVSYFKEKILLECDVGSIVAFYAVMGYLGLLSILCFILAFLARKLPDNFNEAKYITFSMLIFCAVWITFVPAYISSPGKYTVAVEIFAILASSFGLLFCIFAPKCYVILFKPEANTKRNM
IGKMPS
>Sterlet.locus286.1
------------------------------MGILLATFSLFGTCVGIAIAFVFFLYRHTPIVKANNSELSFLLLFSLALCFLCALTFIGQPSEWSCMLRHTAFGITFVLCLSCVLGKTIVVLMAFKATLPGNNVKKHSGSVQWS-
SVFISTLIQVLICTLWLTLSPPFPKKTMTYYKDRIILECDLGSAAAFYAVLGYIGFLAAMCFVLAFLARKLPDNFNEAKYITFSMLIFCAVWITFIPAYISSPGKYTVAVEIFAILASSFGLLFCIFVPKCYIILLKPETNTRKHLMGK
MPS
>Reedfish.locus40490.1
-----------------------------
MMGMLLVLLSLLGASLTIFVTVVFYLNKDTPIVKANNSELSFLLLFSLILCFLCSLTFIGRPSEWSCMLRHTTFGITFVLCISCVLAKTVVVLMAFRATLPGRNVMRWFGPFQQRLSVCAFALVQVIICTIWLSKSPPFPNKNMQHSPE
IIILECHLGSVVAFYAVLGYVGILALFCFVLAFLARKLPDNFNEARFITFSMLIFCAVWLTFIPAYISSPGKYTVAVEIFAILASSFGLLFCIFIPKCYIILLKPQKNTKKNMMGKMPL
>Reedfish.locus27693.1
```

MGMLLVLLSLIGAFLSIIVAIAFFRNRDTPVVKANNSELSFLLLFSLTLCFLCSLVFIGPPSQWSCMLRHTAFGITFALCISCVLGKTLVVLMAFRATLPHNSIMKWFGPIQQRISVFTLTFVQLLICTLWLLISPPFPHKNSRTFKEK
IVFECDMGSTVTFFAVLGYIGFLSAVCFLLAFLARKLPDHFNEAKFITFSMLIFCAVWITFIPAYLNSLGKYTVAVEIFAILASSFGLLFCIFVPKMYILWIKPEKNTKKHIMGKI--
>Reedfish.locus27685.1
-MKCPLDYRSNKEKDACVLKDIEFLSYTEIISLVLEAFSFFGASATTAVMVIFYKHKETPIVKANNSELSFLLLFALTLCFLCSLAFIGEPTQWSCMLRHTAFGISFVLCISCVLGKTVVVLMAFQATLPGNNIMKWFGPTQQRLTVCT
FTAIQVLICTLWLSISPPSPIKNMKYFTEKIILECDIGSGIAFYAVLGYIGFLSVLCFVLAFLARKLPDNFNEAKFITFSMLTFCAVWVTFIPAYISSPGKYTVAVEIFAILASSFGLLSCIFVPKCYIILFRPENNSKKHLMSKMAS
>Reedfish.locus27677.1
CLSCPAEYKSNNVKDKCILKEIEFLSFAEIMGIVLVAFSFLGAGVTVAIIAVFFNHKDTPIVRANNSELSFLLLFSLTLCFLCSLTFIGEPSPWSCILRHTAFGITFVLCISCVLGKTIVVLMAFRATLPGSNIMKWFGPMQQRLSVCS
FTLIQVTICTLWLSVSPPFPIRNMKYYNDRIILECDLGSVVAFYSVLGYIGCLSILCFVLAFLARKLPDNFNEAKFITFSMLIFCAVWITFIPAYISSPGKYTVAVEIFAILASSFGLLFCIFIPKCYVILFKPEHNTKKHLMGKMPS
>Reedfish.locus27679.1

MGISLVVISLLGAGGTIIVASVFFSYKDTPIVRANNSELSFLLLFSLTLCFLCSLTFIGEPSQWSCILRHTAFGITFVLCISCVLGKTIVVLMAFRATLPGSNVMKWFGPTQQRLSVCSFTLIQVSICTLWLTISPPFPIKNMKHSTEK
IILECHVGSVVAFYAVLGYIGFLSAMCFILAFLARKLPDNFNEAKFITFSMLIFCAVWITFIPAYISSPGKYTVAVEIFAILASSFGLLCCIFIPKCYIILMKPEINTKKYLMGKMPS
>Reedfish.locus27671.1
CLMCPVEYKSNYLRDLCILKDTEFLAYEEIMGEILALFSLFGACVTLGIAAVFFIHKDTPIVRANNSELSFLLLFSLTLCFLCSLTFIGRPTDWSCLLRHTAFGITFVLCISCVLGKTIVVLMAFRATLPGSNVMKWFGPVQQRLSVFA
FTSVQILICTLWLTIVPPFPYKNLKSSTEKIILECDLGSVVAFYAVLGYIGFLSIICFVLAFFARKLPDNFNEAKFITFSMLIFCAVWITFIPVYVSSPGKYTVAVEIFAILASSFALLFCIFIPKCYIILLKPENNTKKHLMGKNQL
>Reedfish.locus27661.1

MEFISHELRHQCILKATEFLSSNETMGIILMIVSLLGACVTIAISIIFFCYKDTPIVRANNSELSFLLLFSLILCFLCSLTFIGQPSDWSCILRHTAFGITFAQCISCVLGKTIVVLMAFRATLPGSNLMKWFGPVQQRLSVFFFTFVQ
VLICAFWLIISPPFPIKNMKYYKEKIILECDAGSTVAFYCVLGYIGFLSTMCFVLAFLARKLPDNFNEAKFITFSMLVFCAVWITFIPAYISSPGKYTVAVEIFAILASSFGLLFSIFIPKCYVILLKPEINTRKHIMGKMPS
>Reedfish.locus27603.1
CLKCPLEYRSNDQRDQCILKDVEFLSFGEVMGIIPAIFSLTGASLTTSIICIFFYYRNTPIVKANNTELSFLLLFSLSLCFLCSLTFIGQLTGWSCMLRHTSFGITFELCVSCILGKTIVVLIMFRATKPGNNVMKWFGPRQQRLSVLG
LTLIQVFICIFWITLSPPFPSKNMRSYKEKIILECNVGSTLGFYIVLGYIGLLSIICFILSFLVRNLPDNFNEAKFITFSMLIFCVVWLAFIPAYISSPGKYTVAVEIIAILASSFGLLFCIFAHKCCIILLKPEKNTKKIVMGKMPT
>Reedfish.locus27617.1
CIKCPIEFKSNDQRDTCVLKEMEFLSFKEITGVLLVIFSLIGTFLTVSIAFLFFYYRNTPIVKANNSELSFLLLFSLTMCFLCSLTFIGEPTELSCMLRHTAFGITFALCISCILGKTIVVLMAFRATLPNRNVMKWFGPLQQRLSVLG
LTAVQVLICTLWLTLSPPFPNRNMQHYKEKIILECNLGSSLPFYAVLGYIGFLSSLCFILSFLARKLPDNFNEAKFITFSMLIFCMVWITFIPAYISSPGKFTVAVEIFAILASSFALLFCIFLPKCYIMTIHPEKNTKKQLMGKIPL
>Reedfish.locus27575.1
-MKCPPEYKSNQQRDKCILKEMEFLSFFEIMGILLITFSFFGTCLTMTIAVIFFWHKDTPIVKANNSELSFLLLFSLTLCFTCSLTFIGQPSDWSCILRHITFGITFVLCISCVLGKTIVVLMAFRATLPGSNVMKWFGPKQQRLCVSA
LTFIQVLICFLWLFLSPPFPVKNMEFYKDKVILECNLGSVAAFCAVLGYIGLLSVICFLLAFLARKLPDNFNEAKFITFSMLIFCTVWITFVPAYISSPGKYTVAVEIFAILASAYGLLFCIFTPKMYIILLKPEKNTKKNLMGKISS
>Reedfish.locus27573.1

MEFLSLFEIMGILLIIFSFFGACLTMTIAVIFFWHRDTPIVKANNSELSFLLLFSLTLCFTCSLTFIGQPSDWSCILRHVAFGITFVLCISCVLGKTIVVLMAFRATLPGSNVMKWFGPKQQRLCVSALTFIQVLICSLWLFLSPPFPV
KNMEFYKDKIILECNLGSVTAFSAVLGYIGLLSVICFLLAFLARKLPDNFNEAKFITFSMLIFCAVWITFIPAYISSPGKYTVAVEIFAILASGYGLLFCIFAPKMYVIIFRPQQNTKKHLMGKLPS
>Reedfish.locus27563.1
CILCPVEYKPSKGKNVCILKEVEFLSFGEILGIFLVLFSLAGATLAMTIAVVFFFYRHTPIVKANNSELSFLLLFSLVLCFLSSLIFIGQPSEWSCRLRHTAFGIAFVLCVSCVLAKTIVVLMAFRATLPGNNIMKWFGPTQQRLSVLS
MTFVQIVICTLWLVKSPPFPNKDFLTFKDKIIFECNVGSEATFYSVLGYIGFLSVLCFVLSFLARKLPDNFNEAKFITFSMLIFCAVWITFIPAYISSPGKYEVAVEIFAILASSFGLLFCIFTPKCFNILLRPERNSKRYLMDKR--
>Reedfish.locus40517.1
CILCPVEYKPSKGKNVCILKEVEFLSFGEILGIFLVLFSLVGATLAMTIAVVFFFYRHTPIVKANNSELSFLLLFSLVLCFLSSLIFIGQPSEWSCRLRHTAFGIAFVLCVSCVLAKTIVVLMAFRATLPGNNIMKWFGPTQQRLSVLS
MTFVQIVICTLWLVKSPPFPNKDFLTFKDKIIFECNVGSEATFYSVLGYIGFLSVLCFVLSFLARKLPDNFNEAKFITFSMLIFCAVWITFIPAYISSPGKYEVAVEIFAILASSFGLLFCIFTPKCFNILLRPERNSKRYLMDKR--
>Reedfish.locus27587.1

MGIILVVFSLAGASVSVAVAGIFYRYKDTPIVKANNSELSFLLLLSLSLCFLCSLTFIGEPSEWSCKLRHTTFGITFVLCISCVLGKTIVVLMAFRATLPGNNTMKWFGPTQQRLSVFTLTVIQIMICALWLIVFPPFPSRNLQHYKDR
IILECDLGSTTVFYAVLGYIGVLSIMCFVLSFLARKLPDNFNEAKYITFSMLIFCAVWITFIPAYISSPGKYTVAVEIFAILASSFALLFCIFAPKCYVILLKPEKNTKKHLMAKIPS
>Reedfish.locus27591.1

MGILLVIFSLLGTCMTIAVALVFFLYRDTPIVRANNSELSFLLLLSLALCFLCSLTFIGQPSDWSCMLRHTAFGITFVMCISCVLGKTIVVLMAFRATLPGNNIMKWFGPAQQRLSVFSLSFIQILICALWLIVSPPFPNRNMKHYKDR
IILECDLGSVAVFYAVLGYIGLLSSMCFLLSFLARKLPDNFNEAKYITFSMLIFCAVWVTFIPAYISSPGKYTVAVEIFAILASSFGLLFCIFFPKCHIILLKPENNTKKHLMGKMVS
>Reedfish.locus27596.1
CIKCPLEHTTNHQRDTCILKDIEFLSFGEIMGVLLVVFSLLGACITIAIALVFFLHRDTPIVKANNSELSFLLLFSLTLCFLCSLTFIGQPTDWSCMLRHTAFGIAFVLCISCVLGKTIVVLMAFRATLPGSNMMKWFGPTQQRLSVFS
LTFIQMLICTLWLILSPPFPNKNIVHYKDKIIMECDLGSTAVFYGVLGYIGFLSSMCFVLSFLARKLPDNFNEAKYITFSMLIFCAVWITFIPAYISSPGKYTVAVEIFAILASSFGLLFSIFIPKCYIILLKPERNTKKFLMGKI--
>Reedfish.locus27584.1

MGVILTVFTLLGACLTVSVAFVFFCHRKTPIVRANNSELSFLLLFSLTLCFLCSLTFIGQPSDWSCILRHTVFGITFVMCVSCILAKTIVVLMAFNATRPGANVMKWFGPLRQRLSVFILTFIQAFMCTVWLITSPPFFSSNIKSYKHK
VIFECDLGSTVAFYIMLGYIGVLSALCFMLSFLARKLPDNFNEAKFITFSMIIFCAVWLTFIPAYISSPGKYTVAVEIFAILASSFALLFCIFTPKCYIILLKPEQNTKKSVIGKK--
>Reedfish.locus27585.1

MGVILTVFTLLGACLTVSVAFVFFCHRKTPIVRANNSELSFLLLFSLTLCFLCSLTFIGQPSDWSCILRHTVFGITFVMCVSCILAKTIVVLMAFNATRPGANVMKWFGPLRQRLSVFILTFIQAFMCTVWLITSPPFFSSNIKSYKHK
VIFECDLGSTVDFYIMLGYIGVLSALCFMLSFLARKLPDNFNEAKFITFSMLIFCAVWLTFIPAYISSPGKYTVAVEIFAILASSFALLFCIFTPKCYIILLKPEQNTKKSVIGKK--
>Reedfish.locus27586.1

MGVILTVFTLLGACLTVSVAFVFFCHRKTPIVRANNSELSFLLLFSLTLCFLCSLTFIGQPSDWSCMFRHTVFGITFVMCISCILAKTIVVLIAFNATRPGANVMKWFGPSQQRLSVFILTFIQAFLCMVWLTISPPFFSLNINSYKHK
IIFECDLGSTVAFYIMLGYIGVLSALCFVLSFLARKLPDNFNEAKFITFSMIIFCAVWLTFIPAYISSPGKYTVAVEIFAILASSFALLFCIFSPKCYIILLKPEQNTKKSVIGKK--
>Reedfish.locus27579.1
-MACPLEFKSNDQRNQCILKNTEFLSFGELMGTLLVIFSLVGTCITLVTGIIFYFYRDTPIVKANNSELSFLLLFSLTLCFLCSLTFIGEPTDWSCMLRHTSFGITFVLSISCILAKTIVVLMAFRATLPGNNVMKWFGPTQQRLSVFA
LTLVQAFLCISWLIISPPFPNRNMQHYKDKIILECNFGSTFAFYAMLGYIGLLSTMCFVLSFLARKLPDNFNEAKYITFSMLIFCAVWITFIPAYISSPGKYTVAVEIFAILASSFGILFCFFSPKCYIILLKPEKNTKKYLMGKTTS
>Reedfish.locus27613.1

MGILLVILSILGTMVTLTVATVFFKYRQTPVVKANNSELSFLLLFSLTFCFLCSLTFIGEPSDWSCMLRHTAFGITFVLCISCILGKTLVVLMAFRATLPGNNIMKLFGPRQQRFSVFSLTAIQILICALWLILSPPFPIKNMQHYNDK
IILECDLGSAVTFYSVLGYIGFLSIMCFILAFLARKLPDNFNEAKFITFSMLIFCAVWLAFIPAYISSPGKYTVAVEIFAIVASSFGLLFCIFAPKCYIILLKPEKNTKKFLLGKNQP
>Reedfish.locus40453.1

MGILLVILSILGTMVTLTVATVFFKYRQTPVVKANNSELSFLLLFSLTFCFLCSLTFIGEPSDWSCMLRHTAFGITFVLCISCILGKTLVVLMAFRATLPGNNIMKLFGPRQQRFSVFSLTAIQILICALWLMLSPPFPIKNMQHYNDK
IILECDLGSAVTFYSVLGYIGLLSIMCFILAFLARKLPDNFNEAKFITFSMLIFCSVWLAFIPAYISSPGKYTVAVEIFAILASSFGLLFCIFAPKCYIILLKPEKNTKKVLLGKNQP
>Reedfish.locus27599.1
CIKCSLEYTANEQSDQCTLKEVEFLSSGEIMGMLLTILAILGACKTISIAVVFYYYRDTPIVKANNSELSFLLLFSLTCCFLCSLTFIGRPSDWSCMLRHTAFGITFVLCVSCVLGKTLVVLMAFRATIPSNNVMKYFGPGQQRLSVCI
LTAIQILICAMWLMISPPFPMKNMQHYKNKIILECDLGSAITFYGVLGYIGFLSVICFLLAFLARKLPDNFNEGKFITFSMVIFCAVWLTFIPAYVSSPGKYTVAVEIFAILASSFGLLFCIFLPKCYIILLKPERNTKKFVMGKS--
>Reedfish.locus27597.1
CLKCPIEYKSNDLKSQCILKEIEFLAFEETTGILLVFFSLLGTCFTMAVTMVFLYYRDTPIVRANNSELSFLLLFSLSLCFLCSLTFVGQPSHWSCMLRHTVFGITFVLCISCVLGKTIVVLMAFRATLPGNNIMKWFGPSQQRFCVFI
LTFIQILICALWIIVSPPFPSKNTEHYKDRIILECSLGSTITFCAVLGYIGLLSTMCFILAFLARKLPDNFNEAKFITFSMLIFCAVWITFIPAYISSPGKYTVAVEIFAILASSFGLLFCIFVPKCYVILLKPHKNTKKFLMGKVQS
>Reedfish.locus27608.1
CLKCPIEYKSNDLKSQCILKEIEFLAFEETTGILLVFFSLLGTCFTMAVTMVFLSYRDTPIVRANNSELSFLLLFSLSLCFLCSLTFIGQPSHWSCMLRHMVFGITFVLCISCVLGKTIVVLMAFRATLPGNNIMKWFGPSQQRFCVFI
LTFIQILICALWIILSPPFPSKNTEHYKDRIILECSLGSTITFCAVLGYIGLLSTMCFILAFLARKLPDNFNEAKFITFSMLIFCAVWITFIPAYISSPGKYTVAVEIFAILASSFGLLFCIFVPKCYVILLKPHKNTKKFLMGKVQS
>Reedfish.locus27638.1
-MKCPLEYRANEEKDQCILKDIEFLSYDEIMGMILIIFALLGASLTSVVAVVFFKFRDTPIVKANNSELSFLLLFSLTLCFLCSLTFIREPTQWSCMLRHTAFGITFVLCISCVLGKTIVVLMAFRATMPGSKIMKCFGPTQQRLSVLS
LTLLQTLTCIIWLVKYPPLPHKNMNSNKEVIILECEMGSPVAFYLVLGYIGVLAVMCFVLAFLARKLPDNFNEAKFITFSMLVFCAVWITFIPSYISSPGKYTVAVEVFAILASSFGLLLCIFVPKCYIVLLKPDRNSKKHVISKMQS
>Reedfish.locus27640.1
-MKCPLEYRANEEKDQCILKDIEFLSYDEIMGMILIIFALLGASLTSVVTVVFFKFKDTPIVKANNSELSFLLLFSLTLCFLCSLTFIGEPTQWSCMLRHTAFGITFVLCISCVLGKTIVVLMAFRATVPGSKIMKCFGPTQQRLSVLS
LTLLQTLTCIIWLVKYPPLPHKNMNSNKEVIILECEMGSAIAFYLVLGYIGVLAVMCFVLAFLARKLPDNFNEAKFITFSMLVFCAVWITFIPSYISSPGKYTVAVEVFAILASSFGLLLCIFVPKCYIVLLKPDRNSKKHVIGKMQS
>Reedfish.locus27641.1
-MKCPLEYRSNEEKDQCILKDVEFLSHDEIMGMILIIFALLGVLLTSTVTAVFFKFRDTPIVKANNSELSFLLLFSLTLCFLCSLTFIGEPTQWSCMLRHTAFGITFVLCISCVLGKTIVVLMAFRATMPGSKVMKYFGPTQQRLSVLT
FTLIQTLTCIIWLVKYPPFPQKNINSNKEIIILECEMGSTVAFYIVLGYIGVLAFVCFALAFLARKLPDNFNEAKFITFSMLVFCTVWITFIPSYISSPGKYTVAVEVFAILSSSFGLLLCIFVPKCYIILLKPEKNFKKHVMGKMQS
>Reedfish.locus27646.1
-MKCPLEYRSNEEKDQCILKDVEFLSHDEIMGMILIIFALLGVLLTSTVTAVFFKFRDTPIVKANNSELSFLLLFSLTLCFLCSLTFIGEPTQWSCMLRHTAFGITFVLCISCVLGKTIVVLMAFRATMPGSKVMKYFGPTQQRLSVLT
FTLIQTLTCIIWLVKYPPFPQKNINSNKEIIILECEMGSTVAFYIVLGYIGVLAFVCFALAFLARKLPDNFNEAKFITFSMLVFCTVWITFIPSYISSPGKYTVAVEVFAILSSSFGLLLCIFVPKCYIILLKPEKNFKKHVMGKMQS
>Reedfish.locus27659.1
-MKCPLEYWANEEKDQCILKDIEFLSYDEIMGMILIIFALFGALLTSMITVVFFKFRDTPIVKANNSELSFLLLFALILCFLCSLTFIGEPTQWSCMLRHTAFGITFVLCISCVLGKTTVVLIAFRATMPGSNVIKCFGPTQQRLSVLT
FTLLQTLTCIIWLVQYPPFPQKRINSDKEIIILECNNGSTVIFYIVLGYIGLLAVICFFLAFLARKLPDNFNEAKFITFSMLIFCAVWITFIPSYISSPGKYTVAVEIFAILASSFGLLLCIFVPKCYIILLKPDKNSKKHLMGKIQS
>Reedfish.locus27649.1
-MTCPLEYRANEEKDQCILKDIEFLSHDEIMGMILIIILALLGALITSMVTVVFFKFRDTPIVKANNSELSFLLLFSLTLCFLCSLTFIGEPTQWSCMLRHTAFGITFVLCISCVLGKTIVVLMAFRATMPGSNIMKCFGPTQQRLSVLT
FTLIQTLICIIWLVNYPPFPKKNVNSNKEIIILECEMGSTVAFYIVLGYIGLLSVMCFVLAFLARKLPDNFNEAKFITFSMLVFCTVWITFIPSYISSPGKYTVAVEVFAILASSFGLLLCIFVPKCYIILLKPDKNSKKHLMGKMQT
>Reedfish.locus27651.1
-MTCPLEYRANKEKDQCILKNIEFLSYDEIMGMILIIILAFLGALLTSTVTVVFFKFRDTPIVKANNSELSFLLLFSLTLCFLCSLTFIGEPTQWSCMLRHTAFGITFVLCISCVLGKTIVVLMAFRATMPGNNIMKCFGPTQQRLSVLT
FTLIQTLICIIWLAKYPPVPQKNVNSNKEIIILECEMGSTVAFYIVLGYIGLLSVMCFVLAFLARKLPDNFNEAKFITFSMLVFCSVWITFIPSYISSPGKYTVAVEVFAILASSFGLLLCIFIPKCYIILLKPDKNSKKHLMGKNTL
>Reedfish.locus27654.1

MGMMLVIFALLGASLTSVVTVVFFKFRDTPIVKANNSELSFLLLFSLTLCFLCSLTFIGEPTQWSCMLRHTAFGITFVLCISCVLGKTIVVLMAFRATMPGSNIMKCFGPTQQRLSVLTFTLIQTLICIIWLVKYPPFPQKNVNSNKEI
IILECEMGSTVAFYIVLGYIGLLSVMCFVLAFLARKLPDNFNEAKFITFSMLVFCSVWITFIPSYISSPGKYTVAVEVFAILASSFGLLLCIFIPKCYIILLKPDKNSKKHLMGKVQA
>Reedfish.locus27657.1

MGRILIIFALLGALLSSTVTAVFFKFRDTPIVKANNSELSFLLLFSLTLCFLCSLTFIGEPTQWSCMLRQTAFGITFVLCISCVLGKTIVVLMAFRATMPGSNVMKCFGPTQQRLSVLSFTLLQTLTCIIWLVKHPPFPSKNISSNKEV
IILECEMGSTVAFYIVLGYIGLLSVMCFVLAFLARKLPDNFNEAKFITFSMLVFCSVWITFIPSYISSPGKYTVAVEVFAILASSFGLLLCIFIPKCYIILLKPDKNSKKHLMGKM--
>Reedfish.locus40514.1
CTKCPLEYQANEEKDQCILKDIEFLSYNEIMGMILIIFALLGTLLTSTVTVVFFKFRDTPIVKANNSELSFLLLFSLTLCFLCSLTFIGEPTQWSCMLRHTAFGITFVLCISCVLGKTIVVLMAFRATMPGSNIMKCFGPTQQRLSVLT
FTVLQTLICIVWLVKYPPFPQKNMNFNKKLIILECEMGSTVAFYIVLGYIGLLSVMCFVLAFLARKLPDNFNEAKFITFSMLVFCTVWITFIPSYISSPGKYTVAVEVFAILASSFGLLLCIFAPKCYIILLKSDKNSKKHLMGKTQS
>Reedfish.locus27647.1
CMKCPLEYRANEEKDQCILKDIEFLSYDEIMGMILIIFALLGTLLTSFVTAVFFKFRDTPIVKANNSELSFLLLFSLTLCFLCSLTFIGEPTQWSCMLRHTAFGITFVLCISCVLGKTIVVLMAFRATMPGSNIMKCFGPTQQRLSVLT
FTLVQTLTCIIWLVKYPPLPHKNMNSNKEIIILECDIGSTVGFYIVLGYIGLLSVICFFLAFLARKLPDNFNEAKFITFSMLVFCTVWITFIPSYISSPGKYTVAVEVFAILASSFGLLFCIFAPKCYIILLRPDKNSKKHLIGKVQA
>Reedfish.locus27637.1

```
------------------
MKDTEFLSYEEIMGKILIIFSLLGAFLTLAVIAVFFKFRDTPIVKANNSELSFLLLFSLTLCFLCSLTFIGEPTEWSCMLRHTAFGITFVLCISCVLGKTIVVMMAFRATRPGSDIMKYFGPTQQRISVLIFTLIQALTCIIWLAVSPP
FPKKNMKSNKDRIILECDIGSIIAFYTVLGYIGLLSVMCFVLAFLARKLPDKFNEAKFITFSMLVFCAVWVTFIPSYISSPGKYTVAVEVFAILASSFGLLLCIFAPKCYLILLKPGKNSKKYLMGKMNL
>Reedfish.locus27703.1
------------------------------

MGILMAIFSLSGACLTAATALIFFHYRNTPIVKANNSELSFLLLFSLLLSFLCSLIFIGEPSNWSCVLRHTAFGISFVLCISCVLGKTIVVLVAFRATIPGSNIMKWFGPAQQRLCVSAFTLIQVLICTMWLMLFPPFPNKNAKHYKDR
IILECDLGSTAAFYTVLGYIGFLSIVCFILAFLGRRLPDNFNEAKYITFSMLIFCAVWITFIPAYISSPGKYTVAVEIFAILASSFGLLFCIFAPKCYIILIKPDRNTKKHLMGKITS
>Reedfish.locus27697.1
------------------------------

MGILMAIFSLSGACLTAATALIFFHYRNTPIVKANNSELSFLLLFSLLLSFLCSLIFIGEPSNWSCVLRHTAFGISFVLCISCVLGKTIVVLVAFRATIPGSNIMKWFGPAQQRLCVSAFTLIQVLICSVWLILFPPFPNKNTKYYKDR
IILECDLGSTTAFYTVLGYIGFLSIVCFILAFLGRKLPDNFNEAKYITFSMLIFCAVWITFIPAYISSPGKYTVAVEIFAILASSFGLFFCIFAPKCYIILIKPDRNTKKHLMGKLPS
>Reedfish.locus27665.1
CIKCPLEYKSNRQRDQCILKEIEFLSFEEIMGTVLVIFSLLGTCATIAIASVFFHFKETPIVKANNSELSFLLLFSLVLCFLCSLTFIGEPSDWSCMLRHTAFGITFVLCISCVLGKTIVVLMAFRSTLPGNNMMKWFGPIQQKFTVFS
FTLIQAIVCLIWLLVSPPFPYQNTSYYVEKIILECNLGSSVAFYAVLGYIGFLSAICFVLAFFARKLPDNFNEAKYITFSMLIFCAVWITFIPAYISSPGKYTVAVEIFAILASSFSLLFCIFAPKCYIILLKPEKNTKKHLMGKPT-
>Reedfish.locus27663.1
-MNCPLEYKSNKQRDLCILKEIEFLSCDEIMGVVLVIFSLLGTCATVATASVFFHFRQTPIVKANNSELSFLLLFSLSLCFLCSLTFIGEPSNWSCMLRHTAFGITFVLCISCVLGKTIVVLMAFRSTLPGSNVMKWFGPHQQRFTVFA
FTLIQAIICLLWLILSPPFPYQNTTHYVEKIILECALGSTVAFYAVLGYIGFLSAISFVLAFLARNLPDNFNEAKYITFSMLIFRAVWITFIPAYVSSPGKYTVAVEIFAILVSSFALLFCIFAPKCYIILLKPENNTKKKLIGKSTI
>Reedfish.locus40515.1
CVRCPLEYKSNEQRTECILKEIEYLSFEELMGKLLMTIALLGAISTFAVAVVFFKYRETPVVKANNSELSFLLLFSLMLCFLCSITFIGEPSDWSCMLRHTVFGITFVLCISCVLGKTIVVLMAFRATLPGSNIMKWFGPMQQRLSVFM
CTLIQVLICILWLLLTPPFPNKNTKHYKDKIILECDLGSTIAFYAVLGYIGFLSAMCFILAFLARKLPDNFNEAKYITFSMLIFCAVWITFIPAYISSPGKYTVAVEIFAILASSFSLLFCIFAPKCYIILLKPERNTKKYLMSKGT-
>Reedfish.locus27565.1
CVRCPLEYKSNEQRTECILKEIEYLSFEELMGKLLMTIALLGAISTFAVAVVFFKYRETPVVKANNSELSFLLLFSLMLCFLCSITFIGEPSDWSCMLRHTVFGITFVLCISCVLGKTIVVLMAFRATLPGSNIMKWFGPMQQRLSVFM
CTLIQVLICILWLLLTPPFPNKNTKHYKDKIILECDLGSTIAFYAVLGYIGFLSAMCFILAFLARKLPDNFNEAKYITFSMLIFCAVWITFIPAYISSPGKYTVAVEIFAILASSFSLLFCIFAPKCYIILLKPERNTKKYLMSKGT-
>Reedfish.locus27576.1
------------------------------

MGMLLVTLSLLGTCITMAVAVVFFHYRDTPIVKANNSELSFLLLFALTLCFLCSITFIGEPSEWSCMLRHTAFGITFVLCISCVLGKTIVVLMAFRATLPGSNIMKWFGPTQQRLTVCTFTLIQVVICSIWLITAPPFPNKNMKYFKDR
IILECDLGSTLAFYSVLGYIGFLSGMCFVLSFLARKLPDNFNEAKFITFSMLTFCAVWITFIPAYISSPGKYTVAVEIFAILASSFGLLFCIFSPKCYVILLKPENNTKKHLMGKTSA
>Reedfish.locus27711.1
--------------------------------

MTLVTFSLIGAAVSVSVAVIFFSYRDTPIVKANNSELSFLLLFSLTLCFLCSLTFIGVPSDWSCMLRHTAFGITFVLCISCILGKTVVVLVAFRATLPGNNMMKWFGPTQQRISVCCFTLIQVMICTLWLILSPPSPSKNIQSYKEKII
LECDLGSKIAFYTVLGYIGLLSAMCFLLSFLARKLPDNFNEAKYITFSMLIFCAVWITFIPAYISSPGKYTVAVEIFAILASSFGLLLCIFAPKCYVILFKPNKNTKKHLMSKN--
>Reedfish.locus27559.1
------------------------------

MGTLLVILSLLGSSITFLVVLVFFYYKDTPIVRANNRELSFLLLLSLALCFLCALTFIGEPSTWSCKLRHTAFGITFVLCISCVLGKTTVVLMAFRATLPGSNTMKWFGPTQQRLSVFACTFIQILICSFWLIISPPFPNKNVISHKDK
IIFECDVGSTAAFYAALGYIGFLSSICFILAFLARKLPDNFNEAKFITFSMLLFCAVWITFIPAYISSPGKFTVAVEIFAILASSFGLLFCIFAPKCYIILLKPEENTKRHLMGKNIA
>Reedfish.locus27699.1
------------------------------

MGAFLMVLSLMGAGMTIGITFIFYCYRNTPIVKANNSELSFLLLFSLILSFLCSLTFIGRPSDWSCMLRHTAFGIVFVLCISCVLGKTIVVIMAFKATLPGNNVMKWFGPNQQRLCICIFTLIQVLICTLWLLLSPPFPNSNTAYYKEK
IILECDIGSALAFYAVLGYIGFLSALCFILAFLGRKLPDNFNEAKYITFSMLIFCAVWITFIPAYISSPGKYTVAVEIFAILASSFGLLFCIFAPKCFIILVKPEKNTKKYLMGKRP-
>Reedfish.locus27707.1
------------------------------

MGAFLMVLSLMGAGVTIGITFVFYCNRSTPIVKANNSELSFLLLFSLILSFLCSLTFIGRPSDWSCMLRHTAFGIVFVLCISCVLGKTIVVIMAFKATLPGNNVMKWFGPNQQRLCICIFTLIQVLICTLWLLLSPPFPNSNTAYYKEK
IILECDIGSALAFYAVLGYIGFLSALCFILAFLARKLPDNFNEAKYITFSMLIFCAVWITFIPAYISSPGKYTVAVEIFAILASSYGLLFCIFAPKCFIILVMPEKNTKKYLMGKIP-
>Reedfish.locus40427.1
------------------------------

MGILLLSLSLIGSGISIFVAVVFYYFKDTPIVRANNSELSFLLLFSLTLCFLCSLTFIGQPSEWSCMLRHTAFGISFVLCISCVLGKTIVVLMAFKATLPGSNVMKWFGPSQQRLSVFFFTLVQVLICTIWLIISPPFPFKNMKYYKEK
IILECHVGSSIAFYSVLGYIGFLSVICFVLAFLARKLPDNFNEAKFITFSMIIFCAVWITFIPAYVSTPGKYTVAVELFAILASSFALLLCIFAPKCVVIILKPEQNTKKNLIGKMPS
>Reedfish.locus40424.1
------------------------------

MGLLLLTLALVGTGISIIVAGVFYHYKDTPIVRANNSELSFLLLFSLTLCFLCSLTFIGEPSIWSCMLRHTAFGISFVLCISCVLGKTVVVLMAFKARLPGSNVMKWFGPTQQRLSVWMFTLVQVLICAIWLIISPPFPNRNMKYYKDK
IILECNVGSQIAFYLVLGYIGFLSVICFVLAFLARKLPDNFNEAKFITFSMLIFCAVWITFIPAYISSPGKYTVAVELFAILASSFALLFCIFAPKCVVIILKPEKNTKKGLMTKTSP
>Small-eyed_rabbitfish.locus138.1
CLTCHLEYRSNDKRDHCLLKEIEFLSFEETLGIVLVTLALLGVCLTISIFTAFYVHRDTPIVEANKSELSILLLVALALCFLCSVTFIGEPSAWSCRLRHMAFVITFVWCISCVLGKTIVVVMAINATIPSNNMMKWFGPTQQRLSVFT
LTLIKCLICTVWLSLSPPYPLKNSEYFTERIVLECRVGSALAFYCVLGYIGLLSCVCFRIAFLARRLPENFNEAKHITFSILIFCAVWITFIPACISSAGKYTVAVEIFAILALSFGLLACEFDPKCYIIPLKKEENTKKHMMVKWLP
>Small-eyed_rabbitfish.locus291.1
CLTCPLEYRSNDKRDHSLLKEIEFLSFDETLGIVLVTLALLGVCLNISVLTAFYVHRDTPIVKANKSELSFLLLVALALCFLCSVTFIGEPSVWSYRLRHMAFGITFVLCISCVLGKTIVVVMAFNATHPSNNMMKWFGPTKQRLSVFI
LTLIQCFICTVWLSLSPPYPLKNSEYFTERIVFECHVGFAVAFYCLLGYIGLLSCVCFGVAFLARRLPDNFNEAKHITFSMLIFCAVWITFIPAYVSSPGKY----------------------------------------------
>Small-eyed_rabbitfish.locus396.1
CLTCPLEFRSNDKRDHCLLKEIEFLSFEETLGILLVTLALLGVCLTISVFAAFYVHRDTPIVKANNSELSFLLLVALALCFLCSVTFIGEPSVWSCRLRHMVFGITFVLCISCVLGKTIVVVMAFNATLPSNNMMKWFGPTQQRLSVFI
LTLIQCLICTVWLSLSPPYPLKNSEYFTERIVFECHVGSAVAFYCVLGYIGLLSCVCFGVAFLARRLPDNFNEAKHITFSTLIFCAVWITFIPAYVSSPGKYTVAVEIFAILASSFGLLACIFAPKCYIILLKKEENTKKHMMVKVAS
>Small-eyed_rabbitfish.locus613.1
CLTCPLEYRSNDKRDHCLLKEIEFLSFEETLGIVLVTLALLGVCLTISVFTAFYVHRYTPIVKANNSELSFLLLVALALCFLCSVTFIGEPSVWSCRLRHMAFGITFVQCISCVLGKTIVVVMAFNATLPSINMMKWFGPIQQRLSVFI
LTLMQCLICTAWLSLSPPYPMKNSEYFTERIEFECHVGSAVAFYCVLGYIGLLSCVCFGVAFLARQLPDNFNEAKHITFSMLIFCAVWITFIPAYVSSPGKYTVAVEIFAILASSFGLLACI--------------------------
>Small-eyed_rabbitfish.locus302.1
----------------CLLKEIEFLSFEETLGIVLVTLALLGVCLTISVFTAFYVHRDTPIVKANNSELSFLLIVALALYFLCSVTFIGEPSVWSCGLRHMAFGITFVQCISCVLGKTIVVVMAFNATLPNNNMMKWFG-
TQQRLSVFILTLIQCLICTVWLSLSPPYPLKNSEYFTERIVFECHVGSAVAFYCVLGYIGLLSCVCFGVAFLARRLPVNFNEAKHITFSMLIFCAVWTTFIPAYVSSPGKYTVAVEIFAILASSFGLLACICAPKCYIILLKKEKNTKK
HMMCKVAS
>Small-eyed_rabbitfish.locus226.1
CIECADEYRSNDKRDHCLLKEIEFLSFDETLGIVLVTLAMLGVCLTISVFTAFHVHRDTPIVTANNSELSFLLLVALALCFLCSVTFIGEPSVLSCRLRHIAFGITFVLCISCVLGKTIVVLMAFNATLPSNNMMKWFGPTQQSLSVFI
LTLIQCLICTVWLSLSPPYPLKNSEYFTERIVFECHVGSAVAFYCVLGYIGLLSFVCFGVAFLARRLPDNFNEAKHITFSMLIFCAVWITFIPAYVSSPGKYTVAVEIFAILLSSFGLLACIFAPKCYIILLKKEENTKKHMMVKVAS
>Small-eyed_rabbitfish.locus366.1
CLTCALDYRSNDKRDHCLLKEIEFLSFEETLGIVLVTLAMLGVCLTISVFTAFYVHRNTTIVKANNSELSFLLLVALALCFLCSVTFIGEPSVWSCRLRHMAFGITFVLCISFVLGKTIVVLMAFNATLPSNNIMKWFGPTQQYLSVFI
LNLIQCLICTVWLSLSPPYPQKNSEYFTERIVFECHLGSALAFYCVLGYIGLLSCVCFGVAFLARRLPDNFNEAKHFTFSMLIFCAVWITFIPAYISSPGKYTVAVEIFAILASSFGLLACIFAPKCYIILLKKEENTKKHMMGKVAS
>Small-eyed_rabbitfish.locus209.1
--------------------------

FEETLGIVLVTLALLGVCLTISVFTMFYVHRDTPVVKANDSELSFLLLVTLALCFLYSVTFIGEPSVWSCRLRHMAFGINFVLCISSVLGKTIVVVMAFNATLPSNNMMKWFGPTQQSLSVFILTLLQCFMCTVWLSLSPPYPLKNSEY
FTERIVFECHVGSAVAYYCVLGYIGLLSCVCFGVAFLARRLPDNFNEAKHITFSMLIFCAVWITSIPVYVSSPGK-----------------------------------------------
>Small-eyed_rabbitfish.locus60.1
CLTCPLEYRSNDKRDHCLLKEIEILSFEETLGIVLVTLALLGVCLT-
SVFTAFYVHSFSPIVKANNSELSFPLLVALALCFLCSVTFIGEPVVWSCRLRHIDFGITFVLCISCVLGKTIVVVMAFNATLPSNNMMKWFGPTQQPLSVFILNLIQCLICTVWLSLSPPYPLKNSEYFTERIVFECHVGSSVAFYCVL
GYIGLLSCACFGFAFLARRLPDNINEAKHITFSMLIFCAVWITFIPAYVSSPGKYTVAVEIFAILPSSFRLLASIFTSKCYIILLKKEENTKKHMMGKVAA
>Small-eyed_rabbitfish.locus48.1
CITCPLEYRSNDQRDHCLLKEIEFLSFEETLGIVLVTLALLGVCLTISVFTAFYVHRDTPIVKANNSDLSFLLIVALALCFLCSVTFIGEPSVWSCRLRHMAFGITFVLCISCVLGKKIVVVMAFNATLPSNNMMKCFGPTQQRLSVFI
LTLIQCLICTVWLSLSPPYPLKNSEYFTERIVFEYHVGSAVAFYCVLGYIGLLSCICFGVAFLARRLPDNVNEAKHITFSMLIFCAVWITFIPAYVSSPGKYTVAVEMFAILASSFGLLTCIFAPKCYIILLKKEENTKQHMMGKVAS
>Small-eyed_rabbitfish.locus232.1
CLSCPLEYRSNDKRDHCLLKEVEFLSFEETLVIVLITLGLLGVCLTISVFTAFYVHRDTPMVKANNSELSFLLLVALTLCFLCSVTFIGEPSVWSCRLRHMAFGITFVLCISCVLGKTIVVVMAFNAKLPSNNMMKWFGPTQQRLSVFI
LTLIQCLICTVWLSLSPPYPLKNTEYFTERIVFECHVGSAVAFYCVLGYIGLLSFVCFGVAFLARRLLDNFNEAKHITFSMLIFCAVWITFIPAYVSSPGKYTVAVEIFAILASSFGLLACIFAPKCYIILLKKEENTKQHMMGKVAS
>Small-eyed_rabbitfish.locus263.1
--------------------------------

IVLVTLALLGVCLTISVFTAFYVHRETPIVKANNSELSFLLLVALALCFLCSVTFIGVPSVWSCRLRHMAFGITFVLCISCVLGKTIVVVMAFNATLPSNNMMKWFGPTQQRLGVFILTLIQCLICTVWLSLSPPYPLKNSEYFTEKIV
FECHVGSAVAFYCVLGYIGLLSCVCFGVAFLARRLPDNFNEAKHITISMLIFCAVWITFIPAYVSSPGKYTVAVEIFAILASSFGLLACISTPKCYIILLKKEENTKNHMMGKVAS
>Small-eyed_rabbitfish.locus179.1
-------------------------------

GIVLVTLALLGVCLTISVFTAFYIHRDTPIVKANNSELSFLLLVALALCFLCSATFIGEPSVWSCRLRHMAFGITFVLCISCVLGKIIDVVMAFNATLPSNKMMKWFGPTQQRLSVFILTLIQCLICTVWLSSSPPYPLKNSEYFTESI
VFECHVGSAVAFYCVLGYIGLLSCVCFGVAFLARRLPDNFNEAKHITFSMLIFCAVWITFIPAFVSSPGKYTMAVEIFAILASSFGLLACIFAPKCYIVLLKKEENTKKHMMGKVAS
>Small-eyed_rabbitfish.locus519.1
------------------

LKEIEFLSFEETLGIVLVTLALLGVCLTISVFTAFYVHRDTPIVKANISELSFLLLVALALCFLCSVTFIGEPSVWSCRLRHMAFGITFVLGISCVLGKTIVVVMAFNATIPSNNMMKWFGPTQQRLSVFILTLIQCLICTVWLSLSPP
YPLKNSEYFTERIVFECHVGSAVAFYCVFGYIGLLSCVCFGVAFLARRLPDNFNEVKHITFSMLIVCAVWITFIPAYVSSPGNYTVAVEIFAILASSFGLLASIFAPKCHNIQLKKEENTKKHMMGKMPS
>Small-eyed_rabbitfish.locus539.1
CLTCPLEYQSNDKRDHCLLKEIEFLSFEETLEIVLVTLALLGVCLTISVFTAFYVHRGTPIVKANNSELSFLLLVALALCFLCSVTFIGEPSVWSCRLSHIGIAFYFVLYISCVFGTKVVVIMAFNATIPSNNIMKWFGPTQQRLSVFI
LTLIQCLICTVWLSLSSPYPLKNSEYFTERIVFECHVGSALTFYCVLGYIGLLSCVCFGVAFLARRLPDNLNEAKHITFTRLIVCAVWITFVPSYVNSPGKYTVAVEIFAILASNFGLLACIFAPKCYIILLKKEENTKKHVMGKMVC
>Small-eyed_rabbitfish.locus330.1
--------------------------------------------

LTISVFTAFYVHRDTPIVKANNSELSFLLLVALALCFLCSVTFIGEPSVWSCRLRHMAFGITLVLCISCVLGKTIVVVMAFNATLPCNNMMKWFGPTHQRLSVFILTLIQCLICTVWLSLSPPYPLKNSEYFTERIVFELHVGSPVAFY
CVLGYIGLLSCVCFGVAFLARRLPDNFNEAKHITFSMLIFCAVWITFIPAYVSSPGKYTVAVEIFAILASSFGLLACIFAPKCHIILLKKEENTKKLMMGKMAS
>Small-eyed_rabbitfish.locus95.1
------KLVTSLVR---
IHKAINSFLNSQTLGIFLVTLALLGVCLTIRVFTAFNVHRDTTIVKANNSELSFLLLVALALCFLCSITFIGEPSVWSCRLRHMAFCIIFVLCISCVLGKTIVVVMAFNATLPNNNMMKWFGPTQQLLSVFILTLIQCLICTVWLSLSP
PYPLKNSEYFTERIVFECHVGSAVAFYCVLRYIGLLSCVCFGVAFLTRQLPDNFNEAKHITFSMLIFCAIWITFIPAYVSSPGKYT-------ILASSVGILACIFAPKCYIILLKKEENTKKHLMGKMAS
>Small-eyed_rabbitfish.locus401.1
------------------------------

LGIVLVTLALLGVCLTISVFTAFYVHRDTPIVKANNSELSFLLLVALALCFLCSVTFFGEPSVLSCRLRHMAFGITFVLCISCVLGKTIVVVMAFNATLPSNNMMKWFGPTQQRLSVFIITLIQCLIFTVWLSLSPPYPLKNSEYFTER
IVFECHLGSEVSFYCVLGYIGLLSCVCFGVAFLARRLPDNFNEAKHLTFSMLIFCAVWITFIPAYVSSPGEYTVAVEIFAVLASSFGLLACIFAPKCYIILLKKEGNTRKNMIGKMAS
>Small-eyed_rabbitfish.locus257.1
KLVTSLEYRSNDKRDHCLLKEIEFLSFEETLGIALVTLALLGVCLTISVFTAFYVHRDTPIVKANNSELSFLLLLALALCFLCSVTFIGEPSVWSCRLRHMAFGITFFLCISCVLGKTIVVVMAFNATLPSNNMMKWFGPTQQRLSVFI
LTLIQCLICTVWLSLSPPYPLKNSEYFTERIVFECHLGSAVAFYCVLGYIGLLSCVCFGVAFLARRLPDNFNEAKHITFSMLIFCSVWITFIPAYVSSPGKYTVAVEIFAILASSFGLLACIFAPKCYIILLKNYINTVSDL------
>Elephant_shark.locus2.1
```

```
CLKCPLEYRSNDKRDHCILKEIEFLSFEETLGIALVTLALLGVFITISVFAAFYVHRDTPIVKANNSELSFLLLFALALCFLCSVTFIGEPSVWSCRLRHTAFAITFVLCISCVLGKTIVVVMAFNATLPSNNMMKWFGPTQQRLSVFI
LTLIQCLICTVWLCLSPPYPLKNSEYFTDRIVFECHLGSAVAFYCVLGYIGLLSCVCFGVAFLARRLPDNFNEAKHITFSMLIFCAVWITFIPAYVSSPGKYTVAVEIFAILASSFGLLVFIFAPKCNIILLKKEQNTKKHMMGKMAS
>Elephant_shark.locus14.1
CLKCPLEYRSNDKRDHCILKEIEFLSFEETLGIALVTLALLGVSITFSVFAAFYVHRDTPIVKANNSELSFLLLFALALCFLCSVTFIGEPSVWSCRLCHTAFAITFVLCISCVLGKTIVVVMAFNTTLPSNNMMKWFGPTQQRLSVFI
LTLIQCLICTVWLCLAPPYPLKNSDYFRERIVFECHLGSAVAFYCVLGYIGLLSGVCFVVAFLARRLPDNFNEAKHITFSMLIFCAVWITFIPAYVSSPGKYTVAVEIFAILASSFGLLVCIFAPKCFIILLKKEENTKKHMMGKITS
>Elephant_shark.locus12.1
CLKCPLEYRSNDKRDHCILKEIEFLSFEETLGIVPVIIALLGVCITISVFETFYVHRNTPIVKANNLELSFLLLFALALCFLCSIAFIGEPSVWSCRLRHTAFGITFVLCISCVLGKTIVVVMAFNATLPSNNMMKWFGPTQQRLSVLI
LTLIQCLICTVWLCLAPPYPLKNSEYFKERIVFECHLGSAVAFYCVLGYIGLLSGVCFVVAFLARRLPDNFNEAKHITFSMLIFCAVWITFIPAYVSSPGKYTVAVEIFAILASSFGLLVCIFAPKCYIILLKKEENTKKHMTGKMVS
>Cloudy_catshark.locus44.1
------------------------------
MGIILVTLALFGACISTGVFVTFYLHRHTPIVKANNSELSFLLLLALTLCFLCSIPFIGEPSSWSCALRHIAFGITFVLCISCVLGKTIVVVMAFNATLPNRNLMKWFGPTQQRLTVIILTLVQSLICAAWLTLSPPYPEKNSDYFHEI
IVFECNVGSTIGFYCVLGYIGFLSCVCFVAAFLARKLPDNFNEAKHITFSMLIFCAVWITFIPAYVSSPGKYTVAVEIFAILSSSSGLLICIFAPKCYVVLLRQELNTKRCLMG----
>Cloudy_catshark.locus42.1
------------------------------
MGIILVTLALFAACISTGVFVTFYLHRHTPIVKANNSELSFLLLLALTLCFLCSITFIGEPSSWSCALRHIAFGITFLLCISCVLGKTIVVLMAFNATLPNSNLMKWFGPTQQRLTVLILALVQSLICAAWLTLSPPYPEKNSDYFHEI
IVFECNVGSTIGFYCVLGYIGFLSRVCFVAAFLARKLPDNFNEAKHITFSMLIFCAVWITFIPAYVSSPGKYTVVAEIFAILSSSSGLLI----------------------------
>Brownbanded_bambooshark.locus1595.1
CLKCPLEYRSNHQRDECILKEIQFLSFEETLGIILVAIALFGSSISISVFVTFHRYRDTPIVKANNSELSFLLLSSLTFCFMCSITFIGEPSVWSCPLRHVAFGIIFVLCISCILSKTIVVVMAFQATLPNNNLMKWFGHKQQLLTVLM
LTLVQCLICAAWITISPPFPEKNSDYFNEIIVFECNIGSTIAFYCVFGYVGFLSSVCFVAAFLARKLPDNFNEAKHITFSMLIFCTVWITFIPAYVSSPGKYTVAVEIFAILASGFGLLACIFAPKCYIILLKQELNTKKHMMC----
>Brownbanded_bambooshark.locus1573.1
CIKCPLEFKSNERRDQCVPKEIDFLSFFDIIGIILVTLALFGACTTVGVFVIFYIFRHTPIVKANNSELSFLLLFALTLCFLSSIIFIGEPLVWSCMLCHVAFAVSFVLCISCVLSKTIVVVMAFKATFPSNNMMKWFGPRQQRLMVYI
LTLIQCAICTAWLIVFPPYPLKKTSHSSEIIVFECNVGSLLAFYCVLGYIGFLSCVCFVVAFLARQLPDNFNEAKHITISMLIFCAVWITFIPAYISSPGKYAVAVEVFAILASSFGVLICIFAPKCYIILLKKEDNTKRNMMGSVSF
>Brownbanded_bambooshark.locus1576.1
------------------------------
MGIILMTLALLGASTTVGVFIIFYIHRHTPIVKANNSELSFLLLFALTFCFLCSITFIGEPLVWSCMLCHVAFGISFVLCISCVLSKTIVVVMAFKATFPSNNMMKWFGPRQQQLMVYILTLVQCAICTAWLIVFPPYPLKNTSHSNEI
IVFECDVGSSLAFYCVLGYIGFLSCICFVVAFLARQLPDNFNEAKHITFSMLIFCAVWITFIPAYISSPGKYAVAVEVFAILASSFGLLICIFAPKCYIILLKKENNTKRNMIGSVAS
>Brownbanded_bambooshark.locus1583.1
------------------------------
MGIILVTLALLGVCTTVGIFAIFYINRHTPIVKANNSELSFLLLFALMLCFLSSITFIGEPSVWSCMLCNVAFGISFVLSISCVLSKTIVVVMAFKATFPSNNMMKWFGPRQQRLMVYILTLVQCAICTAWLIAFPPYPLKNTSHSNEI
IVFECHVGSSLAFYSVLGYIGFLSCVCFVVAFLARQLPDNFNEAKHITFSMFIFCTVWITFIPAYISSPGKYAVAVEIFAILASSFSLLICIFTPKCYIILLKKANNTKRNMMGSVTS
>Whale_shark.locus58.1
-----------------------------
MMGITLVALALFGACTTVAVFAIFYVYRXTPIVKANNSELSFLLLFALTLCFLCSITFIGEPLVWSCMLCHVAFGISFVLCIXCVLSKTIVVVMAFKAKFPNNNMMKWFGPRQQRLMVYILTLVQCAICTAWLILFPPYPLRSTSHSHE
IIVFECNVGSSLAFYCVLGYIGFLSCVCFVVAFLARQLPDNFNEAKHITFSMLIFCAVWITFIPAYISSPGKYAVAVEVFAILASSFGLLICIFAPKCYIILLNKEKNTKRNMMGSVSS
>Whale_shark.locus56.1
------------------------------
MGIILVTLALFGACTTVGVCAIFYVYRHTPIVKANNSELSFLLLFALTLCFLCSITFIGEPIVWSCMLCHVAFGISFVLCISCVLSKTIVVVMAFKAAFPNNNMMKWFGPRQQRLMVYILTLVQCAICTAWLILFPPYPLRNTSHSDEI
IVFECNVGSSFAFYCVLGYIGFLSCVCFVVAFLARQLPDNFNEAKHITFSMLIFCAVWITFIPAYVSSPGKYAVAVEVFAILASSFGLLICIFAPKCYIILLNKEKNTKRNMMSSVSS
>Whale_shark.locus62.1
------------------------------
MGIILVALSLLGAGTTVGVFTIFYVYRQTPIVKANNSELSFLLLFALTLCFLCAITFIGEPLVWSCMLCHVAFGISFVLCISCVLSKTIVVVMAFKATFPNKNMMKWFGPRQQRLMVYILTLVQCAICTAWLILLPPYPLKNTRHFNEI
IVFECNVGSPFAFYCVLGYIGFLSCVCFVVAFLARQLPDNFNEAKHITFSMLIFCAVWITFIPAYISSPGKYTVAVEVFAILASSFGLLICIFAPKCYIILLKKEKNTKRNMIGPVTS
>Whale_shark.locus68.1
------------------------------
MGIILVTLALLGACTAVGIFAIFYVYRHTPIVKANNSELSFLLLLALTLCFLSSITFIGEPSVWSCMFCHVAFGISFVFCISCVLSKTIVVVMVFKAKFPNNNVMKWFEPRQQRLMVYILTLVQCAICTAWLIVFPPYPLKNTSHSNEI
IMFECNVGSSFAFYCVLGYIGCLSCVCFLVAFLARQLPDNFNEAKHITFSMLIFCAVWITFIPAYISSPGKYAVAVEIFAILASTFGLLFCIFGPKCYVIVLKKEENTKRNMIGSVAS
>Smalltooth_sawfish.locus37.1
-----------------------------
MMGIILVTLALFGFCITMLVFATFYTHRETPIVKANNSELSFLLLLALMLCFLCSVTFIGEPSTWSCVLRHTSFGITFVLCVSCVLDKTIVVVLAFKATLPNNNIMKWFGPTQQRLAVLSLTLLQCLICTVWLATSPPYPRRNTEYFTE
IIVFECNVGSELAFYCVLGYIGFLSCVCFVVAFLVRQLPDNFNEAKHITFSMLIFCAVWITFIPVYVSSPGKYTVAVEVFAILASSFALLFCIFAPKCYVILLKREQNTKRHVMGKMPS
>Brownbanded_bambooshark.locus1597.1
CIPCPTEYKSNQQRDQCVPKEIEFLSFYEIMGIILVAFALFGCCITLIVFFTFYTHRHTPIVKANNSELSFLLIVTLLLCFLCSITFIGEPSAWSCMLRHTAFGIAFVLCVSCILCKTLIVVLAFKATLPNNNLMKWFGPVQQRLTIFI
LTLLQCLICTTWLATSPPYPLKNSNYFNEVIVFECNVGSSVAFYCVLGYIGFLSCVCFVAAFLARHLPDNFNEAKHITFSMLIFCAVWITFIPAYISSPGKYTVAVEVFAILASSFALLVCIFAPKCYIILLEREKNTRKHLMGKASS
>Whale_shark.locus24.1
------------------------------
MGILFVTLALTGTSFTVTEIIIFCIHINTPLVKVNNAELSFLLLFALVLCFLHSIMFIGKPSDWFCMLCHIIFGITFVLCVSCVLSKTMVVMMAFNATLPNNNMMEWFGPVQQRLAIFILTFVQCLICMAWLITSPPYSLKNANYFKDI
IILECDVGSVTAFSLVLGYIGFLSCVCFVIAFLASRLPNNFNEAKFITFSMFIFCAVWITFIPAYVSSPGKCMVAVEAFAILASSFGLIVCIFVPKCYIILLKPEKNTKKHLMQKVT-
>Whale_shark.locus57.1
------------------------------
MGIVLLKLALTGTSFTVSVIIIFXIHINTPFVKVNNAELSFLFLFALVLCLLHSIMFIGKPSDWFCMLCHIVFGITFVLCVSCVLSKTVVVMMAFNATLPNSNMMKWFGPVQQRLAIFTLTFVQCLICTAWLIASPPYSLKNANYFKDI
IILECDVGSVKAFSLVLGHIGFLSCVCFVVAFLTRRLPNNFNEAKCITFSMFTFCAVWITFIPSDVSSPGKHMVAGEAFAILVSXFGLLVCIFVPKCYIILLKLEKNTKKYLMQKVT-
>Brownbanded_bambooshark.locus1560.1
--------------------
MIEFLSFEEIMGIIIVTLALLGTGFTVTVTVIFCVHINTPIVKANNAELSFLLLFALVLCFLCSITFIGKPSNWSCMLRHTVFGITFVLCVSCVLGKTLVVMMAFNTTLPNNNMMKWFGPVQQRLAIFILTFIQCLICMVWLITSPPYP
LKNANYFKDIIILECDVGSVTTFYLVLGYIGFLSCVCFVVAFLARRLPNNFNEAKFITFSMLIFCAVWITFIPAYVSSPGKYMVAVEVFAILASSFGLLVCIFVPKCYIILLQPEKNTKKHLIRNVT-
>Whale_shark.locus64.1
------------------------------
MGIVLVTLALIGTSFTVTVIVIFCIHINTPLVKANNSELSFLLLFALVLCFLCSIMFIGKPSNWSCMLRHTVFGISFVLCISCVLGKTLVVMVAFKATFPNNKMMKWFGPVQQRLAIFILTFVQCVICTTWVIISPPYPLKNINYFKDI
IILECDVGSVTAFYLVLGYIGFLSCVCFVVAFLARRLPDNFNEAKFITFSMLVFCAVWITFIPAYVSSPGKYMVATEVFAILASSFGLLFCIFVPKCYIILLRPENNTKKHLMCKVT-
>Cloudy_catshark.locus40.1
CLTCPWDQWSNEQRDLCIPKMTEFLSFEEIMGIVLVTLALVGTGFTVTVTIIFYIHINTPIVKANNSELSFLLLSALALCFLCSITFIGKPSIWSCILRHIAFGITFVLCISCVLGKTLVVMMAFNATLPNNNMMKWFGPIQQRLAVLI
LTLVQCLICTVWLITSPPHPLKNVDYFKDIIILECDVGSAAAFYWVIGYIGFLSCVCFLVAFLARKLPDNFNEAKFITFSMLIFCAVWITFIPAYISSPGKYTVAVEVFAILASSFGLLVCIFVPKCYIILLRPETNTKKNLIGKVA-
>Great_white_shark.locus31.1
CLKCPWDYWSNVQRNQCIPKITEFLSFEETMGIVLVTLAIIGTSFTVTVIIIFYIHINSPIVKANNSELSFLLLSALALCFLCSVMFIGKPSNWSCMLRHTVFGITFVLCISCVLGKTLVVMMAFNATLPNNNRMKWFGPMQQRLAVLI
LMFVQCLICTIWLITSPPYPLKNVKYFKDVIILECDVGSVTAFYWVIGYIGFLSCVCFVVAFLARKLPDNFNEAKFITFSMLIFCAVWITFIPVYISSPGKYTVAVEVFAILASSFGLLICIFLPKCYIILLKPEKNTKKNLIGKVT-
>Smalltooth_sawfish.locus31.1
CMRCPSDSWPNARQDRCIQKQVDFLSFAELMGIVLTALALTGTCFTATVAGIFLLHKDTPVVKANNSELSFLLLLALTLCFLCSIAFIGEPSAWSCMLRHTVFGISFVLCVSCVLGKTIVVVMAFQAKLPSNNVAKWFGPLQQRLTVFL
LTLLQCLICTVWLIKSPPLPVKNMAYSEEVILLECDVGSEVAFYCVLGYIGCLSLVCFAVAFLARNLPDNFNEAKYITFSMLTFCAVWISFIPAYVSSPGKFTVAVEVFAILASSFGLLFCIFSPKCYIILLKPERNTKKHLMGKVSS
>Smalltooth_sawfish.locus34.1
CVRCPSDSWPNARQDRCIQKHVDFLSFAELMGIVLTALALTGTCFTATVAGIFLLHKDTPVVKANNSELSFLLLLALTLCFLCSIAFIGEPSTWSCMLRHIVFSISFVLCVSCVLGKTIVVVMAFQTKLPSNNVAKWFGPLQQRLTVFL
LTLLQCLICTVWLIKSPPLPVKNMAYSKEVILLECDVGSEVAFYCVLGYIGCLSLVCFAVAFLARNLPDNFNEAKYITFSMLIFCAVWISFIPAYISSPGKFTVAVEVFAILASSFGLLFCIFVPKCDVILLKPERNTKKYLMSKVSS
>Smalltooth_sawfish.locus33.1
-MRCPSDSWPNARQDRCIQKHVDFLSFAELMGIVLTALALTGTCFTATVAGIFLLHKDTPVVKANNSELSLFLLFALALCFLCSFAFIGEPSAWSCMLRHTMFGISFVLCVSCVLGKTLVVVMAFQAKLPSNNVAKWFGPLQQRLTVFL
LTLLQCLICTVWLIKSPPLPVKNMAYSKEVILLECDVGSEVAFYCVLGYIGCLSLVCFAVAFLARNLPDNFNEAKYITFSMLIFCAVWISFIPAYVSSPGKFTVAVEVFAILASSFGLLLCIFAPKCYIILLKPERNTKKHLMGKVSS
>Cloudy_catshark.locus47.1
CMKCPSESWPNAQQDQCLEKKVDYLSFEDILGIVLTTLALFGASTTATVGCIFLFHKDTPIVKANNSELSFLLLFALTLCFLCSLTFIGQPSGWSCMLRHTMFGVIFVLCVSCVLGKTIVVVMAFQAQLPSSDVAKRFGPSQQRLTVSG
LTLVQCFICTAWLTTSPPFPTQNTKYFQEVIILECDVGSEVAFYCVLGYIGCLSAVCFVVAFLARNLPDSFNEAKYITFSMLIFCAVWISFIPAYVSSPGKFTVAVEVFAILASSFALLFCIFAPKCYVILLRSERNTKKHLMGRVPS
>Brownbanded_bambooshark.locus1599.1
CIKCPSDSWPNSQQDQCIAKKVDYLSFGDTIGVILTTLALVGALATVTVGIIFVFYKDTPVVKANNSELSFLLLFALTLCFLCSLTFIAKPSGWSCALRHTMFGIIFVLCVSCVLGKTVVVVMAFQAKLPSNNVAKWFGPVQQRLTVCC
LTLVQCFICMVWLIISTPFLAQNIKYAKELIILECDVGSEVAFYCVLSYIGCLSAVCFVVAFLARNLPDALNEAKYITFSMLIFCAVWISFIPAYISSPGKFTVAIEVFAILASSFGLLFCIFAPKCYVILLKPEENTRKHLMGRVSS
>Great_white_shark.locus25.1
------------------------------
MGIVLTTLALVGAFATATIGGIFLFYKDTPIVKANNSELSFLLLFALTLCFLCSLAFIGQPSGWSCTLRHTMFGIIFVLCLSCILGKTIVVVMAFQAKFPSSNVAKWFGPVQQRLAVLCLILVQCLICIVWLMTSPPTPVKNTKYFKEV
IVLECDVGSEVAFYCVLGYIGCLSAVCFAVAFLARNLPESFNEAKYITFSMLIFCAVWISFIPAYVSSPGKFTVAVEVFAILASSFGLLFCIFAPKCYIILLKPEKNTKKQLMGRVLS
>Elephant_shark.locus3.1
-MKCLSEYWSSVQRDQCIPKQTEFLSYSEIIGMVIVIFAIVGACITMIVAVLFFVYKHTPIVKANNSELSFLLLLALTFCFLCSLTFIGEPSVPSCILRHTMFGVSFVLCMSCVLGKTIVVVMAFKATHPRKNIMKYFGVTQQRMTVCI
LTLVQCIICTIWLTIAPPFPVKNAISYTDKIILECDVGSVTAFYCVLGYIGCLSCVCFVVAFLARKLPDNFNEAKFITFSMIMFCAVWLSFIPVYISSRGKYTVAAEVFAILASSFGLLICIFAPKCYIILLKPDNNTKQHLMGKVSS
>Small-eyed_rabbitfish.locus83.1
--------------------------------
MVIVVFAIVGAGITMMVAVIFFVYRHTPMVKANNSELSFLLLLALTLCFLCSLTFIGQPSTPSCILRHTMFGVSFVLCMSCVLGKTIVVVMAFKATQPRNNIMKCFGVKQQRLTVCILTLVQCILCTIWLTTASPFPVRNGVSYTDKII
LECDVGSETAFYCVLGYIGCLSCVCFVVAFLARKLPDHFNEAKFITFSMIIFCAVWISFIPVYISSRGKYTVAAEVFAILASSFGLLVCIFAPKCYIILLKPENNTKQHLMGKVSS
>Cloudy_catshark.locus45.1
-----------------------------------
MALTIVGAVLTMGVAALFFVYKRTPIVKANNSELSFFLLFALTLCFLCSFIFMGEPTFWSCIFRHVTFSITFVFCVACVLGKTVVVVMAFKATVPNRNMMKYFGPTQQRLTVCALTMAQSFICTIWLATSPPFPRKNINYFKDKIILEC
DVGSTTAFYCVLGFLGLLSCVCFVVAFLARKLPDNFNEAKFITFSMLIFCTVWITFIPAYTSSHGKFTVAVEVFAILASSFGLLVCIFAPKCYIILLRPEYNTKEHVMGKVNS
>Elephant_shark.locus22.1
------------------------------
MGIFLAIIAVFGACLTIGISALFYYYKDTPIVKANNSELSFLLLFALTLCFLCSLAFIGEPSVWSCMLRHTLFGITFVLCISCILGKTFVVLMAFRATVPSNNIMKWFGPTQQLLTILILTFVQCLICIVWLAISPPFPVRNTKYLKEI
IILECDVGSVTAFYSVLGYLGFLSCVCFVVAFLARKLPDNFNEAKCITFSMLIFCAVWITFIPAYISSPGKYTVAVEVFAILASSSGLLLSIFAPKCYIIIRKPEKNTKKHMMGKKPS
>Brownbanded_bambooshark.locus1603.1
--------------------
MIEFLSFQEVLGSVFVALASVGVCLALGTATIFLRYRETPIVRANNSELSFLLLFALTLCFLCSLTFIGKPTGWSCMLRRTAFGIVFVLCISCILGKTVLAVIAFKSTLPNNNLMKWFGPLQQRLGVFVLTFLQGLICTIWLIVSPPYP
VKNVTYYRDIIILECDVGSLKAFYFVSSYIGLLSTVCFLLAFLARKLPDNFNDAKYITFSMLIFCAVWITFIPAYVSSPGKYTVAVEVFAIWASSFGLLVCIFTPKCYIILLKPENNSKEKIMGRCSS
>Brownbanded_bambooshark.locus1614.1
-MKCPLEYWSNQHKDRCVPKKIEFLSFQEVLGYVLVALALVGVCLALGTAAVFFQYRETPIVRANNSELSFLLLFALTLCFLCSLTFIGKPTGWSCMLRRTAFGIVFVLCISCILGKTVLVVVAFKATLPNNNLMKWFGPMQQRLGVFV
LTFLQGLICTIWLIISPPYPKKNVTYYRDIIILECDVGSLKAFYFVSGYIGLLCTVCFLLAFLARKLPDNFNDAKYITFSMLIFCAVWITFIPSYDSSPGKYTVAVEVFAIWASSFGLLVCIFAPKCYIILLKPENNTKKNMMA----
>Brownbanded_bambooshark.locus1611.1
CVKCPLEYWSNQQKDHCVPKTIEFLSFQEVLGCVLGGFALVGVCLSLCATTVFFRYRETPLVRANNSELSLLLLFALTLCFLCSLTFIGKPTGWSCMLRRTVFAIVFVLCISCILGKTVLVVIAFKSTLPNNNLMNWFGPLQQRLGVFV
```

LTFLQGLICTIWLIVSPPSPVKNMTYYRDIIILECDVGSLKAFYFVSSYIGLLSTVCFLLAFLARKLPDSFNDAKYITFSMLIFCAVWITFIPAYVSSPGKYTVAVEVFAIWASSFGLLACIFAPKCYIILLKPENNTKKNMMNKGSS
>Whale_shark.locus109.1
CMKCPLEYWSNQQKDQCIPKKIEFLSFQEALGYFLVALALVGVCLALAAATIFFRYRETSIVRANNSELSFLLLFALTLCFLCSLTFIGKPTGWSCMLRRTAFGIVFVLCISCILGKTVLVVIAFKATLPNNNLMNWFGPMQQRLSVFV
LTFLQGLICTIWLIVSPPYPVKNMTYYRDMIILECDMGSLKAFYFVSSYIGLLSTVCFLLAFLSRKLPDNFNDAKYITFSMLIFCAVWITFIPAYVSSPGKYTVAVEVFAIWASSFGLLVCIFAPKCYIILLKPENNTKKNMMGKGSS
>Whale_shark.locus36.1
CVKCPLEYWSNQQKDRCIPKKIEFLSFQEVLGYVLVALALVGVCLALGAAAVFFQFRETPIVRANNSELSFLLLFALTLCFLCSLIFIGKPTGWSCMLRRTAFGIVFVLCISCILGKTILVVIAFKATLPNNNLMNWFGPMQQRLGVFG
LTFLQGLICTIWLIVSPPHPMKNMTYYRNVIILECDMGSLKAFYFVSSYIGTLSTVCFLLAFLARKLSDNFNDAKYITFSMLIFCAVWITFIPAYVSSPGKYTVAVEVFAIWASSFGLLICIFAPKCYIILLKPENNTKKNVMGKGSS
>Whale_shark.locus103.1
CVKCPLEYWSNQQKDRCLPKKTEFLSFREVLGYVLVALAIMGVCLALCTAAVFFQFRETPIVRANNSELSFLLLFALTLCFLCPLAFIGKPIGWSCMLRRTAFGIVFVLCISCILGKTILVVIAFKATLPNNNLMSWFGPMQQRLGVFG
LTFLQGLICTIWLIVSPPYPVKNMTYYRDIIILECDVGSLKAFYFVSSYIGLLSTVCFLLAFLARKLPDNFNDAKYITFSMLIFCAVWITFIPAYVSSPGKYTVAVEAFAIWASSFGLLVCIFAPKCYIILLKPENNTRKT-------
>Whale_shark.locus49.1
CIKCPLEYWSNQQKDQCVPKKIEFLSFQEVLGYVLVALAIVGVCLAQGTAAVFFRYRETPIVRANNSELSFLLLFALTLCFLCSLTFIGKPTEWSCMFRRTEFGIVFVLCISCILGKTVLVVIAFKATLPNSNLMNWFGPMQQRLGVFG
LTFLQGLICTIWLIVSPPYPVKNMTYYRDIIILECDVGSLKAFYFVSSYIGFLSTVCFLLAFLSRKLPDNFNDAKYITFSMLIFCAVWITFIPAYVSSPGKYTVAVEVFAIWASSFGLLVCIFAPKCYIILLKPENNTKKNVIGKGPS
>Whale_shark.locus106.1
CIKCPLDYWSNQQKDRCIPKKIEFLSFQEVLGYVLVALASVGACLALGTVAVFFRYRETPIVRANNSELSFLLLFALMLCFLCSLTFIGKPTRWSCMLRRTAFGIVFVLCISCILGKTVLVVIAFKATLPTNNLMNWFGPMQQRLSVFV
LTFLQGLICTIWLIVSPPYPVKNMTYYRDIIILECDVGSLKAFYFVSSYIGLLSTVCFLLAFLARKLPDNFNDAKYITFSMLIFCAVWITFIPAYVSSPGKYTVAVEVFAIWASSFGLLVCIFAPKCYIILLKPENNTKKNMMGKWTS
>Great_white_shark.locus18.1
CTKCPLEYWSNQQKDRCVLKKIEFLSFREILGFVLVTLALVGVGVTLATAAIFFQYRETPIVRANNSELSFLLLFALMLCFLCSLTFIGKPTGWSCMLRRTIFGIVFVLCISCILGKTILVVIAFKATLPNNNLMNWFGPMQQRLLVFV
LTFLQALVCTIWLAVSPPYPLKNMAYYRDIIILECDVGSLKAFYFVSSYIGLLSTICFVLAFLARKLPDNFNDAKYITFSMLIFCAVWITFIPAYVSSPGKYTVAVEVFAIWASSFGLLICIFAPKCYIILLKPQDNTKKNMMGKVTS
>Great_white_shark.locus19.1
CTKCPLEYWSNQQKDRCVLKKIEFLSFREILGFVLVTLALVGVGVTLATAAIFFQYRETPIVRANNSELSFLLLFALMLCFLCSLTFIGKPTGWSCMLRRTIFGIVFVLCISCILGKTILVVIAFKATLPNNNLMNWFGPMQQRLGVFV
LTFLQALVCTIWLAVSPPYPLKNMAYYRDIIILECDVGSLKAFYFVSSYIGLLSTICFVLAFLARKLPDNFNDAKYITFSMLIFCAVWITFIPAYVSSPGKYTVAVEVFAIWASSFGLLVCIFAPKCYIILLKPQDNTKKNIMGKVTS
>Smalltooth_sawfish.locus46.1
CIKCPSEYWSNQPKTQCVLKKVEFLSSGEVLGFVLVTLAAVGVCFTLATAAIFYRYRETPMVKANNSELSFLLLFALMLCFICSLTFIGEPSGWSCMLRRTVFGVVFVLCISCILGKTILVVVAFKATLPNSSVMNWFGPAQQRLGVFI
LTFLQGVVCVVWLSVSPPYPLKNISYYRDIIILECDVGSLTAFYFVSAYIALLTIVCLVLAFLARKLPDSFNDAKYITFSMLIFCAVWITFIPVYVSSPGKYTVAVEVFAIWASSFGLLVCIFAPKCYIILLKPESNTKKHMMAKGTS
>Smalltooth_sawfish.locus55.1
CIKCPSEYWSNQQKTQCVLKKVEFISFGEVLGFVLVTLAAVGVCFTLATAAIFYRYRETPMVKANNSELSFLLLFALMLCFICSLTFIGEPSGWSCMLRRTVFGVVFVLCISCILGKTILVVVAFKATLPNSSVMNWFGPAQQRLGVFI
LTFLQGVVCVVWLSVSPPYPLKNISYYRDIIILECDVGSLTAFYFVSAYIALLSIVCLVLAFLARKLPDSFNDAKYITFSMLIFCAVWITFIPVYVSSPGKYTVAVEVFAIWASSFGLLVCIFAPKCYIILLKPESNTKKHMMAKGTS
>Smalltooth_sawfish.locus51.1
CIKCPSEYWSNQPKTKCVLKKVEFLSFGEVLGFVLVTLAAVGVCFTLATAAIFYRYRETPMVKANNSELSFLLLFALMLCFICSLTFIGEPSGWSCMLRRTVFGVVFVLCISCILGKTILVVVAFKATLPNSSVMNWFGPAQQRLGVFI
LTFLQGVVCVVWLSVSPPYPLKNMSYYRDIIILECDVGSLTAFYFVSAYIALLSIVCLVLAFLARKLPDSFNDAKYITFSMLIFCAVWITFIPVYVSSPGKYTVAVEVFAIWASSFGLLVCIFAPKCYIILLKPESNTKKHMMAKATS
>Smalltooth_sawfish.locus49.1
CIKCPSEYWSNQPKTQCVLKKVEFLSFGEVLGFVLVTLAAVGVCFTLVTAAIFYRYRETPMVKANNSELSFLLLFALMLCFICSLTFIGEPSGWSCMLRRTVFGVVFVLCISCILGKTILVVVAFKATLPNSSVMNWFGPAQQRLGVFI
LTFLQGVVCVVWLSVSPPYPLKNMSYYRDIIILECDVGSLTAFYFVSAYIALLSIVCLVLAFLARKLPDSFNDAKYITFSMLIFCAVWITFIPVYVSSPGKYTVAVEVFAIWASSFGLLVCIFAPKCYIILLKPESNTKKHMMAKATS
>Smalltooth_sawfish.locus57.1
CVKCPSEYWSNKPKTQCVLKKVEFLSFGEVLGFVLVTLAAVGVCFTLATAAIFYRYRETPMVKANNSELSFLLLFALMLCFICSLTFIGEPSGWSCMLRRTVFGVVFVLCISCILGKTILVVVAFKATLPNSSVMNWFGPAQQRLGVFI
LTFLQGVVCVVWLSVSPPYPLKNMSYYRDIIILECDVGSLTAFYFVSAYIALLSIVCLVLAFLARKLPDSFNDAKYITFSMLIFCAVWITFIPAYVSSPGKYTVAVEVFAIWASSFGLLVCIFAPKCYIILLKPESNTKKHMMAKGTS
>Smalltooth_sawfish.locus59.1
CIKCPSEYWSNQPKTQCVLKKVEFLSFGEVLGFVLVTLAAVGVCFTLATAAIFYRYRETPMVKANNSELSFLLLFALMLCFICSLTFIGEPSGWSCMLRRTVFGVVFVLCISCILGKTILVVVAFKATLPNSSVMNWFGPAQQRLGVFI
LTFLQGVVCVVWLSVSPPYPLKNMSYYRDIIILECDVGSLTAFYFVSAYIALLSIVCLVLAFLARKLPDSFNDAKYITFSMLIFCAVWITFVPAYVSSPGKYTVAVEVFAIWASSFGLLVCIFAPKCYIILLKPESNTKKHMMAKGTS
>Smalltooth_sawfish.locus53.1
CIKCPSEYWSNQPKTQCVVKKVEFLSFGEVLGFVLVTLAAVGVCFTLAAAAIFYQYRETPMVKANNSELSFLLLFALMFCFICSLTFIGEPSGWSCMLRRTVFGVVFVLCISCILGKTILVVVAFKATLPNSSVMNWFGPAQQRLGVFI
LTFLQGVVCVVWLSVSPPYPLKNMSYYRDIIILECDVGSLTAFYFVSAYIALLSIVCLVLAFLARKLPDSFNDAKYITFSMLIFCAVWITFIPAYVSSPGKYTVAVEVFAIWASSFGLLVCIFAPKCYIILLKPESNTKKHMMAKGTS
>Smalltooth_sawfish.locus48.1
CIKCPSEYWSNQPKTQCVLKKVEFLSFGEDLGFVLVTLAAVGVCFTLATAAIFYRYRETPMVKANNSELSFLLLFALMLCFICSLTFIGEPSGWSCMLRRTVFGVVFVLCISCILGKTILVVVAFKATLPNNSVRNWFGPAQQRLGVFV
LTFLQGVVCVVWLSVSPPYPLKNMSYYRDIIILECDVGSLTAFYFVSAYISLLSIVCLVLAFLARKLPDSFNDAKYITFSMLIFCAVWITFIPAYVSSPGKYTVAVEVFAIWASSFGLLVCIFAPKCYIILLKPESNTKKHMMAKGTS
>Small-eyed_rabbitfish.locus29.1
CLTCPPRYWSNQGKDHCVEKKVEFLSYGEILGVVLATLAITGVCLTLAVAALFLKHRSSPIVKANNSELSLLLLLSLTLCFLCSLTFIGKPSAWSCMLRRTAFGVVFVLCISCILGKTIVVLVAFKASRPNNKMLKWFNAKQQRLIIFA
LTFVQCFICTLWLTISPPFPLENNKYYREIIIIECDMGSLTAFYCVSGYIALLSSLCFVLAFIARKLPDNFNEAKFITFSMLIFCVVWIAFIPAYVSSPGKYTVAVEVFAILASSFALLVCIFGSKCYIILIKPEQNTKKHLMGKETP
>Elephant_shark.locus180.1
CLTCPPRYWSNQGKDQCEKKKVEFLSYGEILGTVLATLAITGVCLTLAIAALFFKHRSSPIIKANNSELSLLLLLSLTLCFLCSLTFLGEPSAWSCMLRRTAFGVAFVLCISCVLGKTIVVLVAFKASRPNNKMLKWFSAKQQRLTIFA
LTFVQCFTCTLWLTISPPFPVENNMYYREIIIVECDMGSLTAFYCVSGYIALLSCLCFVLAFIARKLPDNFNEAKFITFSMLIFCVVWIAFIPAYVSSPGKYTVAVEVFAILASSFALLVCIFGPKCYIILIKPEQNTKKHLMGKETS
>Brownbanded_bambooshark.locus1601.1
CVKCPTEYWPNISRDKCVPKEIEFLSYTEILGIVLATLALVGVCLAIATLIVFIKYKDTPIVKANNSELSFLLLFAIAHCFLCSLTFIGKPSPWSCILRCTAIGVSFVLSISCILVKTMIVLFVFKARVPNNTMLKYFGVTQQRLSIFV
LTFIQGIISALWLAMAPPFPMKNTRYYNDIIILECNVGSVTAFYLVSVYIGTLSCVCFVLAFLARKLPDNFNEAKFITFSMLTFCAVWIAFIPAYVSSPGKYTTASEVFAILTSNFGLLLCIFVPKCYVVLLKPENNTKKFLMGKELS
>Cloudy_catshark.locus77.1
CFKCPVEYWPNVSRDKCVPKGIEFLSYTEILGIVLTTLALAGICIAMAIAIVFLKYKDTPIVKANNSELSFLLLFAIALCFLCSLTFIGQPTLWSCMLRRTAFGISFVLCISCILVKTIIVLMVFKTRIPNQSMLKRFGAKQQRLSVFV
LTFIQGLISALWLVIAPPFPMKNTRYYKDILILECDTGSVTAFYLVSVYIGTLSCLCFVLAFLARKLPDNFNEAQFITFSMLTFCAVWIAFIPAYVSSPGKYTTASEIFAILTSNFGLLLCIFVPKCYVVLLKPENNTKKFLMGKEPS
>Great_white_shark.locus22.1
CVKCPVEYWPNVSRDKCVPKGIEFLSYTETLGVVLTTVALVGICIAMAIAIVFLKYKDTPIVKANNSELSFLLLSAIAFCFLCSLIFIGQPSPWSCMLRRTAFGISFILCISCILVKTIIVLMVFKTRVPNNSMMKHFGPTQQRLSVFV
LTFIQGLISALWLVIAPPFPMKNTRYYKDMIILECDVGSTTAFYLVSAYIGTLSCVCFVLAFLARKLPDNFNEAKFITFSMLTFCAVWIAFIPAYISSPGKYTAASEVFAILTSNFGLLLCIFVPKCYAVLLKPENNTKKFLMGKETS
>Elephant_shark.locus179.1
CLKCPWEYWSNERRDECIPKKIEFLTFDENIGIALTSLAIVGVGLTVAIAAVFFQYKDTPIVKSNNSELSFLLLFALALCFLCSLTFIGQPSVWSCMLRRTSFGISFVLCISCVLGKTIIVLMAFKTKFPNNNRVKLFGLGRQRFSIFA
LTFIQILLCALWLSQAAPFPLKNGNYYRNIIILECDAGSTTIFYCVSGYIALLSCVSFILAYLARKLPDNFNEAKYITFSMLIVCAVWVTFIPAYVSSPGKYTVAVEMFAILASSFGLLVCIFAPKCYVVLLKPECNTKKHIMGKVPS
>Small-eyed_rabbitfish.locus30.1
CFKCPWEYWPNEQRDECLPKKIEFLTFTENIGIALTSLAIVGVGLTVTIAAIFFQYKDTPIVKANNSELSFLLLFALALCFLCSLTFIGQPSVWSCMLCHTSFGISFVLCISCVLGKTIIVLMAFKTKFPNNNWVKLFGLGRQRFSVFA
LTFFQILLCALWLSLAAPFPLKNINYYRNIIILECDAGSTTMLYCVSGYIALLSCVSFILAFLARKLPDNFNEAKYITFSMIIVCAVWITFIPAYVSSPGKYTVAVEIFAILASSFGLVVCIFVPKCYVILLKPECNTKKHIMGKVPS
>Elephant_shark.locus177.1
CIRCPWDYWSNRLRDQCLPKATDFLSYTEPVGAALASLALTGLCTTLVVAAVFFQSRHTPIVKANNSELSFLLLFALWLCFLCALAFIGEPSGRTCKLRRGAFSVSFVLCLSCVLVKTVLVLLAFKTTVPNNSVLRWFDATKQRLSVFI
LTFVQSFICALWLTLSPPFPLKNTEYYEDIIILECAVGSVTAFYWASAYIALLSCVCFLIAFFTRKLPDNFNEAKCITFSMLIHCAVWITYIPVYVSSPGRHSVAVEVFAILMSSFALLVCIFAPKCYIILLKPAQNTKKHLMGKEPS
>Small-eyed_rabbitfish.locus32.1
CIKCPWDYWSNQLRDQCVPKATDFLSYTETLGVVLVSLSLAGLCTTLFTAAMFFRCRHTPIVKANNSELSFLLLFALCLCFLCALTFIGQPSSQTCGLRRTAFSLSFVLCISCVLVKTILVLMAFKTTMPNNNILKWFDVTKQRLSVFI
LTFVQGLTCFLWLSLSPPFPFKNTEYYKDIIILECAVGSVTAFYWVSAYIALLSCTCFLLAFFARKLPDNFNEAKFITFSMLIFCVVWMTYIPVNISSPGRYTVAVEVFAILMSSFALLVCIFAPKCYIILLKPEHNTKKHLMGKEPS
>Small-eyed_rabbitfish.locus163.1
CIKCSLEYWSNQQKDRCVPKGIEFLSFADTLGIVLVTLALVGVCTTVTVAVVFLKYKHTPIVKANNSELSFLLLFALALCFLSSLTFIGQPSVWSCMLRRTAFSVSFVLCISCILGKTIIVVLAFKATLPSDNVMKWFGPTQQRLSVSI
LTFIQFVMCVLWLTILPPFPLKNSTHYRDIIILECDSGSRTAFYCVSGYIAILSCICFILAFLARKLPDNFNEAKFITFSMLIFCAVWITYIPASVSSPGKYSTTVEVFAILASSF--------------------------------
>Elephant_shark.locus23.1
CVKCSLEYWSNQQKDRCVSKRIEFLSFADTLGIVLVTLALVGVCTTAAVAVVFLQYKHTPIVKANNSELSFLLLFALALCFLSSLTFIGQPSVWSCMLRRTAFSISFVLCISCILGKTIIVVLAFKATLPSNNVMKWFGHTQQRLSVLI
LTSIQFAICVLWLTILPPFPLKNSTHYRDVIILECDSGSRTAFYCVSGYIAILSCICFVLAFLARKLPDNFNEAKFITFSMLIFCAVWITYIPASVSSPGKYSTAVEVFAILSSSFGLLVSIFTPKCYIILLKPETNTKKHLMSKNAE
>Great_white_shark.locus20.1
CIQCPLEYWPSTNKDKCVAKEIEFLDFGETLGIVLVTLALVGICGTIAIAGVFLQHKDTPIVKANNSELSFLLLFALTLCFLCSLTFIGEPSFWSCVLRRVIFSITFVICISCILAKTILVLMAFKATLPNNNLMRWFGPTQQRFGVVG
LTFIQGFICTVWLSIAAPFPMKNTSYYNEIIILECRVGSITAFYCVSGYIAILSCVCFVLAFLARKLPNNFNEAQCITFSMLIFCAVWITFIPAYLSSPGKYTVATEVFAILASSFALLLCIFVPKCIIILFKPENNTRKHVMSKMAS
>Elephant_shark.locus178.1

MLGIVLVTIGLVGIGVTLTTTVIFYQHRDTPIVKANNSELSFLLLFALTLCFLCSLTFIGEPSMWSCKLRRVSFGIAFVLCISCILGKTILVLMAFKATLPNNNMMKWFGPTQQRLSVFLLTLIQSLICALWLTFSPPSPVKNIKYYRD
IIILECDVGSITAFYCVSTYIGLLSAVCFILAFLARNLPDHFNEAKCITFSMLIFCTVWMAFIPAYVSSPGRYTVAVEVFAILASSFGLLLCIFVPKCRIILLKPENNTRQYIMSKDPS
>Small-eyed_rabbitfish.locus31.1
CIKCPLEYWSNQKKDKCIQKEIEFLSFDETLGIVLVTIGLVGVCLTLTTTVIFYQHRDTPIVKANNSELSFLLLFALTLCFLCSLTFIGEPSLLSCMLRRVSFGIAFVLCISCILGKTILVLMAFKATLPNNNMMKWFGPKQQRLSVFL
LTLIQSLTCTLWLTLSPPTPVQNIKYYRNIIILECNVGSITAFYCVSAYIGLLSAVCFILAFLARKLPDNFNEAKCITFSMLIFCTVWMAFIPAYVSSPGKYTVAIEVFAILASSFGLLLCIFVPKCRIILLKPENNTKQYLMSKDPS
>Elephant_shark.locus212.1

MLGGVLLTLALVGVSTTISIAIVFFTHRDTPIVKANNSELSYLLLFALTLCFLCSVTFIGEPSVWACMLCHTAFAIAFVLCISCILIKTILVILAFKATLPNNNMAKLFGPAQQRWSVFLLTSVQCLICTLWLTISHPFPMRNTEYYRE
IIILECDLGSVSAFYSMAGYIAFLAGVSFVLAFLARKLPDNFNEAQCITFSMLIFCAVWITFIPVYISSPGKYTVAVEVFAILASSFGLLFCIFVPKCYIILIKPEMNTKKHLMGKV--
>Elephant_shark.locus191.1

MLGGVLLTLALVGVSTTISIAIVFFTHRDTPIVKANNSELNYLLLFALTLCFLCSVTFIGEPSVWACMLCHTAFAIAFVLCISCILIKTILVILAFKATLPNNNMAKLFGPAQQRWSVFLLTSVQCLICTLWLTISHPFPMRNTEYYRE
IIILECDLGSVSAFYSMAGYIAFLAGVSFVLAFLARKLPDNFNEAQCITFSMLIFCAVWITFIPVYISSPGKYTVAVEVFAILASSFGLLFCIFVPKCYIILIKPEMNTKKHLMGKV--
>Elephant_shark.locus174.1
CIKCPEQYWPNQQRDRCLPKEIEFLSFTEMLGGVLLTLALVGVSTTISITIVFFTHRDTPIVKTNNSELSYLLLFALTLCFLCSVTFIGEPSVWACMLCHTAFAIAFVLCISCILIKTILVILAFKATLPNNNMAKLFGPAQQRWSVFL
LTSVQCLICTLWLTISHPFPMRNTEYYREIIILECDLGSVSAFYSMAGYIAFLAGVSFVLAFLARKLPDNFNEAQCITFSMLIFCAVWITFIPVYISSPGKYTIAVEVFAILASSCGLLFCIFVPKCYIILIKPEMNTKKHLMGKVPA
>Elephant_shark.locus176.1
CIKCPEQYWPNQQRDRCLPKEIEFLSFTEMLGGVLLTLALVGVSTTISIAFVFFTHRDTPIVKANNSELSYLLLFALTLCFLCSVTFIGEPSVWACMLCHTAFAIAFVLCISCILIKTILVILAFKATLPNNNMAKLFGPAQQRWSVFL
LTSVQCLICTLWLTISHPFPMRNTEYYREIIILECDLGSVSAFYSMAGYIAFLAGVSFVLAFLARKLPDNFNEAQCITFSMLIFCAVWITFIPVYISSPGKYTVAVEVFAILASSCGLLFCIFVPKCYIILIKPEMNTKKHLMGKVPV
>Elephant_shark.locus182.1

XLGGVLLTLALVGVSTTISIAFVFFTHRDTPIVKANNSELSYLLLFALTLCFLCSVTFIGEPSVWACMLCHTAFAIAFVLCISCILIKTILVILAFKATLPNNNMAKLFGPAQQRWSVFLLTSVQCLICTLWLTISHPFPMRNTEYYRE
IIILECDLGSVSAFYSMAGYIAFLAGVSFVLAFLARKLPDNFNEAQCITFSMLIFCAVWITFIPVYISSPGKYTVAVEVFAILASSFGLLFCIFVPKCYIILIKPEMNTKKHLMGKV--
>Elephant_shark.locus203.1

MLGGVLLTLALVGVSTTISIAIVFFTHRDTPIVKANNSELSYLLLFALTLCFLCSVTFIGEPSVWACMLCHTAFAIAFVLCISCILIKTILVILAFKATLPNNNMAKLFGPAQQRWSVFLLTSVQCLICTLWLTISHPFPMRNTEYYRE
IIILECDLGSVSAFYSMAGYIAFLAGVSFVLAFLARKLPDNFNEAQCITFSMLIFCAVWITFIPVYISSPGKYTVAVEVFAILASSFGLLFCIFVPKCYIILIKPEMNTRKHLMGKVPV
>Small-eyed_rabbitfish.locus218.1

MLGSVLLTLSLVGVSTTISIATVFFTHKDTPIVKANNSELSYLLLFALTLCFLCSVTFIGEPSVWACMLCHTAFAIAFVLSISCILIKTILVLLAFKATLPNNNMAKLFGPAQQRLSVFLLTSVQCLICTLWLTISHPFPMRNAEYYRE
IIILECDLGSVTAFYSMAGYIAFLAGVSFVLAFLARKLPDKFNEAQCITFSMLIFCAVWITFIPVYVSSPGKYTVAVEVFAILASSFGLLF----------------------------
>Smalltooth_sawfish.locus44.1
CIKCPPLFWSNHRRDQCIPKNIEYLAFTEILGIVLLVLALVGVCLSALTAGLFFRHRETPLVKANNSELSFLLLFALSCCFLCSVTFIGAPSDWSCMLRRTAFGVSFVLCMSCVLVKTILVLTAFKATHPSNNWMKWFGQRQQRISVFL
LASVQGLICVLWLIAAPPLPVINTKYYREMIILECDTGSSAAFYSASGYIAVLSCICFVLAFLARKLPNNFNEAKCITFSMLIFCAVWITFIPASVSSPGKYTVAVEVFAILASSFGLLLCIFAPKCYIMVITPEKNTKKHVMGKVG-

```
>Smalltooth_sawfish.locus43.1
CIKCPPLFWSNHRRDQCTPKNIEYLAFTEILGIVLLVLALAGVCLSALTAGLFFRHRETPLVKANNSELSFLLLFALSCCFFCSVTFIGKPSDWSCMLRRTAFGVSFVLCMSCVLVKTILVLTAFKATHPSNNWMKWFGQRQQRISVFL
LASVQGLICVLWLIIAPPFPVINTKYYREMIILECDTGSSAAFYSASGYIAVLSCVCFVLAFLARKLPDNFNEAKCITFSMLIFCAVWITFIPASVSSPGKYTVAVEAFAILASSFGLLLCIFAPKCYIMVITPEKNTKKHVMGKVG-
>Smalltooth_sawfish.locus42.1
CIKCPPLFWSNHRRDQCIPKNIEYLAFTEILGIVLLVLALVGVCLSALTAGLFFRYRETPLVKANNSELSFLLLFALSCCFLCSVTFIGAPSDWSCMLRRTAFGVSFVLCMSCVLVKTILVLTAFKATHPSNNWMKWFGQRQQRISVFL
LASVQGLICVLWLIIAPPFPVINTKYYREMIILECDTGSSAAFYSASGYISVLSCICFVLAFLARKLPDNFNEAKCITFSMLIFCAVWITFIPASVSSPGKYTVAVEVFAILASSFGLLLCIFAPKCYIMVITPEKNTKKHIMGKVG-
>Smalltooth_sawfish.locus41.1
CIKCPPLFWSNHRRDQCIPKNIEYLAFTEILGIVLVVLALVGVCLSALTAGLFFRYRETPLVKANNSELSFLLLFALSCCFLCSVTFIGAPSDWSCMLRRTAFGVSFVLCMSCVLVKTILVLMAFKATHPSNNWMKWFGQRQQRISVFL
LASVQGLTCVLWLIIAPPFPVINTKYYREMIILECDTGSSAAFYSASGYIAVLSCICFVLAFLARKLPDNFNEAKCITFSMLIFCAVWITFIPASVSSPGKYTVAVEVFAILASSFGLLLCIFAPKCYIMVITPEKNTKKHIMGKVG-
>Brownbanded_bambooshark.locus1619.1
------------------------------
MGSSLLTLAVIGTCLTVATAIIFYKHRQTPLVKANNSELSFLLLFALTLCFLCSLAFIDRPSVWSCMLRHTVFSITFVLCLSCILGKTLVVLMAFKTRLPNNKKIQWFSPMHQRLNVCALTSVQTIICIIWLTTSPPFPVNSTKYYKTI
IILECNVGSMVYFCCVFSYIGALACVCCGLAFLARKLPDNFNEATFITFSMLIFCAVWITFIPAYVSSPGKYTVAVEVFAILASSFGLLFCIFAPKCYIILLQPEKNTKKYLMRRSQA
>Whale_shark.locus2.1
------------------------------
MGSSLMTLAVIGACLTVATAIIFYKHRQTPLVKANNSELSFLLLFALTLCFLCSLAFIDRPSVWSCMLRHTVFSITFVLCLSCILGKTLVVLMAFKTRLPNNKKIQWFSPMHQRLNVCALTSVQTIICIIWLTTSPPFPVNSTKYYKTI
IILECNVGSLVYFCCVFGYIGALACVCCALAFLARKLPDNFNEATFITFSMLIFCAVWITFIPAYVSSPGKYTVAVEVFAILASSFGLLFCIFAPKCYIILFQPEKNTKKYLMRRSHA
>Great_white_shark.locus17.1
-----------------
MPKKIEFLSFDDEMGSSLLTLAVIGACLTVATGLIFYKHRQTPLVKANNSELSFLLLFALTLCFLCSLTFIDQPSVWSCMLRHVVFSITFVLCLSCILGKTLVVLMAFKTRHPNNKRIKWFSPMHQRLNVCALTSVQTIICIIWLTSSP
PFPVNSTKYYKTIIILECNIGSLVYFCCVFSYIGALACLCCALAFLARKLPDNFNEATFITFSMLIFCAVWITFIPAYVSSPGKYTVAVEVFAILASSYSLLFCIFAPKCYIILFQPEKNTKKYLMRRSNT
>Cloudy_catshark.locus53.1
------------------------------
MGSSLMALALIGACLTVATALIFYKHRQTPLVKANNSELSFLLLFALTLCFLCSLAFIDRPSEWSCMLRHTVFSITFVLCLSCILGKTLVVLMAFKARLPNNKNIKWFSRLQQRLNVCALTSVQTIICIIWLTTKPPFPVNSTKYYKTV
IILECNFGSLVYFCCVFSYIGALACLCCALAFLARKLPDNFNEATFITFSMLIFCAVWITFIPAYVSSPGKYTVAVEVFAILASSYSLLFCIFAPKCYIILLQPEKNTKKYLMRRSNA
>Thorny_skate.locus28.1
CLKCSPEFWSNELKTQCVPKRVEFLSFDDEMGSSLVTLAVIGAFLTMATAVVFYKHKSTPLVKANNSELSFLLLFALTLCFLCSLTFIDRPSTWSCMLRHTVFSISFVLCLSCILGKTVVVLMAFKTRLPNRKKIKWFNPMHQRLNVFS
LTSVQTVICIIWLTSSPPFPVNSTKYYKAIIILECNVGSMVYFCCVFSYIGALACLCCALAFLARKLPNNFNEATFITFSMLIFCAVWITFIPAYVSSPGKYSVAVEVFAILASSFGLLFCIFAPKCYIILLQPEKNTRKYLMQRGIK
>Little_skate.locus7.1
CLKCSPEFWSNELKTQCVPKRVEFLSFDDEMGSSLVTLAVIGAFLTIATAVVFYKHKSTPLVKANNSELSFLLLFALTLCFLCSLTFIDRPSTWSCMLRHTVFSISFVLCLSCILGKTVVVLMAFKTRIPNRKKIKWFSPMHQRLNVFS
LTSVQTIICIIWLTSSPPFPVNSTKYYKAIIILECNVGSMVYFCCVFSYIGALAFLCCALAFLARKLPNNFNEATFITFSMLIFCAVWITFIPAYVSSPGKYSVAVEVFAILASSFGLLFCIFAPKCYIILLQPEKNTRKYLMQRGIK
>Small-eyed_rabbitfish.locus25.1
------------------------------
MGKSLLSLALVGAGLTLATAGIFYKFRNTPLVKANNSDLSFLLLFALILCFLCSLTFIDEPSRWSCMLRHTIFSISFVLGLSCILGKTIVVLMAFKTTMPNNKKIKWFSPVRQRINVFILTFIQTLLCVLWLTTSPPFPVKNTSYYKAM
IILECNVGSIVYFCCVFGYIGILSCVCFILAFLARKLPNNFNEATFITFSMLIFCAVWITFIPAYVSSPGKYTVAVEVFAILASSFGLLVCIFAPKCYIIILKPEKNTKKYLMKK---
>Elephant_shark.locus192.1
------------------------------
MGKSLVILAVVGAGLTLATAGIFYKFRSTPLVKANNSDLSFLLLFALILCFLCSLTFIDKPSRWSCMLRHTIFSISFVLCLSCILGKTIVVLMAFKTTMPNSKKIKWFSPIRQRINVFILTFIQTLLCILWLTTSPPFPVKNTSYYKSI
IILECNVGSMAYFCCVFGYIGALSCVCFVLAFLARKLPNNFNEATFITFSMLIFCAVWITFIPAYVSSPGKYTVAVEVFAILASSFGLLVCIFVPKCYIIIFKPEKNTKKYLMKK---
>Little_skate.locus52.1
CTSCPLEYWPNQPKTECLLKDLEFLSFEDTIGATLAGFAVGGTCLTIATIVIFYHYRHTPLVRANNSELSFIILFSLGLCFLCALAFISSPSHWSCILRFTVFGVTFAMCLAGVLGKTIVVLMAFKARSPKNRAMRWFSPNRQRLAVLI
LTFVQCIICIFWIIQSPPYPFKSSTYYANVLSLECQVGSDAYFWGVFIIIFLLTGITLFFAFLARKLPDSFNEATHITFSLLIFCAVWITFFPVYASAPEKYAASVHVFAILASSFSLLLCIFAPKCYIILFKPERNTKKCLFTYSSP
>Thorny_skate.locus27.1
CTSCPLEYWPNQPKTECLLKDLEYLSFEDTIGATLASFAVGGTCLTIATIVIFYHYRHSPLVRANNSELSFIILFSLGLCFLCALAFISSPSHWSCILRFTVFGVTFAMCLAGVLGKTIVVLMAFKTRSPKNQAMRWFSPNRQRLAVLI
LTFVQCIICIFWIIQSPPYPFKSSTYYPNVLSLECQVGSDAYFWGVFIIIFLLTGITLFFAFLARKLPDSFNEATHITFSLLIFCAVWITFFPVYASAPEKYAASVHVFAILASSFSLLLCIFAPKCYIILFKPERNTKKCLFTYSSP
>Smalltooth_sawfish.locus61.1
CTKCPLEYWSNELRTECLLKEIEFLSFSDGMGATLAGFAVGGACLTIATIVVFYNYRHTPLVRANNSELSFIILFSLGLCFLCALTFIASPSHWSCVLRFTVFGVTFAMCLSGILGKTIVVLMAFKARYPQNNAMRWFTPNRQRMGVLI
LTLIQFAICIFWVIDSPPYPFKNSSYYAHILSLECQVGSNVYFWVVFIVIFLLTGITLVFAFFARKLPDTFNEATHITFSLIIFCAVWITFFPVYASAPEKYAASVHVFAILASSFSLLLCIFAPKCYIILFKPQLNSRKLLLTFSTS
>Brownbanded_bambooshark.locus1620.1
-MSCPLEYWPNQYRNECLLKEIEFLSFQDSIGVALTTFTGGGACLTVAMAFIFFKYRHTPLVRANNSELSFFMLLSLFLCFLSALTFIAHPSPWSCGLRYTIFGVTFALCLSSILGKTMVVLLAFKTLAPNKHFVKWFSPKCQRIFVLI
LTSVQCVICILWIKISPPYTFKSLKYYQNVMSLECQVGSTLYFCIVFTYIFFLAGVTLVLAFLARKLPDSFNEATHITFSLLVFFVVWITLFPVYICSPEKYAAAVHAFAILSSSYGLLLCIFAPKCYIMLLKPEKNTRKRLMTFSSN
>Cloudy_catshark.locus54.1
CTKCPLEYWPNQYRNECLLKEIEFLSFQDRIGVTLTSFSVGGACLTITTAVVFFKHRHTPLVRANNSELSFLMLLALLLCFLSALLFSAYPTQWSCTLRFTMFSVTVALCLSAVLGKTVVVLLAFKTVSQSTKLLRWFNPRRQRMGVLI
LTSIQCIICILWITHSPPFPFKSLEYYPHVISLECQIGSVVYFCAIFVYIFLLTGVTLTAAYFARKLPDSFNEATHMTFSLLVFLVVWITFFPVYMSAPEKYTATVHAFAILASSFGFLLCIFAPKCYIILLKPETNTRKNLMRFSSK
>Whale_shark.locus21.1
------------------------------
MGIILTTLSLFGACTSMAVIAVFYYYKDTPIVKANNSELSFLLLFSLVLCFLCSLLFMGQPTTWSCMLRHTAFAIIFVLCISCILGKTITVLVAFSAKYPNNDVMKWFIPLPRSLAVFLLPSIQIVICILWLILSPPFPAKNTEYQSVT
IILECDVGSATAFYCMLGYIGLLSVMCFILAFLARKLPGNFNEAKFITFSMLIFCAVWITFIPVYINS-GKYTVAVEIFAILTSSFSLLACIFAPKVYIILLRPELNTKRNVMGRPSS
>Great_white_shark.locus34.1
------------------------------
MGIILTTLSLFGACLSMAVTAVFYHYKNTPIVKANNSELSFLLLFSLVLCFLCSLLFMGQPTIRSCMLRHTAFAIIFVLCISCILGKTIMVLMAFNARLPNNNVVKWFGPLQRRLSIFFLPAIQVVICILWLNLSPPYPAKNIEYQSAI
IILECNVGSTTAFYSVLGYIGLLSVMCFVLAFLARELPDNFNEAKFITFSMLIFCAVWITFIPVYINS-GKYIVAVEIFAILSSSFGLLTCIFAPKVYIILLRPELNTKKNLMGRPPS
>Zebrafish.locus500.1
------------------------------
MGIILTALSLFGASLTVATMIVFIHYRETPIVKANNSELSSLLLSSLFFCFLCPLTFIGEPTHWSCMLRHTAFGLTFALCISCVLGKTIVVVTAFRATLPGNKLSGKFGPVQQRAIVFLCTAIQIVICALWLLIKPPFPDKALRYDHKK
IILECNTGSDAGFYAALGYVGLLSTICLILAFLGRKLPDNFNEAKFISFSMLIFCAVWVTFIPAYISSPGKYTVAVEIFAILSSAFGLLLCIFAPKCFIILIKPERNTRKHVMGKSKT
>marker.zebrafish_3_1_F
---
CPKEYWSNERRDKCLVKTIEFLSYTETMGIILTALSLFGASLTVATMIVFIHYRETPIVKANNSELSSLLLSSLFFCFLCPLTFIGEPTHWSCMLRHTAFGLTFALCISCVLGKTIVVVTAFRATLPGNKLSGKFGPVQQRAIVFLCTA
IQIVICALWLLIKPPFPDKALRYDHKKIILECNTGSDAGFYAALGYVGLLSTICLILAFLGRKLPDNFNEAKFISFSMLIFCAVWVTFIPAYISSPGKYTVAVEIFAILSSAFGLLLCIFAPKCFIILIKPERNTRKHVMGKS--
>marker.cichlid_3-1aa
---
CPKEYWSNERKDDCIPRTIEFLKYHEPMGIALTLVSLLGASLSLATMVVFINYRETPVIKASNFELSSLLLFSLFLCFLCPLTFLGRPTVWTCMLRHTAFGVTFALCISCVLGKTIVVVTAFKATFPGNNIAGKFGHAQQRIIICSCTL
IQIVICVLWLSLNPPYPHMLFTYSNKIIVLECNTGSQASFYAVLGYIGVLAIICLILAFMARKLPDNFNEAKLITFSLLIFCAVWITFIPAYISSPGKFTVAVEIFAILSSAFGLLSSIFAPKCYIIFLKPEKNTKKHVMGKI--
>Japanese_eel.locus142.1
------------------------------
MGIALTVVSLSGVCLAFATIVVFFNFKTTPIVKANNSELSFLLLFSLLLCFLCPLTFIGEPTIWSCMLRHTAFGITFALCISCVLGKTIVVVTAFRATLPNNNMARKFGPVQQRIIVFSCTTVQICICVLWLTLNPPFPDRTFKQDDKK
IILECNTGSETAFYAVLGYIGLLSAVCLVLAFLARKLPDNFNEAKLITFSMLIFCTVWLTFIPAYVSSPGKYTVAVEIFAILSSTFGLLFCIFAPKCYIILIKPEKNTRNNVMGKLRT
>Japanese_eel.locus141.1
------------------------------
MGIALTVVSLSGVCLALATIVVFFNFKTTPIVKANNSELSFLLLFSLLLCFLCPLTFIGEPTIWSCMLRHTAFGITFALCISCVLGKTIVVVTAFRATLPNNNMARKFGPVQQRIIVFSCTTVQICICVLWLTLNPPFPDRIFKQDDKK
IILECNTGSETAFYAVLGYIGLLSAVCLVLAFLARKLPDNFNEAKLITFSMLIFCAVWLTFIPAYVSSPGKYTVAVEIFAILSSTFGLMFCIFAPKCYIILIKPEKNTRNNVMGKLVK
>Spotted_gar.locus43.1
------------------------------
MGITLTTVSLFGLCLTVATISVFFSFRSTPIVKANNSELSSLLLFSLLLCFLCPLTFIGQPTAWSCMLRHTAFGITFALCISCVLGKTIVVVTAFRATLPNDNMAKNFGPVQQRIIVCSCTAVQVVICVLWLSLKPPFPDKNFKLSNKK
IILECNTGSEIAFYAVLGYIGLLAATCLVLAFLARKLPDNFNEAKLITFSMLIFCAVWVTFIPAYVSSPGKYTVAVEIFAILSSTFGLLFCICAPKCYIVLLKPEKNTRKHIMGKLPS
>Spotted_gar.locus42.1
------------------------------
MGITLTTVSLFGLCLTVATISVFFSFRSTPIVKANNSELSSLLLFSLLLCFLCPLTFIGQPTAWSCMLRHTAFGITFALCISCVLGKTIVVVTAFRATLPSNNMAKNFGPIQQKIIVCSCTAVQVVICVLWLSLKPPFPDKNFKLSNEK
IILECNTGSEIAFYAVLGYIGLLAATCLVLAFLARKLPDNFNEAKLITFSMLIFCAVWVTFIPAYVSSPGKYTVAVEIFAILSSTFGLLLCIFAPKCYIILLKPEKNTRKQIMGKIPS
>Reedfish.locus27719.1
------------------------------
MGIILTAMALAGLGLTTATAAIFMYYKQTPIVKANNSELSALLLLSLFFCFLCPLTFIGEPSVWSCMLRHTVFGITFVVCISCMLGKTVMVVIAFKATLPNNNLAKFFGPSKQRLLVFVCTGVQIIICTLWLNVDPPVPAKNFKHNNEI
IILECNTGSKVAFYAVLGYIGLLSCICFVLAFLARKLPDNFNEAKFITFSMLIFCTVWLTFIPAYISSPGKYTVAVEIFAILSSSFGLLLCIFFPKCYIILIKPEKNTKKHMLGKIPT
>Sterlet.locus278.1
-MKCPEEYWSNAKKDKCIPKEIEFLSFSEAFGITLTTVALMGLCFTIATATVIIYNKNTPIVKANNIELSCLLLFSLVLCFLCSLTFIGEPVVWTCMLRHTFFGIAFALSISCVLGKTIVVVTAFKATLPNSNMAKLFGPVQQQIIVFA
CTAVQIIICALWLNLSPPFPVKNLKHDKEKIILECNIGSAIGYSLVLGYIGMLSCICFVLAFLARKLPDNFNEAKYITFSMLIFCAVWLTFIPAYVSSPGKYTVAVEIFAILSSSFGLLVCIFVPKCYIILLKPEKNTRQYIMGKMPS
>Coelacanth.locus328.1
------------------------------
MGMILVVVAVLGASLTLVIAVIFMCNKHTAIIKANNSELSFLLLFSLLLCFLCSLTFIGKPTLLSCMVRQTFFCVTFVLCVSCILAKTVVVVAAFKATGPKSNIMRWFGPVQQRFIVFICTSMQILICIVWLIRYPPFPEENMNHHNEK
IILECNLGSEVIFYSVLGYIGILSCMCFVLAFLARKLPDNFNETKFITFSMLIFCAVWLTFIPAYISSPGKYTVAVEIFAILFSSFGLLFCIFVPKCYVILFKPEKNTRKHIMGKVAS
>Whale_shark.locus23.1
-MKCPLEYWSSKDSQSCILKEIEFLSFKDTMGIILMTVALFGACITLIIASTFLHHKDTPIVKANNSELSFLLLFSLVLCFLCSLTFIGKPSVWSCVLRHTVFGITFVLCMSCILAKTIVVLVAFKATVPQMNVMKWFGPVQQRAIVCA
CTFIQILICITWLIVAPPFPTKYTKYHTSKVILECNVGSPIAFYCVLGYIGILACLCFXLAFLARKLPGNFNEAKLITFSMLIFCAVWLTFIPAYVSSPGKYTVAVEIFAILSSSFGLLFCIFGPKCYIILFKPEKNTKRNLMRKLPS
>Brownbanded_bambooshark.locus1555.1
------------------------------
MGIILMMVALFGACITLIIASAFLYYKDTPIVKANNSELSFLLLFSLALCFLCSLTFIGEPSVWSCVLRHTVFGITFVLCMSCILGKTIVVLMAFKATVPQMNMMKWFGPVQQRAMVCACTFIQILICTIWLVVAPPFPTKYTKYHNSK
IILECNAGSPVAFYCVLGYIGILACVCFMLAFLARKLPGNFNEAKLITFSMLIFCAVWLIFIPAYVSSPGKYTVAVEIFAILSSSFGLLFCIFGPKCYIILFKPEKNTKQNLMRKIPS
>Great_white_shark.locus32.1
-MKCPLEYWSSDDGQRCLLKEIEFLSFEDTMGIILMMVELIGACITLVIASVFLYHKNTPIVKANNSELSFLLLFSLVICFLCSLTFIGKPSVWSCVLRHTLFGITFVLCMSCLLGKPIVVLMVFKATVPQKNPMKWFGPLQQRLIVCI
CTSIQILICVTCLVVAPPFPTKYMQYHNTKVILECNVGSPIAFYCVLGYIGILSCLCFLLAFLARKLPGNFNEAKLITCSILIFCAVWLKFIPAYVSSPGKYTVAVEILAILSSSFGLLLCIFGPKCYIIFFRPEKNTKRKLMKKIPS
>Cloudy_catshark.locus39.1
------------------------------
MGIILMTFALFGACITLIIASVFLYRKDTPIVKAHNSELSFLLLFSLVLCFLCSLTFMGKPSVWSCILRHTLFSVTFVLCMSCILGKTIVVLMAFKATVPQNNPMKWFGPLQQRLIVCACTFIQIVICVTWLAIAPPFPTKYLQYHNAK
VILECNVGSPIAFYCVLGYIGILSCLCFVLAFLARKLPGNFNEAKLITFSMLIFCAVWLTFIPAYVSSPGKYTVAVEIFAILSSSFGLLFCIFGPKCYIILFRPEKNTKRNLMGKIPS
>Little_skate.locus14.1
CMKCPLEYWSSQDRQQCVDKDIEFLSFDDTMGIILGAVALLGTLNTLVIASLFLYHKDTPIVKGNNAELSFLLLFSLGLCFLCSLTFIGRPSHWSCALRHTLFGIAFALCVSCLLVKTVVVLSAFKATGPQTRTMKWFGAAQQRLIVCG
CTLLQILICVTWLAMAPPFPSKNMRYHNTKILLECDVGSPIAFYCVLGYIGVLSGLCFVLAFLARELPGKFNEAKLITFSMIIFCAVWLTFIPAYVSSPGKYTVAVEIFAILSSSFGLLFCIFVPKCYIILFKPEENTKKGLMGKGTF
```

```
>Thorny_skate.locus31.1
CMKCPLEYWSSQDRQQCVGKDIEFLSFDDTMGMILGAVALLGTLNTLVIASLFLHRKDTPIVKGNNAELSFLLLFSLGLCFLCSLTFIGRPSHWSCALRHTLFGIAFALCVSCLLVKTVVVLSAFKATGPQTRTMKWFGAAQQRLIVCG
CTLLQILICVTWLAMAPPFPSKNMRYHNTKILLECDVGSPIAFYCVLGYIGVLSGLCFVLAFLARELPGKFNEAKLITFSMIIFCAVWLTFIPAYVSSPGKYTVAVEIFAILSSSFGLLFCIFVPKCYIILFKPEENTKKGLMGKGTF
>Smalltooth_sawfish.locus24.1
------------------------------
MGITLTTVSLLGTASTVGVAGVVWYYRRSPIIRANNSELSFLLLFSLALCFLCSLTFIGQPSEWSCVLRHTLFGVSFVLCISCVLAKTLVVLMAFKATLPGNNLMKWFGTLQQILSVLLCTAIQVVTCLVWLLLAAPFPVKQTKYYKDK
IIFECDLGSVTAFCCVLGYIGFLACISFVLAFLARKLPDNFNEAKLITFSMLIFCAVWLAFVPAYASSPGKYAVAVEIFAILASSFGLLICIFVPKCYIILLKPEQNTKKHLMGRTSQ
>Smalltooth_sawfish.locus39.1
------------------------------
MGITLTTVSLLGTASTVGVAGVVWYYRRTPIVRANNSELSFLLLFSLALCFLCSLTFIGQPSEWSCVLRHTLFGVSFVLCISCVLAKTLVVLMAFKATLPGNNLMKWFGTLQQILSVLLCTAVQVVTCLVWLLLAAPFPVKQTKYYKDK
IIFECDLGSVTAFCCVLGYIGFLACISFVLAFLARKLPDNFNEAKLITFSMLIFCVVWLAFVPAYASSPGKYAVAVEIFAILASSFGLLICIFVPKCYIILLKPEQNTKRHLMGRNCP
>Thorny_skate.locus32.1
--------------------------
MEDGTGMALTTVSLLGTASTVLMAGVVWYYRRTPIIRANNSALSFLLLFSLALCFLCSLSFIGRPSAWSCRLRHTLFGISFVLCLSCVLAKTLVVLVAFKSTLPGNKLMRWFGSLQQILSVLLCTAVQGGICLAWLLLAAPFPVKQTKY
YKEKIIYECDVGSMAAFCCVLGYIGFLACVSLALAFLARKLPDNFNEAKFITFSMLIFCAVWLAFIPAYASSPGKYAVAVEIFAILASSFGLLTCLFVPKCYIILLKPEQNTKRHLMGRTG-
>Little_skate.locus16.1
--------------------------
MEDGTGMTLTTVSLLGTASTVLMAGVVWYYRRTPIIRANNSALSFLLLFSLALCFLCSLSFIGRPSAWSCRLRHTLFGISFVLCLSCVLAKTLVVLVAFKSTLPGNKLMRWFGSLQQILSVLLCTAVQGGICLAWLLLAAPFPVKQTKY
YKEKIIYECDVGSMVAFCCVLGYIGFLACVSLALAFLARKLPDNFNEAKFITFSMLIFCAVWLAFIPAYASSPGKYAVAVEIFAILASSFGLLTCLFVPKCYIILLKPEQNTKRHLMGRTG-
>Great_white_shark.locus35.1
CIPCALEDWPNPQRNECLPKEIEFLSFKEAMGITLTTMSLLGTASTVSVAGVLWYYRKTPIVRANNSELSFLLLFSLMLCFLCSLTFIGQPSVWSCMLRHTLFGVSLVLCISCVLAKTLVVLMAFKATFPNNNLMKWFGTLQQIFSVLL
CTLVQVVICVVWLLVSSPFPVKQIKYYKEKIIFECDLGSVIAFCCVLGYIGFLSCMSFFLAFLARKLPDNFNEAKFITFSMLIFCAVWLTFIPAYVSSPGKYTVAVEIFAILASSFGLLICIFAPKCYIILLKPERNTKKHLMNKTG-
>Great_white_shark.locus33.1
------------------------------
MGIILMTMSLLGTANTVSVAGVLWYYRKTPIVRANNSELSFLLLFSLMLCFLCSLTFIGQPSVWSCMLRHTLFGVSLVLCISCVLAKTLVVLMAFKATFPNNNMMKWFGTLQQIFSVLLCTLVQVVICVVCLLVSSPFPVKQIKYYKEK
IIFECDLGSVKAFCCVLGYIGFLSCMSFVLAFLARKLPGNFNEAKFFTFSMLIFCAVWLTFIPAYVSSPGKYTVAVEIFAILASSFGLLICIFAPRCYIILLKPEINTKKYLMDKTCP
>Whale_shark.locus20.1
----------------------------------------------------------
RRPLVRANNSELSFLLLFSLMSCFLCSLIFIGQPSVWSCKLRHTLFGVSFVLCISCVLAKTLVVLMAFRATLPNNNIMKWFGTLQQLLSVLACTVVQGVICVAWLLVSSPFPVKQTKYYNEKIIFECVLGSVTAFCCVLGYIGLLSFVS
FVLAFLARKLPDNFNEAKFITFSMLIFCAVWLTFIPAYISSPGKYSVAVEIFAILASSFGLLICIFAPKCYIILLKPETNTKRHLMNKSGQ
>Whale_shark.locus22.1
------------------------------
VLLELQKIGPTYTASTVSVAGVLWYYRKTPTVRGHNSELSFLLLFSLILCFLCSLIVIGQXSVRSCKLRHTLFGVNFVLCISCVLAKTLVVLLAFRATVPNNNMLKWFGTLQQLLSILACTVVQVVICVAWLRMSSPFPVKQTKYYNEK
IIFECDLGSVTAFCCMLVYIRFLSYMSFVLLFLARKLPDNFNETKFITFSMLIFCAVWLTFIPAYISSPGKYSVTVETFAILASSFGLLICIFAPKCYIILLKPERNRKKYLMGKTCY
>Brownbanded_bambooshark.locus1554.1
CIPCALEDWSNLQKNQCIPKEIEFLTFDEAMGITLTTVSLLGTASTMSVAGVLWYYRKTPIVRANNSELSFLLLVSLMFCFLCSLTFIGQPSVWSCMLRHTLFGVSFVLCISCVLAKTLVVLMAFRATLPNNNMMKWFGTLQQLRSVLA
CTVVQVVICVAWLLVSSPFPVKQTKYYNEKIIFECDLGSVMAFCCVLGYIGLLSCMSFVLAFLARKLPDNFNEAKFITFSMLIFCAVWLAFVPAYISSPGKYAVAIEIFAILASSFGLLICIFAPKCYIILLKPERNTKKYLMAKTCP
>Elephant_shark.locus140.1
CIPCPEDSWSNLQRNKCIPKELEFLSFAEPLGLMLTTGSALGALSTIIVAAVLLYHRDTPIVRANNSKLSFLLLFSLTFCFLCSLTYMGQPSTWTCMLRHTLFGVSFVLCISCVLGKTLVVLIAFKATLPNSNVWKWLGTLQQVLSVFS
CTLIQVIICVVWLLVSPPSAARNVEYSSEKILLECNLGSVSAFCCVLGYIGFLSCMSFVLAFLARKLPDQFNEAKLITFSMLVFGAVWLAFIPAYVSSPGKYTVAVEIFAILSSSFALLICIFAPKCYVILLKPHKNTRKHLMDKTCP
>Zebrafish.locus475.1
CLLCPEDFWSNKHRNYCVPKEIEFLSYLEAFGMVLAAIAILGAVAAISVGVVFFRHRDTPLVRANNSELSFLLLISLTLCFICALTFLGQPSHWACPLRRISFGLTFALCLSCLLSKTLVVLIAFKSTLPGNNTARWFRPPQQRLGVFI
CSLLQGGVCIAWLTTASPYPVKNTWLYRDRIILECHLGSVVYFCCVLGYIGCLAAFCFILAFLARKLPDNFNEAKFITFSMLIFCAVWITFIPAYVSSPGKFTVAVEIFAILASSYGVLLCIFAPKCYIIIFMPEKNSKKYLMTQK--
>marker.zebrafish_1_1_F
---
CPEDFWSNKHRNYCVPKEIEFLSYLEAFGMVLAAIAILGAVAAISVGVVFFRHRDTPLVRANNSELSFLLLISLTLCFICALTFLGQPSHWACPLRRISFGLTFALCLSCLLSKTLVVLIAFKSTLPGNNTARWFRPPQQRLGVFICSL
LQGGVCIAWLTTASPYPVKNTWLYRDRIILECHLGSVVYFCCVLGYIGCLAAFCFILAFLARKLPDNFNEAKFITFSMLIFCAVWITFIPAYVSSPGKFTVAVEIFAILASSYGVLLCIFAPKCYIIIFMPEKNSKKYLMTQK--
>Zebrafish.locus830.1
--------------------------------
MVLAAIAILGAVAAISVGVVFFRHRDTPLVRANNSELSFLLLISLTLCFICALTFLGQPSHWACPLRRISFGLTFALCLSCLLSKTLVVLIAFKSTLPGNNTARWFRPPQQRLGVFICSLLQGGVCIAWLTTASPYPVKNTWLYRDRII
LECHLGSVVYFCCVLGYIGCLAAFCFILAFLARKLPDNFNEAKFITFSMLIFCAVWITFIPAYVSSPGKFTVAVEIFAILASSYGVLWCIFAPKCYIIIFMPEKNSKKYLMTQK--
>Reedfish.locus27724.1
CFQCPVDFWSNKEKDACHPKDIEYLSFSDTLGITSATISIFGAVLTTAVAIIFFLHKSTPIVRANNSELSFLLLLSLVFCFLCALTFIGEPTEWTCRLRRTSFSITFVLCLSCVLGKTIVVLMAFKATLPGNNVMRWFGPAQQRFSVFV
CTAVQCLICLVWLILSPPFPFKSTRYYNDRIILECDLGSVTLFCCVMGYIGFLACICFVLAFLARKLPDNFNEAKFITFSMLIFCAVWITFIPAYISSPGKYVVAVEIFAILSSSFGVLLCIFTPKCYVILVKPEKNTKKHLMSKTSV
>Sterlet.locus208.1
------------
MKNTCIPKVIEFLSFGEIMGIILSVLGVFGACLTLFVAAVFFHFRSTPIVRANNSEISFLLLFAILLCFLCSLLFIGEPSPWSCMLKHAAFGITFVLCISCVLGKTLVVLMAFKSTLPGNNIMKWFGPMQQRLSIFFFTFIQVLICILW
LVIAPPFPQKNAKLFNSRIIFECDLGSVTAFCCVLGYIGILSILCFILAFLARKLPDNFNEAKYITFSMLIFCAVWITFIPAYVSSPGKYTVAVEIFAILSSSFGVLICIFAPKCYIILLNPEKNTKKHLLGRPGT
>Sterlet.locus276.1
CEKCSSNYWSHVQKTKCIPKEIEYLAYEETMGIILAVVAIFGAGITAAVITVFFYYKDTPIVKANNSELSFLLLFSLILCFLCSLTFIGQPLTWSCMLRHTVFGISFVLCISCVLSKTVVVLMAFKAALPGSNVMRYFGPKQQRVGICF
CTLVQVVICMLWLSLYPPFPSKNTQ-QSEKIILECNIGSVLGFASVLGYIGSLACMCFVLAFLARKLPDNFNEAKFITFSMLIFCAVWITFIPAYVSSPGKYTVAVEIFAILSSSYGLLVCIFVPKCYIILLKPEKNTKKQMMSKG--
>Sterlet.locus209.1
CEKCSSNFWSNVQKTKCIPKEIEYLSYEETMGVILAVVAIFGAGITAAVITVFFYYKDTPIVKANNSELSFLLLFSLILCFLCSLTFIGQPLTWSCMLRHTVFGISFVLCISCVLSKTVVVLMAFKAALPGSNVMRYFGPMQQRAGIFF
CTLVQVVICMLWLTLYPPFPSKNTQ-QSEKIILECNIGSVLGFASVLGYIGSLACMCFVLAFLARKLPDNFNEAKFITFSMLIFCAVWITFIPAYVSSPGKYTVAVEIFAILSSSYGLLVCIFVPKCYIILLKPEKNTKKQMMSK---
>Coelacanth.locus335.1
CMKCPLGYWSNLHRNTCISTIVEYLSYKDTLGIIFVIVSVLGASVTITVMVIFYLHKDTPIVKANNSELSFLILMSLIPCFVCSLAFIGQPSVWSCMLRHTVFGISFALCISCVLGKTIVVLMAFQATLPGNNVMRYFGPLQQRGSIFI
CTLVQVIICLLWLTLSPPFPVKNTKYQSVKIILECNLGSKLAFSGLLGYIGFLACMCFAFAFLARKLPDNFNEAKFITFSMLIFCAVWITFIPAYISSPGKYTTAVEIFAILSSSFGLLTCIFLPKCYIILFKPEKNTKQHLMGKSIA
>Small-eyed_rabbitfish.locus24.1
CLKCPLDFWSNQERTQCIAKEIEFLSYEETMGIMLAVIALCGACVTKSVAAIFFCFRKSVIVRANNSELSFYLLFSLTLCFLCSVSFIGKPTHWSCMIRHTAFAISFTLCISCVLAKTVVVLMAFKATLPSNNALKWFGPVQQRAGIFI
SASVQVLICLIWLLSSPPFPSKNTQVSSAKIILECDVGSVVAFCCVFGYIGFLSAMCFILAFLARKLPDNFNEAKYITFSMLIFFVVWITFIPAYVSSPGKYTVAVEIFAILSSSFGLLICIFATKCYIVLLKPERNTKKHLMGRESL
>Elephant_shark.locus193.1
------------------------------
MGIMLTVIALCGACITKAVAAIFFCYRKTVIVRANNSELSFYLLFSLTLCFLCSVSFIGKPTHWSCMIRHTAFAISFTLCISCVLAKTVVVLIVFKATLPNNNTLKWFGPLQQRAGIFISVSVQVLICIIWLVSSPPFPSKNTQVSNAK
NILECDVGSVVDFCCVFGYIGFLSAMCFILAFLARKLPDNFNEAKYITFSMLIFFVVWIAFIPAYVSSPGKYTVAVEIFPILSSSLGMLICIFAPKCYILLLKPETNTKKHLMGRESL
>Reedfish.locus27405.1
------------------------------
MGITLTAVTLSGTCLTLAIGVVFLYYKNTPIVRANNSELSFLLLISLGLCFLCSLFFIGQPSEWSCIVRHAAFGVSFVLCISCILVKTIVVLMAFQATLPGNDIMKWFGPSQQRGSVFVSTLIQVLICVIWLAATPPLVYKNSGIQSSK
IILECTVGSAVAFGCVLGYIGLLGTSCFVLAFLARNLPDNFNEAKFITFSMLIFCAVWITFIPAYVSSPGKYTVAVEIFAILSSSYGLLGCIFAPKCYIILLRPDQNTKKHVMGRAVS
>Spotted_gar.locus167.1
------------------------------
MGITLTTVALSGASITLAVAIIFLYYKNTPIVRANNSELSFLLLFSLMMCFLCSLCFIGKPSIWSCKIQHIAFGVSFVLCISCLLVKTIVVLMAFQATLPGNDVMKWFGPTQQRSSVIVCTLIQVLQCAVWLSVSPPLPNKNTSYQNSK
IILECAVGSIVAFCCVLGYIGLLACLCFTLAFLARNLPDNFNEGKFITFSMLIFCAVWITFIPAYISSPGKYTVAVEIFAILSSSFGLLGCIFAPKCFIILLRPENNTKKFLMGRASA
>Thorny_skate.locus25.1
CLTCPIDYWSNQEHDKCVLKEIEFLSFEDTLGIILTTVALFGVCLTVVILAIFIHYRNTPVVRANNMELSFLLLVSLVLCFLCSLSFIGQPSVWSCMLRHTVFGVSFALCISCILGKTIVVLMAFSATLPNNNMMKYFGPRQQRASVFA
CTFFQVVICTLWLVSAPPFPIKNTKHQSTKVILECDVGSVEAFSCVLGYIGLLAAISVILAFFARKLPDNFNEAKFITFSMLIFSAVWITFIPAYISSPGKYTVAVEIFAILSSSFGLATCNFAPKCYIILLKPEQNTKKHLMGKGG-
>Little_skate.locus4.1
CLTCPIDYWSNQEHDKCVLKEIEFLSFEDTLGIILTTVALFGVCLTVVILAIFIHYRNTPVVRANNMELSFLLLVSLVLCFLCSLSFIGQPSVWSCMLRHTVFGVSFALCISCILGKTIVVLMAFSATLPNNNMMKYFGPRQQRASVFA
CTFFQVVICTLWLVSAPPFPIKNTKHQSTKVILECDVGSVEAFSCVLGYIGLLAAISVILAFFARKLPDNFNEAKFITFSMLIFSAVWITFIPAYISSPGKYTVAVEIFAILSSSFGLATCNFAPKCYIILLKPEQNTKKHLMGKGG-
>Smalltooth_sawfish.locus21.1
CLTCPIDYWSNQEHDKCVLKEVEFLSFEDTLGIILTTVALFGVCITVVILAIFIHYRNTPVVRANNMELSFLLLISLVLCFLCSLTFIGQPSVWSCMLRHTVFGVSFALCISCILGKTIVVLMAFSATLPNNNLMKYFGPQQQRACVFT
CTFLQVIICILWLTNSPPFPVKNIKHQSAKVILECDVGSVMAFSCVLGYIGLLAAISVVLAFLARKLPDNFNEAKFITFSMLIFSAVWITFIPAYISSPGKYTVAVEIFAILSSSFGLAACNFAPKCYIILLKPEQNTKKHLMGKGAK
>Cloudy_catshark.locus65.1
CLTCPIDYWSNQEHDQCVLKEIEFLSFEDTLGIILTTIALFGTGITTVILVIFICYRNTPVVRSNNIELSFLLLVSLLLCFLCSLTFIGQPTTWSCIFRHTVFGVSFALCISCILGKTIVVLMVFSATLPNNDMMKYFGPLQQRASVFA
CTFLQVIICILWLAISPPFPVKNIKYQNAKIILECDVGSVTAFCCVLGYIGLLAAISVVLAFLARKLPDNFNEAKFITFSMLIFSAVWVTFIPAYISSPGKYTVAVEIFAILCSSFGVAACNFAPKCYIILLKPDQNTKKHVMGRPAV
>Brownbanded_bambooshark.locus518.1
CFTCPIDYWPNQEHDQCVLKEIEFLSFEDTLGIILTTIALFGTGITIVMLVIFIHYRNTPVVRANNVELSFLLLISLVLCFLCSLTFIGQPTTWSCILRHTVFGTSFALCISCILGKTIVVLMAFSATLPNNNMMKYFGPLQQRASVFA
CTFLQVIICMLWLATSPPVPVKNTKYQSAKIILECDVGSATAFWCMLGYIGLLAGTSVILAFLARKLPDNFNEAKFITFSMLTFSAVWVTFIPAYISSPGKYTVAVEIFAILSSTFGLAACNFVPKCYIILIKPDQNTKKHLMGRAVA
>Small-eyed_rabbitfish.locus115.1
------------------------------
MGIILTVVALSGTCTTIAVGVVFFYHRHTPIVRANNSELSFLLLLSLCFCFLCSLTFIGQPLVWSCMLRHTAFGISFALCISCILGKTIVVLMAFKATLPNNNHMKWFGPTQQRGIVFVSTGIQVIICTLWLATSPPFPAQN-
KFQSAKLLLECDVGSLTAFSCMLGYIGLLACLCFVLAFFARKLPDNFNEAKFITFSTIIFCVVWLTFIPAYVSTPGKYTVAVEIFAILSSSTGVLGCIFVPKCYVILLKPERNTKKHLMGK---
>marker.zebrafish_2_1_F
---CPEDYWSNVNRTECIQKVIEFLSH-
DAMGMTLTVIAVAGAFLTITVLGIFLYNKGTPIVRVNNSELSFFILVSLTFCFLCALIFIGEPTSWSCMLRHTAFSITFSLCISCILGKTLVVLAAFTATRPGNNIMKWLGPTQQRIIIFCCTLVQVLICTVWLVVSPPFPYRNTKYQQ
SKIILDCSVGSDLAFWCVLGYIGLLACVCFFLAFLARKLPGNFNEAKYITFSMIIFCTVWLAFVPAYVSSPGKFTTAVEIFAILASSFGLLFCLFAPKCYIILVKPEKNTKQHLIGKV--
>Zebrafish.locus505.1
------------------------------
MGMTLTVIAVAGAFLTITVLGIFLYNKGTPIVRVNNSELSFFILVSLTFCFLCALIFIGEPTSWSCMLRHTAFSITFSLCISCILGKTLVVLAAFTATRPGNNIMKWLGPTQQRIIIFCCTLVQVLICTVWLVVSPPFPYRNTKYQQSK
IILDCSVGSDLAFWCVLGYIGLLACVCFFLAFLARKLPGNFNEAKYITFSMIIFCTVWLAFVPAYVSSPGKFTTAVEIFAILASSFGLLFCLFAPKCYIILVKPEKNTKQHLIGKVTK
>Zebrafish.locus844.1
------------------------------
MGMTLTVIAVAGAFLTITVLGIFLYNKGTPIVRVNNSELSFFILVSLTFCFLCALIFIGEPTSWSCMLRHTAFSITFSLCISCILGKTLVVLAAFTATRPGNNIMKWLGPTQQRIIIFCCTLVQVLICTVWLVVSPPFPYRNTKYQQSK
IILDCSVGSDLAFWCVLGYIGLLACVCFFLAFLARKLPGNFNEAKYITFSMIIFCTVWLAFVPAYVSSPGKFTTAVEIFAILASSFGLLLCLFAPKCYIILVKPEKNTKQHLIGKVTK
>Zebrafish.locus506.1
------------------------------
MGMTLTVIAVIGAFLTITVLVIFLYNKGTPIVRVNNSELSFFILLSLTLCFLCALIFIGEPTSWSCMLRHTAFSITFSLCISCILGKTLVVLAAFTATRPGNNIMKWLGPTQQRIIIFCCTLVQVLICTVWLVVSPPFPYRNTKYQQSK
IILDCSVGSDLAFWCVLGYIGLLACVCFFLAFLARKLPGNFNEAKYITFSMIIFCAVWLAFVPAYVSQPGKFTTAVEIFAILASSFGLLLCLFAPKCYIILMKPEKNTKQHLMGKVIT
>Zebrafish.locus845.1
```

```
------------------------------
MGMTLTVIAVIGAFLTITVLVIFLYNKGTPIVRVNNSELSFFILLSLTLCFLCALIFIGEPTSWSCMLRHTAFSITFSLCISCILGKTLVVLAAFTATRPGNNIMKWLGPTQQRIIIFCCTLVQVLICTVWLVVSPPFPYRNTKYQQSK
IILDCSVGSDLAFWCVLGYIGLLACVCFFLAFLARKLPGNFNEAKYITFSMIIFCAVWLAFVPAYVSQPGKFTTAVEIFAILASSFGLLLCLFAPKCYIILMKPEKNTKQHLMGKVIT
>Zebrafish.locus507.1
------------------------------
MGLTLTVIAVAGACLTLAVFSVFLYYKNTPVVRINNSELSFFILLSLTLCFLCALIFIGEPTSWSCMLRHTAFSITFSLCISCILGKTLVVLAAFTATRPGNNLMKWLGPTQQRIIIFCCTLIQVLICTVWLVASPPFPYRNTKYQQSK
IILDCSVGSDLAFWCVLGYIGLLACVCFFLAFLARKLPGNFNEAKYITFSMIIFCAVWLAFVPAYVSSPGKFTTAVEIFAILASSFGLLLCLFTPKVYIILVKPEKNTKQHLMGKD--
>Zebrafish.locus846.1
------------------------------
MGLTLTVIAVAGACLTLAVFSVFLYYKNTPVVRINNSELSFFILLSLTLCFLCALIFIGEPTSWSCMLRHTAFSITFSLCISCILGKTLVVLAAFTATRPGNNIMKWLGPTQQRIIIFCCTLVQVLICTVWLVVSPPFPYRNTKYQQSK
IILDCSVGSDLAFWCVLGYIGLLACVCFFLAFLARKLPGNFNEAKYITFSMIIFCAVWLAFVPAYVSSPGKFTTAVEIFAILASSFGLLLCLFTPKVYIILVKPEKNTKQHLMGKD--
>Japanese_eel.locus123.1
------------------------------
MGLILTVIAVVGACLTVTVLAVFLYHRNTPIVKVNNSELSFFILLSLTLCFLCALVFIGEPTSWSCMLRHTAFSITFSLCISCILGKTLVVLAAFTATRPGNNLMKWLGPKQQRFIISACTFIQVIICAVWLITAPPFPNKNTQYHRSR
IILECSVGSEQAFWCVLGYIGLLASLCFILAFLARKLPGNFNEAKYITFSMLIFCAVWLTFVPAYVSSPGKFTVAVEIFAILASSFGLLLCLFAPKCYIILLKPQKNTKQHLMGRLTN
>Japanese_eel.locus115.1
------------------------------
MGLTLTVIAVVGACLTVAVLAVFLYHRNTPIVKVNNSELSFFILLSLTLCFLCALVFIGEPTSWSCMLRHTAFSITFSLCISCILGKTLVVLAAFTATRPGNNIMKWLGPKQQRFIISACTFIQVIICAVWLITAPPFPNKNTQYHRSR
IILECSVGSEQAFWCVLGYIGLLAALCFILAFLARKLPGNFNEAKYITFSMLIFCAVWLAFIPAYVSSPGKFTVAVEIFAILASSFGLLLCLFAPKCYIILLKPEKNTKQHLMGRVVT
>marker.medaka_2_1_F
---CPEDFWSNEDRTICIPKKVEFLAY-
DSLGIALTVISVVGACLTIGVFLVFFHHRNTAVVRVNNSELSFFILFALTFCFLCSLVFIGEPTRWSCMLRHTAFSITFSLCISCILAKTLVVLAAFTATRPGDNIMKWLGPKHQRVIIFSCTMVQVIICAAWLIDASPYPSRNTKYER
SKIILECSVGSSLAFWCVLGYIGLQACLCFVLAFLARKLPGNFNEAKFITFSILIFCAVWLAFIPAYISSPGIYADAVESFAILASSFGLLFCLFAPKCYIILLKPEKNTKQHLMGKE--
>marker.fugu_2_1_F
---CPEDFWPNPNRTVCIPKKVEYLAY-
DSLGIALMVTSVVGACATIATFAVFFYHRNTAIVRVNNAELSFFILLSLVLCFLCSLVFIGEPTTWSCMLRHTAFSITFSLCFSCILGKTLVVLAAFTATRPGHNIMKWLGPKQQRTIIFSCTLVQVVICTAWLIDAPPAPFRNTEYEH
SKIILECSVGSSVGFWCVLGYIGLQACLSFILAFLARKLPGNFNEAKFITFSMLIFCAVWLAFIPAYVSSPGNYSDAVESFAILASSFGLLFCMFAPKCYIILLKPERNTKQHLMGKE--
>Spotted_gar.locus143.1
CIPCPPNYWSNAAKTDCIPKEVEYLSY-
DEMGITLAVIALLGACLTIGVLVVFLHSRNTPIVRVNNSELSFFILLSLTLCFLCSVAFIGQPTYWSCMFRHTAFSVTFSMCISCILGKTLVVLAAFKATRPGSNVMKWFGPRQQRMIIFACTAVQVVTCTIWLIAAPPFPLKNTVYQR
SRIILECSTGSDLAFWCVLGYIGVLACLCFVLAFLARKLPDNFNEAKYITFSMLIFCVVWLVFIPAYVSSPGKYTVAVEIFAILASSFGLLVCIFAPKCYIILLKPEKNTKHHLMGRVNA
>Sterlet.locus191.1
------------------------------
MGVTLTVIALFGACLTIGVLAVFLHYKNTPMVRVNNSELSFFILLSLALCFLSSIAFIGEPANWSCMFRHTVFSITFSLCISCILGKTVVVLMAFKATQPGSNIMKWFGPIQQRIMISACTSVQIIICAIWLIAMPPFPSKNTKYQSSK
IILECDVGSTLAFWCVLGYIGVLACMCFVLAFLARKLPGNFNEAKYITFSMLIFCAVWIAFIPAYVSSPGKYTVAVEIFAILSSSFGLLFCIFAPKCYIILLKPQKNTKQYIMGKASA
>Sterlet.locus186.1
------------------------------
MGVTLTVIALFGACLTTGVLAVFLHYKNTPIVRVNNSELSFFILLSLALCFLSSIAFIGEPANWSCMFRHTVFSITFSLCISCILGKTVVVLMAFKATQPGSNIMKWFGPIQQRIMISACTSVQIIICAIWLIAMPPFPSKNTKYQSSK
IIVECDVGSTLAFWCVLGYIGVLACMCFVLAFLARKLPGNFNEAKYITFSMLIFSAVWIAFIPAYVSSPGKYTVAVEIFAILSSSFGLLLCIFAPKCYIILLKPKKNTKQYIMGK---
>Sterlet.locus287.1
-MQCPADFWSNAERTECIPKEIEYLTY-
DEMGVTLTVIALFGACLTIGVLAVFLHYKNTPIVRVNNSELSFFILQSLALCFLSSIAFIGQPANWSCMFRHTVFSITFSLCISCILGKTVVVLMAFKSTQPGSNIMKWFGPIQQRIMISACTSVQIIICAIWLIAMPPFPSKNTKYQS
SKIILECDVGSTLAFWCVLGYIGVLACMCFILAFLARKLPGNFNEAKFITFSMLIFCAVWIAFIPAYVSSPGKYTVAVEIFAILSSSFGLLFCIFAPKCYIILLKPEKNTKLYIMGKASG
>Sterlet.locus289.1
------------------------------
MGVTLTVIALFGACLTIGVLAVFLHYKNTPIVRVNNSELSFFILQSLALCFLSSIAFIGQPANWSCMFRHTVFSITFSLCISCILGKTVVVLMAFKSTQPGSNIMKWFGPIQQRIMISACTSVQIIICAIWLIAMPPFPSKNTKYQSSK
IILECDVGSTLAFWCVLGYIGVLACMCFVLAFLARKLPGNFNEAKFITFSMLIFCAVWIAFIPAYVSSPGKYTVAVEIFAILSSSFGLLFCIFAPKCYIILLKPEKNTKQYIMGR---
>Reedfish.locus40415.1
CTHCPSDFWSNAERTECIPKEIEFLTY-
DEIGASLAVIALFGTCLTFGVLGIFLYYKSTPIVRVNNSELSFFILFSLALCFLCSIAFIGEPGSWSCMLRHTVFSVTFSLCISCILGKTVVVLMAFKATQPGNNLMKYFGPLQQRMMILSCTLIQIVICAIWLISTPPFPVKNTKYQN
SKIILECHVGSTLAFWFVLGYVGILACLCFVLAFLARKLPDNFNEAKYITFSMLIFCSVWLAFIPAYVSSPGKYTVAVEIFAILSSSFGLLFCIFAPKCYIILLKPEKNTKQYLMRKVNK
>Reedfish.locus40403.1
CTQCPSDFWSNAERTECIPKEIEFLAY-
DEIGASLTVIALFGACLTFGVLGIFLYYKSTPIVRVNNSELSFFILISLALCFLCSIAFIGEPADWSCMFRHTVFSVTFSLCISCILGKTVVVLMAFKATQPGSTIMKWFGPLQQRIMIFCCTFIQIIICAIWLISMPPFPVKNTKYNN
SKIILECHVGSALAFWCVLGYIGVLACMCFVLAFLARKLPGNFNEAKYITFSMLIFCSVWLAFIPAYVSSPGKYTVAVEIFAILASSFGLLFCIFAPKCYIILLKPEKNTKKGILGKSNT
>Coelacanth.locus332.1
------------------------------
MGIVLTLVALFGVCLTTTVILVFFYYRSTPIVRANNSELSFLILLSLILCFLCSIIFIGQPTTWNCILRYTVFGISFSLCISCILCKTVVVLMAFKVTVPGSSIIKLFGPVQQRAVISACGLIQVIICIVWLTMAPPYAFKNTQYQSGK
IILECGVESALAFWSVLGYIGLLACICFLLAFFVRKLPDNFNEAKYITFSMLIFSAVWIAFIPAYVSSPGKYTVAVQIFAILSSSFSLLTCIFFPKCYIILLKSENNSKQFLMGRK--
>marker.medaka_15_1_F
---
CPAEFWSNTERTVCVPRQLDFLSFNETLGVTLTAVAVSGATVTTAVFVVFLHHRQTPVVRANNSELSFLLLLSLKLCFLCSLLFIGRPSYWACRFQQAAFGISFVLCVSCLQVKTIVVLAAFRSARPGAALMKWFGPGQQRGSVCFFTS
IQVVICIIWLTLSPPIPQPDLEIPGLKVTLKCAMASVVGFSVVLGYIGLLGCTSLLLAFLARKLPDNFNEAKLITFSMLIFCAVWVAFVPAYVSSPGKYAVAVEVFAILASSFGLLFCIFAPKCFIILLRPEKNTKKHLMAR---
>marker.cichlid_15-2aa
---
CPSEFWSNAKRTTCIPRQLDFLSFNETLGITLTAVAVSGVTVTTAVFVVFLHYRQTPMVRANNSELSFLLLVSIKLCFLCSLVFIGRPSDWSCRFQQAAFGISFVLCISCLKVKTIVVLAAFRSARPGAALMKWFGPGQQRGSVFFFTC
VQVIICIIWLSISPPVPKSDLDVPGLKVTLKCATGSVVGFSLVLGYIGLLGCTSFLLAFLARKLPDNFNEAKLITFSMLIFCAVWVAFVPAYVSSPGKYAVAVEIFAILASSYGLLFCIFAPKCFIILLRPEKNTKKHLMGR---
>marker.cichlid_15-1aa
---
CPLELWSNAKRTACIPRQLDFLAFNETLGITLTTAAVTGVTVTTVVFVVFLHYRHTPVVRANNSELSFLLLVSIKLCFLCSLVFIGRPSVWSCRFQQAAFGISFVLCVSCLKVKTIVVLAAFRSARPGAALIRWFGPGQQRGSICLLTC
IQIIICTIWLSLIPPVPRQDLGFQGSKVTLECAMVSVVGFCLILGYIGLLACTSLILAFFARKLPDNFNEAKLITFSMLIFCAVWVAFVPAYLSSPGKYTVAVEVFAILASSYGLLFCIFAPKCFIILLRPERNTKKYMMAR---
>marker.fugu_15_2_F
---
CPPEFWSNDGRTACVPRQLDFLSFNETLGVALTAVAVSGAVVTTAVFVVFLHYRHTPMVRANNSELSFLLLLSLKLCFLCSLVFIGRPSVWSCRFQQAAFGISFVLCVSCILVKTLVVLAVFRSAQPDCATMKWFGPSQQRGSVGLFTS
IQIVICGIWLSVSPPKPERDLGFQGSKVTLECAMASVVGFSLVLGYIGLLACTCLLLAFLARKLPDNFNEAKLITFSMLIFCAVWVAFVPAYVSSPGKYVVAVEIFAILASSYGLLFCIFAPKCFIILLRPEKNTKKHLMSRN--
>marker.zebrafish_9_13_FN
---
CPKEFWSNSYKNECVARETEFLSVKETMGITLMSVAVSGAVMTTTVAVIFLYHRNTPIVKANNSELSFLLLLSLKLCFLCALVFVGQPSLWSCRIQQAAFGISFVLCISCILVKTFVVLIAFHSTRPESSLIKWFGLGKQRGIVLVFTC
VQVVICAIWLCVSPPLPYQNFGMHRSKVILECTIGSVVGFTCVLGYIGFLATVCFLLAFFARKLPDNFNEAKYITFSMLIFCAVWVAFVPAYVSSPGKYSVVVEVFAILASSFGLLFCIFVPKCYIILLKPENNTKKFLMGKE--
>Zebrafish.locus539.1
------------------------------
MGITLMSVAVSGAVMTTTVAVIFLYHRNTPIVKANNSELSFLLLLSLKLCFLCALVFVGQPSLWSCRIQQAAFGISFVLCISCILVKTFVVLIAFHSTRPESSLIKWFGLGKQRGIVLVFTCVQVVICAIWLCVSPPLPYQNFGMHRSK
VILECTIGSVVGFTCVLGYIGFLATVCFLLAFFARKLPDNFNEAKYITFSMLIFCAVWVAFVPAYVSSPGKYSVVVEVFAILASSFGLLFCIFVPKCYIILLKPENNTKKFLMGKE--
>Zebrafish.locus815.1
CYRCPKEFWSNSYKNECVARETEFLSVKETMGITLMSVAVSGAVMTTTVAVIFLYHRNTPIVKANNSELSFLLLLSLKLCFLCALVFVGQPSLWSCRIQQAAFGISFVLCISCILVKTFVVLIAFHSTRPESSLIKWFGLGKQRGIVLV
FTCVQVVICAIWLCVSPPLPYQNFGMHRSKVILECTIGSVVGFTCVLGYIGFLATVCFLLAFFARKLPDNFNEAKFITFSMLIFCAVWVAFVPAYVSSPGKYSVVVEVFAILASSFGLLFCIFVPKCYIILLKPENNTKKFLMGKE--
>Japanese_eel.locus275.1
------------------------------
MGITLTTIALSGSGITVAVGVVFLYHRHTPLVKANNSELSFLLLLSLTLCFLCALVFVGRPSEWSCLAQHLVFGISFVLCLSCILVKTIVVLVAFRSARPGANMIKWFGLVQQRGSVVLFTGIQVFICILWLSLSPPYPHRNTGFQGSK
LILECAVGSALGFGCVLGYIGLLAAICFLLAFLARKLPDNFNEAKFITFSMLIFCAVWIAFVPAYVSSPGKYTVAVEIFAILASSFGLLLCIFAPKCYIILLRPENNTKKFLMGKT--
>Japanese_eel.locus263.1
-------------------------------------------------------
HRHTPLVKANNSELSFLLLLSLTLCFLCALVFVGRPSEWSCLAQHLVFGISFVLCLSCILVKTIVVLVAFRSARPGASMIKWFGLVQQRGSVVLFTGIQVFICILWLSLSPPYPHRNTGFQGSKLILECAVGSTLGFACVLGYIGLLAA
ICFLLAFLARKLPDNFNEAKFITFSMLIFCAVWIAFVPAYVSSPGKYTVAVEIFAILASSFGLLLCIFAPKCYIILLRPENNTKKFLMGKT--
>Japanese_eel.locus266.1
------------------------------
MGITLTTIALSGSGITVAVGVVFLYHRHTPLVKANNSELSFLLLLSLTLCFLCALVFVGRPSEWSCLAQHLVFGISFVLCLSCILVKTIVVLVAFRSARPGASMIKWFGLVQQRGSVVLFTGIQVFICILWLSLSPPYPHRNTGFQGSK
LILECAVGSALGFAFVLGYIGLLAAICFLLAFLARKLPDNFNEAKFITFSMLIFCAVWVAFVPAYVSSPGKYTVAVEIFAILASSFGLLLCIFAPKCYIILLRPENNTKKFLMGKT--
>Japanese_eel.locus360.1
------------------------------
MGIILAALSVSGVVLTTTVIAAFFFHRDTPLIRANNAELSFMLLLSLSLCFLCALAFIGRPTPWSCMLRHTLFGISFVLCISCVLSRTVVVLVAFRASVPGSNIMRYFGPVQQRLAISLCTSVQMLICVLWLILSPPIPAE-
VQERSPRIILKCDVGSGAGFACVLGYIGLLAAICFLLAFLARKLPDNFNETKFITFSMLIFCAVWITFVPAYVSSPGKYTVAVEIFAILASGYGLLLCIFAPKCYIILLRPDKNTKKKIMAK---
>Japanese_eel.locus380.1
------------------------------
MGIILAALSVSGVVLTGCVITTFFFHRDTPLVRANNAELSFLLLLSLSLCFLCALAFIGRPAPWSCMLRHTLFGISFVLCISCVLSRTVVVLVAFRASVPGSNIMRYFGPVQQRLAISLCTSVQVLICVLWLVLSPPVPAE-
VQGRSARIILECDVGSGAGFACVLGYIGLLAAICFLLAFLARKLPDNFNEAKFITFSMLIFCAVWITFVPAYVSSPGKYTVAVEIFAILASSYGLLLCIFAPKCYIILLRPDRNTKKKMMAK---
>Japanese_eel.locus394.1
CQKCPDRFWSNAARTTCIPKEVEFLSFQETMGIILAALSVSGVVLTTTVLGTFFFHRDTPLVRANNAELSFLLLLSLSLCFLCALAFIGRPSLWSCMLRHTLFGISFVLCISCVLSRTVVVLVAFRASVPGSNIMRYFGPVQQRLAISL
CTSVQVLICVLWLVLSPPIPSE-IQGRSARIILECDVGSGAGFACVLGYIGLLAAICFLLAFLARKLPDNFNEAKFITFSMLIFCAVWITFVPAYVSSPGKYTVAVEIFAILASSYGLLLCIFAPKCYIILLRPDKNTKKKMMSKEKS
>Japanese_eel.locus396.1
CQKCPDRFWSNAARTTCIPKEVEFLSFEEIMGIILAALSVSGVVLTTSVLATFFFHRDTPLVRANNAELSFLLLVSLSLCFLCALAFIGRPTPWSCMLRHTLFGISFVLCISCVLSRTVVVLVAFRASVPGSNIMRYFGPVQQRLAISL
CTSVQVLICVLWLVLSPPIPAE-VQGRSARIILECDVGSGAGFACVLGYIGLLAAICFLLAFLARKLPDNFNEAKFITFSMLIFCAVWITFIPAYVSSPGKYTVAVEIFAILASSYGLLLCIFAPKCYIILLRPEKNTKKKMMAK---
>Zebrafish.locus474.1
-MRCPEKFWSNTEKTKCIPKVVEFLSLQDTMGIVLTVLSVTGATLTTTVLATFFHHRDTPLVRAYNSELSFLLLMSLTLCFLCALVFIGRPAAWNCMLRHTLFGVSFVTCIGCILSKTVVVLVAFRATRPGSHLMQYFGPIQQRAAIFI
CTLVQVVICLLWLLLDPPQPTESAGELGARLILQCTVGSVVGFVFVLGYIGMLAVVCFLLAFFARKLPDNFNEAKFITFSMFIFCAVWAAFVPAYISSPGKYTVAVEIFAILASSYGLLICIFTPKCYIILLKPEKNTKKTMMAR---
>Zebrafish.locus828.1
-MRCPEKFWSNTEKTKCIPKVVEFLSLQDTMGIVLTVLSVTGATLTTTVLATFFHHRDTPLVRAYNSELSFLLLMSLTLCFLCALVFIGRPAAWNCMLRHTLFGVSFVTCIGCILSKTVVVLVAFRATRPGSHLMQYFGPIQQRAAIFI
CTLVQVVICLLWLLLDPPQPTESAGELGARLILQCTVGSVVGFVFVLGYIGMLAVVCFLLAFFARKLPDNFNEAKFITFSMFIFCAVWAAFVPAYISSPGKYTVAVEIFAILASSYGLLICIFTPKCYIILLKPEKNTKKTMMAR---
```

```
>marker.zebrafish_11_1_F
---
CPEKFWSNVERTKCVPKIVEFLSLQDTMGIVLTVLSVTGATLTTTVLATFFHHRDTPLVRANNSELSFMLLVSLTLCFLCALVFIGRPATWNCMLRHTLFGVSFVICIACILSKTVVVLVAFQATLPGSNLMQYFGPIQQRAGILVCTM
VQVVICLLWLLLAPPLPTERAGEFGARVILQCTVGSVVGFALVLGYIGLLAVVCFLLAFFARKLPDSFNEAKFITFSMLIFCTVWIVFVPAYVSSPGKYTVAVEIFAILASSYGLLLCIFTPKCYIILLKPEKNTKKNMMAK---
>Zebrafish.locus473.1
CIRCPEKFWSNVERTKCVPKIVEFLSLQDTMGIVLTVLSVTGATLTTTVLATFFHHRDTPLVRANNSELSFMLLVSLTLCFLCALVFIGRPATWNCMLRHTLFGVSFVICIACILSKTVVVLVAFQATLPGSNLMQYFGPIQQRAGILV
CTMVQVVICLLWLLLAPPLPTERAGEFGARVILQCTVGSVVGFALVLGYIGLLAVVCFLLAFFARKLPDSFNEAKFITFSMLIFCTVWIVFVPAYVSSPGKYTVAVEIFAILASSYGLLLCIFTPKCYIILLKPEKNTKKNMMAK---
>marker.fugu_12_1_F
---
CLPEFWSNADRTACVPKQVEFLSFGDTIGIALLVVSLIGSFLTCAVALVFFYHRTSPIVRANNSDLSFLLLFSLTLCFLCSLTFISPPSQWSCMLRHTAFGITFVLCISCILGKTIVVLMAFRATLPGSDVMKWFGPGKQKAIITFSTL
VQVVICTVWLVVAPPTPRQYMPRESAIIILLCDEGSTIAFSLVLGYIGVLACMCFLLAFLARKLPDNFNEARLIAFSMLIFCAVWVAFVPAYISSPGKYSTLTEIFAILASSYGLLGCIFAPKCYIILMKSEKNTRKHLMSKS--
>marker.cichlid_12-1aa
---
CLPEFWSSAEKTACIPKQMEFLSFSDTMGIILMVISLTGIFSTCFVAFIFYCHRSTPIVRANNSELSFLLLLSLTLCFLCSLIFISQPSKLSCLLRHTAFGITFVLCISCILGKTIVVLIAFKATLPGSHVIKWFGPLQQRIIITCCTL
VQVIICTVWLVVAPPMPQKLMPRDSPIVILLCDKGSHLAYALVLGYIGLLAFLCLVLAFLARKLPDNFNEARIITFSMIIFCAVWIAFIPAYISSPGKYSTVTEVFAILASSYGLLGCIFAPKCYIILLRPEKNTRKHLMSKT--
>Japanese_eel.locus384.1
------------------------------
MGIALLTVALLGVCCTVVVAFIFVRHRTTPIVRANNSELSFLILCSLVLCFLCALAFIGQPTTWSCMLRHSIFGIAFVLCISCILGKTIVVLIAFRATLPGSKVMQLFSPKRQRAIIFSCTLMQVLICSLWLALSPPSPQRLLMRESAR
IILLCNVGSALAFSLVLGYIGLLSCVCFLLAFLARKLPGNFNEAKLITFSMLIFFAVWIAFIPAYVSSPGKYTTATEIFAILASSFGLLACLFFPKCYIILLKPEKNTRKHLMPKSEK
>Zebrafish.locus795.1
CVKCSPEFWSNTRRDTCIPKVVEFLSYNDTMGITLLSVALLGSCFTLAVTLIFALKRHTPLVRANNSEISFLILSSLLLCFLCALAFIGRPTTWSCGLRHTAFGIAFSLCLSCVLGKTLVVLMAFKASLPGSNVMRWFGPLQQRGIIFM
CTAVQVVICCTWLCVAPPIPHMLMTHETARIILLCDVGSSFAFSLVLGYIGLLAAVCFLLAFFARKLPDNFNEAKFITFSMLIFCAVWIAFVPAYISSPGKYTVAVEIFAILASSYGLLLCIFTPKCYIILLKPEKNTKRYVMSKSNS
>marker.cichlid_14-3aa
---
CPEYYLSDNDKVKCVAGEEEFLSFYDAMGIILVALTLLGFILTSIITIVFHSFRFTPIIKANNSEISFLLLLSLKLCFLCSLVFIGQPSWWTCRLRQAAFGISFALCLSCVLFKTIVVLLAFRTNVPGSRGRMLFGTAQQRILIICTTA
PQICLCAGWVLAAPPFPFRNPNYQTGRIVVECKEPWPPGFYLLLGYIGLLAFACLLLAFLGRKLPDTFNEAKLITFSMLIFWAVWISFIPAYVSAPGKFSVAVEIFAILSSSFGLLLCIFIPKCYIILLRPERNIKKGMTGKY--
>marker.cichlid_14-1aa
---
CPEYFWSDKEKVKCVAGVEEFLSFYDKMGIILVAITLLGFILTTIITIVFHRFRYTPIVKANNAEISFLLLLSLKLCFLCSLVFIGQPSWWTCRLRQAAFGISFVLCLSCLLVKTIVVLLAFRANVPGSRGQMLFGTSQQRILILCTTA
PQICLCTGWVLGAPPFPFRNPYYQTGKIVVECKEPWPPGFYLLLGYIGLLAFVCLLLAFLGRKLPDRFNEAKFITFSMLIFWAVWISFIPAYVSSPGNFSVAVEIFAILASSYGLLLCIFVPKCYIILLHPEKNIKKGMTRKC--
>marker.pufferfish_14_1_Y
---
CPEYYWPDKDKVKCLPGIEEFLSFSEAMGIVLVILSLLGVILAFTLTIIFHHFRSTAIVKANNSEISFLLLLSLKLCFLCSLLFIGQPSLWKCRLRQAAFGISFVLCLSCLLVKTIVVLFAFRANLKARCLAKLFGPSRQRTLILLTTA
PQVCLCTAWLLAAPSFPFKNPTYQTGKIVVECKEPWPPGFYLVLGI--LLAFIYLLLAFVRRKLPDTFNEAKLITFNMMIKWDVWISLKPAYVRSTGKLTVAVEVFAILASSFGLLLCIFLPKCFILLLHPERNVKRGMTGKY--
>marker.fugu_14_1_F
---
CPEYYWPDKDKVKCLPGIEEFLSFSETMGIILVILTLVGVLLTFSLTIIFLHFRSTPIVKANNSEISFLLLLSLKLCFLCSLLFIGQPSLWKCRLRQAAFGISFVLCLSCLLVKTIVVLFAFRTNLAGCCALKLFGPSRQRTLILCTTA
PQVCLCAGWLLAAPSFPVRNPAYQTGKIVLECKEPWPPGFYLVLGYIGLLAFLCLLLAFVGRKLPNTFNEAKLITFSLLIFWAVWISFIPAYVSSPGKFTVAVEVFAILASSFGLLLCIFLPKCFIILLRPERNVKKGMTGKC--
>marker.zebrafish_14_1_F
---
CPEYYWPNKERVSCVAGIEEFLSYHDVMGIILISLSLFGVAVATVVMVIFFIFRSTPIVKANNSEMSFLLLLSLKLCFLCSLVFVGRPSPLTCRARQAAFGISFVLCISCILVKTIVVLLAFRSTVPGSISLKVFGPPQQRVFIFCCTT
GQVILCACWLALAPPYPYKNTSYQDGRIILECKDILPLGFYLVLGYIGLLSCMCFVLAFLGRKLPDTFNEAKLITFSMLIFCAVWISFIPAYNSSPGKYTVAVEIFAILASTFGLLFSIFIPKCYVILLRPDLNTKKGMTGKL--
>Zebrafish.locus514.1
------------------------------
MGIILISLSLFGVAVATVVMVIFFIFRSTPIVKANNSEMSFLLLLSLKLCFLCSLVFVGRPSPLTCRARQAAFGISFVLCISCILVKTIVVLLAFRSTVPGSISLKVFGPPQQRVFIFCCTTGQVILCACWLALAPPYPYKNTSYQDGR
IILECKDILPLGFYLVLGYIGLLSCMCFVLAFLGRKLPDTFNEAKLITFSMLIFCAVWISFIPAYNSSPGKYTVAVEIFAILASTFGLLFSIFIPKCYVILLRPDLNTKKGMTGKLSK
>Zebrafish.locus853.1
------------------------------
MGIILISLSLFGVAVATVVMVIFFIFRSTPIVKANNSEMSFLLLLSLKLCFLCSLVFVGRPSPLTCRARQAAFGISFVLCISCILVKTIVVLLAFRSTVPGSISQKVFGPPQQRVFIFCCTTGQVILCACWLALAPPYPYKNTSYQDGR
IILECKDILPLGFYLVLGYIGLLSCMCFALAFLGRKLPDTFNEAKLITFSMLIFCAVWISFIPAYNSSPGKYTVAVEIFAILASTFGLLFSIFIPKCYVILLRPDLNTKRGITGKLSK
>Japanese_eel.locus257.1
CTKCPQYYWSNRERVACVASVEEFLSFQDSMGIILVALSLLGVSMTLVISVIFYHFRATAIVKANNSELSFLLLQSLMLCFLCALVFVGRPSEWSCWLRQVAFGVSFVLCISCILVKTIVVLVAFRSTLPGSSTVKLFGPPLQRAVILT
CTAVQVILCAVWLVWTPPYPFKNTSYEAGKIILECKVGSTLGFYLVLGYIGLLSCICFILAFLGRKLPDTFNEAKLITFSMLIFFAVWISFIPAYHTSPGKYAVAVEIFAILASSFGLLLCMFLPKCYIILLRPERNTKKSLVNKPTQ
>Spotted_gar.locus146.1
---------------
MCEPRAIEFLSFQDTLGIILVILTFVGVSVTLATVVIFYNCRSTPLVRANNSELSFLLLIALALCFLCSLLFIGKPSVWSCMARQAAFAISFVLCISCILVKTIVVLVAFRATLPGSNAIKRFGPLQQRAIIFLCTVVQAVLCALWLIV
APPFPFKNTSYQGGKIILECKDGSTLAFYLVLGYIGLLSCVCFVLAFLGRKLPGTFNEAKFITFSMFIFFAVWISFIPAYQSSPGKYTVAVEIFAILSSSFGLLICIFAPKCYIILLRPEKNTKKSITCSKIK
>Sterlet.locus292.1
------------------------------
MGIALVTASLLGVSSTVAVIAIFYCFRSTPLVRANNSEISFLLLFSLILCFLCSITFIGEPSKWSCMTRHTTFGISFVLCISCILAKTVVVLAAFRTTLPASNAMKWFGPLQQKGIISICTVIQVLLCTVWLVMAPPFPFRNTSYQGGK
IVVECNAGSVLAFYLVLGYIGFLSCICFVLAFLGRKLPDNFNEAKFITFSMLIFFAVWITFIPAYESSPGKYTVAVEIFAILSSSFGLLFCIFSPKCYIILLKPETNTKRGLMNKPSS
>Reedfish.locus40383.1
------------------------------
MGVTLTVAAILGAFLTLTVMAIFFYFRSTPLVKANNSELSFLLLGSLELCFLCSLTFIGEPSLWSCMTRHTAFGISFVLCISCILAKSIVVLGAFKSTLPASNAMKWFGPLQQRAIIIICTLLQAFLCITWLTLSPPFPFRNTNNQGGK
IILQCNAGSVLAFYLVLGYIGLISCVCFILAFLGRKLPNNFNEAKLITFSMLIFFAVWIAFIPAYESSPGKYTVAVEVFAILSSSFGLLLCIFIPKCYIILLHPEKNTKKGLIGKAVS
>Coelacanth.locus27.1
------------------------------
MAIVLMAVAVLGACITTATAIIFYYHRNTPLVRANNSELSFLLLGSLLLCFLCSLTFLGQPSAWSCLTRHTAFGISFVLCISCLLTKTVVVLMAFKATLPGSSVIKWFGPLQQRAIIFLCTFIQVLICAIWVAVSPPKPLKNTMHQSAK
IILQCDLGSDWAFYLMLGYIGLLSCICFVLAFLGRRLPNNFNEAKFITFSMLIFLIVWIAFIPAYVSSPGKYTVASEIFAILSSSFGMLLCIFAPKCYIILMKPEKNTKKGLMGKN--
>Elephant_shark.locus194.1
CTKCSTDDRSNPERNAYIPKEVEYLSFQEATGIALVAIALFGACLTVTVSVIFFHYKTTPVVKANNSELSFLLLTSLVLCFLCSIAFIGQPTDWGCKLRYTAFAVIFELCICCVLAKTVVVLMAFRATLPNSNVMQWFSPAQQRAAVFI
AMCVQVLICVAWLMVSPPFPFKNTTHQNAKIILQCHVGSMVAFCTVLGYIGFLSCICFVLAFLARKLPDTFNEAKFITFSMLIFFAVWIVFIPVYLSSPGKYTVAVQVFAILSSSFALLLCIFAPKCYIILLQPERNTRQGLMGKH--
>Small-eyed_rabbitfish.locus64.1
CTKCSVDDRSNPERNACVPKEIEYLSFQEAIGIALLAIALFGACVTVAVTVIFFHYKTTPVVKANNSELSFLLLTSLVLCFLCSIAFISQPTDWACKLRYTAFAVIFELCICCVLTKTVVVLMAFRATLPHSNVMQWFGPAQQRAAVFI
ATCVQVLICAAWLTVSPPFPFKNTTHQNTRIILQCHTGSTVAFCTVLAYIGFLSCICFVLAFLARKLPDTFNEAKFITFSMLIFFAVWIVFIPVYLSSPGKYTVAVQVFAILSSSFALLLCIFAPKCYIILLQPERNTRQGLMGKH--
>Brownbanded_bambooshark.locus2001.1
CTKCSSDDWSNAERIMCIPREIEYLSFEDSAGIILTVIALLGACLTVAVMAIFFRHRNTAIVKANNSELSFLLLSSLVMCFLCSLTFIGRPTSWSCVLSHTAFGVIFVLCISCILAKTVVVLMAFRATLPNSNVMKWFGATQQRTVIFL
CTCVQVGICVTWLVRFPPFPVKNTAYQNDKIILECNVGSVAAFYSVLAYISILSCICFVLAFLARKLPDKFNEAKFVTFSMLIFFAVWTAFIPAYVSSPGKYTVAVEIFAILSSSFGLLLCIFTPKCFIILLRPEKNTKKTLMNK---
>Cloudy_catshark.locus15.1
--------------------------------
MTLAIIALLGACLTVAVLAIFFRHRDTPIVKANNSELSFLLLSSLVLCFLCSLTFIGRPTVWSCMLRHTAFGVIFVLCISCILAKTVVVLMAFRATLPSSNVMKWFGPAQQRAVIFLCTCVQVGICAAWLVRSPPFPIKNTTYQSAKTI
LECNVGSDVAFYTVLGYISFLSCICFVLAFLARKLPDKFNEAKFITFSMLIFFAVWMAFIPAYVSSPGKYTVAVEIFAILSSSFGLLLCIFAPKCFIILLRPENNTKKMLMSKQ--
>Caecillan.locus34699.1
CNKCPSDHWSGHGFTKCIPRQLEYFSLEDSMGIVLTVIALIGVVCTTAVLVIFFHWKHTPVVKGNNAELSFLLLGSLALCFLCSLTFIGQPSAWSCMVRSTAFAISFVLCISCILAKTTVVVAAFKVTTPGSDVMKWFGPLQQKALVFL
CTFTQVVICVTWLIISPPFPSQDTSYHNTKILSHCNVGSALAFYLTLGYIGFLCGVCFLLAFLARRLPDAYNEAKYITFGMIIFFAVWMAFIPTYLSSPEKYTVAVEIFAIQFSSFGLLICIFTYKCYVLLLRPESKHTKYYKEQKVL
>Zebrafish.locus843.1
CLTCSKETWPNQAQDRCIPKTLEFLSFQEPLGIILWVFSALGACAALAVLCVFVMYRKTPVVRGNNIELSFLLLLFLCACFLIGLTFLGKPTDWLCQIRYPAFGISFTLCISCILAKTVVVLMAFRATIPGNNVMKWFGPVKQRSSVIL
CTCVQALICIIWLTTKPPLASYNSKFMSETIIVECSVGSEIGFWCVLGYVGLLACLCFLMAFLARKLPDNFNEAKFITFSMLIFFAVWITFIPVYVSSSGKYMVAVHVFAILASAFGLLMCIFAPKCYVVLLKPERNDKKHMMKK---
>Zebrafish.locus504.1
CLTCSKETWPNQAQDRCIPKTLEFLSFQEPLGIILWVFSAFGACAALAVLCVFVMYRKTPVVRGNNIELSFLLLLFLCACFLIGLTFLGKPTDWLCQIRYPAFGISFTLCISCILAKTVVVLMAFRATIPGNNVMKWFGPVKQRSSVIL
CTCVQALICIIWLTTKPPLASYNSKFMSETIIVECSVGSEIGFWCVLGYVGLLACLCFLMAFLARKLPDNFNEAKFITFSMLIFFAVWVTFIPVYVSSSGKYMVAVHVFAILASAFGLLMCIFAPKCYVVLLKPERNDKKHMMKK---
>Zebrafish.locus503.1
CLTCSEDTWPNQSQDQCIPKTLEFLSFQEPLGIILWVFSAFGACAALAVLCVFVMYRKTPVVRGNNIELSFLLLLFLCACFLIGLTFLGKPTDWLCQIRYPAFGISFTLCISCILAKTVVVLMAFRATIPGNNVMKWFGPVKQRSSVIL
CTCVQALICIIWLTTKPPLASYNSKFMSETIIVECSVGSEIGFWCVLGYVGLLACLCFLMAFLARKLPDNFNEAKFITFSMLIFFAVWITFIPVYLSSSGKYMVAVHVFAILASAFGLLLCIFFPKCYIVLLKPEKNDKKHIMKK---
>Zebrafish.locus842.1
CLTCSKDTWPNQSQDQCIPKTLEFLSFQEPLGIILWVFSALGACAALAVLCVFVMYRKTPVVRGNNIELSFLLLLFLCACFLIGLTFLGKPTDWLCQIRYPAFGISFTLCISCILAKTVVVLMAFRATIPGNNVMKWFGPVKQRSSVIL
CTCVQALICIIWLTTKPPLASYNSKFMSETIIVECSVGSEIGFWCVLGYVGLLACLCFLMAFLARKLPDNFNEAKFITFSMLIFFAVWITFIPVYLSSSGKYMVAVHVFAILASAFGLLLCIFFPKCYIVLLKPEKNDKKHIMKK---
>Zebrafish.locus840.1
CLTCSKDTWPNQSQDQCIPKTLEFLSFQEPLGIILWVFSALGACAALAVLCVFVMYRTTPVIRGNNIELSFLLLLFLCACFLIGLTFLGKPTDWLCQIRYPAFGISFTLCISCILAKTVVVLMAFRATIPGNNVMKWFGPVKQRSSVIL
CTCVQALICIIWLTTKPPLASYNSKFLSATIIVECSVGSEVGFWCVLGYIGFLSSLCFFLAFLARKLPDNFNEAKFITFSMLIFFAVWITFIPVYVSSSGKYMVAVHVFAILASAFGLLMCIFAPKCYVVLLKPERNDKKSMMKK---
>Zebrafish.locus501.1
CLTCSKDTWPNQSQDQCIPKTLEFLSFQEPLGIILWVFSALGACAALAVLCVFVMYRTTPVIRGNNIELSFLLLLFLCACFLIGLTFLGKPTDWLCQIRYPAFGISFTLCISCILAKTVVVLMAFRATIPGNNVMKWFGPVKQRSSVIL
CTCVQALICIIWLTTKPPLASYNSKFLSATIIVECSVGSEVGFWCVLGYIGFLSSLCFFLAFLARKLPDNFNEAKFITFSMLIFFAVWITFIPVYVSSSGKYMVAVHVFAILASAFGLLMCIFAPKCYVVLLKPERNDKKSMMKK---
>Japanese_eel.locus138.1
------------------------------
MGFVLCVVSAVGACATTVVSGVLVAHRTTPVVRGNNMELSFLLLLFLGICFLIGLTFIGEPTDWFCMIRYTAFGISFALCISCILAKTVVVLMAFRATLPGSNVMRLFGPTQQRASVLLCTSVQVVICLVWLTTKPPYAAHNTKYQTAK
IILECAVGSEVGFWCVLGYIGLLACLCFVMAFLARKLPDNFNEAKFITFSMLIFFAVWITFIPVYVSTSGKYTVAVHIFAILASAFGLLICIFAPKCYIVLLKPEKNTRRQMMMMKK-
>Japanese_eel.locus140.1
------------------------------
MGFILCVVSAVGACATTVVSGVLVAHRKTPVVRGNNMELSFLVLLFLGICFLIGLTFIGEPTDWFCMIRYTAFGISFALCISCILAKTVVVLMAFRATLPGRNVMRLFGPTQQRASVLLCTSVQVVICLVWLTTKPPYAAHNTKYQTAK
IILECAVGSEVGFWCVLGYIGLLACLCFVMAFLARKLPDNFNEAKFITFSMLIFFAVWITFIPVYVSTSGKYTVAVHIFAILASAFGLLICIFAPKCYIVLLKPEKNTRRQMMMMKK-
>marker.cichlid_13-1aa
---
CSADTWSNTARNLCIPRTTEYLSYYEFMGILLCVASVFGACLSLFIITIFISFKDTPLVRANNMELSFLLLAFLAICFLVGLLFIGVPSDWLCRIRYPAFGISFALCISCLLAKTAVVLMAFRSTLPRSNVMKWFGSNQQRASVLLGTA
VQVIICFIWLLTSPPHANNNTDYHGATIIKECVPGSEVGFWSVLGYIGILACGCLLMAFLARNLPDNFNEAKFIAFSMLIFFAVWITFIPVYVSTAGKYTVAVHIFAILASAFALLFCIFVPKCYIILLRPEKNCKKKIMQR---
>Spotted_gar.locus140.1
------------------------------
MGMVLTVVSTLGACVTIAVSGVFIFFRNTPIVRANNMELSFLLLLFLILCFLIALTFIGEPTDWSCMVRYTAFGIGFALCISCILAKTVVVLVAFRATLPGSNVMKWFGPTQQRASVFVCTSVQILICIVWLITSPPHAAHNTVYRSAK
```

```
IILECAVGSVLGFWGVLGYIGLLACLCFFIAFLARKLPDNFNEAKFITFSMLIFFAVWITFVPAYVSTPGKYTVAVQVFAILASAFGLLSCIFAPKCYIILLRPEKNSKKYLMDKSNK
>Coelacanth.locus26.1
------------------------------
MGITLTVIAVFGASVTTAVSVIFFNFKNTPIVRANNMELSFLLLISLILCFLCALAFIGEPTTWSCMIRQTVFGISFVLCISCILGKTVVVLMAFKNRLPSSNTMKWFGPPQQRASVFLTTVIQVIICIIWLVTNPPFPSQNSVHQSAK
IILECNMGSALAFCLVLGYIGLLAGTCFFLAFLARKLPDNFNEAKFITFSMLIFFTVWITFIPAYISSPGKYTVAVEIFAILASTLGLLLCIFAPKCYVILLKPEKNTKKHLMDKVPS
>Cloudy_catshark.locus14.1
------------------------------
MGITLAVISLFGACITGVVAVVFLYFLNTPIVRANNSELSFLLLISLILCFLCSIAFIGQPTPWSCMVRHTVFGVSFVLCISCILSKTLVVLMAFKATLPASNVMKWFGPKQQRSIVFLCTLIQIIICVIWLVTSPPLPSKNTKYQSAK
IILECDVGSTVAFWCVLVYIGLLACMCLILAFLARALPDKFNEAKFITFSMLIFFAVWLTFIPTYVSNPGKYTVAVEIFAILASSFGLLICIFAPKCYIILFKSEENSKKHLMGKHAQ
>Cloudy_catshark.locus12.1
------------------------------
MGITLAAISLIGACITGVVAVVFLYFLNTPIVRANNSELSFLLLISLILCFLCSIAFIGQPTPWSCMVRHTVFGVSFVLCISCILSKTLVVLMAFKATLPASNVMKWFGPKQQRSIVFLCTLIQIIICVIWLVTSPPLPSKNTKYQSAK
IILECDVGSTIAFWCVLAYIGLLACMCLILAFLARALPDTFNEAKFITFSMLIFFAVWLTFIPTYVSNPGKYTVAVEIFAILASSFGLLVCIFAPKCYIILFKSEENSKKHLMGKNAP
>Cloudy_catshark.locus8.1
CLRCSIDQWSNNEKNECIPRENEYLSFQDGIGITLAAISLFGACITVVVAIVFLCFVNTPIVRANNSELSFLLLISLILCFLCSIAFIGQPTPWSCMVRHTVFGVSFVLCISCILSKTLVVLMAFKATLPASNVMKWFGPKQQRSIVFL
CTLIQIIICVIWLVTSPPLPSKNTKYQSAKIILECDVGSTVAFCCVFGYIGLLACMCFVLAFLARTLPDKFNEAKFITFSMLIFFAVWVTFIPTYVSTPGKYTVAVEIFAILTSSFGLLCCIFAPKCYIILLKPEENTKKQLTIKTDR
>Cloudy_catshark.locus10.1
CLICPLQQWSNERRDQCIPKDVEFLSFYDTMGITLTAVSLFGACITGVVATVFLYFLNTPIVRANNSELSFLLLISLILCFLCSIAFIGQPTTWSCMVRHTGFGVSFVLCISCILSKTLVVLMAFKATLPASNAMKWFGPKQQRSIVFL
CTLIQIIICVIWLVTSPPLPSKNTKYQSAKIILECDVGSTVAFCCVFGYIGLLAGICLLLAFLARALPDKFNEAKFITFSMLIFFAVWVTFIPAYVSTPGKYTVAVEIFAILASSFGLLSCIFAPKCFIILLRPEENSKKRLMSK---
>Whale_shark.locus9.1
------------------------------
MGITLTAISLLGACITGAVAAVFLYFINTPIVKANNSELSFLLLISLILCFLCSIAFIGQPSPWSCMVQHTVFGVSFALSLSCILGKTLVVLMAFKATLPGSDVMKWFGPKQQRSIVFICALIQVIICVIWLLTSPPLPAKNTKYQNEK
IILECGVGSTVAYCSVFGYIGLLGCICLILALLARKLPDRFNEAKLITFSMLIFFAVWVTFIPAYVSNPGKYTVAVEVFAILASSFGLLSCIFSSKCYIILLKPEENTKKHAIAKNVQ
>Brownbanded_bambooshark.locus2000.1
------------------------------
MGLTLTVISLLGALVSGAVAAVFLHFINTPIVRANNSELSFLLLISLILCFLCSIAFIGQPSPWSYMMRHTVFGVSFALCISCILSKTLVVLMAFKSTLSGSNVMKWFGPKQQRSIVFICALIQVIICVIWLVTSPPFPAKNIQNQNAR
IILECDVGSTTAFGCVFGYIGLLGCICLIQALLARTLPDRFNEAKLITLSMLIFFAVWVTFNPVYVSNPGKYTVAVEIFAILASSFGLLFCMFSSNCYIILLKPEKNTKKHTIAKNEQ
>Brownbanded_bambooshark.locus1998.1
-MKCSLDYWSNDNRNECIPKENEYVSFQDGIGIALTVTSLLGACITVAVAAVFLHFINTPIVKANNLELSFLLLMSLILCFLCSIAFIGQPSPWSCMVRHTVFGISFALCISCILSKTLVVLMAFKSTLPGNDVMKWFGPKQQRSIVFL
STLIQLIICVLWLVISPPVPAKNTRYQSAKVILECSLGSSIAFCCVFGYIGLLACFCLMLAFLARALPDKFNEAKFITFSMLTFFAVWLTFIPAYVSTHGKYTVAVEVFAILTSSFGLLICIFTPKCYIILLKPEENTRKHLTAK---
>Reedfish.locus40409.1
------------------------------
MGISLLAISLLGSCFTIAIISVFAYYRNTPIVRANNSELSFLILFSLVLCFLCSVAFIGQPTVWSCMLRHTIFGIAFVLCISCILGKTIVVLIAFKATLPGKNIMKWFGAAQQRAIIFLCTMIQVVICTVWLVVSPPSPVKTMNYQSSK
IILECHVGSLTAFILVLGYIGFLSSLCFVLAFLARKLPDNFNEAKFITFSMLIFFVVWTAFIPAYVSTPGKYTVATEIFAILSSSFGLLVCLFFPKCYIILIKPENNSKKHLLGKRAS
>Sterlet.locus288.1
------------------------------
MGITLMSVSLLGSCFTFAIAAVFAYYRNTPIVKANNSELSFLILVSLVLCFLCSLAFIGQPTTWSCMLRHTVFGIAFVLCISCLLGKTIVVLFAFKANHPGSNMMKWFGPTQQRAVIFLCTLIQVLICTVWLIISPPSPVKTMNYQSSK
IILGCDVGSVMAFSLVLGYIGLLSCLCFLLAFLARKLPDNFNEAKFITFSMLIFFAVWITFIPAYVSTPGKYTVAVEIFAILSSSFGLLVCLFFPKCYIILIKPEKNTKKHLMGKTTS
>Elephant_shark.locus206.1
CIKCDLDYWSNSQNNQCILKEIEFLSFGDAIGISLATIALLGTGLTITIAAVFTHYRTTPVVRANNSELSFLILFSLALCFMCSLIFIGQPSVWSCSLCNTLSGVSFALCVSCILAKTIVVLMAFKATGPNTNTMKWFGPMQQRAFILL
CTCIQISICVSWLITSPPLPVKNTTYQSGKIILECAIGSVKAFAVVLGYVFVLSSLCLVLAYFARKLPDNFNEAKFITFSMLIFFAVWITFIPAYVSTPGKYTVAVEIFAILSSSFGLLLCIFAPKCYIILFKPERNTKKHLMGTMIT
>Cloudy_catshark.locus7.1
CIKCHLDYWSNSLKDRCLPKDIEFLSFEDPLGISLVTIALCGASLTIGAAAVFTNYRNTPIVRANNSELSFLILLSLVLCFLCSVTFIGKPTVWSCMLRHTLFGISFVLCISCILGKTIVVLMAFRARPPNSDRMKWFGPVQQRVIILL
CTSLQIIICLLWLTTSPPLPVKNTKYQNAKIILECDVGSIKAFAFVLGYIGILSSSCFLLAFFARKLPDNFNEAKFITFSMVIFFAVWVTFIPAYVSTSGKYTVAVEIFAILASSFGILSCIFVPKCYIILFKPEKNNRKHLMCRRAD
>Elephant_shark.locus28.1
CTQCPEDSWSNQKKDECLPKEIEFLSFEEPLGIILTVFSLLGAVITVVIIVIFIKFLNTPIVRANNHELSFLLLFSLFFCFLCCLTFIGEPSVEFCVLQQTGFGISFALCISCILVKTSVVVLAFKMTAPNQNMSKYFRPIHHRIVVTA
ITLVQIGISLGFLISSPLVVQRNSQAVIGKIILECNKEPELALIGILGYIGTLALLCFILAFLARKLPNNFNEAKFISFSLLVFFVVWLSFIPAHASSKGKYLVAVEIFAILSSTFGLLAFIFFPKCYVVLMRPDLNTKQGMMGRAPP
>Small-eyed_rabbitfish.locus66.1
-MQCPEDYWSNQKKDECLPKEIEFLTFEEPLGKFLTVFSLLGAAITVAIIVVFVKYFNTPIVRANNYELSFILLFSLVLCFLCCLAFIGEPSVEFCVLQQMGFSISFTLCVSCILVKTIVVVLAFKMTAPNQNVSKRFRPVHQRLVVIA
LTLVQIGISLGLLILSPMGVHRNSRAVIGKIILECNMEAKLALVSILGYIGILAVLCFILAFLARKLPNNFNEAKFITFSLLVFFVVWLSFIPAHASSKGKYLVAVEIFAILSSTFGLLALIFFPKCYVVLMRPELNTKHGLMGRAPP
>Sterlet.locus295.1
CIKCPADYWSNSKKDKCVAREIEFLSFGESLGIILTVFAVLGSCLTLSVFGIFIKNMNTPIVRANNLELSFLLLFSLVLCFLCALTFIGEPRVELCVLRRTGFAISFALCISCILVKTIVVLLAFKMTVPNQNVMKHFRPVHQRMTVAA
ATLIQIVICLGFMLSSPPSVEKNREAVIAKIILECREGSELTLFCILGYIGFLAVIDFALAFLARNLPDNFNEAKFITFSLLVFVVVWISFIPGYISTKGKYLVAVEIFAILSSSFGLLACIFFPKCYIILLRPELNTKNSLMGRPKK
>Western_clawed_frog.locus569.1
---------------------
MDFLSFEEPLGITFSVLAVIGISQTLAVVVVFIKHIHTPIVRAHNIEMSFFLLFSLILCFLSSLTFIGKPSSGLCVLRQTVFGVSFALCISCVLVKTLVVILAFTMSKPNQNMMKYFKPSHQRAFVGLATLIQIAICVGLISSANLSIK
KNRDAVITKIVLECNLELELVFLFAIGYIALLALPCFVLAFLARNLPDTFNETKFITFSLLLFVMVWVSFIPGYISTKGKYVTAVEIFAILSSAAGLLGCIFFPKYYIILLKPERNTRKNVTGHSSL
>Mouse.locus33.1
CDPCGEDDWSNAEKSKCVPKLVEFLAYEEALGFTLVILSIFGALVVLAVTAVYVVHRHTPLVKANDRELSFLIQMSLGITVLSSMLFIGKPCNWSCMARQITLALGFCLCLSSILGKTISLFFAYRISISKTRLI-
SMHPIFRKLIVLICVLAEICVCAVYLVLEPPKMFKNIEIQNVKIIFECSEGSIEFLCSIFGFDVLLALLCFLTTFVARQLPDNYYEGKCITFGILVFFIVWISFVPAYLSTKGKFKVAVEIFAILASSYGLLGCVFLPKCFIILLRPKR
NTDETVGGRVPT
>Mouse.locus28.1
CDPCGEDDWSNAEKSKCVPKHVEFLAYEEALGFTLVILSIFGALVVLAVTVVYVIHRHTPLVKANDRELSFFIQMSLVITVLSSLLFIGKPCNWSCMARQITLALGFCLCLSSILGKTISLFFAYRISLSKTRLI-
SMHPIFRKLIVLICVIGEICVCAVYLVLEPPSLFKNIEIQNVKIIFECNEGSIEFLCSIFGFDVLLALLCFVTTFVARQLPDNYYEGKCITFGMLVFFIVWISFVPAYLSTKGKFKVAVEIFAILASSYGLLGCLFLPKCFIILLRPKR
NTDETVGGRVPT
>Mouse.locus26.1
CNPCSEDDWSNAEKNKCVPKHVEFLAYEEALGFTLVILSIFGALVVLAVTVVYVIHRHTPLVKANDRELSFLIQISLVITVLSTMLFIGKPCNWSCMARQVTLALGFCLCLSSILGKTISLFFAYRISISKTQLI-
SMHPSFRKLIVLLCVFGEIGVCASYLVLKPPSMFKNIERQNVKIIFECNEGSVEFLCSIFGFDVLLALLCFVTTFMARQLPDNYYEGKCITFGMLFFFIVWISFVPAYLSTKGKFKVAVEIFAILASSYGLLGCLFLPKCFIILLRPKR
NTDETVGGRVPT
>Mouse.locus41.1
CDPCGENDWSNAEKNKCVPKHVEFLAYEEALGFTLVILSIFGALVVLAVTVVYVIHRHTPLVKANDRELSFLIQVSLGITVLSSMLFIGKPCNWSCKTRQVTLALGFCLCLSSILGKTVSLFFAYRMSISKTRLI-
SMHPIFRKLIVLICVVGEIGVCTAYLVLEPPSLFKNIEPQNVKIIFECNEGSIEFLCSIFGFDVLLALLCFVTTFVARQLPDNYYEGKCITFGMLVFFIVWISFVPAYLGTKGKFNVAVEIFAILASSYGLLGCVFLPKCFIILLRPKR
NTDETVGGRVPT
>Mouse.locus35.1
CDPCGENDWSNAEKNKCVPKHVEFLAYEEALGFTLVILSIFGALVVLAVTVVYVIHRHTPLVKANDRELSFLIQVSLGITVLSSMLFIGKPCNWSCKTRQVTLALGFCLCLSSILGKTVSLFFAYRISISKTRLI-
SMHPIFRKLIVLICVVGEIGVCTTYLVLEPPSLFKNIEPQNVKIIFECNEGSIEFLCSIFGFDVLLALLCFVTTFVARQLPDNYYEGKCITFGMLVFFIVWISFVPAYLGTKGKFNVAVEIFAILASSYGLLGCVFLPKCFIILLRPKR
NTDETVGGRVPT
>Anole_lizard.locus40.1
--------------------------------MTLIALSIFGVFVVLAVTGVYIKHRATPLVRANDRDLSFLIQYSLIVTLLTSILFIGKPEDWSCQSRQVTLALGFSLCLSCVLGKTVMLFLTHLAKNAEK-
ARMSFKPIFQKIIVLLSVLTEVGICTAYLILEPPRMYKNTQFTTLKIIFECDEGSIVYLCVMFGFDVFLAILCFLTAIVSRKLPNNFNEAKFVTFGMLVFFIVWISFVPAYLSTRGKFKVAVEIFAILASSFGLLGCIFVPKCYIILLK
PARNTEEIVGGRATT
>Western_clawed_frog.locus568.1
CISCPSDFWSNSHRNECVLKEVEFLATDEALGITLIFLSVFGASLVIAFTVVYIMYRETALVKANSRSLSFLIQVSLVFTFMTSVLFVGKPEQWSCMARQITLALGFSLCLSSILGKTIVLMLRKKSAKAER-
GHDALKPVYQIIVAVIGLTIQAGICTAYLLIYPPWVYKNMESQNVKIILECNEGSIEFLCSMFGFDVFLAILSFITAFIARKLPDNFNEAKFITFGMLVFFIVWISFVPAYLSTRGKFKVAVEIFAILASSFGLLGCIFVPKSYIILLK
PERNTEEIVGGRAAT
>Zebrafish.locus541.1
CIQCDGDYWSNANHDECVPKTIEFLDFSEPLGITLIAIAAFGALATIVVAIIFLMHLNTPLVNVNDPLLTFSLLLGLVITFLCSIVFLGKPQMWSCMTSQMALAVGFALILSSLMGKSALLMLRARAVKPQKNDVDPIHPRHQRAIMIL
CTLIQVVGCTAWLILMPPHPVKNTGVQNIKIILECDPGNIIFICSIFGYDILLALVTFAFAFVARKLEDHFNEGKSVTFGMLVFFIVWSSFVPAYLSTRGKFMVAVQIFAILASSFGLLACVFIPKCYVLLVKPERNKEEMMIPRPKS
>Spotted_gar.locus169.1
CIQCGEDYWSNTKRDACVPKEIEFLDFSEALGITLISISAFGACITIAVAIIFLVHRNTALVKANDRTLSFFLLLSLVITFLSSIVFIGRPQHWSCMTSQVALALGFAMCLSCIMGKTVVLMLRARAAKQEQANGDPIKPIHQKSIALV
CTLIQVCACTVWLILLPPYPVKNTASQNIKIILECNIGSIIFICCIFAYDILLALLGFICAFIARKMPDNFNEAKFVTFGMLVFFIVWISFVPAYLSTRGKFMVAVQIFAILASSFGLLACIFIPKCYIILIKPERNTEELVRGKSQK
>Reedfish.locus27372.1
CIQCGTDYWSNAKKDACVPKEIEFLAYDEALGITLIAVSVFGACVAFAVVIIYIIYRNTPLVKANDIVLTFFIQFSLIITFLSSITFVGQPQPWSCMTRHVSLALGFAICLSCIMGKTIVLMFSARAAKPEKVNSEPIKPIHQKIIALV
GSLIQVCGCTIWLILYPPYPVKNTASQNIKIILECSEGNIVFICCIFGYDVLLALLGFVFAFIARKLPDNFNEGKFVTFGMLVFFIVWSSFVPAYLSTRGKFMVAVHIFAILASSFGLLACIFVPKCYIIVLKPERNTDELVKSQPKL
>Sterlet.locus180.1
CIQCGEDYWSNTNRDACVAKEIEFLAYDEALGITLIAVSAFGACIAIAVAIIFLVYRNTALVKANDAVLSFFIQFSLVITFLSSIIFVGEPVKWSCMTRQVSLALGFCICLSCIMGKTIVLMFTARAAKPEKTNSEPIRPIHQRIIALT
CILIHACACTVWLVLYPPYPVKNTASQNIKILLECDEGSIIFICCIFGYDVLLALLGFVFAFMARKLPDNFNEAKFVTFGMLVFFIVWISFVPAYLSTRGKFMVAVEIFAILASSFGLLACIFIPKCYIILLKPERNTEALVRSKPKM
>Coelacanth.locus28.1
CIQCPEDYWSNANKDECVKKLVEYLAFEEALGITLIALSVFGACIALSIVVVFIIYRDTALVKANDRELSFLIQLALAIIILSSIVFIGKPARWSCMTRQVLLALAFALCLSCVMGKAVLLMLTAKAAKSENTDKELLKPLYQKLIAFT
GTIIQGIICTIYLVLIPPYPIKNTASQNIKIILECNEGSVIFLCCVFGYDVLLALLCFIFAFIGRKLPDNFNEAKFMTFAMLVFFIVWISFVPAYLSTRGKFMVAVEIFAILASSFGLLGCLFVPKCYIIILKPERNTEELVRGKPTG
>Sterlet.locus136.1
CFKCPPDSWSNENHTSCILKQIEYLSWTEPFGIALTLFAALGIFLTSFVLGVFVKFRNTPIVKATNRELSYLLLFSLICCFSSSLIFIGEPKDWTCRLRQPAFGVSFVFCISCILVKTNRVLLVFEAKIPTSLHRKWWGLNLQFLLVFL
CTFVQIMTCVIWLYNAPPSSYWNSELEDEIIFITCNEGSLMALGFLIGYTCLLAAVCFFFAFKSRKLPENFNEAKFITFSMLIFFIVWISFIPAYVSTYGKFVSAVEIIAILASSFGLLACIFFNKIYIILFKPSRNT----------
>Sterlet.locus167.1
CFKCPPDSWSNENHTSCILKQIEYLSWTEPFGIALTLFAALGIFLTSFVLGVFVKFRNTPIVKATNRELSYLLLFSLICCFSSSLIFIGEPKDWTCRLRQPAFGVSFVFCISCILVKTNRVLLVFEAKIPTSLHRKWWGLNLQFLLVFL
CTFVQIMTCVIWLYNAPPSSYWNSELEDEIIFITCNEGSLMALGFLIGYTCLLAAVCFFFAFKSRKLPENFNEAKFITFSMLIFFIVWISFIPAYVSTYGKFVSAVEIIAILASSFGLLACIFFNKIYIILFKPSRNT----------
>Coelacanth.locus225.1
CAKCPLDSWSNENHTDCIKKQIEFLSWTEPFGIALALFAVLGIFMTSFVLGVFVKFRNTPIVKATNRELSYLLLFSLICCFSSSLIFIGEPKDWTCRLRQPAFGISFVLCISCILVKTNRVLLVFEAKIPTSLHRKWWGLNLQFLLVFL
CTFVQIMTCVIWLYSAPPSSYNNYDLEDEIIFITCHEGSTMALGFLIGYTCLLAAICFFFAFKSRKLPENFNEAKFITFSMLIFFIVWISFIPAYVSTYGKFVSAVEIIAILASSFGLLGCIFFNKIYIILFKPSRNT----------
>Reedfish.locus72574.1
CIKCSEDSWSNENHTSCILKQIEFLSWTEPFGIALTLFAALGIFLTSFVLGVFVKFRNTPIVKATNRELSYLLLFSLICCFSSSLIFIGEPQDWTCQLRQPAFGISFVLCISCILVKTNRVLLVFEAKIPTSLHRKWWGLNLQFLLVFL
CTFVQIMTCGIWLYNSPPKSYRNHEMDDEIIFVTCNEGSLMALGFLIGYTCLLAAICFFFAFKSRKLPENFNEAKFITFSMLIFFIVWISFIPAYVSTHGKFVSAVEVIAILASSFGLLACIFFNKIYIILFKPSRNT----------
>Zebrafish.locus111.1
CVKCPNNSWSNGNHTSCFLKQIEFLSWTEPFGIALALFAVLGVLLTAFVLGVFVQFRDTPIVKASNRELSFLLLFSLICCFSSSLIFIGEPQDWTCRVRQPAFGISFVLCISCILVKTNRVLLVFEAKIPTSLHRKWWGLNLQFLLVFL
FTFVQVMICVVWLYNAPPGSYKNYDI-DEIIFITCNEGSMMALGFLIGYTCLLAAICFFFAFKSRKLPENFTEAKFITFSMLIFFIVWISFIPAYFSTYGKFVSAVEVIAILASSFSLLACIFFNKVYIILLKPSRNTIEEVRCSTAA
>Spotted_gar.locus196.1
CTKCPNNSWSNENHTSCLLKEIEFLSWTEPFGIALTLFAVLGVFLTSFVLGVFVKFRNTPIVKATNRELSYLLLFSLICCFSSSLIFIGEPQDWTCRLRQPAFGISFVLCISCILVKTNRVLLVFEAKIPTSLHRKWWGLNLQFLLVFM
CTFVQIMICVVWLYNAPPESYSNHDM-DEIIFITCNEGSLMALGFLIGYTCLLAAVCFFFAFKSRKLPENFNEAKFITFSMLIFFIVWISFIPAYVSTYGKFVSAVEVIAILASSFGLLACIFFNKVYIILFKPSRNT----------
```

```
>NM_013803.3.mouse.CaSR
CDKCPDDFWSNENHTSCIAKEIEFLAWTEPFGIALTLFAVLGIFLTAFVLGVFIKFRNTPIVKATNRELSYLLLFSLLCCFSSSLFFIGEPQDWTCRLRQPAFGISFVLCISCILVKTNRVLLVFEAKIPTSFHRKWWGLNLQFLLVFL
CTFMQIVICIIWLYTAPPSSYRNHELEDEIIFITCHEGSLMALGSLIGYTCLLAAICFFFAFKSRKLPENFNEAKFITFSMLIFFIVWISFIPAYASTYGKFVSAVEVIAILAASFGLLACIFFNKVYIILFKPSRNTIEEVRSSTAA
>Mouse.locus398.1
CDKCPDDFWSNENHTSCIAKEIEFLAWTEPFGIALTLFAVLGIFLTAFVLGVFIKFRNTPIVKATNRELSYLLLFSLLCCFSSSLFFIGEPQDWTCRLRQPAFGISFVLCISCILVKTNRVLLVFEAKIPTSFHRKWWGLNLQFLLVFL
CTFMQIVICIIWLYTAPPSSYRNHELEDEIIFITCHEGSLMALGSLIGYTCLLAAICFFFAFKSRKLPENFNEAKFITFSMLIFFIVWISFIPAYASTYGKFVSAVEVIAILAASFGLLACIFFNKVYIILFKPSRNT----------
>Anole_lizard.locus95.1
CEKCAENFWSNENHTVCIPKQIEFLSWTEPFGIALTLFAILGIFLTSFVLGVFTRFRNTPIVKATNRELSYLLLFSLLCCFSSSLFFIGEPQDWNCRLRQPAFGISFVLCISCILVKTNRVLLVFEAKIPTSLHRKWWGMNLQFLLVFL
CTFVQIVICVIWLYTAPPSSFRNHELEDEIIFITCNEGSLMALGFLIGYTCLLAAICFFFAFKSRKLPENFNEAKFITFSMLIFFIVWISFIPAYASTYGKFVSAVEVIAILAASFGLLACIFFNKVYIILFKPSRNT----------
>Western_clawed_frog.locus333.1
------------------------------------
MFAILGICLTSFVLGVFIKFRNTPIVKATNRELSYLLLFSLICCFSSSLIFLGEPQDWTCRLRQPAFGISFVLCISCILVKTNRILLVFEAKIPTSFQRKWWGLNLQFLLVFLCTFVQIVTCIIWLYTAPPWSYRNQDLEDEIIFITCN
EGSLMALGFLIGYTCLLAAICFFFAFKARKLPENFNEAKFITFSMLIFFIVWISFIPAYASTYGKFVSAVEVIAILASSFGLLACIFFNKVYIILFKPSRNT----------
>Caecillan.locus19184.1
CEKCPPDSWSNENHTACIAKRIEFLSWTEPFGIALTLFAVLGIFLTSFVLGVFIKFRNTPIVKATNRELSYLLLFSLICCFSSSLIFIGEPQDWTCRLRQPAFGISFVLCISCILVKTNRILLVFEAKIPTSFHRKWWGLNLQFLLVFL
CTFVQIVICVIWLYIAPPWSYRNHELEDETIFITCHEGSLMALGFLIGYTCLLAAVCFFFAFKSRKLPENFNEAKFITFSMLIFFIVWISFIPAYASTYGKFVSAVEVIAILASSFGLLACIFFNKIYIILFRPSRNT----------
>Thorny_skate.locus20.1
CTKCPNDFWSNENHTSCIAKEIEYLSWTEPFGIALTIFAVLGILITSFVLGVFIKFRNTPIVKATNRELSYLLLFSLICCFSSSLIFIGEPRDWTCRLRQPAFGISFVLCISCILVKTNRVLLVFEAKIPTSLHRKWVGLNLQFLLVFL
CILVQIVTCIIWLYTAPPSSYRNHELEDEVIFITCDEGSLMALGFLIGYTCLLAAICFFFAFKSRKLPENFNEAKFITFSMLIFFIVWISFIPAYVST--------------------------------------------------
>Little_skate.locus75.1
CTKCPNDFWSNENHTSCIAKEIEYLSWTEPFGIALTIFAVLGILITSFVLGVFIKFRNTPIVKATNRELSYLLLFSLICCFSSSLIFIGEPRDWTCRLRQPAFGISFVLCISCILVKTNRVLLVFEAKIPTSLHRKWVGLNLQFLLVFL
CILVQIVTCIIWLYTAPPSSYRNHELEDEVIFITCDEGSLMALGFLIGYTCLLAAICFFFAFKSRKLPENFNEAKFITFSMLIFFIVWISFIPAYVSTYGKFVSAVEVIAILASSFGLLGCIYFNKCYIILFKPCRNT----------
>Cloudy_catshark.locus93.1
CTKCPNDFWSNENHTSCIAKEIEYLSWTEPFGIALTIFAILGILMTSFVLGVFIKFRNTPIVKATNRELSYLLLFSLICCFSSSLIFIGEPRDWACRLRQPAFGISFVLCISCILVKTNRVLLVFEAKIPTSLHRKWVGLNLQFLLVFL
CILVQIVTCVIWLYTAPPSSYRNHELEDEVIFITCDEGSLMALGFLIGYTCLLAAICFFFAFKSRKLPENFNEAKFITFSMLIFFIVWISFIPAYVSTYGKFVSAVEVIAILASSFGLLGCIYFNKCYIILFKPSRNT----------
>Great_white_shark.locus44.1
CTKCPNDFWSNENHTSCIAKEIEYLSWTEPFGIALTIFAILGILMTSFVLGVFIKFRNTPIVKATNRELSYLLLFSLICCFSSSLIFIGEPRDWTCRLRQPAFGISFVLCISCILVKTNRVLLVFEAKIPTSLHRKWVGLNLQFLLVFL
CILVQIVTCVIWLYTAPPSSYRNHELEDEIIFITCDEGSLMALGFLIGYTCLLAAICFFFAFKSRKLPENFNEAKFITFSMLIFFIVWISFIPAYVSTYGKFVSAVEVIAILASSFGLLGCIYFNKCYIILFKPSRNT----------
>Brownbanded_bambooshark.locus769.1
CTKCPNDFWSNENHTSCIAKEIEYLSWIEPFGIALTIFAILGILMTSFVLGVFIKFRNTPIVKATNRELSYLLLFSLICCFSSSLIFIGEPKDWTCRLRQPAFGISFVLCISCILVKTNRVLLVFEAKIPTSLHRKWVGLNLQFLLVFL
CILVQIVTCVIWLYTAPPSSYRNHELEDEIIFITCDEGSLMALGFLIGYTCLLAAICFFFAFKSRKLPENFNEAKFITFSMLIFFIVWISFIPAYVSTYGKFVSAVEVIAILASSFGLLGCIYFNKCYIILFKPSRNT----------
>Whale_shark.locus27.1
CTKCPNDFWSNENHTSCIAKEIEYLSWIEPFGIALTIFAILGILMTSFVLGVFIKFRNTPIVKATNRELSYLLLFSLICCFSSSLIFIGEPKDWTCRLRQPAFGISFVLCISCILVKTNRVLLVFEAKIPTSLHRKWVGLNLQFLLVFL
CILVQIVTCVIWLYTAPPSSYRNHELEDEIIFIMCDEGSLMALGFLIGYTCLLAAICFFFAFKSRKLPENFNEAKFITFSMLIFFIVWISFIPAYVSTYGKFVSAVEVIAILASSFGLLGCIYFNKCYIILFKPSRNT----------
>Smalltooth_sawfish.locus11.1
CTKCPNDFWSNENHTSCIAKEIEYLSWTEPFGIALTIFAVLGILITSFVLGVFIKFRNTPIVKATNRELSYLLLFSLICCFSSSLIFIGEPKDWTCRLRQPAFGISFVLCISCILVKTNRVLLVFEAKIPTSLHRKWVGLNLQFLLVFL
CILVQIVTCVIWLYTAPPSSYRNHELEDEIIFITCDEGSLMALGFLIGYTCLLAAICFFFAFKSRKLPENFNEAKFITFSMLIFFIVWISFIPAYVSTYGKFVSAVEVIAILASSFGLLGCIYFNKCYIILFKPSRNT----------
>Elephant_shark.locus171.1
CTKCPNDFWSNENHTYCIEKEIEYLSWTEPFGIALTIFAVLGTVMTFFVLGVFIKFRNTPIVKATNRELSYLLLFSLICCFSSSLIFIGEPKDWTCRLRQPAFGISFVLCISCILVKTNRVLLVFEAKIPTSLHRKWVGLNLQFLLVFL
CTLVQIVTCVIWLYTAPPSSYRNHELEDEIIFITCDEGSLMALGFLIGYTCLLAAICFFFAFKSRKLPENFNEAKFITFSMLIFFIVWISFIPAYVSTYGKFVSAVEVIAILASSFGLLGCIYFNKCYIILFKPSRNT----------
>Small-eyed_rabbitfish.locus70.1
CTKCRNDFWSNENHTYCIAKEIEYLSWTEPFGIALTIFAVLGTVMTFFVLGVFIKFRNTPIVKATNRELSYLLLFSLICCFSSSLIFIGEPKDWTCRLRQPAFGISFVLCISCILVKTNRVLLVFEAKIPTSLHRKWVGLNLQFLLVFL
CTLVQIVTCVIWLYTAPPSSYRNHELEDEIIFITCDEGSLMALGFLIGYTCLLAAICFFFAFKSRKLPENFNEAKFITFSMLIFFIVWISFIPAYVSTYGKFVSAVEVIAILASSFGLLGCIYFNKCYIILFKPSRNT----------
```

Supplementary File 2. The Newick format phylogenetic tree of all 1897 intact V2Rs identified by a broad range of vertebrates. Note that grouping of “fish-type” V2Rs, “tetrapod-type” V2Rs, V2R2, and ancV2R was supported in sufficient bootstrap values.

(((Anole_lizard.locus95.1:0.052386,
(((((((Brownbanded_bambooshark.locus769.1:0.000001,Whale_shark.locus27.1:0.004192)80:0.004238
,(Great_white_shark.locus44.1:0.000001,
((Thorny_skate.locus20.1:0.000001,Little_skate.locus75.1:0.000001)95:0.017053,Cloudy_catshark
.locus93.1:0.004307)44:0.004086)50:0.004225)37:0.004131,Smalltooth_sawfish.locus11.1:0.004224
)74:0.004692,(Small-
eyed_rabbitfish.locus70.1:0.004208,Elephant_shark.locus171.1:0.004207)99:0.016857)79:0.035989
,(((((((Small-
eyed_rabbitfish.locus115.1:0.047697,Elephant_shark.locus31.1:0.049762)100:0.179357,
((((Elephant_shark.locus30.1:0.004517,Elephant_shark.locus29.1:0.010574)100:0.137406,
((Smalltooth_sawfish.locus21.1:0.030479,
(Thorny_skate.locus25.1:0.000001,Little_skate.locus4.1:0.000001)100:0.062483)99:0.036867,
(Brownbanded_bambooshark.locus518.1:0.076931,Cloudy_catshark.locus65.1:0.060638)100:0.049842)
91:0.053196)100:0.117949,
((((((((marker.cichlid_15-2aa:0.076480,marker.medaka_15_1_F:0.072902)68:0.032277,marker.fugu_
15_1_F:0.045736)51:0.031968,
(marker.cichlid_15-1aa:0.128976,marker.cichlid_15-3aa:0.083738)30:0.031439)18:0.012377,marker
.fugu_15_2_F:0.085844)99:0.186587,((Zebrafish.locus815.1:0.000001,
(marker.zebrafish_9_13_FN:0.000001,Zebrafish.locus539.1:0.000001)65:0.003814)100:0.260461,
(Japanese_eel.locus266.1:0.005042,
(Japanese_eel.locus275.1:0.009109,Japanese_eel.locus263.1:0.005640)57:0.004012)100:0.153818)4
1:0.055653)97:0.206979,Spotted_gar.locus167.1:0.133492)22:0.028935,Reedfish.locus27405.1:0.17
7657)45:0.104226,
((((((((Reedfish.locus27515.1:0.114336,Reedfish.locus27517.1:0.246000)22:0.018018,
((Reedfish.locus27530.1:0.202991,Reedfish.locus40330.1:0.194473)5:0.029290,
((Reedfish.locus27535.1:0.074924,Reedfish.locus27531.1:0.106518)98:0.042185,Reedfish.locus275
43.1:0.063267)94:0.064940)5:0.025521)0:0.031453,
((((Reedfish.locus27451.1:0.183307,Reedfish.locus27510.1:0.141945)2:0.007388,Reedfish.locus27
514.1:0.173467)1:0.007602,Reedfish.locus40375.1:0.228801)0:0.010132,
(((Reedfish.locus27521.1:0.225821,Reedfish.locus27524.1:0.131136)24:0.032530,
(Reedfish.locus40382.1:0.280247,Reedfish.locus27417.1:0.125389)26:0.048778)2:0.006175,
(Reedfish.locus40354.1:0.196076,(Reedfish.locus27496.1:0.020300,
(Reedfish.locus27498.1:0.000001,Reedfish.locus27501.1:0.013628)98:0.008453)100:0.078887)43:0.
055680)0:0.023747)0:0.012142)0:0.012340,
(((Reedfish.locus27464.1:0.033940,Reedfish.locus27461.1:0.006531)100:0.202120,
((Reedfish.locus27527.1:0.176188,Reedfish.locus27452.1:0.243567)4:0.030297,
((((Reedfish.locus27408.1:0.042448,Reedfish.locus27410.1:0.012470)100:0.120721,Reedfish.locus
40334.1:0.191392)32:0.068369,(Reedfish.locus40350.1:0.079094,
((Reedfish.locus40344.1:0.003851,Reedfish.locus40339.1:0.000001)100:0.015230,Reedfish.locus40
349.1:0.016398)97:0.054903)100:0.113184)0:0.008255,Reedfish.locus40363.1:0.137315)0:0.015934)
0:0.010437)0:0.005807,
((((Reedfish.locus27442.1:0.013104,Reedfish.locus27444.1:0.004199)100:0.169639,
(Reedfish.locus27507.1:0.126365,Reedfish.locus27509.1:0.085327)69:0.036535)6:0.011431,
((Reedfish.locus27418.1:0.124802,
(Reedfish.locus27420.1:0.025386,Reedfish.locus27427.1:0.019699)100:0.206187)52:0.059728,
((Reedfish.locus27432.1:0.024325,Reedfish.locus27426.1:0.012179)100:0.140089,
((Reedfish.locus27441.1:0.004275,Reedfish.locus27436.1:0.000001)100:0.203260,
(Reedfish.locus27423.1:0.018888,Reedfish.locus27424.1:0.040315)100:0.088471)30:0.025153)6:0.0
08107)20:0.017626)0:0.018948,Reedfish.locus27411.1:0.089580)1:0.021765)0:0.016528)0:0.012161,
Reedfish.locus27518.1:0.137554)0:0.010566,
((((Reedfish.locus27482.1:0.028897,Reedfish.locus27487.1:0.010858)62:0.010061,Reedfish.locus2
7491.1:0.009726)71:0.026316,Reedfish.locus27495.1:0.015877)100:0.180893,
((Reedfish.locus27472.1:0.044363,Reedfish.locus27467.1:0.015048)61:0.013369,
(Reedfish.locus27468.1:0.027635,Reedfish.locus27474.1:0.075127)51:0.009505)96:0.082193)11:0.0
27233)0:0.005612,(Reedfish.locus27457.1:0.152757,
((Reedfish.locus40381.1:0.044350,Reedfish.locus40378.1:0.037565)100:0.283447,Reedfish.locus27
478.1:0.144630)17:0.014657)11:0.031348)0:0.010090,((Reedfish.locus40347.1:0.011457,
(Reedfish.locus40342.1:0.019395,Reedfish.locus40336.1:0.003692)88:0.007895)100:0.116640,Reedf
ish.locus27454.1:0.413866)37:0.057518)2:0.018780,(Sterlet.locus293.1:0.142703,
((((((marker.zebrafish_16_10_F:0.000001,Zebrafish.locus527.1:0.000001)100:0.080177,
((((Zebrafish.locus517.1:0.012232,Zebrafish.locus856.1:0.000001)100:0.098150,
(Zebrafish.locus519.1:0.000001,Zebrafish.locus858.1:0.000001)100:0.105898)27:0.019158,
(((Zebrafish.locus516.1:0.000001,marker.zebrafish_16_1_F:0.000001)62:0.003655,Zebrafish.locus
855.1:0.000001)100:0.115645,
(Zebrafish.locus528.1:0.000001,marker.zebrafish_16_11_F:0.000001)100:0.120040)22:0.014745)10:
0.007191,(((((((Zebrafish.locus535.1:0.000001,marker.zebrafish_16_16_F:0.000001)100:0.109003,
(Zebrafish.locus814.1:0.000001,Zebrafish.locus538.1:0.000001)100:0.027353)28:0.012134,Zebrafi
sh.locus534.1:0.038175)12:0.006490,Zebrafish.locus533.1:0.030574)17:0.007677,
((((Zebrafish.locus521.1:0.003770,
(marker.zebrafish_16_2_F:0.000001,Zebrafish.locus520.1:0.000001)64:0.003702)100:0.057147,Zebr
afish.locus526.1:0.012555)57:0.006900,Zebrafish.locus525.1:0.011666)96:0.030131,Zebrafish.loc
us532.1:0.040596)55:0.008368)6:0.009468,((Zebrafish.locus524.1:0.007117,
(marker.zebrafish_16_6_F:0.000001,Zebrafish.locus522.1:0.000001)100:0.015252)100:0.078698,
(Zebrafish.locus536.1:0.000001,marker.zebrafish_16_17_F:0.000001)100:0.062909)12:0.014161)10:

0.004776,((Zebrafish.locus530.1:0.000001,marker.zebrafish_16_12_F:0.000001)100:0.086233,
(marker.zebrafish_16_13_F:0.000001,Zebrafish.locus531.1:0.000001)100:0.106424)28:0.013669)30:
0.019184)34:0.017014)98:0.117186,(marker.medaka_16_1_F:0.099610,
(((marker.pufferfish_16_3_Y:0.086664,marker.fugu_16_1_F:0.053713)100:0.099974,marker.fugu_16_
4_FP:0.044711)58:0.027770,(marker.cichlid_16-1aa:0.064545,((marker.medaka_16_2_F:0.134779,
((marker.cichlid_16-8aa:0.079121,marker.cichlid_16-10aa:0.065597)50:0.009019,marker.cichlid_1
6-4aa:0.087849)98:0.069645)19:0.007944,marker.cichlid_16-3aa:0.054984)32:0.028619)31:0.025475
)80:0.075938)99:0.140434)97:0.110928,
((Japanese_eel.locus270.1:0.034676,Japanese_eel.locus260.1:0.007634)41:0.004020,
(Japanese_eel.locus262.1:0.013021,
(Japanese_eel.locus274.1:0.032980,Japanese_eel.locus272.1:0.009190)32:0.003782)37:0.005604)10
0:0.124834)97:0.135866,
(((((Spotted_gar.locus147.1:0.067985,Spotted_gar.locus148.1:0.023998)67:0.026539,Spotted_gar.
locus152.1:0.051440)17:0.003795,(Spotted_gar.locus150.1:0.079170,
(Spotted_gar.locus163.1:0.018350,
(Spotted_gar.locus156.1:0.075456,Spotted_gar.locus161.1:0.014462)51:0.011656)16:0.003768)4:0.
003833)24:0.018521,Spotted_gar.locus154.1:0.049995)15:0.013255,Spotted_gar.locus166.1:0.07128
4)35:0.021934)96:0.110640,(((Reedfish.locus40368.1:0.019476,
(Reedfish.locus40372.1:0.077711,Reedfish.locus40371.1:0.004578)61:0.013761)79:0.036277,Reedfi
sh.locus40365.1:0.024449)100:0.228177,Reedfish.locus27456.1:0.124522)9:0.028597)3:0.009251)1:
0.019881)95:0.148811)42:0.046106)6:0.024111,
(((((Brownbanded_bambooshark.locus1554.1:0.051775,
(Whale_shark.locus20.1:0.088747,Whale_shark.locus22.1:0.179815)83:0.024471)88:0.029757,
((Great_white_shark.locus35.1:0.023508,Great_white_shark.locus33.1:0.048051)100:0.037240,
((Smalltooth_sawfish.locus39.1:0.011450,Smalltooth_sawfish.locus24.1:0.024670)61:0.024418,
(Thorny_skate.locus32.1:0.006194,Little_skate.locus16.1:0.002814)100:0.165095)100:0.108712)39
:0.008494)97:0.080756,Elephant_shark.locus140.1:0.248932)99:0.113760,
(((Japanese_eel.locus82.1:0.142104,
((marker.zebrafish_1_1_F:0.000001,Zebrafish.locus475.1:0.000001)100:0.000001,Zebrafish.locus8
30.1:0.004510)99:0.218604)99:0.257398,
(Reedfish.locus27724.1:0.139501,Sterlet.locus277.1:0.135893)74:0.037360)91:0.127641,Coelacant
h.locus334.1:0.256726)63:0.051211)30:0.037684,
((((Sterlet.locus208.1:0.076607,Spotted_gar.locus28.1:0.257011)100:0.214702,
(Whale_shark.locus21.1:0.137545,Great_white_shark.locus34.1:0.065673)100:0.201760)9:0.034347,
((((((Cloudy_catshark.locus54.1:0.218007,Brownbanded_bambooshark.locus1620.1:0.212586)90:0.11
6575,
((Thorny_skate.locus27.1:0.023305,Little_skate.locus52.1:0.000001)100:0.113022,Smalltooth_saw
fish.locus61.1:0.123257)100:0.179006)100:0.381402,
(((Thorny_skate.locus28.1:0.009405,Little_skate.locus7.1:0.009382)100:0.090574,
((Whale_shark.locus2.1:0.011377,Brownbanded_bambooshark.locus1619.1:0.018674)50:0.009593,
(Great_white_shark.locus17.1:0.044491,Cloudy_catshark.locus53.1:0.052751)79:0.022354)76:0.034
482)97:0.129677,(Elephant_shark.locus192.1:0.032237,Small-
eyed_rabbitfish.locus25.1:0.053200)99:0.102315)84:0.077831)92:0.144930,
(Elephant_shark.locus22.1:0.214287,
((((Cloudy_catshark.locus40.1:0.083684,Great_white_shark.locus31.1:0.062917)82:0.037907,
((Brownbanded_bambooshark.locus1560.1:0.056302,
(Whale_shark.locus24.1:0.042002,Whale_shark.locus57.1:0.105253)100:0.077731)81:0.021509,Whale
_shark.locus64.1:0.078490)95:0.035322)100:0.118056,(((((Small-
eyed_rabbitfish.locus257.1:0.066267,(Small-eyed_rabbitfish.locus401.1:0.058446,(((Small-
eyed_rabbitfish.locus651.1:0.010033,Small-eyed_rabbitfish.locus376.1:0.018456)40:0.004501,
((((Small-eyed_rabbitfish.locus302.1:0.035807,Small-
eyed_rabbitfish.locus613.1:0.035687)45:0.005320,((Small-
eyed_rabbitfish.locus138.1:0.115791,Small-
eyed_rabbitfish.locus291.1:0.046380)51:0.005294,Small-
eyed_rabbitfish.locus396.1:0.019353)10:0.001834)1:0.001783,(Small-
eyed_rabbitfish.locus60.1:0.082790,(Small-eyed_rabbitfish.locus209.1:0.070414,(Small-
eyed_rabbitfish.locus226.1:0.043818,Small-
eyed_rabbitfish.locus366.1:0.047194)42:0.011214)15:0.000001)6:0.003757)0:0.000001,((((Small-
eyed_rabbitfish.locus332.1:0.070162,Small-eyed_rabbitfish.locus355.1:0.044159)30:0.004329,
(Small-eyed_rabbitfish.locus500.1:0.030242,Small-
eyed_rabbitfish.locus322.1:0.056354)20:0.000001)10:0.003835,Small-
eyed_rabbitfish.locus69.1:0.045210)5:0.001428,(Small-eyed_rabbitfish.locus179.1:0.044580,
((Small-eyed_rabbitfish.locus48.1:0.042555,Small-
eyed_rabbitfish.locus232.1:0.042795)31:0.003630,Small-
eyed_rabbitfish.locus263.1:0.035277)3:0.000001)1:0.000001)0:0.000001)0:0.000001)0:0.003807,
((Small-eyed_rabbitfish.locus95.1:0.148777,Small-
eyed_rabbitfish.locus128.1:0.049753)32:0.004703,((Small-
eyed_rabbitfish.locus539.1:0.123574,Small-
eyed_rabbitfish.locus519.1:0.042340)38:0.007550,Small-
eyed_rabbitfish.locus330.1:0.034374)8:0.000001)1:0.000001)22:0.004004)28:0.000001)90:0.031863
,Elephant_shark.locus2.1:0.026833)52:0.007689,
(Elephant_shark.locus12.1:0.049000,Elephant_shark.locus14.1:0.042127)58:0.009155)100:0.108835
,(Brownbanded_bambooshark.locus1595.1:0.170547,
(Cloudy_catshark.locus44.1:0.016485,Cloudy_catshark.locus42.1:0.040339)100:0.089046)90:0.0684
79)39:0.014913,(((Cloudy_catshark.locus43.1:0.110401,(Whale_shark.locus61.1:0.066532,
(Whale_shark.locus79.1:0.031790,Whale_shark.locus77.1:0.049628)97:0.035440)97:0.066151)99:0.0
80720,(Smalltooth_sawfish.locus36.1:0.147888,
(Cloudy_catshark.locus41.1:0.094204,Great_white_shark.locus29.1:0.050183)65:0.033580)100:0.14
3515)37:0.018973,((Whale_shark.locus62.1:0.067061,

((Whale_shark.locus58.1:0.027106,Whale_shark.locus56.1:0.028810)67:0.014103,
((Brownbanded_bambooshark.locus1573.1:0.074428,
((Whale_shark.locus68.1:0.100904,Brownbanded_bambooshark.locus1583.1:0.065002)47:0.010011,
((Brownbanded_bambooshark.locus1570.1:0.067894,Brownbanded_bambooshark.locus1562.1:0.045750)3
1:0.009498,
((Brownbanded_bambooshark.locus1585.1:0.044247,Brownbanded_bambooshark.locus1588.1:0.034221)8
6:0.014990,Brownbanded_bambooshark.locus1564.1:0.036728)51:0.009454)31:0.007260)6:0.005590)6:
0.003712,Brownbanded_bambooshark.locus1576.1:0.044757)22:0.019546)30:0.010680)100:0.135868,
(Smalltooth_sawfish.locus37.1:0.138137,Brownbanded_bambooshark.locus1597.1:0.153616)71:0.0600
63)16:0.021167)31:0.019957)100:0.091911)57:0.031090,(((Smalltooth_sawfish.locus33.1:0.022416,
(Smalltooth_sawfish.locus31.1:0.014594,Smalltooth_sawfish.locus34.1:0.043042)86:0.009655)100:
0.120206,(Great_white_shark.locus25.1:0.088618,
(Cloudy_catshark.locus47.1:0.097943,Brownbanded_bambooshark.locus1599.1:0.149026)70:0.023641)
93:0.052980)100:0.164842,(Cloudy_catshark.locus45.1:0.250561,(Small-
eyed_rabbitfish.locus83.1:0.071615,Elephant_shark.locus3.1:0.044414)100:0.161743)74:0.056883)
42:0.030562)49:0.016698)54:0.027143)27:0.021333,((Elephant_shark.locus179.1:0.046398,Small-
eyed_rabbitfish.locus30.1:0.056441)100:0.246688,
(((((Elephant_shark.locus177.1:0.155884,Small-
eyed_rabbitfish.locus32.1:0.085223)100:0.192559,
(Brownbanded_bambooshark.locus1601.1:0.110798,
(Great_white_shark.locus22.1:0.061536,Cloudy_catshark.locus77.1:0.054829)36:0.015420)100:0.19
9207)28:0.029872,(Small-
eyed_rabbitfish.locus29.1:0.046091,Elephant_shark.locus180.1:0.036921)100:0.260922)3:0.024748
,(((Small-eyed_rabbitfish.locus163.1:0.040797,Elephant_shark.locus23.1:0.035551)100:0.197163,
((Smalltooth_sawfish.locus48.1:0.012496,(Smalltooth_sawfish.locus53.1:0.014801,
((Smalltooth_sawfish.locus59.1:0.003658,Smalltooth_sawfish.locus57.1:0.007337)20:0.000001,
((Smalltooth_sawfish.locus49.1:0.003631,Smalltooth_sawfish.locus51.1:0.003650)63:0.003658,
(Smalltooth_sawfish.locus55.1:0.007358,Smalltooth_sawfish.locus46.1:0.007350)68:0.003644)65:0
.003660)45:0.000001)62:0.006148)100:0.125109,
(((Whale_shark.locus36.1:0.048480,Whale_shark.locus103.1:0.057489)61:0.012317,
((Whale_shark.locus106.1:0.043412,
((Whale_shark.locus109.1:0.047245,Whale_shark.locus49.1:0.045673)26:0.006175,
(Brownbanded_bambooshark.locus1603.1:0.071124,Brownbanded_bambooshark.locus1611.1:0.074656)29
:0.014299)9:0.002437)17:0.004293,Brownbanded_bambooshark.locus1614.1:0.049992)26:0.003481)78:
0.043315,
((Great_white_shark.locus18.1:0.007473,Great_white_shark.locus19.1:0.003877)100:0.045931,Clou
dy_catshark.locus91.1:0.046302)62:0.022109)62:0.039322)100:0.132499)8:0.016813,
((Cloudy_catshark.locus76.1:0.063841,Great_white_shark.locus20.1:0.110422)100:0.125427,
(Small-
eyed_rabbitfish.locus31.1:0.041236,Elephant_shark.locus178.1:0.057692)100:0.135930)31:0.04608
7)1:0.005770)5:0.020310,
((((((Elephant_shark.locus174.1:0.018723,Elephant_shark.locus176.1:0.000001)44:0.004109,Eleph
ant_shark.locus182.1:0.000001)27:0.004116,
((Elephant_shark.locus191.1:0.004232,Elephant_shark.locus212.1:0.000001)31:0.000001,
(Elephant_shark.locus202.1:0.000001,Elephant_shark.locus223.1:0.016711)42:0.004133)3:0.000001
)12:0.000001,Elephant_shark.locus203.1:0.004127)100:0.024074,(Small-
eyed_rabbitfish.locus629.1:0.024847,Small-
eyed_rabbitfish.locus218.1:0.012638)97:0.013204)100:0.188871,
(Cloudy_catshark.locus78.1:0.149882,(Smalltooth_sawfish.locus41.1:0.004194,
(Smalltooth_sawfish.locus42.1:0.003782,
(Smalltooth_sawfish.locus44.1:0.011830,Smalltooth_sawfish.locus43.1:0.023242)90:0.007476)88:0
.007154)100:0.130210)100:0.162198)40:0.055663)11:0.018141)81:0.038479)76:0.034944,
((((Sterlet.locus286.1:0.139243,((((marker.medaka_10_1_F:0.193754,
(marker.cichlid_10-1aa:0.145205,marker.fugu_10_1_F:0.148829)23:0.028449)94:0.119289,
(marker.zebrafish_10_1_F:0.000001,Zebrafish.locus489.1:0.000001)100:0.261996)93:0.066428,Japa
nese_eel.locus400.1:0.199742)67:0.135736,Spotted_gar.locus135.1:0.122187)33:0.066372)3:0.0448
29,((Reedfish.locus40391.1:0.294014,Reedfish.locus40393.1:0.260265)47:0.080227,
(Reedfish.locus27713.1:0.299257,Reedfish.locus40385.1:0.201990)11:0.061873)1:0.032067)0:0.013
414,((((((Spotted_gar.locus62.1:0.059523,Spotted_gar.locus69.1:0.117556)62:0.049052,
((((Spotted_gar.locus94.1:0.067199,(((Spotted_gar.locus71.1:0.091352,
(Spotted_gar.locus66.1:0.113776,
(Spotted_gar.locus89.1:0.030966,Spotted_gar.locus90.1:0.032131)97:0.057656)31:0.025643)10:0.0
19528,Spotted_gar.locus96.1:0.103704)14:0.020516,
(Spotted_gar.locus76.1:0.115413,Spotted_gar.locus74.1:0.043764)27:0.011369)9:0.016713)9:0.015
297,Spotted_gar.locus92.1:0.032566)8:0.013718,(Spotted_gar.locus88.1:0.088000,
((Spotted_gar.locus87.1:0.040191,
(Spotted_gar.locus81.1:0.038111,Spotted_gar.locus83.1:0.043726)97:0.038865)66:0.017581,
(Spotted_gar.locus78.1:0.021578,Spotted_gar.locus77.1:0.076471)81:0.050718)38:0.011452)15:0.0
10842)6:0.010467,Spotted_gar.locus73.1:0.067614)10:0.027800)10:0.011661,
(((Spotted_gar.locus52.1:0.079725,Spotted_gar.locus48.1:0.091138)39:0.027337,Spotted_gar.locu
s100.1:0.085481)11:0.022991,((((Spotted_gar.locus110.1:0.092491,
(Spotted_gar.locus113.1:0.066041,Spotted_gar.locus105.1:0.098169)43:0.015347)21:0.011414,Spot
ted_gar.locus128.1:0.145432)8:0.017452,Spotted_gar.locus130.1:0.060309)5:0.015038,
((Spotted_gar.locus55.1:0.080875,Spotted_gar.locus120.1:0.128832)22:0.017771,Spotted_gar.locu
s70.1:0.088110)20:0.027487)1:0.019234)1:0.010769)7:0.026269,
(((((Zebrafish.locus492.1:0.070956,Zebrafish.locus495.1:0.095871)100:0.082078,
(marker.fugu_8_2_F:0.104828,
((marker.cichlid_8-1aa:0.040644,marker.cichlid_8-4aa:0.117350)100:0.092648,marker.medaka_8_2_
FP:0.111914)43:0.028370)95:0.115860)50:0.059290,(Japanese_eel.locus155.1:0.021042,

(Japanese_eel.locus298.1:0.026747,Japanese_eel.locus153.1:0.031012)43:0.007991)90:0.071141)25
:0.051408,(((((Japanese_eel.locus219.1:0.012175,(Japanese_eel.locus108.1:0.014420,
(Japanese_eel.locus106.1:0.007636,
(Japanese_eel.locus99.1:0.018547,Japanese_eel.locus217.1:0.018589)88:0.012442)43:0.004518)20:
0.003757)33:0.010731,(Japanese_eel.locus227.1:0.022893,
((((Japanese_eel.locus236.1:0.034412,Japanese_eel.locus234.1:0.038731)34:0.007724,Japanese_ee
l.locus247.1:0.050600)20:0.005106,
(Japanese_eel.locus238.1:0.060112,Japanese_eel.locus250.1:0.030837)15:0.007044)29:0.003850,
(Japanese_eel.locus256.1:0.107612,Japanese_eel.locus110.1:0.030648)11:0.000001)9:0.004543)27:
0.010899)26:0.000001,(Japanese_eel.locus223.1:0.010327,(((marker.fugu_4_3_FP:0.106445,
(marker.cichlid_4-10aa:0.072090,marker.medaka_4_1_F:0.134451)35:0.007072)98:0.159782,
((((Zebrafish.locus509.1:0.000001,marker.zebrafish_4_1_F:0.000001)64:0.003922,Zebrafish.locus
848.1:0.000001)100:0.049252,
(Zebrafish.locus513.1:0.000001,Zebrafish.locus852.1:0.000001)100:0.039204)50:0.022652,
(Zebrafish.locus511.1:0.000001,Zebrafish.locus850.1:0.003876)100:0.036391)100:0.115780)73:0.0
95150,Japanese_eel.locus101.1:0.043196)14:0.004708)21:0.012433)69:0.098951,
(Japanese_eel.locus149.1:0.080715,((((marker.pufferfish_9_1_F:0.115259,
(marker.medaka_9_1_F:0.173894,marker.cichlid_9-1aa:0.087377)67:0.026548)85:0.040255,
(marker.pufferfish_6_1_F:0.056519,marker.fugu_6_2_F:0.080218)100:0.189751)17:0.053777,
(((marker.zebrafish_9_2_F:0.000001,Zebrafish.locus485.1:0.000001)100:0.085659,
((marker.zebrafish_9_10_F:0.075640,
(((Zebrafish.locus487.1:0.000001,marker.zebrafish_9_4_F:0.000001)100:0.103504,
(marker.zebrafish_9_9_F:0.000001,Zebrafish.locus482.1:0.000001)100:0.077018)32:0.012546,
(marker.zebrafish_9_1_F:0.071603,
(((marker.zebrafish_9_7_F:0.000001,Zebrafish.locus480.1:0.000001)100:0.078708,
(marker.zebrafish_9_8_F:0.000001,Zebrafish.locus481.1:0.000001)100:0.074935)36:0.014212,
(((Zebrafish.locus488.1:0.000001,marker.zebrafish_9_5_F:0.000001)100:0.078227,
(Zebrafish.locus478.1:0.000001,marker.zebrafish_9_11_F:0.000001)100:0.078608)34:0.007507,
(marker.zebrafish_9_3_F:0.000001,Zebrafish.locus486.1:0.000001)100:0.108801)27:0.011206)3:0.0
03815)10:0.004586)19:0.010406)18:0.011664,
(Zebrafish.locus479.1:0.000001,marker.zebrafish_9_12_F:0.000001)100:0.114450)22:0.007711)90:0
.061413,
(((Zebrafish.locus502.1:0.000001,Zebrafish.locus841.1:0.000001)100:0.072396,Zebrafish.locus49
3.1:0.078689)100:0.134431,((Zebrafish.locus498.1:0.004387,
(marker.zebrafish_5_1_F:0.000001,Zebrafish.locus497.1:0.000001)99:0.011344)100:0.111812,Zebra
fish.locus494.1:0.195413)12:0.031015)37:0.053469)13:0.014218)6:0.052757,
(((((((Japanese_eel.locus202.1:0.013761,Japanese_eel.locus191.1:0.023324)100:0.050711,Japanes
e_eel.locus404.1:0.041047)16:0.000001,
(Japanese_eel.locus189.1:0.018065,Japanese_eel.locus199.1:0.013566)33:0.004738)13:0.005705,
(Japanese_eel.locus175.1:0.027927,
(Japanese_eel.locus182.1:0.028270,Japanese_eel.locus186.1:0.040621)86:0.028848)22:0.000001)18
:0.007991,
(Japanese_eel.locus184.1:0.045171,Japanese_eel.locus197.1:0.021793)53:0.009802)53:0.018410,
(Japanese_eel.locus162.1:0.053067,Japanese_eel.locus158.1:0.059151)18:0.010978)32:0.019850,
((Japanese_eel.locus165.1:0.031200,Japanese_eel.locus160.1:0.028226)54:0.010043,Japanese_eel.
locus166.1:0.032815)70:0.009595)37:0.021525)2:0.009240)1:0.026570)2:0.033374,
(((Japanese_eel.locus145.1:0.330024,
((Zebrafish.locus490.1:0.000001,marker.zebrafish_7_1_F:0.000001)100:0.183728,
((marker.medaka_7_1_F:0.217240,marker.cichlid_7-1aa:0.105399)81:0.023807,
(marker.pufferfish_7_1_PF:0.132576,marker.fugu_7_1_F:0.026397)99:0.057411)87:0.065711)51:0.04
1330)4:0.014829,
(Japanese_eel.locus159.1:0.004355,Japanese_eel.locus193.1:0.009232)90:0.032087)4:0.024944,Jap
anese_eel.locus151.1:0.116819)0:0.020032)0:0.017233)0:0.005854,
(marker.fugu_6_1_F:0.334558,Japanese_eel.locus143.1:0.039272)42:0.074160)17:0.081371)8:0.0182
70,
(((((((Reedfish.locus27654.1:0.026274,Reedfish.locus27651.1:0.038255)42:0.010133,Reedfish.loc
us27649.1:0.025218)48:0.012579,Reedfish.locus40514.1:0.048890)19:0.005282,
(((Reedfish.locus27640.1:0.011866,Reedfish.locus27638.1:0.014928)100:0.054501,
(Reedfish.locus27646.1:0.000001,Reedfish.locus27641.1:0.000001)99:0.057223)31:0.015418,Reedfi
sh.locus27657.1:0.059792)6:0.000001)10:0.006783,
(Reedfish.locus27647.1:0.051876,Reedfish.locus27659.1:0.091874)17:0.011268)86:0.081582,Reedfi
sh.locus27637.1:0.110358)97:0.136366,(Reedfish.locus27603.1:0.238443,
(Reedfish.locus27586.1:0.022943,
(Reedfish.locus27584.1:0.000001,Reedfish.locus27585.1:0.008898)93:0.048410)100:0.218152)9:0.0
52844)1:0.035541)0:0.019160,
(((((Reedfish.locus27573.1:0.048185,Reedfish.locus27575.1:0.059554)100:0.172956,
(Reedfish.locus27661.1:0.183919,(Reedfish.locus27671.1:0.185473,
(Reedfish.locus27679.1:0.090699,
((Reedfish.locus27689.1:0.000001,Reedfish.locus27685.1:0.030501)100:0.198298,Reedfish.locus27
677.1:0.078265)44:0.055202)34:0.022982)13:0.019064)8:0.039426)0:0.012709,
((Reedfish.locus40420.1:0.225585,Reedfish.locus40419.1:0.160471)7:0.027297,
(Reedfish.locus27570.1:0.128223,
(((Reedfish.locus40490.1:0.204426,Reedfish.locus40486.1:0.147179)6:0.023206,
((((Reedfish.locus40497.1:0.201640,(Reedfish.locus40506.1:0.013002,
(((Reedfish.locus40509.1:0.033294,Reedfish.locus40503.1:0.009785)75:0.023787,Reedfish.locus40
499.1:0.000001)11:0.004712,Reedfish.locus40501.1:0.000001)19:0.007715)81:0.054105)58:0.048732
,Reedfish.locus40475.1:0.122534)41:0.058090,
(Reedfish.locus40513.1:0.088555,Reedfish.locus40492.1:0.051367)64:0.041969)4:0.008949,
((Reedfish.locus40471.1:0.041887,Reedfish.locus40478.1:0.034461)100:0.054519,

((Reedfish.locus40481.1:0.020567,Reedfish.locus40483.1:0.031472)77:0.008696,Reedfish.locus404
79.1:0.064588)98:0.048191)75:0.032359)14:0.006307)57:0.053980,
(Reedfish.locus40417.1:0.363922,
(Reedfish.locus27707.1:0.020659,Reedfish.locus27699.1:0.019775)100:0.153925)6:0.029880)1:0.02
6866)0:0.014023)0:0.000001)0:0.015278,(Reedfish.locus27576.1:0.124723,
(Reedfish.locus27567.1:0.140866,
(Reedfish.locus40427.1:0.083274,Reedfish.locus40424.1:0.101878)100:0.106093)10:0.028299)2:0.0
26686)0:0.014152,
((((Reedfish.locus27626.1:0.000001,Reedfish.locus27607.1:0.000001)100:0.239015,
(((Reedfish.locus27548.1:0.038807,((Reedfish.locus40451.1:0.032393,
(Reedfish.locus27552.1:0.000001,Reedfish.locus40523.1:0.015521)100:0.080524)44:0.017324,
((((Reedfish.locus40443.1:0.015127,Reedfish.locus40437.1:0.000001)100:0.036213,
(Reedfish.locus40464.1:0.020344,(Reedfish.locus40458.1:0.051048,
((Reedfish.locus40470.1:0.007922,Reedfish.locus40462.1:0.007361)74:0.012346,
(Reedfish.locus40468.1:0.010700,Reedfish.locus40460.1:0.016939)45:0.013642)22:0.012767)49:0.0
09319)65:0.015901)47:0.014337,Reedfish.locus40448.1:0.069984)14:0.000001,Reedfish.locus27546.
1:0.043332)10:0.009374)16:0.006124)37:0.023270,
(Reedfish.locus27551.1:0.051649,Reedfish.locus40436.1:0.069918)21:0.006016)100:0.160416,
(Reedfish.locus40429.1:0.010433,Reedfish.locus40435.1:0.031340)99:0.080018)49:0.031303)16:0.0
56554,((Reedfish.locus27636.1:0.008455,Reedfish.locus27634.1:0.000001)100:0.208072,
(Reedfish.locus27693.1:0.285748,(((Reedfish.locus27629.1:0.159923,
(Reedfish.locus27563.1:0.003885,Reedfish.locus40517.1:0.000001)100:0.281721)12:0.037786,
(Reedfish.locus27632.1:0.188271,
(Reedfish.locus27683.1:0.082634,Reedfish.locus27690.1:0.063681)100:0.096060)35:0.025757)3:0.0
19825,Reedfish.locus40433.1:0.173447)0:0.018569)0:0.022169)0:0.012385)0:0.016971,
(((Sterlet.locus205.1:0.007718,Sterlet.locus206.1:0.000001)100:0.135408,
(((Sterlet.locus204.1:0.042707,Sterlet.locus203.1:0.000001)98:0.074145,
(Sterlet.locus290.1:0.108282,
(((Sterlet.locus280.1:0.048194,Sterlet.locus201.1:0.057537)68:0.009719,Sterlet.locus202.1:0.0
34010)82:0.039211,(((Sterlet.locus197.1:0.014160,((Sterlet.locus196.1:0.007890,
((Sterlet.locus285.1:0.019777,Sterlet.locus284.1:0.007823)84:0.023765,
((Sterlet.locus192.1:0.016625,Sterlet.locus193.1:0.027128)27:0.003910,
(Sterlet.locus199.1:0.015664,Sterlet.locus195.1:0.007830)43:0.004652)24:0.007047)10:0.003966)
11:0.003980,Sterlet.locus194.1:0.015875)29:0.005306)91:0.055116,
(Sterlet.locus283.1:0.028891,Sterlet.locus282.1:0.056618)53:0.027172)5:0.000001,
(Sterlet.locus200.1:0.011238,Sterlet.locus281.1:0.030776)94:0.069581)21:0.020208)18:0.024367)
14:0.006542)32:0.034462,
(Reedfish.locus27596.1:0.112959,Reedfish.locus27591.1:0.094868)27:0.049451)1:0.004254)0:0.010
928,((((Reedfish.locus27559.1:0.171605,
(Reedfish.locus27554.1:0.004007,Reedfish.locus40520.1:0.007670)100:0.167151)40:0.068263,
((Reedfish.locus27608.1:0.011358,Reedfish.locus27597.1:0.004469)100:0.186739,
((Reedfish.locus40453.1:0.016779,Reedfish.locus27613.1:0.006157)100:0.101775,Reedfish.locus27
599.1:0.184843)71:0.063589)21:0.031989)0:0.015145,
((Reedfish.locus27617.1:0.239277,Reedfish.locus27579.1:0.146095)12:0.038283,
(Reedfish.locus27587.1:0.149135,Reedfish.locus27711.1:0.153981)23:0.039991)1:0.020413)0:0.010
213,((Reedfish.locus27697.1:0.026746,Reedfish.locus27703.1:0.019920)100:0.192295,
((Reedfish.locus40515.1:0.000001,Reedfish.locus27565.1:0.000001)100:0.158812,
(Reedfish.locus27670.1:0.149182,
(Reedfish.locus27665.1:0.067539,Reedfish.locus27663.1:0.110602)100:0.071831)38:0.040350)5:0.0
17955)0:0.004364)0:0.021113)0:0.009524)0:0.007192)0:0.008312)0:0.009892)3:0.035121,
(Reedfish.locus27623.1:0.212277,
((Coelacanth.locus327.1:0.125309,Coelacanth.locus330.1:0.160817)46:0.031360,Coelacanth.locus3
29.1:0.182650)89:0.060503)32:0.055310)38:0.072778)24:0.035539)8:0.022839,
((((Cloudy_catshark.locus39.1:0.052361,Great_white_shark.locus32.1:0.114160)72:0.017470,
(Brownbanded_bambooshark.locus1555.1:0.066224,Whale_shark.locus23.1:0.036981)92:0.053029)81:0
.031008,
((Thorny_skate.locus31.1:0.015891,Little_skate.locus14.1:0.000001)100:0.155296,Smalltooth_saw
fish.locus38.1:0.057605)98:0.093704)99:0.155427,(Coelacanth.locus328.1:0.324076,
((((Spotted_gar.locus42.1:0.026199,Spotted_gar.locus43.1:0.018021)99:0.071164,
(((marker.zebrafish_3_1_F:0.000001,Zebrafish.locus500.1:0.000001)100:0.180279,
(marker.cichlid_3-1aa:0.046916,marker.cichlid_3-2aa:0.096430)100:0.214159)90:0.091633,
(Japanese_eel.locus142.1:0.007394,Japanese_eel.locus141.1:0.019525)100:0.099099)50:0.046681)9
2:0.111919,Reedfish.locus27719.1:0.245417)38:0.022368,Sterlet.locus278.1:0.177386)84:0.090661
)48:0.067269)53:0.069970)13:0.035891)11:0.029861)1:0.016029)1:0.013583,
((((Reedfish.locus40409.1:0.126078,(((Zebrafish.locus795.1:0.262370,
(marker.cichlid_12-1aa:0.202640,marker.fugu_12_1_F:0.129261)100:0.169202)38:0.052770,Japanese
_eel.locus384.1:0.208725)79:0.100762,Spotted_gar.locus137.1:0.130795)31:0.056434)27:0.021779,
Sterlet.locus288.1:0.070797)51:0.079128,(Coelacanth.locus333.1:0.164263,
((Coelacanth.locus21.1:0.010996,Coelacanth.locus20.1:0.000001)79:0.006712,Coelacanth.locus325
.1:0.004288)100:0.241065)89:0.087107)33:0.046549,
(Elephant_shark.locus206.1:0.241970,Cloudy_catshark.locus7.1:0.218286)97:0.075671)29:0.056639
)1:0.025959,((((Thorny_skate.locus26.1:0.003695,Little_skate.locus54.1:0.000001)100:0.181328,
((((Cloudy_catshark.locus12.1:0.021324,Cloudy_catshark.locus14.1:0.014059)100:0.048714,Cloudy
_catshark.locus8.1:0.099843)34:0.022437,Cloudy_catshark.locus10.1:0.085599)32:0.039581,
(Brownbanded_bambooshark.locus1998.1:0.176615,
(Brownbanded_bambooshark.locus2000.1:0.123776,Whale_shark.locus9.1:0.072712)100:0.064428)23:0
.031104)35:0.020657)100:0.118723,(Coelacanth.locus26.1:0.213415,
((Spotted_gar.locus140.1:0.121645,
(((((Zebrafish.locus842.1:0.000001,Zebrafish.locus503.1:0.007967)91:0.024966,

(Zebrafish.locus843.1:0.000001,Zebrafish.locus504.1:0.007843)95:0.011551)52:0.010116,
(Zebrafish.locus840.1:0.000001,Zebrafish.locus501.1:0.000001)100:0.039113)99:0.264294,
(Japanese_eel.locus138.1:0.003418,Japanese_eel.locus140.1:0.013691)100:0.097603)74:0.039943,m
arker.cichlid_13-1aa:0.408552)98:0.090796)98:0.094994,
(Reedfish.locus40406.1:0.057708,Reedfish.locus40408.1:0.094111)100:0.086750)97:0.110894)55:0.
047107)35:0.040254,((Small-
eyed_rabbitfish.locus24.1:0.033217,Elephant_shark.locus193.1:0.073148)100:0.260495,
((((Elephant_shark.locus194.1:0.029149,Small-eyed_rabbitfish.locus64.1:0.060738)100:0.256699,
(Cloudy_catshark.locus15.1:0.065270,Brownbanded_bambooshark.locus2001.1:0.102959)99:0.063942)
89:0.078689,(Caecillan.locus34699.1:0.508958,(Coelacanth.locus27.1:0.253056,
(((Spotted_gar.locus146.1:0.163389,
((((marker.cichlid_14-1aa:0.086434,marker.cichlid_14-3aa:0.079436)98:0.092352,
(marker.pufferfish_14_1_Y:0.154542,marker.fugu_14_1_F:0.044872)99:0.115914)96:0.262194,
((Zebrafish.locus514.1:0.000001,marker.zebrafish_14_1_F:0.000001)94:0.000001,Zebrafish.locus8
53.1:0.017846)100:0.188910)83:0.101945,Japanese_eel.locus257.1:0.159303)96:0.147088)95:0.1415
28,Sterlet.locus292.1:0.164993)67:0.061397,Reedfish.locus40383.1:0.204424)78:0.078591)64:0.02
8316)27:0.020857)75:0.100866,(((Reedfish.locus27727.1:0.206924,
((Sterlet.locus276.1:0.026870,Sterlet.locus209.1:0.004769)100:0.097847,
((Japanese_eel.locus396.1:0.012666,(Japanese_eel.locus394.1:0.026205,
(Japanese_eel.locus380.1:0.028083,Japanese_eel.locus360.1:0.051129)49:0.004214)42:0.008375)99
:0.157856,((marker.zebrafish_11_1_F:0.000001,Zebrafish.locus473.1:0.000001)100:0.055626,
(Zebrafish.locus828.1:0.000001,Zebrafish.locus474.1:0.000001)100:0.091477)100:0.169255)98:0.2
31572)39:0.042255)28:0.019173,Coelacanth.locus335.1:0.291881)48:0.087832,
(Coelacanth.locus332.1:0.292042,
(((((((Zebrafish.locus506.1:0.000001,Zebrafish.locus845.1:0.000001)100:0.018144,
(Zebrafish.locus844.1:0.000001,
(Zebrafish.locus505.1:0.000001,marker.zebrafish_2_1_F:0.000001)69:0.004514)100:0.030864)91:0.
028512,(Zebrafish.locus507.1:0.013604,Zebrafish.locus846.1:0.000001)98:0.042076)100:0.109917,
(((Japanese_eel.locus128.1:0.011875,Japanese_eel.locus113.1:0.021473)68:0.006626,Japanese_eel
.locus123.1:0.031717)76:0.013076,Japanese_eel.locus115.1:0.000001)100:0.076707)90:0.025299,
(marker.fugu_2_1_F:0.115093,marker.medaka_2_1_F:0.090567)100:0.122834)100:0.083994,Spotted_ga
r.locus143.1:0.138627)77:0.072426,
(((Sterlet.locus289.1:0.004339,Sterlet.locus287.1:0.009938)97:0.019883,
(Sterlet.locus186.1:0.022619,((((Sterlet.locus183.1:0.003899,(Sterlet.locus188.1:0.000001,
(Sterlet.locus189.1:0.004467,Sterlet.locus184.1:0.000001)53:0.000001)53:0.003890)33:0.000001,
(Sterlet.locus187.1:0.016965,Sterlet.locus185.1:0.008975)61:0.003914)37:0.000001,Sterlet.locu
s182.1:0.000001)33:0.000001,Sterlet.locus191.1:0.004352)38:0.008946)64:0.000001)77:0.021607,
(Reedfish.locus40403.1:0.053707,Reedfish.locus40415.1:0.097422)96:0.097875)98:0.074050)98:0.1
02017)87:0.073206)11:0.008524)11:0.033062)2:0.037931)0:0.019106)89:0.191551,(((Small-
eyed_rabbitfish.locus66.1:0.127989,Elephant_shark.locus28.1:0.083622)100:0.138684,
(((Great_white_shark.locus10.1:0.072089,Cloudy_catshark.locus87.1:0.097713)62:0.029122,Brownb
anded_bambooshark.locus519.1:0.173886)84:0.037786,(Smalltooth_sawfish.locus20.1:0.080829,
(Thorny_skate.locus24.1:0.012462,Little_skate.locus5.1:0.010614)100:0.100437)100:0.127630)98:
0.095780)97:0.117727,(Western_clawed_frog.locus569.1:0.536656,
((Spotted_gar.locus168.1:0.293152,Reedfish.locus27375.1:0.203410)38:0.033944,Sterlet.locus295
.1:0.101154)65:0.054400)50:0.053683)100:0.308179)48:0.045087,
(((Western_clawed_frog.locus867.1:0.009329,(Western_clawed_frog.locus864.1:0.008077,
(Western_clawed_frog.locus866.1:0.008000,Western_clawed_frog.locus865.1:0.000001)99:0.028413)
79:0.019413)100:0.230187,
(((Western_clawed_frog.locus847.1:0.035119,Western_clawed_frog.locus849.1:0.022434)98:0.04088
1,
(((Western_clawed_frog.locus854.1:0.025842,Western_clawed_frog.locus853.1:0.013667)41:0.00816
2,
(Western_clawed_frog.locus856.1:0.014519,Western_clawed_frog.locus855.1:0.005324)92:0.022143)
31:0.008003,Western_clawed_frog.locus852.1:0.050466)19:0.006791)72:0.052399,
((Western_clawed_frog.locus863.1:0.094111,
((Western_clawed_frog.locus858.1:0.050758,Western_clawed_frog.locus860.1:0.060772)48:0.024393
,Western_clawed_frog.locus862.1:0.025038)36:0.025927)38:0.026316,Western_clawed_frog.locus857
.1:0.066389)84:0.042361)100:0.223714)100:0.350850,((((Caecillan.locus36974.1:0.011316,
(Caecillan.locus36976.1:0.000001,Caecillan.locus36981.1:0.000001)100:0.033257)100:0.394562,
((((((Coelacanth.locus349.1:0.096721,Coelacanth.locus377.1:0.122435)100:0.116504,Coelacanth.l
ocus301.1:0.135892)15:0.013608,
(((((((Coelacanth.locus274.1:0.026161,Coelacanth.locus276.1:0.011831)100:0.132409,Coelacanth.
locus132.1:0.080965)27:0.016904,Coelacanth.locus270.1:0.191182)5:0.005340,
(Coelacanth.locus375.1:0.125696,
(Coelacanth.locus136.1:0.177962,Coelacanth.locus209.1:0.124401)45:0.038501)10:0.013555)1:0.01
1102,(Coelacanth.locus347.1:0.148295,
(Coelacanth.locus272.1:0.109330,Coelacanth.locus269.1:0.115449)31:0.000347)14:0.023309)1:0.00
8583,((Coelacanth.locus271.1:0.141145,(Coelacanth.locus268.1:0.119090,
(Coelacanth.locus319.1:0.115939,
((Coelacanth.locus362.1:0.076604,Coelacanth.locus361.1:0.039273)56:0.004333,
(Coelacanth.locus370.1:0.087368,Coelacanth.locus199.1:0.053446)57:0.012874)99:0.081736)64:0.0
23830)30:0.000001)14:0.022743,(Coelacanth.locus318.1:0.186710,
(Coelacanth.locus324.1:0.046811,((Coelacanth.locus346.1:0.073243,
(Coelacanth.locus412.1:0.133184,((Coelacanth.locus321.1:0.079551,
(Coelacanth.locus290.1:0.076784,Coelacanth.locus320.1:0.055336)20:0.003040)13:0.002655,
((Coelacanth.locus294.1:0.100711,Coelacanth.locus295.1:0.053767)25:0.016440,
(Coelacanth.locus389.1:0.045990,Coelacanth.locus292.1:0.104674)36:0.006047)39:0.025611)7:0.00
4247)13:0.004758)44:0.014768,Coelacanth.locus322.1:0.057003)71:0.018136)96:0.066311)6:0.01356

4)5:0.012846)1:0.017161,(((Coelacanth.locus206.1:0.030550,
(Coelacanth.locus208.1:0.053105,Coelacanth.locus388.1:0.049704)68:0.007460)99:0.063246,
((Coelacanth.locus201.1:0.114374,Coelacanth.locus200.1:0.134673)35:0.012508,
(Coelacanth.locus203.1:0.051425,Coelacanth.locus205.1:0.079051)100:0.058534)89:0.042685)28:0.
018902,
((Coelacanth.locus277.1:0.153864,Coelacanth.locus278.1:0.019615)99:0.060230,Coelacanth.locus1
31.1:0.086372)100:0.134629)10:0.010317)6:0.014942)5:0.021867,
((Coelacanth.locus341.1:0.132953,Coelacanth.locus353.1:0.083647)93:0.042261,
((((Coelacanth.locus304.1:0.122421,
(((((Coelacanth.locus373.1:0.124355,Coelacanth.locus153.1:0.141510)80:0.024302,Coelacanth.loc
us404.1:0.078812)85:0.032783,(((Coelacanth.locus365.1:0.156091,
(Coelacanth.locus350.1:0.159449,Coelacanth.locus413.1:0.084729)23:0.006910)14:0.023128,
(Coelacanth.locus142.1:0.106394,
(Coelacanth.locus248.1:0.024317,Coelacanth.locus143.1:0.062535)93:0.060188)51:0.024689)5:0.00
3594,
(Coelacanth.locus311.1:0.114819,Coelacanth.locus150.1:0.106642)48:0.030646)16:0.008301)9:0.00
5090,Coelacanth.locus252.1:0.138417)32:0.026155,
(((Coelacanth.locus251.1:0.142148,Coelacanth.locus250.1:0.119748)55:0.026363,Coelacanth.locus
253.1:0.160005)34:0.012824,Coelacanth.locus309.1:0.076138)26:0.013945)45:0.034088)12:0.004954
,
((Coelacanth.locus305.1:0.008641,Coelacanth.locus308.1:0.025957)90:0.020156,Coelacanth.locus3
06.1:0.051779)100:0.091462)16:0.005691,(((Coelacanth.locus374.1:0.021089,
((Coelacanth.locus351.1:0.047626,Coelacanth.locus262.1:0.052523)51:0.017290,Coelacanth.locus1
41.1:0.051622)75:0.027592)55:0.006671,
(Coelacanth.locus145.1:0.051554,Coelacanth.locus147.1:0.077666)84:0.042269)41:0.017963,Coelac
anth.locus380.1:0.092117)100:0.120959)13:0.018941,(Coelacanth.locus395.1:0.122693,
(Coelacanth.locus352.1:0.062529,Coelacanth.locus261.1:0.029616)100:0.085883)57:0.046424)9:0.0
20064)6:0.006703)2:0.012737,Coelacanth.locus302.1:0.156895)14:0.057459,
(((Reedfish.locus56974.1:0.064295,Reedfish.locus56973.1:0.104349)99:0.100656,
((Sterlet.locus416.1:0.000001,Sterlet.locus438.1:0.003754)100:0.195089,Sterlet.locus410.1:0.1
05896)78:0.053237)41:0.051098,Reedfish.locus56889.1:0.181413)99:0.219473)8:0.028000)5:0.05071
9,
((((Caecillan.locus44365.1:0.033212,Caecillan.locus44514.1:0.069118)98:0.202238,Caecillan.loc
us44364.1:0.175044)90:0.087908,
((((Western_clawed_frog.locus426.1:0.121611,Western_clawed_frog.locus427.1:0.094154)100:0.178
462,((Western_clawed_frog.locus428.1:0.081575,
(((Western_clawed_frog.locus443.1:0.065437,Western_clawed_frog.locus442.1:0.006575)92:0.02259
8,Western_clawed_frog.locus444.1:0.077310)20:0.004742,
(((Western_clawed_frog.locus438.1:0.015015,
((((Western_clawed_frog.locus435.1:0.050433,Western_clawed_frog.locus436.1:0.034303)68:0.0239
18,(Western_clawed_frog.locus433.1:0.013917,(Western_clawed_frog.locus429.1:0.027766,
(Western_clawed_frog.locus431.1:0.013394,Western_clawed_frog.locus432.1:0.016333)79:0.010181)
63:0.014523)54:0.021321)34:0.020708,Western_clawed_frog.locus434.1:0.057993)30:0.019651,Weste
rn_clawed_frog.locus437.1:0.004958)17:0.012105)42:0.027243,Western_clawed_frog.locus441.1:0.0
05902)13:0.005945,Western_clawed_frog.locus439.1:0.011718)50:0.029340)43:0.037667)100:0.14543
0,
((Western_clawed_frog.locus423.1:0.119260,Western_clawed_frog.locus424.1:0.141108)98:0.070588
,
((Western_clawed_frog.locus420.1:0.083608,Western_clawed_frog.locus421.1:0.097079)99:0.082505
,
(Western_clawed_frog.locus422.1:0.186243,Western_clawed_frog.locus425.1:0.181611)75:0.037170)
41:0.021263)100:0.182790)76:0.047676)32:0.016932,
(Western_clawed_frog.locus419.1:0.005467,Western_clawed_frog.locus418.1:0.014048)100:0.271724
)64:0.035120,(Western_clawed_frog.locus498.1:0.316338,
(Western_clawed_frog.locus464.1:0.130609,
((Western_clawed_frog.locus497.1:0.152454,Western_clawed_frog.locus458.1:0.094122)26:0.017905
,
((((Western_clawed_frog.locus493.1:0.084481,Western_clawed_frog.locus449.1:0.073336)6:0.00602
8,Western_clawed_frog.locus448.1:0.152432)1:0.005341,
(Western_clawed_frog.locus467.1:0.070708,Western_clawed_frog.locus468.1:0.121712)47:0.036531)
0:0.004449,(((((Western_clawed_frog.locus491.1:0.110786,
(((Western_clawed_frog.locus485.1:0.047001,Western_clawed_frog.locus487.1:0.003999)66:0.00583
0,Western_clawed_frog.locus486.1:0.014015)82:0.026717,Western_clawed_frog.locus488.1:0.024327
)90:0.024455)65:0.033528,Western_clawed_frog.locus457.1:0.112984)7:0.023879,
(Western_clawed_frog.locus445.1:0.162316,
(Western_clawed_frog.locus471.1:0.040616,Western_clawed_frog.locus489.1:0.110679)9:0.002666)2
:0.004820)0:0.004241,((Western_clawed_frog.locus453.1:0.068759,
(Western_clawed_frog.locus451.1:0.155663,
(Western_clawed_frog.locus472.1:0.024596,Western_clawed_frog.locus473.1:0.102841)71:0.028873)
31:0.011628)13:0.016130,
(((((Western_clawed_frog.locus470.1:0.024218,Western_clawed_frog.locus455.1:0.047431)49:0.008
340,Western_clawed_frog.locus456.1:0.107887)23:0.020295,
(Western_clawed_frog.locus463.1:0.140700,Western_clawed_frog.locus469.1:0.061102)34:0.030117)
4:0.007129,(((Western_clawed_frog.locus494.1:0.029250,
(Western_clawed_frog.locus454.1:0.054192,((Western_clawed_frog.locus481.1:0.048590,
(Western_clawed_frog.locus474.1:0.067112,Western_clawed_frog.locus477.1:0.023918)33:0.015904)
45:0.015191,((Western_clawed_frog.locus482.1:0.024417,
(Western_clawed_frog.locus476.1:0.024993,
(Western_clawed_frog.locus479.1:0.024368,Western_clawed_frog.locus480.1:0.014461)51:0.004070)

56:0.010513)55:0.017773,Western_clawed_frog.locus483.1:0.030353)60:0.017312)25:0.016209)29:0.
008294)10:0.000001,Western_clawed_frog.locus484.1:0.035323)52:0.028117,
(Western_clawed_frog.locus478.1:0.008061,Western_clawed_frog.locus475.1:0.015938)100:0.081133
)4:0.009423)1:0.012719,Western_clawed_frog.locus490.1:0.168923)0:0.000001)0:0.009573)0:0.0110
11,
((((Western_clawed_frog.locus460.1:0.029336,Western_clawed_frog.locus459.1:0.037552)100:0.124
912,Western_clawed_frog.locus492.1:0.131979)23:0.029472,Western_clawed_frog.locus446.1:0.1415
44)1:0.003954,(Western_clawed_frog.locus447.1:0.118692,
(Western_clawed_frog.locus496.1:0.069711,
((Western_clawed_frog.locus465.1:0.111710,Western_clawed_frog.locus466.1:0.066215)24:0.025630
,Western_clawed_frog.locus452.1:0.073212)70:0.042163)10:0.016790)2:0.006161)0:0.012375)0:0.00
8934)2:0.019170)11:0.038416)95:0.160670)67:0.045859)97:0.208548)86:0.228237,
((((Western_clawed_frog.locus782.1:0.000001,
(Western_clawed_frog.locus785.1:0.000001,Western_clawed_frog.locus783.1:0.008114)97:0.025517)
100:0.647646,
(((((((((Caecillan.locus44451.1:0.019607,Caecillan.locus44253.1:0.046494)78:0.028310,Caecill
an.locus44252.1:0.038097)27:0.009745,Caecillan.locus44452.1:0.029379)100:0.103636,
((((Caecillan.locus44440.1:0.003917,Caecillan.locus44442.1:0.007914)95:0.007932,Caecillan.loc
us44444.1:0.008023)100:0.130970,((Caecillan.locus44258.1:0.159162,
(Caecillan.locus44450.1:0.004382,
(Caecillan.locus44250.1:0.024672,Caecillan.locus44251.1:0.007346)66:0.007538)100:0.095003)52:
0.013017,Caecillan.locus44261.1:0.124587)69:0.016877)60:0.021552,
(Caecillan.locus44437.1:0.000001,Caecillan.locus44439.1:0.003767)100:0.123781)72:0.039397)70:
0.047140,(Caecillan.locus44290.1:0.022552,
((Caecillan.locus46850.1:0.029735,Caecillan.locus44273.1:0.030173)27:0.009123,
(Caecillan.locus44297.1:0.040847,Caecillan.locus44300.1:0.010614)80:0.011942)17:0.007142)100:
0.137390)71:0.055356,(((Western_clawed_frog.locus381.1:0.007724,
((((Western_clawed_frog.locus385.1:0.019622,Western_clawed_frog.locus380.1:0.000001)30:0.0076
90,Western_clawed_frog.locus379.1:0.007674)6:0.000001,Western_clawed_frog.locus382.1:0.011623
)11:0.003841,Western_clawed_frog.locus383.1:0.000001)20:0.003846)32:0.003584,
((Western_clawed_frog.locus386.1:0.011642,Western_clawed_frog.locus388.1:0.000001)57:0.007783
,Western_clawed_frog.locus389.1:0.015716)21:0.004167)100:0.349954,
((((Western_clawed_frog.locus592.1:0.059354,(Western_clawed_frog.locus589.1:0.056848,
((Western_clawed_frog.locus586.1:0.076476,
(Western_clawed_frog.locus731.1:0.000001,Western_clawed_frog.locus585.1:0.011309)73:0.053093)
100:0.172766,
(((((((Western_clawed_frog.locus625.1:0.011712,Western_clawed_frog.locus699.1:0.000001)100:0.
070461,
(Western_clawed_frog.locus732.1:0.069525,Western_clawed_frog.locus584.1:0.023767)68:0.033994)
54:0.022620,
((Western_clawed_frog.locus701.1:0.000001,Western_clawed_frog.locus623.1:0.000001)100:0.05108
3,
(Western_clawed_frog.locus624.1:0.014963,Western_clawed_frog.locus700.1:0.014129)97:0.045273)
58:0.026052)87:0.039792,
((((Western_clawed_frog.locus614.1:0.006818,Western_clawed_frog.locus710.1:0.035494)90:0.0519
23,
(Western_clawed_frog.locus621.1:0.034611,Western_clawed_frog.locus703.1:0.007600)66:0.005869)
30:0.003773,
(((Western_clawed_frog.locus628.1:0.034204,Western_clawed_frog.locus629.1:0.018556)48:0.00900
9,(Western_clawed_frog.locus626.1:0.051775,
(((Western_clawed_frog.locus632.1:0.007527,Western_clawed_frog.locus695.1:0.000001)98:0.03067
0,
(Western_clawed_frog.locus627.1:0.000001,Western_clawed_frog.locus698.1:0.000001)100:0.019556
)28:0.009248,
((Western_clawed_frog.locus696.1:0.000001,Western_clawed_frog.locus631.1:0.003852)100:0.01342
0,
(Western_clawed_frog.locus630.1:0.003907,Western_clawed_frog.locus697.1:0.011424)100:0.061593
)48:0.017523)15:0.006028)46:0.023906)53:0.029469,
(Western_clawed_frog.locus702.1:0.000001,Western_clawed_frog.locus622.1:0.000001)100:0.050944
)97:0.044608)63:0.022884,
(Western_clawed_frog.locus704.1:0.000001,Western_clawed_frog.locus620.1:0.003801)100:0.044362
)69:0.025363)68:0.053356,
(((Western_clawed_frog.locus615.1:0.003721,Western_clawed_frog.locus709.1:0.011379)100:0.0629
88,Western_clawed_frog.locus618.1:0.018325)57:0.008144,
((Western_clawed_frog.locus616.1:0.011539,Western_clawed_frog.locus708.1:0.000001)100:0.02438
8,Western_clawed_frog.locus706.1:0.002896)31:0.003752)97:0.066409)37:0.018932,
((((Western_clawed_frog.locus580.1:0.009521,Western_clawed_frog.locus737.1:0.015320)72:0.0193
47,
((((Western_clawed_frog.locus735.1:0.003845,Western_clawed_frog.locus581.1:0.003834)100:0.023
493,Western_clawed_frog.locus583.1:0.009031)90:0.011939,
(Western_clawed_frog.locus741.1:0.000001,Western_clawed_frog.locus578.1:0.004426)98:0.019277)
54:0.007796,(Western_clawed_frog.locus736.1:0.018549,
(Western_clawed_frog.locus619.1:0.000001,Western_clawed_frog.locus705.1:0.000001)100:0.019791
)77:0.003779)57:0.011367)37:0.004393,
(Western_clawed_frog.locus734.1:0.000001,Western_clawed_frog.locus582.1:0.000001)100:0.011658
)24:0.005269,Western_clawed_frog.locus739.1:0.010154)99:0.084176)23:0.009565,
((Western_clawed_frog.locus715.1:0.011108,Western_clawed_frog.locus609.1:0.003839)80:0.017681
,
(((Western_clawed_frog.locus717.1:0.000001,Western_clawed_frog.locus607.1:0.000001)100:0.0326

27,
(((Western_clawed_frog.locus729.1:0.003478,Western_clawed_frog.locus590.1:0.020862)100:0.0348
27,(Western_clawed_frog.locus594.1:0.044658,
((Western_clawed_frog.locus727.1:0.000001,Western_clawed_frog.locus597.1:0.000001)100:0.03448
1,
(Western_clawed_frog.locus598.1:0.000001,Western_clawed_frog.locus726.1:0.007381)100:0.024847
)85:0.031276)78:0.039827)29:0.018794,(((Western_clawed_frog.locus596.1:0.034928,
(Western_clawed_frog.locus603.1:0.030852,Western_clawed_frog.locus721.1:0.025637)51:0.010810)
42:0.004310,(Western_clawed_frog.locus728.1:0.018498,
(Western_clawed_frog.locus722.1:0.028894,Western_clawed_frog.locus602.1:0.010723)96:0.045379)
53:0.017127)66:0.009866,Western_clawed_frog.locus605.1:0.035289)72:0.018382)12:0.011157)40:0.
021339,
((((((Western_clawed_frog.locus682.1:0.035925,Western_clawed_frog.locus644.1:0.035389)91:0.0
40477,((((((Western_clawed_frog.locus646.1:0.015337,
(Western_clawed_frog.locus680.1:0.000001,Western_clawed_frog.locus678.1:0.000001)100:0.015546
)79:0.011666,
((Western_clawed_frog.locus679.1:0.000001,Western_clawed_frog.locus681.1:0.015464)99:0.027960
,(Western_clawed_frog.locus647.1:0.026279,(Western_clawed_frog.locus677.1:0.054661,
(Western_clawed_frog.locus676.1:0.020377,Western_clawed_frog.locus675.1:0.034391)44:0.010014)
10:0.010846)42:0.019510)25:0.007958)26:0.006267,((Western_clawed_frog.locus686.1:0.021619,
(Western_clawed_frog.locus641.1:0.014285,Western_clawed_frog.locus685.1:0.013489)100:0.040231
)51:0.011988,
((((Western_clawed_frog.locus655.1:0.031204,Western_clawed_frog.locus651.1:0.031010)5:0.00381
9,(((Western_clawed_frog.locus659.1:0.034602,
((((((Western_clawed_frog.locus748.1:0.042769,Western_clawed_frog.locus751.1:0.030788)39:0.0
09773,(Western_clawed_frog.locus743.1:0.034956,((((((Western_clawed_frog.locus634.1:0.146486,
(Western_clawed_frog.locus613.1:0.000001,Western_clawed_frog.locus711.1:0.003748)100:0.038953
)39:0.011676,Western_clawed_frog.locus714.1:0.069497)18:0.019924,
(Western_clawed_frog.locus612.1:0.003762,Western_clawed_frog.locus712.1:0.007532)99:0.007349)
100:0.205971,
(Western_clawed_frog.locus713.1:0.030847,Western_clawed_frog.locus611.1:0.011137)83:0.004162)
19:0.065525,Western_clawed_frog.locus749.1:0.003729)6:0.000001,
(Western_clawed_frog.locus658.1:0.026818,(Western_clawed_frog.locus661.1:0.042552,
(Western_clawed_frog.locus750.1:0.016635,Western_clawed_frog.locus660.1:0.012908)93:0.017091)
35:0.004091)33:0.015152)1:0.003946)1:0.002860)3:0.010825,Western_clawed_frog.locus745.1:0.034
367)4:0.006447,Western_clawed_frog.locus747.1:0.054908)10:0.003039,Western_clawed_frog.locus7
52.1:0.011269)4:0.007658,Western_clawed_frog.locus744.1:0.034697)8:0.004441,
(Western_clawed_frog.locus753.1:0.015088,Western_clawed_frog.locus742.1:0.026414)89:0.028031)
2:0.004093)14:0.034755,((Western_clawed_frog.locus654.1:0.023007,
(Western_clawed_frog.locus668.1:0.034733,Western_clawed_frog.locus649.1:0.018175)18:0.005410)
7:0.000001,((Western_clawed_frog.locus664.1:0.018904,
(Western_clawed_frog.locus665.1:0.000001,Western_clawed_frog.locus666.1:0.003746)96:0.015218)
72:0.011450,
(Western_clawed_frog.locus667.1:0.030454,Western_clawed_frog.locus653.1:0.007595)28:0.000001)
78:0.015250)19:0.003790)0:0.003960,Western_clawed_frog.locus640.1:0.015418)0:0.000001)0:0.011
429,Western_clawed_frog.locus687.1:0.023235)0:0.000001,
((((Western_clawed_frog.locus674.1:0.036121,
((Western_clawed_frog.locus669.1:0.049014,Western_clawed_frog.locus663.1:0.032370)12:0.007758
,Western_clawed_frog.locus672.1:0.024379)12:0.008251)6:0.005831,
(Western_clawed_frog.locus746.1:0.027076,Western_clawed_frog.locus662.1:0.042980)49:0.021189)
0:0.007510,Western_clawed_frog.locus671.1:0.031346)1:0.007873,
(Western_clawed_frog.locus688.1:0.037772,Western_clawed_frog.locus639.1:0.017777)63:0.018258)
0:0.003966)0:0.011318)0:0.009488)2:0.008160,
(((Western_clawed_frog.locus650.1:0.043692,Western_clawed_frog.locus670.1:0.044702)12:0.00502
9,Western_clawed_frog.locus738.1:0.043049)5:0.005456,
((Western_clawed_frog.locus656.1:0.007451,Western_clawed_frog.locus657.1:0.018901)95:0.051461
,
((Western_clawed_frog.locus648.1:0.026835,Western_clawed_frog.locus673.1:0.007676)92:0.043528
,Western_clawed_frog.locus645.1:0.074855)11:0.000001)7:0.007071)7:0.016280)13:0.032855,
((Western_clawed_frog.locus637.1:0.003504,Western_clawed_frog.locus690.1:0.016172)100:0.04408
8,
(((Western_clawed_frog.locus689.1:0.027870,Western_clawed_frog.locus638.1:0.022421)95:0.04236
1,
((Western_clawed_frog.locus683.1:0.019930,Western_clawed_frog.locus643.1:0.012066)99:0.038881
,
(Western_clawed_frog.locus642.1:0.011268,Western_clawed_frog.locus684.1:0.003666)94:0.049748)
80:0.032936)64:0.011755,
(Western_clawed_frog.locus691.1:0.003882,Western_clawed_frog.locus636.1:0.007678)100:0.079213
)23:0.014008)12:0.010610)3:0.008206,
(Western_clawed_frog.locus635.1:0.000001,Western_clawed_frog.locus692.1:0.007417)100:0.061783
)2:0.007661)35:0.091415,
(((((Western_clawed_frog.locus768.1:0.007714,Western_clawed_frog.locus773.1:0.007609)54:0.007
622,(((((Western_clawed_frog.locus774.1:0.000001,
((Western_clawed_frog.locus780.1:0.011400,Western_clawed_frog.locus779.1:0.000001)79:0.011371
,Western_clawed_frog.locus778.1:0.003778)44:0.003829)9:0.000001,Western_clawed_frog.locus771.
1:0.007620)7:0.003813,Western_clawed_frog.locus767.1:0.011491)1:0.000001,Western_clawed_frog.
locus776.1:0.003806)1:0.000001,(((Western_clawed_frog.locus757.1:0.023258,
((Western_clawed_frog.locus766.1:0.015435,Western_clawed_frog.locus765.1:0.011395)28:0.004027
,

(((Western_clawed_frog.locus760.1:0.000001,Western_clawed_frog.locus761.1:0.000001)86:0.00766
9,Western_clawed_frog.locus759.1:0.007704)29:0.003830,Western_clawed_frog.locus762.1:0.003837
)36:0.003831)13:0.003883)16:0.007607,
(Western_clawed_frog.locus755.1:0.003801,Western_clawed_frog.locus770.1:0.011571)25:0.003826)
5:0.000001,((Western_clawed_frog.locus754.1:0.003811,
(Western_clawed_frog.locus769.1:0.011486,Western_clawed_frog.locus763.1:0.023094)6:0.003810)2
:0.003814,Western_clawed_frog.locus772.1:0.003807)0:0.000001)6:0.007634)11:0.003891)47:0.0038
39,Western_clawed_frog.locus764.1:0.015224)76:0.024912,Western_clawed_frog.locus758.1:0.01423
1)96:0.086712,Western_clawed_frog.locus756.1:0.011461)83:0.064923)18:0.051435,
(Western_clawed_frog.locus591.1:0.016875,Western_clawed_frog.locus587.1:0.039637)84:0.025628)
5:0.011465,
((Western_clawed_frog.locus720.1:0.004327,Western_clawed_frog.locus604.1:0.026259)65:0.011631
,
((Western_clawed_frog.locus718.1:0.011418,Western_clawed_frog.locus606.1:0.019146)100:0.03424
4,Western_clawed_frog.locus600.1:0.023307)34:0.000001)47:0.016272)7:0.017712,
(Western_clawed_frog.locus608.1:0.000001,Western_clawed_frog.locus716.1:0.003816)100:0.068844
)5:0.029048,
((Western_clawed_frog.locus723.1:0.003638,Western_clawed_frog.locus601.1:0.003651)96:0.010334
,((Western_clawed_frog.locus593.1:0.023588,
(Western_clawed_frog.locus730.1:0.064223,Western_clawed_frog.locus588.1:0.000001)100:0.032817
)89:0.050818,
(Western_clawed_frog.locus725.1:0.004196,Western_clawed_frog.locus599.1:0.004020)95:0.025910)
30:0.023005)26:0.015667)1:0.007285)1:0.021890)3:0.044815)9:0.037490)4:0.029634)13:0.062687)99
:0.461405,
(Western_clawed_frog.locus786.1:0.517271,Western_clawed_frog.locus781.1:0.366714)34:0.053430)
21:0.018068,(((Western_clawed_frog.locus506.1:0.031998,
(Western_clawed_frog.locus507.1:0.024661,Western_clawed_frog.locus508.1:0.046522)95:0.027995)
91:0.018308,Western_clawed_frog.locus505.1:0.000001)100:0.578265,
((((((Western_clawed_frog.locus947.1:0.006275,
(Western_clawed_frog.locus945.1:0.039145,Western_clawed_frog.locus946.1:0.028455)39:0.017015)
39:0.019903,Western_clawed_frog.locus944.1:0.030708)38:0.013649,Western_clawed_frog.locus943.
1:0.053073)100:0.373350,
(Western_clawed_frog.locus941.1:0.088334,Western_clawed_frog.locus940.1:0.075903)100:0.364803
)74:0.071642,((Western_clawed_frog.locus980.1:0.114063,
(Western_clawed_frog.locus905.1:0.060195,Western_clawed_frog.locus993.1:0.025959)98:0.097974)
35:0.015077,
((((Western_clawed_frog.locus991.1:0.023508,Western_clawed_frog.locus988.1:0.031258)95:0.0415
14,(Western_clawed_frog.locus992.1:0.016088,
(Western_clawed_frog.locus906.1:0.037985,Western_clawed_frog.locus990.1:0.018009)84:0.007562)
97:0.027131)36:0.017291,(Western_clawed_frog.locus982.1:0.075998,
(((Western_clawed_frog.locus987.1:0.026260,
(Western_clawed_frog.locus985.1:0.010066,Western_clawed_frog.locus984.1:0.063329)79:0.023658)
32:0.003879,
(Western_clawed_frog.locus989.1:0.050559,Western_clawed_frog.locus986.1:0.015296)51:0.007926)
81:0.031043,Western_clawed_frog.locus983.1:0.060907)76:0.027932)27:0.007352)78:0.056673,Weste
rn_clawed_frog.locus981.1:0.082875)68:0.033679)100:0.382509)60:0.035232,
(Western_clawed_frog.locus995.1:0.029690,
((Western_clawed_frog.locus994.1:0.056005,Western_clawed_frog.locus999.1:0.027745)65:0.027677
,Western_clawed_frog.locus998.1:0.086583)67:0.024193)100:0.286870)94:0.148386)19:0.044894)12:
0.062779,(((((Western_clawed_frog.locus919.1:0.027907,
((Western_clawed_frog.locus917.1:0.003527,Western_clawed_frog.locus918.1:0.031458)84:0.018089
,Western_clawed_frog.locus920.1:0.021632)62:0.010668)87:0.078978,
(Western_clawed_frog.locus924.1:0.024256,
(Western_clawed_frog.locus922.1:0.019738,Western_clawed_frog.locus923.1:0.019709)58:0.022545)
81:0.060538)59:0.037625,
(Western_clawed_frog.locus915.1:0.023346,Western_clawed_frog.locus916.1:0.019802)90:0.056952)
99:0.560627,((Western_clawed_frog.locus913.1:0.099558,
(Western_clawed_frog.locus914.1:0.079123,(Western_clawed_frog.locus909.1:0.016682,
(((Western_clawed_frog.locus912.1:0.035823,Western_clawed_frog.locus911.1:0.014601)59:0.02324
4,Western_clawed_frog.locus910.1:0.027374)35:0.014469,Western_clawed_frog.locus903.1:0.025683
)9:0.004578)37:0.022281)22:0.016256)100:0.376238,((Western_clawed_frog.locus939.1:0.011068,
((Western_clawed_frog.locus937.1:0.072351,Western_clawed_frog.locus936.1:0.044632)40:0.008802
,Western_clawed_frog.locus938.1:0.022520)44:0.009112)100:0.262037,
((Western_clawed_frog.locus935.1:0.104156,
((Western_clawed_frog.locus932.1:0.054359,Western_clawed_frog.locus931.1:0.012251)66:0.019539
,
((Western_clawed_frog.locus930.1:0.020629,Western_clawed_frog.locus926.1:0.032984)50:0.011765
,
(((Western_clawed_frog.locus933.1:0.070538,Western_clawed_frog.locus928.1:0.007751)18:0.00446
2,Western_clawed_frog.locus927.1:0.016099)22:0.012301,Western_clawed_frog.locus929.1:0.020670
)50:0.032608)26:0.005826)33:0.018032)34:0.010214,Western_clawed_frog.locus934.1:0.053848)100:
0.228715)84:0.100827)72:0.052648)26:0.049090,(Caecillan.locus22632.1:0.165003,
(Western_clawed_frog.locus848.1:0.147064,Western_clawed_frog.locus850.1:0.113894)100:0.295616
)18:0.006892)4:0.032417)5:0.021508)5:0.019743)3:0.027122,
((((Caecillan.locus36896.1:0.007917,Caecillan.locus36888.1:0.012105)75:0.017842,Caecillan.loc
us36882.1:0.010247)33:0.003971,(((Caecillan.locus36898.1:0.011591,
(Caecillan.locus36885.1:0.000001,Caecillan.locus36895.1:0.000001)99:0.016100)29:0.003842,
(Caecillan.locus36883.1:0.000001,Caecillan.locus36884.1:0.000001)88:0.004190)39:0.004266,
((Caecillan.locus36891.1:0.003988,Caecillan.locus36886.1:0.003959)90:0.015448,

(Caecillan.locus36893.1:0.000001,Caecillan.locus36892.1:0.000001)98:0.008542)31:0.003942)14:0
.004970)43:0.009367,Caecillan.locus36900.1:0.008841)100:0.233937)25:0.042745,
(Western_clawed_frog.locus377.1:0.009895,(Western_clawed_frog.locus376.1:0.000001,
(Western_clawed_frog.locus375.1:0.000001,
(Western_clawed_frog.locus378.1:0.049900,Western_clawed_frog.locus374.1:0.003779)55:0.003897)
50:0.003828)59:0.005678)100:0.565476)72:0.058594,(Western_clawed_frog.locus192.1:0.454316,
(((((Western_clawed_frog.locus167.1:0.052476,
((((((((Western_clawed_frog.locus163.1:0.025151,Western_clawed_frog.locus162.1:0.036345)85:0
.035324,
((((Western_clawed_frog.locus32.1:0.023173,Western_clawed_frog.locus31.1:0.027854)68:0.014517
,((Western_clawed_frog.locus115.1:0.015174,(Western_clawed_frog.locus117.1:0.000001,
(Western_clawed_frog.locus118.1:0.061886,Western_clawed_frog.locus116.1:0.019069)13:0.011207)
6:0.007502)50:0.034602,
((Western_clawed_frog.locus25.1:0.025156,Western_clawed_frog.locus24.1:0.025108)97:0.024182,
(((Western_clawed_frog.locus28.1:0.037170,Western_clawed_frog.locus29.1:0.004089)44:0.011508,
Western_clawed_frog.locus30.1:0.008398)71:0.021116,
(((Western_clawed_frog.locus282.1:0.059617,Western_clawed_frog.locus22.1:0.007956)41:0.008049
,Western_clawed_frog.locus23.1:0.008374)32:0.018167,Western_clawed_frog.locus21.1:0.035282)18
:0.016733)26:0.006666)41:0.017626)5:0.013689)4:0.004729,
(((Western_clawed_frog.locus27.1:0.023441,Western_clawed_frog.locus26.1:0.029792)94:0.031180,
(Western_clawed_frog.locus59.1:0.033197,
(Western_clawed_frog.locus62.1:0.035446,Western_clawed_frog.locus58.1:0.026665)42:0.010159)41
:0.032787)14:0.014320,Western_clawed_frog.locus34.1:0.083871)7:0.019465)0:0.011193,Western_cl
awed_frog.locus35.1:0.099999)0:0.030449)0:0.005566,
(((((((Western_clawed_frog.locus46.1:0.007684,
(Western_clawed_frog.locus40.1:0.011561,Western_clawed_frog.locus45.1:0.007714)69:0.007640)13
:0.000001,Western_clawed_frog.locus51.1:0.011494)9:0.003823,
((((((Western_clawed_frog.locus41.1:0.011579,Western_clawed_frog.locus42.1:0.003873)43:0.0035
95,Western_clawed_frog.locus44.1:0.019401)43:0.003974,Western_clawed_frog.locus52.1:0.000001)
36:0.003812,Western_clawed_frog.locus47.1:0.007701)51:0.007695,Western_clawed_frog.locus39.1:
0.023259)16:0.003808,Western_clawed_frog.locus43.1:0.000001)9:0.000001)25:0.020126,Western_cl
awed_frog.locus48.1:0.026826)46:0.025043,Western_clawed_frog.locus203.1:0.036807)47:0.028741,
((Western_clawed_frog.locus272.1:0.011549,
((Western_clawed_frog.locus275.1:0.000001,Western_clawed_frog.locus277.1:0.011293)88:0.007524
,
((Western_clawed_frog.locus274.1:0.067432,Western_clawed_frog.locus271.1:0.000001)85:0.026780
,Western_clawed_frog.locus278.1:0.014166)48:0.008681)9:0.003683)7:0.003779,
(Western_clawed_frog.locus269.1:0.015073,Western_clawed_frog.locus270.1:0.005553)51:0.001918)
68:0.035257)44:0.030457,
(((Western_clawed_frog.locus234.1:0.007300,Western_clawed_frog.locus237.1:0.007326)74:0.0036
68,
((Western_clawed_frog.locus232.1:0.000001,Western_clawed_frog.locus230.1:0.022184)49:0.003671
,
(Western_clawed_frog.locus233.1:0.010921,Western_clawed_frog.locus231.1:0.007285)16:0.003618)
23:0.003599)99:0.040120,
((((Western_clawed_frog.locus159.1:0.010654,Western_clawed_frog.locus136.1:0.018814)65:0.0122
46,
(Western_clawed_frog.locus114.1:0.019322,Western_clawed_frog.locus160.1:0.051777)52:0.013874)
27:0.014084,(Western_clawed_frog.locus104.1:0.041128,
(Western_clawed_frog.locus112.1:0.000001,
(Western_clawed_frog.locus113.1:0.010827,Western_clawed_frog.locus111.1:0.003572)46:0.007165)
71:0.006029)88:0.019832)40:0.016364,Western_clawed_frog.locus190.1:0.021361)67:0.024203)18:0.
010746,Western_clawed_frog.locus121.1:0.060575)2:0.010896)0:0.007367)0:0.003747,
((Western_clawed_frog.locus188.1:0.069464,
(((Western_clawed_frog.locus157.1:0.011327,Western_clawed_frog.locus127.1:0.040669)97:0.04809
6,((Western_clawed_frog.locus5.1:0.098075,
((Western_clawed_frog.locus82.1:0.007239,Western_clawed_frog.locus83.1:0.003913)81:0.018858,
(Western_clawed_frog.locus84.1:0.000001,Western_clawed_frog.locus85.1:0.015263)85:0.016666)98
:0.049800)3:0.008549,
((Western_clawed_frog.locus229.1:0.030947,Western_clawed_frog.locus228.1:0.071558)45:0.007636
,
(Western_clawed_frog.locus135.1:0.029031,Western_clawed_frog.locus161.1:0.026302)92:0.025847)
51:0.020363)0:0.004648)1:0.011150,((((((Western_clawed_frog.locus74.1:0.003720,
(Western_clawed_frog.locus72.1:0.000001,Western_clawed_frog.locus73.1:0.003764)82:0.003785)10
0:0.022073,
(Western_clawed_frog.locus54.1:0.007307,Western_clawed_frog.locus56.1:0.003611)98:0.030674)83
:0.046596,
((Western_clawed_frog.locus195.1:0.039620,Western_clawed_frog.locus194.1:0.025187)54:0.015977
,
(((Western_clawed_frog.locus187.1:0.013473,Western_clawed_frog.locus185.1:0.004448)63:0.00908
0,
(Western_clawed_frog.locus123.1:0.010586,Western_clawed_frog.locus124.1:0.000001)34:0.005149)
51:0.000001,Western_clawed_frog.locus128.1:0.041693)43:0.016627)41:0.042258)1:0.008875,
(((Western_clawed_frog.locus69.1:0.074700,Western_clawed_frog.locus86.1:0.060655)13:0.012093,
Western_clawed_frog.locus225.1:0.081351)0:0.000001,
((Western_clawed_frog.locus109.1:0.031332,Western_clawed_frog.locus238.1:0.020949)100:0.05274
4,Western_clawed_frog.locus110.1:0.034933)26:0.021396)1:0.003781)0:0.000001,
((((Western_clawed_frog.locus57.1:0.000001,Western_clawed_frog.locus55.1:0.007558)100:0.0596
49,

(Western_clawed_frog.locus36.1:0.000001,Western_clawed_frog.locus38.1:0.007445)100:0.045721)5
2:0.042178,(Western_clawed_frog.locus63.1:0.050985,
(Western_clawed_frog.locus64.1:0.007444,Western_clawed_frog.locus65.1:0.011182)90:0.010693)50
:0.010517)5:0.011208,
((Western_clawed_frog.locus37.1:0.113889,Western_clawed_frog.locus214.1:0.039692)6:0.017195,
(Western_clawed_frog.locus122.1:0.033452,Western_clawed_frog.locus66.1:0.040901)33:0.018115)0
:0.002562)0:0.000001,(((((Western_clawed_frog.locus191.1:0.060526,
(Western_clawed_frog.locus120.1:0.078745,
(Western_clawed_frog.locus50.1:0.023916,Western_clawed_frog.locus60.1:0.029213)92:0.023722)18
:0.010620)11:0.018749,((Western_clawed_frog.locus6.1:0.028430,
(Western_clawed_frog.locus4.1:0.021241,
(Western_clawed_frog.locus2.1:0.016381,Western_clawed_frog.locus3.1:0.003700)98:0.030511)42:0
.009116)25:0.009095,
((((Western_clawed_frog.locus18.1:0.047793,Western_clawed_frog.locus19.1:0.035782)47:0.013681
,Western_clawed_frog.locus20.1:0.057139)30:0.010264,
(Western_clawed_frog.locus16.1:0.013608,Western_clawed_frog.locus17.1:0.038622)50:0.013620)23
:0.007833,
((((Western_clawed_frog.locus13.1:0.016425,Western_clawed_frog.locus12.1:0.011917)50:0.004776
,Western_clawed_frog.locus14.1:0.027283)45:0.013176,Western_clawed_frog.locus15.1:0.025432)29
:0.012447,Western_clawed_frog.locus11.1:0.035503)22:0.003796)38:0.017288)60:0.034858)0:0.0120
96,Western_clawed_frog.locus33.1:0.076827)0:0.000001,
(Western_clawed_frog.locus165.1:0.025818,Western_clawed_frog.locus164.1:0.017697)84:0.024891)
0:0.008477,
(((Western_clawed_frog.locus156.1:0.118113,Western_clawed_frog.locus158.1:0.007564)82:0.02766
2,(Western_clawed_frog.locus788.1:0.018621,(Western_clawed_frog.locus1062.1:0.007630,
(Western_clawed_frog.locus332.1:0.019650,Western_clawed_frog.locus787.1:0.037794)47:0.000001)
76:0.015427)75:0.027925)18:0.003656,(Western_clawed_frog.locus536.1:0.029357,
(Western_clawed_frog.locus502.1:0.006546,Western_clawed_frog.locus1028.1:0.015488)37:0.011859
)45:0.015915)22:0.009950)0:0.012773)0:0.010581)0:0.000001,
(((((((Western_clawed_frog.locus258.1:0.014998,Western_clawed_frog.locus263.1:0.014963)12:0.
000001,
(Western_clawed_frog.locus257.1:0.011522,Western_clawed_frog.locus262.1:0.015733)61:0.003840)
12:0.003750,
(Western_clawed_frog.locus261.1:0.019238,Western_clawed_frog.locus259.1:0.000001)52:0.003801)
3:0.000001,Western_clawed_frog.locus260.1:0.011679)96:0.039115,Western_clawed_frog.locus144.1
:0.078200)8:0.000001,
(((Western_clawed_frog.locus240.1:0.024743,Western_clawed_frog.locus245.1:0.011236)23:0.00000
1,
((Western_clawed_frog.locus236.1:0.018808,Western_clawed_frog.locus250.1:0.022118)24:0.000041
,(((Western_clawed_frog.locus255.1:0.003692,
((Western_clawed_frog.locus243.1:0.058476,Western_clawed_frog.locus241.1:0.007514)41:0.007764
,Western_clawed_frog.locus256.1:0.014752)12:0.000001)9:0.003907,
(Western_clawed_frog.locus252.1:0.018648,Western_clawed_frog.locus244.1:0.028116)34:0.003713)
4:0.000001,
((Western_clawed_frog.locus239.1:0.011069,Western_clawed_frog.locus246.1:0.007310)4:0.000001,
(Western_clawed_frog.locus247.1:0.033862,
(Western_clawed_frog.locus254.1:0.011078,Western_clawed_frog.locus251.1:0.003603)54:0.005227)
48:0.002146)9:0.003665)40:0.007654)25:0.003793)85:0.024300,
(((Western_clawed_frog.locus134.1:0.023968,Western_clawed_frog.locus170.1:0.032631)70:0.03031
0,Western_clawed_frog.locus146.1:0.023742)28:0.007390,Western_clawed_frog.locus145.1:0.031574
)77:0.042324)30:0.011808)25:0.011167,Western_clawed_frog.locus169.1:0.066376)15:0.007594,West
ern_clawed_frog.locus248.1:0.051527)24:0.019572)0:0.003634)0:0.011053)0:0.000001,
(((((Western_clawed_frog.locus207.1:0.019917,
(((Western_clawed_frog.locus204.1:0.000001,Western_clawed_frog.locus205.1:0.000001)92:0.00407
5,Western_clawed_frog.locus211.1:0.019242)38:0.007418,Western_clawed_frog.locus206.1:0.024057
)29:0.003730)61:0.023995,
(Western_clawed_frog.locus209.1:0.007861,Western_clawed_frog.locus208.1:0.023549)43:0.000001)
12:0.013350,Western_clawed_frog.locus210.1:0.022450)67:0.040790,
(Western_clawed_frog.locus9.1:0.027084,Western_clawed_frog.locus8.1:0.023594)99:0.028122)3:0.
015217,
((Western_clawed_frog.locus75.1:0.039553,Western_clawed_frog.locus76.1:0.029931)65:0.027924,
((((Western_clawed_frog.locus198.1:0.017996,
(Western_clawed_frog.locus200.1:0.016055,Western_clawed_frog.locus199.1:0.034430)89:0.035628)
65:0.015845,(((Western_clawed_frog.locus78.1:0.011383,
((Western_clawed_frog.locus80.1:0.000001,Western_clawed_frog.locus79.1:0.011467)93:0.019498,W
estern_clawed_frog.locus81.1:0.014230)62:0.013286)42:0.010473,Western_clawed_frog.locus77.1:0
.024014)99:0.048880,
(Western_clawed_frog.locus88.1:0.020272,Western_clawed_frog.locus87.1:0.037971)83:0.023360)13
:0.001790)26:0.008723,
(Western_clawed_frog.locus196.1:0.001830,Western_clawed_frog.locus197.1:0.019882)100:0.084745
)69:0.018396,Western_clawed_frog.locus280.1:0.025238)73:0.031115)27:0.011706)0:0.005441)0:0.0
03689)0:0.005839,
((((Western_clawed_frog.locus227.1:0.048519,Western_clawed_frog.locus213.1:0.049496)27:0.0103
57,(Western_clawed_frog.locus107.1:0.050811,
(Western_clawed_frog.locus106.1:0.027412,Western_clawed_frog.locus105.1:0.023864)57:0.018751)
73:0.027974)6:0.005509,Western_clawed_frog.locus212.1:0.033569)4:0.004551,
(Western_clawed_frog.locus193.1:0.053258,Western_clawed_frog.locus71.1:0.061487)51:0.026131)2
:0.010873)0:0.002407,((((Western_clawed_frog.locus288.1:0.004014,
((Western_clawed_frog.locus296.1:0.014544,

(Western_clawed_frog.locus294.1:0.011558,Western_clawed_frog.locus295.1:0.011562)68:0.006609)
41:0.009125,Western_clawed_frog.locus286.1:0.022522)20:0.003768)51:0.007616,
((Western_clawed_frog.locus290.1:0.015176,Western_clawed_frog.locus285.1:0.011259)39:0.007651
,
((Western_clawed_frog.locus291.1:0.007410,Western_clawed_frog.locus287.1:0.030926)62:0.007542
,Western_clawed_frog.locus292.1:0.007504)57:0.015135)24:0.007588)100:0.106628,
(Western_clawed_frog.locus166.1:0.141433,Western_clawed_frog.locus215.1:0.064028)6:0.005713)2
:0.016516,((Western_clawed_frog.locus217.1:0.003758,
((((Western_clawed_frog.locus218.1:0.038622,Western_clawed_frog.locus219.1:0.003658)53:0.0036
46,Western_clawed_frog.locus216.1:0.011057)49:0.003651,
(Western_clawed_frog.locus222.1:0.018399,Western_clawed_frog.locus221.1:0.010993)75:0.003621)
9:0.000001,Western_clawed_frog.locus224.1:0.003631)51:0.011012)99:0.051348,
(Western_clawed_frog.locus49.1:0.044392,Western_clawed_frog.locus53.1:0.012345)84:0.038493)53
:0.023174)0:0.023840)0:0.004843,Western_clawed_frog.locus226.1:0.043756)0:0.020052,
(Western_clawed_frog.locus70.1:0.109400,Western_clawed_frog.locus89.1:0.037290)24:0.020220)0:
0.011029,Western_clawed_frog.locus61.1:0.100679)7:0.012757)68:0.068830,Western_clawed_frog.lo
cus249.1:0.006695)22:0.007466,
(Western_clawed_frog.locus152.1:0.004071,Western_clawed_frog.locus151.1:0.012325)98:0.018095)
49:0.064947,Western_clawed_frog.locus100.1:0.046786)11:0.014569,
(Western_clawed_frog.locus91.1:0.005354,((Western_clawed_frog.locus94.1:0.000001,
(Western_clawed_frog.locus95.1:0.019498,
(Western_clawed_frog.locus96.1:0.011748,Western_clawed_frog.locus90.1:0.068951)24:0.012716)18
:0.004168)22:0.011633,
(Western_clawed_frog.locus93.1:0.012785,Western_clawed_frog.locus92.1:0.007702)86:0.006443)23
:0.009541)81:0.105940)64:0.112080,
(((Western_clawed_frog.locus279.1:0.057289,Western_clawed_frog.locus98.1:0.184655)92:0.041290
,(Western_clawed_frog.locus202.1:0.171538,
((Western_clawed_frog.locus68.1:0.032590,Western_clawed_frog.locus67.1:0.009629)100:0.042062,
((Western_clawed_frog.locus103.1:0.047070,Western_clawed_frog.locus102.1:0.037835)72:0.007593
,Western_clawed_frog.locus101.1:0.021663)63:0.000001)94:0.068668)48:0.034903)21:0.017783,
(((Western_clawed_frog.locus131.1:0.014925,
(((Western_clawed_frog.locus133.1:0.020130,Western_clawed_frog.locus130.1:0.019073)79:0.03139
6,Western_clawed_frog.locus132.1:0.004532)42:0.011277,
(Western_clawed_frog.locus150.1:0.007639,(Western_clawed_frog.locus168.1:0.022382,
(Western_clawed_frog.locus148.1:0.004385,Western_clawed_frog.locus149.1:0.010833)59:0.007687)
58:0.004311)53:0.014995)25:0.009286)94:0.058936,
(((Western_clawed_frog.locus153.1:0.032509,Western_clawed_frog.locus154.1:0.014419)44:0.00699
7,Western_clawed_frog.locus108.1:0.027477)85:0.029611,
((Western_clawed_frog.locus183.1:0.021432,
((((Western_clawed_frog.locus176.1:0.015350,Western_clawed_frog.locus173.1:0.011505)50:0.0037
88,Western_clawed_frog.locus180.1:0.023899)41:0.000001,
(((Western_clawed_frog.locus174.1:0.015290,Western_clawed_frog.locus177.1:0.042052)36:0.00000
1,(((Western_clawed_frog.locus178.1:0.030151,(Western_clawed_frog.locus242.1:0.506975,
(Western_clawed_frog.locus142.1:0.031662,Western_clawed_frog.locus172.1:0.042882)66:0.022553)
43:0.022376)1:0.004357,(Western_clawed_frog.locus141.1:0.030843,
(Western_clawed_frog.locus139.1:0.008575,Western_clawed_frog.locus175.1:0.017242)51:0.005728)
25:0.000001)1:0.011459,Western_clawed_frog.locus140.1:0.011599)0:0.007660)0:0.003790,
(Western_clawed_frog.locus129.1:0.027012,Western_clawed_frog.locus138.1:0.007574)10:0.003798)
10:0.007818)15:0.009285,
(Western_clawed_frog.locus184.1:0.026101,Western_clawed_frog.locus126.1:0.013126)34:0.007706)
29:0.012167)65:0.038125,Western_clawed_frog.locus97.1:0.058497)62:0.020485)74:0.032004)55:0.0
36477,((Western_clawed_frog.locus268.1:0.010023,
(Western_clawed_frog.locus265.1:0.000001,Western_clawed_frog.locus266.1:0.010938)83:0.015534)
100:0.150819,Western_clawed_frog.locus283.1:0.221865)34:0.011431)34:0.010987)42:0.039437)97:0
.247874)99:0.223233)17:0.041134,
((((((Anole_lizard.locus96.1:0.019433,Anole_lizard.locus98.1:0.014884)77:0.015241,Anole_lizar
d.locus97.1:0.004286)100:0.360839,
(((Anole_lizard.locus99.1:0.339236,Anole_lizard.locus141.1:0.308064)12:0.068217,
(Anole_lizard.locus145.1:0.343417,Anole_lizard.locus148.1:0.516764)8:0.035970)1:0.033150,
((((Anole_lizard.locus181.1:0.031070,Anole_lizard.locus182.1:0.004171)100:0.208081,
((Anole_lizard.locus180.1:0.325576,Anole_lizard.locus179.1:0.228686)88:0.066614,
(Anole_lizard.locus178.1:0.000001,Anole_lizard.locus177.1:0.007656)100:0.238505)96:0.113602)1
0:0.025109,Anole_lizard.locus127.1:0.325637)7:0.043231,
((((Anole_lizard.locus146.1:0.006501,Anole_lizard.locus149.1:0.033736)100:0.215388,Anole_liza
rd.locus147.1:0.261080)31:0.070850,
(Anole_lizard.locus152.1:0.351847,Anole_lizard.locus144.1:0.283116)29:0.038342)4:0.020862,
((Anole_lizard.locus109.1:0.296429,Anole_lizard.locus150.1:0.288706)18:0.037626,
((((Anole_lizard.locus122.1:0.000570,Anole_lizard.locus121.1:0.018515)100:0.268453,
((Anole_lizard.locus123.1:0.007362,Anole_lizard.locus125.1:0.018624)100:0.310929,
(Anole_lizard.locus108.1:0.072138,Anole_lizard.locus106.1:0.152194)100:0.181499)36:0.043015)1
5:0.028553,((((Anole_lizard.locus107.1:0.502336,Anole_lizard.locus120.1:0.207793)61:0.079062,
(Anole_lizard.locus117.1:0.045943,(((Anole_lizard.locus124.1:0.035411,
(Anole_lizard.locus113.1:0.043647,Anole_lizard.locus112.1:0.024957)99:0.096220)29:0.006902,An
ole_lizard.locus119.1:0.106101)35:0.024931,Anole_lizard.locus115.1:0.039684)31:0.027050)100:0
.234977)23:0.028852,Anole_lizard.locus135.1:0.624369)13:0.038079,
(Anole_lizard.locus114.1:0.257841,(Anole_lizard.locus111.1:0.078238,
(Anole_lizard.locus102.1:0.000001,((Anole_lizard.locus103.1:0.012958,
(Anole_lizard.locus101.1:0.131009,Anole_lizard.locus104.1:0.002600)57:0.049679)21:0.004923,
(Anole_lizard.locus100.1:0.036940,Anole_lizard.locus105.1:0.006672)52:0.006519)30:0.007865)73

:0.071119)100:0.185244)15:0.033536)0:0.004716)14:0.018880,Anole_lizard.locus155.1:0.297450)4:
0.030862)4:0.027271)0:0.028969)0:0.028688)1:0.026327)5:0.019777,
(((Anole_lizard.locus175.1:0.390752,
(((Anole_lizard.locus184.1:0.023865,Anole_lizard.locus183.1:0.039406)100:0.204346,Anole_lizar
d.locus204.1:0.220275)99:0.090088,
(Anole_lizard.locus211.1:0.042751,Anole_lizard.locus37.1:0.047370)100:0.224454)81:0.088439)52
:0.067175,(Anole_lizard.locus143.1:0.457415,(Anole_lizard.locus142.1:0.421066,
(Anole_lizard.locus129.1:0.769751,Anole_lizard.locus130.1:0.286335)72:0.078855)17:0.061010)1:
0.022337)1:0.028311,
((Anole_lizard.locus140.1:0.313686,Anole_lizard.locus137.1:0.415351)8:0.037819,Anole_lizard.l
ocus139.1:0.523882)0:0.009375)1:0.020823)73:0.104260,
(((((Mouse.locus114.1:0.018708,Mouse.locus113.1:0.013617)100:0.066191,
(((Mouse.locus106.1:0.003965,
(Mouse.locus110.1:0.000001,Mouse.locus108.1:0.000001)78:0.003830)100:0.033279,Mouse.locus105.
1:0.075958)89:0.033868,(Mouse.locus117.1:0.069728,
(Mouse.locus116.1:0.086870,Mouse.locus115.1:0.076205)59:0.030390)94:0.053996)32:0.008661)35:0
.012958,(Mouse.locus111.1:0.058146,Mouse.locus112.1:0.040682)88:0.021330)100:0.434428,
(((Mouse.locus221.1:0.024265,Mouse.locus220.1:0.057659)47:0.007368,
(Mouse.locus223.1:0.011967,Mouse.locus222.1:0.003603)98:0.025578)100:0.526036,
(((Mouse.locus93.1:0.055922,Mouse.locus92.1:0.073776)56:0.007380,Mouse.locus317.1:0.073817)10
0:0.305841,(((((Mouse.locus348.1:0.095582,(Mouse.locus346.1:0.015290,
(Mouse.locus347.1:0.027863,Mouse.locus345.1:0.048713)30:0.003624)100:0.204336)100:0.219377,
((((((Mouse.locus246.1:0.018410,
(Mouse.locus248.1:0.010919,Mouse.locus244.1:0.000001)100:0.022257)82:0.012458,Mouse.locus224.
1:0.032134)100:0.066560,(Mouse.locus237.1:0.048286,((Mouse.locus232.1:0.039536,
(Mouse.locus227.1:0.076949,
(Mouse.locus231.1:0.023561,Mouse.locus229.1:0.003265)97:0.017015)76:0.018831)46:0.009232,
(Mouse.locus234.1:0.053622,
(Mouse.locus243.1:0.028886,Mouse.locus239.1:0.048175)65:0.009173)39:0.007205)20:0.010035)94:0
.047398)63:0.027309,Mouse.locus403.1:0.094346)100:0.100232,((Mouse.locus462.1:0.052577,
(((Mouse.locus409.1:0.040572,Mouse.locus448.1:0.088414)37:0.004647,
(Mouse.locus413.1:0.073854,
((Mouse.locus452.1:0.046343,Mouse.locus453.1:0.021967)76:0.019607,Mouse.locus410.1:0.068802)2
9:0.005099)18:0.004459)10:0.005093,
((Mouse.locus440.1:0.045369,Mouse.locus434.1:0.042723)58:0.012661,
(((Mouse.locus415.1:0.046680,Mouse.locus432.1:0.079810)16:0.000001,
((Mouse.locus421.1:0.026734,
(Mouse.locus430.1:0.028150,Mouse.locus426.1:0.031069)30:0.003192)12:0.000001,Mouse.locus419.1
:0.022121)35:0.004394)53:0.008875,Mouse.locus436.1:0.031309)60:0.010979)45:0.013675)36:0.0082
66)93:0.040602,(Mouse.locus456.1:0.154916,
((Mouse.locus467.1:0.014181,Mouse.locus465.1:0.052975)100:0.060804,Mouse.locus458.1:0.082653)
71:0.018833)81:0.023342)99:0.069619)99:0.065982,Mouse.locus90.1:0.259791)55:0.037555)99:0.098
998,(Mouse.locus494.1:0.196181,
(((Mouse.locus218.1:0.011984,Mouse.locus120.1:0.026489)100:0.068966,
((((Mouse.locus155.1:0.000001,Mouse.locus181.1:0.003667)100:0.007430,Mouse.locus214.1:0.01894
8)89:0.015840,(Mouse.locus191.1:0.000001,Mouse.locus140.1:0.003673)100:0.034042)86:0.010730,
((((Mouse.locus159.1:0.007437,Mouse.locus126.1:0.003712)62:0.007437,
((Mouse.locus176.1:0.000001,Mouse.locus208.1:0.003789)96:0.010944,Mouse.locus150.1:0.007293)9
6:0.011837)44:0.000001,
(Mouse.locus133.1:0.000001,Mouse.locus183.1:0.007416)78:0.003703)77:0.007491,
(Mouse.locus122.1:0.000001,
(((Mouse.locus134.1:0.026698,Mouse.locus185.1:0.003774)92:0.018679,
(((((Mouse.locus136.1:0.003712,Mouse.locus187.1:0.003722)73:0.007461,Mouse.locus163.1:0.01517
7)19:0.003735,((Mouse.locus124.1:0.000001,(((Mouse.locus128.1:0.007620,
((Mouse.locus151.1:0.003788,Mouse.locus177.1:0.000001)58:0.000001,
(Mouse.locus210.1:0.000001,Mouse.locus216.1:0.007630)89:0.019110)29:0.003814)24:0.007677,
((Mouse.locus198.1:0.000001,Mouse.locus206.1:0.000001)36:0.000001,Mouse.locus174.1:0.000001)8
0:0.003757)8:0.003772,
((Mouse.locus170.1:0.003768,Mouse.locus143.1:0.000001)57:0.000001,Mouse.locus161.1:0.015172)6
1:0.007621)11:0.000001)33:0.003773,
((Mouse.locus153.1:0.003734,Mouse.locus212.1:0.003767)42:0.000001,Mouse.locus130.1:0.003736)9
3:0.038030)13:0.000001)55:0.022826,
(Mouse.locus192.1:0.000001,Mouse.locus200.1:0.000001)100:0.018863)57:0.018846,Mouse.locus157.
1:0.003691)28:0.007444)9:0.000001,
(Mouse.locus172.1:0.003717,Mouse.locus145.1:0.003709)64:0.003714)0:0.000001)31:0.007461)35:0.
004109)82:0.041050)100:0.118805,
((((((Mouse.locus481.1:0.014589,Mouse.locus484.1:0.023490)92:0.029505,Mouse.locus489.1:0.0057
05)61:0.011978,Mouse.locus487.1:0.022852)93:0.020107,Mouse.locus486.1:0.032788)93:0.022747,
(Mouse.locus469.1:0.007992,Mouse.locus472.1:0.017230)100:0.058832)100:0.056682,
(Mouse.locus477.1:0.051786,
(Mouse.locus474.1:0.016094,Mouse.locus476.1:0.004454)53:0.004520)99:0.067901)100:0.121367)90:
0.058943)45:0.026381)20:0.014175,((((((((Mouse.locus391.1:0.041983,
(Mouse.locus63.1:0.021273,Mouse.locus508.1:0.015680)79:0.011172)46:0.009156,Mouse.locus389.1:
0.064124)82:0.021524,
((Mouse.locus755.1:0.000001,Mouse.locus338.1:0.000001)100:0.025059,Mouse.locus87.1:0.260868)5
8:0.021489)36:0.007540,(Mouse.locus354.1:0.034341,
((Mouse.locus352.1:0.014003,Mouse.locus353.1:0.020127)46:0.007460,Mouse.locus351.1:0.021525)7
0:0.008525)96:0.021956)44:0.007882,Mouse.locus17.1:0.063960)60:0.048004,
((Mouse.locus82.1:0.030570,Mouse.locus85.1:0.040966)54:0.010514,((Mouse.locus77.1:0.056020,

(((Mouse.locus74.1:0.011483,
(Mouse.locus75.1:0.027475,Mouse.locus78.1:0.011375)66:0.003754)71:0.000001,Mouse.locus79.1:0.
034317)76:0.015295,Mouse.locus83.1:0.050776)31:0.003748)61:0.005178,Mouse.locus80.1:0.038597)
63:0.010635)63:0.014327)32:0.003841,Mouse.locus86.1:0.085407)100:0.210888,
((Mouse.locus302.1:0.274040,(((Mouse.locus307.1:0.041456,
(Mouse.locus309.1:0.021916,Mouse.locus311.1:0.056121)50:0.019094)66:0.027899,Mouse.locus305.1
:0.032260)100:0.074594,((Mouse.locus298.1:0.077607,Mouse.locus255.1:0.067821)32:0.004418,
((((Mouse.locus261.1:0.045454,Mouse.locus264.1:0.037360)51:0.009992,Mouse.locus280.1:0.046685
)98:0.023016,((Mouse.locus276.1:0.075730,
(Mouse.locus289.1:0.061097,Mouse.locus271.1:0.048714)57:0.012337)64:0.015460,Mouse.locus268.1
:0.027035)49:0.009352)37:0.004029,Mouse.locus286.1:0.096061)39:0.005563)99:0.082237)100:0.097
728)57:0.023212,Mouse.locus254.1:0.208916)77:0.077973)23:0.015433)60:0.043192,
(Mouse.locus758.1:0.000001,Mouse.locus493.1:0.000001)100:0.276503)65:0.029502)100:0.154740)98
:0.167077)43:0.058356,(Anole_lizard.locus190.1:0.175740,
((Anole_lizard.locus187.1:0.101379,Anole_lizard.locus188.1:0.096281)53:0.021527,Anole_lizard.
locus186.1:0.171463)84:0.039386)100:0.231265)34:0.039498)76:0.082632,
(((Western_clawed_frog.locus942.1:0.307885,
(((Western_clawed_frog.locus1005.1:0.006210,Western_clawed_frog.locus1006.1:0.005403)100:0.11
1258,
(Western_clawed_frog.locus1003.1:0.019977,Western_clawed_frog.locus1004.1:0.018452)100:0.0880
68)100:0.176312,(((((Western_clawed_frog.locus1014.1:0.009395,
(Western_clawed_frog.locus997.1:0.004084,
(Western_clawed_frog.locus1009.1:0.014939,Western_clawed_frog.locus1007.1:0.000001)58:0.00000
1)50:0.005526)92:0.020584,Western_clawed_frog.locus908.1:0.041298)90:0.035849,Western_clawed_
frog.locus1011.1:0.012697)100:0.304228,Western_clawed_frog.locus1000.1:0.259963)46:0.038486,W
estern_clawed_frog.locus1001.1:0.200561)58:0.051866)83:0.070607)100:0.159337,
((((((((Caecillan.locus40424.1:0.000001,Caecillan.locus40426.1:0.003841)100:0.033109,
((Caecillan.locus40565.1:0.011927,
((((Caecillan.locus40573.1:0.019941,Caecillan.locus40574.1:0.023960)5:0.000001,
((Caecillan.locus40585.1:0.015954,Caecillan.locus40568.1:0.020029)16:0.000001,
((Caecillan.locus40580.1:0.036476,((Caecillan.locus40572.1:0.011969,
(Caecillan.locus40581.1:0.015964,Caecillan.locus40571.1:0.007931)61:0.015966)28:0.000001,Caec
illan.locus40584.1:0.020694)44:0.003990)21:0.003948,
(Caecillan.locus40569.1:0.020122,Caecillan.locus40579.1:0.003898)55:0.012053)6:0.000001)6:0.0
03977)3:0.003928,(Caecillan.locus40582.1:0.024188,
(Caecillan.locus40566.1:0.024107,Caecillan.locus40578.1:0.016067)29:0.003986)21:0.004108)30:0
.000001,Caecillan.locus40570.1:0.015993)61:0.004052)97:0.031284,Caecillan.locus40395.1:0.0595
09)27:0.008376)29:0.007419,Caecillan.locus40454.1:0.040521)74:0.035626,
(Caecillan.locus40586.1:0.078883,
(((Caecillan.locus40355.1:0.030444,Caecillan.locus40354.1:0.046278)93:0.021410,Caecillan.locu
s40349.1:0.102997)35:0.006726,Caecillan.locus40294.1:0.067659)70:0.024041)19:0.006659)22:0.02
6999,(Caecillan.locus40288.1:0.145213,
((Caecillan.locus40460.1:0.012225,Caecillan.locus40458.1:0.015206)90:0.015774,Caecillan.locus
40462.1:0.028270)79:0.039904)17:0.005970)28:0.019012,
(((((Caecillan.locus40464.1:0.070740,Caecillan.locus40463.1:0.056849)34:0.011661,Caecillan.lo
cus40335.1:0.035939)33:0.004560,Caecillan.locus40455.1:0.040098)68:0.019645,Caecillan.locus40
291.1:0.041286)22:0.007703,(Caecillan.locus40466.1:0.090410,(Caecillan.locus40334.1:0.110741,
((Caecillan.locus40338.1:0.068117,
(((Caecillan.locus40336.1:0.014151,Caecillan.locus40337.1:0.013325)87:0.019727,
(Caecillan.locus40448.1:0.090674,Caecillan.locus40387.1:0.032231)16:0.001847)17:0.005929,
((Caecillan.locus40450.1:0.040576,Caecillan.locus40390.1:0.032203)49:0.015672,Caecillan.locus
40385.1:0.031679)36:0.004489)10:0.010213)10:0.003969,((Caecillan.locus40363.1:0.050731,
(Caecillan.locus40362.1:0.003699,Caecillan.locus40361.1:0.011861)100:0.035397)35:0.010941,
(Caecillan.locus40339.1:0.024096,(Caecillan.locus40364.1:0.010514,
(Caecillan.locus40371.1:0.000001,Caecillan.locus40365.1:0.007820)100:0.020930)92:0.026602)41:
0.008787)15:0.003988)83:0.030039)23:0.008814)15:0.019730)9:0.011749)13:0.027617,Caecillan.loc
us40356.1:0.103248)95:0.079559,((((((Caecillan.locus40509.1:0.059319,
(Caecillan.locus40508.1:0.028566,Caecillan.locus40513.1:0.047672)71:0.018692)40:0.010992,Caec
illan.locus40511.1:0.027012)57:0.039531,Caecillan.locus40506.1:0.075914)100:0.147302,Caecilla
n.locus40514.1:0.241018)9:0.002554,Caecillan.locus40492.1:0.250303)9:0.021532,
((Caecillan.locus36723.1:0.140553,(Caecillan.locus40541.1:0.071781,
((Caecillan.locus40435.1:0.079209,(Caecillan.locus40434.1:0.063306,
(Caecillan.locus36727.1:0.014794,Caecillan.locus36726.1:0.020453)76:0.020480)81:0.026899)42:0
.017211,Caecillan.locus36728.1:0.053294)91:0.051847)76:0.040054)44:0.020558,
((Caecillan.locus36921.1:0.003891,Caecillan.locus36920.1:0.025039)99:0.085953,
((((((((((((((Caecillan.locus40408.1:0.000001,
(Caecillan.locus40416.1:0.011529,Caecillan.locus40407.1:0.052786)49:0.011554)98:0.058484,Caec
illan.locus40313.1:0.070283)10:0.006091,Caecillan.locus40323.1:0.015569)3:0.003853,
(Caecillan.locus40403.1:0.043993,((Caecillan.locus40404.1:0.062667,
((Caecillan.locus40376.1:0.000001,
(Caecillan.locus40377.1:0.000001,Caecillan.locus40369.1:0.008191)82:0.004021)95:0.004031,
(((Caecillan.locus40384.1:0.000001,Caecillan.locus40370.1:0.003893)77:0.007969,Caecillan.locu
s40379.1:0.003700)84:0.027931,Caecillan.locus40382.1:0.003938)34:0.004001)100:0.074413)21:0.0
16593,Caecillan.locus40402.1:0.085353)4:0.004670)6:0.007616)1:0.000001,
((Caecillan.locus40481.1:0.037402,Caecillan.locus40397.1:0.047317)88:0.015878,Caecillan.locus
40325.1:0.034364)81:0.017295)1:0.008020,((Caecillan.locus40307.1:0.000001,
(Caecillan.locus40311.1:0.000001,
((Caecillan.locus40310.1:0.011345,Caecillan.locus40308.1:0.003741)34:0.000001,Caecillan.locus
40309.1:0.003770)15:0.000001)65:0.003735)100:0.061674,Caecillan.locus40315.1:0.039198)33:0.00

9804)0:0.004883,((Caecillan.locus40406.1:0.087064,((Caecillan.locus36943.1:0.027699,
((Caecillan.locus40477.1:0.039324,Caecillan.locus40480.1:0.038686)94:0.046810,
((((Caecillan.locus36951.1:0.003807,Caecillan.locus36744.1:0.000001)100:0.039500,
(Caecillan.locus36738.1:0.070784,Caecillan.locus36949.1:0.005256)71:0.028948)55:0.008766,
(Caecillan.locus36950.1:0.003856,
(Caecillan.locus36743.1:0.003858,Caecillan.locus36734.1:0.003863)63:0.000001)96:0.024452)12:0
.000001,((Caecillan.locus36944.1:0.044253,
(Caecillan.locus36736.1:0.003869,Caecillan.locus36732.1:0.011594)58:0.003855)28:0.003895,
((Caecillan.locus36948.1:0.000001,(Caecillan.locus36947.1:0.003940,
(Caecillan.locus36946.1:0.003883,Caecillan.locus36735.1:0.007772)73:0.000001)98:0.015725)84:0
.019943,Caecillan.locus36733.1:0.019570)8:0.000001)54:0.007641)60:0.011820)10:0.004026)8:0.00
3770,
(Caecillan.locus36740.1:0.003829,Caecillan.locus36742.1:0.000001)100:0.027682)8:0.015709)1:0.
000001,
(((Caecillan.locus40401.1:0.064117,Caecillan.locus40317.1:0.030244)71:0.022527,Caecillan.locu
s40318.1:0.055039)30:0.012470,
((Caecillan.locus40473.1:0.027914,Caecillan.locus40470.1:0.011596)100:0.023795,Caecillan.locu
s40476.1:0.060901)55:0.019831)7:0.004277)0:0.003839)1:0.000001,Caecillan.locus40321.1:0.06072
6)3:0.014678,(((Caecillan.locus40280.1:0.011366,Caecillan.locus40278.1:0.004079)100:0.122001,
((Caecillan.locus40281.1:0.077970,(Caecillan.locus40306.1:0.074922,
(Caecillan.locus40322.1:0.060072,Caecillan.locus40314.1:0.029118)98:0.034973)79:0.019930)73:0
.020124,
(Caecillan.locus40283.1:0.049636,Caecillan.locus40348.1:0.070624)57:0.025867)15:0.017739)4:0.
004816,
(Caecillan.locus40429.1:0.081130,Caecillan.locus40312.1:0.113826)9:0.006376)26:0.013958)6:0.0
04746,Caecillan.locus40400.1:0.057073)40:0.019993,Caecillan.locus40286.1:0.056408)81:0.038045
,(((Caecillan.locus36963.1:0.017014,Caecillan.locus36960.1:0.059628)84:0.028870,
(Caecillan.locus36959.1:0.051946,Caecillan.locus36962.1:0.031251)32:0.000954)32:0.008252,Caec
illan.locus40302.1:0.056343)90:0.037488)30:0.011696,((((Caecillan.locus36915.1:0.039718,
((Caecillan.locus36909.1:0.014379,Caecillan.locus36910.1:0.015258)77:0.015084,
(Caecillan.locus36911.1:0.030307,Caecillan.locus36912.1:0.040446)53:0.011909)23:0.005368)7:0.
004563,Caecillan.locus36913.1:0.063906)4:0.000001,Caecillan.locus36907.1:0.039516)29:0.004351
,(Caecillan.locus36908.1:0.044960,(Caecillan.locus36955.1:0.043513,
((Caecillan.locus40485.1:0.002315,Caecillan.locus40486.1:0.021247)100:0.045439,
(Caecillan.locus36954.1:0.048094,Caecillan.locus40412.1:0.080140)29:0.000001)12:0.004376)28:0
.000001)36:0.004401)60:0.015429)44:0.028054,
(((Caecillan.locus40499.1:0.010411,Caecillan.locus40498.1:0.040696)79:0.024749,
(Caecillan.locus40494.1:0.006825,
((Caecillan.locus40504.1:0.013855,Caecillan.locus40497.1:0.008975)57:0.011578,Caecillan.locus
40502.1:0.021703)73:0.005897)70:0.023312)100:0.300687,
(Caecillan.locus40443.1:0.098400,Caecillan.locus40431.1:0.065360)86:0.035036)19:0.036165)8:0.
019561,((Caecillan.locus40410.1:0.083222,Caecillan.locus40284.1:0.106272)46:0.017329,
(((((Caecillan.locus40428.1:0.031459,
(Caecillan.locus40421.1:0.000001,Caecillan.locus40422.1:0.000001)99:0.017227)100:0.023643,Cae
cillan.locus40420.1:0.026380)98:0.036391,
((Caecillan.locus40409.1:0.049775,Caecillan.locus40417.1:0.037854)84:0.020941,
(Caecillan.locus40418.1:0.019364,Caecillan.locus40415.1:0.027470)100:0.027157)56:0.004457)98:
0.052686,Caecillan.locus40413.1:0.099718)46:0.013637,Caecillan.locus40414.1:0.086902)71:0.029
147)59:0.017812)12:0.019256,(Caecillan.locus40341.1:0.123824,
((((((((Caecillan.locus40533.1:0.009077,Caecillan.locus40531.1:0.000001)95:0.020747,
((Caecillan.locus40540.1:0.058523,Caecillan.locus40534.1:0.015201)70:0.012140,
((Caecillan.locus40537.1:0.004046,Caecillan.locus40539.1:0.008102)65:0.000001,Caecillan.locus
40538.1:0.016316)86:0.018224)17:0.007803)27:0.011021,
(Caecillan.locus40520.1:0.024271,Caecillan.locus40527.1:0.008077)45:0.008206)11:0.004016,
(Caecillan.locus40528.1:0.012233,Caecillan.locus40530.1:0.020111)99:0.020387)18:0.008526,Caec
illan.locus40522.1:0.020522)31:0.010947,Caecillan.locus40519.1:0.015650)16:0.014906,
(Caecillan.locus40526.1:0.058523,
(Caecillan.locus40517.1:0.029195,Caecillan.locus40518.1:0.033310)55:0.010204)61:0.008078)14:0
.007970,(Caecillan.locus40524.1:0.054633,
(Caecillan.locus40523.1:0.020395,Caecillan.locus40525.1:0.016531)84:0.012646)22:0.004278)57:0
.027032,(Caecillan.locus40433.1:0.089590,(Caecillan.locus40274.1:0.108407,
(Caecillan.locus40276.1:0.033178,Caecillan.locus40432.1:0.078336)52:0.015285)9:0.003041)35:0.
020239)74:0.030844)98:0.053078)5:0.008361,(((Caecillan.locus36939.1:0.043026,
(Caecillan.locus36940.1:0.011566,Caecillan.locus36942.1:0.011463)100:0.039465)10:0.000001,
((Caecillan.locus36937.1:0.074299,Caecillan.locus36860.1:0.034334)30:0.003680,
(Caecillan.locus36862.1:0.008915,Caecillan.locus36730.1:0.017752)100:0.034737)39:0.008765)36:
0.017502,(Caecillan.locus40345.1:0.109234,
(((Caecillan.locus36931.1:0.007582,Caecillan.locus36928.1:0.000001)100:0.043402,
((((Caecillan.locus40438.1:0.016602,Caecillan.locus40440.1:0.034052)99:0.034443,Caecillan.loc
us36934.1:0.036044)25:0.003985,Caecillan.locus36926.1:0.042582)53:0.005166,Caecillan.locus403
44.1:0.043140)27:0.003794)44:0.011155,
(Caecillan.locus40343.1:0.027106,Caecillan.locus40441.1:0.038606)97:0.028096)63:0.025552)22:0
.001349)94:0.085548)5:0.022386,Caecillan.locus40342.1:0.173934)5:0.018039)2:0.019223)8:0.0218
32)10:0.010931)18:0.018638,(((Caecillan.locus40332.1:0.142089,
(Caecillan.locus40296.1:0.098056,Caecillan.locus40393.1:0.093649)58:0.021709)9:0.007021,
((Caecillan.locus40331.1:0.025792,Caecillan.locus40467.1:0.038801)88:0.019480,Caecillan.locus
40394.1:0.066089)98:0.044490)18:0.010929,
(((Caecillan.locus40487.1:0.067735,Caecillan.locus40489.1:0.062343)78:0.026589,
(Caecillan.locus40491.1:0.063775,Caecillan.locus40297.1:0.073735)68:0.017966)52:0.022598,

((Caecillan.locus40360.1:0.079562,(Caecillan.locus40446.1:0.104826,
(Caecillan.locus40303.1:0.055844,Caecillan.locus40490.1:0.057841)61:0.016631)50:0.006813)54:0
.007097,(((Caecillan.locus40447.1:0.035709,Caecillan.locus40328.1:0.081653)100:0.055444,
(Caecillan.locus40327.1:0.110144,Caecillan.locus40392.1:0.016261)56:0.013624)54:0.008344,
(Caecillan.locus40469.1:0.045732,Caecillan.locus40329.1:0.015730)100:0.071596)35:0.011813)62:
0.033151)25:0.013070)97:0.126369)56:0.093860)40:0.041366,Western_clawed_frog.locus890.1:0.660
572)48:0.066650)28:0.053493)35:0.048745)25:0.040627,
(((Western_clawed_frog.locus977.1:0.046922,Western_clawed_frog.locus978.1:0.038040)85:0.04779
5,((Western_clawed_frog.locus1023.1:0.009951,(Western_clawed_frog.locus1021.1:0.007336,
((Western_clawed_frog.locus1017.1:0.007733,
(Western_clawed_frog.locus1022.1:0.011485,Western_clawed_frog.locus1019.1:0.003737)62:0.01119
5)27:0.003852,
(Western_clawed_frog.locus1018.1:0.007728,Western_clawed_frog.locus1020.1:0.000001)67:0.00369
2)38:0.003732)83:0.016470)100:0.049370,((((Western_clawed_frog.locus964.1:0.026493,
((Western_clawed_frog.locus973.1:0.018642,
(Western_clawed_frog.locus976.1:0.050047,Western_clawed_frog.locus952.1:0.012746)32:0.005707)
5:0.004525,(((Western_clawed_frog.locus959.1:0.023398,
(((Western_clawed_frog.locus953.1:0.030376,Western_clawed_frog.locus971.1:0.026594)12:0.00000
1,Western_clawed_frog.locus975.1:0.014967)10:0.003727,Western_clawed_frog.locus969.1:0.007482
)10:0.003814)1:0.003666,Western_clawed_frog.locus972.1:0.034047)1:0.000001,
((Western_clawed_frog.locus957.1:0.015260,(Western_clawed_frog.locus967.1:0.027206,
(Western_clawed_frog.locus963.1:0.003856,
(Western_clawed_frog.locus965.1:0.019225,Western_clawed_frog.locus966.1:0.003464)97:0.015223)
62:0.003778)33:0.011396)4:0.007375,Western_clawed_frog.locus960.1:0.011206)0:0.000001)0:0.003
728)0:0.000001)0:0.003736,(Western_clawed_frog.locus974.1:0.018934,
((Western_clawed_frog.locus961.1:0.026559,
((Western_clawed_frog.locus950.1:0.011393,Western_clawed_frog.locus962.1:0.022614)34:0.007636
,Western_clawed_frog.locus951.1:0.026353)7:0.003603)5:0.007661,Western_clawed_frog.locus968.1
:0.015161)5:0.007457)2:0.003703)54:0.020103,
((Western_clawed_frog.locus948.1:0.020883,Western_clawed_frog.locus955.1:0.044566)95:0.039138
,Western_clawed_frog.locus958.1:0.031646)84:0.022275)98:0.064900,Western_clawed_frog.locus979
.1:0.062400)55:0.018389)92:0.097900)100:0.404190,Western_clawed_frog.locus875.1:0.543430)33:0
.058461)17:0.029603,(Caecillan.locus36917.1:0.438444,
(Western_clawed_frog.locus531.1:0.285383,Caecillan.locus10029.1:0.245922)100:0.253702)32:0.05
5225)52:0.079037)5:0.026353)5:0.024047,
((Western_clawed_frog.locus417.1:0.031794,Western_clawed_frog.locus416.1:0.013340)100:0.55101
8,
(Anole_lizard.locus203.1:0.988059,Western_clawed_frog.locus499.1:0.604860)38:0.147927)22:0.14
5348)10:0.025497)92:0.271844)52:0.070673,(((Elephant_shark.locus27.1:0.031627,Small-
eyed_rabbitfish.locus110.1:0.125245)96:0.151032,((Smalltooth_sawfish.locus19.1:0.052890,
(Little_skate.locus49.1:0.019806,Thorny_skate.locus23.1:0.013179)100:0.151599)79:0.043388,
((Great_white_shark.locus11.1:0.055465,
(Brownbanded_bambooshark.locus520.1:0.026139,Whale_shark.locus98.1:0.004686)100:0.057501)51:0
.011648,Cloudy_catshark.locus79.1:0.070956)55:0.025209)93:0.087479)100:0.136137,
(((Reedfish.locus27372.1:0.134877,((Spotted_gar.locus169.1:0.066913,
(Zebrafish.locus541.1:0.239114,Japanese_eel.locus277.1:0.166619)100:0.199622)100:0.111947,Ste
rlet.locus180.1:0.077158)36:0.020064)95:0.105936,
(((((Mouse.locus35.1:0.003613,Mouse.locus41.1:0.004048)100:0.045422,
(Mouse.locus26.1:0.054462,((Mouse.locus33.1:0.045207,
(Mouse.locus23.1:0.011444,Mouse.locus22.1:0.007797)76:0.006648)18:0.012154,Mouse.locus28.1:0.
023974)44:0.017100)24:0.000001)100:0.366803,Anole_lizard.locus40.1:0.224276)99:0.088675,Caeci
llan.locus34702.1:0.129554)89:0.065890,Western_clawed_frog.locus568.1:0.199678)100:0.209424)4
5:0.039618,Coelacanth.locus28.1:0.212125)88:0.126574)100:0.389686)98:0.718490)73:0.023310,
((Spotted_gar.locus196.1:0.021787,Zebrafish.locus111.1:0.092191)93:0.027909,
(Reedfish.locus72574.1:0.047537,(Coelacanth.locus225.1:0.060024,
(Sterlet.locus167.1:0.000001,Sterlet.locus136.1:0.000001)100:0.035881)50:0.017200)33:0.010878
)63:0.022592)67:0.012170,
(Western_clawed_frog.locus333.1:0.052560,Caecillan.locus19184.1:0.039687)56:0.015518)70:0.015
191)100:0.034683,Mouse.locus398.1:0.000001):0.000001,NM_013803.3.mouse.CaSR:0.000001);
